# Recognition of Immune Cell Markers of COVID-19 Severity with Machine Learning Methods

**DOI:** 10.1155/2022/6089242

**Published:** 2022-04-28

**Authors:** Lei Chen, Zi Mei, Wei Guo, ShiJian Ding, Tao Huang, Yu-Dong Cai

**Affiliations:** ^1^School of Life Sciences, Shanghai University, Shanghai 200444, China; ^2^College of Information Engineering, Shanghai Maritime University, Shanghai 201306, China; ^3^Shanghai Institute of Nutrition and Health, Chinese Academy of Sciences, Shanghai 200031, China; ^4^Key Laboratory of Stem Cell Biology, Shanghai Jiao Tong University School of Medicine (SJTUSM) & Shanghai Institutes for Biological Sciences (SIBS), Chinese Academy of Sciences (CAS), Shanghai 200031, China; ^5^Bio-Med Big Data Center, CAS Key Laboratory of Computational Biology, Shanghai Institute of Nutrition and Health, University of Chinese Academy of Sciences, Chinese Academy of Sciences, Shanghai 200031, China; ^6^CAS Key Laboratory of Tissue Microenvironment and Tumor, Shanghai Institute of Nutrition and Health, University of Chinese Academy of Sciences, Chinese Academy of Sciences, Shanghai 200031, China

## Abstract

COVID-19 is hypothesized to be linked to the host's excessive inflammatory immunological response to SARS-CoV-2 infection, which is regarded to be a major factor in disease severity and mortality. Numerous immune cells play a key role in immune response regulation, and gene expression analysis in these cells could be a useful method for studying disease states, assessing immunological responses, and detecting biomarkers. Here, we developed a machine learning procedure to find biomarkers that discriminate disease severity in individual immune cells (B cell, CD4^+^ cell, CD8^+^ cell, monocyte, and NK cell) using single-cell gene expression profiles of COVID-19. The gene features of each profile were first filtered and ranked using the Boruta feature selection method and mRMR, and the resulting ranked feature lists were then fed into the incremental feature selection method to determine the optimal number of features with decision tree and random forest algorithms. Meanwhile, we extracted the classification rules in each cell type from the optimal decision tree classifiers. The best gene sets discovered in this study were analyzed by GO and KEGG pathway enrichment, and some important biomarkers like TLR2, ITK, CX3CR1, IL1B, and PRDM1 were validated by recent literature. The findings reveal that the optimal gene sets for each cell type can accurately classify COVID-19 disease severity and provide insight into the molecular mechanisms involved in disease progression.

## 1. Introduction

Since the outbreak of the novel coronavirus at the end of 2019, the novel coronavirus has spread to all regions of the world in less than one and a half years, causing more than 156 million confirmed infections and 3.2 million deaths worldwide, leading to the most severe viral pandemic worldwide in the past 100 years [1]. After the outbreak, the new coronavirus was first named 2019-novel coronavirus (2019-nCoV); later, the International Committee on Taxonomy of Viruses (ICTV) classified it as severe acute respiratory syndrome coronavirus- (SARS-CoV-) related virus and renamed it as SARS coronavirus-2 (SARS-CoV-2). The World Health Organization (WHO) named the disease caused by SARS-CoV-2 as coronavirus disease 2019 (COVID-19).

COVID-19 was first reported at the end of 2019. In early January 2020, researchers had isolated and identified the virus for the first time in the world, completed its whole genome sequencing, and submitted the genome sequence information of SARS-CoV-2 to the WHO. According to its genome sequence information, the virus is a new type of coronavirus, which is evolutionarily similar to the SARS coronavirus that caused “SARS” in 2003, and belongs to the *β*-coronavirus genus of the coronavirus family [2]. SARS-CoV-2 has a 79% sequence consistency with SARS coronavirus and a 50% sequence consistency with the Middle East Respiratory syndrome coronavirus (MERS-COV) [3]. Similar to other *β*-coronaviruses, SARS-CoV-2 is a single-stranded positive-strand RNA virus. Its genome is composed of a 30 kb positive-stranded RNA and contains 6 functional open reading frames (ORF), encoding replicase (ORF1a/ORF1b), spike protein (S protein), envelope protein (E protein), membrane protein (M protein), and nucleocapsid protein (N protein) [4].

According to a large-scale cohort study conducted by the Chinese Center for Disease Control and Prevention (CCDC), more than 19% of patients diagnosed with COVID-19 will develop severe or critical illness [5]. The clinical symptoms of SARS-CoV-2 infection include fever, pneumonia, sepsis, respiratory disorders, acute respiratory distress syndrome (ARDS), multiple organ damage, and so on [6]. In addition, there are also many reports of impaired taste and smell all over the world [7]. Patients infected with SARS-CoV-2 may also have no pathogenic symptoms, such as presymptomatic patients and asymptomatic patients. Although most patients only show mild symptoms, the condition of COVID-19 develops rapidly, especially in the absence of adequate medical and nursing care. Several COVID-19 infection-related signatures and rules at different omics levels have been reported by machine learning, which may contributed to exploring the pathology, improving the diagnosis accuracy of COVID-19 and finding new targets for vaccine design [8–11].

SARS-CoV-2 infection will cause a series of physiological and pathological changes in the body, including the stimulation of innate immunity and adaptive immunity. On the one hand, innate immunity and inflammation can produce antiviral effects and further stimulate adaptive immunity, helping the body to resist viral infections; on the other hand, excessive innate immunity and inflammatory response can lead to immune overload, inflammatory factor storms, and microthrombosis, which is considered to be one of the main pathogenic causes of ARDS and multiple organ damage caused by SARS-CoV-2 [12]. COVID-19 induces the production of a large number of inflammatory factors in patients, and its disease process and severity are closely related to the degree of immune response. The infection of COVID-19 can affect the immune cells and inflammation levels in the body including inflammatory factors, chemokines, growth factors, metabolites, and lipids. For example, Xu et al. [13] collected blood samples from COVID-19 patients and found that compared with healthy volunteers, 20 types of cytokines, chemokines, and growth factors (CCGFs) were increased in the plasma of patients with mild, severe, and dead COVID-19 patients. Moreover, there were 16 kinds of CCGFs, including HGF, CXCL8/IL-8, CCL7/cP-3, CCL2/McP-1, CXCL9/MIG, CXCL10/IP-10, IL-6, IL-18, IL-2, M-CSF, IL-1R*α*, IL-2R*α*/CD25, IFN-*γ*, CC L3/MIP-1*α*, FGF, and SCF which were abnormally elevated in patients who died from COVID-19. COVID-19 patients with different disease severity exhibit significantly altered plasma proteins which can be used as biomarkers to predict different clinical outcomes of COVID-19 patients, such as from severe disease to death, from mild disease to severe disease, and from severe or mild disease to recovery. A recent research conducted proteomic systematic analysis of plasma protein in patients with COVID-19 death, severe, and mild disease progression and found a large number of unique protein changes in patients with different clinical outcomes, involving a variety of physiological and pathological pathways, such as platelet shedding, complement and coagulation system, and metabolism [14]. To further explore the variation of various immune cells in blood of COVID-19 patients, here we incorporated single-cell profiles of peripheral blood mononuclear cells (PBMC) from 14 healthy controls (HC) and 33 single-cell profiles of COVID-19 patients including 3 moderate patients, 5 severe patients, and 25 critical patients and identified the decision rules and genes that clearly distinguish the immune cells in HC and COVID-19 patients of different severity, to figure out the complex immune process of SARS-CoV-2 infection and discover the immune response variation in patients with different severities, by which we hope to provide insight into the potential pathogenesis, diagnosis, and prognosis of COVID-19.

## 2. Materials and Methods

### 2.1. Study Design

**Figure 1:**
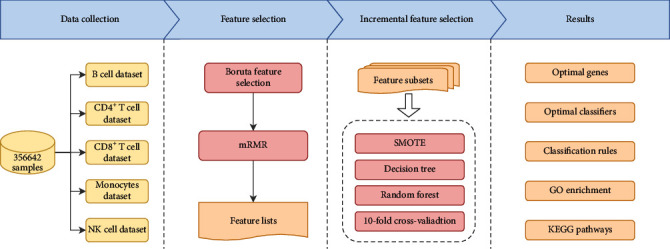
Computational workflow for this study. First, we applied Boruta and mRMR methods to filter and rank features of expression profiles for different immune cells (B cell, CD4^+^ T cell, CD8^+^ T cell, monocyte, and NK cell). Then, using the incremental feature selection method, a series of feature subsets were generated, and training samples made up of these feature subsets were used to train decision tree and random forest with 10-fold cross-validation. Based on the evaluation metrics of the model, the optimal number of features under each cell type was determined, and the optimal classifiers and classification rules were established as well. The GO and KEGG functional analyses were performed on these selected gene sets.

The machine learning-based workflow in this study is shown in Figure 1, which has three main sections: (1) data collection for five types of immune cells, (2) features of each dataset are filtered and ranked using Boruta and mRMR, and (3) identifying key biomarkers and classification rules using incremental feature selection method and performing biofunction analysis.

### 2.2. Datasets

**Figure 2:**
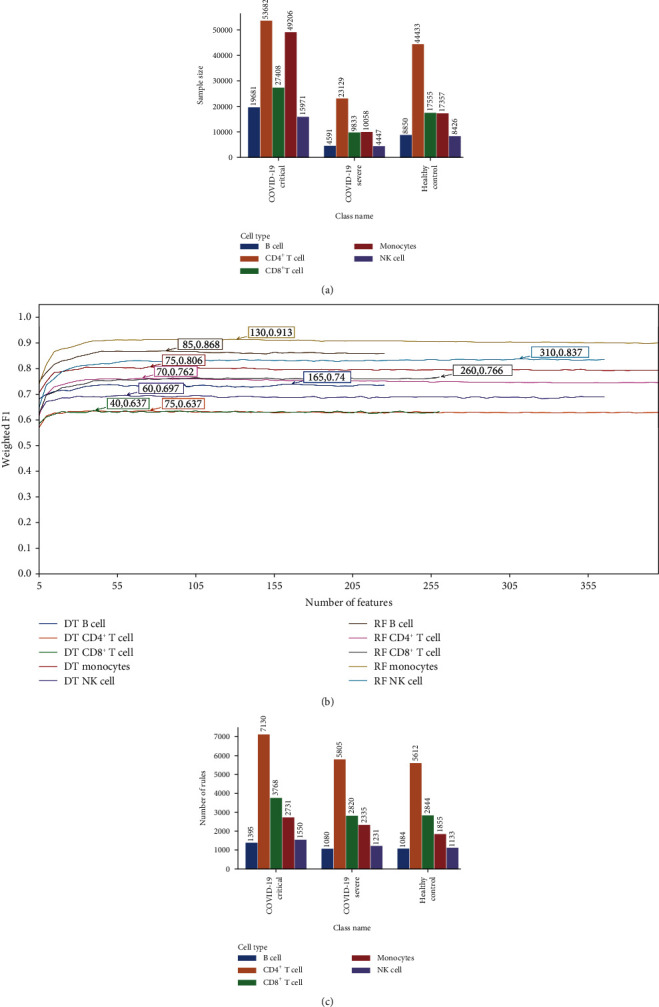
Details of the sample sizes and analysis results. (a) Sample sizes for different disease severity in each immune cell type. (b) IFS curves generated by decision tree and random forest in different immune cell types, the highest point of each curve was marked. (c) Number of classification rules extracted by the optimal decision tree classifiers.

The expression profiles of five cell types (B cell, CD4+ T cell, CD8+ T cell, monocytes, and NK cell) were retrieved from the GEO database with accession GSE161918 (https://www.ncbi.nlm.nih.gov/geo/query/acc.cgi?acc=GSE161918) [15]. These expression profiles include three groups, COVID-19 critical, COVID-19 severe, and healthy control, and the sample size for each group in each profile is provided in Figure 2(a). Furthermore, each expression dataset contains 473 gene attributes, and we will employ a computational workflow to extract the optimal gene set from these gene features that distinguishes COVID-19 states for a specific cell type.

### 2.3. Boruta Feature Filtering

Boruta is a random forest-based feature selection algorithm [16]. To begin, it introduces randomness to the dataset by generating shadow features from the original features. It then uses the extended dataset to train a random forest classification model and assesses the importance of each feature. It analyzes whether a real feature is more important than the best shadow feature in each iteration, removing features it finds trivial. The algorithm comes to a halt after all features have been confirmed or rejected. This work utilized the Boruta tool from https://github.com/scikit-learn-contrib/boruta_py with default parameters.

### 2.4. mRMR

Based on the results of Boruta feature filtering, mRMR [17] was applied to rank the retained features according to maximum relevance with the category and the minimum redundancy between features and features.

The mutual information (MI) is defined as shown in the following equation:

(1) $$\begin{array}{c} I\left(x,y\right)=\iint p\left(x,y\right)\log\frac{p\left(x,y\right)}{p\left(x\right)p\left(y\right)}dxdy, \end{array}$$

where *p*(*x*, *y*) represents the joint probabilistic density of *x* and *y* and *p*(*x*) and *p*(*y*) represent the marginal probabilistic densities of *x* and *y*, respectively.

Let *Ω* denotes the already selected gene features, *f*_*i*_ or *f*_*j*_ represents a gene feature in *Ω*, and *C* is the dataset label. The mRMR function is defined as follows:

(2) $$\begin{array}{c} \text{mRMR}=\frac{1}{\left|\Omega \right|}\underset{f_{i}\in \Omega }{\sum }I\left(f_{i},C\right)-\frac{1}{{\left|\Omega \right|}^{2}}\underset{f_{i}f_{j}\in \Omega }{\sum }I\left(f_{i,}f_{j}\right), \end{array}$$

where *I*(*f*_*i*_, *C*) indicates the mutual information between the *f*_*i*_ gene feature and label *C* and *I*(*f*_*i*_, *f*_*j*_)  represents mutual information between *f*_*i*_ and *f*_*j*_.

The mRMR program was obtained from http://www.home.penglab.com/proj/mRMR/ for this work and run with the default settings.

### 2.5. Incremental Feature Selection

IFS constructs classification models using the supervised algorithms to determine the best number of features in each single cell profiles [18]. In this analysis, a series of feature subsets were created using a step size of 5 based on the ranked feature list acquired from mRMR. For example, the top 5 features are present in the first feature subset, and the top 10 features compose the second feature subset. For each subset, a classifier (e.g., random forest (RF) [19]) is trained and tested by cross-validation on the dataset that is made up of this feature subset. An optimal feature subset is determined when it has the best performance in candidate feature subsets, where the performance is evaluated by tenfold cross-validation [20]. The classifier with such a feature subset can be built and is referred to as the optimum classifier.

### 2.6. SMOTE

Since the sample size for each category in each dataset is greatly different, the SMOTE method was used to balance the dataset [21]. This method uses the *k*-nearest neighbor algorithm to linearly synthesize new sample data for minority classes, resulting in an equal number of samples for each class. During the tenfold cross-validation procedure, these new data are utilized to train the classification model and improve its performance. For this analysis, we used the SMOTE tool from https://github.com/scikit-learn-contrib/imbalanced-learn with default parameters.

### 2.7. Classification Algorithms

#### 2.7.1. Random Forest

RF [9, 11, 19, 22–25] is a classification or regression algorithm that uses the Bagging concept of ensemble learning by incorporating many trees. In RF, the original data is first resampled using the bootstrap method to generate numerous sample datasets; next, for each sample dataset, a decision tree prediction model is built, and the final results are obtained by voting taken from the predictions of each tree. The model can handle data with a lot of dimensions and has a quick convergence rate and few adjustment parameters. In this study, the random forest function in Scikit-learn [26] is utilized with default parameters.

#### 2.7.2. Decision Tree

DT [9, 22, 27–29] is built based on the “IF-THEN” tree structure. Starting with the root node, DT judges the test of the node and assigns the instance to its child nodes based on the judgment result, where each node corresponds to a test of a feature, and so on, recursively, until the instance is assigned to a leaf node. Furthermore, DT can produce comprehensible classification rules that statistically characterize the pattern of feature expression. The DT was run with default parameters using the Scikit-learn software in this work.

### 2.8. Measurement

The classification model's prediction ability was evaluated using the *F*_1_ score [30–32]. It is calculated as follows:

(3) $$\begin{array}{c} F_{1}=2\times \frac{\text{precision}\times \text{recall}}{\text{precision}+\text{recall}},\\ \text{precision}=\frac{\text{TP}}{\text{TP}+\text{FP}},\\ \text{recall}=\frac{\text{TP}}{\text{TP}+\text{FN}}, \end{array}$$

where TP stands for true positive, FP for false positive, and FN for false negative. In a multiple classification task, the precision and recall for each class are determined first, and then, the sample weights are used to calculate the weighted *F*_1_ score.

### 2.9. Enrichment Analysis

In order to investigate the biological significance of the selected optimal genes, we employed ontology (GO) analysis to discover the roles of these genes and applied Kyoto Encyclopedia of Genes and Genomes (KEGG) pathway analysis to determine the essential pathways. The clusterProfiler package [33] in R was used to perform GO and KEGG enrichment analyses, with a threshold of *p* = 0.05.

## 3. Results

### 3.1. Results of Feature Selection

To identify key features in each single-cell expression profile, irrelevant features were first eliminated using Boruta, and then, mRMR was used to rank the retained features according to their importance, and the results are shown in Table S1. The lengths of the feature lists for B cell, CD4^+^ T cell, CD8^+^ T cell, monocytes, and NK cell were 229, 401, 263, 401, and 368, respectively, and these feature lists would be fed into the IFS method to identify the optimum number of features.

### 3.2. Results of IFS Method with DT and RF Classifiers

The IFS method was integrated with the RF and DT classifiers to find the best number of features and build the best classification models based on the feature lists provided by mRMR. During the training process, SMOTE was used to increase the amount of minority class samples, and tenfold cross-validation and weighted *F*_1_ scores were used to assess the model's performance. Table S2 contains the weighted *F*_1_ scores for each classifier in several immune cell types with a various number of attributes. IFS curves (Figure 2(b)) were drawn using the number of features as the *x*-axis and the weighted *F*_1_ scores as the *y*-axis to make the presentation easier. As can be seen from the picture, the RF outperforms DT in each cell dataset. Under the first 85, 70, 260, 130, and 310 features, B cell, CD4^+^ T cell, CD8^+^ T cell, monocytes, and NK cell have the best performance, with weighted *F*_1_ values of 0.87, 0.76, 0.77, 0.91, and 0.84, respectively. The RF that is developed using these features is considered to be the best classifier in each cell dataset. And the ability to distinguish the COVID-19 disease severity at the B cell and monocytes is excellent. All of these optimal RF classifiers perform well, demonstrating the efficacy of the computational methods we devised in this study.

### 3.3. Classification Rules Extracted by Optimal DT Classifier

When using the first 165, 75, 40, 75, and 60 features, B cell, CD4^+^ T cell, CD8^+^ T cell, monocytes, and NK cell created the best DT classifier (Figure 2(b)). DT was able to produce interpretable classification rules that provide the basis for quantitative gene expression. We used the best DT classifier for each dataset to extract classification rules, which are listed in Table S3. The number of rules for different disease states in each cell type is shown in Figure 2(c). It is obvious that the number of rules related to disease states is high in all cell types, which will benefit the diagnosis of COVID-19. Some important rules would be described in Discussion.

### 3.4. Analysis of Biological Functions

**Figure 3:**
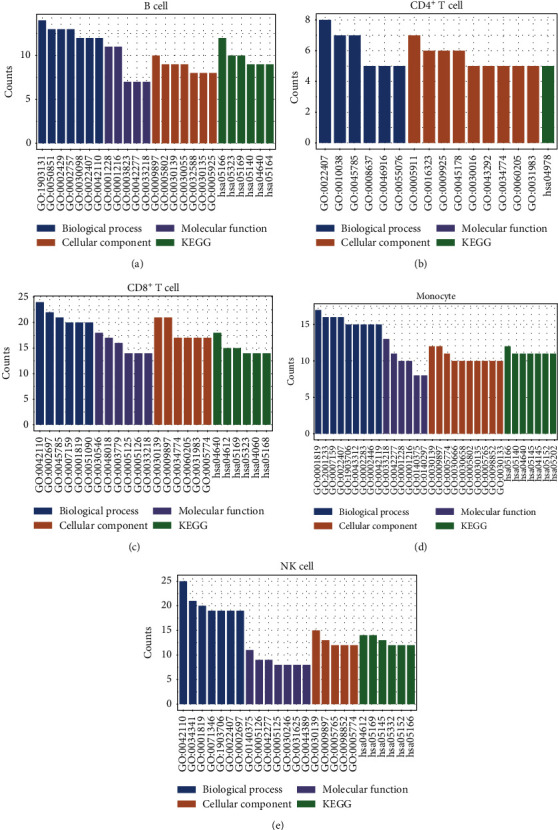
Results of GO and KEGG enrichment analyses in different immune cell types.

To conduct functional analysis of the selected optimal feature subsets, we used GO and KEGG pathway enrichment analyses with the top 85 (B cells), 70 (CD4^+^ T cell), 260 (CD8^+^ T cell), 130 (monocyte), 310 (NK cell) gene features, and all results are shown in Table S4. The main GO terms and KEGG pathways enriched in the different gene sets are provided in Figure 3. Among them, GO terms mainly contain three parts, biological process, molecular function, and cellular component.

## 4. Discussion

It has been reported that SARS-CoV-2 infection can produce cytokine storm or cytokine release syndrome (CRS) in some patients, which can be reflected in the increased inflammatory response and increased levels of a series of inflammatory factors in the blood [34]. Therefore, our study is aimed at finding the discriminative genes and rules between various immune cells (B cell, CD4^+^ T cell, CD8^+^ T cell, monocytes, NK cell) in healthy control, severe, and critical COVID-19 patients to shed new light on the immune response changes during the infection and development of COVID-19. We concentrated on several top features and decision rules because they have a vital influence on the classification. Then, we performed functional enrichment analysis in each cluster to explore the biological function and discussed them further through a wide literature publication to demonstrate our findings are trusted and convincing. Some key genes for next analysis are listed in Table 1.

### 4.1. Discriminative Genes and Rules in B Cell

The top Gene Ontology (GO) terms in B cells include interferon-gamma-mediated signaling pathway (GO:0060333), cellular response to type I interferon (GO:0071357), immune response-activating signal transduction (GO:0002757), and positive regulation of cytokine production (GO:0001819) indicating the alterant immune response in B cell belonging to HC, moderate, severe, and critical COVID-19 patients. KEGG pathways in B cells include antigen processing and presentation (hsa04612), TNF signaling pathway (hsa04668), and NF-kappa B signaling pathway (hsa04064) which represents that the immune alterations may occur through TNF and NF-kappa B signaling pathway. Therefore, based on the GO and KEGG functions, we concentrated on the related genes.

TLR2 belongs to Toll-like receptors (TLRs) family which are the primary receptors for innate immunity and can recognize a variety of microbial-associated molecular patterns (MAMPs). Moreover, TLRs have specific MAMPs. For example, the ligands of TLR2, TLR4, and TLR5 are lipid membrane acid of Gram-positive bacteria, lipopolysaccharide of Gram-negative bacteria, and flagellin of bacterial, respectively [35]. TLRs participate in the activation of mature B lymphocytes. The activation of B cell proliferation and immune globulin secretion by CpG-DNA and lipopolysaccharide of bacterial is known to be mediated by TLR9 and TLR4 [36, 37]. Similarly, the recognition of bacterial lipoproteins by TLR2 has been reported to trigger the activation of B lymphocytes [38, 39]. The latest study found that contrary to the effect of TLR4 in activating T cells, TLR2 is involved in preventing/delaying the maturation of B cells. Simultaneous addition of TLR2 and TLR4 ligands in in vitro experiments revealed the antagonistic effect between these stimuli [40]. Therefore, TLR2 plays an important role in B cell maturation and may be treated as a potential therapeutic target in the treatment of COVID-19.

BCL2A1 encodes a member of BCL2 family which is a group of heterodimers that play key roles in the regulation of various cellular activities, such as the development of embryonic development, homeostasis, and tumorigenesis. The BCL2 protein is a direct transcription target of the NF-kappa B family. It is also upregulated by various extracellular signals including inflammatory cytokine TNF and IL-1. Therefore, it is supposed that BCL2 acts as a cytoprotective protein which is involved in lymphocyte activation and cell survival. Sochalska et al. [41] constructed a BCL2A1 conditional knockdown model and demonstrated that BCL2A1 is a target of several key kinases that regulate the B cell receptor's survival signals. These enzymes include the Brutons tyrosine kinase and the spleen tyrosine kinase. It is believed that BCL2A1 could be utilized as a therapeutic target for the treatment of certain B-cell-related pathologies.

CD79a and CD79b located on the surface of B cells and functions as antigen receptor helper molecules. In the immune system, B lymphocytes mediate humoral immunity through the production of antibodies by plasma cells to destroy antigens. CD79a and CD79b play an important role in the transduction of antigen stimulation signals. Both of them are members of the immunoglobulin superfamily and are composed of extracellular regions, transmembrane regions, and relatively long cytoplasmic regions. CD79a and CD79b formed a dimer by disulfide bonding in the extracellular region near the membrane. Both CD79a and CD79 have polar amino acids in the transmembrane region, which form stable BCR complexes with Ig through electrostatic interaction. The cytoplasmic regions of CD79a and CD79b contain immunoreceptor tyrosine-based activation motif (ITAM), which can recruit downstream signal molecules to transduce signals generated by the binding of specific antigens and BCR into B cells. In addition, CD79a protein is present throughout the life course of B cells and is not present in other healthy cells, so it is regarded as a marker molecule of B cells.

NR4A1 is a member of nuclear hormone receptor NR4A family and has a main expression in immune cells. NUR77 (encoded by NR4A1), NURR1 (NR4A2), and NOR1 (NR4A3) constitute NR4A family, and they are known to be involved in the development of B cells [42]. Immature B cells in bone marrow exhibit low levels of NR4A1 and increase consistently during the transitional stages of the development process. In mature B cells, the expression value of NR4A1 reaches its peak. Park et al. [43] carried on the detailed explanation of the function of the NR4A1 in B cell. They found that the NUR77-deficient mice have elevated levels of all four class antibodies, which can trigger autoimmune response and exhibited an increase in CD38hi memory B cells and GL7+ germinal center B cells. Also, B cells in NUR77-deficient mice that were stimulated with anti-CD40 and anti-IgM survived better than those B cells in C57BL/6 wild-type mice. In conclusion, NUR77^−/−^ mice exhibited an autoimmunogenic B cell response, and NUR77 plays an important role in B cell survival and activation.

### 4.2. Discriminative Genes and Rules in CD4+ T Cell

KEGG pathway analysis of discriminative genes in CD4^+^ T cell leads to an important term, coronavirus disease—COVID-19 (hsa05171), directly supporting the reliability of our classifier that identified features highly related to COVID-19 infection. Hsa05171 pathway refers to that SARS-COV-2 infects alveolar epithelial cells, mainly alveolar epithelial type 2 (AEC2) cells, through angiotensin converting enzyme 2 (ACE2) receptor. And after SARS-COV-2 occupies ACE2, serum free angiotensin II (Ang II) levels increase due to decreased ACE2-mediated degradation, which promotes NF-*κ*B pathway activation via Ang II type 1 receptor (AT1R) and finally leads to the overproduction of proinflammatory cytokines. Therefore, COVID-19 infection may induce the alteration of CD4^+^ T cell effector function and lead to aberrant secretion of cytokines which are discussed further below.

The Tec family tyrosine kinase, ITK, mainly expressed in T cells, is essential for the production and development of CD4^+^ T cell effector function. Signal transducing by TCR results in the activation of ITK and the combination with multimolecular complexes including SLP-76, LAT, Gads, Grb2, and PLC-*γ*1 [44, 45]. It has been proved by several researches that the deficiency of ITK impairs the TCR signal pathway. In ITK-deficient T cells, the phosphorylation and activation of PLC-*γ*1 tyrosine, intracellular calcium mobilization, MAP kinase activation, and NFATc nuclear translocation are blocked [46–49]. Therefore, under the TCR stimulation, the ITK^−/−^ CD4^+^ T cell has a decreased secretion of cytokines such as IL-2, IL-4, IFN-*γ*, and FasL [46, 48, 49].

HPGD gene is located on chromosome 4q34.1 with a length of 31 kb and consists of 7 exons. It encodes the catalytic enzyme, hydroxyprostaglandin dehydrogenase 15-(NAD) (HPGD), which catalyzed the dehydrogenation of prostaglandin E2 (PGE2). PGE2 signaling can increase the ratio of naive CD4+ T cells differentiating into helper T (Th) 1 cells [46]. According to the research of Schmidleithner et al. [50], *Hpgd* conditional knockout in mouse Treg cells leads to the deposition of Treg cells which has functional defect in visceral adipose tissue and triggers an autoimmune response, indicating the important role of HPGD in naive CD4^+^ T cells differentiation.

### 4.3. Discriminative Genes and Rules in CD8+ T Cell

According to the discriminative genes in CD8^+^ T cell, the top Gene Ontology (GO) terms and KEGG pathways consist of T cell activation (GO:0042110), Th17 cell differentiation (hsa04659), and Th1 and Th2 cell differentiation (hsa04658). Therefore, we suspect that the infection of COVID-19 may influence the differentiation of T cell and so we place emphasis on these genes.

CX3CR1 encodes a functional protein with 1065 nucleotides and 355 amino acids, which is a specific and high affinity receptor for the chemokine CX3CL1 and can induce phosphorylation of downstream genes and participate in corresponding signal transduction. Expression of CX3CR1 has been recently found to represent a subset of memory T cell [51]. Memory T cell is a long-lived cell differentiated from effector CD8^+^ T cells, and it can be divided into three types according to the specific expression of phenotypic markers such as KLRG1, CD127, CD27, and CX3CR1. CX3CR1^+^ memory T cell is an effector memory phenotype and has unique abilities in tumor therapy including withstanding the toxicity of chemotherapy and proliferating when using chemoimmunotherapy [51]. What is more, CX3CR1^+^ memory T cell plays a major killing role in secondary infection, while CX3CR1^−^ memory T cell has almost no cytotoxicity [52]. Therefore, we can infer that the magnitude and duration of T-cell immune response form the changes in expression levels of CX3CR1.

The tumor necrosis factor alpha-induced protein 3 (TNFAIP3), as a ubiquitinated editing protein, has been identified as a dual inhibitor of NF-*κ*B activation and cell death. The basic expression of TNFAIP3 was very low, but it was quickly induced after NF-*κ*B activation and gave rise to the disintegration of the K63-linked polyubiquitin from the adapter protein RIP1 replaced by the K48-linked polyubiquitin chain, leading to the inhibition of TNF-induced NF-*κ*B activation, which could be regarded as a negative feedback regulator of NF-*κ*B [53]. TNFAIP3 is associated with the development of multiple inflammatory pathologies. Giordano et al. found that [54] TNFAIP3 selective knockout mice exhibited robust antigen sensitivity and cytotoxicity in CD8 T cells, with an increased generation of IL-2 and IFN*γ*. Meanwhile, in vivo experiment showed more forceful antitumor activity in TNFAIP3 knockout CD8 T cells.

### 4.4. Discriminative Genes and Rules in Monocyte

GO terms of discriminative genes in monocyte includes positive regulation of cytokine production (GO:0001819), regulation of mononuclear cell proliferation (GO:0032944), antigen processing and presentation of peptide or polysaccharide antigen via MHC class II (GO:0002504), and negative regulation of immune system process (GO:0002683). SARS-CoV-2 can activate the innate immune system, triggering the overproduction of proinflammatory cytokines and the “cytokine storm,” which results in systemic inflammatory response syndrome and multiple organ failure. Monocyte is an important component of the innate immune system. Due to the infection of COVID-19, distinct changes have taken place in monocytes including the proliferation and function.

IL1B gene is located on chromosome 2 and encodes interleukin-1*β* (IL-1*β*) which is mainly expressed in activated monocytes [54]. IL-1*β* is derived from the interleukin 1*β* protein precursor (proIL-1*β*), which is synthesized when monocytes are stimulated [54]. ProIL-1*β* has few biological functions, and through intracellular inflammasome or extracellular proteases, it can be converted to mature IL-1*β* by proteolytic processing [55]. As an inflammatory cytokine with multiple effects, IL-1*β* plays a role in a series of inflammatory responses and immune regulation in the body and is closely related to the occurrence of chronic inflammation and the occurrence and development of tumors.

IFITM3 is a member of interferon-induced transmembrane proteins (IFITMs), a family of small molecule homologous proteins located in the cytoplasm and lysosomal intima, which enable cells to develop resistance to a variety of viruses. IFITM3 is a broad-spectrum host limiting factor, highly induced by type I and type II interferon, and shows antiviral activity against a variety of viruses including influenza A virus (IAV), HIV-1, Ebola, SARS coronavirus, and dengue virus. Wellington et al. [56] used CyTOF to measure the expression level of IFITM3 in individual cell types of human adult blood samples. It shows that IFITM3 has a highest expression in CD16^+^ monocytes which can be inferred that IFITM3. Other studies have shown that after viral infection, IFITM3 is induced by type I interferon and promotes the degradation of IFN regulatory factor 3 (IRF3) by enhancing IRF3 autophagy and migration to autophagosome, thus negatively regulating the RIG-I-like receptor- (RLR-) mediated signaling pathway of type I interferon production [57].

### 4.5. Discriminative Genes and Rules in NK

GO and KEGG analyses of discriminative genes in NK cell both point to the functions associated with virus infection including response to virus (GO:0009615), viral life cycle (GO:0019058), and viral protein interaction with cytokine and cytokine receptor (hsa04061). NK cells are an important part of the innate immune system and play a crucial role in the early stage of antitumor and antivirus. There are many ways for NK cells to kill target cells, such as directed exocytosis and specific secretion of lysosomes, which are called cytotoxic particles containing perforation protein, granzyme, and Fas ligand [58]. Another way is to secrete INK-*γ* to enhance the cytotoxicity of NK cells after encountering susceptible target cells or stimulated by IL-2, IL-12, IL-15, and IL-18 released by other cells [58, 59]. PRDM1 is a transcription inhibitor and acts as a key negative regulator of NK function. It synergistically inhibits TNF-*α*, TNF-*β*, and IFN-*γ* by directly binding to multiple conserved regulatory regions. Downregulated expression of PRDM1 causes increased expression of TNF-*α* and IFN-*γ*, whereas upregulated expression blocks cytokine generation [60].

Collectively, in various immune cells (B cell, CD4^+^T cell, CD8^+^T cell, monocyte, NK cell), the top identified discriminative feature genes and settled rules between patients with different disease severity have been confirmed to play a crucial role in individual cell clusters, demonstrating that our method is reliable and convincing. Our results are consistent with a previous systematic proteomic analysis of plasma in different disease processes of patients with death, severe, and mild diseases of COVID-19 that demonstrated that a large number of plasma proteins have changed in COVID-19 patients with different clinical outcomes [13]. Therefore, based on the single-cell expression profiles from blood samples of HC, moderate, severe, and critical COVID-19 patients, our newly presented computational approach provides a new perspective for exploring the mechanism of COVID-19 and predicting different clinical outcomes in patients with COVID-19.

## 5. Conclusions

In summary, to distinguish COVID-19 disease severity at the immune cell level, we used Boruta and mRMR methods to filter and rank features of the expression profiles for each immune cell type. After that, using incremental feature selection, the optimal number of features was identified, and optimal classifiers and classification rules were constructed. Finally, the selected gene sets were subjected to GO and KEGG functional analyses, and some of the key genes were validated using recent literature. The findings suggest that these gene features are important in differentiating disease severity in distinct immune cells, demonstrating the validity of our computational method. The key gene markers identified in this study can serve as potential targets in the clinical prognosis and treatment of COVID-19. Our predictive model can be clinically applied into disease stratification of large cohort of COVID-19 patients according to the disease severity, which can contribute to providing appropriate medical support for the predicted severe patients to help improve the patient survival rate. Together, this research provides a new perspective to explore the mechanisms of COVID-19 and the ability to predict disease outcome.

## Figures and Tables

**Table 1:** Essential genes involved in Discussion.

Cell type	Gene symbol	Description
B cell	TLR2	Toll-like receptor 2
	BCL2A1	BCL2-related protein A1
	CD79A	CD79a molecule
	CD79B	CD79b molecule
	NR4A1	Nuclear receptor subfamily 4 group A member 1
CD4^+^ T cell	ITK	IL2 inducible T cell kinase
	HPGD	15-hydroxyprostaglandin dehydrogenase
CD8+ T cell	CX3CR1	C-X3-C motif chemokine receptor 1
	TNFAIP3	TNF alpha-induced protein 3
Monocyte	IL1B	Interleukin 1 beta
	IFITM3	Interferon-induced transmembrane protein 3
NK cell	PRDM1	PR/SET domain 1

## Data Availability

The original data used to support the findings of this study are available at Gene Expression Omnibus (https://www.ncbi.nlm.nih.gov/geo/query/acc.cgi?acc=GSE161918).

## Supplementary Materials

Supplementary 1 Table S1: feature lists obtained by Boruta and mRMR methods.

Supplementary 2 Table S2: performance of decision tree and random forest with different number of features in each cell type.

Supplementary 3 Table S3: classification rules extracted by the optimal decision tree classifiers in different immune cell type.

Supplementary 4 Table S4: detailed results of GO and KEGG enrichment analyses in different optimal gene sets.
