# Supplementary material for: Recognition of Immune Cell Markers of COVID-19 Severity with Machine Learning Methods
Source: Biomed Res Int. 2022 Apr 28;2022:6089242. doi: 10.1155/2022/6089242 (PMC9073549; doi:10.1155/2022/6089242)
Supplement: Supplementary 1 — Table S1: feature lists obtained by Boruta and mRMR methods. [file 6089242.f1.pdf]

Table S1. Feature lists obtained by Boruta and mRMR methods

Index	B cell	CD4+ T cell	CD8+ T cell	Monocytes cell	NK cell
1	LTB	MT-CO1	MT-CO1	NR4A1	AREG
2	MTRNR2L2	MTRNR2L2	MTRNR2L2	MTRNR2L2	IFI44L
3	CD79B	XIST	OAZ1	THBS1	MTRNR2L2
4	SNX9	OAZ1	IFI44L	G0S2	MT-CO1
5	MT-CO1	AIM1	AIM1	MAFB	CX3CR1
6	PLD4	SNX9	FTH1	ASPH	IFI6
7	TCL1A	IFI44L	IL32	FGL2	RGS1
8	NFKBID	IL32	SLC7A5	IL1R2	IL2RB
9	IFI44L	RGCC	IFI6	TNFRSF12A	MYOM2
10	VPREB3	PELI1	HLA-DQA2	LGALS2	TNFAIP3
11	OAZ1	SLC40A1	IL2RB	NFKBIZ	KLRC1
12	AREGB	SYTL3	MTRNR2L8	PLAUR	HES4
13	FAM129C	CASP8	CX3CR1	IFI27	SYTL3
14	MS4A1	FTH1	MT-CYB	DUSP6	NFKBIZ
15	XIST	PRDM1	MX1	HLA-DQB1	PRDM1
16	STAG3	AQP3	RGS1	CX3CR1	FTH1
17	HLA-DPA1	ILK	SYTL3	NR4A3	IFIT3
18	MEF2C	FERMT3	ILK	SEMA6B	DENND3
19	PDLIM1	LIMS1	CMC1	AREG	MX1
20	RALGPS2	ACTN1	PRDM1	MIR181A1HG	FCER1G
21	IGJ	MTRNR2L8	DUSP4	CLU	OAZ1
22	TP53INP1	MT-CYB	FERMT3	MS4A7	VCL
23	FTH1	SOD2	SNX9	RBKS	MT-CYB
24	FCER2	SESN3	ITK	SERPINB2	SLC7A5
25	BACH1	UTY	VCL	XIST	OASL
26	HLA-DPB1	FAM160B1	TNFAIP3	AGFG1	HOPX
27	ADAM19	IFI6	STOM	PLD4	HBA1
28	PELI1	LTB	DENND3	MNDA	KIR2DL2
29	MTRNR2L8	VCL	HPGD	VSIG4	JUN
30	HLA-DRB5	AP3M2	SLA2	RGCC	ILK
31	STMN1	NFKBIZ	ID1	IFITM3	ISG15
32	RP11-731F5.2	MX1	FCGR3A	HSPA1A	GRASP

33	TSPYL2	INPP4B	CASP8	TMEM176B	CLIC3
34	MT-ND6	RNU12	XIST	CD163	KLRG1
35	NR4A1	FTL	YWHAH	JARID2	IRF4
36	HLA-DQB1	DUSP2	FAM160B1	CD83	ZEB2
37	SESN3	RGS1	RHOB	HLA-DRB5	IFNG
38	FERMT3	MFSD1	RGCC	STXBP2	SLA2
39	CD79A	SLC7A5	AQP3	GRASP	AREGB
40	CCR6	ITK	TSPYL2	FTH1	PTGDS
41	RNF144B	NR4A3	XAF1	PPIF	GPR56
42	ICAM1	MT-CO3	RASGEF1B	VCL	GSN
43	HLA-DQA1	HOPX	KLRG1	CEBPD	XCL1
44	JUN	CD79B	IFIT3	CYP1B1	TSPYL2
45	MT-CO3	MT2A	AREG	LILRB2	FCGR3A
46	SNX29P2	NPIPB4	MFSD1	HLA-DPA1	XAF1
47	PLEC	IFIT3	IFI27	BACH1	MT-CO2
48	PRDM1	NGFRAP1	MT-ND6	RNASE2	EMR2
49	ATF5	ANK3	MYOM2	MTRNR2L8	HBA2
50	ITM2C	GRAP2	XCL1	TMEM176A	HSPA1A
51	MX1	PLA2G12A	ISG15	IL1B	GNLY
52	TLN1	RBKS	NR4A3	RNF144B	HLA-DQA2
53	ILK	LDLR	INPP4B	ITGA5	MTRNR2L8
54	PLA2G12A	HPGD	MT-CO3	FCGR3A	MT2A
55	IRF8	TAF4B	HIST1H3A	LMNA	CCL3
56	1-Mar	HIST1H3A	PLA2G12A	ILK	XIST
57	TNF	ZNF10	KLRF1	EGR1	AIM1
58	IGLL5	ID3	RNU12	LILRA5	MT-CO3
59	HSP90B1	ID1	GZMA	RP11-290F20.3	ENC1
60	GNG11	CMC1	ANK3	S100A9	CD38
61	IFIT3	COQ7	CTSA	NCOA4	IRF8
62	NFKBIZ	MT-CO2	RBKS	ID1	IFIT2
63	MEF2A	ISG15	ATG2A	RBM47	KLRF1
64	VCL	YWHAH	GADD45A	DSE	IFITM3
65	CYBB	JUN	JUN	SESN3	LGALS1
66	APP	CTSA	STMN1	SH2D1B	MMD
67	MT-CO2	MYOM2	CEP78	HLA-DQA1	LMNA

68	UTY	ADAM19	KLRC1	PTGS2	DUSP4
69	RP5-821D11.7	MT1E	OASL	LST1	YWHAH
70	RGS1	MT-ND6	IFITM3	SLC40A1	FERMT3
71	SPI1	TLN1	PELI1	LDLR	HLA-DPA1
72	RELT	FHL1	MS4A1	BCL2A1	HPGD
73	SNORD3B-2	AREGB	MT-CO2	FOLR3	TRAF1
74	TCF4	STMN1	CD38	CD79B	FAM160B1
75	MARCKS	FRMD4B	ACTN1	MT1E	XCL2
76	GPX1	RASGEF1B	CCL4L2	IER3	EGR1
77	CD83	AREG	MCOLN2	DAPP1	LDLR
78	RGCC	SLA2	LMNA	HRH2	HLA-DRB5
79	XAF1	DENND3	PDLIM1	MEF2C	STOM
80	IRF4	ANO6	UTY	MIR4435-1HG	CCL4
81	NT5C3A	PLEC	GZMK	ZEB2	PHLDA1
82	ATF3	RP11-796E2.4	LDLR	RGS18	IL32
83	EGR1	OASL	HSPA1B	ACSL1	S100A11
84	HLA-DRB1	IFI27	ZNF10	LRRK2	CASP8
85	TTN	TP53INP1	XCL2	YWHAH	TSC22D2
86	NAMPT	TSC22D2	SLC40A1	SERPINA1	SH2D1B
87	RP11-685N3.1	FAM13A	GPR56	RP11-796E2.4	CD83
88	RGS2	HRH2	NFKBIZ	CXCL2	KLRB1
89	SLC7A5	RP4-673M15.1	COQ7	RHOB	ATF3
90	RHOB	HSP90B1	EMR2	DUSP2	HLA-DQB1
91	HIST1H2AC	DUSP4	CDKN1A	TNF	RGCC
92	HSPA1A	IL2RB	ENDOD1	OASL	GZMK
93	FAM160B1	RUFY1	HLA-DRB5	CPVL	TPM4
94	KIAA0125	TNFAIP3	MTSS1	MEF2A	MCOLN2
95	LDLRAD4	XAF1	ABHD5	RNU12	HSPA1B
96	WDR74	CDC14A	FHL1	ETV6	LINC00936
97	GRASP	GZMK	NR4A1	PSAP	RP5-821D11.7
98	ANO6	EMR2	KIR2DL2	SGK1	RALGPS2
99	IFI6	PHLDA1	IRF4	PTPN18	ICAM1
100	MT-CYB	CTD-2562J15.6	CTLA4	MTSS1	FGL2
101	CD38	TRAF1	AREGB	HLA-DPB1	IFI27
102	ENDOD1	MMD	IFRD1	ADAM19	GZMB

103	MAP3K2	AGPAT9	ADAM19	SPI1	ATG2A
104	PARP14	TPM1	CCDC88A	THBD	HELLS
105	PPIF	MEF2A	PHLDA1	FAM160B1	PELI1
106	OASL	APP	ANKRD28	AHR	ABHD5
107	RNF11	CEP78	RP5-821D11.7	KIF2A	AP3M2
108	FCRL1	IL2RA	HBA1	IGSF6	CMIP
109	ID3	ENDOD1	ID3	SLC25A37	LGALS3
110	ATG2A	LMNA	FTL	ATG2A	ANKRD28
111	CASP8	ITM2C	TPM1	IFI44L	CD8A
112	DENND3	BACH1	CLIC3	IL1RN	MIR29A
113	HOPX	RALGPS2	TSC22D2	IL8	PPIF
114	HES4	HIST1H2AC	DTHD1	SYNE1	ZNF10
115	BOD1L1	SH3BP2	RGS2	CLEC7A	COTL1
116	ITGAX	MIR24-2	HES4	MCTP1	RP11-291B21.2
117	COTL1	SNORD3B-2	APP	AREGB	CD9
118	NR4A3	HLA-DPA1	DOCK5	TRAF1	HIST1H2BK
119	PTGS1	NABP1	MMD	HLA-DQA2	PARP14
120	ID1	CCDC88A	HBA2	PHLDA1	CTSW
121	GRAP2	S100A6	HLA-DPB1	TNFAIP3	CTD-2562J15.6
122	DUSP4	MCOLN2	DSE	STAB1	MIR181A1HG
123	SLC11A1	SYNE1	CTD-2035E11.3	CDKN1A	DOCK5
124	SYNE1	CTD-2035E11.3	SH2D1B	HPGD	IER3
125	SYTL3	HSPA1B	NPIPB4	LGALS3	SESN3
126	CCDC141	COTL1	HIST1H2AC	RALGPS2	IFRD1
127	CD22	HLA-DQA2	RTKN2	HLA-DRB1	CDKN1A
128	MMD	EGR1	IL1B	AIM1	GZMA
129	ISG15	CXorf57	MIR24-2	S100A8	FRMD4B
130	MIR24-2	SGK1	TRAF1	CCRN4L	HLA-DPB1
131	IL1B	PARP15	PARP14	TPM1	RP6-99M1.2
132	DAPP1	GZMA	HBB	44256	RASGEF1B
133	MT1E	CTLA4	FAM13A	FAM13A	BACH1
134	RBKS	RP11-350N15.5	PARP15	CASP8	TGFBR3
135	TNFAIP3	DUSP6	ATF5	CMC1	ATF5
136	ZNF10	IFITM3	MYO9B	CD36	HIST1H3A
137	ACSL1	ARHGAP18	HELLS	FCAR	ZNF683

138	MYL9	RRP12	NEAT1	PTGS1	KLRD1
139	CCL3	IFIT2	FRMD4B	SH3BP2	NR4A3
140	TPM1	PHLDA2	DUSP2	ENC1	ABCA1
141	TLR2	MS4A1	SGK1	SLC8A1	RP11-367G6.3
142	S100A6	RP5-821D11.7	IGLL5	LTB	DAPK1
143	MCTP1	TMEM140	IFIT2	NKG7	RALGAPA1
144	JARID2	HES4	MZB1	TBC1D8	ENTPD1
145	BANK1	S100A8	ARHGEF40	FRMD4B	DTHD1
146	IL8	MAP3K2	TUBB1	TLN1	IRAK3
147	LDLR	TSC22D1	HOPX	DMXL2	RP11-350N15.5
148	ABCA1	HLA-DQB1	MT2A	CD74	CRY1
149	TRIB1	DSE	CH17-132F21.1	CCL4	44257
150	HBB	BACH2	ABCA1	LINC00936	WARS
151	IRAK3	RALGAPA1	RP11-796E2.4	ABCA1	NFKBID
152	HLA-DQA2	ZNF683	TAF4B	GPX1	TNF
153	CTD-2035E11.3	RP11-138A9.1	ITGAV	IFI6	TYMP
154	TRAF1	ABCA1	ZEB2	SYTL3	ID1
155	AHR	ATG2A	MIR181A1HG	ADAM28	CDC14A
156	YWHAH	ATP6V0A1	VPREB3	CTD-2035E11.3	PRF1
157	NAIP	MLH3	DERL3	RRP12	MIR24-2
158	DAPK1	ABHD5	AQP9	PHLDA2	CD151
159	IFIT2	IGLL5	TNFRSF4	ENDOD1	NABP1
160	BCL2A1	RHOB	CCRN4L	CYBB	S100B
161	CXorf57	HBB	TLN1	LUCAT1	RNU12
162	MIAT	S100A12	C15orf48	CEP78	CCL4L2
163	AP3M2	RTKN2	SERPINB2	WDR74	MT1E
164	C15orf48	RGS2	CD8A	MT2A	CCRN4L
165	CSF3R	RAB27B	ANPEP	RP11-138A9.1	CTD-2035E11.3
166	ARHGAP24	IGJ	BOD1L1	AFF3	HBB
167	MACF1	MIAT	RP5-887A10.1	RP4-673M15.1	GPX1
168	CD93	CX3CR1	SEMA6B	ANPEP	STMN1
169	CD74	ATF5	FAM129C	XAF1	MT-ND6
170	MIR4435-1HG	RP11-206L10.2	HBG2	ATP6V0A1	NR4A1
171	ANKRD36C	ANKRD28	ADAM28	CCL3	HLA-DRB1
172	AGFG1	PDLIM1	CMIP	IRAK3	MTRNR2L12

173	IL1RN	ICAM1	PRKAR2B	SLC7A5	BCL2A1
174	GZMA	ITGA5	TYMP	IFRD1	CD74
175	RP11-367G6.3	PARP14	FOLR3	CTSL	S100A4
176	TNFRSF12A	CTSL	ZFHX3	F13A1	DUSP2
177	SGK1	MAP3K7CL	GNLY	NAIP	NEAT1
178	CD300E	MIR29A	CMTM5	MS4A6A	S100A6
179	PTPN18	STOM	VSIG4	NLRP3	SPON2
180	LUCAT1	CD74	MCTP1	PARP14	GZMH
181	DUSP2	HBA1	RP11-291B21.2	TP53INP1	CST7
182	ZFHX3	CD83	FCER2	RP5-821D11.7	FGFBP2
183	SH2D1B	IRF4	RP6-99M1.2	MIR29A	CMC1
184	CD151	RETN	AFF3	ATF5	NKG7
185	ANKRD28	S100A11	GNG11	MYOM2	AREG
186	RNU12	ARHGEF40	F13A1	CD22	IFI44L
187	AREG	ETV6	GP9	ITGAV	MTRNR2L2
188	ZEB2-AS1	TRBV11-2	PTGS1	HOPX	MT-CO1
189	ZEB2	CCRN4L	TRIB1	FABP5	CX3CR1
190	GSN	NEAT1	TBC1D8	TMEM140	IFI6
191	ETV6	ITGAV	S100A8	CD9	RGS1
192	IL2RB	CH17-132F21.1	CCL3	CST7	IL2RB
193	LMNA	RNF144B	ITGA2B	RP11-367G6.3	MYOM2
194	SPON2	HLA-DRB5	HIST1H3H	MAP3K2	TNFAIP3
195	IER3	LUCAT1	C6orf25	HBG2	KLRC1
196	RASGEF1B	NAMPT	NGFRAP1	RGS1	HES4
197	CCRN4L	BANK1	CD163	NGFRAP1	SYTL3
198	RP5-887A10.1	VPREB3	MT1E	LILRA3	NFKBIZ
199	ANK3	BLK	BACH1	S100B	PRDM1
200	APLP2	HSPA1A	NAMPT	GSN	FTH1
201	AFF3	HBG2	TREML1	SOD2	IFIT3
202	NEAT1	RP11-731F5.2	PTGDS	IGJ	DENND3
203	GLUL	CYP1B1	MACF1	NCF2	MX1
204	ENC1	KLRG1	PLXNB2	LYZ	FCER1G
205	IFITM3	RGS18	EGR1	HBB	OAZ1
206	MT2A	NFKBID	PF4	BANK1	VCL
207	LGALS1	S100A9	CTD-2562J15.6	HBEGF	MT-CYB

208	HRH2	CCL4L2	NFKBID	TGFBR3	SLC7A5
209	CLEC7A	CXCL2	TSHZ2	DUSP4	OASL
210	LINC00926	GNG11	SPARC	TAF4B	HOPX
211	CDKN1A	DERL3	C1QA	HMOX1	HBA1
212	FTL	FGFBP2	ITGA5	IFNG	KIR2DL2 0.01120
213	MS4A7	F13A1	TTN	UTY	JUN
214	BLK	ADAM28	BACH2	GZMH	ILK
215	NLRP3	HIST1H3H	FCAR	PLA2G12A	ISG15
216	KLF4	AFF3	S100B	RETN	GRASP
217	MTRNR2L12	SEMA6B	METTL12	RELT	CLIC3
218	HBEGF	RP5-887A10.1	HLA-DQB1	DERL3	KLRG1
219	FGL2	S100B	HBEGF	FCER2	IRF4
220	AIF1	MIR4435-1HG	THBD	KLF4	ZEB2
221	LGALS3	CD9	MIR4435-1HG	IGLL5	IFNG
222	FPR1	MACF1	FABP5	CRY1	SLA2
223	TIMP1	PTGDS	GRASP	CH17-132F21.1	AREGB
224	CD68	TCF4	ZNF683	KLRF1	PTGDS
225	HMOX1	TSPYL2	TGFBR3	HBA2	GPR56
226	RP11-290F20.3	MZB1	PLD4	CCR6	GSN
227	LILRB2	SLC8A1	ATF3	TTN	XCL1
228	BACH2	CDKN1A	RP11-290D2.6	TCL1A	TSPYL2
229	SERPINA1	PPBP	HLA-DQA1	KIAA0125	FCGR3A
230		IL1R2	PPIF	IL2RA	XAF1
231		KIAA0125	KLRB1	PLEC	MT-CO2
232		TRIB1	IRAK3	HIST1H3A	EMR2
233		SERPINB2	KLRD1	RP11-291B21.2	HBA2
234		CD93	S100A9	TYMP	HSPA1A
235		SH2D1B	CEBPD	MYL9	GNLY
236		CD22	TNF	KLRD1	HLA-DQA2
237		DOCK5	MTRNR2L12	TSHZ2	MTRNR2L8
238		HBA2	SESN3	RP5-887A10.1	MT2A
239		DAPK1	S100A11	MZB1	CCL3
240		CD8B	GPX1	TRBV11-2	XIST
241		ARHGAP24	S100A6	PRDM1	AIM1
242		NLRP12	FPR1	VPREB3	MT-CO3

243		1-Mar	ENC1	OSM	ENC1
244		NRGN	CCL4	HIST1H3H	CD38
245		CSF3R	CD74	P2RX1	IRF8
246		TNFAIP2	TIMP1	MIAT	IFIT2
247		CLIC3	SPON2	MX1	KLRF1
248		LRRK2	COTL1	HELLS	IFITM3
249		TCL1A	IFNG	S100A12	LGALS1
250		FAM129C	HLA-DPA1	CD38	MMD
251		NR4A1	CD8B	GZMK	LMNA
252		ZEB2	HLA-DRB1	NFKBID	DUSP4
253		PRKAR2B	LTB	FCRL1	YWHAH
254		FOLR3	S100A4	ANK3	FERMT3
255		COBLL1	SYNE1	MS4A1	HLA-DPA1
256		IRAK3	LGALS1	CTSW	HPGD
257		TIMP1	CTSW	TNFRSF4	TRAF1
258		FCER2	FGFBP2	FAM129C	FAM160B1
259		TLR2	GZMB	RASGEF1B	XCL2
260		PHACTR1	CST7	XCL2	EGR1
261		IFRD1	PRF1	ZNF683	LDLR
262		RP11-291B21.2	GZMH	RP11-731F5.2	HLA-DRB5
263		ITGAX	NKG7	RGS2	STOM
264		C15orf48		CD8B	CCL4
265		ZEB2-AS1		C6orf25	PHLDA1
266		KIR2DL2		XCL1	IL32
267		VSIG4		KLRC1	S100A11
268		FCRL1		CD8A	CASP8
269		BCL2A1		BLK	TSC22D2
270		LINC00926		RP11-685N3.1	SH2D1B
271		AQP9		KIR2DL2	CD83
272		TUBB1		RTKN2	KLRB1
273		MCTP1		FHL1	ATF3
274		PLXNB2		ICAM1	HLA-DQB1
275		ANPEP		CTLA4	RGCC
276		STAB1		DTHD1	GZMK
277		PTGS1		PTGDS	TPM4

278		PTGS2		SLA2	MCOLN2
279		SPON2		CDC14A	HSPA1B
280		P2RX1		CCR4	LINC00936
281		HELLS		BACH2	RP5-821D11.7
282		MEF2C		HBA1	RALGPS2
283		SOX4		FGFBP2	ICAM1
284		TREML1		TPM4	FGL2
285		KLRC1		LINC00926	IFI27
286		ITGA2B		CXorf57	GZMB
287		EREG		CCDC141	ATG2A
288		RP11-685N3.1		RAB27B	HELLS
289		GP9		SPON2	PELI1
290		NT5C3A		RP11-206L10.2	ABHD5
291		ZFHX3		COBLL1	AP3M2
292		CLU		CLIC3	CMIP
293		MYO15B		IL2RB	LGALS3
294		XCL1		LGALS1	ANKRD28
295		MIR181A1HG		PRF1	CD8A
296		CMTM5		GP9	MIR29A
297		DTHD1		INPP4B	PPIF
298		FCGR3A		CCL4L2	ZNF10
299		MYL9		MCOLN2	COTL1
300		HNRNPU-AS1		GZMB	RP11-291B21.2
301		HLA-DPB1		NPIPB4	CD9
302		PF4		ATF3	HIST1H2BK
303		C6orf25		KLRG1	PARP14
304		SDPR		METTL12	CTSW
305		CCL3		PPBP	CTD-2562J15.6
306		GNLY		GLUL	MIR181A1HG
307		FCAR		CD79A	DOCK5
308		AGFG1		SNX29P2	IER3
309		RP6-99M1.2		ITK	SESN3
310		ATF3		GZMA	IFRD1
311		RP11-325F22.2		GRAP2	CDKN1A
312		2-Mar		WARS	GZMA

313		PGRMC1		ZNF10	FRMD4B
314		RRBP1		ITM2C	HLA-DPB1
315		CCDC141		CMTM5	RP6-99M1.2
316		MXD1		STAG3	RASGEF1B
317		KLRD1		PAXBP1	BACH1
318		RBM47		PADI4	TGFBR3
319		XCL2		PRKAR1B	ATF5
320		GADD45A		AP3M2	HIST1H3A
321		JARID2		VCAN	ZNF683
322		SPARC		ITGA2B	KLRD1
323		GRASP		RP11-325F22.2	NR4A3
324		TNF		TSC22D1	ABCA1
325		KLRF1		TREML1	RP11-367G6.3
326		ENTPD1		MAP3K7CL	DAPK1
327		KIF2A		MLH3	RALGAPA1
328		PLD4		PARP15	ENTPD1
329		HIST1H2BK		SNORD3B-2	DTHD1
330		PRF1		GNG11	IRAK3
331		AHR		TREM1	RP11-350N15.5
332		HLA-DQA1		HNRNPU-AS1	CRY1
333		LDLRAD4		PF4	44257
334		ENC1		MMD	WARS
335		LRP1		HSPA1B	NFKBID
336		PARVB		RP6-99M1.2	TNF
337		CCR4		SPARC	TYMP
338		IL1B		SDPR	ID1
339		CRY1		PELI1	CDC14A
340		CD38		TUBB1	PRF1
341		GPR56		TIMP1	MIR24-2
342		RP11-367G6.3		PDLIM1	CD151
343		TNFRSF4		DAPK1	NABP1
344		FABP5		AGPAT9	S100B
345		CEBPD		PRKAR2B	RNU12
346		TYMP		COTL1	CCL4L2
347		OSM		ANKRD28	MT1E

348		TTN		TSC22D2	CCRN4L
349		TSHZ2		GPR56	CTD-2035E11.3
350		CCL4		EMR2	HBB
351		TBC1D8		RALGAPA1	GPX1
352		IFNG		AQP9	STMN1
353		HLA-DRB1		APOBEC3A	MT-ND6
354		GPX1		ISG15	NR4A1
355		CD163		SNX9	HLA-DRB1
356		MTRNR2L12		ITGAX	MTRNR2L12
357		C1QA		C15orf48	BCL2A1
358		NKG7		IFIT3	CD74
359		RP11-290D2.6		C1QA	S100A4
360		GLUL		SCO2	DUSP2
361		TGFBR3		EREG	NEAT1
362		TPM4		HSP90B1	S100A6
363		NLRP3		MIR24-2	SPON2
364		PPIF		JUN	GZMH
365		GZMB		SPAG9	CST7
366		CCR6		PID1	FGFBP2
367		RBP7		IFIT2	CMC1
368		IL1RN		IFI30	NKG7
369		KLRB1		PHACTR1	
370		TSPO		MTRNR2L12	
371		CTSW		FTL	
372		LGALS1		PLBD1	
373		PLAUR		HES4	
374		PLXDC2		RBP7	
375		CDA		CD14	
376		GZMH		MT-CO3	
377		THBD		SLC43A2	
378		TMEM176A		AQP3	
379		CST7		CD300E	
380		LGALS2		AIF1	
381		FGL2		MARCKS	
382		HBEGF		C5AR1	

383		LGALS3		FCER1G		
384		GSN		NAMPT		
385		KLF4		OAZ1		
386		S100A4		FPR1		
387		FPR1		FCN1		
388		RNASE2		CFD		
389		TMEM176B		MT-CO2		
390		CD14		NEAT1		
391		AIF1		CSF3R		
392		RP11-290F20.3		SLC11A1		
393		CPVL		S100A6		
394		CYBB		MT-CYB		
395		MNDA		APLP2		
396		HMOX1		CD68		
397		PLBD1		S100A11		
398		LILRA3		TSPO		
399		PID1		MT-CO1		
400		LILRA5		CSTA		
401		LILRB2		S100A4		
