# Supplementary material for: Recognition of Immune Cell Markers of COVID-19 Severity with Machine Learning Methods
Source: Biomed Res Int. 2022 Apr 28;2022:6089242. doi: 10.1155/2022/6089242 (PMC9073549; doi:10.1155/2022/6089242)
Supplement: Supplementary 2 — Table S2: performance of decision tree and random forest with different number of features in each cell type. [file 6089242.f2.pdf]

Table S2. Performance of decision tree and random forest with different number of features in each cell type

(1) Decision tree

Number of features	B cell	CD4+ T cell	CD8+ T cell	Monocytes	NK cell
5	0.680803	0.569116	0.585744	0.702642	0.622301
10	0.700318	0.61592	0.611947	0.749962	0.672408
15	0.711142	0.627224	0.6222	0.78421	0.677255
20	0.716253	0.626027	0.633359	0.78705	0.686669
25	0.714056	0.634084	0.629516	0.793018	0.686215
30	0.723342	0.633014	0.629486	0.796121	0.693015
35	0.731441	0.634838	0.629427	0.804753	0.690196
40	0.73402	0.634694	0.637485	0.803432	0.691302
45	0.737045	0.634909	0.631028	0.803176	0.689151
50	0.738222	0.633481	0.632652	0.803553	0.693561
55	0.736997	0.63287	0.629408	0.80329	0.694659
60	0.731616	0.633687	0.636311	0.802146	0.69669
65	0.734796	0.633931	0.629178	0.802541	0.692418
70	0.731838	0.636144	0.633818	0.799594	0.693975
75	0.736554	0.637031	0.63077	0.805518	0.695613
80	0.733498	0.63355	0.630138	0.804304	0.693662
85	0.738603	0.633673	0.634008	0.80368	0.691311
90	0.737403	0.630595	0.634243	0.805449	0.689352
95	0.738884	0.63518	0.634735	0.803776	0.693341
100	0.730269	0.633056	0.631894	0.800604	0.693465
105	0.734954	0.630341	0.635608	0.801789	0.695749
110	0.735952	0.633843	0.63005	0.799475	0.693805
115	0.733464	0.631336	0.630622	0.800976	0.694138
120	0.729462	0.633422	0.631737	0.800742	0.687178
125	0.733225	0.62968	0.631605	0.80086	0.688562
130	0.727991	0.634459	0.632779	0.799177	0.691048
135	0.730887	0.633441	0.630313	0.802351	0.693375
140	0.728048	0.629359	0.632427	0.800978	0.69229
145	0.729256	0.632136	0.630301	0.798035	0.689781
150	0.731774	0.630857	0.631228	0.797978	0.68852
155	0.734119	0.62854	0.631113	0.794315	0.689579
160	0.739055	0.629451	0.632591	0.794694	0.68791
165	0.740066	0.629674	0.629875	0.795805	0.688444
170	0.739469	0.631908	0.630912	0.796247	0.687493
175	0.736988	0.631509	0.629441	0.79489	0.689485
180	0.736376	0.631795	0.633561	0.796123	0.692166
185	0.734112	0.629476	0.633272	0.796387	0.685082

190	0.734475	0.628273	0.629152	0.794383	0.687572
195	0.732549	0.630804	0.634778	0.794921	0.688657
200	0.733112	0.628675	0.63021	0.793285	0.689356
205	0.732708	0.629928	0.628431	0.795083	0.687323
210	0.737932	0.629699	0.634891	0.796308	0.692887
215	0.737294	0.627691	0.626498	0.79582	0.685046
220	0.735226	0.630956	0.62621	0.792842	0.689509
225	0.735741	0.62663	0.630788	0.796381	0.686893
230		0.631153	0.631462	0.79402	0.690129
235		0.629888	0.634754	0.795381	0.689689
240		0.629893	0.629127	0.794566	0.69015
245		0.632689	0.627591	0.793619	0.689132
250		0.63087	0.626913	0.794398	0.6909
255		0.631365	0.628036	0.794235	0.690405
260		0.628206	0.632463	0.795912	0.689829
265		0.630831		0.79411	0.689335
270		0.629484		0.794248	0.691225
275		0.628935		0.796403	0.684734
280		0.63078		0.794355	0.689701
285		0.629917		0.797692	0.690232
290		0.628958		0.796036	0.685239
295		0.629696		0.794876	0.689853
300		0.629408		0.794096	0.688639
305		0.628573		0.794922	0.688871
310		0.628559		0.794522	0.689192
315		0.630636		0.795596	0.686618
320		0.629004		0.793312	0.690079
325		0.629227		0.793037	0.683419
330		0.629097		0.79344	0.68443
335		0.627813		0.79282	0.684917
340		0.629392		0.795072	0.689792
345		0.629691		0.793568	0.687749
350		0.627882		0.796711	0.691997
355		0.629409		0.792616	0.689842
360		0.629496		0.7935	0.690124
365		0.63003		0.7916	0.689886
370		0.628541		0.791641	
375		0.629793		0.792996	
380		0.631471		0.791635	
385		0.626914		0.793526	
390		0.629032		0.792453	
395		0.629432		0.792951	

400		0.629692		0.792768	

(2) Random forest

Number of features	B cell	CD4+ T cell	CD8+ T cell	Monocytes	NK cell
5	0.74692	0.610042	0.619077	0.734757	0.642614
10	0.784086	0.698768	0.697206	0.815384	0.736757
15	0.815868	0.729346	0.708405	0.86538	0.761443
20	0.829299	0.737596	0.726108	0.876008	0.787072
25	0.834942	0.74624	0.730517	0.881172	0.799584
30	0.846267	0.752895	0.737181	0.890816	0.808852
35	0.852537	0.757334	0.741834	0.901413	0.8121
40	0.860275	0.758153	0.753404	0.907378	0.814975
45	0.86692	0.760244	0.754376	0.907623	0.818274
50	0.86553	0.760207	0.75231	0.910169	0.817757
55	0.867021	0.760262	0.756968	0.911358	0.824319
60	0.866226	0.757911	0.756635	0.910837	0.827853
65	0.865994	0.761165	0.755928	0.911256	0.830255
70	0.865739	0.762405	0.757526	0.910584	0.83027
75	0.866906	0.761969	0.759963	0.913301	0.828474
80	0.866988	0.76138	0.757913	0.912296	0.825992
85	0.867794	0.760535	0.760607	0.912227	0.82797
90	0.866779	0.759325	0.762774	0.911828	0.828129
95	0.865582	0.759699	0.761858	0.911743	0.830825
100	0.865579	0.759795	0.761978	0.912286	0.831536
105	0.865343	0.759981	0.763762	0.91176	0.831789
110	0.862276	0.75949	0.761658	0.911498	0.831358
115	0.861607	0.758956	0.76188	0.911289	0.830161
120	0.861482	0.757758	0.760072	0.911768	0.833014
125	0.862572	0.758079	0.762939	0.91324	0.830506
130	0.861265	0.758421	0.761662	0.913326	0.831803
135	0.858482	0.75799	0.761981	0.912094	0.834421
140	0.859405	0.756549	0.762171	0.911628	0.832206
145	0.857369	0.755226	0.761684	0.911807	0.833187
150	0.856428	0.757613	0.762923	0.910322	0.831643
155	0.863237	0.754261	0.761368	0.910545	0.830688
160	0.859301	0.754675	0.758557	0.90973	0.831601
165	0.862301	0.75354	0.761562	0.908859	0.829045
170	0.860649	0.755631	0.761714	0.910904	0.830479
175	0.861898	0.752915	0.760785	0.90934	0.831912
180	0.862072	0.75429	0.76269	0.908957	0.827474
185	0.859451	0.751256	0.763244	0.905331	0.831644
190	0.857237	0.752003	0.763711	0.907843	0.830056

195	0.859421	0.753223	0.761479	0.907547	0.826685
200	0.859744	0.752649	0.763481	0.906391	0.830307
205	0.857089	0.752716	0.760061	0.906794	0.829789
210	0.858179	0.751644	0.760607	0.908061	0.828757
215	0.857924	0.751993	0.761001	0.907964	0.832444
220	0.856895	0.751754	0.759808	0.90716	0.829854
225	0.857626	0.752137	0.76072	0.907107	0.830985
230		0.749871	0.760674	0.906544	0.831384
235		0.75107	0.761342	0.906874	0.832873
240		0.749859	0.762428	0.905528	0.833173
245		0.750232	0.760053	0.905514	0.832063
250		0.751033	0.761157	0.905095	0.832558
255		0.747631	0.762562	0.904842	0.835338
260		0.74918	0.765916	0.903803	0.832572
265		0.750529		0.904693	0.831526
270		0.749667		0.903784	0.835496
275		0.748697		0.90409	0.83544
280		0.748103		0.903562	0.834842
285		0.747649		0.902213	0.832689
290		0.747851		0.902247	0.834198
295		0.748135		0.9021	0.835195
300		0.746905		0.90247	0.833176
305		0.74633		0.903335	0.834136
310		0.747071		0.901533	0.837132
315		0.746157		0.902286	0.837094
320		0.746329		0.901221	0.833474
325		0.746979		0.902922	0.836357
330		0.746582		0.903009	0.835709
335		0.744351		0.902018	0.833239
340		0.745353		0.901575	0.834983
345		0.745384		0.900518	0.832329
350		0.744956		0.900881	0.832391
355		0.745034		0.899467	0.833513
360		0.74604		0.90059	0.832867
365		0.744865		0.900251	0.834382
370		0.74489		0.898828	
375		0.745985		0.899558	
380		0.746233		0.898084	
385		0.745986		0.899004	
390		0.745989		0.897446	
395		0.747076		0.897724	
400		0.745711		0.899076	
