# Supplementary material for: Recognition of Immune Cell Markers of COVID-19 Severity with Machine Learning Methods
Source: Biomed Res Int. 2022 Apr 28;2022:6089242. doi: 10.1155/2022/6089242 (PMC9073549; doi:10.1155/2022/6089242)
Supplement: Supplementary 4 — Table S4: detailed results of GO and KEGG enrichment analyses in different optimal gene sets. [file 6089242.f4.pdf]

Table S4. Detailed results of GO and KEGG enrichment analysis in different optimal gene sets

(1) B cell

ONTOLOGY	ID	Description	GeneRatio	BgRatio	pvalue	p.adjust	qvalue	geneID	Count
BP	GO:1903131	mononuclear cell differentiation	14/73	411/18862	5.01E-10	3.46E-07	2.66E-07	974/84807/931/973/1235/3725/639/3394/64332/6688/9308/3662/1958/3123	14
BP	GO:0050851	antigen receptor-mediated signaling pathway	13/73	323/18862	2.94E-10	3.04E-07	2.34E-07	974/84807/931/3113/4208/3115/3127/3119/973/3117/100423062/64332/3123	13
BP	GO:0002429	immune response-activating cell surface receptor signaling pathway	13/73	481/18862	3.51E-08	1.04E-05	7.99E-06	974/84807/931/3113/4208/3115/3127/3119/973/3117/100423062/64332/3123	13
BP	GO:0002757	immune response-activating signal transduction	13/73	481/18862	3.51E-08	1.04E-05	7.99E-06	974/84807/931/3113/4208/3115/3127/3119/973/3117/100423062/64332/3123	13
BP	GO:0030098	lymphocyte differentiation	12/73	358/18862	1.18E-08	6.11E-06	4.71E-06	974/84807/931/973/1235/639/64332/6688/9308/3662/1958/3123	12
BP	GO:0022407	regulation of cell-cell adhesion	12/73	437/18862	1.05E-07	2.42E-05	1.87E-05	84807/3113/3115/8728/57162/83706/3383/7124/64332/9308/28984/3123	12
BP	GO:0042110	T cell activation	12/73	474/18862	2.53E-07	4.36E-05	3.36E-05	84807/3113/3115/57162/1235/3383/639/64332/9308/3662/1958/3123	12
BP	GO:0002460	adaptive immune response based on somatic recombination of immune receptors	11/73	367/18862	1.58E-07	3.26E-05	2.51E-05	84807/4208/2208/3119/1235/3383/7124/100423062/64332/3662/3123	11

		built from immunoglobulin superfamily domains							
BP	GO:0001819	positive regulation of cytokine production	11/73	437/18862	8.90E-07	0.0001416	0.00010906	4050/3113/3115/57162/3394/7124/351/9308/28984/3662/1958	11
BP	GO:0007159	leukocyte cell-cell adhesion	10/73	366/18862	1.40E-06	0.0001814	0.0001397	84807/3113/3115/57162/83706/3383/7124/64332/9308/3123	10
BP	GO:0006959	humoral immune response	10/73	380/18862	1.97E-06	0.0002033	0.00015656	931/4208/2208/3119/1235/7124/100423062/9308/28984/3123	10
BP	GO:1903706	regulation of hemopoiesis	10/73	415/18862	4.31E-06	0.000307	0.00023645	84807/4208/3725/639/7124/64332/6688/9308/3662/3123	10
BP	GO:0045785	positive regulation of cell adhesion	10/73	425/18862	5.31E-06	0.0003329	0.00025635	84807/3113/3115/8728/3383/3611/7124/64332/9308/3123	10
BP	GO:0060333	interferon-gamma-mediated signaling pathway	9/73	91/18862	7.12E-11	1.47E-07	1.13E-07	3113/3115/3127/3119/3383/3117/3394/3662/3123	9
BP	GO:0071346	cellular response to interferon-gamma	9/73	177/18862	2.67E-08	1.04E-05	7.99E-06	3113/3115/3127/3119/3383/3117/3394/3662/3123	9
BP	GO:0034341	response to interferon-gamma	9/73	197/18862	6.72E-08	1.74E-05	1.34E-05	3113/3115/3127/3119/3383/3117/3394/3662/3123	9
BP	GO:0022409	positive regulation of cell-cell adhesion	9/73	276/18862	1.16E-06	0.0001656	0.00012755	84807/3113/3115/8728/3383/7124/64332/9308/3123	9

BP	GO:0050863	regulation of T cell activation	9/73	327/18862	4.64E-06	0.00032	0.00024647	84807/3113/3115/57162/639/64332/9308/3662/3123	9
BP	GO:1903037	regulation of leukocyte cell-cell adhesion	9/73	330/18862	5.00E-06	0.0003231	0.00024881	84807/3113/3115/57162/3383/7124/64332/9308/3123	9
BP	GO:0051251	positive regulation of lymphocyte activation	9/73	356/18862	9.21E-06	0.0005011	0.00038589	84807/3113/4208/3115/57162/100423062/64332/9308/3123	9
BP	GO:0002696	positive regulation of leukocyte activation	9/73	401/18862	2.37E-05	0.0010641	0.0008195	84807/3113/4208/3115/57162/100423062/64332/9308/3123	9
BP	GO:0050867	positive regulation of cell activation	9/73	412/18862	2.93E-05	0.0012016	0.0009254	84807/3113/4208/3115/57162/100423062/64332/9308/3123	9
BP	GO:0050852	T cell receptor signaling pathway	8/73	204/18862	1.20E-06	0.0001656	0.00012755	84807/3113/3115/3127/3119/3117/64332/3123	8
BP	GO:0019882	antigen processing and presentation	8/73	234/18862	3.34E-06	0.000256	0.00019718	3113/3115/3127/3119/3383/3117/1536/3123	8
BP	GO:1903039	positive regulation of leukocyte cell-cell adhesion	8/73	234/18862	3.34E-06	0.000256	0.00019718	84807/3113/3115/3383/7124/64332/9308/3123	8
BP	GO:0030217	T cell differentiation	8/73	246/18862	4.84E-06	0.0003226	0.00024842	84807/1235/639/64332/9308/3662/1958/3123	8
BP	GO:1902105	regulation of leukocyte differentiation	8/73	279/18862	1.21E-05	0.0006269	0.00048278	84807/3725/639/7124/64332/9308/3662/3123	8

BP	GO:0030099	myeloid cell differentiation	8/73	419/18862	0.000211082	0.0052593	0.00405032	4208/3725/3394/7124/351/6688/3662/3123	8
BP	GO:0007015	actin filament organization	8/73	435/18862	0.000271879	0.0063174	0.0048652	51429/9124/3925/3383/7184/4082/28984/7273	8
BP	GO:0002478	antigen processing and presentation of exogenous peptide antigen	7/73	177/18862	5.50E-06	0.0003347	0.00025778	3113/3115/3127/3119/3117/1536/3123	7
BP	GO:0019884	antigen processing and presentation of exogenous antigen	7/73	185/18862	7.36E-06	0.0004111	0.00031663	3113/3115/3127/3119/3117/1536/3123	7
BP	GO:0048002	antigen processing and presentation of peptide antigen	7/73	194/18862	1.00E-05	0.000532	0.0004097	3113/3115/3127/3119/3117/1536/3123	7
BP	GO:0046651	lymphocyte proliferation	7/73	282/18862	0.000108748	0.0033471	0.00257769	931/3113/4208/3115/57162/973/3123	7
BP	GO:0032943	mononuclear cell proliferation	7/73	285/18862	0.000116131	0.0034546	0.00266047	931/3113/4208/3115/57162/973/3123	7
BP	GO:0070661	leukocyte proliferation	7/73	312/18862	0.000202781	0.005114	0.00393849	931/3113/4208/3115/57162/973/3123	7
BP	GO:0042113	B cell activation	7/73	326/18862	0.000264931	0.0062259	0.00479475	974/931/4208/57162/973/1235/100423062	7
BP	GO:0001666	response to hypoxia	7/73	348/18862	0.000392846	0.0082061	0.00631978	9124/571/3383/7184/1536/28984/1958	7
BP	GO:0010038	response to metal ion	7/73	352/18862	0.000420681	0.0086997	0.0066999	4208/3383/3725/4205/1536/351/7273	7

BP	GO:0002449	lymphocyte mediated immunity	7/73	360/18862	0.000481091	0.0097539	0.00751177	2208/3119/1235/3383/7124/100423062/3123	7
BP	GO:0036293	response to decreased oxygen levels	7/73	360/18862	0.000481091	0.0097539	0.00751177	9124/571/3383/7184/1536/28984/1958	7
BP	GO:0070482	response to oxygen levels	7/73	385/18862	0.000715808	0.0135807	0.01045889	9124/571/3383/7184/1536/28984/1958	7
BP	GO:0034329	cell junction assembly	7/73	425/18862	0.001271898	0.0205491	0.01582551	4208/5339/7094/3611/7124/7414/351	7
BP	GO:0006979	response to oxidative stress	7/73	444/18862	0.001633546	0.0235437	0.01813173	9124/94241/143686/3725/1536/351/2876	7
BP	GO:0009896	positive regulation of catabolic process	7/73	450/18862	0.001763017	0.0243403	0.0187452	51429/4946/94241/143686/255488/7124/351	7
BP	GO:0002697	regulation of immune effector process	7/73	465/18862	0.002121819	0.0266209	0.02050159	84807/2208/3383/7124/64332/3662/3123	7
BP	GO:0001667	ameboidal-type cell migration	7/73	473/18862	0.00233504	0.0284051	0.02187564	4208/9124/3164/1235/7124/2876/28984	7
BP	GO:0046637	regulation of alpha-beta T cell differentiation	6/73	67/18862	2.26E-07	4.24E-05	3.27E-05	84807/639/64332/9308/3662/3123	6
BP	GO:0060337	type I interferon signaling pathway	6/73	95/18862	1.80E-06	0.0002033	0.00015656	4599/3394/3437/54739/3662/1958	6

BP	GO:0071357	cellular response to type I interferon	6/73	96/18862	1.92E-06	0.0002033	0.00015656	4599/3394/3437/54739/3662/1958	6
BP	GO:0019886	antigen processing and presentation of exogenous peptide antigen via MHC class II	6/73	99/18862	2.30E-06	0.0002264	0.00017435	3113/3115/3127/3119/3117/3123	6
BP	GO:0034340	response to type I interferon	6/73	101/18862	2.58E-06	0.0002324	0.00017895	4599/3394/3437/54739/3662/1958	6
BP	GO:0046634	regulation of alpha-beta T cell activation	6/73	101/18862	2.58E-06	0.0002324	0.00017895	84807/639/64332/9308/3662/3123	6
BP	GO:0002495	antigen processing and presentation of peptide antigen via MHC class II	6/73	103/18862	2.90E-06	0.0002497	0.00019231	3113/3115/3127/3119/3117/3123	6
BP	GO:0002504	antigen processing and presentation of peptide or polysaccharide antigen via MHC class II	6/73	104/18862	3.07E-06	0.0002536	0.00019532	3113/3115/3127/3119/3117/3123	6
BP	GO:0046632	alpha-beta T cell differentiation	6/73	107/18862	3.62E-06	0.0002673	0.00020582	84807/639/64332/9308/3662/3123	6
BP	GO:0045580	regulation of T cell differentiation	6/73	146/18862	2.16E-05	0.0009906	0.00076292	84807/639/64332/9308/3662/3123	6

BP	GO:0046631	alpha-beta T cell activation	6/73	149/18862	2.42E-05	0.0010641	0.0008195	84807/639/64332/9308/3662/3123	6
BP	GO:1902107	positive regulation of leukocyte differentiation	6/73	154/18862	2.91E-05	0.0012016	0.0009254	84807/3725/7124/64332/9308/3123	6
BP	GO:1903708	positive regulation of hemopoiesis	6/73	154/18862	2.91E-05	0.0012016	0.0009254	84807/3725/7124/64332/9308/3123	6
BP	GO:0002822	regulation of adaptive immune response based on somatic recombination of immune receptors built from immunoglobulin superfamily domains	6/73	155/18862	3.02E-05	0.0012016	0.0009254	84807/4208/2208/7124/64332/3123	6
BP	GO:0002819	regulation of adaptive immune response	6/73	170/18862	5.07E-05	0.0018073	0.00139186	84807/4208/2208/7124/64332/3123	6
BP	GO:0045619	regulation of lymphocyte differentiation	6/73	175/18862	5.96E-05	0.0020192	0.00155506	84807/639/64332/9308/3662/3123	6
BP	GO:0002285	lymphocyte activation	6/73	189/18862	9.12E-05	0.0029459	0.00226871	84807/1235/3383/64332/3662/3123	6

		involved in immune response							
BP	GO:0002573	myeloid leukocyte differentiation	6/73	204/18862	0.000138563	0.0037214	0.00286598	3725/7124/351/6688/3662/3123	6
BP	GO:0050870	positive regulation of T cell activation	6/73	212/18862	0.000170828	0.0044823	0.00345197	84807/3113/3115/64332/9308/3123	6
BP	GO:0016064	immunoglobulin mediated immune response	6/73	223/18862	0.000224601	0.0055295	0.00425842	2208/3119/1235/7124/100423062/3123	6
BP	GO:0019724	B cell mediated immunity	6/73	226/18862	0.000241364	0.0058723	0.00452241	2208/3119/1235/7124/100423062/3123	6
BP	GO:0007517	muscle organ development	6/73	317/18862	0.001422295	0.0226254	0.01742455	4208/4205/2876/467/1958/7273	6
BP	GO:0032496	response to lipopolysaccharide	6/73	326/18862	0.001639406	0.0235437	0.01813173	4208/57162/3383/3725/3394/7124	6
BP	GO:0002237	response to molecule of bacterial origin	6/73	346/18862	0.002211751	0.0272256	0.02096729	4208/57162/3383/3725/3394/7124	6
BP	GO:0014706	striated muscle tissue development	6/73	351/18862	0.002375905	0.0287332	0.02212831	4208/4205/2876/467/1958/7273	6
BP	GO:0010631	epithelial cell migration	6/73	357/18862	0.002584849	0.030372	0.02339041	4208/3164/1235/7124/2876/28984	6
BP	GO:0009615	response to virus	6/73	359/18862	0.002657483	0.0307843	0.02370796	10964/3925/4599/7124/3437/51251	6

BP	GO:0090132	epithelium migration	6/73	360/18862	0.002694371	0.0307843	0.02370796	4208/3164/1235/7124/2876/28984	6
BP	GO:0090130	tissue migration	6/73	365/18862	0.002884622	0.0327769	0.02524253	4208/3164/1235/7124/2876/28984	6
BP	GO:1902903	regulation of supramolecular fiber organization	6/73	370/18862	0.003084793	0.0341142	0.0262724	51429/3925/3383/351/2876/28984	6
BP	GO:0060537	muscle tissue development	6/73	371/18862	0.003126045	0.0343865	0.02648212	4208/4205/2876/467/1958/7273	6
BP	GO:0031331	positive regulation of cellular catabolic process	6/73	384/18862	0.003700757	0.0386524	0.02976739	51429/94241/143686/255488/7124/351	6
BP	GO:0043370	regulation of CD4-positive, alpha-beta T cell differentiation	5/73	51/18862	1.55E-06	0.000188	0.00014476	84807/64332/9308/3662/3123	5
BP	GO:2000514	regulation of CD4-positive, alpha-beta T cell activation	5/73	67/18862	6.05E-06	0.0003577	0.00027551	84807/64332/9308/3662/3123	5
BP	GO:0043367	CD4-positive, alpha-beta T cell differentiation	5/73	81/18862	1.54E-05	0.0007407	0.00057046	84807/64332/9308/3662/3123	5
BP	GO:0002824	positive regulation of adaptive immune response based on somatic	5/73	99/18862	4.07E-05	0.00156	0.00120144	84807/2208/7124/64332/3123	5

		recombination of immune receptors built from immunoglobulin superfamily domains							
BP	GO:0035710	CD4-positive, alpha-beta T cell activation	5/73	100/18862	4.28E-05	0.0016075	0.00123797	84807/64332/9308/3662/3123	5
BP	GO:0002821	positive regulation of adaptive immune response	5/73	104/18862	5.16E-05	0.0018086	0.00139284	84807/2208/7124/64332/3123	5
BP	GO:0032649	regulation of interferon-gamma production	5/73	107/18862	5.91E-05	0.0020192	0.00155506	3113/3115/3394/7124/3123	5
BP	GO:0002286	T cell activation involved in immune response	5/73	111/18862	7.04E-05	0.0023488	0.00180889	84807/3383/64332/3662/3123	5
BP	GO:0032609	interferon-gamma production	5/73	112/18862	7.35E-05	0.0024123	0.00185779	3113/3115/3394/7124/3123	5
BP	GO:0002576	platelet degranulation	5/73	128/18862	0.000138149	0.0037214	0.00286598	83706/7094/7414/351/7273	5
BP	GO:0050853	B cell receptor signaling pathway	5/73	134/18862	0.000171229	0.0044823	0.00345197	974/931/4208/973/100423062	5
BP	GO:0051017	actin filament bundle assembly	5/73	154/18862	0.000326574	0.0071815	0.00553073	9124/3925/7184/4082/28984	5

BP	GO:2001236	regulation of extrinsic apoptotic signaling pathway	5/73	154/18862	0.000326574	0.0071815	0.00553073	3383/81618/7124/2876/467	5
BP	GO:0002455	humoral immune response mediated by circulating immunoglobulin	5/73	156/18862	0.000346556	0.0074654	0.00574933	2208/3119/7124/100423062/3123	5
BP	GO:0061572	actin filament bundle organization	5/73	158/18862	0.000367451	0.0078339	0.00603313	9124/3925/7184/4082/28984	5
BP	GO:0043534	blood vessel endothelial cell migration	5/73	175/18862	0.000585827	0.011538	0.00888578	4208/3164/7124/2876/28984	5
BP	GO:0010469	regulation of signaling receptor activity	5/73	188/18862	0.000809585	0.0148161	0.01141036	100462981/4208/100463486/7124/351	5
BP	GO:0071248	cellular response to metal ion	5/73	189/18862	0.000829107	0.0150046	0.01155551	4208/3725/4205/1536/351	5
BP	GO:0031032	actomyosin structure organization	5/73	191/18862	0.000869204	0.0152332	0.01173154	9124/3925/4205/28984/7273	5
BP	GO:0002377	immunoglobulin production	5/73	198/18862	0.00102101	0.0175954	0.01355078	29802/3119/1235/7124/3123	5
BP	GO:1902905	positive regulation of supramolecular fiber organization	5/73	203/18862	0.001140928	0.0191824	0.014773	51429/3383/351/2876/28984	5

BP	GO:0071456	cellular response to hypoxia	5/73	206/18862	0.001217718	0.0198287	0.01527068	571/3383/1536/28984/1958		5
BP	GO:0036294	cellular response to decreased oxygen levels	5/73	214/18862	0.001441204	0.0226651	0.01745513	571/3383/1536/28984/1958		5
BP	GO:0071241	cellular response to inorganic substance	5/73	216/18862	0.00150151	0.0226651	0.01745513	4208/3725/4205/1536/351		5
BP	GO:0097191	extrinsic apoptotic signaling pathway	5/73	217/18862	0.001532351	0.0229631	0.01768457	3383/81618/7124/2876/467		5
BP	GO:0002699	positive regulation of immune effector process	5/73	219/18862	0.001595432	0.0235437	0.01813173	84807/2208/7124/64332/3123		5
BP	GO:0050670	regulation of lymphocyte proliferation	5/73	221/18862	0.001660405	0.0236808	0.01823734	3113/4208/3115/57162/3123		5
BP	GO:0032944	regulation of mononuclear cell proliferation	5/73	223/18862	0.001727305	0.0241356	0.01858757	3113/4208/3115/57162/3123		5
BP	GO:0071453	cellular response to oxygen levels	5/73	231/18862	0.002014859	0.0255627	0.01968667	571/3383/1536/28984/1958		5
BP	GO:0070663	regulation of leukocyte proliferation	5/73	241/18862	0.002421795	0.028839	0.02220979	3113/4208/3115/57162/3123		5

BP	GO:0045637	regulation of myeloid cell differentiation	5/73	258/18862	0.003246396	0.0344284	0.02651442	4208/3725/7124/6688/3123	5
BP	GO:0043542	endothelial cell migration	5/73	278/18862	0.004456109	0.044092	0.03395665	4208/3164/7124/2876/28984	5
BP	GO:0002440	production of molecular mediator of immune response	5/73	286/18862	0.00501989	0.0487377	0.03753444	29802/3119/1235/7124/3123	5
BP	GO:0043372	positive regulation of CD4-positive, alpha-beta T cell differentiation	4/73	32/18862	6.84E-06	0.0003929	0.00030257	84807/64332/9308/3123	4
BP	GO:0045622	regulation of T-helper cell differentiation	4/73	39/18862	1.53E-05	0.0007407	0.00057046	84807/64332/3662/3123	4
BP	GO:2000516	positive regulation of CD4-positive, alpha-beta T cell activation	4/73	39/18862	1.53E-05	0.0007407	0.00057046	84807/64332/9308/3123	4
BP	GO:1902895	positive regulation of pri-miRNA transcription by RNA polymerase II	4/73	40/18862	1.70E-05	0.000798	0.0006146	3725/7124/6688/1958	4

BP	GO:0046638	positive regulation of alpha-beta T cell differentiation	4/73	49/18862	3.83E-05	0.0014961	0.00115221	84807/64332/9308/3123	4
BP	GO:1902893	regulation of pri-miRNA transcription by RNA polymerase II	4/73	51/18862	4.50E-05	0.0016604	0.00127869	3725/7124/6688/1958	4
BP	GO:0061614	pri-miRNA transcription by RNA polymerase II	4/73	52/18862	4.86E-05	0.001762	0.00135698	3725/7124/6688/1958	4
BP	GO:0002437	inflammatory response to antigenic stimulus	4/73	62/18862	9.72E-05	0.0030454	0.00234538	3383/7124/2876/3123	4
BP	GO:0070527	platelet aggregation	4/73	62/18862	9.72E-05	0.0030454	0.00234538	83706/7094/3611/7414	4
BP	GO:0042093	T-helper cell differentiation	4/73	64/18862	0.000110059	0.0033471	0.00257769	84807/64332/3662/3123	4
BP	GO:0032729	positive regulation of interferon-gamma production	4/73	65/18862	0.000116934	0.0034546	0.00266047	3113/3115/3394/7124	4
BP	GO:0001885	endothelial cell development	4/73	66/18862	0.000124116	0.003516	0.00270781	3383/7124/7414/2876	4
BP	GO:0002294	CD4-positive, alpha-beta T cell	4/73	66/18862	0.000124116	0.003516	0.00270781	84807/64332/3662/3123	4

		differentiation involved in immune response							
BP	GO:0046635	positive regulation of alpha-beta T cell activation	4/73	66/18862	0.000124116	0.003516	0.00270781	84807/64332/9308/3123	4
BP	GO:0002287	alpha-beta T cell activation involved in immune response	4/73	67/18862	0.000131612	0.003629	0.00279478	84807/64332/3662/3123	4
BP	GO:0002293	alpha-beta T cell differentiation involved in immune response	4/73	67/18862	0.000131612	0.003629	0.00279478	84807/64332/3662/3123	4
BP	GO:0002292	T cell differentiation involved in immune response	4/73	73/18862	0.000183652	0.0047474	0.00365613	84807/64332/3662/3123	4
BP	GO:0008625	extrinsic apoptotic signaling pathway via death domain receptors	4/73	84/18862	0.000315298	0.0070874	0.00545819	3383/7124/2876/467	4
BP	GO:0034109	homotypic cell-cell adhesion	4/73	85/18862	0.000329906	0.0071815	0.00553073	83706/7094/3611/7414	4

BP	GO:0045582	positive regulation of T cell differentiation	4/73	88/18862	0.00037664	0.0079479	0.0061209	84807/64332/9308/3123	4
BP	GO:0042100	B cell proliferation	4/73	96/18862	0.000524205	0.0105248	0.00810549	931/4208/57162/973	4
BP	GO:0045621	positive regulation of lymphocyte differentiation	4/73	101/18862	0.000634983	0.0123882	0.00954052	84807/64332/9308/3123	4
BP	GO:0043200	response to amino acid	4/73	102/18862	0.000658982	0.0126183	0.00971774	143686/3383/7124/1536	4
BP	GO:0043618	regulation of transcription from RNA polymerase II promoter in response to stress	4/73	109/18862	0.000845318	0.0150046	0.01155551	571/3725/467/1958	4
BP	GO:0043620	regulation of DNA-templated transcription in response to stress	4/73	115/18862	0.001032309	0.0176431	0.0135875	571/3725/467/1958	4
BP	GO:0001101	response to acid chemical	4/73	119/18862	0.001172031	0.0193901	0.0149329	143686/3383/7124/1536	4
BP	GO:0045446	endothelial cell differentiation	4/73	119/18862	0.001172031	0.0193901	0.0149329	3383/7124/7414/2876	4
BP	GO:0045471	response to ethanol	4/73	126/18862	0.001447606	0.0226651	0.01745513	94241/3383/1536/6688	4

BP	GO:0030968	endoplasmic reticulum unfolded protein response	4/73	127/18862	0.001490353	0.0226651	0.01745513	64061/7094/7184/467	4
BP	GO:0002687	positive regulation of leukocyte migration	4/73	133/18862	0.001765493	0.0243403	0.0187452	1235/3383/7124/351	4
BP	GO:0050671	positive regulation of lymphocyte proliferation	4/73	135/18862	0.00186454	0.0251144	0.01934141	3113/4208/3115/57162	4
BP	GO:0032946	positive regulation of mononuclear cell proliferation	4/73	136/18862	0.00191548	0.0251144	0.01934141	3113/4208/3115/57162	4
BP	GO:0003158	endothelium development	4/73	137/18862	0.001967378	0.0251144	0.01934141	3383/7124/7414/2876	4
BP	GO:0007519	skeletal muscle tissue development	4/73	147/18862	0.002541024	0.0300276	0.02312523	4208/2876/467/1958	4
BP	GO:0070665	positive regulation of leukocyte proliferation	4/73	148/18862	0.002604057	0.0304248	0.0234311	3113/4208/3115/57162	4
BP	GO:0034620	cellular response to unfolded protein	4/73	149/18862	0.002668157	0.0307843	0.02370796	64061/7094/7184/467	4
BP	GO:0030168	platelet activation	4/73	157/18862	0.003220439	0.0344284	0.02651442	83706/7094/3611/7414	4

BP	GO:0060538	skeletal muscle organ development	4/73	157/18862	0.003220439	0.0344284	0.02651442	4208/2876/467/1958	4
BP	GO:0035967	cellular response to topologically incorrect protein	4/73	168/18862	0.004100561	0.0417732	0.03217086	64061/7094/7184/467	4
BP	GO:0042129	regulation of T cell proliferation	4/73	168/18862	0.004100561	0.0417732	0.03217086	3113/3115/57162/3123	4
BP	GO:0001659	temperature homeostasis	4/73	171/18862	0.004366183	0.0434099	0.03343135	7124/2876/3662/1958	4
BP	GO:0001936	regulation of endothelial cell proliferation	4/73	177/18862	0.004931832	0.0485525	0.03739183	4208/3164/7124/28984	4
BP	GO:0071361	cellular response to ethanol	3/73	16/18862	3.00E-05	0.0012016	0.0009254	94241/1536/6688	3
BP	GO:0072539	T-helper 17 cell differentiation	3/73	29/18862	0.000189046	0.0048265	0.00371705	84807/64332/3662	3
BP	GO:0002861	regulation of inflammatory response to antigenic stimulus	3/73	32/18862	0.000254485	0.0060491	0.00465864	7124/2876/3123	3
BP	GO:0032673	regulation of interleukin-4 production	3/73	32/18862	0.000254485	0.0060491	0.00465864	9308/3662/3123	3
BP	GO:0032633	interleukin-4 production	3/73	33/18862	0.000279158	0.0063439	0.00488567	9308/3662/3123	3

BP	GO:0072538	T-helper 17 type immune response	3/73	33/18862	0.000279158	0.0063439	0.00488567	84807/64332/3662	3
BP	GO:0006509	membrane protein ectodomain proteolysis	3/73	42/18862	0.000572896	0.0113918	0.00877319	51429/8728/7124	3
BP	GO:0030225	macrophage differentiation	3/73	47/18862	0.000798057	0.0147355	0.01134831	351/6688/3123	3
BP	GO:0097028	dendritic cell differentiation	3/73	47/18862	0.000798057	0.0147355	0.01134831	3394/6688/3662	3
BP	GO:0061028	establishment of endothelial barrier	3/73	48/18862	0.000848906	0.0150046	0.01155551	3383/7124/7414	3
BP	GO:2001238	positive regulation of extrinsic apoptotic signaling pathway	3/73	48/18862	0.000848906	0.0150046	0.01155551	81618/7124/467	3
BP	GO:0002381	immunoglobulin production involved in immunoglobulin-mediated immune response	3/73	54/18862	0.001197354	0.0196518	0.01513447	3119/1235/3123	3
BP	GO:0032731	positive regulation of interleukin-1 beta production	3/73	56/18862	0.001330733	0.021333	0.01642921	7124/351/1958	3
BP	GO:0002763	positive regulation of myeloid	3/73	58/18862	0.00147311	0.0226651	0.01745513	3725/7124/3123	3

		leukocyte differentiation							
BP	GO:2000351	regulation of endothelial cell apoptotic process	3/73	58/18862	0.00147311	0.0226651	0.01745513	3383/7124/28984	3
BP	GO:1902041	regulation of extrinsic apoptotic signaling pathway via death domain receptors	3/73	59/18862	0.001547741	0.0230268	0.01773368	3383/2876/467	3
BP	GO:0032653	regulation of interleukin-10 production	3/73	60/18862	0.001624704	0.0235437	0.01813173	9308/3662/3123	3
BP	GO:0032722	positive regulation of chemokine production	3/73	60/18862	0.001624704	0.0235437	0.01813173	7124/351/1958	3
BP	GO:0033619	membrane protein proteolysis	3/73	61/18862	0.001704023	0.0239722	0.01846178	51429/8728/7124	3
BP	GO:0071230	cellular response to amino acid stimulus	3/73	61/18862	0.001704023	0.0239722	0.01846178	143686/7124/1536	3
BP	GO:0032613	interleukin-10 production	3/73	62/18862	0.001785727	0.0244562	0.01883447	9308/3662/3123	3
BP	GO:0071677	positive regulation of mononuclear cell migration	3/73	63/18862	0.001869839	0.0251144	0.01934141	1235/7124/351	3

BP	GO:0032732	positive regulation of interleukin-1 production	3/73	64/18862	0.001956385	0.0251144	0.01934141	7124/351/1958	3
BP	GO:0035914	skeletal muscle cell differentiation	3/73	64/18862	0.001956385	0.0251144	0.01934141	4208/467/1958	3
BP	GO:0072577	endothelial cell apoptotic process	3/73	64/18862	0.001956385	0.0251144	0.01934141	3383/7124/28984	3
BP	GO:0072678	T cell migration	3/73	66/18862	0.00213688	0.0266209	0.02050159	1235/3383/351	3
BP	GO:2000272	negative regulation of signaling receptor activity	3/73	66/18862	0.00213688	0.0266209	0.02050159	100462981/100463486/7124	3
BP	GO:1901224	positive regulation of NIK/NF-kappaB signaling	3/73	67/18862	0.002230877	0.0272985	0.02102347	3611/7124/351	3
BP	GO:0071229	cellular response to acid chemical	3/73	69/18862	0.002426489	0.028839	0.02220979	143686/7124/1536	3
BP	GO:0001937	negative regulation of endothelial cell proliferation	3/73	75/18862	0.003075894	0.0341142	0.0262724	4208/7124/28984	3
BP	GO:0043537	negative regulation of blood vessel endothelial cell migration	3/73	78/18862	0.003436862	0.0362624	0.02792681	4208/7124/28984	3

BP	GO:1904705	regulation of vascular associated smooth muscle cell proliferation	3/73	83/18862	0.004094081	0.0417732	0.03217086	4208/3725/7124	3
BP	GO:1990874	vascular associated smooth muscle cell proliferation	3/73	83/18862	0.004094081	0.0417732	0.03217086	4208/3725/7124	3
BP	GO:0032642	regulation of chemokine production	3/73	89/18862	0.00497734	0.0485525	0.03739183	7124/351/1958	3
BP	GO:0097306	cellular response to alcohol	3/73	89/18862	0.00497734	0.0485525	0.03739183	94241/1536/6688	3
BP	GO:2000318	positive regulation of T-helper 17 type immune response	2/73	10/18862	0.000651618	0.0125939	0.00969895	84807/64332	2
BP	GO:0045657	positive regulation of monocyte differentiation	2/73	11/18862	0.000794428	0.0147355	0.01134831	3725/3123	2
BP	GO:0055012	ventricular cardiac muscle cell differentiation	2/73	12/18862	0.000950927	0.0165254	0.01272669	4208/4205	2
BP	GO:0002923	regulation of humoral immune	2/73	13/18862	0.00112101	0.019002	0.01463406	2208/7124	2

		response mediated by circulating immunoglobulin							
BP	GO:0051044	positive regulation of membrane protein ectodomain proteolysis	2/73	15/18862	0.001501511	0.0226651	0.01745513	51429/7124	2
BP	GO:0033194	response to hydroperoxide	2/73	17/18862	0.0019351	0.0251144	0.01934141	94241/2876	2
BP	GO:0055003	cardiac myofibril assembly	2/73	17/18862	0.0019351	0.0251144	0.01934141	4205/7273	2
BP	GO:1901550	regulation of endothelial cell development	2/73	17/18862	0.0019351	0.0251144	0.01934141	7124/7414	2
BP	GO:1903140	regulation of establishment of endothelial barrier	2/73	17/18862	0.0019351	0.0251144	0.01934141	7124/7414	2
BP	GO:0002862	negative regulation of inflammatory response to antigenic stimulus	2/73	18/18862	0.002171547	0.0268908	0.02070943	2876/3123	2
BP	GO:2000319	regulation of T-helper 17 cell differentiation	2/73	19/18862	0.002420959	0.028839	0.02220979	84807/64332	2

BP	GO:0043011	myeloid dendritic cell differentiation	2/73	20/18862	0.002683235	0.0307843	0.02370796	6688/3662	2
BP	GO:0045655	regulation of monocyte differentiation	2/73	21/18862	0.002958275	0.0330687	0.02546726	3725/3123	2
BP	GO:1900118	negative regulation of execution phase of apoptosis	2/73	21/18862	0.002958275	0.0330687	0.02546726	100462981/100463486	2
BP	GO:2000047	regulation of cell-cell adhesion mediated by cadherin	2/73	21/18862	0.002958275	0.0330687	0.02546726	8728/28984	2
BP	GO:0002922	positive regulation of humoral immune response	2/73	22/18862	0.003245978	0.0344284	0.02651442	2208/7124	2
BP	GO:0045624	positive regulation of T-helper cell differentiation	2/73	22/18862	0.003245978	0.0344284	0.02651442	84807/64332	2
BP	GO:1904996	positive regulation of leukocyte adhesion to vascular endothelial cell	2/73	22/18862	0.003245978	0.0344284	0.02651442	3383/7124	2

BP	GO:2000316	regulation of T-helper 17 type immune response	2/73	22/18862	0.003245978	0.0344284	0.02651442	84807/64332	2
BP	GO:0051043	regulation of membrane protein ectodomain proteolysis	2/73	23/18862	0.003546246	0.0372266	0.02866936	51429/7124	2
BP	GO:0051000	positive regulation of nitric-oxide synthase activity	2/73	24/18862	0.00385898	0.0401024	0.03088409	2208/7124	2
BP	GO:0001562	response to protozoan	2/73	25/18862	0.00418408	0.0418004	0.03219178	3394/3662	2
BP	GO:0033622	integrin activation	2/73	25/18862	0.00418408	0.0418004	0.03219178	83706/7094	2
BP	GO:0042832	defense response to protozoan	2/73	25/18862	0.00418408	0.0418004	0.03219178	3394/3662	2
BP	GO:0048143	astrocyte activation	2/73	25/18862	0.00418408	0.0418004	0.03219178	7124/351	2
CC	GO:0009897	external side of plasma membrane	10/72	402/19520	2.11E-06	3.12E-05	2.14E-05	974/931/2208/973/1235/3383/7124/100423062/9308/3123	10
CC	GO:0005802	trans-Golgi network	9/72	251/19520	3.50E-07	6.11E-06	4.19E-06	51429/122618/3113/3115/3127/3119/3117/351/3123	9
CC	GO:0030139	endocytic vesicle	9/72	307/19520	1.87E-06	2.99E-05	2.05E-05	122618/3113/3115/3127/3119/3117/7184/1536/3123	9
CC	GO:0030055	cell-substrate junction	9/72	423/19520	2.46E-05	0.0002507	0.00017177	9124/83706/3383/5339/7094/3611/7184/7414/4082	9

CC	GO:0032588	trans-Golgi network membrane	8/72	95/19520	2.16E-09	8.30E-08	5.69E-08	122618/3113/3115/3127/3119/3117/351/3123	8
CC	GO:0030135	coated vesicle	8/72	295/19520	1.28E-05	0.0001447	9.91E-05	51429/3113/3115/3127/3119/3117/351/3123	8
CC	GO:0005925	focal adhesion	8/72	416/19520	0.000144591	0.001207	0.00082718	9124/3383/5339/7094/3611/7184/7414/4082	8
CC	GO:0030134	COPII-coated ER to Golgi transport vesicle	7/72	94/19520	5.47E-08	1.31E-06	8.99E-07	3113/3115/3127/3119/3117/351/3123	7
CC	GO:0030666	endocytic vesicle membrane	7/72	163/19520	2.32E-06	3.19E-05	2.18E-05	3113/3115/3127/3119/3117/1536/3123	7
CC	GO:0030136	clathrin-coated vesicle	7/72	192/19520	6.85E-06	8.22E-05	5.64E-05	51429/3113/3115/3127/3119/3117/3123	7
CC	GO:0005765	lysosomal membrane	7/72	378/19520	0.000482649	0.0037067	0.00254026	3113/3115/3127/3119/3117/81618/3123	7
CC	GO:0098852	lytic vacuole membrane	7/72	378/19520	0.000482649	0.0037067	0.00254026	3113/3115/3127/3119/3117/81618/3123	7
CC	GO:0030133	transport vesicle	7/72	402/19520	0.000694846	0.0051312	0.00351643	3113/3115/3127/3119/3117/351/3123	7
CC	GO:0005667	transcription regulator complex	7/72	409/19520	0.00076898	0.0054683	0.00374746	9124/3164/3725/22809/4205/6688/6925	7
CC	GO:0005774	vacuolar membrane	7/72	431/19520	0.001043624	0.0071563	0.00490425	3113/3115/3127/3119/3117/81618/3123	7
CC	GO:0042613	MHC class II protein complex	6/72	16/19520	1.58E-11	3.04E-09	2.08E-09	3113/3115/3127/3119/3117/3123	6
CC	GO:0042611	MHC protein complex	6/72	25/19520	3.41E-10	3.27E-08	2.24E-08	3113/3115/3127/3119/3117/3123	6

CC	GO:0071556	integral component of lumenal side of endoplasmic reticulum membrane	6/72	29/19520	9.04E-10	4.34E-08	2.97E-08	3113/3115/3127/3119/3117/3123	6
CC	GO:0098553	lumenal side of endoplasmic reticulum membrane	6/72	29/19520	9.04E-10	4.34E-08	2.97E-08	3113/3115/3127/3119/3117/3123	6
CC	GO:0098576	lumenal side of membrane	6/72	36/19520	3.63E-09	1.16E-07	7.97E-08	3113/3115/3127/3119/3117/3123	6
CC	GO:0030669	clathrin-coated endocytic vesicle membrane	6/72	39/19520	6.03E-09	1.65E-07	1.13E-07	3113/3115/3127/3119/3117/3123	6
CC	GO:0045334	clathrin-coated endocytic vesicle	6/72	57/19520	6.37E-08	1.36E-06	9.31E-07	3113/3115/3127/3119/3117/3123	6
CC	GO:0012507	ER to Golgi transport vesicle membrane	6/72	62/19520	1.06E-07	2.04E-06	1.40E-06	3113/3115/3127/3119/3117/3123	6
CC	GO:0030665	clathrin-coated vesicle membrane	6/72	117/19520	4.61E-06	5.90E-05	4.05E-05	3113/3115/3127/3119/3117/3123	6
CC	GO:0030176	integral component of endoplasmic	6/72	157/19520	2.48E-05	0.0002507	0.00017177	3113/3115/3127/3119/3117/3123	6

		reticulum membrane							
CC	GO:0031227	intrinsic component of endoplasmic reticulum membrane	6/72	165/19520	3.28E-05	0.000315	0.00021587	3113/3115/3127/3119/3117/3123	6
CC	GO:0030662	coated vesicle membrane	6/72	182/19520	5.67E-05	0.0005188	0.00035557	3113/3115/3127/3119/3117/3123	6
CC	GO:0030658	transport vesicle membrane	6/72	206/19520	0.000112418	0.0009811	0.00067236	3113/3115/3127/3119/3117/3123	6
CC	GO:0031301	integral component of organelle membrane	6/72	371/19520	0.002462742	0.0144724	0.00991801	3113/3115/3127/3119/3117/3123	6
CC	GO:0031300	intrinsic component of organelle membrane	6/72	402/19520	0.003657468	0.0200638	0.01374988	3113/3115/3127/3119/3117/3123	6
CC	GO:0030016	myofibril	5/72	224/19520	0.001426185	0.0094423	0.00647089	9124/5339/3611/7414/7273	5
CC	GO:0043292	contractile fiber	5/72	231/19520	0.001632933	0.0104508	0.00716199	9124/5339/3611/7414/7273	5
CC	GO:0045121	membrane raft	5/72	323/19520	0.006812806	0.0327015	0.02241055	931/973/3383/7124/351	5
CC	GO:0098857	membrane microdomain	5/72	323/19520	0.006812806	0.0327015	0.02241055	931/973/3383/7124/351	5
CC	GO:0032432	actin filament bundle	3/72	73/19520	0.002487437	0.0144724	0.00991801	9124/3611/4082	3

CC	GO:0016528	sarcoplasm	3/72	79/19520	0.003113173	0.0175803	0.01204788	4208/5339/7184	3
CC	GO:0000794	condensed nuclear chromosome	3/72	88/19520	0.004219212	0.0225025	0.0154211	10734/57162/7273	3
CC	GO:0031234	extrinsic component of cytoplasmic side of plasma membrane	3/72	100/19520	0.006024681	0.0304405	0.02086108	51429/2791/5996	3
CC	GO:0043034	costamere	2/72	18/19520	0.001975803	0.0122372	0.00838626	5339/7414	2
CC	GO:0002102	podosome	2/72	28/19520	0.004766548	0.0247345	0.01695074	83706/7414	2
CC	GO:0001772	immunological synapse	2/72	41/19520	0.010025946	0.0469508	0.03217569	3383/3123	2
MF	GO:0001228	DNA-binding transcription activator activity, RNA polymerase II-specific	11/71	443/18337	1.00E-06	5.48E-05	4.30E-05	4208/571/3164/3725/22809/4205/6688/6925/3662/467/1958	11
MF	GO:0001216	DNA-binding transcription activator activity	11/71	447/18337	1.10E-06	5.48E-05	4.30E-05	4208/571/3164/3725/22809/4205/6688/6925/3662/467/1958	11
MF	GO:0003823	antigen binding	7/71	165/18337	3.45E-06	0.0001379	0.00010813	3113/3115/3127/3119/3117/100423062/3123	7
MF	GO:0042277	peptide binding	7/71	315/18337	0.000214285	0.0061224	0.00480127	3113/3115/3127/3119/3117/81618/3123	7
MF	GO:0033218	amide binding	7/71	391/18337	0.000781612	0.0195403	0.01532372	3113/3115/3127/3119/3117/81618/3123	7
MF	GO:0042605	peptide antigen binding	6/71	32/18337	2.27E-09	4.55E-07	3.56E-07	3113/3115/3127/3119/3117/3123	6

MF	GO:0001227	DNA-binding transcription repressor activity, RNA polymerase II-specific	6/71	307/18337	0.001205584	0.0249265	0.01954765	571/3725/639/3394/6688/467	6
MF	GO:0001217	DNA-binding transcription repressor activity	6/71	309/18337	0.001246327	0.0249265	0.01954765	571/3725/639/3394/6688/467	6
MF	GO:0046982	protein heterodimerization activity	6/71	324/18337	0.001586556	0.0264426	0.02073656	4208/3164/4205/1536/6925/467	6
MF	GO:0140375	immune receptor activity	5/71	136/18337	0.000183286	0.0061095	0.00479117	3113/3119/1235/3117/3123	5
MF	GO:0032395	MHC class II receptor activity	4/71	10/18337	4.26E-08	4.26E-06	3.34E-06	3113/3119/3117/3123	4
MF	GO:0005178	integrin binding	4/71	142/18337	0.002241205	0.0320172	0.02510824	2208/83706/3383/7094	4
MF	GO:0046332	SMAD binding	3/71	77/18337	0.003314828	0.0404463	0.03171843	3725/4205/28984	3
MF	GO:0033613	activating transcription factor binding	3/71	78/18337	0.003437937	0.0404463	0.03171843	4208/3725/4205	3
MF	GO:0051371	muscle alpha-actinin binding	2/71	15/18337	0.00150229	0.0264426	0.02073656	9124/7273	2
MF	GO:0023026	MHC class II protein complex binding	2/71	17/18337	0.001936108	0.0297863	0.02335871	931/3123	2
MF	GO:0070412	R-SMAD binding	2/71	22/18337	0.003247682	0.0404463	0.03171843	3725/28984	2

MF	GO:0019865	immunoglobulin binding	2/71	24/18337	0.00386101	0.0429001	0.03364272	931/2208	2
MF	GO:0023023	MHC protein complex binding	2/71	26/18337	0.004523837	0.0464149	0.03639907	931/3123	2
MF	GO:0035035	histone acetyltransferase binding	2/71	27/18337	0.004873567	0.0464149	0.03639907	4205/1958	2
MF	GO:0051393	alpha-actinin binding	2/71	27/18337	0.004873567	0.0464149	0.03639907	9124/7273	2
KEGG	hsa05166	Human T-cell leukemia virus 1 infection	12/46	222/8096	2.30E-09	5.69E-08	4.26E-08	3113/3115/3127/3119/3383/3117/3725/7094/7124/6688/1958/3123	12
KEGG	hsa05323	Rheumatoid arthritis	10/46	93/8096	7.09E-11	1.11E-08	8.28E-09	4050/3113/3115/3127/3119/3383/3117/3725/7124/3123	10
KEGG	hsa05169	Epstein-Barr virus infection	10/46	202/8096	1.39E-07	1.81E-06	1.36E-06	3113/2208/3115/3127/3119/3383/3117/3725/7124/3123	10
KEGG	hsa05140	Leishmaniasis	9/46	77/8096	3.27E-10	1.70E-08	1.27E-08	3113/3115/3127/3119/3117/3725/7124/1536/3123	9
KEGG	hsa04640	Hematopoietic cell lineage	9/46	99/8096	3.21E-09	5.69E-08	4.26E-08	931/3113/2208/3115/3127/3119/3117/7124/3123	9
KEGG	hsa05164	Influenza A	9/46	172/8096	4.01E-07	4.17E-06	3.12E-06	3113/3115/3127/3119/3383/3117/4599/7124/3123	9
KEGG	hsa05321	Inflammatory bowel disease	8/46	65/8096	2.27E-09	5.69E-08	4.26E-08	3113/3115/3127/3119/3117/3725/7124/3123	8
KEGG	hsa04659	Th17 cell differentiation	8/46	108/8096	1.32E-07	1.81E-06	1.36E-06	3113/3115/3127/3119/3117/3725/3662/3123	8
KEGG	hsa05310	Asthma	7/46	31/8096	2.82E-10	1.70E-08	1.27E-08	3113/3115/3127/3119/3117/7124/3123	7
KEGG	hsa05330	Allograft rejection	7/46	38/8096	1.31E-09	5.12E-08	3.84E-08	3113/3115/3127/3119/3117/7124/3123	7

KEGG	hsa05332	Graft-versus-host disease	7/46	42/8096	2.76E-09	5.69E-08	4.26E-08	3113/3115/3127/3119/3117/7124/3123	7
KEGG	hsa04940	Type I diabetes mellitus	7/46	43/8096	3.28E-09	5.69E-08	4.26E-08	3113/3115/3127/3119/3117/7124/3123	7
KEGG	hsa05416	Viral myocarditis	7/46	60/8096	3.66E-08	5.71E-07	4.28E-07	3113/3115/3127/3119/3383/3117/3123	7
KEGG	hsa04612	Antigen processing and presentation	7/46	78/8096	2.32E-07	2.78E-06	2.09E-06	3113/3115/3127/3119/3117/7124/3123	7
KEGG	hsa04658	Th1 and Th2 cell differentiation	7/46	92/8096	7.25E-07	6.65E-06	4.98E-06	3113/3115/3127/3119/3117/3725/3123	7
KEGG	hsa05150	Staphylococcus aureus infection	7/46	96/8096	9.70E-07	8.41E-06	6.30E-06	3113/3115/3127/3119/3383/3117/3123	7
KEGG	hsa05145	Toxoplasmosis	7/46	112/8096	2.75E-06	2.26E-05	1.69E-05	3113/3115/3127/3119/3117/7124/3123	7
KEGG	hsa05322	Systemic lupus erythematosus	7/46	136/8096	1.00E-05	7.82E-05	5.85E-05	3113/3115/3127/3119/3117/7124/3123	7
KEGG	hsa04514	Cell adhesion molecules	7/46	149/8096	1.82E-05	0.0001353	0.00010137	3113/3115/3127/3119/3383/3117/3123	7
KEGG	hsa04145	Phagosome	7/46	152/8096	2.07E-05	0.0001471	0.00011016	3113/3115/3127/3119/3117/1536/3123	7
KEGG	hsa05152	Tuberculosis	7/46	180/8096	6.15E-05	0.000417	0.00031231	3113/3115/3127/3119/3117/7124/3123	7
KEGG	hsa04672	Intestinal immune network for IgA production	6/46	49/8096	2.80E-07	3.11E-06	2.33E-06	3113/3115/3127/3119/3117/3123	6
KEGG	hsa05320	Autoimmune thyroid disease	6/46	53/8096	4.51E-07	4.40E-06	3.29E-06	3113/3115/3127/3119/3117/3123	6

KEGG	hsa05418	Fluid shear stress and atherosclerosis	6/46	139/8096	0.000122408	0.0007957	0.00059593	4208/3383/3725/7124/7184/4205	6
KEGG	hsa04933	AGE-RAGE signaling pathway in diabetic complications	5/46	100/8096	0.000238572	0.0014887	0.00111501	3383/3725/7124/1536/1958	5
KEGG	hsa05417	Lipid and atherosclerosis	5/46	215/8096	0.007130383	0.0427823	0.03204342	3383/3725/7124/7184/1536	5
KEGG	hsa04371	Apelin signaling pathway	4/46	138/8096	0.007578037	0.0437842	0.03279384	4208/2791/4205/1958	4

(2) CD4+ T cell

**ONTOLOGY**	**ID**	**Description**	**GeneRatio**	**BgRatio**	**pvalue**	**p.adjust**	**qvalue**	**geneID**	**Count**
BP	GO:0022407	regulation of cell-cell adhesion	8/58	437/18862	5.41E-05	0.018879527	0.015417379	RGCC/PELI1/FERMT3/NFKBIZ/NR4A3/ANK3/GRAP2/ADAM19	8
BP	GO:0010038	response to metal ion	7/58	352/18862	9.86E-05	0.020679571	0.016887329	CASP8/AQP3/SOD2/MT2A/ANK3/JUN/MT1E	7
BP	GO:0045785	positive regulation of cell adhesion	7/58	425/18862	0.000313246	0.027621159	0.022555961	ILK/LIMS1/NFKBIZ/NR4A3/ANK3/GRAP2/ADAM19	7
BP	GO:0008637	apoptotic mitochondrial changes	5/58	118/18862	3.09E-05	0.017150043	0.014005049	CASP8/SOD2/IFI6/YWHAH/JUN	5
BP	GO:0046916	cellular transition metal ion homeostasis	5/58	118/18862	3.09E-05	0.017150043	0.014005049	SLC40A1/FTH1/FTL/MT2A/MT1E	5
BP	GO:0055076	transition metal ion homeostasis	5/58	139/18862	6.77E-05	0.01890209	0.015435804	SLC40A1/FTH1/FTL/MT2A/MT1E	5
BP	GO:0046686	response to cadmium ion	4/58	61/18862	3.69E-05	0.017150043	0.014005049	SOD2/MT2A/JUN/MT1E	4

BP	GO:1904705	regulation of vascular associated smooth muscle cell proliferation	4/58	83/18862	0.000123417	0.020679571	0.016887329	SOD2/NR4A3/HPGD/JUN	4
BP	GO:1990874	vascular associated smooth muscle cell proliferation	4/58	83/18862	0.000123417	0.020679571	0.016887329	SOD2/NR4A3/HPGD/JUN	4
BP	GO:0034109	homotypic cell-cell adhesion	4/58	85/18862	0.000135367	0.020679571	0.016887329	ILK/FERMT3/VCL/ANK3	4
BP	GO:0051899	membrane depolarization	4/58	87/18862	0.000148134	0.020679571	0.016887329	IFI6/ANK3/YWHAH/JUN	4
BP	GO:0060337	type I interferon signaling pathway	4/58	95/18862	0.000208033	0.023257664	0.018992648	IFI6/MX1/IFIT3/ISG15	4
BP	GO:0071357	cellular response to type I interferon	4/58	96/18862	0.000216583	0.023257664	0.018992648	IFI6/MX1/IFIT3/ISG15	4
BP	GO:0034340	response to type I interferon	4/58	101/18862	0.000263176	0.026242419	0.021430056	IFI6/MX1/IFIT3/ISG15	4
BP	GO:0007229	integrin-mediated signaling pathway	4/58	106/18862	0.000316575	0.027621159	0.022555961	ILK/FERMT3/LIMS1/ISG15	4
BP	GO:0071276	cellular response to cadmium ion	3/58	38/18862	0.00021563	0.023257664	0.018992648	MT2A/JUN/MT1E	3
BP	GO:1904707	positive regulation of vascular associated smooth muscle cell proliferation	3/58	47/18862	0.000406411	0.033373546	0.027253469	NR4A3/HPGD/JUN	3
CC	GO:0005911	cell-cell junction	7/59	485/19520	0.00062539	0.012568871	0.010135562	AQP3/LIMS1/ACTN1/VCL/ITK/ANK3/YWHAH	7
CC	GO:0016323	basolateral plasma membrane	6/59	211/19520	4.15E-05	0.003203237	0.002583097	SLC40A1/AQP3/SLC7A5/ANK3/LDLR/HPGD	6
CC	GO:0009925	basal plasma membrane	6/59	240/19520	8.48E-05	0.003625617	0.002923704	SLC40A1/AQP3/SLC7A5/ANK3/LDLR/HPGD	6
CC	GO:0045178	basal part of cell	6/59	258/19520	0.000126076	0.004311792	0.003477037	SLC40A1/AQP3/SLC7A5/ANK3/LDLR/HPGD	6
CC	GO:0030016	myofibril	5/59	224/19520	0.000575577	0.012568871	0.010135562	ILK/ACTN1/VCL/ANK3/MYOM2	5
CC	GO:0043292	contractile fiber	5/59	231/19520	0.00066152	0.012568871	0.010135562	ILK/ACTN1/VCL/ANK3/MYOM2	5
CC	GO:0034774	secretory granule lumen	5/59	322/19520	0.002861514	0.035342941	0.028500617	FERMT3/ACTN1/VCL/FTL/CTSA	5

CC	GO:0060205	cytoplasmic vesicle lumen	5/59	326/19520	0.003017161	0.035342941	0.028500617	FERMT3/ACTN1/VCL/FTL/CTSA	5
CC	GO:0031983	vesicle lumen	5/59	328/19520	0.003097244	0.035342941	0.028500617	FERMT3/ACTN1/VCL/FTL/CTSA	5
CC	GO:0014704	intercalated disc	4/59	48/19520	1.33E-05	0.002267614	0.001828608	ACTN1/VCL/ANK3/YWHAH	4
CC	GO:0044291	cell-cell contact zone	4/59	69/19520	5.62E-05	0.003203237	0.002583097	ACTN1/VCL/ANK3/YWHAH	4
CC	GO:0030017	sarcomere	4/59	203/19520	0.003306942	0.035342941	0.028500617	ILK/ACTN1/ANK3/MYOM2	4
CC	GO:0044754	autolysosome	2/59	10/19520	0.000397914	0.011340547	0.009145039	FTH1/FTL	2
CC	GO:0005767	secondary lysosome	2/59	16/19520	0.001048795	0.017934398	0.014462334	FTH1/FTL	2
CC	GO:0043034	costamere	2/59	18/19520	0.001332028	0.020706975	0.016698145	VCL/ANK3	2
CC	GO:0002102	podosome	2/59	28/19520	0.003227646	0.035342941	0.028500617	FERMT3/VCL	2
KEGG	hsa04978	Mineral absorption	5/40	60/8096	1.02E-05	0.00129256	0.001221316	30061/2495/2512/4502/4493	5

(3) CD8+ T cell

ONTOLOGY	ID	Description	GeneRatio	BgRatio	pvalue	p.adjust	qvalue	geneID	Count
BP	GO:0042110	T cell activation	24/225	474/18862	2.72E-09	2.14E-06	1.59E-06	639/3702/84174/841/6375/3821/57162/64332/3662/1493/3115/3553/7293/925/11326/1958/84807/257101/972/3458/3113/926/3123/3956	24
BP	GO:0002697	regulation of immune effector process	22/225	465/18862	4.34E-08	1.24E-05	9.22E-06	8140/7128/6375/8013/3821/64332/3662/117157/3553/51237/7293/11326/2208/84807/712/257101/11213/3824/7124/972/3458/3123	22
BP	GO:0045785	positive regulation of cell adhesion	21/225	425/18862	4.29E-08	1.24E-05	9.22E-06	3611/6375/8013/288/64332/1493/8728/7168/80005/3115/3553/3685/463/84807/3678/7124/972/3458/3113/3123/3956	21
BP	GO:0007159	leukocyte cell-cell adhesion	20/225	366/18862	1.74E-08	7.76E-06	5.76E-06	1524/83706/6375/8013/57162/64332/1493/3115/3553/11326/6279/84807/3678/6280/7124/972/3458/3113/3123/3956	20
BP	GO:0001819	positive regulation of cytokine production	20/225	437/18862	3.12E-07	7.55E-05	5.61E-05	8140/841/28984/6375/8013/57162/255231/3304/3662/351/3115/3553/6348/1958/5196/7124/972/3458/3113/4050	20

BP	GO:0051090	regulation of DNA-binding transcription factor activity	20/225	444/18862	4.02E-07	8.16E-05	6.06E-05	1524/7128/3397/28984/6375/3725/57162/3304/3399/351/219790/3553/7185/6446/7293/10221/6279/11213/6280/7124	20
BP	GO:1903706	regulation of hemopoiesis	19/225	415/18862	6.21E-07	0.000102756	7.63E-05	639/841/9636/8013/3725/3304/64332/3662/1493/10221/6348/3674/5196/84807/257101/7124/972/3458/3123	19
BP	GO:0022407	regulation of cell-cell adhesion	19/225	437/18862	1.34E-06	0.000167204	0.000124149	83706/28984/6375/8013/288/57162/64332/1493/8728/3115/3553/11326/84807/7124/972/3458/3113/3123/3956	19
BP	GO:0002429	immune response-activating cell surface receptor signaling pathway	19/225	481/18862	5.43E-06	0.000363183	0.000269664	3118/3702/84174/2214/8013/931/952/64332/3127/1493/3115/100423062/84807/3119/3117/3824/2357/3113/3123	19
BP	GO:0002757	immune response-activating signal transduction	19/225	481/18862	5.43E-06	0.000363183	0.000269664	3118/3702/84174/2214/8013/931/952/64332/3127/1493/3115/100423062/84807/3119/3117/3824/2357/3113/3123	19
BP	GO:0034341	response to interferon-gamma	18/225	197/18862	2.57E-11	8.09E-08	6.01E-08	3118/6375/8638/10410/6846/3127/3662/3115/54625/4502/6348/3119/3117/6351/972/3458/3113/3123	18
BP	GO:0002460	adaptive immune response based on somatic recombination of immune receptors built from immunoglobulin superfamily domains	18/225	367/18862	4.59E-07	8.49E-05	6.31E-05	7128/84174/6375/3821/64332/3662/3553/100423062/925/2208/84807/712/60468/3119/3824/7124/972/3123	18
BP	GO:0002696	positive regulation of leukocyte activation	18/225	401/18862	1.64E-06	0.000177983	0.000132153	6375/8013/57162/952/64332/1026/1493/3115/3553/100423062/7293/6348/84807/972/3458/3113/3123/3956	18
BP	GO:0002683	negative regulation of immune system process	18/225	403/18862	1.76E-06	0.000182669	0.000135632	7128/84174/6375/3821/57162/3949/1493/54625/11326/10221/6348/11213/3824/7124/2876/972/3123/8530	18

BP	GO:0050867	positive regulation of cell activation	18/225	412/18862	2.41E-06	0.000208774	0.000155015	6375/8013/57162/952/64332/1026/1493/3115/3553/100423062/7293/6348/84807/972/3458/3113/3123/3956	18
BP	GO:2001233	regulation of apoptotic signaling pathway	17/225	348/18862	1.02E-06	0.000160727	0.00011934	2537/1524/7128/841/3304/219790/3553/7185/3685/6279/5196/467/10105/6280/7124/2876/972	17
BP	GO:1903131	mononuclear cell differentiation	17/225	411/18862	9.49E-06	0.000505643	0.000375441	639/3702/3725/3821/931/64332/3662/1493/3553/925/1958/84807/257101/972/3458/3123/3956	17
BP	GO:0030099	myeloid cell differentiation	17/225	419/18862	1.22E-05	0.000589384	0.000437619	841/9636/8013/3725/87/3304/3662/351/10221/6348/3674/5196/7049/7124/972/3458/3123	17
BP	GO:0006979	response to oxidative stress	17/225	444/18862	2.56E-05	0.000903215	0.000670639	7128/388/8013/3725/952/9124/3304/3039/7168/351/3040/3043/79772/5742/10105/143686/2876	17
BP	GO:0043312	neutrophil degranulation	17/225	485/18862	7.66E-05	0.001740497	0.001292323	2495/7414/2040/5476/3304/2512/3043/3685/290/2352/6279/2204/2171/6280/6282/2357/23406	17
BP	GO:0002283	neutrophil activation involved in immune response	17/225	488/18862	8.26E-05	0.001802479	0.001338344	2495/7414/2040/5476/3304/2512/3043/3685/290/2352/6279/2204/2171/6280/6282/2357/23406	17
BP	GO:0002446	neutrophil mediated immunity	17/225	499/18862	0.00010814	0.002207679	0.001639206	2495/7414/2040/5476/3304/2512/3043/3685/290/2352/6279/2204/2171/6280/6282/2357/23406	17
BP	GO:0042119	neutrophil activation	17/225	500/18862	0.000110776	0.002246245	0.001667842	2495/7414/2040/5476/3304/2512/3043/3685/290/2352/6279/2204/2171/6280/6282/2357/23406	17
BP	GO:0071346	cellular response to interferon-gamma	16/225	177/18862	3.98E-10	6.26E-07	4.65E-07	3118/6375/8638/6846/3127/3662/3115/54625/4502/6348/3119/3117/6351/3458/3113/3123	16
BP	GO:0070661	leukocyte proliferation	16/225	312/18862	1.14E-06	0.000167204	0.000124149	7128/6375/57162/931/952/1026/1493/3115/3553/3070/51237/7293/11326/972/3113/3123	16
BP	GO:0032496	response to lipopolysaccharide	16/225	326/18862	2.02E-06	0.000192287	0.000142774	1524/7128/3248/841/3725/57162/3553/10221/6279/6348/5196/6678/7056/11213/6280/7124	16

BP	GO:0050863	regulation of T cell activation	16/225	327/18862	2.10E-06	0.000194182	0.00014418	639/6375/57162/64332/3662/1493/3115/3553/11326/84807/257101/972/3458/3113/3123/3956	16
BP	GO:0002237	response to molecule of bacterial origin	16/225	346/18862	4.34E-06	0.000316945	0.000235332	1524/7128/3248/841/3725/57162/3553/10221/6279/6348/5196/6678/7056/11213/6280/7124	16
BP	GO:0019058	viral life cycle	16/225	348/18862	4.67E-06	0.000333284	0.000247464	4599/2040/3429/9636/8638/10410/3949/3304/3685/7293/290/3678/7124/972/3123/3956	16
BP	GO:0051251	positive regulation of lymphocyte activation	16/225	356/18862	6.22E-06	0.00040685	0.000302087	6375/57162/952/64332/1026/1493/3115/3553/100423062/7293/84807/972/3458/3113/3123/3956	16
BP	GO:0030098	lymphocyte differentiation	16/225	358/18862	6.68E-06	0.00040685	0.000302087	639/3702/3821/931/64332/3662/1493/3553/925/1958/84807/257101/972/3458/3123/3956	16
BP	GO:0009615	response to virus	16/225	359/18862	6.91E-06	0.00040685	0.000302087	10964/2537/4599/7128/3437/3429/6375/9636/3925/8638/10410/3433/11213/7124/6351/3458	16
BP	GO:0001667	ameboidal-type cell migration	16/225	473/18862	0.00018703	0.003321112	0.002465933	3397/388/28984/1647/9124/3164/80005/9839/10501/23499/6678/1839/7124/2876/7076/3458	16
BP	GO:0002699	positive regulation of immune effector process	15/225	219/18862	6.17E-08	1.62E-05	1.20E-05	8140/6375/8013/64332/117157/3553/51237/7293/2208/84807/3824/7124/972/3458/3123	15
BP	GO:0022409	positive regulation of cell-cell adhesion	15/225	276/18862	1.21E-06	0.000167204	0.000124149	6375/8013/288/64332/1493/8728/3115/3553/84807/7124/972/3458/3113/3123/3956	15
BP	GO:1902105	regulation of leukocyte differentiation	15/225	279/18862	1.38E-06	0.000167204	0.000124149	639/841/3725/64332/3662/1493/10221/6348/5196/84807/257101/7124/972/3458/3123	15
BP	GO:0046651	lymphocyte proliferation	15/225	282/18862	1.58E-06	0.000177983	0.000132153	6375/57162/931/952/1026/1493/3115/3553/3070/51237/7293/11326/972/3113/3123	15
BP	GO:0032943	mononuclear cell proliferation	15/225	285/18862	1.80E-06	0.000182669	0.000135632	6375/57162/931/952/1026/1493/3115/3553/3070/51237/7293/11326/972/3113/3123	15
BP	GO:0051235	maintenance of location	15/225	319/18862	7.12E-06	0.00040685	0.000302087	2495/6375/288/255231/51099/55704/2512/3553/19/3685/6279/6348/6280/7124/23345	15

BP	GO:0050851	antigen receptor-mediated signaling pathway	15/225	323/18862	8.26E-06	0.000447853	0.000332532	3118/3702/84174/931/952/64332/3127/1493/3115/100423062/84807/3119/3117/3113/3123	15
BP	GO:1903037	regulation of leukocyte cell-cell adhesion	15/225	330/18862	1.07E-05	0.000540778	0.000401529	6375/8013/57162/64332/1493/3115/3553/11326/84807/7124/972/3458/3113/3123/3956	15
BP	GO:0002449	lymphocyte mediated immunity	15/225	360/18862	2.95E-05	0.001012589	0.00075185	84174/6375/3821/117157/3553/100423062/925/2208/712/3119/3824/7124/972/3123/3002	15
BP	GO:0050727	regulation of inflammatory response	15/225	366/18862	3.57E-05	0.001031565	0.000765939	7128/6375/3949/64332/351/3553/283131/6279/6348/6280/7124/2876/3458/3123/8530	15
BP	GO:0006959	humoral immune response	15/225	380/18862	5.47E-05	0.001420781	0.001054933	28984/931/3553/100423062/10578/11326/2208/6279/5196/712/3119/6280/7124/3458/3123	15
BP	GO:0032102	negative regulation of response to external stimulus	15/225	394/18862	8.21E-05	0.001802479	0.001338344	7128/3821/3949/54625/5055/10501/11326/10221/7056/11213/3824/7124/2876/3123/8530	15
BP	GO:1903793	positive regulation of anion transport	15/225	478/18862	0.000648495	0.008457347	0.006279599	4946/2040/841/7533/8013/288/952/3553/19/6279/7273/7124/3458/3123/3002	15
BP	GO:0071900	regulation of protein serine/threonine kinase activity	15/225	492/18862	0.000869213	0.010588896	0.007862278	1846/7128/28984/1647/1026/5997/3553/1844/5577/10221/11213/7124/2357/972/3458	15
BP	GO:0070663	regulation of leukocyte proliferation	14/225	241/18862	1.26E-06	0.000167204	0.000124149	7128/6375/57162/952/1026/1493/3115/3553/51237/7293/11326/972/3113/3123	14
BP	GO:0030217	T cell differentiation	14/225	246/18862	1.61E-06	0.000177983	0.000132153	639/3702/3821/64332/3662/1493/3553/925/1958/84807/257101/972/3458/3123	14
BP	GO:0007596	blood coagulation	14/225	342/18862	6.59E-05	0.001604908	0.001191648	3611/83706/7414/3043/7094/5055/3048/5577/2162/2815/3674/340205/5196/7056	14
BP	GO:0007599	hemostasis	14/225	346/18862	7.46E-05	0.001737861	0.001290366	3611/83706/7414/3043/7094/5055/3048/5577/2162/2815/3674/340205/5196/7056	14

BP	GO:0050817	coagulation	14/225	347/18862	7.70E-05	0.001740497	0.001292323	3611/83706/7414/3043/7094/5055/3048/5577/2162/2815/3674/340205/5196/7056	14
BP	GO:0016049	cell growth	14/225	470/18862	0.001597763	0.016095412	0.011950877	7414/64061/952/3304/1026/2273/5997/351/6446/10501/6279/23499/1839/6280	14
BP	GO:0060284	regulation of cell development	14/225	485/18862	0.002134484	0.020329344	0.015094581	1524/3611/7414/3397/7533/3949/80005/3553/10501/10221/23499/23654/7124/3458	14
BP	GO:0043087	regulation of GTPase activity	14/225	487/18862	0.002216191	0.020486727	0.015211439	5996/51429/6375/3725/3925/6846/5997/80005/10144/4650/11138/6348/23654/6351	14
BP	GO:0072507	divalent inorganic cation homeostasis	14/225	499/18862	0.002762658	0.024667706	0.018315825	1524/6375/288/931/952/255231/351/4502/3685/6279/6348/4493/6280/2357	14
BP	GO:0002576	platelet degranulation	13/225	128/18862	4.42E-09	2.78E-06	2.06E-06	83706/7414/87/23052/351/7094/2162/3674/5196/6678/7273/7076/1521	13
BP	GO:0097191	extrinsic apoptotic signaling pathway	13/225	217/18862	2.16E-06	0.000194404	0.000144346	2537/7128/841/3429/3304/3553/7185/3685/5196/467/7124/2876/3458	13
BP	GO:0050670	regulation of lymphocyte proliferation	13/225	221/18862	2.65E-06	0.000219212	0.000162765	6375/57162/952/1026/1493/3115/3553/51237/7293/11326/972/3113/3123	13
BP	GO:0032944	regulation of mononuclear cell proliferation	13/225	223/18862	2.93E-06	0.000235929	0.000175178	6375/57162/952/1026/1493/3115/3553/51237/7293/11326/972/3113/3123	13
BP	GO:2001234	negative regulation of apoptotic signaling pathway	13/225	224/18862	3.08E-06	0.000241661	0.000179434	2537/1524/7128/841/3304/219790/3553/3685/5196/10105/7124/2876/972	13
BP	GO:1903039	positive regulation of leukocyte cell-cell adhesion	13/225	234/18862	4.96E-06	0.000346646	0.000257385	6375/8013/64332/1493/3115/3553/84807/7124/972/3458/3113/3123/3956	13
BP	GO:0045637	regulation of myeloid cell differentiation	13/225	258/18862	1.42E-05	0.000646237	0.000479832	841/9636/8013/3725/3304/10221/6348/3674/5196/7124/972/3458/3123	13

BP	GO:0072593	reactive oxygen species metabolic process	13/225	281/18862	3.47E-05	0.001031565	0.000765939	2537/1524/1647/10229/1026/3039/3040/3553/3043/3048/7124/2876/3458	13
BP	GO:0034612	response to tumor necrosis factor	13/225	320/18862	0.000129612	0.002499213	0.001855671	3611/7128/841/6375/3304/6846/7185/7293/3899/6348/7124/6351/4050	13
BP	GO:0010038	response to metal ion	13/225	352/18862	0.000327829	0.005177718	0.003844467	841/360/288/3725/351/4502/366/6279/4493/6678/712/7273/10105	13
BP	GO:0051271	negative regulation of cellular component movement	13/225	352/18862	0.000327829	0.005177718	0.003844467	1524/7414/388/28984/1647/87/7168/79772/10221/7049/7124/972/7076	13
BP	GO:0010631	epithelial cell migration	13/225	357/18862	0.000374994	0.005777478	0.00428979	3397/388/28984/1647/3164/80005/9839/23499/6678/1839/7124/2876/3458	13
BP	GO:0090132	epithelium migration	13/225	360/18862	0.000405976	0.006105178	0.004533108	3397/388/28984/1647/3164/80005/9839/23499/6678/1839/7124/2876/3458	13
BP	GO:0090130	tissue migration	13/225	365/18862	0.000462452	0.006698091	0.004973347	3397/388/28984/1647/3164/80005/9839/23499/6678/1839/7124/2876/3458	13
BP	GO:0042176	regulation of protein catabolic process	13/225	383/18862	0.000724243	0.009141751	0.006787769	4946/51429/7128/5476/3949/3304/3553/10221/11213/7124/2876/7076/3458	13
BP	GO:0042326	negative regulation of phosphorylation	13/225	407/18862	0.001258635	0.013364492	0.009923164	1846/7128/1647/1026/5997/3553/54625/1844/5577/10221/10105/11213/3458	13
BP	GO:0072503	cellular divalent inorganic cation homeostasis	13/225	480/18862	0.005189845	0.039116747	0.029044268	1524/6375/931/952/255231/351/4502/3685/6279/6348/4493/6280/2357	13
BP	GO:0060333	interferon-gamma-mediated signaling pathway	12/225	91/18862	8.52E-10	8.93E-07	6.63E-07	3118/8638/3127/3662/3115/54625/4502/3119/3117/3458/3113/3123	12
BP	GO:0050852	T cell receptor signaling pathway	12/225	204/18862	6.46E-06	0.00040685	0.000302087	3118/3702/84174/64332/3127/1493/3115/84807/3119/3117/3113/3123	12

BP	GO:0009636	response to toxic substance	12/225	239/18862	3.15E-05	0.001012589	0.00075185	1026/3039/3040/3043/4502/3048/5742/6348/4493/6280/2876/6351	12
BP	GO:0007249	I-kappaB kinase/NF-kappaB signaling	12/225	282/18862	0.000153095	0.002830456	0.002101619	1524/7128/841/57162/3553/7185/6285/7124/972/3123/6275/3956	12
BP	GO:0002440	production of molecular mediator of immune response	12/225	286/18862	0.000174483	0.00315721	0.002344235	8140/6375/8013/3553/51237/29802/7293/3119/11213/7124/972/3123	12
BP	GO:0071356	cellular response to tumor necrosis factor	12/225	296/18862	0.000239426	0.004096213	0.003041447	3611/7128/841/6375/3304/6846/7185/7293/6348/7124/6351/4050	12
BP	GO:0051222	positive regulation of protein transport	12/225	312/18862	0.000385834	0.005886782	0.004370948	4946/2040/841/7533/288/952/3553/7273/7124/3458/3123/3002	12
BP	GO:0070371	ERK1 and ERK2 cascade	12/225	320/18862	0.000483739	0.006910877	0.005131341	1846/6375/6846/351/3553/3685/6348/467/7124/6351/972/3123	12
BP	GO:0042113	B cell activation	12/225	326/18862	0.000570237	0.007744515	0.005750319	7128/841/57162/931/952/1026/1493/100423062/51237/7293/972/3956	12
BP	GO:1904951	positive regulation of establishment of protein localization	12/225	328/18862	0.000601815	0.008076351	0.005996708	4946/2040/841/7533/288/952/3553/7273/7124/3458/3123/3002	12
BP	GO:0001933	negative regulation of protein phosphorylation	12/225	362/18862	0.001408466	0.014507906	0.010772151	1846/7128/1647/1026/5997/3553/54625/1844/5577/10221/11213/3458	12
BP	GO:0040013	negative regulation of locomotion	12/225	377/18862	0.001977418	0.019110855	0.014189851	1524/7414/388/28984/1647/7168/10501/79772/10221/7124/972/7076	12
BP	GO:0001558	regulation of cell growth	12/225	406/18862	0.003610928	0.029866173	0.022175697	64061/952/3304/1026/2273/5997/6446/10501/6279/23499/1839/6280	12
BP	GO:0043547	positive regulation of GTPase activity	12/225	411/18862	0.003979742	0.031909005	0.023692505	5996/51429/6375/3725/6846/5997/80005/10144/4650/11138/6348/6351	12

BP	GO:0051960	regulation of nervous system development	12/225	422/18862	0.004898232	0.037276378	0.02767779	1524/3397/7533/3949/3553/10501/1890/23499/23654/7124/3458/8530	12
BP	GO:0060337	type I interferon signaling pathway	11/225	95/18862	1.77E-08	7.76E-06	5.76E-06	2537/4599/54739/3437/3429/9636/8638/10410/3662/3433/1958	11
BP	GO:0071357	cellular response to type I interferon	11/225	96/18862	1.98E-08	7.76E-06	5.76E-06	2537/4599/54739/3437/3429/9636/8638/10410/3662/3433/1958	11
BP	GO:0034340	response to type I interferon	11/225	101/18862	3.38E-08	1.18E-05	8.76E-06	2537/4599/54739/3437/3429/9636/8638/10410/3662/3433/1958	11
BP	GO:2001236	regulation of extrinsic apoptotic signaling pathway	11/225	154/18862	2.46E-06	0.000208774	0.000155015	2537/7128/841/3304/3553/7185/3685/5196/467/7124/2876	11
BP	GO:0002573	myeloid leukocyte differentiation	11/225	204/18862	3.53E-05	0.001031565	0.000765939	841/3725/3662/351/10221/6348/5196/7124/972/3458/3123	11
BP	GO:0050870	positive regulation of T cell activation	11/225	212/18862	5.01E-05	0.001323138	0.000982433	6375/64332/1493/3115/3553/84807/972/3458/3113/3123/3956	11
BP	GO:0051701	biological process involved in interaction with host	11/225	219/18862	6.72E-05	0.001611541	0.001196572	841/10410/3949/3304/3685/7293/290/3678/972/3123/3956	11
BP	GO:0033002	muscle cell proliferation	11/225	222/18862	7.59E-05	0.001740497	0.001292323	7128/3248/8013/3725/1026/7168/10221/1839/7049/7124/3458	11
BP	GO:0000302	response to reactive oxygen species	11/225	224/18862	8.22E-05	0.001802479	0.001338344	7128/388/8013/3725/3039/7168/3040/3043/10105/143686/2876	11
BP	GO:0006469	negative regulation of protein kinase activity	11/225	243/18862	0.000168646	0.003099725	0.002301552	1846/7128/1647/1026/5997/3553/1844/5577/10221/11213/3458	11

BP	GO:0043122	regulation of I-kappaB kinase/NF-kappaB signaling	11/225	244/18862	0.000174787	0.00315721	0.002344235	7128/841/57162/3553/7185/6285/7124/972/3123/6275/3956	11
BP	GO:0051607	defense response to virus	11/225	260/18862	0.000302102	0.004844429	0.003596999	10964/2537/4599/7128/3437/3429/9636/8638/10410/3433/3458	11
BP	GO:0140546	defense response to symbiont	11/225	260/18862	0.000302102	0.004844429	0.003596999	10964/2537/4599/7128/3437/3429/9636/8638/10410/3433/3458	11
BP	GO:0051091	positive regulation of DNA-binding transcription factor activity	11/225	266/18862	0.000366694	0.005677439	0.00421551	1524/28984/3304/351/219790/3553/7185/6279/11213/6280/7124	11
BP	GO:0033673	negative regulation of kinase activity	11/225	267/18862	0.000378513	0.005803257	0.004308931	1846/7128/1647/1026/5997/3553/1844/5577/10221/11213/3458	11
BP	GO:0051348	negative regulation of transferase activity	11/225	296/18862	0.000890134	0.010801902	0.008020435	1846/7128/1647/1026/5997/3553/1844/5577/10221/11213/3458	11
BP	GO:0070372	regulation of ERK1 and ERK2 cascade	11/225	301/18862	0.001019597	0.011738438	0.008715816	1846/6375/6846/351/3553/6348/467/7124/6351/972/3123	11
BP	GO:0060326	cell chemotaxis	11/225	306/18862	0.001164248	0.012799106	0.009503364	1524/6375/6846/3164/6279/6348/5196/1839/6280/6351/972	11
BP	GO:0007517	muscle organ development	11/225	317/18862	0.001542586	0.015741394	0.011688018	9172/2273/3399/7168/463/1958/7273/1839/7049/467/2876	11
BP	GO:0030336	negative regulation of cell migration	11/225	330/18862	0.002113941	0.020194881	0.014994743	1524/7414/388/28984/1647/7168/79772/10221/7124/972/7076	11
BP	GO:1901342	regulation of vasculature development	11/225	341/18862	0.00272225	0.024445808	0.018151065	1524/7128/3397/388/28984/1647/3553/5196/6678/3678/7124	11

BP	GO:2000146	negative regulation of cell motility	11/225	345/18862	0.002975711	0.02597961	0.019289916	1524/7414/388/28984/1647/7168/79772/10221/7124/972/7076	11
BP	GO:0031589	cell-substrate adhesion	11/225	359/18862	0.004016484	0.032055643	0.023801383	3611/83706/7414/3397/87/80005/3685/3674/23499/3678/3956	11
BP	GO:0031349	positive regulation of defense response	11/225	361/18862	0.00418635	0.032976687	0.024485261	64332/351/117157/3553/283131/6279/6348/3824/6280/7124/3458	11
BP	GO:1902903	regulation of supramolecular fiber organization	11/225	370/18862	0.005023016	0.038133668	0.02831433	51429/3397/28984/3925/3949/3304/55704/7168/351/2876/23406	11
BP	GO:0050678	regulation of epithelial cell proliferation	11/225	374/18862	0.005435026	0.039911888	0.029634662	7128/3397/28984/374/8013/9788/3164/6678/7049/7124/2876	11
BP	GO:0031331	positive regulation of cellular catabolic process	11/225	384/18862	0.006582505	0.045670671	0.033910571	51429/7128/3949/3304/51099/351/3553/10221/7124/143686/3458	11
BP	GO:0045639	positive regulation of myeloid cell differentiation	10/225	102/18862	3.91E-07	8.16E-05	6.06E-05	841/9636/3725/3304/10221/5196/7124/972/3458/3123	10
BP	GO:0046718	viral entry into host cell	10/225	140/18862	7.09E-06	0.00040685	0.000302087	10410/3949/3304/3685/7293/290/3678/972/3123/3956	10
BP	GO:0050777	negative regulation of immune response	10/225	150/18862	1.30E-05	0.000602693	0.000447501	7128/6375/3821/1493/54625/11326/11213/3824/2876/3123	10
BP	GO:0044409	entry into host	10/225	153/18862	1.55E-05	0.000686329	0.000509601	10410/3949/3304/3685/7293/290/3678/972/3123/3956	10
BP	GO:1902107	positive regulation of leukocyte differentiation	10/225	154/18862	1.64E-05	0.000706533	0.000524602	841/3725/64332/10221/5196/84807/7124/972/3458/3123	10
BP	GO:1903708	positive regulation of hemopoiesis	10/225	154/18862	1.64E-05	0.000706533	0.000524602	841/3725/64332/10221/5196/84807/7124/972/3458/3123	10

BP	GO:0002822	regulation of adaptive immune response based on somatic recombination of immune receptors built from immunoglobulin superfamily domains	10/225	155/18862	1.74E-05	0.00071798	0.000533101	7128/6375/3821/64332/3553/2208/84807/3824/7124/3123	10
BP	GO:0030168	platelet activation	10/225	157/18862	1.94E-05	0.000775404	0.000575739	3611/83706/7414/3043/7094/2815/3674/340205/5196/7056	10
BP	GO:0051092	positive regulation of NF-kappaB transcription factor activity	10/225	159/18862	2.17E-05	0.00083424	0.000619425	1524/3304/351/219790/3553/7185/6279/11213/6280/7124	10
BP	GO:0048660	regulation of smooth muscle cell proliferation	10/225	160/18862	2.29E-05	0.000865714	0.000642794	7128/3248/8013/3725/1026/7168/10221/1839/7124/3458	10
BP	GO:0048659	smooth muscle cell proliferation	10/225	162/18862	2.54E-05	0.000903215	0.000670639	7128/3248/8013/3725/1026/7168/10221/1839/7124/3458	10
BP	GO:0002819	regulation of adaptive immune response	10/225	170/18862	3.85E-05	0.001088904	0.000808514	7128/6375/3821/64332/3553/2208/84807/3824/7124/3123	10
BP	GO:0052126	movement in host environment	10/225	175/18862	4.92E-05	0.001310063	0.000972724	10410/3949/3304/3685/7293/290/3678/972/3123/3956	10
BP	GO:0001906	cell killing	10/225	179/18862	5.95E-05	0.001508496	0.001120061	841/6375/3821/10578/2208/5196/3824/3458/3123/3002	10
BP	GO:0050866	negative regulation of cell activation	10/225	200/18862	0.000149319	0.002776989	0.00206192	7128/6375/57162/3949/1493/11326/7056/972/3123/8530	10
BP	GO:0070555	response to interleukin-1	10/225	206/18862	0.000189914	0.003334631	0.002475971	6375/57162/952/6846/351/3553/6348/1958/11213/6351	10

BP	GO:0051651	maintenance of location in cell	10/225	209/18862	0.000213462	0.003706696	0.00275223	2495/6375/288/255231/55704/2512/6279/6348/6280/23345	10
BP	GO:0097529	myeloid leukocyte migration	10/225	218/18862	0.000299315	0.004844429	0.003596999	1524/6375/255231/6846/6279/6348/5196/6280/6351/972	10
BP	GO:0019882	antigen processing and presentation	10/225	234/18862	0.000522999	0.007437945	0.00552269	3118/3127/3115/3685/925/3119/3117/972/3113/3123	10
BP	GO:0031348	negative regulation of defense response	10/225	236/18862	0.000558798	0.007669446	0.00569458	7128/3821/3949/54625/11326/11213/3824/2876/3123/8530	10
BP	GO:0033157	regulation of intracellular protein transport	10/225	248/18862	0.000818823	0.010132124	0.007523124	4946/2040/841/7533/3429/288/3553/91319/3458/3002	10
BP	GO:0042063	gliogenesis	10/225	283/18862	0.002190648	0.020334737	0.015098586	1524/3949/351/3553/6279/6348/712/6280/7124/3458	10
BP	GO:0043405	regulation of MAP kinase activity	10/225	307/18862	0.003917075	0.031648755	0.023499268	1846/1647/5997/3553/1844/10221/11213/7124/2357/972	10
BP	GO:0043271	negative regulation of ion transport	10/225	319/18862	0.005113281	0.0387254	0.028753692	4946/288/5997/3553/3685/91319/2171/10105/7124/972	10
BP	GO:0045765	regulation of angiogenesis	10/225	335/18862	0.00713435	0.048434032	0.035962372	1524/7128/388/28984/1647/3553/5196/6678/3678/7124	10
BP	GO:0032602	chemokine production	9/225	95/18862	2.01E-06	0.000192287	0.000142774	255231/351/3553/6279/1958/6280/7124/972/3458	9
BP	GO:0042116	macrophage activation	9/225	102/18862	3.65E-06	0.000279811	0.00020776	3949/351/11326/6348/712/7124/972/3458/8530	9
BP	GO:2001237	negative regulation of extrinsic apoptotic signaling pathway	9/225	103/18862	3.96E-06	0.000296234	0.000219955	2537/7128/841/3304/3553/3685/5196/7124/2876	9

BP	GO:0002761	regulation of myeloid leukocyte differentiation	9/225	118/18862	1.21E-05	0.000589384	0.000437619	841/3725/10221/6348/5196/7124/972/3458/3123	9
BP	GO:0046916	cellular transition metal ion homeostasis	9/225	118/18862	1.21E-05	0.000589384	0.000437619	2495/30061/2512/351/4502/6279/4493/6280/3458	9
BP	GO:0035821	modulation of process of other organism	9/225	123/18862	1.69E-05	0.000708013	0.000525701	2040/841/3429/3725/2208/6348/6280/6351/3458	9
BP	GO:1990266	neutrophil migration	9/225	123/18862	1.69E-05	0.000708013	0.000525701	6375/255231/6846/6279/6348/5196/6280/6351/972	9
BP	GO:0002687	positive regulation of leukocyte migration	9/225	133/18862	3.15E-05	0.001012589	0.00075185	1524/6375/6846/351/6348/3674/7124/6351/972	9
BP	GO:0050729	positive regulation of inflammatory response	9/225	133/18862	3.15E-05	0.001012589	0.00075185	64332/351/3553/283131/6279/6348/6280/7124/3458	9
BP	GO:0042542	response to hydrogen peroxide	9/225	135/18862	3.55E-05	0.001031565	0.000765939	7128/388/8013/3725/3039/3040/3043/10105/2876	9
BP	GO:0050671	positive regulation of lymphocyte proliferation	9/225	135/18862	3.55E-05	0.001031565	0.000765939	6375/57162/952/1026/3115/3553/7293/972/3113	9
BP	GO:0032946	positive regulation of mononuclear cell proliferation	9/225	136/18862	3.76E-05	0.001074223	0.000797613	6375/57162/952/1026/3115/3553/7293/972/3113	9
BP	GO:0098754	detoxification	9/225	138/18862	4.22E-05	0.001170479	0.000869083	3039/3040/3043/4502/3048/5742/4493/6280/2876	9
BP	GO:0055076	transition metal ion homeostasis	9/225	139/18862	4.46E-05	0.001209254	0.000897874	2495/30061/2512/351/4502/6279/4493/6280/3458	9
BP	GO:0071901	negative regulation of protein serine/threonine kinase activity	9/225	145/18862	6.21E-05	0.001560767	0.001158873	1846/7128/1647/1026/5997/3553/1844/5577/11213	9

BP	GO:0002700	regulation of production of molecular mediator of immune response	9/225	146/18862	6.55E-05	0.001604908	0.001191648	8140/6375/8013/3553/51237/7293/11213/7124/972	9
BP	GO:0045580	regulation of T cell differentiation	9/225	146/18862	6.55E-05	0.001604908	0.001191648	639/64332/3662/1493/84807/257101/972/3458/3123	9
BP	GO:0070665	positive regulation of leukocyte proliferation	9/225	148/18862	7.28E-05	0.00171926	0.001276555	6375/57162/952/1026/3115/3553/7293/972/3113	9
BP	GO:0097530	granulocyte migration	9/225	148/18862	7.28E-05	0.00171926	0.001276555	6375/255231/6846/6279/6348/5196/6280/6351/972	9
BP	GO:0046631	alpha-beta T cell activation	9/225	149/18862	7.66E-05	0.001740497	0.001292323	639/3702/6375/64332/3662/84807/257101/3458/3123	9
BP	GO:0045619	regulation of lymphocyte differentiation	9/225	175/18862	0.000259147	0.004286831	0.003182981	639/64332/3662/1493/84807/257101/972/3458/3123	9
BP	GO:0001959	regulation of cytokine-mediated signaling pathway	9/225	177/18862	0.000281913	0.004590943	0.003408785	7128/841/3304/7185/54625/11213/7124/972/3458	9
BP	GO:0002478	antigen processing and presentation of exogenous peptide antigen	9/225	177/18862	0.000281913	0.004590943	0.003408785	3118/3127/3115/3685/3119/3117/972/3113/3123	9
BP	GO:0002695	negative regulation of leukocyte activation	9/225	182/18862	0.000346166	0.005402061	0.004011042	7128/6375/57162/3949/1493/11326/972/3123/8530	9
BP	GO:0019884	antigen processing and presentation of exogenous antigen	9/225	185/18862	0.00039021	0.00592478	0.004399161	3118/3127/3115/3685/3119/3117/972/3113/3123	9
BP	GO:0060759	regulation of response to cytokine stimulus	9/225	189/18862	0.000456027	0.006697632	0.004973006	7128/841/3304/7185/54625/11213/7124/972/3458	9

BP	GO:0050864	regulation of B cell activation	9/225	193/18862	0.000530715	0.007513683	0.005578925	7128/57162/952/1026/1493/100423062/51237/7293/972	9
BP	GO:0048002	antigen processing and presentation of peptide antigen	9/225	194/18862	0.000550876	0.007626168	0.005662446	3118/3127/3115/3685/3119/3117/972/3113/3123	9
BP	GO:0042098	T cell proliferation	9/225	195/18862	0.00057166	0.007744515	0.005750319	6375/57162/1493/3115/3553/7293/11326/3113/3123	9
BP	GO:0043903	regulation of biological process involved in symbiotic interaction	9/225	197/18862	0.000615159	0.008157991	0.006057326	4599/9636/8638/10410/3685/7124/972/3123/3956	9
BP	GO:0002685	regulation of leukocyte migration	9/225	205/18862	0.000817123	0.010132124	0.007523124	1524/6375/6846/351/6348/3674/7124/6351/972	9
BP	GO:0045732	positive regulation of protein catabolic process	9/225	225/18862	0.001565124	0.0158517	0.011769921	4946/51429/7128/3949/3304/3553/10221/7124/3458	9
BP	GO:0019724	B cell mediated immunity	9/225	226/18862	0.001613521	0.016202222	0.012030184	84174/6375/100423062/2208/712/3119/7124/972/3123	9
BP	GO:0097305	response to alcohol	9/225	233/18862	0.001986944	0.019110855	0.014189851	3248/841/1026/5997/6446/6279/6348/6678/7049	9
BP	GO:0050890	cognition	9/225	276/18862	0.00604367	0.043010459	0.031935358	1524/3725/3949/351/6446/5577/3678/6285/7124	9
BP	GO:0097193	intrinsic apoptotic signaling pathway	9/225	283/18862	0.007077218	0.048355863	0.035904331	2537/1026/219790/6279/10105/6280/7124/2876/972	9
BP	GO:0002763	positive regulation of myeloid leukocyte differentiation	8/225	58/18862	4.15E-07	8.16E-05	6.06E-05	841/3725/10221/5196/7124/972/3458/3123	8

BP	GO:0034109	homotypic cell-cell adhesion	8/225	85/18862	7.91E-06	0.000436369	0.000324005	3611/83706/7414/288/3043/7094/3674/3956	8
BP	GO:0042100	B cell proliferation	8/225	96/18862	1.95E-05	0.000775404	0.000575739	57162/931/952/1026/1493/51237/7293/972	8
BP	GO:0019886	antigen processing and presentation of exogenous peptide antigen via MHC class II	8/225	99/18862	2.44E-05	0.000881674	0.000654645	3118/3127/3115/3119/3117/972/3113/3123	8
BP	GO:0046634	regulation of alpha-beta T cell activation	8/225	101/18862	2.82E-05	0.00098578	0.000731944	639/6375/64332/3662/84807/257101/3458/3123	8
BP	GO:0002495	antigen processing and presentation of peptide antigen via MHC class II	8/225	103/18862	3.25E-05	0.001012589	0.00075185	3118/3127/3115/3119/3117/972/3113/3123	8
BP	GO:0030593	neutrophil chemotaxis	8/225	103/18862	3.25E-05	0.001012589	0.00075185	6375/6846/6279/6348/5196/6280/6351/972	8
BP	GO:0032651	regulation of interleukin-1 beta production	8/225	103/18862	3.25E-05	0.001012589	0.00075185	1524/7128/841/351/6348/1958/7124/3458	8
BP	GO:0002504	antigen processing and presentation of peptide or polysaccharide antigen via MHC class II	8/225	104/18862	3.49E-05	0.001031565	0.000765939	3118/3127/3115/3119/3117/972/3113/3123	8
BP	GO:0007229	integrin-mediated signaling pathway	8/225	106/18862	4.00E-05	0.001123361	0.000834098	3611/83706/9636/3685/7094/3674/3678/7076	8
BP	GO:0032649	regulation of interferon-gamma production	8/225	107/18862	4.28E-05	0.001170479	0.000869083	8140/6375/9636/3115/3553/7124/3113/3123	8

BP	GO:0046632	alpha-beta T cell differentiation	8/225	107/18862	4.28E-05	0.001170479	0.000869083	639/3702/64332/3662/84807/257101/3458/3123	8
BP	GO:0032611	interleukin-1 beta production	8/225	108/18862	4.58E-05	0.001229915	0.000913214	1524/7128/841/351/6348/1958/7124/3458	8
BP	GO:0032609	interferon-gamma production	8/225	112/18862	5.94E-05	0.001508496	0.001120061	8140/6375/9636/3115/3553/7124/3113/3123	8
BP	GO:0008637	apoptotic mitochondrial changes	8/225	118/18862	8.59E-05	0.001862651	0.001383022	2537/841/7533/3725/3433/10105/2876/3002	8
BP	GO:0032652	regulation of interleukin-1 production	8/225	119/18862	9.12E-05	0.001949788	0.001447722	1524/7128/841/351/6348/1958/7124/3458	8
BP	GO:0071621	granulocyte chemotaxis	8/225	124/18862	0.000121666	0.002392776	0.001776641	6375/6846/6279/6348/5196/6280/6351/972	8
BP	GO:0032612	interleukin-1 production	8/225	126/18862	0.000136004	0.002606469	0.001935309	1524/7128/841/351/6348/1958/7124/3458	8
BP	GO:1903900	regulation of viral life cycle	8/225	148/18862	0.000407919	0.006105182	0.00453311	4599/9636/8638/10410/7124/972/3123/3956	8
BP	GO:0002706	regulation of lymphocyte mediated immunity	8/225	157/18862	0.000603863	0.008076351	0.005996708	6375/3821/117157/3553/2208/3824/7124/3123	8
BP	GO:0042129	regulation of T cell proliferation	8/225	168/18862	0.000939937	0.011148013	0.008277423	6375/57162/1493/3115/3553/11326/3113/3123	8
BP	GO:0033209	tumor necrosis factor-mediated signaling pathway	8/225	174/18862	0.001178274	0.012858734	0.009547637	3611/7128/841/3304/7185/7293/7124/4050	8
BP	GO:0043433	negative regulation of DNA-binding	8/225	177/18862	0.001314298	0.01381551	0.010258046	7128/3397/6375/57162/3399/7293/10221/11213	8

		transcription factor activity							
BP	GO:0090316	positive regulation of intracellular protein transport	8/225	179/18862	0.00141172	0.014507906	0.010772151	4946/2040/841/7533/288/3553/3458/3002	8
BP	GO:0071347	cellular response to interleukin-1	8/225	180/18862	0.001462537	0.014973141	0.011117589	6375/57162/6846/3553/6348/1958/11213/6351	8
BP	GO:0043123	positive regulation of I-kappaB kinase/NF-kappaB signaling	8/225	182/18862	0.001568527	0.0158517	0.011769921	841/57162/6285/7124/972/3123/6275/3956	8
BP	GO:0050792	regulation of viral process	8/225	186/18862	0.001798744	0.017667034	0.013117811	4599/9636/8638/10410/7124/972/3123/3956	8
BP	GO:0010469	regulation of signaling receptor activity	8/225	188/18862	0.001923449	0.018658641	0.013854081	100462981/100463486/374/351/1839/7124/100463498/3458	8
BP	GO:0002285	lymphocyte activation involved in immune response	8/225	189/18862	0.001988307	0.019110855	0.014189851	64332/3662/84807/257101/972/3458/3123/3956	8
BP	GO:2000377	regulation of reactive oxygen species metabolic process	8/225	192/18862	0.002193279	0.020334737	0.015098586	1524/1647/10229/1026/3553/3043/7124/3458	8
BP	GO:1902905	positive regulation of supramolecular fiber organization	8/225	203/18862	0.00309065	0.026760093	0.019869426	51429/3397/28984/3304/55704/7168/351/2876	8
BP	GO:0002221	pattern recognition receptor signaling pathway	8/225	208/18862	0.003582734	0.029748226	0.022088121	7128/841/57162/3304/3662/6279/11213/6280	8

BP	GO:0002703	regulation of leukocyte mediated immunity	8/225	209/18862	0.003688049	0.030186296	0.022413389	6375/3821/117157/3553/2208/3824/7124/3123	8
BP	GO:0070374	positive regulation of ERK1 and ERK2 cascade	8/225	210/18862	0.003795746	0.030987092	0.023007982	6375/6846/351/6348/7124/6351/972/3123	8
BP	GO:0007623	circadian rhythm	8/225	212/18862	0.004018429	0.032055643	0.023801383	3397/3725/3399/22809/463/10135/5730/1958	8
BP	GO:1903792	negative regulation of anion transport	8/225	217/18862	0.004619334	0.035760019	0.02655189	4946/5997/3553/3685/91319/2171/7124/972	8
BP	GO:0032388	positive regulation of intracellular transport	8/225	219/18862	0.00487814	0.037213578	0.027631161	4946/2040/841/7533/288/3553/3458/3002	8
BP	GO:0016064	immunoglobulin mediated immune response	8/225	223/18862	0.005428992	0.039911888	0.029634662	6375/100423062/2208/712/3119/7124/972/3123	8
BP	GO:0030595	leukocyte chemotaxis	8/225	226/18862	0.005872391	0.042718372	0.031718482	6375/6846/6279/6348/5196/6280/6351/972	8
BP	GO:0007611	learning or memory	8/225	235/18862	0.007369073	0.049069907	0.03643451	1524/3725/3949/351/6446/5577/3678/6285	8
BP	GO:0014002	astrocyte development	7/225	42/18862	5.92E-07	0.000102756	7.63E-05	3949/351/6279/712/6280/7124/3458	7
BP	GO:0001774	microglial cell activation	7/225	47/18862	1.31E-06	0.000167204	0.000124149	3949/351/6348/712/7124/3458/8530	7
BP	GO:0032722	positive regulation of chemokine production	7/225	60/18862	7.07E-06	0.00040685	0.000302087	255231/351/3553/1958/7124/972/3458	7
BP	GO:0030888	regulation of B cell proliferation	7/225	61/18862	7.90E-06	0.000436369	0.000324005	57162/952/1026/1493/51237/7293/972	7
BP	GO:0046637	regulation of alpha-beta T cell differentiation	7/225	67/18862	1.48E-05	0.000665736	0.00049431	639/64332/3662/84807/257101/3458/3123	7

BP	GO:0048708	astrocyte differentiation	7/225	72/18862	2.39E-05	0.000881674	0.000654645	3949/351/6279/712/6280/7124/3458	7
BP	GO:0002718	regulation of cytokine production involved in immune response	7/225	84/18862	6.51E-05	0.001604908	0.001191648	8140/6375/8013/3553/11213/7124/972	7
BP	GO:0032642	regulation of chemokine production	7/225	89/18862	9.41E-05	0.001998724	0.001484057	255231/351/3553/1958/7124/972/3458	7
BP	GO:0051702	biological process involved in interaction with symbiont	7/225	92/18862	0.000116107	0.002309657	0.001714925	2040/3429/3725/6348/5196/2876/6351	7
BP	GO:1901216	positive regulation of neuron death	7/225	92/18862	0.000116107	0.002309657	0.001714925	841/3725/6348/1958/712/7124/3458	7
BP	GO:0002367	cytokine production involved in immune response	7/225	93/18862	0.000124302	0.002411609	0.001790625	8140/6375/8013/3553/11213/7124/972	7
BP	GO:0031341	regulation of cell killing	7/225	98/18862	0.000172622	0.003154365	0.002342123	841/6375/3821/2208/3824/3458/3123	7
BP	GO:0002702	positive regulation of production of molecular mediator of immune response	7/225	99/18862	0.000183899	0.003284065	0.002438426	8140/6375/8013/3553/51237/7293/972	7
BP	GO:0002824	positive regulation of adaptive immune response based on somatic recombination of immune receptors built from	7/225	99/18862	0.000183899	0.003284065	0.002438426	6375/64332/3553/2208/84807/7124/3123	7

		immunoglobulin superfamily domains							
BP	GO:0002832	negative regulation of response to biotic stimulus	7/225	100/18862	0.000195765	0.00341827	0.002538073	7128/3821/54625/11326/10221/11213/3824	7
BP	GO:0098869	cellular oxidant detoxification	7/225	102/18862	0.000221351	0.00382256	0.002838259	3039/3040/3043/3048/5742/6280/2876	7
BP	GO:0002821	positive regulation of adaptive immune response	7/225	104/18862	0.000249568	0.004239951	0.003148173	6375/64332/3553/2208/84807/7124/3123	7
BP	GO:0002708	positive regulation of lymphocyte mediated immunity	7/225	108/18862	0.000314684	0.00502057	0.003727784	6375/117157/3553/2208/3824/7124/3123	7
BP	GO:0021782	glial cell development	7/225	112/18862	0.000392783	0.005935173	0.004406879	3949/351/6279/712/6280/7124/3458	7
BP	GO:0033559	unsaturated fatty acid metabolic process	7/225	115/18862	0.000460957	0.006698091	0.004973347	3248/3553/5742/5730/2171/2876/972	7
BP	GO:1990748	cellular detoxification	7/225	115/18862	0.000460957	0.006698091	0.004973347	3039/3040/3043/3048/5742/6280/2876	7
BP	GO:0034766	negative regulation of ion transmembrane transport	7/225	119/18862	0.000566199	0.007737232	0.005744912	4946/288/5997/3553/2171/10105/7124	7
BP	GO:0034763	negative regulation of transmembrane transport	7/225	120/18862	0.000595282	0.008029919	0.005962232	4946/288/5997/3553/2171/10105/7124	7
BP	GO:0097237	cellular response to toxic substance	7/225	122/18862	0.000657005	0.008532924	0.006335714	3039/3040/3043/3048/5742/6280/2876	7
BP	GO:0006690	icosanoid metabolic process	7/225	123/18862	0.000689714	0.008741008	0.006490217	3248/3553/5742/5730/2171/2876/972	7

BP	GO:0002705	positive regulation of leukocyte mediated immunity	7/225	125/18862	0.000758998	0.00954212	0.007085044	6375/117157/3553/2208/3824/7124/3123	7
BP	GO:1905477	positive regulation of protein localization to membrane	7/225	127/18862	0.000833676	0.010266798	0.007623119	2040/841/7533/288/7124/3458/3002	7
BP	GO:0019079	viral genome replication	7/225	129/18862	0.00091404	0.011016475	0.008179756	4599/2040/3429/9636/8638/10410/7124	7
BP	GO:0045834	positive regulation of lipid metabolic process	7/225	150/18862	0.002181014	0.020334737	0.015098586	8013/3949/51099/3553/7124/972/3458	7
BP	GO:0051250	negative regulation of lymphocyte activation	7/225	153/18862	0.002438419	0.022150148	0.016446533	7128/6375/57162/1493/11326/972/3123	7
BP	GO:0051017	actin filament bundle assembly	7/225	154/18862	0.002529222	0.022908777	0.017009816	3397/28984/3925/87/9124/55704/7168	7
BP	GO:0002224	toll-like receptor signaling pathway	7/225	157/18862	0.002817306	0.02498237	0.018549463	7128/841/57162/3662/6279/11213/6280	7
BP	GO:0032675	regulation of interleukin-6 production	7/225	158/18862	0.00291872	0.025553028	0.018973178	7128/351/3553/11213/7124/972/3458	7
BP	GO:0061572	actin filament bundle organization	7/225	158/18862	0.00291872	0.025553028	0.018973178	3397/28984/3925/87/9124/55704/7168	7
BP	GO:0032680	regulation of tumor necrosis factor production	7/225	160/18862	0.003129921	0.027025663	0.020066613	1524/7128/351/6348/5196/11213/3458	7
BP	GO:0032635	interleukin-6 production	7/225	162/18862	0.003352615	0.028402346	0.021088802	7128/351/3553/11213/7124/972/3458	7
BP	GO:0032640	tumor necrosis factor production	7/225	162/18862	0.003352615	0.028402346	0.021088802	1524/7128/351/6348/5196/11213/3458	7

BP	GO:1903555	regulation of tumor necrosis factor superfamily cytokine production	7/225	164/18862	0.003587203	0.029748226	0.022088121	1524/7128/351/6348/5196/11213/3458	7
BP	GO:0071706	tumor necrosis factor superfamily cytokine production	7/225	167/18862	0.003962264	0.031878891	0.023670145	1524/7128/351/6348/5196/11213/3458	7
BP	GO:0001659	temperature homeostasis	7/225	171/18862	0.004507759	0.034982433	0.025974532	3397/3662/3553/1958/2171/7124/2876	7
BP	GO:0007565	female pregnancy	7/225	177/18862	0.00543002	0.039911888	0.029634662	3248/952/5997/3553/3678/7056/7076	7
BP	GO:0006509	membrane protein ectodomain proteolysis	6/225	42/18862	9.88E-06	0.000517326	0.000384115	51429/8728/3553/7124/7076/3458	6
BP	GO:0150076	neuroinflammatory response	6/225	44/18862	1.30E-05	0.000602693	0.000447501	3949/3553/6348/7124/3458/8530	6
BP	GO:0030225	macrophage differentiation	6/225	47/18862	1.92E-05	0.000775404	0.000575739	841/351/10221/5196/3458/3123	6
BP	GO:0006692	prostanoid metabolic process	6/225	48/18862	2.18E-05	0.00083424	0.000619425	3248/3553/5742/5730/2171/972	6
BP	GO:0006693	prostaglandin metabolic process	6/225	48/18862	2.18E-05	0.00083424	0.000619425	3248/3553/5742/5730/2171/972	6
BP	GO:0032731	positive regulation of interleukin-1 beta production	6/225	56/18862	5.32E-05	0.001393161	0.001034425	841/351/6348/1958/7124/3458	6
BP	GO:0033619	membrane protein proteolysis	6/225	61/18862	8.66E-05	0.001863513	0.001383663	51429/8728/3553/7124/7076/3458	6

BP	GO:0070527	platelet aggregation	6/225	62/18862	9.49E-05	0.002001704	0.001486269	3611/83706/7414/3043/7094/3674	6
BP	GO:0071677	positive regulation of mononuclear cell migration	6/225	63/18862	0.00010385	0.002161586	0.001604983	6375/6846/351/6348/7124/6351	6
BP	GO:0032732	positive regulation of interleukin-1 production	6/225	64/18862	0.000113463	0.002285997	0.001697357	841/351/6348/1958/7124/3458	6
BP	GO:0045824	negative regulation of innate immune response	6/225	65/18862	0.00012377	0.002411609	0.001790625	7128/3821/54625/11326/11213/3824	6
BP	GO:0038034	signal transduction in absence of ligand	6/225	67/18862	0.000146607	0.002742779	0.002036519	2537/3304/3553/3685/5196/7124	6
BP	GO:0097192	extrinsic apoptotic signaling pathway in absence of ligand	6/225	67/18862	0.000146607	0.002742779	0.002036519	2537/3304/3553/3685/5196/7124	6
BP	GO:2000514	regulation of CD4-positive, alpha-beta T cell activation	6/225	67/18862	0.000146607	0.002742779	0.002036519	6375/64332/3662/84807/3458/3123	6
BP	GO:1904705	regulation of vascular associated smooth muscle cell proliferation	6/225	83/18862	0.000473405	0.00679412	0.005044649	3248/8013/3725/1026/7168/7124	6
BP	GO:1990874	vascular associated smooth muscle cell proliferation	6/225	83/18862	0.000473405	0.00679412	0.005044649	3248/8013/3725/1026/7168/7124	6
BP	GO:0070098	chemokine-mediated signaling pathway	6/225	88/18862	0.000647005	0.008457347	0.006279599	1524/6375/6846/6348/5196/6351	6

BP	GO:0070664	negative regulation of leukocyte proliferation	6/225	88/18862	0.000647005	0.008457347	0.006279599	7128/6375/57162/1493/11326/3123	6
BP	GO:1990868	response to chemokine	6/225	97/18862	0.001079712	0.012163208	0.009031208	1524/6375/6846/6348/5196/6351	6
BP	GO:1990869	cellular response to chemokine	6/225	97/18862	0.001079712	0.012163208	0.009031208	1524/6375/6846/6348/5196/6351	6
BP	GO:0051817	modulation of process of other organism involved in symbiotic interaction	6/225	98/18862	0.00113887	0.012694823	0.009425933	2040/841/3429/3725/6348/6351	6
BP	GO:0035710	CD4-positive, alpha-beta T cell activation	6/225	100/18862	0.001264612	0.013382746	0.009936717	6375/64332/3662/84807/3458/3123	6
BP	GO:2000379	positive regulation of reactive oxygen species metabolic process	6/225	101/18862	0.00133133	0.013947903	0.010356347	1647/1026/3553/3043/7124/3458	6
BP	GO:0002456	T cell mediated immunity	6/225	104/18862	0.001547694	0.015742401	0.011688766	6375/3821/3553/925/3824/3123	6
BP	GO:0002286	T cell activation involved in immune response	6/225	111/18862	0.002156121	0.020334737	0.015098586	64332/3662/84807/972/3458/3123	6
BP	GO:0071675	regulation of mononuclear cell migration	6/225	111/18862	0.002156121	0.020334737	0.015098586	6375/6846/351/6348/7124/6351	6
BP	GO:1903959	regulation of anion transmembrane transport	6/225	111/18862	0.002156121	0.020334737	0.015098586	4946/8013/5997/3553/2171/7124	6
BP	GO:0050868	negative regulation of T cell activation	6/225	119/18862	0.003055245	0.02652662	0.019696072	6375/57162/1493/11326/972/3123	6
BP	GO:0002698	negative regulation of immune effector process	6/225	125/18862	0.003894381	0.03162801	0.023483865	6375/3821/11326/11213/3824/7124	6

BP	GO:0050768	negative regulation of neurogenesis	6/225	133/18862	0.005264721	0.039586166	0.029392812	3397/7533/3949/3553/10501/7124	6
BP	GO:0003158	endothelium development	6/225	137/18862	0.006068938	0.043057952	0.031970622	7414/3397/388/3553/7124/2876	6
BP	GO:0051961	negative regulation of nervous system development	6/225	138/18862	0.006283242	0.044378042	0.032950791	3397/7533/3949/3553/10501/7124	6
BP	GO:1903038	negative regulation of leukocyte cell-cell adhesion	6/225	138/18862	0.006283242	0.044378042	0.032950791	6375/57162/1493/11326/972/3123	6
BP	GO:0062013	positive regulation of small molecule metabolic process	6/225	141/18862	0.006959198	0.047966576	0.035615285	8013/351/3553/7124/972/3458	6
BP	GO:0010212	response to ionizing radiation	6/225	142/18862	0.00719579	0.048434032	0.035962372	388/1647/1026/1958/7056/2876	6
BP	GO:0051043	regulation of membrane protein ectodomain proteolysis	5/225	23/18862	6.52E-06	0.00040685	0.000302087	51429/3553/7124/7076/3458	5
BP	GO:0048143	astrocyte activation	5/225	25/18862	1.01E-05	0.000520547	0.000386507	3949/351/712/7124/3458	5
BP	GO:0001516	prostaglandin biosynthetic process	5/225	31/18862	3.05E-05	0.001012589	0.00075185	3553/5742/5730/2171/972	5
BP	GO:0042744	hydrogen peroxide catabolic process	5/225	31/18862	3.05E-05	0.001012589	0.00075185	3039/3040/3043/3048/2876	5
BP	GO:0046457	prostanoid biosynthetic process	5/225	31/18862	3.05E-05	0.001012589	0.00075185	3553/5742/5730/2171/972	5

BP	GO:1901099	negative regulation of signal transduction in absence of ligand	5/225	32/18862	3.58E-05	0.001031565	0.000765939	2537/3304/3553/5196/7124	5
BP	GO:2001240	negative regulation of extrinsic apoptotic signaling pathway in absence of ligand	5/225	32/18862	3.58E-05	0.001031565	0.000765939	2537/3304/3553/5196/7124	5
BP	GO:1903960	negative regulation of anion transmembrane transport	5/225	35/18862	5.60E-05	0.001443128	0.001071526	4946/5997/3553/2171/7124	5
BP	GO:2000403	positive regulation of lymphocyte migration	5/225	37/18862	7.38E-05	0.001730335	0.001284777	6375/6846/351/6348/6351	5
BP	GO:2000516	positive regulation of CD4-positive, alpha-beta T cell activation	5/225	39/18862	9.56E-05	0.002002539	0.001486889	6375/64332/84807/3458/3123	5
BP	GO:0030890	positive regulation of B cell proliferation	5/225	40/18862	0.000108171	0.002207679	0.001639206	57162/952/1026/7293/972	5
BP	GO:0150077	regulation of neuroinflammatory response	5/225	40/18862	0.000108171	0.002207679	0.001639206	3949/3553/6348/7124/8530	5
BP	GO:2001239	regulation of extrinsic apoptotic signaling pathway in absence of ligand	5/225	42/18862	0.000137161	0.002612703	0.001939938	2537/3304/3553/5196/7124	5
BP	GO:0052372	modulation by symbiont of entry into host	5/225	48/18862	0.000260521	0.004286998	0.003183105	10410/3685/972/3123/3956	5

BP	GO:0006636	unsaturated fatty acid biosynthetic process	5/225	51/18862	0.000347189	0.005402061	0.004011042	3553/5742/5730/2171/972	5
BP	GO:0043370	regulation of CD4-positive, alpha-beta T cell differentiation	5/225	51/18862	0.000347189	0.005402061	0.004011042	64332/3662/84807/3458/3123	5
BP	GO:0002720	positive regulation of cytokine production involved in immune response	5/225	53/18862	0.000416025	0.006196999	0.004601285	8140/6375/8013/3553/972	5
BP	GO:0045071	negative regulation of viral genome replication	5/225	54/18862	0.000454073	0.006697632	0.004973006	4599/9636/8638/10410/7124	5
BP	GO:0042743	hydrogen peroxide metabolic process	5/225	56/18862	0.000537967	0.007548348	0.005604665	3039/3040/3043/3048/2876	5
BP	GO:0046456	icosanoid biosynthetic process	5/225	56/18862	0.000537967	0.007548348	0.005604665	3553/5742/5730/2171/972	5
BP	GO:0031663	lipopolysaccharide-mediated signaling pathway	5/225	58/18862	0.000633	0.008359319	0.006206812	7128/3553/10221/6348/7124	5
BP	GO:0032663	regulation of interleukin-2 production	5/225	59/18862	0.000684976	0.008722214	0.006476262	7128/6375/3662/3553/11326	5
BP	GO:0043030	regulation of macrophage activation	5/225	59/18862	0.000684976	0.008722214	0.006476262	3949/11326/6348/972/8530	5
BP	GO:0045428	regulation of nitric oxide biosynthetic process	5/225	59/18862	0.000684976	0.008722214	0.006476262	1524/3553/3043/7124/3458	5
BP	GO:0032623	interleukin-2 production	5/225	61/18862	0.00079843	0.009958201	0.007393986	7128/6375/3662/3553/11326	5

BP	GO:0080164	regulation of nitric oxide metabolic process	5/225	61/18862	0.00079843	0.009958201	0.007393986	1524/3553/3043/7124/3458	5
BP	GO:0051851	modulation by host of symbiont process	5/225	62/18862	0.000860144	0.010519194	0.007810524	2040/3429/3725/6348/6351	5
BP	GO:0010803	regulation of tumor necrosis factor-mediated signaling pathway	5/225	63/18862	0.000925342	0.011016475	0.008179756	7128/841/3304/7185/7124	5
BP	GO:0031343	positive regulation of cell killing	5/225	63/18862	0.000925342	0.011016475	0.008179756	6375/2208/3824/3458/3123	5
BP	GO:0048662	negative regulation of smooth muscle cell proliferation	5/225	63/18862	0.000925342	0.011016475	0.008179756	7128/1026/7168/10221/3458	5
BP	GO:2000401	regulation of lymphocyte migration	5/225	63/18862	0.000925342	0.011016475	0.008179756	6375/6846/351/6348/6351	5
BP	GO:0032729	positive regulation of interferon-gamma production	5/225	65/18862	0.001066676	0.012103118	0.008986591	8140/3115/3553/7124/3113	5
BP	GO:0002294	CD4-positive, alpha-beta T cell differentiation involved in immune response	5/225	66/18862	0.001143059	0.012694823	0.009425933	64332/3662/84807/3458/3123	5
BP	GO:0042130	negative regulation of T cell proliferation	5/225	66/18862	0.001143059	0.012694823	0.009425933	6375/57162/1493/11326/3123	5
BP	GO:0046635	positive regulation of alpha-beta T cell activation	5/225	66/18862	0.001143059	0.012694823	0.009425933	6375/64332/84807/3458/3123	5

BP	GO:0002287	alpha-beta T cell activation involved in immune response	5/225	67/18862	0.001223418	0.013034587	0.009678209	64332/3662/84807/3458/3123	5
BP	GO:0002293	alpha-beta T cell differentiation involved in immune response	5/225	67/18862	0.001223418	0.013034587	0.009678209	64332/3662/84807/3458/3123	5
BP	GO:0031640	killing of cells of other organism	5/225	67/18862	0.001223418	0.013034587	0.009678209	841/10578/2208/5196/3458	5
BP	GO:0032922	circadian regulation of gene expression	5/225	67/18862	0.001223418	0.013034587	0.009678209	3397/3399/463/10135/1958	5
BP	GO:1901224	positive regulation of NIK/NF-kappaB signaling	5/225	67/18862	0.001223418	0.013034587	0.009678209	3611/351/219790/3553/7124	5
BP	GO:0002292	T cell differentiation involved in immune response	5/225	73/18862	0.001796166	0.017667034	0.013117811	64332/3662/84807/3458/3123	5
BP	GO:0006809	nitric oxide biosynthetic process	5/225	73/18862	0.001796166	0.017667034	0.013117811	1524/3553/3043/7124/3458	5
BP	GO:0043407	negative regulation of MAP kinase activity	5/225	77/18862	0.002273727	0.020895688	0.015515093	1846/5997/3553/1844/11213	5
BP	GO:0046209	nitric oxide metabolic process	5/225	77/18862	0.002273727	0.020895688	0.015515093	1524/3553/3043/7124/3458	5
BP	GO:0002709	regulation of T cell mediated immunity	5/225	78/18862	0.002406281	0.021921566	0.01627681	6375/3821/3553/3824/3123	5
BP	GO:2001057	reactive nitrogen species metabolic process	5/225	78/18862	0.002406281	0.021921566	0.01627681	1524/3553/3043/7124/3458	5

BP	GO:0045445	myoblast differentiation	5/225	80/18862	0.002688105	0.024208353	0.017974754	3475/3399/116173/7124/3956	5
BP	GO:0014910	regulation of smooth muscle cell migration	5/225	81/18862	0.002837641	0.02498237	0.018549463	8013/7168/80005/10221/6282	5
BP	GO:0043367	CD4-positive, alpha-beta T cell differentiation	5/225	81/18862	0.002837641	0.02498237	0.018549463	64332/3662/84807/3458/3123	5
BP	GO:0050672	negative regulation of lymphocyte proliferation	5/225	81/18862	0.002837641	0.02498237	0.018549463	6375/57162/1493/11326/3123	5
BP	GO:0032945	negative regulation of mononuclear cell proliferation	5/225	82/18862	0.002993102	0.02605906	0.019348908	6375/57162/1493/11326/3123	5
BP	GO:0045069	regulation of viral genome replication	5/225	83/18862	0.003154621	0.027164316	0.020169562	4599/9636/8638/10410/7124	5
BP	GO:0008625	extrinsic apoptotic signaling pathway via death domain receptors	5/225	84/18862	0.003322331	0.028298334	0.021011573	7128/841/467/7124/2876	5
BP	GO:0046330	positive regulation of JNK cascade	5/225	84/18862	0.003322331	0.028298334	0.021011573	1647/351/3553/7185/7124	5
BP	GO:0019915	lipid storage	5/225	85/18862	0.003496362	0.029461304	0.021875081	51099/3553/19/3685/7124	5
BP	GO:0046889	positive regulation of lipid biosynthetic process	5/225	85/18862	0.003496362	0.029461304	0.021875081	3949/3553/7124/972/3458	5
BP	GO:0032755	positive regulation of interleukin-6 production	5/225	86/18862	0.003676848	0.030173195	0.022403662	351/3553/7124/972/3458	5
BP	GO:0051899	membrane depolarization	5/225	87/18862	0.00386392	0.031461922	0.023360544	2537/7533/288/3725/2273	5

BP	GO:0014909	smooth muscle cell migration	5/225	88/18862	0.004057711	0.032205515	0.023912664	8013/7168/80005/10221/6282	5
BP	GO:0045582	positive regulation of T cell differentiation	5/225	88/18862	0.004057711	0.032205515	0.023912664	64332/84807/972/3458/3123	5
BP	GO:0048525	negative regulation of viral process	5/225	89/18862	0.00425835	0.033459982	0.024844108	4599/9636/8638/10410/7124	5
BP	GO:0002690	positive regulation of leukocyte chemotaxis	5/225	91/18862	0.004680698	0.03614603	0.026838504	6375/6846/6348/6351/972	5
BP	GO:0051591	response to cAMP	5/225	93/18862	0.005132007	0.038773792	0.028789624	3725/351/366/6678/7056	5
BP	GO:0050764	regulation of phagocytosis	5/225	94/18862	0.005368844	0.039737442	0.029505136	3560/3553/3685/7124/3458	5
BP	GO:0048661	positive regulation of smooth muscle cell proliferation	5/225	96/18862	0.005865527	0.042718372	0.031718482	3248/8013/3725/1839/7124	5
BP	GO:1903426	regulation of reactive oxygen species biosynthetic process	5/225	99/18862	0.006669967	0.046175564	0.034285455	1524/3553/3043/7124/3458	5
BP	GO:0007044	cell-substrate junction assembly	5/225	100/18862	0.006954459	0.047966576	0.035615285	7414/87/7094/23499/3678	5
BP	GO:0014812	muscle cell migration	5/225	101/18862	0.007247331	0.048434032	0.035962372	8013/7168/80005/10221/6282	5
BP	GO:0045621	positive regulation of lymphocyte differentiation	5/225	101/18862	0.007247331	0.048434032	0.035962372	64332/84807/972/3458/3123	5
BP	GO:0032231	regulation of actin filament bundle assembly	5/225	102/18862	0.007548703	0.049835739	0.037003142	3397/28984/3925/55704/7168	5

BP	GO:0070486	leukocyte aggregation	4/225	13/18862	1.30E-05	0.000602693	0.000447501	8013/3553/6279/6280	4
BP	GO:0015671	oxygen transport	4/225	15/18862	2.43E-05	0.000881674	0.000654645	3039/3040/3043/3048	4
BP	GO:0051044	positive regulation of membrane protein ectodomain proteolysis	4/225	15/18862	2.43E-05	0.000881674	0.000654645	51429/3553/7124/3458	4
BP	GO:0051238	sequestering of metal ion	4/225	16/18862	3.21E-05	0.001012589	0.00075185	2495/2512/6279/6280	4
BP	GO:0002544	chronic inflammatory response	4/225	19/18862	6.64E-05	0.001604908	0.001191648	7128/6279/6280/7124	4
BP	GO:0015669	gas transport	4/225	20/18862	8.22E-05	0.001802479	0.001338344	3039/3040/3043/3048	4
BP	GO:0140131	positive regulation of lymphocyte chemotaxis	4/225	20/18862	8.22E-05	0.001802479	0.001338344	6375/6846/6348/6351	4
BP	GO:0010888	negative regulation of lipid storage	4/225	22/18862	0.000121809	0.002392776	0.001776641	51099/19/3685/7124	4
BP	GO:1901623	regulation of lymphocyte chemotaxis	4/225	26/18862	0.000239804	0.004096213	0.003041447	6375/6846/6348/6351	4
BP	GO:0043372	positive regulation of CD4-positive, alpha-beta T cell differentiation	4/225	32/18862	0.00054539	0.007618494	0.005656748	64332/84807/3458/3123	4
BP	GO:0032770	positive regulation of monooxygenase activity	4/225	33/18862	0.000614857	0.008157991	0.006057326	3553/2208/7124/3458	4
BP	GO:0001914	regulation of T cell mediated cytotoxicity	4/225	38/18862	0.00105867	0.012055792	0.008951451	6375/3821/3824/3123	4

BP	GO:0006882	cellular zinc ion homeostasis	4/225	38/18862	0.00105867	0.012055792	0.008951451	4502/6279/4493/6280	4
BP	GO:0045429	positive regulation of nitric oxide biosynthetic process	4/225	38/18862	0.00105867	0.012055792	0.008951451	3553/3043/7124/3458	4
BP	GO:0045622	regulation of T-helper cell differentiation	4/225	39/18862	0.001168738	0.012799106	0.009503364	64332/3662/84807/3123	4
BP	GO:0050832	defense response to fungus	4/225	39/18862	0.001168738	0.012799106	0.009503364	10578/6279/6280/23406	4
BP	GO:1904407	positive regulation of nitric oxide metabolic process	4/225	39/18862	0.001168738	0.012799106	0.009503364	3553/3043/7124/3458	4
BP	GO:0055069	zinc ion homeostasis	4/225	40/18862	0.001286579	0.01356952	0.010075397	4502/6279/4493/6280	4
BP	GO:0046596	regulation of viral entry into host cell	4/225	41/18862	0.001412478	0.014507906	0.010772151	10410/972/3123/3956	4
BP	GO:0070266	necroptotic process	4/225	43/18862	0.001689595	0.016752041	0.012438426	841/57162/10105/7124	4
BP	GO:0045601	regulation of endothelial cell differentiation	4/225	45/18862	0.002002357	0.019187222	0.014246553	7414/3397/3553/7124	4
BP	GO:1904707	positive regulation of vascular associated smooth muscle cell proliferation	4/225	47/18862	0.002353008	0.02156124	0.016009267	3248/8013/3725/7124	4
BP	GO:0001913	T cell mediated cytotoxicity	4/225	48/18862	0.002543235	0.022969501	0.017054903	6375/3821/3824/3123	4

BP	GO:0046638	positive regulation of alpha-beta T cell differentiation	4/225	49/18862	0.002743759	0.024568759	0.018242356	64332/84807/3458/3123	4
BP	GO:0097300	programmed necrotic cell death	4/225	51/18862	0.003176781	0.027206053	0.020200552	841/57162/10105/7124	4
BP	GO:0001706	endoderm formation	4/225	53/18862	0.0036542	0.030065841	0.022323951	1846/1844/3685/3678	4
BP	GO:0009620	response to fungus	4/225	53/18862	0.0036542	0.030065841	0.022323951	10578/6279/6280/23406	4
BP	GO:0006968	cellular defense response	4/225	54/18862	0.00391021	0.031648755	0.023499268	1524/3702/10219/10578	4
BP	GO:0001836	release of cytochrome c from mitochondria	4/225	55/18862	0.004178095	0.032976687	0.024485261	2537/3725/10105/2876	4
BP	GO:1903428	positive regulation of reactive oxygen species biosynthetic process	4/225	55/18862	0.004178095	0.032976687	0.024485261	3553/3043/7124/3458	4
BP	GO:0010883	regulation of lipid storage	4/225	56/18862	0.004458105	0.03468273	0.025752001	51099/19/3685/7124	4
BP	GO:0051353	positive regulation of oxidoreductase activity	4/225	56/18862	0.004458105	0.03468273	0.025752001	3553/2208/7124/3458	4
BP	GO:0032757	positive regulation of interleukin-8 production	4/225	57/18862	0.004750487	0.036595053	0.027171905	3304/3553/7124/972	4
BP	GO:1902041	regulation of extrinsic apoptotic signaling pathway via death domain receptors	4/225	59/18862	0.005373342	0.039737442	0.029505136	7128/841/467/2876	4

BP	GO:0034113	heterotypic cell-cell adhesion	4/225	60/18862	0.005704289	0.041694372	0.03095816	3553/3685/3678/7124	4
BP	GO:0050994	regulation of lipid catabolic process	4/225	60/18862	0.005704289	0.041694372	0.03095816	3949/51099/3553/7124	4
BP	GO:0046686	response to cadmium ion	4/225	61/18862	0.00604856	0.043010459	0.031935358	3725/4502/4493/6678	4
BP	GO:0032233	positive regulation of actin filament bundle assembly	4/225	62/18862	0.006406381	0.044763753	0.033237182	3397/28984/55704/7168	4
BP	GO:0032613	interleukin-10 production	4/225	62/18862	0.006406381	0.044763753	0.033237182	6375/9636/3662/3123	4
BP	GO:1902686	mitochondrial outer membrane permeabilization involved in programmed cell death	4/225	62/18862	0.006406381	0.044763753	0.033237182	841/7533/10105/3002	4
BP	GO:0035794	positive regulation of mitochondrial membrane permeability	4/225	64/18862	0.007163566	0.048434032	0.035962372	841/7533/10105/3002	4
BP	GO:0042093	T-helper cell differentiation	4/225	64/18862	0.007163566	0.048434032	0.035962372	64332/3662/84807/3123	4
BP	GO:0048247	lymphocyte chemotaxis	4/225	64/18862	0.007163566	0.048434032	0.035962372	6375/6846/6348/6351	4
BP	GO:0032768	regulation of monooxygenase activity	4/225	65/18862	0.007563362	0.049835739	0.037003142	3553/2208/7124/3458	4
BP	GO:0050795	regulation of behavior	4/225	65/18862	0.007563362	0.049835739	0.037003142	8013/463/6348/5730	4

BP	GO:0070265	necrotic cell death	4/225	65/18862	0.007563362	0.049835739	0.037003142	841/57162/10105/7124	4
BP	GO:0071479	cellular response to ionizing radiation	4/225	65/18862	0.007563362	0.049835739	0.037003142	388/1647/1026/1958	4
BP	GO:2000343	positive regulation of chemokine (C-X-C motif) ligand 2 production	3/225	10/18862	0.000188933	0.003334631	0.002475971	255231/7124/972	3
BP	GO:0035747	natural killer cell chemotaxis	3/225	11/18862	0.000257499	0.004282108	0.003179474	6375/6348/6351	3
BP	GO:0045657	positive regulation of monocyte differentiation	3/225	11/18862	0.000257499	0.004282108	0.003179474	3725/972/3123	3
BP	GO:0046598	positive regulation of viral entry into host cell	3/225	11/18862	0.000257499	0.004282108	0.003179474	972/3123/3956	3
BP	GO:0075294	positive regulation by symbiont of entry into host	3/225	11/18862	0.000257499	0.004282108	0.003179474	972/3123/3956	3
BP	GO:0031392	regulation of prostaglandin biosynthetic process	3/225	13/18862	0.000438522	0.006501292	0.004827224	3553/2171/972	3
BP	GO:0043922	negative regulation by host of viral transcription	3/225	14/18862	0.000553219	0.007626168	0.005662446	3725/6348/6351	3
BP	GO:0045651	positive regulation of macrophage differentiation	3/225	14/18862	0.000553219	0.007626168	0.005662446	841/10221/5196	3

BP	GO:00517<br>70	positive regulation of nitric-oxide synthase biosynthetic process	3/225	15/18862	0.00068545<br>6	0.00872221<br>4	0.00647626<br>2	2208/10135/3458	3
BP	GO:20012<br>79	regulation of unsaturated fatty acid biosynthetic process	3/225	15/18862	0.00068545<br>6	0.00872221<br>4	0.00647626<br>2	3553/2171/972	3
BP	GO:00517<br>09	regulation of killing of cells of other organism	3/225	16/18862	0.00083623<br>9	0.01026679<br>8	0.00762311<br>9	841/2208/3458	3
BP	GO:00307<br>30	sequestering of triglyceride	3/225	17/18862	0.00100653<br>4	0.01163064<br>5	0.00863577<br>9	51099/3553/7124	3
BP	GO:00718<br>50	mitotic cell cycle arrest	3/225	17/18862	0.00100653<br>4	0.01163064<br>5	0.00863577<br>9	28984/1647/1026	3
BP	GO:00725<br>67	chemokine (C-X-C motif) ligand 2 production	3/225	17/18862	0.00100653<br>4	0.01163064<br>5	0.00863577<br>9	255231/7124/972	3
BP	GO:01500<br>78	positive regulation of neuroinflammatory response	3/225	17/18862	0.00100653<br>4	0.01163064<br>5	0.00863577<br>9	3553/6348/7124	3
BP	GO:19015<br>50	regulation of endothelial cell development	3/225	17/18862	0.00100653<br>4	0.01163064<br>5	0.00863577<br>9	7414/3553/7124	3
BP	GO:19031<br>40	regulation of establishment of endothelial barrier	3/225	17/18862	0.00100653<br>4	0.01163064<br>5	0.00863577<br>9	7414/3553/7124	3
BP	GO:20003<br>41	regulation of chemokine (C-X-C motif) ligand 2 production	3/225	17/18862	0.00100653<br>4	0.01163064<br>5	0.00863577<br>9	255231/7124/972	3

BP	GO:0070269	pyroptosis	3/225	18/18862	0.00119726	0.012975826	0.009634579	841/3001/3002	3
BP	GO:0070293	renal absorption	3/225	18/18862	0.00119726	0.012975826	0.009634579	360/3043/6446	3
BP	GO:0010829	negative regulation of glucose transmembrane transport	3/225	19/18862	0.0014093	0.014507906	0.010772151	3553/2171/7124	3
BP	GO:0034138	toll-like receptor 3 signaling pathway	3/225	19/18862	0.0014093	0.014507906	0.010772151	7128/841/57162	3
BP	GO:0043031	negative regulation of macrophage activation	3/225	19/18862	0.0014093	0.014507906	0.010772151	3949/11326/8530	3
BP	GO:0051767	nitric-oxide synthase biosynthetic process	3/225	20/18862	0.001643494	0.016346521	0.012137327	2208/10135/3458	3
BP	GO:0051769	regulation of nitric-oxide synthase biosynthetic process	3/225	20/18862	0.001643494	0.016346521	0.012137327	2208/10135/3458	3
BP	GO:1903978	regulation of microglial cell activation	3/225	20/18862	0.001643494	0.016346521	0.012137327	3949/6348/8530	3
BP	GO:0035455	response to interferon-alpha	3/225	21/18862	0.001900643	0.018494491	0.013732199	3437/10410/3433	3
BP	GO:0045655	regulation of monocyte differentiation	3/225	21/18862	0.001900643	0.018494491	0.013732199	3725/972/3123	3
BP	GO:1900118	negative regulation of execution phase of apoptosis	3/225	21/18862	0.001900643	0.018494491	0.013732199	100462981/100463486/100463498	3

BP	GO:0002710	negative regulation of T cell mediated immunity	3/225	22/18862	0.002181511	0.020334737	0.015098586	6375/3821/3824	3
BP	GO:0002922	positive regulation of humoral immune response	3/225	22/18862	0.002181511	0.020334737	0.015098586	3553/2208/7124	3
BP	GO:0045649	regulation of macrophage differentiation	3/225	22/18862	0.002181511	0.020334737	0.015098586	841/10221/5196	3
BP	GO:0045662	negative regulation of myoblast differentiation	3/225	24/18862	0.002817278	0.02498237	0.018549463	3399/116173/7124	3
BP	GO:0033622	integrin activation	3/225	25/18862	0.003173523	0.027206053	0.020200552	83706/51237/7094	3
BP	GO:0002719	negative regulation of cytokine production involved in immune response	3/225	26/18862	0.003556182	0.029647428	0.022013279	6375/11213/7124	3
BP	GO:0061082	myeloid leukocyte cytokine production	3/225	26/18862	0.003556182	0.029647428	0.022013279	8013/11213/972	3
BP	GO:1900739	regulation of protein insertion into mitochondrial membrane involved in apoptotic signaling pathway	3/225	26/18862	0.003556182	0.029647428	0.022013279	841/7533/3002	3
BP	GO:1900740	positive regulation of protein insertion into mitochondrial membrane involved in apoptotic signaling pathway	3/225	26/18862	0.003556182	0.029647428	0.022013279	841/7533/3002	3

BP	GO:0010818	T cell chemotaxis	3/225	27/18862	0.003965844	0.031878891	0.023670145	6375/6846/6348	3
BP	GO:0032703	negative regulation of interleukin-2 production	3/225	28/18862	0.004403063	0.034424944	0.025560595	7128/6375/11326	3
BP	GO:0071280	cellular response to copper ion	3/225	28/18862	0.004403063	0.034424944	0.025560595	351/4502/4493	3
BP	GO:0060055	angiogenesis involved in wound healing	3/225	29/18862	0.004868365	0.037213578	0.027631161	7128/7124/2876	3
BP	GO:0072539	T-helper 17 cell differentiation	3/225	29/18862	0.004868365	0.037213578	0.027631161	64332/3662/84807	3
BP	GO:1900117	regulation of execution phase of apoptosis	3/225	29/18862	0.004868365	0.037213578	0.027631161	100462981/100463486/100463498	3
BP	GO:0001844	protein insertion into mitochondrial membrane involved in apoptotic signaling pathway	3/225	30/18862	0.005362243	0.039737442	0.029505136	841/7533/3002	3
BP	GO:0007176	regulation of epidermal growth factor-activated receptor activity	3/225	30/18862	0.005362243	0.039737442	0.029505136	374/351/1839	3
BP	GO:0046685	response to arsenic-containing substance	3/225	30/18862	0.005362243	0.039737442	0.029505136	1026/467/10105	3
BP	GO:0050850	positive regulation of calcium-mediated signaling	3/225	30/18862	0.005362243	0.039737442	0.029505136	6348/7124/6351	3
BP	GO:2000406	positive regulation of T cell migration	3/225	30/18862	0.005362243	0.039737442	0.029505136	6375/6846/351	3

BP	GO:0019835	cytolysis	3/225	31/18862	0.005885159	0.042718372	0.031718482	3001/23531/3002	3
BP	GO:0002861	regulation of inflammatory response to antigenic stimulus	3/225	32/18862	0.006437549	0.044763753	0.033237182	7124/2876/3123	3
BP	GO:0010743	regulation of macrophage derived foam cell differentiation	3/225	32/18862	0.006437549	0.044763753	0.033237182	19/3685/5196	3
BP	GO:0032673	regulation of interleukin-4 production	3/225	32/18862	0.006437549	0.044763753	0.033237182	8140/3662/3123	3
BP	GO:1903902	positive regulation of viral life cycle	3/225	32/18862	0.006437549	0.044763753	0.033237182	972/3123/3956	3
BP	GO:0032633	interleukin-4 production	3/225	33/18862	0.007019818	0.048068167	0.035690716	8140/3662/3123	3
BP	GO:0045589	regulation of regulatory T cell differentiation	3/225	33/18862	0.007019818	0.048068167	0.035690716	1493/3458/3123	3
BP	GO:0072538	T-helper 17 type immune response	3/225	33/18862	0.007019818	0.048068167	0.035690716	64332/3662/84807	3
BP	GO:0001660	fever generation	2/225	10/18862	0.005985321	0.042754235	0.031745111	3553/7124	2
BP	GO:0030656	regulation of vitamin metabolic process	2/225	10/18862	0.005985321	0.042754235	0.031745111	7124/3458	2
BP	GO:0031652	positive regulation of heat generation	2/225	10/18862	0.005985321	0.042754235	0.031745111	3553/7124	2
BP	GO:0032823	regulation of natural killer cell differentiation	2/225	10/18862	0.005985321	0.042754235	0.031745111	639/257101	2

BP	GO:0042368	vitamin D biosynthetic process	2/225	10/18862	0.005985321	0.042754235	0.031745111	7124/3458	2
BP	GO:0051712	positive regulation of killing of cells of other organism	2/225	10/18862	0.005985321	0.042754235	0.031745111	2208/3458	2
BP	GO:2000318	positive regulation of T-helper 17 type immune response	2/225	10/18862	0.005985321	0.042754235	0.031745111	64332/84807	2
BP	GO:0019371	cyclooxygenase pathway	2/225	11/18862	0.00725817	0.048434032	0.035962372	5742/5730	2
BP	GO:0035457	cellular response to interferon-alpha	2/225	11/18862	0.00725817	0.048434032	0.035962372	3437/3433	2
BP	GO:0098883	synapse pruning	2/225	11/18862	0.00725817	0.048434032	0.035962372	1524/712	2
BP	GO:1900222	negative regulation of amyloid-beta clearance	2/225	11/18862	0.00725817	0.048434032	0.035962372	7124/3458	2
CC	GO:0030139	endocytic vesicle	21/224	307/19520	6.49E-11	5.14E-09	3.96E-09	3118/3949/3127/9788/1493/3039/3040/3115/3043/19/3685/10578/9332/6678/3119/1839/122618/3117/972/3113/3123	21
CC	GO:0009897	external side of plasma membrane	21/224	402/19520	8.48E-09	2.99E-07	2.30E-07	8140/3560/1524/2214/931/3949/1493/100423062/3685/7293/925/2352/2208/3674/9332/3678/7056/7049/7124/972/3123	21
CC	GO:0034774	secretory granule lumen	17/224	322/19520	2.03E-07	4.59E-06	3.54E-06	83706/7414/5476/87/2512/351/2352/2162/6279/5196/6678/2171/6280/6282/7076/23406/1521	17
CC	GO:0060205	cytoplasmic vesicle lumen	17/224	326/19520	2.42E-07	5.11E-06	3.93E-06	83706/7414/5476/87/2512/351/2352/2162/6279/5196/6678/2171/6280/6282/7076/23406/1521	17
CC	GO:0031983	vesicle lumen	17/224	328/19520	2.63E-07	5.22E-06	4.02E-06	83706/7414/5476/87/2512/351/2352/2162/6279/5196/6678/2171/6280/6282/7076/23406/1521	17

CC	GO:0005774	vacuolar membrane	17/224	431/19520	1.07E-05	0.000121066	9.33E-05	8140/3118/2040/5476/10410/3949/3127/23531/3115/290/3119/7056/3117/2357/972/3113/3123	17
CC	GO:0005765	lysosomal membrane	16/224	378/19520	8.17E-06	9.96E-05	7.67E-05	8140/3118/2040/5476/10410/3949/3127/23531/3115/290/3119/3117/2357/972/3113/3123	16
CC	GO:0098852	lytic vacuole membrane	16/224	378/19520	8.17E-06	9.96E-05	7.67E-05	8140/3118/2040/5476/10410/3949/3127/23531/3115/290/3119/3117/2357/972/3113/3123	16
CC	GO:0030135	coated vesicle	15/224	295/19520	1.72E-06	2.73E-05	2.10E-05	3118/51429/374/3949/3127/1493/55704/351/3115/3119/1839/3117/972/3113/3123	15
CC	GO:0031252	cell leading edge	15/224	411/19520	8.57E-05	0.000876224	0.000675025	3611/51429/87/9788/55704/7168/351/23150/3685/7094/23499/3678/6285/6282/6277	15
CC	GO:0030136	clathrin-coated vesicle	13/224	192/19520	3.54E-07	6.60E-06	5.08E-06	3118/51429/374/3949/3127/1493/3115/3119/1839/3117/972/3113/3123	13
CC	GO:0030133	transport vesicle	13/224	402/19520	0.000793788	0.006621865	0.005101354	3118/94120/374/3127/55704/351/3115/79772/3119/3117/972/3113/3123	13
CC	GO:0001726	ruffle	12/224	175/19520	8.80E-07	1.55E-05	1.19E-05	51429/87/9788/7168/23150/3685/7094/23499/3678/6285/6282/6277	12
CC	GO:0030055	cell-substrate junction	12/224	423/19520	0.003683666	0.026539142	0.02044523	3611/83706/7414/388/87/9124/3304/2273/3685/7094/3674/3678	12
CC	GO:0045334	clathrin-coated endocytic vesicle	11/224	57/19520	4.13E-11	4.36E-09	3.36E-09	3118/3949/3127/1493/3115/3119/1839/3117/972/3113/3123	11
CC	GO:0030665	clathrin-coated vesicle membrane	11/224	117/19520	1.06E-07	3.06E-06	2.36E-06	3118/374/3949/3127/3115/3119/1839/3117/972/3113/3123	11
CC	GO:0030666	endocytic vesicle membrane	11/224	163/19520	2.96E-06	4.26E-05	3.28E-05	3118/3949/3127/3115/9332/3119/1839/3117/972/3113/3123	11
CC	GO:0030662	coated vesicle membrane	11/224	182/19520	8.53E-06	0.000100168	7.72E-05	3118/374/3949/3127/3115/3119/1839/3117/972/3113/3123	11

CC	GO:0005802	trans-Golgi network	11/224	251/19520	0.00016025	0.001494098	0.001151024	3118/51429/3127/351/3115/3119/122618/3117/972/3113/3123	11
CC	GO:0005770	late endosome	11/224	275/19520	0.00035138	0.0030941	0.002383634	84174/388/153020/10410/255231/3949/3127/23531/972/3123/8530	11
CC	GO:0030669	clathrin-coated endocytic vesicle membrane	10/224	39/19520	1.54E-11	2.44E-09	1.88E-09	3118/3949/3127/3115/3119/1839/3117/972/3113/3123	10
CC	GO:0030134	COPII-coated ER to Golgi transport vesicle	10/224	94/19520	1.26E-07	3.34E-06	2.57E-06	3118/374/3127/351/3115/3119/3117/972/3113/3123	10
CC	GO:0032588	trans-Golgi network membrane	10/224	95/19520	1.40E-07	3.41E-06	2.63E-06	3118/3127/351/3115/3119/122618/3117/972/3113/3123	10
CC	GO:0030658	transport vesicle membrane	10/224	206/19520	0.000139173	0.001336907	0.001029927	3118/374/3127/3115/79772/3119/3117/972/3113/3123	10
CC	GO:0098576	lumenal side of membrane	9/224	36/19520	2.11E-10	1.28E-08	9.88E-09	3118/5476/3127/3115/3119/3117/972/3113/3123	9
CC	GO:0012507	ER to Golgi transport vesicle membrane	9/224	62/19520	3.52E-08	1.11E-06	8.59E-07	3118/374/3127/3115/3119/3117/972/3113/3123	9
CC	GO:0031091	platelet alpha granule	9/224	91/19520	1.02E-06	1.70E-05	1.31E-05	83706/87/351/2162/3674/340205/5196/6678/7076	9
CC	GO:0030176	integral component of endoplasmic reticulum membrane	9/224	157/19520	8.56E-05	0.000876224	0.000675025	3118/3127/3115/91319/3119/3117/972/3113/3123	9
CC	GO:0031227	intrinsic component of endoplasmic reticulum membrane	9/224	165/19520	0.000125111	0.001239381	0.000954794	3118/3127/3115/91319/3119/3117/972/3113/3123	9
CC	GO:0030016	myofibril	9/224	224/19520	0.001164026	0.00946144	0.007288906	3611/7414/9172/288/87/9124/7168/7273/23345	9

CC	GO:0043292	contractile fiber	9/224	231/19520	0.00144083	0.011418578	0.008796646	3611/7414/9172/288/87/9124/7168/7273/23345	9
CC	GO:0042613	MHC class II protein complex	8/224	16/19520	3.16E-12	1.00E-09	7.71E-10	3118/3127/3115/3119/3117/972/3113/3123	8
CC	GO:0042611	MHC protein complex	8/224	25/19520	2.43E-10	1.28E-08	9.88E-09	3118/3127/3115/3119/3117/972/3113/3123	8
CC	GO:0071556	integral component of lumenal side of endoplasmic reticulum membrane	8/224	29/19520	9.26E-10	3.67E-08	2.83E-08	3118/3127/3115/3119/3117/972/3113/3123	8
CC	GO:0098553	lumenal side of endoplasmic reticulum membrane	8/224	29/19520	9.26E-10	3.67E-08	2.83E-08	3118/3127/3115/3119/3117/972/3113/3123	8
CC	GO:0072562	blood microparticle	8/224	146/19520	0.000287993	0.002608391	0.002009452	2040/3304/3039/3040/3043/3048/2162/3674	8
CC	GO:0030017	sarcomere	8/224	203/19520	0.002442704	0.018436602	0.014203193	3611/9172/288/87/9124/7168/7273/23345	8
CC	GO:0016323	basolateral plasma membrane	8/224	211/19520	0.003096282	0.022826079	0.01758476	8140/3248/360/288/952/3949/30061/366	8
CC	GO:0009925	basal plasma membrane	8/224	240/19520	0.006663903	0.040624179	0.031296063	8140/3248/360/288/952/3949/30061/366	8
CC	GO:0031093	platelet alpha granule lumen	7/224	67/19520	1.16E-05	0.000126419	9.74E-05	83706/87/351/2162/5196/6678/7076	7
CC	GO:0101002	ficolin-1-rich granule	7/224	185/19520	0.005602864	0.036257425	0.027932003	2495/7414/3304/3043/2204/2357/23406	7

CC	GO:0031902	late endosome membrane	6/224	140/19520	0.00560446	0.036257425	0.027932003	388/10410/255231/3127/23531/3123	6
CC	GO:0071682	endocytic vesicle lumen	5/224	20/19520	2.56E-06	3.87E-05	2.98E-05	3039/3040/3043/10578/6678	5
CC	GO:0032587	ruffle membrane	5/224	95/19520	0.004778266	0.033660228	0.025931174	7168/3685/7094/23499/3678	5
CC	GO:0031838	haptoglobin-hemoglobin complex	4/224	11/19520	5.23E-06	7.21E-05	5.55E-05	3039/3040/3043/3048	4
CC	GO:0005833	hemoglobin complex	4/224	12/19520	7.77E-06	9.96E-05	7.67E-05	3039/3040/3043/3048	4
CC	GO:0098636	protein complex involved in cell adhesion	4/224	36/19520	0.00074549	0.006387033	0.004920444	1493/3685/3674/3678	4
CC	GO:0014704	intercalated disc	4/224	48/19520	0.002212082	0.017103174	0.013175947	7414/7533/288/87	4
CC	GO:0001725	stress fiber	4/224	64/19520	0.006265414	0.038943846	0.030001568	3611/87/9124/7168	4
CC	GO:0097517	contractile actin filament bundle	4/224	64/19520	0.006265414	0.038943846	0.030001568	3611/87/9124/7168	4
CC	GO:0044291	cell-cell contact zone	4/224	69/19520	0.00815561	0.048779779	0.037578977	7414/7533/288/87	4
CC	GO:0008305	integrin complex	3/224	31/19520	0.005288224	0.036257425	0.027932003	3685/3674/3678	3
CC	GO:0044754	autolysosome	2/224	10/19520	0.005552236	0.036257425	0.027932003	2495/2512	2
MF	GO:0030546	signaling receptor activator activity	18/217	492/18337	2.40E-05	0.000958225	0.00081323	9235/374/6375/6846/351/3553/10501/1890/116173/6348/10135/5196/1839/7124/6351/7076/3458/4050	18

MF	GO:0048018	receptor ligand activity	17/217	486/18337	7.06E-05	0.002027468	0.00172068	9235/374/6375/6846/3553/10501/1890/116173/6348/10135/5196/1839/7124/6351/7076/3458/4050	17
MF	GO:0003779	actin binding	16/217	439/18337	7.17E-05	0.002027468	0.00172068	7414/7533/9172/87/9124/9788/55704/7168/4650/7094/23499/7273/8507/23406/6275/23345	16
MF	GO:0005125	cytokine activity	14/217	235/18337	8.42E-07	6.74E-05	5.72E-05	9235/374/6375/6846/3553/116173/6348/10135/5196/7124/6351/7076/3458/4050	14
MF	GO:0005126	cytokine receptor binding	14/217	270/18337	4.31E-06	0.000251799	0.000213698	841/6375/6846/55704/3553/7185/6348/5196/3678/7049/7124/6351/3458/4050	14
MF	GO:0033218	amide binding	14/217	391/18337	0.000244038	0.005578021	0.004733978	8140/3949/30061/3127/3115/290/2352/712/3119/3117/10105/972/3113/3123	14
MF	GO:0042277	peptide binding	13/217	315/18337	0.000101751	0.002713357	0.002302783	8140/3949/30061/3127/3115/290/712/3119/3117/10105/972/3113/3123	13
MF	GO:0140375	immune receptor activity	12/217	136/18337	7.79E-08	9.34E-06	7.93E-06	3118/3560/1524/51348/3821/3119/3117/3824/2357/972/3113/3123	12
MF	GO:0031625	ubiquitin protein ligase binding	10/217	293/18337	0.002650209	0.031974536	0.027136284	51429/7414/841/9636/3725/3304/1026/7185/5577/10221	10
MF	GO:0044389	ubiquitin-like protein ligase binding	10/217	312/18337	0.004136954	0.044127511	0.037450322	51429/7414/841/9636/3725/3304/1026/7185/5577/10221	10
MF	GO:0043177	organic acid binding	9/217	114/18337	8.53E-06	0.000409548	0.000347577	3039/3399/3040/3043/3048/2352/6279/2171/6280	9
MF	GO:0003823	antigen binding	9/217	165/18337	0.000156733	0.003959564	0.003360419	8140/3127/3115/100423062/3119/3117/3824/3113/3123	9
MF	GO:0005178	integrin binding	8/217	142/18337	0.000291965	0.005796749	0.004919609	83706/9636/87/3553/3685/7094/2208/3678	8
MF	GO:0051015	actin filament binding	8/217	208/18337	0.003407871	0.038041354	0.032285097	9172/87/7168/7094/23499/7273/23406/23345	8

MF	GO:0042605	peptide antigen binding	7/217	32/18337	7.72E-08	9.34E-06	7.93E-06	8140/3127/3115/3119/3117/3113/3123	7
MF	GO:0016684	oxidoreductase activity, acting on peroxide as acceptor	7/217	57/18337	4.72E-06	0.000251799	0.000213698	3039/3040/3043/3048/5742/143686/2876	7
MF	GO:0016209	antioxidant activity	7/217	86/18337	7.18E-05	0.002027468	0.00172068	3039/3040/3043/3048/5742/6280/2876	7
MF	GO:0002020	protease binding	7/217	131/18337	0.000953422	0.014749228	0.012517437	7128/3949/3685/91319/7273/7124/7076	7
MF	GO:0070851	growth factor receptor binding	7/217	141/18337	0.001462257	0.019496763	0.016546595	374/931/55704/351/3553/3678/1839	7
MF	GO:0023023	MHC protein complex binding	6/217	26/18337	4.84E-07	4.65E-05	3.95E-05	3821/931/925/3824/972/3123	6
MF	GO:0004601	peroxidase activity	6/217	53/18337	3.70E-05	0.001366095	0.001159383	3039/3040/3043/3048/5742/2876	6
MF	GO:0001618	virus receptor activity	6/217	76/18337	0.000281128	0.005796749	0.004919609	3949/3304/3685/7293/290/3678	6
MF	GO:0140272	exogenous protein binding	6/217	77/18337	0.000301914	0.005796749	0.004919609	3949/3304/3685/7293/290/3678	6
MF	GO:0048306	calcium-dependent protein binding	6/217	84/18337	0.000483344	0.008285904	0.007032116	6279/6285/6280/6282/6277/6275	6
MF	GO:0032395	MHC class II receptor activity	5/217	10/18337	5.32E-08	9.34E-06	7.93E-06	3118/3119/3117/3113/3123	5
MF	GO:0050786	RAGE receptor binding	5/217	10/18337	5.32E-08	9.34E-06	7.93E-06	6279/6285/6280/2357/6275	5

MF	GO:0005154	epidermal growth factor receptor binding	5/217	33/18337	4.02E-05	0.001376696	0.001168381	374/931/55704/3678/1839	5
MF	GO:0008009	chemokine activity	5/217	49/18337	0.00027668	0.005796749	0.004919609	6375/6846/6348/5196/6351	5
MF	GO:0042379	chemokine receptor binding	5/217	69/18337	0.001347008	0.018473251	0.015677957	6375/6846/6348/5196/6351	5
MF	GO:0031720	haptoglobin binding	4/217	10/18337	3.79E-06	0.000251799	0.000213698	3039/3040/3043/3048	4
MF	GO:0005344	oxygen carrier activity	4/217	14/18337	1.74E-05	0.000759252	0.000644365	3039/3040/3043/3048	4
MF	GO:0019865	immunoglobulin binding	4/217	24/18337	0.000168316	0.004039587	0.003428333	2214/931/2208/2204	4
MF	GO:0005164	tumor necrosis factor receptor binding	4/217	31/18337	0.00046712	0.008285904	0.007032116	841/7185/7124/4050	4
MF	GO:0005504	fatty acid binding	4/217	37/18337	0.00092761	0.014749228	0.012517437	6279/5730/2171/6280	4
MF	GO:0019825	oxygen binding	4/217	39/18337	0.001134107	0.016496097	0.013999977	3039/3040/3043/3048	4
MF	GO:0042287	MHC protein binding	4/217	40/18337	0.001248554	0.017626643	0.014959453	925/3824/972/926	4
MF	GO:0048020	CCR chemokine receptor binding	4/217	46/18337	0.002109583	0.027367563	0.023226419	6375/6846/6348/6351	4
MF	GO:0015026	coreceptor activity	4/217	48/18337	0.002469617	0.031195161	0.02647484	3685/925/7049/926	4

MF	GO:0032813	tumor necrosis factor receptor superfamily binding	4/217	49/18337	0.002664545	0.031974536	0.027136284	841/7185/7124/4050	4
MF	GO:0016763	transferase activity, transferring pentosyl groups	4/217	50/18337	0.002869767	0.033597269	0.028513472	54625/165631/1890/10135	4
MF	GO:0050840	extracellular matrix binding	4/217	55/18337	0.004059362	0.044127511	0.037450322	3685/3674/6678/3956	4
MF	GO:0035259	glucocorticoid receptor binding	3/217	12/18337	0.000332329	0.006135311	0.005206941	7533/8013/3164	3
MF	GO:0044548	S100 protein binding	3/217	14/18337	0.000540319	0.00894321	0.007589961	6285/6282/6277	3
MF	GO:0023026	MHC class II protein complex binding	3/217	17/18337	0.000983282	0.014749228	0.012517437	931/972/3123	3
MF	GO:0001965	G-protein alpha-subunit binding	3/217	25/18337	0.003102032	0.035451794	0.030087378	5996/55704/5997	3
KEGG	hsa04640	Hematopoietic cell lineage	18/135	99/8096	2.53E-14	5.39E-12	3.97E-12	3118/931/952/3127/3115/3553/925/290/2208/2815/3674/3678/3119/3117/7124/3113/926/3123	18
KEGG	hsa04612	Antigen processing and presentation	15/135	78/8096	1.75E-12	1.24E-10	9.13E-11	3118/3821/3304/3127/3115/925/3119/3117/3824/7124/972/3458/3113/926/3123	15
KEGG	hsa05169	Epstein-Barr virus infection	15/135	202/8096	1.19E-06	1.80E-05	1.33E-05	3118/7128/841/9636/1647/3725/1026/3127/3115/2208/3119/3117/7124/3113/3123	15
KEGG	hsa05323	Rheumatoid arthritis	14/135	93/8096	3.15E-10	1.34E-08	9.88E-09	3118/3725/3127/1493/3115/3553/6348/3119/3117/7124/3458/3113/3123/4050	14
KEGG	hsa04060	Cytokine-cytokine receptor interaction	14/135	295/8096	0.000384502	0.002824104	0.002079523	9235/3560/1524/6375/9560/6846/3553/7293/6348/5196/7124/6351/3458/4050	14
KEGG	hsa05332	Graft-versus-host disease	13/135	42/8096	7.26E-14	7.73E-12	5.69E-12	3118/3821/3127/3115/3553/3119/3117/3824/7124/3458/3113/3123/3002	13

KEGG	hsa05145	Toxoplasmosis	13/135	112/8096	3.59E-08	7.65E-07	5.63E-07	3118/841/3949/3304/3127/3115/3119/3117/10105/7124/3458/3113/3123	13
KEGG	hsa05152	Tuberculosis	13/135	180/8096	8.65E-06	9.70E-05	7.14E-05	3118/2214/841/3127/3115/3553/3119/3117/7124/972/3458/3113/3123	13
KEGG	hsa05166	Human T-cell leukemia virus 1 infection	13/135	222/8096	7.93E-05	0.000649877	0.000478535	3118/3560/3725/1026/3127/3115/7094/1958/3119/3117/7124/3113/3123	13
KEGG	hsa05140	Leishmaniasis	12/135	77/8096	4.12E-09	1.25E-07	9.24E-08	3118/2214/3725/3127/3115/3553/3119/3117/7124/3458/3113/3123	12
KEGG	hsa04659	Th17 cell differentiation	12/135	108/8096	2.02E-07	3.58E-06	2.64E-06	3118/3560/3725/3127/3662/3115/3553/3119/3117/3458/3113/3123	12
KEGG	hsa05322	Systemic lupus erythematosus	12/135	136/8096	2.47E-06	3.50E-05	2.58E-05	3118/2214/87/3127/3115/712/3119/3117/7124/3458/3113/3123	12
KEGG	hsa04145	Phagosome	12/135	152/8096	7.88E-06	9.33E-05	6.87E-05	3118/2214/3127/3115/81027/3685/3678/2204/3119/3117/3113/3123	12
KEGG	hsa05164	Influenza A	12/135	172/8096	2.76E-05	0.000267155	0.000196719	3118/4599/841/3127/3115/3553/3119/3117/7124/3458/3113/3123	12
KEGG	hsa04940	Type I diabetes mellitus	11/135	43/8096	6.71E-11	3.57E-09	2.63E-09	3118/3127/3115/3553/3119/3117/7124/3458/3113/3123/3002	11
KEGG	hsa05321	Inflammatory bowel disease	11/135	65/8096	7.65E-09	2.04E-07	1.50E-07	3118/3725/3127/3115/3553/3119/3117/7124/3458/3113/3123	11
KEGG	hsa05150	Staphylococcus aureus infection	11/135	96/8096	4.86E-07	7.96E-06	5.86E-06	3118/2214/3127/3115/712/2204/3119/3117/2357/3113/3123	11
KEGG	hsa04514	Cell adhesion molecules	11/135	149/8096	3.59E-05	0.000318444	0.000234486	3118/3127/1493/3115/3685/925/3119/3117/3113/926/3123	11
KEGG	hsa05330	Allograft rejection	10/135	38/8096	3.78E-10	1.34E-08	9.88E-09	3118/3127/3115/3119/3117/7124/3458/3113/3123/3002	10
KEGG	hsa04658	Th1 and Th2 cell differentiation	10/135	92/8096	2.69E-06	3.57E-05	2.63E-05	3118/3560/3725/3127/3115/3119/3117/3458/3113/3123	10
KEGG	hsa05320	Autoimmune thyroid disease	9/135	53/8096	1.82E-07	3.52E-06	2.59E-06	3118/3127/1493/3115/3119/3117/3113/3123/3002	9
KEGG	hsa04061	Viral protein interaction with cytokine and cytokine receptor	9/135	100/8096	4.02E-05	0.000342664	0.00025232	3560/1524/6375/9560/6846/6348/5196/7124/6351	9

KEGG	hsa04062	Chemokine signaling pathway	9/135	192/8096	0.004709587	0.027111946	0.019963825	1524/3702/6375/9560/6846/2791/6348/5196/6351	9
KEGG	hsa05310	Asthma	8/135	31/8096	2.77E-08	6.56E-07	4.83E-07	3118/3127/3115/3119/3117/7124/3113/3123	8
KEGG	hsa05416	Viral myocarditis	8/135	60/8096	5.99E-06	7.50E-05	5.53E-05	3118/841/3127/3115/3119/3117/3113/3123	8
KEGG	hsa04657	IL-17 signaling pathway	8/135	94/8096	0.00016203	0.001278235	0.000941226	7128/841/3725/3553/6279/6280/7124/3458	8
KEGG	hsa04064	NF-kappa B signaling pathway	8/135	104/8096	0.000326137	0.002480968	0.001826855	7128/1647/9560/3553/7185/7124/6351/4050	8
KEGG	hsa04210	Apoptosis	8/135	136/8096	0.001909611	0.011621348	0.008557355	841/1647/3725/4000/7185/7124/1521/3002	8
KEGG	hsa04672	Intestinal immune network for IgA production	7/135	49/8096	1.47E-05	0.000156519	0.000115253	3118/3127/3115/3119/3117/3113/3123	7
KEGG	hsa05144	Malaria	7/135	50/8096	1.69E-05	0.000170945	0.000125875	3039/3040/3553/3043/3820/7124/3458	7
KEGG	hsa05410	Hypertrophic cardiomyopathy	7/135	90/8096	0.000725187	0.005148824	0.003791326	4000/7168/3685/3674/3678/7273/7124	7
KEGG	hsa05414	Dilated cardiomyopathy	7/135	96/8096	0.001065152	0.007318622	0.005389052	4000/7168/3685/3674/3678/7273/7124	7
KEGG	hsa05142	Chagas disease	7/135	102/8096	0.001519548	0.01011449	0.007447783	841/3725/3553/6348/712/7124/3458	7
KEGG	hsa04620	Toll-like receptor signaling pathway	7/135	104/8096	0.001700601	0.010653763	0.007844876	841/3725/9560/3553/6348/7124/6351	7
KEGG	hsa04660	T cell receptor signaling pathway	7/135	104/8096	0.001700601	0.010653763	0.007844876	3702/3725/1493/925/7124/3458/926	7
KEGG	hsa04650	Natural killer cell mediated cytotoxicity	7/135	131/8096	0.006170509	0.034587329	0.025468307	2214/3821/117157/3824/7124/3458/3002	7

KEGG	hsa05162	Measles	7/135	139/8096	0.008457005	0.044608434	0.032847328	3560/4599/7128/841/3725/3304/3553	7
KEGG	hsa05418	Fluid shear stress and atherosclerosis	7/135	139/8096	0.008457005	0.044608434	0.032847328	3725/3553/3685/3674/7056/7124/3458	7
KEGG	hsa05143	African trypanosomiasis	6/135	37/8096	2.92E-05	0.000270736	0.000199356	3039/3040/3553/3043/7124/3458	6
KEGG	hsa04978	Mineral absorption	5/135	60/8096	0.003135588	0.018552226	0.013660893	2495/30061/2512/4502/4493	5
KEGG	hsa05133	Pertussis	5/135	76/8096	0.0085866	0.044608434	0.032847328	3725/3553/712/3678/7124	5

(4) Monocyte

ONTOLOGY	ID	Description	Gene Ratio	BgRatio	pvalue	p.adjust	qvalue	geneID	Count
BP	GO:0001819	positive regulation of cytokine production	17/117	437/18862	1.68E-09	1.51E-06	1.09E-06	7057/8013/1191/4332/28984/3303/9308/1545/10288/3113/3553/1958/353514/5743/7124/3115/64581	17
BP	GO:2001233	regulation of apoptotic signaling pathway	16/117	348/18862	4.76E-10	1.29E-06	9.30E-07	7057/50486/51330/5329/1524/1191/3303/10105/3553/6280/120892/7124/7185/7128/3958/6279	16
BP	GO:0007159	leukocyte cell-cell adhesion	16/117	366/18862	9.90E-10	1.34E-06	9.68E-07	10875/64332/1524/8013/11326/9308/10288/3113/3553/3678/6280/7124/3115/3958/3123/6279	16

BP	GO:0022407	regulation of cell-cell adhesion	16/117	437/18862	1.24E-08	4.84E-06	3.49E-06	10875/64332/5329/8013/11326/28984/9308/10288/3113/3553/7124/3115/8728/3557/3958/3123	16
BP	GO:1903706	regulation of hemopoiesis	15/117	415/18862	4.35E-08	1.18E-05	8.51E-06	7057/9935/10875/64332/8013/3303/28959/9308/10288/55365/4208/7124/6688/3958/3123	15
BP	GO:0043312	neutrophil degranulation	15/117	485/18862	3.27E-07	3.99E-05	2.88E-05	10875/5329/4332/3303/6813/2495/7414/10288/6036/6280/2352/5265/5660/3958/6279	15
BP	GO:0002283	neutrophil activation involved in immune response	15/117	488/18862	3.53E-07	3.99E-05	2.88E-05	10875/5329/4332/3303/6813/2495/7414/10288/6036/6280/2352/5265/5660/3958/6279	15
BP	GO:0002446	neutrophil mediated immunity	15/117	499/18862	4.69E-07	4.65E-05	3.35E-05	10875/5329/4332/3303/6813/2495/7414/10288/6036/6280/2352/5265/5660/3958/6279	15
BP	GO:0042119	neutrophil activation	15/117	500/18862	4.81E-07	4.65E-05	3.35E-05	10875/5329/4332/3303/6813/2495/7414/10288/6036/6280/2352/5265/5660/3958/6279	15
BP	GO:0070661	leukocyte proliferation	14/117	312/18862	8.55E-09	4.64E-06	3.34E-06	1191/4332/11326/10288/3113/3553/7940/4208/3115/196/7128/1026/3958/3123	14
BP	GO:0002683	negative regulation of immune system process	14/117	403/18862	2.06E-07	3.17E-05	2.28E-05	7057/9935/10875/4332/11326/28959/10288/55365/7940/3949/7124/7128/3958/3123	14
BP	GO:0050867	positive regulation of cell activation	14/117	412/18862	2.69E-07	3.47E-05	2.50E-05	7057/64332/8013/9308/10288/3113/3553/353514/4208/120892/3115/64581/1026/3123	14

BP	GO:0002429	immune response-activating cell surface receptor signaling pathway	14/117	481/18862	1.70E-06	0.000111409	8.03E-05	64332/3119/8013/4332/3127/3113/2214/3117/974/4208/3115/3118/3958/3123	14
BP	GO:0002757	immune response-activating signal transduction	14/117	481/18862	1.70E-06	0.000111409	8.03E-05	64332/3119/8013/4332/3127/3113/2214/3117/974/4208/3115/3118/3958/3123	14
BP	GO:0070663	regulation of leukocyte proliferation	13/117	241/18862	3.34E-09	2.27E-06	1.63E-06	4332/11326/10288/3113/3553/7940/4208/3115/196/7128/1026/3958/3123	13
BP	GO:0002696	positive regulation of leukocyte activation	13/117	401/18862	1.25E-06	9.17E-05	6.61E-05	7057/64332/8013/9308/10288/3113/3553/4208/120892/3115/64581/1026/3123	13
BP	GO:0045785	positive regulation of cell adhesion	13/117	425/18862	2.38E-06	0.000140289	0.000101117	64332/5329/8013/9308/10288/3113/3553/3678/3611/7124/3115/8728/3123	13
BP	GO:0051090	regulation of DNA-binding transcription factor activity	13/117	444/18862	3.84E-06	0.000200757	0.0001447	1524/1191/28984/3303/1545/3553/6280/3397/7124/6446/7185/7128/6279	13

BP	GO:0002697	regulation of immune effector process	13/117	465/18862	6.33E-06	0.000286093	0.000206208	10875/64332/8013/1191/11326/6813/3553/117157/7124/64581/7128/3958/3123	13
BP	GO:0042110	T cell activation	13/117	474/18862	7.78E-06	0.000334744	0.000241275	9935/10875/64332/11326/9308/10288/3113/3553/1958/3115/64581/3958/3123	13
BP	GO:0050670	regulation of lymphocyte proliferation	12/117	221/18862	1.29E-08	4.84E-06	3.49E-06	4332/11326/10288/3113/3553/7940/4208/3115/196/1026/3958/3123	12
BP	GO:0032944	regulation of mononuclear cell proliferation	12/117	223/18862	1.43E-08	4.84E-06	3.49E-06	4332/11326/10288/3113/3553/7940/4208/3115/196/1026/3958/3123	12
BP	GO:0046651	lymphocyte proliferation	12/117	282/18862	1.88E-07	3.17E-05	2.28E-05	4332/11326/10288/3113/3553/7940/4208/3115/196/1026/3958/3123	12
BP	GO:0032943	mononuclear cell proliferation	12/117	285/18862	2.10E-07	3.17E-05	2.28E-05	4332/11326/10288/3113/3553/7940/4208/3115/196/1026/3958/3123	12
BP	GO:0050851	antigen receptor-mediated signaling pathway	12/117	323/18862	7.97E-07	6.76E-05	4.87E-05	64332/3119/4332/3127/3113/3117/974/4208/3115/3118/3958/3123	12
BP	GO:1903037	regulation of leukocyte cell-cell adhesion	12/117	330/18862	9.99E-07	7.97E-05	5.74E-05	10875/64332/8013/11326/9308/10288/3113/3553/7124/3115/3958/3123	12

BP	GO:0006959	humoral immune response	12/117	380/18862	4.30E-06	0.00021618	0.000155816	3119/1191/11326/28984/9308/3553/6280/4208/2920/7124/3123/6279	12
BP	GO:1903131	mononuclear cell differentiation	12/117	411/18862	9.52E-06	0.000385368	0.000277763	9935/10875/64332/28959/9308/10288/55365/3553/1958/974/6688/3123	12
BP	GO:0097191	extrinsic apoptotic signaling pathway	11/117	217/18862	1.08E-07	2.26E-05	1.63E-05	7057/50486/51330/3429/3303/3553/597/7124/7185/7128/3958	11
BP	GO:2001234	negative regulation of apoptotic signaling pathway	11/117	224/18862	1.49E-07	2.89E-05	2.08E-05	7057/5329/1524/1191/3303/10105/3553/120892/7124/7128/3958	11
BP	GO:0019882	antigen processing and presentation	11/117	234/18862	2.32E-07	3.31E-05	2.38E-05	7057/10875/3119/3127/10288/3113/3117/3115/3796/3118/3123	11
BP	GO:0022409	positive regulation of cell-cell adhesion	11/117	276/18862	1.19E-06	8.95E-05	6.45E-05	64332/5329/8013/9308/10288/3113/3553/7124/3115/8728/3123	11
BP	GO:0097193	intrinsic apoptotic signaling pathway	11/117	283/18862	1.52E-06	0.000108281	7.80E-05	5329/1191/3303/10105/1545/6280/597/120892/7124/1026/6279	11

BP	GO:0032496	response to lipopolysaccharide	11/117	326/18862	5.91E-06	0.000271577	0.000195745	1524/10288/3553/6280/4208/2920/7124/7056/7128/3248/6279	11
BP	GO:1901342	regulation of vasculature development	11/117	341/18862	9.03E-06	0.00037695	0.000271695	7057/1524/28984/1545/3553/3678/3397/388/7124/7128/23166	11
BP	GO:0002237	response to molecule of bacterial origin	11/117	346/18862	1.04E-05	0.000407073	0.000293407	1524/10288/3553/6280/4208/2920/7124/7056/7128/3248/6279	11
BP	GO:0031349	positive regulation of defense response	11/117	361/18862	1.54E-05	0.000565178	0.000407365	64332/4332/3553/353514/6280/117157/5743/4208/120892/7124/6279	11
BP	GO:0050727	regulation of inflammatory response	11/117	366/18862	1.75E-05	0.000623759	0.000449588	64332/3553/353514/6280/5743/3949/120892/7124/7128/3123/6279	11
BP	GO:0032102	negative regulation of response to external stimulus	11/117	394/18862	3.44E-05	0.001045482	0.000753555	7057/10875/5329/10501/5055/11326/3949/7124/7056/7128/3123	11
BP	GO:0001667	ameboidal-type cell migration	11/117	473/18862	0.000174601	0.003243266	0.002337659	3164/7057/10501/28984/1545/3397/5743/4208/9839/388/7124	11
BP	GO:0060284	regulation of cell development	11/117	485/18862	0.000216777	0.003751728	0.002704145	1524/10501/7414/3553/3611/3397/3949/120892/7533/7124/64581	11

BP	GO:0050866	negative regulation of cell activation	10/117	200/18862	4.78E-07	4.65E-05	3.35E-05	10875/4332/11326/10288/7940/3949/7056/7128/3958/3123	10
BP	GO:0072593	reactive oxygen species metabolic process	10/117	281/18862	1.01E-05	0.000401803	0.000289609	7057/1524/1191/1545/3553/5743/120892/7124/64581/1026	10
BP	GO:0050863	regulation of T cell activation	10/117	327/18862	3.70E-05	0.001090992	0.000786358	10875/64332/11326/9308/10288/3113/3553/3115/3958/3123	10
BP	GO:0045765	regulation of angiogenesis	10/117	335/18862	4.54E-05	0.001268524	0.000914318	7057/1524/28984/1545/3553/3678/388/7124/7128/23166	10
BP	GO:0051251	positive regulation of lymphocyte activation	10/117	356/18862	7.54E-05	0.001792969	0.001292324	64332/9308/10288/3113/3553/4208/3115/64581/1026/3123	10
BP	GO:0010631	epithelial cell migration	10/117	357/18862	7.71E-05	0.001819171	0.001311209	3164/7057/28984/1545/3397/5743/4208/9839/388/7124	10
BP	GO:0030098	lymphocyte differentiation	10/117	358/18862	7.89E-05	0.001845747	0.001330365	9935/10875/64332/9308/10288/3553/1958/974/6688/3123	10
BP	GO:0090132	epithelium migration	10/117	360/18862	8.27E-05	0.001900035	0.001369494	3164/7057/28984/1545/3397/5743/4208/9839/388/7124	10
BP	GO:0090130	tissue migration	10/117	365/18862	9.26E-05	0.002084436	0.001502406	3164/7057/28984/1545/3397/5743/4208/9839/388/7124	10
BP	GO:0001818	negative regulation of	10/117	367/18862	9.69E-05	0.002085685	0.001503306	7057/7850/1524/11326/28984/9308/353514/7124/7128/3123	10

		cytokine production							
BP	GO:0045862	positive regulation of proteolysis	10/117	367/18862	9.69E-05	0.002085685	0.001503306	444/1191/3303/3553/255488/6280/120892/7124/64581/6279	10
BP	GO:0050678	regulation of epithelial cell proliferation	10/117	374/18862	0.000113158	0.002360656	0.001701498	3164/7057/8013/374/28984/3397/4208/7124/9788/7128	10
BP	GO:0040013	negative regulation of locomotion	10/117	377/18862	0.000120802	0.002500873	0.001802562	7057/1524/10501/28984/7414/1545/4208/388/7124/79772	10
BP	GO:0050673	epithelial cell proliferation	10/117	428/18862	0.000334921	0.005342981	0.003851077	3164/7057/8013/374/28984/3397/4208/7124/9788/7128	10
BP	GO:0006979	response to oxidative stress	10/117	444/18862	0.000446809	0.006769531	0.004879296	8013/3303/10105/1545/143686/5743/120892/388/79772/7128	10
BP	GO:0071900	regulation of protein serine/threonine kinase activity	10/117	492/18862	0.000983345	0.011695702	0.008429948	7057/1848/28984/3553/2180/120892/1844/7124/7128/1026	10
BP	GO:0002504	antigen processing and presentation of peptide or polysaccharide	9/117	104/18862	1.67E-08	5.04E-06	3.63E-06	7057/3119/3127/3113/3117/3115/3796/3118/3123	9

		antigen via MHC class II							
BP	GO:2001236	regulation of extrinsic apoptotic signaling pathway	9/117	154/18862	4.97E-07	4.65E-05	3.35E-05	7057/50486/51330/3303/3553/7124/7185/7128/3958	9
BP	GO:0002695	negative regulation of leukocyte activation	9/117	182/18862	2.01E-06	0.000123749	8.92E-05	10875/4332/11326/10288/7940/3949/7128/3958/3123	9
BP	GO:2000377	regulation of reactive oxygen species metabolic process	9/117	192/18862	3.12E-06	0.000177107	0.000127654	7057/1524/1191/1545/3553/5743/7124/64581/1026	9
BP	GO:0034341	response to interferon-gamma	9/117	197/18862	3.85E-06	0.000200757	0.0001447	3119/10410/3127/3113/3117/8638/3115/3118/3123	9
BP	GO:0050852	T cell receptor signaling pathway	9/117	204/18862	5.12E-06	0.000239242	0.000172439	64332/3119/3127/3113/3117/3115/3118/3958/3123	9
BP	GO:1903039	positive regulation of leukocyte cell-cell adhesion	9/117	234/18862	1.54E-05	0.000565178	0.000407365	64332/8013/9308/10288/3113/3553/7124/3115/3123	9

BP	GO:0045637	regulation of myeloid cell differentiation	9/117	258/18862	3.33E-05	0.001026099	0.000739585	7057/9935/8013/3303/4208/7124/6688/3958/3123	9
BP	GO:0051091	positive regulation of DNA-binding transcription factor activity	9/117	266/18862	4.22E-05	0.001205757	0.000869077	1524/1191/28984/3303/3553/6280/7124/7185/6279	9
BP	GO:0043542	endothelial cell migration	9/117	278/18862	5.94E-05	0.001579737	0.001138632	3164/7057/28984/1545/3397/5743/4208/388/7124	9
BP	GO:1902105	regulation of leukocyte differentiation	9/117	279/18862	6.11E-05	0.001608238	0.001159175	9935/10875/64332/28959/9308/10288/55365/7124/3123	9
BP	GO:0007162	negative regulation of cell adhesion	9/117	295/18862	9.36E-05	0.002084436	0.001502406	7057/10875/11326/28984/1545/10288/3557/3958/3123	9
BP	GO:0030336	negative regulation of cell migration	9/117	330/18862	0.000217191	0.003751728	0.002704145	7057/1524/28984/7414/1545/4208/388/7124/79772	9
BP	GO:2000146	negative regulation of cell motility	9/117	345/18862	0.00030141	0.004942553	0.00356246	7057/1524/28984/7414/1545/4208/388/7124/79772	9
BP	GO:0051271	negative regulation of cellular	9/117	352/18862	0.000349096	0.005504355	0.003967391	7057/1524/28984/7414/1545/4208/388/7124/79772	9

		component movement								
BP	GO:0009615	response to virus	9/117	359/18862	0.000402859	0.006207694	0.004474339	10875/3429/1191/10410/6036/7124/8638/10964/7128		9
BP	GO:0031331	positive regulation of cellular catabolic process	9/117	384/18862	0.000653637	0.008775561	0.006325189	1191/3303/3553/255488/143686/3949/120892/7124/7128		9
BP	GO:0019216	regulation of lipid metabolic process	9/117	402/18862	0.000903791	0.010845491	0.007817139	50486/8013/3553/1958/5743/3949/2180/7124/5660		9
BP	GO:0030099	myeloid cell differentiation	9/117	419/18862	0.00120681	0.013524251	0.009747918	7057/9935/8013/3303/4208/7124/6688/3958/3123		9
BP	GO:0052548	regulation of endopeptidase activity	9/117	426/18862	0.001353355	0.014919914	0.010753874	7057/444/5329/5055/6280/5265/7124/64581/6279		9
BP	GO:0009896	positive regulation of catabolic process	9/117	450/18862	0.00196848	0.019626901	0.014146543	1191/3303/3553/255488/143686/3949/120892/7124/7128		9
BP	GO:0052547	regulation of peptidase activity	9/117	455/18862	0.002121138	0.020692543	0.01491463	7057/444/5329/5055/6280/5265/7124/64581/6279		9
BP	GO:0016311	dephosphorylation	9/117	491/18862	0.003520532	0.027357259	0.019718379	1848/10288/27071/4208/120892/1844/7124/26469/3958		9

BP	GO:0060333	interferon-gamma-mediated signaling pathway	8/117	91/18862	9.53E-08	2.15E-05	1.55E-05	3119/3127/3113/3117/8638/3115/3118/3123	8
BP	GO:0019886	antigen processing and presentation of exogenous peptide antigen via MHC class II	8/117	99/18862	1.84E-07	3.17E-05	2.28E-05	3119/3127/3113/3117/3115/3796/3118/3123	8
BP	GO:0002495	antigen processing and presentation of peptide antigen via MHC class II	8/117	103/18862	2.51E-07	3.40E-05	2.45E-05	3119/3127/3113/3117/3115/3796/3118/3123	8
BP	GO:0032652	regulation of interleukin-1 production	8/117	119/18862	7.61E-07	6.66E-05	4.80E-05	7850/1524/4332/1958/353514/7124/64581/7128	8
BP	GO:0032612	interleukin-1 production	8/117	126/18862	1.18E-06	8.95E-05	6.45E-05	7850/1524/4332/1958/353514/7124/64581/7128	8
BP	GO:0061041	regulation of wound healing	8/117	131/18862	1.58E-06	0.000109784	7.91E-05	7057/51330/5329/5055/7124/7056/64581/7128	8

BP	GO:0050729	positive regulation of inflammatory response	8/117	133/18862	1.77E-06	0.000111626	8.05E-05	64332/3553/353514/6280/5743/120892/7124/6279	8
BP	GO:0051250	negative regulation of lymphocyte activation	8/117	153/18862	5.04E-06	0.000239242	0.000172439	10875/4332/11326/10288/7940/7128/3958/3123	8
BP	GO:0051092	positive regulation of NF-kappaB transcription factor activity	8/117	159/18862	6.70E-06	0.000297681	0.00021456	1524/1191/3303/3553/6280/7124/7185/6279	8
BP	GO:1903034	regulation of response to wounding	8/117	164/18862	8.41E-06	0.000356231	0.000256762	7057/51330/5329/5055/7124/7056/64581/7128	8
BP	GO:0002478	antigen processing and presentation of exogenous peptide antigen	8/117	177/18862	1.47E-05	0.000552387	0.000398146	3119/3127/3113/3117/3115/3796/3118/3123	8
BP	GO:0071346	cellular response to interferon-gamma	8/117	177/18862	1.47E-05	0.000552387	0.000398146	3119/3127/3113/3117/8638/3115/3118/3123	8

BP	GO:0019884	antigen processing and presentation of exogenous antigen	8/117	185/18862	2.02E-05	0.000700142	0.000504643	3119/3127/3113/3117/3115/3796/3118/3123	8
BP	GO:0048002	antigen processing and presentation of peptide antigen	8/117	194/18862	2.84E-05	0.000895386	0.00064537	3119/3127/3113/3117/3115/3796/3118/3123	8
BP	GO:0002699	positive regulation of immune effector process	8/117	219/18862	6.70E-05	0.001651639	0.001190457	64332/8013/6813/3553/117157/7124/64581/3123	8
BP	GO:0033002	muscle cell proliferation	8/117	222/18862	7.37E-05	0.001768439	0.001274643	7057/8013/3720/4208/7124/7128/1026/3248	8
BP	GO:0045732	positive regulation of protein catabolic process	8/117	225/18862	8.09E-05	0.00187581	0.001352034	1191/3303/3553/255488/3949/120892/7124/7128	8
BP	GO:0071216	cellular response to biotic stimulus	8/117	233/18862	0.000103173	0.002185981	0.001575597	1524/10288/3553/4208/2920/7124/64581/7128	8
BP	GO:0030217	T cell differentiation	8/117	246/18862	0.000150002	0.002885152	0.00207954	9935/10875/64332/9308/10288/3553/1958/3123	8

BP	GO:0006470	protein dephosphorylation	8/117	325/18862	0.000956045	0.011421996	0.008232668	1848/10288/27071/120892/1844/7124/26469/3958	8
BP	GO:0048545	response to steroid hormone	8/117	330/18862	0.001054419	0.01207171	0.008700965	7057/8013/8031/7533/7124/6446/3557/1026	8
BP	GO:0019058	viral life cycle	8/117	348/18862	0.00147769	0.016159255	0.011647157	3429/10410/3303/3678/3949/7124/8638/3123	8
BP	GO:0002460	adaptive immune response based on somatic recombination of immune receptors built from immunoglobulin superfamily domains	8/117	367/18862	0.002060841	0.020323636	0.014648732	64332/3119/1191/3553/4208/7124/7128/3123	8
BP	GO:0042176	regulation of protein catabolic process	8/117	383/18862	0.002681093	0.023876805	0.01720976	1191/3303/3553/255488/3949/120892/7124/7128	8
BP	GO:0050804	modulation of chemical	8/117	405/18862	0.00376255	0.028545075	0.020574525	1524/10288/3553/4208/120892/7533/7124/79772	8

		synaptic transmission							
BP	GO:0099177	regulation of trans-synaptic signaling	8/117	406/18862	0.003818706	0.028608648	0.020620346	1524/10288/3553/4208/120892/7533/7124/79772	8
BP	GO:0051051	negative regulation of transport	8/117	438/18862	0.005985308	0.037144517	0.026772771	7057/10105/10288/3553/120892/7124/79772/3958	8
BP	GO:0051346	negative regulation of hydrolase activity	8/117	456/18862	0.007558663	0.042441186	0.030590468	7057/5329/5055/10105/120892/5265/7124/3958	8
BP	GO:0023061	signal release	8/117	475/18862	0.009539308	0.049560541	0.035721908	6813/3553/4208/120892/7124/3557/79772/3123	8
BP	GO:0006809	nitric oxide biosynthetic process	7/117	73/18862	3.43E-07	3.99E-05	2.88E-05	1524/1191/1545/3553/5743/7124/64581	7
BP	GO:0046209	nitric oxide metabolic process	7/117	77/18862	4.95E-07	4.65E-05	3.35E-05	1524/1191/1545/3553/5743/7124/64581	7
BP	GO:2001057	reactive nitrogen species metabolic process	7/117	78/18862	5.41E-07	4.89E-05	3.53E-05	1524/1191/1545/3553/5743/7124/64581	7

BP	GO:2000379	positive regulation of reactive oxygen species metabolic process	7/117	101/18862	3.13E-06	0.000177107	0.000127654	7057/1191/3553/5743/7124/64581/1026	7
BP	GO:0032651	regulation of interleukin-1 beta production	7/117	103/18862	3.57E-06	0.000194801	0.000140407	1524/4332/1958/353514/7124/64581/7128	7
BP	GO:0032611	interleukin-1 beta production	7/117	108/18862	4.90E-06	0.000237472	0.000171164	1524/4332/1958/353514/7124/64581/7128	7
BP	GO:1903409	reactive oxygen species biosynthetic process	7/117	123/18862	1.16E-05	0.00044831	0.00032313	1524/1191/1545/3553/5743/7124/64581	7
BP	GO:0055076	transition metal ion homeostasis	7/117	139/18862	2.56E-05	0.00084737	0.000610762	2495/6280/8031/30061/4493/51312/6279	7
BP	GO:0048660	regulation of smooth muscle cell proliferation	7/117	160/18862	6.29E-05	0.00161142	0.001161468	7057/8013/4208/7124/7128/1026/3248	7
BP	GO:2001242	regulation of intrinsic apoptotic	7/117	160/18862	6.29E-05	0.00161142	0.001161468	5329/1191/3303/10105/6280/120892/6279	7

		signaling pathway							
BP	GO:0048659	smooth muscle cell proliferation	7/117	162/18862	6.81E-05	0.001662797	0.0011985	7057/8013/4208/7124/7128/1026/3248	7
BP	GO:0042129	regulation of T cell proliferation	7/117	168/18862	8.56E-05	0.001949809	0.00140537	11326/10288/3113/3553/3115/3958/3123	7
BP	GO:0001659	temperature homeostasis	7/117	171/18862	9.56E-05	0.002085685	0.001503306	50486/3553/1958/3397/5743/2180/7124	7
BP	GO:0043534	blood vessel endothelial cell migration	7/117	175/18862	0.00011044	0.002321815	0.001673502	3164/7057/28984/3397/5743/4208/7124	7
BP	GO:0022408	negative regulation of cell-cell adhesion	7/117	189/18862	0.000177895	0.003281977	0.002365561	10875/11326/28984/10288/3557/3958/3123	7
BP	GO:0042098	T cell proliferation	7/117	195/18862	0.000215507	0.003751728	0.002704145	11326/10288/3113/3553/3115/3958/3123	7
BP	GO:0071222	cellular response to lipopolysaccharide	7/117	197/18862	0.000229382	0.003863883	0.002784983	1524/10288/3553/4208/2920/7124/7128	7
BP	GO:0071219	cellular response to	7/117	209/18862	0.000328544	0.005272248	0.003800095	1524/10288/3553/4208/2920/7124/7128	7

		molecule of bacterial origin							
BP	GO:0050870	positive regulation of T cell activation	7/117	212/18862	0.000358049	0.005596392	0.004033729	64332/9308/10288/3113/3553/3115/3123	7
BP	GO:0000302	response to reactive oxygen species	7/117	224/18862	0.000498031	0.007300869	0.005262271	8013/10105/1545/143686/120892/388/7128	7
BP	GO:2000116	regulation of cysteine-type endopeptidase activity	7/117	230/18862	0.000582765	0.008188912	0.005902348	7057/444/5329/6280/7124/64581/6279	7
BP	GO:0051607	defense response to virus	7/117	260/18862	0.001193762	0.013433543	0.009682537	10875/3429/10410/6036/8638/10964/7128	7
BP	GO:0140546	defense response to symbiont	7/117	260/18862	0.001193762	0.013433543	0.009682537	10875/3429/10410/6036/8638/10964/7128	7
BP	GO:0030522	intracellular receptor signaling pathway	7/117	274/18862	0.00161229	0.017490123	0.012606411	3164/8013/3303/8031/7533/196/7128	7
BP	GO:0050890	cognition	7/117	276/18862	0.001680386	0.017527718	0.012633509	1524/10288/3678/3949/4208/7124/6446	7
BP	GO:0042063	gliogenesis	7/117	283/18862	0.00193653	0.019456217	0.014023519	1524/1191/3553/6280/3949/7124/6279	7

BP	GO:0071356	cellular response to tumor necrosis factor	7/117	296/18862	0.002491707	0.022752552	0.016399429	7057/51330/3303/3611/7124/7185/7128	7
BP	GO:0034599	cellular response to oxidative stress	7/117	299/18862	0.002635683	0.023747417	0.017116501	8013/3303/10105/1545/120892/388/7128	7
BP	GO:1901214	regulation of neuron death	7/117	302/18862	0.002785983	0.024419909	0.017601215	1524/8013/1191/1958/4208/120892/7124	7
BP	GO:0060326	cell chemotaxis	7/117	306/18862	0.002996533	0.025369114	0.018285376	3164/7057/1524/6280/2920/3958/6279	7
BP	GO:0071902	positive regulation of protein serine/threonine kinase activity	7/117	311/18862	0.003276646	0.026451466	0.019065507	7057/1848/28984/3553/2180/120892/7124	7
BP	GO:0034612	response to tumor necrosis factor	7/117	320/18862	0.003830851	0.028620574	0.020628942	7057/51330/3303/3611/7124/7185/7128	7
BP	GO:0007596	blood coagulation	7/117	342/18862	0.00548514	0.035250471	0.025407594	7057/5329/5055/7414/3611/5265/7056	7
BP	GO:0070997	neuron death	7/117	342/18862	0.00548514	0.035250471	0.025407594	1524/8013/1191/1958/4208/120892/7124	7
BP	GO:0007599	hemostasis	7/117	346/18862	0.005835966	0.036807303	0.026529717	7057/5329/5055/7414/3611/5265/7056	7

BP	GO:0045861	negative regulation of proteolysis	7/117	346/18862	0.005835966	0.036807303	0.026529717	7057/7850/5329/5055/120892/5265/7124	7
BP	GO:0050817	coagulation	7/117	347/18862	0.005926243	0.036862318	0.02656937	7057/5329/5055/7414/3611/5265/7056	7
BP	GO:0062197	cellular response to chemical stress	7/117	347/18862	0.005926243	0.036862318	0.02656937	8013/3303/10105/1545/120892/388/7128	7
BP	GO:0050767	regulation of neurogenesis	7/117	348/18862	0.006017562	0.037259425	0.026855594	1524/10501/3553/3397/3949/7533/7124	7
BP	GO:0010038	response to metal ion	7/117	352/18862	0.006393413	0.038499861	0.027749667	7057/10105/4493/4208/120892/4205/6279	7
BP	GO:0006909	phagocytosis	7/117	381/18862	0.009664185	0.04963877	0.035778294	7057/122618/3553/2214/3949/7124/64581	7
BP	GO:0045428	regulation of nitric oxide biosynthetic process	6/117	59/18862	1.73E-06	0.000111409	8.03E-05	1524/1191/3553/5743/7124/64581	6
BP	GO:0080164	regulation of nitric oxide metabolic process	6/117	61/18862	2.10E-06	0.000126854	9.14E-05	1524/1191/3553/5743/7124/64581	6
BP	GO:0070664	negative regulation of leukocyte proliferation	6/117	88/18862	1.79E-05	0.000631564	0.000455215	4332/11326/10288/7940/7128/3123	6

BP	GO:0070301	cellular response to hydrogen peroxide	6/117	90/18862	2.04E-05	0.000700142	0.000504643	8013/10105/1545/120892/388/7128	6
BP	GO:0032602	chemokine production	6/117	95/18862	2.78E-05	0.000885779	0.000638446	3553/1958/6280/7124/64581/6279	6
BP	GO:1903426	regulation of reactive oxygen species biosynthetic process	6/117	99/18862	3.51E-05	0.001045482	0.000753555	1524/1191/3553/5743/7124/64581	6
BP	GO:0042116	macrophage activation	6/117	102/18862	4.15E-05	0.00119806	0.00086353	7057/1191/11326/3949/120892/7124	6
BP	GO:2001237	negative regulation of extrinsic apoptotic signaling pathway	6/117	103/18862	4.39E-05	0.001239418	0.000893339	7057/3303/3553/7124/7128/3958	6
BP	GO:0002526	acute inflammatory response	6/117	107/18862	5.43E-05	0.001459373	0.001051877	9332/3553/5743/5265/7124/6279	6
BP	GO:0032649	regulation of interferon-gamma production	6/117	107/18862	5.43E-05	0.001459373	0.001051877	3113/3553/7124/3115/64581/3123	6

BP	GO:0007041	lysosomal transport	6/117	111/18862	6.67E-05	0.001651639	0.001190457	1191/3303/8031/120892/388/5660	6
BP	GO:1903959	regulation of anion transmembrane transport	6/117	111/18862	6.67E-05	0.001651639	0.001190457	7057/8013/3553/2180/7124/4205	6
BP	GO:0032609	interferon-gamma production	6/117	112/18862	7.02E-05	0.001698939	0.00122455	3113/3553/7124/3115/64581/3123	6
BP	GO:0046916	cellular transition metal ion homeostasis	6/117	118/18862	9.38E-05	0.002084436	0.001502406	2495/6280/8031/30061/4493/6279	6
BP	GO:2001235	positive regulation of apoptotic signaling pathway	6/117	126/18862	0.000134637	0.002704709	0.001949481	7057/50486/51330/6280/7124/6279	6
BP	GO:0002576	platelet degranulation	6/117	128/18862	0.000146783	0.00287566	0.002072699	7057/1191/6813/7414/5265/5660	6
BP	GO:0050768	negative regulation of neurogenesis	6/117	133/18862	0.000180968	0.003316108	0.002390162	10501/3553/3397/3949/7533/7124	6
BP	GO:0042542	response to hydrogen peroxide	6/117	135/18862	0.000196283	0.003531827	0.002545646	8013/10105/1545/120892/388/7128	6

BP	GO:0050671	positive regulation of lymphocyte proliferation	6/117	135/18862	0.000196283	0.003531827	0.002545646	10288/3113/3553/4208/3115/1026	6
BP	GO:0032946	positive regulation of mononuclear cell proliferation	6/117	136/18862	0.000204316	0.003598077	0.002593397	10288/3113/3553/4208/3115/1026	6
BP	GO:0051961	negative regulation of nervous system development	6/117	138/18862	0.000221157	0.003756602	0.002707658	10501/3553/3397/3949/7533/7124	6
BP	GO:0070665	positive regulation of leukocyte proliferation	6/117	148/18862	0.000322482	0.005205785	0.00375219	10288/3113/3553/4208/3115/1026	6
BP	GO:1903364	positive regulation of cellular protein catabolic process	6/117	150/18862	0.000346524	0.005495757	0.003961194	1191/3303/255488/3949/120892/7128	6
BP	GO:0010821	regulation of mitochondrion organization	6/117	151/18862	0.000359061	0.005596392	0.004033729	5329/1191/3303/10105/120892/7533	6

BP	GO:0007034	vacuolar transport	6/117	153/18862	0.000385197	0.005969449	0.004302619	1191/3303/8031/120892/388/5660	6
BP	GO:0002822	regulation of adaptive immune response based on somatic recombination of immune receptors built from immunoglobulin superfamily domains	6/117	155/18862	0.000412798	0.00632491	0.004558825	64332/3553/4208/7124/7128/3123	6
BP	GO:0032675	regulation of interleukin-6 production	6/117	158/18862	0.000457072	0.006886557	0.004963646	10288/3553/353514/7124/64581/7128	6
BP	GO:0034614	cellular response to reactive oxygen species	6/117	159/18862	0.000472625	0.007042635	0.005076142	8013/10105/1545/120892/388/7128	6
BP	GO:0032680	regulation of tumor necrosis factor production	6/117	160/18862	0.000488588	0.007240709	0.005218909	7057/1524/1191/353514/64581/7128	6

BP	GO:0032635	interleukin-6 production	6/117	162/18862	0.000521771	0.007526784	0.005425104	10288/3553/353514/7124/64581/7128	6
BP	GO:0032640	tumor necrosis factor production	6/117	162/18862	0.000521771	0.007526784	0.005425104	7057/1524/1191/353514/64581/7128	6
BP	GO:1903555	regulation of tumor necrosis factor superfamily cytokine production	6/117	164/18862	0.000556684	0.007891458	0.005687951	7057/1524/1191/353514/64581/7128	6
BP	GO:0071706	tumor necrosis factor superfamily cytokine production	6/117	167/18862	0.00061243	0.008304558	0.005985702	7057/1524/1191/353514/64581/7128	6
BP	GO:0002819	regulation of adaptive immune response	6/117	170/18862	0.000672418	0.008895594	0.006411705	64332/3553/4208/7124/7128/3123	6
BP	GO:0010721	negative regulation of cell development	6/117	174/18862	0.000759374	0.009806769	0.007068456	10501/3553/3397/3949/7533/7124	6
BP	GO:0033209	tumor necrosis factor-mediated	6/117	174/18862	0.000759374	0.009806769	0.007068456	51330/3303/3611/7124/7185/7128	6

		signaling pathway							
BP	GO:0045766	positive regulation of angiogenesis	6/117	175/18862	0.000782413	0.009961988	0.007180334	7057/1524/1545/3553/3678/388	6
BP	GO:1904018	positive regulation of vasculature development	6/117	175/18862	0.000782413	0.009961988	0.007180334	7057/1524/1545/3553/3678/388	6
BP	GO:0001959	regulation of cytokine-mediated signaling pathway	6/117	177/18862	0.00083011	0.010267316	0.007400406	7850/3303/7124/3557/7185/7128	6
BP	GO:0060759	regulation of response to cytokine stimulus	6/117	189/18862	0.001165248	0.01322239	0.009530344	7850/3303/7124/3557/7185/7128	6
BP	GO:0043281	regulation of cysteine-type endopeptidase activity involved in apoptotic process	6/117	205/18862	0.00176296	0.018248652	0.013153138	7057/5329/6280/7124/64581/6279	6

BP	GO:0002703	regulation of leukocyte mediated immunity	6/117	209/18862	0.001943373	0.019456217	0.014023519	6813/3553/117157/7124/64581/3123	6
BP	GO:0097529	myeloid leukocyte migration	6/117	218/18862	0.002400233	0.022364297	0.016119585	7057/1524/6280/2920/3958/6279	6
BP	GO:0010594	regulation of endothelial cell migration	6/117	231/18862	0.003197389	0.026436946	0.019055042	7057/28984/5743/4208/388/7124	6
BP	GO:0097305	response to alcohol	6/117	233/18862	0.003335684	0.026606982	0.019177599	6446/6688/196/1026/3248/6279	6
BP	GO:0016358	dendrite development	6/117	234/18862	0.003406482	0.026855751	0.019356905	3397/7940/4208/120892/7533/4205	6
BP	GO:0007611	learning or memory	6/117	235/18862	0.003478394	0.027189012	0.019597111	1524/10288/3678/3949/4208/6446	6
BP	GO:0009636	response to toxic substance	6/117	239/18862	0.003777408	0.028545075	0.020574525	1545/6280/5743/4493/196/1026	6
BP	GO:0071560	cellular response to transforming growth factor beta stimulus	6/117	251/18862	0.004789409	0.032449833	0.023388969	7057/1524/3303/3397/4208/3248	6
BP	GO:1903362	regulation of cellular protein	6/117	251/18862	0.004789409	0.032449833	0.023388969	1191/3303/255488/3949/120892/7128	6

		catabolic process							
BP	GO:0071559	response to transforming growth factor beta	6/117	257/18862	0.005364376	0.035250471	0.025407594	7057/1524/3303/3397/4208/3248	6
BP	GO:0043491	protein kinase B signaling	6/117	273/18862	0.007144126	0.04096167	0.029524072	7057/1524/374/3553/143686/7124	6
BP	GO:0007249	I-kappaB kinase/NF-kappaB signaling	6/117	282/18862	0.008315402	0.044942916	0.032393648	1524/3553/7124/7185/7128/3123	6
BP	GO:0002440	production of molecular mediator of immune response	6/117	286/18862	0.008877995	0.046751695	0.033697368	3119/8013/3553/7124/64581/3123	6
BP	GO:0002544	chronic inflammatory response	5/117	19/18862	9.14E-08	2.15E-05	1.55E-05	7057/6280/7124/7128/6279	5
BP	GO:0045429	positive regulation of nitric oxide biosynthetic process	5/117	38/18862	3.59E-06	0.000194801	0.000140407	1191/3553/5743/7124/64581	5

BP	GO:1904407	positive regulation of nitric oxide metabolic process	5/117	39/18862	4.10E-06	0.00020976	0.000151189	1191/3553/5743/7124/64581	5
BP	GO:0150077	regulation of neuroinflammatory response	5/117	40/18862	4.66E-06	0.000229869	0.000165683	3553/5743/3949/120892/7124	5
BP	GO:0150076	neuroinflammatory response	5/117	44/18862	7.54E-06	0.000329963	0.000237828	3553/5743/3949/120892/7124	5
BP	GO:0006953	acute-phase response	5/117	46/18862	9.43E-06	0.000385368	0.000277763	9332/3553/5743/5265/7124	5
BP	GO:1903428	positive regulation of reactive oxygen species biosynthetic process	5/117	55/18862	2.29E-05	0.000775817	0.000559188	1191/3553/5743/7124/64581	5
BP	GO:0032731	positive regulation of interleukin-1 beta production	5/117	56/18862	2.50E-05	0.000837218	0.000603444	4332/1958/353514/7124/64581	5
BP	GO:0032663	regulation of interleukin-2 production	5/117	59/18862	3.23E-05	0.001006596	0.000725528	11326/9308/3553/64581/7128	5

BP	GO:0034113	heterotypic cell-cell adhesion	5/117	60/18862	3.51E-05	0.001045482	0.000753555	10288/3553/3678/7124/3557	5
BP	GO:0032623	interleukin-2 production	5/117	61/18862	3.80E-05	0.001107991	0.00079861	11326/9308/3553/64581/7128	5
BP	GO:0032732	positive regulation of interleukin-1 production	5/117	64/18862	4.80E-05	0.001327771	0.000957022	4332/1958/353514/7124/64581	5
BP	GO:0032729	positive regulation of interferon-gamma production	5/117	65/18862	5.17E-05	0.001416884	0.001021252	3113/3553/7124/3115/64581	5
BP	GO:0061045	negative regulation of wound healing	5/117	74/18862	9.65E-05	0.002085685	0.001503306	7057/5329/5055/7124/7056	5
BP	GO:0050672	negative regulation of lymphocyte proliferation	5/117	81/18862	0.000148277	0.00287566	0.002072699	4332/11326/10288/7940/3123	5
BP	GO:0032945	negative regulation of mononuclear cell proliferation	5/117	82/18862	0.00015713	0.003000954	0.002163007	4332/11326/10288/7940/3123	5

BP	GO:1904705	regulation of vascular associated smooth muscle cell proliferation	5/117	83/18862	0.000166381	0.003133509	0.002258549	8013/4208/7124/1026/3248	5
BP	GO:1990874	vascular associated smooth muscle cell proliferation	5/117	83/18862	0.000166381	0.003133509	0.002258549	8013/4208/7124/1026/3248	5
BP	GO:0032755	positive regulation of interleukin-6 production	5/117	86/18862	0.000196647	0.003531827	0.002545646	10288/3553/353514/7124/64581	5
BP	GO:1903035	negative regulation of response to wounding	5/117	90/18862	0.000243324	0.004048439	0.002918006	7057/5329/5055/7124/7056	5
BP	GO:2001243	negative regulation of intrinsic apoptotic signaling pathway	5/117	95/18862	0.000312997	0.005082916	0.00366363	5329/1191/3303/10105/120892	5

BP	GO:0021782	glial cell development	5/117	112/18862	0.000666662	0.008895594	0.006411705	1191/6280/3949/7124/6279		5
BP	GO:0033559	unsaturated fatty acid metabolic process	5/117	115/18862	0.000751543	0.009806769	0.007068456	1545/3553/5743/2180/3248		5
BP	GO:0032874	positive regulation of stress-activated MAPK cascade	5/117	116/18862	0.000781554	0.009961988	0.007180334	3553/7124/6688/64581/7185		5
BP	GO:0008637	apoptotic mitochondrial changes	5/117	118/18862	0.000844252	0.010267316	0.007400406	5329/1191/3303/10105/7533		5
BP	GO:0043500	muscle adaptation	5/117	118/18862	0.000844252	0.010267316	0.007400406	8013/3720/3553/4000/4205		5
BP	GO:0070304	positive regulation of stress-activated protein kinase signaling cascade	5/117	118/18862	0.000844252	0.010267316	0.007400406	3553/7124/6688/64581/7185		5
BP	GO:0050868	negative regulation of T cell activation	5/117	119/18862	0.000876971	0.010617616	0.007652893	10875/11326/10288/3958/3123		5
BP	GO:0071621	granulocyte chemotaxis	5/117	124/18862	0.001054939	0.01207171	0.008700965	7057/6280/2920/3958/6279		5

BP	GO:0002705	positive regulation of leukocyte mediated immunity	5/117	125/18862	0.001093525	0.012460677	0.008981321	3553/117157/7124/64581/3123	5
BP	GO:0003158	endothelium development	5/117	137/18862	0.001642437	0.017527718	0.012633509	7414/3553/3397/388/7124	5
BP	GO:1903038	negative regulation of leukocyte cell-cell adhesion	5/117	138/18862	0.001695868	0.017621437	0.012701059	10875/11326/10288/3958/3123	5
BP	GO:0046718	viral entry into host cell	5/117	140/18862	0.001806547	0.018558165	0.013376227	10410/3303/3678/3949/3123	5
BP	GO:0071901	negative regulation of protein serine/threonine kinase activity	5/117	145/18862	0.00210634	0.020622364	0.014864047	1848/3553/1844/7128/1026	5
BP	GO:0045580	regulation of T cell differentiation	5/117	146/18862	0.002170403	0.021097252	0.015206333	10875/64332/9308/10288/3123	5
BP	GO:0097530	granulocyte migration	5/117	148/18862	0.002302778	0.021836135	0.015738901	7057/6280/2920/3958/6279	5
BP	GO:0043535	regulation of blood vessel	5/117	150/18862	0.002440941	0.022364297	0.016119585	7057/28984/5743/4208/7124	5

		endothelial cell migration							
BP	GO:0045834	positive regulation of lipid metabolic process	5/117	150/18862	0.002440941	0.022364297	0.016119585	8013/3553/5743/3949/7124	5
BP	GO:0050777	negative regulation of immune response	5/117	150/18862	0.002440941	0.022364297	0.016119585	10875/11326/7128/3958/3123	5
BP	GO:2001056	positive regulation of cysteine-type endopeptidase activity	5/117	151/18862	0.002512238	0.022863054	0.016479076	444/6280/7124/64581/6279	5
BP	GO:0044409	entry into host	5/117	153/18862	0.002659356	0.023876805	0.01720976	10410/3303/3678/3949/3123	5
BP	GO:0000187	activation of MAPK activity	5/117	154/18862	0.002735213	0.024084086	0.017359163	7057/1848/3553/120892/7124	5
BP	GO:1902107	positive regulation of leukocyte differentiation	5/117	154/18862	0.002735213	0.024084086	0.017359163	64332/9308/10288/7124/3123	5
BP	GO:1903708	positive regulation of hemopoiesis	5/117	154/18862	0.002735213	0.024084086	0.017359163	64332/9308/10288/7124/3123	5

BP	GO:0050680	negative regulation of epithelial cell proliferation	5/117	160/18862	0.003223687	0.026451466	0.019065507	7057/28984/4208/7124/9788	5
BP	GO:0045619	regulation of lymphocyte differentiation	5/117	175/18862	0.004715993	0.03237917	0.023338037	10875/64332/9308/10288/3123	5
BP	GO:0052126	movement in host environment	5/117	175/18862	0.004715993	0.03237917	0.023338037	10410/3303/3678/3949/3123	5
BP	GO:0048167	regulation of synaptic plasticity	5/117	176/18862	0.00483032	0.032449833	0.023388969	1524/10288/4208/7533/79772	5
BP	GO:0001936	regulation of endothelial cell proliferation	5/117	177/18862	0.004946606	0.032799986	0.02364135	3164/7057/28984/4208/7124	5
BP	GO:0032872	regulation of stress-activated MAPK cascade	5/117	181/18862	0.00543168	0.035250471	0.025407594	3553/7124/6688/64581/7185	5
BP	GO:0010950	positive regulation of endopeptidase activity	5/117	182/18862	0.005558022	0.035466719	0.025563459	444/6280/7124/64581/6279	5
BP	GO:0070302	regulation of stress-activated protein kinase	5/117	184/18862	0.005816916	0.036807303	0.026529717	3553/7124/6688/64581/7185	5

		signaling cascade							
BP	GO:0010469	regulation of signaling receptor activity	5/117	188/18862	0.006360044	0.038499861	0.027749667	100462981/374/100463486/4208/7124	5
BP	GO:0071248	cellular response to metal ion	5/117	189/18862	0.006501207	0.038665073	0.027868747	10105/4493/4208/120892/4205	5
BP	GO:0001935	endothelial cell proliferation	5/117	191/18862	0.006790112	0.039432083	0.028421588	3164/7057/28984/4208/7124	5
BP	GO:0050731	positive regulation of peptidyl-tyrosine phosphorylation	5/117	193/18862	0.007087903	0.040725408	0.029353782	374/3678/353514/7124/64581	5
BP	GO:0050864	regulation of B cell activation	5/117	193/18862	0.007087903	0.040725408	0.029353782	4332/4208/196/7128/1026	5
BP	GO:1901654	response to ketone	5/117	193/18862	0.007087903	0.040725408	0.029353782	7057/8031/6446/196/1026	5
BP	GO:1901888	regulation of cell junction assembly	5/117	195/18862	0.007394718	0.041733556	0.030080428	7057/7414/3553/4208/7124	5
BP	GO:0010952	positive regulation of	5/117	200/18862	0.008202121	0.044756847	0.032259534	444/6280/7124/64581/6279	5

		peptidase activity							
BP	GO:0046890	regulation of lipid biosynthetic process	5/117	203/18862	0.008714911	0.046617041	0.033600312	3553/1958/5743/3949/7124	5
BP	GO:0050679	positive regulation of epithelial cell proliferation	5/117	203/18862	0.008714911	0.046617041	0.033600312	3164/8013/374/3397/7128	5
BP	GO:0071456	cellular response to hypoxia	5/117	206/18862	0.009249505	0.048332676	0.034836896	28984/571/4000/1958/5743	5
BP	GO:0070486	leukocyte aggregation	4/117	13/18862	9.63E-07	7.91E-05	5.70E-05	8013/3553/6280/6279	4
BP	GO:0042730	fibrinolysis	4/117	25/18862	1.61E-05	0.000581564	0.000419176	7057/5329/5055/7056	4
BP	GO:0014002	astrocyte development	4/117	42/18862	0.000131194	0.002655204	0.0019138	6280/3949/7124/6279	4
BP	GO:0045601	regulation of endothelial cell differentiation	4/117	45/18862	0.000172154	0.003219872	0.002320798	7414/3553/3397/7124	4
BP	GO:0001774	microglial cell activation	4/117	47/18862	0.000204131	0.003598077	0.002593397	1191/3949/120892/7124	4
BP	GO:0097028	dendritic cell differentiation	4/117	47/18862	0.000204131	0.003598077	0.002593397	28959/10288/55365/6688	4

BP	GO:0030195	negative regulation of blood coagulation	4/117	48/18862	0.000221628	0.003756602	0.002707658	7057/5329/5055/7056	4
BP	GO:2001238	positive regulation of extrinsic apoptotic signaling pathway	4/117	48/18862	0.000221628	0.003756602	0.002707658	7057/50486/51330/7124	4
BP	GO:1900047	negative regulation of hemostasis	4/117	49/18862	0.00024018	0.004020793	0.002898079	7057/5329/5055/7056	4
BP	GO:0050819	negative regulation of coagulation	4/117	52/18862	0.000302531	0.004942553	0.00356246	7057/5329/5055/7056	4
BP	GO:0032757	positive regulation of interleukin-8 production	4/117	57/18862	0.000431023	0.006567042	0.004733348	3303/3553/7124/64581	4
BP	GO:2000351	regulation of endothelial cell apoptotic process	4/117	58/18862	0.00046075	0.006903617	0.004975942	7057/28984/7124/7128	4

BP	GO:0043030	regulation of macrophage activation	4/117	59/18862	0.000491911	0.007250339	0.00522585	7057/11326/3949/120892	4
BP	GO:0032653	regulation of interleukin-10 production	4/117	60/18862	0.000524544	0.007526784	0.005425104	9308/353514/64581/3123	4
BP	GO:0032722	positive regulation of chemokine production	4/117	60/18862	0.000524544	0.007526784	0.005425104	3553/1958/7124/64581	4
BP	GO:0030888	regulation of B cell proliferation	4/117	61/18862	0.000558687	0.007891458	0.005687951	4332/4208/196/1026	4
BP	GO:0010573	vascular endothelial growth factor production	4/117	62/18862	0.000594381	0.008266472	0.005958251	1545/3553/5743/7124	4
BP	GO:0032613	interleukin-10 production	4/117	62/18862	0.000594381	0.008266472	0.005958251	9308/353514/64581/3123	4
BP	GO:0010803	regulation of tumor necrosis factor-mediated signaling pathway	4/117	63/18862	0.000631665	0.00852276	0.006142977	3303/7124/7185/7128	4

BP	GO:0072577	endothelial cell apoptotic process	4/117	64/18862	0.000670577	0.008895594	0.006411705	7057/28984/7124/7128	4
BP	GO:0030193	regulation of blood coagulation	4/117	66/18862	0.000753446	0.009806769	0.007068456	7057/5329/5055/7056	4
BP	GO:0038034	signal transduction in absence of ligand	4/117	67/18862	0.000797482	0.010012829	0.007216978	3303/3553/597/7124	4
BP	GO:0097192	extrinsic apoptotic signaling pathway in absence of ligand	4/117	67/18862	0.000797482	0.010012829	0.007216978	3303/3553/597/7124	4
BP	GO:1900046	regulation of hemostasis	4/117	67/18862	0.000797482	0.010012829	0.007216978	7057/5329/5055/7056	4
BP	GO:0010611	regulation of cardiac muscle hypertrophy	4/117	68/18862	0.000843305	0.010267316	0.007400406	8013/3720/4000/4205	4
BP	GO:1900015	regulation of cytokine production involved in	4/117	68/18862	0.000843305	0.010267316	0.007400406	7850/122618/7124/64581	4

		inflammatory response							
BP	GO:000 2534	cytokine production involved in inflammatory response	4/117	69/18 862	0.00089 0954	0.01073 8969	0.00774 0361	7850/122618/7124/64581	4
BP	GO:001 4743	regulation of muscle hypertrophy	4/117	71/18 862	0.00099 1892	0.01169 5702	0.00842 9948	8013/3720/4000/4205	4
BP	GO:005 0818	regulation of coagulation	4/117	71/18 862	0.00099 1892	0.01169 5702	0.00842 9948	7057/5329/5055/7056	4
BP	GO:004 8708	astrocyte differentiation	4/117	72/18 862	0.00104 526	0.01206 2743	0.00869 4501	6280/3949/7124/6279	4
BP	GO:000 1937	negative regulation of endothelial cell proliferation	4/117	75/18 862	0.00121 7434	0.01358 7166	0.00979 3264	7057/28984/4208/7124	4
BP	GO:001 0827	regulation of glucose transmembrane transport	4/117	77/18 862	0.00134 2673	0.01486 2564	0.01071 2537	8013/3553/7124/4205	4
BP	GO:004 3537	negative regulation of blood vessel	4/117	78/18 862	0.00140 8547	0.01546 5507	0.01114 7123	7057/28984/4208/7124	4

		endothelial cell migration							
BP	GO:0002718	regulation of cytokine production involved in immune response	4/117	84/18862	0.001851849	0.01888051	0.013608565	8013/3553/7124/64581	4
BP	GO:0008625	extrinsic apoptotic signaling pathway via death domain receptors	4/117	84/18862	0.001851849	0.01888051	0.013608565	7057/7124/7128/3958	4
BP	GO:0046889	positive regulation of lipid biosynthetic process	4/117	85/18862	0.001934107	0.019456217	0.014023519	3553/5743/3949/7124	4
BP	GO:0055072	iron ion homeostasis	4/117	86/18862	0.0020188861	0.020021753	0.014431143	2495/8031/30061/51312	4
BP	GO:0032760	positive regulation of tumor necrosis factor production	4/117	87/18862	0.002106151	0.020622364	0.014864047	7057/1191/353514/64581	4

BP	GO:0002532	production of molecular mediator involved in inflammatory response	4/117	88/18862	0.002196013	0.021119103	0.015222083	7850/122618/7124/64581	4
BP	GO:0032436	positive regulation of proteasomal ubiquitin-dependent protein catabolic process	4/117	88/18862	0.002196013	0.021119103	0.015222083	1191/3303/255488/120892	4
BP	GO:0045582	positive regulation of T cell differentiation	4/117	88/18862	0.002196013	0.021119103	0.015222083	64332/9308/10288/3123	4
BP	GO:0032642	regulation of chemokine production	4/117	89/18862	0.002288487	0.02177676	0.015696105	3553/1958/7124/64581	4
BP	GO:1903557	positive regulation of tumor necrosis factor superfamily	4/117	90/18862	0.002383611	0.022364297	0.016119585	7057/1191/353514/64581	4

		cytokine production							
BP	GO:0002367	cytokine production involved in immune response	4/117	93/18862	0.00268526	0.023876805	0.01720976	8013/3553/7124/64581	4
BP	GO:1902882	regulation of response to oxidative stress	4/117	93/18862	0.00268526	0.023876805	0.01720976	8013/143686/120892/79772	4
BP	GO:1904035	regulation of epithelial cell apoptotic process	4/117	94/18862	0.002791361	0.024419909	0.017601215	7057/28984/7124/7128	4
BP	GO:0060337	type I interferon signaling pathway	4/117	95/18862	0.0029003	0.024875314	0.017929459	3429/10410/1958/8638	4
BP	GO:0003300	cardiac muscle hypertrophy	4/117	96/18862	0.003012115	0.025369114	0.018285376	8013/3720/4000/4205	4
BP	GO:0042100	B cell proliferation	4/117	96/18862	0.003012115	0.025369114	0.018285376	4332/4208/196/1026	4
BP	GO:0048661	positive regulation of smooth muscle	4/117	96/18862	0.003012115	0.025369114	0.018285376	7057/8013/7124/3248	4

		cell proliferation							
BP	GO:0071357	cellular response to type I interferon	4/117	96/18862	0.003012115	0.025369114	0.018285376	3429/10410/1958/8638	4
BP	GO:0010596	negative regulation of endothelial cell migration	4/117	97/18862	0.003126841	0.025932696	0.018691591	7057/28984/4208/7124	4
BP	GO:0032677	regulation of interleukin-8 production	4/117	97/18862	0.003126841	0.025932696	0.018691591	3303/3553/7124/64581	4
BP	GO:0043502	regulation of muscle adaptation	4/117	97/18862	0.003126841	0.025932696	0.018691591	8013/3720/4000/4205	4
BP	GO:0002824	positive regulation of adaptive immune response based on somatic recombination of immune receptors built from	4/117	99/18862	0.003365175	0.026607446	0.019177933	64332/3553/7124/3123	4

		immunoglobulin superfamily domains							
BP	GO:0014897	striated muscle hypertrophy	4/117	99/18862	0.003365175	0.026607446	0.019177933	8013/3720/4000/4205	4
BP	GO:0042102	positive regulation of T cell proliferation	4/117	99/18862	0.003365175	0.026607446	0.019177933	10288/3113/3553/3115	4
BP	GO:0008630	intrinsic apoptotic signaling pathway in response to DNA damage	4/117	100/18862	0.003488856	0.027189012	0.019597111	1191/597/7124/1026	4
BP	GO:0014896	muscle hypertrophy	4/117	101/18862	0.003615594	0.027777592	0.020021343	8013/3720/4000/4205	4
BP	GO:0034340	response to type I interferon	4/117	101/18862	0.003615594	0.027777592	0.020021343	3429/10410/1958/8638	4
BP	GO:0045621	positive regulation of lymphocyte differentiation	4/117	101/18862	0.003615594	0.027777592	0.020021343	64332/9308/10288/3123	4
BP	GO:1902106	negative regulation of	4/117	101/18862	0.003615594	0.027777592	0.020021343	9935/10875/28959/55365	4

		leukocyte differentiation							
BP	GO:0030593	neutrophil chemotaxis	4/117	103/18862	0.003878384	0.028738192	0.020713718	6280/2920/3958/6279	4
BP	GO:0032637	interleukin-8 production	4/117	103/18862	0.003878384	0.028738192	0.020713718	3303/3553/7124/64581	4
BP	GO:0070498	interleukin-1-mediated signaling pathway	4/117	103/18862	0.003878384	0.028738192	0.020713718	7850/3553/1958/3557	4
BP	GO:0002821	positive regulation of adaptive immune response	4/117	104/18862	0.004014508	0.029267056	0.021094909	64332/3553/7124/3123	4
BP	GO:0071868	cellular response to monoamine stimulus	4/117	104/18862	0.004014508	0.029267056	0.021094909	8013/3397/3274/120892	4
BP	GO:0071870	cellular response to catecholamine stimulus	4/117	104/18862	0.004014508	0.029267056	0.021094909	8013/3397/3274/120892	4
BP	GO:2000060	positive regulation of ubiquitin-	4/117	104/18862	0.004014508	0.029267056	0.021094909	1191/3303/255488/120892	4

		dependent protein catabolic process							
BP	GO:190 3707	negative regulation of hemopoiesis	4/117	105/1 8862	0.00415 383	0.03004 0499	0.02165 2386	9935/10875/28959/55365	4
BP	GO:000 7613	memory	4/117	107/1 8862	0.00444 221	0.03104 9677	0.02237 9774	1524/3678/3949/6446	4
BP	GO:000 2708	positive regulation of lymphocyte mediated immunity	4/117	108/1 8862	0.00459 1337	0.03168 3731	0.02283 6784	3553/117157/7124/3123	4
BP	GO:007 1867	response to monoamine	4/117	108/1 8862	0.00459 1337	0.03168 3731	0.02283 6784	8013/3397/3274/120892	4
BP	GO:007 1869	response to catecholamine	4/117	108/1 8862	0.00459 1337	0.03168 3731	0.02283 6784	8013/3397/3274/120892	4
BP	GO:190 1800	positive regulation of proteasomal protein catabolic process	4/117	109/1 8862	0.00474 3801	0.03240 6013	0.02335 7385	1191/3303/255488/120892	4

BP	GO:1904659	glucose transmembrane transport	4/117	109/18862	0.004743801	0.032406013	0.023357385	8013/3553/7124/4205	4
BP	GO:0002286	T cell activation involved in immune response	4/117	111/18862	0.005058872	0.033462585	0.024118934	10875/64332/3958/3123	4
BP	GO:0008645	hexose transmembrane transport	4/117	112/18862	0.005221546	0.034454581	0.024833938	8013/3553/7124/4205	4
BP	GO:0010633	negative regulation of epithelial cell migration	4/117	114/18862	0.005557337	0.035466719	0.025563459	7057/28984/4208/7124	4
BP	GO:0015749	monosaccharide transmembrane transport	4/117	114/18862	0.005557337	0.035466719	0.025563459	8013/3553/7124/4205	4
BP	GO:0034219	carbohydrate transmembrane transport	4/117	116/18862	0.005907268	0.036862318	0.02656937	8013/3553/7124/4205	4
BP	GO:1904019	epithelial cell apoptotic process	4/117	116/18862	0.005907268	0.036862318	0.02656937	7057/28984/7124/7128	4

BP	GO:0034766	negative regulation of ion transmembrane transport	4/117	119/18862	0.006459232	0.038499861	0.027749667	7057/10105/3553/7124	4
BP	GO:0045446	endothelial cell differentiation	4/117	119/18862	0.006459232	0.038499861	0.027749667	7414/3553/3397/7124	4
BP	GO:0120254	olefinic compound metabolic process	4/117	119/18862	0.006459232	0.038499861	0.027749667	1545/1958/5743/2180	4
BP	GO:0001676	long-chain fatty acid metabolic process	4/117	120/18862	0.006650563	0.039241836	0.028284463	1545/5743/2180/3248	4
BP	GO:0034763	negative regulation of transmembrane transport	4/117	120/18862	0.006650563	0.039241836	0.028284463	7057/10105/3553/7124	4
BP	GO:0006690	icosanoid metabolic process	4/117	123/18862	0.007247014	0.041376636	0.029823169	1545/3553/5743/3248	4
BP	GO:1990266	neutrophil migration	4/117	123/18862	0.007247014	0.041376636	0.029823169	6280/2920/3958/6279	4
BP	GO:0002698	negative regulation of	4/117	125/18862	0.007663661	0.042941836	0.030951323	10875/11326/7124/3958	4

		immune effector process								
BP	GO:1903052	positive regulation of proteolysis involved in cellular protein catabolic process	4/117	127/18862	0.008095791	0.044431443	0.032024992	1191/3303/255488/120892		4
BP	GO:0019079	viral genome replication	4/117	129/18862	0.008543631	0.045881837	0.033070397	3429/10410/7124/8638		4
BP	GO:0032434	regulation of proteasomal ubiquitin-dependent protein catabolic process	4/117	132/18862	0.009245335	0.048332676	0.034836896	1191/3303/255488/120892		4
BP	GO:0002687	positive regulation of leukocyte migration	4/117	133/18862	0.00948733	0.049385103	0.035595457	7057/1524/7124/3958		4
BP	GO:0001660	fever generation	3/117	10/18862	2.70E-05	0.000873044	0.000629267	3553/5743/7124		3

BP	GO:0031652	positive regulation of heat generation	3/117	10/18862	2.70E-05	0.000873044	0.000629267	3553/5743/7124	3
BP	GO:0031650	regulation of heat generation	3/117	13/18862	6.36E-05	0.00161142	0.001161468	3553/5743/7124	3
BP	GO:2001198	regulation of dendritic cell differentiation	3/117	13/18862	6.36E-05	0.00161142	0.001161468	28959/10288/55365	3
BP	GO:0002468	dendritic cell antigen processing and presentation	3/117	15/18862	0.000100233	0.002140416	0.001542755	7057/10875/3123	3
BP	GO:0031649	heat generation	3/117	16/18862	0.000122807	0.002504147	0.001804922	3553/5743/7124	3
BP	GO:0051238	sequestering of metal ion	3/117	16/18862	0.000122807	0.002504147	0.001804922	2495/6280/6279	3
BP	GO:0150078	positive regulation of neuroinflammatory response	3/117	17/18862	0.000148449	0.00287566	0.002072699	3553/120892/7124	3
BP	GO:1901550	regulation of endothelial cell development	3/117	17/18862	0.000148449	0.00287566	0.002072699	7414/3553/7124	3
BP	GO:1903140	regulation of establishment	3/117	17/18862	0.000148449	0.00287566	0.002072699	7414/3553/7124	3

		of endothelial barrier							
BP	GO:0002577	regulation of antigen processing and presentation	3/117	20/18862	0.000245513	0.004059953	0.002926305	7057/10875/10288	3
BP	GO:0034114	regulation of heterotypic cell-cell adhesion	3/117	26/18862	0.00054496	0.007778583	0.005606594	3553/7124/3557	3
BP	GO:0002675	positive regulation of acute inflammatory response	3/117	27/18862	0.000610317	0.008304558	0.005985702	3553/5743/7124	3
BP	GO:0010955	negative regulation of protein processing	3/117	27/18862	0.000610317	0.008304558	0.005985702	7057/7850/120892	3
BP	GO:1902883	negative regulation of response to oxidative stress	3/117	27/18862	0.000610317	0.008304558	0.005985702	8013/120892/79772	3
BP	GO:1903318	negative regulation of	3/117	27/18862	0.000610317	0.008304558	0.005985702	7057/7850/120892	3

		protein maturation							
BP	GO:0010575	positive regulation of vascular endothelial growth factor production	3/117	28/18862	0.000680476	0.008958499	0.006457046	1545/3553/5743	3
BP	GO:0032743	positive regulation of interleukin-2 production	3/117	30/18862	0.000835747	0.010267316	0.007400406	9308/3553/64581	3
BP	GO:0043372	positive regulation of CD4-positive, alpha-beta T cell differentiation	3/117	32/18862	0.001011842	0.011726989	0.008452499	64332/9308/3123	3
BP	GO:1901099	negative regulation of signal transduction in absence of ligand	3/117	32/18862	0.001011842	0.011726989	0.008452499	3303/3553/7124	3
BP	GO:1901889	negative regulation of	3/117	32/18862	0.001011842	0.011726989	0.008452499	7057/3553/7124	3

		cell junction assembly							
BP	GO:2001240	negative regulation of extrinsic apoptotic signaling pathway in absence of ligand	3/117	32/18862	0.001011842	0.011726989	0.008452499	3303/3553/7124	3
BP	GO:1903960	negative regulation of anion transmembrane transport	3/117	35/18862	0.001317249	0.014640899	0.010552767	7057/3553/7124	3
BP	GO:1901031	regulation of response to reactive oxygen species	3/117	37/18862	0.001549768	0.016879404	0.012166221	8013/143686/120892	3
BP	GO:0006882	cellular zinc ion homeostasis	3/117	38/18862	0.001675047	0.017527718	0.012633509	6280/4493/6279	3
BP	GO:0032733	positive regulation of interleukin-10 production	3/117	38/18862	0.001675047	0.017527718	0.012633509	9308/353514/64581	3

BP	GO:0045923	positive regulation of fatty acid metabolic process	3/117	38/18862	0.001675047	0.017527718	0.012633509	8013/3553/5743	3
BP	GO:0097242	amyloid-beta clearance	3/117	38/18862	0.001675047	0.017527718	0.012633509	1191/3949/7124	3
BP	GO:2000516	positive regulation of CD4-positive, alpha-beta T cell activation	3/117	39/18862	0.001806484	0.018558165	0.013376227	64332/9308/3123	3
BP	GO:0055069	zinc ion homeostasis	3/117	40/18862	0.001944187	0.019456217	0.014023519	6280/4493/6279	3
BP	GO:1902895	positive regulation of pri-miRNA transcription by RNA polymerase II	3/117	40/18862	0.001944187	0.019456217	0.014023519	1958/7124/6688	3
BP	GO:0006509	membrane protein ectodomain proteolysis	3/117	42/18862	0.002238803	0.021378992	0.015409404	3553/7124/8728	3
BP	GO:2001239	regulation of extrinsic	3/117	42/18862	0.002238803	0.021378992	0.015409404	3303/3553/7124	3

		apoptotic signaling pathway in absence of ligand							
BP	GO:0090199	regulation of release of cytochrome c from mitochondria	3/117	44/18862	0.002559695	0.023139645	0.016678435	5329/1191/10105	3
BP	GO:1903573	negative regulation of response to endoplasmic reticulum stress	3/117	44/18862	0.002559695	0.023139645	0.016678435	1191/3303/120892	3
BP	GO:0010823	negative regulation of mitochondrion organization	3/117	46/18862	0.002907623	0.024875314	0.017929459	1191/3303/10105	3
BP	GO:0045646	regulation of erythrocyte differentiation	3/117	47/18862	0.003091952	0.025880787	0.018654177	9935/3303/6688	3
BP	GO:1904707	positive regulation of vascular associated	3/117	47/18862	0.003091952	0.025880787	0.018654177	8013/7124/3248	3

		smooth muscle<br>cell<br>proliferation							
BP	GO:000<br>2673	regulation of<br>acute<br>inflammatory<br>response	3/117	48/18<br>862	0.00328<br>3307	0.02645<br>1466	0.01906<br>5507	3553/5743/7124	3
BP	GO:000<br>6692	prostanoid<br>metabolic<br>process	3/117	48/18<br>862	0.00328<br>3307	0.02645<br>1466	0.01906<br>5507	3553/5743/3248	3
BP	GO:000<br>6693	prostaglandin<br>metabolic<br>process	3/117	48/18<br>862	0.00328<br>3307	0.02645<br>1466	0.01906<br>5507	3553/5743/3248	3
BP	GO:001<br>4009	glial cell<br>proliferation	3/117	48/18<br>862	0.00328<br>3307	0.02645<br>1466	0.01906<br>5507	1191/3553/7124	3
BP	GO:006<br>1028	establishment<br>of endothelial<br>barrier	3/117	48/18<br>862	0.00328<br>3307	0.02645<br>1466	0.01906<br>5507	7414/3553/7124	3
BP	GO:200<br>0191	regulation of<br>fatty acid<br>transport	3/117	48/18<br>862	0.00328<br>3307	0.02645<br>1466	0.01906<br>5507	7057/3553/2180	3
BP	GO:003<br>2692	negative<br>regulation of<br>interleukin-1<br>production	3/117	49/18<br>862	0.00348<br>1772	0.02718<br>9012	0.01959<br>7111	7850/1524/7128	3

BP	GO:0046638	positive regulation of alpha-beta T cell differentiation	3/117	49/18862	0.003481772	0.027189012	0.019597111	64332/9308/3123	3
BP	GO:0002931	response to ischemia	3/117	50/18862	0.003687428	0.028249452	0.020361447	1524/10105/1958	3
BP	GO:0043370	regulation of CD4-positive, alpha-beta T cell differentiation	3/117	51/18862	0.003900356	0.028743925	0.02071785	64332/9308/3123	3
BP	GO:1902893	regulation of pri-miRNA transcription by RNA polymerase II	3/117	51/18862	0.003900356	0.028743925	0.02071785	1958/7124/6688	3
BP	GO:0061614	pri-miRNA transcription by RNA polymerase II	3/117	52/18862	0.004120631	0.029880081	0.021536761	1958/7124/6688	3
BP	GO:0072666	establishment of protein localization to vacuole	3/117	52/18862	0.004120631	0.029880081	0.021536761	1191/8031/7128	3

BP	GO:0002720	positive regulation of cytokine production involved in immune response	3/117	53/18862	0.004348328	0.030472005	0.021963404	8013/3553/64581	3
BP	GO:0009620	response to fungus	3/117	53/18862	0.004348328	0.030472005	0.021963404	6280/64581/6279	3
BP	GO:1905517	macrophage migration	3/117	53/18862	0.004348328	0.030472005	0.021963404	7057/1524/3958	3
BP	GO:0006968	cellular defense response	3/117	54/18862	0.004583521	0.031683731	0.022836784	1524/4332/10288	3
BP	GO:0045071	negative regulation of viral genome replication	3/117	54/18862	0.004583521	0.031683731	0.022836784	10410/7124/8638	3
BP	GO:0001836	release of cytochrome c from mitochondria	3/117	55/18862	0.004826279	0.032449833	0.023388969	5329/1191/10105	3
BP	GO:0010574	regulation of vascular endothelial growth factor production	3/117	58/18862	0.005600618	0.035571137	0.025638721	1545/3553/5743	3

BP	GO:0031663	lipopolysaccharide-mediated signaling pathway	3/117	58/18862	0.005600618	0.035571137	0.025638721	3553/7124/7128	3
BP	GO:0090303	positive regulation of wound healing	3/117	59/18862	0.005874299	0.036862318	0.02656937	7057/7056/64581	3
BP	GO:1902041	regulation of extrinsic apoptotic signaling pathway via death domain receptors	3/117	59/18862	0.005874299	0.036862318	0.02656937	7057/7128/3958	3
BP	GO:0032615	interleukin-12 production	3/117	60/18862	0.006155865	0.037348333	0.026919676	7057/353514/64581	3
BP	GO:0032655	regulation of interleukin-12 production	3/117	60/18862	0.006155865	0.037348333	0.026919676	7057/353514/64581	3
BP	GO:0032890	regulation of organic acid transport	3/117	60/18862	0.006155865	0.037348333	0.026919676	7057/3553/2180	3
BP	GO:0050994	regulation of lipid catabolic process	3/117	60/18862	0.006155865	0.037348333	0.026919676	3553/3949/7124	3

BP	GO:0033619	membrane protein proteolysis	3/117	61/18862	0.006445374	0.038499861	0.027749667	3553/7124/8728	3
BP	GO:0070613	regulation of protein processing	3/117	61/18862	0.006445374	0.038499861	0.027749667	7057/7850/120892	3
BP	GO:1903961	positive regulation of anion transmembrane transport	3/117	61/18862	0.006445374	0.038499861	0.027749667	8013/2180/4205	3
BP	GO:0002437	inflammatory response to antigenic stimulus	3/117	62/18862	0.006742882	0.039241836	0.028284463	7124/3557/3123	3
BP	GO:0032835	glomerulus development	3/117	62/18862	0.006742882	0.039241836	0.028284463	1958/4208/9788	3
BP	GO:1902686	mitochondrial outer membrane permeabilization involved in programmed cell death	3/117	62/18862	0.006742882	0.039241836	0.028284463	3303/10105/7533	3
BP	GO:0048662	negative regulation of	3/117	63/18862	0.007048443	0.040725408	0.029353782	4208/7128/1026	3

		smooth muscle cell proliferation							
BP	GO:1903317	regulation of protein maturation	3/117	63/18862	0.007048443	0.040725408	0.029353782	7057/7850/120892	3
BP	GO:0035794	positive regulation of mitochondrial membrane permeability	3/117	64/18862	0.007362108	0.041733556	0.030080428	3303/10105/7533	3
BP	GO:0071479	cellular response to ionizing radiation	3/117	65/18862	0.007683928	0.042966626	0.030969192	1958/388/1026	3
BP	GO:0001885	endothelial cell development	3/117	66/18862	0.008013951	0.044354768	0.031969726	7414/3553/7124	3
BP	GO:0042130	negative regulation of T cell proliferation	3/117	66/18862	0.008013951	0.044354768	0.031969726	11326/10288/3123	3
BP	GO:0046635	positive regulation of alpha-beta T cell activation	3/117	66/18862	0.008013951	0.044354768	0.031969726	64332/9308/3123	3

BP	GO:0050805	negative regulation of synaptic transmission	3/117	66/18862	0.008013951	0.044354768	0.031969726	10288/3553/120892	3
BP	GO:2000272	negative regulation of signaling receptor activity	3/117	66/18862	0.008013951	0.044354768	0.031969726	100462981/100463486/7124	3
BP	GO:0032922	circadian regulation of gene expression	3/117	67/18862	0.008352223	0.044942916	0.032393648	1958/3397/196	3
BP	GO:0042987	amyloid precursor protein catabolic process	3/117	67/18862	0.008352223	0.044942916	0.032393648	1191/7124/8728	3
BP	GO:0046637	regulation of alpha-beta T cell differentiation	3/117	67/18862	0.008352223	0.044942916	0.032393648	64332/9308/3123	3
BP	GO:0050766	positive regulation of phagocytosis	3/117	67/18862	0.008352223	0.044942916	0.032393648	3553/7124/64581	3

BP	GO:1901224	positive regulation of NIK/NF-kappaB signaling	3/117	67/18862	0.008352223	0.044942916	0.032393648	3553/3611/7124	3
BP	GO:2000514	regulation of CD4-positive, alpha-beta T cell activation	3/117	67/18862	0.008352223	0.044942916	0.032393648	64332/9308/3123	3
BP	GO:0051966	regulation of synaptic transmission, glutamatergic	3/117	69/18862	0.00905369	0.047492473	0.0342313	4208/120892/7124	3
BP	GO:1905710	positive regulation of membrane permeability	3/117	69/18862	0.00905369	0.047492473	0.0342313	3303/10105/7533	3
BP	GO:0006879	cellular iron ion homeostasis	3/117	70/18862	0.009416969	0.049113115	0.035399416	2495/8031/30061	3
BP	GO:0002291	T cell activation via T cell receptor contact with antigen bound to MHC molecule on	2/117	10/18862	0.001661786	0.017527718	0.012633509	10875/3958	2

		antigen presenting cell							
BP	GO:0015911	long-chain fatty acid import across plasma membrane	2/117	10/18862	0.001661786	0.017527718	0.012633509	7057/2180	2
BP	GO:0051918	negative regulation of fibrinolysis	2/117	10/18862	0.001661786	0.017527718	0.012633509	7057/7056	2
BP	GO:2000659	regulation of interleukin-1-mediated signaling pathway	2/117	10/18862	0.001661786	0.017527718	0.012633509	7850/3557	2
BP	GO:0002604	regulation of dendritic cell antigen processing and presentation	2/117	11/18862	0.002022847	0.020021753	0.014431143	7057/10875	2
BP	GO:0043380	regulation of memory T cell differentiation	2/117	12/18862	0.002417591	0.022364297	0.016119585	10875/3123	2
BP	GO:0055012	ventricular cardiac muscle cell differentiation	2/117	12/18862	0.002417591	0.022364297	0.016119585	4208/4205	2

BP	GO:0061469	regulation of type B pancreatic cell proliferation	2/117	12/18862	0.002417591	0.022364297	0.016119585	3164/8013	2
BP	GO:0071639	positive regulation of monocyte chemotactic protein-1 production	2/117	12/18862	0.002417591	0.022364297	0.016119585	3553/64581	2
BP	GO:1905907	negative regulation of amyloid fibril formation	2/117	12/18862	0.002417591	0.022364297	0.016119585	1191/3949	2
BP	GO:0031392	regulation of prostaglandin biosynthetic process	2/117	13/18862	0.002845593	0.0244992	0.017658366	3553/5743	2
BP	GO:0043379	memory T cell differentiation	2/117	13/18862	0.002845593	0.0244992	0.017658366	10875/3123	2
BP	GO:0061043	regulation of vascular wound healing	2/117	13/18862	0.002845593	0.0244992	0.017658366	7124/7128	2
BP	GO:0070424	regulation of nucleotide-binding	2/117	13/18862	0.002845593	0.0244992	0.017658366	3303/7128	2

		oligomerization domain containing signaling pathway							
BP	GO:190 2947	regulation of tau-protein kinase activity	2/117	13/18 862	0.00284 5593	0.02449 92	0.01765 8366	1191/1958	2
BP	GO:000 2467	germinal center formation	2/117	14/18 862	0.00330 6433	0.02645 1466	0.01906 5507	4208/7128	2
BP	GO:005 1917	regulation of fibrinolysis	2/117	14/18 862	0.00330 6433	0.02645 1466	0.01906 5507	7057/7056	2
BP	GO:009 0715	immunological memory formation process	2/117	14/18 862	0.00330 6433	0.02645 1466	0.01906 5507	10875/3123	2
BP	GO:002 3035	CD40 signaling pathway	2/117	15/18 862	0.00379 9695	0.02854 5075	0.02057 4525	3678/7128	2
BP	GO:005 1044	positive regulation of membrane protein ectodomain proteolysis	2/117	15/18 862	0.00379 9695	0.02854 5075	0.02057 4525	3553/7124	2

BP	GO:0072160	nephron tubule epithelial cell differentiation	2/117	15/18862	0.003799695	0.028545075	0.020574525	4208/9788	2
BP	GO:1905906	regulation of amyloid fibril formation	2/117	15/18862	0.003799695	0.028545075	0.020574525	1191/3949	2
BP	GO:2001279	regulation of unsaturated fatty acid biosynthetic process	2/117	15/18862	0.003799695	0.028545075	0.020574525	3553/5743	2
BP	GO:0017014	protein nitrosylation	2/117	16/18862	0.004324965	0.030472005	0.021963404	6280/6279	2
BP	GO:0018119	peptidyl-cysteine S-nitrosylation	2/117	16/18862	0.004324965	0.030472005	0.021963404	6280/6279	2
BP	GO:0019372	lipoxygenase pathway	2/117	16/18862	0.004324965	0.030472005	0.021963404	5743/3248	2
BP	GO:0030949	positive regulation of vascular endothelial growth factor receptor signaling pathway	2/117	16/18862	0.004324965	0.030472005	0.021963404	3553/3678	2

BP	GO:0034116	positive regulation of heterotypic cell-cell adhesion	2/117	16/18862	0.004324965	0.030472005	0.021963404	3553/7124		2
BP	GO:0045591	positive regulation of regulatory T cell differentiation	2/117	16/18862	0.004324965	0.030472005	0.021963404	10288/3123		2
BP	GO:0048311	mitochondrion distribution	2/117	16/18862	0.004324965	0.030472005	0.021963404	120892/4205		2
BP	GO:0070431	nucleotide-binding oligomerization domain containing 2 signaling pathway	2/117	16/18862	0.004324965	0.030472005	0.021963404	3303/7128		2
BP	GO:0090083	regulation of inclusion body assembly	2/117	16/18862	0.004324965	0.030472005	0.021963404	1191/3303		2
BP	GO:0001696	gastric acid secretion	2/117	17/18862	0.004881833	0.032449833	0.023388969	3274/6446		2
BP	GO:0030730	sequestering of triglyceride	2/117	17/18862	0.004881833	0.032449833	0.023388969	3553/7124		2

BP	GO:0044539	long-chain fatty acid import into cell	2/117	17/18862	0.004881833	0.032449833	0.023388969	7057/2180	2
BP	GO:0071850	mitotic cell cycle arrest	2/117	17/18862	0.004881833	0.032449833	0.023388969	28984/1026	2
BP	GO:0090136	epithelial cell-cell adhesion	2/117	17/18862	0.004881833	0.032449833	0.023388969	7414/1545	2
BP	GO:0090201	negative regulation of release of cytochrome c from mitochondria	2/117	17/18862	0.004881833	0.032449833	0.023388969	1191/10105	2
BP	GO:0090713	immunological memory process	2/117	17/18862	0.004881833	0.032449833	0.023388969	10875/3123	2
BP	GO:0002523	leukocyte migration involved in inflammatory response	2/117	18/18862	0.005469896	0.035250471	0.025407594	6280/6279	2
BP	GO:0032695	negative regulation of interleukin-12 production	2/117	18/18862	0.005469896	0.035250471	0.025407594	7057/353514	2

BP	GO:0060252	positive regulation of glial cell proliferation	2/117	18/18862	0.005469896	0.035250471	0.025407594	3553/7124	2
BP	GO:0071498	cellular response to fluid shear stress	2/117	18/18862	0.005469896	0.035250471	0.025407594	4208/9788	2
BP	GO:0071605	monocyte chemotactic protein-1 production	2/117	18/18862	0.005469896	0.035250471	0.025407594	3553/64581	2
BP	GO:0071637	regulation of monocyte chemotactic protein-1 production	2/117	18/18862	0.005469896	0.035250471	0.025407594	3553/64581	2
BP	GO:1902236	negative regulation of endoplasmic reticulum stress-induced intrinsic apoptotic signaling pathway	2/117	18/18862	0.005469896	0.035250471	0.025407594	3303/120892	2

BP	GO:0010829	negative regulation of glucose transmembrane transport	2/117	19/18862	0.00608875	0.037274697	0.026866601	3553/7124	2
BP	GO:0034755	iron ion transmembrane transport	2/117	19/18862	0.00608875	0.037274697	0.026866601	30061/51312	2
BP	GO:0043031	negative regulation of macrophage activation	2/117	19/18862	0.00608875	0.037274697	0.026866601	11326/3949	2
BP	GO:0098543	detection of other organism	2/117	19/18862	0.00608875	0.037274697	0.026866601	64581/3123	2
BP	GO:1900221	regulation of amyloid-beta clearance	2/117	19/18862	0.00608875	0.037274697	0.026866601	1191/7124	2
BP	GO:0007252	I-kappaB phosphorylation	2/117	20/18862	0.006737999	0.039241836	0.028284463	1524/7124	2
BP	GO:0044342	type B pancreatic cell proliferation	2/117	20/18862	0.006737999	0.039241836	0.028284463	3164/8013	2
BP	GO:1902004	positive regulation of	2/117	20/18862	0.006737999	0.039241836	0.028284463	1191/7124	2

		amyloid-beta formation							
BP	GO:1903206	negative regulation of hydrogen peroxide-induced cell death	2/117	20/18862	0.006737999	0.039241836	0.028284463	8013/120892	2
BP	GO:1903978	regulation of microglial cell activation	2/117	20/18862	0.006737999	0.039241836	0.028284463	3949/120892	2
BP	GO:0061042	vascular wound healing	2/117	21/18862	0.007417247	0.041733556	0.030080428	7124/7128	2
BP	GO:0071636	positive regulation of transforming growth factor beta production	2/117	21/18862	0.007417247	0.041733556	0.030080428	7057/5743	2
BP	GO:1900118	negative regulation of execution phase of apoptosis	2/117	21/18862	0.007417247	0.041733556	0.030080428	100462981/100463486	2
BP	GO:1903429	regulation of cell maturation	2/117	21/18862	0.007417247	0.041733556	0.030080428	120892/64581	2

BP	GO:2000047	regulation of cell-cell adhesion mediated by cadherin	2/117	21/18862	0.007417247	0.041733556	0.030080428	28984/8728	2
BP	GO:0002922	positive regulation of humoral immune response	2/117	22/18862	0.008126105	0.044431443	0.032024992	3553/7124	2
BP	GO:0032069	regulation of nuclease activity	2/117	22/18862	0.008126105	0.044431443	0.032024992	3303/8638	2
BP	GO:0035970	peptidyl-threonine dephosphorylation	2/117	22/18862	0.008126105	0.044431443	0.032024992	1848/1844	2
BP	GO:0051131	chaperone-mediated protein complex assembly	2/117	22/18862	0.008126105	0.044431443	0.032024992	1191/3303	2
BP	GO:1901032	negative regulation of response to	2/117	22/18862	0.008126105	0.044431443	0.032024992	8013/120892	2

		reactive oxygen species							
BP	GO:0006622	protein targeting to lysosome	2/117	23/18862	0.008864184	0.046751695	0.033697368	1191/8031	2
BP	GO:0030194	positive regulation of blood coagulation	2/117	23/18862	0.008864184	0.046751695	0.033697368	7057/7056	2
BP	GO:0045723	positive regulation of fatty acid biosynthetic process	2/117	23/18862	0.008864184	0.046751695	0.033697368	3553/5743	2
BP	GO:0051043	regulation of membrane protein ectodomain proteolysis	2/117	23/18862	0.008864184	0.046751695	0.033697368	3553/7124	2
BP	GO:0070841	inclusion body assembly	2/117	23/18862	0.008864184	0.046751695	0.033697368	1191/3303	2
BP	GO:1900048	positive regulation of hemostasis	2/117	23/18862	0.008864184	0.046751695	0.033697368	7057/7056	2
BP	GO:2000353	positive regulation of	2/117	23/18862	0.008864184	0.046751695	0.033697368	7057/28984	2

		endothelial cell apoptotic process							
BP	GO:0030318	melanocyte differentiation	2/117	24/18862	0.009631103	0.049562715	0.035723476	4208/9839	2
BP	GO:0050820	positive regulation of coagulation	2/117	24/18862	0.009631103	0.049562715	0.035723476	7057/7056	2
BP	GO:0098581	detection of external biotic stimulus	2/117	24/18862	0.009631103	0.049562715	0.035723476	64581/3123	2
BP	GO:1900017	positive regulation of cytokine production involved in inflammatory response	2/117	24/18862	0.009631103	0.049562715	0.035723476	7124/64581	2
BP	GO:1905523	positive regulation of macrophage migration	2/117	24/18862	0.009631103	0.049562715	0.035723476	7057/1524	2
CC	GO:0030139	endocytic vesicle	12/118	307/19520	3.57E-07	5.95E-06	4.48E-06	3119/122618/9332/3127/3113/3117/3949/9788/3115/3118/23166/3123	12

CC	GO:0009897	external side of plasma membrane	12/118	402/19520	5.91E-06	7.12E-05	5.35E-05	7057/1524/9332/9308/3678/2214/3949/2352/974/7124/7056/3123	12
CC	GO:0005774	vacuolar membrane	11/118	431/19520	6.16E-05	0.000580851	0.00043673	3119/10410/3127/3113/3117/3949/5660/3115/7056/3118/3123	11
CC	GO:0030666	endocytic vesicle membrane	10/118	163/19520	5.61E-08	1.11E-06	8.33E-07	3119/9332/3127/3113/3117/3949/3115/3118/23166/3123	10
CC	GO:0030658	transport vesicle membrane	10/118	206/19520	4.99E-07	7.73E-06	5.81E-06	3119/374/3127/3113/3117/120892/3115/79772/3118/3123	10
CC	GO:0005802	trans-Golgi network	10/118	251/19520	2.98E-06	3.81E-05	2.86E-05	3119/58475/122618/3127/3113/3117/120892/3115/3118/3123	10
CC	GO:0030135	coated vesicle	10/118	295/19520	1.24E-05	0.000141063	0.000106062	3119/374/3127/3113/3117/3949/5265/3115/3118/3123	10
CC	GO:0005765	lysosomal membrane	10/118	378/19520	0.000100344	0.000837482	0.000629686	3119/10410/3127/3113/3117/3949/5660/3115/3118/3123	10
CC	GO:0098852	lytic vacuole membrane	10/118	378/19520	0.000100344	0.000837482	0.000629686	3119/10410/3127/3113/3117/3949/5660/3115/3118/3123	10
CC	GO:0030133	transport vesicle	10/118	402/19520	0.000165747	0.001160231	0.000872354	3119/374/3127/3113/3117/120892/3115/79772/3118/3123	10
CC	GO:0030134	COPII-coated ER to Golgi transport vesicle	9/118	94/19520	5.48E-09	1.32E-07	9.93E-08	3119/374/3127/3113/3117/5265/3115/3118/3123	9

CC	GO:0030665	clathrin-coated vesicle membrane	9/118	117/19520	3.79E-08	8.22E-07	6.18E-07	3119/374/3127/3113/3117/3949/3115/3118/3123	9
CC	GO:0030662	coated vesicle membrane	9/118	182/19520	1.63E-06	2.36E-05	1.78E-05	3119/374/3127/3113/3117/3949/3115/3118/3123	9
CC	GO:0030136	clathrin-coated vesicle	9/118	192/19520	2.54E-06	3.44E-05	2.59E-05	3119/374/3127/3113/3117/3949/3115/3118/3123	9
CC	GO:0034774	secretory granule lumen	9/118	322/19520	0.000149773	0.001120717	0.000842645	7057/1191/4332/7414/6036/6280/2352/5265/6279	9
CC	GO:0060205	cytoplasmic vesicle lumen	9/118	326/19520	0.000164276	0.001160231	0.000872354	7057/1191/4332/7414/6036/6280/2352/5265/6279	9
CC	GO:0031983	vesicle lumen	9/118	328/19520	0.000171957	0.00116608	0.000876752	7057/1191/4332/7414/6036/6280/2352/5265/6279	9
CC	GO:0062023	collagen-containing extracellular matrix	9/118	423/19520	0.001080501	0.007105114	0.005342191	7057/10875/1191/6280/5265/5660/8728/3958/6279	9
CC	GO:0030669	clathrin-coated endocytic vesicle membrane	8/118	39/19520	7.38E-11	7.83E-09	5.89E-09	3119/3127/3113/3117/3949/3115/3118/3123	8
CC	GO:0045334	clathrin-coated endocytic vesicle	8/118	57/19520	1.81E-09	5.61E-08	4.22E-08	3119/3127/3113/3117/3949/3115/3118/3123	8
CC	GO:0012507	ER to Golgi transport	8/118	62/19520	3.61E-09	9.80E-08	7.37E-08	3119/374/3127/3113/3117/3115/3118/3123	8

		vesicle membrane							
CC	GO:0032588	trans-Golgi network membrane	8/118	95/19520	1.10E-07	1.99E-06	1.50E-06	3119/122618/3127/3113/3117/3115/3118/3123	8
CC	GO:0101002	ficolin-1-rich granule	8/118	185/19520	1.68E-05	0.000182686	0.000137358	10875/4332/3303/2495/7414/10288/5265/3958	8
CC	GO:0005635	nuclear envelope	8/118	462/19520	0.00703626	0.039150475	0.029436447	3164/3429/3267/28959/4000/5743/1844/23345	8
CC	GO:0042613	MHC class II protein complex	7/118	16/19520	2.69E-12	5.85E-10	4.40E-10	3119/3127/3113/3117/3115/3118/3123	7
CC	GO:0042611	MHC protein complex	7/118	25/19520	1.08E-10	7.83E-09	5.89E-09	3119/3127/3113/3117/3115/3118/3123	7
CC	GO:0071556	integral component of lumenal side of endoplasmic reticulum membrane	7/118	29/19520	3.45E-10	1.50E-08	1.12E-08	3119/3127/3113/3117/3115/3118/3123	7
CC	GO:0098553	lumenal side of endoplasmic reticulum membrane	7/118	29/19520	3.45E-10	1.50E-08	1.12E-08	3119/3127/3113/3117/3115/3118/3123	7
CC	GO:0098576	lumenal side of membrane	7/118	36/19520	1.78E-09	5.61E-08	4.22E-08	3119/3127/3113/3117/3115/3118/3123	7

CC	GO:0030176	integral component of endoplasmic reticulum membrane	7/118	157/19520	4.76E-05	0.000469239	0.000352811	3119/3127/3113/3117/3115/3118/3123	7
CC	GO:0031227	intrinsic component of endoplasmic reticulum membrane	7/118	165/19520	6.52E-05	0.000589335	0.000443109	3119/3127/3113/3117/3115/3118/3123	7
CC	GO:0005770	late endosome	7/118	275/19520	0.001423904	0.009087859	0.006832976	10410/3127/3949/120892/388/5660/3123	7
CC	GO:0031965	nuclear membrane	7/118	295/19520	0.002120367	0.013146273	0.009884416	3164/3429/28959/4000/5743/1844/23345	7
CC	GO:0031301	integral component of organelle membrane	7/118	371/19520	0.007372994	0.039998491	0.030074053	3119/3127/3113/3117/3115/3118/3123	7
CC	GO:1904813	ficolin-1-rich granule lumen	6/118	124/19520	0.000107331	0.000862627	0.000648592	10875/4332/3303/2495/7414/5265	6
CC	GO:0031968	organelle outer membrane	6/118	220/19520	0.002212725	0.013337813	0.010028431	3429/5743/597/2180/120892/23345	6
CC	GO:0019867	outer membrane	6/118	222/19520	0.002315062	0.013577527	0.010208667	3429/5743/597/2180/120892/23345	6
CC	GO:0070820	tertiary granule	5/118	164/19520	0.003213587	0.018351275	0.013797951	6813/2495/10288/2352/3958	5

CC	GO:0044754	autolysosome	3/118	10/19520	2.51E-05	0.000258893	0.000194657	2495/8031/120892	3
CC	GO:0005767	secondary lysosome	3/118	16/19520	0.000113861	0.000882421	0.000663475	2495/8031/120892	3
CC	GO:0031093	platelet alpha granule lumen	3/118	67/19520	0.007788418	0.041221626	0.030993704	7057/1191/5265	3
MF	GO:0033218	amide binding	13/116	391/18337	1.17E-06	0.000132716	0.000108975	3119/1191/3127/10105/10288/3113/3117/30061/3949/2352/5660/3115/3123	13
MF	GO:0042277	peptide binding	11/116	315/18337	5.11E-06	0.000348629	0.000286264	3119/1191/3127/10105/10288/3113/3117/30061/3949/3115/3123	11
MF	GO:0001228	DNA-binding transcription activator activity, RNA polymerase II-specific	10/116	443/18337	0.000510114	0.020724155	0.017016902	3164/9935/8013/1052/571/1958/4208/4205/2120/6688	10
MF	GO:0001216	DNA-binding transcription activator activity	10/116	447/18337	0.000546972	0.020724155	0.017016902	3164/9935/8013/1052/571/1958/4208/4205/2120/6688	10
MF	GO:0140375	immune receptor activity	8/116	136/18337	2.41E-06	0.00020576	0.000168953	7850/3119/1524/10288/3113/3117/3118/3123	8
MF	GO:0140297	DNA-binding transcription factor binding	8/116	376/18337	0.002695892	0.043837798	0.035995846	3164/3429/8013/4208/7533/8638/4205/6688	8

MF	GO:0042605	peptide antigen binding	6/116	32/18337	4.46E-08	7.60E-06	6.24E-06	3119/3127/3113/3117/3115/3123	6
MF	GO:0003823	antigen binding	6/116	165/18337	0.000636072	0.021690048	0.01781001	3119/3127/3113/3117/3115/3123	6
MF	GO:0032395	MHC class II receptor activity	5/116	10/18337	2.28E-09	7.78E-07	6.39E-07	3119/3113/3117/3118/3123	5
MF	GO:0002020	protease binding	5/116	131/18337	0.001467616	0.034644463	0.028447066	3949/5265/7124/5660/7128	5
MF	GO:0035259	glucocorticoid receptor binding	3/116	12/18337	5.21E-05	0.002958572	0.002429326	3164/8013/7533	3
MF	GO:0030169	low-density lipoprotein particle binding	3/116	17/18337	0.000157229	0.007659322	0.00628918	7057/3949/23166	3
MF	GO:0071813	lipoprotein particle binding	3/116	30/18337	0.000884166	0.025125058	0.020630546	7057/3949/23166	3
MF	GO:0071814	protein-lipid complex binding	3/116	30/18337	0.000884166	0.025125058	0.020630546	7057/3949/23166	3
MF	GO:0048019	receptor antagonist activity	3/116	32/18337	0.001070275	0.028074127	0.023052069	100462981/100463486/3557	3
MF	GO:0030547	receptor inhibitor activity	3/116	43/18337	0.002531833	0.043837798	0.035995846	100462981/100463486/3557	3

MF	GO:0005381	iron ion transmembrane transporter activity	2/116	10/18337	0.001727143	0.034644463	0.028447066	30061/51312	2
MF	GO:0008330	protein tyrosine/threonine phosphatase activity	2/116	10/18337	0.001727143	0.034644463	0.028447066	1848/1844	2
MF	GO:0050786	RAGE receptor binding	2/116	10/18337	0.001727143	0.034644463	0.028447066	6280/6279	2
MF	GO:0035325	Toll-like receptor binding	2/116	12/18337	0.002512272	0.043837798	0.035995846	6280/6279	2
MF	GO:0005521	lamin binding	2/116	13/18337	0.002956802	0.043837798	0.035995846	3429/23345	2
MF	GO:0017017	MAP kinase tyrosine/serine/threonine phosphatase activity	2/116	13/18337	0.002956802	0.043837798	0.035995846	1848/1844	2
MF	GO:0036041	long-chain fatty acid binding	2/116	13/18337	0.002956802	0.043837798	0.035995846	6280/6279	2
MF	GO:0005041	low-density lipoprotein particle	2/116	14/18337	0.003435379	0.048811008	0.04007942	3949/23166	2

		receptor activity							
KEGG	hsa05166	Human T-cell leukemia virus 1 infection	12/80	222/8096	1.56E-06	1.75E-05	1.33E-05	7850/3119/3127/3113/1958/3117/7124/3115/6688/3118/1026/3123	12
KEGG	hsa05140	Leishmaniasis	11/80	77/8096	1.71E-10	1.45E-08	1.11E-08	3119/3127/3113/3553/2214/3117/5743/7124/3115/3118/3123	11
KEGG	hsa04640	Hematopoietic cell lineage	11/80	99/8096	2.71E-09	8.09E-08	6.18E-08	7850/3119/3127/3113/3553/3678/3117/7124/3115/3118/3123	11
KEGG	hsa05145	Toxoplasmosis	11/80	112/8096	1.02E-08	2.59E-07	1.98E-07	3119/3303/3127/10105/3113/3117/3949/7124/3115/3118/3123	11
KEGG	hsa04145	Phagosome	11/80	152/8096	2.45E-07	3.98E-06	3.05E-06	7057/3119/3127/3113/3678/2214/3117/3115/64581/3118/3123	11
KEGG	hsa05152	Tuberculosis	11/80	180/8096	1.34E-06	1.59E-05	1.22E-05	3119/3127/3113/3553/2214/3117/7124/3115/64581/3118/3123	11
KEGG	hsa05202	Transcriptional misregulation in cancer	11/80	192/8096	2.52E-06	2.51E-05	1.92E-05	7850/64332/1848/8013/597/4208/2120/6688/7185/1026/3248	11
KEGG	hsa05323	Rheumatoid arthritis	10/80	93/8096	2.08E-08	4.14E-07	3.16E-07	3119/3127/3113/3553/3117/2920/7124/3115/3118/3123	10
KEGG	hsa05169	Epstein-Barr virus infection	10/80	202/8096	2.69E-05	0.000229457	0.000175415	3119/3127/3113/3117/7124/3115/3118/7128/1026/3123	10
KEGG	hsa05332	Graft-versus-host disease	9/80	42/8096	1.94E-10	1.45E-08	1.11E-08	3119/3127/3113/3553/3117/7124/3115/3118/3123	9
KEGG	hsa04940	Type I diabetes mellitus	9/80	43/8096	2.44E-10	1.45E-08	1.11E-08	3119/3127/3113/3553/3117/7124/3115/3118/3123	9

KEGG	hsa05321	Inflammatory bowel disease	9/80	65/8096	1.16E-08	2.59E-07	1.98E-07	3119/3127/3113/3553/3117/7124/3115/3118/3123	9
KEGG	hsa04612	Antigen processing and presentation	9/80	78/8096	5.96E-08	1.07E-06	8.16E-07	3119/3303/3127/3113/3117/7124/3115/3118/3123	9
KEGG	hsa04659	Th17 cell differentiation	9/80	108/8096	1.01E-06	1.29E-05	9.85E-06	3119/3127/3113/3553/3117/3115/196/3118/3123	9
KEGG	hsa05322	Systemic lupus erythematosus	9/80	136/8096	6.90E-06	6.18E-05	4.72E-05	3119/3127/3113/2214/3117/7124/3115/3118/3123	9
KEGG	hsa05164	Influenza A	9/80	172/8096	4.54E-05	0.000338885	0.000259071	3119/3127/3113/3553/3117/7124/3115/3118/3123	9
KEGG	hsa05310	Asthma	8/80	31/8096	4.18E-10	1.87E-08	1.43E-08	3119/3127/3113/3117/7124/3115/3118/3123	8
KEGG	hsa05330	Allograft rejection	8/80	38/8096	2.45E-09	8.09E-08	6.18E-08	3119/3127/3113/3117/7124/3115/3118/3123	8
KEGG	hsa05150	Staphylococcus aureus infection	8/80	96/8096	4.19E-06	3.94E-05	3.02E-05	3119/3127/3113/2214/3117/3115/3118/3123	8
KEGG	hsa04010	MAPK signaling pathway	8/80	294/8096	0.008255803	0.04104969	0.031381709	3164/1848/374/3303/3553/4208/1844/7124	8
KEGG	hsa04672	Intestinal immune network for IgA production	7/80	49/8096	4.34E-07	6.47E-06	4.94E-06	3119/3127/3113/3117/3115/3118/3123	7

KEGG	hsa05320	Autoimmune thyroid disease	7/80	53/8096	7.54E-07	1.04E-05	7.93E-06	3119/3127/3113/3117/3115/3118/3123	7
KEGG	hsa05416	Viral myocarditis	7/80	60/8096	1.79E-06	1.88E-05	1.44E-05	3119/3127/3113/3117/3115/3118/3123	7
KEGG	hsa04658	Th1 and Th2 cell differentiation	7/80	92/8096	3.15E-05	0.000255994	0.000195703	3119/3127/3113/3117/3115/3118/3123	7
KEGG	hsa04657	IL-17 signaling pathway	7/80	94/8096	3.62E-05	0.00028161	0.000215286	3553/6280/5743/2920/7124/7128/6279	7
KEGG	hsa04064	NF-kappa B signaling pathway	7/80	104/8096	6.94E-05	0.00049681	0.000379802	3553/5743/597/2920/7124/7185/7128	7
KEGG	hsa04514	Cell adhesion molecules	7/80	149/8096	0.00064187	0.004255362	0.003253144	3119/3127/3113/3117/3115/3118/3123	7
KEGG	hsa04610	Complement and coagulation cascades	6/80	85/8096	0.000181059	0.001246524	0.000952944	5329/1191/5055/11326/5265/7056	6
KEGG	hsa04668	TNF signaling pathway	6/80	112/8096	0.000801933	0.00494986	0.003784074	3553/5743/2920/7124/7185/7128	6
KEGG	hsa04380	Osteoclast differentiation	6/80	128/8096	0.001604601	0.009574119	0.007319233	10288/3553/2214/353514/7124/6688	6
KEGG	hsa05418	Fluid shear stress and atherosclerosis	6/80	139/8096	0.002438423	0.01363993	0.010427468	7850/3553/4208/7124/4205/7056	6
KEGG	hsa05146	Amoebiasis	5/80	102/8096	0.00327563	0.017767811	0.013583154	7850/7414/3553/2920/7124	5

KEGG	hsa04928	Parathyroid hormone synthesis, secretion and action	5/80	106/8096	0.003865387	0.019768691	0.01511279	9935/1958/4208/4205/1026	5
KEGG	hsa04216	Ferroptosis	4/80	41/8096	0.000678133	0.004335206	0.003314182	2495/8031/30061/2180	4
KEGG	hsa05134	Legionellosis	4/80	57/8096	0.002347293	0.013553725	0.010361566	3303/3553/2920/7124	4
KEGG	hsa04662	B cell receptor signaling pathway	4/80	82/8096	0.008632399	0.041762148	0.03192637	10288/353514/974/27071	4
KEGG	hsa01523	Antifolate resistance	3/80	31/8096	0.003421887	0.018015231	0.013772302	3553/2352/7124	3

(5) NK cell

ONTOLOGY	ID	Description	GeneRatio	BgRatio	pvalue	p.adjust	qvalue	geneID	Count
BP	GO:0042110	T cell activation	25/154	474/18862	1.08E-13	1.01E-10	7.37E-11	3821/64332/639/2207/3662/3458/84174/2934/6375/3956/3113/1958/841/9308/3383/10875/57162/3958/925/3115/257101/84807/977/3123/972	25
BP	GO:0034341	response to interferon-gamma	21/154	197/18862	1.03E-17	2.87E-14	2.10E-14	8638/3662/3458/2934/6375/3118/4502/6348/3394/10410/3113/6846/3127/6351/3119/3383/54625/3115/1612/3123/972	21
BP	GO:0001819	positive regulation of cytokine production	20/154	437/18862	4.74E-10	1.65E-07	1.21E-07	2207/8140/3662/3458/6375/3303/6348/3394/3113/1958/841/9308/28984/255231/3304/57162/3115/8013/7124/972	20
BP	GO:0071346	cellular response to interferon-gamma	19/154	177/18862	3.54E-16	4.94E-13	3.62E-13	8638/3662/3458/2934/6375/3118/4502/6348/3394/3113/6846/3127/6351/3119/3383/54625/3115/1612/3123	19

BP	GO:1903706	regulation of hemopoiesis	19/154	415/18862	1.32E-09	3.36E-07	2.46E-07	64332/639/3725/9636/3662/3458/3303/6348/841/9308/3304/10875/3958/257101/8013/84807/7124/3123/972	19
BP	GO:0022407	regulation of cell-cell adhesion	19/154	437/18862	3.10E-09	6.65E-07	4.87E-07	64332/3458/6375/3956/83706/3113/9308/28984/3383/10875/57162/3958/928/3115/8013/84807/7124/3123/972	19
BP	GO:0002697	regulation of immune effector process	19/154	465/18862	8.52E-09	1.70E-06	1.24E-06	7128/3821/64332/8140/3662/3458/6375/117157/3383/10875/3958/257101/3824/8013/11213/84807/7124/3123/972	19
BP	GO:0009615	response to virus	18/154	359/18862	8.82E-10	2.73E-07	2.00E-07	10964/2537/7128/3437/4599/8638/9636/3458/6375/3433/10410/6351/10875/3429/11213/7124/5551/3925	18
BP	GO:0007159	leukocyte cell-cell adhesion	18/154	366/18862	1.20E-09	3.34E-07	2.45E-07	1524/64332/3458/6375/3956/83706/3113/9308/3383/10875/57162/3958/3115/8013/84807/7124/3123/972	18
BP	GO:0002683	negative regulation of immune system process	17/154	403/18862	3.45E-08	5.06E-06	3.71E-06	7128/3821/84174/6375/6348/3949/10875/57162/3958/54625/3824/11213/7124/2876/3123/972/8530	17
BP	GO:1903131	mononuclear cell differentiation	17/154	411/18862	4.59E-08	6.09E-06	4.46E-06	3821/64332/639/2207/3725/3662/3458/3394/3956/1958/9308/10875/925/257101/84807/3123/972	17
BP	GO:0050863	regulation of T cell activation	16/154	327/18862	1.14E-08	2.11E-06	1.55E-06	64332/639/3662/3458/6375/3956/3113/9308/10875/57162/3958/3115/257101/84807/3123/972	16
BP	GO:1903037	regulation of leukocyte cell-cell adhesion	16/154	330/18862	1.29E-08	2.25E-06	1.65E-06	64332/3458/6375/3956/3113/9308/3383/10875/57162/3958/3115/8013/84807/7124/3123/972	16
BP	GO:0002460	adaptive immune response based on somatic recombination of immune receptors built from immunoglobulin superfamily domains	16/154	367/18862	5.69E-08	6.60E-06	4.84E-06	7128/3821/64332/2207/3662/84174/6375/3119/3383/925/3824/84807/7124/5551/3123/972	16
BP	GO:0019058	viral life cycle	15/154	348/18862	1.75E-07	1.57E-05	1.15E-05	4599/8638/9636/2934/3303/10410/3956/3949/2040/3304/3383/3429/7124/3123/972	15
BP	GO:0030098	lymphocyte differentiation	15/154	358/18862	2.52E-07	2.06E-05	1.51E-05	3821/64332/639/2207/3662/3458/3956/1958/9308/10875/925/257101/84807/3123/972	15

BP	GO:0002696	positive regulation of leukocyte activation	15/154	401/18862	1.06E-06	7.18E-05	5.26E-05	64332/3458/6375/6348/952/3956/3113/9308/57162/1026/3115/8013/84807/3123/972	15
BP	GO:0050867	positive regulation of cell activation	15/154	412/18862	1.48E-06	8.91E-05	6.53E-05	64332/3458/6375/6348/952/3956/3113/9308/57162/1026/3115/8013/84807/3123/972	15
BP	GO:0030099	myeloid cell differentiation	15/154	419/18862	1.82E-06	0.00010145	7.43E-05	3725/9636/3662/3458/3303/6348/3394/841/3304/3958/7049/8013/7124/3123/972	15
BP	GO:0045785	positive regulation of cell adhesion	15/154	425/18862	2.17E-06	0.000107011	7.84E-05	64332/3611/3458/6375/3956/3113/9308/3383/80005/3115/8013/84807/7124/3123/972	15
BP	GO:0002429	immune response-activating cell surface receptor signaling pathway	15/154	481/18862	9.67E-06	0.00032068	0.00023497	64332/2207/84174/2214/3118/952/3113/3127/3119/3958/3115/3824/8013/84807/3123	15
BP	GO:0002757	immune response-activating signal transduction	15/154	481/18862	9.67E-06	0.00032068	0.00023497	64332/2207/84174/2214/3118/952/3113/3127/3119/3958/3115/3824/8013/84807/3123	15
BP	GO:0097191	extrinsic apoptotic signaling pathway	14/154	217/18862	2.98E-09	6.65E-07	4.87E-07	2537/7128/3458/3303/7185/841/467/3304/3383/3429/3958/7124/2876/597	14
BP	GO:0030217	T cell differentiation	14/154	246/18862	1.48E-08	2.43E-06	1.78E-06	3821/64332/639/2207/3662/3458/1958/9308/10875/925/257101/84807/3123/972	14
BP	GO:1902105	regulation of leukocyte differentiation	14/154	279/18862	7.18E-08	7.42E-06	5.43E-06	64332/639/3725/3662/3458/6348/841/9308/10875/257101/84807/7124/3123/972	14
BP	GO:2001233	regulation of apoptotic signaling pathway	14/154	348/18862	1.05E-06	7.18E-05	5.26E-05	1524/2537/7128/3303/7185/841/467/3304/3383/3958/10105/7124/2876/972	14
BP	GO:0002449	lymphocyte mediated immunity	14/154	360/18862	1.56E-06	9.06E-05	6.64E-05	3821/2207/84174/6375/117157/3119/3383/3002/925/3824/7124/5551/3123/972	14
BP	GO:0060333	interferon-gamma-mediated signaling pathway	13/154	91/18862	4.76E-13	3.31E-10	2.43E-10	8638/3662/3458/3118/4502/3394/3113/3127/3119/3383/54625/3115/3123	13
BP	GO:1903039	positive regulation of leukocyte cell-cell adhesion	13/154	234/18862	6.59E-08	7.35E-06	5.38E-06	64332/3458/6375/3956/3113/9308/3383/3115/8013/84807/7124/3123/972	13

BP	GO:0051607	defense response to virus	13/154	260/18862	2.24E-07	1.89E-05	1.39E-05	10964/2537/7128/3437/4599/8638/9636/3458/3433/10410/10875/3429/5551	13
BP	GO:0140546	defense response to symbiont	13/154	260/18862	2.24E-07	1.89E-05	1.39E-05	10964/2537/7128/3437/4599/8638/9636/3458/3433/10410/10875/3429/5551	13
BP	GO:0022409	positive regulation of cell-cell adhesion	13/154	276/18862	4.43E-07	3.25E-05	2.38E-05	64332/3458/6375/3956/3113/9308/3383/3115/8013/84807/7124/3123/972	13
BP	GO:0051251	positive regulation of lymphocyte activation	13/154	356/18862	7.40E-06	0.000263728	0.0001932 4	64332/3458/6375/952/3956/3113/9308/57162/1026/3115/84807/3123/972	13
BP	GO:0006979	response to oxidative stress	13/154	444/18862	7.39E-05	0.001513781	0.001109186	7128/3039/3725/3040/3303/952/3304/10105/143686/8013/1612/3043/2876	13
BP	GO:0051090	regulation of DNA-binding transcription factor activity	13/154	444/18862	7.39E-05	0.001513781	0.001109186	1524/7128/3725/6375/3303/7185/28984/3304/3383/57162/11213/7124/3397	13
BP	GO:0060337	type I interferon signaling pathway	12/154	95/18862	1.74E-11	9.16E-09	6.71E-09	2537/3437/4599/8638/9636/3662/54739/3394/3433/10410/1958/3429	12
BP	GO:0071357	cellular response to type I interferon	12/154	96/18862	1.97E-11	9.16E-09	6.71E-09	2537/3437/4599/8638/9636/3662/54739/3394/3433/10410/1958/3429	12
BP	GO:0034340	response to type I interferon	12/154	101/18862	3.63E-11	1.45E-08	1.06E-08	2537/3437/4599/8638/9636/3662/54739/3394/3433/10410/1958/3429	12
BP	GO:0002285	lymphocyte activation involved in immune response	12/154	189/18862	5.01E-08	6.34E-06	4.65E-06	64332/2207/3662/3458/3956/3383/10875/3958/257101/84807/3123/972	12
BP	GO:2001234	negative regulation of apoptotic signaling pathway	12/154	224/18862	3.19E-07	2.54E-05	1.86E-05	1524/2537/7128/3303/841/3304/3383/3958/10105/7124/2876/972	12
BP	GO:0045637	regulation of myeloid cell differentiation	12/154	258/18862	1.43E-06	8.91E-05	6.53E-05	3725/9636/3458/3303/6348/841/3304/3958/8013/7124/3123/972	12
BP	GO:0071356	cellular response to tumor necrosis factor	12/154	296/18862	5.89E-06	0.000218703	0.000160249	7128/3611/6375/3303/6348/7185/6846/6351/841/3304/3383/7124	12

BP	GO:0070661	leukocyte proliferation	12/154	312/18862	1.00E-05	0.000324617	0.000237855	7128/6375/952/3113/3070/57162/3958/1026/3115/977/3123/972	12
BP	GO:0034612	response to tumor necrosis factor	12/154	320/18862	1.29E-05	0.000397615	0.000291343	7128/3611/6375/3303/6348/7185/6846/6351/841/3304/3383/7124	12
BP	GO:0032102	negative regulation of response to external stimulus	12/154	394/18862	9.73E-05	0.001737714	0.001273267	7128/3821/3949/10875/928/54625/3824/11213/7124/2876/3123/8530	12
BP	GO:0043312	neutrophil degranulation	12/154	485/18862	0.000644849	0.007047819	0.005164115	2495/2207/7414/2934/3303/2040/6282/3304/10875/3958/23406/3043	12
BP	GO:0002283	neutrophil activation involved in immune response	12/154	488/18862	0.000680604	0.007380714	0.005408035	2495/2207/7414/2934/3303/2040/6282/3304/10875/3958/23406/3043	12
BP	GO:0002446	neutrophil mediated immunity	12/154	499/18862	0.000826282	0.008689991	0.006367376	2495/2207/7414/2934/3303/2040/6282/3304/10875/3958/23406/3043	12
BP	GO:0042119	neutrophil activation	12/154	500/18862	0.000840729	0.0087757	0.006430177	2495/2207/7414/2934/3303/2040/6282/3304/10875/3958/23406/3043	12
BP	GO:2001236	regulation of extrinsic apoptotic signaling pathway	11/154	154/18862	5.54E-08	6.60E-06	4.84E-06	2537/7128/3303/7185/841/467/3304/3383/3958/7124/2876	11
BP	GO:0002699	positive regulation of immune effector process	11/154	219/18862	1.89E-06	0.000103308	7.57E-05	64332/8140/3458/6375/117157/3824/8013/84807/7124/3123/972	11
BP	GO:0019882	antigen processing and presentation	11/154	234/18862	3.59E-06	0.000156255	0.000114492	2207/3118/3113/3127/3119/3383/10875/925/3115/3123/972	11
BP	GO:0007249	I-kappaB kinase/NF-kappaB signaling	11/154	282/18862	2.08E-05	0.000575078	0.000421374	1524/7128/3956/7185/841/57162/7124/6285/3123/972/6275	11
BP	GO:0046651	lymphocyte proliferation	11/154	282/18862	2.08E-05	0.000575078	0.000421374	6375/952/3113/3070/57162/3958/1026/3115/977/3123/972	11
BP	GO:0032943	mononuclear cell proliferation	11/154	285/18862	2.30E-05	0.000601843	0.000440985	6375/952/3113/3070/57162/3958/1026/3115/977/3123/972	11

BP	GO:0050851	antigen receptor-mediated signaling pathway	11/154	323/18862	7.15E-05	0.001487691	0.001090069	64332/84174/3118/952/3113/3127/3119/3958/3115/84807/3123	11
BP	GO:0032496	response to lipopolysaccharide	11/154	326/18862	7.77E-05	0.001550144	0.00113583	1524/7128/3725/6348/3394/3248/841/3383/57162/11213/7124	11
BP	GO:0002237	response to molecule of bacterial origin	11/154	346/18862	0.000131413	0.002129343	0.001560223	1524/7128/3725/6348/3394/3248/841/3383/57162/11213/7124	11
BP	GO:0050727	regulation of inflammatory response	11/154	366/18862	0.000214071	0.003013023	0.002207718	7128/64332/3458/6375/6348/3949/7124/2876/3123/283131/8530	11
BP	GO:1903793	positive regulation of anion transport	11/154	478/18862	0.001923063	0.015951123	0.011687791	4946/3458/952/7533/2040/841/3002/8013/19/7124/3123	11
BP	GO:0002286	T cell activation involved in immune response	10/154	111/18862	2.50E-08	3.87E-06	2.83E-06	64332/2207/3662/3458/3383/10875/3958/84807/3123/972	10
BP	GO:0008637	apoptotic mitochondrial changes	10/154	118/18862	4.50E-08	6.09E-06	4.46E-06	2537/3725/3303/3433/7533/841/3002/407021/10105/2876	10
BP	GO:0045580	regulation of T cell differentiation	10/154	146/18862	3.36E-07	2.60E-05	1.91E-05	64332/639/3662/3458/9308/10875/257101/84807/3123/972	10
BP	GO:0050777	negative regulation of immune response	10/154	150/18862	4.32E-07	3.25E-05	2.38E-05	7128/3821/6375/10875/3958/54625/3824/11213/2876/3123	10
BP	GO:0045619	regulation of lymphocyte differentiation	10/154	175/18862	1.77E-06	0.000100878	7.39E-05	64332/639/3662/3458/9308/10875/257101/84807/3123/972	10
BP	GO:0001959	regulation of cytokine-mediated signaling pathway	10/154	177/18862	1.97E-06	0.000105345	7.72E-05	7128/3458/3303/7185/841/3304/54625/11213/7124/972	10
BP	GO:0001906	cell killing	10/154	179/18862	2.18E-06	0.000107011	7.84E-05	3821/3458/6375/10578/841/3383/3002/3824/5551/3123	10
BP	GO:0060759	regulation of response to cytokine stimulus	10/154	189/18862	3.54E-06	0.000156255	0.000114492	7128/3458/3303/7185/841/3304/54625/11213/7124/972	10

BP	GO:0050866	negative regulation of cell activation	10/154	200/18862	5.86E-06	0.000218703	0.000160249	7128/6375/3949/10875/57162/3958/928/3123/972/8530	10
BP	GO:0050852	T cell receptor signaling pathway	10/154	204/18862	6.98E-06	0.000252549	0.000185049	64332/84174/3118/3113/3127/3119/3958/3115/84807/3123	10
BP	GO:0050870	positive regulation of T cell activation	10/154	212/18862	9.78E-06	0.000320682	0.000234972	64332/3458/6375/3956/3113/9308/3115/84807/3123/972	10
BP	GO:0051701	biological process involved in interaction with host	10/154	219/18862	1.30E-05	0.000397615	0.000291343	2934/3303/10410/3956/3949/841/3304/3383/3123/972	10
BP	GO:0031348	negative regulation of defense response	10/154	236/18862	2.47E-05	0.000638447	0.000467806	7128/3821/3949/10875/54625/3824/11213/2876/3123/8530	10
BP	GO:0070663	regulation of leukocyte proliferation	10/154	241/18862	2.96E-05	0.000729823	0.000534759	7128/6375/952/3113/57162/3958/1026/3115/3123/972	10
BP	GO:0043122	regulation of I-kappaB kinase/NF-kappaB signaling	10/154	244/18862	3.29E-05	0.000803716	0.000588903	7128/3956/7185/841/57162/7124/6285/3123/972/6275	10
BP	GO:0072593	reactive oxygen species metabolic process	10/154	281/18862	0.000107008	0.001871703	0.001371444	1524/2537/3039/3458/3040/3383/1026/7124/3043/2876	10
BP	GO:0070372	regulation of ERK1 and ERK2 cascade	10/154	301/18862	0.000187313	0.002754914	0.002018595	6375/6348/1846/6846/6351/467/3383/7124/3123/972	10
BP	GO:0070371	ERK1 and ERK2 cascade	10/154	320/18862	0.000305639	0.003907411	0.002863058	6375/6348/1846/6846/6351/467/3383/7124/3123/972	10
BP	GO:0001818	negative regulation of cytokine production	10/154	367/18862	0.000885506	0.009208605	0.006747377	1524/7128/9636/3458/6375/9308/28984/11213/7124/3123	10
BP	GO:1902903	regulation of supramolecular fiber organization	10/154	370/18862	0.000941862	0.009545341	0.006994112	2934/3303/3949/28984/3304/3383/23406/3397/2876/3925	10
BP	GO:0050678	regulation of epithelial cell proliferation	10/154	374/18862	0.001021555	0.010131937	0.007423926	374/7128/28984/407021/7049/8013/7124/3397/2876/3164	10

BP	GO:0002831	regulation of response to biotic stimulus	10/154	420/18862	0.002404472	0.019037681	0.013949389	7128/3821/2207/3458/117157/10875/54625/3824/11213/3123	10
BP	GO:0050673	epithelial cell proliferation	10/154	428/18862	0.002753632	0.021083444	0.015448371	374/7128/28984/407021/7049/8013/7124/3397/2876/3164	10
BP	GO:0043254	regulation of protein-containing complex assembly	10/154	446/18862	0.003688526	0.025828949	0.018925523	3458/2934/3303/3304/3383/3958/23406/19/7124/3925	10
BP	GO:0009896	positive regulation of catabolic process	10/154	450/18862	0.003927064	0.02726518	0.019977886	7128/4946/3458/3303/3949/3304/51099/143686/1612/7124	10
BP	GO:0043087	regulation of GTPase activity	10/154	487/18862	0.006762382	0.040530667	0.029697843	5996/3725/6375/6348/6846/6351/3383/80005/253959/3925	10
BP	GO:0046634	regulation of alpha-beta T cell activation	9/154	101/18862	1.40E-07	1.40E-05	1.02E-05	64332/639/3662/3458/6375/9308/257101/84807/3123	9
BP	GO:0045639	positive regulation of myeloid cell differentiation	9/154	102/18862	1.53E-07	1.47E-05	1.08E-05	3725/9636/3458/3303/841/3304/7124/3123/972	9
BP	GO:2001237	negative regulation of extrinsic apoptotic signaling pathway	9/154	103/18862	1.66E-07	1.55E-05	1.13E-05	2537/7128/3303/841/3304/3383/3958/7124/2876	9
BP	GO:0002687	positive regulation of leukocyte migration	9/154	133/18862	1.47E-06	8.91E-05	6.53E-05	1524/6375/6348/6846/6351/3383/3958/7124/972	9
BP	GO:0046718	viral entry into host cell	9/154	140/18862	2.25E-06	0.000107986	7.91E-05	2934/3303/10410/3956/3949/3304/3383/3123/972	9
BP	GO:1903900	regulation of viral life cycle	9/154	148/18862	3.56E-06	0.000156255	0.000114492	4599/8638/9636/2934/10410/3956/7124/3123/972	9
BP	GO:0046631	alpha-beta T cell activation	9/154	149/18862	3.76E-06	0.000161172	0.000118095	64332/639/3662/3458/6375/9308/257101/84807/3123	9
BP	GO:0044409	entry into host	9/154	153/18862	4.67E-06	0.000194358	0.000142411	2934/3303/10410/3956/3949/3304/3383/3123/972	9

BP	GO:1902107	positive regulation of leukocyte differentiation	9/154	154/18862	4.93E-06	0.000196213	0.00014377	64332/3725/3458/841/9308/84807/7124/3123/972	9
BP	GO:1903708	positive regulation of hemopoiesis	9/154	154/18862	4.93E-06	0.000196213	0.00014377	64332/3725/3458/841/9308/84807/7124/3123/972	9
BP	GO:0052126	movement in host environment	9/154	175/18862	1.39E-05	0.000415491	0.000304441	2934/3303/10410/3956/3949/3304/3383/3123/972	9
BP	GO:0002695	negative regulation of leukocyte activation	9/154	182/18862	1.90E-05	0.000539088	0.000395003	7128/6375/3949/10875/57162/3958/3123/972/8530	9
BP	GO:0050792	regulation of viral process	9/154	186/18862	2.25E-05	0.000597825	0.000438042	4599/8638/9636/2934/10410/3956/7124/3123/972	9
BP	GO:0043903	regulation of biological process involved in symbiotic interaction	9/154	197/18862	3.54E-05	0.000850601	0.000623257	4599/8638/9636/2934/10410/3956/7124/3123/972	9
BP	GO:0002685	regulation of leukocyte migration	9/154	205/18862	4.83E-05	0.001099343	0.000805516	1524/6375/6348/6846/6351/3383/3958/7124/972	9
BP	GO:0070555	response to interleukin-1	9/154	206/18862	5.01E-05	0.001127077	0.000825838	6375/6348/952/6846/1958/6351/3383/57162/11213	9
BP	GO:0097529	myeloid leukocyte migration	9/154	218/18862	7.76E-05	0.001550144	0.00113583	1524/2207/6375/6348/6846/6351/255231/3958/972	9
BP	GO:0050670	regulation of lymphocyte proliferation	9/154	221/18862	8.62E-05	0.001611586	0.00118085	6375/952/3113/57162/3958/1026/3115/3123/972	9
BP	GO:0032944	regulation of mononuclear cell proliferation	9/154	223/18862	9.23E-05	0.001690924	0.001238982	6375/952/3113/57162/3958/1026/3115/3123/972	9
BP	GO:0000302	response to reactive oxygen species	9/154	224/18862	9.55E-05	0.001717098	0.001258161	7128/3039/3725/3040/10105/143686/8013/3043/2876	9
BP	GO:0009636	response to toxic substance	9/154	239/18862	0.000155924	0.00240088	0.001759185	3039/3040/4502/6348/6351/1026/4493/3043/2876	9

BP	GO:0007162	negative regulation of cell adhesion	9/154	295/18862	0.000726128	0.007831431	0.005738287	6375/3956/28984/10875/57162/3958/928/3123/972		9
BP	GO:0060326	cell chemotaxis	9/154	306/18862	0.000940206	0.009545341	0.006994112	1524/2207/6375/6348/6846/6351/3958/3164/972		9
BP	GO:0051222	positive regulation of protein transport	9/154	312/18862	0.001077024	0.010458766	0.007663402	4946/3458/952/7533/2040/841/3002/7124/3123		9
BP	GO:0051235	maintenance of location	9/154	319/18862	0.001256601	0.011635041	0.008525289	2495/2934/6375/6348/255231/51099/19/1407/7124		9
BP	GO:1904951	positive regulation of establishment of protein localization	9/154	328/18862	0.001522267	0.013511335	0.009900096	4946/3458/952/7533/2040/841/3002/7124/3123		9
BP	GO:0010631	epithelial cell migration	9/154	357/18862	0.002699245	0.020723954	0.015184964	9839/3458/28984/407021/80005/7124/3397/2876/3164		9
BP	GO:0051098	regulation of binding	9/154	357/18862	0.002699245	0.020723954	0.015184964	3725/3662/3458/3433/1026/3001/7049/3397/3925		9
BP	GO:0090132	epithelium migration	9/154	360/18862	0.00285364	0.021299416	0.01560662	9839/3458/28984/407021/80005/7124/3397/2876/3164		9
BP	GO:0090130	tissue migration	9/154	365/18862	0.003126437	0.022880067	0.016764803	9839/3458/28984/407021/80005/7124/3397/2876/3164		9
BP	GO:0042176	regulation of protein catabolic process	9/154	383/18862	0.004283163	0.028833756	0.021127221	7128/4946/3458/3303/3949/3304/11213/7124/2876		9
BP	GO:0031331	positive regulation of cellular catabolic process	9/154	384/18862	0.004356073	0.029183594	0.021383556	7128/3458/3303/3949/3304/51099/143686/1612/7124		9
BP	GO:0043547	positive regulation of GTPase activity	9/154	411/18862	0.006719023	0.040357581	0.029571018	5996/3725/6375/6348/6846/6351/3383/80005/253959		9
BP	GO:0046637	regulation of alpha-beta T cell differentiation	8/154	67/18862	7.14E-08	7.42E-06	5.43E-06	64332/639/3662/3458/9308/257101/84807/3123		8

BP	GO:0031341	regulation of cell killing	8/154	98/18862	1.39E-06	8.91E-05	6.53E-05	3821/3458/6375/841/3383/3824/5551/3123	8
BP	GO:0019886	antigen processing and presentation of exogenous peptide antigen via MHC class II	8/154	99/18862	1.50E-06	8.91E-05	6.53E-05	2207/3118/3113/3127/3119/3115/3123/972	8
BP	GO:0002495	antigen processing and presentation of peptide antigen via MHC class II	8/154	103/18862	2.03E-06	0.000106766	7.82E-05	2207/3118/3113/3127/3119/3115/3123/972	8
BP	GO:0002504	antigen processing and presentation of peptide or polysaccharide antigen via MHC class II	8/154	104/18862	2.18E-06	0.000107011	7.84E-05	2207/3118/3113/3127/3119/3115/3123/972	8
BP	GO:0032649	regulation of interferon-gamma production	8/154	107/18862	2.71E-06	0.000125783	9.22E-05	8140/9636/6375/3394/3113/3115/7124/3123	8
BP	GO:0046632	alpha-beta T cell differentiation	8/154	107/18862	2.71E-06	0.000125783	9.22E-05	64332/639/3662/3458/9308/257101/84807/3123	8
BP	GO:0032609	interferon-gamma production	8/154	112/18862	3.82E-06	0.000161172	0.000118095	8140/9636/6375/3394/3113/3115/7124/3123	8
BP	GO:0035821	modulation of process of other organism	8/154	123/18862	7.66E-06	0.000263728	0.00019324	3725/3458/6348/2040/6351/841/3429/5551	8
BP	GO:1990266	neutrophil migration	8/154	123/18862	7.66E-06	0.000263728	0.00019324	2207/6375/6348/6846/6351/255231/3958/972	8
BP	GO:0042542	response to hydrogen peroxide	8/154	135/18862	1.52E-05	0.000450083	0.000329787	7128/3039/3725/3040/10105/8013/3043/2876	8
BP	GO:0097530	granulocyte migration	8/154	148/18862	2.95E-05	0.000729823	0.000534759	2207/6375/6348/6846/6351/255231/3958/972	8
BP	GO:0002822	regulation of adaptive immune response based on somatic	8/154	155/18862	4.11E-05	0.000962259	0.000705071	7128/3821/64332/6375/3824/84807/7124/3123	8

		recombination of immune receptors built from immunoglobulin superfamily domains							
BP	GO:0002819	regulation of adaptive immune response	8/154	170/18862	7.89E-05	0.001552222	0.001137352	7128/3821/64332/6375/3824/84807/7124/3123	8
BP	GO:0002478	antigen processing and presentation of exogenous peptide antigen	8/154	177/18862	0.000104569	0.001856269	0.001360135	2207/3118/3113/3127/3119/3115/3123/972	8
BP	GO:0071347	cellular response to interleukin-1	8/154	180/18862	0.000117504	0.002009101	0.001472119	6375/6348/6846/1958/6351/3383/57162/11213	8
BP	GO:0043123	positive regulation of I-kappaB kinase/NF-kappaB signaling	8/154	182/18862	0.000126841	0.002107196	0.001543996	3956/841/57162/7124/6285/3123/972/6275	8
BP	GO:0019884	antigen processing and presentation of exogenous antigen	8/154	185/18862	0.000141989	0.002274274	0.001666418	2207/3118/3113/3127/3119/3115/3123/972	8
BP	GO:0022408	negative regulation of cell-cell adhesion	8/154	189/18862	0.00016448	0.002491339	0.001825467	6375/28984/10875/57162/3958/928/3123/972	8
BP	GO:0048002	antigen processing and presentation of peptide antigen	8/154	194/18862	0.000196642	0.002824951	0.002069913	2207/3118/3113/3127/3119/3115/3123/972	8
BP	GO:1905475	regulation of protein localization to membrane	8/154	194/18862	0.000196642	0.002824951	0.002069913	3458/2934/7533/2040/841/3002/3958/7124	8
BP	GO:0002573	myeloid leukocyte differentiation	8/154	204/18862	0.000276534	0.003601409	0.002638843	3725/3662/3458/6348/841/7124/3123/972	8
BP	GO:0070374	positive regulation of ERK1 and ERK2 cascade	8/154	210/18862	0.000336042	0.004107669	0.003009793	6375/6348/6846/6351/3383/7124/3123/972	8
BP	GO:0033002	muscle cell proliferation	8/154	222/18862	0.000486474	0.005556573	0.004071442	7128/3725/3458/3248/1026/7049/8013/7124	8

BP	GO:0031334	positive regulation of protein-containing complex assembly	8/154	254/18862	0.001167207	0.011140427	0.008162872	3458/2934/3303/3304/3383/3958/19/7124	8
BP	GO:0051091	positive regulation of DNA-binding transcription factor activity	8/154	266/18862	0.001564	0.013707135	0.010043564	1524/3303/7185/28984/3304/3383/11213/7124	8
BP	GO:0030522	intracellular receptor signaling pathway	8/154	274/18862	0.001883223	0.015667291	0.01147982	7128/3303/7533/841/3304/8013/1407/3164	8
BP	GO:0097193	intrinsic apoptotic signaling pathway	8/154	283/18862	0.00230166	0.018275571	0.013390972	2537/3303/10105/1026/7124/2876/597/972	8
BP	GO:0002440	production of molecular mediator of immune response	8/154	286/18862	0.002456288	0.019338061	0.014169485	8140/6375/3119/8013/11213/7124/3123/972	8
BP	GO:0034599	cellular response to oxidative stress	8/154	299/18862	0.003222874	0.023452091	0.017183939	7128/3725/3303/3304/10105/8013/1612/2876	8
BP	GO:1901214	regulation of neuron death	8/154	302/18862	0.003423688	0.024675709	0.018080515	1524/3725/3458/6348/1958/841/8013/7124	8
BP	GO:0045088	regulation of innate immune response	8/154	315/18862	0.004408638	0.029464924	0.021589694	7128/3821/2207/3458/117157/54625/3824/11213	8
BP	GO:0070997	neuron death	8/154	342/18862	0.007139977	0.041892877	0.030695968	1524/3725/3458/6348/1958/841/8013/7124	8
BP	GO:0062197	cellular response to chemical stress	8/154	347/18862	0.007761679	0.043967072	0.032215784	7128/3725/3303/3304/10105/8013/1612/2876	8
BP	GO:0051271	negative regulation of cellular component movement	8/154	352/18862	0.00842335	0.046031132	0.033728172	1524/7414/28984/407021/928/7049/7124/972	8
BP	GO:2000514	regulation of CD4-positive, alpha-beta T cell activation	7/154	67/18862	1.22E-06	8.08E-05	5.92E-05	64332/3662/3458/6375/9308/84807/3123	7
BP	GO:0002292	T cell differentiation involved in immune response	7/154	73/18862	2.19E-06	0.000107011	7.84E-05	64332/2207/3662/3458/10875/84807/3123	7

BP	GO:0008625	extrinsic apoptotic signaling pathway via death domain receptors	7/154	84/18862	5.64E-06	0.000218703	0.000160249	7128/841/467/3383/3958/7124/2876	7
BP	GO:0035710	CD4-positive, alpha-beta T cell activation	7/154	100/18862	1.79E-05	0.000519411	0.000380585	64332/3662/3458/6375/9308/84807/3123	7
BP	GO:0030593	neutrophil chemotaxis	7/154	103/18862	2.17E-05	0.000581466	0.000426055	2207/6375/6348/6846/6351/3958/972	7
BP	GO:0032651	regulation of interleukin-1 beta production	7/154	103/18862	2.17E-05	0.000581466	0.000426055	1524/7128/3458/6348/1958/841/7124	7
BP	GO:0002456	T cell mediated immunity	7/154	104/18862	2.31E-05	0.000601843	0.000440985	3821/6375/3383/925/3824/5551/3123	7
BP	GO:0032611	interleukin-1 beta production	7/154	108/18862	2.95E-05	0.000729823	0.000534759	1524/7128/3458/6348/1958/841/7124	7
BP	GO:0001909	leukocyte mediated cytotoxicity	7/154	113/18862	3.95E-05	0.000933989	0.000684357	3821/6375/3383/3002/3824/5551/3123	7
BP	GO:0002761	regulation of myeloid leukocyte differentiation	7/154	118/18862	5.21E-05	0.001162551	0.00085183	3725/3458/6348/841/7124/3123/972	7
BP	GO:0032652	regulation of interleukin-1 production	7/154	119/18862	5.50E-05	0.001207529	0.000884787	1524/7128/3458/6348/1958/841/7124	7
BP	GO:0071621	granulocyte chemotaxis	7/154	124/18862	7.15E-05	0.001487691	0.001090069	2207/6375/6348/6846/6351/3958/972	7
BP	GO:0002698	negative regulation of immune effector process	7/154	125/18862	7.52E-05	0.001529839	0.001120952	3821/6375/10875/3958/3824/11213/7124	7
BP	GO:0032612	interleukin-1 production	7/154	126/18862	7.91E-05	0.001552222	0.001137352	1524/7128/3458/6348/1958/841/7124	7
BP	GO:1905477	positive regulation of protein localization to membrane	7/154	127/18862	8.31E-05	0.001580595	0.001158142	3458/7533/2040/841/3002/3958/7124	7

BP	GO:0019079	viral genome replication	7/154	129/18862	9.17E-05	0.001690924	0.001238982	4599/8638/9636/10410/2040/3429/7124	7
BP	GO:0050671	positive regulation of lymphocyte proliferation	7/154	135/18862	0.000121989	0.002060509	0.001509787	6375/952/3113/57162/1026/3115/972	7
BP	GO:0032946	positive regulation of mononuclear cell proliferation	7/154	136/18862	0.00012774	0.002107196	0.001543996	6375/952/3113/57162/1026/3115/972	7
BP	GO:0070665	positive regulation of leukocyte proliferation	7/154	148/18862	0.000215611	0.003013023	0.002207718	6375/952/3113/57162/1026/3115/972	7
BP	GO:0051250	negative regulation of lymphocyte activation	7/154	153/18862	0.000264268	0.003490597	0.002557648	7128/6375/10875/57162/3958/3123/972	7
BP	GO:0051092	positive regulation of NF-kappaB transcription factor activity	7/154	159/18862	0.000333932	0.004099866	0.003004075	1524/3303/7185/3304/3383/11213/7124	7
BP	GO:0048660	regulation of smooth muscle cell proliferation	7/154	160/18862	0.000346855	0.004184783	0.003066296	7128/3725/3458/3248/1026/8013/7124	7
BP	GO:0048659	smooth muscle cell proliferation	7/154	162/18862	0.000373903	0.004491671	0.00329116	7128/3725/3458/3248/1026/8013/7124	7
BP	GO:0033209	tumor necrosis factor-mediated signaling pathway	7/154	174/18862	0.000573775	0.006474131	0.00474376	7128/3611/3303/7185/841/3304/7124	7
BP	GO:0010469	regulation of signaling receptor activity	7/154	188/18862	0.000905594	0.009313249	0.006824053	374/100462981/3458/100463486/1612/7124/100463498	7
BP	GO:0071674	mononuclear cell migration	7/154	190/18862	0.000963306	0.009622697	0.007050793	6375/6348/6846/6351/3383/3958/7124	7
BP	GO:0042098	T cell proliferation	7/154	195/18862	0.001120313	0.010803846	0.007916251	6375/3113/57162/3958/3115/977/3123	7
BP	GO:1902905	positive regulation of supramolecular fiber organization	7/154	203/18862	0.001412624	0.012741043	0.009335684	2934/3303/28984/3304/3383/3397/2876	7

BP	GO:0002221	pattern recognition receptor signaling pathway	7/154	208/18862	0.00162356	0.014184517	0.010393354	7128/3662/3303/841/3304/57162/11213	7
BP	GO:0002703	regulation of leukocyte mediated immunity	7/154	209/18862	0.001668543	0.014311445	0.010486357	3821/6375/117157/3383/3824/7124/3123	7
BP	GO:0045732	positive regulation of protein catabolic process	7/154	225/18862	0.002529222	0.019856176	0.014549122	7128/4946/3458/3303/3949/3304/7124	7
BP	GO:0019724	B cell mediated immunity	7/154	226/18862	0.002592571	0.020126729	0.014747363	2207/84174/6375/3119/7124/3123/972	7
BP	GO:0030595	leukocyte chemotaxis	7/154	226/18862	0.002592571	0.020126729	0.014747363	2207/6375/6348/6846/6351/3958/972	7
BP	GO:2000116	regulation of cysteine-type endopeptidase activity	7/154	230/18862	0.002858264	0.021299416	0.01560662	2537/2934/841/1612/7124/2876/8530	7
BP	GO:0097305	response to alcohol	7/154	233/18862	0.003070879	0.022581901	0.016546329	2934/6348/3248/841/3383/1026/7049	7
BP	GO:1903320	regulation of protein modification by small protein conjugation or removal	7/154	237/18862	0.003372964	0.024480341	0.017937364	7128/9636/3303/1958/3304/57162/1407	7
BP	GO:0051896	regulation of protein kinase B signaling	7/154	247/18862	0.004227487	0.028527863	0.020903085	374/1524/6348/407021/143686/7124/2876	7
BP	GO:0033157	regulation of intracellular protein transport	7/154	248/18862	0.004321187	0.029019634	0.021263419	4946/3458/7533/2040/841/3429/3002	7
BP	GO:0071560	cellular response to transforming growth factor beta stimulus	7/154	251/18862	0.004611741	0.030675229	0.022476515	1524/3725/6375/3303/3248/7049/3397	7
BP	GO:0071559	response to transforming growth factor beta	7/154	257/18862	0.005236881	0.033246439	0.024360505	1524/3725/6375/3303/3248/7049/3397	7
BP	GO:0043491	protein kinase B signaling	7/154	273/18862	0.00721541	0.042243779	0.030953083	374/1524/6348/407021/143686/7124/2876	7

BP	GO:0042063	gliogenesis	7/154	283/18862	0.008705355	0.047479106	0.03478914	1524/3458/2934/6348/3949/928/7124	7
BP	GO:2000516	positive regulation of CD4-positive, alpha-beta T cell activation	6/154	39/18862	7.02E-07	5.01E-05	3.67E-05	64332/3458/6375/9308/84807/3123	6
BP	GO:0043370	regulation of CD4-positive, alpha-beta T cell differentiation	6/154	51/18862	3.57E-06	0.000156255	0.000114492	64332/3662/3458/9308/84807/3123	6
BP	GO:0002763	positive regulation of myeloid leukocyte differentiation	6/154	58/18862	7.66E-06	0.000263728	0.00019324	3725/3458/841/7124/3123/972	6
BP	GO:1902041	regulation of extrinsic apoptotic signaling pathway via death domain receptors	6/154	59/18862	8.47E-06	0.000287899	0.000210951	7128/841/467/3383/3958/2876	6
BP	GO:1902686	mitochondrial outer membrane permeabilization involved in programmed cell death	6/154	62/18862	1.13E-05	0.00036284	0.000265862	3303/7533/841/3002/407021/10105	6
BP	GO:0010803	regulation of tumor necrosis factor-mediated signaling pathway	6/154	63/18862	1.24E-05	0.000389399	0.000285323	7128/3303/7185/841/3304/7124	6
BP	GO:0071677	positive regulation of mononuclear cell migration	6/154	63/18862	1.24E-05	0.000389399	0.000285323	6375/6348/6846/6351/3958/7124	6
BP	GO:0035794	positive regulation of mitochondrial membrane permeability	6/154	64/18862	1.36E-05	0.00041289	0.000302535	3303/7533/841/3002/407021/10105	6
BP	GO:0046635	positive regulation of alpha-beta T cell activation	6/154	66/18862	1.63E-05	0.000478106	0.00035032	64332/3458/6375/9308/84807/3123	6
BP	GO:1905710	positive regulation of membrane permeability	6/154	69/18862	2.11E-05	0.000575808	0.000421909	3303/7533/841/3002/407021/10105	6
BP	GO:0046902	regulation of mitochondrial membrane permeability	6/154	75/18862	3.40E-05	0.000824028	0.000603786	3303/7533/841/3002/407021/10105	6

BP	GO:0043367	CD4-positive, alpha-beta T cell differentiation	6/154	81/18862	5.26E-05	0.001164407	0.00085319	64332/3662/3458/9308/84807/3123	6
BP	GO:0002718	regulation of cytokine production involved in immune response	6/154	84/18862	6.46E-05	0.001374834	0.001007376	8140/6375/8013/11213/7124/972	6
BP	GO:0034109	homotypic cell-cell adhesion	6/154	85/18862	6.91E-05	0.001458248	0.001068495	7414/3611/3956/83706/928/3043	6
BP	GO:0045582	positive regulation of T cell differentiation	6/154	88/18862	8.39E-05	0.001580595	0.001158142	64332/3458/9308/84807/3123/972	6
BP	GO:0048525	negative regulation of viral process	6/154	89/18862	8.93E-05	0.001660003	0.001216326	4599/8638/9636/2934/10410/7124	6
BP	GO:0090559	regulation of membrane permeability	6/154	90/18862	9.51E-05	0.001717098	0.001258161	3303/7533/841/3002/407021/10105	6
BP	GO:0051702	biological process involved in interaction with symbiont	6/154	92/18862	0.000107453	0.001871703	0.001371444	3725/6348/2040/6351/3429/2876	6
BP	GO:1901216	positive regulation of neuron death	6/154	92/18862	0.000107453	0.001871703	0.001371444	3725/3458/6348/1958/841/7124	6
BP	GO:0002367	cytokine production involved in immune response	6/154	93/18862	0.000114098	0.00196291	0.001438274	8140/6375/8013/11213/7124/972	6
BP	GO:0051817	modulation of process of other organism involved in symbiotic interaction	6/154	98/18862	0.000152382	0.002359376	0.001728774	3725/6348/2040/6351/841/3429	6
BP	GO:0002832	negative regulation of response to biotic stimulus	6/154	100/18862	0.000170275	0.002565168	0.001879564	7128/3821/10875/54625/3824/11213	6
BP	GO:0045621	positive regulation of lymphocyte differentiation	6/154	101/18862	0.000179823	0.002680039	0.001963732	64332/3458/9308/84807/3123/972	6

BP	GO:0042116	macrophage activation	6/154	102/18862	0.00018979	0.002754914	0.002018595	3458/6348/3949/7124/972/8530	6
BP	GO:0062207	regulation of pattern recognition receptor signaling pathway	6/154	105/18862	0.000222328	0.003052356	0.002236538	7128/3662/3303/3304/57162/11213	6
BP	GO:0071675	regulation of mononuclear cell migration	6/154	111/18862	0.000300516	0.003859623	0.002828044	6375/6348/6846/6351/3958/7124	6
BP	GO:0050868	negative regulation of T cell activation	6/154	119/18862	0.000436623	0.005134461	0.003762149	6375/10875/57162/3958/3123/972	6
BP	GO:0098754	detoxification	6/154	138/18862	0.000952565	0.009549638	0.006997261	3039/3040/4502/4493/3043/2876	6
BP	GO:1903038	negative regulation of leukocyte cell-cell adhesion	6/154	138/18862	0.000952565	0.009549638	0.006997261	6375/10875/57162/3958/3123/972	6
BP	GO:0007006	mitochondrial membrane organization	6/154	144/18862	0.001187093	0.01123126	0.008229428	3303/7533/841/3002/407021/10105	6
BP	GO:0002700	regulation of production of molecular mediator of immune response	6/154	146/18862	0.001274351	0.011760318	0.008617082	8140/6375/8013/11213/7124/972	6
BP	GO:0045834	positive regulation of lipid metabolic process	6/154	150/18862	0.001463513	0.013073113	0.009579	3458/3949/51099/8013/7124/972	6
BP	GO:0010821	regulation of mitochondrion organization	6/154	151/18862	0.001513982	0.01348073	0.009877671	3303/7533/841/3002/10105/2876	6
BP	GO:0002706	regulation of lymphocyte mediated immunity	6/154	157/18862	0.001845207	0.015489398	0.011349473	3821/6375/117157/3824/7124/3123	6
BP	GO:0030168	platelet activation	6/154	157/18862	0.001845207	0.015489398	0.011349473	2207/7414/3611/83706/928/3043	6
BP	GO:0042129	regulation of T cell proliferation	6/154	168/18862	0.002591792	0.020126729	0.014747363	6375/3113/57162/3958/3115/3123	6

BP	GO:0051897	positive regulation of protein kinase B signaling	6/154	175/18862	0.003172637	0.023146964	0.016960365	374/1524/6348/407021/7124/2876	6
BP	GO:0090316	positive regulation of intracellular protein transport	6/154	179/18862	0.003545369	0.025142351	0.018422435	4946/3458/7533/2040/841/3002	6
BP	GO:0031099	regeneration	6/154	192/18862	0.004983554	0.032076591	0.023503328	2934/928/1026/7049/8013/2876	6
BP	GO:2000377	regulation of reactive oxygen species metabolic process	6/154	192/18862	0.004983554	0.032076591	0.023503328	1524/3458/3383/1026/7124/3043	6
BP	GO:0050731	positive regulation of peptidyl-tyrosine phosphorylation	6/154	193/18862	0.005109595	0.032812076	0.024042236	374/3458/3383/54625/7124/972	6
BP	GO:0071222	cellular response to lipopolysaccharide	6/154	197/18862	0.005637274	0.035471313	0.025990727	1524/7128/6348/3394/3383/7124	6
BP	GO:0050679	positive regulation of epithelial cell proliferation	6/154	203/18862	0.006502075	0.039394091	0.028865045	374/7128/407021/8013/3397/3164	6
BP	GO:0031396	regulation of protein ubiquitination	6/154	205/18862	0.00681068	0.040558471	0.029718216	7128/9636/3303/3304/57162/1407	6
BP	GO:0043281	regulation of cysteine-type endopeptidase activity involved in apoptotic process	6/154	205/18862	0.00681068	0.040558471	0.029718216	2537/2934/841/1612/7124/2876	6
BP	GO:0071219	cellular response to molecule of bacterial origin	6/154	209/18862	0.007459585	0.042584205	0.031202522	1524/7128/6348/3394/3383/7124	6
BP	GO:0007623	circadian rhythm	6/154	212/18862	0.007974773	0.044629907	0.03270146	3725/5730/1958/22809/1407/3397	6
BP	GO:0032388	positive regulation of intracellular transport	6/154	219/18862	0.009275891	0.048997837	0.035901952	4946/3458/7533/2040/841/3002	6

BP	GO:0051495	positive regulation of cytoskeleton organization	6/154	220/18862	0.009473435	0.049722152	0.036432676	2934/3303/28984/3304/3383/3397	6
BP	GO:0019835	cytolysis	5/154	31/18862	4.87E-06	0.000196213	0.00014377	23531/3002/3001/5551/2999	5
BP	GO:0032673	regulation of interleukin-4 production	5/154	32/18862	5.73E-06	0.000218703	0.000160249	2207/8140/3662/9308/3123	5
BP	GO:0043372	positive regulation of CD4-positive, alpha-beta T cell differentiation	5/154	32/18862	5.73E-06	0.000218703	0.000160249	64332/3458/9308/84807/3123	5
BP	GO:0032633	interleukin-4 production	5/154	33/18862	6.71E-06	0.000245995	0.000180247	2207/8140/3662/9308/3123	5
BP	GO:0046596	regulation of viral entry into host cell	5/154	41/18862	2.01E-05	0.000565729	0.000414524	2934/10410/3956/3123/972	5
BP	GO:0150076	neuroinflammatory response	5/154	44/18862	2.86E-05	0.000723455	0.000530094	3458/6348/3949/7124/8530	5
BP	GO:0001774	microglial cell activation	5/154	47/18862	3.95E-05	0.000933989	0.000684357	3458/6348/3949/7124/8530	5
BP	GO:0001913	T cell mediated cytotoxicity	5/154	48/18862	4.39E-05	0.00101009	0.000740118	3821/6375/3824/5551/3123	5
BP	GO:0052372	modulation by symbiont of entry into host	5/154	48/18862	4.39E-05	0.00101009	0.000740118	2934/10410/3956/3123/972	5
BP	GO:0046638	positive regulation of alpha-beta T cell differentiation	5/154	49/18862	4.85E-05	0.001099343	0.000805516	64332/3458/9308/84807/3123	5
BP	GO:0045071	negative regulation of viral genome replication	5/154	54/18862	7.79E-05	0.001550144	0.00113583	4599/8638/9636/10410/7124	5
BP	GO:0032731	positive regulation of interleukin-1 beta production	5/154	56/18862	9.28E-05	0.001690924	0.001238982	3458/6348/1958/841/7124	5

BP	GO:0045428	regulation of nitric oxide biosynthetic process	5/154	59/18862	0.000119295	0.002027282	0.001485441	1524/3458/3383/7124/3043	5
BP	GO:0032722	positive regulation of chemokine production	5/154	60/18862	0.00012929	0.002107196	0.001543996	3458/1958/255231/7124/972	5
BP	GO:1902110	positive regulation of mitochondrial membrane permeability involved in apoptotic process	5/154	60/18862	0.00012929	0.002107196	0.001543996	3303/7533/841/3002/407021	5
BP	GO:0080164	regulation of nitric oxide metabolic process	5/154	61/18862	0.000139914	0.002253988	0.001651554	1524/3458/3383/7124/3043	5
BP	GO:0032613	interleukin-10 production	5/154	62/18862	0.000151193	0.002354057	0.001724877	9636/3662/6375/9308/3123	5
BP	GO:0051851	modulation by host of symbiont process	5/154	62/18862	0.000151193	0.002354057	0.001724877	3725/6348/2040/6351/3429	5
BP	GO:0070527	platelet aggregation	5/154	62/18862	0.000151193	0.002354057	0.001724877	7414/3611/83706/928/3043	5
BP	GO:0031343	positive regulation of cell killing	5/154	63/18862	0.000163155	0.002491339	0.001825467	3458/6375/3824/5551/3123	5
BP	GO:0032732	positive regulation of interleukin-1 production	5/154	64/18862	0.000175827	0.00263457	0.001930416	3458/6348/1958/841/7124	5
BP	GO:0032729	positive regulation of interferon-gamma production	5/154	65/18862	0.000189236	0.002754914	0.002018595	8140/3394/3113/3115/7124	5
BP	GO:0045824	negative regulation of innate immune response	5/154	65/18862	0.000189236	0.002754914	0.002018595	7128/3821/54625/3824/11213	5
BP	GO:0002294	CD4-positive, alpha-beta T cell differentiation involved in immune response	5/154	66/18862	0.000203412	0.00289239	0.002119327	64332/3662/3458/84807/3123	5

BP	GO:1902108	regulation of mitochondrial membrane permeability involved in apoptotic process	5/154	66/18862	0.000203412	0.00289239	0.002119327	3303/7533/841/3002/407021	5
BP	GO:0002287	alpha-beta T cell activation involved in immune response	5/154	67/18862	0.000218382	0.003013023	0.002207718	64332/3662/3458/84807/3123	5
BP	GO:0002293	alpha-beta T cell differentiation involved in immune response	5/154	67/18862	0.000218382	0.003013023	0.002207718	64332/3662/3458/84807/3123	5
BP	GO:0038034	signal transduction in absence of ligand	5/154	67/18862	0.000218382	0.003013023	0.002207718	2537/3303/3304/7124/597	5
BP	GO:0097192	extrinsic apoptotic signaling pathway in absence of ligand	5/154	67/18862	0.000218382	0.003013023	0.002207718	2537/3303/3304/7124/597	5
BP	GO:0002548	monocyte chemotaxis	5/154	68/18862	0.000234177	0.003152899	0.002310209	6375/6348/6846/6351/3958	5
BP	GO:0006809	nitric oxide biosynthetic process	5/154	73/18862	0.000326574	0.004099825	0.003004045	1524/3458/3383/7124/3043	5
BP	GO:0046209	nitric oxide metabolic process	5/154	77/18862	0.000418492	0.004942111	0.0036212 1	1524/3458/3383/7124/3043	5
BP	GO:2001057	reactive nitrogen species metabolic process	5/154	78/18862	0.000444245	0.005202142	0.003811741	1524/3458/3383/7124/3043	5
BP	GO:0001910	regulation of leukocyte mediated cytotoxicity	5/154	79/18862	0.000471174	0.005403955	0.003959614	3821/6375/3383/3824/3123	5
BP	GO:0031397	negative regulation of protein ubiquitination	5/154	79/18862	0.000471174	0.005403955	0.003959614	7128/9636/3303/3304/1407	5
BP	GO:0045069	regulation of viral genome replication	5/154	83/18862	0.000591352	0.006566129	0.004811168	4599/8638/9636/10410/7124	5

BP	GO:1904705	regulation of vascular associated smooth muscle cell proliferation	5/154	83/18862	0.000591352	0.006566129	0.004811168	3725/3248/1026/8013/7124	5
BP	GO:1990874	vascular associated smooth muscle cell proliferation	5/154	83/18862	0.000591352	0.006566129	0.004811168	3725/3248/1026/8013/7124	5
BP	GO:0070098	chemokine-mediated signaling pathway	5/154	88/18862	0.000772297	0.00821524	0.006019514	1524/6375/6348/6846/6351	5
BP	GO:0032642	regulation of chemokine production	5/154	89/18862	0.000812949	0.008582159	0.006288365	3458/1958/255231/7124/972	5
BP	GO:0002690	positive regulation of leukocyte chemotaxis	5/154	91/18862	0.000899017	0.009279855	0.006799584	6375/6348/6846/6351/972	5
BP	GO:1903321	negative regulation of protein modification by small protein conjugation or removal	5/154	91/18862	0.000899017	0.009279855	0.006799584	7128/9636/3303/3304/1407	5
BP	GO:1904035	regulation of epithelial cell apoptotic process	5/154	94/18862	0.001040603	0.010284261	0.007535538	7128/2934/28984/3383/7124	5
BP	GO:0032602	chemokine production	5/154	95/18862	0.001091283	0.010560439	0.0077379	3458/1958/255231/7124/972	5
BP	GO:1990868	response to chemokine	5/154	97/18862	0.001198106	0.01123126	0.008229428	1524/6375/6348/6846/6351	5
BP	GO:1990869	cellular response to chemokine	5/154	97/18862	0.001198106	0.01123126	0.008229428	1524/6375/6348/6846/6351	5
BP	GO:0002824	positive regulation of adaptive immune response based on somatic recombination of immune receptors built from immunoglobulin superfamily domains	5/154	99/18862	0.001312476	0.011993018	0.008787587	64332/6375/84807/7124/3123	5

BP	GO:1903426	regulation of reactive oxygen species biosynthetic process	5/154	99/18862	0.001312476	0.011993018	0.008787587	1524/3458/3383/7124/3043	5
BP	GO:2000379	positive regulation of reactive oxygen species metabolic process	5/154	101/18862	0.001434715	0.012898552	0.009451095	3458/3383/1026/7124/3043	5
BP	GO:0002821	positive regulation of adaptive immune response	5/154	104/18862	0.001633537	0.014227082	0.010424542	64332/6375/84807/7124/3123	5
BP	GO:0002708	positive regulation of lymphocyte mediated immunity	5/154	108/18862	0.001929214	0.015954657	0.0116903<br>8	6375/117157/3824/7124/3123	5
BP	GO:0043618	regulation of transcription from RNA polymerase II promoter in response to stress	5/154	109/18862	0.0020088<br>7	0.016515397	0.012101248	3725/3303/1958/467/571	5
BP	GO:0043620	regulation of DNA-templated transcription in response to stress	5/154	115/18862	0.002538191	0.019870616	0.014559702	3725/3303/1958/467/571	5
BP	GO:1904019	epithelial cell apoptotic process	5/154	116/18862	0.002635367	0.020402133	0.014949158	7128/2934/28984/3383/7124	5
BP	GO:0051101	regulation of DNA binding	5/154	118/18862	0.002837772	0.021299416	0.0156066<br>2	3725/3662/3458/3001/3397	5
BP	GO:0072676	lymphocyte migration	5/154	118/18862	0.002837772	0.021299416	0.0156066<br>2	6375/6348/6846/6351/3383	5
BP	GO:0002688	regulation of leukocyte chemotaxis	5/154	119/18862	0.002943086	0.021756976	0.015941886	6375/6348/6846/6351/972	5
BP	GO:0045446	endothelial cell differentiation	5/154	119/18862	0.002943086	0.021756976	0.015941886	7414/3383/7124/3397/2876	5
BP	GO:1903409	reactive oxygen species biosynthetic process	5/154	123/18862	0.003392728	0.024559828	0.017995606	1524/3458/3383/7124/3043	5

BP	GO:0002705	positive regulation of leukocyte mediated immunity	5/154	125/18862	0.003635171	0.025636786	0.01878472	6375/117157/3824/7124/3123	5
BP	GO:0050729	positive regulation of inflammatory response	5/154	133/18862	0.004730236	0.031388493	0.022999141	64332/3458/6348/7124/283131	5
BP	GO:0003158	endothelium development	5/154	137/18862	0.005357599	0.033935522	0.024865414	7414/3383/7124/3397/2876	5
BP	GO:0050921	positive regulation of chemotaxis	5/154	139/18862	0.005692391	0.035650999	0.026122387	6375/6348/6846/6351/972	5
BP	GO:0010212	response to ionizing radiation	5/154	142/18862	0.006221879	0.038448732	0.028172357	1958/3383/1026/64859/2876	5
BP	GO:0071901	negative regulation of protein serine/threonine kinase activity	5/154	145/18862	0.006785091	0.040558471	0.029718216	7128/1846/1026/11213/1844	5
BP	GO:0034620	cellular response to unfolded protein	5/154	149/18862	0.007590342	0.043172009	0.031633221	64061/3303/4000/467/3304	5
BP	GO:0002224	toll-like receptor signaling pathway	5/154	157/18862	0.009396294	0.049410326	0.036204193	7128/3662/841/57162/11213	5
BP	GO:0140131	positive regulation of lymphocyte chemotaxis	4/154	20/18862	1.87E-05	0.000537274	0.000393674	6375/6348/6846/6351	4
BP	GO:0071404	cellular response to low-density lipoprotein particle stimulus	4/154	22/18862	2.79E-05	0.00071275	0.00052225	2207/3949/928/19	4
BP	GO:1901623	regulation of lymphocyte chemotaxis	4/154	26/18862	5.55E-05	0.001209317	0.000886097	6375/6348/6846/6351	4
BP	GO:0042744	hydrogen peroxide catabolic process	4/154	31/18862	0.000113244	0.001960311	0.001436369	3039/3040/3043/2876	4
BP	GO:1901099	negative regulation of signal transduction in absence of ligand	4/154	32/18862	0.000128603	0.002107196	0.001543996	2537/3303/3304/7124	4

BP	GO:2001240	negative regulation of extrinsic apoptotic signaling pathway in absence of ligand	4/154	32/18862	0.000128603	0.002107196	0.001543996	2537/3303/3304/7124	4
BP	GO:0055094	response to lipoprotein particle	4/154	33/18862	0.000145416	0.002302693	0.001687242	2207/3949/928/19	4
BP	GO:0071402	cellular response to lipoprotein particle stimulus	4/154	34/18862	0.000163763	0.002491339	0.001825467	2207/3949/928/19	4
BP	GO:2000403	positive regulation of lymphocyte migration	4/154	37/18862	0.000228825	0.003095808	0.002268377	6375/6348/6846/6351	4
BP	GO:0001914	regulation of T cell mediated cytotoxicity	4/154	38/18862	0.000254131	0.003372681	0.002471249	3821/6375/3824/3123	4
BP	GO:0045429	positive regulation of nitric oxide biosynthetic process	4/154	38/18862	0.000254131	0.003372681	0.002471249	3458/3383/7124/3043	4
BP	GO:0071276	cellular response to cadmium ion	4/154	38/18862	0.000254131	0.003372681	0.002471249	3725/2934/4502/4493	4
BP	GO:0045622	regulation of T-helper cell differentiation	4/154	39/18862	0.000281387	0.003630669	0.002660283	64332/3662/84807/3123	4
BP	GO:1904407	positive regulation of nitric oxide metabolic process	4/154	39/18862	0.000281387	0.003630669	0.002660283	3458/3383/7124/3043	4
BP	GO:0030890	positive regulation of B cell proliferation	4/154	40/18862	0.000310678	0.003917921	0.00287076	952/57162/1026/972	4
BP	GO:0070423	nucleotide-binding oligomerization domain containing signaling pathway	4/154	40/18862	0.000310678	0.003917921	0.00287076	7128/3303/841/3304	4
BP	GO:0150077	regulation of neuroinflammatory response	4/154	40/18862	0.000310678	0.003917921	0.00287076	6348/3949/7124/8530	4

BP	GO:0035872	nucleotide-binding domain, leucine rich repeat containing receptor signaling pathway	4/154	41/18862	0.000342093	0.004145271	0.003037344	7128/3303/841/3304	4
BP	GO:1902042	negative regulation of extrinsic apoptotic signaling pathway via death domain receptors	4/154	41/18862	0.000342093	0.004145271	0.003037344	7128/841/3383/2876	4
BP	GO:2001239	regulation of extrinsic apoptotic signaling pathway in absence of ligand	4/154	42/18862	0.000375717	0.00449409	0.003292934	2537/3303/3304/7124	4
BP	GO:0070266	necroptotic process	4/154	43/18862	0.000411639	0.004881864	0.003577065	841/57162/10105/7124	4
BP	GO:1901028	regulation of mitochondrial outer membrane permeabilization involved in apoptotic signaling pathway	4/154	44/18862	0.000449948	0.005246878	0.00384452	3303/7533/841/3002	4
BP	GO:1904707	positive regulation of vascular associated smooth muscle cell proliferation	4/154	47/18862	0.000580083	0.006518921	0.004776578	3725/3248/8013/7124	4
BP	GO:0001961	positive regulation of cytokine-mediated signaling pathway	4/154	49/18862	0.000680415	0.007380714	0.005408035	3303/3304/54625/972	4
BP	GO:0097300	programmed necrotic cell death	4/154	51/18862	0.000792451	0.008397569	0.006153111	841/57162/10105/7124	4
BP	GO:0002720	positive regulation of cytokine production involved in immune response	4/154	53/18862	0.000916919	0.009360634	0.006858773	8140/6375/8013/972	4
BP	GO:1905517	macrophage migration	4/154	53/18862	0.000916919	0.009360634	0.006858773	1524/6348/255231/3958	4

BP	GO:0006968	cellular defense response	4/154	54/18862	0.000984043	0.009794741	0.007176854	1524/10219/10578/5551		4
BP	GO:0001836	release of cytochrome c from mitochondria	4/154	55/18862	0.001054549	0.010312375	0.007556137	2537/3725/10105/2876		4
BP	GO:0097345	mitochondrial outer membrane permeabilization	4/154	55/18862	0.001054549	0.010312375	0.007556137	3303/7533/841/3002		4
BP	GO:1903428	positive regulation of reactive oxygen species biosynthetic process	4/154	55/18862	0.001054549	0.010312375	0.007556137	3458/3383/7124/3043		4
BP	GO:0042743	hydrogen peroxide metabolic process	4/154	56/18862	0.001128527	0.010808265	0.007919488	3039/3040/3043/2876		4
BP	GO:0060760	positive regulation of response to cytokine stimulus	4/154	56/18862	0.001128527	0.010808265	0.007919488	3303/3304/54625/972		4
BP	GO:0032757	positive regulation of interleukin-8 production	4/154	57/18862	0.00120607	0.01123126	0.008229428	3303/3304/7124/972		4
BP	GO:2000351	regulation of endothelial cell apoptotic process	4/154	58/18862	0.001287267	0.011840309	0.008675693	7128/28984/3383/7124		4
BP	GO:0032663	regulation of interleukin-2 production	4/154	59/18862	0.001372211	0.012416724	0.009098047	7128/3662/6375/9308		4
BP	GO:0043030	regulation of macrophage activation	4/154	59/18862	0.001372211	0.012416724	0.009098047	6348/3949/972/8530		4
BP	GO:0032653	regulation of interleukin-10 production	4/154	60/18862	0.001460991	0.013073113	0.009579	3662/6375/9308/3123		4
BP	GO:0030888	regulation of B cell proliferation	4/154	61/18862	0.001553698	0.013659797	0.010008878	952/57162/1026/972		4
BP	GO:0032623	interleukin-2 production	4/154	61/18862	0.001553698	0.013659797	0.010008878	7128/3662/6375/9308		4

BP	GO:0046686	response to cadmium ion	4/154	61/18862	0.001553698	0.013659797	0.010008878	3725/2934/4502/4493	4
BP	GO:0002437	inflammatory response to antigenic stimulus	4/154	62/18862	0.001650422	0.014311445	0.010486357	3383/7124/2876/3123	4
BP	GO:2000401	regulation of lymphocyte migration	4/154	63/18862	0.001751254	0.014971609	0.010970076	6375/6348/6846/6351	4
BP	GO:0042093	T-helper cell differentiation	4/154	64/18862	0.001856282	0.015489398	0.011349473	64332/3662/84807/3123	4
BP	GO:0048247	lymphocyte chemotaxis	4/154	64/18862	0.001856282	0.015489398	0.011349473	6375/6348/6846/6351	4
BP	GO:0072577	endothelial cell apoptotic process	4/154	64/18862	0.001856282	0.015489398	0.011349473	7128/28984/3383/7124	4
BP	GO:0070265	necrotic cell death	4/154	65/18862	0.001965597	0.016207451	0.011875608	841/57162/10105/7124	4
BP	GO:0001885	endothelial cell development	4/154	66/18862	0.002079286	0.016944358	0.012415559	7414/3383/7124/2876	4
BP	GO:0072678	T cell migration	4/154	66/18862	0.002079286	0.016944358	0.012415559	6375/6348/6846/3383	4
BP	GO:2000272	negative regulation of signaling receptor activity	4/154	66/18862	0.002079286	0.016944358	0.012415559	100462981/100463486/7124/100463498	4
BP	GO:0002228	natural killer cell mediated immunity	4/154	67/18862	0.002197439	0.0176491	0.012931992	3821/117157/3002/3824	4
BP	GO:0031640	killing of cells of other organism	4/154	67/18862	0.002197439	0.0176491	0.012931992	3458/10578/841/5551	4
BP	GO:0050766	positive regulation of phagocytosis	4/154	67/18862	0.002197439	0.0176491	0.012931992	3560/2207/3458/7124	4

BP	GO:0050848	regulation of calcium-mediated signaling	4/154	67/18862	0.002197439	0.0176491 7	0.012931992	84174/6348/6351/7124	4
BP	GO:1905953	negative regulation of lipid localization	4/154	67/18862	0.002197439	0.0176491 7	0.012931992	51099/19/1407/7124	4
BP	GO:0002753	cytoplasmic pattern recognition receptor signaling pathway	4/154	72/18862	0.002858212	0.021299416	0.0156066 2	7128/3303/841/3304	4
BP	GO:0042246	tissue regeneration	4/154	73/18862	0.003004974	0.022155722	0.016234057	2934/928/1026/2876	4
BP	GO:0034121	regulation of toll-like receptor signaling pathway	4/154	77/18862	0.003643537	0.025636786	0.0187847 2	7128/3662/57162/11213	4
BP	GO:0002709	regulation of T cell mediated immunity	4/154	78/18862	0.003816472	0.0266579 1	0.019532924	3821/6375/3824/3123	4
BP	GO:0048145	regulation of fibroblast proliferation	4/154	79/18862	0.003994887	0.027627174	0.020243128	2495/1026/972/6277	4
BP	GO:0048144	fibroblast proliferation	4/154	80/18862	0.004178865	0.028336975	0.020763217	2495/1026/972/6277	4
BP	GO:2000117	negative regulation of cysteine-type endopeptidase activity	4/154	80/18862	0.004178865	0.028336975	0.020763217	2537/7124/2876/8530	4
BP	GO:0010822	positive regulation of mitochondrion organization	4/154	84/18862	0.004971975	0.032076591	0.023503328	7533/841/3002/10105	4
BP	GO:0019915	lipid storage	4/154	85/18862	0.0051849 4	0.032998557	0.024178876	51099/19/1407/7124	4
BP	GO:0046889	positive regulation of lipid biosynthetic process	4/154	85/18862	0.0051849 4	0.032998557	0.024178876	3458/3949/7124/972	4
BP	GO:0070664	negative regulation of leukocyte proliferation	4/154	88/18862	0.005860304	0.036495232	0.026740978	7128/6375/57162/3123	4

BP	GO:0050764	regulation of phagocytosis	4/154	94/18862	0.007381672	0.042243779	0.030953083	3560/2207/3458/7124	4
BP	GO:0097194	execution phase of apoptosis	4/154	94/18862	0.007381672	0.042243779	0.030953083	100462981/100463486/841/100463498	4
BP	GO:2001243	negative regulation of intrinsic apoptotic signaling pathway	4/154	95/18862	0.007658173	0.043469099	0.031850906	3303/10105/2876/972	4
BP	GO:0042100	B cell proliferation	4/154	96/18862	0.007941408	0.044532602	0.032630161	952/57162/1026/972	4
BP	GO:0048661	positive regulation of smooth muscle cell proliferation	4/154	96/18862	0.007941408	0.044532602	0.032630161	3725/3248/8013/7124	4
BP	GO:0032677	regulation of interleukin-8 production	4/154	97/18862	0.008231442	0.045596709	0.03340986	3303/3304/7124/972	4
BP	GO:0002702	positive regulation of production of molecular mediator of immune response	4/154	99/18862	0.008832166	0.047796598	0.035021774	8140/6375/8013/972	4
BP	GO:0008630	intrinsic apoptotic signaling pathway in response to DNA damage	4/154	100/18862	0.009142982	0.048997837	0.035901952	1026/7124/597/972	4
BP	GO:0002291	T cell activation via T cell receptor contact with antigen bound to MHC molecule on antigen presenting cell	3/154	10/18862	6.14E-05	0.001316575	0.000964688	3383/10875/3958	3
BP	GO:2000343	positive regulation of chemokine (C-X-C motif) ligand 2 production	3/154	10/18862	6.14E-05	0.001316575	0.000964688	255231/7124/972	3
BP	GO:0035747	natural killer cell chemotaxis	3/154	11/18862	8.39E-05	0.001580595	0.001158142	6375/6348/6351	3
BP	GO:0045657	positive regulation of monocyte differentiation	3/154	11/18862	8.39E-05	0.001580595	0.001158142	3725/3123/972	3

BP	GO:0046598	positive regulation of viral entry into host cell	3/154	11/18862	8.39E-05	0.001580595	0.001158142	3956/3123/972	3
BP	GO:0075294	positive regulation by symbiont of entry into host	3/154	11/18862	8.39E-05	0.001580595	0.001158142	3956/3123/972	3
BP	GO:0070424	regulation of nucleotide-binding oligomerization domain containing signaling pathway	3/154	13/18862	0.000143751	0.002289343	0.00167746	7128/3303/3304	3
BP	GO:0043922	negative regulation by host of viral transcription	3/154	14/18862	0.000181862	0.002696002	0.001975428	3725/6348/6351	3
BP	GO:0002468	dendritic cell antigen processing and presentation	3/154	15/18862	0.000225967	0.003072054	0.002250971	10875/3123/972	3
BP	GO:0015671	oxygen transport	3/154	15/18862	0.000225967	0.003072054	0.002250971	3039/3040/3043	3
BP	GO:0051709	regulation of killing of cells of other organism	3/154	16/18862	0.000276451	0.003601409	0.002638843	3458/841/5551	3
BP	GO:0070431	nucleotide-binding oligomerization domain containing 2 signaling pathway	3/154	16/18862	0.000276451	0.003601409	0.002638843	7128/3303/3304	3
BP	GO:0033194	response to hydroperoxide	3/154	17/18862	0.000333684	0.004099866	0.003004075	952/1612/2876	3
BP	GO:0071850	mitotic cell cycle arrest	3/154	17/18862	0.000333684	0.004099866	0.003004075	28984/1026/8556	3
BP	GO:0072567	chemokine (C-X-C motif) ligand 2 production	3/154	17/18862	0.000333684	0.004099866	0.003004075	255231/7124/972	3
BP	GO:2000341	regulation of chemokine (C-X-C motif) ligand 2 production	3/154	17/18862	0.000333684	0.004099866	0.003004075	255231/7124/972	3

BP	GO:0070269	pyroptosis	3/154	18/18862	0.000398029	0.004740622	0.003473573	841/3002/3001	3
BP	GO:0034138	toll-like receptor 3 signaling pathway	3/154	19/18862	0.000469836	0.005403955	0.003959614	7128/841/57162	3
BP	GO:0048245	eosinophil chemotaxis	3/154	19/18862	0.000469836	0.005403955	0.003959614	6348/6351/3958	3
BP	GO:0015669	gas transport	3/154	20/18862	0.000549448	0.006224848	0.004561103	3039/3040/3043	3
BP	GO:1903978	regulation of microglial cell activation	3/154	20/18862	0.000549448	0.006224848	0.004561103	6348/3949/8530	3
BP	GO:0035455	response to interferon-alpha	3/154	21/18862	0.000637198	0.006991613	0.005122931	3437/3433/10410	3
BP	GO:0045655	regulation of monocyte differentiation	3/154	21/18862	0.000637198	0.006991613	0.005122931	3725/3123/972	3
BP	GO:1900118	negative regulation of execution phase of apoptosis	3/154	21/18862	0.000637198	0.006991613	0.005122931	100462981/100463486/100463498	3
BP	GO:0002710	negative regulation of T cell mediated immunity	3/154	22/18862	0.000733406	0.007831431	0.005738287	3821/6375/3824	3
BP	GO:0010888	negative regulation of lipid storage	3/154	22/18862	0.000733406	0.007831431	0.005738287	51099/19/7124	3
BP	GO:0032069	regulation of nuclease activity	3/154	22/18862	0.000733406	0.007831431	0.005738287	8638/3303/3001	3
BP	GO:0072677	eosinophil migration	3/154	23/18862	0.000838388	0.0087757	0.006430177	6348/6351/3958	3
BP	GO:0032753	positive regulation of interleukin-4 production	3/154	24/18862	0.000952446	0.009549638	0.006997261	2207/8140/3662	3

BP	GO:0048143	astrocyte activation	3/154	25/18862	0.001075875	0.010458766	0.007663402	3458/3949/7124	3
BP	GO:0002719	negative regulation of cytokine production involved in immune response	3/154	26/18862	0.001208962	0.01123126	0.008229428	6375/11213/7124	3
BP	GO:0061082	myeloid leukocyte cytokine production	3/154	26/18862	0.001208962	0.01123126	0.008229428	8013/11213/972	3
BP	GO:1900739	regulation of protein insertion into mitochondrial membrane involved in apoptotic signaling pathway	3/154	26/18862	0.001208962	0.01123126	0.008229428	7533/841/3002	3
BP	GO:1900740	positive regulation of protein insertion into mitochondrial membrane involved in apoptotic signaling pathway	3/154	26/18862	0.001208962	0.01123126	0.008229428	7533/841/3002	3
BP	GO:0010818	T cell chemotaxis	3/154	27/18862	0.001351984	0.01231366	0.009022529	6375/6348/6846	3
BP	GO:0060055	angiogenesis involved in wound healing	3/154	29/18862	0.001668898	0.014311445	0.010486357	7128/7124/2876	3
BP	GO:0072539	T-helper 17 cell differentiation	3/154	29/18862	0.001668898	0.014311445	0.010486357	64332/3662/84807	3
BP	GO:1900117	regulation of execution phase of apoptosis	3/154	29/18862	0.001668898	0.014311445	0.010486357	100462981/100463486/100463498	3
BP	GO:0001844	protein insertion into mitochondrial membrane involved in apoptotic signaling pathway	3/154	30/18862	0.001843303	0.015489398	0.011349473	7533/841/3002	3

BP	GO:0046685	response to arsenic-containing substance	3/154	30/18862	0.001843303	0.015489398	0.011349473	467/10105/1026	3
BP	GO:0050850	positive regulation of calcium-mediated signaling	3/154	30/18862	0.001843303	0.015489398	0.011349473	6348/6351/7124	3
BP	GO:0002861	regulation of inflammatory response to antigenic stimulus	3/154	32/18862	0.002225224	0.017719144	0.012983264	7124/2876/3123	3
BP	GO:0045648	positive regulation of erythrocyte differentiation	3/154	32/18862	0.002225224	0.017719144	0.012983264	9636/3303/3304	3
BP	GO:1903902	positive regulation of viral life cycle	3/154	32/18862	0.002225224	0.017719144	0.012983264	3956/3123/972	3
BP	GO:0072538	T-helper 17 type immune response	3/154	33/18862	0.002433205	0.019210603	0.014076094	64332/3662/84807	3
BP	GO:0051085	chaperone cofactor-dependent protein refolding	3/154	34/18862	0.002652829	0.020480425	0.015006525	3303/3304/972	3
BP	GO:1901030	positive regulation of mitochondrial outer membrane permeabilization involved in apoptotic signaling pathway	3/154	35/18862	0.002884307	0.021436171	0.015706824	7533/841/3002	3
BP	GO:0030224	monocyte differentiation	3/154	36/18862	0.003127846	0.022880067	0.016764803	3725/3123/972	3
BP	GO:0032733	positive regulation of interleukin-10 production	3/154	38/18862	0.003651885	0.025636786	0.01878472	3662/6375/9308	3
BP	GO:0097242	amyloid-beta clearance	3/154	38/18862	0.003651885	0.025636786	0.01878472	3458/3949/7124	3

BP	GO:0002701	negative regulation of production of molecular mediator of immune response	3/154	39/18862	0.00393276	0.02726518	0.019977886	6375/11213/7124	3
BP	GO:0051084	'de novo' posttranslational protein folding	3/154	39/18862	0.00393276	0.02726518	0.019977886	3303/3304/972	3
BP	GO:1902895	positive regulation of pri-miRNA transcription by RNA polymerase II	3/154	40/18862	0.004226442	0.028527863	0.020903085	3725/1958/7124	3
BP	GO:0010939	regulation of necrotic cell death	3/154	41/18862	0.004533102	0.030224292	0.022146102	841/57162/10105	3
BP	GO:0014002	astrocyte development	3/154	42/18862	0.0048529	0.031562268	0.023126471	3458/3949/7124	3
BP	GO:0046688	response to copper ion	3/154	42/18862	0.0048529	0.031562268	0.023126471	4502/3383/4493	3
BP	GO:0062208	positive regulation of pattern recognition receptor signaling pathway	3/154	42/18862	0.0048529	0.031562268	0.023126471	3303/3304/57162	3
BP	GO:0006458	'de novo' protein folding	3/154	43/18862	0.005185995	0.032998557	0.024178876	3303/3304/972	3
BP	GO:0015701	bicarbonate transport	3/154	43/18862	0.005185995	0.032998557	0.024178876	3039/3040/3043	3
BP	GO:0002707	negative regulation of lymphocyte mediated immunity	3/154	45/18862	0.005892664	0.036495232	0.026740978	3821/6375/3824	3
BP	GO:0014911	positive regulation of smooth muscle cell migration	3/154	45/18862	0.005892664	0.036495232	0.026740978	6282/80005/8013	3
BP	GO:0034122	negative regulation of toll-like receptor signaling pathway	3/154	45/18862	0.005892664	0.036495232	0.026740978	7128/3662/11213	3

BP	GO:0045601	regulation of endothelial cell differentiation	3/154	45/18862	0.005892664	0.036495232	0.026740978	7414/7124/3397	3
BP	GO:0010823	negative regulation of mitochondrion organization	3/154	46/18862	0.006266519	0.038468694	0.028186984	3303/10105/2876	3
BP	GO:0034198	cellular response to amino acid starvation	3/154	46/18862	0.006266519	0.038468694	0.028186984	467/143686/1026	3
BP	GO:0048146	positive regulation of fibroblast proliferation	3/154	46/18862	0.006266519	0.038468694	0.028186984	1026/972/6277	3
BP	GO:0002823	negative regulation of adaptive immune response based on somatic recombination of immune receptors built from immunoglobulin superfamily domains	3/154	47/18862	0.00665423	0.040054728	0.029349111	3821/6375/3824	3
BP	GO:0030225	macrophage differentiation	3/154	47/18862	0.00665423	0.040054728	0.029349111	3458/841/3123	3
BP	GO:0045646	regulation of erythrocyte differentiation	3/154	47/18862	0.00665423	0.040054728	0.029349111	9636/3303/3304	3
BP	GO:0002715	regulation of natural killer cell mediated immunity	3/154	48/18862	0.007055923	0.041487043	0.030398604	3821/117157/3824	3
BP	GO:0006692	prostanoid metabolic process	3/154	48/18862	0.007055923	0.041487043	0.030398604	5730/3248/972	3
BP	GO:0006693	prostaglandin metabolic process	3/154	48/18862	0.007055923	0.041487043	0.030398604	5730/3248/972	3
BP	GO:0051204	protein insertion into mitochondrial membrane	3/154	48/18862	0.007055923	0.041487043	0.030398604	7533/841/3002	3

BP	GO:0061028	establishment of endothelial barrier	3/154	48/18862	0.007055923	0.041487043	0.030398604	7414/3383/7124	3
BP	GO:1902003	regulation of amyloid-beta formation	3/154	48/18862	0.007055923	0.041487043	0.030398604	3458/407021/7124	3
BP	GO:1990928	response to amino acid starvation	3/154	49/18862	0.007471717	0.042584205	0.031202522	467/143686/1026	3
BP	GO:0002931	response to ischemia	3/154	50/18862	0.007901725	0.044489108	0.032598293	1524/1958/10105	3
BP	GO:0008347	glial cell migration	3/154	50/18862	0.007901725	0.044489108	0.032598293	1524/6348/928	3
BP	GO:0031113	regulation of microtubule polymerization	3/154	50/18862	0.007901725	0.044489108	0.032598293	3303/3304/3925	3
BP	GO:1902893	regulation of pri-miRNA transcription by RNA polymerase II	3/154	51/18862	0.008346055	0.045698341	0.033484328	3725/1958/7124	3
BP	GO:0002820	negative regulation of adaptive immune response	3/154	52/18862	0.008804808	0.047741246	0.034981217	3821/6375/3824	3
BP	GO:0043392	negative regulation of DNA binding	3/154	52/18862	0.008804808	0.047741246	0.034981217	3725/3001/3397	3
BP	GO:0061614	pri-miRNA transcription by RNA polymerase II	3/154	52/18862	0.008804808	0.047741246	0.034981217	3725/1958/7124	3
BP	GO:0002704	negative regulation of leukocyte mediated immunity	3/154	53/18862	0.009278082	0.048997837	0.035901952	3821/6375/3824	3
BP	GO:0010332	response to gamma radiation	3/154	53/18862	0.009278082	0.048997837	0.035901952	1958/1026/2876	3
BP	GO:0090151	establishment of protein localization to mitochondrial membrane	3/154	53/18862	0.009278082	0.048997837	0.035901952	7533/841/3002	3

BP	GO:0030656	regulation of vitamin metabolic process	2/154	10/18862	0.002854935	0.021299416	0.01560662	3458/7124	2
BP	GO:0032823	regulation of natural killer cell differentiation	2/154	10/18862	0.002854935	0.021299416	0.01560662	639/257101	2
BP	GO:0042368	vitamin D biosynthetic process	2/154	10/18862	0.002854935	0.021299416	0.01560662	3458/7124	2
BP	GO:0051712	positive regulation of killing of cells of other organism	2/154	10/18862	0.002854935	0.021299416	0.01560662	3458/5551	2
BP	GO:2000318	positive regulation of T-helper 17 type immune response	2/154	10/18862	0.002854935	0.021299416	0.01560662	64332/84807	2
BP	GO:0002604	regulation of dendritic cell antigen processing and presentation	2/154	11/18862	0.003470713	0.024675709	0.018080515	10875/972	2
BP	GO:0035457	cellular response to interferon-alpha	2/154	11/18862	0.003470713	0.024675709	0.018080515	3437/3433	2
BP	GO:0090084	negative regulation of inclusion body assembly	2/154	11/18862	0.003470713	0.024675709	0.018080515	3303/3304	2
BP	GO:1900222	negative regulation of amyloid-beta clearance	2/154	11/18862	0.003470713	0.024675709	0.018080515	3458/7124	2
BP	GO:1903265	positive regulation of tumor necrosis factor-mediated signaling pathway	2/154	11/18862	0.003470713	0.024675709	0.018080515	3303/3304	2
BP	GO:2001269	positive regulation of cysteine-type endopeptidase activity involved in apoptotic signaling pathway	2/154	11/18862	0.003470713	0.024675709	0.018080515	2934/841	2
BP	GO:0036462	TRAIL-activated apoptotic signaling pathway	2/154	12/18862	0.004142607	0.028228474	0.020683716	841/467	2

BP	GO:0043380	regulation of memory T cell differentiation	2/154	12/18862	0.004142607	0.028228474	0.020683716	10875/3123	2
BP	GO:0061469	regulation of type B pancreatic cell proliferation	2/154	12/18862	0.004142607	0.028228474	0.020683716	8013/3164	2
BP	GO:0070493	thrombin-activated receptor signaling pathway	2/154	12/18862	0.004142607	0.028228474	0.020683716	3248/3925	2
BP	GO:0071801	regulation of podosome assembly	2/154	12/18862	0.004142607	0.028228474	0.020683716	2934/7124	2
BP	GO:0097201	negative regulation of transcription from RNA polymerase II promoter in response to stress	2/154	12/18862	0.004142607	0.028228474	0.020683716	3725/3303	2
BP	GO:0001771	immunological synapse formation	2/154	13/18862	0.004869672	0.031562268	0.023126471	3958/5551	2
BP	GO:0042362	fat-soluble vitamin biosynthetic process	2/154	13/18862	0.004869672	0.031562268	0.023126471	3458/7124	2
BP	GO:0042921	glucocorticoid receptor signaling pathway	2/154	13/18862	0.004869672	0.031562268	0.023126471	7533/1407	2
BP	GO:0043379	memory T cell differentiation	2/154	13/18862	0.004869672	0.031562268	0.023126471	10875/3123	2
BP	GO:0061043	regulation of vascular wound healing	2/154	13/18862	0.004869672	0.031562268	0.023126471	7128/7124	2
BP	GO:1902947	regulation of tau-protein kinase activity	2/154	13/18862	0.004869672	0.031562268	0.023126471	3458/1958	2
BP	GO:1903977	positive regulation of glial cell migration	2/154	13/18862	0.004869672	0.031562268	0.023126471	1524/6348	2

BP	GO:0010935	regulation of macrophage cytokine production	2/154	14/18862	0.005650974	0.035471313	0.025990727	11213/972	2
BP	GO:0031958	corticosteroid receptor signaling pathway	2/154	14/18862	0.005650974	0.035471313	0.025990727	7533/1407	2
BP	GO:0090715	immunological memory formation process	2/154	14/18862	0.005650974	0.035471313	0.025990727	10875/3123	2
BP	GO:0010273	detoxification of copper ion	2/154	15/18862	0.00648559	0.039379823	0.028854591	4502/4493	2
BP	GO:0010820	positive regulation of T cell chemotaxis	2/154	15/18862	0.00648559	0.039379823	0.028854591	6375/6846	2
BP	GO:0010934	macrophage cytokine production	2/154	15/18862	0.00648559	0.039379823	0.028854591	11213/972	2
BP	GO:0051044	positive regulation of membrane protein ectodomain proteolysis	2/154	15/18862	0.00648559	0.039379823	0.028854591	3458/7124	2
BP	GO:1990169	stress response to copper ion	2/154	15/18862	0.00648559	0.039379823	0.028854591	4502/4493	2
BP	GO:0002830	positive regulation of type 2 immune response	2/154	16/18862	0.00737261	0.042243779	0.030953083	6375/972	2
BP	GO:0007096	regulation of exit from mitosis	2/154	16/18862	0.00737261	0.042243779	0.030953083	28984/8556	2
BP	GO:0010819	regulation of T cell chemotaxis	2/154	16/18862	0.00737261	0.042243779	0.030953083	6375/6846	2
BP	GO:0019372	lipoxygenase pathway	2/154	16/18862	0.00737261	0.042243779	0.030953083	3248/2876	2
BP	GO:0045591	positive regulation of regulatory T cell differentiation	2/154	16/18862	0.00737261	0.042243779	0.030953083	3458/3123	2

BP	GO:0045779	negative regulation of bone resorption	2/154	16/18862	0.00737261	0.042243779	0.030953083	7128/952	2
BP	GO:0061081	positive regulation of myeloid leukocyte cytokine production involved in immune response	2/154	16/18862	0.00737261	0.042243779	0.030953083	8013/972	2
BP	GO:0071800	podosome assembly	2/154	16/18862	0.00737261	0.042243779	0.030953083	2934/7124	2
BP	GO:0090083	regulation of inclusion body assembly	2/154	16/18862	0.00737261	0.042243779	0.030953083	3303/3304	2
BP	GO:0030730	sequestering of triglyceride	2/154	17/18862	0.008311133	0.045596709	0.03340986	51099/7124	2
BP	GO:0061687	detoxification of inorganic compound	2/154	17/18862	0.008311133	0.045596709	0.03340986	4502/4493	2
BP	GO:0090201	negative regulation of release of cytochrome c from mitochondria	2/154	17/18862	0.008311133	0.045596709	0.03340986	10105/2876	2
BP	GO:0090713	immunological memory process	2/154	17/18862	0.008311133	0.045596709	0.03340986	10875/3123	2
BP	GO:0150078	positive regulation of neuroinflammatory response	2/154	17/18862	0.008311133	0.045596709	0.03340986	6348/7124	2
BP	GO:0150079	negative regulation of neuroinflammatory response	2/154	17/18862	0.008311133	0.045596709	0.03340986	3949/8530	2
BP	GO:1901550	regulation of endothelial cell development	2/154	17/18862	0.008311133	0.045596709	0.03340986	7414/7124	2
BP	GO:1903140	regulation of establishment of endothelial barrier	2/154	17/18862	0.008311133	0.045596709	0.03340986	7414/7124	2

BP	GO:2001267	regulation of cysteine-type endopeptidase activity involved in apoptotic signaling pathway	2/154	17/18862	0.008311133	0.045596709	0.03340986	2934/841	2
BP	GO:0001779	natural killer cell differentiation	2/154	18/18862	0.009300271	0.048997837	0.035901952	639/257101	2
BP	GO:0002862	negative regulation of inflammatory response to antigenic stimulus	2/154	18/18862	0.009300271	0.048997837	0.035901952	2876/3123	2
BP	GO:0045953	negative regulation of natural killer cell mediated cytotoxicity	2/154	18/18862	0.009300271	0.048997837	0.035901952	3821/3824	2
BP	GO:0046851	negative regulation of bone remodeling	2/154	18/18862	0.009300271	0.048997837	0.035901952	7128/952	2
BP	GO:0060546	negative regulation of necroptotic process	2/154	18/18862	0.009300271	0.048997837	0.035901952	841/57162	2
BP	GO:0070293	renal absorption	2/154	18/18862	0.009300271	0.048997837	0.035901952	2934/3043	2
BP	GO:0071243	cellular response to arsenic-containing substance	2/154	18/18862	0.009300271	0.048997837	0.035901952	467/10105	2
BP	GO:0097501	stress response to metal ion	2/154	18/18862	0.009300271	0.048997837	0.035901952	4502/4493	2
BP	GO:1901673	regulation of mitotic spindle assembly	2/154	18/18862	0.009300271	0.048997837	0.035901952	3303/3304	2
CC	GO:0030139	endocytic vesicle	15/153	307/19520	2.00E-08	6.05E-07	4.97E-07	3039/2934/3040/10578/3118/3113/3949/3127/3119/928/3115/19/3043/3123/972	15
CC	GO:0009897	external side of plasma membrane	13/153	402/19520	1.75E-05	0.000211599	0.000173955	1524/3560/2207/8140/2214/3949/9308/3383/925/7049/7124/3123/972	13

CC	GO:0005765	lysosomal membrane	12/153	378/19520	4.45E-05	0.000489382	0.00040232	8140/3118/10410/23531/3113/3949/3127/2040/3119/3115/3123/972	12
CC	GO:0098852	lytic vacuole membrane	12/153	378/19520	4.45E-05	0.000489382	0.00040232	8140/3118/10410/23531/3113/3949/3127/2040/3119/3115/3123/972	12
CC	GO:0005774	vacuolar membrane	12/153	431/19520	0.000154027	0.001336743	0.001098932	8140/3118/10410/23531/3113/3949/3127/2040/3119/3115/3123/972	12
CC	GO:0030665	clathrin-coated vesicle membrane	10/153	117/19520	2.83E-08	6.86E-07	5.64E-07	374/3118/3113/3949/3127/3119/928/3115/3123/972	10
CC	GO:0030662	coated vesicle membrane	10/153	182/19520	1.76E-06	3.55E-05	2.92E-05	374/3118/3113/3949/3127/3119/928/3115/3123/972	10
CC	GO:0101002	ficolin-1-rich granule	10/153	185/19520	2.04E-06	3.80E-05	3.12E-05	2495/2207/7414/2934/3303/3304/10875/3958/23406/3043	10
CC	GO:0030136	clathrin-coated vesicle	10/153	192/19520	2.85E-06	4.92E-05	4.05E-05	374/3118/3113/3949/3127/3119/928/3115/3123/972	10
CC	GO:0005770	late endosome	10/153	275/19520	6.40E-05	0.000673753	0.00055389	84174/10410/23531/3949/3127/255231/153020/3123/972/8530	10
CC	GO:0030135	coated vesicle	10/153	295/19520	0.000114409	0.001064888	0.000875441	374/3118/3113/3949/3127/3119/928/3115/3123/972	10
CC	GO:0030055	cell-substrate junction	10/153	423/19520	0.00188477	0.013821647	0.011362728	7414/3611/2934/3303/83706/7171/3304/3383/928/977	10
CC	GO:0030669	clathrin-coated endocytic vesicle membrane	9/153	39/19520	1.53E-11	2.07E-09	1.70E-09	3118/3113/3949/3127/3119/928/3115/3123/972	9
CC	GO:0045334	clathrin-coated endocytic vesicle	9/153	57/19520	5.75E-10	4.09E-08	3.36E-08	3118/3113/3949/3127/3119/928/3115/3123/972	9
CC	GO:0030666	endocytic vesicle membrane	9/153	163/19520	5.65E-06	9.11E-05	7.49E-05	3118/3113/3949/3127/3119/928/3115/3123/972	9

CC	GO:0030133	transport vesicle	9/153	402/19520	0.004503571	0.031138975	0.025599244	374/94120/3118/3113/3127/3119/3115/3123/972	9
CC	GO:0005925	focal adhesion	9/153	416/19520	0.005608186	0.035715291	0.029361418	7414/3611/2934/3303/7171/3304/3383/928/977	9
CC	GO:0012507	ER to Golgi transport vesicle membrane	8/153	62/19520	2.80E-08	6.86E-07	5.64E-07	374/3118/3113/3127/3119/3115/3123/972	8
CC	GO:0030134	COPII-coated ER to Golgi transport vesicle	8/153	94/19520	7.46E-07	1.64E-05	1.35E-05	374/3118/3113/3127/3119/3115/3123/972	8
CC	GO:1904813	ficolin-1-rich granule lumen	8/153	124/19520	6.06E-06	9.16E-05	7.53E-05	2495/7414/2934/3303/3304/10875/23406/3043	8
CC	GO:0030658	transport vesicle membrane	8/153	206/19520	0.00022478	0.001875751	0.001542048	374/3118/3113/3127/3119/3115/3123/972	8
CC	GO:0042613	MHC class II protein complex	7/153	16/19520	1.71E-11	2.07E-09	1.70E-09	3118/3113/3127/3119/3115/3123/972	7
CC	GO:0042611	MHC protein complex	7/153	25/19520	6.76E-10	4.09E-08	3.36E-08	3118/3113/3127/3119/3115/3123/972	7
CC	GO:0071556	integral component of lumenal side of endoplasmic reticulum membrane	7/153	29/19520	2.14E-09	8.63E-08	7.09E-08	3118/3113/3127/3119/3115/3123/972	7
CC	GO:0098553	lumenal side of endoplasmic reticulum membrane	7/153	29/19520	2.14E-09	8.63E-08	7.09E-08	3118/3113/3127/3119/3115/3123/972	7
CC	GO:0098576	lumenal side of membrane	7/153	36/19520	1.09E-08	3.78E-07	3.10E-07	3118/3113/3127/3119/3115/3123/972	7
CC	GO:0032588	trans-Golgi network membrane	7/153	95/19520	9.83E-06	0.000139917	0.000115025	3118/3113/3127/3119/3115/3123/972	7
CC	GO:0072562	blood microparticle	7/153	146/19520	0.000154664	0.001336743	0.001098932	3039/2934/3040/3303/2040/3304/3043	7

CC	GO:0030176	integral component of endoplasmic reticulum membrane	7/153	157/19520	0.000241914	0.001951443	0.001604274	3118/3113/3127/3119/3115/3123/972	7
CC	GO:0031227	intrinsic component of endoplasmic reticulum membrane	7/153	165/19520	0.000327396	0.0025558	0.002101115	3118/3113/3127/3119/3115/3123/972	7
CC	GO:0005802	trans-Golgi network	7/153	251/19520	0.003699317	0.026330434	0.021646159	3118/3113/3127/3119/3115/3123/972	7
CC	GO:0001772	immunological synapse	5/153	41/19520	1.65E-05	0.000210625	0.000173154	3383/3002/3958/3001/3123	5
CC	GO:0031902	late endosome membrane	5/153	140/19520	0.004951719	0.033286555	0.027364762	10410/23531/3127/255231/3123	5
CC	GO:0071682	endocytic vesicle lumen	4/153	20/19520	1.59E-05	0.000210625	0.000173154	3039/3040/10578/3043	4
CC	GO:0031838	haptoglobin-hemoglobin complex	3/153	11/19520	7.44E-05	0.000750183	0.000616722	3039/3040/3043	3
CC	GO:0005833	hemoglobin complex	3/153	12/19520	9.86E-05	0.000954714	0.000784867	3039/3040/3043	3
CC	GO:0002102	podosome	3/153	28/19520	0.001339622	0.010130894	0.008328573	7414/2934/83706	3
CC	GO:0022627	cytosolic small ribosomal subunit	3/153	46/19520	0.00560091	0.035715291	0.029361418	3039/9636/3040	3
MF	GO:0140375	immune receptor activity	11/149	136/18337	1.44E-08	5.28E-06	4.41E-06	1524/3560/3821/2207/3118/51348/3113/3119/3824/3123/972	11
MF	GO:0005126	cytokine receptor binding	9/149	270/18337	0.000369437	0.013538583	0.01130797	3458/6375/6348/7185/6846/6351/841/7049/7124	9
MF	GO:0042277	peptide binding	9/149	315/18337	0.001109159	0.02401004	0.020054153	8140/3113/3949/3127/3119/10105/3115/3123/972	9

MF	GO:0005125	cytokine activity	8/149	235/18337	0.000682861	0.017949492	0.014992139	374/3458/6375/6348/6846/6351/9235/7124	8
MF	GO:0030246	carbohydrate binding	8/149	267/18337	0.001549053	0.031669519	0.026451657	3821/10219/51348/3956/3820/3958/3824/3123	8
MF	GO:0031625	ubiquitin protein ligase binding	8/149	293/18337	0.002758696	0.043359613	0.036215695	7414/3725/9636/3303/7185/841/3304/1026	8
MF	GO:0044389	ubiquitin-like protein ligase binding	8/149	312/18337	0.004035416	0.049484384	0.04133135	7414/3725/9636/3303/7185/841/3304/1026	8
MF	GO:0003823	antigen binding	7/149	165/18337	0.000404686	0.013538583	0.01130797	8140/3113/3127/3119/3115/3824/3123	7
MF	GO:0042605	peptide antigen binding	6/149	32/18337	1.98E-07	3.64E-05	3.04E-05	8140/3113/3127/3119/3115/3123	6
MF	GO:0023023	MHC protein complex binding	5/149	26/18337	1.90E-06	0.000174644	0.00014587	3821/925/3824/3123/972	5
MF	GO:0016684	oxidoreductase activity, acting on peroxide as acceptor	5/149	57/18337	9.87E-05	0.005762928	0.00481343	3039/3040/143686/3043/2876	5
MF	GO:0032395	MHC class II receptor activity	4/149	10/18337	8.47E-07	0.000103839	8.67E-05	3118/3113/3119/3123	4
MF	GO:0048020	CCR chemokine receptor binding	4/149	46/18337	0.000524064	0.016071281	0.013423381	6375/6348/6846/6351	4
MF	GO:0008009	chemokine activity	4/149	49/18337	0.000667724	0.017949492	0.014992139	6375/6348/6846/6351	4
MF	GO:0004601	peroxidase activity	4/149	53/18337	0.000899943	0.021595036	0.018037044	3039/3040/3043/2876	4
MF	GO:0042379	chemokine receptor binding	4/149	69/18337	0.002403505	0.042118557	0.035179115	6375/6348/6846/6351	4

MF	GO:0001618	virus receptor activity	4/149	76/18337	0.00341436	0.046856233	0.039136213	3303/3949/3304/3383	4
MF	GO:0140272	exogenous protein binding	4/149	77/18337	0.003579046	0.047038896	0.03928878	3303/3949/3304/3383	4
MF	GO:0031720	haptoglobin binding	3/149	10/18337	6.05E-05	0.00445368	0.003719893	3039/3040/3043	3
MF	GO:0035259	glucocorticoid receptor binding	3/149	12/18337	0.000109621	0.005762928	0.00481343	7533/8013/3164	3
MF	GO:0005344	oxygen carrier activity	3/149	14/18337	0.00017922	0.007328111	0.006120734	3039/3040/3043	3
MF	GO:0044548	S100 protein binding	3/149	14/18337	0.00017922	0.007328111	0.006120734	6282/6285/6277	3
MF	GO:0019865	immunoglobulin binding	3/149	24/18337	0.000938915	0.021595036	0.018037044	2207/2214/3958	3
MF	GO:0005164	tumor necrosis factor receptor binding	3/149	31/18337	0.002000293	0.038742524	0.032359316	7185/841/7124	3
MF	GO:0048019	receptor antagonist activity	3/149	32/18337	0.002194172	0.040372773	0.033720966	100462981/100463486/100463498	3
MF	GO:0044183	protein folding chaperone	3/149	36/18337	0.00308459	0.045405164	0.037924222	3303/3304/972	3
MF	GO:0019825	oxygen binding	3/149	39/18337	0.003878741	0.049219891	0.041110435	3039/3040/3043	3
MF	GO:0042287	MHC protein binding	3/149	40/18337	0.004168521	0.049484384	0.04133135	925/3824/972	3
MF	GO:0008330	protein tyrosine/threonine phosphatase activity	2/149	10/18337	0.002827801	0.043359613	0.036215695	1846/1844	2

MF	GO:0050786	RAGE receptor binding	2/149	10/18337	0.002827801	0.043359613	0.036215695	6285/6275	2
MF	GO:0019864	IgG binding	2/149	11/18337	0.003437821	0.046856233	0.039136213	2207/2214	2
KEGG	hsa04612	Antigen processing and presentation	14/88	78/8096	6.44E-14	5.96E-12	4.47E-12	3821/3458/3303/3118/3113/3127/3119/3304/925/3115/3824/7124/3123/972	14
KEGG	hsa05169	Epstein-Barr virus infection	14/88	202/8096	2.93E-08	4.92E-07	3.70E-07	7128/3725/9636/3118/3113/3127/841/3119/3383/1026/3115/953/7124/3123	14
KEGG	hsa05145	Toxoplasmosis	13/88	112/8096	1.69E-10	6.26E-09	4.70E-09	3458/3303/3118/3113/3949/3127/841/3119/3304/10105/3115/7124/3123	13
KEGG	hsa05332	Graft-versus-host disease	12/88	42/8096	1.06E-14	1.97E-12	1.48E-12	3821/3458/3118/3113/3127/3119/3002/3115/3824/7124/5551/3123	12
KEGG	hsa05152	Tuberculosis	12/88	180/8096	4.77E-07	5.88E-06	4.42E-06	2207/3458/2214/3118/3113/3127/841/3119/3115/7124/3123/972	12
KEGG	hsa05166	Human T-cell leukemia virus 1 infection	12/88	222/8096	4.38E-06	3.86E-05	2.90E-05	3560/3725/3118/3113/1958/3127/3119/3383/1026/3115/7124/3123	12
KEGG	hsa05323	Rheumatoid arthritis	11/88	93/8096	3.90E-09	1.03E-07	7.73E-08	3725/3458/3118/6348/3113/3127/3119/3383/3115/7124/3123	11
KEGG	hsa05164	Influenza A	11/88	172/8096	2.24E-06	2.18E-05	1.64E-05	4599/3458/3118/3113/3127/841/3119/3383/3115/7124/3123	11
KEGG	hsa05330	Allograft rejection	10/88	38/8096	5.03E-12	3.10E-10	2.33E-10	3458/3118/3113/3127/3119/3002/3115/7124/5551/3123	10
KEGG	hsa04940	Type I diabetes mellitus	10/88	43/8096	1.95E-11	9.03E-10	6.78E-10	3458/3118/3113/3127/3119/3002/3115/7124/5551/3123	10
KEGG	hsa05140	Leishmaniasis	10/88	77/8096	8.27E-09	1.91E-07	1.44E-07	3725/3458/2214/3118/3113/3127/3119/3115/7124/3123	10
KEGG	hsa04640	Hematopoietic cell lineage	10/88	99/8096	9.67E-08	1.38E-06	1.03E-06	3118/952/3113/3127/3119/925/928/3115/7124/3123	10
KEGG	hsa04659	Th17 cell differentiation	10/88	108/8096	2.22E-07	2.93E-06	2.20E-06	3560/3725/3662/3458/3118/3113/3127/3119/3115/3123	10
KEGG	hsa04650	Natural killer cell mediated cytotoxicity	10/88	131/8096	1.35E-06	1.39E-05	1.04E-05	3821/2207/3458/2214/117157/3383/3002/3824/7124/5551	10

KEGG	hsa04060	Cytokine-cytokine receptor interaction	10/88	295/8096	0.001291399	0.006991497	0.005251081	1524/3560/3458/6375/6348/6846/6351/9235/7124/9560	10
KEGG	hsa05416	Viral myocarditis	9/88	60/8096	1.31E-08	2.70E-07	2.03E-07	3118/3113/3127/841/3119/3383/3115/5551/3123	9
KEGG	hsa05321	Inflammatory bowel disease	9/88	65/8096	2.72E-08	4.92E-07	3.70E-07	3725/3458/3118/3113/3127/3119/3115/7124/3123	9
KEGG	hsa04658	Th1 and Th2 cell differentiation	9/88	92/8096	5.81E-07	6.72E-06	5.05E-06	3560/3725/3458/3118/3113/3127/3119/3115/3123	9
KEGG	hsa04210	Apoptosis	9/88	136/8096	1.52E-05	0.000108008	8.11E-05	3725/4000/7185/841/3002/1521/7124/5551/597	9
KEGG	hsa05322	Systemic lupus erythematosus	9/88	136/8096	1.52E-05	0.000108008	8.11E-05	3458/2214/3118/3113/3127/3119/3115/7124/3123	9
KEGG	hsa05417	Lipid and atherosclerosis	9/88	215/8096	0.000514851	0.003284392	0.002466798	3725/3303/6348/3949/841/3304/3383/19/7124	9
KEGG	hsa04010	MAPK signaling pathway	9/88	294/8096	0.004485764	0.021278624	0.015981669	374/3725/3303/1846/3304/7124/3925/3164/1844	9
KEGG	hsa05310	Asthma	8/88	31/8096	9.07E-10	2.80E-08	2.10E-08	2207/3118/3113/3127/3119/3115/7124/3123	8
KEGG	hsa05320	Autoimmune thyroid disease	8/88	53/8096	8.39E-08	1.29E-06	9.72E-07	3118/3113/3127/3119/3002/3115/5551/3123	8
KEGG	hsa05150	Staphylococcus aureus infection	8/88	96/8096	8.59E-06	7.22E-05	5.43E-05	2214/3118/3113/3127/3119/3383/3115/3123	8
KEGG	hsa04061	Viral protein interaction with cytokine and cytokine receptor	8/88	100/8096	1.16E-05	9.36E-05	7.03E-05	1524/3560/6375/6348/6846/6351/7124/9560	8
KEGG	hsa04514	Cell adhesion molecules	8/88	149/8096	0.000202051	0.00133498	0.001002659	3118/3113/3127/3119/3383/925/3115/3123	8
KEGG	hsa05202	Transcriptional misregulation in cancer	8/88	192/8096	0.001098467	0.006350513	0.00476966	3560/64332/3248/7185/3002/1026/8013/597	8
KEGG	hsa05144	Malaria	7/88	50/8096	9.63E-07	1.05E-05	7.87E-06	3039/3458/3040/3820/3383/7124/3043	7
KEGG	hsa04064	NF-kappa B signaling pathway	7/88	104/8096	0.000127503	0.00087363	0.000656154	7128/7185/6351/3383/7124/9560/597	7

KEGG	hsa05162	Measles	7/88	139/8096	0.000753816	0.00464853	0.003491357	3560/7128/4599/3725/3303/841/3304	7
KEGG	hsa04145	Phagosome	7/88	152/8096	0.001275022	0.006991497	0.005251081	2214/3118/3113/3127/3119/3115/3123	7
KEGG	hsa05160	Hepatitis C	7/88	157/8096	0.001537853	0.007902858	0.005935575	4599/3458/7533/3949/841/1026/7124	7
KEGG	hsa05143	African trypanosomiasis	6/88	37/8096	2.46E-06	2.28E-05	1.71E-05	3039/3458/3040/3383/7124/3043	6
KEGG	hsa04672	Intestinal immune network for IgA production	6/88	49/8096	1.33E-05	0.000102888	7.73E-05	3118/3113/3127/3119/3115/3123	6
KEGG	hsa04620	Toll-like receptor signaling pathway	6/88	104/8096	0.000898979	0.005364874	0.004029379	3725/6348/6351/841/7124/9560	6
KEGG	hsa04668	TNF signaling pathway	6/88	112/8096	0.001322716	0.006991497	0.005251081	7128/3725/7185/841/3383/7124	6
KEGG	hsa04657	IL-17 signaling pathway	5/88	94/8096	0.0034781	0.016932855	0.012717706	7128/3725/3458/841/7124	5
KEGG	hsa05142	Chagas disease	5/88	102/8096	0.0049326	0.022813274	0.017134294	3725/3458/6348/841/7124	5
KEGG	hsa05134	Legionellosis	4/88	57/8096	0.003319873	0.016599366	0.012467234	3303/841/3304/7124	4
KEGG	hsa05219	Bladder cancer	3/88	41/8096	0.009821371	0.044315941	0.033284234	1026/1612/1890	3
