# Supplementary material for: Recognition of Immune Cell Markers of COVID-19 Severity with Machine Learning Methods
Source: Biomed Res Int. 2022 Apr 28;2022:6089242. doi: 10.1155/2022/6089242 (PMC9073549; doi:10.1155/2022/6089242)
Supplement: Supplementary 3 — Table S3: classification rules extracted by the optimal decision tree classifiers in different immune cell type. [file 6089242.f3.pdf]

Table S3. Classification rules extracted by the optimal decision tree classifiers in different immune cell type

(1) B cell

Rules_0	passed counts:1355
node_0: feature_name=LTB	LTB.value <= threshold=0.994031548500061
node_1: feature_name=MTRNR2L2	MTRNR2L2.value > threshold=2.8784297704696655
node_5207: feature_name=MTRNR2L8	MTRNR2L8.value <= threshold=3.1915172338485718
node_5208: feature_name=MT-CYB	MT-CYB.value <= threshold=1.3929389119148254
node_5209: feature_name=SLC7A5	SLC7A5.value <= threshold=3.205444812774658
node_5210: feature_name=MT-CO2	MT-CO2.value <= threshold=2.065874218940735
node_5211: feature_name=HLA-DRB5	HLA-DRB5.value <= threshold=2.276706099510193
node_5212: feature_name=TLR2	TLR2.value <= threshold=4.805860757827759
node_5213: feature_name=XIST	XIST.value <= threshold=4.313443303108215
node_5214: feature_name=TTN	TTN.value <= threshold=7.185946702957153
node_5215: feature_name=ITGAX	ITGAX.value <= threshold=3.106598973274231
node_5216: feature_name=PLEC	PLEC.value <= threshold=3.848032832145691
node_5217: feature_name=BCL2A1	BCL2A1.value <= threshold=2.4758780002593994
node_5218: feature_name=ATG2A	ATG2A.value <= threshold=3.2491225004196167
node_5219: feature_name=MT-CO3	MT-CO3.value > threshold=-1.363724172115326
Class: COVID-19 Severe	
Rules_1	passed counts:1097
node_0: feature_name=LTB	LTB.value > threshold=0.994031548500061
node_5264: feature_name=MT-CO1	MT-CO1.value > threshold=-0.4127991199493408
node_5638: feature_name=OAZ1	OAZ1.value > threshold=-0.2640867084264755
node_6112: feature_name=IFI44L	IFI44L.value <= threshold=2.5893263816833496
node_6113: feature_name=TCL1A	TCL1A.value > threshold=4.201832294464111
node_6991: feature_name=XAF1	XAF1.value <= threshold=3.4585667848587036
node_6992: feature_name=STAG3	STAG3.value <= threshold=7.686291694641113
node_6993: feature_name=RALGPS2	RALGPS2.value > threshold=-0.4090157449245453
node_6997: feature_name=MTRNR2L2	MTRNR2L2.value <= threshold=3.23723828792572

node_6998: feature_name=TP53INP1	TP53INP1.value <= threshold=6.239925146102905
node_6999: feature_name=MEF2C	MEF2C.value > threshold=-0.44642284512519836
node_7003: feature_name=HSPA1A	HSPA1A.value <= threshold=7.404359817504883
node_7004: feature_name=COTL1	COTL1.value > threshold=-1.6598749160766602
node_7006: feature_name=HIST1H2AC	HIST1H2AC.value > threshold=-0.34910088777542114
node_7008: feature_name=MARCH1	MARCH1.value <= threshold=5.393903970718384
node_7009: feature_name=PELI1	PELI1.value <= threshold=3.565749168395996
node_7010: feature_name=RGS1	RGS1.value <= threshold=2.851950168609619
node_7011: feature_name=HLA-DRB5	HLA-DRB5.value <= threshold=3.0528379678726196
node_7012: feature_name=MEF2A	MEF2A.value <= threshold=3.228689193725586
node_7013: feature_name=MT-CO1	MT-CO1.value > threshold=-0.37944722175598145
node_7017: feature_name=ATG2A	ATG2A.value <= threshold=2.790610194206238
node_7018: feature_name=MEF2C	MEF2C.value > threshold=-0.404978945851326
node_7026: feature_name=MT-CO3	MT-CO3.value > threshold=-1.699916422367096
node_7030: feature_name=CD79A	CD79A.value > threshold=0.17886222898960114
node_7034: feature_name=CCDC141	CCDC141.value <= threshold=5.498055934906006
node_7035: feature_name=SNX9	SNX9.value <= threshold=2.924591064453125
node_7036: feature_name=RP5-821D11.7	RP5-821D11.7.value <= threshold=4.530828952789307
node_7037: feature_name=IRAK3	IRAK3.value <= threshold=3.0760087966918945
Class: Healthy Control	
Rules_2	passed counts:1026
node_0: feature_name=LTB	LTB.value <= threshold=0.994031548500061
node_1: feature_name=MTRNR2L2	MTRNR2L2.value <= threshold=2.8784297704696655
node_2: feature_name=TCL1A	TCL1A.value <= threshold=4.798312664031982
node_3: feature_name=MT-CO1	MT-CO1.value <= threshold=0.08947191759943962
node_4: feature_name=NR4A1	NR4A1.value <= threshold=1.0725348591804504
node_5: feature_name=HLA-DPA1	HLA-DPA1.value <= threshold=1.6885469555854797
node_6: feature_name=HLA-DRB5	HLA-DRB5.value <= threshold=2.1886194944381714
node_7: feature_name=XIST	XIST.value <= threshold=-0.12328458577394485

node_8: feature_name=SYNE1	SYNE1.value > threshold=-0.7835594415664673
node_16: feature_name=MTRNR2L2	MTRNR2L2.value <= threshold=2.4483247995376587
node_17: feature_name=HLA-DQA2	HLA-DQA2.value <= threshold=2.227178931236267
node_18: feature_name=NR4A1	NR4A1.value <= threshold=-0.21380723267793655
node_19: feature_name=IRAK3	IRAK3.value <= threshold=4.27159571647644
node_20: feature_name=IFIT2	IFIT2.value <= threshold=7.8175740242004395
node_21: feature_name=CD79A	CD79A.value <= threshold=2.857242226600647
node_22: feature_name=HIST1H2AC	HIST1H2AC.value <= threshold=5.077470302581787
node_23: feature_name=FTH1	FTH1.value <= threshold=0.4158056080341339
node_24: feature_name=ATF3	ATF3.value <= threshold=3.8028353452682495
node_25: feature_name=OASL	OASL.value <= threshold=3.387580633163452
node_26: feature_name=MT-CO1	MT-CO1.value > threshold=-0.7942646145820618
node_74: feature_name=HOPX	HOPX.value <= threshold=0.8682331740856171
node_75: feature_name=RGS2	RGS2.value <= threshold=1.4589954018592834
node_76: feature_name=AHR	AHR.value <= threshold=3.870481848716736
node_77: feature_name=IGJ	IGJ.value <= threshold=4.902882814407349
node_78: feature_name=CD79B	CD79B.value <= threshold=4.87716269493103
node_79: feature_name=HLA-DQB1	HLA-DQB1.value <= threshold=3.6925491094589233
node_80: feature_name=HLA-DRB5	HLA-DRB5.value <= threshold=2.0926843881607056
node_81: feature_name=MCTP1	MCTP1.value <= threshold=4.010472774505615
node_82: feature_name=GNG11	GNG11.value <= threshold=3.627470850944519
node_83: feature_name=HSP90B1	HSP90B1.value <= threshold=2.0835131406784058
node_84: feature_name=ATF3	ATF3.value <= threshold=1.1142374873161316
node_85: feature_name=MT-ND6	MT-ND6.value <= threshold=2.7328014373779297
node_86: feature_name=COTL1	COTL1.value > threshold=-1.6524587869644165
node_90: feature_name=MT-CYB	MT-CYB.value <= threshold=1.099629819393158
node_91: feature_name=RGCC	RGCC.value <= threshold=1.6908138990402222
node_92: feature_name=ILK	ILK.value <= threshold=3.9497616291046143
node_93: feature_name=CTD-2035E11.3	CTD-2035E11.3.value <= threshold=2.4543306827545166
node_94: feature_name=S100A6	S100A6.value <= threshold=0.2867250442504883

node_95: feature_name=HLA-DRB5	HLA-DRB5.value > threshold=-0.9496813416481018
node_99: feature_name=MT-CO3	MT-CO3.value > threshold=-1.55654776096344
node_103: feature_name=ITGAX	ITGAX.value <= threshold=1.8544010519981384
node_104: feature_name=MEF2C	MEF2C.value <= threshold=4.22724461555481
node_105: feature_name=CXorf57	CXorf57.value <= threshold=5.114444732666016
node_106: feature_name=IRAK3	IRAK3.value <= threshold=3.1017903089523315
node_107: feature_name=DUSP4	DUSP4.value <= threshold=2.9122174978256226
node_108: feature_name=OAZ1	OAZ1.value > threshold=-1.8057305812835693
node_112: feature_name=DAPP1	DAPP1.value <= threshold=4.016228437423706
node_113: feature_name=HSP90B1	HSP90B1.value > threshold=-1.2981283068656921
node_119: feature_name=MT-CYB	MT-CYB.value <= threshold=0.9500376582145691
node_120: feature_name=HSP90B1	HSP90B1.value <= threshold=1.7814298272132874
node_121: feature_name=ACSL1	ACSL1.value <= threshold=2.289203405380249
Class: COVID-19 Critical	
Rules_3	passed counts:970
node_0: feature_name=LTB	LTB.value > threshold=0.994031548500061
node_5264: feature_name=MT-CO1	MT-CO1.value > threshold=-0.4127991199493408
node_5638: feature_name=OAZ1	OAZ1.value > threshold=-0.2640867084264755
node_6112: feature_name=IFI44L	IFI44L.value <= threshold=2.5893263816833496
node_6113: feature_name=TCL1A	TCL1A.value <= threshold=4.201832294464111
node_6114: feature_name=HLA-DQA2	HLA-DQA2.value <= threshold=-0.2270243540406227
node_6115: feature_name=MTRNR2L2	MTRNR2L2.value <= threshold=2.548917293548584
node_6116: feature_name=ATF5	ATF5.value <= threshold=2.041263461112976
node_6117: feature_name=JUN	JUN.value > threshold=-0.616259902715683
node_6189: feature_name=MT-CO3	MT-CO3.value > threshold=0.13483521342277527
node_6351: feature_name=XAF1	XAF1.value <= threshold=3.061717987060547
node_6352: feature_name=MT-CO2	MT-CO2.value <= threshold=1.2605181336402893
node_6353: feature_name=ICAM1	ICAM1.value <= threshold=-0.3340446650981903
node_6354: feature_name=OAZ1	OAZ1.value > threshold=-0.24055946618318558

node_6360: feature_name=PPIF	PPIF.value <= threshold=2.070030093193054
node_6361: feature_name=RALGPS2	RALGPS2.value > threshold=-0.3684315085411072
node_6369: feature_name=ANO6	ANO6.value <= threshold=3.504153251647949
node_6370: feature_name=YWHAH	YWHAH.value <= threshold=4.3182291984558105
node_6371: feature_name=LDLR	LDLR.value <= threshold=2.494291305541992
node_6372: feature_name=HLA-DQA1	HLA-DQA1.value > threshold=-0.5716990232467651
node_6374: feature_name=ABCA1	ABCA1.value <= threshold=3.7662240266799927
node_6375: feature_name=MS4A1	MS4A1.value > threshold=1.4995208978652954
node_6387: feature_name=SNORD3B-2	SNORD3B-2.value <= threshold=4.154061555862427
node_6388: feature_name=LTB	LTB.value <= threshold=2.1739100217819214
node_6389: feature_name=BACH1	BACH1.value <= threshold=2.0371285676956177
node_6390: feature_name=HLA-DQB1	HLA-DQB1.value <= threshold=3.0258322954177856
node_6391: feature_name=HLA-DPB1	HLA-DPB1.value > threshold=0.2591289207339287
node_6395: feature_name=CXorf57	CXorf57.value <= threshold=3.3742183446884155
node_6396: feature_name=MMD	MMD.value <= threshold=3.8883349895477295
node_6397: feature_name=IRF8	IRF8.value <= threshold=3.939465045928955
node_6398: feature_name=PPIF	PPIF.value <= threshold=1.1284508109092712
node_6399: feature_name=S100A6	S100A6.value > threshold=-2.1265565156936646
node_6403: feature_name=MEF2A	MEF2A.value <= threshold=2.771904706954956
Class: Healthy Control	
Rules_4	passed counts:950
node_0: feature_name=LTB	LTB.value <= threshold=0.994031548500061
node_1: feature_name=MTRNR2L2	MTRNR2L2.value <= threshold=2.8784297704696655
node_2: feature_name=TCL1A	TCL1A.value <= threshold=4.798312664031982
node_3: feature_name=MT-CO1	MT-CO1.value <= threshold=0.08947191759943962
node_4: feature_name=NR4A1	NR4A1.value <= threshold=1.0725348591804504
node_5: feature_name=HLA-DPA1	HLA-DPA1.value <= threshold=1.6885469555854797
node_6: feature_name=HLA-DRB5	HLA-DRB5.value <= threshold=2.1886194944381714
node_7: feature_name=XIST	XIST.value <= threshold=-0.12328458577394485

node_8: feature_name=SYNE1	SYNE1.value > threshold=-0.7835594415664673
node_16: feature_name=MTRNR2L2	MTRNR2L2.value <= threshold=2.4483247995376587
node_17: feature_name=HLA-DQA2	HLA-DQA2.value <= threshold=2.227178931236267
node_18: feature_name=NR4A1	NR4A1.value <= threshold=-0.21380723267793655
node_19: feature_name=IRAK3	IRAK3.value <= threshold=4.27159571647644
node_20: feature_name=IFIT2	IFIT2.value <= threshold=7.8175740242004395
node_21: feature_name=CD79A	CD79A.value <= threshold=2.857242226600647
node_22: feature_name=HIST1H2AC	HIST1H2AC.value <= threshold=5.077470302581787
node_23: feature_name=FTH1	FTH1.value <= threshold=0.4158056080341339
node_24: feature_name=ATF3	ATF3.value <= threshold=3.8028353452682495
node_25: feature_name=OASL	OASL.value <= threshold=3.387580633163452
node_26: feature_name=MT-CO1	MT-CO1.value <= threshold=-0.7942646145820618
node_27: feature_name=FCER2	FCER2.value <= threshold=7.268347978591919
node_28: feature_name=CCL3	CCL3.value <= threshold=3.2822797298431396
node_29: feature_name=BCL2A1	BCL2A1.value <= threshold=3.1504160165786743
node_30: feature_name=GRAP2	GRAP2.value <= threshold=3.0517499446868896
node_31: feature_name=EGR1	EGR1.value <= threshold=7.645251512527466
node_32: feature_name=GPX1	GPX1.value <= threshold=0.6752602756023407
node_33: feature_name=CD79B	CD79B.value <= threshold=4.365710258483887
node_34: feature_name=S100A6	S100A6.value <= threshold=0.4172801077365875
node_35: feature_name=ID3	ID3.value <= threshold=3.093442916870117
node_36: feature_name=ATF3	ATF3.value <= threshold=2.6047290563583374
node_37: feature_name=PLA2G12A	PLA2G12A.value <= threshold=3.5468435287475586
Class: COVID-19 Critical	
Rules_5	passed counts:579
node_0: feature_name=LTB	LTB.value <= threshold=0.994031548500061
node_1: feature_name=MTRNR2L2	MTRNR2L2.value <= threshold=2.8784297704696655
node_2: feature_name=TCL1A	TCL1A.value <= threshold=4.798312664031982
node_3: feature_name=MT-CO1	MT-CO1.value > threshold=0.08947191759943962

node_2521: feature_name=LTB	LTB.value <= threshold=0.6038667261600494
node_2522: feature_name=HLA-DQB1	HLA-DQB1.value <= threshold=2.1053054332733154
node_2523: feature_name=SNX9	SNX9.value > threshold=-0.5346486568450928
node_2907: feature_name=NR4A1	NR4A1.value <= threshold=2.5433837175369263
node_2908: feature_name=XIST	XIST.value <= threshold=0.7664470989257097
node_2909: feature_name=CD79B	CD79B.value <= threshold=2.9326847791671753
node_2910: feature_name=NR4A1	NR4A1.value <= threshold=-0.2875557690858841
node_2911: feature_name=CTD-2035E11.3	CTD-2035E11.3.value <= threshold=5.262048006057739
node_2912: feature_name=VPREB3	VPREB3.value <= threshold=4.937594890594482
node_2913: feature_name=IRAK3	IRAK3.value <= threshold=4.592883586883545
node_2914: feature_name=IGJ	IGJ.value <= threshold=7.093410015106201
node_2915: feature_name=WDR74	WDR74.value <= threshold=4.077916383743286
node_2916: feature_name=MEF2C	MEF2C.value <= threshold=4.374382972717285
node_2917: feature_name=HIST1H2AC	HIST1H2AC.value <= threshold=3.743621349334717
node_2918: feature_name=STMN1	STMN1.value <= threshold=6.266510248184204
node_2919: feature_name=RALGPS2	RALGPS2.value <= threshold=5.72194766998291
node_2920: feature_name=HOPX	HOPX.value <= threshold=2.5944807529449463
node_2921: feature_name=MARCKS	MARCKS.value <= threshold=4.622651815414429
node_2922: feature_name=CCL3	CCL3.value <= threshold=0.7484713792800903
node_2923: feature_name=TPM1	TPM1.value <= threshold=6.446193695068359
node_2924: feature_name=PLD4	PLD4.value <= threshold=8.038907289505005
node_2925: feature_name=COTL1	COTL1.value > threshold=-1.8123814463615417
node_2929: feature_name=ID3	ID3.value <= threshold=7.534881353378296
node_2930: feature_name=IRF8	IRF8.value <= threshold=4.855781078338623
node_2931: feature_name=AP3M2	AP3M2.value <= threshold=1.8868122100830078
node_2932: feature_name=FCER2	FCER2.value <= threshold=6.877448558807373
node_2933: feature_name=MTRNR2L2	MTRNR2L2.value <= threshold=2.710945725440979
node_2934: feature_name=GNG11	GNG11.value <= threshold=2.510925054550171
node_2935: feature_name=CD83	CD83.value <= threshold=1.9873459935188293
node_2936: feature_name=MARCH1	MARCH1.value <= threshold=5.006200551986694

node_2937: feature_name=MEF2C	MEF2C.value > threshold=-0.5498070418834686
node_2941: feature_name=FCRL1	FCRL1.value <= threshold=7.093972444534302
node_2942: feature_name=HSP90B1	HSP90B1.value > threshold=-1.4724047780036926
node_2946: feature_name=HLA-DQA1	HLA-DQA1.value > threshold=-0.45666155219078064
node_2950: feature_name=RELT	RELT.value <= threshold=1.5976553559303284
Class: COVID-19 Critical	
Rules_6	passed counts:459
node_0: feature_name=LTB	LTB.value <= threshold=0.994031548500061
node_1: feature_name=MTRNR2L2	MTRNR2L2.value <= threshold=2.8784297704696655
node_2: feature_name=TCL1A	TCL1A.value <= threshold=4.798312664031982
node_3: feature_name=MT-CO1	MT-CO1.value <= threshold=0.08947191759943962
node_4: feature_name=NR4A1	NR4A1.value <= threshold=1.0725348591804504
node_5: feature_name=HLA-DPA1	HLA-DPA1.value > threshold=1.6885469555854797
node_861: feature_name=CD83	CD83.value <= threshold=3.0563642978668213
node_862: feature_name=HLA-DQB1	HLA-DQB1.value <= threshold=2.61081063747406
node_863: feature_name=MTRNR2L2	MTRNR2L2.value <= threshold=2.0919997692108154
node_864: feature_name=BCL2A1	BCL2A1.value <= threshold=1.852618932723999
node_865: feature_name=CD79B	CD79B.value <= threshold=4.352142333984375
node_866: feature_name=TNFAIP3	TNFAIP3.value <= threshold=-0.0449496041983366
node_867: feature_name=MT-CO1	MT-CO1.value <= threshold=-0.08531957119703293
node_868: feature_name=MTRNR2L8	MTRNR2L8.value <= threshold=0.8770350813865662
node_869: feature_name=EGR1	EGR1.value <= threshold=-0.32270750403404236
node_870: feature_name=IGJ	IGJ.value <= threshold=4.618318557739258
node_871: feature_name=DUSP4	DUSP4.value <= threshold=2.8197922706604004
node_872: feature_name=HOPX	HOPX.value <= threshold=2.10397732257843
node_873: feature_name=RGCC	RGCC.value <= threshold=1.2070720195770264
node_874: feature_name=OAZ1	OAZ1.value <= threshold=1.02116060256958
node_875: feature_name=NAIP	NAIP.value <= threshold=3.768904685974121
node_876: feature_name=LDLR	LDLR.value <= threshold=2.9582698345184326

node_877: feature_name=HLA-DPA1	HLA-DPA1.value <= threshold=3.0588693618774414
node_878: feature_name=RHOB	RHOB.value <= threshold=5.08755898475647
node_879: feature_name=SYTL3	SYTL3.value <= threshold=1.5127440690994263
node_880: feature_name=FTH1	FTH1.value > threshold=-1.4152275919914246
node_882: feature_name=CCL3	CCL3.value <= threshold=2.253131687641144
node_883: feature_name=CD83	CD83.value <= threshold=2.8566592931747437
node_884: feature_name=HLA-DRB5	HLA-DRB5.value > threshold=-0.6464254856109619
node_888: feature_name=HSP90B1	HSP90B1.value > threshold=-1.186358630657196
node_892: feature_name=ATG2A	ATG2A.value <= threshold=2.3624722957611084
node_893: feature_name=ICAM1	ICAM1.value <= threshold=2.4306468963623047
node_894: feature_name=CD38	CD38.value <= threshold=3.4977954626083374
node_895: feature_name=HLA-DRB1	HLA-DRB1.value > threshold=0.28603747487068176
node_899: feature_name=XIST	XIST.value <= threshold=1.4272559583187103
node_900: feature_name=BANK1	BANK1.value <= threshold=5.060973644256592
node_901: feature_name=IRF4	IRF4.value <= threshold=6.117950916290283
node_902: feature_name=MTRNR2L2	MTRNR2L2.value <= threshold=1.8410876989364624
node_903: feature_name=TPM1	TPM1.value <= threshold=4.590091228485107
node_904: feature_name=SNORD3B-2	SNORD3B-2.value <= threshold=4.349201917648315
node_905: feature_name=AHR	AHR.value <= threshold=2.268427610397339
node_906: feature_name=OAZ1	OAZ1.value > threshold=-1.4369714260101318
node_914: feature_name=MT-ND6	MT-ND6.value <= threshold=1.7712779641151428
node_915: feature_name=HLA-DPB1	HLA-DPB1.value <= threshold=2.6004384756088257
node_916: feature_name=COTL1	COTL1.value > threshold=-1.1624112129211426
Class: COVID-19 Critical	
Rules_7	passed counts:419
node_0: feature_name=LTB	LTB.value <= threshold=0.994031548500061
node_1: feature_name=MTRNR2L2	MTRNR2L2.value <= threshold=2.8784297704696655
node_2: feature_name=TCL1A	TCL1A.value <= threshold=4.798312664031982
node_3: feature_name=MT-CO1	MT-CO1.value <= threshold=0.08947191759943962

node_4: feature_name=NR4A1	NR4A1.value > threshold=1.0725348591804504
node_1622: feature_name=HLA-DQA2	HLA-DQA2.value <= threshold=1.9496816992759705
node_1623: feature_name=HLA-DRB5	HLA-DRB5.value <= threshold=2.715766429901123
node_1624: feature_name=HLA-DPA1	HLA-DPA1.value <= threshold=1.7050301432609558
node_1625: feature_name=CD83	CD83.value <= threshold=2.783489227294922
node_1626: feature_name=XIST	XIST.value <= threshold=0.9589543007314205
node_1627: feature_name=PLD4	PLD4.value <= threshold=5.865932464599609
node_1628: feature_name=OAZ1	OAZ1.value <= threshold=0.6137169003486633
node_1629: feature_name=MTRNR2L2	MTRNR2L2.value <= threshold=1.6102792620658875
node_1630: feature_name=IFI6	IFI6.value > threshold=-0.8404486179351807
node_1632: feature_name=NR4A1	NR4A1.value > threshold=1.0916320085525513
node_1638: feature_name=DENND3	DENND3.value <= threshold=3.988452672958374
node_1639: feature_name=HLA-DRB1	HLA-DRB1.value > threshold=1.4092575907707214
node_1749: feature_name=HLA-DQB1	HLA-DQB1.value <= threshold=3.4929215908050537
node_1750: feature_name=RP11-685N3.1	RP11-685N3.1.value <= threshold=9.97533893585205
node_1751: feature_name=RGS1	RGS1.value > threshold=-0.5999330878257751
node_1765: feature_name=MT-ND6	MT-ND6.value <= threshold=2.4838690757751465
node_1766: feature_name=DAPK1	DAPK1.value <= threshold=4.920811414718628
node_1767: feature_name=RGS1	RGS1.value <= threshold=3.3605728149414062
node_1768: feature_name=ANO6	ANO6.value <= threshold=4.00420081615448
node_1769: feature_name=FTH1	FTH1.value <= threshold=1.6050249934196472
node_1770: feature_name=APP	APP.value <= threshold=2.417694091796875
node_1771: feature_name=ITGAX	ITGAX.value <= threshold=2.950524091720581
node_1772: feature_name=PDLIM1	PDLIM1.value <= threshold=5.856243848800659
node_1773: feature_name=RELT	RELT.value <= threshold=2.891391396522522
node_1774: feature_name=RHOB	RHOB.value <= threshold=3.2315229177474976
node_1775: feature_name=NR4A1	NR4A1.value > threshold=1.125234842300415
node_1781: feature_name=LTB	LTB.value <= threshold=0.7502643764019012
node_1782: feature_name=HSP90B1	HSP90B1.value <= threshold=1.9601801037788391
Class: COVID-19 Critical	

Rules_8	passed counts:374
node_0: feature_name=LTB	LTB.value <= threshold=0.994031548500061
node_1: feature_name=MTRNR2L2	MTRNR2L2.value <= threshold=2.8784297704696655
node_2: feature_name=TCL1A	TCL1A.value <= threshold=4.798312664031982
node_3: feature_name=MT-CO1	MT-CO1.value <= threshold=0.08947191759943962
node_4: feature_name=NR4A1	NR4A1.value <= threshold=1.0725348591804504
node_5: feature_name=HLA-DPA1	HLA-DPA1.value <= threshold=1.6885469555854797
node_6: feature_name=HLA-DRB5	HLA-DRB5.value <= threshold=2.1886194944381714
node_7: feature_name=XIST	XIST.value <= threshold=-0.12328458577394485
node_8: feature_name=SYNE1	SYNE1.value > threshold=-0.7835594415664673
node_16: feature_name=MTRNR2L2	MTRNR2L2.value <= threshold=2.4483247995376587
node_17: feature_name=HLA-DQA2	HLA-DQA2.value <= threshold=2.227178931236267
node_18: feature_name=NR4A1	NR4A1.value <= threshold=-0.21380723267793655
node_19: feature_name=IRAK3	IRAK3.value <= threshold=4.27159571647644
node_20: feature_name=IFIT2	IFIT2.value <= threshold=7.8175740242004395
node_21: feature_name=CD79A	CD79A.value <= threshold=2.857242226600647
node_22: feature_name=HIST1H2AC	HIST1H2AC.value <= threshold=5.077470302581787
node_23: feature_name=FTH1	FTH1.value > threshold=0.4158056080341339
node_285: feature_name=EGR1	EGR1.value <= threshold=4.240467071533203
node_286: feature_name=HLA-DRB1	HLA-DRB1.value > threshold=-0.7553382813930511
node_290: feature_name=TLR2	TLR2.value <= threshold=2.879140257835388
node_291: feature_name=SYNE1	SYNE1.value <= threshold=2.323199510574341
node_292: feature_name=BCL2A1	BCL2A1.value <= threshold=1.8940600156784058
node_293: feature_name=RALGPS2	RALGPS2.value <= threshold=4.808189153671265
node_294: feature_name=IGLL5	IGLL5.value <= threshold=4.116017818450928
node_295: feature_name=FTH1	FTH1.value > threshold=0.41612209379673004
node_297: feature_name=MCTP1	MCTP1.value <= threshold=4.601342678070068
node_298: feature_name=MT-CO2	MT-CO2.value <= threshold=0.6201252341270447
node_299: feature_name=MARCKS	MARCKS.value <= threshold=3.471073269844055

node_300: feature_name=PPIF	PPIF.value <= threshold=2.8470659255981445
node_301: feature_name=CCL3	CCL3.value <= threshold=1.9901156425476074
node_302: feature_name=MT-CO2	MT-CO2.value > threshold=-2.867768883705139
node_306: feature_name=RHOB	RHOB.value <= threshold=3.9839000701904297
node_307: feature_name=SNX29P2	SNX29P2.value <= threshold=5.035826921463013
node_308: feature_name=MT-CO3	MT-CO3.value <= threshold=0.7808255851268768
node_309: feature_name=RGCC	RGCC.value <= threshold=1.070733904838562
node_310: feature_name=SESN3	SESN3.value <= threshold=2.5199365615844727
node_311: feature_name=ABCA1	ABCA1.value <= threshold=2.989766836166382
Class: COVID-19 Critical	
Rules_9	passed counts:347
node_0: feature_name=LTB	LTB.value <= threshold=0.994031548500061
node_1: feature_name=MTRNR2L2	MTRNR2L2.value <= threshold=2.8784297704696655
node_2: feature_name=TCL1A	TCL1A.value > threshold=4.798312664031982
node_4912: feature_name=MT-CO1	MT-CO1.value > threshold=-0.3664844036102295
node_5002: feature_name=IFI44L	IFI44L.value <= threshold=2.3101747035980225
node_5003: feature_name=SNX9	SNX9.value <= threshold=2.0247421264648438
node_5004: feature_name=STAG3	STAG3.value <= threshold=6.482141733169556
node_5005: feature_name=BACH1	BACH1.value <= threshold=-0.5237349569797516
node_5006: feature_name=OAZ1	OAZ1.value > threshold=-0.6512158215045929
node_5014: feature_name=MEF2A	MEF2A.value <= threshold=3.61627995967865
node_5015: feature_name=HLA-DPB1	HLA-DPB1.value > threshold=-0.2871522083878517
node_5019: feature_name=FTH1	FTH1.value <= threshold=1.0194584727287292
node_5020: feature_name=RNF144B	RNF144B.value <= threshold=3.085212469100952
node_5021: feature_name=TTN	TTN.value <= threshold=5.307270765304565
node_5022: feature_name=CCL3	CCL3.value <= threshold=1.4737252593040466
node_5023: feature_name=LTB	LTB.value > threshold=-1.121648371219635
node_5025: feature_name=HLA-DQB1	HLA-DQB1.value <= threshold=3.169318914413452
node_5026: feature_name=ATF3	ATF3.value <= threshold=2.082080602645874

node_5027: feature_name=SNX9	SNX9.value <= threshold=1.8630352020263672
node_5028: feature_name=MT-CO3	MT-CO3.value > threshold=-1.0862728953361511
Class: Healthy Control	
Rules_10	passed counts:342
node_0: feature_name=LTB	LTB.value <= threshold=0.994031548500061
node_1: feature_name=MTRNR2L2	MTRNR2L2.value <= threshold=2.8784297704696655
node_2: feature_name=TCL1A	TCL1A.value <= threshold=4.798312664031982
node_3: feature_name=MT-CO1	MT-CO1.value > threshold=0.08947191759943962
node_2521: feature_name=LTB	LTB.value <= threshold=0.6038667261600494
node_2522: feature_name=HLA-DQB1	HLA-DQB1.value <= threshold=2.1053054332733154
node_2523: feature_name=SNX9	SNX9.value > threshold=-0.5346486568450928
node_2907: feature_name=NR4A1	NR4A1.value <= threshold=2.5433837175369263
node_2908: feature_name=XIST	XIST.value <= threshold=0.7664470989257097
node_2909: feature_name=CD79B	CD79B.value <= threshold=2.9326847791671753
node_2910: feature_name=NR4A1	NR4A1.value <= threshold=-0.2875557690858841
node_2911: feature_name=CTD-2035E11.3	CTD-2035E11.3.value <= threshold=5.262048006057739
node_2912: feature_name=VPREB3	VPREB3.value <= threshold=4.937594890594482
node_2913: feature_name=IRAK3	IRAK3.value <= threshold=4.592883586883545
node_2914: feature_name=IGJ	IGJ.value <= threshold=7.093410015106201
node_2915: feature_name=WDR74	WDR74.value <= threshold=4.077916383743286
node_2916: feature_name=MEF2C	MEF2C.value <= threshold=4.374382972717285
node_2917: feature_name=HIST1H2AC	HIST1H2AC.value <= threshold=3.743621349334717
node_2918: feature_name=STMN1	STMN1.value <= threshold=6.266510248184204
node_2919: feature_name=RALGPS2	RALGPS2.value <= threshold=5.72194766998291
node_2920: feature_name=HOPX	HOPX.value <= threshold=2.5944807529449463
node_2921: feature_name=MARCKS	MARCKS.value <= threshold=4.622651815414429
node_2922: feature_name=CCL3	CCL3.value <= threshold=0.7484713792800903
node_2923: feature_name=TPM1	TPM1.value <= threshold=6.446193695068359
node_2924: feature_name=PLD4	PLD4.value <= threshold=8.038907289505005

node_2925: feature_name=COTL1	COTL1.value > threshold=-1.8123814463615417
node_2929: feature_name=ID3	ID3.value <= threshold=7.534881353378296
node_2930: feature_name=IRF8	IRF8.value <= threshold=4.855781078338623
node_2931: feature_name=AP3M2	AP3M2.value <= threshold=1.8868122100830078
node_2932: feature_name=FCER2	FCER2.value <= threshold=6.877448558807373
node_2933: feature_name=MTRNR2L2	MTRNR2L2.value <= threshold=2.710945725440979
node_2934: feature_name=GNG11	GNG11.value <= threshold=2.510925054550171
node_2935: feature_name=CD83	CD83.value > threshold=1.9873459935188293
node_2961: feature_name=CD83	CD83.value > threshold=2.0049850940704346
node_2965: feature_name=HES4	HES4.value <= threshold=4.076817035675049
node_2966: feature_name=BCL2A1	BCL2A1.value <= threshold=1.1745486855506897
node_2967: feature_name=NT5C3A	NT5C3A.value <= threshold=3.6911903619766235
node_2968: feature_name=TLN1	TLN1.value <= threshold=2.122964382171631
node_2969: feature_name=MT-CYB	MT-CYB.value > threshold=-0.9192460179328918
node_2973: feature_name=KIAA0125	KIAA0125.value <= threshold=6.9272027015686035
node_2974: feature_name=OAZ1	OAZ1.value > threshold=-1.8451763987541199
node_2978: feature_name=ATG2A	ATG2A.value <= threshold=1.4784215688705444
node_2979: feature_name=MMD	MMD.value <= threshold=4.39089298248291
Class: COVID-19 Critical	
Rules_11	passed counts:338
node_0: feature_name=LTB	LTB.value > threshold=0.994031548500061
node_5264: feature_name=MT-CO1	MT-CO1.value <= threshold=-0.4127991199493408
node_5265: feature_name=MTRNR2L2	MTRNR2L2.value <= threshold=2.4473838806152344
node_5266: feature_name=TCL1A	TCL1A.value <= threshold=5.787256956100464
node_5267: feature_name=PLD4	PLD4.value <= threshold=3.544657826423645
node_5268: feature_name=NR4A1	NR4A1.value <= threshold=0.8490704298019409
node_5269: feature_name=LTB	LTB.value <= threshold=1.5252206921577454
node_5270: feature_name=HLA-DRB5	HLA-DRB5.value <= threshold=2.5117164850234985
node_5271: feature_name=CD79A	CD79A.value <= threshold=3.685201406478882

node_5272: feature_name=BCL2A1	BCL2A1.value <= threshold=2.081526756286621
node_5273: feature_name=HLA-DPA1	HLA-DPA1.value <= threshold=1.8042166829109192
node_5274: feature_name=HSP90B1	HSP90B1.value > threshold=-1.3115270137786865
node_5278: feature_name=OAZ1	OAZ1.value <= threshold=1.0802008509635925
node_5279: feature_name=CD79B	CD79B.value <= threshold=5.875581979751587
node_5280: feature_name=CCDC141	CCDC141.value <= threshold=6.439151048660278
node_5281: feature_name=SYTL3	SYTL3.value <= threshold=1.149337351322174
node_5282: feature_name=GPX1	GPX1.value > threshold=-1.0243326425552368
node_5284: feature_name=MMD	MMD.value <= threshold=6.431635856628418
node_5285: feature_name=MARCKS	MARCKS.value <= threshold=4.034798860549927
node_5286: feature_name=XIST	XIST.value <= threshold=1.148451752960682
node_5287: feature_name=CD83	CD83.value <= threshold=2.894522190093994
node_5288: feature_name=MIR24-2	MIR24-2.value <= threshold=2.76771080493927
node_5289: feature_name=GRASP	GRASP.value > threshold=-0.4039058983325958
node_5303: feature_name=CD38	CD38.value <= threshold=3.721052408218384
node_5304: feature_name=ATF5	ATF5.value <= threshold=6.64192533493042
node_5305: feature_name=CD22	CD22.value <= threshold=5.268923759460449
node_5306: feature_name=CASP8	CASP8.value <= threshold=1.738209843635559
node_5307: feature_name=HSP90B1	HSP90B1.value > threshold=-1.2544044852256775
node_5311: feature_name=HLA-DPB1	HLA-DPB1.value <= threshold=2.4081677198410034
node_5312: feature_name=HSP90B1	HSP90B1.value <= threshold=1.8984123468399048
Class: COVID-19 Critical	
Rules_12	passed counts:324
node_0: feature_name=LTB	LTB.value <= threshold=0.994031548500061
node_1: feature_name=MTRNR2L2	MTRNR2L2.value <= threshold=2.8784297704696655
node_2: feature_name=TCL1A	TCL1A.value <= threshold=4.798312664031982
node_3: feature_name=MT-CO1	MT-CO1.value <= threshold=0.08947191759943962
node_4: feature_name=NR4A1	NR4A1.value <= threshold=1.0725348591804504
node_5: feature_name=HLA-DPA1	HLA-DPA1.value <= threshold=1.6885469555854797

node_6: feature_name=HLA-DRB5	HLA-DRB5.value <= threshold=2.1886194944381714
node_7: feature_name=XIST	XIST.value <= threshold=-0.12328458577394485
node_8: feature_name=SYNE1	SYNE1.value > threshold=-0.7835594415664673
node_16: feature_name=MTRNR2L2	MTRNR2L2.value <= threshold=2.4483247995376587
node_17: feature_name=HLA-DQA2	HLA-DQA2.value > threshold=2.227178931236267
node_591: feature_name=BCL2A1	BCL2A1.value <= threshold=1.0648428201675415
node_592: feature_name=FTH1	FTH1.value <= threshold=0.8842596709728241
node_593: feature_name=SNX9	SNX9.value <= threshold=3.232524275779724
node_594: feature_name=OAZ1	OAZ1.value <= threshold=1.704061508178711
node_595: feature_name=MT-CYB	MT-CYB.value <= threshold=0.5040059983730316
node_596: feature_name=CD83	CD83.value <= threshold=2.535894751548767
node_597: feature_name=ATF5	ATF5.value <= threshold=5.664362907409668
node_598: feature_name=IGLL5	IGLL5.value <= threshold=6.548790216445923
node_599: feature_name=EGR1	EGR1.value <= threshold=6.419021844863892
node_600: feature_name=PLEC	PLEC.value <= threshold=1.6971505880355835
node_601: feature_name=CD22	CD22.value <= threshold=6.541341304779053
node_602: feature_name=HSPA1A	HSPA1A.value <= threshold=3.400114059448242
node_603: feature_name=HBB	HBB.value <= threshold=6.7660768032073975
node_604: feature_name=FCRL1	FCRL1.value <= threshold=7.900821685791016
node_605: feature_name=STAG3	STAG3.value <= threshold=9.824122905731201
node_606: feature_name=SPI1	SPI1.value <= threshold=1.346154272556305
node_607: feature_name=TRIB1	TRIB1.value <= threshold=2.0271907448768616
Class: COVID-19 Critical	
Rules_13	passed counts:309
node_0: feature_name=LTB	LTB.value <= threshold=0.994031548500061
node_1: feature_name=MTRNR2L2	MTRNR2L2.value <= threshold=2.8784297704696655
node_2: feature_name=TCL1A	TCL1A.value <= threshold=4.798312664031982
node_3: feature_name=MT-CO1	MT-CO1.value <= threshold=0.08947191759943962
node_4: feature_name=NR4A1	NR4A1.value <= threshold=1.0725348591804504

node_5: feature_name=HLA-DPA1	HLA-DPA1.value <= threshold=1.6885469555854797
node_6: feature_name=HLA-DRB5	HLA-DRB5.value <= threshold=2.1886194944381714
node_7: feature_name=XIST	XIST.value <= threshold=-0.12328458577394485
node_8: feature_name=SYNE1	SYNE1.value > threshold=-0.7835594415664673
node_16: feature_name=MTRNR2L2	MTRNR2L2.value <= threshold=2.4483247995376587
node_17: feature_name=HLA-DQA2	HLA-DQA2.value <= threshold=2.227178931236267
node_18: feature_name=NR4A1	NR4A1.value <= threshold=-0.21380723267793655
node_19: feature_name=IRAK3	IRAK3.value <= threshold=4.27159571647644
node_20: feature_name=IFIT2	IFIT2.value <= threshold=7.8175740242004395
node_21: feature_name=CD79A	CD79A.value > threshold=2.857242226600647
node_375: feature_name=LTB	LTB.value <= threshold=0.7405790388584137
node_376: feature_name=NFKBIZ	NFKBIZ.value <= threshold=1.7718515396118164
node_377: feature_name=OAZ1	OAZ1.value <= threshold=1.3302325010299683
node_378: feature_name=RHOB	RHOB.value <= threshold=3.0459729433059692
node_379: feature_name=MT-CO3	MT-CO3.value <= threshold=1.6619641780853271
node_380: feature_name=MTRNR2L2	MTRNR2L2.value <= threshold=2.1229071617126465
node_381: feature_name=MAP3K2	MAP3K2.value <= threshold=3.626914143562317
node_382: feature_name=LDLR	LDLR.value <= threshold=2.571519374847412
node_383: feature_name=HLA-DRB1	HLA-DRB1.value > threshold=-0.7626054286956787
node_385: feature_name=CD79A	CD79A.value > threshold=2.857717990875244
node_387: feature_name=FCRL1	FCRL1.value <= threshold=6.850295543670654
node_388: feature_name=PDLIM1	PDLIM1.value <= threshold=4.4097795486450195
node_389: feature_name=MARCH1	MARCH1.value > threshold=-0.35520173609256744
node_413: feature_name=RP11-731F5.2	RP11-731F5.2.value <= threshold=6.423685789108276
node_414: feature_name=IL1B	IL1B.value <= threshold=1.5673747062683105
node_415: feature_name=MARCKS	MARCKS.value <= threshold=4.112927675247192
node_416: feature_name=TCF4	TCF4.value <= threshold=5.713138103485107
node_417: feature_name=HLA-DPB1	HLA-DPB1.value <= threshold=2.425311803817749
node_418: feature_name=CD79B	CD79B.value <= threshold=4.059849739074707
node_419: feature_name=MEF2C	MEF2C.value <= threshold=3.9403620958328247

Class: COVID-19 Critical	
Rules_14	passed counts:306
node_0: feature_name=LTB	LTB.value > threshold=0.994031548500061
node_5264: feature_name=MT-CO1	MT-CO1.value > threshold=-0.4127991199493408
node_5638: feature_name=OAZ1	OAZ1.value > threshold=-0.2640867084264755
node_6112: feature_name=IFI44L	IFI44L.value <= threshold=2.5893263816833496
node_6113: feature_name=TCL1A	TCL1A.value <= threshold=4.201832294464111
node_6114: feature_name=HLA-DQA2	HLA-DQA2.value <= threshold=-0.2270243540406227
node_6115: feature_name=MTRNR2L2	MTRNR2L2.value <= threshold=2.548917293548584
node_6116: feature_name=ATF5	ATF5.value <= threshold=2.041263461112976
node_6117: feature_name=JUN	JUN.value > threshold=-0.616259902715683
node_6189: feature_name=MT-CO3	MT-CO3.value <= threshold=0.13483521342277527
node_6190: feature_name=XAF1	XAF1.value <= threshold=2.584952235221863
node_6191: feature_name=HLA-DRB5	HLA-DRB5.value > threshold=0.6955901980400085
node_6239: feature_name=TNFAIP3	TNFAIP3.value <= threshold=0.11930759996175766
node_6240: feature_name=TP53INP1	TP53INP1.value <= threshold=2.9654784202575684
node_6241: feature_name=HLA-DRB5	HLA-DRB5.value <= threshold=2.5141202211380005
node_6242: feature_name=NFKBID	NFKBID.value <= threshold=3.358878970146179
node_6243: feature_name=JARID2	JARID2.value <= threshold=1.7160060405731201
node_6244: feature_name=HLA-DPA1	HLA-DPA1.value > threshold=0.49491266906261444
node_6246: feature_name=MIR24-2	MIR24-2.value <= threshold=1.8348549008369446
node_6247: feature_name=HSP90B1	HSP90B1.value <= threshold=1.8861054182052612
node_6248: feature_name=NR4A1	NR4A1.value > threshold=-0.5434737503528595
node_6274: feature_name=CD79A	CD79A.value > threshold=1.2718155980110168
node_6278: feature_name=RP5-821D11.7	RP5-821D11.7.value <= threshold=4.768282890319824
node_6279: feature_name=FAM160B1	FAM160B1.value <= threshold=2.6933162212371826
node_6280: feature_name=ICAM1	ICAM1.value <= threshold=3.578627824783325
node_6281: feature_name=CASP8	CASP8.value <= threshold=1.7487988471984863
node_6282: feature_name=TRAF1	TRAF1.value <= threshold=3.18716561794281

node_6283: feature_name=RNF11	RNF11.value <= threshold=1.9705576300621033
node_6284: feature_name=SNX9	SNX9.value <= threshold=3.318251371383667
node_6285: feature_name=PARP14	PARP14.value <= threshold=2.4342962503433228
node_6286: feature_name=GPX1	GPX1.value <= threshold=1.119508445262909
node_6287: feature_name=IRF8	IRF8.value <= threshold=3.6463273763656616
node_6288: feature_name=TNF	TNF.value <= threshold=1.8232123255729675
Class: Healthy Control	
Rules_15	passed counts:303
node_0: feature_name=LTB	LTB.value <= threshold=0.994031548500061
node_1: feature_name=MTRNR2L2	MTRNR2L2.value <= threshold=2.8784297704696655
node_2: feature_name=TCL1A	TCL1A.value <= threshold=4.798312664031982
node_3: feature_name=MT-CO1	MT-CO1.value > threshold=0.08947191759943962
node_2521: feature_name=LTB	LTB.value <= threshold=0.6038667261600494
node_2522: feature_name=HLA-DQB1	HLA-DQB1.value <= threshold=2.1053054332733154
node_2523: feature_name=SNX9	SNX9.value > threshold=-0.5346486568450928
node_2907: feature_name=NR4A1	NR4A1.value <= threshold=2.5433837175369263
node_2908: feature_name=XIST	XIST.value <= threshold=0.7664470989257097
node_2909: feature_name=CD79B	CD79B.value <= threshold=2.9326847791671753
node_2910: feature_name=NR4A1	NR4A1.value > threshold=-0.2875557690858841
node_3096: feature_name=NR4A3	NR4A3.value <= threshold=3.314968705177307
node_3097: feature_name=VPREB3	VPREB3.value <= threshold=3.3549938201904297
node_3098: feature_name=RALGPS2	RALGPS2.value <= threshold=5.807050704956055
node_3099: feature_name=CTD-2035E11.3	CTD-2035E11.3.value <= threshold=6.4090416431427
node_3100: feature_name=FAM129C	FAM129C.value <= threshold=6.966361045837402
node_3101: feature_name=HES4	HES4.value <= threshold=4.139557242393494
node_3102: feature_name=CD83	CD83.value <= threshold=1.8343153595924377
node_3103: feature_name=FCER2	FCER2.value <= threshold=7.255846738815308
node_3104: feature_name=MT-CYB	MT-CYB.value <= threshold=2.3888577222824097
node_3105: feature_name=RGCC	RGCC.value > threshold=-1.2040565013885498

node_3107: feature_name=MCTP1	MCTP1.value <= threshold=6.221277952194214
node_3108: feature_name=JUN	JUN.value > threshold=-0.629857987165451
node_3120: feature_name=RGS2	RGS2.value > threshold=-0.9065633714199066
node_3124: feature_name=MT1E	MT1E.value <= threshold=0.8591758906841278
node_3125: feature_name=SPI1	SPI1.value <= threshold=1.2638847827911377
Class: COVID-19 Critical	
Rules_16	passed counts:252
node_0: feature_name=LTB	LTB.value <= threshold=0.994031548500061
node_1: feature_name=MTRNR2L2	MTRNR2L2.value <= threshold=2.8784297704696655
node_2: feature_name=TCL1A	TCL1A.value <= threshold=4.798312664031982
node_3: feature_name=MT-CO1	MT-CO1.value <= threshold=0.08947191759943962
node_4: feature_name=NR4A1	NR4A1.value <= threshold=1.0725348591804504
node_5: feature_name=HLA-DPA1	HLA-DPA1.value > threshold=1.6885469555854797
node_861: feature_name=CD83	CD83.value <= threshold=3.0563642978668213
node_862: feature_name=HLA-DQB1	HLA-DQB1.value <= threshold=2.61081063747406
node_863: feature_name=MTRNR2L2	MTRNR2L2.value <= threshold=2.0919997692108154
node_864: feature_name=BCL2A1	BCL2A1.value <= threshold=1.852618932723999
node_865: feature_name=CD79B	CD79B.value <= threshold=4.352142333984375
node_866: feature_name=TNFAIP3	TNFAIP3.value <= threshold=-0.0449496041983366
node_867: feature_name=MT-CO1	MT-CO1.value <= threshold=-0.08531957119703293
node_868: feature_name=MTRNR2L8	MTRNR2L8.value > threshold=0.8770350813865662
node_1148: feature_name=NR4A1	NR4A1.value <= threshold=0.3121328055858612
node_1149: feature_name=SNX29P2	SNX29P2.value <= threshold=4.279992580413818
Class: COVID-19 Critical	
Rules_17	passed counts:250
node_0: feature_name=LTB	LTB.value <= threshold=0.994031548500061
node_1: feature_name=MTRNR2L2	MTRNR2L2.value <= threshold=2.8784297704696655
node_2: feature_name=TCL1A	TCL1A.value <= threshold=4.798312664031982

node_3: feature_name=MT-CO1	MT-CO1.value > threshold=0.08947191759943962
node_2521: feature_name=LTB	LTB.value <= threshold=0.6038667261600494
node_2522: feature_name=HLA-DQB1	HLA-DQB1.value <= threshold=2.1053054332733154
node_2523: feature_name=SNX9	SNX9.value <= threshold=-0.5346486568450928
node_2524: feature_name=HLA-DPB1	HLA-DPB1.value <= threshold=1.9548270106315613
node_2525: feature_name=FAM129C	FAM129C.value <= threshold=3.3393388986587524
node_2526: feature_name=XIST	XIST.value <= threshold=3.6911416053771973
node_2527: feature_name=NR4A1	NR4A1.value <= threshold=-0.2557755410671234
node_2528: feature_name=HOPX	HOPX.value <= threshold=2.776473879814148
node_2529: feature_name=AREGB	AREGB.value <= threshold=-0.10896047577261925
node_2530: feature_name=KIAA0125	KIAA0125.value <= threshold=7.652706623077393
node_2531: feature_name=MT-CO3	MT-CO3.value > threshold=-1.3726763725280762
node_2533: feature_name=TNF	TNF.value <= threshold=3.1101887226104736
node_2534: feature_name=NFKBID	NFKBID.value > threshold=-0.39706873893737793
node_2568: feature_name=TCF4	TCF4.value <= threshold=6.289925575256348
node_2569: feature_name=CD22	CD22.value <= threshold=8.377277374267578
node_2570: feature_name=FERMT3	FERMT3.value <= threshold=2.260508179664612
node_2571: feature_name=MT-ND6	MT-ND6.value <= threshold=3.413356304168701
node_2572: feature_name=ID1	ID1.value <= threshold=1.9121195077896118
node_2573: feature_name=DAPK1	DAPK1.value <= threshold=1.6979314014315605
node_2574: feature_name=IRF4	IRF4.value <= threshold=8.765593528747559
node_2575: feature_name=PLD4	PLD4.value <= threshold=6.6784141063690186
node_2576: feature_name=GPX1	GPX1.value > threshold=-0.9480071663856506
node_2582: feature_name=MYL9	MYL9.value <= threshold=2.9503014087677
node_2583: feature_name=ID1	ID1.value <= threshold=1.3511708974838257
node_2584: feature_name=HLA-DRB5	HLA-DRB5.value > threshold=-0.8129743039608002
node_2588: feature_name=HLA-DQA1	HLA-DQA1.value > threshold=-0.44121430814266205
Class: COVID-19 Critical	
Rules_18	passed counts:248

node_0: feature_name=LTB	LTB.value <= threshold=0.994031548500061
node_1: feature_name=MTRNR2L2	MTRNR2L2.value <= threshold=2.8784297704696655
node_2: feature_name=TCL1A	TCL1A.value <= threshold=4.798312664031982
node_3: feature_name=MT-CO1	MT-CO1.value > threshold=0.08947191759943962
node_2521: feature_name=LTB	LTB.value <= threshold=0.6038667261600494
node_2522: feature_name=HLA-DQB1	HLA-DQB1.value <= threshold=2.1053054332733154
node_2523: feature_name=SNX9	SNX9.value > threshold=-0.5346486568450928
node_2907: feature_name=NR4A1	NR4A1.value <= threshold=2.5433837175369263
node_2908: feature_name=XIST	XIST.value <= threshold=0.7664470989257097
node_2909: feature_name=CD79B	CD79B.value <= threshold=2.9326847791671753
node_2910: feature_name=NR4A1	NR4A1.value > threshold=-0.2875557690858841
node_3096: feature_name=NR4A3	NR4A3.value <= threshold=3.314968705177307
node_3097: feature_name=VPREB3	VPREB3.value <= threshold=3.3549938201904297
node_3098: feature_name=RALGPS2	RALGPS2.value <= threshold=5.807050704956055
node_3099: feature_name=CTD-2035E11.3	CTD-2035E11.3.value <= threshold=6.4090416431427
node_3100: feature_name=FAM129C	FAM129C.value <= threshold=6.966361045837402
node_3101: feature_name=HES4	HES4.value <= threshold=4.139557242393494
node_3102: feature_name=CD83	CD83.value > threshold=1.8343153595924377
node_3136: feature_name=PLD4	PLD4.value <= threshold=4.453821897506714
node_3137: feature_name=HLA-DQA2	HLA-DQA2.value <= threshold=-0.10758286714553833
node_3138: feature_name=STAG3	STAG3.value <= threshold=7.365770578384399
node_3139: feature_name=LDLR	LDLR.value <= threshold=2.3423796892166138
node_3140: feature_name=CD79A	CD79A.value <= threshold=4.038331866264343
node_3141: feature_name=DUSP4	DUSP4.value <= threshold=4.829863548278809
node_3142: feature_name=OAZ1	OAZ1.value <= threshold=-0.34704728424549103
node_3143: feature_name=CSF3R	CSF3R.value <= threshold=1.0170481204986572
node_3144: feature_name=IGLL5	IGLL5.value <= threshold=6.979742765426636
node_3145: feature_name=ANO6	ANO6.value <= threshold=4.378111124038696
node_3146: feature_name=NAMPT	NAMPT.value <= threshold=3.490760326385498
node_3147: feature_name=RP11-731F5.2	RP11-731F5.2.value <= threshold=8.205055236816406

node_3148: feature_name=COTL1	COTL1.value > threshold=-1.5100116729736328
node_3152: feature_name=LTB	LTB.value > threshold=-1.0809237957000732
node_3156: feature_name=BOD1L1	BOD1L1.value <= threshold=4.357234954833984
node_3157: feature_name=DUSP4	DUSP4.value <= threshold=4.34939432144165
node_3158: feature_name=TTN	TTN.value <= threshold=7.321672439575195
Class: COVID-19 Critical	
Rules_19	passed counts:248
node_0: feature_name=LTB	LTB.value <= threshold=0.994031548500061
node_1: feature_name=MTRNR2L2	MTRNR2L2.value <= threshold=2.8784297704696655
node_2: feature_name=TCL1A	TCL1A.value <= threshold=4.798312664031982
node_3: feature_name=MT-CO1	MT-CO1.value <= threshold=0.08947191759943962
node_4: feature_name=NR4A1	NR4A1.value <= threshold=1.0725348591804504
node_5: feature_name=HLA-DPA1	HLA-DPA1.value > threshold=1.6885469555854797
node_861: feature_name=CD83	CD83.value <= threshold=3.0563642978668213
node_862: feature_name=HLA-DQB1	HLA-DQB1.value > threshold=2.61081063747406
node_1358: feature_name=HLA-DRB5	HLA-DRB5.value > threshold=0.7542744874954224
node_1390: feature_name=HLA-DRB5	HLA-DRB5.value <= threshold=2.6244447231292725
node_1391: feature_name=XIST	XIST.value <= threshold=-0.22017984092235565
node_1392: feature_name=MT-CO3	MT-CO3.value <= threshold=0.12647542357444763
node_1393: feature_name=CD79B	CD79B.value <= threshold=4.338301658630371
node_1394: feature_name=LTB	LTB.value <= threshold=0.9865012466907501
node_1395: feature_name=MEF2C	MEF2C.value <= threshold=4.133931398391724
node_1396: feature_name=MEF2C	MEF2C.value > threshold=-0.38076959550380707
node_1418: feature_name=HLA-DPB1	HLA-DPB1.value > threshold=0.8961269855499268
node_1422: feature_name=HLA-DPA1	HLA-DPA1.value <= threshold=2.9194568395614624
node_1423: feature_name=IL1B	IL1B.value <= threshold=2.321333408355713
node_1424: feature_name=TRIB1	TRIB1.value <= threshold=4.043357253074646
node_1425: feature_name=NT5C3A	NT5C3A.value <= threshold=3.4580591917037964
node_1426: feature_name=MT1E	MT1E.value <= threshold=1.7696760445833206

node_1427: feature_name=SESN3	SESN3.value <= threshold=3.1229121685028076
node_1428: feature_name=HLA-DRB1	HLA-DRB1.value <= threshold=2.7549219131469727
node_1429: feature_name=OASL	OASL.value <= threshold=3.688907504081726
node_1430: feature_name=ITM2C	ITM2C.value <= threshold=3.4028767347335815
node_1431: feature_name=HLA-DRB1	HLA-DRB1.value > threshold=0.7038701176643372
node_1435: feature_name=ATF5	ATF5.value <= threshold=3.428827404975891
node_1436: feature_name=DAPP1	DAPP1.value <= threshold=5.408607244491577
node_1437: feature_name=PDLIM1	PDLIM1.value <= threshold=4.337203502655029
Class: COVID-19 Critical	
Rules_20	passed counts:241
node_0: feature_name=LTB	LTB.value <= threshold=0.994031548500061
node_1: feature_name=MTRNR2L2	MTRNR2L2.value <= threshold=2.8784297704696655
node_2: feature_name=TCL1A	TCL1A.value <= threshold=4.798312664031982
node_3: feature_name=MT-CO1	MT-CO1.value > threshold=0.08947191759943962
node_2521: feature_name=LTB	LTB.value > threshold=0.6038667261600494
node_4173: feature_name=OAZ1	OAZ1.value > threshold=0.143283873796463
node_4615: feature_name=NR4A1	NR4A1.value > threshold=0.6621004343032837
node_4817: feature_name=HSP90B1	HSP90B1.value <= threshold=1.1699737310409546
node_4818: feature_name=XAF1	XAF1.value <= threshold=2.6752071380615234
node_4819: feature_name=SNX9	SNX9.value <= threshold=2.939147114753723
node_4820: feature_name=HLA-DQA2	HLA-DQA2.value <= threshold=2.9822239875793457
node_4821: feature_name=ATF5	ATF5.value <= threshold=4.116653323173523
node_4822: feature_name=RALGPS2	RALGPS2.value > threshold=-0.3627814054489136
node_4834: feature_name=CD79B	CD79B.value > threshold=-0.4220905303955078
node_4838: feature_name=MIAT	MIAT.value <= threshold=2.609323263168335
node_4839: feature_name=YWHAH	YWHAH.value <= threshold=4.648661851882935
node_4840: feature_name=HLA-DRB5	HLA-DRB5.value > threshold=-0.8319411873817444
node_4842: feature_name=MX1	MX1.value <= threshold=3.420834183692932
node_4843: feature_name=SLC7A5	SLC7A5.value <= threshold=2.6673996448516846

node_4844: feature_name=PTGS1	PTGS1.value <= threshold=5.0393829345703125
node_4845: feature_name=ATG2A	ATG2A.value <= threshold=2.2149808406829834
node_4846: feature_name=MT-CO2	MT-CO2.value > threshold=-0.6727012395858765
node_4850: feature_name=AHR	AHR.value <= threshold=1.3810421824455261
node_4851: feature_name=CASP8	CASP8.value <= threshold=1.853389322757721
node_4852: feature_name=MT-ND6	MT-ND6.value > threshold=-0.9859366714954376
Class: Healthy Control	
Rules_21	passed counts:241
node_0: feature_name=LTB	LTB.value <= threshold=0.994031548500061
node_1: feature_name=MTRNR2L2	MTRNR2L2.value <= threshold=2.8784297704696655
node_2: feature_name=TCL1A	TCL1A.value <= threshold=4.798312664031982
node_3: feature_name=MT-CO1	MT-CO1.value <= threshold=0.08947191759943962
node_4: feature_name=NR4A1	NR4A1.value <= threshold=1.0725348591804504
node_5: feature_name=HLA-DPA1	HLA-DPA1.value <= threshold=1.6885469555854797
node_6: feature_name=HLA-DRB5	HLA-DRB5.value <= threshold=2.1886194944381714
node_7: feature_name=XIST	XIST.value <= threshold=-0.12328458577394485
node_8: feature_name=SYNE1	SYNE1.value > threshold=-0.7835594415664673
node_16: feature_name=MTRNR2L2	MTRNR2L2.value <= threshold=2.4483247995376587
node_17: feature_name=HLA-DQA2	HLA-DQA2.value <= threshold=2.227178931236267
node_18: feature_name=NR4A1	NR4A1.value > threshold=-0.21380723267793655
node_504: feature_name=LTB	LTB.value <= threshold=0.8758118152618408
node_505: feature_name=TNFAIP3	TNFAIP3.value <= threshold=0.4779366999864578
node_506: feature_name=HLA-DQB1	HLA-DQB1.value <= threshold=2.9689468145370483
node_507: feature_name=MS4A1	MS4A1.value <= threshold=3.7428637742996216
node_508: feature_name=SNX9	SNX9.value <= threshold=3.304862380027771
node_509: feature_name=MARCKS	MARCKS.value > threshold=-0.519626647233963
node_513: feature_name=CD79B	CD79B.value <= threshold=3.0890846252441406
node_514: feature_name=GNG11	GNG11.value <= threshold=3.3202054500579834
node_515: feature_name=BANK1	BANK1.value > threshold=-0.3250492364168167

node_521: feature_name=VPREB3	VPREB3.value <= threshold=8.097821950912476
node_522: feature_name=IRF4	IRF4.value <= threshold=6.5894060134887695
node_523: feature_name=BCL2A1	BCL2A1.value <= threshold=2.7336734533309937
node_524: feature_name=HSPA1A	HSPA1A.value <= threshold=3.192288041114807
node_525: feature_name=MS4A1	MS4A1.value > threshold=-0.3445378392934799
node_529: feature_name=MS4A1	MS4A1.value <= threshold=3.4282803535461426
node_530: feature_name=NR4A3	NR4A3.value <= threshold=2.2900307178497314
node_531: feature_name=OAZ1	OAZ1.value <= threshold=0.8763319551944733
node_532: feature_name=JUN	JUN.value > threshold=-1.1919074058532715
node_536: feature_name=HOPX	HOPX.value <= threshold=1.6209625601768494
node_537: feature_name=IL1B	IL1B.value <= threshold=0.27510731667280197
Class: COVID-19 Critical	
Rules_22	passed counts:237
node_0: feature_name=LTB	LTB.value <= threshold=0.994031548500061
node_1: feature_name=MTRNR2L2	MTRNR2L2.value <= threshold=2.8784297704696655
node_2: feature_name=TCL1A	TCL1A.value <= threshold=4.798312664031982
node_3: feature_name=MT-CO1	MT-CO1.value > threshold=0.08947191759943962
node_2521: feature_name=LTB	LTB.value > threshold=0.6038667261600494
node_4173: feature_name=OAZ1	OAZ1.value <= threshold=0.143283873796463
node_4174: feature_name=PLD4	PLD4.value <= threshold=2.921932101249695
node_4175: feature_name=XIST	XIST.value <= threshold=-0.05067425221204758
node_4176: feature_name=MT-CO1	MT-CO1.value <= threshold=1.345594048500061
node_4177: feature_name=HLA-DQB1	HLA-DQB1.value <= threshold=1.927736520767212
node_4178: feature_name=BACH1	BACH1.value > threshold=-0.5349778532981873
node_4236: feature_name=TPM1	TPM1.value <= threshold=3.516477584838867
node_4237: feature_name=NR4A1	NR4A1.value <= threshold=-0.274935707449913
node_4238: feature_name=VPREB3	VPREB3.value <= threshold=5.024149179458618
node_4239: feature_name=YWHAH	YWHAH.value <= threshold=4.7504096031188965
node_4240: feature_name=NAIP	NAIP.value <= threshold=4.030007481575012

node_4241: feature_name=CD38	CD38.value > threshold=-0.3387429714202881
node_4243: feature_name=MS4A1	MS4A1.value <= threshold=5.195746660232544
node_4244: feature_name=TRIB1	TRIB1.value <= threshold=2.9039160013198853
node_4245: feature_name=RGS2	RGS2.value > threshold=-0.8980970978736877
node_4249: feature_name=PLD4	PLD4.value <= threshold=2.8284080028533936
node_4250: feature_name=GRAP2	GRAP2.value <= threshold=2.0700820088386536
node_4251: feature_name=ATF3	ATF3.value <= threshold=2.327788233757019
node_4252: feature_name=ITM2C	ITM2C.value <= threshold=3.576403021812439
Class: COVID-19 Critical	
Rules_23	passed counts:221
node_0: feature_name=LTB	LTB.value > threshold=0.994031548500061
node_5264: feature_name=MT-CO1	MT-CO1.value > threshold=-0.4127991199493408
node_5638: feature_name=OAZ1	OAZ1.value > threshold=-0.2640867084264755
node_6112: feature_name=IFI44L	IFI44L.value <= threshold=2.5893263816833496
node_6113: feature_name=TCL1A	TCL1A.value <= threshold=4.201832294464111
node_6114: feature_name=HLA-DQA2	HLA-DQA2.value > threshold=-0.2270243540406227
node_6542: feature_name=CD79B	CD79B.value > threshold=3.096232771873474
node_6872: feature_name=ICAM1	ICAM1.value <= threshold=-0.2232399433851242
node_6873: feature_name=STAG3	STAG3.value <= threshold=-0.19312061369419098
node_6874: feature_name=CD79A	CD79A.value > threshold=1.57558012008667
node_6880: feature_name=MEF2A	MEF2A.value <= threshold=2.676826596260071
node_6881: feature_name=MTRNR2L2	MTRNR2L2.value <= threshold=2.9562686681747437
node_6882: feature_name=SNX9	SNX9.value <= threshold=3.162803888320923
node_6883: feature_name=MARCKS	MARCKS.value <= threshold=4.3125433921813965
node_6884: feature_name=MX1	MX1.value <= threshold=2.1828908920288086
node_6885: feature_name=ATF5	ATF5.value <= threshold=3.838430166244507
node_6886: feature_name=PELI1	PELI1.value <= threshold=1.8930315971374512
node_6887: feature_name=BCL2A1	BCL2A1.value <= threshold=2.259818911552429
node_6888: feature_name=LDLR	LDLR.value <= threshold=0.9009743332862854

node_6889: feature_name=CD38	CD38.value <= threshold=3.9168299436569214
node_6890: feature_name=NFKBID	NFKBID.value <= threshold=3.940497040748596
node_6891: feature_name=HLA-DQA2	HLA-DQA2.value > threshold=-0.22470510751008987
node_6895: feature_name=GRASP	GRASP.value <= threshold=3.424399256706238
Class: Healthy Control	
Rules_24	passed counts:216
node_0: feature_name=LTB	LTB.value <= threshold=0.994031548500061
node_1: feature_name=MTRNR2L2	MTRNR2L2.value <= threshold=2.8784297704696655
node_2: feature_name=TCL1A	TCL1A.value <= threshold=4.798312664031982
node_3: feature_name=MT-CO1	MT-CO1.value <= threshold=0.08947191759943962
node_4: feature_name=NR4A1	NR4A1.value > threshold=1.0725348591804504
node_1622: feature_name=HLA-DQA2	HLA-DQA2.value <= threshold=1.9496816992759705
node_1623: feature_name=HLA-DRB5	HLA-DRB5.value <= threshold=2.715766429901123
node_1624: feature_name=HLA-DPA1	HLA-DPA1.value <= threshold=1.7050301432609558
node_1625: feature_name=CD83	CD83.value <= threshold=2.783489227294922
node_1626: feature_name=XIST	XIST.value <= threshold=0.9589543007314205
node_1627: feature_name=PLD4	PLD4.value <= threshold=5.865932464599609
node_1628: feature_name=OAZ1	OAZ1.value <= threshold=0.6137169003486633
node_1629: feature_name=MTRNR2L2	MTRNR2L2.value <= threshold=1.6102792620658875
node_1630: feature_name=IFI6	IFI6.value > threshold=-0.8404486179351807
node_1632: feature_name=NR4A1	NR4A1.value > threshold=1.0916320085525513
node_1638: feature_name=DENND3	DENND3.value <= threshold=3.988452672958374
node_1639: feature_name=HLA-DRB1	HLA-DRB1.value <= threshold=1.4092575907707214
node_1640: feature_name=HLA-DPA1	HLA-DPA1.value <= threshold=1.2675696015357971
node_1641: feature_name=TRAF1	TRAF1.value <= threshold=2.918454885482788
node_1642: feature_name=HLA-DPA1	HLA-DPA1.value > threshold=-0.917132705450058
node_1648: feature_name=LTB	LTB.value <= threshold=0.9716593027114868
node_1649: feature_name=LDLRAD4	LDLRAD4.value <= threshold=4.4251909255981445
node_1650: feature_name=BCL2A1	BCL2A1.value > threshold=-0.7959703803062439

node_1652: feature_name=PRDM1	PRDM1.value <= threshold=2.6821553707122803
node_1653: feature_name=FAM129C	FAM129C.value <= threshold=4.9084858894348145
node_1654: feature_name=BOD1L1	BOD1L1.value <= threshold=2.9830023050308228
node_1655: feature_name=HSP90B1	HSP90B1.value <= threshold=2.581766724586487
node_1656: feature_name=HLA-DQB1	HLA-DQB1.value <= threshold=3.3723777532577515
node_1657: feature_name=ANO6	ANO6.value <= threshold=2.491254210472107
node_1658: feature_name=RP5-821D11.7	RP5-821D11.7.value <= threshold=3.2165164947509766
node_1659: feature_name=MARCH1	MARCH1.value <= threshold=4.2638514041900635
node_1660: feature_name=ABCA1	ABCA1.value <= threshold=3.618377923965454
node_1661: feature_name=LTB	LTB.value <= threshold=0.8862588405609131
node_1662: feature_name=SNORD3B-2	SNORD3B-2.value <= threshold=4.273592233657837
node_1663: feature_name=S100A6	S100A6.value <= threshold=0.29081472009420395
node_1664: feature_name=IGLL5	IGLL5.value <= threshold=6.102234125137329
node_1665: feature_name=STAG3	STAG3.value <= threshold=6.671069860458374
node_1666: feature_name=HLA-DQA1	HLA-DQA1.value > threshold=-0.5025019943714142
Class: COVID-19 Critical	
Rules_25	passed counts:213
node_0: feature_name=LTB	LTB.value <= threshold=0.994031548500061
node_1: feature_name=MTRNR2L2	MTRNR2L2.value <= threshold=2.8784297704696655
node_2: feature_name=TCL1A	TCL1A.value <= threshold=4.798312664031982
node_3: feature_name=MT-CO1	MT-CO1.value <= threshold=0.08947191759943962
node_4: feature_name=NR4A1	NR4A1.value <= threshold=1.0725348591804504
node_5: feature_name=HLA-DPA1	HLA-DPA1.value > threshold=1.6885469555854797
node_861: feature_name=CD83	CD83.value <= threshold=3.0563642978668213
node_862: feature_name=HLA-DQB1	HLA-DQB1.value <= threshold=2.61081063747406
node_863: feature_name=MTRNR2L2	MTRNR2L2.value <= threshold=2.0919997692108154
node_864: feature_name=BCL2A1	BCL2A1.value <= threshold=1.852618932723999
node_865: feature_name=CD79B	CD79B.value <= threshold=4.352142333984375
node_866: feature_name=TNFAIP3	TNFAIP3.value <= threshold=-0.0449496041983366

node_867: feature_name=MT-CO1	MT-CO1.value <= threshold=-0.08531957119703293
node_868: feature_name=MTRNR2L8	MTRNR2L8.value <= threshold=0.8770350813865662
node_869: feature_name=EGR1	EGR1.value > threshold=-0.32270750403404236
node_1015: feature_name=NR4A3	NR4A3.value <= threshold=1.9085431098937988
node_1016: feature_name=ID3	ID3.value <= threshold=2.776647686958313
node_1017: feature_name=XIST	XIST.value <= threshold=2.618046522140503
node_1018: feature_name=CD79B	CD79B.value <= threshold=3.818994164466858
node_1019: feature_name=NFKBID	NFKBID.value <= threshold=3.4191306829452515
node_1020: feature_name=MIAT	MIAT.value <= threshold=3.532225251197815
node_1021: feature_name=STAG3	STAG3.value <= threshold=9.534917831420898
node_1022: feature_name=OASL	OASL.value <= threshold=2.3923760652542114
node_1023: feature_name=HLA-DQB1	HLA-DQB1.value <= threshold=2.572031259536743
node_1024: feature_name=CD79A	CD79A.value > threshold=0.1879405975341797
node_1028: feature_name=NR4A1	NR4A1.value <= threshold=0.7387348711490631
node_1029: feature_name=SLC7A5	SLC7A5.value <= threshold=2.0677570104599
node_1030: feature_name=HLA-DPB1	HLA-DPB1.value <= threshold=2.17708420753479
node_1031: feature_name=MARCH1	MARCH1.value <= threshold=4.443183898925781
node_1032: feature_name=ATF5	ATF5.value <= threshold=6.411617755889893
node_1033: feature_name=MYL9	MYL9.value <= threshold=3.8704605102539062
node_1034: feature_name=LTB	LTB.value <= threshold=0.9670123755931854
node_1035: feature_name=PLD4	PLD4.value <= threshold=7.310958623886108
node_1036: feature_name=CTD-2035E11.3	CTD-2035E11.3.value <= threshold=2.9847536087036133
node_1037: feature_name=PDLIM1	PDLIM1.value > threshold=-0.278710201382637
node_1051: feature_name=MTRNR2L2	MTRNR2L2.value <= threshold=1.1439209580421448
Class: COVID-19 Critical	
Rules_26	passed counts:208
node_0: feature_name=LTB	LTB.value <= threshold=0.994031548500061
node_1: feature_name=MTRNR2L2	MTRNR2L2.value <= threshold=2.8784297704696655
node_2: feature_name=TCL1A	TCL1A.value > threshold=4.798312664031982

node_4912: feature_name=MT-CO1	MT-CO1.value <= threshold=-0.3664844036102295
node_4913: feature_name=CD79A	CD79A.value <= threshold=2.987208843231201
node_4914: feature_name=HLA-DPB1	HLA-DPB1.value <= threshold=2.0926655530929565
node_4915: feature_name=MX1	MX1.value > threshold=-0.4660506546497345
node_4921: feature_name=NR4A1	NR4A1.value <= threshold=1.2700347900390625
node_4922: feature_name=CD79B	CD79B.value <= threshold=5.17732834815979
node_4923: feature_name=HLA-DRB5	HLA-DRB5.value <= threshold=2.903197169303894
node_4924: feature_name=FTH1	FTH1.value > threshold=-1.6512951254844666
node_4926: feature_name=CCL3	CCL3.value <= threshold=4.140803575515747
node_4927: feature_name=MTRNR2L2	MTRNR2L2.value <= threshold=2.1290477514266968
node_4928: feature_name=SLC7A5	SLC7A5.value <= threshold=1.9653798341751099
node_4929: feature_name=GRASP	GRASP.value <= threshold=4.255978584289551
node_4930: feature_name=SLC11A1	SLC11A1.value <= threshold=1.3972728252410889
node_4931: feature_name=CXorf57	CXorf57.value <= threshold=4.044520854949951
Class: COVID-19 Critical	
Rules_27	passed counts:195
node_0: feature_name=LTB	LTB.value <= threshold=0.994031548500061
node_1: feature_name=MTRNR2L2	MTRNR2L2.value <= threshold=2.8784297704696655
node_2: feature_name=TCL1A	TCL1A.value <= threshold=4.798312664031982
node_3: feature_name=MT-CO1	MT-CO1.value > threshold=0.08947191759943962
node_2521: feature_name=LTB	LTB.value <= threshold=0.6038667261600494
node_2522: feature_name=HLA-DQB1	HLA-DQB1.value > threshold=2.1053054332733154
node_3550: feature_name=XIST	XIST.value <= threshold=0.7716337237507105
node_3551: feature_name=CD79B	CD79B.value <= threshold=2.9426796436309814
node_3552: feature_name=HLA-DQB1	HLA-DQB1.value <= threshold=2.713674306869507
node_3553: feature_name=SNX9	SNX9.value > threshold=-0.5404067635536194
node_3653: feature_name=LTB	LTB.value <= threshold=0.021708179265260696
node_3654: feature_name=HLA-DRB5	HLA-DRB5.value > threshold=-0.5633223652839661
node_3660: feature_name=NR4A1	NR4A1.value <= threshold=2.2327845096588135

node_3661: feature_name=FCRL1	FCRL1.value <= threshold=8.662389755249023
node_3662: feature_name=BANK1	BANK1.value <= threshold=4.776419401168823
node_3663: feature_name=IGLL5	IGLL5.value <= threshold=1.4806077480316162
node_3664: feature_name=COTL1	COTL1.value > threshold=-1.2335613369941711
node_3670: feature_name=VCL	VCL.value <= threshold=4.393002271652222
node_3671: feature_name=HLA-DQA1	HLA-DQA1.value > threshold=-0.3948168456554413
node_3673: feature_name=HLA-DRB1	HLA-DRB1.value > threshold=-0.6657836139202118
node_3677: feature_name=GNG11	GNG11.value <= threshold=3.942102313041687
node_3678: feature_name=MT-CO2	MT-CO2.value > threshold=0.10530227422714233
node_3692: feature_name=PLA2G12A	PLA2G12A.value <= threshold=1.5832663029432297
node_3693: feature_name=ABCA1	ABCA1.value <= threshold=5.887498140335083
Class: COVID-19 Critical	
Rules_28	passed counts:189
node_0: feature_name=LTB	LTB.value <= threshold=0.994031548500061
node_1: feature_name=MTRNR2L2	MTRNR2L2.value <= threshold=2.8784297704696655
node_2: feature_name=TCL1A	TCL1A.value <= threshold=4.798312664031982
node_3: feature_name=MT-CO1	MT-CO1.value <= threshold=0.08947191759943962
node_4: feature_name=NR4A1	NR4A1.value > threshold=1.0725348591804504
node_1622: feature_name=HLA-DQA2	HLA-DQA2.value <= threshold=1.9496816992759705
node_1623: feature_name=HLA-DRB5	HLA-DRB5.value <= threshold=2.715766429901123
node_1624: feature_name=HLA-DPA1	HLA-DPA1.value > threshold=1.7050301432609558
node_2056: feature_name=OAZ1	OAZ1.value <= threshold=0.456103190779686
node_2057: feature_name=HLA-DRB5	HLA-DRB5.value > threshold=0.6839984953403473
node_2101: feature_name=HLA-DRB1	HLA-DRB1.value > threshold=1.6199676990509033
node_2151: feature_name=HLA-DQB1	HLA-DQB1.value <= threshold=3.1961567401885986
node_2152: feature_name=PLD4	PLD4.value <= threshold=-0.03259922005236149
node_2153: feature_name=XIST	XIST.value <= threshold=-0.138966154307127
node_2154: feature_name=HLA-DQA1	HLA-DQA1.value > threshold=2.0848299264907837
node_2190: feature_name=RALGPS2	RALGPS2.value <= threshold=4.276920795440674

node_2191: feature_name=RGS2	RGS2.value > threshold=-0.7087429165840149
node_2195: feature_name=GRAP2	GRAP2.value <= threshold=2.2513980865478516
node_2196: feature_name=ID3	ID3.value <= threshold=5.227530002593994
node_2197: feature_name=CD79B	CD79B.value <= threshold=4.226724624633789
node_2198: feature_name=ENDOD1	ENDOD1.value <= threshold=4.877119541168213
node_2199: feature_name=AP3M2	AP3M2.value <= threshold=2.6568219661712646
node_2200: feature_name=ICAM1	ICAM1.value <= threshold=4.384357690811157
node_2201: feature_name=ILK	ILK.value <= threshold=2.051138460636139
node_2202: feature_name=DUSP4	DUSP4.value <= threshold=5.8240966796875
node_2203: feature_name=NR4A1	NR4A1.value > threshold=1.132164180278778
node_2205: feature_name=MEF2C	MEF2C.value <= threshold=4.1522674560546875
node_2206: feature_name=PLA2G12A	PLA2G12A.value <= threshold=1.7114491239190102
node_2207: feature_name=SNX29P2	SNX29P2.value <= threshold=5.11986231803894
node_2208: feature_name=ADAM19	ADAM19.value <= threshold=3.4296538829803467
node_2209: feature_name=PPIF	PPIF.value <= threshold=2.486689567565918
Class: COVID-19 Critical	
Rules_29	passed counts:189
node_0: feature_name=LTB	LTB.value <= threshold=0.994031548500061
node_1: feature_name=MTRNR2L2	MTRNR2L2.value <= threshold=2.8784297704696655
node_2: feature_name=TCL1A	TCL1A.value <= threshold=4.798312664031982
node_3: feature_name=MT-CO1	MT-CO1.value <= threshold=0.08947191759943962
node_4: feature_name=NR4A1	NR4A1.value <= threshold=1.0725348591804504
node_5: feature_name=HLA-DPA1	HLA-DPA1.value <= threshold=1.6885469555854797
node_6: feature_name=HLA-DRB5	HLA-DRB5.value > threshold=2.1886194944381714
node_738: feature_name=HLA-DQA2	HLA-DQA2.value > threshold=-0.20105575770139694
node_804: feature_name=CYBB	CYBB.value <= threshold=2.9090086221694946
node_805: feature_name=VPREB3	VPREB3.value <= threshold=7.757248401641846
node_806: feature_name=IL8	IL8.value <= threshold=1.7155421376228333
node_807: feature_name=ATF5	ATF5.value <= threshold=6.124105930328369

node_808: feature_name=RP11-731F5.2	RP11-731F5.2.value <= threshold=7.633600234985352
node_809: feature_name=OASL	OASL.value <= threshold=0.9932635426521301
node_810: feature_name=ID1	ID1.value <= threshold=2.8093127012252808
node_811: feature_name=GRASP	GRASP.value <= threshold=2.3934966325759888
node_812: feature_name=KIAA0125	KIAA0125.value <= threshold=9.68092966079712
node_813: feature_name=MTRNR2L2	MTRNR2L2.value <= threshold=1.901122272014618
node_814: feature_name=HLA-DPA1	HLA-DPA1.value > threshold=-0.7204518914222717
node_816: feature_name=NT5C3A	NT5C3A.value <= threshold=4.120891809463501
node_817: feature_name=IRAK3	IRAK3.value <= threshold=4.327898621559143
node_818: feature_name=PLA2G12A	PLA2G12A.value <= threshold=4.008805990219116
node_819: feature_name=CD79A	CD79A.value > threshold=1.521829903125763
Class: COVID-19 Critical	
Rules_30	passed counts:175
node_0: feature_name=LTB	LTB.value <= threshold=0.994031548500061
node_1: feature_name=MTRNR2L2	MTRNR2L2.value <= threshold=2.8784297704696655
node_2: feature_name=TCL1A	TCL1A.value > threshold=4.798312664031982
node_4912: feature_name=MT-CO1	MT-CO1.value > threshold=-0.3664844036102295
node_5002: feature_name=IFI44L	IFI44L.value <= threshold=2.3101747035980225
node_5003: feature_name=SNX9	SNX9.value <= threshold=2.0247421264648438
node_5004: feature_name=STAG3	STAG3.value <= threshold=6.482141733169556
node_5005: feature_name=BACH1	BACH1.value > threshold=-0.5237349569797516
node_5047: feature_name=RHOB	RHOB.value > threshold=-0.4843696355819702
node_5055: feature_name=FAM129C	FAM129C.value > threshold=-0.2832885980606079
node_5079: feature_name=TP53INP1	TP53INP1.value <= threshold=2.9482468366622925
node_5080: feature_name=HLA-DRB5	HLA-DRB5.value <= threshold=2.6279215812683105
node_5081: feature_name=IFI6	IFI6.value <= threshold=1.4884688258171082
node_5082: feature_name=MARCKS	MARCKS.value <= threshold=3.0851633548736572
node_5083: feature_name=JUN	JUN.value <= threshold=1.6659854054450989
node_5084: feature_name=MT-ND6	MT-ND6.value <= threshold=2.70896577835083

node_5085: feature_name=NAIP	NAIP.value <= threshold=3.280133605003357
node_5086: feature_name=LDLR	LDLR.value <= threshold=2.3318628072738647
node_5087: feature_name=RP11-731F5.2	RP11-731F5.2.value <= threshold=5.905111789703369
node_5088: feature_name=ATF5	ATF5.value <= threshold=3.1376168727874756
node_5089: feature_name=IFIT3	IFIT3.value <= threshold=3.4996854066848755
node_5090: feature_name=CCDC141	CCDC141.value <= threshold=5.474728345870972
node_5091: feature_name=HLA-DQB1	HLA-DQB1.value > threshold=-0.5577107667922974
node_5095: feature_name=SNORD3B-2	SNORD3B-2.value <= threshold=4.051119565963745
node_5096: feature_name=IGLL5	IGLL5.value <= threshold=6.897646188735962
node_5097: feature_name=SESN3	SESN3.value <= threshold=2.8603135347366333
Class: Healthy Control	
Rules_31	passed counts:159
node_0: feature_name=LTB	LTB.value <= threshold=0.994031548500061
node_1: feature_name=MTRNR2L2	MTRNR2L2.value <= threshold=2.8784297704696655
node_2: feature_name=TCL1A	TCL1A.value <= threshold=4.798312664031982
node_3: feature_name=MT-CO1	MT-CO1.value <= threshold=0.08947191759943962
node_4: feature_name=NR4A1	NR4A1.value <= threshold=1.0725348591804504
node_5: feature_name=HLA-DPA1	HLA-DPA1.value > threshold=1.6885469555854797
node_861: feature_name=CD83	CD83.value <= threshold=3.0563642978668213
node_862: feature_name=HLA-DQB1	HLA-DQB1.value <= threshold=2.61081063747406
node_863: feature_name=MTRNR2L2	MTRNR2L2.value <= threshold=2.0919997692108154
node_864: feature_name=BCL2A1	BCL2A1.value <= threshold=1.852618932723999
node_865: feature_name=CD79B	CD79B.value <= threshold=4.352142333984375
node_866: feature_name=TNFAIP3	TNFAIP3.value <= threshold=-0.0449496041983366
node_867: feature_name=MT-CO1	MT-CO1.value > threshold=-0.08531957119703293
node_1155: feature_name=IRF8	IRF8.value > threshold=-0.39239931106567383
node_1177: feature_name=PELI1	PELI1.value > threshold=-0.6234017610549927
node_1203: feature_name=STMN1	STMN1.value <= threshold=-0.2129739671945572
node_1204: feature_name=HLA-DPA1	HLA-DPA1.value > threshold=1.6937929391860962

node_1210: feature_name=MT-CO2	MT-CO2.value > threshold=-0.5078963339328766
node_1218: feature_name=KIAA0125	KIAA0125.value <= threshold=7.932265043258667
node_1219: feature_name=CD83	CD83.value <= threshold=3.0211856365203857
node_1220: feature_name=CXorf57	CXorf57.value <= threshold=4.241424322128296
node_1221: feature_name=VCL	VCL.value <= threshold=2.1459813117980957
Class: COVID-19 Critical	
Rules_32	passed counts:153
node_0: feature_name=LTB	LTB.value <= threshold=0.994031548500061
node_1: feature_name=MTRNR2L2	MTRNR2L2.value <= threshold=2.8784297704696655
node_2: feature_name=TCL1A	TCL1A.value <= threshold=4.798312664031982
node_3: feature_name=MT-CO1	MT-CO1.value <= threshold=0.08947191759943962
node_4: feature_name=NR4A1	NR4A1.value > threshold=1.0725348591804504
node_1622: feature_name=HLA-DQA2	HLA-DQA2.value <= threshold=1.9496816992759705
node_1623: feature_name=HLA-DRB5	HLA-DRB5.value <= threshold=2.715766429901123
node_1624: feature_name=HLA-DPA1	HLA-DPA1.value <= threshold=1.7050301432609558
node_1625: feature_name=CD83	CD83.value > threshold=2.783489227294922
node_1873: feature_name=IFI44L	IFI44L.value <= threshold=1.5938722491264343
node_1874: feature_name=PLD4	PLD4.value <= threshold=4.101001739501953
node_1875: feature_name=RHOB	RHOB.value <= threshold=1.304835319519043
node_1876: feature_name=PPIF	PPIF.value <= threshold=2.2943923473358154
node_1877: feature_name=OAZ1	OAZ1.value <= threshold=0.04260866530239582
node_1878: feature_name=ID3	ID3.value <= threshold=5.290210247039795
node_1879: feature_name=NFKBID	NFKBID.value > threshold=-0.40532417595386505
node_1883: feature_name=MEF2C	MEF2C.value > threshold=-0.4597593992948532
node_1887: feature_name=SNX9	SNX9.value > threshold=1.010923683643341
node_1901: feature_name=RBKS	RBKS.value <= threshold=4.642704963684082
node_1902: feature_name=TSPYL2	TSPYL2.value <= threshold=2.4923402070999146
node_1903: feature_name=MARCKS	MARCKS.value <= threshold=4.944992542266846
node_1904: feature_name=RELT	RELT.value <= threshold=2.6679846048355103

node_1905: feature_name=TCL1A	TCL1A.value <= threshold=4.318726301193237
node_1906: feature_name=ID1	ID1.value <= threshold=4.261367559432983
node_1907: feature_name=IGJ	IGJ.value > threshold=-0.3538896441459656
node_1915: feature_name=HLA-DRB5	HLA-DRB5.value <= threshold=2.2267305850982666
Class: COVID-19 Critical	
Rules_33	passed counts:152
node_0: feature_name=LTB	LTB.value > threshold=0.994031548500061
node_5264: feature_name=MT-CO1	MT-CO1.value > threshold=-0.4127991199493408
node_5638: feature_name=OAZ1	OAZ1.value <= threshold=-0.2640867084264755
node_5639: feature_name=CD79B	CD79B.value <= threshold=2.2998517751693726
node_5640: feature_name=MTRNR2L2	MTRNR2L2.value <= threshold=3.5499863624572754
node_5641: feature_name=MT-CO1	MT-CO1.value <= threshold=0.22567133605480194
node_5642: feature_name=RGS2	RGS2.value <= threshold=1.2649686336517334
node_5643: feature_name=TCL1A	TCL1A.value <= threshold=4.771023273468018
node_5644: feature_name=IGJ	IGJ.value <= threshold=3.91206431388855
node_5645: feature_name=AREGB	AREGB.value <= threshold=1.5142876282334328
node_5646: feature_name=HLA-DRB1	HLA-DRB1.value > threshold=-0.6253005266189575
node_5648: feature_name=ITM2C	ITM2C.value <= threshold=3.2904402017593384
node_5649: feature_name=PLA2G12A	PLA2G12A.value <= threshold=3.833786725997925
node_5650: feature_name=RGCC	RGCC.value <= threshold=-0.10016544535756111
node_5651: feature_name=CD79B	CD79B.value <= threshold=2.278481364250183
node_5652: feature_name=HIST1H2AC	HIST1H2AC.value <= threshold=3.4233893156051636
node_5653: feature_name=RHOB	RHOB.value <= threshold=4.956467151641846
node_5654: feature_name=CD83	CD83.value <= threshold=2.970836043357849
node_5655: feature_name=MT-CYB	MT-CYB.value > threshold=-1.610063374042511
node_5657: feature_name=AP3M2	AP3M2.value <= threshold=1.5549840927124023
node_5658: feature_name=LDLR	LDLR.value <= threshold=2.029319405555725
node_5659: feature_name=IFIT2	IFIT2.value <= threshold=3.3327656984329224
Class: COVID-19 Critical	

Rules_34	passed counts:150
node_0: feature_name=LTB	LTB.value > threshold=0.994031548500061
node_5264: feature_name=MT-CO1	MT-CO1.value <= threshold=-0.4127991199493408
node_5265: feature_name=MTRNR2L2	MTRNR2L2.value <= threshold=2.4473838806152344
node_5266: feature_name=TCL1A	TCL1A.value <= threshold=5.787256956100464
node_5267: feature_name=PLD4	PLD4.value <= threshold=3.544657826423645
node_5268: feature_name=NR4A1	NR4A1.value <= threshold=0.8490704298019409
node_5269: feature_name=LTB	LTB.value <= threshold=1.5252206921577454
node_5270: feature_name=HLA-DRB5	HLA-DRB5.value <= threshold=2.5117164850234985
node_5271: feature_name=CD79A	CD79A.value <= threshold=3.685201406478882
node_5272: feature_name=BCL2A1	BCL2A1.value <= threshold=2.081526756286621
node_5273: feature_name=HLA-DPA1	HLA-DPA1.value > threshold=1.8042166829109192
node_5363: feature_name=MS4A1	MS4A1.value <= threshold=3.5283708572387695
node_5364: feature_name=MT-CO3	MT-CO3.value <= threshold=0.3994715213775635
node_5365: feature_name=TNFAIP3	TNFAIP3.value <= threshold=0.2631131559610367
node_5366: feature_name=MT-CO1	MT-CO1.value <= threshold=-0.44641776382923126
node_5367: feature_name=GPX1	GPX1.value <= threshold=0.7746693789958954
node_5368: feature_name=MMD	MMD.value <= threshold=3.176570177078247
node_5369: feature_name=FERMT3	FERMT3.value > threshold=-0.7207392454147339
node_5373: feature_name=HBB	HBB.value > threshold=-0.42021533846855164
node_5375: feature_name=MT-CYB	MT-CYB.value <= threshold=0.6712252795696259
node_5376: feature_name=IGLL5	IGLL5.value <= threshold=8.24229383468628
node_5377: feature_name=PLA2G12A	PLA2G12A.value <= threshold=3.9736461639404297
node_5378: feature_name=MEF2C	MEF2C.value <= threshold=4.8988196849823
node_5379: feature_name=ATF3	ATF3.value <= threshold=3.2426276206970215
node_5380: feature_name=RELT	RELT.value <= threshold=2.1399883031845093
node_5381: feature_name=SLC7A5	SLC7A5.value <= threshold=2.0621291399002075
node_5382: feature_name=TPM1	TPM1.value <= threshold=1.94612605124712
node_5383: feature_name=HLA-DPA1	HLA-DPA1.value > threshold=1.8114917278289795

node_5387: feature_name=EGR1	EGR1.value <= threshold=3.533252716064453
node_5388: feature_name=SNX9	SNX9.value <= threshold=3.2163097858428955
Class: COVID-19 Critical	
Rules_35	passed counts:147
node_0: feature_name=LTB	LTB.value > threshold=0.994031548500061
node_5264: feature_name=MT-CO1	MT-CO1.value > threshold=-0.4127991199493408
node_5638: feature_name=OAZ1	OAZ1.value > threshold=-0.2640867084264755
node_6112: feature_name=IFI44L	IFI44L.value > threshold=2.5893263816833496
node_7078: feature_name=XIST	XIST.value <= threshold=1.1465829275548458
node_7079: feature_name=TCL1A	TCL1A.value <= threshold=7.115930080413818
node_7080: feature_name=XAF1	XAF1.value > threshold=-0.45034870505332947
node_7088: feature_name=FERMT3	FERMT3.value <= threshold=1.994587481021881
node_7089: feature_name=CD22	CD22.value <= threshold=6.903364181518555
node_7090: feature_name=MARCH1	MARCH1.value <= threshold=4.378922939300537
node_7091: feature_name=HSPA1A	HSPA1A.value <= threshold=4.0261393785476685
node_7092: feature_name=ACSL1	ACSL1.value <= threshold=1.867650032043457
node_7093: feature_name=MARCKS	MARCKS.value > threshold=-0.5142948925495148
node_7095: feature_name=JUN	JUN.value <= threshold=1.7509565353393555
node_7096: feature_name=RELT	RELT.value <= threshold=2.2516160011291504
Class: COVID-19 Critical	
Rules_36	passed counts:144
node_0: feature_name=LTB	LTB.value <= threshold=0.994031548500061
node_1: feature_name=MTRNR2L2	MTRNR2L2.value <= threshold=2.8784297704696655
node_2: feature_name=TCL1A	TCL1A.value <= threshold=4.798312664031982
node_3: feature_name=MT-CO1	MT-CO1.value > threshold=0.08947191759943962
node_2521: feature_name=LTB	LTB.value > threshold=0.6038667261600494
node_4173: feature_name=OAZ1	OAZ1.value > threshold=0.143283873796463
node_4615: feature_name=NR4A1	NR4A1.value <= threshold=0.6621004343032837

node_4616: feature_name=NFKBID	NFKBID.value <= threshold=3.283741593360901
node_4617: feature_name=HLA-DQA1	HLA-DQA1.value > threshold=2.3435752391815186
node_4719: feature_name=MX1	MX1.value <= threshold=1.851993978023529
node_4720: feature_name=JUN	JUN.value > threshold=-0.5025404393672943
node_4740: feature_name=IRF8	IRF8.value <= threshold=3.838419556617737
node_4741: feature_name=BACH1	BACH1.value <= threshold=0.9568033516407013
node_4742: feature_name=HLA-DQA2	HLA-DQA2.value <= threshold=4.834465265274048
node_4743: feature_name=STAG3	STAG3.value <= threshold=6.6092634201049805
node_4744: feature_name=IFIT2	IFIT2.value <= threshold=0.700381800532341
node_4745: feature_name=BCL2A1	BCL2A1.value <= threshold=2.0025230646133423
node_4746: feature_name=HIST1H2AC	HIST1H2AC.value <= threshold=3.086765766143799
node_4747: feature_name=RNF144B	RNF144B.value <= threshold=3.0571365356445312
node_4748: feature_name=MS4A1	MS4A1.value > threshold=0.27620622515678406
node_4752: feature_name=MT-CO3	MT-CO3.value > threshold=-0.6347786486148834
Class: Healthy Control	
Rules_37	passed counts:142
node_0: feature_name=LTB	LTB.value <= threshold=0.994031548500061
node_1: feature_name=MTRNR2L2	MTRNR2L2.value <= threshold=2.8784297704696655
node_2: feature_name=TCL1A	TCL1A.value <= threshold=4.798312664031982
node_3: feature_name=MT-CO1	MT-CO1.value > threshold=0.08947191759943962
node_2521: feature_name=LTB	LTB.value > threshold=0.6038667261600494
node_4173: feature_name=OAZ1	OAZ1.value <= threshold=0.143283873796463
node_4174: feature_name=PLD4	PLD4.value <= threshold=2.921932101249695
node_4175: feature_name=XIST	XIST.value <= threshold=-0.05067425221204758
node_4176: feature_name=MT-CO1	MT-CO1.value <= threshold=1.345594048500061
node_4177: feature_name=HLA-DQB1	HLA-DQB1.value <= threshold=1.927736520767212
node_4178: feature_name=BACH1	BACH1.value > threshold=-0.5349778532981873
node_4236: feature_name=TPM1	TPM1.value <= threshold=3.516477584838867
node_4237: feature_name=NR4A1	NR4A1.value > threshold=-0.274935707449913

node_4277: feature_name=HLA-DPA1	HLA-DPA1.value <= threshold=1.8894080519676208
node_4278: feature_name=TRAF1	TRAF1.value <= threshold=2.091539978981018
node_4279: feature_name=STMN1	STMN1.value <= threshold=2.2198781967163086
node_4280: feature_name=PLD4	PLD4.value <= threshold=2.7647364139556885
node_4281: feature_name=IGJ	IGJ.value <= threshold=4.225788354873657
node_4282: feature_name=MEF2C	MEF2C.value <= threshold=3.573865532875061
node_4283: feature_name=ILK	ILK.value <= threshold=3.675175428390503
node_4284: feature_name=MT-CO3	MT-CO3.value > threshold=-0.7448426485061646
node_4288: feature_name=TCF4	TCF4.value <= threshold=3.9125311374664307
node_4289: feature_name=HES4	HES4.value <= threshold=1.3096457719802856
node_4290: feature_name=KIAA0125	KIAA0125.value <= threshold=6.644903659820557
node_4291: feature_name=FTH1	FTH1.value > threshold=-3.568124532699585
node_4293: feature_name=STAG3	STAG3.value <= threshold=8.591830015182495
node_4294: feature_name=MYL9	MYL9.value <= threshold=2.8391387462615967
node_4295: feature_name=HLA-DPB1	HLA-DPB1.value > threshold=-0.869614988565445
node_4297: feature_name=TLR2	TLR2.value <= threshold=0.7944257706403732
node_4298: feature_name=RP11-685N3.1	RP11-685N3.1.value <= threshold=9.965410232543945
Class: COVID-19 Critical	
Rules_38	passed counts:133
node_0: feature_name=LTB	LTB.value <= threshold=0.994031548500061
node_1: feature_name=MTRNR2L2	MTRNR2L2.value <= threshold=2.8784297704696655
node_2: feature_name=TCL1A	TCL1A.value <= threshold=4.798312664031982
node_3: feature_name=MT-CO1	MT-CO1.value <= threshold=0.08947191759943962
node_4: feature_name=NR4A1	NR4A1.value <= threshold=1.0725348591804504
node_5: feature_name=HLA-DPA1	HLA-DPA1.value > threshold=1.6885469555854797
node_861: feature_name=CD83	CD83.value <= threshold=3.0563642978668213
node_862: feature_name=HLA-DQB1	HLA-DQB1.value <= threshold=2.61081063747406
node_863: feature_name=MTRNR2L2	MTRNR2L2.value <= threshold=2.0919997692108154
node_864: feature_name=BCL2A1	BCL2A1.value <= threshold=1.852618932723999

node_865: feature_name=CD79B	CD79B.value <= threshold=4.352142333984375
node_866: feature_name=TNFAIP3	TNFAIP3.value > threshold=-0.0449496041983366
node_1236: feature_name=HLA-DRB5	HLA-DRB5.value <= threshold=1.710871696472168
node_1237: feature_name=HLA-DQA2	HLA-DQA2.value <= threshold=3.6443899869918823
node_1238: feature_name=FAM160B1	FAM160B1.value <= threshold=-0.36028963327407837
node_1239: feature_name=ID1	ID1.value <= threshold=2.7296812534332275
node_1240: feature_name=TLR2	TLR2.value <= threshold=2.888237714767456
node_1241: feature_name=HLA-DQA1	HLA-DQA1.value > threshold=-0.45326389372348785
node_1243: feature_name=CSF3R	CSF3R.value <= threshold=1.47954660654068
node_1244: feature_name=LTB	LTB.value > threshold=-1.0752127170562744
node_1246: feature_name=GNG11	GNG11.value <= threshold=3.7192968130111694
node_1247: feature_name=MAP3K2	MAP3K2.value <= threshold=2.3261473178863525
Class: COVID-19 Critical	
Rules_39	passed counts:129
node_0: feature_name=LTB	LTB.value <= threshold=0.994031548500061
node_1: feature_name=MTRNR2L2	MTRNR2L2.value <= threshold=2.8784297704696655
node_2: feature_name=TCL1A	TCL1A.value <= threshold=4.798312664031982
node_3: feature_name=MT-CO1	MT-CO1.value > threshold=0.08947191759943962
node_2521: feature_name=LTB	LTB.value <= threshold=0.6038667261600494
node_2522: feature_name=HLA-DQB1	HLA-DQB1.value <= threshold=2.1053054332733154
node_2523: feature_name=SNX9	SNX9.value > threshold=-0.5346486568450928
node_2907: feature_name=NR4A1	NR4A1.value > threshold=2.5433837175369263
node_3459: feature_name=VPREB3	VPREB3.value <= threshold=4.410779237747192
node_3460: feature_name=RALGPS2	RALGPS2.value <= threshold=3.064661979675293
node_3461: feature_name=MT-CO1	MT-CO1.value <= threshold=1.7489010095596313
node_3462: feature_name=HLA-DQA2	HLA-DQA2.value <= threshold=1.794841468334198
node_3463: feature_name=CYBB	CYBB.value <= threshold=1.5984815955162048
node_3464: feature_name=MARCKS	MARCKS.value > threshold=-0.38392288982868195
node_3474: feature_name=PLD4	PLD4.value <= threshold=7.326558828353882

node_3475: feature_name=IRAK3	IRAK3.value <= threshold=4.625715494155884
node_3476: feature_name=CCL3	CCL3.value <= threshold=1.9986491203308105
node_3477: feature_name=ID1	ID1.value <= threshold=3.170982599258423
node_3478: feature_name=AHR	AHR.value <= threshold=4.220391273498535
node_3479: feature_name=OAZ1	OAZ1.value > threshold=-1.7742292881011963
Class: COVID-19 Critical	
Rules_40	passed counts:128
node_0: feature_name=LTB	LTB.value <= threshold=0.994031548500061
node_1: feature_name=MTRNR2L2	MTRNR2L2.value <= threshold=2.8784297704696655
node_2: feature_name=TCL1A	TCL1A.value <= threshold=4.798312664031982
node_3: feature_name=MT-CO1	MT-CO1.value > threshold=0.08947191759943962
node_2521: feature_name=LTB	LTB.value <= threshold=0.6038667261600494
node_2522: feature_name=HLA-DQB1	HLA-DQB1.value <= threshold=2.1053054332733154
node_2523: feature_name=SNX9	SNX9.value > threshold=-0.5346486568450928
node_2907: feature_name=NR4A1	NR4A1.value <= threshold=2.5433837175369263
node_2908: feature_name=XIST	XIST.value <= threshold=0.7664470989257097
node_2909: feature_name=CD79B	CD79B.value > threshold=2.9326847791671753
node_3353: feature_name=VPREB3	VPREB3.value <= threshold=3.0226117372512817
node_3354: feature_name=CD83	CD83.value <= threshold=1.9074636101722717
node_3355: feature_name=FERMT3	FERMT3.value <= threshold=1.6086163520812988
node_3356: feature_name=IGJ	IGJ.value <= threshold=4.493098735809326
node_3357: feature_name=HLA-DQA1	HLA-DQA1.value <= threshold=3.502458691596985
node_3358: feature_name=ID1	ID1.value <= threshold=1.2194700837135315
node_3359: feature_name=OAZ1	OAZ1.value <= threshold=1.6739212274551392
node_3360: feature_name=HLA-DPB1	HLA-DPB1.value <= threshold=2.48960018157959
node_3361: feature_name=TPM1	TPM1.value <= threshold=3.764936327934265
node_3362: feature_name=DUSP4	DUSP4.value <= threshold=3.401969313621521
node_3363: feature_name=PDLIM1	PDLIM1.value <= threshold=4.827272415161133
Class: COVID-19 Critical	

Rules_41	passed counts:124
node_0: feature_name=LTB	LTB.value > threshold=0.994031548500061
node_5264: feature_name=MT-CO1	MT-CO1.value > threshold=-0.4127991199493408
node_5638: feature_name=OAZ1	OAZ1.value > threshold=-0.2640867084264755
node_6112: feature_name=IFI44L	IFI44L.value <= threshold=2.5893263816833496
node_6113: feature_name=TCL1A	TCL1A.value <= threshold=4.201832294464111
node_6114: feature_name=HLA-DQA2	HLA-DQA2.value > threshold=-0.2270243540406227
node_6542: feature_name=CD79B	CD79B.value <= threshold=3.096232771873474
node_6543: feature_name=NR4A1	NR4A1.value <= threshold=1.421015739440918
node_6544: feature_name=LTB	LTB.value <= threshold=1.359755277633667
node_6545: feature_name=NFKBID	NFKBID.value > threshold=1.4294711351394653
node_6643: feature_name=FAM129C	FAM129C.value <= threshold=4.799884080886841
node_6644: feature_name=XIST	XIST.value <= threshold=0.9931697957217693
node_6645: feature_name=GPX1	GPX1.value <= threshold=0.3628883808851242
node_6646: feature_name=STMN1	STMN1.value <= threshold=2.3225364685058594
node_6647: feature_name=APP	APP.value <= threshold=4.124613881111145
node_6648: feature_name=FCER2	FCER2.value <= threshold=5.776939868927002
node_6649: feature_name=MEF2C	MEF2C.value <= threshold=3.8539518117904663
node_6650: feature_name=RHOB	RHOB.value <= threshold=1.7790308594703674
node_6651: feature_name=HSPA1A	HSPA1A.value <= threshold=3.537449836730957
node_6652: feature_name=MTRNR2L2	MTRNR2L2.value <= threshold=3.4327136278152466
node_6653: feature_name=MT-CO3	MT-CO3.value > threshold=-2.1226823925971985
node_6655: feature_name=TLR2	TLR2.value <= threshold=1.0477308258414268
node_6656: feature_name=PLEC	PLEC.value <= threshold=2.035267412662506
node_6657: feature_name=IGJ	IGJ.value > threshold=-0.38578981161117554
node_6661: feature_name=PPIF	PPIF.value <= threshold=1.9370648860931396
Class: COVID-19 Critical	
Rules_42	passed counts:120

node_0: feature_name=LTB	LTB.value > threshold=0.994031548500061
node_5264: feature_name=MT-CO1	MT-CO1.value > threshold=-0.4127991199493408
node_5638: feature_name=OAZ1	OAZ1.value > threshold=-0.2640867084264755
node_6112: feature_name=IFI44L	IFI44L.value <= threshold=2.5893263816833496
node_6113: feature_name=TCL1A	TCL1A.value <= threshold=4.201832294464111
node_6114: feature_name=HLA-DQA2	HLA-DQA2.value > threshold=-0.2270243540406227
node_6542: feature_name=CD79B	CD79B.value <= threshold=3.096232771873474
node_6543: feature_name=NR4A1	NR4A1.value <= threshold=1.421015739440918
node_6544: feature_name=LTB	LTB.value > threshold=1.359755277633667
node_6720: feature_name=NFKBIZ	NFKBIZ.value <= threshold=0.2720414251089096
node_6721: feature_name=ATF5	ATF5.value <= threshold=2.4672781229019165
node_6722: feature_name=SNX9	SNX9.value <= threshold=2.0814030170440674
node_6723: feature_name=HLA-DQA2	HLA-DQA2.value <= threshold=4.360614776611328
node_6724: feature_name=MT-CO1	MT-CO1.value > threshold=-0.12188276648521423
node_6736: feature_name=MT1E	MT1E.value <= threshold=-0.1352209895849228
node_6737: feature_name=PELI1	PELI1.value <= threshold=3.1761642694473267
node_6738: feature_name=TRAF1	TRAF1.value <= threshold=-0.2332744374871254
node_6739: feature_name=HLA-DQA2	HLA-DQA2.value > threshold=-0.22670809924602509
node_6741: feature_name=HLA-DRB5	HLA-DRB5.value > threshold=-0.7810345888137817
node_6745: feature_name=GPX1	GPX1.value <= threshold=0.5079085677862167
node_6746: feature_name=MMD	MMD.value <= threshold=2.110599994659424
node_6747: feature_name=FAM160B1	FAM160B1.value <= threshold=1.345553994178772
Class: Healthy Control	
Rules_43	passed counts:118
node_0: feature_name=LTB	LTB.value > threshold=0.994031548500061
node_5264: feature_name=MT-CO1	MT-CO1.value > threshold=-0.4127991199493408
node_5638: feature_name=OAZ1	OAZ1.value <= threshold=-0.2640867084264755
node_5639: feature_name=CD79B	CD79B.value > threshold=2.2998517751693726
node_5925: feature_name=SNX9	SNX9.value <= threshold=2.4445992708206177

node_5926: feature_name=TCL1A	TCL1A.value > threshold=2.6938670873641968
node_6052: feature_name=IGLL5	IGLL5.value > threshold=-0.26075856387615204
node_6054: feature_name=IFI44L	IFI44L.value <= threshold=1.9543575644493103
node_6055: feature_name=LTB	LTB.value > threshold=1.0038496255874634
node_6059: feature_name=RGS1	RGS1.value <= threshold=2.333457112312317
node_6060: feature_name=ITGAX	ITGAX.value <= threshold=0.9978828206658363
node_6061: feature_name=DENND3	DENND3.value <= threshold=3.04962956905365
node_6062: feature_name=S100A6	S100A6.value <= threshold=0.4195839613676071
node_6063: feature_name=CCDC141	CCDC141.value <= threshold=6.152930974960327
node_6064: feature_name=YWHAH	YWHAH.value <= threshold=2.891282320022583
node_6065: feature_name=FTH1	FTH1.value <= threshold=0.9599592983722687
Class: Healthy Control	
Rules_44	passed counts:114
node_0: feature_name=LTB	LTB.value <= threshold=0.994031548500061
node_1: feature_name=MTRNR2L2	MTRNR2L2.value <= threshold=2.8784297704696655
node_2: feature_name=TCL1A	TCL1A.value <= threshold=4.798312664031982
node_3: feature_name=MT-CO1	MT-CO1.value <= threshold=0.08947191759943962
node_4: feature_name=NR4A1	NR4A1.value <= threshold=1.0725348591804504
node_5: feature_name=HLA-DPA1	HLA-DPA1.value <= threshold=1.6885469555854797
node_6: feature_name=HLA-DRB5	HLA-DRB5.value <= threshold=2.1886194944381714
node_7: feature_name=XIST	XIST.value <= threshold=-0.12328458577394485
node_8: feature_name=SYNE1	SYNE1.value > threshold=-0.7835594415664673
node_16: feature_name=MTRNR2L2	MTRNR2L2.value <= threshold=2.4483247995376587
node_17: feature_name=HLA-DQA2	HLA-DQA2.value <= threshold=2.227178931236267
node_18: feature_name=NR4A1	NR4A1.value <= threshold=-0.21380723267793655
node_19: feature_name=IRAK3	IRAK3.value <= threshold=4.27159571647644
node_20: feature_name=IFIT2	IFIT2.value <= threshold=7.8175740242004395
node_21: feature_name=CD79A	CD79A.value <= threshold=2.857242226600647
node_22: feature_name=HIST1H2AC	HIST1H2AC.value <= threshold=5.077470302581787

node_23: feature_name=FTH1	FTH1.value <= threshold=0.4158056080341339
node_24: feature_name=ATF3	ATF3.value <= threshold=3.8028353452682495
node_25: feature_name=OASL	OASL.value <= threshold=3.387580633163452
node_26: feature_name=MT-CO1	MT-CO1.value > threshold=-0.7942646145820618
node_74: feature_name=HOPX	HOPX.value <= threshold=0.8682331740856171
node_75: feature_name=RGS2	RGS2.value > threshold=1.4589954018592834
node_213: feature_name=TRAF1	TRAF1.value <= threshold=2.4759315252304077
node_214: feature_name=IRF8	IRF8.value > threshold=-0.4509534388780594
node_218: feature_name=EGR1	EGR1.value <= threshold=4.4260780811309814
node_219: feature_name=PPIF	PPIF.value <= threshold=1.8276893496513367
node_220: feature_name=RGS2	RGS2.value <= threshold=2.9868245124816895
node_221: feature_name=CD79B	CD79B.value <= threshold=4.210061550140381
node_222: feature_name=CD79A	CD79A.value <= threshold=2.8370617628097534
node_223: feature_name=RP5-821D11.7	RP5-821D11.7.value <= threshold=4.518633961677551
node_224: feature_name=HLA-DQB1	HLA-DQB1.value <= threshold=3.2811548709869385
node_225: feature_name=LDLRAD4	LDLRAD4.value <= threshold=4.308608055114746
node_226: feature_name=MYL9	MYL9.value <= threshold=4.106156587600708
node_227: feature_name=RELT	RELT.value <= threshold=1.972962498664856
Class: COVID-19 Critical	
Rules_45	passed counts:111
node_0: feature_name=LTB	LTB.value > threshold=0.994031548500061
node_5264: feature_name=MT-CO1	MT-CO1.value > threshold=-0.4127991199493408
node_5638: feature_name=OAZ1	OAZ1.value > threshold=-0.2640867084264755
node_6112: feature_name=IFI44L	IFI44L.value <= threshold=2.5893263816833496
node_6113: feature_name=TCL1A	TCL1A.value <= threshold=4.201832294464111
node_6114: feature_name=HLA-DQA2	HLA-DQA2.value <= threshold=-0.2270243540406227
node_6115: feature_name=MTRNR2L2	MTRNR2L2.value <= threshold=2.548917293548584
node_6116: feature_name=ATF5	ATF5.value <= threshold=2.041263461112976
node_6117: feature_name=JUN	JUN.value > threshold=-0.616259902715683

node_6189: feature_name=MT-CO3	MT-CO3.value > threshold=0.13483521342277527
node_6351: feature_name=XAF1	XAF1.value <= threshold=3.061717987060547
node_6352: feature_name=MT-CO2	MT-CO2.value <= threshold=1.2605181336402893
node_6353: feature_name=ICAM1	ICAM1.value > threshold=-0.3340446650981903
node_6439: feature_name=FAM129C	FAM129C.value > threshold=-0.24657993018627167
node_6455: feature_name=HSP90B1	HSP90B1.value <= threshold=1.5712243914604187
node_6456: feature_name=NR4A1	NR4A1.value > threshold=-0.6648433208465576
node_6462: feature_name=IFIT3	IFIT3.value <= threshold=3.3345084190368652
node_6463: feature_name=GPX1	GPX1.value <= threshold=0.91975337266922
node_6464: feature_name=MEF2A	MEF2A.value <= threshold=3.2376389503479004
node_6465: feature_name=HLA-DPA1	HLA-DPA1.value > threshold=-0.04563889652490616
node_6467: feature_name=NAIP	NAIP.value <= threshold=1.7054211497306824
Class: Healthy Control	
Rules_46	passed counts:108
node_0: feature_name=LTB	LTB.value > threshold=0.994031548500061
node_5264: feature_name=MT-CO1	MT-CO1.value > threshold=-0.4127991199493408
node_5638: feature_name=OAZ1	OAZ1.value > threshold=-0.2640867084264755
node_6112: feature_name=IFI44L	IFI44L.value <= threshold=2.5893263816833496
node_6113: feature_name=TCL1A	TCL1A.value <= threshold=4.201832294464111
node_6114: feature_name=HLA-DQA2	HLA-DQA2.value > threshold=-0.2270243540406227
node_6542: feature_name=CD79B	CD79B.value <= threshold=3.096232771873474
node_6543: feature_name=NR4A1	NR4A1.value > threshold=1.421015739440918
node_6811: feature_name=SLC7A5	SLC7A5.value <= threshold=1.570515513420105
node_6812: feature_name=XAF1	XAF1.value <= threshold=2.218220591545105
node_6813: feature_name=MEF2C	MEF2C.value > threshold=-0.35489463806152344
node_6831: feature_name=ACSL1	ACSL1.value <= threshold=2.9197680950164795
node_6832: feature_name=SNX9	SNX9.value <= threshold=4.184023022651672
node_6833: feature_name=HLA-DQB1	HLA-DQB1.value > threshold=-0.5399456918239594
node_6837: feature_name=SYTL3	SYTL3.value <= threshold=1.365163803100586

node_6838: feature_name=MIAT	MIAT.value <= threshold=1.9473694376647472
node_6839: feature_name=TRIB1	TRIB1.value <= threshold=2.461132228374481
node_6840: feature_name=ATF5	ATF5.value <= threshold=5.3038389682769775
node_6841: feature_name=S100A6	S100A6.value > threshold=-1.8476895093917847
node_6845: feature_name=SNORD3B-2	SNORD3B-2.value <= threshold=4.938738822937012
node_6846: feature_name=RNF144B	RNF144B.value <= threshold=2.939046025276184
node_6847: feature_name=MS4A1	MS4A1.value > threshold=1.0266582369804382
Class: Healthy Control	
Rules_47	passed counts:106
node_0: feature_name=LTB	LTB.value > threshold=0.994031548500061
node_5264: feature_name=MT-CO1	MT-CO1.value > threshold=-0.4127991199493408
node_5638: feature_name=OAZ1	OAZ1.value <= threshold=-0.2640867084264755
node_5639: feature_name=CD79B	CD79B.value > threshold=2.2998517751693726
node_5925: feature_name=SNX9	SNX9.value <= threshold=2.4445992708206177
node_5926: feature_name=TCL1A	TCL1A.value <= threshold=2.6938670873641968
node_5927: feature_name=PLD4	PLD4.value > threshold=-0.06434785947203636
node_6009: feature_name=MX1	MX1.value <= threshold=3.600893020629883
node_6010: feature_name=PRDM1	PRDM1.value <= threshold=-0.407244011759758
node_6011: feature_name=SNORD3B-2	SNORD3B-2.value <= threshold=2.7082353830337524
node_6012: feature_name=NFKBID	NFKBID.value <= threshold=3.0716724395751953
node_6013: feature_name=HLA-DQA2	HLA-DQA2.value <= threshold=3.7589778900146484
node_6014: feature_name=HLA-DQB1	HLA-DQB1.value <= threshold=3.291059136390686
node_6015: feature_name=ATG2A	ATG2A.value <= threshold=1.4668332934379578
node_6016: feature_name=IRF4	IRF4.value <= threshold=4.835575103759766
node_6017: feature_name=IL1B	IL1B.value <= threshold=2.2611767649650574
node_6018: feature_name=IFI44L	IFI44L.value <= threshold=1.2665085792541504
Class: Healthy Control	
Rules_48	passed counts:103

node_0: feature_name=LTB	LTB.value <= threshold=0.994031548500061
node_1: feature_name=MTRNR2L2	MTRNR2L2.value <= threshold=2.8784297704696655
node_2: feature_name=TCL1A	TCL1A.value <= threshold=4.798312664031982
node_3: feature_name=MT-CO1	MT-CO1.value > threshold=0.08947191759943962
node_2521: feature_name=LTB	LTB.value <= threshold=0.6038667261600494
node_2522: feature_name=HLA-DQB1	HLA-DQB1.value <= threshold=2.1053054332733154
node_2523: feature_name=SNX9	SNX9.value > threshold=-0.5346486568450928
node_2907: feature_name=NR4A1	NR4A1.value <= threshold=2.5433837175369263
node_2908: feature_name=XIST	XIST.value <= threshold=0.7664470989257097
node_2909: feature_name=CD79B	CD79B.value <= threshold=2.9326847791671753
node_2910: feature_name=NR4A1	NR4A1.value > threshold=-0.2875557690858841
node_3096: feature_name=NR4A3	NR4A3.value <= threshold=3.314968705177307
node_3097: feature_name=VPREB3	VPREB3.value <= threshold=3.3549938201904297
node_3098: feature_name=RALGPS2	RALGPS2.value <= threshold=5.807050704956055
node_3099: feature_name=CTD-2035E11.3	CTD-2035E11.3.value <= threshold=6.4090416431427
node_3100: feature_name=FAM129C	FAM129C.value <= threshold=6.966361045837402
node_3101: feature_name=HES4	HES4.value <= threshold=4.139557242393494
node_3102: feature_name=CD83	CD83.value > threshold=1.8343153595924377
node_3136: feature_name=PLD4	PLD4.value <= threshold=4.453821897506714
node_3137: feature_name=HLA-DQA2	HLA-DQA2.value <= threshold=-0.10758286714553833
node_3138: feature_name=STAG3	STAG3.value <= threshold=7.365770578384399
node_3139: feature_name=LDLR	LDLR.value <= threshold=2.3423796892166138
node_3140: feature_name=CD79A	CD79A.value <= threshold=4.038331866264343
node_3141: feature_name=DUSP4	DUSP4.value <= threshold=4.829863548278809
node_3142: feature_name=OAZ1	OAZ1.value > threshold=-0.34704728424549103
node_3178: feature_name=MX1	MX1.value > threshold=-0.48719069361686707
node_3184: feature_name=HLA-DPB1	HLA-DPB1.value <= threshold=2.151633381843567
node_3185: feature_name=RGS2	RGS2.value <= threshold=1.594672977924347
node_3186: feature_name=RELT	RELT.value <= threshold=2.0173692107200623
node_3187: feature_name=RALGPS2	RALGPS2.value > threshold=-0.4155271500349045

node_3189: feature_name=ID3	ID3.value <= threshold=6.75140643119812
node_3190: feature_name=ENDOD1	ENDOD1.value <= threshold=7.847548723220825
node_3191: feature_name=MARCKS	MARCKS.value <= threshold=4.328244686126709
Class: COVID-19 Critical	
Rules_49	passed counts:98
node_0: feature_name=LTB	LTB.value <= threshold=0.994031548500061
node_1: feature_name=MTRNR2L2	MTRNR2L2.value <= threshold=2.8784297704696655
node_2: feature_name=TCL1A	TCL1A.value <= threshold=4.798312664031982
node_3: feature_name=MT-CO1	MT-CO1.value > threshold=0.08947191759943962
node_2521: feature_name=LTB	LTB.value <= threshold=0.6038667261600494
node_2522: feature_name=HLA-DQB1	HLA-DQB1.value > threshold=2.1053054332733154
node_3550: feature_name=XIST	XIST.value <= threshold=0.7716337237507105
node_3551: feature_name=CD79B	CD79B.value <= threshold=2.9426796436309814
node_3552: feature_name=HLA-DQB1	HLA-DQB1.value <= threshold=2.713674306869507
node_3553: feature_name=SNX9	SNX9.value > threshold=-0.5404067635536194
node_3653: feature_name=LTB	LTB.value > threshold=0.021708179265260696
node_3759: feature_name=TCL1A	TCL1A.value <= threshold=3.3921085596084595
node_3760: feature_name=HLA-DRB5	HLA-DRB5.value > threshold=0.5265218019485474
node_3776: feature_name=ID1	ID1.value <= threshold=-0.21235637366771698
node_3777: feature_name=RP11-731F5.2	RP11-731F5.2.value <= threshold=2.830894112586975
node_3778: feature_name=CASP8	CASP8.value <= threshold=2.222941279411316
node_3779: feature_name=MT-CO3	MT-CO3.value > threshold=-0.41117167472839355
node_3785: feature_name=HLA-DRB5	HLA-DRB5.value > threshold=0.8465962707996368
node_3793: feature_name=PDLIM1	PDLIM1.value <= threshold=2.90118670463562
node_3794: feature_name=WDR74	WDR74.value <= threshold=3.335801362991333
node_3795: feature_name=MEF2C	MEF2C.value <= threshold=4.397150039672852
node_3796: feature_name=HLA-DPA1	HLA-DPA1.value > threshold=-0.10480117797851562
node_3798: feature_name=FCER2	FCER2.value <= threshold=5.688235521316528
node_3799: feature_name=FTH1	FTH1.value <= threshold=1.0842017531394958

node_3800: feature_name=ILK	ILK.value <= threshold=2.2077265977859497
Class: COVID-19 Critical	
Rules_50	passed counts:97
node_0: feature_name=LTB	LTB.value <= threshold=0.994031548500061
node_1: feature_name=MTRNR2L2	MTRNR2L2.value <= threshold=2.8784297704696655
node_2: feature_name=TCL1A	TCL1A.value <= threshold=4.798312664031982
node_3: feature_name=MT-CO1	MT-CO1.value > threshold=0.08947191759943962
node_2521: feature_name=LTB	LTB.value <= threshold=0.6038667261600494
node_2522: feature_name=HLA-DQB1	HLA-DQB1.value <= threshold=2.1053054332733154
node_2523: feature_name=SNX9	SNX9.value <= threshold=-0.5346486568450928
node_2524: feature_name=HLA-DPB1	HLA-DPB1.value <= threshold=1.9548270106315613
node_2525: feature_name=FAM129C	FAM129C.value > threshold=3.3393388986587524
node_2721: feature_name=ADAM19	ADAM19.value <= threshold=-0.29525794088840485
node_2722: feature_name=NR4A1	NR4A1.value <= threshold=2.1566513776779175
node_2723: feature_name=CD22	CD22.value <= threshold=8.345317840576172
node_2724: feature_name=PLD4	PLD4.value <= threshold=7.507996082305908
node_2725: feature_name=RALGPS2	RALGPS2.value <= threshold=4.3808369636535645
node_2726: feature_name=VPREB3	VPREB3.value <= threshold=4.1944358348846436
node_2727: feature_name=LDLRAD4	LDLRAD4.value <= threshold=3.293746590614319
node_2728: feature_name=MT-CO3	MT-CO3.value > threshold=-0.9921484291553497
node_2730: feature_name=PDLIM1	PDLIM1.value <= threshold=5.490554571151733
node_2731: feature_name=GRASP	GRASP.value <= threshold=5.188978672027588
node_2732: feature_name=RP11-731F5.2	RP11-731F5.2.value <= threshold=6.8664562702178955
Class: COVID-19 Critical	
Rules_51	passed counts:95
node_0: feature_name=LTB	LTB.value <= threshold=0.994031548500061
node_1: feature_name=MTRNR2L2	MTRNR2L2.value <= threshold=2.8784297704696655
node_2: feature_name=TCL1A	TCL1A.value <= threshold=4.798312664031982

node_3: feature_name=MT-CO1	MT-CO1.value <= threshold=0.08947191759943962
node_4: feature_name=NR4A1	NR4A1.value > threshold=1.0725348591804504
node_1622: feature_name=HLA-DQA2	HLA-DQA2.value > threshold=1.9496816992759705
node_2350: feature_name=BCL2A1	BCL2A1.value > threshold=1.1322240233421326
node_2466: feature_name=MS4A1	MS4A1.value <= threshold=2.7321707010269165
node_2467: feature_name=HSP90B1	HSP90B1.value > threshold=0.8215908706188202
node_2495: feature_name=RP11-731F5.2	RP11-731F5.2.value <= threshold=5.027556896209717
node_2496: feature_name=HLA-DQB1	HLA-DQB1.value > threshold=-0.5962571501731873
node_2500: feature_name=HLA-DRB5	HLA-DRB5.value <= threshold=2.8970696926116943
node_2501: feature_name=VPREB3	VPREB3.value <= threshold=6.056820392608643
node_2502: feature_name=ID3	ID3.value <= threshold=6.186669826507568
Class: COVID-19 Severe	
Rules_52	passed counts:94
node_0: feature_name=LTB	LTB.value <= threshold=0.994031548500061
node_1: feature_name=MTRNR2L2	MTRNR2L2.value <= threshold=2.8784297704696655
node_2: feature_name=TCL1A	TCL1A.value <= threshold=4.798312664031982
node_3: feature_name=MT-CO1	MT-CO1.value > threshold=0.08947191759943962
node_2521: feature_name=LTB	LTB.value <= threshold=0.6038667261600494
node_2522: feature_name=HLA-DQB1	HLA-DQB1.value <= threshold=2.1053054332733154
node_2523: feature_name=SNX9	SNX9.value <= threshold=-0.5346486568450928
node_2524: feature_name=HLA-DPB1	HLA-DPB1.value <= threshold=1.9548270106315613
node_2525: feature_name=FAM129C	FAM129C.value <= threshold=3.3393388986587524
node_2526: feature_name=XIST	XIST.value <= threshold=3.6911416053771973
node_2527: feature_name=NR4A1	NR4A1.value > threshold=-0.2557755410671234
node_2625: feature_name=HLA-DPA1	HLA-DPA1.value <= threshold=0.9335225224494934
node_2626: feature_name=CD83	CD83.value > threshold=-0.585537850856781
node_2628: feature_name=PDLIM1	PDLIM1.value <= threshold=5.292127370834351
node_2629: feature_name=ISG15	ISG15.value <= threshold=3.357967495918274
node_2630: feature_name=BACH1	BACH1.value <= threshold=4.870309114456177

node_2631: feature_name=LDLRAD4	LDLRAD4.value <= threshold=-0.2641522139310837
node_2632: feature_name=CD83	CD83.value <= threshold=3.8994957208633423
node_2633: feature_name=HIST1H2AC	HIST1H2AC.value <= threshold=7.211487054824829
node_2634: feature_name=NR4A3	NR4A3.value <= threshold=4.066339612007141
node_2635: feature_name=RBKS	RBKS.value <= threshold=7.189899444580078
Class: COVID-19 Critical	
Rules_53	passed counts:86
node_0: feature_name=LTB	LTB.value <= threshold=0.994031548500061
node_1: feature_name=MTRNR2L2	MTRNR2L2.value <= threshold=2.8784297704696655
node_2: feature_name=TCL1A	TCL1A.value <= threshold=4.798312664031982
node_3: feature_name=MT-CO1	MT-CO1.value > threshold=0.08947191759943962
node_2521: feature_name=LTB	LTB.value <= threshold=0.6038667261600494
node_2522: feature_name=HLA-DQB1	HLA-DQB1.value > threshold=2.1053054332733154
node_3550: feature_name=XIST	XIST.value <= threshold=0.7716337237507105
node_3551: feature_name=CD79B	CD79B.value <= threshold=2.9426796436309814
node_3552: feature_name=HLA-DQB1	HLA-DQB1.value <= threshold=2.713674306869507
node_3553: feature_name=SNX9	SNX9.value <= threshold=-0.5404067635536194
node_3554: feature_name=VPREB3	VPREB3.value <= threshold=4.4475414752960205
node_3555: feature_name=RALGPS2	RALGPS2.value <= threshold=2.7017585039138794
node_3556: feature_name=MEF2C	MEF2C.value <= threshold=3.997420072555542
node_3557: feature_name=NR4A1	NR4A1.value <= threshold=3.9266018867492676
node_3558: feature_name=CD22	CD22.value <= threshold=5.738929986953735
node_3559: feature_name=IRF4	IRF4.value <= threshold=6.277133464813232
node_3560: feature_name=MTRNR2L2	MTRNR2L2.value <= threshold=1.1907066702842712
node_3561: feature_name=BACH1	BACH1.value > threshold=-0.6081153750419617
node_3567: feature_name=DUSP4	DUSP4.value > threshold=-0.27051687240600586
node_3569: feature_name=FAM160B1	FAM160B1.value <= threshold=3.54995596408844
node_3570: feature_name=MT-CO2	MT-CO2.value > threshold=-0.5464511513710022
node_3572: feature_name=TLR2	TLR2.value <= threshold=1.2082695588469505

node_3573: feature_name=SYNE1	SYNE1.value <= threshold=1.1966111063957214
node_3574: feature_name=PPIF	PPIF.value <= threshold=1.97076016664505
node_3575: feature_name=PDLIM1	PDLIM1.value <= threshold=4.590035915374756
node_3576: feature_name=NR4A1	NR4A1.value <= threshold=2.7961448431015015
node_3577: feature_name=RGS2	RGS2.value <= threshold=2.236423373222351
Class: COVID-19 Critical	
Rules_54	passed counts:84
node_0: feature_name=LTB	LTB.value <= threshold=0.994031548500061
node_1: feature_name=MTRNR2L2	MTRNR2L2.value <= threshold=2.8784297704696655
node_2: feature_name=TCL1A	TCL1A.value <= threshold=4.798312664031982
node_3: feature_name=MT-CO1	MT-CO1.value > threshold=0.08947191759943962
node_2521: feature_name=LTB	LTB.value > threshold=0.6038667261600494
node_4173: feature_name=OAZ1	OAZ1.value <= threshold=0.143283873796463
node_4174: feature_name=PLD4	PLD4.value <= threshold=2.921932101249695
node_4175: feature_name=XIST	XIST.value <= threshold=-0.05067425221204758
node_4176: feature_name=MT-CO1	MT-CO1.value <= threshold=1.345594048500061
node_4177: feature_name=HLA-DQB1	HLA-DQB1.value <= threshold=1.927736520767212
node_4178: feature_name=BACH1	BACH1.value <= threshold=-0.5349778532981873
node_4179: feature_name=CD79B	CD79B.value <= threshold=1.6518195271492004
node_4180: feature_name=FTH1	FTH1.value > threshold=-1.4486562609672546
node_4184: feature_name=FAM160B1	FAM160B1.value <= threshold=1.850276529788971
node_4185: feature_name=HSPA1A	HSPA1A.value <= threshold=1.0704439878463745
node_4186: feature_name=NR4A1	NR4A1.value <= threshold=1.1393861174583435
node_4187: feature_name=MEF2C	MEF2C.value > threshold=-0.45953963696956635
node_4189: feature_name=CD38	CD38.value <= threshold=2.5397236347198486
Class: COVID-19 Critical	
Rules_55	passed counts:84
node_0: feature_name=LTB	LTB.value <= threshold=0.994031548500061

node_1: feature_name=MTRNR2L2	MTRNR2L2.value <= threshold=2.8784297704696655
node_2: feature_name=TCL1A	TCL1A.value <= threshold=4.798312664031982
node_3: feature_name=MT-CO1	MT-CO1.value <= threshold=0.08947191759943962
node_4: feature_name=NR4A1	NR4A1.value <= threshold=1.0725348591804504
node_5: feature_name=HLA-DPA1	HLA-DPA1.value <= threshold=1.6885469555854797
node_6: feature_name=HLA-DRB5	HLA-DRB5.value > threshold=2.1886194944381714
node_738: feature_name=HLA-DQA2	HLA-DQA2.value <= threshold=-0.20105575770139694
node_739: feature_name=HLA-DQA1	HLA-DQA1.value > threshold=1.7414769530296326
node_763: feature_name=HLA-DRB5	HLA-DRB5.value <= threshold=2.6777851581573486
node_764: feature_name=ICAM1	ICAM1.value > threshold=-0.3619510233402252
node_770: feature_name=SESN3	SESN3.value > threshold=-0.473929226398468
node_772: feature_name=PTGS1	PTGS1.value <= threshold=5.343248605728149
node_773: feature_name=KIAA0125	KIAA0125.value <= threshold=6.498627185821533
node_774: feature_name=CD83	CD83.value <= threshold=3.549751400947571
node_775: feature_name=ENDOD1	ENDOD1.value <= threshold=5.09564471244812
node_776: feature_name=HLA-DRB1	HLA-DRB1.value > threshold=0.8218721747398376
node_778: feature_name=MIR24-2	MIR24-2.value <= threshold=1.968224048614502
node_779: feature_name=STMN1	STMN1.value <= threshold=3.082697033882141
node_780: feature_name=MT-ND6	MT-ND6.value <= threshold=1.1761196851730347
node_781: feature_name=MMD	MMD.value <= threshold=4.159241437911987
Class: COVID-19 Critical	
Rules_56	passed counts:82
node_0: feature_name=LTB	LTB.value > threshold=0.994031548500061
node_5264: feature_name=MT-CO1	MT-CO1.value <= threshold=-0.4127991199493408
node_5265: feature_name=MTRNR2L2	MTRNR2L2.value <= threshold=2.4473838806152344
node_5266: feature_name=TCL1A	TCL1A.value > threshold=5.787256956100464
node_5616: feature_name=XAF1	XAF1.value <= threshold=3.0923240184783936
node_5617: feature_name=CD79A	CD79A.value > threshold=2.297448992729187
node_5623: feature_name=MT-CO1	MT-CO1.value > threshold=-1.1357948780059814

node_5629: feature_name=BOD1L1	BOD1L1.value <= threshold=2.8688247203826904
node_5630: feature_name=CYBB	CYBB.value <= threshold=2.8891000747680664
node_5631: feature_name=MAP3K2	MAP3K2.value <= threshold=2.492444157600403
Class: Healthy Control	
Rules_57	passed counts:82
node_0: feature_name=LTB	LTB.value <= threshold=0.994031548500061
node_1: feature_name=MTRNR2L2	MTRNR2L2.value <= threshold=2.8784297704696655
node_2: feature_name=TCL1A	TCL1A.value <= threshold=4.798312664031982
node_3: feature_name=MT-CO1	MT-CO1.value <= threshold=0.08947191759943962
node_4: feature_name=NR4A1	NR4A1.value <= threshold=1.0725348591804504
node_5: feature_name=HLA-DPA1	HLA-DPA1.value <= threshold=1.6885469555854797
node_6: feature_name=HLA-DRB5	HLA-DRB5.value <= threshold=2.1886194944381714
node_7: feature_name=XIST	XIST.value <= threshold=-0.12328458577394485
node_8: feature_name=SYNE1	SYNE1.value > threshold=-0.7835594415664673
node_16: feature_name=MTRNR2L2	MTRNR2L2.value <= threshold=2.4483247995376587
node_17: feature_name=HLA-DQA2	HLA-DQA2.value <= threshold=2.227178931236267
node_18: feature_name=NR4A1	NR4A1.value <= threshold=-0.21380723267793655
node_19: feature_name=IRAK3	IRAK3.value <= threshold=4.27159571647644
node_20: feature_name=IFIT2	IFIT2.value <= threshold=7.8175740242004395
node_21: feature_name=CD79A	CD79A.value > threshold=2.857242226600647
node_375: feature_name=LTB	LTB.value <= threshold=0.7405790388584137
node_376: feature_name=NFKBIZ	NFKBIZ.value <= threshold=1.7718515396118164
node_377: feature_name=OAZ1	OAZ1.value <= threshold=1.3302325010299683
node_378: feature_name=RHOB	RHOB.value <= threshold=3.0459729433059692
node_379: feature_name=MT-CO3	MT-CO3.value <= threshold=1.6619641780853271
node_380: feature_name=MTRNR2L2	MTRNR2L2.value <= threshold=2.1229071617126465
node_381: feature_name=MAP3K2	MAP3K2.value <= threshold=3.626914143562317
node_382: feature_name=LDLR	LDLR.value <= threshold=2.571519374847412
node_383: feature_name=HLA-DRB1	HLA-DRB1.value > threshold=-0.7626054286956787

node_385: feature_name=CD79A	CD79A.value > threshold=2.857717990875244
node_387: feature_name=FCRL1	FCRL1.value <= threshold=6.850295543670654
node_388: feature_name=PDLIM1	PDLIM1.value <= threshold=4.4097795486450195
node_389: feature_name=MARCH1	MARCH1.value <= threshold=-0.35520173609256744
node_390: feature_name=ICAM1	ICAM1.value <= threshold=-0.33725936710834503
node_391: feature_name=HES4	HES4.value <= threshold=3.0498239994049072
node_392: feature_name=HLA-DRB5	HLA-DRB5.value > threshold=-0.7931511402130127
node_394: feature_name=STMN1	STMN1.value <= threshold=2.673145294189453
node_395: feature_name=HBB	HBB.value <= threshold=1.8341119289398193
node_396: feature_name=FAM129C	FAM129C.value <= threshold=4.626989841461182
Class: COVID-19 Critical	
Rules_58	passed counts:79
node_0: feature_name=LTB	LTB.value > threshold=0.994031548500061
node_5264: feature_name=MT-CO1	MT-CO1.value > threshold=-0.4127991199493408
node_5638: feature_name=OAZ1	OAZ1.value > threshold=-0.2640867084264755
node_6112: feature_name=IFI44L	IFI44L.value <= threshold=2.5893263816833496
node_6113: feature_name=TCL1A	TCL1A.value <= threshold=4.201832294464111
node_6114: feature_name=HLA-DQA2	HLA-DQA2.value <= threshold=-0.2270243540406227
node_6115: feature_name=MTRNR2L2	MTRNR2L2.value <= threshold=2.548917293548584
node_6116: feature_name=ATF5	ATF5.value <= threshold=2.041263461112976
node_6117: feature_name=JUN	JUN.value <= threshold=-0.616259902715683
node_6118: feature_name=NFKBID	NFKBID.value <= threshold=1.5264258980751038
node_6119: feature_name=MT-CO1	MT-CO1.value > threshold=0.1564532369375229
node_6133: feature_name=LTB	LTB.value > threshold=1.2431999444961548
node_6149: feature_name=MT-CO1	MT-CO1.value <= threshold=1.1254711747169495
node_6150: feature_name=RP5-821D11.7	RP5-821D11.7.value <= threshold=2.683489441871643
node_6151: feature_name=SLC7A5	SLC7A5.value <= threshold=1.7370842099189758
node_6152: feature_name=OAZ1	OAZ1.value > threshold=-0.12006772682070732
node_6156: feature_name=ICAM1	ICAM1.value <= threshold=0.616551861166954

Class: Healthy Control	
Rules_59	passed counts:78
node_0: feature_name=LTB	LTB.value <= threshold=0.994031548500061
node_1: feature_name=MTRNR2L2	MTRNR2L2.value <= threshold=2.8784297704696655
node_2: feature_name=TCL1A	TCL1A.value <= threshold=4.798312664031982
node_3: feature_name=MT-CO1	MT-CO1.value > threshold=0.08947191759943962
node_2521: feature_name=LTB	LTB.value <= threshold=0.6038667261600494
node_2522: feature_name=HLA-DQB1	HLA-DQB1.value <= threshold=2.1053054332733154
node_2523: feature_name=SNX9	SNX9.value <= threshold=-0.5346486568450928
node_2524: feature_name=HLA-DPB1	HLA-DPB1.value <= threshold=1.9548270106315613
node_2525: feature_name=FAM129C	FAM129C.value <= threshold=3.3393388986587524
node_2526: feature_name=XIST	XIST.value <= threshold=3.6911416053771973
node_2527: feature_name=NR4A1	NR4A1.value <= threshold=-0.2557755410671234
node_2528: feature_name=HOPX	HOPX.value <= threshold=2.776473879814148
node_2529: feature_name=AREGB	AREGB.value <= threshold=-0.10896047577261925
node_2530: feature_name=KIAA0125	KIAA0125.value <= threshold=7.652706623077393
node_2531: feature_name=MT-CO3	MT-CO3.value > threshold=-1.3726763725280762
node_2533: feature_name=TNF	TNF.value <= threshold=3.1101887226104736
node_2534: feature_name=NFKBID	NFKBID.value <= threshold=-0.39706873893737793
node_2535: feature_name=KIAA0125	KIAA0125.value > threshold=-0.17713353037834167
node_2543: feature_name=XAF1	XAF1.value <= threshold=1.7887146472930908
node_2544: feature_name=TLR2	TLR2.value <= threshold=2.253062665462494
node_2545: feature_name=HLA-DRB5	HLA-DRB5.value <= threshold=3.099335193634033
node_2546: feature_name=HLA-DQB1	HLA-DQB1.value <= threshold=2.057031512260437
node_2547: feature_name=CCL3	CCL3.value <= threshold=2.8681111335754395
node_2548: feature_name=MIAT	MIAT.value <= threshold=8.097615242004395
node_2549: feature_name=NT5C3A	NT5C3A.value <= threshold=3.9882057905197144
node_2550: feature_name=PLD4	PLD4.value <= threshold=2.694515687879175
node_2551: feature_name=MT-ND6	MT-ND6.value <= threshold=2.745527148246765

Class: COVID-19 Critical	
Rules_60	passed counts:77
node_0: feature_name=LTB	LTB.value <= threshold=0.994031548500061
node_1: feature_name=MTRNR2L2	MTRNR2L2.value <= threshold=2.8784297704696655
node_2: feature_name=TCL1A	TCL1A.value <= threshold=4.798312664031982
node_3: feature_name=MT-CO1	MT-CO1.value > threshold=0.08947191759943962
node_2521: feature_name=LTB	LTB.value > threshold=0.6038667261600494
node_4173: feature_name=OAZ1	OAZ1.value <= threshold=0.143283873796463
node_4174: feature_name=PLD4	PLD4.value <= threshold=2.921932101249695
node_4175: feature_name=XIST	XIST.value <= threshold=-0.05067425221204758
node_4176: feature_name=MT-CO1	MT-CO1.value <= threshold=1.345594048500061
node_4177: feature_name=HLA-DQB1	HLA-DQB1.value > threshold=1.927736520767212
node_4353: feature_name=ITM2C	ITM2C.value <= threshold=-0.12329206243157387
node_4354: feature_name=MX1	MX1.value > threshold=-0.4356398284435272
node_4430: feature_name=CD83	CD83.value <= threshold=3.929547071456909
node_4431: feature_name=SNX9	SNX9.value <= threshold=3.212221622467041
node_4432: feature_name=SYNE1	SYNE1.value <= threshold=1.6369847059249878
node_4433: feature_name=ID1	ID1.value <= threshold=0.8923406675457954
node_4434: feature_name=MEF2A	MEF2A.value > threshold=-0.4683011621236801
node_4438: feature_name=HLA-DQB1	HLA-DQB1.value > threshold=1.9348734021186829
Class: COVID-19 Critical	
Rules_61	passed counts:77
node_0: feature_name=LTB	LTB.value <= threshold=0.994031548500061
node_1: feature_name=MTRNR2L2	MTRNR2L2.value <= threshold=2.8784297704696655
node_2: feature_name=TCL1A	TCL1A.value <= threshold=4.798312664031982
node_3: feature_name=MT-CO1	MT-CO1.value <= threshold=0.08947191759943962
node_4: feature_name=NR4A1	NR4A1.value > threshold=1.0725348591804504
node_1622: feature_name=HLA-DQA2	HLA-DQA2.value > threshold=1.9496816992759705

node_2350: feature_name=BCL2A1	BCL2A1.value <= threshold=1.1322240233421326
node_2351: feature_name=CD83	CD83.value > threshold=2.3162450790405273
node_2391: feature_name=CD79B	CD79B.value <= threshold=1.7111427783966064
node_2392: feature_name=TTN	TTN.value <= threshold=-0.28553803265094757
node_2393: feature_name=LDLRAD4	LDLRAD4.value <= threshold=3.3328956365585327
node_2394: feature_name=BCL2A1	BCL2A1.value > threshold=-0.5811292827129364
node_2404: feature_name=SLC7A5	SLC7A5.value <= threshold=1.3126214146614075
node_2405: feature_name=AP3M2	AP3M2.value <= threshold=1.617304801940918
node_2406: feature_name=MT-ND6	MT-ND6.value <= threshold=0.7549090385437012
node_2407: feature_name=HLA-DPB1	HLA-DPB1.value <= threshold=2.3294938802719116
node_2408: feature_name=ZNF10	ZNF10.value > threshold=-0.3644643574953079
node_2410: feature_name=RNF144B	RNF144B.value <= threshold=3.029694676399231
node_2411: feature_name=RP11-685N3.1	RP11-685N3.1.value <= threshold=5.6112189292907715
Class: COVID-19 Severe	
Rules_62	passed counts:76
node_0: feature_name=LTB	LTB.value > threshold=0.994031548500061
node_5264: feature_name=MT-CO1	MT-CO1.value > threshold=-0.4127991199493408
node_5638: feature_name=OAZ1	OAZ1.value <= threshold=-0.2640867084264755
node_5639: feature_name=CD79B	CD79B.value <= threshold=2.2998517751693726
node_5640: feature_name=MTRNR2L2	MTRNR2L2.value <= threshold=3.5499863624572754
node_5641: feature_name=MT-CO1	MT-CO1.value > threshold=0.22567133605480194
node_5729: feature_name=MARCH1	MARCH1.value <= threshold=1.5154643654823303
node_5730: feature_name=MT-CO1	MT-CO1.value <= threshold=1.7823500633239746
node_5731: feature_name=HLA-DPB1	HLA-DPB1.value <= threshold=2.0096163749694824
node_5732: feature_name=NR4A3	NR4A3.value <= threshold=3.509189486503601
node_5733: feature_name=OAZ1	OAZ1.value <= threshold=-0.4392261207103729
node_5734: feature_name=SESN3	SESN3.value > threshold=-0.45661941170692444
node_5786: feature_name=ANO6	ANO6.value <= threshold=1.6851763129234314
node_5787: feature_name=RALGPS2	RALGPS2.value <= threshold=5.7725324630737305

node_5788: feature_name=XIST	XIST.value <= threshold=2.581266798079014
node_5789: feature_name=WDR74	WDR74.value > threshold=-0.5116851031780243
node_5791: feature_name=TCL1A	TCL1A.value <= threshold=5.243391036987305
node_5792: feature_name=IL8	IL8.value <= threshold=0.2682276964187622
Class: COVID-19 Critical	
Rules_63	passed counts:76
node_0: feature_name=LTB	LTB.value <= threshold=0.994031548500061
node_1: feature_name=MTRNR2L2	MTRNR2L2.value <= threshold=2.8784297704696655
node_2: feature_name=TCL1A	TCL1A.value <= threshold=4.798312664031982
node_3: feature_name=MT-CO1	MT-CO1.value <= threshold=0.08947191759943962
node_4: feature_name=NR4A1	NR4A1.value > threshold=1.0725348591804504
node_1622: feature_name=HLA-DQA2	HLA-DQA2.value > threshold=1.9496816992759705
node_2350: feature_name=BCL2A1	BCL2A1.value <= threshold=1.1322240233421326
node_2351: feature_name=CD83	CD83.value <= threshold=2.3162450790405273
node_2352: feature_name=LTB	LTB.value <= threshold=0.7341383993625641
node_2353: feature_name=TCL1A	TCL1A.value <= threshold=3.942829728126526
node_2354: feature_name=MARCKS	MARCKS.value <= threshold=2.9345329999923706
node_2355: feature_name=ACSL1	ACSL1.value <= threshold=2.564934253692627
node_2356: feature_name=HLA-DQB1	HLA-DQB1.value > threshold=-0.0751032829284668
node_2360: feature_name=FTH1	FTH1.value > threshold=-0.976179450750351
node_2364: feature_name=ISG15	ISG15.value <= threshold=2.5562429428100586
node_2365: feature_name=NR4A3	NR4A3.value <= threshold=2.5293972492218018
node_2366: feature_name=RBKS	RBKS.value <= threshold=4.449531316757202
node_2367: feature_name=MYL9	MYL9.value <= threshold=1.8219271362759173
Class: COVID-19 Critical	
Rules_64	passed counts:76
node_0: feature_name=LTB	LTB.value <= threshold=0.994031548500061
node_1: feature_name=MTRNR2L2	MTRNR2L2.value <= threshold=2.8784297704696655

node_2: feature_name=TCL1A	TCL1A.value <= threshold=4.798312664031982
node_3: feature_name=MT-CO1	MT-CO1.value <= threshold=0.08947191759943962
node_4: feature_name=NR4A1	NR4A1.value <= threshold=1.0725348591804504
node_5: feature_name=HLA-DPA1	HLA-DPA1.value > threshold=1.6885469555854797
node_861: feature_name=CD83	CD83.value <= threshold=3.0563642978668213
node_862: feature_name=HLA-DQB1	HLA-DQB1.value <= threshold=2.61081063747406
node_863: feature_name=MTRNR2L2	MTRNR2L2.value <= threshold=2.0919997692108154
node_864: feature_name=BCL2A1	BCL2A1.value <= threshold=1.852618932723999
node_865: feature_name=CD79B	CD79B.value <= threshold=4.352142333984375
node_866: feature_name=TNFAIP3	TNFAIP3.value <= threshold=-0.0449496041983366
node_867: feature_name=MT-CO1	MT-CO1.value <= threshold=-0.08531957119703293
node_868: feature_name=MTRNR2L8	MTRNR2L8.value <= threshold=0.8770350813865662
node_869: feature_name=EGR1	EGR1.value > threshold=-0.32270750403404236
node_1015: feature_name=NR4A3	NR4A3.value <= threshold=1.9085431098937988
node_1016: feature_name=ID3	ID3.value <= threshold=2.776647686958313
node_1017: feature_name=XIST	XIST.value <= threshold=2.618046522140503
node_1018: feature_name=CD79B	CD79B.value <= threshold=3.818994164466858
node_1019: feature_name=NFKBID	NFKBID.value <= threshold=3.4191306829452515
node_1020: feature_name=MIAT	MIAT.value <= threshold=3.532225251197815
node_1021: feature_name=STAG3	STAG3.value <= threshold=9.534917831420898
node_1022: feature_name=OASL	OASL.value <= threshold=2.3923760652542114
node_1023: feature_name=HLA-DQB1	HLA-DQB1.value <= threshold=2.572031259536743
node_1024: feature_name=CD79A	CD79A.value > threshold=0.1879405975341797
node_1028: feature_name=NR4A1	NR4A1.value <= threshold=0.7387348711490631
node_1029: feature_name=SLC7A5	SLC7A5.value <= threshold=2.0677570104599
node_1030: feature_name=HLA-DPB1	HLA-DPB1.value <= threshold=2.17708420753479
node_1031: feature_name=MARCH1	MARCH1.value <= threshold=4.443183898925781
node_1032: feature_name=ATF5	ATF5.value <= threshold=6.411617755889893
node_1033: feature_name=MYL9	MYL9.value <= threshold=3.8704605102539062
node_1034: feature_name=LTB	LTB.value <= threshold=0.9670123755931854

node_1035: feature_name=PLD4	PLD4.value <= threshold=7.310958623886108
node_1036: feature_name=CTD-2035E11.3	CTD-2035E11.3.value <= threshold=2.9847536087036133
node_1037: feature_name=PDLIM1	PDLIM1.value <= threshold=-0.278710201382637
node_1038: feature_name=TTN	TTN.value > threshold=-0.3065446764230728
node_1042: feature_name=FAM160B1	FAM160B1.value <= threshold=1.677986741065979
node_1043: feature_name=IRAK3	IRAK3.value <= threshold=1.8925572633743286
node_1044: feature_name=MARCKS	MARCKS.value <= threshold=3.0563639402389526
Class: COVID-19 Critical	
Rules_65	passed counts:75
node_0: feature_name=LTB	LTB.value <= threshold=0.994031548500061
node_1: feature_name=MTRNR2L2	MTRNR2L2.value <= threshold=2.8784297704696655
node_2: feature_name=TCL1A	TCL1A.value <= threshold=4.798312664031982
node_3: feature_name=MT-CO1	MT-CO1.value <= threshold=0.08947191759943962
node_4: feature_name=NR4A1	NR4A1.value > threshold=1.0725348591804504
node_1622: feature_name=HLA-DQA2	HLA-DQA2.value <= threshold=1.9496816992759705
node_1623: feature_name=HLA-DRB5	HLA-DRB5.value <= threshold=2.715766429901123
node_1624: feature_name=HLA-DPA1	HLA-DPA1.value <= threshold=1.7050301432609558
node_1625: feature_name=CD83	CD83.value > threshold=2.783489227294922
node_1873: feature_name=IFI44L	IFI44L.value > threshold=1.5938722491264343
node_2047: feature_name=RNF11	RNF11.value <= threshold=2.110256612300873
node_2048: feature_name=HLA-DQA1	HLA-DQA1.value > threshold=-0.395967960357666
Class: COVID-19 Critical	
Rules_66	passed counts:73
node_0: feature_name=LTB	LTB.value <= threshold=0.994031548500061
node_1: feature_name=MTRNR2L2	MTRNR2L2.value <= threshold=2.8784297704696655
node_2: feature_name=TCL1A	TCL1A.value <= threshold=4.798312664031982
node_3: feature_name=MT-CO1	MT-CO1.value <= threshold=0.08947191759943962
node_4: feature_name=NR4A1	NR4A1.value <= threshold=1.0725348591804504

node_5: feature_name=HLA-DPA1	HLA-DPA1.value > threshold=1.6885469555854797
node_861: feature_name=CD83	CD83.value > threshold=3.0563642978668213
node_1539: feature_name=ID1	ID1.value <= threshold=1.4626862406730652
node_1540: feature_name=FTH1	FTH1.value <= threshold=0.2985198348760605
node_1541: feature_name=SESN3	SESN3.value <= threshold=2.7280690670013428
node_1542: feature_name=HOPX	HOPX.value <= threshold=-0.46606285870075226
node_1543: feature_name=ATF3	ATF3.value <= threshold=1.6206613183021545
node_1544: feature_name=HLA-DQA1	HLA-DQA1.value <= threshold=2.5666390657424927
node_1545: feature_name=STMN1	STMN1.value <= threshold=0.9797938093543053
node_1546: feature_name=MEF2C	MEF2C.value <= threshold=4.716001033782959
node_1547: feature_name=BCL2A1	BCL2A1.value <= threshold=2.306094765663147
node_1548: feature_name=FAM160B1	FAM160B1.value <= threshold=3.3123955726623535
node_1549: feature_name=MYL9	MYL9.value <= threshold=3.9540880918502808
node_1550: feature_name=STAG3	STAG3.value <= threshold=8.4168062210083
Class: COVID-19 Critical	
Rules_67	passed counts:70
node_0: feature_name=LTB	LTB.value <= threshold=0.994031548500061
node_1: feature_name=MTRNR2L2	MTRNR2L2.value <= threshold=2.8784297704696655
node_2: feature_name=TCL1A	TCL1A.value <= threshold=4.798312664031982
node_3: feature_name=MT-CO1	MT-CO1.value > threshold=0.08947191759943962
node_2521: feature_name=LTB	LTB.value > threshold=0.6038667261600494
node_4173: feature_name=OAZ1	OAZ1.value > threshold=0.143283873796463
node_4615: feature_name=NR4A1	NR4A1.value <= threshold=0.6621004343032837
node_4616: feature_name=NFKBID	NFKBID.value > threshold=3.283741593360901
node_4804: feature_name=FAM129C	FAM129C.value <= threshold=6.735232830047607
node_4805: feature_name=ATF3	ATF3.value <= threshold=3.1994469165802
node_4806: feature_name=CD83	CD83.value <= threshold=4.217104196548462
node_4807: feature_name=CYBB	CYBB.value <= threshold=4.050122618675232
node_4808: feature_name=RP11-731F5.2	RP11-731F5.2.value <= threshold=5.314566373825073

node_4809: feature_name=SESN3	SESN3.value <= threshold=4.074076771736145
Class: COVID-19 Critical	
Rules_68	passed counts:70
node_0: feature_name=LTB	LTB.value <= threshold=0.994031548500061
node_1: feature_name=MTRNR2L2	MTRNR2L2.value <= threshold=2.8784297704696655
node_2: feature_name=TCL1A	TCL1A.value <= threshold=4.798312664031982
node_3: feature_name=MT-CO1	MT-CO1.value > threshold=0.08947191759943962
node_2521: feature_name=LTB	LTB.value > threshold=0.6038667261600494
node_4173: feature_name=OAZ1	OAZ1.value > threshold=0.143283873796463
node_4615: feature_name=NR4A1	NR4A1.value <= threshold=0.6621004343032837
node_4616: feature_name=NFKBID	NFKBID.value <= threshold=3.283741593360901
node_4617: feature_name=HLA-DQA1	HLA-DQA1.value <= threshold=2.3435752391815186
node_4618: feature_name=PELI1	PELI1.value > threshold=1.3271203637123108
node_4680: feature_name=BANK1	BANK1.value > threshold=-0.3607018291950226
node_4682: feature_name=RGCC	RGCC.value <= threshold=0.60355344414711
node_4683: feature_name=SNX29P2	SNX29P2.value <= threshold=4.192138910293579
node_4684: feature_name=XIST	XIST.value <= threshold=2.375336170196533
node_4685: feature_name=TCF4	TCF4.value <= threshold=2.2667007446289062
node_4686: feature_name=FTH1	FTH1.value <= threshold=0.9238187372684479
node_4687: feature_name=SESN3	SESN3.value > threshold=-0.4816848784685135
node_4689: feature_name=NT5C3A	NT5C3A.value <= threshold=3.1949219703674316
node_4690: feature_name=STMN1	STMN1.value <= threshold=2.830788731575012
node_4691: feature_name=COTL1	COTL1.value > threshold=-1.5321864485740662
Class: COVID-19 Critical	
Rules_69	passed counts:70
node_0: feature_name=LTB	LTB.value <= threshold=0.994031548500061
node_1: feature_name=MTRNR2L2	MTRNR2L2.value <= threshold=2.8784297704696655
node_2: feature_name=TCL1A	TCL1A.value <= threshold=4.798312664031982

node_3: feature_name=MT-CO1	MT-CO1.value > threshold=0.08947191759943962
node_2521: feature_name=LTB	LTB.value <= threshold=0.6038667261600494
node_2522: feature_name=HLA-DQB1	HLA-DQB1.value <= threshold=2.1053054332733154
node_2523: feature_name=SNX9	SNX9.value > threshold=-0.5346486568450928
node_2907: feature_name=NR4A1	NR4A1.value <= threshold=2.5433837175369263
node_2908: feature_name=XIST	XIST.value <= threshold=0.7664470989257097
node_2909: feature_name=CD79B	CD79B.value <= threshold=2.9326847791671753
node_2910: feature_name=NR4A1	NR4A1.value > threshold=-0.2875557690858841
node_3096: feature_name=NR4A3	NR4A3.value <= threshold=3.314968705177307
node_3097: feature_name=VPREB3	VPREB3.value <= threshold=3.3549938201904297
node_3098: feature_name=RALGPS2	RALGPS2.value <= threshold=5.807050704956055
node_3099: feature_name=CTD-2035E11.3	CTD-2035E11.3.value <= threshold=6.4090416431427
node_3100: feature_name=FAM129C	FAM129C.value <= threshold=6.966361045837402
node_3101: feature_name=HES4	HES4.value <= threshold=4.139557242393494
node_3102: feature_name=CD83	CD83.value > threshold=1.8343153595924377
node_3136: feature_name=PLD4	PLD4.value <= threshold=4.453821897506714
node_3137: feature_name=HLA-DQA2	HLA-DQA2.value > threshold=-0.10758286714553833
node_3229: feature_name=FAM160B1	FAM160B1.value <= threshold=1.61697119474411
node_3230: feature_name=BCL2A1	BCL2A1.value <= threshold=0.9140156805515289
node_3231: feature_name=IFI6	IFI6.value <= threshold=0.9741227328777313
node_3232: feature_name=MTRNR2L8	MTRNR2L8.value <= threshold=3.1325559616088867
node_3233: feature_name=TLN1	TLN1.value <= threshold=2.827813982963562
node_3234: feature_name=ATF5	ATF5.value <= threshold=5.219146251678467
node_3235: feature_name=HBB	HBB.value <= threshold=7.074732303619385
node_3236: feature_name=STMN1	STMN1.value <= threshold=3.467090129852295
Class: COVID-19 Critical	
Rules_70	passed counts:70
node_0: feature_name=LTB	LTB.value <= threshold=0.994031548500061
node_1: feature_name=MTRNR2L2	MTRNR2L2.value <= threshold=2.8784297704696655

node_2: feature_name=TCL1A	TCL1A.value <= threshold=4.798312664031982
node_3: feature_name=MT-CO1	MT-CO1.value <= threshold=0.08947191759943962
node_4: feature_name=NR4A1	NR4A1.value > threshold=1.0725348591804504
node_1622: feature_name=HLA-DQA2	HLA-DQA2.value <= threshold=1.9496816992759705
node_1623: feature_name=HLA-DRB5	HLA-DRB5.value <= threshold=2.715766429901123
node_1624: feature_name=HLA-DPA1	HLA-DPA1.value <= threshold=1.7050301432609558
node_1625: feature_name=CD83	CD83.value <= threshold=2.783489227294922
node_1626: feature_name=XIST	XIST.value <= threshold=0.9589543007314205
node_1627: feature_name=PLD4	PLD4.value <= threshold=5.865932464599609
node_1628: feature_name=OAZ1	OAZ1.value <= threshold=0.6137169003486633
node_1629: feature_name=MTRNR2L2	MTRNR2L2.value <= threshold=1.6102792620658875
node_1630: feature_name=IFI6	IFI6.value > threshold=-0.8404486179351807
node_1632: feature_name=NR4A1	NR4A1.value > threshold=1.0916320085525513
node_1638: feature_name=DENND3	DENND3.value <= threshold=3.988452672958374
node_1639: feature_name=HLA-DRB1	HLA-DRB1.value > threshold=1.4092575907707214
node_1749: feature_name=HLA-DQB1	HLA-DQB1.value <= threshold=3.4929215908050537
node_1750: feature_name=RP11-685N3.1	RP11-685N3.1.value <= threshold=9.97533893585205
node_1751: feature_name=RGS1	RGS1.value > threshold=-0.5999330878257751
node_1765: feature_name=MT-ND6	MT-ND6.value <= threshold=2.4838690757751465
node_1766: feature_name=DAPK1	DAPK1.value <= threshold=4.920811414718628
node_1767: feature_name=RGS1	RGS1.value <= threshold=3.3605728149414062
node_1768: feature_name=ANO6	ANO6.value <= threshold=4.00420081615448
node_1769: feature_name=FTH1	FTH1.value <= threshold=1.6050249934196472
node_1770: feature_name=APP	APP.value <= threshold=2.417694091796875
node_1771: feature_name=ITGAX	ITGAX.value <= threshold=2.950524091720581
node_1772: feature_name=PDLIM1	PDLIM1.value <= threshold=5.856243848800659
node_1773: feature_name=RELT	RELT.value <= threshold=2.891391396522522
node_1774: feature_name=RHOB	RHOB.value <= threshold=3.2315229177474976
node_1775: feature_name=NR4A1	NR4A1.value > threshold=1.125234842300415
node_1781: feature_name=LTB	LTB.value > threshold=0.7502643764019012

node_1787: feature_name=LTB	LTB.value > threshold=0.7552008926868439
node_1791: feature_name=ID1	ID1.value <= threshold=3.2292819023132324
node_1792: feature_name=MARCKS	MARCKS.value <= threshold=4.350169897079468
node_1793: feature_name=HLA-DQA1	HLA-DQA1.value <= threshold=3.0671645402908325
node_1794: feature_name=MS4A1	MS4A1.value <= threshold=3.1135826110839844
Class: COVID-19 Critical	
Rules_71	passed counts:69
node_0: feature_name=LTB	LTB.value > threshold=0.994031548500061
node_5264: feature_name=MT-CO1	MT-CO1.value > threshold=-0.4127991199493408
node_5638: feature_name=OAZ1	OAZ1.value > threshold=-0.2640867084264755
node_6112: feature_name=IFI44L	IFI44L.value <= threshold=2.5893263816833496
node_6113: feature_name=TCL1A	TCL1A.value <= threshold=4.201832294464111
node_6114: feature_name=HLA-DQA2	HLA-DQA2.value > threshold=-0.2270243540406227
node_6542: feature_name=CD79B	CD79B.value > threshold=3.096232771873474
node_6872: feature_name=ICAM1	ICAM1.value <= threshold=-0.2232399433851242
node_6873: feature_name=STAG3	STAG3.value > threshold=-0.19312061369419098
node_6939: feature_name=HLA-DRB5	HLA-DRB5.value <= threshold=2.209904909133911
node_6940: feature_name=STAG3	STAG3.value <= threshold=7.8730878829956055
node_6941: feature_name=FAM129C	FAM129C.value > threshold=1.668714702129364
node_6953: feature_name=ENDOD1	ENDOD1.value <= threshold=2.171262264251709
node_6954: feature_name=ATG2A	ATG2A.value <= threshold=2.183647572994232
node_6955: feature_name=IRF8	IRF8.value <= threshold=3.6009104251861572
Class: Healthy Control	
Rules_72	passed counts:69
node_0: feature_name=LTB	LTB.value > threshold=0.994031548500061
node_5264: feature_name=MT-CO1	MT-CO1.value <= threshold=-0.4127991199493408
node_5265: feature_name=MTRNR2L2	MTRNR2L2.value <= threshold=2.4473838806152344
node_5266: feature_name=TCL1A	TCL1A.value <= threshold=5.787256956100464

node_5267: feature_name=PLD4	PLD4.value <= threshold=3.544657826423645
node_5268: feature_name=NR4A1	NR4A1.value > threshold=0.8490704298019409
node_5498: feature_name=HLA-DPA1	HLA-DPA1.value <= threshold=1.9079211950302124
node_5499: feature_name=ID3	ID3.value <= threshold=-0.17505786567926407
node_5500: feature_name=PPIF	PPIF.value > threshold=-0.5180355906486511
node_5512: feature_name=PDLIM1	PDLIM1.value <= threshold=2.5938297510147095
node_5513: feature_name=HLA-DRB5	HLA-DRB5.value > threshold=-0.5597687363624573
node_5519: feature_name=MARCKS	MARCKS.value > threshold=-0.4281132072210312
node_5521: feature_name=ILK	ILK.value <= threshold=2.261121392250061
node_5522: feature_name=LTB	LTB.value <= threshold=1.790786623954773
Class: COVID-19 Critical	
Rules_73	passed counts:69
node_0: feature_name=LTB	LTB.value <= threshold=0.994031548500061
node_1: feature_name=MTRNR2L2	MTRNR2L2.value <= threshold=2.8784297704696655
node_2: feature_name=TCL1A	TCL1A.value > threshold=4.798312664031982
node_4912: feature_name=MT-CO1	MT-CO1.value > threshold=-0.3664844036102295
node_5002: feature_name=IFI44L	IFI44L.value > threshold=2.3101747035980225
node_5198: feature_name=MX1	MX1.value > threshold=-0.4572612792253494
node_5200: feature_name=HSPA1A	HSPA1A.value <= threshold=4.940417528152466
node_5201: feature_name=LDLRAD4	LDLRAD4.value <= threshold=5.265421390533447
node_5202: feature_name=OAZ1	OAZ1.value > threshold=-1.4393892884254456
Class: COVID-19 Critical	
Rules_74	passed counts:69
node_0: feature_name=LTB	LTB.value <= threshold=0.994031548500061
node_1: feature_name=MTRNR2L2	MTRNR2L2.value <= threshold=2.8784297704696655
node_2: feature_name=TCL1A	TCL1A.value <= threshold=4.798312664031982
node_3: feature_name=MT-CO1	MT-CO1.value > threshold=0.08947191759943962
node_2521: feature_name=LTB	LTB.value <= threshold=0.6038667261600494

node_2522: feature_name=HLA-DQB1	HLA-DQB1.value <= threshold=2.1053054332733154
node_2523: feature_name=SNX9	SNX9.value > threshold=-0.5346486568450928
node_2907: feature_name=NR4A1	NR4A1.value <= threshold=2.5433837175369263
node_2908: feature_name=XIST	XIST.value <= threshold=0.7664470989257097
node_2909: feature_name=CD79B	CD79B.value <= threshold=2.9326847791671753
node_2910: feature_name=NR4A1	NR4A1.value <= threshold=-0.2875557690858841
node_2911: feature_name=CTD-2035E11.3	CTD-2035E11.3.value <= threshold=5.262048006057739
node_2912: feature_name=VPREB3	VPREB3.value > threshold=4.937594890594482
node_3072: feature_name=LTB	LTB.value <= threshold=0.5297379791736603
node_3073: feature_name=HLA-DPB1	HLA-DPB1.value > threshold=-0.7085362374782562
node_3075: feature_name=GNG11	GNG11.value <= threshold=1.377760574221611
node_3076: feature_name=PLD4	PLD4.value <= threshold=6.698240280151367
node_3077: feature_name=LTB	LTB.value > threshold=-1.0393452048301697
node_3081: feature_name=FTH1	FTH1.value <= threshold=1.2618587613105774
node_3082: feature_name=MT1E	MT1E.value <= threshold=1.5809853002429008
node_3083: feature_name=CXorf57	CXorf57.value <= threshold=1.89571101218462
Class: COVID-19 Critical	
Rules_75	passed counts:68
node_0: feature_name=LTB	LTB.value <= threshold=0.994031548500061
node_1: feature_name=MTRNR2L2	MTRNR2L2.value <= threshold=2.8784297704696655
node_2: feature_name=TCL1A	TCL1A.value <= threshold=4.798312664031982
node_3: feature_name=MT-CO1	MT-CO1.value <= threshold=0.08947191759943962
node_4: feature_name=NR4A1	NR4A1.value <= threshold=1.0725348591804504
node_5: feature_name=HLA-DPA1	HLA-DPA1.value <= threshold=1.6885469555854797
node_6: feature_name=HLA-DRB5	HLA-DRB5.value <= threshold=2.1886194944381714
node_7: feature_name=XIST	XIST.value <= threshold=-0.12328458577394485
node_8: feature_name=SYNE1	SYNE1.value > threshold=-0.7835594415664673
node_16: feature_name=MTRNR2L2	MTRNR2L2.value <= threshold=2.4483247995376587
node_17: feature_name=HLA-DQA2	HLA-DQA2.value > threshold=2.227178931236267

node_591: feature_name=BCL2A1	BCL2A1.value <= threshold=1.0648428201675415
node_592: feature_name=FTH1	FTH1.value <= threshold=0.8842596709728241
node_593: feature_name=SNX9	SNX9.value <= threshold=3.232524275779724
node_594: feature_name=OAZ1	OAZ1.value <= threshold=1.704061508178711
node_595: feature_name=MT-CYB	MT-CYB.value <= threshold=0.5040059983730316
node_596: feature_name=CD83	CD83.value > threshold=2.535894751548767
node_638: feature_name=MTRNR2L8	MTRNR2L8.value <= threshold=1.581753134727478
node_639: feature_name=FCER2	FCER2.value > threshold=-0.253058984875679
node_643: feature_name=ID3	ID3.value <= threshold=2.6175442934036255
node_644: feature_name=PELI1	PELI1.value <= threshold=2.353822112083435
node_645: feature_name=HOPX	HOPX.value <= threshold=1.0554000735282898
node_646: feature_name=OASL	OASL.value <= threshold=1.1027036309242249
Class: COVID-19 Critical	
Rules_76	passed counts:67
node_0: feature_name=LTB	LTB.value <= threshold=0.994031548500061
node_1: feature_name=MTRNR2L2	MTRNR2L2.value <= threshold=2.8784297704696655
node_2: feature_name=TCL1A	TCL1A.value <= threshold=4.798312664031982
node_3: feature_name=MT-CO1	MT-CO1.value > threshold=0.08947191759943962
node_2521: feature_name=LTB	LTB.value <= threshold=0.6038667261600494
node_2522: feature_name=HLA-DQB1	HLA-DQB1.value > threshold=2.1053054332733154
node_3550: feature_name=XIST	XIST.value <= threshold=0.7716337237507105
node_3551: feature_name=CD79B	CD79B.value <= threshold=2.9426796436309814
node_3552: feature_name=HLA-DQB1	HLA-DQB1.value > threshold=2.713674306869507
node_3854: feature_name=MEF2C	MEF2C.value > threshold=-0.3721291869878769
node_3882: feature_name=IFI44L	IFI44L.value <= threshold=2.5516022443771362
node_3883: feature_name=FCER2	FCER2.value <= threshold=-0.21487552672624588
node_3884: feature_name=HLA-DQA2	HLA-DQA2.value > threshold=-0.12580305337905884
node_3954: feature_name=SNX9	SNX9.value > threshold=-0.5673478543758392
node_3964: feature_name=MX1	MX1.value <= threshold=3.7867738008499146

node_3965: feature_name=ZNF10	ZNF10.value <= threshold=7.291811943054199
node_3966: feature_name=MARCH1	MARCH1.value <= threshold=6.366525888442993
node_3967: feature_name=PDLIM1	PDLIM1.value <= threshold=6.026795864105225
node_3968: feature_name=SPI1	SPI1.value <= threshold=2.3112276792526245
Class: COVID-19 Critical	
Rules_77	passed counts:67
node_0: feature_name=LTB	LTB.value <= threshold=0.994031548500061
node_1: feature_name=MTRNR2L2	MTRNR2L2.value <= threshold=2.8784297704696655
node_2: feature_name=TCL1A	TCL1A.value <= threshold=4.798312664031982
node_3: feature_name=MT-CO1	MT-CO1.value <= threshold=0.08947191759943962
node_4: feature_name=NR4A1	NR4A1.value <= threshold=1.0725348591804504
node_5: feature_name=HLA-DPA1	HLA-DPA1.value > threshold=1.6885469555854797
node_861: feature_name=CD83	CD83.value <= threshold=3.0563642978668213
node_862: feature_name=HLA-DQB1	HLA-DQB1.value <= threshold=2.61081063747406
node_863: feature_name=MTRNR2L2	MTRNR2L2.value <= threshold=2.0919997692108154
node_864: feature_name=BCL2A1	BCL2A1.value <= threshold=1.852618932723999
node_865: feature_name=CD79B	CD79B.value <= threshold=4.352142333984375
node_866: feature_name=TNFAIP3	TNFAIP3.value <= threshold=-0.0449496041983366
node_867: feature_name=MT-CO1	MT-CO1.value <= threshold=-0.08531957119703293
node_868: feature_name=MTRNR2L8	MTRNR2L8.value <= threshold=0.8770350813865662
node_869: feature_name=EGR1	EGR1.value <= threshold=-0.32270750403404236
node_870: feature_name=IGJ	IGJ.value <= threshold=4.618318557739258
node_871: feature_name=DUSP4	DUSP4.value <= threshold=2.8197922706604004
node_872: feature_name=HOPX	HOPX.value <= threshold=2.10397732257843
node_873: feature_name=RGCC	RGCC.value <= threshold=1.2070720195770264
node_874: feature_name=OAZ1	OAZ1.value > threshold=1.02116060256958
node_980: feature_name=IFIT3	IFIT3.value > threshold=-0.2093840092420578
node_986: feature_name=ID1	ID1.value <= threshold=0.9667705297470093
node_987: feature_name=HLA-DQB1	HLA-DQB1.value > threshold=-0.5031426399946213

node_989: feature_name=BANK1	BANK1.value <= threshold=4.076449275016785
node_990: feature_name=DAPK1	DAPK1.value <= threshold=2.0046065635979176
node_991: feature_name=HLA-DRB1	HLA-DRB1.value <= threshold=2.4354681968688965
node_992: feature_name=APP	APP.value <= threshold=3.4017211198806763
Class: COVID-19 Critical	
Rules_78	passed counts:66
node_0: feature_name=LTB	LTB.value <= threshold=0.994031548500061
node_1: feature_name=MTRNR2L2	MTRNR2L2.value <= threshold=2.8784297704696655
node_2: feature_name=TCL1A	TCL1A.value <= threshold=4.798312664031982
node_3: feature_name=MT-CO1	MT-CO1.value > threshold=0.08947191759943962
node_2521: feature_name=LTB	LTB.value <= threshold=0.6038667261600494
node_2522: feature_name=HLA-DQB1	HLA-DQB1.value > threshold=2.1053054332733154
node_3550: feature_name=XIST	XIST.value > threshold=0.7716337237507105
node_4140: feature_name=HLA-DQA2	HLA-DQA2.value <= threshold=-0.1354314684867859
node_4141: feature_name=MS4A1	MS4A1.value <= threshold=3.907364010810852
node_4142: feature_name=XAF1	XAF1.value <= threshold=2.253366529941559
node_4143: feature_name=HOPX	HOPX.value <= threshold=2.271597385406494
node_4144: feature_name=TCL1A	TCL1A.value <= threshold=4.321073532104492
node_4145: feature_name=PDLIM1	PDLIM1.value <= threshold=3.787937045097351
Class: COVID-19 Severe	
Rules_79	passed counts:66
node_0: feature_name=LTB	LTB.value <= threshold=0.994031548500061
node_1: feature_name=MTRNR2L2	MTRNR2L2.value <= threshold=2.8784297704696655
node_2: feature_name=TCL1A	TCL1A.value <= threshold=4.798312664031982
node_3: feature_name=MT-CO1	MT-CO1.value > threshold=0.08947191759943962
node_2521: feature_name=LTB	LTB.value <= threshold=0.6038667261600494
node_2522: feature_name=HLA-DQB1	HLA-DQB1.value <= threshold=2.1053054332733154
node_2523: feature_name=SNX9	SNX9.value > threshold=-0.5346486568450928

node_2907: feature_name=NR4A1	NR4A1.value <= threshold=2.5433837175369263
node_2908: feature_name=XIST	XIST.value <= threshold=0.7664470989257097
node_2909: feature_name=CD79B	CD79B.value <= threshold=2.9326847791671753
node_2910: feature_name=NR4A1	NR4A1.value > threshold=-0.2875557690858841
node_3096: feature_name=NR4A3	NR4A3.value <= threshold=3.314968705177307
node_3097: feature_name=VPREB3	VPREB3.value > threshold=3.3549938201904297
node_3285: feature_name=OAZ1	OAZ1.value <= threshold=0.11267071589827538
node_3286: feature_name=STMN1	STMN1.value <= threshold=2.106989860534668
node_3287: feature_name=PLD4	PLD4.value <= threshold=4.0678054094314575
node_3288: feature_name=APP	APP.value <= threshold=2.4729559421539307
node_3289: feature_name=JUN	JUN.value > threshold=-1.9935784935951233
node_3291: feature_name=MT-CO2	MT-CO2.value > threshold=-0.4252379387617111
node_3295: feature_name=CD79B	CD79B.value <= threshold=2.8821581602096558
node_3296: feature_name=SNX9	SNX9.value > threshold=-0.01219281554222107
node_3298: feature_name=HLA-DRB5	HLA-DRB5.value > threshold=-1.0200685262680054
Class: COVID-19 Critical	
Rules_80	passed counts:66
node_0: feature_name=LTB	LTB.value <= threshold=0.994031548500061
node_1: feature_name=MTRNR2L2	MTRNR2L2.value <= threshold=2.8784297704696655
node_2: feature_name=TCL1A	TCL1A.value <= threshold=4.798312664031982
node_3: feature_name=MT-CO1	MT-CO1.value <= threshold=0.08947191759943962
node_4: feature_name=NR4A1	NR4A1.value > threshold=1.0725348591804504
node_1622: feature_name=HLA-DQA2	HLA-DQA2.value <= threshold=1.9496816992759705
node_1623: feature_name=HLA-DRB5	HLA-DRB5.value <= threshold=2.715766429901123
node_1624: feature_name=HLA-DPA1	HLA-DPA1.value <= threshold=1.7050301432609558
node_1625: feature_name=CD83	CD83.value <= threshold=2.783489227294922
node_1626: feature_name=XIST	XIST.value <= threshold=0.9589543007314205
node_1627: feature_name=PLD4	PLD4.value <= threshold=5.865932464599609
node_1628: feature_name=OAZ1	OAZ1.value <= threshold=0.6137169003486633

node_1629: feature_name=MTRNR2L2	MTRNR2L2.value <= threshold=1.6102792620658875
node_1630: feature_name=IFI6	IFI6.value > threshold=-0.8404486179351807
node_1632: feature_name=NR4A1	NR4A1.value > threshold=1.0916320085525513
node_1638: feature_name=DENND3	DENND3.value <= threshold=3.988452672958374
node_1639: feature_name=HLA-DRB1	HLA-DRB1.value <= threshold=1.4092575907707214
node_1640: feature_name=HLA-DPA1	HLA-DPA1.value > threshold=1.2675696015357971
node_1708: feature_name=CYBB	CYBB.value <= threshold=1.8550206422805786
node_1709: feature_name=JUN	JUN.value > threshold=-0.04836222808808088
node_1723: feature_name=NT5C3A	NT5C3A.value <= threshold=1.9380813241004944
node_1724: feature_name=FAM129C	FAM129C.value <= threshold=4.6762073040008545
node_1725: feature_name=FERMT3	FERMT3.value <= threshold=1.1511099338531494
node_1726: feature_name=GPX1	GPX1.value <= threshold=0.10868745669722557
node_1727: feature_name=MTRNR2L8	MTRNR2L8.value <= threshold=3.6919054985046387
node_1728: feature_name=NAMPT	NAMPT.value <= threshold=2.033546030521393
node_1729: feature_name=HLA-DPA1	HLA-DPA1.value <= threshold=1.7016383409500122
Class: COVID-19 Critical	
Rules_81	passed counts:65
node_0: feature_name=LTB	LTB.value <= threshold=0.994031548500061
node_1: feature_name=MTRNR2L2	MTRNR2L2.value <= threshold=2.8784297704696655
node_2: feature_name=TCL1A	TCL1A.value <= threshold=4.798312664031982
node_3: feature_name=MT-CO1	MT-CO1.value > threshold=0.08947191759943962
node_2521: feature_name=LTB	LTB.value <= threshold=0.6038667261600494
node_2522: feature_name=HLA-DQB1	HLA-DQB1.value <= threshold=2.1053054332733154
node_2523: feature_name=SNX9	SNX9.value <= threshold=-0.5346486568450928
node_2524: feature_name=HLA-DPB1	HLA-DPB1.value <= threshold=1.9548270106315613
node_2525: feature_name=FAM129C	FAM129C.value <= threshold=3.3393388986587524
node_2526: feature_name=XIST	XIST.value <= threshold=3.6911416053771973
node_2527: feature_name=NR4A1	NR4A1.value > threshold=-0.2557755410671234
node_2625: feature_name=HLA-DPA1	HLA-DPA1.value > threshold=0.9335225224494934

node_2657: feature_name=RALGPS2	RALGPS2.value <= threshold=2.9413715600967407
node_2658: feature_name=HLA-DQB1	HLA-DQB1.value <= threshold=1.7503233551979065
node_2659: feature_name=TP53INP1	TP53INP1.value > threshold=-0.2649386376142502
node_2675: feature_name=VPREB3	VPREB3.value <= threshold=5.346607446670532
node_2676: feature_name=MT-CO3	MT-CO3.value > threshold=-0.4287712126970291
node_2678: feature_name=NR4A1	NR4A1.value <= threshold=3.887486696243286
node_2679: feature_name=ITM2C	ITM2C.value <= threshold=3.06315279006958
node_2680: feature_name=MAP3K2	MAP3K2.value <= threshold=2.1286245584487915
node_2681: feature_name=NR4A3	NR4A3.value <= threshold=2.2177510857582092
Class: COVID-19 Critical	
Rules_82	passed counts:63
node_0: feature_name=LTB	LTB.value > threshold=0.994031548500061
node_5264: feature_name=MT-CO1	MT-CO1.value <= threshold=-0.4127991199493408
node_5265: feature_name=MTRNR2L2	MTRNR2L2.value > threshold=2.4473838806152344
Class: COVID-19 Severe	
Rules_83	passed counts:63
node_0: feature_name=LTB	LTB.value <= threshold=0.994031548500061
node_1: feature_name=MTRNR2L2	MTRNR2L2.value <= threshold=2.8784297704696655
node_2: feature_name=TCL1A	TCL1A.value <= threshold=4.798312664031982
node_3: feature_name=MT-CO1	MT-CO1.value <= threshold=0.08947191759943962
node_4: feature_name=NR4A1	NR4A1.value > threshold=1.0725348591804504
node_1622: feature_name=HLA-DQA2	HLA-DQA2.value <= threshold=1.9496816992759705
node_1623: feature_name=HLA-DRB5	HLA-DRB5.value > threshold=2.715766429901123
node_2337: feature_name=HLA-DRB1	HLA-DRB1.value <= threshold=1.9765487909317017
node_2338: feature_name=VCL	VCL.value > threshold=-0.3584357351064682
node_2340: feature_name=MT-CYB	MT-CYB.value <= threshold=0.6708013713359833
Class: COVID-19 Severe	

Rules_84	passed counts:63
node_0: feature_name=LTB	LTB.value <= threshold=0.994031548500061
node_1: feature_name=MTRNR2L2	MTRNR2L2.value <= threshold=2.8784297704696655
node_2: feature_name=TCL1A	TCL1A.value <= threshold=4.798312664031982
node_3: feature_name=MT-CO1	MT-CO1.value <= threshold=0.08947191759943962
node_4: feature_name=NR4A1	NR4A1.value <= threshold=1.0725348591804504
node_5: feature_name=HLA-DPA1	HLA-DPA1.value <= threshold=1.6885469555854797
node_6: feature_name=HLA-DRB5	HLA-DRB5.value <= threshold=2.1886194944381714
node_7: feature_name=XIST	XIST.value <= threshold=-0.12328458577394485
node_8: feature_name=SYNE1	SYNE1.value > threshold=-0.7835594415664673
node_16: feature_name=MTRNR2L2	MTRNR2L2.value <= threshold=2.4483247995376587
node_17: feature_name=HLA-DQA2	HLA-DQA2.value <= threshold=2.227178931236267
node_18: feature_name=NR4A1	NR4A1.value <= threshold=-0.21380723267793655
node_19: feature_name=IRAK3	IRAK3.value <= threshold=4.27159571647644
node_20: feature_name=IFIT2	IFIT2.value <= threshold=7.8175740242004395
node_21: feature_name=CD79A	CD79A.value <= threshold=2.857242226600647
node_22: feature_name=HIST1H2AC	HIST1H2AC.value <= threshold=5.077470302581787
node_23: feature_name=FTH1	FTH1.value <= threshold=0.4158056080341339
node_24: feature_name=ATF3	ATF3.value <= threshold=3.8028353452682495
node_25: feature_name=OASL	OASL.value <= threshold=3.387580633163452
node_26: feature_name=MT-CO1	MT-CO1.value <= threshold=-0.7942646145820618
node_27: feature_name=FCER2	FCER2.value <= threshold=7.268347978591919
node_28: feature_name=CCL3	CCL3.value <= threshold=3.2822797298431396
node_29: feature_name=BCL2A1	BCL2A1.value <= threshold=3.1504160165786743
node_30: feature_name=GRAP2	GRAP2.value <= threshold=3.0517499446868896
node_31: feature_name=EGR1	EGR1.value <= threshold=7.645251512527466
node_32: feature_name=GPX1	GPX1.value <= threshold=0.6752602756023407
node_33: feature_name=CD79B	CD79B.value <= threshold=4.365710258483887
node_34: feature_name=S100A6	S100A6.value <= threshold=0.4172801077365875
node_35: feature_name=ID3	ID3.value > threshold=3.093442916870117

node_45: feature_name=ID3	ID3.value > threshold=3.0995997190475464
node_47: feature_name=GRAP2	GRAP2.value <= threshold=0.7355649620294571
node_48: feature_name=ATF5	ATF5.value <= threshold=3.7072882652282715
Class: COVID-19 Critical	
Rules_85	passed counts:62
node_0: feature_name=LTB	LTB.value > threshold=0.994031548500061
node_5264: feature_name=MT-CO1	MT-CO1.value > threshold=-0.4127991199493408
node_5638: feature_name=OAZ1	OAZ1.value > threshold=-0.2640867084264755
node_6112: feature_name=IFI44L	IFI44L.value <= threshold=2.5893263816833496
node_6113: feature_name=TCL1A	TCL1A.value <= threshold=4.201832294464111
node_6114: feature_name=HLA-DQA2	HLA-DQA2.value <= threshold=-0.2270243540406227
node_6115: feature_name=MTRNR2L2	MTRNR2L2.value <= threshold=2.548917293548584
node_6116: feature_name=ATF5	ATF5.value > threshold=2.041263461112976
node_6490: feature_name=HLA-DPB1	HLA-DPB1.value <= threshold=1.5343759655952454
node_6491: feature_name=MT-ND6	MT-ND6.value <= threshold=1.0284687876701355
node_6492: feature_name=FAM129C	FAM129C.value <= threshold=3.5579545497894287
node_6493: feature_name=WDR74	WDR74.value <= threshold=1.888875424861908
node_6494: feature_name=HSP90B1	HSP90B1.value > threshold=-1.1869465708732605
Class: COVID-19 Critical	
Rules_86	passed counts:61
node_0: feature_name=LTB	LTB.value <= threshold=0.994031548500061
node_1: feature_name=MTRNR2L2	MTRNR2L2.value <= threshold=2.8784297704696655
node_2: feature_name=TCL1A	TCL1A.value <= threshold=4.798312664031982
node_3: feature_name=MT-CO1	MT-CO1.value > threshold=0.08947191759943962
node_2521: feature_name=LTB	LTB.value <= threshold=0.6038667261600494
node_2522: feature_name=HLA-DQB1	HLA-DQB1.value > threshold=2.1053054332733154
node_3550: feature_name=XIST	XIST.value <= threshold=0.7716337237507105
node_3551: feature_name=CD79B	CD79B.value > threshold=2.9426796436309814

node_4039: feature_name=IFI44L	IFI44L.value <= threshold=1.8637521862983704
node_4040: feature_name=SNX9	SNX9.value <= threshold=1.274232804775238
node_4041: feature_name=CASP8	CASP8.value <= threshold=-0.574097603559494
node_4042: feature_name=NFKBID	NFKBID.value <= threshold=1.49756920337677
node_4043: feature_name=MIAT	MIAT.value <= threshold=3.7295743227005005
node_4044: feature_name=HLA-DRB5	HLA-DRB5.value <= threshold=2.8214967250823975
node_4045: feature_name=OAZ1	OAZ1.value > threshold=-1.574858844280243
node_4047: feature_name=TP53INP1	TP53INP1.value <= threshold=5.462192058563232
node_4048: feature_name=STAG3	STAG3.value <= threshold=8.407716989517212
Class: Healthy Control	
Rules_87	passed counts:61
node_0: feature_name=LTB	LTB.value <= threshold=0.994031548500061
node_1: feature_name=MTRNR2L2	MTRNR2L2.value <= threshold=2.8784297704696655
node_2: feature_name=TCL1A	TCL1A.value <= threshold=4.798312664031982
node_3: feature_name=MT-CO1	MT-CO1.value <= threshold=0.08947191759943962
node_4: feature_name=NR4A1	NR4A1.value <= threshold=1.0725348591804504
node_5: feature_name=HLA-DPA1	HLA-DPA1.value <= threshold=1.6885469555854797
node_6: feature_name=HLA-DRB5	HLA-DRB5.value <= threshold=2.1886194944381714
node_7: feature_name=XIST	XIST.value <= threshold=-0.12328458577394485
node_8: feature_name=SYNE1	SYNE1.value > threshold=-0.7835594415664673
node_16: feature_name=MTRNR2L2	MTRNR2L2.value <= threshold=2.4483247995376587
node_17: feature_name=HLA-DQA2	HLA-DQA2.value <= threshold=2.227178931236267
node_18: feature_name=NR4A1	NR4A1.value <= threshold=-0.21380723267793655
node_19: feature_name=IRAK3	IRAK3.value <= threshold=4.27159571647644
node_20: feature_name=IFIT2	IFIT2.value <= threshold=7.8175740242004395
node_21: feature_name=CD79A	CD79A.value <= threshold=2.857242226600647
node_22: feature_name=HIST1H2AC	HIST1H2AC.value <= threshold=5.077470302581787
node_23: feature_name=FTH1	FTH1.value <= threshold=0.4158056080341339
node_24: feature_name=ATF3	ATF3.value <= threshold=3.8028353452682495

node_25: feature_name=OASL	OASL.value <= threshold=3.387580633163452
node_26: feature_name=MT-CO1	MT-CO1.value > threshold=-0.7942646145820618
node_74: feature_name=HOPX	HOPX.value > threshold=0.8682331740856171
node_250: feature_name=CD22	CD22.value > threshold=-0.23878339678049088
node_254: feature_name=MT-CO2	MT-CO2.value <= threshold=0.39438967406749725
node_255: feature_name=COTL1	COTL1.value > threshold=-0.0382253173738718
Class: COVID-19 Critical	
Rules_88	passed counts:60
node_0: feature_name=LTB	LTB.value <= threshold=0.994031548500061
node_1: feature_name=MTRNR2L2	MTRNR2L2.value <= threshold=2.8784297704696655
node_2: feature_name=TCL1A	TCL1A.value <= threshold=4.798312664031982
node_3: feature_name=MT-CO1	MT-CO1.value <= threshold=0.08947191759943962
node_4: feature_name=NR4A1	NR4A1.value > threshold=1.0725348591804504
node_1622: feature_name=HLA-DQA2	HLA-DQA2.value <= threshold=1.9496816992759705
node_1623: feature_name=HLA-DRB5	HLA-DRB5.value <= threshold=2.715766429901123
node_1624: feature_name=HLA-DPA1	HLA-DPA1.value <= threshold=1.7050301432609558
node_1625: feature_name=CD83	CD83.value <= threshold=2.783489227294922
node_1626: feature_name=XIST	XIST.value <= threshold=0.9589543007314205
node_1627: feature_name=PLD4	PLD4.value <= threshold=5.865932464599609
node_1628: feature_name=OAZ1	OAZ1.value > threshold=0.6137169003486633
node_1840: feature_name=MT-CO1	MT-CO1.value <= threshold=-0.28350088000297546
node_1841: feature_name=AHR	AHR.value <= threshold=1.9306254982948303
node_1842: feature_name=SYNE1	SYNE1.value <= threshold=0.17462080717086792
Class: COVID-19 Critical	
Rules_89	passed counts:58
node_0: feature_name=LTB	LTB.value > threshold=0.994031548500061
node_5264: feature_name=MT-CO1	MT-CO1.value > threshold=-0.4127991199493408
node_5638: feature_name=OAZ1	OAZ1.value > threshold=-0.2640867084264755

node_6112: feature_name=IFI44L	IFI44L.value <= threshold=2.5893263816833496
node_6113: feature_name=TCL1A	TCL1A.value <= threshold=4.201832294464111
node_6114: feature_name=HLA-DQA2	HLA-DQA2.value <= threshold=-0.2270243540406227
node_6115: feature_name=MTRNR2L2	MTRNR2L2.value <= threshold=2.548917293548584
node_6116: feature_name=ATF5	ATF5.value <= threshold=2.041263461112976
node_6117: feature_name=JUN	JUN.value > threshold=-0.616259902715683
node_6189: feature_name=MT-CO3	MT-CO3.value <= threshold=0.13483521342277527
node_6190: feature_name=XAF1	XAF1.value <= threshold=2.584952235221863
node_6191: feature_name=HLA-DRB5	HLA-DRB5.value > threshold=0.6955901980400085
node_6239: feature_name=TNFAIP3	TNFAIP3.value <= threshold=0.11930759996175766
node_6240: feature_name=TP53INP1	TP53INP1.value <= threshold=2.9654784202575684
node_6241: feature_name=HLA-DRB5	HLA-DRB5.value <= threshold=2.5141202211380005
node_6242: feature_name=NFKBID	NFKBID.value <= threshold=3.358878970146179
node_6243: feature_name=JARID2	JARID2.value <= threshold=1.7160060405731201
node_6244: feature_name=HLA-DPA1	HLA-DPA1.value > threshold=0.49491266906261444
node_6246: feature_name=MIR24-2	MIR24-2.value <= threshold=1.8348549008369446
node_6247: feature_name=HSP90B1	HSP90B1.value <= threshold=1.8861054182052612
node_6248: feature_name=NR4A1	NR4A1.value <= threshold=-0.5434737503528595
node_6249: feature_name=MT-CO1	MT-CO1.value > threshold=0.002460678806528449
node_6261: feature_name=PLD4	PLD4.value <= threshold=5.953886985778809
node_6262: feature_name=STAG3	STAG3.value <= threshold=6.350517749786377
node_6263: feature_name=AHR	AHR.value <= threshold=1.2266070246696472
node_6264: feature_name=ITM2C	ITM2C.value <= threshold=3.805675506591797
Class: Healthy Control	
Rules_90	passed counts:58
node_0: feature_name=LTB	LTB.value <= threshold=0.994031548500061
node_1: feature_name=MTRNR2L2	MTRNR2L2.value <= threshold=2.8784297704696655
node_2: feature_name=TCL1A	TCL1A.value <= threshold=4.798312664031982
node_3: feature_name=MT-CO1	MT-CO1.value <= threshold=0.08947191759943962

node_4: feature_name=NR4A1	NR4A1.value <= threshold=1.0725348591804504
node_5: feature_name=HLA-DPA1	HLA-DPA1.value <= threshold=1.6885469555854797
node_6: feature_name=HLA-DRB5	HLA-DRB5.value <= threshold=2.1886194944381714
node_7: feature_name=XIST	XIST.value <= threshold=-0.12328458577394485
node_8: feature_name=SYNE1	SYNE1.value > threshold=-0.7835594415664673
node_16: feature_name=MTRNR2L2	MTRNR2L2.value <= threshold=2.4483247995376587
node_17: feature_name=HLA-DQA2	HLA-DQA2.value <= threshold=2.227178931236267
node_18: feature_name=NR4A1	NR4A1.value <= threshold=-0.21380723267793655
node_19: feature_name=IRAK3	IRAK3.value <= threshold=4.27159571647644
node_20: feature_name=IFIT2	IFIT2.value <= threshold=7.8175740242004395
node_21: feature_name=CD79A	CD79A.value > threshold=2.857242226600647
node_375: feature_name=LTB	LTB.value > threshold=0.7405790388584137
node_473: feature_name=MS4A1	MS4A1.value <= threshold=2.8195884227752686
node_474: feature_name=HLA-DQA1	HLA-DQA1.value > threshold=-0.44217973947525024
node_478: feature_name=IGLL5	IGLL5.value > threshold=-0.25717009603977203
node_480: feature_name=RGS2	RGS2.value <= threshold=1.253660798072815
node_481: feature_name=PTGS1	PTGS1.value <= threshold=2.5437903478741646
Class: COVID-19 Critical	
Rules_91	passed counts:57
node_0: feature_name=LTB	LTB.value > threshold=0.994031548500061
node_5264: feature_name=MT-CO1	MT-CO1.value > threshold=-0.4127991199493408
node_5638: feature_name=OAZ1	OAZ1.value <= threshold=-0.2640867084264755
node_5639: feature_name=CD79B	CD79B.value <= threshold=2.2998517751693726
node_5640: feature_name=MTRNR2L2	MTRNR2L2.value <= threshold=3.5499863624572754
node_5641: feature_name=MT-CO1	MT-CO1.value > threshold=0.22567133605480194
node_5729: feature_name=MARCH1	MARCH1.value > threshold=1.5154643654823303
node_5867: feature_name=NFKBID	NFKBID.value <= threshold=2.6947624683380127
node_5868: feature_name=UTY	UTY.value <= threshold=3.29777729511261
node_5869: feature_name=IFI44L	IFI44L.value <= threshold=-0.29640993475914

node_5870: feature_name=FTH1	FTH1.value <= threshold=0.6966870129108429
node_5871: feature_name=ILK	ILK.value <= threshold=1.5719919204711914
node_5872: feature_name=MARCKS	MARCKS.value <= threshold=1.20343416929245
node_5873: feature_name=GRAP2	GRAP2.value <= threshold=3.6710673570632935
node_5874: feature_name=AHR	AHR.value <= threshold=2.3661739826202393
node_5875: feature_name=SNX9	SNX9.value <= threshold=3.658568263053894
Class: Healthy Control	
Rules_92	passed counts:57
node_0: feature_name=LTB	LTB.value <= threshold=0.994031548500061
node_1: feature_name=MTRNR2L2	MTRNR2L2.value <= threshold=2.8784297704696655
node_2: feature_name=TCL1A	TCL1A.value <= threshold=4.798312664031982
node_3: feature_name=MT-CO1	MT-CO1.value > threshold=0.08947191759943962
node_2521: feature_name=LTB	LTB.value > threshold=0.6038667261600494
node_4173: feature_name=OAZ1	OAZ1.value <= threshold=0.143283873796463
node_4174: feature_name=PLD4	PLD4.value > threshold=2.921932101249695
node_4558: feature_name=HLA-DQA2	HLA-DQA2.value <= threshold=1.5485953092575073
node_4559: feature_name=COTL1	COTL1.value > threshold=-0.42844848334789276
node_4573: feature_name=CD83	CD83.value > threshold=1.9224650263786316
node_4593: feature_name=IFIT2	IFIT2.value <= threshold=2.8800936937332153
node_4594: feature_name=EGR1	EGR1.value <= threshold=5.897513151168823
Class: Healthy Control	
Rules_93	passed counts:57
node_0: feature_name=LTB	LTB.value <= threshold=0.994031548500061
node_1: feature_name=MTRNR2L2	MTRNR2L2.value <= threshold=2.8784297704696655
node_2: feature_name=TCL1A	TCL1A.value <= threshold=4.798312664031982
node_3: feature_name=MT-CO1	MT-CO1.value <= threshold=0.08947191759943962
node_4: feature_name=NR4A1	NR4A1.value > threshold=1.0725348591804504
node_1622: feature_name=HLA-DQA2	HLA-DQA2.value <= threshold=1.9496816992759705

node_1623: feature_name=HLA-DRB5	HLA-DRB5.value <= threshold=2.715766429901123
node_1624: feature_name=HLA-DPA1	HLA-DPA1.value > threshold=1.7050301432609558
node_2056: feature_name=OAZ1	OAZ1.value > threshold=0.456103190779686
node_2266: feature_name=XAF1	XAF1.value <= threshold=-0.4431648850440979
node_2267: feature_name=MT-CO1	MT-CO1.value > threshold=-0.3505740910768509
node_2293: feature_name=LTB	LTB.value > threshold=-0.31744880229234695
node_2301: feature_name=CD79A	CD79A.value > threshold=2.299592971801758
node_2307: feature_name=HLA-DRB1	HLA-DRB1.value <= threshold=2.3275738954544067
node_2308: feature_name=AP3M2	AP3M2.value <= threshold=1.6965308785438538
Class: Healthy Control	
Rules_94	passed counts:56
node_0: feature_name=LTB	LTB.value > threshold=0.994031548500061
node_5264: feature_name=MT-CO1	MT-CO1.value > threshold=-0.4127991199493408
node_5638: feature_name=OAZ1	OAZ1.value > threshold=-0.2640867084264755
node_6112: feature_name=IFI44L	IFI44L.value <= threshold=2.5893263816833496
node_6113: feature_name=TCL1A	TCL1A.value <= threshold=4.201832294464111
node_6114: feature_name=HLA-DQA2	HLA-DQA2.value <= threshold=-0.2270243540406227
node_6115: feature_name=MTRNR2L2	MTRNR2L2.value <= threshold=2.548917293548584
node_6116: feature_name=ATF5	ATF5.value > threshold=2.041263461112976
node_6490: feature_name=HLA-DPB1	HLA-DPB1.value > threshold=1.5343759655952454
node_6508: feature_name=OAZ1	OAZ1.value > threshold=0.21525950729846954
node_6514: feature_name=NT5C3A	NT5C3A.value > threshold=-0.556839257478714
node_6516: feature_name=S100A6	S100A6.value > threshold=-1.8120158910751343
node_6518: feature_name=NFKBID	NFKBID.value <= threshold=3.4249380826950073
node_6519: feature_name=XAF1	XAF1.value <= threshold=3.1939691305160522
node_6520: feature_name=MT1E	MT1E.value <= threshold=-0.21073266118764877
node_6521: feature_name=CD79B	CD79B.value <= threshold=4.386048793792725
node_6522: feature_name=MT-CYB	MT-CYB.value > threshold=-1.8555787801742554
node_6524: feature_name=HLA-DPA1	HLA-DPA1.value > threshold=1.061785340309143

Class: Healthy Control	
Rules_95	passed counts:56
node_0: feature_name=LTB	LTB.value > threshold=0.994031548500061
node_5264: feature_name=MT-CO1	MT-CO1.value > threshold=-0.4127991199493408
node_5638: feature_name=OAZ1	OAZ1.value <= threshold=-0.2640867084264755
node_5639: feature_name=CD79B	CD79B.value <= threshold=2.2998517751693726
node_5640: feature_name=MTRNR2L2	MTRNR2L2.value <= threshold=3.5499863624572754
node_5641: feature_name=MT-CO1	MT-CO1.value > threshold=0.22567133605480194
node_5729: feature_name=MARCH1	MARCH1.value <= threshold=1.5154643654823303
node_5730: feature_name=MT-CO1	MT-CO1.value <= threshold=1.7823500633239746
node_5731: feature_name=HLA-DPB1	HLA-DPB1.value <= threshold=2.0096163749694824
node_5732: feature_name=NR4A3	NR4A3.value <= threshold=3.509189486503601
node_5733: feature_name=OAZ1	OAZ1.value <= threshold=-0.4392261207103729
node_5734: feature_name=SESN3	SESN3.value <= threshold=-0.45661941170692444
node_5735: feature_name=WDR74	WDR74.value > threshold=-0.4893670231103897
node_5761: feature_name=FCER2	FCER2.value <= threshold=6.041448354721069
node_5762: feature_name=TCL1A	TCL1A.value <= threshold=-0.1774759516119957
node_5763: feature_name=LTB	LTB.value <= threshold=2.0274250507354736
node_5764: feature_name=MT-ND6	MT-ND6.value <= threshold=3.5135587453842163
node_5765: feature_name=TSPYL2	TSPYL2.value <= threshold=2.319188117980957
node_5766: feature_name=CCL3	CCL3.value <= threshold=0.9654240012168884
node_5767: feature_name=FAM129C	FAM129C.value <= threshold=8.283316135406494
node_5768: feature_name=LTB	LTB.value > threshold=1.006561040878296
node_5770: feature_name=VPREB3	VPREB3.value <= threshold=3.576285481452942
Class: COVID-19 Critical	
Rules_96	passed counts:56
node_0: feature_name=LTB	LTB.value <= threshold=0.994031548500061
node_1: feature_name=MTRNR2L2	MTRNR2L2.value <= threshold=2.8784297704696655

node_2: feature_name=TCL1A	TCL1A.value <= threshold=4.798312664031982
node_3: feature_name=MT-CO1	MT-CO1.value > threshold=0.08947191759943962
node_2521: feature_name=LTB	LTB.value <= threshold=0.6038667261600494
node_2522: feature_name=HLA-DQB1	HLA-DQB1.value <= threshold=2.1053054332733154
node_2523: feature_name=SNX9	SNX9.value <= threshold=-0.5346486568450928
node_2524: feature_name=HLA-DPB1	HLA-DPB1.value > threshold=1.9548270106315613
node_2814: feature_name=CD79B	CD79B.value > threshold=-0.28186772763729095
node_2826: feature_name=MT-ND6	MT-ND6.value > threshold=0.17273088544607162
node_2872: feature_name=IFI44L	IFI44L.value <= threshold=-0.31519079208374023
node_2873: feature_name=RALGPS2	RALGPS2.value > threshold=1.6798744797706604
node_2887: feature_name=HLA-DQA2	HLA-DQA2.value <= threshold=5.12495756149292
node_2888: feature_name=MS4A1	MS4A1.value <= threshold=3.8452248573303223
node_2889: feature_name=PLEC	PLEC.value <= threshold=2.50480055809021
node_2890: feature_name=ATF3	ATF3.value <= threshold=1.5363580584526062
node_2891: feature_name=CYBB	CYBB.value > threshold=-0.7289507687091827
node_2893: feature_name=MT-CO2	MT-CO2.value > threshold=-1.0604701340198517
Class: Healthy Control	
Rules_97	passed counts:55
node_0: feature_name=LTB	LTB.value <= threshold=0.994031548500061
node_1: feature_name=MTRNR2L2	MTRNR2L2.value <= threshold=2.8784297704696655
node_2: feature_name=TCL1A	TCL1A.value <= threshold=4.798312664031982
node_3: feature_name=MT-CO1	MT-CO1.value <= threshold=0.08947191759943962
node_4: feature_name=NR4A1	NR4A1.value <= threshold=1.0725348591804504
node_5: feature_name=HLA-DPA1	HLA-DPA1.value > threshold=1.6885469555854797
node_861: feature_name=CD83	CD83.value <= threshold=3.0563642978668213
node_862: feature_name=HLA-DQB1	HLA-DQB1.value <= threshold=2.61081063747406
node_863: feature_name=MTRNR2L2	MTRNR2L2.value <= threshold=2.0919997692108154
node_864: feature_name=BCL2A1	BCL2A1.value <= threshold=1.852618932723999
node_865: feature_name=CD79B	CD79B.value <= threshold=4.352142333984375

node_866: feature_name=TNFAIP3	TNFAIP3.value <= threshold=-0.0449496041983366
node_867: feature_name=MT-CO1	MT-CO1.value <= threshold=-0.08531957119703293
node_868: feature_name=MTRNR2L8	MTRNR2L8.value <= threshold=0.8770350813865662
node_869: feature_name=EGR1	EGR1.value <= threshold=-0.32270750403404236
node_870: feature_name=IGJ	IGJ.value <= threshold=4.618318557739258
node_871: feature_name=DUSP4	DUSP4.value <= threshold=2.8197922706604004
node_872: feature_name=HOPX	HOPX.value <= threshold=2.10397732257843
node_873: feature_name=RGCC	RGCC.value <= threshold=1.2070720195770264
node_874: feature_name=OAZ1	OAZ1.value <= threshold=1.02116060256958
node_875: feature_name=NAIP	NAIP.value <= threshold=3.768904685974121
node_876: feature_name=LDLR	LDLR.value <= threshold=2.9582698345184326
node_877: feature_name=HLA-DPA1	HLA-DPA1.value <= threshold=3.0588693618774414
node_878: feature_name=RHOB	RHOB.value <= threshold=5.08755898475647
node_879: feature_name=SYTL3	SYTL3.value <= threshold=1.5127440690994263
node_880: feature_name=FTH1	FTH1.value > threshold=-1.4152275919914246
node_882: feature_name=CCL3	CCL3.value <= threshold=2.253131687641144
node_883: feature_name=CD83	CD83.value <= threshold=2.8566592931747437
node_884: feature_name=HLA-DRB5	HLA-DRB5.value > threshold=-0.6464254856109619
node_888: feature_name=HSP90B1	HSP90B1.value > threshold=-1.186358630657196
node_892: feature_name=ATG2A	ATG2A.value <= threshold=2.3624722957611084
node_893: feature_name=ICAM1	ICAM1.value <= threshold=2.4306468963623047
node_894: feature_name=CD38	CD38.value <= threshold=3.4977954626083374
node_895: feature_name=HLA-DRB1	HLA-DRB1.value > threshold=0.28603747487068176
node_899: feature_name=XIST	XIST.value <= threshold=1.4272559583187103
node_900: feature_name=BANK1	BANK1.value <= threshold=5.060973644256592
node_901: feature_name=IRF4	IRF4.value <= threshold=6.117950916290283
node_902: feature_name=MTRNR2L2	MTRNR2L2.value <= threshold=1.8410876989364624
node_903: feature_name=TPM1	TPM1.value <= threshold=4.590091228485107
node_904: feature_name=SNORD3B-2	SNORD3B-2.value <= threshold=4.349201917648315
node_905: feature_name=AHR	AHR.value <= threshold=2.268427610397339

node_906: feature_name=OAZ1	OAZ1.value <= threshold=-1.4369714260101318
node_907: feature_name=PELI1	PELI1.value <= threshold=1.937936544418335
node_908: feature_name=MT-CO3	MT-CO3.value > threshold=-1.687304139137268
Class: COVID-19 Critical	
Rules_98	passed counts:53
node_0: feature_name=LTB	LTB.value <= threshold=0.994031548500061
node_1: feature_name=MTRNR2L2	MTRNR2L2.value <= threshold=2.8784297704696655
node_2: feature_name=TCL1A	TCL1A.value <= threshold=4.798312664031982
node_3: feature_name=MT-CO1	MT-CO1.value > threshold=0.08947191759943962
node_2521: feature_name=LTB	LTB.value <= threshold=0.6038667261600494
node_2522: feature_name=HLA-DQB1	HLA-DQB1.value <= threshold=2.1053054332733154
node_2523: feature_name=SNX9	SNX9.value > threshold=-0.5346486568450928
node_2907: feature_name=NR4A1	NR4A1.value <= threshold=2.5433837175369263
node_2908: feature_name=XIST	XIST.value <= threshold=0.7664470989257097
node_2909: feature_name=CD79B	CD79B.value > threshold=2.9326847791671753
node_3353: feature_name=VPREB3	VPREB3.value <= threshold=3.0226117372512817
node_3354: feature_name=CD83	CD83.value > threshold=1.9074636101722717
node_3388: feature_name=BOD1L1	BOD1L1.value > threshold=-0.4742381125688553
node_3404: feature_name=RALGPS2	RALGPS2.value <= threshold=4.456665992736816
node_3405: feature_name=MEF2A	MEF2A.value > threshold=-0.43542933464050293
node_3409: feature_name=MMD	MMD.value <= threshold=3.3664941787719727
node_3410: feature_name=CTD-2035E11.3	CTD-2035E11.3.value <= threshold=3.5381768196821213
node_3411: feature_name=CD83	CD83.value > threshold=1.9435778260231018
Class: COVID-19 Critical	
Rules_99	passed counts:51
node_0: feature_name=LTB	LTB.value > threshold=0.994031548500061
node_5264: feature_name=MT-CO1	MT-CO1.value > threshold=-0.4127991199493408
node_5638: feature_name=OAZ1	OAZ1.value > threshold=-0.2640867084264755

node_6112: feature_name=IFI44L	IFI44L.value <= threshold=2.5893263816833496
node_6113: feature_name=TCL1A	TCL1A.value <= threshold=4.201832294464111
node_6114: feature_name=HLA-DQA2	HLA-DQA2.value <= threshold=-0.2270243540406227
node_6115: feature_name=MTRNR2L2	MTRNR2L2.value <= threshold=2.548917293548584
node_6116: feature_name=ATF5	ATF5.value <= threshold=2.041263461112976
node_6117: feature_name=JUN	JUN.value > threshold=-0.616259902715683
node_6189: feature_name=MT-CO3	MT-CO3.value <= threshold=0.13483521342277527
node_6190: feature_name=XAF1	XAF1.value <= threshold=2.584952235221863
node_6191: feature_name=HLA-DRB5	HLA-DRB5.value <= threshold=0.6955901980400085
node_6192: feature_name=NR4A1	NR4A1.value > threshold=-0.4496448189020157
node_6222: feature_name=HSP90B1	HSP90B1.value <= threshold=1.1745065450668335
node_6223: feature_name=NFKBID	NFKBID.value <= threshold=3.3718907833099365
node_6224: feature_name=CCDC141	CCDC141.value <= threshold=2.79733145236969
node_6225: feature_name=FAM160B1	FAM160B1.value <= threshold=1.5568975806236267
Class: Healthy Control	
Rules_100	passed counts:51
node_0: feature_name=LTB	LTB.value <= threshold=0.994031548500061
node_1: feature_name=MTRNR2L2	MTRNR2L2.value <= threshold=2.8784297704696655
node_2: feature_name=TCL1A	TCL1A.value <= threshold=4.798312664031982
node_3: feature_name=MT-CO1	MT-CO1.value <= threshold=0.08947191759943962
node_4: feature_name=NR4A1	NR4A1.value <= threshold=1.0725348591804504
node_5: feature_name=HLA-DPA1	HLA-DPA1.value > threshold=1.6885469555854797
node_861: feature_name=CD83	CD83.value <= threshold=3.0563642978668213
node_862: feature_name=HLA-DQB1	HLA-DQB1.value <= threshold=2.61081063747406
node_863: feature_name=MTRNR2L2	MTRNR2L2.value <= threshold=2.0919997692108154
node_864: feature_name=BCL2A1	BCL2A1.value <= threshold=1.852618932723999
node_865: feature_name=CD79B	CD79B.value <= threshold=4.352142333984375
node_866: feature_name=TNFAIP3	TNFAIP3.value > threshold=-0.0449496041983366
node_1236: feature_name=HLA-DRB5	HLA-DRB5.value > threshold=1.710871696472168

node_1268: feature_name=MT-CO1	MT-CO1.value > threshold=-0.8004095554351807
node_1282: feature_name=HLA-DQA2	HLA-DQA2.value > threshold=-0.1890905275940895
node_1296: feature_name=HSP90B1	HSP90B1.value <= threshold=1.671745777130127
node_1297: feature_name=ID3	ID3.value <= threshold=5.183009624481201
node_1298: feature_name=TCF4	TCF4.value <= threshold=4.291216850280762
Class: COVID-19 Critical	
Rules_101	passed counts:50
node_0: feature_name=LTB	LTB.value <= threshold=0.994031548500061
node_1: feature_name=MTRNR2L2	MTRNR2L2.value <= threshold=2.8784297704696655
node_2: feature_name=TCL1A	TCL1A.value <= threshold=4.798312664031982
node_3: feature_name=MT-CO1	MT-CO1.value <= threshold=0.08947191759943962
node_4: feature_name=NR4A1	NR4A1.value > threshold=1.0725348591804504
node_1622: feature_name=HLA-DQA2	HLA-DQA2.value <= threshold=1.9496816992759705
node_1623: feature_name=HLA-DRB5	HLA-DRB5.value <= threshold=2.715766429901123
node_1624: feature_name=HLA-DPA1	HLA-DPA1.value > threshold=1.7050301432609558
node_2056: feature_name=OAZ1	OAZ1.value <= threshold=0.456103190779686
node_2057: feature_name=HLA-DRB5	HLA-DRB5.value > threshold=0.6839984953403473
node_2101: feature_name=HLA-DRB1	HLA-DRB1.value > threshold=1.6199676990509033
node_2151: feature_name=HLA-DQB1	HLA-DQB1.value <= threshold=3.1961567401885986
node_2152: feature_name=PLD4	PLD4.value <= threshold=-0.03259922005236149
node_2153: feature_name=XIST	XIST.value <= threshold=-0.138966154307127
node_2154: feature_name=HLA-DQA1	HLA-DQA1.value <= threshold=2.0848299264907837
node_2155: feature_name=LTB	LTB.value > threshold=-0.9993145167827606
node_2161: feature_name=CYBB	CYBB.value <= threshold=1.514441728591919
node_2162: feature_name=CD83	CD83.value <= threshold=3.348180890083313
node_2163: feature_name=ID1	ID1.value <= threshold=1.9755518436431885
node_2164: feature_name=LDLRAD4	LDLRAD4.value <= threshold=3.572142720222473
node_2165: feature_name=BACH1	BACH1.value > threshold=-0.5848218500614166
node_2167: feature_name=MS4A1	MS4A1.value <= threshold=3.2828351259231567

node_2168: feature_name=CXorf57	CXorf57.value <= threshold=1.7314302325248718
Class: COVID-19 Critical	
Rules_102	passed counts:50
node_0: feature_name=LTB	LTB.value <= threshold=0.994031548500061
node_1: feature_name=MTRNR2L2	MTRNR2L2.value <= threshold=2.8784297704696655
node_2: feature_name=TCL1A	TCL1A.value <= threshold=4.798312664031982
node_3: feature_name=MT-CO1	MT-CO1.value <= threshold=0.08947191759943962
node_4: feature_name=NR4A1	NR4A1.value <= threshold=1.0725348591804504
node_5: feature_name=HLA-DPA1	HLA-DPA1.value > threshold=1.6885469555854797
node_861: feature_name=CD83	CD83.value <= threshold=3.0563642978668213
node_862: feature_name=HLA-DQB1	HLA-DQB1.value > threshold=2.61081063747406
node_1358: feature_name=HLA-DRB5	HLA-DRB5.value <= threshold=0.7542744874954224
node_1359: feature_name=MT-CYB	MT-CYB.value > threshold=-0.06432783603668213
node_1375: feature_name=RP11-731F5.2	RP11-731F5.2.value <= threshold=-0.09639483317732811
node_1376: feature_name=CD79B	CD79B.value <= threshold=3.379225730895996
node_1377: feature_name=ENDOD1	ENDOD1.value <= threshold=3.936734437942505
node_1378: feature_name=IL1B	IL1B.value <= threshold=1.4339272379875183
Class: COVID-19 Severe	
Rules_103	passed counts:48
node_0: feature_name=LTB	LTB.value <= threshold=0.994031548500061
node_1: feature_name=MTRNR2L2	MTRNR2L2.value <= threshold=2.8784297704696655
node_2: feature_name=TCL1A	TCL1A.value <= threshold=4.798312664031982
node_3: feature_name=MT-CO1	MT-CO1.value > threshold=0.08947191759943962
node_2521: feature_name=LTB	LTB.value > threshold=0.6038667261600494
node_4173: feature_name=OAZ1	OAZ1.value <= threshold=0.143283873796463
node_4174: feature_name=PLD4	PLD4.value <= threshold=2.921932101249695
node_4175: feature_name=XIST	XIST.value <= threshold=-0.05067425221204758
node_4176: feature_name=MT-CO1	MT-CO1.value <= threshold=1.345594048500061

node_4177: feature_name=HLA-DQB1	HLA-DQB1.value > threshold=1.927736520767212
node_4353: feature_name=ITM2C	ITM2C.value <= threshold=-0.12329206243157387
node_4354: feature_name=MX1	MX1.value <= threshold=-0.4356398284435272
node_4355: feature_name=HLA-DQB1	HLA-DQB1.value <= threshold=2.8865538835525513
node_4356: feature_name=SNX9	SNX9.value > threshold=1.002076417207718
node_4384: feature_name=HLA-DRB5	HLA-DRB5.value > threshold=1.0937854647636414
node_4396: feature_name=SLC7A5	SLC7A5.value <= threshold=1.3017424941062927
node_4397: feature_name=BACH1	BACH1.value <= threshold=2.4516913890838623
node_4398: feature_name=BANK1	BANK1.value <= threshold=3.624985694885254
node_4399: feature_name=CCR6	CCR6.value <= threshold=2.8670331239700317
node_4400: feature_name=FCRL1	FCRL1.value <= threshold=6.179790735244751
node_4401: feature_name=MS4A1	MS4A1.value <= threshold=3.6583468914031982
node_4402: feature_name=NFKBID	NFKBID.value <= threshold=4.10752272605896
Class: COVID-19 Critical	
Rules_104	passed counts:48
node_0: feature_name=LTB	LTB.value <= threshold=0.994031548500061
node_1: feature_name=MTRNR2L2	MTRNR2L2.value <= threshold=2.8784297704696655
node_2: feature_name=TCL1A	TCL1A.value <= threshold=4.798312664031982
node_3: feature_name=MT-CO1	MT-CO1.value > threshold=0.08947191759943962
node_2521: feature_name=LTB	LTB.value <= threshold=0.6038667261600494
node_2522: feature_name=HLA-DQB1	HLA-DQB1.value > threshold=2.1053054332733154
node_3550: feature_name=XIST	XIST.value <= threshold=0.7716337237507105
node_3551: feature_name=CD79B	CD79B.value <= threshold=2.9426796436309814
node_3552: feature_name=HLA-DQB1	HLA-DQB1.value > threshold=2.713674306869507
node_3854: feature_name=MEF2C	MEF2C.value > threshold=-0.3721291869878769
node_3882: feature_name=IFI44L	IFI44L.value > threshold=2.5516022443771362
node_4030: feature_name=DENND3	DENND3.value <= threshold=3.481819987297058
node_4031: feature_name=ATF5	ATF5.value > threshold=-0.397869735956192
node_4033: feature_name=BOD1L1	BOD1L1.value <= threshold=4.707000970840454

Class: COVID-19 Critical	
Rules_105	passed counts:48
node_0: feature_name=LTB	LTB.value <= threshold=0.994031548500061
node_1: feature_name=MTRNR2L2	MTRNR2L2.value <= threshold=2.8784297704696655
node_2: feature_name=TCL1A	TCL1A.value <= threshold=4.798312664031982
node_3: feature_name=MT-CO1	MT-CO1.value <= threshold=0.08947191759943962
node_4: feature_name=NR4A1	NR4A1.value <= threshold=1.0725348591804504
node_5: feature_name=HLA-DPA1	HLA-DPA1.value <= threshold=1.6885469555854797
node_6: feature_name=HLA-DRB5	HLA-DRB5.value <= threshold=2.1886194944381714
node_7: feature_name=XIST	XIST.value <= threshold=-0.12328458577394485
node_8: feature_name=SYNE1	SYNE1.value > threshold=-0.7835594415664673
node_16: feature_name=MTRNR2L2	MTRNR2L2.value <= threshold=2.4483247995376587
node_17: feature_name=HLA-DQA2	HLA-DQA2.value <= threshold=2.227178931236267
node_18: feature_name=NR4A1	NR4A1.value <= threshold=-0.21380723267793655
node_19: feature_name=IRAK3	IRAK3.value <= threshold=4.27159571647644
node_20: feature_name=IFIT2	IFIT2.value <= threshold=7.8175740242004395
node_21: feature_name=CD79A	CD79A.value <= threshold=2.857242226600647
node_22: feature_name=HIST1H2AC	HIST1H2AC.value <= threshold=5.077470302581787
node_23: feature_name=FTH1	FTH1.value <= threshold=0.4158056080341339
node_24: feature_name=ATF3	ATF3.value <= threshold=3.8028353452682495
node_25: feature_name=OASL	OASL.value <= threshold=3.387580633163452
node_26: feature_name=MT-CO1	MT-CO1.value > threshold=-0.7942646145820618
node_74: feature_name=HOPX	HOPX.value <= threshold=0.8682331740856171
node_75: feature_name=RGS2	RGS2.value <= threshold=1.4589954018592834
node_76: feature_name=AHR	AHR.value <= threshold=3.870481848716736
node_77: feature_name=IGJ	IGJ.value <= threshold=4.902882814407349
node_78: feature_name=CD79B	CD79B.value <= threshold=4.87716269493103
node_79: feature_name=HLA-DQB1	HLA-DQB1.value <= threshold=3.6925491094589233
node_80: feature_name=HLA-DRB5	HLA-DRB5.value <= threshold=2.0926843881607056

node_81: feature_name=MCTP1	MCTP1.value <= threshold=4.010472774505615
node_82: feature_name=GNG11	GNG11.value <= threshold=3.627470850944519
node_83: feature_name=HSP90B1	HSP90B1.value <= threshold=2.0835131406784058
node_84: feature_name=ATF3	ATF3.value > threshold=1.1142374873161316
node_178: feature_name=AHR	AHR.value <= threshold=1.9248778223991394
node_179: feature_name=MEF2C	MEF2C.value <= threshold=3.2153685092926025
node_180: feature_name=ATG2A	ATG2A.value <= threshold=1.9718633890151978
Class: COVID-19 Critical	
Rules_106	passed counts:47
node_0: feature_name=LTB	LTB.value > threshold=0.994031548500061
node_5264: feature_name=MT-CO1	MT-CO1.value > threshold=-0.4127991199493408
node_5638: feature_name=OAZ1	OAZ1.value > threshold=-0.2640867084264755
node_6112: feature_name=IFI44L	IFI44L.value <= threshold=2.5893263816833496
node_6113: feature_name=TCL1A	TCL1A.value <= threshold=4.201832294464111
node_6114: feature_name=HLA-DQA2	HLA-DQA2.value > threshold=-0.2270243540406227
node_6542: feature_name=CD79B	CD79B.value <= threshold=3.096232771873474
node_6543: feature_name=NR4A1	NR4A1.value <= threshold=1.421015739440918
node_6544: feature_name=LTB	LTB.value <= threshold=1.359755277633667
node_6545: feature_name=NFKBID	NFKBID.value <= threshold=1.4294711351394653
node_6546: feature_name=HLA-DRB1	HLA-DRB1.value > threshold=1.0398632287979126
node_6556: feature_name=CD79B	CD79B.value > threshold=-0.2919560521841049
node_6582: feature_name=PELI1	PELI1.value <= threshold=-0.6156984269618988
node_6583: feature_name=LTB	LTB.value > threshold=1.0177513360977173
node_6585: feature_name=CD22	CD22.value > threshold=-0.24851123988628387
node_6593: feature_name=HLA-DQB1	HLA-DQB1.value > threshold=-0.6246480345726013
node_6595: feature_name=UTY	UTY.value <= threshold=2.418617367744446
node_6596: feature_name=MMD	MMD.value <= threshold=2.8966281414031982
Class: Healthy Control	

Rules_107	passed counts:47
node_0: feature_name=LTB	LTB.value > threshold=0.994031548500061
node_5264: feature_name=MT-CO1	MT-CO1.value > threshold=-0.4127991199493408
node_5638: feature_name=OAZ1	OAZ1.value > threshold=-0.2640867084264755
node_6112: feature_name=IFI44L	IFI44L.value <= threshold=2.5893263816833496
node_6113: feature_name=TCL1A	TCL1A.value <= threshold=4.201832294464111
node_6114: feature_name=HLA-DQA2	HLA-DQA2.value <= threshold=-0.2270243540406227
node_6115: feature_name=MTRNR2L2	MTRNR2L2.value <= threshold=2.548917293548584
node_6116: feature_name=ATF5	ATF5.value <= threshold=2.041263461112976
node_6117: feature_name=JUN	JUN.value > threshold=-0.616259902715683
node_6189: feature_name=MT-CO3	MT-CO3.value > threshold=0.13483521342277527
node_6351: feature_name=XAF1	XAF1.value <= threshold=3.061717987060547
node_6352: feature_name=MT-CO2	MT-CO2.value <= threshold=1.2605181336402893
node_6353: feature_name=ICAM1	ICAM1.value <= threshold=-0.3340446650981903
node_6354: feature_name=OAZ1	OAZ1.value > threshold=-0.24055946618318558
node_6360: feature_name=PPIF	PPIF.value <= threshold=2.070030093193054
node_6361: feature_name=RALGPS2	RALGPS2.value > threshold=-0.3684315085411072
node_6369: feature_name=ANO6	ANO6.value <= threshold=3.504153251647949
node_6370: feature_name=YWHAH	YWHAH.value <= threshold=4.3182291984558105
node_6371: feature_name=LDLR	LDLR.value <= threshold=2.494291305541992
node_6372: feature_name=HLA-DQA1	HLA-DQA1.value > threshold=-0.5716990232467651
node_6374: feature_name=ABCA1	ABCA1.value <= threshold=3.7662240266799927
node_6375: feature_name=MS4A1	MS4A1.value > threshold=1.4995208978652954
node_6387: feature_name=SNORD3B-2	SNORD3B-2.value <= threshold=4.154061555862427
node_6388: feature_name=LTB	LTB.value <= threshold=2.1739100217819214
node_6389: feature_name=BACH1	BACH1.value <= threshold=2.0371285676956177
node_6390: feature_name=HLA-DQB1	HLA-DQB1.value <= threshold=3.0258322954177856
node_6391: feature_name=HLA-DPB1	HLA-DPB1.value > threshold=0.2591289207339287
node_6395: feature_name=CXorf57	CXorf57.value <= threshold=3.3742183446884155
node_6396: feature_name=MMD	MMD.value <= threshold=3.8883349895477295

node_6397: feature_name=IRF8	IRF8.value <= threshold=3.939465045928955
node_6398: feature_name=PPIF	PPIF.value > threshold=1.1284508109092712
node_6408: feature_name=JUN	JUN.value > threshold=-0.1225685365498066
Class: Healthy Control	
Rules_108	passed counts:47
node_0: feature_name=LTB	LTB.value > threshold=0.994031548500061
node_5264: feature_name=MT-CO1	MT-CO1.value > threshold=-0.4127991199493408
node_5638: feature_name=OAZ1	OAZ1.value <= threshold=-0.2640867084264755
node_5639: feature_name=CD79B	CD79B.value > threshold=2.2998517751693726
node_5925: feature_name=SNX9	SNX9.value <= threshold=2.4445992708206177
node_5926: feature_name=TCL1A	TCL1A.value <= threshold=2.6938670873641968
node_5927: feature_name=PLD4	PLD4.value <= threshold=-0.06434785947203636
node_5928: feature_name=ICAM1	ICAM1.value <= threshold=-0.2820792943239212
node_5929: feature_name=MS4A1	MS4A1.value > threshold=2.747320055961609
node_5963: feature_name=RNF144B	RNF144B.value <= threshold=-0.2324080914258957
node_5964: feature_name=HLA-DRB5	HLA-DRB5.value <= threshold=2.0430362224578857
node_5965: feature_name=MX1	MX1.value <= threshold=1.2721255421638489
node_5966: feature_name=LDLRAD4	LDLRAD4.value <= threshold=2.3228561878204346
node_5967: feature_name=LTB	LTB.value > threshold=1.1045992970466614
Class: Healthy Control	
Rules_109	passed counts:46
node_0: feature_name=LTB	LTB.value <= threshold=0.994031548500061
node_1: feature_name=MTRNR2L2	MTRNR2L2.value <= threshold=2.8784297704696655
node_2: feature_name=TCL1A	TCL1A.value <= threshold=4.798312664031982
node_3: feature_name=MT-CO1	MT-CO1.value > threshold=0.08947191759943962
node_2521: feature_name=LTB	LTB.value > threshold=0.6038667261600494
node_4173: feature_name=OAZ1	OAZ1.value > threshold=0.143283873796463
node_4615: feature_name=NR4A1	NR4A1.value <= threshold=0.6621004343032837

node_4616: feature_name=NFKBID	NFKBID.value <= threshold=3.283741593360901
node_4617: feature_name=HLA-DQA1	HLA-DQA1.value <= threshold=2.3435752391815186
node_4618: feature_name=PELI1	PELI1.value <= threshold=1.3271203637123108
node_4619: feature_name=PLD4	PLD4.value <= threshold=3.3385335206985474
node_4620: feature_name=MEF2A	MEF2A.value <= threshold=1.5926940441131592
node_4621: feature_name=DAPP1	DAPP1.value <= threshold=2.647637367248535
node_4622: feature_name=STAG3	STAG3.value <= threshold=-0.16521219909191132
node_4623: feature_name=OAZ1	OAZ1.value > threshold=0.2042311355471611
node_4625: feature_name=FAM129C	FAM129C.value > threshold=-0.23615043610334396
node_4639: feature_name=TP53INP1	TP53INP1.value <= threshold=4.941076993942261
node_4640: feature_name=KIAA0125	KIAA0125.value <= threshold=7.376631736755371
node_4641: feature_name=SESN3	SESN3.value <= threshold=1.000320941209793
Class: Healthy Control	
Rules_110	passed counts:46
node_0: feature_name=LTB	LTB.value <= threshold=0.994031548500061
node_1: feature_name=MTRNR2L2	MTRNR2L2.value <= threshold=2.8784297704696655
node_2: feature_name=TCL1A	TCL1A.value <= threshold=4.798312664031982
node_3: feature_name=MT-CO1	MT-CO1.value <= threshold=0.08947191759943962
node_4: feature_name=NR4A1	NR4A1.value <= threshold=1.0725348591804504
node_5: feature_name=HLA-DPA1	HLA-DPA1.value <= threshold=1.6885469555854797
node_6: feature_name=HLA-DRB5	HLA-DRB5.value <= threshold=2.1886194944381714
node_7: feature_name=XIST	XIST.value <= threshold=-0.12328458577394485
node_8: feature_name=SYNE1	SYNE1.value > threshold=-0.7835594415664673
node_16: feature_name=MTRNR2L2	MTRNR2L2.value <= threshold=2.4483247995376587
node_17: feature_name=HLA-DQA2	HLA-DQA2.value <= threshold=2.227178931236267
node_18: feature_name=NR4A1	NR4A1.value <= threshold=-0.21380723267793655
node_19: feature_name=IRAK3	IRAK3.value <= threshold=4.27159571647644
node_20: feature_name=IFIT2	IFIT2.value <= threshold=7.8175740242004395
node_21: feature_name=CD79A	CD79A.value <= threshold=2.857242226600647

node_22: feature_name=HIST1H2AC	HIST1H2AC.value <= threshold=5.077470302581787
node_23: feature_name=FTH1	FTH1.value <= threshold=0.4158056080341339
node_24: feature_name=ATF3	ATF3.value <= threshold=3.8028353452682495
node_25: feature_name=OASL	OASL.value <= threshold=3.387580633163452
node_26: feature_name=MT-CO1	MT-CO1.value <= threshold=-0.7942646145820618
node_27: feature_name=FCER2	FCER2.value <= threshold=7.268347978591919
node_28: feature_name=CCL3	CCL3.value <= threshold=3.2822797298431396
node_29: feature_name=BCL2A1	BCL2A1.value <= threshold=3.1504160165786743
node_30: feature_name=GRAP2	GRAP2.value <= threshold=3.0517499446868896
node_31: feature_name=EGR1	EGR1.value <= threshold=7.645251512527466
node_32: feature_name=GPX1	GPX1.value > threshold=0.6752602756023407
node_58: feature_name=GPX1	GPX1.value > threshold=0.6858049035072327
node_60: feature_name=HLA-DQB1	HLA-DQB1.value > threshold=-0.5587622821331024
node_62: feature_name=IGJ	IGJ.value <= threshold=3.6438305377960205
Class: COVID-19 Critical	
Rules_111	passed counts:45
node_0: feature_name=LTB	LTB.value <= threshold=0.994031548500061
node_1: feature_name=MTRNR2L2	MTRNR2L2.value <= threshold=2.8784297704696655
node_2: feature_name=TCL1A	TCL1A.value > threshold=4.798312664031982
node_4912: feature_name=MT-CO1	MT-CO1.value <= threshold=-0.3664844036102295
node_4913: feature_name=CD79A	CD79A.value > threshold=2.987208843231201
node_4973: feature_name=IFI44L	IFI44L.value <= threshold=0.7183398455381393
node_4974: feature_name=CD79B	CD79B.value > threshold=1.7606428265571594
node_4982: feature_name=TNFAIP3	TNFAIP3.value <= threshold=0.0729537159204483
node_4983: feature_name=HLA-DRB5	HLA-DRB5.value <= threshold=2.942599296569824
node_4984: feature_name=MT-CO3	MT-CO3.value > threshold=-1.767779290676117
node_4988: feature_name=IRF4	IRF4.value <= threshold=4.741645812988281
node_4989: feature_name=MT-CO3	MT-CO3.value <= threshold=0.6114157736301422
Class: Healthy Control	

Rules_112	passed counts:45
node_0: feature_name=LTB	LTB.value <= threshold=0.994031548500061
node_1: feature_name=MTRNR2L2	MTRNR2L2.value <= threshold=2.8784297704696655
node_2: feature_name=TCL1A	TCL1A.value <= threshold=4.798312664031982
node_3: feature_name=MT-CO1	MT-CO1.value > threshold=0.08947191759943962
node_2521: feature_name=LTB	LTB.value > threshold=0.6038667261600494
node_4173: feature_name=OAZ1	OAZ1.value <= threshold=0.143283873796463
node_4174: feature_name=PLD4	PLD4.value <= threshold=2.921932101249695
node_4175: feature_name=XIST	XIST.value <= threshold=-0.05067425221204758
node_4176: feature_name=MT-CO1	MT-CO1.value > threshold=1.345594048500061
node_4486: feature_name=RALGPS2	RALGPS2.value > threshold=2.7596691846847534
node_4508: feature_name=SNX9	SNX9.value <= threshold=2.5712826251983643
node_4509: feature_name=NFKBIZ	NFKBIZ.value > threshold=-0.8055458068847656
node_4511: feature_name=MT-CO3	MT-CO3.value > threshold=0.9913833141326904
node_4515: feature_name=MT-CO2	MT-CO2.value <= threshold=2.31252920627594
node_4516: feature_name=SLC7A5	SLC7A5.value <= threshold=3.756393551826477
node_4517: feature_name=PPIF	PPIF.value <= threshold=3.0951626300811768
node_4518: feature_name=PARP14	PARP14.value <= threshold=4.886617422103882
Class: Healthy Control	
Rules_113	passed counts:44
node_0: feature_name=LTB	LTB.value <= threshold=0.994031548500061
node_1: feature_name=MTRNR2L2	MTRNR2L2.value <= threshold=2.8784297704696655
node_2: feature_name=TCL1A	TCL1A.value <= threshold=4.798312664031982
node_3: feature_name=MT-CO1	MT-CO1.value <= threshold=0.08947191759943962
node_4: feature_name=NR4A1	NR4A1.value > threshold=1.0725348591804504
node_1622: feature_name=HLA-DQA2	HLA-DQA2.value <= threshold=1.9496816992759705
node_1623: feature_name=HLA-DRB5	HLA-DRB5.value <= threshold=2.715766429901123
node_1624: feature_name=HLA-DPA1	HLA-DPA1.value <= threshold=1.7050301432609558

node_1625: feature_name=CD83	CD83.value > threshold=2.783489227294922
node_1873: feature_name=IFI44L	IFI44L.value <= threshold=1.5938722491264343
node_1874: feature_name=PLD4	PLD4.value <= threshold=4.101001739501953
node_1875: feature_name=RHOB	RHOB.value > threshold=1.304835319519043
node_1965: feature_name=HLA-DQB1	HLA-DQB1.value > threshold=1.5989137291908264
node_1991: feature_name=COTL1	COTL1.value > threshold=-1.2334879636764526
node_1997: feature_name=HSP90B1	HSP90B1.value <= threshold=1.903715431690216
node_1998: feature_name=NFKBID	NFKBID.value > threshold=1.8163637518882751
node_2012: feature_name=UTY	UTY.value > threshold=-0.5331911742687225
node_2014: feature_name=ILK	ILK.value <= threshold=2.458114504814148
node_2015: feature_name=MYL9	MYL9.value <= threshold=2.1646361108869314
node_2016: feature_name=MT-ND6	MT-ND6.value <= threshold=1.4324160814285278
Class: COVID-19 Critical	
Rules_114	passed counts:43
node_0: feature_name=LTB	LTB.value > threshold=0.994031548500061
node_5264: feature_name=MT-CO1	MT-CO1.value <= threshold=-0.4127991199493408
node_5265: feature_name=MTRNR2L2	MTRNR2L2.value <= threshold=2.4473838806152344
node_5266: feature_name=TCL1A	TCL1A.value <= threshold=5.787256956100464
node_5267: feature_name=PLD4	PLD4.value <= threshold=3.544657826423645
node_5268: feature_name=NR4A1	NR4A1.value <= threshold=0.8490704298019409
node_5269: feature_name=LTB	LTB.value <= threshold=1.5252206921577454
node_5270: feature_name=HLA-DRB5	HLA-DRB5.value <= threshold=2.5117164850234985
node_5271: feature_name=CD79A	CD79A.value <= threshold=3.685201406478882
node_5272: feature_name=BCL2A1	BCL2A1.value <= threshold=2.081526756286621
node_5273: feature_name=HLA-DPA1	HLA-DPA1.value <= threshold=1.8042166829109192
node_5274: feature_name=HSP90B1	HSP90B1.value > threshold=-1.3115270137786865
node_5278: feature_name=OAZ1	OAZ1.value <= threshold=1.0802008509635925
node_5279: feature_name=CD79B	CD79B.value <= threshold=5.875581979751587
node_5280: feature_name=CCDC141	CCDC141.value <= threshold=6.439151048660278

node_5281: feature_name=SYTL3	SYTL3.value <= threshold=1.149337351322174
node_5282: feature_name=GPX1	GPX1.value > threshold=-1.0243326425552368
node_5284: feature_name=MMD	MMD.value <= threshold=6.431635856628418
node_5285: feature_name=MARCKS	MARCKS.value <= threshold=4.034798860549927
node_5286: feature_name=XIST	XIST.value <= threshold=1.148451752960682
node_5287: feature_name=CD83	CD83.value <= threshold=2.894522190093994
node_5288: feature_name=MIR24-2	MIR24-2.value <= threshold=2.76771080493927
node_5289: feature_name=GRASP	GRASP.value <= threshold=-0.4039058983325958
node_5290: feature_name=SLC7A5	SLC7A5.value > threshold=-0.4947117418050766
node_5294: feature_name=CD83	CD83.value <= threshold=2.1984872817993164
node_5295: feature_name=ID3	ID3.value <= threshold=3.3465518951416016
Class: COVID-19 Critical	
Rules_115	passed counts:43
node_0: feature_name=LTB	LTB.value <= threshold=0.994031548500061
node_1: feature_name=MTRNR2L2	MTRNR2L2.value <= threshold=2.8784297704696655
node_2: feature_name=TCL1A	TCL1A.value <= threshold=4.798312664031982
node_3: feature_name=MT-CO1	MT-CO1.value <= threshold=0.08947191759943962
node_4: feature_name=NR4A1	NR4A1.value <= threshold=1.0725348591804504
node_5: feature_name=HLA-DPA1	HLA-DPA1.value <= threshold=1.6885469555854797
node_6: feature_name=HLA-DRB5	HLA-DRB5.value <= threshold=2.1886194944381714
node_7: feature_name=XIST	XIST.value > threshold=-0.12328458577394485
node_711: feature_name=HLA-DQB1	HLA-DQB1.value <= threshold=2.337415099143982
node_712: feature_name=TNF	TNF.value <= threshold=0.626040518283844
node_713: feature_name=NR4A3	NR4A3.value <= threshold=1.637395977973938
node_714: feature_name=TCL1A	TCL1A.value <= threshold=4.2311248779296875
node_715: feature_name=IGJ	IGJ.value <= threshold=2.6652711629867554
Class: COVID-19 Critical	
Rules_116	passed counts:42

node_0: feature_name=LTB	LTB.value > threshold=0.994031548500061
node_5264: feature_name=MT-CO1	MT-CO1.value > threshold=-0.4127991199493408
node_5638: feature_name=OAZ1	OAZ1.value > threshold=-0.2640867084264755
node_6112: feature_name=IFI44L	IFI44L.value <= threshold=2.5893263816833496
node_6113: feature_name=TCL1A	TCL1A.value > threshold=4.201832294464111
node_6991: feature_name=XAF1	XAF1.value <= threshold=3.4585667848587036
node_6992: feature_name=STAG3	STAG3.value <= threshold=7.686291694641113
node_6993: feature_name=RALGPS2	RALGPS2.value > threshold=-0.4090157449245453
node_6997: feature_name=MTRNR2L2	MTRNR2L2.value <= threshold=3.23723828792572
node_6998: feature_name=TP53INP1	TP53INP1.value <= threshold=6.239925146102905
node_6999: feature_name=MEF2C	MEF2C.value > threshold=-0.44642284512519836
node_7003: feature_name=HSPA1A	HSPA1A.value <= threshold=7.404359817504883
node_7004: feature_name=COTL1	COTL1.value > threshold=-1.6598749160766602
node_7006: feature_name=HIST1H2AC	HIST1H2AC.value > threshold=-0.34910088777542114
node_7008: feature_name=MARCH1	MARCH1.value <= threshold=5.393903970718384
node_7009: feature_name=PELI1	PELI1.value <= threshold=3.565749168395996
node_7010: feature_name=RGS1	RGS1.value <= threshold=2.851950168609619
node_7011: feature_name=HLA-DRB5	HLA-DRB5.value <= threshold=3.0528379678726196
node_7012: feature_name=MEF2A	MEF2A.value <= threshold=3.228689193725586
node_7013: feature_name=MT-CO1	MT-CO1.value > threshold=-0.37944722175598145
node_7017: feature_name=ATG2A	ATG2A.value <= threshold=2.790610194206238
node_7018: feature_name=MEF2C	MEF2C.value <= threshold=-0.404978945851326
node_7019: feature_name=NAIP	NAIP.value <= threshold=-0.33305495977401733
node_7020: feature_name=MIR24-2	MIR24-2.value <= threshold=1.1773051023483276
Class: Healthy Control	
Rules_117	passed counts:42
node_0: feature_name=LTB	LTB.value <= threshold=0.994031548500061
node_1: feature_name=MTRNR2L2	MTRNR2L2.value <= threshold=2.8784297704696655
node_2: feature_name=TCL1A	TCL1A.value > threshold=4.798312664031982

node_4912: feature_name=MT-CO1	MT-CO1.value > threshold=-0.3664844036102295
node_5002: feature_name=IFI44L	IFI44L.value <= threshold=2.3101747035980225
node_5003: feature_name=SNX9	SNX9.value > threshold=2.0247421264648438
node_5147: feature_name=CD79A	CD79A.value > threshold=2.554050326347351
node_5169: feature_name=CD79B	CD79B.value > threshold=-0.2480710819363594
node_5185: feature_name=OAZ1	OAZ1.value > threshold=0.2523931488394737
node_5193: feature_name=MEF2A	MEF2A.value <= threshold=2.9668554067611694
node_5194: feature_name=MARCH1	MARCH1.value <= threshold=3.9670889377593994
Class: Healthy Control	
Rules_118	passed counts:42
node_0: feature_name=LTB	LTB.value <= threshold=0.994031548500061
node_1: feature_name=MTRNR2L2	MTRNR2L2.value <= threshold=2.8784297704696655
node_2: feature_name=TCL1A	TCL1A.value <= threshold=4.798312664031982
node_3: feature_name=MT-CO1	MT-CO1.value > threshold=0.08947191759943962
node_2521: feature_name=LTB	LTB.value <= threshold=0.6038667261600494
node_2522: feature_name=HLA-DQB1	HLA-DQB1.value <= threshold=2.1053054332733154
node_2523: feature_name=SNX9	SNX9.value <= threshold=-0.5346486568450928
node_2524: feature_name=HLA-DPB1	HLA-DPB1.value > threshold=1.9548270106315613
node_2814: feature_name=CD79B	CD79B.value > threshold=-0.28186772763729095
node_2826: feature_name=MT-ND6	MT-ND6.value <= threshold=0.17273088544607162
node_2827: feature_name=NR4A1	NR4A1.value <= threshold=-0.24634335190057755
node_2828: feature_name=NR4A3	NR4A3.value <= threshold=1.609707236289978
node_2829: feature_name=CD79B	CD79B.value <= threshold=4.004396319389343
node_2830: feature_name=ICAM1	ICAM1.value <= threshold=0.6128160282969475
node_2831: feature_name=HOPX	HOPX.value <= threshold=2.623421788215637
node_2832: feature_name=HLA-DRB1	HLA-DRB1.value <= threshold=2.767460584640503
node_2833: feature_name=XIST	XIST.value <= threshold=1.2089489996433258
Class: COVID-19 Critical	

Rules_119	passed counts:40
node_0: feature_name=LTB	LTB.value > threshold=0.994031548500061
node_5264: feature_name=MT-CO1	MT-CO1.value > threshold=-0.4127991199493408
node_5638: feature_name=OAZ1	OAZ1.value > threshold=-0.2640867084264755
node_6112: feature_name=IFI44L	IFI44L.value <= threshold=2.5893263816833496
node_6113: feature_name=TCL1A	TCL1A.value <= threshold=4.201832294464111
node_6114: feature_name=HLA-DQA2	HLA-DQA2.value <= threshold=-0.2270243540406227
node_6115: feature_name=MTRNR2L2	MTRNR2L2.value > threshold=2.548917293548584
node_6535: feature_name=RGS1	RGS1.value > threshold=-0.6410573720932007
node_6539: feature_name=SPI1	SPI1.value <= threshold=0.7604411840438843
Class: COVID-19 Severe	
Rules_120	passed counts:40
node_0: feature_name=LTB	LTB.value > threshold=0.994031548500061
node_5264: feature_name=MT-CO1	MT-CO1.value > threshold=-0.4127991199493408
node_5638: feature_name=OAZ1	OAZ1.value <= threshold=-0.2640867084264755
node_5639: feature_name=CD79B	CD79B.value > threshold=2.2998517751693726
node_5925: feature_name=SNX9	SNX9.value > threshold=2.4445992708206177
node_6083: feature_name=RALGPS2	RALGPS2.value <= threshold=4.061077356338501
node_6084: feature_name=HLA-DQB1	HLA-DQB1.value <= threshold=2.593139410018921
node_6085: feature_name=RP11-731F5.2	RP11-731F5.2.value <= threshold=5.4822447299957275
node_6086: feature_name=ZNF10	ZNF10.value <= threshold=5.895071268081665
node_6087: feature_name=IFIT2	IFIT2.value <= threshold=1.681579053401947
node_6088: feature_name=TNF	TNF.value <= threshold=2.0882365703582764
Class: COVID-19 Critical	
Rules_121	passed counts:40
node_0: feature_name=LTB	LTB.value > threshold=0.994031548500061
node_5264: feature_name=MT-CO1	MT-CO1.value <= threshold=-0.4127991199493408
node_5265: feature_name=MTRNR2L2	MTRNR2L2.value <= threshold=2.4473838806152344

node_5266: feature_name=TCL1A	TCL1A.value <= threshold=5.787256956100464
node_5267: feature_name=PLD4	PLD4.value > threshold=3.544657826423645
node_5577: feature_name=MT-CO1	MT-CO1.value > threshold=-0.6592064797878265
node_5601: feature_name=OAZ1	OAZ1.value > threshold=-0.21202879399061203
node_5605: feature_name=JUN	JUN.value <= threshold=1.5750274658203125
node_5606: feature_name=COTL1	COTL1.value <= threshold=1.0928653478622437
node_5607: feature_name=MMD	MMD.value <= threshold=2.549311637878418
node_5608: feature_name=FTH1	FTH1.value > threshold=-1.1575720310211182
Class: Healthy Control	
Rules_122	passed counts:40
node_0: feature_name=LTB	LTB.value <= threshold=0.994031548500061
node_1: feature_name=MTRNR2L2	MTRNR2L2.value <= threshold=2.8784297704696655
node_2: feature_name=TCL1A	TCL1A.value <= threshold=4.798312664031982
node_3: feature_name=MT-CO1	MT-CO1.value > threshold=0.08947191759943962
node_2521: feature_name=LTB	LTB.value > threshold=0.6038667261600494
node_4173: feature_name=OAZ1	OAZ1.value <= threshold=0.143283873796463
node_4174: feature_name=PLD4	PLD4.value <= threshold=2.921932101249695
node_4175: feature_name=XIST	XIST.value <= threshold=-0.05067425221204758
node_4176: feature_name=MT-CO1	MT-CO1.value > threshold=1.345594048500061
node_4486: feature_name=RALGPS2	RALGPS2.value <= threshold=2.7596691846847534
node_4487: feature_name=IFIT2	IFIT2.value <= threshold=-0.1915586143732071
node_4488: feature_name=MT-CO1	MT-CO1.value > threshold=1.3644989132881165
node_4490: feature_name=IFI6	IFI6.value <= threshold=1.1558569073677063
node_4491: feature_name=TCL1A	TCL1A.value <= threshold=2.8633137941360474
node_4492: feature_name=TTN	TTN.value <= threshold=4.178668260574341
Class: COVID-19 Critical	
Rules_123	passed counts:39
node_0: feature_name=LTB	LTB.value > threshold=0.994031548500061

node_5264: feature_name=MT-CO1	MT-CO1.value > threshold=-0.4127991199493408
node_5638: feature_name=OAZ1	OAZ1.value > threshold=-0.2640867084264755
node_6112: feature_name=IFI44L	IFI44L.value <= threshold=2.5893263816833496
node_6113: feature_name=TCL1A	TCL1A.value <= threshold=4.201832294464111
node_6114: feature_name=HLA-DQA2	HLA-DQA2.value <= threshold=-0.2270243540406227
node_6115: feature_name=MTRNR2L2	MTRNR2L2.value <= threshold=2.548917293548584
node_6116: feature_name=ATF5	ATF5.value <= threshold=2.041263461112976
node_6117: feature_name=JUN	JUN.value > threshold=-0.616259902715683
node_6189: feature_name=MT-CO3	MT-CO3.value <= threshold=0.13483521342277527
node_6190: feature_name=XAF1	XAF1.value <= threshold=2.584952235221863
node_6191: feature_name=HLA-DRB5	HLA-DRB5.value <= threshold=0.6955901980400085
node_6192: feature_name=NR4A1	NR4A1.value <= threshold=-0.4496448189020157
node_6193: feature_name=HLA-DPB1	HLA-DPB1.value <= threshold=1.7609722018241882
node_6194: feature_name=JUN	JUN.value <= threshold=1.172904908657074
node_6195: feature_name=MT-ND6	MT-ND6.value <= threshold=0.6287261247634888
node_6196: feature_name=ACSL1	ACSL1.value <= threshold=-0.31894539296627045
node_6197: feature_name=STMN1	STMN1.value <= threshold=1.9789239764213562
node_6198: feature_name=AP3M2	AP3M2.value <= threshold=1.1014634370803833
Class: COVID-19 Critical	
Rules_124	passed counts:38
node_0: feature_name=LTB	LTB.value > threshold=0.994031548500061
node_5264: feature_name=MT-CO1	MT-CO1.value > threshold=-0.4127991199493408
node_5638: feature_name=OAZ1	OAZ1.value > threshold=-0.2640867084264755
node_6112: feature_name=IFI44L	IFI44L.value <= threshold=2.5893263816833496
node_6113: feature_name=TCL1A	TCL1A.value <= threshold=4.201832294464111
node_6114: feature_name=HLA-DQA2	HLA-DQA2.value > threshold=-0.2270243540406227
node_6542: feature_name=CD79B	CD79B.value <= threshold=3.096232771873474
node_6543: feature_name=NR4A1	NR4A1.value <= threshold=1.421015739440918
node_6544: feature_name=LTB	LTB.value <= threshold=1.359755277633667

node_6545: feature_name=NFKBID	NFKBID.value <= threshold=1.4294711351394653
node_6546: feature_name=HLA-DRB1	HLA-DRB1.value > threshold=1.0398632287979126
node_6556: feature_name=CD79B	CD79B.value <= threshold=-0.2919560521841049
node_6557: feature_name=MTRNR2L2	MTRNR2L2.value <= threshold=2.7710195183753967
node_6558: feature_name=JUN	JUN.value <= threshold=0.5767147243022919
node_6559: feature_name=TLN1	TLN1.value <= threshold=-0.08378086984157562
node_6560: feature_name=LDLRAD4	LDLRAD4.value <= threshold=2.972443103790283
node_6561: feature_name=MT-CO1	MT-CO1.value > threshold=-0.3593759834766388
node_6563: feature_name=MAP3K2	MAP3K2.value <= threshold=1.9669477939605713
node_6564: feature_name=HSP90B1	HSP90B1.value > threshold=-1.168101727962494
Class: COVID-19 Critical	
Rules_125	passed counts:38
node_0: feature_name=LTB	LTB.value > threshold=0.994031548500061
node_5264: feature_name=MT-CO1	MT-CO1.value > threshold=-0.4127991199493408
node_5638: feature_name=OAZ1	OAZ1.value > threshold=-0.2640867084264755
node_6112: feature_name=IFI44L	IFI44L.value <= threshold=2.5893263816833496
node_6113: feature_name=TCL1A	TCL1A.value <= threshold=4.201832294464111
node_6114: feature_name=HLA-DQA2	HLA-DQA2.value > threshold=-0.2270243540406227
node_6542: feature_name=CD79B	CD79B.value <= threshold=3.096232771873474
node_6543: feature_name=NR4A1	NR4A1.value <= threshold=1.421015739440918
node_6544: feature_name=LTB	LTB.value <= threshold=1.359755277633667
node_6545: feature_name=NFKBID	NFKBID.value <= threshold=1.4294711351394653
node_6546: feature_name=HLA-DRB1	HLA-DRB1.value <= threshold=1.0398632287979126
node_6547: feature_name=CASP8	CASP8.value <= threshold=0.6416637301445007
node_6548: feature_name=OAZ1	OAZ1.value > threshold=-0.24654901772737503
node_6550: feature_name=HLA-DPB1	HLA-DPB1.value > threshold=-0.6567609012126923
Class: COVID-19 Critical	
Rules_126	passed counts:38

node_0: feature_name=LTB	LTB.value <= threshold=0.994031548500061
node_1: feature_name=MTRNR2L2	MTRNR2L2.value <= threshold=2.8784297704696655
node_2: feature_name=TCL1A	TCL1A.value <= threshold=4.798312664031982
node_3: feature_name=MT-CO1	MT-CO1.value > threshold=0.08947191759943962
node_2521: feature_name=LTB	LTB.value <= threshold=0.6038667261600494
node_2522: feature_name=HLA-DQB1	HLA-DQB1.value > threshold=2.1053054332733154
node_3550: feature_name=XIST	XIST.value <= threshold=0.7716337237507105
node_3551: feature_name=CD79B	CD79B.value <= threshold=2.9426796436309814
node_3552: feature_name=HLA-DQB1	HLA-DQB1.value <= threshold=2.713674306869507
node_3553: feature_name=SNX9	SNX9.value > threshold=-0.5404067635536194
node_3653: feature_name=LTB	LTB.value <= threshold=0.021708179265260696
node_3654: feature_name=HLA-DRB5	HLA-DRB5.value > threshold=-0.5633223652839661
node_3660: feature_name=NR4A1	NR4A1.value <= threshold=2.2327845096588135
node_3661: feature_name=FCRL1	FCRL1.value <= threshold=8.662389755249023
node_3662: feature_name=BANK1	BANK1.value <= threshold=4.776419401168823
node_3663: feature_name=IGLL5	IGLL5.value > threshold=1.4806077480316162
node_3705: feature_name=NR4A1	NR4A1.value <= threshold=1.2165279388427734
node_3706: feature_name=NAIP	NAIP.value <= threshold=-0.26606951653957367
node_3707: feature_name=ABCA1	ABCA1.value <= threshold=2.952040672302246
node_3708: feature_name=TSPYL2	TSPYL2.value <= threshold=1.8636173009872437
node_3709: feature_name=STMN1	STMN1.value <= threshold=2.621256947517395
node_3710: feature_name=DUSP4	DUSP4.value <= threshold=3.1131497621536255
Class: COVID-19 Critical	
Rules_127	passed counts:38
node_0: feature_name=LTB	LTB.value <= threshold=0.994031548500061
node_1: feature_name=MTRNR2L2	MTRNR2L2.value <= threshold=2.8784297704696655
node_2: feature_name=TCL1A	TCL1A.value <= threshold=4.798312664031982
node_3: feature_name=MT-CO1	MT-CO1.value <= threshold=0.08947191759943962
node_4: feature_name=NR4A1	NR4A1.value > threshold=1.0725348591804504

node_1622: feature_name=HLA-DQA2	HLA-DQA2.value <= threshold=1.9496816992759705
node_1623: feature_name=HLA-DRB5	HLA-DRB5.value <= threshold=2.715766429901123
node_1624: feature_name=HLA-DPA1	HLA-DPA1.value > threshold=1.7050301432609558
node_2056: feature_name=OAZ1	OAZ1.value <= threshold=0.456103190779686
node_2057: feature_name=HLA-DRB5	HLA-DRB5.value > threshold=0.6839984953403473
node_2101: feature_name=HLA-DRB1	HLA-DRB1.value <= threshold=1.6199676990509033
node_2102: feature_name=CD83	CD83.value <= threshold=2.5568599700927734
node_2103: feature_name=MT-CO1	MT-CO1.value <= threshold=-0.08588453382253647
node_2104: feature_name=FAM129C	FAM129C.value <= threshold=3.9770365953445435
node_2105: feature_name=OAZ1	OAZ1.value <= threshold=0.13203191384673119
node_2106: feature_name=SESN3	SESN3.value <= threshold=2.733139395713806
node_2107: feature_name=ID3	ID3.value <= threshold=3.629174590110779
node_2108: feature_name=PELI1	PELI1.value <= threshold=2.723706841468811
Class: COVID-19 Critical	
Rules_128	passed counts:37
node_0: feature_name=LTB	LTB.value > threshold=0.994031548500061
node_5264: feature_name=MT-CO1	MT-CO1.value > threshold=-0.4127991199493408
node_5638: feature_name=OAZ1	OAZ1.value > threshold=-0.2640867084264755
node_6112: feature_name=IFI44L	IFI44L.value <= threshold=2.5893263816833496
node_6113: feature_name=TCL1A	TCL1A.value <= threshold=4.201832294464111
node_6114: feature_name=HLA-DQA2	HLA-DQA2.value <= threshold=-0.2270243540406227
node_6115: feature_name=MTRNR2L2	MTRNR2L2.value <= threshold=2.548917293548584
node_6116: feature_name=ATF5	ATF5.value <= threshold=2.041263461112976
node_6117: feature_name=JUN	JUN.value > threshold=-0.616259902715683
node_6189: feature_name=MT-CO3	MT-CO3.value <= threshold=0.13483521342277527
node_6190: feature_name=XAF1	XAF1.value <= threshold=2.584952235221863
node_6191: feature_name=HLA-DRB5	HLA-DRB5.value <= threshold=0.6955901980400085
node_6192: feature_name=NR4A1	NR4A1.value <= threshold=-0.4496448189020157
node_6193: feature_name=HLA-DPB1	HLA-DPB1.value > threshold=1.7609722018241882

node_6213: feature_name=SNX9	SNX9.value <= threshold=1.1182581186294556
node_6214: feature_name=MX1	MX1.value <= threshold=1.2671910524368286
node_6215: feature_name=ICAM1	ICAM1.value <= threshold=0.8275804966688156
Class: Healthy Control	
Rules_129	passed counts:37
node_0: feature_name=LTB	LTB.value > threshold=0.994031548500061
node_5264: feature_name=MT-CO1	MT-CO1.value > threshold=-0.4127991199493408
node_5638: feature_name=OAZ1	OAZ1.value <= threshold=-0.2640867084264755
node_5639: feature_name=CD79B	CD79B.value > threshold=2.2998517751693726
node_5925: feature_name=SNX9	SNX9.value <= threshold=2.4445992708206177
node_5926: feature_name=TCL1A	TCL1A.value <= threshold=2.6938670873641968
node_5927: feature_name=PLD4	PLD4.value <= threshold=-0.06434785947203636
node_5928: feature_name=ICAM1	ICAM1.value <= threshold=-0.2820792943239212
node_5929: feature_name=MS4A1	MS4A1.value <= threshold=2.747320055961609
node_5930: feature_name=LTB	LTB.value <= threshold=1.3533939123153687
node_5931: feature_name=CD83	CD83.value <= threshold=3.0086376667022705
node_5932: feature_name=HLA-DQB1	HLA-DQB1.value <= threshold=2.98651123046875
node_5933: feature_name=MTRNR2L2	MTRNR2L2.value <= threshold=0.8930560350418091
node_5934: feature_name=S100A6	S100A6.value <= threshold=0.22000961005687714
node_5935: feature_name=SYNE1	SYNE1.value <= threshold=0.772991269826889
node_5936: feature_name=JUN	JUN.value <= threshold=1.2428678274154663
Class: COVID-19 Critical	
Rules_130	passed counts:37
node_0: feature_name=LTB	LTB.value <= threshold=0.994031548500061
node_1: feature_name=MTRNR2L2	MTRNR2L2.value <= threshold=2.8784297704696655
node_2: feature_name=TCL1A	TCL1A.value > threshold=4.798312664031982
node_4912: feature_name=MT-CO1	MT-CO1.value > threshold=-0.3664844036102295
node_5002: feature_name=IFI44L	IFI44L.value <= threshold=2.3101747035980225

node_5003: feature_name=SNX9	SNX9.value > threshold=2.0247421264648438
node_5147: feature_name=CD79A	CD79A.value <= threshold=2.554050326347351
node_5148: feature_name=HLA-DQB1	HLA-DQB1.value <= threshold=2.6719621419906616
node_5149: feature_name=BANK1	BANK1.value <= threshold=4.278962850570679
node_5150: feature_name=SNX29P2	SNX29P2.value <= threshold=4.986646890640259
node_5151: feature_name=MIAT	MIAT.value <= threshold=-0.14716000854969025
node_5152: feature_name=CD79B	CD79B.value <= threshold=4.372928857803345
Class: COVID-19 Critical	
Rules_131	passed counts:37
node_0: feature_name=LTB	LTB.value <= threshold=0.994031548500061
node_1: feature_name=MTRNR2L2	MTRNR2L2.value <= threshold=2.8784297704696655
node_2: feature_name=TCL1A	TCL1A.value <= threshold=4.798312664031982
node_3: feature_name=MT-CO1	MT-CO1.value > threshold=0.08947191759943962
node_2521: feature_name=LTB	LTB.value > threshold=0.6038667261600494
node_4173: feature_name=OAZ1	OAZ1.value <= threshold=0.143283873796463
node_4174: feature_name=PLD4	PLD4.value > threshold=2.921932101249695
node_4558: feature_name=HLA-DQA2	HLA-DQA2.value <= threshold=1.5485953092575073
node_4559: feature_name=COTL1	COTL1.value > threshold=-0.42844848334789276
node_4573: feature_name=CD83	CD83.value <= threshold=1.9224650263786316
node_4574: feature_name=ICAM1	ICAM1.value <= threshold=-0.3564278930425644
node_4575: feature_name=MARCKS	MARCKS.value <= threshold=2.6073683500289917
node_4576: feature_name=MARCH1	MARCH1.value > threshold=-0.4961168020963669
node_4578: feature_name=FAM129C	FAM129C.value > threshold=-0.2491237074136734
Class: Healthy Control	
Rules_132	passed counts:37
node_0: feature_name=LTB	LTB.value <= threshold=0.994031548500061
node_1: feature_name=MTRNR2L2	MTRNR2L2.value <= threshold=2.8784297704696655
node_2: feature_name=TCL1A	TCL1A.value <= threshold=4.798312664031982

node_3: feature_name=MT-CO1	MT-CO1.value > threshold=0.08947191759943962
node_2521: feature_name=LTB	LTB.value <= threshold=0.6038667261600494
node_2522: feature_name=HLA-DQB1	HLA-DQB1.value > threshold=2.1053054332733154
node_3550: feature_name=XIST	XIST.value <= threshold=0.7716337237507105
node_3551: feature_name=CD79B	CD79B.value <= threshold=2.9426796436309814
node_3552: feature_name=HLA-DQB1	HLA-DQB1.value > threshold=2.713674306869507
node_3854: feature_name=MEF2C	MEF2C.value > threshold=-0.3721291869878769
node_3882: feature_name=IFI44L	IFI44L.value <= threshold=2.5516022443771362
node_3883: feature_name=FCER2	FCER2.value > threshold=-0.21487552672624588
node_3977: feature_name=IGJ	IGJ.value <= threshold=2.123470664024353
node_3978: feature_name=S100A6	S100A6.value > threshold=-1.7107264399528503
node_3982: feature_name=MT-CO1	MT-CO1.value <= threshold=1.5725501775741577
node_3983: feature_name=SNX29P2	SNX29P2.value <= threshold=3.1593780517578125
node_3984: feature_name=IL8	IL8.value <= threshold=-0.28026726841926575
node_3985: feature_name=NAMPT	NAMPT.value > threshold=-0.5902779400348663
node_3991: feature_name=PELI1	PELI1.value <= threshold=3.5653313398361206
node_3992: feature_name=IRF8	IRF8.value > threshold=-0.34819670021533966
node_3998: feature_name=BACH1	BACH1.value <= threshold=3.9624054431915283
node_3999: feature_name=CD38	CD38.value <= threshold=3.3956143856048584
node_4000: feature_name=ID1	ID1.value <= threshold=2.621088981628418
Class: COVID-19 Critical	
Rules_133	passed counts:37
node_0: feature_name=LTB	LTB.value <= threshold=0.994031548500061
node_1: feature_name=MTRNR2L2	MTRNR2L2.value <= threshold=2.8784297704696655
node_2: feature_name=TCL1A	TCL1A.value <= threshold=4.798312664031982
node_3: feature_name=MT-CO1	MT-CO1.value > threshold=0.08947191759943962
node_2521: feature_name=LTB	LTB.value <= threshold=0.6038667261600494
node_2522: feature_name=HLA-DQB1	HLA-DQB1.value <= threshold=2.1053054332733154
node_2523: feature_name=SNX9	SNX9.value > threshold=-0.5346486568450928

node_2907: feature_name=NR4A1	NR4A1.value <= threshold=2.5433837175369263
node_2908: feature_name=XIST	XIST.value <= threshold=0.7664470989257097
node_2909: feature_name=CD79B	CD79B.value <= threshold=2.9326847791671753
node_2910: feature_name=NR4A1	NR4A1.value > threshold=-0.2875557690858841
node_3096: feature_name=NR4A3	NR4A3.value <= threshold=3.314968705177307
node_3097: feature_name=VPREB3	VPREB3.value <= threshold=3.3549938201904297
node_3098: feature_name=RALGPS2	RALGPS2.value <= threshold=5.807050704956055
node_3099: feature_name=CTD-2035E11.3	CTD-2035E11.3.value <= threshold=6.4090416431427
node_3100: feature_name=FAM129C	FAM129C.value <= threshold=6.966361045837402
node_3101: feature_name=HES4	HES4.value <= threshold=4.139557242393494
node_3102: feature_name=CD83	CD83.value <= threshold=1.8343153595924377
node_3103: feature_name=FCER2	FCER2.value <= threshold=7.255846738815308
node_3104: feature_name=MT-CYB	MT-CYB.value <= threshold=2.3888577222824097
node_3105: feature_name=RGCC	RGCC.value > threshold=-1.2040565013885498
node_3107: feature_name=MCTP1	MCTP1.value <= threshold=6.221277952194214
node_3108: feature_name=JUN	JUN.value <= threshold=-0.629857987165451
node_3109: feature_name=JUN	JUN.value <= threshold=-0.6661905348300934
node_3110: feature_name=MT-CO3	MT-CO3.value > threshold=-0.2565731704235077
node_3112: feature_name=MT-ND6	MT-ND6.value > threshold=-1.055504322052002
node_3114: feature_name=ID1	ID1.value <= threshold=3.4776406288146973
node_3115: feature_name=IRF4	IRF4.value <= threshold=5.238286733627319
Class: COVID-19 Critical	
Rules_134	passed counts:37
node_0: feature_name=LTB	LTB.value <= threshold=0.994031548500061
node_1: feature_name=MTRNR2L2	MTRNR2L2.value <= threshold=2.8784297704696655
node_2: feature_name=TCL1A	TCL1A.value <= threshold=4.798312664031982
node_3: feature_name=MT-CO1	MT-CO1.value > threshold=0.08947191759943962
node_2521: feature_name=LTB	LTB.value <= threshold=0.6038667261600494
node_2522: feature_name=HLA-DQB1	HLA-DQB1.value <= threshold=2.1053054332733154

node_2523: feature_name=SNX9	SNX9.value <= threshold=-0.5346486568450928
node_2524: feature_name=HLA-DPB1	HLA-DPB1.value > threshold=1.9548270106315613
node_2814: feature_name=CD79B	CD79B.value <= threshold=-0.28186772763729095
node_2815: feature_name=HLA-DRB5	HLA-DRB5.value > threshold=0.7097251415252686
node_2821: feature_name=FAM129C	FAM129C.value <= threshold=4.518173694610596
Class: COVID-19 Critical	
Rules_135	passed counts:36
node_0: feature_name=LTB	LTB.value <= threshold=0.994031548500061
node_1: feature_name=MTRNR2L2	MTRNR2L2.value <= threshold=2.8784297704696655
node_2: feature_name=TCL1A	TCL1A.value <= threshold=4.798312664031982
node_3: feature_name=MT-CO1	MT-CO1.value > threshold=0.08947191759943962
node_2521: feature_name=LTB	LTB.value <= threshold=0.6038667261600494
node_2522: feature_name=HLA-DQB1	HLA-DQB1.value <= threshold=2.1053054332733154
node_2523: feature_name=SNX9	SNX9.value <= threshold=-0.5346486568450928
node_2524: feature_name=HLA-DPB1	HLA-DPB1.value <= threshold=1.9548270106315613
node_2525: feature_name=FAM129C	FAM129C.value > threshold=3.3393388986587524
node_2721: feature_name=ADAM19	ADAM19.value > threshold=-0.29525794088840485
node_2781: feature_name=XAF1	XAF1.value > threshold=-0.4296249598264694
node_2793: feature_name=PELI1	PELI1.value <= threshold=1.8806812763214111
node_2794: feature_name=MT-CO2	MT-CO2.value > threshold=-0.5086818039417267
node_2796: feature_name=ISG15	ISG15.value <= threshold=1.3241910338401794
node_2797: feature_name=RP5-821D11.7	RP5-821D11.7.value <= threshold=5.538547039031982
Class: Healthy Control	
Rules_136	passed counts:36
node_0: feature_name=LTB	LTB.value <= threshold=0.994031548500061
node_1: feature_name=MTRNR2L2	MTRNR2L2.value <= threshold=2.8784297704696655
node_2: feature_name=TCL1A	TCL1A.value <= threshold=4.798312664031982
node_3: feature_name=MT-CO1	MT-CO1.value <= threshold=0.08947191759943962

node_4: feature_name=NR4A1	NR4A1.value > threshold=1.0725348591804504
node_1622: feature_name=HLA-DQA2	HLA-DQA2.value > threshold=1.9496816992759705
node_2350: feature_name=BCL2A1	BCL2A1.value <= threshold=1.1322240233421326
node_2351: feature_name=CD83	CD83.value > threshold=2.3162450790405273
node_2391: feature_name=CD79B	CD79B.value > threshold=1.7111427783966064
node_2443: feature_name=MT-CO1	MT-CO1.value > threshold=-1.1614157557487488
node_2447: feature_name=JUN	JUN.value > threshold=0.19632036238908768
node_2457: feature_name=MARCH1	MARCH1.value > threshold=-0.3794829994440079
node_2461: feature_name=MTRNR2L8	MTRNR2L8.value <= threshold=1.2178178429603577
node_2462: feature_name=FCRL1	FCRL1.value <= threshold=6.0251624584198
Class: COVID-19 Critical	
Rules_137	passed counts:35
node_0: feature_name=LTB	LTB.value <= threshold=0.994031548500061
node_1: feature_name=MTRNR2L2	MTRNR2L2.value <= threshold=2.8784297704696655
node_2: feature_name=TCL1A	TCL1A.value <= threshold=4.798312664031982
node_3: feature_name=MT-CO1	MT-CO1.value > threshold=0.08947191759943962
node_2521: feature_name=LTB	LTB.value > threshold=0.6038667261600494
node_4173: feature_name=OAZ1	OAZ1.value <= threshold=0.143283873796463
node_4174: feature_name=PLD4	PLD4.value > threshold=2.921932101249695
node_4558: feature_name=HLA-DQA2	HLA-DQA2.value > threshold=1.5485953092575073
node_4598: feature_name=MTRNR2L8	MTRNR2L8.value <= threshold=2.9248507022857666
node_4599: feature_name=HLA-DRB1	HLA-DRB1.value <= threshold=1.4262019395828247
node_4600: feature_name=CD83	CD83.value <= threshold=2.5374964475631714
node_4601: feature_name=PLD4	PLD4.value > threshold=2.9592150449752808
Class: COVID-19 Critical	
Rules_138	passed counts:35
node_0: feature_name=LTB	LTB.value <= threshold=0.994031548500061
node_1: feature_name=MTRNR2L2	MTRNR2L2.value <= threshold=2.8784297704696655

node_2: feature_name=TCL1A	TCL1A.value <= threshold=4.798312664031982
node_3: feature_name=MT-CO1	MT-CO1.value <= threshold=0.08947191759943962
node_4: feature_name=NR4A1	NR4A1.value <= threshold=1.0725348591804504
node_5: feature_name=HLA-DPA1	HLA-DPA1.value <= threshold=1.6885469555854797
node_6: feature_name=HLA-DRB5	HLA-DRB5.value > threshold=2.1886194944381714
node_738: feature_name=HLA-DQA2	HLA-DQA2.value > threshold=-0.20105575770139694
node_804: feature_name=CYBB	CYBB.value <= threshold=2.9090086221694946
node_805: feature_name=VPREB3	VPREB3.value <= threshold=7.757248401641846
node_806: feature_name=IL8	IL8.value <= threshold=1.7155421376228333
node_807: feature_name=ATF5	ATF5.value <= threshold=6.124105930328369
node_808: feature_name=RP11-731F5.2	RP11-731F5.2.value <= threshold=7.633600234985352
node_809: feature_name=OASL	OASL.value <= threshold=0.9932635426521301
node_810: feature_name=ID1	ID1.value <= threshold=2.8093127012252808
node_811: feature_name=GRASP	GRASP.value > threshold=2.3934966325759888
node_837: feature_name=HLA-DQA2	HLA-DQA2.value > threshold=-0.19460754096508026
node_839: feature_name=RGS2	RGS2.value <= threshold=1.362936019897461
node_840: feature_name=MMD	MMD.value <= threshold=5.1446380615234375
node_841: feature_name=HLA-DRB5	HLA-DRB5.value > threshold=2.198097825050354
Class: COVID-19 Critical	
Rules_139	passed counts:34
node_0: feature_name=LTB	LTB.value > threshold=0.994031548500061
node_5264: feature_name=MT-CO1	MT-CO1.value > threshold=-0.4127991199493408
node_5638: feature_name=OAZ1	OAZ1.value <= threshold=-0.2640867084264755
node_5639: feature_name=CD79B	CD79B.value <= threshold=2.2998517751693726
node_5640: feature_name=MTRNR2L2	MTRNR2L2.value > threshold=3.5499863624572754
node_5922: feature_name=PLEC	PLEC.value <= threshold=3.029274344444275
Class: COVID-19 Severe	
Rules_140	passed counts:34

node_0: feature_name=LTB	LTB.value <= threshold=0.994031548500061
node_1: feature_name=MTRNR2L2	MTRNR2L2.value > threshold=2.8784297704696655
node_5207: feature_name=MTRNR2L8	MTRNR2L8.value <= threshold=3.1915172338485718
node_5208: feature_name=MT-CYB	MT-CYB.value > threshold=1.3929389119148254
node_5242: feature_name=MTRNR2L2	MTRNR2L2.value > threshold=3.8612314462661743
node_5252: feature_name=IRF4	IRF4.value <= threshold=4.855696439743042
Class: COVID-19 Severe	
Rules_141	passed counts:34
node_0: feature_name=LTB	LTB.value <= threshold=0.994031548500061
node_1: feature_name=MTRNR2L2	MTRNR2L2.value <= threshold=2.8784297704696655
node_2: feature_name=TCL1A	TCL1A.value <= threshold=4.798312664031982
node_3: feature_name=MT-CO1	MT-CO1.value > threshold=0.08947191759943962
node_2521: feature_name=LTB	LTB.value <= threshold=0.6038667261600494
node_2522: feature_name=HLA-DQB1	HLA-DQB1.value > threshold=2.1053054332733154
node_3550: feature_name=XIST	XIST.value <= threshold=0.7716337237507105
node_3551: feature_name=CD79B	CD79B.value <= threshold=2.9426796436309814
node_3552: feature_name=HLA-DQB1	HLA-DQB1.value > threshold=2.713674306869507
node_3854: feature_name=MEF2C	MEF2C.value <= threshold=-0.3721291869878769
node_3855: feature_name=HLA-DRB5	HLA-DRB5.value <= threshold=0.7929701805114746
node_3856: feature_name=CTD-2035E11.3	CTD-2035E11.3.value <= threshold=3.802809715270996
Class: COVID-19 Severe	
Rules_142	passed counts:34
node_0: feature_name=LTB	LTB.value <= threshold=0.994031548500061
node_1: feature_name=MTRNR2L2	MTRNR2L2.value <= threshold=2.8784297704696655
node_2: feature_name=TCL1A	TCL1A.value <= threshold=4.798312664031982
node_3: feature_name=MT-CO1	MT-CO1.value <= threshold=0.08947191759943962
node_4: feature_name=NR4A1	NR4A1.value > threshold=1.0725348591804504
node_1622: feature_name=HLA-DQA2	HLA-DQA2.value > threshold=1.9496816992759705

node_2350: feature_name=BCL2A1	BCL2A1.value > threshold=1.1322240233421326
node_2466: feature_name=MS4A1	MS4A1.value <= threshold=2.7321707010269165
node_2467: feature_name=HSP90B1	HSP90B1.value <= threshold=0.8215908706188202
node_2468: feature_name=HLA-DQA1	HLA-DQA1.value > threshold=1.4212530851364136
node_2476: feature_name=MT-CO3	MT-CO3.value <= threshold=0.05051383376121521
node_2477: feature_name=CCR6	CCR6.value <= threshold=-0.2537315636873245
node_2478: feature_name=S100A6	S100A6.value <= threshold=-0.21191135048866272
Class: COVID-19 Severe	
Rules_143	passed counts:33
node_0: feature_name=LTB	LTB.value <= threshold=0.994031548500061
node_1: feature_name=MTRNR2L2	MTRNR2L2.value <= threshold=2.8784297704696655
node_2: feature_name=TCL1A	TCL1A.value <= threshold=4.798312664031982
node_3: feature_name=MT-CO1	MT-CO1.value > threshold=0.08947191759943962
node_2521: feature_name=LTB	LTB.value > threshold=0.6038667261600494
node_4173: feature_name=OAZ1	OAZ1.value <= threshold=0.143283873796463
node_4174: feature_name=PLD4	PLD4.value <= threshold=2.921932101249695
node_4175: feature_name=XIST	XIST.value <= threshold=-0.05067425221204758
node_4176: feature_name=MT-CO1	MT-CO1.value <= threshold=1.345594048500061
node_4177: feature_name=HLA-DQB1	HLA-DQB1.value <= threshold=1.927736520767212
node_4178: feature_name=BACH1	BACH1.value <= threshold=-0.5349778532981873
node_4179: feature_name=CD79B	CD79B.value > threshold=1.6518195271492004
node_4201: feature_name=MT-CO2	MT-CO2.value > threshold=0.5007440596818924
node_4223: feature_name=FAM129C	FAM129C.value <= threshold=4.054540395736694
node_4224: feature_name=MEF2A	MEF2A.value > threshold=-0.4423168897628784
node_4232: feature_name=MEF2C	MEF2C.value <= threshold=3.456770658493042
Class: COVID-19 Critical	
Rules_144	passed counts:33
node_0: feature_name=LTB	LTB.value <= threshold=0.994031548500061

node_1: feature_name=MTRNR2L2	MTRNR2L2.value <= threshold=2.8784297704696655
node_2: feature_name=TCL1A	TCL1A.value <= threshold=4.798312664031982
node_3: feature_name=MT-CO1	MT-CO1.value > threshold=0.08947191759943962
node_2521: feature_name=LTB	LTB.value <= threshold=0.6038667261600494
node_2522: feature_name=HLA-DQB1	HLA-DQB1.value > threshold=2.1053054332733154
node_3550: feature_name=XIST	XIST.value <= threshold=0.7716337237507105
node_3551: feature_name=CD79B	CD79B.value <= threshold=2.9426796436309814
node_3552: feature_name=HLA-DQB1	HLA-DQB1.value <= threshold=2.713674306869507
node_3553: feature_name=SNX9	SNX9.value <= threshold=-0.5404067635536194
node_3554: feature_name=VPREB3	VPREB3.value <= threshold=4.4475414752960205
node_3555: feature_name=RALGPS2	RALGPS2.value > threshold=2.7017585039138794
node_3609: feature_name=MX1	MX1.value <= threshold=1.5204657316207886
node_3610: feature_name=FAM129C	FAM129C.value > threshold=-0.30572274327278137
node_3622: feature_name=CASP8	CASP8.value <= threshold=-0.5367972254753113
node_3623: feature_name=XAF1	XAF1.value <= threshold=1.7484372854232788
node_3624: feature_name=HLA-DRB5	HLA-DRB5.value <= threshold=2.5034819841384888
node_3625: feature_name=MS4A1	MS4A1.value > threshold=2.348857879638672
node_3631: feature_name=RALGPS2	RALGPS2.value <= threshold=6.489912033081055
node_3632: feature_name=FERMT3	FERMT3.value <= threshold=2.727337956428528
Class: Healthy Control	
Rules_145	passed counts:33
node_0: feature_name=LTB	LTB.value <= threshold=0.994031548500061
node_1: feature_name=MTRNR2L2	MTRNR2L2.value <= threshold=2.8784297704696655
node_2: feature_name=TCL1A	TCL1A.value <= threshold=4.798312664031982
node_3: feature_name=MT-CO1	MT-CO1.value > threshold=0.08947191759943962
node_2521: feature_name=LTB	LTB.value <= threshold=0.6038667261600494
node_2522: feature_name=HLA-DQB1	HLA-DQB1.value <= threshold=2.1053054332733154
node_2523: feature_name=SNX9	SNX9.value > threshold=-0.5346486568450928
node_2907: feature_name=NR4A1	NR4A1.value <= threshold=2.5433837175369263

node_2908: feature_name=XIST	XIST.value <= threshold=0.7664470989257097
node_2909: feature_name=CD79B	CD79B.value <= threshold=2.9326847791671753
node_2910: feature_name=NR4A1	NR4A1.value <= threshold=-0.2875557690858841
node_2911: feature_name=CTD-2035E11.3	CTD-2035E11.3.value <= threshold=5.262048006057739
node_2912: feature_name=VPREB3	VPREB3.value <= threshold=4.937594890594482
node_2913: feature_name=IRAK3	IRAK3.value <= threshold=4.592883586883545
node_2914: feature_name=IGJ	IGJ.value <= threshold=7.093410015106201
node_2915: feature_name=WDR74	WDR74.value <= threshold=4.077916383743286
node_2916: feature_name=MEF2C	MEF2C.value <= threshold=4.374382972717285
node_2917: feature_name=HIST1H2AC	HIST1H2AC.value <= threshold=3.743621349334717
node_2918: feature_name=STMN1	STMN1.value <= threshold=6.266510248184204
node_2919: feature_name=RALGPS2	RALGPS2.value <= threshold=5.72194766998291
node_2920: feature_name=HOPX	HOPX.value <= threshold=2.5944807529449463
node_2921: feature_name=MARCKS	MARCKS.value <= threshold=4.622651815414429
node_2922: feature_name=CCL3	CCL3.value <= threshold=0.7484713792800903
node_2923: feature_name=TPM1	TPM1.value <= threshold=6.446193695068359
node_2924: feature_name=PLD4	PLD4.value <= threshold=8.038907289505005
node_2925: feature_name=COTL1	COTL1.value > threshold=-1.8123814463615417
node_2929: feature_name=ID3	ID3.value <= threshold=7.534881353378296
node_2930: feature_name=IRF8	IRF8.value <= threshold=4.855781078338623
node_2931: feature_name=AP3M2	AP3M2.value <= threshold=1.8868122100830078
node_2932: feature_name=FCER2	FCER2.value <= threshold=6.877448558807373
node_2933: feature_name=MTRNR2L2	MTRNR2L2.value <= threshold=2.710945725440979
node_2934: feature_name=GNG11	GNG11.value <= threshold=2.510925054550171
node_2935: feature_name=CD83	CD83.value > threshold=1.9873459935188293
node_2961: feature_name=CD83	CD83.value > threshold=2.0049850940704346
node_2965: feature_name=HES4	HES4.value <= threshold=4.076817035675049
node_2966: feature_name=BCL2A1	BCL2A1.value > threshold=1.1745486855506897
node_2996: feature_name=LTB	LTB.value > threshold=-0.9909347593784332
node_2998: feature_name=VPREB3	VPREB3.value <= threshold=2.2705172896385193

Class: COVID-19 Critical	
Rules_146	passed counts:33
node_0: feature_name=LTB	LTB.value <= threshold=0.994031548500061
node_1: feature_name=MTRNR2L2	MTRNR2L2.value <= threshold=2.8784297704696655
node_2: feature_name=TCL1A	TCL1A.value <= threshold=4.798312664031982
node_3: feature_name=MT-CO1	MT-CO1.value <= threshold=0.08947191759943962
node_4: feature_name=NR4A1	NR4A1.value <= threshold=1.0725348591804504
node_5: feature_name=HLA-DPA1	HLA-DPA1.value > threshold=1.6885469555854797
node_861: feature_name=CD83	CD83.value <= threshold=3.0563642978668213
node_862: feature_name=HLA-DQB1	HLA-DQB1.value > threshold=2.61081063747406
node_1358: feature_name=HLA-DRB5	HLA-DRB5.value > threshold=0.7542744874954224
node_1390: feature_name=HLA-DRB5	HLA-DRB5.value > threshold=2.6244447231292725
node_1516: feature_name=HLA-DQA2	HLA-DQA2.value <= threshold=-0.18513725697994232
node_1517: feature_name=HLA-DPB1	HLA-DPB1.value <= threshold=2.381898283958435
node_1518: feature_name=CYBB	CYBB.value <= threshold=2.3256553411483765
Class: COVID-19 Severe	
Rules_147	passed counts:33
node_0: feature_name=LTB	LTB.value <= threshold=0.994031548500061
node_1: feature_name=MTRNR2L2	MTRNR2L2.value <= threshold=2.8784297704696655
node_2: feature_name=TCL1A	TCL1A.value <= threshold=4.798312664031982
node_3: feature_name=MT-CO1	MT-CO1.value <= threshold=0.08947191759943962
node_4: feature_name=NR4A1	NR4A1.value <= threshold=1.0725348591804504
node_5: feature_name=HLA-DPA1	HLA-DPA1.value <= threshold=1.6885469555854797
node_6: feature_name=HLA-DRB5	HLA-DRB5.value <= threshold=2.1886194944381714
node_7: feature_name=XIST	XIST.value <= threshold=-0.12328458577394485
node_8: feature_name=SYNE1	SYNE1.value > threshold=-0.7835594415664673
node_16: feature_name=MTRNR2L2	MTRNR2L2.value <= threshold=2.4483247995376587
node_17: feature_name=HLA-DQA2	HLA-DQA2.value > threshold=2.227178931236267

node_591: feature_name=BCL2A1	BCL2A1.value > threshold=1.0648428201675415
node_687: feature_name=CD79A	CD79A.value <= threshold=2.49657940864563
node_688: feature_name=MT-ND6	MT-ND6.value <= threshold=0.16280143707990646
node_689: feature_name=TRAF1	TRAF1.value <= threshold=0.8259864822030067
node_690: feature_name=S100A6	S100A6.value <= threshold=-0.15830080211162567
node_691: feature_name=RBKS	RBKS.value <= threshold=4.868927240371704
Class: COVID-19 Severe	
Rules_148	passed counts:32
node_0: feature_name=LTB	LTB.value > threshold=0.994031548500061
node_5264: feature_name=MT-CO1	MT-CO1.value > threshold=-0.4127991199493408
node_5638: feature_name=OAZ1	OAZ1.value > threshold=-0.2640867084264755
node_6112: feature_name=IFI44L	IFI44L.value <= threshold=2.5893263816833496
node_6113: feature_name=TCL1A	TCL1A.value <= threshold=4.201832294464111
node_6114: feature_name=HLA-DQA2	HLA-DQA2.value <= threshold=-0.2270243540406227
node_6115: feature_name=MTRNR2L2	MTRNR2L2.value <= threshold=2.548917293548584
node_6116: feature_name=ATF5	ATF5.value <= threshold=2.041263461112976
node_6117: feature_name=JUN	JUN.value > threshold=-0.616259902715683
node_6189: feature_name=MT-CO3	MT-CO3.value > threshold=0.13483521342277527
node_6351: feature_name=XAF1	XAF1.value <= threshold=3.061717987060547
node_6352: feature_name=MT-CO2	MT-CO2.value <= threshold=1.2605181336402893
node_6353: feature_name=ICAM1	ICAM1.value <= threshold=-0.3340446650981903
node_6354: feature_name=OAZ1	OAZ1.value > threshold=-0.24055946618318558
node_6360: feature_name=PPIF	PPIF.value <= threshold=2.070030093193054
node_6361: feature_name=RALGPS2	RALGPS2.value > threshold=-0.3684315085411072
node_6369: feature_name=ANO6	ANO6.value <= threshold=3.504153251647949
node_6370: feature_name=YWHAH	YWHAH.value <= threshold=4.3182291984558105
node_6371: feature_name=LDLR	LDLR.value <= threshold=2.494291305541992
node_6372: feature_name=HLA-DQA1	HLA-DQA1.value > threshold=-0.5716990232467651
node_6374: feature_name=ABCA1	ABCA1.value <= threshold=3.7662240266799927

node_6375: feature_name=MS4A1	MS4A1.value <= threshold=1.4995208978652954
node_6376: feature_name=MEF2A	MEF2A.value <= threshold=1.7946665287017822
node_6377: feature_name=MT-CYB	MT-CYB.value > threshold=-0.18870697915554047
node_6381: feature_name=CD79B	CD79B.value > threshold=-0.4110361337661743
Class: Healthy Control	
Rules_149	passed counts:32
node_0: feature_name=LTB	LTB.value > threshold=0.994031548500061
node_5264: feature_name=MT-CO1	MT-CO1.value <= threshold=-0.4127991199493408
node_5265: feature_name=MTRNR2L2	MTRNR2L2.value <= threshold=2.4473838806152344
node_5266: feature_name=TCL1A	TCL1A.value <= threshold=5.787256956100464
node_5267: feature_name=PLD4	PLD4.value <= threshold=3.544657826423645
node_5268: feature_name=NR4A1	NR4A1.value <= threshold=0.8490704298019409
node_5269: feature_name=LTB	LTB.value > threshold=1.5252206921577454
node_5471: feature_name=CD79A	CD79A.value <= threshold=2.510846734046936
node_5472: feature_name=CD83	CD83.value > threshold=-0.5842534303665161
node_5476: feature_name=NAIP	NAIP.value <= threshold=3.2324195131659508
node_5477: feature_name=ENDOD1	ENDOD1.value <= threshold=1.9476485773921013
node_5478: feature_name=NT5C3A	NT5C3A.value > threshold=-0.5154461860656738
Class: COVID-19 Critical	
Rules_150	passed counts:32
node_0: feature_name=LTB	LTB.value <= threshold=0.994031548500061
node_1: feature_name=MTRNR2L2	MTRNR2L2.value <= threshold=2.8784297704696655
node_2: feature_name=TCL1A	TCL1A.value <= threshold=4.798312664031982
node_3: feature_name=MT-CO1	MT-CO1.value <= threshold=0.08947191759943962
node_4: feature_name=NR4A1	NR4A1.value > threshold=1.0725348591804504
node_1622: feature_name=HLA-DQA2	HLA-DQA2.value <= threshold=1.9496816992759705
node_1623: feature_name=HLA-DRB5	HLA-DRB5.value <= threshold=2.715766429901123
node_1624: feature_name=HLA-DPA1	HLA-DPA1.value > threshold=1.7050301432609558

node_2056: feature_name=OAZ1	OAZ1.value <= threshold=0.456103190779686
node_2057: feature_name=HLA-DRB5	HLA-DRB5.value <= threshold=0.6839984953403473
node_2058: feature_name=IFI6	IFI6.value <= threshold=-0.5314981639385223
node_2059: feature_name=CD83	CD83.value > threshold=2.6418607234954834
node_2075: feature_name=NFKBID	NFKBID.value <= threshold=3.104505181312561
node_2076: feature_name=MS4A1	MS4A1.value <= threshold=3.1743357181549072
Class: COVID-19 Severe	
Rules_151	passed counts:31
node_0: feature_name=LTB	LTB.value <= threshold=0.994031548500061
node_1: feature_name=MTRNR2L2	MTRNR2L2.value <= threshold=2.8784297704696655
node_2: feature_name=TCL1A	TCL1A.value <= threshold=4.798312664031982
node_3: feature_name=MT-CO1	MT-CO1.value > threshold=0.08947191759943962
node_2521: feature_name=LTB	LTB.value <= threshold=0.6038667261600494
node_2522: feature_name=HLA-DQB1	HLA-DQB1.value > threshold=2.1053054332733154
node_3550: feature_name=XIST	XIST.value <= threshold=0.7716337237507105
node_3551: feature_name=CD79B	CD79B.value <= threshold=2.9426796436309814
node_3552: feature_name=HLA-DQB1	HLA-DQB1.value > threshold=2.713674306869507
node_3854: feature_name=MEF2C	MEF2C.value > threshold=-0.3721291869878769
node_3882: feature_name=IFI44L	IFI44L.value <= threshold=2.5516022443771362
node_3883: feature_name=FCER2	FCER2.value <= threshold=-0.21487552672624588
node_3884: feature_name=HLA-DQA2	HLA-DQA2.value <= threshold=-0.12580305337905884
node_3885: feature_name=SLC7A5	SLC7A5.value > threshold=-0.4906871169805527
node_3945: feature_name=HLA-DQA1	HLA-DQA1.value > threshold=1.6766853332519531
node_3949: feature_name=CCL3	CCL3.value <= threshold=1.0531102418899536
node_3950: feature_name=IGJ	IGJ.value <= threshold=3.4067777395248413
Class: COVID-19 Critical	
Rules_152	passed counts:31
node_0: feature_name=LTB	LTB.value <= threshold=0.994031548500061

node_1: feature_name=MTRNR2L2	MTRNR2L2.value <= threshold=2.8784297704696655
node_2: feature_name=TCL1A	TCL1A.value <= threshold=4.798312664031982
node_3: feature_name=MT-CO1	MT-CO1.value <= threshold=0.08947191759943962
node_4: feature_name=NR4A1	NR4A1.value > threshold=1.0725348591804504
node_1622: feature_name=HLA-DQA2	HLA-DQA2.value <= threshold=1.9496816992759705
node_1623: feature_name=HLA-DRB5	HLA-DRB5.value <= threshold=2.715766429901123
node_1624: feature_name=HLA-DPA1	HLA-DPA1.value > threshold=1.7050301432609558
node_2056: feature_name=OAZ1	OAZ1.value > threshold=0.456103190779686
node_2266: feature_name=XAF1	XAF1.value > threshold=-0.4431648850440979
node_2312: feature_name=ISG15	ISG15.value <= threshold=-0.3511900007724762
node_2313: feature_name=RALGPS2	RALGPS2.value <= threshold=2.2695717811584473
node_2314: feature_name=FCRL1	FCRL1.value <= threshold=4.402489423751831
node_2315: feature_name=PLD4	PLD4.value <= threshold=-0.02754829917103052
node_2316: feature_name=SNX9	SNX9.value <= threshold=3.468294143676758
node_2317: feature_name=ITM2C	ITM2C.value <= threshold=4.142468214035034
Class: COVID-19 Critical	
Rules_153	passed counts:31
node_0: feature_name=LTB	LTB.value <= threshold=0.994031548500061
node_1: feature_name=MTRNR2L2	MTRNR2L2.value <= threshold=2.8784297704696655
node_2: feature_name=TCL1A	TCL1A.value <= threshold=4.798312664031982
node_3: feature_name=MT-CO1	MT-CO1.value <= threshold=0.08947191759943962
node_4: feature_name=NR4A1	NR4A1.value <= threshold=1.0725348591804504
node_5: feature_name=HLA-DPA1	HLA-DPA1.value > threshold=1.6885469555854797
node_861: feature_name=CD83	CD83.value > threshold=3.0563642978668213
node_1539: feature_name=ID1	ID1.value <= threshold=1.4626862406730652
node_1540: feature_name=FTH1	FTH1.value <= threshold=0.2985198348760605
node_1541: feature_name=SESN3	SESN3.value <= threshold=2.7280690670013428
node_1542: feature_name=HOPX	HOPX.value <= threshold=-0.46606285870075226
node_1543: feature_name=ATF3	ATF3.value <= threshold=1.6206613183021545

node_1544: feature_name=HLA-DQA1	HLA-DQA1.value > threshold=2.5666390657424927
node_1562: feature_name=HLA-DQA1	HLA-DQA1.value > threshold=2.702773928642273
node_1564: feature_name=MARCKS	MARCKS.value > threshold=-0.3456065207719803
node_1566: feature_name=MT-CO2	MT-CO2.value > threshold=-1.9448366165161133
node_1568: feature_name=MAP3K2	MAP3K2.value <= threshold=2.7196837663650513
node_1569: feature_name=TCL1A	TCL1A.value <= threshold=3.104050397872925
node_1570: feature_name=PLD4	PLD4.value <= threshold=1.9513476360589266
Class: COVID-19 Critical	
Rules_154	passed counts:31
node_0: feature_name=LTB	LTB.value <= threshold=0.994031548500061
node_1: feature_name=MTRNR2L2	MTRNR2L2.value <= threshold=2.8784297704696655
node_2: feature_name=TCL1A	TCL1A.value <= threshold=4.798312664031982
node_3: feature_name=MT-CO1	MT-CO1.value <= threshold=0.08947191759943962
node_4: feature_name=NR4A1	NR4A1.value <= threshold=1.0725348591804504
node_5: feature_name=HLA-DPA1	HLA-DPA1.value > threshold=1.6885469555854797
node_861: feature_name=CD83	CD83.value <= threshold=3.0563642978668213
node_862: feature_name=HLA-DQB1	HLA-DQB1.value <= threshold=2.61081063747406
node_863: feature_name=MTRNR2L2	MTRNR2L2.value <= threshold=2.0919997692108154
node_864: feature_name=BCL2A1	BCL2A1.value > threshold=1.852618932723999
node_1328: feature_name=ATF5	ATF5.value <= threshold=1.995988368988037
node_1329: feature_name=S100A6	S100A6.value > threshold=-1.6572160124778748
node_1337: feature_name=IFI6	IFI6.value > threshold=-0.6458532810211182
node_1339: feature_name=PELI1	PELI1.value <= threshold=2.5579013824462890
node_1340: feature_name=GPX1	GPX1.value <= threshold=0.6632944941520691
node_1341: feature_name=SPI1	SPI1.value <= threshold=0.8252966105937958
Class: COVID-19 Critical	
Rules_155	passed counts:31
node_0: feature_name=LTB	LTB.value <= threshold=0.994031548500061

node_1: feature_name=MTRNR2L2	MTRNR2L2.value <= threshold=2.8784297704696655
node_2: feature_name=TCL1A	TCL1A.value <= threshold=4.798312664031982
node_3: feature_name=MT-CO1	MT-CO1.value <= threshold=0.08947191759943962
node_4: feature_name=NR4A1	NR4A1.value <= threshold=1.0725348591804504
node_5: feature_name=HLA-DPA1	HLA-DPA1.value <= threshold=1.6885469555854797
node_6: feature_name=HLA-DRB5	HLA-DRB5.value <= threshold=2.1886194944381714
node_7: feature_name=XIST	XIST.value <= threshold=-0.12328458577394485
node_8: feature_name=SYNE1	SYNE1.value > threshold=-0.7835594415664673
node_16: feature_name=MTRNR2L2	MTRNR2L2.value <= threshold=2.4483247995376587
node_17: feature_name=HLA-DQA2	HLA-DQA2.value <= threshold=2.227178931236267
node_18: feature_name=NR4A1	NR4A1.value <= threshold=-0.21380723267793655
node_19: feature_name=IRAK3	IRAK3.value <= threshold=4.27159571647644
node_20: feature_name=IFIT2	IFIT2.value <= threshold=7.8175740242004395
node_21: feature_name=CD79A	CD79A.value <= threshold=2.857242226600647
node_22: feature_name=HIST1H2AC	HIST1H2AC.value <= threshold=5.077470302581787
node_23: feature_name=FTH1	FTH1.value <= threshold=0.4158056080341339
node_24: feature_name=ATF3	ATF3.value <= threshold=3.8028353452682495
node_25: feature_name=OASL	OASL.value <= threshold=3.387580633163452
node_26: feature_name=MT-CO1	MT-CO1.value > threshold=-0.7942646145820618
node_74: feature_name=HOPX	HOPX.value <= threshold=0.8682331740856171
node_75: feature_name=RGS2	RGS2.value <= threshold=1.4589954018592834
node_76: feature_name=AHR	AHR.value <= threshold=3.870481848716736
node_77: feature_name=IGJ	IGJ.value <= threshold=4.902882814407349
node_78: feature_name=CD79B	CD79B.value <= threshold=4.87716269493103
node_79: feature_name=HLA-DQB1	HLA-DQB1.value <= threshold=3.6925491094589233
node_80: feature_name=HLA-DRB5	HLA-DRB5.value <= threshold=2.0926843881607056
node_81: feature_name=MCTP1	MCTP1.value <= threshold=4.010472774505615
node_82: feature_name=GNG11	GNG11.value <= threshold=3.627470850944519
node_83: feature_name=HSP90B1	HSP90B1.value <= threshold=2.0835131406784058
node_84: feature_name=ATF3	ATF3.value <= threshold=1.1142374873161316

node_85: feature_name=MT-ND6	MT-ND6.value <= threshold=2.7328014373779297
node_86: feature_name=COTL1	COTL1.value > threshold=-1.6524587869644165
node_90: feature_name=MT-CYB	MT-CYB.value <= threshold=1.099629819393158
node_91: feature_name=RGCC	RGCC.value <= threshold=1.6908138990402222
node_92: feature_name=ILK	ILK.value <= threshold=3.9497616291046143
node_93: feature_name=CTD-2035E11.3	CTD-2035E11.3.value <= threshold=2.4543306827545166
node_94: feature_name=S100A6	S100A6.value <= threshold=0.2867250442504883
node_95: feature_name=HLA-DRB5	HLA-DRB5.value > threshold=-0.9496813416481018
node_99: feature_name=MT-CO3	MT-CO3.value > threshold=-1.55654776096344
node_103: feature_name=ITGAX	ITGAX.value <= threshold=1.8544010519981384
node_104: feature_name=MEF2C	MEF2C.value <= threshold=4.22724461555481
node_105: feature_name=CXorf57	CXorf57.value <= threshold=5.114444732666016
node_106: feature_name=IRAK3	IRAK3.value <= threshold=3.1017903089523315
node_107: feature_name=DUSP4	DUSP4.value <= threshold=2.9122174978256226
node_108: feature_name=OAZ1	OAZ1.value > threshold=-1.8057305812835693
node_112: feature_name=DAPP1	DAPP1.value <= threshold=4.016228437423706
node_113: feature_name=HSP90B1	HSP90B1.value <= threshold=-1.2981283068656921
node_114: feature_name=JARID2	JARID2.value <= threshold=1.0991151928901672
Class: COVID-19 Critical	
Rules_156	passed counts:30
node_0: feature_name=LTB	LTB.value <= threshold=0.994031548500061
node_1: feature_name=MTRNR2L2	MTRNR2L2.value <= threshold=2.8784297704696655
node_2: feature_name=TCL1A	TCL1A.value <= threshold=4.798312664031982
node_3: feature_name=MT-CO1	MT-CO1.value > threshold=0.08947191759943962
node_2521: feature_name=LTB	LTB.value > threshold=0.6038667261600494
node_4173: feature_name=OAZ1	OAZ1.value > threshold=0.143283873796463
node_4615: feature_name=NR4A1	NR4A1.value <= threshold=0.6621004343032837
node_4616: feature_name=NFKBID	NFKBID.value <= threshold=3.283741593360901
node_4617: feature_name=HLA-DQA1	HLA-DQA1.value <= threshold=2.3435752391815186

node_4618: feature_name=PELI1	PELI1.value <= threshold=1.3271203637123108
node_4619: feature_name=PLD4	PLD4.value > threshold=3.3385335206985474
node_4671: feature_name=LDLRAD4	LDLRAD4.value <= threshold=2.8038166761398315
node_4672: feature_name=CYBB	CYBB.value > threshold=-0.5434646904468536
Class: Healthy Control	
Rules_157	passed counts:30
node_0: feature_name=LTB	LTB.value <= threshold=0.994031548500061
node_1: feature_name=MTRNR2L2	MTRNR2L2.value <= threshold=2.8784297704696655
node_2: feature_name=TCL1A	TCL1A.value <= threshold=4.798312664031982
node_3: feature_name=MT-CO1	MT-CO1.value > threshold=0.08947191759943962
node_2521: feature_name=LTB	LTB.value <= threshold=0.6038667261600494
node_2522: feature_name=HLA-DQB1	HLA-DQB1.value > threshold=2.1053054332733154
node_3550: feature_name=XIST	XIST.value <= threshold=0.7716337237507105
node_3551: feature_name=CD79B	CD79B.value <= threshold=2.9426796436309814
node_3552: feature_name=HLA-DQB1	HLA-DQB1.value > threshold=2.713674306869507
node_3854: feature_name=MEF2C	MEF2C.value > threshold=-0.3721291869878769
node_3882: feature_name=IFI44L	IFI44L.value <= threshold=2.5516022443771362
node_3883: feature_name=FCER2	FCER2.value <= threshold=-0.21487552672624588
node_3884: feature_name=HLA-DQA2	HLA-DQA2.value <= threshold=-0.12580305337905884
node_3885: feature_name=SLC7A5	SLC7A5.value <= threshold=-0.4906871169805527
node_3886: feature_name=VPREB3	VPREB3.value <= threshold=5.492124557495117
node_3887: feature_name=CYBB	CYBB.value <= threshold=2.720046043395996
node_3888: feature_name=CD79A	CD79A.value <= threshold=3.3531476259231567
node_3889: feature_name=HLA-DPA1	HLA-DPA1.value <= threshold=1.6232598423957825
node_3890: feature_name=MT-CYB	MT-CYB.value <= threshold=1.3801624178886414
node_3891: feature_name=HLA-DPB1	HLA-DPB1.value > threshold=-0.6351701319217682
node_3895: feature_name=HLA-DPA1	HLA-DPA1.value > threshold=-0.6622203588485718
node_3899: feature_name=SNX9	SNX9.value > threshold=-0.5651427507400513
node_3903: feature_name=XAF1	XAF1.value > threshold=-0.4552993029356003

Class: COVID-19 Critical	
Rules_158	passed counts:30
node_0: feature_name=LTB	LTB.value <= threshold=0.994031548500061
node_1: feature_name=MTRNR2L2	MTRNR2L2.value <= threshold=2.8784297704696655
node_2: feature_name=TCL1A	TCL1A.value <= threshold=4.798312664031982
node_3: feature_name=MT-CO1	MT-CO1.value > threshold=0.08947191759943962
node_2521: feature_name=LTB	LTB.value <= threshold=0.6038667261600494
node_2522: feature_name=HLA-DQB1	HLA-DQB1.value <= threshold=2.1053054332733154
node_2523: feature_name=SNX9	SNX9.value > threshold=-0.5346486568450928
node_2907: feature_name=NR4A1	NR4A1.value <= threshold=2.5433837175369263
node_2908: feature_name=XIST	XIST.value <= threshold=0.7664470989257097
node_2909: feature_name=CD79B	CD79B.value <= threshold=2.9326847791671753
node_2910: feature_name=NR4A1	NR4A1.value > threshold=-0.2875557690858841
node_3096: feature_name=NR4A3	NR4A3.value > threshold=3.314968705177307
node_3328: feature_name=MT-CO3	MT-CO3.value > threshold=0.35808728635311127
node_3332: feature_name=STAG3	STAG3.value <= threshold=-0.18329568207263947
node_3333: feature_name=SLC7A5	SLC7A5.value <= threshold=2.876398801803589
node_3334: feature_name=SNX29P2	SNX29P2.value <= threshold=5.85232138633728
node_3335: feature_name=GPX1	GPX1.value <= threshold=0.11650833487510681
Class: COVID-19 Critical	
Rules_159	passed counts:30
node_0: feature_name=LTB	LTB.value <= threshold=0.994031548500061
node_1: feature_name=MTRNR2L2	MTRNR2L2.value <= threshold=2.8784297704696655
node_2: feature_name=TCL1A	TCL1A.value <= threshold=4.798312664031982
node_3: feature_name=MT-CO1	MT-CO1.value > threshold=0.08947191759943962
node_2521: feature_name=LTB	LTB.value <= threshold=0.6038667261600494
node_2522: feature_name=HLA-DQB1	HLA-DQB1.value <= threshold=2.1053054332733154
node_2523: feature_name=SNX9	SNX9.value > threshold=-0.5346486568450928

node_2907: feature_name=NR4A1	NR4A1.value <= threshold=2.5433837175369263
node_2908: feature_name=XIST	XIST.value <= threshold=0.7664470989257097
node_2909: feature_name=CD79B	CD79B.value <= threshold=2.9326847791671753
node_2910: feature_name=NR4A1	NR4A1.value <= threshold=-0.2875557690858841
node_2911: feature_name=CTD-2035E11.3	CTD-2035E11.3.value <= threshold=5.262048006057739
node_2912: feature_name=VPREB3	VPREB3.value <= threshold=4.937594890594482
node_2913: feature_name=IRAK3	IRAK3.value <= threshold=4.592883586883545
node_2914: feature_name=IGJ	IGJ.value <= threshold=7.093410015106201
node_2915: feature_name=WDR74	WDR74.value <= threshold=4.077916383743286
node_2916: feature_name=MEF2C	MEF2C.value <= threshold=4.374382972717285
node_2917: feature_name=HIST1H2AC	HIST1H2AC.value <= threshold=3.743621349334717
node_2918: feature_name=STMN1	STMN1.value <= threshold=6.266510248184204
node_2919: feature_name=RALGPS2	RALGPS2.value <= threshold=5.72194766998291
node_2920: feature_name=HOPX	HOPX.value <= threshold=2.5944807529449463
node_2921: feature_name=MARCKS	MARCKS.value <= threshold=4.622651815414429
node_2922: feature_name=CCL3	CCL3.value > threshold=0.7484713792800903
node_3032: feature_name=VCL	VCL.value <= threshold=2.014119505882263
node_3033: feature_name=HBB	HBB.value <= threshold=1.5370113849639893
node_3034: feature_name=HOPX	HOPX.value <= threshold=1.666459083557129
Class: COVID-19 Critical	
Rules_160	passed counts:30
node_0: feature_name=LTB	LTB.value <= threshold=0.994031548500061
node_1: feature_name=MTRNR2L2	MTRNR2L2.value <= threshold=2.8784297704696655
node_2: feature_name=TCL1A	TCL1A.value <= threshold=4.798312664031982
node_3: feature_name=MT-CO1	MT-CO1.value <= threshold=0.08947191759943962
node_4: feature_name=NR4A1	NR4A1.value > threshold=1.0725348591804504
node_1622: feature_name=HLA-DQA2	HLA-DQA2.value <= threshold=1.9496816992759705
node_1623: feature_name=HLA-DRB5	HLA-DRB5.value <= threshold=2.715766429901123
node_1624: feature_name=HLA-DPA1	HLA-DPA1.value <= threshold=1.7050301432609558

node_1625: feature_name=CD83	CD83.value > threshold=2.783489227294922
node_1873: feature_name=IFI44L	IFI44L.value <= threshold=1.5938722491264343
node_1874: feature_name=PLD4	PLD4.value <= threshold=4.101001739501953
node_1875: feature_name=RHOB	RHOB.value <= threshold=1.304835319519043
node_1876: feature_name=PPIF	PPIF.value <= threshold=2.2943923473358154
node_1877: feature_name=OAZ1	OAZ1.value > threshold=0.04260866530239582
node_1935: feature_name=RGS1	RGS1.value <= threshold=-0.13325228542089462
node_1936: feature_name=BCL2A1	BCL2A1.value <= threshold=2.528669595718384
node_1937: feature_name=RALGPS2	RALGPS2.value <= threshold=2.1082775592803955
node_1938: feature_name=IRF8	IRF8.value > threshold=-0.41594912111759186
node_1940: feature_name=NR4A1	NR4A1.value <= threshold=4.344374179840088
node_1941: feature_name=CD83	CD83.value > threshold=2.801012873649597
Class: COVID-19 Critical	
Rules_161	passed counts:30
node_0: feature_name=LTB	LTB.value <= threshold=0.994031548500061
node_1: feature_name=MTRNR2L2	MTRNR2L2.value <= threshold=2.8784297704696655
node_2: feature_name=TCL1A	TCL1A.value <= threshold=4.798312664031982
node_3: feature_name=MT-CO1	MT-CO1.value <= threshold=0.08947191759943962
node_4: feature_name=NR4A1	NR4A1.value <= threshold=1.0725348591804504
node_5: feature_name=HLA-DPA1	HLA-DPA1.value <= threshold=1.6885469555854797
node_6: feature_name=HLA-DRB5	HLA-DRB5.value <= threshold=2.1886194944381714
node_7: feature_name=XIST	XIST.value <= threshold=-0.12328458577394485
node_8: feature_name=SYNE1	SYNE1.value > threshold=-0.7835594415664673
node_16: feature_name=MTRNR2L2	MTRNR2L2.value <= threshold=2.4483247995376587
node_17: feature_name=HLA-DQA2	HLA-DQA2.value <= threshold=2.227178931236267
node_18: feature_name=NR4A1	NR4A1.value <= threshold=-0.21380723267793655
node_19: feature_name=IRAK3	IRAK3.value <= threshold=4.27159571647644
node_20: feature_name=IFIT2	IFIT2.value <= threshold=7.8175740242004395
node_21: feature_name=CD79A	CD79A.value <= threshold=2.857242226600647

node_22: feature_name=HIST1H2AC	HIST1H2AC.value <= threshold=5.077470302581787
node_23: feature_name=FTH1	FTH1.value <= threshold=0.4158056080341339
node_24: feature_name=ATF3	ATF3.value <= threshold=3.8028353452682495
node_25: feature_name=OASL	OASL.value <= threshold=3.387580633163452
node_26: feature_name=MT-CO1	MT-CO1.value > threshold=-0.7942646145820618
node_74: feature_name=HOPX	HOPX.value <= threshold=0.8682331740856171
node_75: feature_name=RGS2	RGS2.value <= threshold=1.4589954018592834
node_76: feature_name=AHR	AHR.value <= threshold=3.870481848716736
node_77: feature_name=IGJ	IGJ.value <= threshold=4.902882814407349
node_78: feature_name=CD79B	CD79B.value <= threshold=4.87716269493103
node_79: feature_name=HLA-DQB1	HLA-DQB1.value <= threshold=3.6925491094589233
node_80: feature_name=HLA-DRB5	HLA-DRB5.value <= threshold=2.0926843881607056
node_81: feature_name=MCTP1	MCTP1.value <= threshold=4.010472774505615
node_82: feature_name=GNG11	GNG11.value <= threshold=3.627470850944519
node_83: feature_name=HSP90B1	HSP90B1.value <= threshold=2.0835131406784058
node_84: feature_name=ATF3	ATF3.value <= threshold=1.1142374873161316
node_85: feature_name=MT-ND6	MT-ND6.value <= threshold=2.7328014373779297
node_86: feature_name=COTL1	COTL1.value > threshold=-1.6524587869644165
node_90: feature_name=MT-CYB	MT-CYB.value <= threshold=1.099629819393158
node_91: feature_name=RGCC	RGCC.value <= threshold=1.6908138990402222
node_92: feature_name=ILK	ILK.value <= threshold=3.9497616291046143
node_93: feature_name=CTD-2035E11.3	CTD-2035E11.3.value <= threshold=2.4543306827545166
node_94: feature_name=S100A6	S100A6.value <= threshold=0.2867250442504883
node_95: feature_name=HLA-DRB5	HLA-DRB5.value > threshold=-0.9496813416481018
node_99: feature_name=MT-CO3	MT-CO3.value > threshold=-1.55654776096344
node_103: feature_name=ITGAX	ITGAX.value <= threshold=1.8544010519981384
node_104: feature_name=MEF2C	MEF2C.value <= threshold=4.22724461555481
node_105: feature_name=CXorf57	CXorf57.value <= threshold=5.114444732666016
node_106: feature_name=IRAK3	IRAK3.value <= threshold=3.1017903089523315
node_107: feature_name=DUSP4	DUSP4.value <= threshold=2.9122174978256226

node_108: feature_name=OAZ1	OAZ1.value > threshold=-1.8057305812835693
node_112: feature_name=DAPP1	DAPP1.value > threshold=4.016228437423706
node_132: feature_name=ANO6	ANO6.value > threshold=-0.36521002650260925
node_134: feature_name=FTH1	FTH1.value <= threshold=0.30795927345752716
Class: COVID-19 Critical	
Rules_162	passed counts:29
node_0: feature_name=LTB	LTB.value > threshold=0.994031548500061
node_5264: feature_name=MT-CO1	MT-CO1.value > threshold=-0.4127991199493408
node_5638: feature_name=OAZ1	OAZ1.value > threshold=-0.2640867084264755
node_6112: feature_name=IFI44L	IFI44L.value <= threshold=2.5893263816833496
node_6113: feature_name=TCL1A	TCL1A.value <= threshold=4.201832294464111
node_6114: feature_name=HLA-DQA2	HLA-DQA2.value <= threshold=-0.2270243540406227
node_6115: feature_name=MTRNR2L2	MTRNR2L2.value <= threshold=2.548917293548584
node_6116: feature_name=ATF5	ATF5.value <= threshold=2.041263461112976
node_6117: feature_name=JUN	JUN.value <= threshold=-0.616259902715683
node_6118: feature_name=NFKBID	NFKBID.value > threshold=1.5264258980751038
node_6166: feature_name=NR4A1	NR4A1.value <= threshold=0.6553736329078674
node_6167: feature_name=WDR74	WDR74.value > threshold=-0.49743133783340454
node_6179: feature_name=XIST	XIST.value <= threshold=1.3986704498529434
node_6180: feature_name=HLA-DRB5	HLA-DRB5.value <= threshold=3.0251117944717407
Class: COVID-19 Critical	
Rules_163	passed counts:29
node_0: feature_name=LTB	LTB.value <= threshold=0.994031548500061
node_1: feature_name=MTRNR2L2	MTRNR2L2.value <= threshold=2.8784297704696655
node_2: feature_name=TCL1A	TCL1A.value <= threshold=4.798312664031982
node_3: feature_name=MT-CO1	MT-CO1.value > threshold=0.08947191759943962
node_2521: feature_name=LTB	LTB.value > threshold=0.6038667261600494
node_4173: feature_name=OAZ1	OAZ1.value > threshold=0.143283873796463

node_4615: feature_name=NR4A1	NR4A1.value <= threshold=0.6621004343032837
node_4616: feature_name=NFKBID	NFKBID.value <= threshold=3.283741593360901
node_4617: feature_name=HLA-DQA1	HLA-DQA1.value <= threshold=2.3435752391815186
node_4618: feature_name=PELI1	PELI1.value <= threshold=1.3271203637123108
node_4619: feature_name=PLD4	PLD4.value <= threshold=3.3385335206985474
node_4620: feature_name=MEF2A	MEF2A.value > threshold=1.5926940441131592
node_4658: feature_name=STAG3	STAG3.value > threshold=-0.2016931176185608
node_4668: feature_name=HLA-DQA2	HLA-DQA2.value > threshold=-0.3469753861427307
Class: COVID-19 Critical	
Rules_164	passed counts:29
node_0: feature_name=LTB	LTB.value <= threshold=0.994031548500061
node_1: feature_name=MTRNR2L2	MTRNR2L2.value <= threshold=2.8784297704696655
node_2: feature_name=TCL1A	TCL1A.value <= threshold=4.798312664031982
node_3: feature_name=MT-CO1	MT-CO1.value > threshold=0.08947191759943962
node_2521: feature_name=LTB	LTB.value <= threshold=0.6038667261600494
node_2522: feature_name=HLA-DQB1	HLA-DQB1.value > threshold=2.1053054332733154
node_3550: feature_name=XIST	XIST.value <= threshold=0.7716337237507105
node_3551: feature_name=CD79B	CD79B.value <= threshold=2.9426796436309814
node_3552: feature_name=HLA-DQB1	HLA-DQB1.value <= threshold=2.713674306869507
node_3553: feature_name=SNX9	SNX9.value > threshold=-0.5404067635536194
node_3653: feature_name=LTB	LTB.value <= threshold=0.021708179265260696
node_3654: feature_name=HLA-DRB5	HLA-DRB5.value > threshold=-0.5633223652839661
node_3660: feature_name=NR4A1	NR4A1.value > threshold=2.2327845096588135
node_3736: feature_name=NFKBID	NFKBID.value > threshold=-0.4191274344921112
node_3738: feature_name=PARP14	PARP14.value > threshold=-0.49970389902591705
node_3752: feature_name=STAG3	STAG3.value <= threshold=2.935676693916321
node_3753: feature_name=MT-ND6	MT-ND6.value <= threshold=1.952991008758545
Class: COVID-19 Critical	

Rules_165	passed counts:29
node_0: feature_name=LTB	LTB.value <= threshold=0.994031548500061
node_1: feature_name=MTRNR2L2	MTRNR2L2.value <= threshold=2.8784297704696655
node_2: feature_name=TCL1A	TCL1A.value <= threshold=4.798312664031982
node_3: feature_name=MT-CO1	MT-CO1.value <= threshold=0.08947191759943962
node_4: feature_name=NR4A1	NR4A1.value > threshold=1.0725348591804504
node_1622: feature_name=HLA-DQA2	HLA-DQA2.value <= threshold=1.9496816992759705
node_1623: feature_name=HLA-DRB5	HLA-DRB5.value <= threshold=2.715766429901123
node_1624: feature_name=HLA-DPA1	HLA-DPA1.value > threshold=1.7050301432609558
node_2056: feature_name=OAZ1	OAZ1.value <= threshold=0.456103190779686
node_2057: feature_name=HLA-DRB5	HLA-DRB5.value <= threshold=0.6839984953403473
node_2058: feature_name=IFI6	IFI6.value > threshold=-0.5314981639385223
node_2082: feature_name=XAF1	XAF1.value > threshold=-0.4537480026483536
node_2086: feature_name=RALGPS2	RALGPS2.value <= threshold=3.8138545751571655
node_2087: feature_name=MMD	MMD.value <= threshold=3.2687419652938843
node_2088: feature_name=PLEC	PLEC.value <= threshold=1.4888519048690796
node_2089: feature_name=TLN1	TLN1.value <= threshold=1.424235463142395
node_2090: feature_name=CD38	CD38.value <= threshold=1.6792335882782936
Class: COVID-19 Critical	
Rules_166	passed counts:29
node_0: feature_name=LTB	LTB.value <= threshold=0.994031548500061
node_1: feature_name=MTRNR2L2	MTRNR2L2.value <= threshold=2.8784297704696655
node_2: feature_name=TCL1A	TCL1A.value <= threshold=4.798312664031982
node_3: feature_name=MT-CO1	MT-CO1.value <= threshold=0.08947191759943962
node_4: feature_name=NR4A1	NR4A1.value <= threshold=1.0725348591804504
node_5: feature_name=HLA-DPA1	HLA-DPA1.value > threshold=1.6885469555854797
node_861: feature_name=CD83	CD83.value <= threshold=3.0563642978668213
node_862: feature_name=HLA-DQB1	HLA-DQB1.value <= threshold=2.61081063747406
node_863: feature_name=MTRNR2L2	MTRNR2L2.value <= threshold=2.0919997692108154

node_864: feature_name=BCL2A1	BCL2A1.value <= threshold=1.852618932723999
node_865: feature_name=CD79B	CD79B.value <= threshold=4.352142333984375
node_866: feature_name=TNFAIP3	TNFAIP3.value <= threshold=-0.0449496041983366
node_867: feature_name=MT-CO1	MT-CO1.value <= threshold=-0.08531957119703293
node_868: feature_name=MTRNR2L8	MTRNR2L8.value <= threshold=0.8770350813865662
node_869: feature_name=EGR1	EGR1.value > threshold=-0.32270750403404236
node_1015: feature_name=NR4A3	NR4A3.value <= threshold=1.9085431098937988
node_1016: feature_name=ID3	ID3.value <= threshold=2.776647686958313
node_1017: feature_name=XIST	XIST.value <= threshold=2.618046522140503
node_1018: feature_name=CD79B	CD79B.value <= threshold=3.818994164466858
node_1019: feature_name=NFKBID	NFKBID.value <= threshold=3.4191306829452515
node_1020: feature_name=MIAT	MIAT.value <= threshold=3.532225251197815
node_1021: feature_name=STAG3	STAG3.value <= threshold=9.534917831420898
node_1022: feature_name=OASL	OASL.value <= threshold=2.3923760652542114
node_1023: feature_name=HLA-DQB1	HLA-DQB1.value <= threshold=2.572031259536743
node_1024: feature_name=CD79A	CD79A.value > threshold=0.1879405975341797
node_1028: feature_name=NR4A1	NR4A1.value > threshold=0.7387348711490631
node_1086: feature_name=WDR74	WDR74.value > threshold=-0.501454085111618
node_1090: feature_name=VCL	VCL.value <= threshold=0.7375603318214417
node_1091: feature_name=HSP90B1	HSP90B1.value <= threshold=1.842017650604248
node_1092: feature_name=MT-CO3	MT-CO3.value <= threshold=0.5361095666885376
node_1093: feature_name=GPX1	GPX1.value <= threshold=0.5040512979030609
Class: COVID-19 Critical	
Rules_167	passed counts:29
node_0: feature_name=LTB	LTB.value <= threshold=0.994031548500061
node_1: feature_name=MTRNR2L2	MTRNR2L2.value <= threshold=2.8784297704696655
node_2: feature_name=TCL1A	TCL1A.value <= threshold=4.798312664031982
node_3: feature_name=MT-CO1	MT-CO1.value <= threshold=0.08947191759943962
node_4: feature_name=NR4A1	NR4A1.value <= threshold=1.0725348591804504

node_5: feature_name=HLA-DPA1	HLA-DPA1.value <= threshold=1.6885469555854797
node_6: feature_name=HLA-DRB5	HLA-DRB5.value > threshold=2.1886194944381714
node_738: feature_name=HLA-DQA2	HLA-DQA2.value > threshold=-0.20105575770139694
node_804: feature_name=CYBB	CYBB.value <= threshold=2.9090086221694946
node_805: feature_name=VPREB3	VPREB3.value <= threshold=7.757248401641846
node_806: feature_name=IL8	IL8.value <= threshold=1.7155421376228333
node_807: feature_name=ATF5	ATF5.value <= threshold=6.124105930328369
node_808: feature_name=RP11-731F5.2	RP11-731F5.2.value <= threshold=7.633600234985352
node_809: feature_name=OASL	OASL.value <= threshold=0.9932635426521301
node_810: feature_name=ID1	ID1.value <= threshold=2.8093127012252808
node_811: feature_name=GRASP	GRASP.value <= threshold=2.3934966325759888
node_812: feature_name=KIAA0125	KIAA0125.value <= threshold=9.68092966079712
node_813: feature_name=MTRNR2L2	MTRNR2L2.value <= threshold=1.901122272014618
node_814: feature_name=HLA-DPA1	HLA-DPA1.value > threshold=-0.7204518914222717
node_816: feature_name=NT5C3A	NT5C3A.value <= threshold=4.120891809463501
node_817: feature_name=IRAK3	IRAK3.value <= threshold=4.327898621559143
node_818: feature_name=PLA2G12A	PLA2G12A.value <= threshold=4.008805990219116
node_819: feature_name=CD79A	CD79A.value <= threshold=1.521829903125763
node_820: feature_name=MEF2C	MEF2C.value > threshold=-0.37839841842651367
node_824: feature_name=TCL1A	TCL1A.value <= threshold=1.8323483243584633
Class: COVID-19 Critical	
Rules_168	passed counts:28
node_0: feature_name=LTB	LTB.value > threshold=0.994031548500061
node_5264: feature_name=MT-CO1	MT-CO1.value > threshold=-0.4127991199493408
node_5638: feature_name=OAZ1	OAZ1.value <= threshold=-0.2640867084264755
node_5639: feature_name=CD79B	CD79B.value > threshold=2.2998517751693726
node_5925: feature_name=SNX9	SNX9.value <= threshold=2.4445992708206177
node_5926: feature_name=TCL1A	TCL1A.value <= threshold=2.6938670873641968
node_5927: feature_name=PLD4	PLD4.value <= threshold=-0.06434785947203636

node_5928: feature_name=ICAM1	ICAM1.value > threshold=-0.2820792943239212
node_5996: feature_name=MT-CO2	MT-CO2.value > threshold=-0.4205213189125061
node_6000: feature_name=HSP90B1	HSP90B1.value > threshold=-1.2168694138526917
node_6006: feature_name=MEF2C	MEF2C.value <= threshold=3.4065632820129395
Class: COVID-19 Critical	
Rules_169	passed counts:28
node_0: feature_name=LTB	LTB.value <= threshold=0.994031548500061
node_1: feature_name=MTRNR2L2	MTRNR2L2.value <= threshold=2.8784297704696655
node_2: feature_name=TCL1A	TCL1A.value <= threshold=4.798312664031982
node_3: feature_name=MT-CO1	MT-CO1.value > threshold=0.08947191759943962
node_2521: feature_name=LTB	LTB.value > threshold=0.6038667261600494
node_4173: feature_name=OAZ1	OAZ1.value <= threshold=0.143283873796463
node_4174: feature_name=PLD4	PLD4.value <= threshold=2.921932101249695
node_4175: feature_name=XIST	XIST.value > threshold=-0.05067425221204758
node_4539: feature_name=HLA-DQB1	HLA-DQB1.value > threshold=2.1246572732925415
node_4549: feature_name=HLA-DQA2	HLA-DQA2.value <= threshold=-0.12850885093212128
node_4550: feature_name=MT-CO1	MT-CO1.value <= threshold=1.8228539824485779
node_4551: feature_name=BANK1	BANK1.value <= threshold=4.882656097412109
node_4552: feature_name=RNF144B	RNF144B.value <= threshold=2.4011234045028687
Class: COVID-19 Severe	
Rules_170	passed counts:28
node_0: feature_name=LTB	LTB.value <= threshold=0.994031548500061
node_1: feature_name=MTRNR2L2	MTRNR2L2.value <= threshold=2.8784297704696655
node_2: feature_name=TCL1A	TCL1A.value <= threshold=4.798312664031982
node_3: feature_name=MT-CO1	MT-CO1.value > threshold=0.08947191759943962
node_2521: feature_name=LTB	LTB.value <= threshold=0.6038667261600494
node_2522: feature_name=HLA-DQB1	HLA-DQB1.value > threshold=2.1053054332733154
node_3550: feature_name=XIST	XIST.value <= threshold=0.7716337237507105

node_3551: feature_name=CD79B	CD79B.value <= threshold=2.9426796436309814
node_3552: feature_name=HLA-DQB1	HLA-DQB1.value <= threshold=2.713674306869507
node_3553: feature_name=SNX9	SNX9.value > threshold=-0.5404067635536194
node_3653: feature_name=LTB	LTB.value <= threshold=0.021708179265260696
node_3654: feature_name=HLA-DRB5	HLA-DRB5.value > threshold=-0.5633223652839661
node_3660: feature_name=NR4A1	NR4A1.value <= threshold=2.2327845096588135
node_3661: feature_name=FCRL1	FCRL1.value <= threshold=8.662389755249023
node_3662: feature_name=BANK1	BANK1.value <= threshold=4.776419401168823
node_3663: feature_name=IGLL5	IGLL5.value <= threshold=1.4806077480316162
node_3664: feature_name=COTL1	COTL1.value > threshold=-1.2335613369941711
node_3670: feature_name=VCL	VCL.value <= threshold=4.393002271652222
node_3671: feature_name=HLA-DQA1	HLA-DQA1.value > threshold=-0.3948168456554413
node_3673: feature_name=HLA-DRB1	HLA-DRB1.value > threshold=-0.6657836139202118
node_3677: feature_name=GNG11	GNG11.value <= threshold=3.942102313041687
node_3678: feature_name=MT-CO2	MT-CO2.value <= threshold=0.10530227422714233
node_3679: feature_name=FCER2	FCER2.value <= threshold=3.9506202936172485
node_3680: feature_name=ISG15	ISG15.value <= threshold=2.3231236934661865
node_3681: feature_name=S100A6	S100A6.value > threshold=-1.7819254994392395
Class: COVID-19 Critical	
Rules_171	passed counts:28
node_0: feature_name=LTB	LTB.value <= threshold=0.994031548500061
node_1: feature_name=MTRNR2L2	MTRNR2L2.value <= threshold=2.8784297704696655
node_2: feature_name=TCL1A	TCL1A.value <= threshold=4.798312664031982
node_3: feature_name=MT-CO1	MT-CO1.value <= threshold=0.08947191759943962
node_4: feature_name=NR4A1	NR4A1.value > threshold=1.0725348591804504
node_1622: feature_name=HLA-DQA2	HLA-DQA2.value <= threshold=1.9496816992759705
node_1623: feature_name=HLA-DRB5	HLA-DRB5.value <= threshold=2.715766429901123
node_1624: feature_name=HLA-DPA1	HLA-DPA1.value > threshold=1.7050301432609558
node_2056: feature_name=OAZ1	OAZ1.value > threshold=0.456103190779686

node_2266: feature_name=XAF1	XAF1.value > threshold=-0.4431648850440979
node_2312: feature_name=ISG15	ISG15.value > threshold=-0.3511900007724762
Class: COVID-19 Critical	
Rules_172	passed counts:28
node_0: feature_name=LTB	LTB.value <= threshold=0.994031548500061
node_1: feature_name=MTRNR2L2	MTRNR2L2.value <= threshold=2.8784297704696655
node_2: feature_name=TCL1A	TCL1A.value <= threshold=4.798312664031982
node_3: feature_name=MT-CO1	MT-CO1.value <= threshold=0.08947191759943962
node_4: feature_name=NR4A1	NR4A1.value <= threshold=1.0725348591804504
node_5: feature_name=HLA-DPA1	HLA-DPA1.value <= threshold=1.6885469555854797
node_6: feature_name=HLA-DRB5	HLA-DRB5.value > threshold=2.1886194944381714
node_738: feature_name=HLA-DQA2	HLA-DQA2.value <= threshold=-0.20105575770139694
node_739: feature_name=HLA-DQA1	HLA-DQA1.value <= threshold=1.7414769530296326
node_740: feature_name=TNFAIP3	TNFAIP3.value <= threshold=-0.5488012433052063
node_741: feature_name=MS4A1	MS4A1.value > threshold=0.3420938551425934
node_745: feature_name=FCER2	FCER2.value > threshold=-0.24763920158147812
node_747: feature_name=HLA-DPA1	HLA-DPA1.value <= threshold=1.6616552472114563
Class: COVID-19 Severe	
Rules_173	passed counts:28
node_0: feature_name=LTB	LTB.value <= threshold=0.994031548500061
node_1: feature_name=MTRNR2L2	MTRNR2L2.value <= threshold=2.8784297704696655
node_2: feature_name=TCL1A	TCL1A.value <= threshold=4.798312664031982
node_3: feature_name=MT-CO1	MT-CO1.value <= threshold=0.08947191759943962
node_4: feature_name=NR4A1	NR4A1.value <= threshold=1.0725348591804504
node_5: feature_name=HLA-DPA1	HLA-DPA1.value <= threshold=1.6885469555854797
node_6: feature_name=HLA-DRB5	HLA-DRB5.value <= threshold=2.1886194944381714
node_7: feature_name=XIST	XIST.value <= threshold=-0.12328458577394485
node_8: feature_name=SYNE1	SYNE1.value > threshold=-0.7835594415664673

node_16: feature_name=MTRNR2L2	MTRNR2L2.value <= threshold=2.4483247995376587
node_17: feature_name=HLA-DQA2	HLA-DQA2.value <= threshold=2.227178931236267
node_18: feature_name=NR4A1	NR4A1.value <= threshold=-0.21380723267793655
node_19: feature_name=IRAK3	IRAK3.value <= threshold=4.27159571647644
node_20: feature_name=IFIT2	IFIT2.value <= threshold=7.8175740242004395
node_21: feature_name=CD79A	CD79A.value <= threshold=2.857242226600647
node_22: feature_name=HIST1H2AC	HIST1H2AC.value <= threshold=5.077470302581787
node_23: feature_name=FTH1	FTH1.value <= threshold=0.4158056080341339
node_24: feature_name=ATF3	ATF3.value <= threshold=3.8028353452682495
node_25: feature_name=OASL	OASL.value <= threshold=3.387580633163452
node_26: feature_name=MT-CO1	MT-CO1.value > threshold=-0.7942646145820618
node_74: feature_name=HOPX	HOPX.value > threshold=0.8682331740856171
node_250: feature_name=CD22	CD22.value > threshold=-0.23878339678049088
node_254: feature_name=MT-CO2	MT-CO2.value <= threshold=0.39438967406749725
node_255: feature_name=COTL1	COTL1.value <= threshold=-0.0382253173738718
node_256: feature_name=HLA-DQB1	HLA-DQB1.value <= threshold=2.292426109313965
node_257: feature_name=CD79B	CD79B.value <= threshold=3.1534523963928223
node_258: feature_name=FTH1	FTH1.value > threshold=-1.4676445126533508
node_260: feature_name=CD79B	CD79B.value > threshold=-0.4302150160074234
node_262: feature_name=HOPX	HOPX.value <= threshold=3.0209115743637085
Class: COVID-19 Critical	
Rules_174	passed counts:28
node_0: feature_name=LTB	LTB.value <= threshold=0.9940315485000061
node_1: feature_name=MTRNR2L2	MTRNR2L2.value <= threshold=2.8784297704696655
node_2: feature_name=TCL1A	TCL1A.value <= threshold=4.798312664031982
node_3: feature_name=MT-CO1	MT-CO1.value <= threshold=0.08947191759943962
node_4: feature_name=NR4A1	NR4A1.value <= threshold=1.0725348591804504
node_5: feature_name=HLA-DPA1	HLA-DPA1.value <= threshold=1.6885469555854797
node_6: feature_name=HLA-DRB5	HLA-DRB5.value <= threshold=2.1886194944381714

node_7: feature_name=XIST	XIST.value <= threshold=-0.12328458577394485
node_8: feature_name=SYNE1	SYNE1.value > threshold=-0.7835594415664673
node_16: feature_name=MTRNR2L2	MTRNR2L2.value <= threshold=2.4483247995376587
node_17: feature_name=HLA-DQA2	HLA-DQA2.value <= threshold=2.227178931236267
node_18: feature_name=NR4A1	NR4A1.value <= threshold=-0.21380723267793655
node_19: feature_name=IRAK3	IRAK3.value <= threshold=4.27159571647644
node_20: feature_name=IFIT2	IFIT2.value <= threshold=7.8175740242004395
node_21: feature_name=CD79A	CD79A.value <= threshold=2.857242226600647
node_22: feature_name=HIST1H2AC	HIST1H2AC.value <= threshold=5.077470302581787
node_23: feature_name=FTH1	FTH1.value <= threshold=0.4158056080341339
node_24: feature_name=ATF3	ATF3.value <= threshold=3.8028353452682495
node_25: feature_name=OASL	OASL.value <= threshold=3.387580633163452
node_26: feature_name=MT-CO1	MT-CO1.value > threshold=-0.7942646145820618
node_74: feature_name=HOPX	HOPX.value <= threshold=0.8682331740856171
node_75: feature_name=RGS2	RGS2.value <= threshold=1.4589954018592834
node_76: feature_name=AHR	AHR.value <= threshold=3.870481848716736
node_77: feature_name=IGJ	IGJ.value <= threshold=4.902882814407349
node_78: feature_name=CD79B	CD79B.value <= threshold=4.87716269493103
node_79: feature_name=HLA-DQB1	HLA-DQB1.value <= threshold=3.6925491094589233
node_80: feature_name=HLA-DRB5	HLA-DRB5.value <= threshold=2.0926843881607056
node_81: feature_name=MCTP1	MCTP1.value <= threshold=4.010472774505615
node_82: feature_name=GNG11	GNG11.value <= threshold=3.627470850944519
node_83: feature_name=HSP90B1	HSP90B1.value <= threshold=2.0835131406784058
node_84: feature_name=ATF3	ATF3.value <= threshold=1.1142374873161316
node_85: feature_name=MT-ND6	MT-ND6.value <= threshold=2.7328014373779297
node_86: feature_name=COTL1	COTL1.value > threshold=-1.6524587869644165
node_90: feature_name=MT-CYB	MT-CYB.value <= threshold=1.099629819393158
node_91: feature_name=RGCC	RGCC.value <= threshold=1.6908138990402222
node_92: feature_name=ILK	ILK.value <= threshold=3.9497616291046143
node_93: feature_name=CTD-2035E11.3	CTD-2035E11.3.value > threshold=2.4543306827545166

node_157: feature_name=HLA-DPA1	HLA-DPA1.value <= threshold=1.546810269355774
node_158: feature_name=CXorf57	CXorf57.value <= threshold=-0.2012881115078926
Class: COVID-19 Critical	
Rules_175	passed counts:27
node_0: feature_name=LTB	LTB.value > threshold=0.994031548500061
node_5264: feature_name=MT-CO1	MT-CO1.value > threshold=-0.4127991199493408
node_5638: feature_name=OAZ1	OAZ1.value > threshold=-0.2640867084264755
node_6112: feature_name=IFI44L	IFI44L.value <= threshold=2.5893263816833496
node_6113: feature_name=TCL1A	TCL1A.value <= threshold=4.201832294464111
node_6114: feature_name=HLA-DQA2	HLA-DQA2.value > threshold=-0.2270243540406227
node_6542: feature_name=CD79B	CD79B.value <= threshold=3.096232771873474
node_6543: feature_name=NR4A1	NR4A1.value > threshold=1.421015739440918
node_6811: feature_name=SLC7A5	SLC7A5.value <= threshold=1.570515513420105
node_6812: feature_name=XAF1	XAF1.value <= threshold=2.218220591545105
node_6813: feature_name=MEF2C	MEF2C.value <= threshold=-0.35489463806152344
node_6814: feature_name=MIR24-2	MIR24-2.value <= threshold=1.5691230297088623
node_6815: feature_name=HLA-DQB1	HLA-DQB1.value <= threshold=2.6548476219177246
node_6816: feature_name=PELI1	PELI1.value <= threshold=2.055415987968445
node_6817: feature_name=DENND3	DENND3.value <= threshold=-0.35369469225406647
node_6818: feature_name=FTH1	FTH1.value <= threshold=1.0994620621204376
Class: Healthy Control	
Rules_176	passed counts:27
node_0: feature_name=LTB	LTB.value > threshold=0.994031548500061
node_5264: feature_name=MT-CO1	MT-CO1.value > threshold=-0.4127991199493408
node_5638: feature_name=OAZ1	OAZ1.value > threshold=-0.2640867084264755
node_6112: feature_name=IFI44L	IFI44L.value <= threshold=2.5893263816833496
node_6113: feature_name=TCL1A	TCL1A.value <= threshold=4.201832294464111
node_6114: feature_name=HLA-DQA2	HLA-DQA2.value > threshold=-0.2270243540406227

node_6542: feature_name=CD79B	CD79B.value <= threshold=3.096232771873474
node_6543: feature_name=NR4A1	NR4A1.value <= threshold=1.421015739440918
node_6544: feature_name=LTB	LTB.value <= threshold=1.359755277633667
node_6545: feature_name=NFKBID	NFKBID.value <= threshold=1.4294711351394653
node_6546: feature_name=HLA-DRB1	HLA-DRB1.value > threshold=1.0398632287979126
node_6556: feature_name=CD79B	CD79B.value > threshold=-0.2919560521841049
node_6582: feature_name=PELI1	PELI1.value > threshold=-0.6156984269618988
node_6602: feature_name=MT-CO3	MT-CO3.value > threshold=0.46141014993190765
node_6632: feature_name=FAM160B1	FAM160B1.value <= threshold=-0.3204421401023865
node_6633: feature_name=TNFAIP3	TNFAIP3.value <= threshold=0.3588470220565796
Class: Healthy Control	
Rules_177	passed counts:27
node_0: feature_name=LTB	LTB.value > threshold=0.994031548500061
node_5264: feature_name=MT-CO1	MT-CO1.value > threshold=-0.4127991199493408
node_5638: feature_name=OAZ1	OAZ1.value > threshold=-0.2640867084264755
node_6112: feature_name=IFI44L	IFI44L.value <= threshold=2.5893263816833496
node_6113: feature_name=TCL1A	TCL1A.value <= threshold=4.201832294464111
node_6114: feature_name=HLA-DQA2	HLA-DQA2.value > threshold=-0.2270243540406227
node_6542: feature_name=CD79B	CD79B.value <= threshold=3.096232771873474
node_6543: feature_name=NR4A1	NR4A1.value <= threshold=1.421015739440918
node_6544: feature_name=LTB	LTB.value <= threshold=1.359755277633667
node_6545: feature_name=NFKBID	NFKBID.value <= threshold=1.4294711351394653
node_6546: feature_name=HLA-DRB1	HLA-DRB1.value > threshold=1.0398632287979126
node_6556: feature_name=CD79B	CD79B.value > threshold=-0.2919560521841049
node_6582: feature_name=PELI1	PELI1.value > threshold=-0.6156984269618988
node_6602: feature_name=MT-CO3	MT-CO3.value <= threshold=0.46141014993190765
node_6603: feature_name=NFKBIZ	NFKBIZ.value <= threshold=0.6470938920974731
node_6604: feature_name=HSP90B1	HSP90B1.value <= threshold=0.4268011748790741
node_6605: feature_name=FCER2	FCER2.value > threshold=-0.2290492281317711

node_6613: feature_name=HLA-DRB5	HLA-DRB5.value <= threshold=2.3765095472335815
node_6614: feature_name=HLA-DQA2	HLA-DQA2.value > threshold=-0.2047279104590416
node_6616: feature_name=ATF5	ATF5.value <= threshold=2.6682212352752686
Class: Healthy Control	
Rules_178	passed counts:27
node_0: feature_name=LTB	LTB.value <= threshold=0.994031548500061
node_1: feature_name=MTRNR2L2	MTRNR2L2.value <= threshold=2.8784297704696655
node_2: feature_name=TCL1A	TCL1A.value <= threshold=4.798312664031982
node_3: feature_name=MT-CO1	MT-CO1.value > threshold=0.08947191759943962
node_2521: feature_name=LTB	LTB.value > threshold=0.6038667261600494
node_4173: feature_name=OAZ1	OAZ1.value > threshold=0.143283873796463
node_4615: feature_name=NR4A1	NR4A1.value <= threshold=0.6621004343032837
node_4616: feature_name=NFKBID	NFKBID.value <= threshold=3.283741593360901
node_4617: feature_name=HLA-DQA1	HLA-DQA1.value <= threshold=2.3435752391815186
node_4618: feature_name=PELI1	PELI1.value > threshold=1.3271203637123108
node_4680: feature_name=BANK1	BANK1.value > threshold=-0.3607018291950226
node_4682: feature_name=RGCC	RGCC.value <= threshold=0.60355344414711
node_4683: feature_name=SNX29P2	SNX29P2.value <= threshold=4.192138910293579
node_4684: feature_name=XIST	XIST.value <= threshold=2.375336170196533
node_4685: feature_name=TCF4	TCF4.value > threshold=2.2667007446289062
node_4697: feature_name=MEF2A	MEF2A.value > threshold=-0.4261530190706253
node_4707: feature_name=RP11-731F5.2	RP11-731F5.2.value <= threshold=5.673919916152954
node_4708: feature_name=DAPP1	DAPP1.value <= threshold=2.9182801246643066
Class: COVID-19 Critical	
Rules_179	passed counts:27
node_0: feature_name=LTB	LTB.value <= threshold=0.994031548500061
node_1: feature_name=MTRNR2L2	MTRNR2L2.value <= threshold=2.8784297704696655
node_2: feature_name=TCL1A	TCL1A.value <= threshold=4.798312664031982

node_3: feature_name=MT-CO1	MT-CO1.value > threshold=0.08947191759943962
node_2521: feature_name=LTB	LTB.value > threshold=0.6038667261600494
node_4173: feature_name=OAZ1	OAZ1.value <= threshold=0.143283873796463
node_4174: feature_name=PLD4	PLD4.value <= threshold=2.921932101249695
node_4175: feature_name=XIST	XIST.value <= threshold=-0.05067425221204758
node_4176: feature_name=MT-CO1	MT-CO1.value <= threshold=1.345594048500061
node_4177: feature_name=HLA-DQB1	HLA-DQB1.value <= threshold=1.927736520767212
node_4178: feature_name=BACH1	BACH1.value <= threshold=-0.5349778532981873
node_4179: feature_name=CD79B	CD79B.value > threshold=1.6518195271492004
node_4201: feature_name=MT-CO2	MT-CO2.value <= threshold=0.5007440596818924
node_4202: feature_name=PELI1	PELI1.value <= threshold=0.9399837851524353
node_4203: feature_name=STAG3	STAG3.value <= threshold=4.226563453674316
node_4204: feature_name=ISG15	ISG15.value <= threshold=1.6907170414924622
node_4205: feature_name=JARID2	JARID2.value <= threshold=1.9471108317375183
Class: Healthy Control	
Rules_180	passed counts:27
node_0: feature_name=LTB	LTB.value <= threshold=0.994031548500061
node_1: feature_name=MTRNR2L2	MTRNR2L2.value <= threshold=2.8784297704696655
node_2: feature_name=TCL1A	TCL1A.value <= threshold=4.798312664031982
node_3: feature_name=MT-CO1	MT-CO1.value > threshold=0.08947191759943962
node_2521: feature_name=LTB	LTB.value <= threshold=0.6038667261600494
node_2522: feature_name=HLA-DQB1	HLA-DQB1.value <= threshold=2.1053054332733154
node_2523: feature_name=SNX9	SNX9.value > threshold=-0.5346486568450928
node_2907: feature_name=NR4A1	NR4A1.value <= threshold=2.5433837175369263
node_2908: feature_name=XIST	XIST.value <= threshold=0.7664470989257097
node_2909: feature_name=CD79B	CD79B.value <= threshold=2.9326847791671753
node_2910: feature_name=NR4A1	NR4A1.value <= threshold=-0.2875557690858841
node_2911: feature_name=CTD-2035E11.3	CTD-2035E11.3.value <= threshold=5.262048006057739
node_2912: feature_name=VPREB3	VPREB3.value <= threshold=4.937594890594482

node_2913: feature_name=IRAK3	IRAK3.value <= threshold=4.592883586883545
node_2914: feature_name=IGJ	IGJ.value <= threshold=7.093410015106201
node_2915: feature_name=WDR74	WDR74.value <= threshold=4.077916383743286
node_2916: feature_name=MEF2C	MEF2C.value <= threshold=4.374382972717285
node_2917: feature_name=HIST1H2AC	HIST1H2AC.value <= threshold=3.743621349334717
node_2918: feature_name=STMN1	STMN1.value <= threshold=6.266510248184204
node_2919: feature_name=RALGPS2	RALGPS2.value <= threshold=5.72194766998291
node_2920: feature_name=HOPX	HOPX.value <= threshold=2.5944807529449463
node_2921: feature_name=MARCKS	MARCKS.value <= threshold=4.622651815414429
node_2922: feature_name=CCL3	CCL3.value <= threshold=0.7484713792800903
node_2923: feature_name=TPM1	TPM1.value <= threshold=6.446193695068359
node_2924: feature_name=PLD4	PLD4.value <= threshold=8.038907289505005
node_2925: feature_name=COTL1	COTL1.value > threshold=-1.8123814463615417
node_2929: feature_name=ID3	ID3.value <= threshold=7.534881353378296
node_2930: feature_name=IRF8	IRF8.value <= threshold=4.855781078338623
node_2931: feature_name=AP3M2	AP3M2.value <= threshold=1.8868122100830078
node_2932: feature_name=FCER2	FCER2.value <= threshold=6.877448558807373
node_2933: feature_name=MTRNR2L2	MTRNR2L2.value <= threshold=2.710945725440979
node_2934: feature_name=GNG11	GNG11.value <= threshold=2.510925054550171
node_2935: feature_name=CD83	CD83.value <= threshold=1.9873459935188293
node_2936: feature_name=MARCH1	MARCH1.value <= threshold=5.006200551986694
node_2937: feature_name=MEF2C	MEF2C.value > threshold=-0.5498070418834686
node_2941: feature_name=FCRL1	FCRL1.value <= threshold=7.093972444534302
node_2942: feature_name=HSP90B1	HSP90B1.value > threshold=-1.4724047780036926
node_2946: feature_name=HLA-DQA1	HLA-DQA1.value > threshold=-0.45666155219078064
node_2950: feature_name=RELT	RELT.value > threshold=1.5976553559303284
node_2952: feature_name=RELT	RELT.value > threshold=1.6147183775901794
Class: COVID-19 Critical	
Rules_181	passed counts:27

node_0: feature_name=LTB	LTB.value <= threshold=0.994031548500061
node_1: feature_name=MTRNR2L2	MTRNR2L2.value <= threshold=2.8784297704696655
node_2: feature_name=TCL1A	TCL1A.value <= threshold=4.798312664031982
node_3: feature_name=MT-CO1	MT-CO1.value <= threshold=0.08947191759943962
node_4: feature_name=NR4A1	NR4A1.value <= threshold=1.0725348591804504
node_5: feature_name=HLA-DPA1	HLA-DPA1.value <= threshold=1.6885469555854797
node_6: feature_name=HLA-DRB5	HLA-DRB5.value <= threshold=2.1886194944381714
node_7: feature_name=XIST	XIST.value <= threshold=-0.12328458577394485
node_8: feature_name=SYNE1	SYNE1.value > threshold=-0.7835594415664673
node_16: feature_name=MTRNR2L2	MTRNR2L2.value <= threshold=2.4483247995376587
node_17: feature_name=HLA-DQA2	HLA-DQA2.value > threshold=2.227178931236267
node_591: feature_name=BCL2A1	BCL2A1.value > threshold=1.0648428201675415
node_687: feature_name=CD79A	CD79A.value > threshold=2.49657940864563
node_703: feature_name=SNX9	SNX9.value > threshold=-0.5558206737041473
node_705: feature_name=ACSL1	ACSL1.value <= threshold=3.0947455167770386
Class: COVID-19 Critical	
Rules_182	passed counts:26
node_0: feature_name=LTB	LTB.value > threshold=0.994031548500061
node_5264: feature_name=MT-CO1	MT-CO1.value > threshold=-0.4127991199493408
node_5638: feature_name=OAZ1	OAZ1.value > threshold=-0.2640867084264755
node_6112: feature_name=IFI44L	IFI44L.value <= threshold=2.5893263816833496
node_6113: feature_name=TCL1A	TCL1A.value <= threshold=4.201832294464111
node_6114: feature_name=HLA-DQA2	HLA-DQA2.value > threshold=-0.2270243540406227
node_6542: feature_name=CD79B	CD79B.value > threshold=3.096232771873474
node_6872: feature_name=ICAM1	ICAM1.value <= threshold=-0.2232399433851242
node_6873: feature_name=STAG3	STAG3.value > threshold=-0.19312061369419098
node_6939: feature_name=HLA-DRB5	HLA-DRB5.value > threshold=2.209904909133911
node_6967: feature_name=TSPYL2	TSPYL2.value > threshold=-0.6224541068077087
Class: COVID-19 Critical	

Rules_183	passed counts:26
node_0: feature_name=LTB	LTB.value > threshold=0.994031548500061
node_5264: feature_name=MT-CO1	MT-CO1.value > threshold=-0.4127991199493408
node_5638: feature_name=OAZ1	OAZ1.value > threshold=-0.2640867084264755
node_6112: feature_name=IFI44L	IFI44L.value <= threshold=2.5893263816833496
node_6113: feature_name=TCL1A	TCL1A.value <= threshold=4.201832294464111
node_6114: feature_name=HLA-DQA2	HLA-DQA2.value > threshold=-0.2270243540406227
node_6542: feature_name=CD79B	CD79B.value > threshold=3.096232771873474
node_6872: feature_name=ICAM1	ICAM1.value <= threshold=-0.2232399433851242
node_6873: feature_name=STAG3	STAG3.value <= threshold=-0.19312061369419098
node_6874: feature_name=CD79A	CD79A.value > threshold=1.57558012008667
node_6880: feature_name=MEF2A	MEF2A.value <= threshold=2.676826596260071
node_6881: feature_name=MTRNR2L2	MTRNR2L2.value <= threshold=2.9562686681747437
node_6882: feature_name=SNX9	SNX9.value <= threshold=3.162803888320923
node_6883: feature_name=MARCKS	MARCKS.value <= threshold=4.3125433921813965
node_6884: feature_name=MX1	MX1.value <= threshold=2.1828908920288086
node_6885: feature_name=ATF5	ATF5.value <= threshold=3.838430166244507
node_6886: feature_name=PELI1	PELI1.value > threshold=1.8930315971374512
node_6910: feature_name=RHOB	RHOB.value > threshold=-0.40518850088119507
node_6914: feature_name=RBKS	RBKS.value <= threshold=5.056721448898315
node_6915: feature_name=MT-CO3	MT-CO3.value > threshold=-0.9622466862201691
Class: Healthy Control	
Rules_184	passed counts:26
node_0: feature_name=LTB	LTB.value > threshold=0.994031548500061
node_5264: feature_name=MT-CO1	MT-CO1.value <= threshold=-0.4127991199493408
node_5265: feature_name=MTRNR2L2	MTRNR2L2.value <= threshold=2.4473838806152344
node_5266: feature_name=TCL1A	TCL1A.value <= threshold=5.787256956100464
node_5267: feature_name=PLD4	PLD4.value > threshold=3.544657826423645

node_5577: feature_name=MT-CO1	MT-CO1.value <= threshold=-0.6592064797878265
node_5578: feature_name=HOPX	HOPX.value <= threshold=-0.47573523223400116
node_5579: feature_name=MARCKS	MARCKS.value <= threshold=2.597380518913269
node_5580: feature_name=NR4A3	NR4A3.value <= threshold=3.236576199531555
Class: COVID-19 Critical	
Rules_185	passed counts:25
node_0: feature_name=LTB	LTB.value > threshold=0.994031548500061
node_5264: feature_name=MT-CO1	MT-CO1.value <= threshold=-0.4127991199493408
node_5265: feature_name=MTRNR2L2	MTRNR2L2.value <= threshold=2.4473838806152344
node_5266: feature_name=TCL1A	TCL1A.value <= threshold=5.787256956100464
node_5267: feature_name=PLD4	PLD4.value <= threshold=3.544657826423645
node_5268: feature_name=NR4A1	NR4A1.value <= threshold=0.8490704298019409
node_5269: feature_name=LTB	LTB.value <= threshold=1.5252206921577454
node_5270: feature_name=HLA-DRB5	HLA-DRB5.value <= threshold=2.5117164850234985
node_5271: feature_name=CD79A	CD79A.value <= threshold=3.685201406478882
node_5272: feature_name=BCL2A1	BCL2A1.value <= threshold=2.081526756286621
node_5273: feature_name=HLA-DPA1	HLA-DPA1.value <= threshold=1.8042166829109192
node_5274: feature_name=HSP90B1	HSP90B1.value > threshold=-1.3115270137786865
node_5278: feature_name=OAZ1	OAZ1.value > threshold=1.0802008509635925
node_5350: feature_name=XAF1	XAF1.value > threshold=-0.4393231123685837
node_5358: feature_name=NR4A3	NR4A3.value <= threshold=0.7696607857942581
Class: COVID-19 Critical	
Rules_186	passed counts:25
node_0: feature_name=LTB	LTB.value <= threshold=0.994031548500061
node_1: feature_name=MTRNR2L2	MTRNR2L2.value <= threshold=2.8784297704696655
node_2: feature_name=TCL1A	TCL1A.value <= threshold=4.798312664031982
node_3: feature_name=MT-CO1	MT-CO1.value > threshold=0.08947191759943962
node_2521: feature_name=LTB	LTB.value > threshold=0.6038667261600494

node_4173: feature_name=OAZ1	OAZ1.value > threshold=0.143283873796463
node_4615: feature_name=NR4A1	NR4A1.value > threshold=0.6621004343032837
node_4817: feature_name=HSP90B1	HSP90B1.value <= threshold=1.1699737310409546
node_4818: feature_name=XAF1	XAF1.value <= threshold=2.6752071380615234
node_4819: feature_name=SNX9	SNX9.value <= threshold=2.939147114753723
node_4820: feature_name=HLA-DQA2	HLA-DQA2.value <= threshold=2.9822239875793457
node_4821: feature_name=ATF5	ATF5.value <= threshold=4.116653323173523
node_4822: feature_name=RALGPS2	RALGPS2.value <= threshold=-0.3627814054489136
node_4823: feature_name=HIST1H2AC	HIST1H2AC.value <= threshold=-0.2637694925069809
node_4824: feature_name=HLA-DRB5	HLA-DRB5.value > threshold=0.4284958690404892
node_4828: feature_name=DAPP1	DAPP1.value <= threshold=3.946416974067688
Class: Healthy Control	
Rules_187	passed counts:25
node_0: feature_name=LTB	LTB.value <= threshold=0.994031548500061
node_1: feature_name=MTRNR2L2	MTRNR2L2.value <= threshold=2.8784297704696655
node_2: feature_name=TCL1A	TCL1A.value <= threshold=4.798312664031982
node_3: feature_name=MT-CO1	MT-CO1.value > threshold=0.08947191759943962
node_2521: feature_name=LTB	LTB.value > threshold=0.6038667261600494
node_4173: feature_name=OAZ1	OAZ1.value > threshold=0.143283873796463
node_4615: feature_name=NR4A1	NR4A1.value <= threshold=0.6621004343032837
node_4616: feature_name=NFKBID	NFKBID.value <= threshold=3.283741593360901
node_4617: feature_name=HLA-DQA1	HLA-DQA1.value > threshold=2.3435752391815186
node_4719: feature_name=MX1	MX1.value > threshold=1.851993978023529
node_4795: feature_name=FCRL1	FCRL1.value <= threshold=5.9853057861328125
node_4796: feature_name=COTL1	COTL1.value > threshold=-1.1470651030540466
node_4798: feature_name=S100A6	S100A6.value <= threshold=0.44754520058631897
Class: COVID-19 Critical	
Rules_188	passed counts:25

node_0: feature_name=LTB	LTB.value <= threshold=0.994031548500061
node_1: feature_name=MTRNR2L2	MTRNR2L2.value <= threshold=2.8784297704696655
node_2: feature_name=TCL1A	TCL1A.value <= threshold=4.798312664031982
node_3: feature_name=MT-CO1	MT-CO1.value > threshold=0.08947191759943962
node_2521: feature_name=LTB	LTB.value > threshold=0.6038667261600494
node_4173: feature_name=OAZ1	OAZ1.value > threshold=0.143283873796463
node_4615: feature_name=NR4A1	NR4A1.value <= threshold=0.6621004343032837
node_4616: feature_name=NFKBID	NFKBID.value <= threshold=3.283741593360901
node_4617: feature_name=HLA-DQA1	HLA-DQA1.value > threshold=2.3435752391815186
node_4719: feature_name=MX1	MX1.value <= threshold=1.851993978023529
node_4720: feature_name=JUN	JUN.value <= threshold=-0.5025404393672943
node_4721: feature_name=RELT	RELT.value <= threshold=-0.28893138468265533
node_4722: feature_name=SNX29P2	SNX29P2.value <= threshold=1.9070574045181274
node_4723: feature_name=APP	APP.value <= threshold=1.4650495648384094
node_4724: feature_name=CYBB	CYBB.value <= threshold=2.0514156818389893
node_4725: feature_name=BCL2A1	BCL2A1.value <= threshold=1.422773003578186
Class: COVID-19 Critical	
Rules_189	passed counts:25
node_0: feature_name=LTB	LTB.value <= threshold=0.994031548500061
node_1: feature_name=MTRNR2L2	MTRNR2L2.value <= threshold=2.8784297704696655
node_2: feature_name=TCL1A	TCL1A.value <= threshold=4.798312664031982
node_3: feature_name=MT-CO1	MT-CO1.value <= threshold=0.08947191759943962
node_4: feature_name=NR4A1	NR4A1.value <= threshold=1.0725348591804504
node_5: feature_name=HLA-DPA1	HLA-DPA1.value > threshold=1.6885469555854797
node_861: feature_name=CD83	CD83.value <= threshold=3.0563642978668213
node_862: feature_name=HLA-DQB1	HLA-DQB1.value > threshold=2.61081063747406
node_1358: feature_name=HLA-DRB5	HLA-DRB5.value > threshold=0.7542744874954224
node_1390: feature_name=HLA-DRB5	HLA-DRB5.value <= threshold=2.6244447231292725
node_1391: feature_name=XIST	XIST.value <= threshold=-0.22017984092235565

node_1392: feature_name=MT-CO3	MT-CO3.value <= threshold=0.12647542357444763
node_1393: feature_name=CD79B	CD79B.value <= threshold=4.338301658630371
node_1394: feature_name=LTB	LTB.value <= threshold=0.9865012466907501
node_1395: feature_name=MEF2C	MEF2C.value <= threshold=4.133931398391724
node_1396: feature_name=MEF2C	MEF2C.value <= threshold=-0.38076959550380707
node_1397: feature_name=UTY	UTY.value > threshold=-0.4634757786989212
Class: COVID-19 Critical	
Rules_190	passed counts:25
node_0: feature_name=LTB	LTB.value <= threshold=0.994031548500061
node_1: feature_name=MTRNR2L2	MTRNR2L2.value <= threshold=2.8784297704696655
node_2: feature_name=TCL1A	TCL1A.value <= threshold=4.798312664031982
node_3: feature_name=MT-CO1	MT-CO1.value <= threshold=0.08947191759943962
node_4: feature_name=NR4A1	NR4A1.value <= threshold=1.0725348591804504
node_5: feature_name=HLA-DPA1	HLA-DPA1.value > threshold=1.6885469555854797
node_861: feature_name=CD83	CD83.value <= threshold=3.0563642978668213
node_862: feature_name=HLA-DQB1	HLA-DQB1.value <= threshold=2.61081063747406
node_863: feature_name=MTRNR2L2	MTRNR2L2.value <= threshold=2.0919997692108154
node_864: feature_name=BCL2A1	BCL2A1.value <= threshold=1.852618932723999
node_865: feature_name=CD79B	CD79B.value > threshold=4.352142333984375
node_1309: feature_name=COTL1	COTL1.value <= threshold=0.48328976333141327
node_1310: feature_name=MARCH1	MARCH1.value > threshold=-0.3511400669813156
node_1314: feature_name=ID3	ID3.value <= threshold=1.150353416800499
node_1315: feature_name=NR4A3	NR4A3.value <= threshold=0.853674978017807
Class: COVID-19 Critical	
Rules_191	passed counts:25
node_0: feature_name=LTB	LTB.value <= threshold=0.994031548500061
node_1: feature_name=MTRNR2L2	MTRNR2L2.value <= threshold=2.8784297704696655
node_2: feature_name=TCL1A	TCL1A.value <= threshold=4.798312664031982

node_3: feature_name=MT-CO1	MT-CO1.value <= threshold=0.08947191759943962
node_4: feature_name=NR4A1	NR4A1.value <= threshold=1.0725348591804504
node_5: feature_name=HLA-DPA1	HLA-DPA1.value > threshold=1.6885469555854797
node_861: feature_name=CD83	CD83.value <= threshold=3.0563642978668213
node_862: feature_name=HLA-DQB1	HLA-DQB1.value <= threshold=2.61081063747406
node_863: feature_name=MTRNR2L2	MTRNR2L2.value <= threshold=2.0919997692108154
node_864: feature_name=BCL2A1	BCL2A1.value <= threshold=1.852618932723999
node_865: feature_name=CD79B	CD79B.value <= threshold=4.352142333984375
node_866: feature_name=TNFAIP3	TNFAIP3.value <= threshold=-0.0449496041983366
node_867: feature_name=MT-CO1	MT-CO1.value <= threshold=-0.08531957119703293
node_868: feature_name=MTRNR2L8	MTRNR2L8.value <= threshold=0.8770350813865662
node_869: feature_name=EGR1	EGR1.value > threshold=-0.32270750403404236
node_1015: feature_name=NR4A3	NR4A3.value <= threshold=1.9085431098937988
node_1016: feature_name=ID3	ID3.value <= threshold=2.776647686958313
node_1017: feature_name=XIST	XIST.value <= threshold=2.618046522140503
node_1018: feature_name=CD79B	CD79B.value <= threshold=3.818994164466858
node_1019: feature_name=NFKBID	NFKBID.value <= threshold=3.4191306829452515
node_1020: feature_name=MIAT	MIAT.value <= threshold=3.532225251197815
node_1021: feature_name=STAG3	STAG3.value <= threshold=9.534917831420898
node_1022: feature_name=OASL	OASL.value <= threshold=2.3923760652542114
node_1023: feature_name=HLA-DQB1	HLA-DQB1.value <= threshold=2.572031259536743
node_1024: feature_name=CD79A	CD79A.value > threshold=0.1879405975341797
node_1028: feature_name=NR4A1	NR4A1.value <= threshold=0.7387348711490631
node_1029: feature_name=SLC7A5	SLC7A5.value <= threshold=2.0677570104599
node_1030: feature_name=HLA-DPB1	HLA-DPB1.value > threshold=2.17708420753479
node_1068: feature_name=FAM129C	FAM129C.value <= threshold=1.734559953212738
node_1069: feature_name=ENDOD1	ENDOD1.value > threshold=-0.22715244442224503
Class: COVID-19 Critical	
Rules_192	passed counts:24

node_0: feature_name=LTB	LTB.value > threshold=0.994031548500061
node_5264: feature_name=MT-CO1	MT-CO1.value > threshold=-0.4127991199493408
node_5638: feature_name=OAZ1	OAZ1.value > threshold=-0.2640867084264755
node_6112: feature_name=IFI44L	IFI44L.value <= threshold=2.5893263816833496
node_6113: feature_name=TCL1A	TCL1A.value <= threshold=4.201832294464111
node_6114: feature_name=HLA-DQA2	HLA-DQA2.value <= threshold=-0.2270243540406227
node_6115: feature_name=MTRNR2L2	MTRNR2L2.value <= threshold=2.548917293548584
node_6116: feature_name=ATF5	ATF5.value <= threshold=2.041263461112976
node_6117: feature_name=JUN	JUN.value <= threshold=-0.616259902715683
node_6118: feature_name=NFKBID	NFKBID.value <= threshold=1.5264258980751038
node_6119: feature_name=MT-CO1	MT-CO1.value <= threshold=0.1564532369375229
node_6120: feature_name=HLA-DPA1	HLA-DPA1.value > threshold=1.569844663143158
node_6126: feature_name=CASP8	CASP8.value <= threshold=0.8988403975963593
node_6127: feature_name=IRF8	IRF8.value <= threshold=2.923409581184387
Class: Healthy Control	
Rules_193	passed counts:24
node_0: feature_name=LTB	LTB.value > threshold=0.994031548500061
node_5264: feature_name=MT-CO1	MT-CO1.value <= threshold=-0.4127991199493408
node_5265: feature_name=MTRNR2L2	MTRNR2L2.value <= threshold=2.4473838806152344
node_5266: feature_name=TCL1A	TCL1A.value <= threshold=5.787256956100464
node_5267: feature_name=PLD4	PLD4.value <= threshold=3.544657826423645
node_5268: feature_name=NR4A1	NR4A1.value <= threshold=0.8490704298019409
node_5269: feature_name=LTB	LTB.value <= threshold=1.5252206921577454
node_5270: feature_name=HLA-DRB5	HLA-DRB5.value <= threshold=2.5117164850234985
node_5271: feature_name=CD79A	CD79A.value <= threshold=3.685201406478882
node_5272: feature_name=BCL2A1	BCL2A1.value <= threshold=2.081526756286621
node_5273: feature_name=HLA-DPA1	HLA-DPA1.value <= threshold=1.8042166829109192
node_5274: feature_name=HSP90B1	HSP90B1.value > threshold=-1.3115270137786865
node_5278: feature_name=OAZ1	OAZ1.value <= threshold=1.0802008509635925

node_5279: feature_name=CD79B	CD79B.value <= threshold=5.875581979751587
node_5280: feature_name=CCDC141	CCDC141.value <= threshold=6.439151048660278
node_5281: feature_name=SYTL3	SYTL3.value <= threshold=1.149337351322174
node_5282: feature_name=GPX1	GPX1.value > threshold=-1.0243326425552368
node_5284: feature_name=MMD	MMD.value <= threshold=6.431635856628418
node_5285: feature_name=MARCKS	MARCKS.value <= threshold=4.034798860549927
node_5286: feature_name=XIST	XIST.value <= threshold=1.148451752960682
node_5287: feature_name=CD83	CD83.value > threshold=2.894522190093994
node_5333: feature_name=RNF11	RNF11.value <= threshold=1.2521337270736694
node_5334: feature_name=HLA-DRB5	HLA-DRB5.value <= threshold=2.06169056892395
node_5335: feature_name=HIST1H2AC	HIST1H2AC.value <= threshold=2.748376488685608
Class: COVID-19 Critical	
Rules_194	passed counts:24
node_0: feature_name=LTB	LTB.value <= threshold=0.994031548500061
node_1: feature_name=MTRNR2L2	MTRNR2L2.value <= threshold=2.8784297704696655
node_2: feature_name=TCL1A	TCL1A.value <= threshold=4.798312664031982
node_3: feature_name=MT-CO1	MT-CO1.value > threshold=0.08947191759943962
node_2521: feature_name=LTB	LTB.value > threshold=0.6038667261600494
node_4173: feature_name=OAZ1	OAZ1.value <= threshold=0.143283873796463
node_4174: feature_name=PLD4	PLD4.value <= threshold=2.921932101249695
node_4175: feature_name=XIST	XIST.value <= threshold=-0.05067425221204758
node_4176: feature_name=MT-CO1	MT-CO1.value <= threshold=1.345594048500061
node_4177: feature_name=HLA-DQB1	HLA-DQB1.value > threshold=1.927736520767212
node_4353: feature_name=ITM2C	ITM2C.value > threshold=-0.12329206243157387
node_4459: feature_name=SNX9	SNX9.value > threshold=0.6323382556438446
node_4469: feature_name=PELI1	PELI1.value > threshold=0.4953474849462509
node_4479: feature_name=HLA-DQB1	HLA-DQB1.value > threshold=1.9635218977928162
node_4481: feature_name=CD79B	CD79B.value <= threshold=3.104057788848877
node_4482: feature_name=DUSP4	DUSP4.value <= threshold=2.7126036882400513

Class: COVID-19 Critical	
Rules_195	passed counts:24
node_0: feature_name=LTB	LTB.value <= threshold=0.994031548500061
node_1: feature_name=MTRNR2L2	MTRNR2L2.value <= threshold=2.8784297704696655
node_2: feature_name=TCL1A	TCL1A.value <= threshold=4.798312664031982
node_3: feature_name=MT-CO1	MT-CO1.value > threshold=0.08947191759943962
node_2521: feature_name=LTB	LTB.value <= threshold=0.6038667261600494
node_2522: feature_name=HLA-DQB1	HLA-DQB1.value > threshold=2.1053054332733154
node_3550: feature_name=XIST	XIST.value <= threshold=0.7716337237507105
node_3551: feature_name=CD79B	CD79B.value > threshold=2.9426796436309814
node_4039: feature_name=IFI44L	IFI44L.value > threshold=1.8637521862983704
node_4137: feature_name=OAZ1	OAZ1.value <= threshold=1.3411704897880554
Class: COVID-19 Critical	
Rules_196	passed counts:24
node_0: feature_name=LTB	LTB.value <= threshold=0.994031548500061
node_1: feature_name=MTRNR2L2	MTRNR2L2.value <= threshold=2.8784297704696655
node_2: feature_name=TCL1A	TCL1A.value <= threshold=4.798312664031982
node_3: feature_name=MT-CO1	MT-CO1.value > threshold=0.08947191759943962
node_2521: feature_name=LTB	LTB.value <= threshold=0.6038667261600494
node_2522: feature_name=HLA-DQB1	HLA-DQB1.value <= threshold=2.1053054332733154
node_2523: feature_name=SNX9	SNX9.value > threshold=-0.5346486568450928
node_2907: feature_name=NR4A1	NR4A1.value <= threshold=2.5433837175369263
node_2908: feature_name=XIST	XIST.value <= threshold=0.7664470989257097
node_2909: feature_name=CD79B	CD79B.value > threshold=2.9326847791671753
node_3353: feature_name=VPREB3	VPREB3.value > threshold=3.0226117372512817
node_3425: feature_name=OAZ1	OAZ1.value <= threshold=0.47689591348171234
node_3426: feature_name=HLA-DQA1	HLA-DQA1.value <= threshold=2.271668791770935
node_3427: feature_name=GNG11	GNG11.value <= threshold=1.8491391688585281

node_3428: feature_name=GPX1	GPX1.value <= threshold=0.6236739754676819
node_3429: feature_name=S100A6	S100A6.value > threshold=-1.923483669757843
Class: COVID-19 Critical	
Rules_197	passed counts:24
node_0: feature_name=LTB	LTB.value <= threshold=0.994031548500061
node_1: feature_name=MTRNR2L2	MTRNR2L2.value <= threshold=2.8784297704696655
node_2: feature_name=TCL1A	TCL1A.value <= threshold=4.798312664031982
node_3: feature_name=MT-CO1	MT-CO1.value <= threshold=0.08947191759943962
node_4: feature_name=NR4A1	NR4A1.value <= threshold=1.0725348591804504
node_5: feature_name=HLA-DPA1	HLA-DPA1.value > threshold=1.6885469555854797
node_861: feature_name=CD83	CD83.value <= threshold=3.0563642978668213
node_862: feature_name=HLA-DQB1	HLA-DQB1.value > threshold=2.61081063747406
node_1358: feature_name=HLA-DRB5	HLA-DRB5.value <= threshold=0.7542744874954224
node_1359: feature_name=MT-CYB	MT-CYB.value <= threshold=-0.06432783603668213
node_1360: feature_name=NFKBID	NFKBID.value <= threshold=2.1380220651626587
node_1361: feature_name=FTH1	FTH1.value <= threshold=0.9248916804790497
node_1362: feature_name=VCL	VCL.value <= threshold=2.499361515045166
node_1363: feature_name=FAM160B1	FAM160B1.value <= threshold=1.9471526741981506
Class: COVID-19 Critical	
Rules_198	passed counts:24
node_0: feature_name=LTB	LTB.value <= threshold=0.994031548500061
node_1: feature_name=MTRNR2L2	MTRNR2L2.value <= threshold=2.8784297704696655
node_2: feature_name=TCL1A	TCL1A.value <= threshold=4.798312664031982
node_3: feature_name=MT-CO1	MT-CO1.value <= threshold=0.08947191759943962
node_4: feature_name=NR4A1	NR4A1.value <= threshold=1.0725348591804504
node_5: feature_name=HLA-DPA1	HLA-DPA1.value > threshold=1.6885469555854797
node_861: feature_name=CD83	CD83.value <= threshold=3.0563642978668213
node_862: feature_name=HLA-DQB1	HLA-DQB1.value <= threshold=2.61081063747406

node_863: feature_name=MTRNR2L2	MTRNR2L2.value <= threshold=2.0919997692108154
node_864: feature_name=BCL2A1	BCL2A1.value <= threshold=1.852618932723999
node_865: feature_name=CD79B	CD79B.value <= threshold=4.352142333984375
node_866: feature_name=TNFAIP3	TNFAIP3.value <= threshold=-0.0449496041983366
node_867: feature_name=MT-CO1	MT-CO1.value > threshold=-0.08531957119703293
node_1155: feature_name=IRF8	IRF8.value > threshold=-0.39239931106567383
node_1177: feature_name=PELI1	PELI1.value <= threshold=-0.6234017610549927
node_1178: feature_name=HLA-DRB1	HLA-DRB1.value <= threshold=1.3807418942451477
node_1179: feature_name=MT-CO1	MT-CO1.value <= threshold=0.05383153632283211
Class: COVID-19 Critical	
Rules_199	passed counts:24
node_0: feature_name=LTB	LTB.value <= threshold=0.994031548500061
node_1: feature_name=MTRNR2L2	MTRNR2L2.value <= threshold=2.8784297704696655
node_2: feature_name=TCL1A	TCL1A.value <= threshold=4.798312664031982
node_3: feature_name=MT-CO1	MT-CO1.value <= threshold=0.08947191759943962
node_4: feature_name=NR4A1	NR4A1.value <= threshold=1.0725348591804504
node_5: feature_name=HLA-DPA1	HLA-DPA1.value <= threshold=1.6885469555854797
node_6: feature_name=HLA-DRB5	HLA-DRB5.value <= threshold=2.1886194944381714
node_7: feature_name=XIST	XIST.value <= threshold=-0.12328458577394485
node_8: feature_name=SYNE1	SYNE1.value > threshold=-0.7835594415664673
node_16: feature_name=MTRNR2L2	MTRNR2L2.value <= threshold=2.4483247995376587
node_17: feature_name=HLA-DQA2	HLA-DQA2.value <= threshold=2.227178931236267
node_18: feature_name=NR4A1	NR4A1.value <= threshold=-0.21380723267793655
node_19: feature_name=IRAK3	IRAK3.value <= threshold=4.27159571647644
node_20: feature_name=IFIT2	IFIT2.value <= threshold=7.8175740242004395
node_21: feature_name=CD79A	CD79A.value <= threshold=2.857242226600647
node_22: feature_name=HIST1H2AC	HIST1H2AC.value <= threshold=5.077470302581787
node_23: feature_name=FTH1	FTH1.value <= threshold=0.4158056080341339
node_24: feature_name=ATF3	ATF3.value <= threshold=3.8028353452682495

node_25: feature_name=OASL	OASL.value <= threshold=3.387580633163452
node_26: feature_name=MT-CO1	MT-CO1.value > threshold=-0.7942646145820618
node_74: feature_name=HOPX	HOPX.value <= threshold=0.8682331740856171
node_75: feature_name=RGS2	RGS2.value <= threshold=1.4589954018592834
node_76: feature_name=AHR	AHR.value <= threshold=3.870481848716736
node_77: feature_name=IGJ	IGJ.value <= threshold=4.902882814407349
node_78: feature_name=CD79B	CD79B.value <= threshold=4.87716269493103
node_79: feature_name=HLA-DQB1	HLA-DQB1.value <= threshold=3.6925491094589233
node_80: feature_name=HLA-DRB5	HLA-DRB5.value <= threshold=2.0926843881607056
node_81: feature_name=MCTP1	MCTP1.value <= threshold=4.010472774505615
node_82: feature_name=GNG11	GNG11.value <= threshold=3.627470850944519
node_83: feature_name=HSP90B1	HSP90B1.value <= threshold=2.0835131406784058
node_84: feature_name=ATF3	ATF3.value <= threshold=1.1142374873161316
node_85: feature_name=MT-ND6	MT-ND6.value <= threshold=2.7328014373779297
node_86: feature_name=COTL1	COTL1.value > threshold=-1.6524587869644165
node_90: feature_name=MT-CYB	MT-CYB.value <= threshold=1.099629819393158
node_91: feature_name=RGCC	RGCC.value <= threshold=1.6908138990402222
node_92: feature_name=ILK	ILK.value <= threshold=3.9497616291046143
node_93: feature_name=CTD-2035E11.3	CTD-2035E11.3.value <= threshold=2.4543306827545166
node_94: feature_name=S100A6	S100A6.value > threshold=0.2867250442504883
node_154: feature_name=ILK	ILK.value > threshold=-0.37570807337760925
Class: COVID-19 Critical	
Rules_200	passed counts:23
node_0: feature_name=LTB	LTB.value > threshold=0.994031548500061
node_5264: feature_name=MT-CO1	MT-CO1.value > threshold=-0.4127991199493408
node_5638: feature_name=OAZ1	OAZ1.value > threshold=-0.2640867084264755
node_6112: feature_name=IFI44L	IFI44L.value <= threshold=2.5893263816833496
node_6113: feature_name=TCL1A	TCL1A.value <= threshold=4.201832294464111
node_6114: feature_name=HLA-DQA2	HLA-DQA2.value > threshold=-0.2270243540406227

node_6542: feature_name=CD79B	CD79B.value <= threshold=3.096232771873474
node_6543: feature_name=NR4A1	NR4A1.value <= threshold=1.421015739440918
node_6544: feature_name=LTB	LTB.value > threshold=1.359755277633667
node_6720: feature_name=NFKBIZ	NFKBIZ.value > threshold=0.2720414251089096
node_6794: feature_name=PELI1	PELI1.value > threshold=1.5861127376556396
node_6806: feature_name=TNF	TNF.value <= threshold=0.9266208410263062
node_6807: feature_name=FCER2	FCER2.value <= threshold=5.030398368835449
Class: COVID-19 Critical	
Rules_201	passed counts:23
node_0: feature_name=LTB	LTB.value > threshold=0.994031548500061
node_5264: feature_name=MT-CO1	MT-CO1.value > threshold=-0.4127991199493408
node_5638: feature_name=OAZ1	OAZ1.value > threshold=-0.2640867084264755
node_6112: feature_name=IFI44L	IFI44L.value <= threshold=2.5893263816833496
node_6113: feature_name=TCL1A	TCL1A.value <= threshold=4.201832294464111
node_6114: feature_name=HLA-DQA2	HLA-DQA2.value <= threshold=-0.2270243540406227
node_6115: feature_name=MTRNR2L2	MTRNR2L2.value <= threshold=2.548917293548584
node_6116: feature_name=ATF5	ATF5.value <= threshold=2.041263461112976
node_6117: feature_name=JUN	JUN.value <= threshold=-0.616259902715683
node_6118: feature_name=NFKBID	NFKBID.value <= threshold=1.5264258980751038
node_6119: feature_name=MT-CO1	MT-CO1.value > threshold=0.1564532369375229
node_6133: feature_name=LTB	LTB.value <= threshold=1.2431999444961548
node_6134: feature_name=MT-ND6	MT-ND6.value > threshold=0.5990295112133026
node_6144: feature_name=HSP90B1	HSP90B1.value <= threshold=1.085684895515442
node_6145: feature_name=COTL1	COTL1.value <= threshold=0.9165144860744476
Class: Healthy Control	
Rules_202	passed counts:23
node_0: feature_name=LTB	LTB.value > threshold=0.994031548500061
node_5264: feature_name=MT-CO1	MT-CO1.value > threshold=-0.4127991199493408

node_5638: feature_name=OAZ1	OAZ1.value <= threshold=-0.2640867084264755
node_5639: feature_name=CD79B	CD79B.value <= threshold=2.2998517751693726
node_5640: feature_name=MTRNR2L2	MTRNR2L2.value <= threshold=3.5499863624572754
node_5641: feature_name=MT-CO1	MT-CO1.value > threshold=0.22567133605480194
node_5729: feature_name=MARCH1	MARCH1.value > threshold=1.5154643654823303
node_5867: feature_name=NFKBID	NFKBID.value > threshold=2.6947624683380127
node_5903: feature_name=RGCC	RGCC.value <= threshold=-0.730547159910202
node_5904: feature_name=BOD1L1	BOD1L1.value <= threshold=2.7324291467666626
node_5905: feature_name=BANK1	BANK1.value > threshold=-0.37174978852272034
Class: COVID-19 Critical	
Rules_203	passed counts:23
node_0: feature_name=LTB	LTB.value > threshold=0.994031548500061
node_5264: feature_name=MT-CO1	MT-CO1.value <= threshold=-0.4127991199493408
node_5265: feature_name=MTRNR2L2	MTRNR2L2.value <= threshold=2.4473838806152344
node_5266: feature_name=TCL1A	TCL1A.value <= threshold=5.787256956100464
node_5267: feature_name=PLD4	PLD4.value <= threshold=3.544657826423645
node_5268: feature_name=NR4A1	NR4A1.value <= threshold=0.8490704298019409
node_5269: feature_name=LTB	LTB.value <= threshold=1.5252206921577454
node_5270: feature_name=HLA-DRB5	HLA-DRB5.value > threshold=2.5117164850234985
node_5448: feature_name=HLA-DQB1	HLA-DQB1.value <= threshold=2.092399835586548
node_5449: feature_name=APP	APP.value <= threshold=0.8784182295203209
node_5450: feature_name=VPREB3	VPREB3.value <= threshold=6.6706461906433105
Class: COVID-19 Critical	
Rules_204	passed counts:23
node_0: feature_name=LTB	LTB.value <= threshold=0.994031548500061
node_1: feature_name=MTRNR2L2	MTRNR2L2.value <= threshold=2.8784297704696655
node_2: feature_name=TCL1A	TCL1A.value <= threshold=4.798312664031982
node_3: feature_name=MT-CO1	MT-CO1.value > threshold=0.08947191759943962

node_2521: feature_name=LTB	LTB.value > threshold=0.6038667261600494
node_4173: feature_name=OAZ1	OAZ1.value <= threshold=0.143283873796463
node_4174: feature_name=PLD4	PLD4.value <= threshold=2.921932101249695
node_4175: feature_name=XIST	XIST.value <= threshold=-0.05067425221204758
node_4176: feature_name=MT-CO1	MT-CO1.value <= threshold=1.345594048500061
node_4177: feature_name=HLA-DQB1	HLA-DQB1.value <= threshold=1.927736520767212
node_4178: feature_name=BACH1	BACH1.value > threshold=-0.5349778532981873
node_4236: feature_name=TPM1	TPM1.value <= threshold=3.516477584838867
node_4237: feature_name=NR4A1	NR4A1.value > threshold=-0.274935707449913
node_4277: feature_name=HLA-DPA1	HLA-DPA1.value <= threshold=1.8894080519676208
node_4278: feature_name=TRAF1	TRAF1.value <= threshold=2.091539978981018
node_4279: feature_name=STMN1	STMN1.value <= threshold=2.2198781967163086
node_4280: feature_name=PLD4	PLD4.value <= threshold=2.7647364139556885
node_4281: feature_name=IGJ	IGJ.value <= threshold=4.225788354873657
node_4282: feature_name=MEF2C	MEF2C.value <= threshold=3.573865532875061
node_4283: feature_name=ILK	ILK.value <= threshold=3.675175428390503
node_4284: feature_name=MT-CO3	MT-CO3.value > threshold=-0.7448426485061646
node_4288: feature_name=TCF4	TCF4.value > threshold=3.9125311374664307
node_4310: feature_name=BOD1L1	BOD1L1.value > threshold=-0.4781903773546219
node_4314: feature_name=SLC7A5	SLC7A5.value <= threshold=2.6468170881271362
node_4315: feature_name=APP	APP.value <= threshold=1.8160242438316345
node_4316: feature_name=RNF11	RNF11.value <= threshold=1.6061962246894836
Class: COVID-19 Critical	
Rules_205	passed counts:23
node_0: feature_name=LTB	LTB.value <= threshold=0.994031548500061
node_1: feature_name=MTRNR2L2	MTRNR2L2.value <= threshold=2.8784297704696655
node_2: feature_name=TCL1A	TCL1A.value <= threshold=4.798312664031982
node_3: feature_name=MT-CO1	MT-CO1.value > threshold=0.08947191759943962
node_2521: feature_name=LTB	LTB.value <= threshold=0.6038667261600494

node_2522: feature_name=HLA-DQB1	HLA-DQB1.value > threshold=2.1053054332733154
node_3550: feature_name=XIST	XIST.value <= threshold=0.7716337237507105
node_3551: feature_name=CD79B	CD79B.value > threshold=2.9426796436309814
node_4039: feature_name=IFI44L	IFI44L.value <= threshold=1.8637521862983704
node_4040: feature_name=SNX9	SNX9.value > threshold=1.274232804775238
node_4096: feature_name=RALGPS2	RALGPS2.value <= threshold=2.3599878549575806
node_4097: feature_name=HLA-DQB1	HLA-DQB1.value <= threshold=2.9625227451324463
node_4098: feature_name=CD79A	CD79A.value > threshold=2.383400559425354
node_4108: feature_name=MX1	MX1.value <= threshold=1.535942792892456
Class: COVID-19 Critical	
Rules_206	passed counts:23
node_0: feature_name=LTB	LTB.value <= threshold=0.994031548500061
node_1: feature_name=MTRNR2L2	MTRNR2L2.value <= threshold=2.8784297704696655
node_2: feature_name=TCL1A	TCL1A.value <= threshold=4.798312664031982
node_3: feature_name=MT-CO1	MT-CO1.value <= threshold=0.08947191759943962
node_4: feature_name=NR4A1	NR4A1.value <= threshold=1.0725348591804504
node_5: feature_name=HLA-DPA1	HLA-DPA1.value > threshold=1.6885469555854797
node_861: feature_name=CD83	CD83.value > threshold=3.0563642978668213
node_1539: feature_name=ID1	ID1.value > threshold=1.4626862406730652
node_1615: feature_name=CD79B	CD79B.value <= threshold=2.151446223258972
node_1616: feature_name=TNFAIP3	TNFAIP3.value <= threshold=0.7088594734668732
Class: COVID-19 Severe	
Rules_207	passed counts:23
node_0: feature_name=LTB	LTB.value <= threshold=0.994031548500061
node_1: feature_name=MTRNR2L2	MTRNR2L2.value <= threshold=2.8784297704696655
node_2: feature_name=TCL1A	TCL1A.value <= threshold=4.798312664031982
node_3: feature_name=MT-CO1	MT-CO1.value <= threshold=0.08947191759943962
node_4: feature_name=NR4A1	NR4A1.value <= threshold=1.0725348591804504

node_5: feature_name=HLA-DPA1	HLA-DPA1.value > threshold=1.6885469555854797
node_861: feature_name=CD83	CD83.value > threshold=3.0563642978668213
node_1539: feature_name=ID1	ID1.value <= threshold=1.4626862406730652
node_1540: feature_name=FTH1	FTH1.value > threshold=0.2985198348760605
node_1590: feature_name=RP5-821D11.7	RP5-821D11.7.value <= threshold=-0.2580558806657791
node_1591: feature_name=HSP90B1	HSP90B1.value <= threshold=1.1008275151252747
node_1592: feature_name=S100A6	S100A6.value <= threshold=-0.45454199612140656
node_1593: feature_name=HLA-DPA1	HLA-DPA1.value > threshold=1.7763205170631409
node_1595: feature_name=ENDOD1	ENDOD1.value <= threshold=1.9235998392105103
Class: COVID-19 Critical	
Rules_208	passed counts:23
node_0: feature_name=LTB	LTB.value <= threshold=0.994031548500061
node_1: feature_name=MTRNR2L2	MTRNR2L2.value <= threshold=2.8784297704696655
node_2: feature_name=TCL1A	TCL1A.value <= threshold=4.798312664031982
node_3: feature_name=MT-CO1	MT-CO1.value <= threshold=0.08947191759943962
node_4: feature_name=NR4A1	NR4A1.value <= threshold=1.0725348591804504
node_5: feature_name=HLA-DPA1	HLA-DPA1.value > threshold=1.6885469555854797
node_861: feature_name=CD83	CD83.value <= threshold=3.0563642978668213
node_862: feature_name=HLA-DQB1	HLA-DQB1.value > threshold=2.61081063747406
node_1358: feature_name=HLA-DRB5	HLA-DRB5.value > threshold=0.7542744874954224
node_1390: feature_name=HLA-DRB5	HLA-DRB5.value <= threshold=2.6244447231292725
node_1391: feature_name=XIST	XIST.value > threshold=-0.22017984092235565
node_1497: feature_name=HLA-DRB5	HLA-DRB5.value > threshold=1.2674232721328735
node_1503: feature_name=HLA-DQA1	HLA-DQA1.value > threshold=2.4817473888397217
node_1509: feature_name=MT-CO2	MT-CO2.value <= threshold=0.5640499591827393
node_1510: feature_name=MS4A1	MS4A1.value > threshold=2.00724059343338
Class: COVID-19 Critical	
Rules_209	passed counts:23

node_0: feature_name=LTB	LTB.value <= threshold=0.994031548500061
node_1: feature_name=MTRNR2L2	MTRNR2L2.value <= threshold=2.8784297704696655
node_2: feature_name=TCL1A	TCL1A.value <= threshold=4.798312664031982
node_3: feature_name=MT-CO1	MT-CO1.value <= threshold=0.08947191759943962
node_4: feature_name=NR4A1	NR4A1.value <= threshold=1.0725348591804504
node_5: feature_name=HLA-DPA1	HLA-DPA1.value > threshold=1.6885469555854797
node_861: feature_name=CD83	CD83.value <= threshold=3.0563642978668213
node_862: feature_name=HLA-DQB1	HLA-DQB1.value <= threshold=2.61081063747406
node_863: feature_name=MTRNR2L2	MTRNR2L2.value <= threshold=2.0919997692108154
node_864: feature_name=BCL2A1	BCL2A1.value <= threshold=1.852618932723999
node_865: feature_name=CD79B	CD79B.value <= threshold=4.352142333984375
node_866: feature_name=TNFAIP3	TNFAIP3.value <= threshold=-0.0449496041983366
node_867: feature_name=MT-CO1	MT-CO1.value > threshold=-0.08531957119703293
node_1155: feature_name=IRF8	IRF8.value > threshold=-0.39239931106567383
node_1177: feature_name=PELI1	PELI1.value <= threshold=-0.6234017610549927
node_1178: feature_name=HLA-DRB1	HLA-DRB1.value > threshold=1.3807418942451477
node_1182: feature_name=ANO6	ANO6.value <= threshold=-0.3352542072534561
node_1183: feature_name=MS4A1	MS4A1.value > threshold=1.8863030076026917
node_1187: feature_name=MT-CO2	MT-CO2.value <= threshold=0.32131922245025635
node_1188: feature_name=BANK1	BANK1.value <= threshold=3.6148797273635864
node_1189: feature_name=NFKBID	NFKBID.value <= threshold=3.1294965744018555
node_1190: feature_name=ICAM1	ICAM1.value <= threshold=1.0546877682209015
node_1191: feature_name=GRASP	GRASP.value > threshold=-0.3996799439191818
Class: Healthy Control	
Rules_210	passed counts:22
node_0: feature_name=LTB	LTB.value > threshold=0.994031548500061
node_5264: feature_name=MT-CO1	MT-CO1.value > threshold=-0.4127991199493408
node_5638: feature_name=OAZ1	OAZ1.value > threshold=-0.2640867084264755
node_6112: feature_name=IFI44L	IFI44L.value <= threshold=2.5893263816833496

node_6113: feature_name=TCL1A	TCL1A.value <= threshold=4.201832294464111
node_6114: feature_name=HLA-DQA2	HLA-DQA2.value > threshold=-0.2270243540406227
node_6542: feature_name=CD79B	CD79B.value > threshold=3.096232771873474
node_6872: feature_name=ICAM1	ICAM1.value > threshold=-0.2232399433851242
node_6974: feature_name=HSP90B1	HSP90B1.value > threshold=-0.9936705231666565
node_6980: feature_name=TCL1A	TCL1A.value <= threshold=-0.17627128213644028
node_6981: feature_name=MT-CO2	MT-CO2.value > threshold=-0.5288586318492889
node_6985: feature_name=MS4A1	MS4A1.value <= threshold=4.967900037765503
Class: COVID-19 Critical	
Rules_211	passed counts:22
node_0: feature_name=LTB	LTB.value <= threshold=0.994031548500061
node_1: feature_name=MTRNR2L2	MTRNR2L2.value <= threshold=2.8784297704696655
node_2: feature_name=TCL1A	TCL1A.value <= threshold=4.798312664031982
node_3: feature_name=MT-CO1	MT-CO1.value > threshold=0.08947191759943962
node_2521: feature_name=LTB	LTB.value > threshold=0.6038667261600494
node_4173: feature_name=OAZ1	OAZ1.value <= threshold=0.143283873796463
node_4174: feature_name=PLD4	PLD4.value > threshold=2.921932101249695
node_4558: feature_name=HLA-DQA2	HLA-DQA2.value <= threshold=1.5485953092575073
node_4559: feature_name=COTL1	COTL1.value <= threshold=-0.42844848334789276
node_4560: feature_name=VPREB3	VPREB3.value <= threshold=0.7973381504416466
node_4561: feature_name=ZNF10	ZNF10.value <= threshold=3.9046400785446167
node_4562: feature_name=NAMPT	NAMPT.value <= threshold=0.5289976298809052
Class: COVID-19 Critical	
Rules_212	passed counts:22
node_0: feature_name=LTB	LTB.value <= threshold=0.994031548500061
node_1: feature_name=MTRNR2L2	MTRNR2L2.value <= threshold=2.8784297704696655
node_2: feature_name=TCL1A	TCL1A.value <= threshold=4.798312664031982
node_3: feature_name=MT-CO1	MT-CO1.value > threshold=0.08947191759943962

node_2521: feature_name=LTB	LTB.value <= threshold=0.6038667261600494
node_2522: feature_name=HLA-DQB1	HLA-DQB1.value > threshold=2.1053054332733154
node_3550: feature_name=XIST	XIST.value <= threshold=0.7716337237507105
node_3551: feature_name=CD79B	CD79B.value > threshold=2.9426796436309814
node_4039: feature_name=IFI44L	IFI44L.value <= threshold=1.8637521862983704
node_4040: feature_name=SNX9	SNX9.value <= threshold=1.274232804775238
node_4041: feature_name=CASP8	CASP8.value > threshold=-0.574097603559494
node_4073: feature_name=HLA-DRB5	HLA-DRB5.value <= threshold=1.4346495270729065
node_4074: feature_name=STMN1	STMN1.value <= threshold=-0.21146973967552185
node_4075: feature_name=GRASP	GRASP.value <= threshold=1.8942881226539612
node_4076: feature_name=TLN1	TLN1.value > threshold=-0.76496422290802
Class: COVID-19 Critical	
Rules_213	passed counts:22
node_0: feature_name=LTB	LTB.value <= threshold=0.994031548500061
node_1: feature_name=MTRNR2L2	MTRNR2L2.value <= threshold=2.8784297704696655
node_2: feature_name=TCL1A	TCL1A.value <= threshold=4.798312664031982
node_3: feature_name=MT-CO1	MT-CO1.value > threshold=0.08947191759943962
node_2521: feature_name=LTB	LTB.value <= threshold=0.6038667261600494
node_2522: feature_name=HLA-DQB1	HLA-DQB1.value > threshold=2.1053054332733154
node_3550: feature_name=XIST	XIST.value <= threshold=0.7716337237507105
node_3551: feature_name=CD79B	CD79B.value <= threshold=2.9426796436309814
node_3552: feature_name=HLA-DQB1	HLA-DQB1.value <= threshold=2.713674306869507
node_3553: feature_name=SNX9	SNX9.value <= threshold=-0.5404067635536194
node_3554: feature_name=VPREB3	VPREB3.value > threshold=4.4475414752960205
node_3646: feature_name=FCRL1	FCRL1.value > threshold=-0.21100489795207977
Class: Healthy Control	
Rules_214	passed counts:22
node_0: feature_name=LTB	LTB.value <= threshold=0.994031548500061

node_1: feature_name=MTRNR2L2	MTRNR2L2.value <= threshold=2.8784297704696655
node_2: feature_name=TCL1A	TCL1A.value <= threshold=4.798312664031982
node_3: feature_name=MT-CO1	MT-CO1.value > threshold=0.08947191759943962
node_2521: feature_name=LTB	LTB.value <= threshold=0.6038667261600494
node_2522: feature_name=HLA-DQB1	HLA-DQB1.value <= threshold=2.1053054332733154
node_2523: feature_name=SNX9	SNX9.value > threshold=-0.5346486568450928
node_2907: feature_name=NR4A1	NR4A1.value > threshold=2.5433837175369263
node_3459: feature_name=VPREB3	VPREB3.value <= threshold=4.410779237747192
node_3460: feature_name=RALGPS2	RALGPS2.value > threshold=3.064661979675293
node_3520: feature_name=RGS2	RGS2.value > threshold=-0.5165578126907349
node_3522: feature_name=MT-ND6	MT-ND6.value <= threshold=1.692318856716156
node_3523: feature_name=ID1	ID1.value <= threshold=-0.16399746388196945
node_3524: feature_name=HBB	HBB.value <= threshold=1.082573562860489
node_3525: feature_name=RBKS	RBKS.value > threshold=-0.2546686381101608
node_3529: feature_name=BOD1L1	BOD1L1.value <= threshold=2.1491535305976868
node_3530: feature_name=MYL9	MYL9.value <= threshold=3.3608904629945755
node_3531: feature_name=PDLIM1	PDLIM1.value <= threshold=4.061182856559753
Class: COVID-19 Critical	
Rules_215	passed counts:22
node_0: feature_name=LTB	LTB.value <= threshold=0.994031548500061
node_1: feature_name=MTRNR2L2	MTRNR2L2.value <= threshold=2.8784297704696655
node_2: feature_name=TCL1A	TCL1A.value <= threshold=4.798312664031982
node_3: feature_name=MT-CO1	MT-CO1.value > threshold=0.08947191759943962
node_2521: feature_name=LTB	LTB.value <= threshold=0.6038667261600494
node_2522: feature_name=HLA-DQB1	HLA-DQB1.value <= threshold=2.1053054332733154
node_2523: feature_name=SNX9	SNX9.value > threshold=-0.5346486568450928
node_2907: feature_name=NR4A1	NR4A1.value <= threshold=2.5433837175369263
node_2908: feature_name=XIST	XIST.value <= threshold=0.7664470989257097
node_2909: feature_name=CD79B	CD79B.value <= threshold=2.9326847791671753

node_2910: feature_name=NR4A1	NR4A1.value <= threshold=-0.2875557690858841
node_2911: feature_name=CTD-2035E11.3	CTD-2035E11.3.value <= threshold=5.262048006057739
node_2912: feature_name=VPREB3	VPREB3.value <= threshold=4.937594890594482
node_2913: feature_name=IRAK3	IRAK3.value <= threshold=4.592883586883545
node_2914: feature_name=IGJ	IGJ.value <= threshold=7.093410015106201
node_2915: feature_name=WDR74	WDR74.value <= threshold=4.077916383743286
node_2916: feature_name=MEF2C	MEF2C.value <= threshold=4.374382972717285
node_2917: feature_name=HIST1H2AC	HIST1H2AC.value <= threshold=3.743621349334717
node_2918: feature_name=STMN1	STMN1.value <= threshold=6.266510248184204
node_2919: feature_name=RALGPS2	RALGPS2.value <= threshold=5.72194766998291
node_2920: feature_name=HOPX	HOPX.value <= threshold=2.5944807529449463
node_2921: feature_name=MARCKS	MARCKS.value <= threshold=4.622651815414429
node_2922: feature_name=CCL3	CCL3.value <= threshold=0.7484713792800903
node_2923: feature_name=TPM1	TPM1.value <= threshold=6.446193695068359
node_2924: feature_name=PLD4	PLD4.value <= threshold=8.038907289505005
node_2925: feature_name=COTL1	COTL1.value > threshold=-1.8123814463615417
node_2929: feature_name=ID3	ID3.value <= threshold=7.534881353378296
node_2930: feature_name=IRF8	IRF8.value <= threshold=4.855781078338623
node_2931: feature_name=AP3M2	AP3M2.value <= threshold=1.8868122100830078
node_2932: feature_name=FCER2	FCER2.value <= threshold=6.877448558807373
node_2933: feature_name=MTRNR2L2	MTRNR2L2.value <= threshold=2.710945725440979
node_2934: feature_name=GNG11	GNG11.value <= threshold=2.510925054550171
node_2935: feature_name=CD83	CD83.value > threshold=1.9873459935188293
node_2961: feature_name=CD83	CD83.value > threshold=2.0049850940704346
node_2965: feature_name=HES4	HES4.value <= threshold=4.076817035675049
node_2966: feature_name=BCL2A1	BCL2A1.value <= threshold=1.1745486855506897
node_2967: feature_name=NT5C3A	NT5C3A.value <= threshold=3.6911903619766235
node_2968: feature_name=TLN1	TLN1.value <= threshold=2.122964382171631
node_2969: feature_name=MT-CYB	MT-CYB.value > threshold=-0.9192460179328918
node_2973: feature_name=KIAA0125	KIAA0125.value <= threshold=6.9272027015686035

node_2974: feature_name=OAZ1	OAZ1.value > threshold=-1.8451763987541199
node_2978: feature_name=ATG2A	ATG2A.value > threshold=1.4784215688705444
node_2984: feature_name=ID3	ID3.value <= threshold=0.9155698791146278
Class: COVID-19 Critical	
Rules_216	passed counts:22
node_0: feature_name=LTB	LTB.value <= threshold=0.994031548500061
node_1: feature_name=MTRNR2L2	MTRNR2L2.value <= threshold=2.8784297704696655
node_2: feature_name=TCL1A	TCL1A.value <= threshold=4.798312664031982
node_3: feature_name=MT-CO1	MT-CO1.value > threshold=0.08947191759943962
node_2521: feature_name=LTB	LTB.value <= threshold=0.6038667261600494
node_2522: feature_name=HLA-DQB1	HLA-DQB1.value <= threshold=2.1053054332733154
node_2523: feature_name=SNX9	SNX9.value <= threshold=-0.5346486568450928
node_2524: feature_name=HLA-DPB1	HLA-DPB1.value <= threshold=1.9548270106315613
node_2525: feature_name=FAM129C	FAM129C.value <= threshold=3.3393388986587524
node_2526: feature_name=XIST	XIST.value <= threshold=3.6911416053771973
node_2527: feature_name=NR4A1	NR4A1.value > threshold=-0.2557755410671234
node_2625: feature_name=HLA-DPA1	HLA-DPA1.value > threshold=0.9335225224494934
node_2657: feature_name=RALGPS2	RALGPS2.value > threshold=2.9413715600967407
node_2701: feature_name=DUSP4	DUSP4.value <= threshold=-0.24480421096086502
node_2702: feature_name=MS4A1	MS4A1.value > threshold=1.7336164116859436
node_2704: feature_name=TLN1	TLN1.value <= threshold=0.8644028902053833
node_2705: feature_name=NT5C3A	NT5C3A.value <= threshold=2.03645521402359
node_2706: feature_name=ISG15	ISG15.value <= threshold=1.4324508309364319
Class: Healthy Control	
Rules_217	passed counts:22
node_0: feature_name=LTB	LTB.value <= threshold=0.994031548500061
node_1: feature_name=MTRNR2L2	MTRNR2L2.value <= threshold=2.8784297704696655
node_2: feature_name=TCL1A	TCL1A.value <= threshold=4.798312664031982

node_3: feature_name=MT-CO1	MT-CO1.value <= threshold=0.08947191759943962
node_4: feature_name=NR4A1	NR4A1.value > threshold=1.0725348591804504
node_1622: feature_name=HLA-DQA2	HLA-DQA2.value <= threshold=1.9496816992759705
node_1623: feature_name=HLA-DRB5	HLA-DRB5.value <= threshold=2.715766429901123
node_1624: feature_name=HLA-DPA1	HLA-DPA1.value <= threshold=1.7050301432609558
node_1625: feature_name=CD83	CD83.value > threshold=2.783489227294922
node_1873: feature_name=IFI44L	IFI44L.value <= threshold=1.5938722491264343
node_1874: feature_name=PLD4	PLD4.value <= threshold=4.101001739501953
node_1875: feature_name=RHOB	RHOB.value <= threshold=1.304835319519043
node_1876: feature_name=PPIF	PPIF.value <= threshold=2.2943923473358154
node_1877: feature_name=OAZ1	OAZ1.value <= threshold=0.04260866530239582
node_1878: feature_name=ID3	ID3.value <= threshold=5.290210247039795
node_1879: feature_name=NFKBID	NFKBID.value > threshold=-0.40532417595386505
node_1883: feature_name=MEF2C	MEF2C.value > threshold=-0.4597593992948532
node_1887: feature_name=SNX9	SNX9.value <= threshold=1.010923683643341
node_1888: feature_name=HLA-DRB5	HLA-DRB5.value > threshold=-0.4844578504562378
node_1890: feature_name=HLA-DPB1	HLA-DPB1.value <= threshold=1.4238839745521545
node_1891: feature_name=ATF5	ATF5.value <= threshold=5.457140207290649
Class: COVID-19 Critical	
Rules_218	passed counts:22
node_0: feature_name=LTB	LTB.value <= threshold=0.994031548500061
node_1: feature_name=MTRNR2L2	MTRNR2L2.value <= threshold=2.8784297704696655
node_2: feature_name=TCL1A	TCL1A.value <= threshold=4.798312664031982
node_3: feature_name=MT-CO1	MT-CO1.value <= threshold=0.08947191759943962
node_4: feature_name=NR4A1	NR4A1.value > threshold=1.0725348591804504
node_1622: feature_name=HLA-DQA2	HLA-DQA2.value <= threshold=1.9496816992759705
node_1623: feature_name=HLA-DRB5	HLA-DRB5.value <= threshold=2.715766429901123
node_1624: feature_name=HLA-DPA1	HLA-DPA1.value <= threshold=1.7050301432609558
node_1625: feature_name=CD83	CD83.value <= threshold=2.783489227294922

node_1626: feature_name=XIST	XIST.value <= threshold=0.9589543007314205
node_1627: feature_name=PLD4	PLD4.value <= threshold=5.865932464599609
node_1628: feature_name=OAZ1	OAZ1.value <= threshold=0.6137169003486633
node_1629: feature_name=MTRNR2L2	MTRNR2L2.value <= threshold=1.6102792620658875
node_1630: feature_name=IFI6	IFI6.value > threshold=-0.8404486179351807
node_1632: feature_name=NR4A1	NR4A1.value > threshold=1.0916320085525513
node_1638: feature_name=DENND3	DENND3.value <= threshold=3.988452672958374
node_1639: feature_name=HLA-DRB1	HLA-DRB1.value > threshold=1.4092575907707214
node_1749: feature_name=HLA-DQB1	HLA-DQB1.value <= threshold=3.4929215908050537
node_1750: feature_name=RP11-685N3.1	RP11-685N3.1.value <= threshold=9.97533893585205
node_1751: feature_name=RGS1	RGS1.value <= threshold=-0.5999330878257751
node_1752: feature_name=BCL2A1	BCL2A1.value <= threshold=1.773327887058258
node_1753: feature_name=BANK1	BANK1.value <= threshold=3.7594480514526367
node_1754: feature_name=MIAT	MIAT.value <= threshold=2.827533006668091
Class: COVID-19 Critical	
Rules_219	passed counts:21
node_0: feature_name=LTB	LTB.value > threshold=0.994031548500061
node_5264: feature_name=MT-CO1	MT-CO1.value > threshold=-0.4127991199493408
node_5638: feature_name=OAZ1	OAZ1.value > threshold=-0.2640867084264755
node_6112: feature_name=IFI44L	IFI44L.value <= threshold=2.5893263816833496
node_6113: feature_name=TCL1A	TCL1A.value <= threshold=4.201832294464111
node_6114: feature_name=HLA-DQA2	HLA-DQA2.value > threshold=-0.2270243540406227
node_6542: feature_name=CD79B	CD79B.value <= threshold=3.096232771873474
node_6543: feature_name=NR4A1	NR4A1.value <= threshold=1.421015739440918
node_6544: feature_name=LTB	LTB.value <= threshold=1.359755277633667
node_6545: feature_name=NFKBID	NFKBID.value > threshold=1.4294711351394653
node_6643: feature_name=FAM129C	FAM129C.value <= threshold=4.799884080886841
node_6644: feature_name=XIST	XIST.value <= threshold=0.9931697957217693
node_6645: feature_name=GPX1	GPX1.value <= threshold=0.3628883808851242

node_6646: feature_name=STMN1	STMN1.value <= threshold=2.3225364685058594
node_6647: feature_name=APP	APP.value <= threshold=4.124613881111145
node_6648: feature_name=FCER2	FCER2.value <= threshold=5.776939868927002
node_6649: feature_name=MEF2C	MEF2C.value <= threshold=3.8539518117904663
node_6650: feature_name=RHOB	RHOB.value > threshold=1.7790308594703674
node_6672: feature_name=CD79B	CD79B.value > threshold=-0.3273680806159973
node_6674: feature_name=MT-ND6	MT-ND6.value <= threshold=1.1112667322158813
node_6675: feature_name=RHOB	RHOB.value > threshold=1.8166040778160095
node_6679: feature_name=SNORD3B-2	SNORD3B-2.value <= threshold=1.751964621245861
Class: COVID-19 Critical	
Rules_220	passed counts:21
node_0: feature_name=LTB	LTB.value > threshold=0.994031548500061
node_5264: feature_name=MT-CO1	MT-CO1.value > threshold=-0.4127991199493408
node_5638: feature_name=OAZ1	OAZ1.value <= threshold=-0.2640867084264755
node_5639: feature_name=CD79B	CD79B.value <= threshold=2.2998517751693726
node_5640: feature_name=MTRNR2L2	MTRNR2L2.value <= threshold=3.5499863624572754
node_5641: feature_name=MT-CO1	MT-CO1.value > threshold=0.22567133605480194
node_5729: feature_name=MARCH1	MARCH1.value <= threshold=1.5154643654823303
node_5730: feature_name=MT-CO1	MT-CO1.value > threshold=1.7823500633239746
node_5856: feature_name=TTN	TTN.value <= threshold=-0.38264039158821106
node_5857: feature_name=IFI44L	IFI44L.value <= threshold=2.535575807094574
node_5858: feature_name=MTRNR2L2	MTRNR2L2.value <= threshold=2.948097348213196
Class: Healthy Control	
Rules_221	passed counts:21
node_0: feature_name=LTB	LTB.value > threshold=0.994031548500061
node_5264: feature_name=MT-CO1	MT-CO1.value <= threshold=-0.4127991199493408
node_5265: feature_name=MTRNR2L2	MTRNR2L2.value <= threshold=2.4473838806152344
node_5266: feature_name=TCL1A	TCL1A.value <= threshold=5.787256956100464

node_5267: feature_name=PLD4	PLD4.value <= threshold=3.544657826423645
node_5268: feature_name=NR4A1	NR4A1.value > threshold=0.8490704298019409
node_5498: feature_name=HLA-DPA1	HLA-DPA1.value <= threshold=1.9079211950302124
node_5499: feature_name=ID3	ID3.value <= threshold=-0.17505786567926407
node_5500: feature_name=PPIF	PPIF.value > threshold=-0.5180355906486511
node_5512: feature_name=PDLIM1	PDLIM1.value > threshold=2.5938297510147095
node_5526: feature_name=FTH1	FTH1.value <= threshold=0.17593900486826897
node_5527: feature_name=MIR24-2	MIR24-2.value <= threshold=1.0514202117919922
node_5528: feature_name=CD22	CD22.value <= threshold=3.0746376514434814
Class: COVID-19 Critical	
Rules_222	passed counts:21
node_0: feature_name=LTB	LTB.value <= threshold=0.994031548500061
node_1: feature_name=MTRNR2L2	MTRNR2L2.value <= threshold=2.8784297704696655
node_2: feature_name=TCL1A	TCL1A.value > threshold=4.798312664031982
node_4912: feature_name=MT-CO1	MT-CO1.value <= threshold=-0.3664844036102295
node_4913: feature_name=CD79A	CD79A.value > threshold=2.987208843231201
node_4973: feature_name=IFI44L	IFI44L.value > threshold=0.7183398455381393
node_4999: feature_name=MT-ND6	MT-ND6.value <= threshold=1.2110604643821716
Class: COVID-19 Critical	
Rules_223	passed counts:21
node_0: feature_name=LTB	LTB.value <= threshold=0.994031548500061
node_1: feature_name=MTRNR2L2	MTRNR2L2.value <= threshold=2.8784297704696655
node_2: feature_name=TCL1A	TCL1A.value <= threshold=4.798312664031982
node_3: feature_name=MT-CO1	MT-CO1.value > threshold=0.08947191759943962
node_2521: feature_name=LTB	LTB.value > threshold=0.6038667261600494
node_4173: feature_name=OAZ1	OAZ1.value > threshold=0.143283873796463
node_4615: feature_name=NR4A1	NR4A1.value <= threshold=0.6621004343032837
node_4616: feature_name=NFKBID	NFKBID.value <= threshold=3.283741593360901

node_4617: feature_name=HLA-DQA1	HLA-DQA1.value > threshold=2.3435752391815186
node_4719: feature_name=MX1	MX1.value <= threshold=1.851993978023529
node_4720: feature_name=JUN	JUN.value > threshold=-0.5025404393672943
node_4740: feature_name=IRF8	IRF8.value <= threshold=3.838419556617737
node_4741: feature_name=BACH1	BACH1.value > threshold=0.9568033516407013
node_4769: feature_name=TCF4	TCF4.value > threshold=-0.23278788477182388
node_4781: feature_name=ICAM1	ICAM1.value <= threshold=-0.24220328778028488
node_4782: feature_name=STAG3	STAG3.value <= threshold=3.4589697122573853
node_4783: feature_name=HLA-DRB5	HLA-DRB5.value <= threshold=2.5528095960617065
Class: Healthy Control	
Rules_224	passed counts:21
node_0: feature_name=LTB	LTB.value <= threshold=0.994031548500061
node_1: feature_name=MTRNR2L2	MTRNR2L2.value <= threshold=2.8784297704696655
node_2: feature_name=TCL1A	TCL1A.value <= threshold=4.798312664031982
node_3: feature_name=MT-CO1	MT-CO1.value > threshold=0.08947191759943962
node_2521: feature_name=LTB	LTB.value <= threshold=0.6038667261600494
node_2522: feature_name=HLA-DQB1	HLA-DQB1.value > threshold=2.1053054332733154
node_3550: feature_name=XIST	XIST.value <= threshold=0.7716337237507105
node_3551: feature_name=CD79B	CD79B.value <= threshold=2.9426796436309814
node_3552: feature_name=HLA-DQB1	HLA-DQB1.value > threshold=2.713674306869507
node_3854: feature_name=MEF2C	MEF2C.value > threshold=-0.3721291869878769
node_3882: feature_name=IFI44L	IFI44L.value <= threshold=2.5516022443771362
node_3883: feature_name=FCER2	FCER2.value <= threshold=-0.21487552672624588
node_3884: feature_name=HLA-DQA2	HLA-DQA2.value <= threshold=-0.12580305337905884
node_3885: feature_name=SLC7A5	SLC7A5.value <= threshold=-0.4906871169805527
node_3886: feature_name=VPREB3	VPREB3.value <= threshold=5.492124557495117
node_3887: feature_name=CYBB	CYBB.value <= threshold=2.720046043395996
node_3888: feature_name=CD79A	CD79A.value > threshold=3.3531476259231567
node_3930: feature_name=OAZ1	OAZ1.value <= threshold=0.8667621314525604

node_3931: feature_name=ADAM19	ADAM19.value <= threshold=4.43106484413147
Class: COVID-19 Critical	
Rules_225	passed counts:21
node_0: feature_name=LTB	LTB.value <= threshold=0.994031548500061
node_1: feature_name=MTRNR2L2	MTRNR2L2.value <= threshold=2.8784297704696655
node_2: feature_name=TCL1A	TCL1A.value <= threshold=4.798312664031982
node_3: feature_name=MT-CO1	MT-CO1.value > threshold=0.08947191759943962
node_2521: feature_name=LTB	LTB.value <= threshold=0.6038667261600494
node_2522: feature_name=HLA-DQB1	HLA-DQB1.value > threshold=2.1053054332733154
node_3550: feature_name=XIST	XIST.value <= threshold=0.7716337237507105
node_3551: feature_name=CD79B	CD79B.value <= threshold=2.9426796436309814
node_3552: feature_name=HLA-DQB1	HLA-DQB1.value > threshold=2.713674306869507
node_3854: feature_name=MEF2C	MEF2C.value <= threshold=-0.3721291869878769
node_3855: feature_name=HLA-DRB5	HLA-DRB5.value > threshold=0.7929701805114746
node_3859: feature_name=LTB	LTB.value > threshold=-0.9689712226390839
node_3863: feature_name=RP11-731F5.2	RP11-731F5.2.value <= threshold=3.578703761100769
node_3864: feature_name=COTL1	COTL1.value <= threshold=0.38136887550354004
node_3865: feature_name=HLA-DQA1	HLA-DQA1.value <= threshold=2.6825207471847534
node_3866: feature_name=FCER2	FCER2.value <= threshold=4.623371601104736
node_3867: feature_name=IFI44L	IFI44L.value <= threshold=1.8092790842056274
Class: COVID-19 Severe	
Rules_226	passed counts:21
node_0: feature_name=LTB	LTB.value <= threshold=0.994031548500061
node_1: feature_name=MTRNR2L2	MTRNR2L2.value <= threshold=2.8784297704696655
node_2: feature_name=TCL1A	TCL1A.value <= threshold=4.798312664031982
node_3: feature_name=MT-CO1	MT-CO1.value > threshold=0.08947191759943962
node_2521: feature_name=LTB	LTB.value <= threshold=0.6038667261600494
node_2522: feature_name=HLA-DQB1	HLA-DQB1.value <= threshold=2.1053054332733154

node_2523: feature_name=SNX9	SNX9.value > threshold=-0.5346486568450928
node_2907: feature_name=NR4A1	NR4A1.value <= threshold=2.5433837175369263
node_2908: feature_name=XIST	XIST.value > threshold=0.7664470989257097
node_3440: feature_name=MT-CO1	MT-CO1.value <= threshold=0.9336088299751282
node_3441: feature_name=WDR74	WDR74.value <= threshold=1.7392029166221619
node_3442: feature_name=SNX29P2	SNX29P2.value <= threshold=4.647986650466919
node_3443: feature_name=OAZ1	OAZ1.value > threshold=-1.6502272486686707
Class: COVID-19 Critical	
Rules_227	passed counts:21
node_0: feature_name=LTB	LTB.value <= threshold=0.994031548500061
node_1: feature_name=MTRNR2L2	MTRNR2L2.value <= threshold=2.8784297704696655
node_2: feature_name=TCL1A	TCL1A.value <= threshold=4.798312664031982
node_3: feature_name=MT-CO1	MT-CO1.value <= threshold=0.08947191759943962
node_4: feature_name=NR4A1	NR4A1.value > threshold=1.0725348591804504
node_1622: feature_name=HLA-DQA2	HLA-DQA2.value <= threshold=1.9496816992759705
node_1623: feature_name=HLA-DRB5	HLA-DRB5.value <= threshold=2.715766429901123
node_1624: feature_name=HLA-DPA1	HLA-DPA1.value > threshold=1.7050301432609558
node_2056: feature_name=OAZ1	OAZ1.value <= threshold=0.456103190779686
node_2057: feature_name=HLA-DRB5	HLA-DRB5.value > threshold=0.6839984953403473
node_2101: feature_name=HLA-DRB1	HLA-DRB1.value <= threshold=1.6199676990509033
node_2102: feature_name=CD83	CD83.value > threshold=2.5568599700927734
node_2124: feature_name=MARCH1	MARCH1.value <= threshold=-0.34506022930145264
node_2125: feature_name=FTH1	FTH1.value > threshold=-0.19983553141355515
node_2129: feature_name=TCL1A	TCL1A.value <= threshold=1.2124098315835
node_2130: feature_name=HLA-DQA1	HLA-DQA1.value <= threshold=2.700020432472229
node_2131: feature_name=MT-CO1	MT-CO1.value <= threshold=0.07234566658735275
node_2132: feature_name=MT-CO3	MT-CO3.value <= threshold=0.4440104067325592
Class: COVID-19 Severe	

Rules_228	passed counts:21
node_0: feature_name=LTB	LTB.value <= threshold=0.994031548500061
node_1: feature_name=MTRNR2L2	MTRNR2L2.value <= threshold=2.8784297704696655
node_2: feature_name=TCL1A	TCL1A.value <= threshold=4.798312664031982
node_3: feature_name=MT-CO1	MT-CO1.value <= threshold=0.08947191759943962
node_4: feature_name=NR4A1	NR4A1.value <= threshold=1.0725348591804504
node_5: feature_name=HLA-DPA1	HLA-DPA1.value > threshold=1.6885469555854797
node_861: feature_name=CD83	CD83.value <= threshold=3.0563642978668213
node_862: feature_name=HLA-DQB1	HLA-DQB1.value <= threshold=2.61081063747406
node_863: feature_name=MTRNR2L2	MTRNR2L2.value <= threshold=2.0919997692108154
node_864: feature_name=BCL2A1	BCL2A1.value <= threshold=1.852618932723999
node_865: feature_name=CD79B	CD79B.value <= threshold=4.352142333984375
node_866: feature_name=TNFAIP3	TNFAIP3.value > threshold=-0.0449496041983366
node_1236: feature_name=HLA-DRB5	HLA-DRB5.value > threshold=1.710871696472168
node_1268: feature_name=MT-CO1	MT-CO1.value <= threshold=-0.8004095554351807
node_1269: feature_name=MTRNR2L8	MTRNR2L8.value <= threshold=-0.3383208364248276
node_1270: feature_name=PELI1	PELI1.value > threshold=-0.6302085220813751
node_1276: feature_name=UTY	UTY.value <= threshold=0.6420612782239914
Class: COVID-19 Severe	
Rules_229	passed counts:21
node_0: feature_name=LTB	LTB.value <= threshold=0.994031548500061
node_1: feature_name=MTRNR2L2	MTRNR2L2.value <= threshold=2.8784297704696655
node_2: feature_name=TCL1A	TCL1A.value <= threshold=4.798312664031982
node_3: feature_name=MT-CO1	MT-CO1.value <= threshold=0.08947191759943962
node_4: feature_name=NR4A1	NR4A1.value <= threshold=1.0725348591804504
node_5: feature_name=HLA-DPA1	HLA-DPA1.value > threshold=1.6885469555854797
node_861: feature_name=CD83	CD83.value <= threshold=3.0563642978668213
node_862: feature_name=HLA-DQB1	HLA-DQB1.value <= threshold=2.61081063747406
node_863: feature_name=MTRNR2L2	MTRNR2L2.value <= threshold=2.0919997692108154

node_864: feature_name=BCL2A1	BCL2A1.value <= threshold=1.852618932723999
node_865: feature_name=CD79B	CD79B.value <= threshold=4.352142333984375
node_866: feature_name=TNFAIP3	TNFAIP3.value <= threshold=-0.0449496041983366
node_867: feature_name=MT-CO1	MT-CO1.value > threshold=-0.08531957119703293
node_1155: feature_name=IRF8	IRF8.value > threshold=-0.39239931106567383
node_1177: feature_name=PELI1	PELI1.value <= threshold=-0.6234017610549927
node_1178: feature_name=HLA-DRB1	HLA-DRB1.value > threshold=1.3807418942451477
node_1182: feature_name=ANO6	ANO6.value > threshold=-0.3352542072534561
node_1198: feature_name=FTH1	FTH1.value <= threshold=0.6898834407329559
node_1199: feature_name=OAZ1	OAZ1.value <= threshold=1.3686169981956482
Class: COVID-19 Critical	
Rules_230	passed counts:21
node_0: feature_name=LTB	LTB.value <= threshold=0.994031548500061
node_1: feature_name=MTRNR2L2	MTRNR2L2.value <= threshold=2.8784297704696655
node_2: feature_name=TCL1A	TCL1A.value <= threshold=4.798312664031982
node_3: feature_name=MT-CO1	MT-CO1.value <= threshold=0.08947191759943962
node_4: feature_name=NR4A1	NR4A1.value <= threshold=1.0725348591804504
node_5: feature_name=HLA-DPA1	HLA-DPA1.value <= threshold=1.6885469555854797
node_6: feature_name=HLA-DRB5	HLA-DRB5.value <= threshold=2.1886194944381714
node_7: feature_name=XIST	XIST.value <= threshold=-0.12328458577394485
node_8: feature_name=SYNE1	SYNE1.value > threshold=-0.7835594415664673
node_16: feature_name=MTRNR2L2	MTRNR2L2.value <= threshold=2.4483247995376587
node_17: feature_name=HLA-DQA2	HLA-DQA2.value <= threshold=2.227178931236267
node_18: feature_name=NR4A1	NR4A1.value <= threshold=-0.21380723267793655
node_19: feature_name=IRAK3	IRAK3.value <= threshold=4.27159571647644
node_20: feature_name=IFIT2	IFIT2.value <= threshold=7.8175740242004395
node_21: feature_name=CD79A	CD79A.value <= threshold=2.857242226600647
node_22: feature_name=HIST1H2AC	HIST1H2AC.value <= threshold=5.077470302581787
node_23: feature_name=FTH1	FTH1.value <= threshold=0.4158056080341339

node_24: feature_name=ATF3	ATF3.value <= threshold=3.8028353452682495
node_25: feature_name=OASL	OASL.value <= threshold=3.387580633163452
node_26: feature_name=MT-CO1	MT-CO1.value > threshold=-0.7942646145820618
node_74: feature_name=HOPX	HOPX.value <= threshold=0.8682331740856171
node_75: feature_name=RGS2	RGS2.value <= threshold=1.4589954018592834
node_76: feature_name=AHR	AHR.value <= threshold=3.870481848716736
node_77: feature_name=IGJ	IGJ.value <= threshold=4.902882814407349
node_78: feature_name=CD79B	CD79B.value <= threshold=4.87716269493103
node_79: feature_name=HLA-DQB1	HLA-DQB1.value <= threshold=3.6925491094589233
node_80: feature_name=HLA-DRB5	HLA-DRB5.value <= threshold=2.0926843881607056
node_81: feature_name=MCTP1	MCTP1.value <= threshold=4.010472774505615
node_82: feature_name=GNG11	GNG11.value <= threshold=3.627470850944519
node_83: feature_name=HSP90B1	HSP90B1.value <= threshold=2.0835131406784058
node_84: feature_name=ATF3	ATF3.value <= threshold=1.1142374873161316
node_85: feature_name=MT-ND6	MT-ND6.value <= threshold=2.7328014373779297
node_86: feature_name=COTL1	COTL1.value > threshold=-1.6524587869644165
node_90: feature_name=MT-CYB	MT-CYB.value <= threshold=1.099629819393158
node_91: feature_name=RGCC	RGCC.value <= threshold=1.6908138990402222
node_92: feature_name=ILK	ILK.value <= threshold=3.9497616291046143
node_93: feature_name=CTD-2035E11.3	CTD-2035E11.3.value <= threshold=2.4543306827545166
node_94: feature_name=S100A6	S100A6.value <= threshold=0.2867250442504883
node_95: feature_name=HLA-DRB5	HLA-DRB5.value > threshold=-0.9496813416481018
node_99: feature_name=MT-CO3	MT-CO3.value > threshold=-1.55654776096344
node_103: feature_name=ITGAX	ITGAX.value <= threshold=1.8544010519981384
node_104: feature_name=MEF2C	MEF2C.value <= threshold=4.22724461555481
node_105: feature_name=CXorf57	CXorf57.value <= threshold=5.114444732666016
node_106: feature_name=IRAK3	IRAK3.value <= threshold=3.1017903089523315
node_107: feature_name=DUSP4	DUSP4.value > threshold=2.9122174978256226
node_137: feature_name=HLA-DRB5	HLA-DRB5.value <= threshold=1.718787968158722
Class: COVID-19 Critical	

Rules_231	passed counts:20
node_0: feature_name=LTB	LTB.value > threshold=0.994031548500061
node_5264: feature_name=MT-CO1	MT-CO1.value > threshold=-0.4127991199493408
node_5638: feature_name=OAZ1	OAZ1.value > threshold=-0.2640867084264755
node_6112: feature_name=IFI44L	IFI44L.value <= threshold=2.5893263816833496
node_6113: feature_name=TCL1A	TCL1A.value <= threshold=4.201832294464111
node_6114: feature_name=HLA-DQA2	HLA-DQA2.value <= threshold=-0.2270243540406227
node_6115: feature_name=MTRNR2L2	MTRNR2L2.value <= threshold=2.548917293548584
node_6116: feature_name=ATF5	ATF5.value <= threshold=2.041263461112976
node_6117: feature_name=JUN	JUN.value > threshold=-0.616259902715683
node_6189: feature_name=MT-CO3	MT-CO3.value > threshold=0.13483521342277527
node_6351: feature_name=XAF1	XAF1.value <= threshold=3.061717987060547
node_6352: feature_name=MT-CO2	MT-CO2.value <= threshold=1.2605181336402893
node_6353: feature_name=ICAM1	ICAM1.value <= threshold=-0.3340446650981903
node_6354: feature_name=OAZ1	OAZ1.value > threshold=-0.24055946618318558
node_6360: feature_name=PPIF	PPIF.value <= threshold=2.070030093193054
node_6361: feature_name=RALGPS2	RALGPS2.value <= threshold=-0.3684315085411072
node_6362: feature_name=JUN	JUN.value > threshold=-0.10340499877929688
node_6366: feature_name=TCF4	TCF4.value <= threshold=4.341203451156616
Class: Healthy Control	
Rules_232	passed counts:20
node_0: feature_name=LTB	LTB.value > threshold=0.994031548500061
node_5264: feature_name=MT-CO1	MT-CO1.value > threshold=-0.4127991199493408
node_5638: feature_name=OAZ1	OAZ1.value <= threshold=-0.2640867084264755
node_5639: feature_name=CD79B	CD79B.value <= threshold=2.2998517751693726
node_5640: feature_name=MTRNR2L2	MTRNR2L2.value <= threshold=3.5499863624572754
node_5641: feature_name=MT-CO1	MT-CO1.value > threshold=0.22567133605480194
node_5729: feature_name=MARCH1	MARCH1.value <= threshold=1.5154643654823303

node_5730: feature_name=MT-CO1	MT-CO1.value <= threshold=1.7823500633239746
node_5731: feature_name=HLA-DPB1	HLA-DPB1.value > threshold=2.0096163749694824
node_5829: feature_name=LTB	LTB.value > threshold=1.2124510407447815
node_5843: feature_name=TTN	TTN.value > threshold=-0.3821280151605606
node_5851: feature_name=SNX9	SNX9.value <= threshold=1.9138266444206238
Class: Healthy Control	
Rules_233	passed counts:20
node_0: feature_name=LTB	LTB.value > threshold=0.994031548500061
node_5264: feature_name=MT-CO1	MT-CO1.value <= threshold=-0.4127991199493408
node_5265: feature_name=MTRNR2L2	MTRNR2L2.value <= threshold=2.4473838806152344
node_5266: feature_name=TCL1A	TCL1A.value <= threshold=5.787256956100464
node_5267: feature_name=PLD4	PLD4.value <= threshold=3.544657826423645
node_5268: feature_name=NR4A1	NR4A1.value <= threshold=0.8490704298019409
node_5269: feature_name=LTB	LTB.value > threshold=1.5252206921577454
node_5471: feature_name=CD79A	CD79A.value > threshold=2.510846734046936
node_5483: feature_name=XAF1	XAF1.value <= threshold=1.7000007629394531
node_5484: feature_name=RALGPS2	RALGPS2.value > threshold=2.08430278301239
node_5492: feature_name=FTH1	FTH1.value <= threshold=-0.024074797984212637
node_5493: feature_name=MT-CYB	MT-CYB.value <= threshold=0.4046303331851959
Class: Healthy Control	
Rules_234	passed counts:20
node_0: feature_name=LTB	LTB.value <= threshold=0.994031548500061
node_1: feature_name=MTRNR2L2	MTRNR2L2.value <= threshold=2.8784297704696655
node_2: feature_name=TCL1A	TCL1A.value > threshold=4.798312664031982
node_4912: feature_name=MT-CO1	MT-CO1.value > threshold=-0.3664844036102295
node_5002: feature_name=IFI44L	IFI44L.value <= threshold=2.3101747035980225
node_5003: feature_name=SNX9	SNX9.value <= threshold=2.0247421264648438
node_5004: feature_name=STAG3	STAG3.value <= threshold=6.482141733169556

node_5005: feature_name=BACH1	BACH1.value > threshold=-0.5237349569797516
node_5047: feature_name=RHOB	RHOB.value > threshold=-0.4843696355819702
node_5055: feature_name=FAM129C	FAM129C.value <= threshold=-0.2832885980606079
node_5056: feature_name=CD83	CD83.value > threshold=-0.5592367649078369
node_5060: feature_name=OAZ1	OAZ1.value <= threshold=0.7524859011173248
node_5061: feature_name=FCRL1	FCRL1.value <= threshold=5.677775859832764
node_5062: feature_name=RNF144B	RNF144B.value <= threshold=1.2688461616635323
node_5063: feature_name=ID1	ID1.value <= threshold=-0.1296444833278656
node_5064: feature_name=LDLRAD4	LDLRAD4.value <= threshold=3.7435786724090576
Class: COVID-19 Critical	
Rules_235	passed counts:20
node_0: feature_name=LTB	LTB.value <= threshold=0.994031548500061
node_1: feature_name=MTRNR2L2	MTRNR2L2.value <= threshold=2.8784297704696655
node_2: feature_name=TCL1A	TCL1A.value <= threshold=4.798312664031982
node_3: feature_name=MT-CO1	MT-CO1.value > threshold=0.08947191759943962
node_2521: feature_name=LTB	LTB.value > threshold=0.6038667261600494
node_4173: feature_name=OAZ1	OAZ1.value > threshold=0.143283873796463
node_4615: feature_name=NR4A1	NR4A1.value > threshold=0.6621004343032837
node_4817: feature_name=HSP90B1	HSP90B1.value > threshold=1.1699737310409546
node_4895: feature_name=MS4A1	MS4A1.value > threshold=2.627432107925415
node_4903: feature_name=MARCH1	MARCH1.value <= threshold=3.4180619716644287
node_4904: feature_name=DAPP1	DAPP1.value <= threshold=-0.28873395919799805
node_4905: feature_name=MEF2A	MEF2A.value <= threshold=3.0424721240997314
Class: Healthy Control	
Rules_236	passed counts:20
node_0: feature_name=LTB	LTB.value <= threshold=0.994031548500061
node_1: feature_name=MTRNR2L2	MTRNR2L2.value <= threshold=2.8784297704696655
node_2: feature_name=TCL1A	TCL1A.value <= threshold=4.798312664031982

node_3: feature_name=MT-CO1	MT-CO1.value > threshold=0.08947191759943962
node_2521: feature_name=LTB	LTB.value <= threshold=0.6038667261600494
node_2522: feature_name=HLA-DQB1	HLA-DQB1.value <= threshold=2.1053054332733154
node_2523: feature_name=SNX9	SNX9.value <= threshold=-0.5346486568450928
node_2524: feature_name=HLA-DPB1	HLA-DPB1.value > threshold=1.9548270106315613
node_2814: feature_name=CD79B	CD79B.value > threshold=-0.28186772763729095
node_2826: feature_name=MT-ND6	MT-ND6.value <= threshold=0.17273088544607162
node_2827: feature_name=NR4A1	NR4A1.value > threshold=-0.24634335190057755
node_2849: feature_name=FAM129C	FAM129C.value <= threshold=3.089340925216675
node_2850: feature_name=COTL1	COTL1.value > threshold=-0.5968812704086304
node_2856: feature_name=DENND3	DENND3.value > threshold=-0.3556941896677017
node_2864: feature_name=HSP90B1	HSP90B1.value <= threshold=1.98911714553833
Class: Healthy Control	
Rules_237	passed counts:20
node_0: feature_name=LTB	LTB.value <= threshold=0.994031548500061
node_1: feature_name=MTRNR2L2	MTRNR2L2.value <= threshold=2.8784297704696655
node_2: feature_name=TCL1A	TCL1A.value <= threshold=4.798312664031982
node_3: feature_name=MT-CO1	MT-CO1.value > threshold=0.08947191759943962
node_2521: feature_name=LTB	LTB.value <= threshold=0.6038667261600494
node_2522: feature_name=HLA-DQB1	HLA-DQB1.value <= threshold=2.1053054332733154
node_2523: feature_name=SNX9	SNX9.value <= threshold=-0.5346486568450928
node_2524: feature_name=HLA-DPB1	HLA-DPB1.value <= threshold=1.9548270106315613
node_2525: feature_name=FAM129C	FAM129C.value > threshold=3.3393388986587524
node_2721: feature_name=ADAM19	ADAM19.value > threshold=-0.29525794088840485
node_2781: feature_name=XAF1	XAF1.value <= threshold=-0.4296249598264694
node_2782: feature_name=CYBB	CYBB.value > threshold=-0.5005911737680435
node_2790: feature_name=MX1	MX1.value > threshold=-0.4843023121356964
Class: COVID-19 Critical	

Rules_238	passed counts:20
node_0: feature_name=LTB	LTB.value <= threshold=0.994031548500061
node_1: feature_name=MTRNR2L2	MTRNR2L2.value <= threshold=2.8784297704696655
node_2: feature_name=TCL1A	TCL1A.value <= threshold=4.798312664031982
node_3: feature_name=MT-CO1	MT-CO1.value > threshold=0.08947191759943962
node_2521: feature_name=LTB	LTB.value <= threshold=0.6038667261600494
node_2522: feature_name=HLA-DQB1	HLA-DQB1.value <= threshold=2.1053054332733154
node_2523: feature_name=SNX9	SNX9.value <= threshold=-0.5346486568450928
node_2524: feature_name=HLA-DPB1	HLA-DPB1.value <= threshold=1.9548270106315613
node_2525: feature_name=FAM129C	FAM129C.value > threshold=3.3393388986587524
node_2721: feature_name=ADAM19	ADAM19.value <= threshold=-0.29525794088840485
node_2722: feature_name=NR4A1	NR4A1.value <= threshold=2.1566513776779175
node_2723: feature_name=CD22	CD22.value <= threshold=8.345317840576172
node_2724: feature_name=PLD4	PLD4.value <= threshold=7.507996082305908
node_2725: feature_name=RALGPS2	RALGPS2.value > threshold=4.3808369636535645
node_2751: feature_name=NFKBID	NFKBID.value > threshold=-0.4283874034881592
node_2757: feature_name=MT-CO1	MT-CO1.value <= threshold=1.7044780254364014
node_2758: feature_name=NAMPT	NAMPT.value <= threshold=0.7461279034614563
node_2759: feature_name=CCR6	CCR6.value <= threshold=2.5590972006320953
Class: COVID-19 Critical	
Rules_239	passed counts:20
node_0: feature_name=LTB	LTB.value <= threshold=0.994031548500061
node_1: feature_name=MTRNR2L2	MTRNR2L2.value <= threshold=2.8784297704696655
node_2: feature_name=TCL1A	TCL1A.value <= threshold=4.798312664031982
node_3: feature_name=MT-CO1	MT-CO1.value <= threshold=0.08947191759943962
node_4: feature_name=NR4A1	NR4A1.value > threshold=1.0725348591804504
node_1622: feature_name=HLA-DQA2	HLA-DQA2.value <= threshold=1.9496816992759705
node_1623: feature_name=HLA-DRB5	HLA-DRB5.value <= threshold=2.715766429901123
node_1624: feature_name=HLA-DPA1	HLA-DPA1.value <= threshold=1.7050301432609558

node_1625: feature_name=CD83	CD83.value > threshold=2.783489227294922
node_1873: feature_name=IFI44L	IFI44L.value <= threshold=1.5938722491264343
node_1874: feature_name=PLD4	PLD4.value <= threshold=4.101001739501953
node_1875: feature_name=RHOB	RHOB.value > threshold=1.304835319519043
node_1965: feature_name=HLA-DQB1	HLA-DQB1.value <= threshold=1.5989137291908264
node_1966: feature_name=CD79A	CD79A.value <= threshold=2.189441442489624
node_1967: feature_name=TNF	TNF.value <= threshold=2.0489413142204285
node_1968: feature_name=MT1E	MT1E.value <= threshold=-0.14949391037225723
Class: COVID-19 Severe	
Rules_240	passed counts:20
node_0: feature_name=LTB	LTB.value <= threshold=0.994031548500061
node_1: feature_name=MTRNR2L2	MTRNR2L2.value <= threshold=2.8784297704696655
node_2: feature_name=TCL1A	TCL1A.value <= threshold=4.798312664031982
node_3: feature_name=MT-CO1	MT-CO1.value <= threshold=0.08947191759943962
node_4: feature_name=NR4A1	NR4A1.value <= threshold=1.0725348591804504
node_5: feature_name=HLA-DPA1	HLA-DPA1.value > threshold=1.6885469555854797
node_861: feature_name=CD83	CD83.value <= threshold=3.0563642978668213
node_862: feature_name=HLA-DQB1	HLA-DQB1.value > threshold=2.61081063747406
node_1358: feature_name=HLA-DRB5	HLA-DRB5.value > threshold=0.7542744874954224
node_1390: feature_name=HLA-DRB5	HLA-DRB5.value <= threshold=2.6244447231292725
node_1391: feature_name=XIST	XIST.value <= threshold=-0.22017984092235565
node_1392: feature_name=MT-CO3	MT-CO3.value > threshold=0.12647542357444763
node_1472: feature_name=IFI44L	IFI44L.value > threshold=-0.34079885482788086
node_1494: feature_name=DAPP1	DAPP1.value <= threshold=4.9949791431427
Class: COVID-19 Critical	
Rules_241	passed counts:20
node_0: feature_name=LTB	LTB.value <= threshold=0.994031548500061
node_1: feature_name=MTRNR2L2	MTRNR2L2.value <= threshold=2.8784297704696655

node_2: feature_name=TCL1A	TCL1A.value <= threshold=4.798312664031982
node_3: feature_name=MT-CO1	MT-CO1.value <= threshold=0.08947191759943962
node_4: feature_name=NR4A1	NR4A1.value <= threshold=1.0725348591804504
node_5: feature_name=HLA-DPA1	HLA-DPA1.value > threshold=1.6885469555854797
node_861: feature_name=CD83	CD83.value <= threshold=3.0563642978668213
node_862: feature_name=HLA-DQB1	HLA-DQB1.value <= threshold=2.61081063747406
node_863: feature_name=MTRNR2L2	MTRNR2L2.value <= threshold=2.0919997692108154
node_864: feature_name=BCL2A1	BCL2A1.value <= threshold=1.852618932723999
node_865: feature_name=CD79B	CD79B.value <= threshold=4.352142333984375
node_866: feature_name=TNFAIP3	TNFAIP3.value <= threshold=-0.0449496041983366
node_867: feature_name=MT-CO1	MT-CO1.value <= threshold=-0.08531957119703293
node_868: feature_name=MTRNR2L8	MTRNR2L8.value <= threshold=0.8770350813865662
node_869: feature_name=EGR1	EGR1.value <= threshold=-0.32270750403404236
node_870: feature_name=IGJ	IGJ.value <= threshold=4.618318557739258
node_871: feature_name=DUSP4	DUSP4.value <= threshold=2.8197922706604004
node_872: feature_name=HOPX	HOPX.value <= threshold=2.10397732257843
node_873: feature_name=RGCC	RGCC.value <= threshold=1.2070720195770264
node_874: feature_name=OAZ1	OAZ1.value <= threshold=1.02116060256958
node_875: feature_name=NAIP	NAIP.value <= threshold=3.768904685974121
node_876: feature_name=LDLR	LDLR.value <= threshold=2.9582698345184326
node_877: feature_name=HLA-DPA1	HLA-DPA1.value <= threshold=3.0588693618774414
node_878: feature_name=RHOB	RHOB.value <= threshold=5.08755898475647
node_879: feature_name=SYTL3	SYTL3.value <= threshold=1.5127440690994263
node_880: feature_name=FTH1	FTH1.value > threshold=-1.4152275919914246
node_882: feature_name=CCL3	CCL3.value <= threshold=2.253131687641144
node_883: feature_name=CD83	CD83.value > threshold=2.8566592931747437
node_967: feature_name=MT-CO3	MT-CO3.value > threshold=-0.9992981255054474
node_971: feature_name=SPI1	SPI1.value <= threshold=0.7543019354343414
Class: COVID-19 Critical	

Rules_242	passed counts:20
node_0: feature_name=LTB	LTB.value <= threshold=0.994031548500061
node_1: feature_name=MTRNR2L2	MTRNR2L2.value <= threshold=2.8784297704696655
node_2: feature_name=TCL1A	TCL1A.value <= threshold=4.798312664031982
node_3: feature_name=MT-CO1	MT-CO1.value <= threshold=0.08947191759943962
node_4: feature_name=NR4A1	NR4A1.value <= threshold=1.0725348591804504
node_5: feature_name=HLA-DPA1	HLA-DPA1.value <= threshold=1.6885469555854797
node_6: feature_name=HLA-DRB5	HLA-DRB5.value <= threshold=2.1886194944381714
node_7: feature_name=XIST	XIST.value <= threshold=-0.12328458577394485
node_8: feature_name=SYNE1	SYNE1.value > threshold=-0.7835594415664673
node_16: feature_name=MTRNR2L2	MTRNR2L2.value <= threshold=2.4483247995376587
node_17: feature_name=HLA-DQA2	HLA-DQA2.value > threshold=2.227178931236267
node_591: feature_name=BCL2A1	BCL2A1.value <= threshold=1.0648428201675415
node_592: feature_name=FTH1	FTH1.value <= threshold=0.8842596709728241
node_593: feature_name=SNX9	SNX9.value <= threshold=3.232524275779724
node_594: feature_name=OAZ1	OAZ1.value <= threshold=1.704061508178711
node_595: feature_name=MT-CYB	MT-CYB.value > threshold=0.5040059983730316
node_657: feature_name=BCL2A1	BCL2A1.value <= threshold=0.7144843935966492
node_658: feature_name=CTD-2035E11.3	CTD-2035E11.3.value <= threshold=-0.25902947783470154
node_659: feature_name=LTB	LTB.value <= threshold=0.6877703368663788
Class: COVID-19 Critical	
Rules_243	passed counts:20
node_0: feature_name=LTB	LTB.value <= threshold=0.994031548500061
node_1: feature_name=MTRNR2L2	MTRNR2L2.value <= threshold=2.8784297704696655
node_2: feature_name=TCL1A	TCL1A.value <= threshold=4.798312664031982
node_3: feature_name=MT-CO1	MT-CO1.value <= threshold=0.08947191759943962
node_4: feature_name=NR4A1	NR4A1.value <= threshold=1.0725348591804504
node_5: feature_name=HLA-DPA1	HLA-DPA1.value <= threshold=1.6885469555854797
node_6: feature_name=HLA-DRB5	HLA-DRB5.value <= threshold=2.1886194944381714

node_7: feature_name=XIST	XIST.value <= threshold=-0.12328458577394485
node_8: feature_name=SYNE1	SYNE1.value > threshold=-0.7835594415664673
node_16: feature_name=MTRNR2L2	MTRNR2L2.value <= threshold=2.4483247995376587
node_17: feature_name=HLA-DQA2	HLA-DQA2.value <= threshold=2.227178931236267
node_18: feature_name=NR4A1	NR4A1.value <= threshold=-0.21380723267793655
node_19: feature_name=IRAK3	IRAK3.value <= threshold=4.27159571647644
node_20: feature_name=IFIT2	IFIT2.value <= threshold=7.8175740242004395
node_21: feature_name=CD79A	CD79A.value <= threshold=2.857242226600647
node_22: feature_name=HIST1H2AC	HIST1H2AC.value <= threshold=5.077470302581787
node_23: feature_name=FTH1	FTH1.value > threshold=0.4158056080341339
node_285: feature_name=EGR1	EGR1.value <= threshold=4.240467071533203
node_286: feature_name=HLA-DRB1	HLA-DRB1.value > threshold=-0.7553382813930511
node_290: feature_name=TLR2	TLR2.value <= threshold=2.879140257835388
node_291: feature_name=SYNE1	SYNE1.value <= threshold=2.323199510574341
node_292: feature_name=BCL2A1	BCL2A1.value <= threshold=1.8940600156784058
node_293: feature_name=RALGPS2	RALGPS2.value <= threshold=4.808189153671265
node_294: feature_name=IGLL5	IGLL5.value <= threshold=4.116017818450928
node_295: feature_name=FTH1	FTH1.value > threshold=0.41612209379673004
node_297: feature_name=MCTP1	MCTP1.value <= threshold=4.601342678070068
node_298: feature_name=MT-CO2	MT-CO2.value <= threshold=0.6201252341270447
node_299: feature_name=MARCKS	MARCKS.value <= threshold=3.471073269844055
node_300: feature_name=PPIF	PPIF.value <= threshold=2.8470659255981445
node_301: feature_name=CCL3	CCL3.value <= threshold=1.9901156425476074
node_302: feature_name=MT-CO2	MT-CO2.value > threshold=-2.867768883705139
node_306: feature_name=RHOB	RHOB.value <= threshold=3.9839000701904297
node_307: feature_name=SNX29P2	SNX29P2.value <= threshold=5.035826921463013
node_308: feature_name=MT-CO3	MT-CO3.value <= threshold=0.7808255851268768
node_309: feature_name=RGCC	RGCC.value <= threshold=1.070733904838562
node_310: feature_name=SESN3	SESN3.value <= threshold=2.5199365615844727
node_311: feature_name=ABCA1	ABCA1.value > threshold=2.989766836166382

node_313: feature_name=NR4A3	NR4A3.value > threshold=-0.4175230860710144
Class: COVID-19 Critical	
Rules_244	passed counts:20
node_0: feature_name=LTB	LTB.value <= threshold=0.994031548500061
node_1: feature_name=MTRNR2L2	MTRNR2L2.value <= threshold=2.8784297704696655
node_2: feature_name=TCL1A	TCL1A.value <= threshold=4.798312664031982
node_3: feature_name=MT-CO1	MT-CO1.value <= threshold=0.08947191759943962
node_4: feature_name=NR4A1	NR4A1.value <= threshold=1.0725348591804504
node_5: feature_name=HLA-DPA1	HLA-DPA1.value <= threshold=1.6885469555854797
node_6: feature_name=HLA-DRB5	HLA-DRB5.value <= threshold=2.1886194944381714
node_7: feature_name=XIST	XIST.value <= threshold=-0.12328458577394485
node_8: feature_name=SYNE1	SYNE1.value > threshold=-0.7835594415664673
node_16: feature_name=MTRNR2L2	MTRNR2L2.value <= threshold=2.4483247995376587
node_17: feature_name=HLA-DQA2	HLA-DQA2.value <= threshold=2.227178931236267
node_18: feature_name=NR4A1	NR4A1.value <= threshold=-0.21380723267793655
node_19: feature_name=IRAK3	IRAK3.value <= threshold=4.27159571647644
node_20: feature_name=IFIT2	IFIT2.value <= threshold=7.8175740242004395
node_21: feature_name=CD79A	CD79A.value <= threshold=2.857242226600647
node_22: feature_name=HIST1H2AC	HIST1H2AC.value <= threshold=5.077470302581787
node_23: feature_name=FTH1	FTH1.value <= threshold=0.4158056080341339
node_24: feature_name=ATF3	ATF3.value <= threshold=3.8028353452682495
node_25: feature_name=OASL	OASL.value <= threshold=3.387580633163452
node_26: feature_name=MT-CO1	MT-CO1.value > threshold=-0.7942646145820618
node_74: feature_name=HOPX	HOPX.value <= threshold=0.8682331740856171
node_75: feature_name=RGS2	RGS2.value <= threshold=1.4589954018592834
node_76: feature_name=AHR	AHR.value <= threshold=3.870481848716736
node_77: feature_name=IGJ	IGJ.value <= threshold=4.902882814407349
node_78: feature_name=CD79B	CD79B.value <= threshold=4.87716269493103
node_79: feature_name=HLA-DQB1	HLA-DQB1.value <= threshold=3.6925491094589233

node_80: feature_name=HLA-DRB5	HLA-DRB5.value <= threshold=2.0926843881607056
node_81: feature_name=MCTP1	MCTP1.value <= threshold=4.010472774505615
node_82: feature_name=GNG11	GNG11.value <= threshold=3.627470850944519
node_83: feature_name=HSP90B1	HSP90B1.value <= threshold=2.0835131406784058
node_84: feature_name=ATF3	ATF3.value <= threshold=1.1142374873161316
node_85: feature_name=MT-ND6	MT-ND6.value <= threshold=2.7328014373779297
node_86: feature_name=COTL1	COTL1.value > threshold=-1.6524587869644165
node_90: feature_name=MT-CYB	MT-CYB.value <= threshold=1.099629819393158
node_91: feature_name=RGCC	RGCC.value <= threshold=1.6908138990402222
node_92: feature_name=ILK	ILK.value <= threshold=3.9497616291046143
node_93: feature_name=CTD-2035E11.3	CTD-2035E11.3.value <= threshold=2.4543306827545166
node_94: feature_name=S100A6	S100A6.value <= threshold=0.2867250442504883
node_95: feature_name=HLA-DRB5	HLA-DRB5.value > threshold=-0.9496813416481018
node_99: feature_name=MT-CO3	MT-CO3.value > threshold=-1.55654776096344
node_103: feature_name=ITGAX	ITGAX.value <= threshold=1.8544010519981384
node_104: feature_name=MEF2C	MEF2C.value <= threshold=4.22724461555481
node_105: feature_name=CXorf57	CXorf57.value <= threshold=5.114444732666016
node_106: feature_name=IRAK3	IRAK3.value <= threshold=3.1017903089523315
node_107: feature_name=DUSP4	DUSP4.value <= threshold=2.9122174978256226
node_108: feature_name=OAZ1	OAZ1.value <= threshold=-1.8057305812835693
node_109: feature_name=MEF2C	MEF2C.value <= threshold=2.702623724937439
Class: COVID-19 Critical	
Rules_245	passed counts:19
node_0: feature_name=LTB	LTB.value > threshold=0.994031548500061
node_5264: feature_name=MT-CO1	MT-CO1.value > threshold=-0.4127991199493408
node_5638: feature_name=OAZ1	OAZ1.value > threshold=-0.2640867084264755
node_6112: feature_name=IFI44L	IFI44L.value <= threshold=2.5893263816833496
node_6113: feature_name=TCL1A	TCL1A.value <= threshold=4.201832294464111
node_6114: feature_name=HLA-DQA2	HLA-DQA2.value > threshold=-0.2270243540406227

node_6542: feature_name=CD79B	CD79B.value <= threshold=3.096232771873474
node_6543: feature_name=NR4A1	NR4A1.value <= threshold=1.421015739440918
node_6544: feature_name=LTB	LTB.value > threshold=1.359755277633667
node_6720: feature_name=NFKBIZ	NFKBIZ.value > threshold=0.2720414251089096
node_6794: feature_name=PELI1	PELI1.value <= threshold=1.5861127376556396
node_6795: feature_name=LTB	LTB.value > threshold=1.5073508024215698
node_6801: feature_name=IGJ	IGJ.value > threshold=-0.3395490199327469
node_6803: feature_name=GPX1	GPX1.value <= threshold=0.7495340406894684
Class: Healthy Control	
Rules_246	passed counts:19
node_0: feature_name=LTB	LTB.value > threshold=0.994031548500061
node_5264: feature_name=MT-CO1	MT-CO1.value > threshold=-0.4127991199493408
node_5638: feature_name=OAZ1	OAZ1.value > threshold=-0.2640867084264755
node_6112: feature_name=IFI44L	IFI44L.value <= threshold=2.5893263816833496
node_6113: feature_name=TCL1A	TCL1A.value <= threshold=4.201832294464111
node_6114: feature_name=HLA-DQA2	HLA-DQA2.value <= threshold=-0.2270243540406227
node_6115: feature_name=MTRNR2L2	MTRNR2L2.value <= threshold=2.548917293548584
node_6116: feature_name=ATF5	ATF5.value <= threshold=2.041263461112976
node_6117: feature_name=JUN	JUN.value <= threshold=-0.616259902715683
node_6118: feature_name=NFKBID	NFKBID.value <= threshold=1.5264258980751038
node_6119: feature_name=MT-CO1	MT-CO1.value <= threshold=0.1564532369375229
node_6120: feature_name=HLA-DPA1	HLA-DPA1.value <= threshold=1.569844663143158
node_6121: feature_name=ADAM19	ADAM19.value <= threshold=0.856033593416214
Class: COVID-19 Critical	
Rules_247	passed counts:19
node_0: feature_name=LTB	LTB.value <= threshold=0.994031548500061
node_1: feature_name=MTRNR2L2	MTRNR2L2.value <= threshold=2.8784297704696655
node_2: feature_name=TCL1A	TCL1A.value <= threshold=4.798312664031982

node_3: feature_name=MT-CO1	MT-CO1.value > threshold=0.08947191759943962
node_2521: feature_name=LTB	LTB.value > threshold=0.6038667261600494
node_4173: feature_name=OAZ1	OAZ1.value > threshold=0.143283873796463
node_4615: feature_name=NR4A1	NR4A1.value > threshold=0.6621004343032837
node_4817: feature_name=HSP90B1	HSP90B1.value > threshold=1.1699737310409546
node_4895: feature_name=MS4A1	MS4A1.value <= threshold=2.627432107925415
node_4896: feature_name=FTH1	FTH1.value <= threshold=0.49300217628479004
Class: COVID-19 Critical	
Rules_248	passed counts:19
node_0: feature_name=LTB	LTB.value <= threshold=0.994031548500061
node_1: feature_name=MTRNR2L2	MTRNR2L2.value <= threshold=2.8784297704696655
node_2: feature_name=TCL1A	TCL1A.value <= threshold=4.798312664031982
node_3: feature_name=MT-CO1	MT-CO1.value > threshold=0.08947191759943962
node_2521: feature_name=LTB	LTB.value > threshold=0.6038667261600494
node_4173: feature_name=OAZ1	OAZ1.value <= threshold=0.143283873796463
node_4174: feature_name=PLD4	PLD4.value <= threshold=2.921932101249695
node_4175: feature_name=XIST	XIST.value > threshold=-0.05067425221204758
node_4539: feature_name=HLA-DQB1	HLA-DQB1.value <= threshold=2.1246572732925415
node_4540: feature_name=ICAM1	ICAM1.value <= threshold=-0.22761741280555725
node_4541: feature_name=MARCH1	MARCH1.value > threshold=-0.3849789649248123
Class: Healthy Control	
Rules_249	passed counts:19
node_0: feature_name=LTB	LTB.value <= threshold=0.994031548500061
node_1: feature_name=MTRNR2L2	MTRNR2L2.value <= threshold=2.8784297704696655
node_2: feature_name=TCL1A	TCL1A.value <= threshold=4.798312664031982
node_3: feature_name=MT-CO1	MT-CO1.value > threshold=0.08947191759943962
node_2521: feature_name=LTB	LTB.value <= threshold=0.6038667261600494
node_2522: feature_name=HLA-DQB1	HLA-DQB1.value > threshold=2.1053054332733154

node_3550: feature_name=XIST	XIST.value <= threshold=0.7716337237507105
node_3551: feature_name=CD79B	CD79B.value <= threshold=2.9426796436309814
node_3552: feature_name=HLA-DQB1	HLA-DQB1.value <= threshold=2.713674306869507
node_3553: feature_name=SNX9	SNX9.value <= threshold=-0.5404067635536194
node_3554: feature_name=VPREB3	VPREB3.value <= threshold=4.4475414752960205
node_3555: feature_name=RALGPS2	RALGPS2.value > threshold=2.7017585039138794
node_3609: feature_name=MX1	MX1.value <= threshold=1.5204657316207886
node_3610: feature_name=FAM129C	FAM129C.value <= threshold=-0.30572274327278137
node_3611: feature_name=LDLRAD4	LDLRAD4.value <= threshold=-0.26634007692337036
node_3612: feature_name=S100A6	S100A6.value > threshold=-1.4843817353248596
node_3614: feature_name=MTRNR2L8	MTRNR2L8.value <= threshold=3.7056519389152527
node_3615: feature_name=AREGB	AREGB.value <= threshold=3.793744370341301
Class: COVID-19 Critical	
Rules_250	passed counts:19
node_0: feature_name=LTB	LTB.value <= threshold=0.994031548500061
node_1: feature_name=MTRNR2L2	MTRNR2L2.value <= threshold=2.8784297704696655
node_2: feature_name=TCL1A	TCL1A.value <= threshold=4.798312664031982
node_3: feature_name=MT-CO1	MT-CO1.value > threshold=0.08947191759943962
node_2521: feature_name=LTB	LTB.value <= threshold=0.6038667261600494
node_2522: feature_name=HLA-DQB1	HLA-DQB1.value <= threshold=2.1053054332733154
node_2523: feature_name=SNX9	SNX9.value <= threshold=-0.5346486568450928
node_2524: feature_name=HLA-DPB1	HLA-DPB1.value > threshold=1.9548270106315613
node_2814: feature_name=CD79B	CD79B.value > threshold=-0.28186772763729095
node_2826: feature_name=MT-ND6	MT-ND6.value > threshold=0.17273088544607162
node_2872: feature_name=IFI44L	IFI44L.value <= threshold=-0.31519079208374023
node_2873: feature_name=RALGPS2	RALGPS2.value <= threshold=1.6798744797706604
node_2874: feature_name=MARCH1	MARCH1.value <= threshold=1.8208032846450806
node_2875: feature_name=MT-ND6	MT-ND6.value <= threshold=2.5143303871154785
node_2876: feature_name=RELT	RELT.value <= threshold=-0.231830894947052

Class: COVID-19 Critical	
Rules_251	passed counts:19
node_0: feature_name=LTB	LTB.value <= threshold=0.994031548500061
node_1: feature_name=MTRNR2L2	MTRNR2L2.value <= threshold=2.8784297704696655
node_2: feature_name=TCL1A	TCL1A.value <= threshold=4.798312664031982
node_3: feature_name=MT-CO1	MT-CO1.value <= threshold=0.08947191759943962
node_4: feature_name=NR4A1	NR4A1.value > threshold=1.0725348591804504
node_1622: feature_name=HLA-DQA2	HLA-DQA2.value <= threshold=1.9496816992759705
node_1623: feature_name=HLA-DRB5	HLA-DRB5.value <= threshold=2.715766429901123
node_1624: feature_name=HLA-DPA1	HLA-DPA1.value > threshold=1.7050301432609558
node_2056: feature_name=OAZ1	OAZ1.value <= threshold=0.456103190779686
node_2057: feature_name=HLA-DRB5	HLA-DRB5.value > threshold=0.6839984953403473
node_2101: feature_name=HLA-DRB1	HLA-DRB1.value > threshold=1.6199676990509033
node_2151: feature_name=HLA-DQB1	HLA-DQB1.value > threshold=3.1961567401885986
node_2257: feature_name=XAF1	XAF1.value <= threshold=2.217243790626526
node_2258: feature_name=MT-CO1	MT-CO1.value > threshold=-1.0810123085975647
node_2260: feature_name=FCRL1	FCRL1.value <= threshold=5.213473558425903
Class: COVID-19 Severe	
Rules_252	passed counts:19
node_0: feature_name=LTB	LTB.value <= threshold=0.994031548500061
node_1: feature_name=MTRNR2L2	MTRNR2L2.value <= threshold=2.8784297704696655
node_2: feature_name=TCL1A	TCL1A.value <= threshold=4.798312664031982
node_3: feature_name=MT-CO1	MT-CO1.value <= threshold=0.08947191759943962
node_4: feature_name=NR4A1	NR4A1.value <= threshold=1.0725348591804504
node_5: feature_name=HLA-DPA1	HLA-DPA1.value > threshold=1.6885469555854797
node_861: feature_name=CD83	CD83.value <= threshold=3.0563642978668213
node_862: feature_name=HLA-DQB1	HLA-DQB1.value > threshold=2.61081063747406
node_1358: feature_name=HLA-DRB5	HLA-DRB5.value > threshold=0.7542744874954224

node_1390: feature_name=HLA-DRB5	HLA-DRB5.value > threshold=2.6244447231292725
node_1516: feature_name=HLA-DQA2	HLA-DQA2.value > threshold=-0.18513725697994232
node_1528: feature_name=IRF8	IRF8.value > threshold=-0.35322973132133484
node_1530: feature_name=ID1	ID1.value <= threshold=0.9785051867365837
node_1531: feature_name=LTB	LTB.value <= threshold=0.7293897569179535
node_1532: feature_name=ITM2C	ITM2C.value <= threshold=3.2393689155578613
Class: COVID-19 Critical	
Rules_253	passed counts:18
node_0: feature_name=LTB	LTB.value > threshold=0.994031548500061
node_5264: feature_name=MT-CO1	MT-CO1.value <= threshold=-0.4127991199493408
node_5265: feature_name=MTRNR2L2	MTRNR2L2.value <= threshold=2.4473838806152344
node_5266: feature_name=TCL1A	TCL1A.value <= threshold=5.787256956100464
node_5267: feature_name=PLD4	PLD4.value <= threshold=3.544657826423645
node_5268: feature_name=NR4A1	NR4A1.value > threshold=0.8490704298019409
node_5498: feature_name=HLA-DPA1	HLA-DPA1.value > threshold=1.9079211950302124
node_5556: feature_name=CD83	CD83.value > threshold=1.6135562062263489
node_5560: feature_name=OAZ1	OAZ1.value > threshold=0.11598343029618263
node_5568: feature_name=MT-CO1	MT-CO1.value > threshold=-0.8431997299194336
node_5574: feature_name=NFKBID	NFKBID.value <= threshold=3.9016520977020264
Class: Healthy Control	
Rules_254	passed counts:18
node_0: feature_name=LTB	LTB.value <= threshold=0.994031548500061
node_1: feature_name=MTRNR2L2	MTRNR2L2.value <= threshold=2.8784297704696655
node_2: feature_name=TCL1A	TCL1A.value > threshold=4.798312664031982
node_4912: feature_name=MT-CO1	MT-CO1.value > threshold=-0.3664844036102295
node_5002: feature_name=IFI44L	IFI44L.value <= threshold=2.3101747035980225
node_5003: feature_name=SNX9	SNX9.value > threshold=2.0247421264648438
node_5147: feature_name=CD79A	CD79A.value > threshold=2.554050326347351

node_5169: feature_name=CD79B	CD79B.value <= threshold=-0.2480710819363594
node_5170: feature_name=HLA-DQB1	HLA-DQB1.value <= threshold=2.405891537666321
node_5171: feature_name=RALGPS2	RALGPS2.value <= threshold=3.8303792476654053
node_5172: feature_name=RGS1	RGS1.value > threshold=-0.5179850161075592
Class: COVID-19 Critical	
Rules_255	passed counts:18
node_0: feature_name=LTB	LTB.value <= threshold=0.994031548500061
node_1: feature_name=MTRNR2L2	MTRNR2L2.value <= threshold=2.8784297704696655
node_2: feature_name=TCL1A	TCL1A.value > threshold=4.798312664031982
node_4912: feature_name=MT-CO1	MT-CO1.value > threshold=-0.3664844036102295
node_5002: feature_name=IFI44L	IFI44L.value <= threshold=2.3101747035980225
node_5003: feature_name=SNX9	SNX9.value <= threshold=2.0247421264648438
node_5004: feature_name=STAG3	STAG3.value <= threshold=6.482141733169556
node_5005: feature_name=BACH1	BACH1.value > threshold=-0.5237349569797516
node_5047: feature_name=RHOB	RHOB.value > threshold=-0.4843696355819702
node_5055: feature_name=FAM129C	FAM129C.value <= threshold=-0.2832885980606079
node_5056: feature_name=CD83	CD83.value <= threshold=-0.5592367649078369
node_5057: feature_name=RNF144B	RNF144B.value <= threshold=3.1223526000976562
Class: Healthy Control	
Rules_256	passed counts:18
node_0: feature_name=LTB	LTB.value <= threshold=0.994031548500061
node_1: feature_name=MTRNR2L2	MTRNR2L2.value <= threshold=2.8784297704696655
node_2: feature_name=TCL1A	TCL1A.value > threshold=4.798312664031982
node_4912: feature_name=MT-CO1	MT-CO1.value <= threshold=-0.3664844036102295
node_4913: feature_name=CD79A	CD79A.value <= threshold=2.987208843231201
node_4914: feature_name=HLA-DPB1	HLA-DPB1.value <= threshold=2.0926655530929565
node_4915: feature_name=MX1	MX1.value > threshold=-0.4660506546497345
node_4921: feature_name=NR4A1	NR4A1.value > threshold=1.2700347900390625

node_4949: feature_name=LTB	LTB.value <= threshold=0.7105090916156769
node_4950: feature_name=TNF	TNF.value <= threshold=1.568493664264679
node_4951: feature_name=HSP90B1	HSP90B1.value > threshold=-1.188246250152588
Class: COVID-19 Critical	
Rules_257	passed counts:18
node_0: feature_name=LTB	LTB.value <= threshold=0.994031548500061
node_1: feature_name=MTRNR2L2	MTRNR2L2.value <= threshold=2.8784297704696655
node_2: feature_name=TCL1A	TCL1A.value <= threshold=4.798312664031982
node_3: feature_name=MT-CO1	MT-CO1.value > threshold=0.08947191759943962
node_2521: feature_name=LTB	LTB.value <= threshold=0.6038667261600494
node_2522: feature_name=HLA-DQB1	HLA-DQB1.value > threshold=2.1053054332733154
node_3550: feature_name=XIST	XIST.value <= threshold=0.7716337237507105
node_3551: feature_name=CD79B	CD79B.value > threshold=2.9426796436309814
node_4039: feature_name=IFI44L	IFI44L.value <= threshold=1.8637521862983704
node_4040: feature_name=SNX9	SNX9.value > threshold=1.274232804775238
node_4096: feature_name=RALGPS2	RALGPS2.value > threshold=2.3599878549575806
node_4118: feature_name=SNX29P2	SNX29P2.value <= threshold=0.880477637052536
node_4119: feature_name=FAM129C	FAM129C.value <= threshold=3.415250778198242
node_4120: feature_name=FTH1	FTH1.value > threshold=-1.350711464881897
node_4122: feature_name=OAZ1	OAZ1.value <= threshold=1.1596062183380127
Class: COVID-19 Critical	
Rules_258	passed counts:18
node_0: feature_name=LTB	LTB.value <= threshold=0.994031548500061
node_1: feature_name=MTRNR2L2	MTRNR2L2.value <= threshold=2.8784297704696655
node_2: feature_name=TCL1A	TCL1A.value <= threshold=4.798312664031982
node_3: feature_name=MT-CO1	MT-CO1.value > threshold=0.08947191759943962
node_2521: feature_name=LTB	LTB.value <= threshold=0.6038667261600494
node_2522: feature_name=HLA-DQB1	HLA-DQB1.value > threshold=2.1053054332733154

node_3550: feature_name=XIST	XIST.value <= threshold=0.7716337237507105
node_3551: feature_name=CD79B	CD79B.value <= threshold=2.9426796436309814
node_3552: feature_name=HLA-DQB1	HLA-DQB1.value <= threshold=2.713674306869507
node_3553: feature_name=SNX9	SNX9.value > threshold=-0.5404067635536194
node_3653: feature_name=LTB	LTB.value > threshold=0.021708179265260696
node_3759: feature_name=TCL1A	TCL1A.value <= threshold=3.3921085596084595
node_3760: feature_name=HLA-DRB5	HLA-DRB5.value <= threshold=0.5265218019485474
node_3761: feature_name=ATF5	ATF5.value > threshold=-0.32008348405361176
node_3769: feature_name=CD83	CD83.value <= threshold=3.0822256803512573
node_3770: feature_name=RGS1	RGS1.value <= threshold=1.4936688542366028
Class: COVID-19 Critical	
Rules_259	passed counts:18
node_0: feature_name=LTB	LTB.value <= threshold=0.994031548500061
node_1: feature_name=MTRNR2L2	MTRNR2L2.value <= threshold=2.8784297704696655
node_2: feature_name=TCL1A	TCL1A.value <= threshold=4.798312664031982
node_3: feature_name=MT-CO1	MT-CO1.value <= threshold=0.08947191759943962
node_4: feature_name=NR4A1	NR4A1.value <= threshold=1.0725348591804504
node_5: feature_name=HLA-DPA1	HLA-DPA1.value > threshold=1.6885469555854797
node_861: feature_name=CD83	CD83.value <= threshold=3.0563642978668213
node_862: feature_name=HLA-DQB1	HLA-DQB1.value > threshold=2.61081063747406
node_1358: feature_name=HLA-DRB5	HLA-DRB5.value > threshold=0.7542744874954224
node_1390: feature_name=HLA-DRB5	HLA-DRB5.value <= threshold=2.6244447231292725
node_1391: feature_name=XIST	XIST.value <= threshold=-0.22017984092235565
node_1392: feature_name=MT-CO3	MT-CO3.value <= threshold=0.12647542357444763
node_1393: feature_name=CD79B	CD79B.value <= threshold=4.338301658630371
node_1394: feature_name=LTB	LTB.value <= threshold=0.9865012466907501
node_1395: feature_name=MEF2C	MEF2C.value <= threshold=4.133931398391724
node_1396: feature_name=MEF2C	MEF2C.value <= threshold=-0.38076959550380707
node_1397: feature_name=UTY	UTY.value <= threshold=-0.4634757786989212

node_1398: feature_name=MT-CO1	MT-CO1.value <= threshold=-0.8070613443851471
node_1399: feature_name=RNF144B	RNF144B.value > threshold=-0.28872863948345184
Class: COVID-19 Critical	
Rules_260	passed counts:18
node_0: feature_name=LTB	LTB.value <= threshold=0.994031548500061
node_1: feature_name=MTRNR2L2	MTRNR2L2.value <= threshold=2.8784297704696655
node_2: feature_name=TCL1A	TCL1A.value <= threshold=4.798312664031982
node_3: feature_name=MT-CO1	MT-CO1.value <= threshold=0.08947191759943962
node_4: feature_name=NR4A1	NR4A1.value <= threshold=1.0725348591804504
node_5: feature_name=HLA-DPA1	HLA-DPA1.value <= threshold=1.6885469555854797
node_6: feature_name=HLA-DRB5	HLA-DRB5.value <= threshold=2.1886194944381714
node_7: feature_name=XIST	XIST.value <= threshold=-0.12328458577394485
node_8: feature_name=SYNE1	SYNE1.value > threshold=-0.7835594415664673
node_16: feature_name=MTRNR2L2	MTRNR2L2.value <= threshold=2.4483247995376587
node_17: feature_name=HLA-DQA2	HLA-DQA2.value <= threshold=2.227178931236267
node_18: feature_name=NR4A1	NR4A1.value <= threshold=-0.21380723267793655
node_19: feature_name=IRAK3	IRAK3.value <= threshold=4.27159571647644
node_20: feature_name=IFIT2	IFIT2.value <= threshold=7.8175740242004395
node_21: feature_name=CD79A	CD79A.value > threshold=2.857242226600647
node_375: feature_name=LTB	LTB.value <= threshold=0.7405790388584137
node_376: feature_name=NFKBIZ	NFKBIZ.value <= threshold=1.7718515396118164
node_377: feature_name=OAZ1	OAZ1.value <= threshold=1.3302325010299683
node_378: feature_name=RHOB	RHOB.value > threshold=3.0459729433059692
node_456: feature_name=CD83	CD83.value <= threshold=2.2091671228408813
node_457: feature_name=ITM2C	ITM2C.value > threshold=-0.2793172299861908
Class: COVID-19 Critical	
Rules_261	passed counts:17
node_0: feature_name=LTB	LTB.value > threshold=0.994031548500061

node_5264: feature_name=MT-CO1	MT-CO1.value > threshold=-0.4127991199493408
node_5638: feature_name=OAZ1	OAZ1.value > threshold=-0.2640867084264755
node_6112: feature_name=IFI44L	IFI44L.value <= threshold=2.5893263816833496
node_6113: feature_name=TCL1A	TCL1A.value > threshold=4.201832294464111
node_6991: feature_name=XAF1	XAF1.value <= threshold=3.4585667848587036
node_6992: feature_name=STAG3	STAG3.value <= threshold=7.686291694641113
node_6993: feature_name=RALGPS2	RALGPS2.value > threshold=-0.4090157449245453
node_6997: feature_name=MTRNR2L2	MTRNR2L2.value <= threshold=3.23723828792572
node_6998: feature_name=TP53INP1	TP53INP1.value <= threshold=6.239925146102905
node_6999: feature_name=MEF2C	MEF2C.value > threshold=-0.44642284512519836
node_7003: feature_name=HSPA1A	HSPA1A.value <= threshold=7.404359817504883
node_7004: feature_name=COTL1	COTL1.value > threshold=-1.6598749160766602
node_7006: feature_name=HIST1H2AC	HIST1H2AC.value > threshold=-0.34910088777542114
node_7008: feature_name=MARCH1	MARCH1.value <= threshold=5.393903970718384
node_7009: feature_name=PELI1	PELI1.value <= threshold=3.565749168395996
node_7010: feature_name=RGS1	RGS1.value <= threshold=2.851950168609619
node_7011: feature_name=HLA-DRB5	HLA-DRB5.value <= threshold=3.0528379678726196
node_7012: feature_name=MEF2A	MEF2A.value <= threshold=3.228689193725586
node_7013: feature_name=MT-CO1	MT-CO1.value <= threshold=-0.37944722175598145
node_7014: feature_name=STAG3	STAG3.value <= threshold=1.8744874000549316
Class: Healthy Control	
Rules_262	passed counts:17
node_0: feature_name=LTB	LTB.value <= threshold=0.994031548500061
node_1: feature_name=MTRNR2L2	MTRNR2L2.value <= threshold=2.8784297704696655
node_2: feature_name=TCL1A	TCL1A.value > threshold=4.798312664031982
node_4912: feature_name=MT-CO1	MT-CO1.value > threshold=-0.3664844036102295
node_5002: feature_name=IFI44L	IFI44L.value <= threshold=2.3101747035980225
node_5003: feature_name=SNX9	SNX9.value <= threshold=2.0247421264648438
node_5004: feature_name=STAG3	STAG3.value > threshold=6.482141733169556

node_5138: feature_name=CCDC141	CCDC141.value <= threshold=-0.2267386093735695
node_5139: feature_name=RGCC	RGCC.value <= threshold=-0.7110134959220886
Class: COVID-19 Critical	
Rules_263	passed counts:17
node_0: feature_name=LTB	LTB.value <= threshold=0.994031548500061
node_1: feature_name=MTRNR2L2	MTRNR2L2.value <= threshold=2.8784297704696655
node_2: feature_name=TCL1A	TCL1A.value > threshold=4.798312664031982
node_4912: feature_name=MT-CO1	MT-CO1.value <= threshold=-0.3664844036102295
node_4913: feature_name=CD79A	CD79A.value <= threshold=2.987208843231201
node_4914: feature_name=HLA-DPB1	HLA-DPB1.value > threshold=2.0926655530929565
node_4958: feature_name=KIAA0125	KIAA0125.value <= threshold=-0.15961305052042007
node_4959: feature_name=PDLIM1	PDLIM1.value <= threshold=3.826790690422058
node_4960: feature_name=MS4A1	MS4A1.value <= threshold=3.352075457572937
node_4961: feature_name=MT-CO3	MT-CO3.value <= threshold=-0.053408265113830566
Class: COVID-19 Critical	
Rules_264	passed counts:17
node_0: feature_name=LTB	LTB.value <= threshold=0.994031548500061
node_1: feature_name=MTRNR2L2	MTRNR2L2.value <= threshold=2.8784297704696655
node_2: feature_name=TCL1A	TCL1A.value <= threshold=4.798312664031982
node_3: feature_name=MT-CO1	MT-CO1.value > threshold=0.08947191759943962
node_2521: feature_name=LTB	LTB.value > threshold=0.6038667261600494
node_4173: feature_name=OAZ1	OAZ1.value <= threshold=0.143283873796463
node_4174: feature_name=PLD4	PLD4.value <= threshold=2.921932101249695
node_4175: feature_name=XIST	XIST.value <= threshold=-0.05067425221204758
node_4176: feature_name=MT-CO1	MT-CO1.value > threshold=1.345594048500061
node_4486: feature_name=RALGPS2	RALGPS2.value > threshold=2.7596691846847534
node_4508: feature_name=SNX9	SNX9.value > threshold=2.5712826251983643
node_4526: feature_name=APP	APP.value <= threshold=-0.2580544352531433

node_4527: feature_name=TNFAIP3	TNFAIP3.value <= threshold=1.1829917430877686
node_4528: feature_name=RGCC	RGCC.value <= threshold=1.4720749258995056
Class: COVID-19 Critical	
Rules_265	passed counts:17
node_0: feature_name=LTB	LTB.value <= threshold=0.994031548500061
node_1: feature_name=MTRNR2L2	MTRNR2L2.value <= threshold=2.8784297704696655
node_2: feature_name=TCL1A	TCL1A.value <= threshold=4.798312664031982
node_3: feature_name=MT-CO1	MT-CO1.value > threshold=0.08947191759943962
node_2521: feature_name=LTB	LTB.value > threshold=0.6038667261600494
node_4173: feature_name=OAZ1	OAZ1.value <= threshold=0.143283873796463
node_4174: feature_name=PLD4	PLD4.value <= threshold=2.921932101249695
node_4175: feature_name=XIST	XIST.value <= threshold=-0.05067425221204758
node_4176: feature_name=MT-CO1	MT-CO1.value <= threshold=1.345594048500061
node_4177: feature_name=HLA-DQB1	HLA-DQB1.value > threshold=1.927736520767212
node_4353: feature_name=ITM2C	ITM2C.value <= threshold=-0.12329206243157387
node_4354: feature_name=MX1	MX1.value <= threshold=-0.4356398284435272
node_4355: feature_name=HLA-DQB1	HLA-DQB1.value > threshold=2.8865538835525513
node_4419: feature_name=DAPP1	DAPP1.value <= threshold=-0.30035753548145294
node_4420: feature_name=RHOB	RHOB.value <= threshold=1.2027948498725891
Class: COVID-19 Severe	
Rules_266	passed counts:17
node_0: feature_name=LTB	LTB.value <= threshold=0.994031548500061
node_1: feature_name=MTRNR2L2	MTRNR2L2.value <= threshold=2.8784297704696655
node_2: feature_name=TCL1A	TCL1A.value <= threshold=4.798312664031982
node_3: feature_name=MT-CO1	MT-CO1.value > threshold=0.08947191759943962
node_2521: feature_name=LTB	LTB.value <= threshold=0.6038667261600494
node_2522: feature_name=HLA-DQB1	HLA-DQB1.value > threshold=2.1053054332733154
node_3550: feature_name=XIST	XIST.value <= threshold=0.7716337237507105

node_3551: feature_name=CD79B	CD79B.value <= threshold=2.9426796436309814
node_3552: feature_name=HLA-DQB1	HLA-DQB1.value > threshold=2.713674306869507
node_3854: feature_name=MEF2C	MEF2C.value > threshold=-0.3721291869878769
node_3882: feature_name=IFI44L	IFI44L.value <= threshold=2.5516022443771362
node_3883: feature_name=FCER2	FCER2.value <= threshold=-0.21487552672624588
node_3884: feature_name=HLA-DQA2	HLA-DQA2.value <= threshold=-0.12580305337905884
node_3885: feature_name=SLC7A5	SLC7A5.value <= threshold=-0.4906871169805527
node_3886: feature_name=VPREB3	VPREB3.value <= threshold=5.492124557495117
node_3887: feature_name=CYBB	CYBB.value <= threshold=2.720046043395996
node_3888: feature_name=CD79A	CD79A.value <= threshold=3.3531476259231567
node_3889: feature_name=HLA-DPA1	HLA-DPA1.value > threshold=1.6232598423957825
node_3911: feature_name=HLA-DQA1	HLA-DQA1.value <= threshold=3.2045756578445435
node_3912: feature_name=GPX1	GPX1.value <= threshold=0.37351667881011963
node_3913: feature_name=IGLL5	IGLL5.value <= threshold=3.4207619428634644
node_3914: feature_name=ICAM1	ICAM1.value <= threshold=3.929016351699829
Class: COVID-19 Severe	
Rules_267	passed counts:17
node_0: feature_name=LTB	LTB.value <= threshold=0.994031548500061
node_1: feature_name=MTRNR2L2	MTRNR2L2.value <= threshold=2.8784297704696655
node_2: feature_name=TCL1A	TCL1A.value <= threshold=4.798312664031982
node_3: feature_name=MT-CO1	MT-CO1.value <= threshold=0.08947191759943962
node_4: feature_name=NR4A1	NR4A1.value > threshold=1.0725348591804504
node_1622: feature_name=HLA-DQA2	HLA-DQA2.value > threshold=1.9496816992759705
node_2350: feature_name=BCL2A1	BCL2A1.value > threshold=1.1322240233421326
node_2466: feature_name=MS4A1	MS4A1.value > threshold=2.7321707010269165
node_2510: feature_name=RHOB	RHOB.value > threshold=-0.35927484929561615
node_2516: feature_name=COTL1	COTL1.value > threshold=-1.2514774203300476
node_2518: feature_name=MT-CO1	MT-CO1.value > threshold=-1.744008183479309
Class: COVID-19 Critical	

Rules_268	passed counts:17
node_0: feature_name=LTB	LTB.value <= threshold=0.994031548500061
node_1: feature_name=MTRNR2L2	MTRNR2L2.value <= threshold=2.8784297704696655
node_2: feature_name=TCL1A	TCL1A.value <= threshold=4.798312664031982
node_3: feature_name=MT-CO1	MT-CO1.value <= threshold=0.08947191759943962
node_4: feature_name=NR4A1	NR4A1.value > threshold=1.0725348591804504
node_1622: feature_name=HLA-DQA2	HLA-DQA2.value <= threshold=1.9496816992759705
node_1623: feature_name=HLA-DRB5	HLA-DRB5.value <= threshold=2.715766429901123
node_1624: feature_name=HLA-DPA1	HLA-DPA1.value <= threshold=1.7050301432609558
node_1625: feature_name=CD83	CD83.value > threshold=2.783489227294922
node_1873: feature_name=IFI44L	IFI44L.value <= threshold=1.5938722491264343
node_1874: feature_name=PLD4	PLD4.value <= threshold=4.101001739501953
node_1875: feature_name=RHOB	RHOB.value > threshold=1.304835319519043
node_1965: feature_name=HLA-DQB1	HLA-DQB1.value <= threshold=1.5989137291908264
node_1966: feature_name=CD79A	CD79A.value > threshold=2.189441442489624
node_1974: feature_name=IGJ	IGJ.value <= threshold=1.4252705574035645
node_1975: feature_name=PRDM1	PRDM1.value <= threshold=-0.4423440992832184
node_1976: feature_name=CCR6	CCR6.value <= threshold=0.9063289687037468
node_1977: feature_name=GRASP	GRASP.value > threshold=-0.39720863103866577
Class: COVID-19 Critical	
Rules_269	passed counts:17
node_0: feature_name=LTB	LTB.value <= threshold=0.994031548500061
node_1: feature_name=MTRNR2L2	MTRNR2L2.value <= threshold=2.8784297704696655
node_2: feature_name=TCL1A	TCL1A.value <= threshold=4.798312664031982
node_3: feature_name=MT-CO1	MT-CO1.value <= threshold=0.08947191759943962
node_4: feature_name=NR4A1	NR4A1.value <= threshold=1.0725348591804504
node_5: feature_name=HLA-DPA1	HLA-DPA1.value > threshold=1.6885469555854797
node_861: feature_name=CD83	CD83.value <= threshold=3.0563642978668213

node_862: feature_name=HLA-DQB1	HLA-DQB1.value <= threshold=2.61081063747406
node_863: feature_name=MTRNR2L2	MTRNR2L2.value <= threshold=2.0919997692108154
node_864: feature_name=BCL2A1	BCL2A1.value <= threshold=1.852618932723999
node_865: feature_name=CD79B	CD79B.value <= threshold=4.352142333984375
node_866: feature_name=TNFAIP3	TNFAIP3.value <= threshold=-0.0449496041983366
node_867: feature_name=MT-CO1	MT-CO1.value <= threshold=-0.08531957119703293
node_868: feature_name=MTRNR2L8	MTRNR2L8.value <= threshold=0.8770350813865662
node_869: feature_name=EGR1	EGR1.value > threshold=-0.32270750403404236
node_1015: feature_name=NR4A3	NR4A3.value <= threshold=1.9085431098937988
node_1016: feature_name=ID3	ID3.value <= threshold=2.776647686958313
node_1017: feature_name=XIST	XIST.value <= threshold=2.618046522140503
node_1018: feature_name=CD79B	CD79B.value <= threshold=3.818994164466858
node_1019: feature_name=NFKBID	NFKBID.value > threshold=3.4191306829452515
node_1111: feature_name=RBKS	RBKS.value <= threshold=-0.2493925839662552
node_1112: feature_name=CYBB	CYBB.value > threshold=-0.5100959241390228
node_1116: feature_name=CCDC141	CCDC141.value <= threshold=3.9731972217559814
Class: COVID-19 Critical	
Rules_270	passed counts:17
node_0: feature_name=LTB	LTB.value <= threshold=0.994031548500061
node_1: feature_name=MTRNR2L2	MTRNR2L2.value <= threshold=2.8784297704696655
node_2: feature_name=TCL1A	TCL1A.value <= threshold=4.798312664031982
node_3: feature_name=MT-CO1	MT-CO1.value <= threshold=0.08947191759943962
node_4: feature_name=NR4A1	NR4A1.value <= threshold=1.0725348591804504
node_5: feature_name=HLA-DPA1	HLA-DPA1.value > threshold=1.6885469555854797
node_861: feature_name=CD83	CD83.value <= threshold=3.0563642978668213
node_862: feature_name=HLA-DQB1	HLA-DQB1.value <= threshold=2.61081063747406
node_863: feature_name=MTRNR2L2	MTRNR2L2.value <= threshold=2.0919997692108154
node_864: feature_name=BCL2A1	BCL2A1.value <= threshold=1.852618932723999
node_865: feature_name=CD79B	CD79B.value <= threshold=4.352142333984375

node_866: feature_name=TNFAIP3	TNFAIP3.value <= threshold=-0.0449496041983366
node_867: feature_name=MT-CO1	MT-CO1.value <= threshold=-0.08531957119703293
node_868: feature_name=MTRNR2L8	MTRNR2L8.value <= threshold=0.8770350813865662
node_869: feature_name=EGR1	EGR1.value <= threshold=-0.32270750403404236
node_870: feature_name=IGJ	IGJ.value <= threshold=4.618318557739258
node_871: feature_name=DUSP4	DUSP4.value <= threshold=2.8197922706604004
node_872: feature_name=HOPX	HOPX.value <= threshold=2.10397732257843
node_873: feature_name=RGCC	RGCC.value <= threshold=1.2070720195770264
node_874: feature_name=OAZ1	OAZ1.value <= threshold=1.02116060256958
node_875: feature_name=NAIP	NAIP.value <= threshold=3.768904685974121
node_876: feature_name=LDLR	LDLR.value <= threshold=2.9582698345184326
node_877: feature_name=HLA-DPA1	HLA-DPA1.value <= threshold=3.0588693618774414
node_878: feature_name=RHOB	RHOB.value <= threshold=5.08755898475647
node_879: feature_name=SYTL3	SYTL3.value <= threshold=1.5127440690994263
node_880: feature_name=FTH1	FTH1.value > threshold=-1.4152275919914246
node_882: feature_name=CCL3	CCL3.value <= threshold=2.253131687641144
node_883: feature_name=CD83	CD83.value <= threshold=2.8566592931747437
node_884: feature_name=HLA-DRB5	HLA-DRB5.value > threshold=-0.6464254856109619
node_888: feature_name=HSP90B1	HSP90B1.value > threshold=-1.186358630657196
node_892: feature_name=ATG2A	ATG2A.value <= threshold=2.3624722957611084
node_893: feature_name=ICAM1	ICAM1.value <= threshold=2.4306468963623047
node_894: feature_name=CD38	CD38.value <= threshold=3.4977954626083374
node_895: feature_name=HLA-DRB1	HLA-DRB1.value > threshold=0.28603747487068176
node_899: feature_name=XIST	XIST.value > threshold=1.4272559583187103
node_947: feature_name=FCER2	FCER2.value <= threshold=5.149282455444336
node_948: feature_name=JUN	JUN.value <= threshold=1.3003323674201965
Class: COVID-19 Critical	
Rules_271	passed counts:17
node_0: feature_name=LTB	LTB.value <= threshold=0.994031548500061

node_1: feature_name=MTRNR2L2	MTRNR2L2.value <= threshold=2.8784297704696655
node_2: feature_name=TCL1A	TCL1A.value <= threshold=4.798312664031982
node_3: feature_name=MT-CO1	MT-CO1.value <= threshold=0.08947191759943962
node_4: feature_name=NR4A1	NR4A1.value <= threshold=1.0725348591804504
node_5: feature_name=HLA-DPA1	HLA-DPA1.value <= threshold=1.6885469555854797
node_6: feature_name=HLA-DRB5	HLA-DRB5.value <= threshold=2.1886194944381714
node_7: feature_name=XIST	XIST.value > threshold=-0.12328458577394485
node_711: feature_name=HLA-DQB1	HLA-DQB1.value > threshold=2.337415099143982
node_731: feature_name=OAZ1	OAZ1.value <= threshold=0.6314055621623993
node_732: feature_name=MTRNR2L8	MTRNR2L8.value <= threshold=-0.2771559953689575
node_733: feature_name=TLN1	TLN1.value <= threshold=1.6143433451652527
Class: COVID-19 Severe	
Rules_272	passed counts:17
node_0: feature_name=LTB	LTB.value <= threshold=0.994031548500061
node_1: feature_name=MTRNR2L2	MTRNR2L2.value <= threshold=2.8784297704696655
node_2: feature_name=TCL1A	TCL1A.value <= threshold=4.798312664031982
node_3: feature_name=MT-CO1	MT-CO1.value <= threshold=0.08947191759943962
node_4: feature_name=NR4A1	NR4A1.value <= threshold=1.0725348591804504
node_5: feature_name=HLA-DPA1	HLA-DPA1.value <= threshold=1.6885469555854797
node_6: feature_name=HLA-DRB5	HLA-DRB5.value <= threshold=2.1886194944381714
node_7: feature_name=XIST	XIST.value <= threshold=-0.12328458577394485
node_8: feature_name=SYNE1	SYNE1.value > threshold=-0.7835594415664673
node_16: feature_name=MTRNR2L2	MTRNR2L2.value <= threshold=2.4483247995376587
node_17: feature_name=HLA-DQA2	HLA-DQA2.value <= threshold=2.227178931236267
node_18: feature_name=NR4A1	NR4A1.value <= threshold=-0.21380723267793655
node_19: feature_name=IRAK3	IRAK3.value <= threshold=4.27159571647644
node_20: feature_name=IFIT2	IFIT2.value <= threshold=7.8175740242004395
node_21: feature_name=CD79A	CD79A.value <= threshold=2.857242226600647
node_22: feature_name=HIST1H2AC	HIST1H2AC.value <= threshold=5.077470302581787

node_23: feature_name=FTH1	FTH1.value > threshold=0.4158056080341339
node_285: feature_name=EGR1	EGR1.value <= threshold=4.240467071533203
node_286: feature_name=HLA-DRB1	HLA-DRB1.value > threshold=-0.7553382813930511
node_290: feature_name=TLR2	TLR2.value <= threshold=2.879140257835388
node_291: feature_name=SYNE1	SYNE1.value <= threshold=2.323199510574341
node_292: feature_name=BCL2A1	BCL2A1.value <= threshold=1.8940600156784058
node_293: feature_name=RALGPS2	RALGPS2.value <= threshold=4.808189153671265
node_294: feature_name=IGLL5	IGLL5.value <= threshold=4.116017818450928
node_295: feature_name=FTH1	FTH1.value > threshold=0.41612209379673004
node_297: feature_name=MCTP1	MCTP1.value <= threshold=4.601342678070068
node_298: feature_name=MT-CO2	MT-CO2.value > threshold=0.6201252341270447
node_342: feature_name=TCF4	TCF4.value > threshold=-0.24748335778713226
Class: COVID-19 Critical	
Rules_273	passed counts:16
node_0: feature_name=LTB	LTB.value > threshold=0.994031548500061
node_5264: feature_name=MT-CO1	MT-CO1.value > threshold=-0.4127991199493408
node_5638: feature_name=OAZ1	OAZ1.value > threshold=-0.2640867084264755
node_6112: feature_name=IFI44L	IFI44L.value <= threshold=2.5893263816833496
node_6113: feature_name=TCL1A	TCL1A.value <= threshold=4.201832294464111
node_6114: feature_name=HLA-DQA2	HLA-DQA2.value > threshold=-0.2270243540406227
node_6542: feature_name=CD79B	CD79B.value <= threshold=3.096232771873474
node_6543: feature_name=NR4A1	NR4A1.value <= threshold=1.421015739440918
node_6544: feature_name=LTB	LTB.value <= threshold=1.359755277633667
node_6545: feature_name=NFKBID	NFKBID.value <= threshold=1.4294711351394653
node_6546: feature_name=HLA-DRB1	HLA-DRB1.value > threshold=1.0398632287979126
node_6556: feature_name=CD79B	CD79B.value > threshold=-0.2919560521841049
node_6582: feature_name=PELI1	PELI1.value > threshold=-0.6156984269618988
node_6602: feature_name=MT-CO3	MT-CO3.value <= threshold=0.46141014993190765
node_6603: feature_name=NFKBIZ	NFKBIZ.value <= threshold=0.6470938920974731

node_6604: feature_name=HSP90B1	HSP90B1.value > threshold=0.4268011748790741
node_6620: feature_name=MT-CO3	MT-CO3.value <= threshold=0.2716837227344513
node_6621: feature_name=KIAA0125	KIAA0125.value <= threshold=7.491191387176514
Class: COVID-19 Critical	
Rules_274	passed counts:16
node_0: feature_name=LTB	LTB.value > threshold=0.994031548500061
node_5264: feature_name=MT-CO1	MT-CO1.value > threshold=-0.4127991199493408
node_5638: feature_name=OAZ1	OAZ1.value > threshold=-0.2640867084264755
node_6112: feature_name=IFI44L	IFI44L.value <= threshold=2.5893263816833496
node_6113: feature_name=TCL1A	TCL1A.value <= threshold=4.201832294464111
node_6114: feature_name=HLA-DQA2	HLA-DQA2.value <= threshold=-0.2270243540406227
node_6115: feature_name=MTRNR2L2	MTRNR2L2.value <= threshold=2.548917293548584
node_6116: feature_name=ATF5	ATF5.value <= threshold=2.041263461112976
node_6117: feature_name=JUN	JUN.value > threshold=-0.616259902715683
node_6189: feature_name=MT-CO3	MT-CO3.value > threshold=0.13483521342277527
node_6351: feature_name=XAF1	XAF1.value <= threshold=3.061717987060547
node_6352: feature_name=MT-CO2	MT-CO2.value > threshold=1.2605181336402893
node_6476: feature_name=ATF5	ATF5.value <= threshold=-0.3569986820220947
node_6477: feature_name=DUSP4	DUSP4.value <= threshold=0.5886635780334473
Class: Healthy Control	
Rules_275	passed counts:16
node_0: feature_name=LTB	LTB.value > threshold=0.994031548500061
node_5264: feature_name=MT-CO1	MT-CO1.value > threshold=-0.4127991199493408
node_5638: feature_name=OAZ1	OAZ1.value <= threshold=-0.2640867084264755
node_5639: feature_name=CD79B	CD79B.value <= threshold=2.2998517751693726
node_5640: feature_name=MTRNR2L2	MTRNR2L2.value <= threshold=3.5499863624572754
node_5641: feature_name=MT-CO1	MT-CO1.value > threshold=0.22567133605480194
node_5729: feature_name=MARCH1	MARCH1.value <= threshold=1.5154643654823303

node_5730: feature_name=MT-CO1	MT-CO1.value <= threshold=1.7823500633239746
node_5731: feature_name=HLA-DPB1	HLA-DPB1.value <= threshold=2.0096163749694824
node_5732: feature_name=NR4A3	NR4A3.value <= threshold=3.509189486503601
node_5733: feature_name=OAZ1	OAZ1.value > threshold=-0.4392261207103729
node_5803: feature_name=BACH1	BACH1.value <= threshold=1.211863934993744
node_5804: feature_name=MEF2A	MEF2A.value <= threshold=0.8741590976715088
node_5805: feature_name=CYBB	CYBB.value <= threshold=2.0083687901496887
Class: Healthy Control	
Rules_276	passed counts:16
node_0: feature_name=LTB	LTB.value <= threshold=0.994031548500061
node_1: feature_name=MTRNR2L2	MTRNR2L2.value <= threshold=2.8784297704696655
node_2: feature_name=TCL1A	TCL1A.value <= threshold=4.798312664031982
node_3: feature_name=MT-CO1	MT-CO1.value > threshold=0.08947191759943962
node_2521: feature_name=LTB	LTB.value > threshold=0.6038667261600494
node_4173: feature_name=OAZ1	OAZ1.value <= threshold=0.143283873796463
node_4174: feature_name=PLD4	PLD4.value <= threshold=2.921932101249695
node_4175: feature_name=XIST	XIST.value <= threshold=-0.05067425221204758
node_4176: feature_name=MT-CO1	MT-CO1.value <= threshold=1.345594048500061
node_4177: feature_name=HLA-DQB1	HLA-DQB1.value > threshold=1.927736520767212
node_4353: feature_name=ITM2C	ITM2C.value <= threshold=-0.12329206243157387
node_4354: feature_name=MX1	MX1.value <= threshold=-0.4356398284435272
node_4355: feature_name=HLA-DQB1	HLA-DQB1.value <= threshold=2.8865538835525513
node_4356: feature_name=SNX9	SNX9.value > threshold=1.002076417207718
node_4384: feature_name=HLA-DRB5	HLA-DRB5.value <= threshold=1.0937854647636414
node_4385: feature_name=IFI6	IFI6.value <= threshold=0.09842458367347717
node_4386: feature_name=FCRL1	FCRL1.value <= threshold=3.549554228782654
node_4387: feature_name=HLA-DPB1	HLA-DPB1.value > threshold=1.561116874217987
node_4391: feature_name=ICAM1	ICAM1.value <= threshold=1.5051074624061584
Class: COVID-19 Severe	

Rules_277	passed counts:16
node_0: feature_name=LTB	LTB.value <= threshold=0.994031548500061
node_1: feature_name=MTRNR2L2	MTRNR2L2.value <= threshold=2.8784297704696655
node_2: feature_name=TCL1A	TCL1A.value <= threshold=4.798312664031982
node_3: feature_name=MT-CO1	MT-CO1.value > threshold=0.08947191759943962
node_2521: feature_name=LTB	LTB.value <= threshold=0.6038667261600494
node_2522: feature_name=HLA-DQB1	HLA-DQB1.value > threshold=2.1053054332733154
node_3550: feature_name=XIST	XIST.value <= threshold=0.7716337237507105
node_3551: feature_name=CD79B	CD79B.value <= threshold=2.9426796436309814
node_3552: feature_name=HLA-DQB1	HLA-DQB1.value > threshold=2.713674306869507
node_3854: feature_name=MEF2C	MEF2C.value <= threshold=-0.3721291869878769
node_3855: feature_name=HLA-DRB5	HLA-DRB5.value > threshold=0.7929701805114746
node_3859: feature_name=LTB	LTB.value <= threshold=-0.9689712226390839
node_3860: feature_name=PELI1	PELI1.value > threshold=-0.6289232671260834
Class: COVID-19 Critical	
Rules_278	passed counts:16
node_0: feature_name=LTB	LTB.value <= threshold=0.994031548500061
node_1: feature_name=MTRNR2L2	MTRNR2L2.value <= threshold=2.8784297704696655
node_2: feature_name=TCL1A	TCL1A.value <= threshold=4.798312664031982
node_3: feature_name=MT-CO1	MT-CO1.value > threshold=0.08947191759943962
node_2521: feature_name=LTB	LTB.value <= threshold=0.6038667261600494
node_2522: feature_name=HLA-DQB1	HLA-DQB1.value <= threshold=2.1053054332733154
node_2523: feature_name=SNX9	SNX9.value > threshold=-0.5346486568450928
node_2907: feature_name=NR4A1	NR4A1.value <= threshold=2.5433837175369263
node_2908: feature_name=XIST	XIST.value <= threshold=0.7664470989257097
node_2909: feature_name=CD79B	CD79B.value <= threshold=2.9326847791671753
node_2910: feature_name=NR4A1	NR4A1.value <= threshold=-0.2875557690858841
node_2911: feature_name=CTD-2035E11.3	CTD-2035E11.3.value <= threshold=5.262048006057739

node_2912: feature_name=VPREB3	VPREB3.value <= threshold=4.937594890594482
node_2913: feature_name=IRAK3	IRAK3.value <= threshold=4.592883586883545
node_2914: feature_name=IGJ	IGJ.value <= threshold=7.093410015106201
node_2915: feature_name=WDR74	WDR74.value <= threshold=4.077916383743286
node_2916: feature_name=MEF2C	MEF2C.value <= threshold=4.374382972717285
node_2917: feature_name=HIST1H2AC	HIST1H2AC.value <= threshold=3.743621349334717
node_2918: feature_name=STMN1	STMN1.value <= threshold=6.266510248184204
node_2919: feature_name=RALGPS2	RALGPS2.value <= threshold=5.72194766998291
node_2920: feature_name=HOPX	HOPX.value <= threshold=2.5944807529449463
node_2921: feature_name=MARCKS	MARCKS.value <= threshold=4.622651815414429
node_2922: feature_name=CCL3	CCL3.value <= threshold=0.7484713792800903
node_2923: feature_name=TPM1	TPM1.value <= threshold=6.446193695068359
node_2924: feature_name=PLD4	PLD4.value <= threshold=8.038907289505005
node_2925: feature_name=COTL1	COTL1.value > threshold=-1.8123814463615417
node_2929: feature_name=ID3	ID3.value <= threshold=7.534881353378296
node_2930: feature_name=IRF8	IRF8.value <= threshold=4.855781078338623
node_2931: feature_name=AP3M2	AP3M2.value <= threshold=1.8868122100830078
node_2932: feature_name=FCER2	FCER2.value <= threshold=6.877448558807373
node_2933: feature_name=MTRNR2L2	MTRNR2L2.value <= threshold=2.710945725440979
node_2934: feature_name=GNG11	GNG11.value <= threshold=2.510925054550171
node_2935: feature_name=CD83	CD83.value <= threshold=1.9873459935188293
node_2936: feature_name=MARCH1	MARCH1.value <= threshold=5.006200551986694
node_2937: feature_name=MEF2C	MEF2C.value > threshold=-0.5498070418834686
node_2941: feature_name=FCRL1	FCRL1.value <= threshold=7.093972444534302
node_2942: feature_name=HSP90B1	HSP90B1.value > threshold=-1.4724047780036926
node_2946: feature_name=HLA-DQA1	HLA-DQA1.value <= threshold=-0.45666155219078064
node_2947: feature_name=FAM160B1	FAM160B1.value > threshold=-0.3966478109359741
Class: COVID-19 Critical	
Rules_279	passed counts:16

node_0: feature_name=LTB	LTB.value <= threshold=0.994031548500061
node_1: feature_name=MTRNR2L2	MTRNR2L2.value <= threshold=2.8784297704696655
node_2: feature_name=TCL1A	TCL1A.value <= threshold=4.798312664031982
node_3: feature_name=MT-CO1	MT-CO1.value <= threshold=0.08947191759943962
node_4: feature_name=NR4A1	NR4A1.value > threshold=1.0725348591804504
node_1622: feature_name=HLA-DQA2	HLA-DQA2.value <= threshold=1.9496816992759705
node_1623: feature_name=HLA-DRB5	HLA-DRB5.value <= threshold=2.715766429901123
node_1624: feature_name=HLA-DPA1	HLA-DPA1.value > threshold=1.7050301432609558
node_2056: feature_name=OAZ1	OAZ1.value > threshold=0.456103190779686
node_2266: feature_name=XAF1	XAF1.value > threshold=-0.4431648850440979
node_2312: feature_name=ISG15	ISG15.value <= threshold=-0.3511900007724762
node_2313: feature_name=RALGPS2	RALGPS2.value > threshold=2.2695717811584473
node_2329: feature_name=LTB	LTB.value > threshold=-0.9258447885513306
node_2331: feature_name=PLEC	PLEC.value <= threshold=1.3002272844314575
Class: Healthy Control	
Rules_280	passed counts:16
node_0: feature_name=LTB	LTB.value <= threshold=0.994031548500061
node_1: feature_name=MTRNR2L2	MTRNR2L2.value <= threshold=2.8784297704696655
node_2: feature_name=TCL1A	TCL1A.value <= threshold=4.798312664031982
node_3: feature_name=MT-CO1	MT-CO1.value <= threshold=0.08947191759943962
node_4: feature_name=NR4A1	NR4A1.value > threshold=1.0725348591804504
node_1622: feature_name=HLA-DQA2	HLA-DQA2.value <= threshold=1.9496816992759705
node_1623: feature_name=HLA-DRB5	HLA-DRB5.value <= threshold=2.715766429901123
node_1624: feature_name=HLA-DPA1	HLA-DPA1.value <= threshold=1.7050301432609558
node_1625: feature_name=CD83	CD83.value > threshold=2.783489227294922
node_1873: feature_name=IFI44L	IFI44L.value <= threshold=1.5938722491264343
node_1874: feature_name=PLD4	PLD4.value <= threshold=4.101001739501953
node_1875: feature_name=RHOB	RHOB.value <= threshold=1.304835319519043
node_1876: feature_name=PPIF	PPIF.value <= threshold=2.2943923473358154

node_1877: feature_name=OAZ1	OAZ1.value > threshold=0.04260866530239582
node_1935: feature_name=RGS1	RGS1.value > threshold=-0.13325228542089462
node_1955: feature_name=DUSP4	DUSP4.value <= threshold=1.2541037425398827
node_1956: feature_name=BACH1	BACH1.value <= threshold=2.0189278721809387
node_1957: feature_name=IGJ	IGJ.value > threshold=-0.36648716032505035
Class: COVID-19 Severe	
Rules_281	passed counts:16
node_0: feature_name=LTB	LTB.value <= threshold=0.994031548500061
node_1: feature_name=MTRNR2L2	MTRNR2L2.value <= threshold=2.8784297704696655
node_2: feature_name=TCL1A	TCL1A.value <= threshold=4.798312664031982
node_3: feature_name=MT-CO1	MT-CO1.value <= threshold=0.08947191759943962
node_4: feature_name=NR4A1	NR4A1.value > threshold=1.0725348591804504
node_1622: feature_name=HLA-DQA2	HLA-DQA2.value <= threshold=1.9496816992759705
node_1623: feature_name=HLA-DRB5	HLA-DRB5.value <= threshold=2.715766429901123
node_1624: feature_name=HLA-DPA1	HLA-DPA1.value <= threshold=1.7050301432609558
node_1625: feature_name=CD83	CD83.value <= threshold=2.783489227294922
node_1626: feature_name=XIST	XIST.value <= threshold=0.9589543007314205
node_1627: feature_name=PLD4	PLD4.value <= threshold=5.865932464599609
node_1628: feature_name=OAZ1	OAZ1.value > threshold=0.6137169003486633
node_1840: feature_name=MT-CO1	MT-CO1.value > threshold=-0.28350088000297546
node_1850: feature_name=CD83	CD83.value > threshold=2.1227927207946777
node_1856: feature_name=WDR74	WDR74.value <= threshold=1.5303557515144348
node_1857: feature_name=BCL2A1	BCL2A1.value <= threshold=1.396297037601471
Class: Healthy Control	
Rules_282	passed counts:16
node_0: feature_name=LTB	LTB.value <= threshold=0.994031548500061
node_1: feature_name=MTRNR2L2	MTRNR2L2.value <= threshold=2.8784297704696655
node_2: feature_name=TCL1A	TCL1A.value <= threshold=4.798312664031982

node_3: feature_name=MT-CO1	MT-CO1.value <= threshold=0.08947191759943962
node_4: feature_name=NR4A1	NR4A1.value <= threshold=1.0725348591804504
node_5: feature_name=HLA-DPA1	HLA-DPA1.value > threshold=1.6885469555854797
node_861: feature_name=CD83	CD83.value > threshold=3.0563642978668213
node_1539: feature_name=ID1	ID1.value <= threshold=1.4626862406730652
node_1540: feature_name=FTH1	FTH1.value > threshold=0.2985198348760605
node_1590: feature_name=RP5-821D11.7	RP5-821D11.7.value > threshold=-0.2580558806657791
node_1610: feature_name=CD79B	CD79B.value <= threshold=2.037744402885437
Class: COVID-19 Severe	
Rules_283	passed counts:16
node_0: feature_name=LTB	LTB.value <= threshold=0.994031548500061
node_1: feature_name=MTRNR2L2	MTRNR2L2.value <= threshold=2.8784297704696655
node_2: feature_name=TCL1A	TCL1A.value <= threshold=4.798312664031982
node_3: feature_name=MT-CO1	MT-CO1.value <= threshold=0.08947191759943962
node_4: feature_name=NR4A1	NR4A1.value <= threshold=1.0725348591804504
node_5: feature_name=HLA-DPA1	HLA-DPA1.value > threshold=1.6885469555854797
node_861: feature_name=CD83	CD83.value <= threshold=3.0563642978668213
node_862: feature_name=HLA-DQB1	HLA-DQB1.value <= threshold=2.61081063747406
node_863: feature_name=MTRNR2L2	MTRNR2L2.value <= threshold=2.0919997692108154
node_864: feature_name=BCL2A1	BCL2A1.value <= threshold=1.852618932723999
node_865: feature_name=CD79B	CD79B.value > threshold=4.352142333984375
node_1309: feature_name=COTL1	COTL1.value > threshold=0.48328976333141327
node_1321: feature_name=MS4A1	MS4A1.value > threshold=2.450835347175598
node_1323: feature_name=TNFAIP3	TNFAIP3.value <= threshold=-0.4280368238687515
Class: Healthy Control	
Rules_284	passed counts:16
node_0: feature_name=LTB	LTB.value <= threshold=0.994031548500061
node_1: feature_name=MTRNR2L2	MTRNR2L2.value <= threshold=2.8784297704696655

node_2: feature_name=TCL1A	TCL1A.value <= threshold=4.798312664031982
node_3: feature_name=MT-CO1	MT-CO1.value <= threshold=0.08947191759943962
node_4: feature_name=NR4A1	NR4A1.value <= threshold=1.0725348591804504
node_5: feature_name=HLA-DPA1	HLA-DPA1.value > threshold=1.6885469555854797
node_861: feature_name=CD83	CD83.value <= threshold=3.0563642978668213
node_862: feature_name=HLA-DQB1	HLA-DQB1.value <= threshold=2.61081063747406
node_863: feature_name=MTRNR2L2	MTRNR2L2.value <= threshold=2.0919997692108154
node_864: feature_name=BCL2A1	BCL2A1.value <= threshold=1.852618932723999
node_865: feature_name=CD79B	CD79B.value <= threshold=4.352142333984375
node_866: feature_name=TNFAIP3	TNFAIP3.value <= threshold=-0.0449496041983366
node_867: feature_name=MT-CO1	MT-CO1.value > threshold=-0.08531957119703293
node_1155: feature_name=IRF8	IRF8.value <= threshold=-0.39239931106567383
node_1156: feature_name=OAZ1	OAZ1.value <= threshold=0.027674595825374126
node_1157: feature_name=IGLL5	IGLL5.value > threshold=-0.2645253688097
node_1159: feature_name=HLA-DPA1	HLA-DPA1.value <= threshold=2.097427725791931
node_1160: feature_name=KIAA0125	KIAA0125.value <= threshold=4.518599271774292
Class: COVID-19 Critical	
Rules_285	passed counts:16
node_0: feature_name=LTB	LTB.value <= threshold=0.994031548500061
node_1: feature_name=MTRNR2L2	MTRNR2L2.value <= threshold=2.8784297704696655
node_2: feature_name=TCL1A	TCL1A.value <= threshold=4.798312664031982
node_3: feature_name=MT-CO1	MT-CO1.value <= threshold=0.08947191759943962
node_4: feature_name=NR4A1	NR4A1.value <= threshold=1.0725348591804504
node_5: feature_name=HLA-DPA1	HLA-DPA1.value <= threshold=1.6885469555854797
node_6: feature_name=HLA-DRB5	HLA-DRB5.value > threshold=2.1886194944381714
node_738: feature_name=HLA-DQA2	HLA-DQA2.value <= threshold=-0.20105575770139694
node_739: feature_name=HLA-DQA1	HLA-DQA1.value <= threshold=1.7414769530296326
node_740: feature_name=TNFAIP3	TNFAIP3.value > threshold=-0.5488012433052063
node_750: feature_name=SNX9	SNX9.value > threshold=0.20590564608573914

node_754: feature_name=HSP90B1	HSP90B1.value > threshold=-1.1355486512184143
node_758: feature_name=CCR6	CCR6.value <= threshold=2.731774926185608
node_759: feature_name=HLA-DPB1	HLA-DPB1.value > threshold=-0.2631095051765442
Class: COVID-19 Critical	
Rules_286	passed counts:16
node_0: feature_name=LTB	LTB.value <= threshold=0.994031548500061
node_1: feature_name=MTRNR2L2	MTRNR2L2.value <= threshold=2.8784297704696655
node_2: feature_name=TCL1A	TCL1A.value <= threshold=4.798312664031982
node_3: feature_name=MT-CO1	MT-CO1.value <= threshold=0.08947191759943962
node_4: feature_name=NR4A1	NR4A1.value <= threshold=1.0725348591804504
node_5: feature_name=HLA-DPA1	HLA-DPA1.value <= threshold=1.6885469555854797
node_6: feature_name=HLA-DRB5	HLA-DRB5.value <= threshold=2.1886194944381714
node_7: feature_name=XIST	XIST.value <= threshold=-0.12328458577394485
node_8: feature_name=SYNE1	SYNE1.value > threshold=-0.7835594415664673
node_16: feature_name=MTRNR2L2	MTRNR2L2.value <= threshold=2.4483247995376587
node_17: feature_name=HLA-DQA2	HLA-DQA2.value <= threshold=2.227178931236267
node_18: feature_name=NR4A1	NR4A1.value > threshold=-0.21380723267793655
node_504: feature_name=LTB	LTB.value > threshold=0.8758118152618408
node_584: feature_name=BACH1	BACH1.value > threshold=-0.5905149877071381
node_588: feature_name=RALGPS2	RALGPS2.value <= threshold=3.0709422826766968
Class: COVID-19 Critical	
Rules_287	passed counts:16
node_0: feature_name=LTB	LTB.value <= threshold=0.994031548500061
node_1: feature_name=MTRNR2L2	MTRNR2L2.value <= threshold=2.8784297704696655
node_2: feature_name=TCL1A	TCL1A.value <= threshold=4.798312664031982
node_3: feature_name=MT-CO1	MT-CO1.value <= threshold=0.08947191759943962
node_4: feature_name=NR4A1	NR4A1.value <= threshold=1.0725348591804504
node_5: feature_name=HLA-DPA1	HLA-DPA1.value <= threshold=1.6885469555854797

node_6: feature_name=HLA-DRB5	HLA-DRB5.value <= threshold=2.1886194944381714
node_7: feature_name=XIST	XIST.value <= threshold=-0.12328458577394485
node_8: feature_name=SYNE1	SYNE1.value > threshold=-0.7835594415664673
node_16: feature_name=MTRNR2L2	MTRNR2L2.value <= threshold=2.4483247995376587
node_17: feature_name=HLA-DQA2	HLA-DQA2.value <= threshold=2.227178931236267
node_18: feature_name=NR4A1	NR4A1.value <= threshold=-0.21380723267793655
node_19: feature_name=IRAK3	IRAK3.value <= threshold=4.27159571647644
node_20: feature_name=IFIT2	IFIT2.value <= threshold=7.8175740242004395
node_21: feature_name=CD79A	CD79A.value <= threshold=2.857242226600647
node_22: feature_name=HIST1H2AC	HIST1H2AC.value <= threshold=5.077470302581787
node_23: feature_name=FTH1	FTH1.value <= threshold=0.4158056080341339
node_24: feature_name=ATF3	ATF3.value <= threshold=3.8028353452682495
node_25: feature_name=OASL	OASL.value <= threshold=3.387580633163452
node_26: feature_name=MT-CO1	MT-CO1.value > threshold=-0.7942646145820618
node_74: feature_name=HOPX	HOPX.value <= threshold=0.8682331740856171
node_75: feature_name=RGS2	RGS2.value <= threshold=1.4589954018592834
node_76: feature_name=AHR	AHR.value <= threshold=3.870481848716736
node_77: feature_name=IGJ	IGJ.value <= threshold=4.902882814407349
node_78: feature_name=CD79B	CD79B.value <= threshold=4.87716269493103
node_79: feature_name=HLA-DQB1	HLA-DQB1.value <= threshold=3.6925491094589233
node_80: feature_name=HLA-DRB5	HLA-DRB5.value <= threshold=2.0926843881607056
node_81: feature_name=MCTP1	MCTP1.value <= threshold=4.010472774505615
node_82: feature_name=GNG11	GNG11.value > threshold=3.627470850944519
node_194: feature_name=SNX9	SNX9.value > threshold=-0.5442804098129272
Class: COVID-19 Critical	
Rules_288	passed counts:16
node_0: feature_name=LTB	LTB.value <= threshold=0.994031548500061
node_1: feature_name=MTRNR2L2	MTRNR2L2.value <= threshold=2.8784297704696655
node_2: feature_name=TCL1A	TCL1A.value <= threshold=4.798312664031982

node_3: feature_name=MT-CO1	MT-CO1.value <= threshold=0.08947191759943962
node_4: feature_name=NR4A1	NR4A1.value <= threshold=1.0725348591804504
node_5: feature_name=HLA-DPA1	HLA-DPA1.value <= threshold=1.6885469555854797
node_6: feature_name=HLA-DRB5	HLA-DRB5.value <= threshold=2.1886194944381714
node_7: feature_name=XIST	XIST.value <= threshold=-0.12328458577394485
node_8: feature_name=SYNE1	SYNE1.value > threshold=-0.7835594415664673
node_16: feature_name=MTRNR2L2	MTRNR2L2.value <= threshold=2.4483247995376587
node_17: feature_name=HLA-DQA2	HLA-DQA2.value <= threshold=2.227178931236267
node_18: feature_name=NR4A1	NR4A1.value <= threshold=-0.21380723267793655
node_19: feature_name=IRAK3	IRAK3.value <= threshold=4.27159571647644
node_20: feature_name=IFIT2	IFIT2.value <= threshold=7.8175740242004395
node_21: feature_name=CD79A	CD79A.value <= threshold=2.857242226600647
node_22: feature_name=HIST1H2AC	HIST1H2AC.value <= threshold=5.077470302581787
node_23: feature_name=FTH1	FTH1.value <= threshold=0.4158056080341339
node_24: feature_name=ATF3	ATF3.value <= threshold=3.8028353452682495
node_25: feature_name=OASL	OASL.value <= threshold=3.387580633163452
node_26: feature_name=MT-CO1	MT-CO1.value > threshold=-0.7942646145820618
node_74: feature_name=HOPX	HOPX.value <= threshold=0.8682331740856171
node_75: feature_name=RGS2	RGS2.value <= threshold=1.4589954018592834
node_76: feature_name=AHR	AHR.value <= threshold=3.870481848716736
node_77: feature_name=IGJ	IGJ.value <= threshold=4.902882814407349
node_78: feature_name=CD79B	CD79B.value <= threshold=4.87716269493103
node_79: feature_name=HLA-DQB1	HLA-DQB1.value <= threshold=3.6925491094589233
node_80: feature_name=HLA-DRB5	HLA-DRB5.value <= threshold=2.0926843881607056
node_81: feature_name=MCTP1	MCTP1.value <= threshold=4.010472774505615
node_82: feature_name=GNG11	GNG11.value <= threshold=3.627470850944519
node_83: feature_name=HSP90B1	HSP90B1.value <= threshold=2.0835131406784058
node_84: feature_name=ATF3	ATF3.value <= threshold=1.1142374873161316
node_85: feature_name=MT-ND6	MT-ND6.value <= threshold=2.7328014373779297
node_86: feature_name=COTL1	COTL1.value > threshold=-1.6524587869644165

node_90: feature_name=MT-CYB	MT-CYB.value <= threshold=1.099629819393158
node_91: feature_name=RGCC	RGCC.value <= threshold=1.6908138990402222
node_92: feature_name=ILK	ILK.value <= threshold=3.9497616291046143
node_93: feature_name=CTD-2035E11.3	CTD-2035E11.3.value <= threshold=2.4543306827545166
node_94: feature_name=S100A6	S100A6.value <= threshold=0.2867250442504883
node_95: feature_name=HLA-DRB5	HLA-DRB5.value > threshold=-0.9496813416481018
node_99: feature_name=MT-CO3	MT-CO3.value > threshold=-1.55654776096344
node_103: feature_name=ITGAX	ITGAX.value <= threshold=1.8544010519981384
node_104: feature_name=MEF2C	MEF2C.value <= threshold=4.22724461555481
node_105: feature_name=CXorf57	CXorf57.value <= threshold=5.114444732666016
node_106: feature_name=IRAK3	IRAK3.value <= threshold=3.1017903089523315
node_107: feature_name=DUSP4	DUSP4.value <= threshold=2.9122174978256226
node_108: feature_name=OAZ1	OAZ1.value > threshold=-1.8057305812835693
node_112: feature_name=DAPP1	DAPP1.value <= threshold=4.016228437423706
node_113: feature_name=HSP90B1	HSP90B1.value > threshold=-1.2981283068656921
node_119: feature_name=MT-CYB	MT-CYB.value <= threshold=0.9500376582145691
node_120: feature_name=HSP90B1	HSP90B1.value <= threshold=1.7814298272132874
node_121: feature_name=ACSL1	ACSL1.value > threshold=2.289203405380249
node_123: feature_name=ACSL1	ACSL1.value > threshold=2.323211908340454
Class: COVID-19 Critical	
Rules_289	passed counts:15
node_0: feature_name=LTB	LTB.value > threshold=0.994031548500061
node_5264: feature_name=MT-CO1	MT-CO1.value > threshold=-0.4127991199493408
node_5638: feature_name=OAZ1	OAZ1.value > threshold=-0.2640867084264755
node_6112: feature_name=IFI44L	IFI44L.value <= threshold=2.5893263816833496
node_6113: feature_name=TCL1A	TCL1A.value <= threshold=4.201832294464111
node_6114: feature_name=HLA-DQA2	HLA-DQA2.value > threshold=-0.2270243540406227
node_6542: feature_name=CD79B	CD79B.value > threshold=3.096232771873474
node_6872: feature_name=ICAM1	ICAM1.value <= threshold=-0.2232399433851242

node_6873: feature_name=STAG3	STAG3.value > threshold=-0.19312061369419098
node_6939: feature_name=HLA-DRB5	HLA-DRB5.value <= threshold=2.209904909133911
node_6940: feature_name=STAG3	STAG3.value <= threshold=7.8730878829956055
node_6941: feature_name=FAM129C	FAM129C.value <= threshold=1.668714702129364
node_6942: feature_name=HLA-DPB1	HLA-DPB1.value > threshold=1.4723529815673828
node_6944: feature_name=BOD1L1	BOD1L1.value <= threshold=0.9118953347206116
node_6945: feature_name=ACSL1	ACSL1.value <= threshold=0.4698426127433777
Class: Healthy Control	
Rules_290	passed counts:15
node_0: feature_name=LTB	LTB.value > threshold=0.994031548500061
node_5264: feature_name=MT-CO1	MT-CO1.value > threshold=-0.4127991199493408
node_5638: feature_name=OAZ1	OAZ1.value > threshold=-0.2640867084264755
node_6112: feature_name=IFI44L	IFI44L.value <= threshold=2.5893263816833496
node_6113: feature_name=TCL1A	TCL1A.value <= threshold=4.201832294464111
node_6114: feature_name=HLA-DQA2	HLA-DQA2.value <= threshold=-0.2270243540406227
node_6115: feature_name=MTRNR2L2	MTRNR2L2.value <= threshold=2.548917293548584
node_6116: feature_name=ATF5	ATF5.value <= threshold=2.041263461112976
node_6117: feature_name=JUN	JUN.value > threshold=-0.616259902715683
node_6189: feature_name=MT-CO3	MT-CO3.value > threshold=0.13483521342277527
node_6351: feature_name=XAF1	XAF1.value <= threshold=3.061717987060547
node_6352: feature_name=MT-CO2	MT-CO2.value <= threshold=1.2605181336402893
node_6353: feature_name=ICAM1	ICAM1.value > threshold=-0.3340446650981903
node_6439: feature_name=FAM129C	FAM129C.value <= threshold=-0.24657993018627167
node_6440: feature_name=ITM2C	ITM2C.value > threshold=-0.2592049688100815
node_6446: feature_name=S100A6	S100A6.value > threshold=-0.7434525489807129
node_6452: feature_name=SYTL3	SYTL3.value <= threshold=0.2586190104484558
Class: Healthy Control	
Rules_291	passed counts:15

node_0: feature_name=LTB	LTB.value > threshold=0.994031548500061
node_5264: feature_name=MT-CO1	MT-CO1.value > threshold=-0.4127991199493408
node_5638: feature_name=OAZ1	OAZ1.value > threshold=-0.2640867084264755
node_6112: feature_name=IFI44L	IFI44L.value <= threshold=2.5893263816833496
node_6113: feature_name=TCL1A	TCL1A.value <= threshold=4.201832294464111
node_6114: feature_name=HLA-DQA2	HLA-DQA2.value <= threshold=-0.2270243540406227
node_6115: feature_name=MTRNR2L2	MTRNR2L2.value <= threshold=2.548917293548584
node_6116: feature_name=ATF5	ATF5.value <= threshold=2.041263461112976
node_6117: feature_name=JUN	JUN.value <= threshold=-0.616259902715683
node_6118: feature_name=NFKBID	NFKBID.value > threshold=1.5264258980751038
node_6166: feature_name=NR4A1	NR4A1.value > threshold=0.6553736329078674
node_6184: feature_name=RP5-821D11.7	RP5-821D11.7.value <= threshold=-0.22415155917406082
Class: Healthy Control	
Rules_292	passed counts:15
node_0: feature_name=LTB	LTB.value > threshold=0.994031548500061
node_5264: feature_name=MT-CO1	MT-CO1.value > threshold=-0.4127991199493408
node_5638: feature_name=OAZ1	OAZ1.value <= threshold=-0.2640867084264755
node_5639: feature_name=CD79B	CD79B.value > threshold=2.2998517751693726
node_5925: feature_name=SNX9	SNX9.value <= threshold=2.4445992708206177
node_5926: feature_name=TCL1A	TCL1A.value <= threshold=2.6938670873641968
node_5927: feature_name=PLD4	PLD4.value <= threshold=-0.06434785947203636
node_5928: feature_name=ICAM1	ICAM1.value <= threshold=-0.2820792943239212
node_5929: feature_name=MS4A1	MS4A1.value <= threshold=2.747320055961609
node_5930: feature_name=LTB	LTB.value > threshold=1.3533939123153687
node_5954: feature_name=HLA-DPB1	HLA-DPB1.value > threshold=1.5530678033828735
node_5958: feature_name=FCER2	FCER2.value > threshold=-0.24438364803791046
node_5960: feature_name=MT-CYB	MT-CYB.value > threshold=-0.4359520673751831
Class: Healthy Control	

Rules_293	passed counts:15
node_0: feature_name=LTB	LTB.value > threshold=0.994031548500061
node_5264: feature_name=MT-CO1	MT-CO1.value > threshold=-0.4127991199493408
node_5638: feature_name=OAZ1	OAZ1.value <= threshold=-0.2640867084264755
node_5639: feature_name=CD79B	CD79B.value <= threshold=2.2998517751693726
node_5640: feature_name=MTRNR2L2	MTRNR2L2.value <= threshold=3.5499863624572754
node_5641: feature_name=MT-CO1	MT-CO1.value > threshold=0.22567133605480194
node_5729: feature_name=MARCH1	MARCH1.value > threshold=1.5154643654823303
node_5867: feature_name=NFKBID	NFKBID.value <= threshold=2.6947624683380127
node_5868: feature_name=UTY	UTY.value <= threshold=3.29777729511261
node_5869: feature_name=IFI44L	IFI44L.value <= threshold=-0.29640993475914
node_5870: feature_name=FTH1	FTH1.value <= threshold=0.6966870129108429
node_5871: feature_name=ILK	ILK.value <= threshold=1.5719919204711914
node_5872: feature_name=MARCKS	MARCKS.value > threshold=1.20343416929245
node_5882: feature_name=RALGPS2	RALGPS2.value > threshold=2.361847758293152
node_5888: feature_name=DAPP1	DAPP1.value <= threshold=3.4492971897125244
Class: Healthy Control	
Rules_294	passed counts:15
node_0: feature_name=LTB	LTB.value > threshold=0.994031548500061
node_5264: feature_name=MT-CO1	MT-CO1.value > threshold=-0.4127991199493408
node_5638: feature_name=OAZ1	OAZ1.value <= threshold=-0.2640867084264755
node_5639: feature_name=CD79B	CD79B.value <= threshold=2.2998517751693726
node_5640: feature_name=MTRNR2L2	MTRNR2L2.value <= threshold=3.5499863624572754
node_5641: feature_name=MT-CO1	MT-CO1.value > threshold=0.22567133605480194
node_5729: feature_name=MARCH1	MARCH1.value <= threshold=1.5154643654823303
node_5730: feature_name=MT-CO1	MT-CO1.value <= threshold=1.7823500633239746
node_5731: feature_name=HLA-DPB1	HLA-DPB1.value > threshold=2.0096163749694824
node_5829: feature_name=LTB	LTB.value <= threshold=1.2124510407447815
node_5830: feature_name=MT-CO1	MT-CO1.value > threshold=0.41994138062000275

node_5834: feature_name=WDR74	WDR74.value <= threshold=-0.48832663893699646
node_5835: feature_name=COTL1	COTL1.value > threshold=-0.8589395880699158
Class: COVID-19 Critical	
Rules_295	passed counts:15
node_0: feature_name=LTB	LTB.value > threshold=0.994031548500061
node_5264: feature_name=MT-CO1	MT-CO1.value <= threshold=-0.4127991199493408
node_5265: feature_name=MTRNR2L2	MTRNR2L2.value <= threshold=2.4473838806152344
node_5266: feature_name=TCL1A	TCL1A.value <= threshold=5.787256956100464
node_5267: feature_name=PLD4	PLD4.value <= threshold=3.544657826423645
node_5268: feature_name=NR4A1	NR4A1.value <= threshold=0.8490704298019409
node_5269: feature_name=LTB	LTB.value > threshold=1.5252206921577454
node_5471: feature_name=CD79A	CD79A.value > threshold=2.510846734046936
node_5483: feature_name=XAF1	XAF1.value <= threshold=1.7000007629394531
node_5484: feature_name=RALGPS2	RALGPS2.value <= threshold=2.08430278301239
node_5485: feature_name=HLA-DPB1	HLA-DPB1.value <= threshold=2.0922847390174866
node_5486: feature_name=XAF1	XAF1.value <= threshold=-0.4127534031867981
node_5487: feature_name=LTB	LTB.value > threshold=1.548236906528473
Class: COVID-19 Critical	
Rules_296	passed counts:15
node_0: feature_name=LTB	LTB.value > threshold=0.994031548500061
node_5264: feature_name=MT-CO1	MT-CO1.value <= threshold=-0.4127991199493408
node_5265: feature_name=MTRNR2L2	MTRNR2L2.value <= threshold=2.4473838806152344
node_5266: feature_name=TCL1A	TCL1A.value <= threshold=5.787256956100464
node_5267: feature_name=PLD4	PLD4.value <= threshold=3.544657826423645
node_5268: feature_name=NR4A1	NR4A1.value <= threshold=0.8490704298019409
node_5269: feature_name=LTB	LTB.value <= threshold=1.5252206921577454
node_5270: feature_name=HLA-DRB5	HLA-DRB5.value <= threshold=2.5117164850234985
node_5271: feature_name=CD79A	CD79A.value <= threshold=3.685201406478882

node_5272: feature_name=BCL2A1	BCL2A1.value <= threshold=2.081526756286621
node_5273: feature_name=HLA-DPA1	HLA-DPA1.value <= threshold=1.8042166829109192
node_5274: feature_name=HSP90B1	HSP90B1.value > threshold=-1.3115270137786865
node_5278: feature_name=OAZ1	OAZ1.value <= threshold=1.0802008509635925
node_5279: feature_name=CD79B	CD79B.value <= threshold=5.875581979751587
node_5280: feature_name=CCDC141	CCDC141.value <= threshold=6.439151048660278
node_5281: feature_name=SYTL3	SYTL3.value <= threshold=1.149337351322174
node_5282: feature_name=GPX1	GPX1.value > threshold=-1.0243326425552368
node_5284: feature_name=MMD	MMD.value <= threshold=6.431635856628418
node_5285: feature_name=MARCKS	MARCKS.value <= threshold=4.034798860549927
node_5286: feature_name=XIST	XIST.value <= threshold=1.148451752960682
node_5287: feature_name=CD83	CD83.value <= threshold=2.894522190093994
node_5288: feature_name=MIR24-2	MIR24-2.value <= threshold=2.76771080493927
node_5289: feature_name=GRASP	GRASP.value > threshold=-0.4039058983325958
node_5303: feature_name=CD38	CD38.value <= threshold=3.721052408218384
node_5304: feature_name=ATF5	ATF5.value <= threshold=6.64192533493042
node_5305: feature_name=CD22	CD22.value > threshold=5.268923759460449
node_5323: feature_name=TNFAIP3	TNFAIP3.value <= threshold=-0.3317747786641121
Class: COVID-19 Critical	
Rules_297	passed counts:15
node_0: feature_name=LTB	LTB.value <= threshold=0.994031548500061
node_1: feature_name=MTRNR2L2	MTRNR2L2.value <= threshold=2.8784297704696655
node_2: feature_name=TCL1A	TCL1A.value > threshold=4.798312664031982
node_4912: feature_name=MT-CO1	MT-CO1.value > threshold=-0.3664844036102295
node_5002: feature_name=IFI44L	IFI44L.value <= threshold=2.3101747035980225
node_5003: feature_name=SNX9	SNX9.value <= threshold=2.0247421264648438
node_5004: feature_name=STAG3	STAG3.value <= threshold=6.482141733169556
node_5005: feature_name=BACH1	BACH1.value > threshold=-0.5237349569797516
node_5047: feature_name=RHOB	RHOB.value <= threshold=-0.4843696355819702

node_5048: feature_name=MT-CO1	MT-CO1.value <= threshold=0.6870226263999939
node_5049: feature_name=HIST1H2AC	HIST1H2AC.value <= threshold=1.6820606589317322
node_5050: feature_name=STMN1	STMN1.value <= threshold=1.5689300894737244
Class: COVID-19 Critical	
Rules_298	passed counts:15
node_0: feature_name=LTB	LTB.value <= threshold=0.994031548500061
node_1: feature_name=MTRNR2L2	MTRNR2L2.value <= threshold=2.8784297704696655
node_2: feature_name=TCL1A	TCL1A.value > threshold=4.798312664031982
node_4912: feature_name=MT-CO1	MT-CO1.value <= threshold=-0.3664844036102295
node_4913: feature_name=CD79A	CD79A.value > threshold=2.987208843231201
node_4973: feature_name=IFI44L	IFI44L.value <= threshold=0.7183398455381393
node_4974: feature_name=CD79B	CD79B.value <= threshold=1.7606428265571594
node_4975: feature_name=HLA-DPA1	HLA-DPA1.value <= threshold=1.9396945238113403
Class: COVID-19 Critical	
Rules_299	passed counts:15
node_0: feature_name=LTB	LTB.value <= threshold=0.994031548500061
node_1: feature_name=MTRNR2L2	MTRNR2L2.value <= threshold=2.8784297704696655
node_2: feature_name=TCL1A	TCL1A.value <= threshold=4.798312664031982
node_3: feature_name=MT-CO1	MT-CO1.value > threshold=0.08947191759943962
node_2521: feature_name=LTB	LTB.value > threshold=0.6038667261600494
node_4173: feature_name=OAZ1	OAZ1.value > threshold=0.143283873796463
node_4615: feature_name=NR4A1	NR4A1.value <= threshold=0.6621004343032837
node_4616: feature_name=NFKBID	NFKBID.value <= threshold=3.283741593360901
node_4617: feature_name=HLA-DQA1	HLA-DQA1.value <= threshold=2.3435752391815186
node_4618: feature_name=PELI1	PELI1.value <= threshold=1.3271203637123108
node_4619: feature_name=PLD4	PLD4.value <= threshold=3.3385335206985474
node_4620: feature_name=MEF2A	MEF2A.value <= threshold=1.5926940441131592
node_4621: feature_name=DAPP1	DAPP1.value <= threshold=2.647637367248535

node_4622: feature_name=STAG3	STAG3.value > threshold=-0.16521219909191132
node_4648: feature_name=CD22	CD22.value > threshold=-0.21456258744001389
node_4652: feature_name=JUN	JUN.value > threshold=-1.9156492948532104
Class: COVID-19 Critical	
Rules_300	passed counts:15
node_0: feature_name=LTB	LTB.value <= threshold=0.994031548500061
node_1: feature_name=MTRNR2L2	MTRNR2L2.value <= threshold=2.8784297704696655
node_2: feature_name=TCL1A	TCL1A.value <= threshold=4.798312664031982
node_3: feature_name=MT-CO1	MT-CO1.value > threshold=0.08947191759943962
node_2521: feature_name=LTB	LTB.value > threshold=0.6038667261600494
node_4173: feature_name=OAZ1	OAZ1.value <= threshold=0.143283873796463
node_4174: feature_name=PLD4	PLD4.value <= threshold=2.921932101249695
node_4175: feature_name=XIST	XIST.value <= threshold=-0.05067425221204758
node_4176: feature_name=MT-CO1	MT-CO1.value <= threshold=1.345594048500061
node_4177: feature_name=HLA-DQB1	HLA-DQB1.value > threshold=1.927736520767212
node_4353: feature_name=ITM2C	ITM2C.value > threshold=-0.12329206243157387
node_4459: feature_name=SNX9	SNX9.value <= threshold=0.6323382556438446
node_4460: feature_name=NR4A1	NR4A1.value > threshold=0.9784677922725677
Class: Healthy Control	
Rules_301	passed counts:15
node_0: feature_name=LTB	LTB.value <= threshold=0.994031548500061
node_1: feature_name=MTRNR2L2	MTRNR2L2.value <= threshold=2.8784297704696655
node_2: feature_name=TCL1A	TCL1A.value <= threshold=4.798312664031982
node_3: feature_name=MT-CO1	MT-CO1.value > threshold=0.08947191759943962
node_2521: feature_name=LTB	LTB.value > threshold=0.6038667261600494
node_4173: feature_name=OAZ1	OAZ1.value <= threshold=0.143283873796463
node_4174: feature_name=PLD4	PLD4.value <= threshold=2.921932101249695
node_4175: feature_name=XIST	XIST.value <= threshold=-0.05067425221204758

node_4176: feature_name=MT-CO1	MT-CO1.value <= threshold=1.345594048500061
node_4177: feature_name=HLA-DQB1	HLA-DQB1.value > threshold=1.927736520767212
node_4353: feature_name=ITM2C	ITM2C.value <= threshold=-0.12329206243157387
node_4354: feature_name=MX1	MX1.value <= threshold=-0.4356398284435272
node_4355: feature_name=HLA-DQB1	HLA-DQB1.value <= threshold=2.8865538835525513
node_4356: feature_name=SNX9	SNX9.value <= threshold=1.002076417207718
node_4357: feature_name=FAM160B1	FAM160B1.value > threshold=-0.42920351028442383
node_4361: feature_name=SNX9	SNX9.value <= threshold=0.011789441108703613
node_4362: feature_name=MT-CO3	MT-CO3.value > threshold=0.4059640020132065
node_4376: feature_name=NFKBID	NFKBID.value <= threshold=3.8887706995010376
Class: COVID-19 Critical	
Rules_302	passed counts:15
node_0: feature_name=LTB	LTB.value <= threshold=0.994031548500061
node_1: feature_name=MTRNR2L2	MTRNR2L2.value <= threshold=2.8784297704696655
node_2: feature_name=TCL1A	TCL1A.value <= threshold=4.798312664031982
node_3: feature_name=MT-CO1	MT-CO1.value > threshold=0.08947191759943962
node_2521: feature_name=LTB	LTB.value > threshold=0.6038667261600494
node_4173: feature_name=OAZ1	OAZ1.value <= threshold=0.143283873796463
node_4174: feature_name=PLD4	PLD4.value <= threshold=2.921932101249695
node_4175: feature_name=XIST	XIST.value <= threshold=-0.05067425221204758
node_4176: feature_name=MT-CO1	MT-CO1.value <= threshold=1.345594048500061
node_4177: feature_name=HLA-DQB1	HLA-DQB1.value <= threshold=1.927736520767212
node_4178: feature_name=BACH1	BACH1.value <= threshold=-0.5349778532981873
node_4179: feature_name=CD79B	CD79B.value > threshold=1.6518195271492004
node_4201: feature_name=MT-CO2	MT-CO2.value <= threshold=0.5007440596818924
node_4202: feature_name=PELI1	PELI1.value > threshold=0.9399837851524353
node_4212: feature_name=TTN	TTN.value > threshold=-0.3203035891056061
node_4214: feature_name=RGS2	RGS2.value <= threshold=0.8960320353507996
node_4215: feature_name=ABCA1	ABCA1.value <= threshold=1.0574960261583328

Class: COVID-19 Critical	
Rules_303	passed counts:15
node_0: feature_name=LTB	LTB.value <= threshold=0.994031548500061
node_1: feature_name=MTRNR2L2	MTRNR2L2.value <= threshold=2.8784297704696655
node_2: feature_name=TCL1A	TCL1A.value <= threshold=4.798312664031982
node_3: feature_name=MT-CO1	MT-CO1.value > threshold=0.08947191759943962
node_2521: feature_name=LTB	LTB.value > threshold=0.6038667261600494
node_4173: feature_name=OAZ1	OAZ1.value <= threshold=0.143283873796463
node_4174: feature_name=PLD4	PLD4.value <= threshold=2.921932101249695
node_4175: feature_name=XIST	XIST.value <= threshold=-0.05067425221204758
node_4176: feature_name=MT-CO1	MT-CO1.value <= threshold=1.345594048500061
node_4177: feature_name=HLA-DQB1	HLA-DQB1.value <= threshold=1.927736520767212
node_4178: feature_name=BACH1	BACH1.value <= threshold=-0.5349778532981873
node_4179: feature_name=CD79B	CD79B.value <= threshold=1.6518195271492004
node_4180: feature_name=FTH1	FTH1.value > threshold=-1.4486562609672546
node_4184: feature_name=FAM160B1	FAM160B1.value <= threshold=1.850276529788971
node_4185: feature_name=HSPA1A	HSPA1A.value <= threshold=1.0704439878463745
node_4186: feature_name=NR4A1	NR4A1.value > threshold=1.1393861174583435
node_4192: feature_name=HLA-DPA1	HLA-DPA1.value <= threshold=1.9337060451507568
node_4193: feature_name=HOPX	HOPX.value <= threshold=-0.46209463477134705
Class: COVID-19 Critical	
Rules_304	passed counts:15
node_0: feature_name=LTB	LTB.value <= threshold=0.994031548500061
node_1: feature_name=MTRNR2L2	MTRNR2L2.value <= threshold=2.8784297704696655
node_2: feature_name=TCL1A	TCL1A.value <= threshold=4.798312664031982
node_3: feature_name=MT-CO1	MT-CO1.value > threshold=0.08947191759943962
node_2521: feature_name=LTB	LTB.value <= threshold=0.6038667261600494
node_2522: feature_name=HLA-DQB1	HLA-DQB1.value > threshold=2.1053054332733154

node_3550: feature_name=XIST	XIST.value <= threshold=0.7716337237507105
node_3551: feature_name=CD79B	CD79B.value <= threshold=2.9426796436309814
node_3552: feature_name=HLA-DQB1	HLA-DQB1.value <= threshold=2.713674306869507
node_3553: feature_name=SNX9	SNX9.value > threshold=-0.5404067635536194
node_3653: feature_name=LTB	LTB.value > threshold=0.021708179265260696
node_3759: feature_name=TCL1A	TCL1A.value <= threshold=3.3921085596084595
node_3760: feature_name=HLA-DRB5	HLA-DRB5.value <= threshold=0.5265218019485474
node_3761: feature_name=ATF5	ATF5.value <= threshold=-0.32008348405361176
node_3762: feature_name=MX1	MX1.value <= threshold=0.7805783450603485
node_3763: feature_name=RBKS	RBKS.value <= threshold=4.537207365036011
Class: COVID-19 Severe	
Rules_305	passed counts:15
node_0: feature_name=LTB	LTB.value <= threshold=0.994031548500061
node_1: feature_name=MTRNR2L2	MTRNR2L2.value <= threshold=2.8784297704696655
node_2: feature_name=TCL1A	TCL1A.value <= threshold=4.798312664031982
node_3: feature_name=MT-CO1	MT-CO1.value > threshold=0.08947191759943962
node_2521: feature_name=LTB	LTB.value <= threshold=0.6038667261600494
node_2522: feature_name=HLA-DQB1	HLA-DQB1.value <= threshold=2.1053054332733154
node_2523: feature_name=SNX9	SNX9.value > threshold=-0.5346486568450928
node_2907: feature_name=NR4A1	NR4A1.value > threshold=2.5433837175369263
node_3459: feature_name=VPREB3	VPREB3.value <= threshold=4.410779237747192
node_3460: feature_name=RALGPS2	RALGPS2.value <= threshold=3.064661979675293
node_3461: feature_name=MT-CO1	MT-CO1.value <= threshold=1.7489010095596313
node_3462: feature_name=HLA-DQA2	HLA-DQA2.value > threshold=1.794841468334198
node_3502: feature_name=MARCKS	MARCKS.value <= threshold=-0.14459624886512756
node_3503: feature_name=PLA2G12A	PLA2G12A.value <= threshold=1.2089874222874641
node_3504: feature_name=TP53INP1	TP53INP1.value <= threshold=1.2963200956583023
Class: COVID-19 Critical	

Rules_306	passed counts:15
node_0: feature_name=LTB	LTB.value <= threshold=0.994031548500061
node_1: feature_name=MTRNR2L2	MTRNR2L2.value <= threshold=2.8784297704696655
node_2: feature_name=TCL1A	TCL1A.value <= threshold=4.798312664031982
node_3: feature_name=MT-CO1	MT-CO1.value > threshold=0.08947191759943962
node_2521: feature_name=LTB	LTB.value <= threshold=0.6038667261600494
node_2522: feature_name=HLA-DQB1	HLA-DQB1.value <= threshold=2.1053054332733154
node_2523: feature_name=SNX9	SNX9.value <= threshold=-0.5346486568450928
node_2524: feature_name=HLA-DPB1	HLA-DPB1.value > threshold=1.9548270106315613
node_2814: feature_name=CD79B	CD79B.value > threshold=-0.28186772763729095
node_2826: feature_name=MT-ND6	MT-ND6.value > threshold=0.17273088544607162
node_2872: feature_name=IFI44L	IFI44L.value <= threshold=-0.31519079208374023
node_2873: feature_name=RALGPS2	RALGPS2.value <= threshold=1.6798744797706604
node_2874: feature_name=MARCH1	MARCH1.value > threshold=1.8208032846450806
node_2882: feature_name=MX1	MX1.value <= threshold=1.581712245941162
Class: Healthy Control	
Rules_307	passed counts:15
node_0: feature_name=LTB	LTB.value <= threshold=0.994031548500061
node_1: feature_name=MTRNR2L2	MTRNR2L2.value <= threshold=2.8784297704696655
node_2: feature_name=TCL1A	TCL1A.value <= threshold=4.798312664031982
node_3: feature_name=MT-CO1	MT-CO1.value > threshold=0.08947191759943962
node_2521: feature_name=LTB	LTB.value <= threshold=0.6038667261600494
node_2522: feature_name=HLA-DQB1	HLA-DQB1.value <= threshold=2.1053054332733154
node_2523: feature_name=SNX9	SNX9.value <= threshold=-0.5346486568450928
node_2524: feature_name=HLA-DPB1	HLA-DPB1.value <= threshold=1.9548270106315613
node_2525: feature_name=FAM129C	FAM129C.value > threshold=3.3393388986587524
node_2721: feature_name=ADAM19	ADAM19.value > threshold=-0.29525794088840485
node_2781: feature_name=XAF1	XAF1.value > threshold=-0.4296249598264694
node_2793: feature_name=PELI1	PELI1.value > threshold=1.8806812763214111

node_2801: feature_name=HLA-DRB1	HLA-DRB1.value > threshold=0.921924501657486
node_2805: feature_name=MS4A1	MS4A1.value > threshold=2.953020453453064
node_2809: feature_name=HLA-DQA1	HLA-DQA1.value > threshold=-0.34216251969337463
node_2811: feature_name=IFI44L	IFI44L.value <= threshold=1.860201895236969
Class: Healthy Control	
Rules_308	passed counts:15
node_0: feature_name=LTB	LTB.value <= threshold=0.994031548500061
node_1: feature_name=MTRNR2L2	MTRNR2L2.value <= threshold=2.8784297704696655
node_2: feature_name=TCL1A	TCL1A.value <= threshold=4.798312664031982
node_3: feature_name=MT-CO1	MT-CO1.value > threshold=0.08947191759943962
node_2521: feature_name=LTB	LTB.value <= threshold=0.6038667261600494
node_2522: feature_name=HLA-DQB1	HLA-DQB1.value <= threshold=2.1053054332733154
node_2523: feature_name=SNX9	SNX9.value <= threshold=-0.5346486568450928
node_2524: feature_name=HLA-DPB1	HLA-DPB1.value <= threshold=1.9548270106315613
node_2525: feature_name=FAM129C	FAM129C.value > threshold=3.3393388986587524
node_2721: feature_name=ADAM19	ADAM19.value <= threshold=-0.29525794088840485
node_2722: feature_name=NR4A1	NR4A1.value > threshold=2.1566513776779175
node_2774: feature_name=HLA-DRB1	HLA-DRB1.value > threshold=0.8161951005458832
node_2776: feature_name=UTY	UTY.value <= threshold=2.9337798357009888
node_2777: feature_name=ACSL1	ACSL1.value <= threshold=1.5818637162446976
Class: Healthy Control	
Rules_309	passed counts:15
node_0: feature_name=LTB	LTB.value <= threshold=0.994031548500061
node_1: feature_name=MTRNR2L2	MTRNR2L2.value <= threshold=2.8784297704696655
node_2: feature_name=TCL1A	TCL1A.value <= threshold=4.798312664031982
node_3: feature_name=MT-CO1	MT-CO1.value > threshold=0.08947191759943962
node_2521: feature_name=LTB	LTB.value <= threshold=0.6038667261600494
node_2522: feature_name=HLA-DQB1	HLA-DQB1.value <= threshold=2.1053054332733154

node_2523: feature_name=SNX9	SNX9.value <= threshold=-0.5346486568450928
node_2524: feature_name=HLA-DPB1	HLA-DPB1.value <= threshold=1.9548270106315613
node_2525: feature_name=FAM129C	FAM129C.value <= threshold=3.3393388986587524
node_2526: feature_name=XIST	XIST.value <= threshold=3.6911416053771973
node_2527: feature_name=NR4A1	NR4A1.value <= threshold=-0.2557755410671234
node_2528: feature_name=HOPX	HOPX.value <= threshold=2.776473879814148
node_2529: feature_name=AREGB	AREGB.value <= threshold=-0.10896047577261925
node_2530: feature_name=KIAA0125	KIAA0125.value <= threshold=7.652706623077393
node_2531: feature_name=MT-CO3	MT-CO3.value > threshold=-1.3726763725280762
node_2533: feature_name=TNF	TNF.value <= threshold=3.1101887226104736
node_2534: feature_name=NFKBID	NFKBID.value > threshold=-0.39706873893737793
node_2568: feature_name=TCF4	TCF4.value <= threshold=6.289925575256348
node_2569: feature_name=CD22	CD22.value <= threshold=8.377277374267578
node_2570: feature_name=FERMT3	FERMT3.value <= threshold=2.260508179664612
node_2571: feature_name=MT-ND6	MT-ND6.value <= threshold=3.413356304168701
node_2572: feature_name=ID1	ID1.value <= threshold=1.9121195077896118
node_2573: feature_name=DAPK1	DAPK1.value <= threshold=1.6979314014315605
node_2574: feature_name=IRF4	IRF4.value <= threshold=8.765593528747559
node_2575: feature_name=PLD4	PLD4.value <= threshold=6.6784141063690186
node_2576: feature_name=GPX1	GPX1.value <= threshold=-0.9480071663856506
node_2577: feature_name=ILK	ILK.value <= threshold=-0.4419245272874832
Class: COVID-19 Critical	
Rules_310	passed counts:15
node_0: feature_name=LTB	LTB.value <= threshold=0.994031548500061
node_1: feature_name=MTRNR2L2	MTRNR2L2.value <= threshold=2.8784297704696655
node_2: feature_name=TCL1A	TCL1A.value <= threshold=4.798312664031982
node_3: feature_name=MT-CO1	MT-CO1.value <= threshold=0.08947191759943962
node_4: feature_name=NR4A1	NR4A1.value > threshold=1.0725348591804504
node_1622: feature_name=HLA-DQA2	HLA-DQA2.value <= threshold=1.9496816992759705

node_1623: feature_name=HLA-DRB5	HLA-DRB5.value <= threshold=2.715766429901123
node_1624: feature_name=HLA-DPA1	HLA-DPA1.value > threshold=1.7050301432609558
node_2056: feature_name=OAZ1	OAZ1.value <= threshold=0.456103190779686
node_2057: feature_name=HLA-DRB5	HLA-DRB5.value <= threshold=0.6839984953403473
node_2058: feature_name=IFI6	IFI6.value <= threshold=-0.5314981639385223
node_2059: feature_name=CD83	CD83.value <= threshold=2.6418607234954834
node_2060: feature_name=HLA-DRB1	HLA-DRB1.value <= threshold=1.92746239900589
node_2061: feature_name=FCRL1	FCRL1.value <= threshold=1.318458966910839
node_2062: feature_name=HLA-DPB1	HLA-DPB1.value <= threshold=2.4035574197769165
Class: COVID-19 Critical	
Rules_311	passed counts:15
node_0: feature_name=LTB	LTB.value <= threshold=0.994031548500061
node_1: feature_name=MTRNR2L2	MTRNR2L2.value <= threshold=2.8784297704696655
node_2: feature_name=TCL1A	TCL1A.value <= threshold=4.798312664031982
node_3: feature_name=MT-CO1	MT-CO1.value <= threshold=0.08947191759943962
node_4: feature_name=NR4A1	NR4A1.value > threshold=1.0725348591804504
node_1622: feature_name=HLA-DQA2	HLA-DQA2.value <= threshold=1.9496816992759705
node_1623: feature_name=HLA-DRB5	HLA-DRB5.value <= threshold=2.715766429901123
node_1624: feature_name=HLA-DPA1	HLA-DPA1.value <= threshold=1.7050301432609558
node_1625: feature_name=CD83	CD83.value <= threshold=2.783489227294922
node_1626: feature_name=XIST	XIST.value <= threshold=0.9589543007314205
node_1627: feature_name=PLD4	PLD4.value <= threshold=5.865932464599609
node_1628: feature_name=OAZ1	OAZ1.value > threshold=0.6137169003486633
node_1840: feature_name=MT-CO1	MT-CO1.value > threshold=-0.28350088000297546
node_1850: feature_name=CD83	CD83.value <= threshold=2.1227927207946777
node_1851: feature_name=MS4A1	MS4A1.value <= threshold=2.708497166633606
node_1852: feature_name=PARP14	PARP14.value <= threshold=1.3996936082839966
Class: COVID-19 Critical	

Rules_312	passed counts:15
node_0: feature_name=LTB	LTB.value <= threshold=0.994031548500061
node_1: feature_name=MTRNR2L2	MTRNR2L2.value <= threshold=2.8784297704696655
node_2: feature_name=TCL1A	TCL1A.value <= threshold=4.798312664031982
node_3: feature_name=MT-CO1	MT-CO1.value <= threshold=0.08947191759943962
node_4: feature_name=NR4A1	NR4A1.value > threshold=1.0725348591804504
node_1622: feature_name=HLA-DQA2	HLA-DQA2.value <= threshold=1.9496816992759705
node_1623: feature_name=HLA-DRB5	HLA-DRB5.value <= threshold=2.715766429901123
node_1624: feature_name=HLA-DPA1	HLA-DPA1.value <= threshold=1.7050301432609558
node_1625: feature_name=CD83	CD83.value <= threshold=2.783489227294922
node_1626: feature_name=XIST	XIST.value <= threshold=0.9589543007314205
node_1627: feature_name=PLD4	PLD4.value <= threshold=5.865932464599609
node_1628: feature_name=OAZ1	OAZ1.value <= threshold=0.6137169003486633
node_1629: feature_name=MTRNR2L2	MTRNR2L2.value <= threshold=1.6102792620658875
node_1630: feature_name=IFI6	IFI6.value > threshold=-0.8404486179351807
node_1632: feature_name=NR4A1	NR4A1.value > threshold=1.0916320085525513
node_1638: feature_name=DENND3	DENND3.value <= threshold=3.988452672958374
node_1639: feature_name=HLA-DRB1	HLA-DRB1.value > threshold=1.4092575907707214
node_1749: feature_name=HLA-DQB1	HLA-DQB1.value <= threshold=3.4929215908050537
node_1750: feature_name=RP11-685N3.1	RP11-685N3.1.value <= threshold=9.97533893585205
node_1751: feature_name=RGS1	RGS1.value > threshold=-0.5999330878257751
node_1765: feature_name=MT-ND6	MT-ND6.value <= threshold=2.4838690757751465
node_1766: feature_name=DAPK1	DAPK1.value <= threshold=4.920811414718628
node_1767: feature_name=RGS1	RGS1.value <= threshold=3.3605728149414062
node_1768: feature_name=ANO6	ANO6.value <= threshold=4.00420081615448
node_1769: feature_name=FTH1	FTH1.value <= threshold=1.6050249934196472
node_1770: feature_name=APP	APP.value <= threshold=2.417694091796875
node_1771: feature_name=ITGAX	ITGAX.value <= threshold=2.950524091720581
node_1772: feature_name=PDLIM1	PDLIM1.value <= threshold=5.856243848800659
node_1773: feature_name=RELT	RELT.value <= threshold=2.891391396522522

node_1774: feature_name=RHOB	RHOB.value > threshold=3.2315229177474976
node_1804: feature_name=ATF5	ATF5.value > threshold=-0.37370941042900085
node_1806: feature_name=HIST1H2AC	HIST1H2AC.value <= threshold=1.139873281121254
Class: COVID-19 Critical	
Rules_313	passed counts:15
node_0: feature_name=LTB	LTB.value <= threshold=0.994031548500061
node_1: feature_name=MTRNR2L2	MTRNR2L2.value <= threshold=2.8784297704696655
node_2: feature_name=TCL1A	TCL1A.value <= threshold=4.798312664031982
node_3: feature_name=MT-CO1	MT-CO1.value <= threshold=0.08947191759943962
node_4: feature_name=NR4A1	NR4A1.value <= threshold=1.0725348591804504
node_5: feature_name=HLA-DPA1	HLA-DPA1.value > threshold=1.6885469555854797
node_861: feature_name=CD83	CD83.value <= threshold=3.0563642978668213
node_862: feature_name=HLA-DQB1	HLA-DQB1.value > threshold=2.61081063747406
node_1358: feature_name=HLA-DRB5	HLA-DRB5.value > threshold=0.7542744874954224
node_1390: feature_name=HLA-DRB5	HLA-DRB5.value <= threshold=2.6244447231292725
node_1391: feature_name=XIST	XIST.value > threshold=-0.22017984092235565
node_1497: feature_name=HLA-DRB5	HLA-DRB5.value <= threshold=1.2674232721328735
node_1498: feature_name=IGJ	IGJ.value <= threshold=1.5823739767074585
node_1499: feature_name=MS4A1	MS4A1.value <= threshold=3.1170977354049683
Class: COVID-19 Severe	
Rules_314	passed counts:15
node_0: feature_name=LTB	LTB.value <= threshold=0.994031548500061
node_1: feature_name=MTRNR2L2	MTRNR2L2.value <= threshold=2.8784297704696655
node_2: feature_name=TCL1A	TCL1A.value <= threshold=4.798312664031982
node_3: feature_name=MT-CO1	MT-CO1.value <= threshold=0.08947191759943962
node_4: feature_name=NR4A1	NR4A1.value <= threshold=1.0725348591804504
node_5: feature_name=HLA-DPA1	HLA-DPA1.value > threshold=1.6885469555854797
node_861: feature_name=CD83	CD83.value <= threshold=3.0563642978668213

node_862: feature_name=HLA-DQB1	HLA-DQB1.value > threshold=2.61081063747406
node_1358: feature_name=HLA-DRB5	HLA-DRB5.value > threshold=0.7542744874954224
node_1390: feature_name=HLA-DRB5	HLA-DRB5.value <= threshold=2.6244447231292725
node_1391: feature_name=XIST	XIST.value <= threshold=-0.22017984092235565
node_1392: feature_name=MT-CO3	MT-CO3.value > threshold=0.12647542357444763
node_1472: feature_name=IFI44L	IFI44L.value <= threshold=-0.34079885482788086
node_1473: feature_name=TNFAIP3	TNFAIP3.value <= threshold=-0.0701381303369999
node_1474: feature_name=HSP90B1	HSP90B1.value <= threshold=0.9771479368209839
node_1475: feature_name=CD79A	CD79A.value <= threshold=2.6819008588790894
node_1476: feature_name=HLA-DPA1	HLA-DPA1.value > threshold=1.8044293522834778
node_1478: feature_name=BCL2A1	BCL2A1.value <= threshold=1.153512716293335
node_1479: feature_name=STAG3	STAG3.value > threshold=-0.21443584561347961
Class: COVID-19 Critical	
Rules_315	passed counts:14
node_0: feature_name=LTB	LTB.value > threshold=0.994031548500061
node_5264: feature_name=MT-CO1	MT-CO1.value > threshold=-0.4127991199493408
node_5638: feature_name=OAZ1	OAZ1.value > threshold=-0.2640867084264755
node_6112: feature_name=IFI44L	IFI44L.value <= threshold=2.5893263816833496
node_6113: feature_name=TCL1A	TCL1A.value <= threshold=4.201832294464111
node_6114: feature_name=HLA-DQA2	HLA-DQA2.value <= threshold=-0.2270243540406227
node_6115: feature_name=MTRNR2L2	MTRNR2L2.value <= threshold=2.548917293548584
node_6116: feature_name=ATF5	ATF5.value > threshold=2.041263461112976
node_6490: feature_name=HLA-DPB1	HLA-DPB1.value > threshold=1.5343759655952454
node_6508: feature_name=OAZ1	OAZ1.value <= threshold=0.21525950729846954
node_6509: feature_name=RHOB	RHOB.value <= threshold=0.046925410628318790
Class: COVID-19 Critical	
Rules_316	passed counts:14
node_0: feature_name=LTB	LTB.value > threshold=0.994031548500061

node_5264: feature_name=MT-CO1	MT-CO1.value > threshold=-0.4127991199493408
node_5638: feature_name=OAZ1	OAZ1.value > threshold=-0.2640867084264755
node_6112: feature_name=IFI44L	IFI44L.value <= threshold=2.5893263816833496
node_6113: feature_name=TCL1A	TCL1A.value <= threshold=4.201832294464111
node_6114: feature_name=HLA-DQA2	HLA-DQA2.value <= threshold=-0.2270243540406227
node_6115: feature_name=MTRNR2L2	MTRNR2L2.value <= threshold=2.548917293548584
node_6116: feature_name=ATF5	ATF5.value <= threshold=2.041263461112976
node_6117: feature_name=JUN	JUN.value > threshold=-0.616259902715683
node_6189: feature_name=MT-CO3	MT-CO3.value <= threshold=0.13483521342277527
node_6190: feature_name=XAF1	XAF1.value > threshold=2.584952235221863
node_6346: feature_name=RELT	RELT.value <= threshold=-0.33669313788414
Class: COVID-19 Critical	
Rules_317	passed counts:14
node_0: feature_name=LTB	LTB.value > threshold=0.994031548500061
node_5264: feature_name=MT-CO1	MT-CO1.value > threshold=-0.4127991199493408
node_5638: feature_name=OAZ1	OAZ1.value <= threshold=-0.2640867084264755
node_5639: feature_name=CD79B	CD79B.value <= threshold=2.2998517751693726
node_5640: feature_name=MTRNR2L2	MTRNR2L2.value <= threshold=3.5499863624572754
node_5641: feature_name=MT-CO1	MT-CO1.value > threshold=0.22567133605480194
node_5729: feature_name=MARCH1	MARCH1.value > threshold=1.5154643654823303
node_5867: feature_name=NFKBID	NFKBID.value > threshold=2.6947624683380127
node_5903: feature_name=RGCC	RGCC.value > threshold=-0.730547159910202
node_5909: feature_name=SPI1	SPI1.value <= threshold=-0.2540571391582489
node_5910: feature_name=BOD1L1	BOD1L1.value <= threshold=1.3663210272789001
Class: Healthy Control	
Rules_318	passed counts:14
node_0: feature_name=LTB	LTB.value > threshold=0.994031548500061
node_5264: feature_name=MT-CO1	MT-CO1.value > threshold=-0.4127991199493408

node_5638: feature_name=OAZ1	OAZ1.value <= threshold=-0.2640867084264755
node_5639: feature_name=CD79B	CD79B.value <= threshold=2.2998517751693726
node_5640: feature_name=MTRNR2L2	MTRNR2L2.value <= threshold=3.5499863624572754
node_5641: feature_name=MT-CO1	MT-CO1.value > threshold=0.22567133605480194
node_5729: feature_name=MARCH1	MARCH1.value <= threshold=1.5154643654823303
node_5730: feature_name=MT-CO1	MT-CO1.value <= threshold=1.7823500633239746
node_5731: feature_name=HLA-DPB1	HLA-DPB1.value <= threshold=2.0096163749694824
node_5732: feature_name=NR4A3	NR4A3.value <= threshold=3.509189486503601
node_5733: feature_name=OAZ1	OAZ1.value <= threshold=-0.4392261207103729
node_5734: feature_name=SESN3	SESN3.value <= threshold=-0.45661941170692444
node_5735: feature_name=WDR74	WDR74.value <= threshold=-0.4893670231103897
node_5736: feature_name=NR4A1	NR4A1.value <= threshold=-0.2724379748106003
node_5737: feature_name=HLA-DPA1	HLA-DPA1.value <= threshold=1.6106070280075073
node_5738: feature_name=HLA-DRB1	HLA-DRB1.value <= threshold=1.5730668902397156
Class: COVID-19 Critical	
Rules_319	passed counts:14
node_0: feature_name=LTB	LTB.value > threshold=0.994031548500061
node_5264: feature_name=MT-CO1	MT-CO1.value <= threshold=-0.4127991199493408
node_5265: feature_name=MTRNR2L2	MTRNR2L2.value <= threshold=2.4473838806152344
node_5266: feature_name=TCL1A	TCL1A.value <= threshold=5.787256956100464
node_5267: feature_name=PLD4	PLD4.value > threshold=3.544657826423645
node_5577: feature_name=MT-CO1	MT-CO1.value <= threshold=-0.6592064797878265
node_5578: feature_name=HOPX	HOPX.value > threshold=-0.47573523223400116
node_5584: feature_name=OAZ1	OAZ1.value <= threshold=0.5679030418395996
node_5585: feature_name=MS4A1	MS4A1.value <= threshold=2.869006037712097
node_5586: feature_name=MT-CO2	MT-CO2.value > threshold=-0.9090828001499176
node_5590: feature_name=PLD4	PLD4.value <= threshold=5.566271543502808
Class: COVID-19 Critical	

Rules_320	passed counts:14
node_0: feature_name=LTB	LTB.value > threshold=0.994031548500061
node_5264: feature_name=MT-CO1	MT-CO1.value <= threshold=-0.4127991199493408
node_5265: feature_name=MTRNR2L2	MTRNR2L2.value <= threshold=2.4473838806152344
node_5266: feature_name=TCL1A	TCL1A.value <= threshold=5.787256956100464
node_5267: feature_name=PLD4	PLD4.value <= threshold=3.544657826423645
node_5268: feature_name=NR4A1	NR4A1.value <= threshold=0.8490704298019409
node_5269: feature_name=LTB	LTB.value <= threshold=1.5252206921577454
node_5270: feature_name=HLA-DRB5	HLA-DRB5.value > threshold=2.5117164850234985
node_5448: feature_name=HLA-DQB1	HLA-DQB1.value > threshold=2.092399835586548
node_5456: feature_name=SNX9	SNX9.value <= threshold=2.15211021900177
node_5457: feature_name=MX1	MX1.value <= threshold=0.5428433567285538
node_5458: feature_name=CD79B	CD79B.value <= threshold=4.320064902305603
Class: COVID-19 Severe	
Rules_321	passed counts:14
node_0: feature_name=LTB	LTB.value <= threshold=0.994031548500061
node_1: feature_name=MTRNR2L2	MTRNR2L2.value > threshold=2.8784297704696655
node_5207: feature_name=MTRNR2L8	MTRNR2L8.value <= threshold=3.1915172338485718
node_5208: feature_name=MT-CYB	MT-CYB.value <= threshold=1.3929389119148254
node_5209: feature_name=SLC7A5	SLC7A5.value <= threshold=3.205444812774658
node_5210: feature_name=MT-CO2	MT-CO2.value <= threshold=2.065874218940735
node_5211: feature_name=HLA-DRB5	HLA-DRB5.value <= threshold=2.276706099510193
node_5212: feature_name=TLR2	TLR2.value <= threshold=4.805860757827759
node_5213: feature_name=XIST	XIST.value <= threshold=4.313443303108215
node_5214: feature_name=TTN	TTN.value <= threshold=7.185946702957153
node_5215: feature_name=ITGAX	ITGAX.value <= threshold=3.106598973274231
node_5216: feature_name=PLEC	PLEC.value <= threshold=3.848032832145691
node_5217: feature_name=BCL2A1	BCL2A1.value <= threshold=2.4758780002593994
node_5218: feature_name=ATG2A	ATG2A.value <= threshold=3.2491225004196167

node_5219: feature_name=MT-CO3	MT-CO3.value <= threshold=-1.363724172115326
node_5220: feature_name=CCDC141	CCDC141.value <= threshold=-0.20780528336763382
Class: COVID-19 Severe	
Rules_322	passed counts:14
node_0: feature_name=LTB	LTB.value <= threshold=0.994031548500061
node_1: feature_name=MTRNR2L2	MTRNR2L2.value <= threshold=2.8784297704696655
node_2: feature_name=TCL1A	TCL1A.value <= threshold=4.798312664031982
node_3: feature_name=MT-CO1	MT-CO1.value > threshold=0.08947191759943962
node_2521: feature_name=LTB	LTB.value <= threshold=0.6038667261600494
node_2522: feature_name=HLA-DQB1	HLA-DQB1.value > threshold=2.1053054332733154
node_3550: feature_name=XIST	XIST.value <= threshold=0.7716337237507105
node_3551: feature_name=CD79B	CD79B.value > threshold=2.9426796436309814
node_4039: feature_name=IFI44L	IFI44L.value <= threshold=1.8637521862983704
node_4040: feature_name=SNX9	SNX9.value > threshold=1.274232804775238
node_4096: feature_name=RALGPS2	RALGPS2.value > threshold=2.3599878549575806
node_4118: feature_name=SNX29P2	SNX29P2.value > threshold=0.880477637052536
node_4132: feature_name=BOD1L1	BOD1L1.value <= threshold=1.5053239464759827
node_4133: feature_name=NR4A3	NR4A3.value <= threshold=3.728569507598877
Class: Healthy Control	
Rules_323	passed counts:14
node_0: feature_name=LTB	LTB.value <= threshold=0.994031548500061
node_1: feature_name=MTRNR2L2	MTRNR2L2.value <= threshold=2.8784297704696655
node_2: feature_name=TCL1A	TCL1A.value <= threshold=4.798312664031982
node_3: feature_name=MT-CO1	MT-CO1.value > threshold=0.08947191759943962
node_2521: feature_name=LTB	LTB.value <= threshold=0.6038667261600494
node_2522: feature_name=HLA-DQB1	HLA-DQB1.value > threshold=2.1053054332733154
node_3550: feature_name=XIST	XIST.value <= threshold=0.7716337237507105
node_3551: feature_name=CD79B	CD79B.value <= threshold=2.9426796436309814

node_3552: feature_name=HLA-DQB1	HLA-DQB1.value <= threshold=2.713674306869507
node_3553: feature_name=SNX9	SNX9.value > threshold=-0.5404067635536194
node_3653: feature_name=LTB	LTB.value > threshold=0.021708179265260696
node_3759: feature_name=TCL1A	TCL1A.value > threshold=3.3921085596084595
node_3841: feature_name=NFKBIZ	NFKBIZ.value <= threshold=0.3082800954580307
node_3842: feature_name=DAPP1	DAPP1.value <= threshold=2.181488871574402
Class: Healthy Control	
Rules_324	passed counts:14
node_0: feature_name=LTB	LTB.value <= threshold=0.994031548500061
node_1: feature_name=MTRNR2L2	MTRNR2L2.value <= threshold=2.8784297704696655
node_2: feature_name=TCL1A	TCL1A.value <= threshold=4.798312664031982
node_3: feature_name=MT-CO1	MT-CO1.value > threshold=0.08947191759943962
node_2521: feature_name=LTB	LTB.value <= threshold=0.6038667261600494
node_2522: feature_name=HLA-DQB1	HLA-DQB1.value > threshold=2.1053054332733154
node_3550: feature_name=XIST	XIST.value <= threshold=0.7716337237507105
node_3551: feature_name=CD79B	CD79B.value <= threshold=2.9426796436309814
node_3552: feature_name=HLA-DQB1	HLA-DQB1.value <= threshold=2.713674306869507
node_3553: feature_name=SNX9	SNX9.value > threshold=-0.5404067635536194
node_3653: feature_name=LTB	LTB.value <= threshold=0.021708179265260696
node_3654: feature_name=HLA-DRB5	HLA-DRB5.value > threshold=-0.5633223652839661
node_3660: feature_name=NR4A1	NR4A1.value > threshold=2.2327845096588135
node_3736: feature_name=NFKBID	NFKBID.value > threshold=-0.4191274344921112
node_3738: feature_name=PARP14	PARP14.value <= threshold=-0.49970389902591705
node_3739: feature_name=LDLRAD4	LDLRAD4.value <= threshold=-0.268046572804451
node_3740: feature_name=IGLL5	IGLL5.value > threshold=-0.24249956011772156
node_3744: feature_name=HLA-DRB5	HLA-DRB5.value > threshold=-0.43410786986351013
Class: COVID-19 Critical	
Rules_325	passed counts:14

node_0: feature_name=LTB	LTB.value <= threshold=0.994031548500061
node_1: feature_name=MTRNR2L2	MTRNR2L2.value <= threshold=2.8784297704696655
node_2: feature_name=TCL1A	TCL1A.value <= threshold=4.798312664031982
node_3: feature_name=MT-CO1	MT-CO1.value > threshold=0.08947191759943962
node_2521: feature_name=LTB	LTB.value <= threshold=0.6038667261600494
node_2522: feature_name=HLA-DQB1	HLA-DQB1.value > threshold=2.1053054332733154
node_3550: feature_name=XIST	XIST.value <= threshold=0.7716337237507105
node_3551: feature_name=CD79B	CD79B.value <= threshold=2.9426796436309814
node_3552: feature_name=HLA-DQB1	HLA-DQB1.value <= threshold=2.713674306869507
node_3553: feature_name=SNX9	SNX9.value <= threshold=-0.5404067635536194
node_3554: feature_name=VPREB3	VPREB3.value <= threshold=4.4475414752960205
node_3555: feature_name=RALGPS2	RALGPS2.value > threshold=2.7017585039138794
node_3609: feature_name=MX1	MX1.value > threshold=1.5204657316207886
Class: COVID-19 Critical	
Rules_326	passed counts:14
node_0: feature_name=LTB	LTB.value <= threshold=0.994031548500061
node_1: feature_name=MTRNR2L2	MTRNR2L2.value <= threshold=2.8784297704696655
node_2: feature_name=TCL1A	TCL1A.value <= threshold=4.798312664031982
node_3: feature_name=MT-CO1	MT-CO1.value > threshold=0.08947191759943962
node_2521: feature_name=LTB	LTB.value <= threshold=0.6038667261600494
node_2522: feature_name=HLA-DQB1	HLA-DQB1.value <= threshold=2.1053054332733154
node_2523: feature_name=SNX9	SNX9.value > threshold=-0.5346486568450928
node_2907: feature_name=NR4A1	NR4A1.value <= threshold=2.5433837175369263
node_2908: feature_name=XIST	XIST.value <= threshold=0.7664470989257097
node_2909: feature_name=CD79B	CD79B.value > threshold=2.9326847791671753
node_3353: feature_name=VPREB3	VPREB3.value <= threshold=3.0226117372512817
node_3354: feature_name=CD83	CD83.value > threshold=1.9074636101722717
node_3388: feature_name=BOD1L1	BOD1L1.value <= threshold=-0.4742381125688553
node_3389: feature_name=MT-ND6	MT-ND6.value > threshold=0.3542506694793701

node_3399: feature_name=RALGPS2	RALGPS2.value > threshold=1.4945479035377502
node_3401: feature_name=BACH1	BACH1.value <= threshold=2.4402166604995728
Class: Healthy Control	
Rules_327	passed counts:14
node_0: feature_name=LTB	LTB.value <= threshold=0.994031548500061
node_1: feature_name=MTRNR2L2	MTRNR2L2.value <= threshold=2.8784297704696655
node_2: feature_name=TCL1A	TCL1A.value <= threshold=4.798312664031982
node_3: feature_name=MT-CO1	MT-CO1.value <= threshold=0.08947191759943962
node_4: feature_name=NR4A1	NR4A1.value > threshold=1.0725348591804504
node_1622: feature_name=HLA-DQA2	HLA-DQA2.value <= threshold=1.9496816992759705
node_1623: feature_name=HLA-DRB5	HLA-DRB5.value <= threshold=2.715766429901123
node_1624: feature_name=HLA-DPA1	HLA-DPA1.value <= threshold=1.7050301432609558
node_1625: feature_name=CD83	CD83.value <= threshold=2.783489227294922
node_1626: feature_name=XIST	XIST.value <= threshold=0.9589543007314205
node_1627: feature_name=PLD4	PLD4.value <= threshold=5.865932464599609
node_1628: feature_name=OAZ1	OAZ1.value <= threshold=0.6137169003486633
node_1629: feature_name=MTRNR2L2	MTRNR2L2.value <= threshold=1.6102792620658875
node_1630: feature_name=IFI6	IFI6.value > threshold=-0.8404486179351807
node_1632: feature_name=NR4A1	NR4A1.value > threshold=1.0916320085525513
node_1638: feature_name=DENND3	DENND3.value <= threshold=3.988452672958374
node_1639: feature_name=HLA-DRB1	HLA-DRB1.value > threshold=1.4092575907707214
node_1749: feature_name=HLA-DQB1	HLA-DQB1.value <= threshold=3.4929215908050537
node_1750: feature_name=RP11-685N3.1	RP11-685N3.1.value <= threshold=9.97533893585205
node_1751: feature_name=RGS1	RGS1.value > threshold=-0.5999330878257751
node_1765: feature_name=MT-ND6	MT-ND6.value <= threshold=2.4838690757751465
node_1766: feature_name=DAPK1	DAPK1.value <= threshold=4.920811414718628
node_1767: feature_name=RGS1	RGS1.value <= threshold=3.3605728149414062
node_1768: feature_name=ANO6	ANO6.value <= threshold=4.00420081615448
node_1769: feature_name=FTH1	FTH1.value <= threshold=1.6050249934196472

node_1770: feature_name=APP	APP.value > threshold=2.417694091796875
node_1818: feature_name=CXorf57	CXorf57.value <= threshold=-0.21061210334300995
node_1819: feature_name=TLN1	TLN1.value <= threshold=0.9299276471138
Class: COVID-19 Critical	
Rules_328	passed counts:14
node_0: feature_name=LTB	LTB.value <= threshold=0.994031548500061
node_1: feature_name=MTRNR2L2	MTRNR2L2.value <= threshold=2.8784297704696655
node_2: feature_name=TCL1A	TCL1A.value <= threshold=4.798312664031982
node_3: feature_name=MT-CO1	MT-CO1.value <= threshold=0.08947191759943962
node_4: feature_name=NR4A1	NR4A1.value <= threshold=1.0725348591804504
node_5: feature_name=HLA-DPA1	HLA-DPA1.value > threshold=1.6885469555854797
node_861: feature_name=CD83	CD83.value <= threshold=3.0563642978668213
node_862: feature_name=HLA-DQB1	HLA-DQB1.value > threshold=2.61081063747406
node_1358: feature_name=HLA-DRB5	HLA-DRB5.value > threshold=0.7542744874954224
node_1390: feature_name=HLA-DRB5	HLA-DRB5.value <= threshold=2.6244447231292725
node_1391: feature_name=XIST	XIST.value <= threshold=-0.22017984092235565
node_1392: feature_name=MT-CO3	MT-CO3.value > threshold=0.12647542357444763
node_1472: feature_name=IFI44L	IFI44L.value <= threshold=-0.34079885482788086
node_1473: feature_name=TNFAIP3	TNFAIP3.value > threshold=-0.0701381303369999
node_1491: feature_name=GRAP2	GRAP2.value <= threshold=-0.36840446293354034
Class: COVID-19 Critical	
Rules_329	passed counts:14
node_0: feature_name=LTB	LTB.value <= threshold=0.994031548500061
node_1: feature_name=MTRNR2L2	MTRNR2L2.value <= threshold=2.8784297704696655
node_2: feature_name=TCL1A	TCL1A.value <= threshold=4.798312664031982
node_3: feature_name=MT-CO1	MT-CO1.value <= threshold=0.08947191759943962
node_4: feature_name=NR4A1	NR4A1.value <= threshold=1.0725348591804504
node_5: feature_name=HLA-DPA1	HLA-DPA1.value > threshold=1.6885469555854797

node_861: feature_name=CD83	CD83.value <= threshold=3.0563642978668213
node_862: feature_name=HLA-DQB1	HLA-DQB1.value <= threshold=2.61081063747406
node_863: feature_name=MTRNR2L2	MTRNR2L2.value <= threshold=2.0919997692108154
node_864: feature_name=BCL2A1	BCL2A1.value <= threshold=1.852618932723999
node_865: feature_name=CD79B	CD79B.value <= threshold=4.352142333984375
node_866: feature_name=TNFAIP3	TNFAIP3.value > threshold=-0.0449496041983366
node_1236: feature_name=HLA-DRB5	HLA-DRB5.value > threshold=1.710871696472168
node_1268: feature_name=MT-CO1	MT-CO1.value > threshold=-0.8004095554351807
node_1282: feature_name=HLA-DQA2	HLA-DQA2.value <= threshold=-0.1890905275940895
node_1283: feature_name=OAZ1	OAZ1.value > threshold=-0.14406292885541916
node_1289: feature_name=HOPX	HOPX.value <= threshold=-0.474257692694664
node_1290: feature_name=MIR24-2	MIR24-2.value <= threshold=-0.5045886039733887
Class: COVID-19 Critical	
Rules_330	passed counts:14
node_0: feature_name=LTB	LTB.value <= threshold=0.994031548500061
node_1: feature_name=MTRNR2L2	MTRNR2L2.value <= threshold=2.8784297704696655
node_2: feature_name=TCL1A	TCL1A.value <= threshold=4.798312664031982
node_3: feature_name=MT-CO1	MT-CO1.value <= threshold=0.08947191759943962
node_4: feature_name=NR4A1	NR4A1.value <= threshold=1.0725348591804504
node_5: feature_name=HLA-DPA1	HLA-DPA1.value <= threshold=1.6885469555854797
node_6: feature_name=HLA-DRB5	HLA-DRB5.value <= threshold=2.1886194944381714
node_7: feature_name=XIST	XIST.value <= threshold=-0.12328458577394485
node_8: feature_name=SYNE1	SYNE1.value > threshold=-0.7835594415664673
node_16: feature_name=MTRNR2L2	MTRNR2L2.value <= threshold=2.4483247995376587
node_17: feature_name=HLA-DQA2	HLA-DQA2.value <= threshold=2.227178931236267
node_18: feature_name=NR4A1	NR4A1.value <= threshold=-0.21380723267793655
node_19: feature_name=IRAK3	IRAK3.value <= threshold=4.27159571647644
node_20: feature_name=IFIT2	IFIT2.value <= threshold=7.8175740242004395
node_21: feature_name=CD79A	CD79A.value > threshold=2.857242226600647

node_375: feature_name=LTB	LTB.value <= threshold=0.7405790388584137
node_376: feature_name=NFKBIZ	NFKBIZ.value <= threshold=1.7718515396118164
node_377: feature_name=OAZ1	OAZ1.value <= threshold=1.3302325010299683
node_378: feature_name=RHOB	RHOB.value <= threshold=3.0459729433059692
node_379: feature_name=MT-CO3	MT-CO3.value <= threshold=1.6619641780853271
node_380: feature_name=MTRNR2L2	MTRNR2L2.value <= threshold=2.1229071617126465
node_381: feature_name=MAP3K2	MAP3K2.value <= threshold=3.626914143562317
node_382: feature_name=LDLR	LDLR.value <= threshold=2.571519374847412
node_383: feature_name=HLA-DRB1	HLA-DRB1.value > threshold=-0.7626054286956787
node_385: feature_name=CD79A	CD79A.value > threshold=2.857717990875244
node_387: feature_name=FCRL1	FCRL1.value <= threshold=6.850295543670654
node_388: feature_name=PDLIM1	PDLIM1.value <= threshold=4.4097795486450195
node_389: feature_name=MARCH1	MARCH1.value <= threshold=-0.35520173609256744
node_390: feature_name=ICAM1	ICAM1.value > threshold=-0.33725936710834503
node_404: feature_name=APP	APP.value <= threshold=-0.37299394607543945
node_405: feature_name=HLA-DPB1	HLA-DPB1.value <= threshold=1.9074877500534058
node_406: feature_name=HSP90B1	HSP90B1.value > threshold=-1.2896496653556824
Class: COVID-19 Critical	
Rules_331	passed counts:14
node_0: feature_name=LTB	LTB.value <= threshold=0.994031548500061
node_1: feature_name=MTRNR2L2	MTRNR2L2.value <= threshold=2.8784297704696655
node_2: feature_name=TCL1A	TCL1A.value <= threshold=4.798312664031982
node_3: feature_name=MT-CO1	MT-CO1.value <= threshold=0.08947191759943962
node_4: feature_name=NR4A1	NR4A1.value <= threshold=1.0725348591804504
node_5: feature_name=HLA-DPA1	HLA-DPA1.value <= threshold=1.6885469555854797
node_6: feature_name=HLA-DRB5	HLA-DRB5.value <= threshold=2.1886194944381714
node_7: feature_name=XIST	XIST.value <= threshold=-0.12328458577394485
node_8: feature_name=SYNE1	SYNE1.value > threshold=-0.7835594415664673
node_16: feature_name=MTRNR2L2	MTRNR2L2.value <= threshold=2.4483247995376587

node_17: feature_name=HLA-DQA2	HLA-DQA2.value <= threshold=2.227178931236267
node_18: feature_name=NR4A1	NR4A1.value <= threshold=-0.21380723267793655
node_19: feature_name=IRAK3	IRAK3.value <= threshold=4.27159571647644
node_20: feature_name=IFIT2	IFIT2.value <= threshold=7.8175740242004395
node_21: feature_name=CD79A	CD79A.value <= threshold=2.857242226600647
node_22: feature_name=HIST1H2AC	HIST1H2AC.value <= threshold=5.077470302581787
node_23: feature_name=FTH1	FTH1.value > threshold=0.4158056080341339
node_285: feature_name=EGR1	EGR1.value <= threshold=4.240467071533203
node_286: feature_name=HLA-DRB1	HLA-DRB1.value > threshold=-0.7553382813930511
node_290: feature_name=TLR2	TLR2.value <= threshold=2.879140257835388
node_291: feature_name=SYNE1	SYNE1.value <= threshold=2.323199510574341
node_292: feature_name=BCL2A1	BCL2A1.value <= threshold=1.8940600156784058
node_293: feature_name=RALGPS2	RALGPS2.value <= threshold=4.808189153671265
node_294: feature_name=IGLL5	IGLL5.value > threshold=4.116017818450928
node_346: feature_name=JUN	JUN.value <= threshold=1.1506463885307312
node_347: feature_name=HLA-DPB1	HLA-DPB1.value > threshold=0.8467520177364349
Class: COVID-19 Critical	
Rules_332	passed counts:14
node_0: feature_name=LTB	LTB.value <= threshold=0.994031548500061
node_1: feature_name=MTRNR2L2	MTRNR2L2.value <= threshold=2.8784297704696655
node_2: feature_name=TCL1A	TCL1A.value <= threshold=4.798312664031982
node_3: feature_name=MT-CO1	MT-CO1.value <= threshold=0.08947191759943962
node_4: feature_name=NR4A1	NR4A1.value <= threshold=1.0725348591804504
node_5: feature_name=HLA-DPA1	HLA-DPA1.value <= threshold=1.6885469555854797
node_6: feature_name=HLA-DRB5	HLA-DRB5.value <= threshold=2.1886194944381714
node_7: feature_name=XIST	XIST.value <= threshold=-0.12328458577394485
node_8: feature_name=SYNE1	SYNE1.value > threshold=-0.7835594415664673
node_16: feature_name=MTRNR2L2	MTRNR2L2.value <= threshold=2.4483247995376587
node_17: feature_name=HLA-DQA2	HLA-DQA2.value <= threshold=2.227178931236267

node_18: feature_name=NR4A1	NR4A1.value <= threshold=-0.21380723267793655
node_19: feature_name=IRAK3	IRAK3.value <= threshold=4.27159571647644
node_20: feature_name=IFIT2	IFIT2.value <= threshold=7.8175740242004395
node_21: feature_name=CD79A	CD79A.value <= threshold=2.857242226600647
node_22: feature_name=HIST1H2AC	HIST1H2AC.value <= threshold=5.077470302581787
node_23: feature_name=FTH1	FTH1.value <= threshold=0.4158056080341339
node_24: feature_name=ATF3	ATF3.value <= threshold=3.8028353452682495
node_25: feature_name=OASL	OASL.value <= threshold=3.387580633163452
node_26: feature_name=MT-CO1	MT-CO1.value > threshold=-0.7942646145820618
node_74: feature_name=HOPX	HOPX.value <= threshold=0.8682331740856171
node_75: feature_name=RGS2	RGS2.value <= threshold=1.4589954018592834
node_76: feature_name=AHR	AHR.value <= threshold=3.870481848716736
node_77: feature_name=IGJ	IGJ.value <= threshold=4.902882814407349
node_78: feature_name=CD79B	CD79B.value <= threshold=4.87716269493103
node_79: feature_name=HLA-DQB1	HLA-DQB1.value <= threshold=3.6925491094589233
node_80: feature_name=HLA-DRB5	HLA-DRB5.value > threshold=2.0926843881607056
node_204: feature_name=DAPP1	DAPP1.value > threshold=-0.3013480007648468
Class: COVID-19 Critical	
Rules_333	passed counts:14
node_0: feature_name=LTB	LTB.value <= threshold=0.994031548500061
node_1: feature_name=MTRNR2L2	MTRNR2L2.value <= threshold=2.8784297704696655
node_2: feature_name=TCL1A	TCL1A.value <= threshold=4.798312664031982
node_3: feature_name=MT-CO1	MT-CO1.value <= threshold=0.08947191759943962
node_4: feature_name=NR4A1	NR4A1.value <= threshold=1.0725348591804504
node_5: feature_name=HLA-DPA1	HLA-DPA1.value <= threshold=1.6885469555854797
node_6: feature_name=HLA-DRB5	HLA-DRB5.value <= threshold=2.1886194944381714
node_7: feature_name=XIST	XIST.value <= threshold=-0.12328458577394485
node_8: feature_name=SYNE1	SYNE1.value > threshold=-0.7835594415664673
node_16: feature_name=MTRNR2L2	MTRNR2L2.value <= threshold=2.4483247995376587

node_17: feature_name=HLA-DQA2	HLA-DQA2.value <= threshold=2.227178931236267
node_18: feature_name=NR4A1	NR4A1.value <= threshold=-0.21380723267793655
node_19: feature_name=IRAK3	IRAK3.value <= threshold=4.27159571647644
node_20: feature_name=IFIT2	IFIT2.value <= threshold=7.8175740242004395
node_21: feature_name=CD79A	CD79A.value <= threshold=2.857242226600647
node_22: feature_name=HIST1H2AC	HIST1H2AC.value <= threshold=5.077470302581787
node_23: feature_name=FTH1	FTH1.value <= threshold=0.4158056080341339
node_24: feature_name=ATF3	ATF3.value <= threshold=3.8028353452682495
node_25: feature_name=OASL	OASL.value <= threshold=3.387580633163452
node_26: feature_name=MT-CO1	MT-CO1.value > threshold=-0.7942646145820618
node_74: feature_name=HOPX	HOPX.value <= threshold=0.8682331740856171
node_75: feature_name=RGS2	RGS2.value <= threshold=1.4589954018592834
node_76: feature_name=AHR	AHR.value <= threshold=3.870481848716736
node_77: feature_name=IGJ	IGJ.value <= threshold=4.902882814407349
node_78: feature_name=CD79B	CD79B.value <= threshold=4.87716269493103
node_79: feature_name=HLA-DQB1	HLA-DQB1.value <= threshold=3.6925491094589233
node_80: feature_name=HLA-DRB5	HLA-DRB5.value <= threshold=2.0926843881607056
node_81: feature_name=MCTP1	MCTP1.value <= threshold=4.010472774505615
node_82: feature_name=GNG11	GNG11.value <= threshold=3.627470850944519
node_83: feature_name=HSP90B1	HSP90B1.value <= threshold=2.0835131406784058
node_84: feature_name=ATF3	ATF3.value <= threshold=1.1142374873161316
node_85: feature_name=MT-ND6	MT-ND6.value <= threshold=2.7328014373779297
node_86: feature_name=COTL1	COTL1.value > threshold=-1.6524587869644165
node_90: feature_name=MT-CYB	MT-CYB.value <= threshold=1.099629819393158
node_91: feature_name=RGCC	RGCC.value <= threshold=1.6908138990402222
node_92: feature_name=ILK	ILK.value <= threshold=3.9497616291046143
node_93: feature_name=CTD-2035E11.3	CTD-2035E11.3.value <= threshold=2.4543306827545166
node_94: feature_name=S100A6	S100A6.value <= threshold=0.2867250442504883
node_95: feature_name=HLA-DRB5	HLA-DRB5.value > threshold=-0.9496813416481018
node_99: feature_name=MT-CO3	MT-CO3.value <= threshold=-1.55654776096344

node_100: feature_name=MX1	MX1.value > threshold=-0.46766048669815063
Class: COVID-19 Critical	
Rules_334	passed counts:13
node_0: feature_name=LTB	LTB.value > threshold=0.994031548500061
node_5264: feature_name=MT-CO1	MT-CO1.value > threshold=-0.4127991199493408
node_5638: feature_name=OAZ1	OAZ1.value > threshold=-0.2640867084264755
node_6112: feature_name=IFI44L	IFI44L.value <= threshold=2.5893263816833496
node_6113: feature_name=TCL1A	TCL1A.value <= threshold=4.201832294464111
node_6114: feature_name=HLA-DQA2	HLA-DQA2.value > threshold=-0.2270243540406227
node_6542: feature_name=CD79B	CD79B.value <= threshold=3.096232771873474
node_6543: feature_name=NR4A1	NR4A1.value <= threshold=1.421015739440918
node_6544: feature_name=LTB	LTB.value <= threshold=1.359755277633667
node_6545: feature_name=NFKBID	NFKBID.value <= threshold=1.4294711351394653
node_6546: feature_name=HLA-DRB1	HLA-DRB1.value > threshold=1.0398632287979126
node_6556: feature_name=CD79B	CD79B.value > threshold=-0.2919560521841049
node_6582: feature_name=PELI1	PELI1.value > threshold=-0.6156984269618988
node_6602: feature_name=MT-CO3	MT-CO3.value <= threshold=0.46141014993190765
node_6603: feature_name=NFKBIZ	NFKBIZ.value > threshold=0.6470938920974731
node_6629: feature_name=HLA-DQA2	HLA-DQA2.value > threshold=-0.21238847821950912
Class: COVID-19 Critical	
Rules_335	passed counts:13
node_0: feature_name=LTB	LTB.value > threshold=0.994031548500061
node_5264: feature_name=MT-CO1	MT-CO1.value > threshold=-0.4127991199493408
node_5638: feature_name=OAZ1	OAZ1.value <= threshold=-0.2640867084264755
node_5639: feature_name=CD79B	CD79B.value <= threshold=2.2998517751693726
node_5640: feature_name=MTRNR2L2	MTRNR2L2.value <= threshold=3.5499863624572754
node_5641: feature_name=MT-CO1	MT-CO1.value > threshold=0.22567133605480194
node_5729: feature_name=MARCH1	MARCH1.value <= threshold=1.5154643654823303

node_5730: feature_name=MT-CO1	MT-CO1.value <= threshold=1.7823500633239746
node_5731: feature_name=HLA-DPB1	HLA-DPB1.value <= threshold=2.0096163749694824
node_5732: feature_name=NR4A3	NR4A3.value <= threshold=3.509189486503601
node_5733: feature_name=OAZ1	OAZ1.value <= threshold=-0.4392261207103729
node_5734: feature_name=SESN3	SESN3.value <= threshold=-0.45661941170692444
node_5735: feature_name=WDR74	WDR74.value <= threshold=-0.4893670231103897
node_5736: feature_name=NR4A1	NR4A1.value > threshold=-0.2724379748106003
node_5754: feature_name=NAMPT	NAMPT.value <= threshold=1.352931261062622
node_5755: feature_name=HSP90B1	HSP90B1.value <= threshold=1.765532672405243
Class: Healthy Control	
Rules_336	passed counts:13
node_0: feature_name=LTB	LTB.value > threshold=0.994031548500061
node_5264: feature_name=MT-CO1	MT-CO1.value > threshold=-0.4127991199493408
node_5638: feature_name=OAZ1	OAZ1.value <= threshold=-0.2640867084264755
node_5639: feature_name=CD79B	CD79B.value <= threshold=2.2998517751693726
node_5640: feature_name=MTRNR2L2	MTRNR2L2.value <= threshold=3.5499863624572754
node_5641: feature_name=MT-CO1	MT-CO1.value <= threshold=0.22567133605480194
node_5642: feature_name=RGS2	RGS2.value <= threshold=1.2649686336517334
node_5643: feature_name=TCL1A	TCL1A.value <= threshold=4.771023273468018
node_5644: feature_name=IGJ	IGJ.value <= threshold=3.91206431388855
node_5645: feature_name=AREGB	AREGB.value <= threshold=1.5142876282334328
node_5646: feature_name=HLA-DRB1	HLA-DRB1.value > threshold=-0.6253005266189575
node_5648: feature_name=ITM2C	ITM2C.value <= threshold=3.2904402017593384
node_5649: feature_name=PLA2G12A	PLA2G12A.value <= threshold=3.833786725997925
node_5650: feature_name=RGCC	RGCC.value > threshold=-0.10016544535756111
node_5680: feature_name=DENND3	DENND3.value <= threshold=1.5598239302635193
node_5681: feature_name=NFKBIZ	NFKBIZ.value > threshold=-0.8164795935153961
Class: COVID-19 Critical	

Rules_337	passed counts:13
node_0: feature_name=LTB	LTB.value <= threshold=0.994031548500061
node_1: feature_name=MTRNR2L2	MTRNR2L2.value <= threshold=2.8784297704696655
node_2: feature_name=TCL1A	TCL1A.value <= threshold=4.798312664031982
node_3: feature_name=MT-CO1	MT-CO1.value > threshold=0.08947191759943962
node_2521: feature_name=LTB	LTB.value > threshold=0.6038667261600494
node_4173: feature_name=OAZ1	OAZ1.value > threshold=0.143283873796463
node_4615: feature_name=NR4A1	NR4A1.value > threshold=0.6621004343032837
node_4817: feature_name=HSP90B1	HSP90B1.value <= threshold=1.1699737310409546
node_4818: feature_name=XAF1	XAF1.value <= threshold=2.6752071380615234
node_4819: feature_name=SNX9	SNX9.value <= threshold=2.939147114753723
node_4820: feature_name=HLA-DQA2	HLA-DQA2.value > threshold=2.9822239875793457
node_4874: feature_name=MARCH1	MARCH1.value > threshold=-0.3389042466878891
node_4882: feature_name=RHOB	RHOB.value <= threshold=3.008488178253174
Class: Healthy Control	
Rules_338	passed counts:13
node_0: feature_name=LTB	LTB.value <= threshold=0.994031548500061
node_1: feature_name=MTRNR2L2	MTRNR2L2.value <= threshold=2.8784297704696655
node_2: feature_name=TCL1A	TCL1A.value <= threshold=4.798312664031982
node_3: feature_name=MT-CO1	MT-CO1.value > threshold=0.08947191759943962
node_2521: feature_name=LTB	LTB.value > threshold=0.6038667261600494
node_4173: feature_name=OAZ1	OAZ1.value > threshold=0.143283873796463
node_4615: feature_name=NR4A1	NR4A1.value <= threshold=0.6621004343032837
node_4616: feature_name=NFKBID	NFKBID.value <= threshold=3.283741593360901
node_4617: feature_name=HLA-DQA1	HLA-DQA1.value > threshold=2.3435752391815186
node_4719: feature_name=MX1	MX1.value <= threshold=1.851993978023529
node_4720: feature_name=JUN	JUN.value > threshold=-0.5025404393672943
node_4740: feature_name=IRF8	IRF8.value <= threshold=3.838419556617737
node_4741: feature_name=BACH1	BACH1.value > threshold=0.9568033516407013

node_4769: feature_name=TCF4	TCF4.value <= threshold=-0.23278788477182388
node_4770: feature_name=FERMT3	FERMT3.value <= threshold=1.1583285331726074
node_4771: feature_name=MS4A1	MS4A1.value > threshold=2.3401901721954346
node_4777: feature_name=HLA-DPB1	HLA-DPB1.value <= threshold=2.264760732650757
Class: COVID-19 Critical	
Rules_339	passed counts:13
node_0: feature_name=LTB	LTB.value <= threshold=0.994031548500061
node_1: feature_name=MTRNR2L2	MTRNR2L2.value <= threshold=2.8784297704696655
node_2: feature_name=TCL1A	TCL1A.value <= threshold=4.798312664031982
node_3: feature_name=MT-CO1	MT-CO1.value > threshold=0.08947191759943962
node_2521: feature_name=LTB	LTB.value > threshold=0.6038667261600494
node_4173: feature_name=OAZ1	OAZ1.value <= threshold=0.143283873796463
node_4174: feature_name=PLD4	PLD4.value > threshold=2.921932101249695
node_4558: feature_name=HLA-DQA2	HLA-DQA2.value <= threshold=1.5485953092575073
node_4559: feature_name=COTL1	COTL1.value > threshold=-0.42844848334789276
node_4573: feature_name=CD83	CD83.value <= threshold=1.9224650263786316
node_4574: feature_name=ICAM1	ICAM1.value > threshold=-0.3564278930425644
node_4586: feature_name=MARCKS	MARCKS.value > threshold=-0.035114869475364685
node_4590: feature_name=BCL2A1	BCL2A1.value > threshold=-0.648458868265152
Class: COVID-19 Critical	
Rules_340	passed counts:13
node_0: feature_name=LTB	LTB.value <= threshold=0.994031548500061
node_1: feature_name=MTRNR2L2	MTRNR2L2.value <= threshold=2.8784297704696655
node_2: feature_name=TCL1A	TCL1A.value <= threshold=4.798312664031982
node_3: feature_name=MT-CO1	MT-CO1.value > threshold=0.08947191759943962
node_2521: feature_name=LTB	LTB.value <= threshold=0.6038667261600494
node_2522: feature_name=HLA-DQB1	HLA-DQB1.value <= threshold=2.1053054332733154
node_2523: feature_name=SNX9	SNX9.value > threshold=-0.5346486568450928

node_2907: feature_name=NR4A1	NR4A1.value > threshold=2.5433837175369263
node_3459: feature_name=VPREB3	VPREB3.value > threshold=4.410779237747192
node_3545: feature_name=ZNF10	ZNF10.value <= threshold=2.669158458709717
Class: Healthy Control	
Rules_341	passed counts:13
node_0: feature_name=LTB	LTB.value <= threshold=0.994031548500061
node_1: feature_name=MTRNR2L2	MTRNR2L2.value <= threshold=2.8784297704696655
node_2: feature_name=TCL1A	TCL1A.value <= threshold=4.798312664031982
node_3: feature_name=MT-CO1	MT-CO1.value > threshold=0.08947191759943962
node_2521: feature_name=LTB	LTB.value <= threshold=0.6038667261600494
node_2522: feature_name=HLA-DQB1	HLA-DQB1.value <= threshold=2.1053054332733154
node_2523: feature_name=SNX9	SNX9.value > threshold=-0.5346486568450928
node_2907: feature_name=NR4A1	NR4A1.value <= threshold=2.5433837175369263
node_2908: feature_name=XIST	XIST.value <= threshold=0.7664470989257097
node_2909: feature_name=CD79B	CD79B.value <= threshold=2.9326847791671753
node_2910: feature_name=NR4A1	NR4A1.value > threshold=-0.2875557690858841
node_3096: feature_name=NR4A3	NR4A3.value <= threshold=3.314968705177307
node_3097: feature_name=VPREB3	VPREB3.value > threshold=3.3549938201904297
node_3285: feature_name=OAZ1	OAZ1.value > threshold=0.11267071589827538
node_3309: feature_name=SNX9	SNX9.value <= threshold=1.7356606721878052
node_3310: feature_name=RALGPS2	RALGPS2.value > threshold=2.1317856311798096
Class: Healthy Control	
Rules_342	passed counts:13
node_0: feature_name=LTB	LTB.value <= threshold=0.994031548500061
node_1: feature_name=MTRNR2L2	MTRNR2L2.value <= threshold=2.8784297704696655
node_2: feature_name=TCL1A	TCL1A.value <= threshold=4.798312664031982
node_3: feature_name=MT-CO1	MT-CO1.value <= threshold=0.08947191759943962
node_4: feature_name=NR4A1	NR4A1.value > threshold=1.0725348591804504

node_1622: feature_name=HLA-DQA2	HLA-DQA2.value > threshold=1.9496816992759705
node_2350: feature_name=BCL2A1	BCL2A1.value <= threshold=1.1322240233421326
node_2351: feature_name=CD83	CD83.value > threshold=2.3162450790405273
node_2391: feature_name=CD79B	CD79B.value <= threshold=1.7111427783966064
node_2392: feature_name=TTN	TTN.value > threshold=-0.28553803265094757
node_2430: feature_name=LTB	LTB.value > threshold=0.3538805693387985
node_2440: feature_name=NAIP	NAIP.value <= threshold=0.8108409643173218
Class: COVID-19 Critical	
Rules_343	passed counts:13
node_0: feature_name=LTB	LTB.value <= threshold=0.994031548500061
node_1: feature_name=MTRNR2L2	MTRNR2L2.value <= threshold=2.8784297704696655
node_2: feature_name=TCL1A	TCL1A.value <= threshold=4.798312664031982
node_3: feature_name=MT-CO1	MT-CO1.value <= threshold=0.08947191759943962
node_4: feature_name=NR4A1	NR4A1.value > threshold=1.0725348591804504
node_1622: feature_name=HLA-DQA2	HLA-DQA2.value <= threshold=1.9496816992759705
node_1623: feature_name=HLA-DRB5	HLA-DRB5.value <= threshold=2.715766429901123
node_1624: feature_name=HLA-DPA1	HLA-DPA1.value > threshold=1.7050301432609558
node_2056: feature_name=OAZ1	OAZ1.value > threshold=0.456103190779686
node_2266: feature_name=XAF1	XAF1.value <= threshold=-0.4431648850440979
node_2267: feature_name=MT-CO1	MT-CO1.value <= threshold=-0.3505740910768509
node_2268: feature_name=HLA-DRB1	HLA-DRB1.value <= threshold=2.1576695442199707
node_2269: feature_name=MARCH1	MARCH1.value > threshold=1.492592692375183
node_2283: feature_name=FAM129C	FAM129C.value > threshold=-0.2708045244216919
Class: Healthy Control	
Rules_344	passed counts:13
node_0: feature_name=LTB	LTB.value <= threshold=0.994031548500061
node_1: feature_name=MTRNR2L2	MTRNR2L2.value <= threshold=2.8784297704696655
node_2: feature_name=TCL1A	TCL1A.value <= threshold=4.798312664031982

node_3: feature_name=MT-CO1	MT-CO1.value <= threshold=0.08947191759943962
node_4: feature_name=NR4A1	NR4A1.value > threshold=1.0725348591804504
node_1622: feature_name=HLA-DQA2	HLA-DQA2.value <= threshold=1.9496816992759705
node_1623: feature_name=HLA-DRB5	HLA-DRB5.value <= threshold=2.715766429901123
node_1624: feature_name=HLA-DPA1	HLA-DPA1.value <= threshold=1.7050301432609558
node_1625: feature_name=CD83	CD83.value <= threshold=2.783489227294922
node_1626: feature_name=XIST	XIST.value <= threshold=0.9589543007314205
node_1627: feature_name=PLD4	PLD4.value <= threshold=5.865932464599609
node_1628: feature_name=OAZ1	OAZ1.value <= threshold=0.6137169003486633
node_1629: feature_name=MTRNR2L2	MTRNR2L2.value <= threshold=1.6102792620658875
node_1630: feature_name=IFI6	IFI6.value > threshold=-0.8404486179351807
node_1632: feature_name=NR4A1	NR4A1.value > threshold=1.0916320085525513
node_1638: feature_name=DENND3	DENND3.value <= threshold=3.988452672958374
node_1639: feature_name=HLA-DRB1	HLA-DRB1.value <= threshold=1.4092575907707214
node_1640: feature_name=HLA-DPA1	HLA-DPA1.value > threshold=1.2675696015357971
node_1708: feature_name=CYBB	CYBB.value <= threshold=1.8550206422805786
node_1709: feature_name=JUN	JUN.value <= threshold=-0.04836222808808088
node_1710: feature_name=MARCH1	MARCH1.value > threshold=-0.3826435059309006
node_1716: feature_name=CD83	CD83.value <= threshold=2.438913345336914
node_1717: feature_name=MX1	MX1.value <= threshold=1.6370581984519958
Class: COVID-19 Critical	
Rules_345	passed counts:13
node_0: feature_name=LTB	LTB.value <= threshold=0.994031548500061
node_1: feature_name=MTRNR2L2	MTRNR2L2.value <= threshold=2.8784297704696655
node_2: feature_name=TCL1A	TCL1A.value <= threshold=4.798312664031982
node_3: feature_name=MT-CO1	MT-CO1.value <= threshold=0.08947191759943962
node_4: feature_name=NR4A1	NR4A1.value <= threshold=1.0725348591804504
node_5: feature_name=HLA-DPA1	HLA-DPA1.value > threshold=1.6885469555854797
node_861: feature_name=CD83	CD83.value <= threshold=3.0563642978668213

node_862: feature_name=HLA-DQB1	HLA-DQB1.value > threshold=2.61081063747406
node_1358: feature_name=HLA-DRB5	HLA-DRB5.value > threshold=0.7542744874954224
node_1390: feature_name=HLA-DRB5	HLA-DRB5.value <= threshold=2.6244447231292725
node_1391: feature_name=XIST	XIST.value <= threshold=-0.22017984092235565
node_1392: feature_name=MT-CO3	MT-CO3.value <= threshold=0.12647542357444763
node_1393: feature_name=CD79B	CD79B.value <= threshold=4.338301658630371
node_1394: feature_name=LTB	LTB.value <= threshold=0.9865012466907501
node_1395: feature_name=MEF2C	MEF2C.value <= threshold=4.133931398391724
node_1396: feature_name=MEF2C	MEF2C.value <= threshold=-0.38076959550380707
node_1397: feature_name=UTY	UTY.value <= threshold=-0.4634757786989212
node_1398: feature_name=MT-CO1	MT-CO1.value > threshold=-0.8070613443851471
node_1402: feature_name=ATF3	ATF3.value > threshold=-0.4212478846311569
node_1404: feature_name=MT-CO2	MT-CO2.value > threshold=-0.6675371527671814
node_1406: feature_name=RP11-731F5.2	RP11-731F5.2.value <= threshold=-0.1286414936184883
node_1407: feature_name=SESN3	SESN3.value <= threshold=1.502801775932312
node_1408: feature_name=MS4A1	MS4A1.value <= threshold=3.278305411338806
Class: COVID-19 Critical	
Rules_346	passed counts:13
node_0: feature_name=LTB	LTB.value <= threshold=0.994031548500061
node_1: feature_name=MTRNR2L2	MTRNR2L2.value <= threshold=2.8784297704696655
node_2: feature_name=TCL1A	TCL1A.value <= threshold=4.798312664031982
node_3: feature_name=MT-CO1	MT-CO1.value <= threshold=0.08947191759943962
node_4: feature_name=NR4A1	NR4A1.value <= threshold=1.0725348591804504
node_5: feature_name=HLA-DPA1	HLA-DPA1.value > threshold=1.6885469555854797
node_861: feature_name=CD83	CD83.value <= threshold=3.0563642978668213
node_862: feature_name=HLA-DQB1	HLA-DQB1.value <= threshold=2.61081063747406
node_863: feature_name=MTRNR2L2	MTRNR2L2.value > threshold=2.0919997692108154
node_1355: feature_name=MEF2C	MEF2C.value <= threshold=3.305505633354187
Class: COVID-19 Severe	

Rules_347	passed counts:13
node_0: feature_name=LTB	LTB.value <= threshold=0.994031548500061
node_1: feature_name=MTRNR2L2	MTRNR2L2.value <= threshold=2.8784297704696655
node_2: feature_name=TCL1A	TCL1A.value <= threshold=4.798312664031982
node_3: feature_name=MT-CO1	MT-CO1.value <= threshold=0.08947191759943962
node_4: feature_name=NR4A1	NR4A1.value <= threshold=1.0725348591804504
node_5: feature_name=HLA-DPA1	HLA-DPA1.value > threshold=1.6885469555854797
node_861: feature_name=CD83	CD83.value <= threshold=3.0563642978668213
node_862: feature_name=HLA-DQB1	HLA-DQB1.value <= threshold=2.61081063747406
node_863: feature_name=MTRNR2L2	MTRNR2L2.value <= threshold=2.0919997692108154
node_864: feature_name=BCL2A1	BCL2A1.value <= threshold=1.852618932723999
node_865: feature_name=CD79B	CD79B.value <= threshold=4.352142333984375
node_866: feature_name=TNFAIP3	TNFAIP3.value <= threshold=-0.0449496041983366
node_867: feature_name=MT-CO1	MT-CO1.value > threshold=-0.08531957119703293
node_1155: feature_name=IRF8	IRF8.value <= threshold=-0.39239931106567383
node_1156: feature_name=OAZ1	OAZ1.value > threshold=0.027674595825374126
node_1168: feature_name=TNFAIP3	TNFAIP3.value <= threshold=-0.6463479399681091
node_1169: feature_name=HLA-DQB1	HLA-DQB1.value <= threshold=2.5379010438919067
Class: Healthy Control	
Rules_348	passed counts:13
node_0: feature_name=LTB	LTB.value <= threshold=0.994031548500061
node_1: feature_name=MTRNR2L2	MTRNR2L2.value <= threshold=2.8784297704696655
node_2: feature_name=TCL1A	TCL1A.value <= threshold=4.798312664031982
node_3: feature_name=MT-CO1	MT-CO1.value <= threshold=0.08947191759943962
node_4: feature_name=NR4A1	NR4A1.value <= threshold=1.0725348591804504
node_5: feature_name=HLA-DPA1	HLA-DPA1.value > threshold=1.6885469555854797
node_861: feature_name=CD83	CD83.value <= threshold=3.0563642978668213
node_862: feature_name=HLA-DQB1	HLA-DQB1.value <= threshold=2.61081063747406

node_863: feature_name=MTRNR2L2	MTRNR2L2.value <= threshold=2.0919997692108154
node_864: feature_name=BCL2A1	BCL2A1.value <= threshold=1.852618932723999
node_865: feature_name=CD79B	CD79B.value <= threshold=4.352142333984375
node_866: feature_name=TNFAIP3	TNFAIP3.value <= threshold=-0.0449496041983366
node_867: feature_name=MT-CO1	MT-CO1.value <= threshold=-0.08531957119703293
node_868: feature_name=MTRNR2L8	MTRNR2L8.value <= threshold=0.8770350813865662
node_869: feature_name=EGR1	EGR1.value > threshold=-0.32270750403404236
node_1015: feature_name=NR4A3	NR4A3.value <= threshold=1.9085431098937988
node_1016: feature_name=ID3	ID3.value > threshold=2.776647686958313
node_1128: feature_name=MARCKS	MARCKS.value <= threshold=-0.29620493948459625
node_1129: feature_name=SNX29P2	SNX29P2.value > threshold=-0.2881411612033844
node_1133: feature_name=PLD4	PLD4.value <= threshold=2.106159593909979
Class: COVID-19 Critical	
Rules_349	passed counts:13
node_0: feature_name=LTB	LTB.value <= threshold=0.994031548500061
node_1: feature_name=MTRNR2L2	MTRNR2L2.value <= threshold=2.8784297704696655
node_2: feature_name=TCL1A	TCL1A.value <= threshold=4.798312664031982
node_3: feature_name=MT-CO1	MT-CO1.value <= threshold=0.08947191759943962
node_4: feature_name=NR4A1	NR4A1.value <= threshold=1.0725348591804504
node_5: feature_name=HLA-DPA1	HLA-DPA1.value > threshold=1.6885469555854797
node_861: feature_name=CD83	CD83.value <= threshold=3.0563642978668213
node_862: feature_name=HLA-DQB1	HLA-DQB1.value <= threshold=2.61081063747406
node_863: feature_name=MTRNR2L2	MTRNR2L2.value <= threshold=2.0919997692108154
node_864: feature_name=BCL2A1	BCL2A1.value <= threshold=1.852618932723999
node_865: feature_name=CD79B	CD79B.value <= threshold=4.352142333984375
node_866: feature_name=TNFAIP3	TNFAIP3.value <= threshold=-0.0449496041983366
node_867: feature_name=MT-CO1	MT-CO1.value <= threshold=-0.08531957119703293
node_868: feature_name=MTRNR2L8	MTRNR2L8.value <= threshold=0.8770350813865662
node_869: feature_name=EGR1	EGR1.value <= threshold=-0.32270750403404236

node_870: feature_name=IGJ	IGJ.value <= threshold=4.618318557739258
node_871: feature_name=DUSP4	DUSP4.value <= threshold=2.8197922706604004
node_872: feature_name=HOPX	HOPX.value <= threshold=2.10397732257843
node_873: feature_name=RGCC	RGCC.value <= threshold=1.2070720195770264
node_874: feature_name=OAZ1	OAZ1.value <= threshold=1.02116060256958
node_875: feature_name=NAIP	NAIP.value <= threshold=3.768904685974121
node_876: feature_name=LDLR	LDLR.value <= threshold=2.9582698345184326
node_877: feature_name=HLA-DPA1	HLA-DPA1.value <= threshold=3.0588693618774414
node_878: feature_name=RHOB	RHOB.value <= threshold=5.08755898475647
node_879: feature_name=SYTL3	SYTL3.value <= threshold=1.5127440690994263
node_880: feature_name=FTH1	FTH1.value > threshold=-1.4152275919914246
node_882: feature_name=CCL3	CCL3.value <= threshold=2.253131687641144
node_883: feature_name=CD83	CD83.value <= threshold=2.8566592931747437
node_884: feature_name=HLA-DRB5	HLA-DRB5.value > threshold=-0.6464254856109619
node_888: feature_name=HSP90B1	HSP90B1.value > threshold=-1.186358630657196
node_892: feature_name=ATG2A	ATG2A.value <= threshold=2.3624722957611084
node_893: feature_name=ICAM1	ICAM1.value <= threshold=2.4306468963623047
node_894: feature_name=CD38	CD38.value > threshold=3.4977954626083374
node_954: feature_name=IGJ	IGJ.value > threshold=-0.30224837362766266
Class: COVID-19 Critical	
Rules_350	passed counts:12
node_0: feature_name=LTB	LTB.value > threshold=0.994031548500061
node_5264: feature_name=MT-CO1	MT-CO1.value > threshold=-0.4127991199493408
node_5638: feature_name=OAZ1	OAZ1.value > threshold=-0.2640867084264755
node_6112: feature_name=IFI44L	IFI44L.value <= threshold=2.5893263816833496
node_6113: feature_name=TCL1A	TCL1A.value <= threshold=4.201832294464111
node_6114: feature_name=HLA-DQA2	HLA-DQA2.value > threshold=-0.2270243540406227
node_6542: feature_name=CD79B	CD79B.value <= threshold=3.096232771873474
node_6543: feature_name=NR4A1	NR4A1.value <= threshold=1.421015739440918

node_6544: feature_name=LTB	LTB.value > threshold=1.359755277633667
node_6720: feature_name=NFKBIZ	NFKBIZ.value <= threshold=0.2720414251089096
node_6721: feature_name=ATF5	ATF5.value > threshold=2.4672781229019165
node_6787: feature_name=SPI1	SPI1.value > threshold=-0.4216492027044296
Class: COVID-19 Critical	
Rules_351	passed counts:12
node_0: feature_name=LTB	LTB.value > threshold=0.994031548500061
node_5264: feature_name=MT-CO1	MT-CO1.value > threshold=-0.4127991199493408
node_5638: feature_name=OAZ1	OAZ1.value > threshold=-0.2640867084264755
node_6112: feature_name=IFI44L	IFI44L.value <= threshold=2.5893263816833496
node_6113: feature_name=TCL1A	TCL1A.value <= threshold=4.201832294464111
node_6114: feature_name=HLA-DQA2	HLA-DQA2.value > threshold=-0.2270243540406227
node_6542: feature_name=CD79B	CD79B.value <= threshold=3.096232771873474
node_6543: feature_name=NR4A1	NR4A1.value <= threshold=1.421015739440918
node_6544: feature_name=LTB	LTB.value > threshold=1.359755277633667
node_6720: feature_name=NFKBIZ	NFKBIZ.value <= threshold=0.2720414251089096
node_6721: feature_name=ATF5	ATF5.value <= threshold=2.4672781229019165
node_6722: feature_name=SNX9	SNX9.value > threshold=2.0814030170440674
node_6780: feature_name=JUN	JUN.value <= threshold=0.45471879839897156
node_6781: feature_name=MTRNR2L2	MTRNR2L2.value <= threshold=2.6171658039093018
Class: COVID-19 Critical	
Rules_352	passed counts:12
node_0: feature_name=LTB	LTB.value > threshold=0.994031548500061
node_5264: feature_name=MT-CO1	MT-CO1.value > threshold=-0.4127991199493408
node_5638: feature_name=OAZ1	OAZ1.value > threshold=-0.2640867084264755
node_6112: feature_name=IFI44L	IFI44L.value <= threshold=2.5893263816833496
node_6113: feature_name=TCL1A	TCL1A.value <= threshold=4.201832294464111
node_6114: feature_name=HLA-DQA2	HLA-DQA2.value > threshold=-0.2270243540406227

node_6542: feature_name=CD79B	CD79B.value <= threshold=3.096232771873474
node_6543: feature_name=NR4A1	NR4A1.value <= threshold=1.421015739440918
node_6544: feature_name=LTB	LTB.value > threshold=1.359755277633667
node_6720: feature_name=NFKBIZ	NFKBIZ.value <= threshold=0.2720414251089096
node_6721: feature_name=ATF5	ATF5.value <= threshold=2.4672781229019165
node_6722: feature_name=SNX9	SNX9.value <= threshold=2.0814030170440674
node_6723: feature_name=HLA-DQA2	HLA-DQA2.value <= threshold=4.360614776611328
node_6724: feature_name=MT-CO1	MT-CO1.value > threshold=-0.12188276648521423
node_6736: feature_name=MT1E	MT1E.value <= threshold=-0.1352209895849228
node_6737: feature_name=PELI1	PELI1.value <= threshold=3.1761642694473267
node_6738: feature_name=TRAF1	TRAF1.value <= threshold=-0.2332744374871254
node_6739: feature_name=HLA-DQA2	HLA-DQA2.value > threshold=-0.22670809924602509
node_6741: feature_name=HLA-DRB5	HLA-DRB5.value > threshold=-0.7810345888137817
node_6745: feature_name=GPX1	GPX1.value > threshold=0.5079085677862167
node_6757: feature_name=FAM129C	FAM129C.value > threshold=-0.24598311632871628
Class: Healthy Control	
Rules_353	passed counts:12
node_0: feature_name=LTB	LTB.value > threshold=0.994031548500061
node_5264: feature_name=MT-CO1	MT-CO1.value > threshold=-0.4127991199493408
node_5638: feature_name=OAZ1	OAZ1.value > threshold=-0.2640867084264755
node_6112: feature_name=IFI44L	IFI44L.value <= threshold=2.5893263816833496
node_6113: feature_name=TCL1A	TCL1A.value <= threshold=4.201832294464111
node_6114: feature_name=HLA-DQA2	HLA-DQA2.value > threshold=-0.2270243540406227
node_6542: feature_name=CD79B	CD79B.value <= threshold=3.096232771873474
node_6543: feature_name=NR4A1	NR4A1.value <= threshold=1.421015739440918
node_6544: feature_name=LTB	LTB.value <= threshold=1.359755277633667
node_6545: feature_name=NFKBID	NFKBID.value > threshold=1.4294711351394653
node_6643: feature_name=FAM129C	FAM129C.value > threshold=4.799884080886841
node_6709: feature_name=MT-ND6	MT-ND6.value > threshold=-0.9825485348701477

node_6717: feature_name=PARP14	PARP14.value <= threshold=3.3823366165161133
Class: Healthy Control	
Rules_354	passed counts:12
node_0: feature_name=LTB	LTB.value > threshold=0.994031548500061
node_5264: feature_name=MT-CO1	MT-CO1.value > threshold=-0.4127991199493408
node_5638: feature_name=OAZ1	OAZ1.value > threshold=-0.2640867084264755
node_6112: feature_name=IFI44L	IFI44L.value <= threshold=2.5893263816833496
node_6113: feature_name=TCL1A	TCL1A.value <= threshold=4.201832294464111
node_6114: feature_name=HLA-DQA2	HLA-DQA2.value <= threshold=-0.2270243540406227
node_6115: feature_name=MTRNR2L2	MTRNR2L2.value <= threshold=2.548917293548584
node_6116: feature_name=ATF5	ATF5.value <= threshold=2.041263461112976
node_6117: feature_name=JUN	JUN.value > threshold=-0.616259902715683
node_6189: feature_name=MT-CO3	MT-CO3.value <= threshold=0.13483521342277527
node_6190: feature_name=XAF1	XAF1.value <= threshold=2.584952235221863
node_6191: feature_name=HLA-DRB5	HLA-DRB5.value > threshold=0.6955901980400085
node_6239: feature_name=TNFAIP3	TNFAIP3.value <= threshold=0.11930759996175766
node_6240: feature_name=TP53INP1	TP53INP1.value <= threshold=2.9654784202575684
node_6241: feature_name=HLA-DRB5	HLA-DRB5.value <= threshold=2.5141202211380005
node_6242: feature_name=NFKBID	NFKBID.value <= threshold=3.358878970146179
node_6243: feature_name=JARID2	JARID2.value <= threshold=1.7160060405731201
node_6244: feature_name=HLA-DPA1	HLA-DPA1.value > threshold=0.49491266906261444
node_6246: feature_name=MIR24-2	MIR24-2.value <= threshold=1.8348549008369446
node_6247: feature_name=HSP90B1	HSP90B1.value <= threshold=1.8861054182052612
node_6248: feature_name=NR4A1	NR4A1.value <= threshold=-0.5434737503528595
node_6249: feature_name=MT-CO1	MT-CO1.value <= threshold=0.002460678806528449
node_6250: feature_name=FTH1	FTH1.value <= threshold=-0.5194586217403412
node_6251: feature_name=STMN1	STMN1.value <= threshold=2.071332335472107
Class: Healthy Control	

Rules_355	passed counts:12
node_0: feature_name=LTB	LTB.value > threshold=0.994031548500061
node_5264: feature_name=MT-CO1	MT-CO1.value > threshold=-0.4127991199493408
node_5638: feature_name=OAZ1	OAZ1.value > threshold=-0.2640867084264755
node_6112: feature_name=IFI44L	IFI44L.value <= threshold=2.5893263816833496
node_6113: feature_name=TCL1A	TCL1A.value <= threshold=4.201832294464111
node_6114: feature_name=HLA-DQA2	HLA-DQA2.value <= threshold=-0.2270243540406227
node_6115: feature_name=MTRNR2L2	MTRNR2L2.value <= threshold=2.548917293548584
node_6116: feature_name=ATF5	ATF5.value <= threshold=2.041263461112976
node_6117: feature_name=JUN	JUN.value > threshold=-0.616259902715683
node_6189: feature_name=MT-CO3	MT-CO3.value <= threshold=0.13483521342277527
node_6190: feature_name=XAF1	XAF1.value <= threshold=2.584952235221863
node_6191: feature_name=HLA-DRB5	HLA-DRB5.value <= threshold=0.6955901980400085
node_6192: feature_name=NR4A1	NR4A1.value <= threshold=-0.4496448189020157
node_6193: feature_name=HLA-DPB1	HLA-DPB1.value <= threshold=1.7609722018241882
node_6194: feature_name=JUN	JUN.value <= threshold=1.172904908657074
node_6195: feature_name=MT-ND6	MT-ND6.value > threshold=0.6287261247634888
node_6207: feature_name=RGCC	RGCC.value <= threshold=-0.8717423379421234
node_6208: feature_name=UTY	UTY.value <= threshold=1.2420618534088135
Class: Healthy Control	
Rules_356	passed counts:12
node_0: feature_name=LTB	LTB.value > threshold=0.994031548500061
node_5264: feature_name=MT-CO1	MT-CO1.value > threshold=-0.4127991199493408
node_5638: feature_name=OAZ1	OAZ1.value > threshold=-0.2640867084264755
node_6112: feature_name=IFI44L	IFI44L.value <= threshold=2.5893263816833496
node_6113: feature_name=TCL1A	TCL1A.value <= threshold=4.201832294464111
node_6114: feature_name=HLA-DQA2	HLA-DQA2.value <= threshold=-0.2270243540406227
node_6115: feature_name=MTRNR2L2	MTRNR2L2.value <= threshold=2.548917293548584
node_6116: feature_name=ATF5	ATF5.value <= threshold=2.041263461112976

node_6117: feature_name=JUN	JUN.value <= threshold=-0.616259902715683
node_6118: feature_name=NFKBID	NFKBID.value <= threshold=1.5264258980751038
node_6119: feature_name=MT-CO1	MT-CO1.value > threshold=0.1564532369375229
node_6133: feature_name=LTB	LTB.value <= threshold=1.2431999444961548
node_6134: feature_name=MT-ND6	MT-ND6.value <= threshold=0.5990295112133026
node_6135: feature_name=MEF2A	MEF2A.value <= threshold=1.0292169749736786
node_6136: feature_name=MX1	MX1.value <= threshold=-0.4555272161960602
node_6137: feature_name=BACH1	BACH1.value <= threshold=1.2474263310432434
node_6138: feature_name=AP3M2	AP3M2.value <= threshold=0.7636791169643402
Class: Healthy Control	
Rules_357	passed counts:12
node_0: feature_name=LTB	LTB.value > threshold=0.994031548500061
node_5264: feature_name=MT-CO1	MT-CO1.value > threshold=-0.4127991199493408
node_5638: feature_name=OAZ1	OAZ1.value <= threshold=-0.2640867084264755
node_5639: feature_name=CD79B	CD79B.value > threshold=2.2998517751693726
node_5925: feature_name=SNX9	SNX9.value <= threshold=2.4445992708206177
node_5926: feature_name=TCL1A	TCL1A.value <= threshold=2.6938670873641968
node_5927: feature_name=PLD4	PLD4.value > threshold=-0.06434785947203636
node_6009: feature_name=MX1	MX1.value <= threshold=3.600893020629883
node_6010: feature_name=PRDM1	PRDM1.value > threshold=-0.407244011759758
node_6046: feature_name=LTB	LTB.value > threshold=1.087718427181244
node_6048: feature_name=CD79B	CD79B.value > threshold=2.662985920906067
Class: COVID-19 Critical	
Rules_358	passed counts:12
node_0: feature_name=LTB	LTB.value > threshold=0.994031548500061
node_5264: feature_name=MT-CO1	MT-CO1.value > threshold=-0.4127991199493408
node_5638: feature_name=OAZ1	OAZ1.value <= threshold=-0.2640867084264755
node_5639: feature_name=CD79B	CD79B.value <= threshold=2.2998517751693726

node_5640: feature_name=MTRNR2L2	MTRNR2L2.value <= threshold=3.5499863624572754
node_5641: feature_name=MT-CO1	MT-CO1.value <= threshold=0.22567133605480194
node_5642: feature_name=RGS2	RGS2.value > threshold=1.2649686336517334
node_5710: feature_name=HLA-DPB1	HLA-DPB1.value > threshold=1.09694242477417
node_5712: feature_name=PPIF	PPIF.value > threshold=-0.4469884932041168
node_5720: feature_name=CD83	CD83.value > threshold=1.302907645702362
node_5722: feature_name=COTL1	COTL1.value <= threshold=0.1188640259206295
node_5723: feature_name=ICAM1	ICAM1.value <= threshold=-0.24770931899547577
Class: COVID-19 Critical	
Rules_359	passed counts:12
node_0: feature_name=LTB	LTB.value > threshold=0.994031548500061
node_5264: feature_name=MT-CO1	MT-CO1.value > threshold=-0.4127991199493408
node_5638: feature_name=OAZ1	OAZ1.value <= threshold=-0.2640867084264755
node_5639: feature_name=CD79B	CD79B.value <= threshold=2.2998517751693726
node_5640: feature_name=MTRNR2L2	MTRNR2L2.value <= threshold=3.5499863624572754
node_5641: feature_name=MT-CO1	MT-CO1.value <= threshold=0.22567133605480194
node_5642: feature_name=RGS2	RGS2.value > threshold=1.2649686336517334
node_5710: feature_name=HLA-DPB1	HLA-DPB1.value > threshold=1.09694242477417
node_5712: feature_name=PPIF	PPIF.value <= threshold=-0.4469884932041168
node_5713: feature_name=TCF4	TCF4.value > threshold=-0.2837526798248291
node_5717: feature_name=FCRL1	FCRL1.value > threshold=-0.20456314086914062
Class: Healthy Control	
Rules_360	passed counts:12
node_0: feature_name=LTB	LTB.value > threshold=0.994031548500061
node_5264: feature_name=MT-CO1	MT-CO1.value <= threshold=-0.4127991199493408
node_5265: feature_name=MTRNR2L2	MTRNR2L2.value <= threshold=2.4473838806152344
node_5266: feature_name=TCL1A	TCL1A.value <= threshold=5.787256956100464
node_5267: feature_name=PLD4	PLD4.value > threshold=3.544657826423645

node_5577: feature_name=MT-CO1	MT-CO1.value <= threshold=-0.6592064797878265
node_5578: feature_name=HOPX	HOPX.value > threshold=-0.47573523223400116
node_5584: feature_name=OAZ1	OAZ1.value > threshold=0.5679030418395996
node_5598: feature_name=MS4A1	MS4A1.value > threshold=2.3023334741592407
Class: Healthy Control	
Rules_361	passed counts:12
node_0: feature_name=LTB	LTB.value <= threshold=0.994031548500061
node_1: feature_name=MTRNR2L2	MTRNR2L2.value > threshold=2.8784297704696655
node_5207: feature_name=MTRNR2L8	MTRNR2L8.value <= threshold=3.1915172338485718
node_5208: feature_name=MT-CYB	MT-CYB.value > threshold=1.3929389119148254
node_5242: feature_name=MTRNR2L2	MTRNR2L2.value <= threshold=3.8612314462661743
node_5243: feature_name=RALGPS2	RALGPS2.value <= threshold=4.833884000778198
node_5244: feature_name=HLA-DQB1	HLA-DQB1.value <= threshold=2.408530831336975
node_5245: feature_name=NR4A1	NR4A1.value > threshold=-0.45447592437267303
Class: COVID-19 Critical	
Rules_362	passed counts:12
node_0: feature_name=LTB	LTB.value <= threshold=0.994031548500061
node_1: feature_name=MTRNR2L2	MTRNR2L2.value <= threshold=2.8784297704696655
node_2: feature_name=TCL1A	TCL1A.value > threshold=4.798312664031982
node_4912: feature_name=MT-CO1	MT-CO1.value > threshold=-0.3664844036102295
node_5002: feature_name=IFI44L	IFI44L.value <= threshold=2.3101747035980225
node_5003: feature_name=SNX9	SNX9.value <= threshold=2.0247421264648438
node_5004: feature_name=STAG3	STAG3.value <= threshold=6.482141733169556
node_5005: feature_name=BACH1	BACH1.value > threshold=-0.5237349569797516
node_5047: feature_name=RHOB	RHOB.value > threshold=-0.4843696355819702
node_5055: feature_name=FAM129C	FAM129C.value <= threshold=-0.2832885980606079
node_5056: feature_name=CD83	CD83.value > threshold=-0.5592367649078369
node_5060: feature_name=OAZ1	OAZ1.value > threshold=0.7524859011173248

node_5074: feature_name=MS4A1	MS4A1.value > threshold=1.7299362421035767
node_5076: feature_name=CD79B	CD79B.value > threshold=-0.32401059567928314
Class: Healthy Control	
Rules_363	passed counts:12
node_0: feature_name=LTB	LTB.value <= threshold=0.994031548500061
node_1: feature_name=MTRNR2L2	MTRNR2L2.value <= threshold=2.8784297704696655
node_2: feature_name=TCL1A	TCL1A.value > threshold=4.798312664031982
node_4912: feature_name=MT-CO1	MT-CO1.value > threshold=-0.3664844036102295
node_5002: feature_name=IFI44L	IFI44L.value <= threshold=2.3101747035980225
node_5003: feature_name=SNX9	SNX9.value <= threshold=2.0247421264648438
node_5004: feature_name=STAG3	STAG3.value <= threshold=6.482141733169556
node_5005: feature_name=BACH1	BACH1.value <= threshold=-0.5237349569797516
node_5006: feature_name=OAZ1	OAZ1.value > threshold=-0.6512158215045929
node_5014: feature_name=MEF2A	MEF2A.value <= threshold=3.61627995967865
node_5015: feature_name=HLA-DPB1	HLA-DPB1.value > threshold=-0.2871522083878517
node_5019: feature_name=FTH1	FTH1.value <= threshold=1.0194584727287292
node_5020: feature_name=RNF144B	RNF144B.value <= threshold=3.085212469100952
node_5021: feature_name=TTN	TTN.value <= threshold=5.307270765304565
node_5022: feature_name=CCL3	CCL3.value <= threshold=1.4737252593040466
node_5023: feature_name=LTB	LTB.value > threshold=-1.121648371219635
node_5025: feature_name=HLA-DQB1	HLA-DQB1.value <= threshold=3.169318914413452
node_5026: feature_name=ATF3	ATF3.value <= threshold=2.082080602645874
node_5027: feature_name=SNX9	SNX9.value > threshold=1.8630352020263672
node_5033: feature_name=HLA-DQA2	HLA-DQA2.value <= threshold=1.6821948364377022
node_5034: feature_name=TCL1A	TCL1A.value > threshold=5.027214288711548
Class: Healthy Control	
Rules_364	passed counts:12
node_0: feature_name=LTB	LTB.value <= threshold=0.994031548500061

node_1: feature_name=MTRNR2L2	MTRNR2L2.value <= threshold=2.8784297704696655
node_2: feature_name=TCL1A	TCL1A.value > threshold=4.798312664031982
node_4912: feature_name=MT-CO1	MT-CO1.value > threshold=-0.3664844036102295
node_5002: feature_name=IFI44L	IFI44L.value <= threshold=2.3101747035980225
node_5003: feature_name=SNX9	SNX9.value <= threshold=2.0247421264648438
node_5004: feature_name=STAG3	STAG3.value <= threshold=6.482141733169556
node_5005: feature_name=BACH1	BACH1.value <= threshold=-0.5237349569797516
node_5006: feature_name=OAZ1	OAZ1.value <= threshold=-0.6512158215045929
node_5007: feature_name=HLA-DQA1	HLA-DQA1.value > threshold=1.5983093976974487
node_5011: feature_name=YWHAH	YWHAH.value > threshold=-0.3913958817720413
Class: Healthy Control	
Rules_365	passed counts:12
node_0: feature_name=LTB	LTB.value <= threshold=0.994031548500061
node_1: feature_name=MTRNR2L2	MTRNR2L2.value <= threshold=2.8784297704696655
node_2: feature_name=TCL1A	TCL1A.value <= threshold=4.798312664031982
node_3: feature_name=MT-CO1	MT-CO1.value > threshold=0.08947191759943962
node_2521: feature_name=LTB	LTB.value > threshold=0.6038667261600494
node_4173: feature_name=OAZ1	OAZ1.value > threshold=0.143283873796463
node_4615: feature_name=NR4A1	NR4A1.value <= threshold=0.6621004343032837
node_4616: feature_name=NFKBID	NFKBID.value <= threshold=3.283741593360901
node_4617: feature_name=HLA-DQA1	HLA-DQA1.value <= threshold=2.3435752391815186
node_4618: feature_name=PELI1	PELI1.value <= threshold=1.3271203637123108
node_4619: feature_name=PLD4	PLD4.value <= threshold=3.3385335206985474
node_4620: feature_name=MEF2A	MEF2A.value <= threshold=1.5926940441131592
node_4621: feature_name=DAPP1	DAPP1.value > threshold=2.647637367248535
node_4655: feature_name=FAM160B1	FAM160B1.value <= threshold=1.5589128136634827
Class: COVID-19 Critical	
Rules_366	passed counts:12

node_0: feature_name=LTB	LTB.value <= threshold=0.994031548500061
node_1: feature_name=MTRNR2L2	MTRNR2L2.value <= threshold=2.8784297704696655
node_2: feature_name=TCL1A	TCL1A.value <= threshold=4.798312664031982
node_3: feature_name=MT-CO1	MT-CO1.value > threshold=0.08947191759943962
node_2521: feature_name=LTB	LTB.value > threshold=0.6038667261600494
node_4173: feature_name=OAZ1	OAZ1.value <= threshold=0.143283873796463
node_4174: feature_name=PLD4	PLD4.value <= threshold=2.921932101249695
node_4175: feature_name=XIST	XIST.value <= threshold=-0.05067425221204758
node_4176: feature_name=MT-CO1	MT-CO1.value <= threshold=1.345594048500061
node_4177: feature_name=HLA-DQB1	HLA-DQB1.value <= threshold=1.927736520767212
node_4178: feature_name=BACH1	BACH1.value > threshold=-0.5349778532981873
node_4236: feature_name=TPM1	TPM1.value <= threshold=3.516477584838867
node_4237: feature_name=NR4A1	NR4A1.value > threshold=-0.274935707449913
node_4277: feature_name=HLA-DPA1	HLA-DPA1.value > threshold=1.8894080519676208
node_4339: feature_name=PARP14	PARP14.value <= threshold=0.2787386327981949
node_4340: feature_name=RGS2	RGS2.value <= threshold=0.10973784327507019
Class: COVID-19 Critical	
Rules_367	passed counts:12
node_0: feature_name=LTB	LTB.value <= threshold=0.994031548500061
node_1: feature_name=MTRNR2L2	MTRNR2L2.value <= threshold=2.8784297704696655
node_2: feature_name=TCL1A	TCL1A.value <= threshold=4.798312664031982
node_3: feature_name=MT-CO1	MT-CO1.value > threshold=0.08947191759943962
node_2521: feature_name=LTB	LTB.value > threshold=0.6038667261600494
node_4173: feature_name=OAZ1	OAZ1.value <= threshold=0.143283873796463
node_4174: feature_name=PLD4	PLD4.value <= threshold=2.921932101249695
node_4175: feature_name=XIST	XIST.value <= threshold=-0.05067425221204758
node_4176: feature_name=MT-CO1	MT-CO1.value <= threshold=1.345594048500061
node_4177: feature_name=HLA-DQB1	HLA-DQB1.value <= threshold=1.927736520767212
node_4178: feature_name=BACH1	BACH1.value > threshold=-0.5349778532981873

node_4236: feature_name=TPM1	TPM1.value <= threshold=3.516477584838867
node_4237: feature_name=NR4A1	NR4A1.value <= threshold=-0.274935707449913
node_4238: feature_name=VPREB3	VPREB3.value > threshold=5.024149179458618
node_4270: feature_name=IRF8	IRF8.value > threshold=0.23405013978481293
node_4274: feature_name=SNORD3B-2	SNORD3B-2.value <= threshold=1.8730536252260208
Class: COVID-19 Critical	
Rules_368	passed counts:12
node_0: feature_name=LTB	LTB.value <= threshold=0.994031548500061
node_1: feature_name=MTRNR2L2	MTRNR2L2.value <= threshold=2.8784297704696655
node_2: feature_name=TCL1A	TCL1A.value <= threshold=4.798312664031982
node_3: feature_name=MT-CO1	MT-CO1.value > threshold=0.08947191759943962
node_2521: feature_name=LTB	LTB.value <= threshold=0.6038667261600494
node_2522: feature_name=HLA-DQB1	HLA-DQB1.value > threshold=2.1053054332733154
node_3550: feature_name=XIST	XIST.value <= threshold=0.7716337237507105
node_3551: feature_name=CD79B	CD79B.value > threshold=2.9426796436309814
node_4039: feature_name=IFI44L	IFI44L.value <= threshold=1.8637521862983704
node_4040: feature_name=SNX9	SNX9.value <= threshold=1.274232804775238
node_4041: feature_name=CASP8	CASP8.value > threshold=-0.574097603559494
node_4073: feature_name=HLA-DRB5	HLA-DRB5.value > threshold=1.4346495270729065
node_4083: feature_name=NT5C3A	NT5C3A.value > threshold=-0.45546136796474457
Class: Healthy Control	
Rules_369	passed counts:12
node_0: feature_name=LTB	LTB.value <= threshold=0.994031548500061
node_1: feature_name=MTRNR2L2	MTRNR2L2.value <= threshold=2.8784297704696655
node_2: feature_name=TCL1A	TCL1A.value <= threshold=4.798312664031982
node_3: feature_name=MT-CO1	MT-CO1.value > threshold=0.08947191759943962
node_2521: feature_name=LTB	LTB.value <= threshold=0.6038667261600494
node_2522: feature_name=HLA-DQB1	HLA-DQB1.value > threshold=2.1053054332733154

node_3550: feature_name=XIST	XIST.value <= threshold=0.7716337237507105
node_3551: feature_name=CD79B	CD79B.value > threshold=2.9426796436309814
node_4039: feature_name=IFI44L	IFI44L.value <= threshold=1.8637521862983704
node_4040: feature_name=SNX9	SNX9.value <= threshold=1.274232804775238
node_4041: feature_name=CASP8	CASP8.value > threshold=-0.574097603559494
node_4073: feature_name=HLA-DRB5	HLA-DRB5.value > threshold=1.4346495270729065
node_4083: feature_name=NT5C3A	NT5C3A.value <= threshold=-0.45546136796474457
node_4084: feature_name=OAZ1	OAZ1.value <= threshold=0.1348155364394188
node_4085: feature_name=NAMPT	NAMPT.value > threshold=-0.6454389691352844
Class: COVID-19 Critical	
Rules_370	passed counts:12
node_0: feature_name=LTB	LTB.value <= threshold=0.994031548500061
node_1: feature_name=MTRNR2L2	MTRNR2L2.value <= threshold=2.8784297704696655
node_2: feature_name=TCL1A	TCL1A.value <= threshold=4.798312664031982
node_3: feature_name=MT-CO1	MT-CO1.value > threshold=0.08947191759943962
node_2521: feature_name=LTB	LTB.value <= threshold=0.6038667261600494
node_2522: feature_name=HLA-DQB1	HLA-DQB1.value > threshold=2.1053054332733154
node_3550: feature_name=XIST	XIST.value <= threshold=0.7716337237507105
node_3551: feature_name=CD79B	CD79B.value > threshold=2.9426796436309814
node_4039: feature_name=IFI44L	IFI44L.value <= threshold=1.8637521862983704
node_4040: feature_name=SNX9	SNX9.value <= threshold=1.274232804775238
node_4041: feature_name=CASP8	CASP8.value <= threshold=-0.574097603559494
node_4042: feature_name=NFKBID	NFKBID.value > threshold=1.49756920337677
node_4056: feature_name=MIR24-2	MIR24-2.value <= threshold=0.2797542065382004
node_4057: feature_name=MEF2C	MEF2C.value > threshold=2.6051090955734253
Class: Healthy Control	
Rules_371	passed counts:12
node_0: feature_name=LTB	LTB.value <= threshold=0.994031548500061

node_1: feature_name=MTRNR2L2	MTRNR2L2.value <= threshold=2.8784297704696655
node_2: feature_name=TCL1A	TCL1A.value <= threshold=4.798312664031982
node_3: feature_name=MT-CO1	MT-CO1.value > threshold=0.08947191759943962
node_2521: feature_name=LTB	LTB.value <= threshold=0.6038667261600494
node_2522: feature_name=HLA-DQB1	HLA-DQB1.value > threshold=2.1053054332733154
node_3550: feature_name=XIST	XIST.value <= threshold=0.7716337237507105
node_3551: feature_name=CD79B	CD79B.value <= threshold=2.9426796436309814
node_3552: feature_name=HLA-DQB1	HLA-DQB1.value > threshold=2.713674306869507
node_3854: feature_name=MEF2C	MEF2C.value > threshold=-0.3721291869878769
node_3882: feature_name=IFI44L	IFI44L.value <= threshold=2.5516022443771362
node_3883: feature_name=FCER2	FCER2.value <= threshold=-0.21487552672624588
node_3884: feature_name=HLA-DQA2	HLA-DQA2.value <= threshold=-0.12580305337905884
node_3885: feature_name=SLC7A5	SLC7A5.value <= threshold=-0.4906871169805527
node_3886: feature_name=VPREB3	VPREB3.value <= threshold=5.492124557495117
node_3887: feature_name=CYBB	CYBB.value > threshold=2.720046043395996
node_3935: feature_name=MT-CO1	MT-CO1.value > threshold=1.21793794631958
node_3939: feature_name=MEF2A	MEF2A.value <= threshold=4.265384912490845
Class: Healthy Control	
Rules_372	passed counts:12
node_0: feature_name=LTB	LTB.value <= threshold=0.994031548500061
node_1: feature_name=MTRNR2L2	MTRNR2L2.value <= threshold=2.8784297704696655
node_2: feature_name=TCL1A	TCL1A.value <= threshold=4.798312664031982
node_3: feature_name=MT-CO1	MT-CO1.value > threshold=0.08947191759943962
node_2521: feature_name=LTB	LTB.value <= threshold=0.6038667261600494
node_2522: feature_name=HLA-DQB1	HLA-DQB1.value > threshold=2.1053054332733154
node_3550: feature_name=XIST	XIST.value <= threshold=0.7716337237507105
node_3551: feature_name=CD79B	CD79B.value <= threshold=2.9426796436309814
node_3552: feature_name=HLA-DQB1	HLA-DQB1.value <= threshold=2.713674306869507
node_3553: feature_name=SNX9	SNX9.value > threshold=-0.5404067635536194

node_3653: feature_name=LTB	LTB.value > threshold=0.021708179265260696
node_3759: feature_name=TCL1A	TCL1A.value <= threshold=3.3921085596084595
node_3760: feature_name=HLA-DRB5	HLA-DRB5.value > threshold=0.5265218019485474
node_3776: feature_name=ID1	ID1.value > threshold=-0.21235637366771698
node_3828: feature_name=RALGPS2	RALGPS2.value > threshold=2.589961290359497
node_3836: feature_name=BACH1	BACH1.value <= threshold=2.4661574363708496
Class: Healthy Control	
Rules_373	passed counts:12
node_0: feature_name=LTB	LTB.value <= threshold=0.994031548500061
node_1: feature_name=MTRNR2L2	MTRNR2L2.value <= threshold=2.8784297704696655
node_2: feature_name=TCL1A	TCL1A.value <= threshold=4.798312664031982
node_3: feature_name=MT-CO1	MT-CO1.value > threshold=0.08947191759943962
node_2521: feature_name=LTB	LTB.value <= threshold=0.6038667261600494
node_2522: feature_name=HLA-DQB1	HLA-DQB1.value <= threshold=2.1053054332733154
node_2523: feature_name=SNX9	SNX9.value > threshold=-0.5346486568450928
node_2907: feature_name=NR4A1	NR4A1.value > threshold=2.5433837175369263
node_3459: feature_name=VPREB3	VPREB3.value <= threshold=4.410779237747192
node_3460: feature_name=RALGPS2	RALGPS2.value <= threshold=3.064661979675293
node_3461: feature_name=MT-CO1	MT-CO1.value <= threshold=1.7489010095596313
node_3462: feature_name=HLA-DQA2	HLA-DQA2.value > threshold=1.794841468334198
node_3502: feature_name=MARCKS	MARCKS.value > threshold=-0.14459624886512756
node_3510: feature_name=SPI1	SPI1.value <= threshold=0.00490250438451767
node_3511: feature_name=UTY	UTY.value <= threshold=1.4687455296516418
Class: COVID-19 Severe	
Rules_374	passed counts:12
node_0: feature_name=LTB	LTB.value <= threshold=0.994031548500061
node_1: feature_name=MTRNR2L2	MTRNR2L2.value <= threshold=2.8784297704696655
node_2: feature_name=TCL1A	TCL1A.value <= threshold=4.798312664031982

node_3: feature_name=MT-CO1	MT-CO1.value > threshold=0.08947191759943962
node_2521: feature_name=LTB	LTB.value <= threshold=0.6038667261600494
node_2522: feature_name=HLA-DQB1	HLA-DQB1.value <= threshold=2.1053054332733154
node_2523: feature_name=SNX9	SNX9.value > threshold=-0.5346486568450928
node_2907: feature_name=NR4A1	NR4A1.value <= threshold=2.5433837175369263
node_2908: feature_name=XIST	XIST.value <= threshold=0.7664470989257097
node_2909: feature_name=CD79B	CD79B.value > threshold=2.9326847791671753
node_3353: feature_name=VPREB3	VPREB3.value > threshold=3.0226117372512817
node_3425: feature_name=OAZ1	OAZ1.value > threshold=0.47689591348171234
Class: Healthy Control	
Rules_375	passed counts:12
node_0: feature_name=LTB	LTB.value <= threshold=0.994031548500061
node_1: feature_name=MTRNR2L2	MTRNR2L2.value <= threshold=2.8784297704696655
node_2: feature_name=TCL1A	TCL1A.value <= threshold=4.798312664031982
node_3: feature_name=MT-CO1	MT-CO1.value > threshold=0.08947191759943962
node_2521: feature_name=LTB	LTB.value <= threshold=0.6038667261600494
node_2522: feature_name=HLA-DQB1	HLA-DQB1.value <= threshold=2.1053054332733154
node_2523: feature_name=SNX9	SNX9.value > threshold=-0.5346486568450928
node_2907: feature_name=NR4A1	NR4A1.value <= threshold=2.5433837175369263
node_2908: feature_name=XIST	XIST.value <= threshold=0.7664470989257097
node_2909: feature_name=CD79B	CD79B.value <= threshold=2.9326847791671753
node_2910: feature_name=NR4A1	NR4A1.value > threshold=-0.2875557690858841
node_3096: feature_name=NR4A3	NR4A3.value <= threshold=3.314968705177307
node_3097: feature_name=VPREB3	VPREB3.value <= threshold=3.3549938201904297
node_3098: feature_name=RALGPS2	RALGPS2.value <= threshold=5.807050704956055
node_3099: feature_name=CTD-2035E11.3	CTD-2035E11.3.value <= threshold=6.4090416431427
node_3100: feature_name=FAM129C	FAM129C.value <= threshold=6.966361045837402
node_3101: feature_name=HES4	HES4.value <= threshold=4.139557242393494
node_3102: feature_name=CD83	CD83.value > threshold=1.8343153595924377

node_3136: feature_name=PLD4	PLD4.value <= threshold=4.453821897506714
node_3137: feature_name=HLA-DQA2	HLA-DQA2.value > threshold=-0.10758286714553833
node_3229: feature_name=FAM160B1	FAM160B1.value <= threshold=1.61697119474411
node_3230: feature_name=BCL2A1	BCL2A1.value > threshold=0.9140156805515289
node_3250: feature_name=RNF11	RNF11.value > threshold=-0.5307398736476898
node_3258: feature_name=HLA-DPB1	HLA-DPB1.value <= threshold=2.44118869304657
Class: COVID-19 Critical	
Rules_376	passed counts:12
node_0: feature_name=LTB	LTB.value <= threshold=0.994031548500061
node_1: feature_name=MTRNR2L2	MTRNR2L2.value <= threshold=2.8784297704696655
node_2: feature_name=TCL1A	TCL1A.value <= threshold=4.798312664031982
node_3: feature_name=MT-CO1	MT-CO1.value > threshold=0.08947191759943962
node_2521: feature_name=LTB	LTB.value <= threshold=0.6038667261600494
node_2522: feature_name=HLA-DQB1	HLA-DQB1.value <= threshold=2.1053054332733154
node_2523: feature_name=SNX9	SNX9.value <= threshold=-0.5346486568450928
node_2524: feature_name=HLA-DPB1	HLA-DPB1.value > threshold=1.9548270106315613
node_2814: feature_name=CD79B	CD79B.value > threshold=-0.28186772763729095
node_2826: feature_name=MT-ND6	MT-ND6.value <= threshold=0.17273088544607162
node_2827: feature_name=NR4A1	NR4A1.value > threshold=-0.24634335190057755
node_2849: feature_name=FAM129C	FAM129C.value > threshold=3.089340925216675
node_2867: feature_name=ADAM19	ADAM19.value <= threshold=2.1224595308303833
node_2868: feature_name=SYNE1	SYNE1.value <= threshold=1.6539024710655212
Class: COVID-19 Critical	
Rules_377	passed counts:12
node_0: feature_name=LTB	LTB.value <= threshold=0.994031548500061
node_1: feature_name=MTRNR2L2	MTRNR2L2.value <= threshold=2.8784297704696655
node_2: feature_name=TCL1A	TCL1A.value <= threshold=4.798312664031982
node_3: feature_name=MT-CO1	MT-CO1.value <= threshold=0.08947191759943962

node_4: feature_name=NR4A1	NR4A1.value > threshold=1.0725348591804504
node_1622: feature_name=HLA-DQA2	HLA-DQA2.value <= threshold=1.9496816992759705
node_1623: feature_name=HLA-DRB5	HLA-DRB5.value <= threshold=2.715766429901123
node_1624: feature_name=HLA-DPA1	HLA-DPA1.value > threshold=1.7050301432609558
node_2056: feature_name=OAZ1	OAZ1.value <= threshold=0.456103190779686
node_2057: feature_name=HLA-DRB5	HLA-DRB5.value > threshold=0.6839984953403473
node_2101: feature_name=HLA-DRB1	HLA-DRB1.value <= threshold=1.6199676990509033
node_2102: feature_name=CD83	CD83.value > threshold=2.5568599700927734
node_2124: feature_name=MARCH1	MARCH1.value > threshold=-0.34506022930145264
node_2140: feature_name=GRASP	GRASP.value <= threshold=2.12925124168396
node_2141: feature_name=KIAA0125	KIAA0125.value > threshold=-0.18390044569969177
node_2145: feature_name=FERMT3	FERMT3.value <= threshold=0.48793651163578033
Class: COVID-19 Critical	
Rules_378	passed counts:12
node_0: feature_name=LTB	LTB.value <= threshold=0.994031548500061
node_1: feature_name=MTRNR2L2	MTRNR2L2.value <= threshold=2.8784297704696655
node_2: feature_name=TCL1A	TCL1A.value <= threshold=4.798312664031982
node_3: feature_name=MT-CO1	MT-CO1.value <= threshold=0.08947191759943962
node_4: feature_name=NR4A1	NR4A1.value > threshold=1.0725348591804504
node_1622: feature_name=HLA-DQA2	HLA-DQA2.value <= threshold=1.9496816992759705
node_1623: feature_name=HLA-DRB5	HLA-DRB5.value <= threshold=2.715766429901123
node_1624: feature_name=HLA-DPA1	HLA-DPA1.value > threshold=1.7050301432609558
node_2056: feature_name=OAZ1	OAZ1.value <= threshold=0.456103190779686
node_2057: feature_name=HLA-DRB5	HLA-DRB5.value <= threshold=0.6839984953403473
node_2058: feature_name=IFI6	IFI6.value <= threshold=-0.5314981639385223
node_2059: feature_name=CD83	CD83.value <= threshold=2.6418607234954834
node_2060: feature_name=HLA-DRB1	HLA-DRB1.value > threshold=1.92746239900589
node_2070: feature_name=FTH1	FTH1.value <= threshold=0.9439846873283386
node_2071: feature_name=HLA-DRB5	HLA-DRB5.value > threshold=-0.6903533041477203

Class: COVID-19 Severe	
Rules_379	passed counts:12
node_0: feature_name=LTB	LTB.value <= threshold=0.994031548500061
node_1: feature_name=MTRNR2L2	MTRNR2L2.value <= threshold=2.8784297704696655
node_2: feature_name=TCL1A	TCL1A.value <= threshold=4.798312664031982
node_3: feature_name=MT-CO1	MT-CO1.value <= threshold=0.08947191759943962
node_4: feature_name=NR4A1	NR4A1.value > threshold=1.0725348591804504
node_1622: feature_name=HLA-DQA2	HLA-DQA2.value <= threshold=1.9496816992759705
node_1623: feature_name=HLA-DRB5	HLA-DRB5.value <= threshold=2.715766429901123
node_1624: feature_name=HLA-DPA1	HLA-DPA1.value <= threshold=1.7050301432609558
node_1625: feature_name=CD83	CD83.value > threshold=2.783489227294922
node_1873: feature_name=IFI44L	IFI44L.value <= threshold=1.5938722491264343
node_1874: feature_name=PLD4	PLD4.value <= threshold=4.101001739501953
node_1875: feature_name=RHOB	RHOB.value <= threshold=1.304835319519043
node_1876: feature_name=PPIF	PPIF.value <= threshold=2.2943923473358154
node_1877: feature_name=OAZ1	OAZ1.value <= threshold=0.04260866530239582
node_1878: feature_name=ID3	ID3.value <= threshold=5.290210247039795
node_1879: feature_name=NFKBID	NFKBID.value > threshold=-0.40532417595386505
node_1883: feature_name=MEF2C	MEF2C.value > threshold=-0.4597593992948532
node_1887: feature_name=SNX9	SNX9.value > threshold=1.010923683643341
node_1901: feature_name=RBKS	RBKS.value <= threshold=4.642704963684082
node_1902: feature_name=TSPYL2	TSPYL2.value <= threshold=2.4923402070999146
node_1903: feature_name=MARCKS	MARCKS.value <= threshold=4.944992542266846
node_1904: feature_name=RELT	RELT.value <= threshold=2.6679846048355103
node_1905: feature_name=TCL1A	TCL1A.value <= threshold=4.318726301193237
node_1906: feature_name=ID1	ID1.value <= threshold=4.261367559432983
node_1907: feature_name=IGJ	IGJ.value <= threshold=-0.3538896441459656
node_1908: feature_name=JARID2	JARID2.value <= threshold=1.1454014778137207
node_1909: feature_name=GRASP	GRASP.value <= threshold=3.5555613040924072

Class: COVID-19 Critical	
Rules_380	passed counts:12
node_0: feature_name=LTB	LTB.value <= threshold=0.994031548500061
node_1: feature_name=MTRNR2L2	MTRNR2L2.value <= threshold=2.8784297704696655
node_2: feature_name=TCL1A	TCL1A.value <= threshold=4.798312664031982
node_3: feature_name=MT-CO1	MT-CO1.value <= threshold=0.08947191759943962
node_4: feature_name=NR4A1	NR4A1.value <= threshold=1.0725348591804504
node_5: feature_name=HLA-DPA1	HLA-DPA1.value > threshold=1.6885469555854797
node_861: feature_name=CD83	CD83.value <= threshold=3.0563642978668213
node_862: feature_name=HLA-DQB1	HLA-DQB1.value > threshold=2.61081063747406
node_1358: feature_name=HLA-DRB5	HLA-DRB5.value > threshold=0.7542744874954224
node_1390: feature_name=HLA-DRB5	HLA-DRB5.value <= threshold=2.6244447231292725
node_1391: feature_name=XIST	XIST.value <= threshold=-0.22017984092235565
node_1392: feature_name=MT-CO3	MT-CO3.value > threshold=0.12647542357444763
node_1472: feature_name=IFI44L	IFI44L.value <= threshold=-0.34079885482788086
node_1473: feature_name=TNFAIP3	TNFAIP3.value <= threshold=-0.0701381303369999
node_1474: feature_name=HSP90B1	HSP90B1.value <= threshold=0.9771479368209839
node_1475: feature_name=CD79A	CD79A.value > threshold=2.6819008588790894
node_1483: feature_name=LTB	LTB.value > threshold=0.06463655456900597
node_1485: feature_name=HLA-DRB5	HLA-DRB5.value > threshold=1.404764175415039
Class: Healthy Control	
Rules_381	passed counts:12
node_0: feature_name=LTB	LTB.value <= threshold=0.994031548500061
node_1: feature_name=MTRNR2L2	MTRNR2L2.value <= threshold=2.8784297704696655
node_2: feature_name=TCL1A	TCL1A.value <= threshold=4.798312664031982
node_3: feature_name=MT-CO1	MT-CO1.value <= threshold=0.08947191759943962
node_4: feature_name=NR4A1	NR4A1.value <= threshold=1.0725348591804504
node_5: feature_name=HLA-DPA1	HLA-DPA1.value > threshold=1.6885469555854797

node_861: feature_name=CD83	CD83.value <= threshold=3.0563642978668213
node_862: feature_name=HLA-DQB1	HLA-DQB1.value <= threshold=2.61081063747406
node_863: feature_name=MTRNR2L2	MTRNR2L2.value <= threshold=2.0919997692108154
node_864: feature_name=BCL2A1	BCL2A1.value <= threshold=1.852618932723999
node_865: feature_name=CD79B	CD79B.value <= threshold=4.352142333984375
node_866: feature_name=TNFAIP3	TNFAIP3.value <= threshold=-0.0449496041983366
node_867: feature_name=MT-CO1	MT-CO1.value > threshold=-0.08531957119703293
node_1155: feature_name=IRF8	IRF8.value > threshold=-0.39239931106567383
node_1177: feature_name=PELI1	PELI1.value > threshold=-0.6234017610549927
node_1203: feature_name=STMN1	STMN1.value <= threshold=-0.2129739671945572
node_1204: feature_name=HLA-DPA1	HLA-DPA1.value > threshold=1.6937929391860962
node_1210: feature_name=MT-CO2	MT-CO2.value <= threshold=-0.5078963339328766
node_1211: feature_name=OAZ1	OAZ1.value > threshold=-0.9088589698076248
node_1213: feature_name=LTB	LTB.value <= threshold=0.30608798563480377
Class: COVID-19 Critical	
Rules_382	passed counts:12
node_0: feature_name=LTB	LTB.value <= threshold=0.994031548500061
node_1: feature_name=MTRNR2L2	MTRNR2L2.value <= threshold=2.8784297704696655
node_2: feature_name=TCL1A	TCL1A.value <= threshold=4.798312664031982
node_3: feature_name=MT-CO1	MT-CO1.value <= threshold=0.08947191759943962
node_4: feature_name=NR4A1	NR4A1.value <= threshold=1.0725348591804504
node_5: feature_name=HLA-DPA1	HLA-DPA1.value <= threshold=1.6885469555854797
node_6: feature_name=HLA-DRB5	HLA-DRB5.value <= threshold=2.1886194944381714
node_7: feature_name=XIST	XIST.value <= threshold=-0.12328458577394485
node_8: feature_name=SYNE1	SYNE1.value > threshold=-0.7835594415664673
node_16: feature_name=MTRNR2L2	MTRNR2L2.value <= threshold=2.4483247995376587
node_17: feature_name=HLA-DQA2	HLA-DQA2.value > threshold=2.227178931236267
node_591: feature_name=BCL2A1	BCL2A1.value > threshold=1.0648428201675415
node_687: feature_name=CD79A	CD79A.value <= threshold=2.49657940864563

node_688: feature_name=MT-ND6	MT-ND6.value > threshold=0.16280143707990646
node_698: feature_name=PARP14	PARP14.value > threshold=-0.49051935970783234
Class: COVID-19 Critical	
Rules_383	passed counts:12
node_0: feature_name=LTB	LTB.value <= threshold=0.994031548500061
node_1: feature_name=MTRNR2L2	MTRNR2L2.value <= threshold=2.8784297704696655
node_2: feature_name=TCL1A	TCL1A.value <= threshold=4.798312664031982
node_3: feature_name=MT-CO1	MT-CO1.value <= threshold=0.08947191759943962
node_4: feature_name=NR4A1	NR4A1.value <= threshold=1.0725348591804504
node_5: feature_name=HLA-DPA1	HLA-DPA1.value <= threshold=1.6885469555854797
node_6: feature_name=HLA-DRB5	HLA-DRB5.value <= threshold=2.1886194944381714
node_7: feature_name=XIST	XIST.value <= threshold=-0.12328458577394485
node_8: feature_name=SYNE1	SYNE1.value > threshold=-0.7835594415664673
node_16: feature_name=MTRNR2L2	MTRNR2L2.value <= threshold=2.4483247995376587
node_17: feature_name=HLA-DQA2	HLA-DQA2.value <= threshold=2.227178931236267
node_18: feature_name=NR4A1	NR4A1.value > threshold=-0.21380723267793655
node_504: feature_name=LTB	LTB.value > threshold=0.8758118152618408
node_584: feature_name=BACH1	BACH1.value <= threshold=-0.5905149877071381
node_585: feature_name=OAZ1	OAZ1.value > threshold=-0.40205591917037964
Class: Healthy Control	
Rules_384	passed counts:12
node_0: feature_name=LTB	LTB.value <= threshold=0.994031548500061
node_1: feature_name=MTRNR2L2	MTRNR2L2.value <= threshold=2.8784297704696655
node_2: feature_name=TCL1A	TCL1A.value <= threshold=4.798312664031982
node_3: feature_name=MT-CO1	MT-CO1.value <= threshold=0.08947191759943962
node_4: feature_name=NR4A1	NR4A1.value <= threshold=1.0725348591804504
node_5: feature_name=HLA-DPA1	HLA-DPA1.value <= threshold=1.6885469555854797
node_6: feature_name=HLA-DRB5	HLA-DRB5.value <= threshold=2.1886194944381714

node_7: feature_name=XIST	XIST.value <= threshold=-0.12328458577394485
node_8: feature_name=SYNE1	SYNE1.value > threshold=-0.7835594415664673
node_16: feature_name=MTRNR2L2	MTRNR2L2.value <= threshold=2.4483247995376587
node_17: feature_name=HLA-DQA2	HLA-DQA2.value <= threshold=2.227178931236267
node_18: feature_name=NR4A1	NR4A1.value <= threshold=-0.21380723267793655
node_19: feature_name=IRAK3	IRAK3.value <= threshold=4.27159571647644
node_20: feature_name=IFIT2	IFIT2.value <= threshold=7.8175740242004395
node_21: feature_name=CD79A	CD79A.value <= threshold=2.857242226600647
node_22: feature_name=HIST1H2AC	HIST1H2AC.value <= threshold=5.077470302581787
node_23: feature_name=FTH1	FTH1.value > threshold=0.4158056080341339
node_285: feature_name=EGR1	EGR1.value <= threshold=4.240467071533203
node_286: feature_name=HLA-DRB1	HLA-DRB1.value > threshold=-0.7553382813930511
node_290: feature_name=TLR2	TLR2.value <= threshold=2.879140257835388
node_291: feature_name=SYNE1	SYNE1.value <= threshold=2.323199510574341
node_292: feature_name=BCL2A1	BCL2A1.value > threshold=1.8940600156784058
node_352: feature_name=HLA-DQB1	HLA-DQB1.value > threshold=1.2327778339385986
Class: COVID-19 Critical	
Rules_385	passed counts:12
node_0: feature_name=LTB	LTB.value <= threshold=0.994031548500061
node_1: feature_name=MTRNR2L2	MTRNR2L2.value <= threshold=2.8784297704696655
node_2: feature_name=TCL1A	TCL1A.value <= threshold=4.798312664031982
node_3: feature_name=MT-CO1	MT-CO1.value <= threshold=0.08947191759943962
node_4: feature_name=NR4A1	NR4A1.value <= threshold=1.0725348591804504
node_5: feature_name=HLA-DPA1	HLA-DPA1.value <= threshold=1.6885469555854797
node_6: feature_name=HLA-DRB5	HLA-DRB5.value <= threshold=2.1886194944381714
node_7: feature_name=XIST	XIST.value <= threshold=-0.12328458577394485
node_8: feature_name=SYNE1	SYNE1.value > threshold=-0.7835594415664673
node_16: feature_name=MTRNR2L2	MTRNR2L2.value <= threshold=2.4483247995376587
node_17: feature_name=HLA-DQA2	HLA-DQA2.value <= threshold=2.227178931236267

node_18: feature_name=NR4A1	NR4A1.value <= threshold=-0.21380723267793655
node_19: feature_name=IRAK3	IRAK3.value <= threshold=4.27159571647644
node_20: feature_name=IFIT2	IFIT2.value <= threshold=7.8175740242004395
node_21: feature_name=CD79A	CD79A.value <= threshold=2.857242226600647
node_22: feature_name=HIST1H2AC	HIST1H2AC.value <= threshold=5.077470302581787
node_23: feature_name=FTH1	FTH1.value <= threshold=0.4158056080341339
node_24: feature_name=ATF3	ATF3.value <= threshold=3.8028353452682495
node_25: feature_name=OASL	OASL.value <= threshold=3.387580633163452
node_26: feature_name=MT-CO1	MT-CO1.value > threshold=-0.7942646145820618
node_74: feature_name=HOPX	HOPX.value <= threshold=0.8682331740856171
node_75: feature_name=RGS2	RGS2.value <= threshold=1.4589954018592834
node_76: feature_name=AHR	AHR.value <= threshold=3.870481848716736
node_77: feature_name=IGJ	IGJ.value <= threshold=4.902882814407349
node_78: feature_name=CD79B	CD79B.value <= threshold=4.87716269493103
node_79: feature_name=HLA-DQB1	HLA-DQB1.value <= threshold=3.6925491094589233
node_80: feature_name=HLA-DRB5	HLA-DRB5.value <= threshold=2.0926843881607056
node_81: feature_name=MCTP1	MCTP1.value <= threshold=4.010472774505615
node_82: feature_name=GNG11	GNG11.value <= threshold=3.627470850944519
node_83: feature_name=HSP90B1	HSP90B1.value <= threshold=2.0835131406784058
node_84: feature_name=ATF3	ATF3.value <= threshold=1.1142374873161316
node_85: feature_name=MT-ND6	MT-ND6.value <= threshold=2.7328014373779297
node_86: feature_name=COTL1	COTL1.value > threshold=-1.6524587869644165
node_90: feature_name=MT-CYB	MT-CYB.value <= threshold=1.099629819393158
node_91: feature_name=RGCC	RGCC.value <= threshold=1.6908138990402222
node_92: feature_name=ILK	ILK.value <= threshold=3.9497616291046143
node_93: feature_name=CTD-2035E11.3	CTD-2035E11.3.value <= threshold=2.4543306827545166
node_94: feature_name=S100A6	S100A6.value <= threshold=0.2867250442504883
node_95: feature_name=HLA-DRB5	HLA-DRB5.value > threshold=-0.9496813416481018
node_99: feature_name=MT-CO3	MT-CO3.value > threshold=-1.55654776096344
node_103: feature_name=ITGAX	ITGAX.value <= threshold=1.8544010519981384

node_104: feature_name=MEF2C	MEF2C.value <= threshold=4.22724461555481
node_105: feature_name=CXorf57	CXorf57.value <= threshold=5.114444732666016
node_106: feature_name=IRAK3	IRAK3.value <= threshold=3.1017903089523315
node_107: feature_name=DUSP4	DUSP4.value <= threshold=2.9122174978256226
node_108: feature_name=OAZ1	OAZ1.value > threshold=-1.8057305812835693
node_112: feature_name=DAPP1	DAPP1.value <= threshold=4.016228437423706
node_113: feature_name=HSP90B1	HSP90B1.value > threshold=-1.2981283068656921
node_119: feature_name=MT-CYB	MT-CYB.value <= threshold=0.9500376582145691
node_120: feature_name=HSP90B1	HSP90B1.value > threshold=1.7814298272132874
node_126: feature_name=HIST1H2AC	HIST1H2AC.value <= threshold=0.9089176803827286
Class: COVID-19 Critical	
Rules_386	passed counts:12
node_0: feature_name=LTB	LTB.value <= threshold=0.994031548500061
node_1: feature_name=MTRNR2L2	MTRNR2L2.value <= threshold=2.8784297704696655
node_2: feature_name=TCL1A	TCL1A.value <= threshold=4.798312664031982
node_3: feature_name=MT-CO1	MT-CO1.value <= threshold=0.08947191759943962
node_4: feature_name=NR4A1	NR4A1.value <= threshold=1.0725348591804504
node_5: feature_name=HLA-DPA1	HLA-DPA1.value <= threshold=1.6885469555854797
node_6: feature_name=HLA-DRB5	HLA-DRB5.value <= threshold=2.1886194944381714
node_7: feature_name=XIST	XIST.value <= threshold=-0.12328458577394485
node_8: feature_name=SYNE1	SYNE1.value > threshold=-0.7835594415664673
node_16: feature_name=MTRNR2L2	MTRNR2L2.value <= threshold=2.4483247995376587
node_17: feature_name=HLA-DQA2	HLA-DQA2.value <= threshold=2.227178931236267
node_18: feature_name=NR4A1	NR4A1.value <= threshold=-0.21380723267793655
node_19: feature_name=IRAK3	IRAK3.value <= threshold=4.27159571647644
node_20: feature_name=IFIT2	IFIT2.value <= threshold=7.8175740242004395
node_21: feature_name=CD79A	CD79A.value <= threshold=2.857242226600647
node_22: feature_name=HIST1H2AC	HIST1H2AC.value <= threshold=5.077470302581787
node_23: feature_name=FTH1	FTH1.value <= threshold=0.4158056080341339

node_24: feature_name=ATF3	ATF3.value <= threshold=3.8028353452682495
node_25: feature_name=OASL	OASL.value <= threshold=3.387580633163452
node_26: feature_name=MT-CO1	MT-CO1.value <= threshold=-0.7942646145820618
node_27: feature_name=FCER2	FCER2.value <= threshold=7.268347978591919
node_28: feature_name=CCL3	CCL3.value <= threshold=3.2822797298431396
node_29: feature_name=BCL2A1	BCL2A1.value <= threshold=3.1504160165786743
node_30: feature_name=GRAP2	GRAP2.value <= threshold=3.0517499446868896
node_31: feature_name=EGR1	EGR1.value <= threshold=7.645251512527466
node_32: feature_name=GPX1	GPX1.value <= threshold=0.6752602756023407
node_33: feature_name=CD79B	CD79B.value <= threshold=4.365710258483887
node_34: feature_name=S100A6	S100A6.value <= threshold=0.4172801077365875
node_35: feature_name=ID3	ID3.value <= threshold=3.093442916870117
node_36: feature_name=ATF3	ATF3.value <= threshold=2.6047290563583374
node_37: feature_name=PLA2G12A	PLA2G12A.value > threshold=3.5468435287475586
node_39: feature_name=PARP14	PARP14.value <= threshold=2.4915021657943726
Class: COVID-19 Critical	
Rules_387	passed counts:11
node_0: feature_name=LTB	LTB.value > threshold=0.994031548500061
node_5264: feature_name=MT-CO1	MT-CO1.value > threshold=-0.4127991199493408
node_5638: feature_name=OAZ1	OAZ1.value > threshold=-0.2640867084264755
node_6112: feature_name=IFI44L	IFI44L.value <= threshold=2.5893263816833496
node_6113: feature_name=TCL1A	TCL1A.value > threshold=4.201832294464111
node_6991: feature_name=XAF1	XAF1.value <= threshold=3.4585667848587036
node_6992: feature_name=STAG3	STAG3.value <= threshold=7.686291694641113
node_6993: feature_name=RALGPS2	RALGPS2.value > threshold=-0.4090157449245453
node_6997: feature_name=MTRNR2L2	MTRNR2L2.value <= threshold=3.23723828792572
node_6998: feature_name=TP53INP1	TP53INP1.value <= threshold=6.239925146102905
node_6999: feature_name=MEF2C	MEF2C.value > threshold=-0.44642284512519836
node_7003: feature_name=HSPA1A	HSPA1A.value <= threshold=7.404359817504883

node_7004: feature_name=COTL1	COTL1.value > threshold=-1.6598749160766602
node_7006: feature_name=HIST1H2AC	HIST1H2AC.value > threshold=-0.34910088777542114
node_7008: feature_name=MARCH1	MARCH1.value <= threshold=5.393903970718384
node_7009: feature_name=PELI1	PELI1.value <= threshold=3.565749168395996
node_7010: feature_name=RGS1	RGS1.value <= threshold=2.851950168609619
node_7011: feature_name=HLA-DRB5	HLA-DRB5.value <= threshold=3.0528379678726196
node_7012: feature_name=MEF2A	MEF2A.value > threshold=3.228689193725586
node_7056: feature_name=TCL1A	TCL1A.value > threshold=4.69646143913269
Class: Healthy Control	
Rules_388	passed counts:11
node_0: feature_name=LTB	LTB.value > threshold=0.994031548500061
node_5264: feature_name=MT-CO1	MT-CO1.value > threshold=-0.4127991199493408
node_5638: feature_name=OAZ1	OAZ1.value > threshold=-0.2640867084264755
node_6112: feature_name=IFI44L	IFI44L.value <= threshold=2.5893263816833496
node_6113: feature_name=TCL1A	TCL1A.value <= threshold=4.201832294464111
node_6114: feature_name=HLA-DQA2	HLA-DQA2.value > threshold=-0.2270243540406227
node_6542: feature_name=CD79B	CD79B.value > threshold=3.096232771873474
node_6872: feature_name=ICAM1	ICAM1.value > threshold=-0.2232399433851242
node_6974: feature_name=HSP90B1	HSP90B1.value <= threshold=-0.9936705231666565
node_6975: feature_name=FTH1	FTH1.value > threshold=-0.6905665695667267
node_6977: feature_name=MARCKS	MARCKS.value <= threshold=1.9962652921676636
Class: Healthy Control	
Rules_389	passed counts:11
node_0: feature_name=LTB	LTB.value > threshold=0.994031548500061
node_5264: feature_name=MT-CO1	MT-CO1.value > threshold=-0.4127991199493408
node_5638: feature_name=OAZ1	OAZ1.value > threshold=-0.2640867084264755
node_6112: feature_name=IFI44L	IFI44L.value <= threshold=2.5893263816833496
node_6113: feature_name=TCL1A	TCL1A.value <= threshold=4.201832294464111

node_6114: feature_name=HLA-DQA2	HLA-DQA2.value > threshold=-0.2270243540406227
node_6542: feature_name=CD79B	CD79B.value <= threshold=3.096232771873474
node_6543: feature_name=NR4A1	NR4A1.value <= threshold=1.421015739440918
node_6544: feature_name=LTB	LTB.value <= threshold=1.359755277633667
node_6545: feature_name=NFKBID	NFKBID.value > threshold=1.4294711351394653
node_6643: feature_name=FAM129C	FAM129C.value <= threshold=4.799884080886841
node_6644: feature_name=XIST	XIST.value <= threshold=0.9931697957217693
node_6645: feature_name=GPX1	GPX1.value > threshold=0.3628883808851242
node_6695: feature_name=CD79A	CD79A.value <= threshold=2.4297949075698853
node_6696: feature_name=RGCC	RGCC.value <= threshold=0.4576346427202225
Class: COVID-19 Critical	
Rules_390	passed counts:11
node_0: feature_name=LTB	LTB.value > threshold=0.994031548500061
node_5264: feature_name=MT-CO1	MT-CO1.value > threshold=-0.4127991199493408
node_5638: feature_name=OAZ1	OAZ1.value <= threshold=-0.2640867084264755
node_5639: feature_name=CD79B	CD79B.value > threshold=2.2998517751693726
node_5925: feature_name=SNX9	SNX9.value > threshold=2.4445992708206177
node_6083: feature_name=RALGPS2	RALGPS2.value > threshold=4.061077356338501
node_6105: feature_name=XAF1	XAF1.value > threshold=-0.4288857579231262
node_6109: feature_name=PARP14	PARP14.value <= threshold=3.2161394357681274
Class: Healthy Control	
Rules_391	passed counts:11
node_0: feature_name=LTB	LTB.value > threshold=0.994031548500061
node_5264: feature_name=MT-CO1	MT-CO1.value > threshold=-0.4127991199493408
node_5638: feature_name=OAZ1	OAZ1.value <= threshold=-0.2640867084264755
node_5639: feature_name=CD79B	CD79B.value > threshold=2.2998517751693726
node_5925: feature_name=SNX9	SNX9.value <= threshold=2.4445992708206177
node_5926: feature_name=TCL1A	TCL1A.value <= threshold=2.6938670873641968

node_5927: feature_name=PLD4	PLD4.value > threshold=-0.06434785947203636
node_6009: feature_name=MX1	MX1.value <= threshold=3.600893020629883
node_6010: feature_name=PRDM1	PRDM1.value <= threshold=-0.407244011759758
node_6011: feature_name=SNORD3B-2	SNORD3B-2.value <= threshold=2.7082353830337524
node_6012: feature_name=NFKBID	NFKBID.value > threshold=3.0716724395751953
node_6034: feature_name=BANK1	BANK1.value > threshold=2.832552671432495
node_6036: feature_name=MAP3K2	MAP3K2.value <= threshold=2.1622473001480103
node_6037: feature_name=STMN1	STMN1.value <= threshold=-0.20975803583860397
Class: Healthy Control	
Rules_392	passed counts:11
node_0: feature_name=LTB	LTB.value > threshold=0.994031548500061
node_5264: feature_name=MT-CO1	MT-CO1.value > threshold=-0.4127991199493408
node_5638: feature_name=OAZ1	OAZ1.value <= threshold=-0.2640867084264755
node_5639: feature_name=CD79B	CD79B.value <= threshold=2.2998517751693726
node_5640: feature_name=MTRNR2L2	MTRNR2L2.value <= threshold=3.5499863624572754
node_5641: feature_name=MT-CO1	MT-CO1.value > threshold=0.22567133605480194
node_5729: feature_name=MARCH1	MARCH1.value <= threshold=1.5154643654823303
node_5730: feature_name=MT-CO1	MT-CO1.value <= threshold=1.7823500633239746
node_5731: feature_name=HLA-DPB1	HLA-DPB1.value > threshold=2.0096163749694824
node_5829: feature_name=LTB	LTB.value > threshold=1.2124510407447815
node_5843: feature_name=TTN	TTN.value <= threshold=-0.3821280151605606
node_5844: feature_name=FAM129C	FAM129C.value <= threshold=6.290640115737915
node_5845: feature_name=FCER2	FCER2.value > threshold=-0.27513572573661804
node_5847: feature_name=MT-CO3	MT-CO3.value > threshold=0.2649626359343529
Class: COVID-19 Critical	
Rules_393	passed counts:11
node_0: feature_name=LTB	LTB.value > threshold=0.994031548500061
node_5264: feature_name=MT-CO1	MT-CO1.value > threshold=-0.4127991199493408

node_5638: feature_name=OAZ1	OAZ1.value <= threshold=-0.2640867084264755
node_5639: feature_name=CD79B	CD79B.value <= threshold=2.2998517751693726
node_5640: feature_name=MTRNR2L2	MTRNR2L2.value <= threshold=3.5499863624572754
node_5641: feature_name=MT-CO1	MT-CO1.value > threshold=0.22567133605480194
node_5729: feature_name=MARCH1	MARCH1.value <= threshold=1.5154643654823303
node_5730: feature_name=MT-CO1	MT-CO1.value <= threshold=1.7823500633239746
node_5731: feature_name=HLA-DPB1	HLA-DPB1.value <= threshold=2.0096163749694824
node_5732: feature_name=NR4A3	NR4A3.value <= threshold=3.509189486503601
node_5733: feature_name=OAZ1	OAZ1.value > threshold=-0.4392261207103729
node_5803: feature_name=BACH1	BACH1.value > threshold=1.211863934993744
node_5815: feature_name=S100A6	S100A6.value > threshold=-1.7154203653335571
node_5819: feature_name=FCER2	FCER2.value > threshold=-0.24423103779554367
Class: COVID-19 Critical	
Rules_394	passed counts:11
node_0: feature_name=LTB	LTB.value > threshold=0.994031548500061
node_5264: feature_name=MT-CO1	MT-CO1.value <= threshold=-0.4127991199493408
node_5265: feature_name=MTRNR2L2	MTRNR2L2.value <= threshold=2.4473838806152344
node_5266: feature_name=TCL1A	TCL1A.value <= threshold=5.787256956100464
node_5267: feature_name=PLD4	PLD4.value <= threshold=3.544657826423645
node_5268: feature_name=NR4A1	NR4A1.value <= threshold=0.8490704298019409
node_5269: feature_name=LTB	LTB.value <= threshold=1.5252206921577454
node_5270: feature_name=HLA-DRB5	HLA-DRB5.value <= threshold=2.5117164850234985
node_5271: feature_name=CD79A	CD79A.value <= threshold=3.685201406478882
node_5272: feature_name=BCL2A1	BCL2A1.value > threshold=2.081526756286621
node_5438: feature_name=RALGPS2	RALGPS2.value > threshold=2.112855315208435
node_5444: feature_name=HLA-DRB1	HLA-DRB1.value > threshold=0.8363427221775055
Class: COVID-19 Critical	
Rules_395	passed counts:11

node_0: feature_name=LTB	LTB.value <= threshold=0.994031548500061
node_1: feature_name=MTRNR2L2	MTRNR2L2.value <= threshold=2.8784297704696655
node_2: feature_name=TCL1A	TCL1A.value > threshold=4.798312664031982
node_4912: feature_name=MT-CO1	MT-CO1.value > threshold=-0.3664844036102295
node_5002: feature_name=IFI44L	IFI44L.value <= threshold=2.3101747035980225
node_5003: feature_name=SNX9	SNX9.value <= threshold=2.0247421264648438
node_5004: feature_name=STAG3	STAG3.value <= threshold=6.482141733169556
node_5005: feature_name=BACH1	BACH1.value > threshold=-0.5237349569797516
node_5047: feature_name=RHOB	RHOB.value > threshold=-0.4843696355819702
node_5055: feature_name=FAM129C	FAM129C.value > threshold=-0.2832885980606079
node_5079: feature_name=TP53INP1	TP53INP1.value > threshold=2.9482468366622925
node_5129: feature_name=CD79B	CD79B.value > threshold=2.402374267578125
node_5135: feature_name=ABCA1	ABCA1.value > threshold=-0.2616613954305649
Class: Healthy Control	
Rules_396	passed counts:11
node_0: feature_name=LTB	LTB.value <= threshold=0.994031548500061
node_1: feature_name=MTRNR2L2	MTRNR2L2.value <= threshold=2.8784297704696655
node_2: feature_name=TCL1A	TCL1A.value <= threshold=4.798312664031982
node_3: feature_name=MT-CO1	MT-CO1.value > threshold=0.08947191759943962
node_2521: feature_name=LTB	LTB.value > threshold=0.6038667261600494
node_4173: feature_name=OAZ1	OAZ1.value > threshold=0.143283873796463
node_4615: feature_name=NR4A1	NR4A1.value > threshold=0.6621004343032837
node_4817: feature_name=HSP90B1	HSP90B1.value <= threshold=1.1699737310409546
node_4818: feature_name=XAF1	XAF1.value <= threshold=2.6752071380615234
node_4819: feature_name=SNX9	SNX9.value <= threshold=2.939147114753723
node_4820: feature_name=HLA-DQA2	HLA-DQA2.value > threshold=2.9822239875793457
node_4874: feature_name=MARCH1	MARCH1.value <= threshold=-0.3389042466878891
node_4875: feature_name=ICAM1	ICAM1.value > threshold=-0.36889341473579407
node_4879: feature_name=SNX9	SNX9.value <= threshold=2.27950519323349

Class: COVID-19 Critical	
Rules_397	passed counts:11
node_0: feature_name=LTB	LTB.value <= threshold=0.994031548500061
node_1: feature_name=MTRNR2L2	MTRNR2L2.value <= threshold=2.8784297704696655
node_2: feature_name=TCL1A	TCL1A.value <= threshold=4.798312664031982
node_3: feature_name=MT-CO1	MT-CO1.value > threshold=0.08947191759943962
node_2521: feature_name=LTB	LTB.value <= threshold=0.6038667261600494
node_2522: feature_name=HLA-DQB1	HLA-DQB1.value > threshold=2.1053054332733154
node_3550: feature_name=XIST	XIST.value > threshold=0.7716337237507105
node_4140: feature_name=HLA-DQA2	HLA-DQA2.value > threshold=-0.1354314684867859
node_4158: feature_name=MT-CO3	MT-CO3.value > threshold=0.5339524000883102
node_4166: feature_name=IL1B	IL1B.value <= threshold=-0.16591781377792358
Class: Healthy Control	
Rules_398	passed counts:11
node_0: feature_name=LTB	LTB.value <= threshold=0.994031548500061
node_1: feature_name=MTRNR2L2	MTRNR2L2.value <= threshold=2.8784297704696655
node_2: feature_name=TCL1A	TCL1A.value <= threshold=4.798312664031982
node_3: feature_name=MT-CO1	MT-CO1.value > threshold=0.08947191759943962
node_2521: feature_name=LTB	LTB.value <= threshold=0.6038667261600494
node_2522: feature_name=HLA-DQB1	HLA-DQB1.value <= threshold=2.1053054332733154
node_2523: feature_name=SNX9	SNX9.value > threshold=-0.5346486568450928
node_2907: feature_name=NR4A1	NR4A1.value <= threshold=2.5433837175369263
node_2908: feature_name=XIST	XIST.value <= threshold=0.7664470989257097
node_2909: feature_name=CD79B	CD79B.value > threshold=2.9326847791671753
node_3353: feature_name=VPREB3	VPREB3.value <= threshold=3.0226117372512817
node_3354: feature_name=CD83	CD83.value > threshold=1.9074636101722717
node_3388: feature_name=BOD1L1	BOD1L1.value <= threshold=-0.4742381125688553
node_3389: feature_name=MT-ND6	MT-ND6.value <= threshold=0.3542506694793701

node_3390: feature_name=JUN	JUN.value > threshold=0.5178887248039246
node_3396: feature_name=SNX9	SNX9.value <= threshold=2.9811075925827026
Class: COVID-19 Critical	
Rules_399	passed counts:11
node_0: feature_name=LTB	LTB.value <= threshold=0.994031548500061
node_1: feature_name=MTRNR2L2	MTRNR2L2.value <= threshold=2.8784297704696655
node_2: feature_name=TCL1A	TCL1A.value <= threshold=4.798312664031982
node_3: feature_name=MT-CO1	MT-CO1.value > threshold=0.08947191759943962
node_2521: feature_name=LTB	LTB.value <= threshold=0.6038667261600494
node_2522: feature_name=HLA-DQB1	HLA-DQB1.value <= threshold=2.1053054332733154
node_2523: feature_name=SNX9	SNX9.value > threshold=-0.5346486568450928
node_2907: feature_name=NR4A1	NR4A1.value <= threshold=2.5433837175369263
node_2908: feature_name=XIST	XIST.value <= threshold=0.7664470989257097
node_2909: feature_name=CD79B	CD79B.value <= threshold=2.9326847791671753
node_2910: feature_name=NR4A1	NR4A1.value > threshold=-0.2875557690858841
node_3096: feature_name=NR4A3	NR4A3.value <= threshold=3.314968705177307
node_3097: feature_name=VPREB3	VPREB3.value > threshold=3.3549938201904297
node_3285: feature_name=OAZ1	OAZ1.value > threshold=0.11267071589827538
node_3309: feature_name=SNX9	SNX9.value > threshold=1.7356606721878052
node_3317: feature_name=BCL2A1	BCL2A1.value <= threshold=0.8642410039901733
node_3318: feature_name=TLN1	TLN1.value <= threshold=0.7824493050575256
Class: COVID-19 Critical	
Rules_400	passed counts:11
node_0: feature_name=LTB	LTB.value <= threshold=0.994031548500061
node_1: feature_name=MTRNR2L2	MTRNR2L2.value <= threshold=2.8784297704696655
node_2: feature_name=TCL1A	TCL1A.value <= threshold=4.798312664031982
node_3: feature_name=MT-CO1	MT-CO1.value > threshold=0.08947191759943962
node_2521: feature_name=LTB	LTB.value <= threshold=0.6038667261600494

node_2522: feature_name=HLA-DQB1	HLA-DQB1.value <= threshold=2.1053054332733154
node_2523: feature_name=SNX9	SNX9.value <= threshold=-0.5346486568450928
node_2524: feature_name=HLA-DPB1	HLA-DPB1.value <= threshold=1.9548270106315613
node_2525: feature_name=FAM129C	FAM129C.value <= threshold=3.3393388986587524
node_2526: feature_name=XIST	XIST.value <= threshold=3.6911416053771973
node_2527: feature_name=NR4A1	NR4A1.value > threshold=-0.2557755410671234
node_2625: feature_name=HLA-DPA1	HLA-DPA1.value > threshold=0.9335225224494934
node_2657: feature_name=RALGPS2	RALGPS2.value > threshold=2.9413715600967407
node_2701: feature_name=DUSP4	DUSP4.value > threshold=-0.24480421096086502
node_2715: feature_name=BACH1	BACH1.value <= threshold=3.6674370765686035
Class: COVID-19 Critical	
Rules_401	passed counts:11
node_0: feature_name=LTB	LTB.value <= threshold=0.994031548500061
node_1: feature_name=MTRNR2L2	MTRNR2L2.value <= threshold=2.8784297704696655
node_2: feature_name=TCL1A	TCL1A.value <= threshold=4.798312664031982
node_3: feature_name=MT-CO1	MT-CO1.value > threshold=0.08947191759943962
node_2521: feature_name=LTB	LTB.value <= threshold=0.6038667261600494
node_2522: feature_name=HLA-DQB1	HLA-DQB1.value <= threshold=2.1053054332733154
node_2523: feature_name=SNX9	SNX9.value <= threshold=-0.5346486568450928
node_2524: feature_name=HLA-DPB1	HLA-DPB1.value <= threshold=1.9548270106315613
node_2525: feature_name=FAM129C	FAM129C.value <= threshold=3.3393388986587524
node_2526: feature_name=XIST	XIST.value <= threshold=3.6911416053771973
node_2527: feature_name=NR4A1	NR4A1.value > threshold=-0.2557755410671234
node_2625: feature_name=HLA-DPA1	HLA-DPA1.value <= threshold=0.9335225224494934
node_2626: feature_name=CD83	CD83.value > threshold=-0.585537850856781
node_2628: feature_name=PDLIM1	PDLIM1.value <= threshold=5.292127370834351
node_2629: feature_name=ISG15	ISG15.value <= threshold=3.357967495918274
node_2630: feature_name=BACH1	BACH1.value <= threshold=4.870309114456177
node_2631: feature_name=LDLRAD4	LDLRAD4.value > threshold=-0.2641522139310837

node_2641: feature_name=CD79B	CD79B.value > threshold=-0.23006416112184525
node_2649: feature_name=MT-CO3	MT-CO3.value <= threshold=2.134994387626648
Class: COVID-19 Critical	
Rules_402	passed counts:11
node_0: feature_name=LTB	LTB.value <= threshold=0.994031548500061
node_1: feature_name=MTRNR2L2	MTRNR2L2.value <= threshold=2.8784297704696655
node_2: feature_name=TCL1A	TCL1A.value <= threshold=4.798312664031982
node_3: feature_name=MT-CO1	MT-CO1.value <= threshold=0.08947191759943962
node_4: feature_name=NR4A1	NR4A1.value > threshold=1.0725348591804504
node_1622: feature_name=HLA-DQA2	HLA-DQA2.value > threshold=1.9496816992759705
node_2350: feature_name=BCL2A1	BCL2A1.value > threshold=1.1322240233421326
node_2466: feature_name=MS4A1	MS4A1.value <= threshold=2.7321707010269165
node_2467: feature_name=HSP90B1	HSP90B1.value <= threshold=0.8215908706188202
node_2468: feature_name=HLA-DQA1	HLA-DQA1.value <= threshold=1.4212530851364136
node_2469: feature_name=STMN1	STMN1.value <= threshold=-0.25645384192466736
node_2470: feature_name=RALGPS2	RALGPS2.value <= threshold=3.605595350265503
Class: COVID-19 Critical	
Rules_403	passed counts:11
node_0: feature_name=LTB	LTB.value <= threshold=0.994031548500061
node_1: feature_name=MTRNR2L2	MTRNR2L2.value <= threshold=2.8784297704696655
node_2: feature_name=TCL1A	TCL1A.value <= threshold=4.798312664031982
node_3: feature_name=MT-CO1	MT-CO1.value <= threshold=0.08947191759943962
node_4: feature_name=NR4A1	NR4A1.value > threshold=1.0725348591804504
node_1622: feature_name=HLA-DQA2	HLA-DQA2.value <= threshold=1.9496816992759705
node_1623: feature_name=HLA-DRB5	HLA-DRB5.value <= threshold=2.715766429901123
node_1624: feature_name=HLA-DPA1	HLA-DPA1.value > threshold=1.7050301432609558
node_2056: feature_name=OAZ1	OAZ1.value > threshold=0.456103190779686
node_2266: feature_name=XAF1	XAF1.value <= threshold=-0.4431648850440979

node_2267: feature_name=MT-CO1	MT-CO1.value <= threshold=-0.3505740910768509
node_2268: feature_name=HLA-DRB1	HLA-DRB1.value <= threshold=2.1576695442199707
node_2269: feature_name=MARCH1	MARCH1.value <= threshold=1.492592692375183
node_2270: feature_name=CD83	CD83.value > threshold=1.7977983355522156
node_2274: feature_name=HLA-DRB5	HLA-DRB5.value <= threshold=1.7145949006080627
node_2275: feature_name=EGR1	EGR1.value <= threshold=4.1976988315582275
Class: COVID-19 Severe	
Rules_404	passed counts:11
node_0: feature_name=LTB	LTB.value <= threshold=0.994031548500061
node_1: feature_name=MTRNR2L2	MTRNR2L2.value <= threshold=2.8784297704696655
node_2: feature_name=TCL1A	TCL1A.value <= threshold=4.798312664031982
node_3: feature_name=MT-CO1	MT-CO1.value <= threshold=0.08947191759943962
node_4: feature_name=NR4A1	NR4A1.value > threshold=1.0725348591804504
node_1622: feature_name=HLA-DQA2	HLA-DQA2.value <= threshold=1.9496816992759705
node_1623: feature_name=HLA-DRB5	HLA-DRB5.value <= threshold=2.715766429901123
node_1624: feature_name=HLA-DPA1	HLA-DPA1.value > threshold=1.7050301432609558
node_2056: feature_name=OAZ1	OAZ1.value <= threshold=0.456103190779686
node_2057: feature_name=HLA-DRB5	HLA-DRB5.value > threshold=0.6839984953403473
node_2101: feature_name=HLA-DRB1	HLA-DRB1.value > threshold=1.6199676990509033
node_2151: feature_name=HLA-DQB1	HLA-DQB1.value <= threshold=3.1961567401885986
node_2152: feature_name=PLD4	PLD4.value > threshold=-0.03259922005236149
node_2244: feature_name=LTB	LTB.value <= threshold=0.3717459514737129
node_2245: feature_name=HSP90B1	HSP90B1.value <= threshold=1.4108100533485413
node_2246: feature_name=ICAM1	ICAM1.value <= threshold=1.767152488231659
Class: COVID-19 Critical	
Rules_405	passed counts:11
node_0: feature_name=LTB	LTB.value <= threshold=0.994031548500061
node_1: feature_name=MTRNR2L2	MTRNR2L2.value <= threshold=2.8784297704696655

node_2: feature_name=TCL1A	TCL1A.value <= threshold=4.798312664031982
node_3: feature_name=MT-CO1	MT-CO1.value <= threshold=0.08947191759943962
node_4: feature_name=NR4A1	NR4A1.value > threshold=1.0725348591804504
node_1622: feature_name=HLA-DQA2	HLA-DQA2.value <= threshold=1.9496816992759705
node_1623: feature_name=HLA-DRB5	HLA-DRB5.value <= threshold=2.715766429901123
node_1624: feature_name=HLA-DPA1	HLA-DPA1.value > threshold=1.7050301432609558
node_2056: feature_name=OAZ1	OAZ1.value <= threshold=0.456103190779686
node_2057: feature_name=HLA-DRB5	HLA-DRB5.value > threshold=0.6839984953403473
node_2101: feature_name=HLA-DRB1	HLA-DRB1.value <= threshold=1.6199676990509033
node_2102: feature_name=CD83	CD83.value <= threshold=2.5568599700927734
node_2103: feature_name=MT-CO1	MT-CO1.value > threshold=-0.08588453382253647
node_2119: feature_name=LTB	LTB.value > threshold=0.19341976940631866
Class: Healthy Control	
Rules_406	passed counts:11
node_0: feature_name=LTB	LTB.value <= threshold=0.994031548500061
node_1: feature_name=MTRNR2L2	MTRNR2L2.value <= threshold=2.8784297704696655
node_2: feature_name=TCL1A	TCL1A.value <= threshold=4.798312664031982
node_3: feature_name=MT-CO1	MT-CO1.value <= threshold=0.08947191759943962
node_4: feature_name=NR4A1	NR4A1.value > threshold=1.0725348591804504
node_1622: feature_name=HLA-DQA2	HLA-DQA2.value <= threshold=1.9496816992759705
node_1623: feature_name=HLA-DRB5	HLA-DRB5.value <= threshold=2.715766429901123
node_1624: feature_name=HLA-DPA1	HLA-DPA1.value <= threshold=1.7050301432609558
node_1625: feature_name=CD83	CD83.value > threshold=2.783489227294922
node_1873: feature_name=IFI44L	IFI44L.value <= threshold=1.5938722491264343
node_1874: feature_name=PLD4	PLD4.value > threshold=4.101001739501953
node_2028: feature_name=SNX9	SNX9.value <= threshold=1.4460421800613403
node_2029: feature_name=MT-CO3	MT-CO3.value > threshold=-0.40799812972545624
Class: Healthy Control	

Rules_407	passed counts:11
node_0: feature_name=LTB	LTB.value <= threshold=0.994031548500061
node_1: feature_name=MTRNR2L2	MTRNR2L2.value <= threshold=2.8784297704696655
node_2: feature_name=TCL1A	TCL1A.value <= threshold=4.798312664031982
node_3: feature_name=MT-CO1	MT-CO1.value <= threshold=0.08947191759943962
node_4: feature_name=NR4A1	NR4A1.value > threshold=1.0725348591804504
node_1622: feature_name=HLA-DQA2	HLA-DQA2.value <= threshold=1.9496816992759705
node_1623: feature_name=HLA-DRB5	HLA-DRB5.value <= threshold=2.715766429901123
node_1624: feature_name=HLA-DPA1	HLA-DPA1.value <= threshold=1.7050301432609558
node_1625: feature_name=CD83	CD83.value > threshold=2.783489227294922
node_1873: feature_name=IFI44L	IFI44L.value <= threshold=1.5938722491264343
node_1874: feature_name=PLD4	PLD4.value <= threshold=4.101001739501953
node_1875: feature_name=RHOB	RHOB.value > threshold=1.304835319519043
node_1965: feature_name=HLA-DQB1	HLA-DQB1.value > threshold=1.5989137291908264
node_1991: feature_name=COTL1	COTL1.value > threshold=-1.2334879636764526
node_1997: feature_name=HSP90B1	HSP90B1.value <= threshold=1.903715431690216
node_1998: feature_name=NFKBID	NFKBID.value <= threshold=1.8163637518882751
node_1999: feature_name=IFI6	IFI6.value > threshold=-0.5193088352680206
Class: COVID-19 Critical	
Rules_408	passed counts:11
node_0: feature_name=LTB	LTB.value <= threshold=0.994031548500061
node_1: feature_name=MTRNR2L2	MTRNR2L2.value <= threshold=2.8784297704696655
node_2: feature_name=TCL1A	TCL1A.value <= threshold=4.798312664031982
node_3: feature_name=MT-CO1	MT-CO1.value <= threshold=0.08947191759943962
node_4: feature_name=NR4A1	NR4A1.value > threshold=1.0725348591804504
node_1622: feature_name=HLA-DQA2	HLA-DQA2.value <= threshold=1.9496816992759705
node_1623: feature_name=HLA-DRB5	HLA-DRB5.value <= threshold=2.715766429901123
node_1624: feature_name=HLA-DPA1	HLA-DPA1.value <= threshold=1.7050301432609558
node_1625: feature_name=CD83	CD83.value > threshold=2.783489227294922

node_1873: feature_name=IFI44L	IFI44L.value <= threshold=1.5938722491264343
node_1874: feature_name=PLD4	PLD4.value <= threshold=4.101001739501953
node_1875: feature_name=RHOB	RHOB.value <= threshold=1.304835319519043
node_1876: feature_name=PPIF	PPIF.value <= threshold=2.2943923473358154
node_1877: feature_name=OAZ1	OAZ1.value <= threshold=0.04260866530239582
node_1878: feature_name=ID3	ID3.value <= threshold=5.290210247039795
node_1879: feature_name=NFKBID	NFKBID.value > threshold=-0.40532417595386505
node_1883: feature_name=MEF2C	MEF2C.value > threshold=-0.4597593992948532
node_1887: feature_name=SNX9	SNX9.value > threshold=1.010923683643341
node_1901: feature_name=RBKS	RBKS.value <= threshold=4.642704963684082
node_1902: feature_name=TSPYL2	TSPYL2.value <= threshold=2.4923402070999146
node_1903: feature_name=MARCKS	MARCKS.value <= threshold=4.944992542266846
node_1904: feature_name=RELT	RELT.value <= threshold=2.6679846048355103
node_1905: feature_name=TCL1A	TCL1A.value <= threshold=4.318726301193237
node_1906: feature_name=ID1	ID1.value <= threshold=4.261367559432983
node_1907: feature_name=IGJ	IGJ.value > threshold=-0.3538896441459656
node_1915: feature_name=HLA-DRB5	HLA-DRB5.value > threshold=2.2267305850982666
node_1917: feature_name=CD22	CD22.value <= threshold=-0.22111180424690247
Class: COVID-19 Critical	
Rules_409	passed counts:11
node_0: feature_name=LTB	LTB.value <= threshold=0.994031548500061
node_1: feature_name=MTRNR2L2	MTRNR2L2.value <= threshold=2.8784297704696655
node_2: feature_name=TCL1A	TCL1A.value <= threshold=4.798312664031982
node_3: feature_name=MT-CO1	MT-CO1.value <= threshold=0.08947191759943962
node_4: feature_name=NR4A1	NR4A1.value > threshold=1.0725348591804504
node_1622: feature_name=HLA-DQA2	HLA-DQA2.value <= threshold=1.9496816992759705
node_1623: feature_name=HLA-DRB5	HLA-DRB5.value <= threshold=2.715766429901123
node_1624: feature_name=HLA-DPA1	HLA-DPA1.value <= threshold=1.7050301432609558
node_1625: feature_name=CD83	CD83.value <= threshold=2.783489227294922

node_1626: feature_name=XIST	XIST.value <= threshold=0.9589543007314205
node_1627: feature_name=PLD4	PLD4.value <= threshold=5.865932464599609
node_1628: feature_name=OAZ1	OAZ1.value <= threshold=0.6137169003486633
node_1629: feature_name=MTRNR2L2	MTRNR2L2.value <= threshold=1.6102792620658875
node_1630: feature_name=IFI6	IFI6.value > threshold=-0.8404486179351807
node_1632: feature_name=NR4A1	NR4A1.value > threshold=1.0916320085525513
node_1638: feature_name=DENND3	DENND3.value <= threshold=3.988452672958374
node_1639: feature_name=HLA-DRB1	HLA-DRB1.value <= threshold=1.4092575907707214
node_1640: feature_name=HLA-DPA1	HLA-DPA1.value <= threshold=1.2675696015357971
node_1641: feature_name=TRAF1	TRAF1.value <= threshold=2.918454885482788
node_1642: feature_name=HLA-DPA1	HLA-DPA1.value > threshold=-0.917132705450058
node_1648: feature_name=LTB	LTB.value <= threshold=0.9716593027114868
node_1649: feature_name=LDLRAD4	LDLRAD4.value <= threshold=4.4251909255981445
node_1650: feature_name=BCL2A1	BCL2A1.value > threshold=-0.7959703803062439
node_1652: feature_name=PRDM1	PRDM1.value <= threshold=2.6821553707122803
node_1653: feature_name=FAM129C	FAM129C.value <= threshold=4.9084858894348145
node_1654: feature_name=BOD1L1	BOD1L1.value <= threshold=2.9830023050308228
node_1655: feature_name=HSP90B1	HSP90B1.value <= threshold=2.581766724586487
node_1656: feature_name=HLA-DQB1	HLA-DQB1.value <= threshold=3.3723777532577515
node_1657: feature_name=ANO6	ANO6.value <= threshold=2.491254210472107
node_1658: feature_name=RP5-821D11.7	RP5-821D11.7.value <= threshold=3.2165164947509766
node_1659: feature_name=MARCH1	MARCH1.value <= threshold=4.2638514041900635
node_1660: feature_name=ABCA1	ABCA1.value <= threshold=3.618377923965454
node_1661: feature_name=LTB	LTB.value <= threshold=0.8862588405609131
node_1662: feature_name=SNORD3B-2	SNORD3B-2.value <= threshold=4.273592233657837
node_1663: feature_name=S100A6	S100A6.value <= threshold=0.29081472009420395
node_1664: feature_name=IGLL5	IGLL5.value <= threshold=6.102234125137329
node_1665: feature_name=STAG3	STAG3.value > threshold=6.671069860458374
node_1671: feature_name=GRASP	GRASP.value <= threshold=1.8713648319244385
Class: COVID-19 Critical	

Rules_410	passed counts:11
node_0: feature_name=LTB	LTB.value <= threshold=0.994031548500061
node_1: feature_name=MTRNR2L2	MTRNR2L2.value <= threshold=2.8784297704696655
node_2: feature_name=TCL1A	TCL1A.value <= threshold=4.798312664031982
node_3: feature_name=MT-CO1	MT-CO1.value <= threshold=0.08947191759943962
node_4: feature_name=NR4A1	NR4A1.value <= threshold=1.0725348591804504
node_5: feature_name=HLA-DPA1	HLA-DPA1.value <= threshold=1.6885469555854797
node_6: feature_name=HLA-DRB5	HLA-DRB5.value <= threshold=2.1886194944381714
node_7: feature_name=XIST	XIST.value <= threshold=-0.12328458577394485
node_8: feature_name=SYNE1	SYNE1.value > threshold=-0.7835594415664673
node_16: feature_name=MTRNR2L2	MTRNR2L2.value <= threshold=2.4483247995376587
node_17: feature_name=HLA-DQA2	HLA-DQA2.value <= threshold=2.227178931236267
node_18: feature_name=NR4A1	NR4A1.value <= threshold=-0.21380723267793655
node_19: feature_name=IRAK3	IRAK3.value <= threshold=4.27159571647644
node_20: feature_name=IFIT2	IFIT2.value <= threshold=7.8175740242004395
node_21: feature_name=CD79A	CD79A.value <= threshold=2.857242226600647
node_22: feature_name=HIST1H2AC	HIST1H2AC.value <= threshold=5.077470302581787
node_23: feature_name=FTH1	FTH1.value > threshold=0.4158056080341339
node_285: feature_name=EGR1	EGR1.value <= threshold=4.240467071533203
node_286: feature_name=HLA-DRB1	HLA-DRB1.value > threshold=-0.7553382813930511
node_290: feature_name=TLR2	TLR2.value <= threshold=2.879140257835388
node_291: feature_name=SYNE1	SYNE1.value <= threshold=2.323199510574341
node_292: feature_name=BCL2A1	BCL2A1.value <= threshold=1.8940600156784058
node_293: feature_name=RALGPS2	RALGPS2.value <= threshold=4.808189153671265
node_294: feature_name=IGLL5	IGLL5.value <= threshold=4.116017818450928
node_295: feature_name=FTH1	FTH1.value > threshold=0.41612209379673004
node_297: feature_name=MCTP1	MCTP1.value <= threshold=4.601342678070068
node_298: feature_name=MT-CO2	MT-CO2.value <= threshold=0.6201252341270447
node_299: feature_name=MARCKS	MARCKS.value > threshold=3.471073269844055

node_337: feature_name=HLA-DRB1	HLA-DRB1.value > threshold=0.9078778922557831
node_339: feature_name=RHOB	RHOB.value <= threshold=2.8503750562667847
Class: COVID-19 Critical	
Rules_411	passed counts:11
node_0: feature_name=LTB	LTB.value <= threshold=0.994031548500061
node_1: feature_name=MTRNR2L2	MTRNR2L2.value <= threshold=2.8784297704696655
node_2: feature_name=TCL1A	TCL1A.value <= threshold=4.798312664031982
node_3: feature_name=MT-CO1	MT-CO1.value <= threshold=0.08947191759943962
node_4: feature_name=NR4A1	NR4A1.value <= threshold=1.0725348591804504
node_5: feature_name=HLA-DPA1	HLA-DPA1.value <= threshold=1.6885469555854797
node_6: feature_name=HLA-DRB5	HLA-DRB5.value <= threshold=2.1886194944381714
node_7: feature_name=XIST	XIST.value <= threshold=-0.12328458577394485
node_8: feature_name=SYNE1	SYNE1.value > threshold=-0.7835594415664673
node_16: feature_name=MTRNR2L2	MTRNR2L2.value <= threshold=2.4483247995376587
node_17: feature_name=HLA-DQA2	HLA-DQA2.value <= threshold=2.227178931236267
node_18: feature_name=NR4A1	NR4A1.value <= threshold=-0.21380723267793655
node_19: feature_name=IRAK3	IRAK3.value <= threshold=4.27159571647644
node_20: feature_name=IFIT2	IFIT2.value <= threshold=7.8175740242004395
node_21: feature_name=CD79A	CD79A.value <= threshold=2.857242226600647
node_22: feature_name=HIST1H2AC	HIST1H2AC.value <= threshold=5.077470302581787
node_23: feature_name=FTH1	FTH1.value > threshold=0.4158056080341339
node_285: feature_name=EGR1	EGR1.value <= threshold=4.240467071533203
node_286: feature_name=HLA-DRB1	HLA-DRB1.value > threshold=-0.7553382813930511
node_290: feature_name=TLR2	TLR2.value <= threshold=2.879140257835388
node_291: feature_name=SYNE1	SYNE1.value <= threshold=2.323199510574341
node_292: feature_name=BCL2A1	BCL2A1.value <= threshold=1.8940600156784058
node_293: feature_name=RALGPS2	RALGPS2.value <= threshold=4.808189153671265
node_294: feature_name=IGLL5	IGLL5.value <= threshold=4.116017818450928
node_295: feature_name=FTH1	FTH1.value > threshold=0.41612209379673004

node_297: feature_name=MCTP1	MCTP1.value <= threshold=4.601342678070068
node_298: feature_name=MT-CO2	MT-CO2.value <= threshold=0.6201252341270447
node_299: feature_name=MARCKS	MARCKS.value <= threshold=3.471073269844055
node_300: feature_name=PPIF	PPIF.value <= threshold=2.8470659255981445
node_301: feature_name=CCL3	CCL3.value <= threshold=1.9901156425476074
node_302: feature_name=MT-CO2	MT-CO2.value > threshold=-2.867768883705139
node_306: feature_name=RHOB	RHOB.value <= threshold=3.9839000701904297
node_307: feature_name=SNX29P2	SNX29P2.value <= threshold=5.035826921463013
node_308: feature_name=MT-CO3	MT-CO3.value <= threshold=0.7808255851268768
node_309: feature_name=RGCC	RGCC.value <= threshold=1.070733904838562
node_310: feature_name=SESN3	SESN3.value > threshold=2.5199365615844727
node_316: feature_name=CCDC141	CCDC141.value <= threshold=1.964890070259571
Class: COVID-19 Critical	
Rules_412	passed counts:11
node_0: feature_name=LTB	LTB.value <= threshold=0.994031548500061
node_1: feature_name=MTRNR2L2	MTRNR2L2.value <= threshold=2.8784297704696655
node_2: feature_name=TCL1A	TCL1A.value <= threshold=4.798312664031982
node_3: feature_name=MT-CO1	MT-CO1.value <= threshold=0.08947191759943962
node_4: feature_name=NR4A1	NR4A1.value <= threshold=1.0725348591804504
node_5: feature_name=HLA-DPA1	HLA-DPA1.value <= threshold=1.6885469555854797
node_6: feature_name=HLA-DRB5	HLA-DRB5.value <= threshold=2.1886194944381714
node_7: feature_name=XIST	XIST.value <= threshold=-0.12328458577394485
node_8: feature_name=SYNE1	SYNE1.value > threshold=-0.7835594415664673
node_16: feature_name=MTRNR2L2	MTRNR2L2.value <= threshold=2.4483247995376587
node_17: feature_name=HLA-DQA2	HLA-DQA2.value <= threshold=2.227178931236267
node_18: feature_name=NR4A1	NR4A1.value <= threshold=-0.21380723267793655
node_19: feature_name=IRAK3	IRAK3.value <= threshold=4.27159571647644
node_20: feature_name=IFIT2	IFIT2.value <= threshold=7.8175740242004395
node_21: feature_name=CD79A	CD79A.value <= threshold=2.857242226600647

node_22: feature_name=HIST1H2AC	HIST1H2AC.value <= threshold=5.077470302581787
node_23: feature_name=FTH1	FTH1.value <= threshold=0.4158056080341339
node_24: feature_name=ATF3	ATF3.value <= threshold=3.8028353452682495
node_25: feature_name=OASL	OASL.value <= threshold=3.387580633163452
node_26: feature_name=MT-CO1	MT-CO1.value > threshold=-0.7942646145820618
node_74: feature_name=HOPX	HOPX.value <= threshold=0.8682331740856171
node_75: feature_name=RGS2	RGS2.value <= threshold=1.4589954018592834
node_76: feature_name=AHR	AHR.value <= threshold=3.870481848716736
node_77: feature_name=IGJ	IGJ.value <= threshold=4.902882814407349
node_78: feature_name=CD79B	CD79B.value <= threshold=4.87716269493103
node_79: feature_name=HLA-DQB1	HLA-DQB1.value <= threshold=3.6925491094589233
node_80: feature_name=HLA-DRB5	HLA-DRB5.value <= threshold=2.0926843881607056
node_81: feature_name=MCTP1	MCTP1.value <= threshold=4.010472774505615
node_82: feature_name=GNG11	GNG11.value <= threshold=3.627470850944519
node_83: feature_name=HSP90B1	HSP90B1.value <= threshold=2.0835131406784058
node_84: feature_name=ATF3	ATF3.value <= threshold=1.1142374873161316
node_85: feature_name=MT-ND6	MT-ND6.value <= threshold=2.7328014373779297
node_86: feature_name=COTL1	COTL1.value > threshold=-1.6524587869644165
node_90: feature_name=MT-CYB	MT-CYB.value <= threshold=1.099629819393158
node_91: feature_name=RGCC	RGCC.value <= threshold=1.6908138990402222
node_92: feature_name=ILK	ILK.value <= threshold=3.9497616291046143
node_93: feature_name=CTD-2035E11.3	CTD-2035E11.3.value <= threshold=2.4543306827545166
node_94: feature_name=S100A6	S100A6.value <= threshold=0.2867250442504883
node_95: feature_name=HLA-DRB5	HLA-DRB5.value > threshold=-0.9496813416481018
node_99: feature_name=MT-CO3	MT-CO3.value > threshold=-1.55654776096344
node_103: feature_name=ITGAX	ITGAX.value > threshold=1.8544010519981384
node_149: feature_name=PPIF	PPIF.value <= threshold=1.4860219359397888
node_150: feature_name=S100A6	S100A6.value > threshold=-1.7994658946990967
Class: COVID-19 Critical	

Rules_413	passed counts:10
node_0: feature_name=LTB	LTB.value > threshold=0.994031548500061
node_5264: feature_name=MT-CO1	MT-CO1.value > threshold=-0.4127991199493408
node_5638: feature_name=OAZ1	OAZ1.value > threshold=-0.2640867084264755
node_6112: feature_name=IFI44L	IFI44L.value <= threshold=2.5893263816833496
node_6113: feature_name=TCL1A	TCL1A.value <= threshold=4.201832294464111
node_6114: feature_name=HLA-DQA2	HLA-DQA2.value > threshold=-0.2270243540406227
node_6542: feature_name=CD79B	CD79B.value > threshold=3.096232771873474
node_6872: feature_name=ICAM1	ICAM1.value <= threshold=-0.2232399433851242
node_6873: feature_name=STAG3	STAG3.value <= threshold=-0.19312061369419098
node_6874: feature_name=CD79A	CD79A.value > threshold=1.57558012008667
node_6880: feature_name=MEF2A	MEF2A.value > threshold=2.676826596260071
node_6932: feature_name=FAM129C	FAM129C.value > threshold=2.9109067916870117
node_6934: feature_name=YWHAH	YWHAH.value > threshold=-0.2668233811855316
node_6936: feature_name=SNX9	SNX9.value <= threshold=3.379360556602478
Class: Healthy Control	
Rules_414	passed counts:10
node_0: feature_name=LTB	LTB.value > threshold=0.994031548500061
node_5264: feature_name=MT-CO1	MT-CO1.value > threshold=-0.4127991199493408
node_5638: feature_name=OAZ1	OAZ1.value > threshold=-0.2640867084264755
node_6112: feature_name=IFI44L	IFI44L.value <= threshold=2.5893263816833496
node_6113: feature_name=TCL1A	TCL1A.value <= threshold=4.201832294464111
node_6114: feature_name=HLA-DQA2	HLA-DQA2.value > threshold=-0.2270243540406227
node_6542: feature_name=CD79B	CD79B.value <= threshold=3.096232771873474
node_6543: feature_name=NR4A1	NR4A1.value <= threshold=1.421015739440918
node_6544: feature_name=LTB	LTB.value > threshold=1.359755277633667
node_6720: feature_name=NFKBIZ	NFKBIZ.value <= threshold=0.2720414251089096
node_6721: feature_name=ATF5	ATF5.value <= threshold=2.4672781229019165
node_6722: feature_name=SNX9	SNX9.value <= threshold=2.0814030170440674

node_6723: feature_name=HLA-DQA2	HLA-DQA2.value > threshold=4.360614776611328
node_6775: feature_name=RP11-731F5.2	RP11-731F5.2.value > threshold=1.6779262274503708
Class: COVID-19 Critical	
Rules_415	passed counts:10
node_0: feature_name=LTB	LTB.value > threshold=0.994031548500061
node_5264: feature_name=MT-CO1	MT-CO1.value > threshold=-0.4127991199493408
node_5638: feature_name=OAZ1	OAZ1.value > threshold=-0.2640867084264755
node_6112: feature_name=IFI44L	IFI44L.value <= threshold=2.5893263816833496
node_6113: feature_name=TCL1A	TCL1A.value <= threshold=4.201832294464111
node_6114: feature_name=HLA-DQA2	HLA-DQA2.value > threshold=-0.2270243540406227
node_6542: feature_name=CD79B	CD79B.value <= threshold=3.096232771873474
node_6543: feature_name=NR4A1	NR4A1.value <= threshold=1.421015739440918
node_6544: feature_name=LTB	LTB.value > threshold=1.359755277633667
node_6720: feature_name=NFKBIZ	NFKBIZ.value <= threshold=0.2720414251089096
node_6721: feature_name=ATF5	ATF5.value <= threshold=2.4672781229019165
node_6722: feature_name=SNX9	SNX9.value <= threshold=2.0814030170440674
node_6723: feature_name=HLA-DQA2	HLA-DQA2.value > threshold=4.360614776611328
node_6775: feature_name=RP11-731F5.2	RP11-731F5.2.value <= threshold=1.6779262274503708
node_6776: feature_name=TTN	TTN.value <= threshold=-0.2564083933830261
Class: Healthy Control	
Rules_416	passed counts:10
node_0: feature_name=LTB	LTB.value > threshold=0.994031548500061
node_5264: feature_name=MT-CO1	MT-CO1.value > threshold=-0.4127991199493408
node_5638: feature_name=OAZ1	OAZ1.value > threshold=-0.2640867084264755
node_6112: feature_name=IFI44L	IFI44L.value <= threshold=2.5893263816833496
node_6113: feature_name=TCL1A	TCL1A.value <= threshold=4.201832294464111
node_6114: feature_name=HLA-DQA2	HLA-DQA2.value <= threshold=-0.2270243540406227
node_6115: feature_name=MTRNR2L2	MTRNR2L2.value <= threshold=2.548917293548584

node_6116: feature_name=ATF5	ATF5.value <= threshold=2.041263461112976
node_6117: feature_name=JUN	JUN.value > threshold=-0.616259902715683
node_6189: feature_name=MT-CO3	MT-CO3.value > threshold=0.13483521342277527
node_6351: feature_name=XAF1	XAF1.value <= threshold=3.061717987060547
node_6352: feature_name=MT-CO2	MT-CO2.value <= threshold=1.2605181336402893
node_6353: feature_name=ICAM1	ICAM1.value <= threshold=-0.3340446650981903
node_6354: feature_name=OAZ1	OAZ1.value > threshold=-0.24055946618318558
node_6360: feature_name=PPIF	PPIF.value <= threshold=2.070030093193054
node_6361: feature_name=RALGPS2	RALGPS2.value > threshold=-0.3684315085411072
node_6369: feature_name=ANO6	ANO6.value <= threshold=3.504153251647949
node_6370: feature_name=YWHAH	YWHAH.value <= threshold=4.3182291984558105
node_6371: feature_name=LDLR	LDLR.value <= threshold=2.494291305541992
node_6372: feature_name=HLA-DQA1	HLA-DQA1.value > threshold=-0.5716990232467651
node_6374: feature_name=ABCA1	ABCA1.value <= threshold=3.7662240266799927
node_6375: feature_name=MS4A1	MS4A1.value > threshold=1.4995208978652954
node_6387: feature_name=SNORD3B-2	SNORD3B-2.value <= threshold=4.154061555862427
node_6388: feature_name=LTB	LTB.value <= threshold=2.1739100217819214
node_6389: feature_name=BACH1	BACH1.value <= threshold=2.0371285676956177
node_6390: feature_name=HLA-DQB1	HLA-DQB1.value <= threshold=3.0258322954177856
node_6391: feature_name=HLA-DPB1	HLA-DPB1.value > threshold=0.2591289207339287
node_6395: feature_name=CXorf57	CXorf57.value <= threshold=3.3742183446884155
node_6396: feature_name=MMD	MMD.value <= threshold=3.8883349895477295
node_6397: feature_name=IRF8	IRF8.value <= threshold=3.939465045928955
node_6398: feature_name=PPIF	PPIF.value <= threshold=1.1284508109092712
node_6399: feature_name=S100A6	S100A6.value > threshold=-2.1265565156936646
node_6403: feature_name=MEF2A	MEF2A.value > threshold=2.771904706954956
node_6405: feature_name=PARP14	PARP14.value > threshold=-0.5069229006767273
Class: Healthy Control	
Rules_417	passed counts:10

node_0: feature_name=LTB	LTB.value > threshold=0.994031548500061
node_5264: feature_name=MT-CO1	MT-CO1.value > threshold=-0.4127991199493408
node_5638: feature_name=OAZ1	OAZ1.value > threshold=-0.2640867084264755
node_6112: feature_name=IFI44L	IFI44L.value <= threshold=2.5893263816833496
node_6113: feature_name=TCL1A	TCL1A.value <= threshold=4.201832294464111
node_6114: feature_name=HLA-DQA2	HLA-DQA2.value <= threshold=-0.2270243540406227
node_6115: feature_name=MTRNR2L2	MTRNR2L2.value <= threshold=2.548917293548584
node_6116: feature_name=ATF5	ATF5.value <= threshold=2.041263461112976
node_6117: feature_name=JUN	JUN.value > threshold=-0.616259902715683
node_6189: feature_name=MT-CO3	MT-CO3.value <= threshold=0.13483521342277527
node_6190: feature_name=XAF1	XAF1.value <= threshold=2.584952235221863
node_6191: feature_name=HLA-DRB5	HLA-DRB5.value > threshold=0.6955901980400085
node_6239: feature_name=TNFAIP3	TNFAIP3.value <= threshold=0.11930759996175766
node_6240: feature_name=TP53INP1	TP53INP1.value > threshold=2.9654784202575684
node_6330: feature_name=LTB	LTB.value > threshold=1.14329594373703
node_6334: feature_name=BANK1	BANK1.value <= threshold=3.5240423679351807
node_6335: feature_name=NR4A3	NR4A3.value <= threshold=1.4398073554039001
node_6336: feature_name=JARID2	JARID2.value <= threshold=1.196907877922058
Class: Healthy Control	
Rules_418	passed counts:10
node_0: feature_name=LTB	LTB.value > threshold=0.994031548500061
node_5264: feature_name=MT-CO1	MT-CO1.value > threshold=-0.4127991199493408
node_5638: feature_name=OAZ1	OAZ1.value > threshold=-0.2640867084264755
node_6112: feature_name=IFI44L	IFI44L.value <= threshold=2.5893263816833496
node_6113: feature_name=TCL1A	TCL1A.value <= threshold=4.201832294464111
node_6114: feature_name=HLA-DQA2	HLA-DQA2.value <= threshold=-0.2270243540406227
node_6115: feature_name=MTRNR2L2	MTRNR2L2.value <= threshold=2.548917293548584
node_6116: feature_name=ATF5	ATF5.value <= threshold=2.041263461112976
node_6117: feature_name=JUN	JUN.value <= threshold=-0.616259902715683

node_6118: feature_name=NFKBID	NFKBID.value <= threshold=1.5264258980751038
node_6119: feature_name=MT-CO1	MT-CO1.value > threshold=0.1564532369375229
node_6133: feature_name=LTB	LTB.value <= threshold=1.2431999444961548
node_6134: feature_name=MT-ND6	MT-ND6.value <= threshold=0.5990295112133026
node_6135: feature_name=MEF2A	MEF2A.value > threshold=1.0292169749736786
Class: COVID-19 Critical	
Rules_419	passed counts:10
node_0: feature_name=LTB	LTB.value > threshold=0.994031548500061
node_5264: feature_name=MT-CO1	MT-CO1.value > threshold=-0.4127991199493408
node_5638: feature_name=OAZ1	OAZ1.value <= threshold=-0.2640867084264755
node_5639: feature_name=CD79B	CD79B.value > threshold=2.2998517751693726
node_5925: feature_name=SNX9	SNX9.value <= threshold=2.4445992708206177
node_5926: feature_name=TCL1A	TCL1A.value <= threshold=2.6938670873641968
node_5927: feature_name=PLD4	PLD4.value <= threshold=-0.06434785947203636
node_5928: feature_name=ICAM1	ICAM1.value <= threshold=-0.2820792943239212
node_5929: feature_name=MS4A1	MS4A1.value > threshold=2.747320055961609
node_5963: feature_name=RNF144B	RNF144B.value > threshold=-0.2324080914258957
node_5989: feature_name=YWHAH	YWHAH.value <= threshold=-0.27859707176685333
node_5990: feature_name=CD83	CD83.value <= threshold=2.019383668899536
Class: COVID-19 Critical	
Rules_420	passed counts:10
node_0: feature_name=LTB	LTB.value > threshold=0.994031548500061
node_5264: feature_name=MT-CO1	MT-CO1.value > threshold=-0.4127991199493408
node_5638: feature_name=OAZ1	OAZ1.value <= threshold=-0.2640867084264755
node_5639: feature_name=CD79B	CD79B.value <= threshold=2.2998517751693726
node_5640: feature_name=MTRNR2L2	MTRNR2L2.value <= threshold=3.5499863624572754
node_5641: feature_name=MT-CO1	MT-CO1.value > threshold=0.22567133605480194
node_5729: feature_name=MARCH1	MARCH1.value > threshold=1.5154643654823303

node_5867: feature_name=NFKBID	NFKBID.value > threshold=2.6947624683380127
node_5903: feature_name=RGCC	RGCC.value > threshold=-0.730547159910202
node_5909: feature_name=SPI1	SPI1.value > threshold=-0.2540571391582489
node_5915: feature_name=MEF2C	MEF2C.value <= threshold=3.5967005491256714
node_5916: feature_name=SNX29P2	SNX29P2.value <= threshold=5.403664827346802
Class: COVID-19 Critical	
Rules_421	passed counts:10
node_0: feature_name=LTB	LTB.value > threshold=0.994031548500061
node_5264: feature_name=MT-CO1	MT-CO1.value > threshold=-0.4127991199493408
node_5638: feature_name=OAZ1	OAZ1.value <= threshold=-0.2640867084264755
node_5639: feature_name=CD79B	CD79B.value <= threshold=2.2998517751693726
node_5640: feature_name=MTRNR2L2	MTRNR2L2.value <= threshold=3.5499863624572754
node_5641: feature_name=MT-CO1	MT-CO1.value <= threshold=0.22567133605480194
node_5642: feature_name=RGS2	RGS2.value > threshold=1.2649686336517334
node_5710: feature_name=HLA-DPB1	HLA-DPB1.value <= threshold=1.09694242477417
Class: COVID-19 Critical	
Rules_422	passed counts:10
node_0: feature_name=LTB	LTB.value > threshold=0.994031548500061
node_5264: feature_name=MT-CO1	MT-CO1.value > threshold=-0.4127991199493408
node_5638: feature_name=OAZ1	OAZ1.value <= threshold=-0.2640867084264755
node_5639: feature_name=CD79B	CD79B.value <= threshold=2.2998517751693726
node_5640: feature_name=MTRNR2L2	MTRNR2L2.value <= threshold=3.5499863624572754
node_5641: feature_name=MT-CO1	MT-CO1.value <= threshold=0.22567133605480194
node_5642: feature_name=RGS2	RGS2.value <= threshold=1.2649686336517334
node_5643: feature_name=TCL1A	TCL1A.value <= threshold=4.771023273468018
node_5644: feature_name=IGJ	IGJ.value <= threshold=3.91206431388855
node_5645: feature_name=AREGB	AREGB.value <= threshold=1.5142876282334328
node_5646: feature_name=HLA-DRB1	HLA-DRB1.value > threshold=-0.6253005266189575

node_5648: feature_name=ITM2C	ITM2C.value <= threshold=3.2904402017593384
node_5649: feature_name=PLA2G12A	PLA2G12A.value <= threshold=3.833786725997925
node_5650: feature_name=RGCC	RGCC.value <= threshold=-0.10016544535756111
node_5651: feature_name=CD79B	CD79B.value <= threshold=2.278481364250183
node_5652: feature_name=HIST1H2AC	HIST1H2AC.value <= threshold=3.4233893156051636
node_5653: feature_name=RHOB	RHOB.value <= threshold=4.956467151641846
node_5654: feature_name=CD83	CD83.value > threshold=2.970836043357849
node_5670: feature_name=HLA-DRB1	HLA-DRB1.value > threshold=1.513556182384491
Class: COVID-19 Critical	
Rules_423	passed counts:10
node_0: feature_name=LTB	LTB.value > threshold=0.994031548500061
node_5264: feature_name=MT-CO1	MT-CO1.value <= threshold=-0.4127991199493408
node_5265: feature_name=MTRNR2L2	MTRNR2L2.value <= threshold=2.4473838806152344
node_5266: feature_name=TCL1A	TCL1A.value <= threshold=5.787256956100464
node_5267: feature_name=PLD4	PLD4.value <= threshold=3.544657826423645
node_5268: feature_name=NR4A1	NR4A1.value > threshold=0.8490704298019409
node_5498: feature_name=HLA-DPA1	HLA-DPA1.value <= threshold=1.9079211950302124
node_5499: feature_name=ID3	ID3.value > threshold=-0.17505786567926407
node_5543: feature_name=BCL2A1	BCL2A1.value <= threshold=-0.4924587309360504
node_5544: feature_name=TNFAIP3	TNFAIP3.value <= threshold=-0.4164377674460411
Class: Healthy Control	
Rules_424	passed counts:10
node_0: feature_name=LTB	LTB.value > threshold=0.994031548500061
node_5264: feature_name=MT-CO1	MT-CO1.value <= threshold=-0.4127991199493408
node_5265: feature_name=MTRNR2L2	MTRNR2L2.value <= threshold=2.4473838806152344
node_5266: feature_name=TCL1A	TCL1A.value <= threshold=5.787256956100464
node_5267: feature_name=PLD4	PLD4.value <= threshold=3.544657826423645
node_5268: feature_name=NR4A1	NR4A1.value <= threshold=0.8490704298019409

node_5269: feature_name=LTB	LTB.value > threshold=1.5252206921577454
node_5471: feature_name=CD79A	CD79A.value > threshold=2.510846734046936
node_5483: feature_name=XAF1	XAF1.value > threshold=1.7000007629394531
Class: COVID-19 Critical	
Rules_425	passed counts:10
node_0: feature_name=LTB	LTB.value > threshold=0.994031548500061
node_5264: feature_name=MT-CO1	MT-CO1.value <= threshold=-0.4127991199493408
node_5265: feature_name=MTRNR2L2	MTRNR2L2.value <= threshold=2.4473838806152344
node_5266: feature_name=TCL1A	TCL1A.value <= threshold=5.787256956100464
node_5267: feature_name=PLD4	PLD4.value <= threshold=3.544657826423645
node_5268: feature_name=NR4A1	NR4A1.value <= threshold=0.8490704298019409
node_5269: feature_name=LTB	LTB.value <= threshold=1.5252206921577454
node_5270: feature_name=HLA-DRB5	HLA-DRB5.value > threshold=2.5117164850234985
node_5448: feature_name=HLA-DQB1	HLA-DQB1.value > threshold=2.092399835586548
node_5456: feature_name=SNX9	SNX9.value > threshold=2.15211021900177
node_5464: feature_name=AHR	AHR.value <= threshold=-0.3730653375387192
node_5465: feature_name=NT5C3A	NT5C3A.value <= threshold=0.8068774491548538
Class: COVID-19 Critical	
Rules_426	passed counts:10
node_0: feature_name=LTB	LTB.value <= threshold=0.994031548500061
node_1: feature_name=MTRNR2L2	MTRNR2L2.value > threshold=2.8784297704696655
node_5207: feature_name=MTRNR2L8	MTRNR2L8.value > threshold=3.1915172338485718
node_5257: feature_name=SNX29P2	SNX29P2.value <= threshold=-0.2883431911468506
node_5258: feature_name=MARCH1	MARCH1.value > threshold=-0.2905067503452301
Class: COVID-19 Critical	
Rules_427	passed counts:10
node_0: feature_name=LTB	LTB.value <= threshold=0.994031548500061

node_1: feature_name=MTRNR2L2	MTRNR2L2.value <= threshold=2.8784297704696655
node_2: feature_name=TCL1A	TCL1A.value > threshold=4.798312664031982
node_4912: feature_name=MT-CO1	MT-CO1.value > threshold=-0.3664844036102295
node_5002: feature_name=IFI44L	IFI44L.value <= threshold=2.3101747035980225
node_5003: feature_name=SNX9	SNX9.value > threshold=2.0247421264648438
node_5147: feature_name=CD79A	CD79A.value > threshold=2.554050326347351
node_5169: feature_name=CD79B	CD79B.value > threshold=-0.2480710819363594
node_5185: feature_name=OAZ1	OAZ1.value <= threshold=0.2523931488394737
node_5186: feature_name=PDLIM1	PDLIM1.value <= threshold=2.7835850715637207
node_5187: feature_name=CTD-2035E11.3	CTD-2035E11.3.value > threshold=-0.26431451737880707
node_5189: feature_name=TCL1A	TCL1A.value <= threshold=6.438810348510742
Class: COVID-19 Critical	
Rules_428	passed counts:10
node_0: feature_name=LTB	LTB.value <= threshold=0.994031548500061
node_1: feature_name=MTRNR2L2	MTRNR2L2.value <= threshold=2.8784297704696655
node_2: feature_name=TCL1A	TCL1A.value > threshold=4.798312664031982
node_4912: feature_name=MT-CO1	MT-CO1.value > threshold=-0.3664844036102295
node_5002: feature_name=IFI44L	IFI44L.value <= threshold=2.3101747035980225
node_5003: feature_name=SNX9	SNX9.value > threshold=2.0247421264648438
node_5147: feature_name=CD79A	CD79A.value > threshold=2.554050326347351
node_5169: feature_name=CD79B	CD79B.value <= threshold=-0.2480710819363594
node_5170: feature_name=HLA-DQB1	HLA-DQB1.value > threshold=2.405891537666321
node_5180: feature_name=HLA-DQA2	HLA-DQA2.value <= threshold=-0.12562735378742218
Class: Healthy Control	
Rules_429	passed counts:10
node_0: feature_name=LTB	LTB.value <= threshold=0.994031548500061
node_1: feature_name=MTRNR2L2	MTRNR2L2.value <= threshold=2.8784297704696655
node_2: feature_name=TCL1A	TCL1A.value <= threshold=4.798312664031982

node_3: feature_name=MT-CO1	MT-CO1.value > threshold=0.08947191759943962
node_2521: feature_name=LTB	LTB.value > threshold=0.6038667261600494
node_4173: feature_name=OAZ1	OAZ1.value > threshold=0.143283873796463
node_4615: feature_name=NR4A1	NR4A1.value <= threshold=0.6621004343032837
node_4616: feature_name=NFKBID	NFKBID.value <= threshold=3.283741593360901
node_4617: feature_name=HLA-DQA1	HLA-DQA1.value > threshold=2.3435752391815186
node_4719: feature_name=MX1	MX1.value <= threshold=1.851993978023529
node_4720: feature_name=JUN	JUN.value > threshold=-0.5025404393672943
node_4740: feature_name=IRF8	IRF8.value > threshold=3.838419556617737
node_4790: feature_name=MT-ND6	MT-ND6.value > threshold=-1.0028773248195648
Class: COVID-19 Critical	
Rules_430	passed counts:10
node_0: feature_name=LTB	LTB.value <= threshold=0.994031548500061
node_1: feature_name=MTRNR2L2	MTRNR2L2.value <= threshold=2.8784297704696655
node_2: feature_name=TCL1A	TCL1A.value <= threshold=4.798312664031982
node_3: feature_name=MT-CO1	MT-CO1.value > threshold=0.08947191759943962
node_2521: feature_name=LTB	LTB.value > threshold=0.6038667261600494
node_4173: feature_name=OAZ1	OAZ1.value > threshold=0.143283873796463
node_4615: feature_name=NR4A1	NR4A1.value <= threshold=0.6621004343032837
node_4616: feature_name=NFKBID	NFKBID.value <= threshold=3.283741593360901
node_4617: feature_name=HLA-DQA1	HLA-DQA1.value <= threshold=2.3435752391815186
node_4618: feature_name=PELI1	PELI1.value <= threshold=1.3271203637123108
node_4619: feature_name=PLD4	PLD4.value <= threshold=3.3385335206985474
node_4620: feature_name=MEF2A	MEF2A.value <= threshold=1.5926940441131592
node_4621: feature_name=DAPP1	DAPP1.value <= threshold=2.647637367248535
node_4622: feature_name=STAG3	STAG3.value <= threshold=-0.16521219909191132
node_4623: feature_name=OAZ1	OAZ1.value > threshold=0.2042311355471611
node_4625: feature_name=FAM129C	FAM129C.value <= threshold=-0.23615043610334396
node_4626: feature_name=TCL1A	TCL1A.value <= threshold=-0.18263818323612213

node_4627: feature_name=RALGPS2	RALGPS2.value > threshold=0.4531548172235489
node_4633: feature_name=SNX9	SNX9.value <= threshold=1.6314868927001953
Class: Healthy Control	
Rules_431	passed counts:10
node_0: feature_name=LTB	LTB.value <= threshold=0.994031548500061
node_1: feature_name=MTRNR2L2	MTRNR2L2.value <= threshold=2.8784297704696655
node_2: feature_name=TCL1A	TCL1A.value <= threshold=4.798312664031982
node_3: feature_name=MT-CO1	MT-CO1.value > threshold=0.08947191759943962
node_2521: feature_name=LTB	LTB.value > threshold=0.6038667261600494
node_4173: feature_name=OAZ1	OAZ1.value <= threshold=0.143283873796463
node_4174: feature_name=PLD4	PLD4.value > threshold=2.921932101249695
node_4558: feature_name=HLA-DQA2	HLA-DQA2.value <= threshold=1.5485953092575073
node_4559: feature_name=COTL1	COTL1.value <= threshold=-0.42844848334789276
node_4560: feature_name=VPREB3	VPREB3.value > threshold=0.7973381504416466
node_4570: feature_name=NAMPT	NAMPT.value <= threshold=0.7296004593372345
Class: Healthy Control	
Rules_432	passed counts:10
node_0: feature_name=LTB	LTB.value <= threshold=0.994031548500061
node_1: feature_name=MTRNR2L2	MTRNR2L2.value <= threshold=2.8784297704696655
node_2: feature_name=TCL1A	TCL1A.value <= threshold=4.798312664031982
node_3: feature_name=MT-CO1	MT-CO1.value > threshold=0.08947191759943962
node_2521: feature_name=LTB	LTB.value > threshold=0.6038667261600494
node_4173: feature_name=OAZ1	OAZ1.value <= threshold=0.143283873796463
node_4174: feature_name=PLD4	PLD4.value <= threshold=2.921932101249695
node_4175: feature_name=XIST	XIST.value <= threshold=-0.05067425221204758
node_4176: feature_name=MT-CO1	MT-CO1.value <= threshold=1.345594048500061
node_4177: feature_name=HLA-DQB1	HLA-DQB1.value > threshold=1.927736520767212
node_4353: feature_name=ITM2C	ITM2C.value > threshold=-0.12329206243157387

node_4459: feature_name=SNX9	SNX9.value > threshold=0.6323382556438446
node_4469: feature_name=PELI1	PELI1.value <= threshold=0.4953474849462509
node_4470: feature_name=HSP90B1	HSP90B1.value <= threshold=0.24037402868270874
node_4471: feature_name=BANK1	BANK1.value > threshold=1.3680726885795593
Class: Healthy Control	
Rules_433	passed counts:10
node_0: feature_name=LTB	LTB.value <= threshold=0.994031548500061
node_1: feature_name=MTRNR2L2	MTRNR2L2.value <= threshold=2.8784297704696655
node_2: feature_name=TCL1A	TCL1A.value <= threshold=4.798312664031982
node_3: feature_name=MT-CO1	MT-CO1.value > threshold=0.08947191759943962
node_2521: feature_name=LTB	LTB.value > threshold=0.6038667261600494
node_4173: feature_name=OAZ1	OAZ1.value <= threshold=0.143283873796463
node_4174: feature_name=PLD4	PLD4.value <= threshold=2.921932101249695
node_4175: feature_name=XIST	XIST.value <= threshold=-0.05067425221204758
node_4176: feature_name=MT-CO1	MT-CO1.value <= threshold=1.345594048500061
node_4177: feature_name=HLA-DQB1	HLA-DQB1.value > threshold=1.927736520767212
node_4353: feature_name=ITM2C	ITM2C.value <= threshold=-0.12329206243157387
node_4354: feature_name=MX1	MX1.value <= threshold=-0.4356398284435272
node_4355: feature_name=HLA-DQB1	HLA-DQB1.value <= threshold=2.8865538835525513
node_4356: feature_name=SNX9	SNX9.value <= threshold=1.002076417207718
node_4357: feature_name=FAM160B1	FAM160B1.value > threshold=-0.42920351028442383
node_4361: feature_name=SNX9	SNX9.value <= threshold=0.011789441108703613
node_4362: feature_name=MT-CO3	MT-CO3.value <= threshold=0.4059640020132065
node_4363: feature_name=RNF11	RNF11.value > threshold=-0.5651727318763733
node_4365: feature_name=IGJ	IGJ.value <= threshold=-0.3220001757144928
node_4366: feature_name=TSPYL2	TSPYL2.value <= threshold=0.8485699594020844
node_4367: feature_name=RBKS	RBKS.value > threshold=-0.3015036880970001
Class: COVID-19 Critical	

Rules_434	passed counts:10
node_0: feature_name=LTB	LTB.value <= threshold=0.994031548500061
node_1: feature_name=MTRNR2L2	MTRNR2L2.value <= threshold=2.8784297704696655
node_2: feature_name=TCL1A	TCL1A.value <= threshold=4.798312664031982
node_3: feature_name=MT-CO1	MT-CO1.value > threshold=0.08947191759943962
node_2521: feature_name=LTB	LTB.value > threshold=0.6038667261600494
node_4173: feature_name=OAZ1	OAZ1.value <= threshold=0.143283873796463
node_4174: feature_name=PLD4	PLD4.value <= threshold=2.921932101249695
node_4175: feature_name=XIST	XIST.value <= threshold=-0.05067425221204758
node_4176: feature_name=MT-CO1	MT-CO1.value <= threshold=1.345594048500061
node_4177: feature_name=HLA-DQB1	HLA-DQB1.value > threshold=1.927736520767212
node_4353: feature_name=ITM2C	ITM2C.value <= threshold=-0.12329206243157387
node_4354: feature_name=MX1	MX1.value <= threshold=-0.4356398284435272
node_4355: feature_name=HLA-DQB1	HLA-DQB1.value <= threshold=2.8865538835525513
node_4356: feature_name=SNX9	SNX9.value <= threshold=1.002076417207718
node_4357: feature_name=FAM160B1	FAM160B1.value <= threshold=-0.42920351028442383
node_4358: feature_name=PELI1	PELI1.value <= threshold=2.166169285774231
Class: Healthy Control	
Rules_435	passed counts:10
node_0: feature_name=LTB	LTB.value <= threshold=0.994031548500061
node_1: feature_name=MTRNR2L2	MTRNR2L2.value <= threshold=2.8784297704696655
node_2: feature_name=TCL1A	TCL1A.value <= threshold=4.798312664031982
node_3: feature_name=MT-CO1	MT-CO1.value > threshold=0.08947191759943962
node_2521: feature_name=LTB	LTB.value <= threshold=0.6038667261600494
node_2522: feature_name=HLA-DQB1	HLA-DQB1.value > threshold=2.1053054332733154
node_3550: feature_name=XIST	XIST.value <= threshold=0.7716337237507105
node_3551: feature_name=CD79B	CD79B.value > threshold=2.9426796436309814
node_4039: feature_name=IFI44L	IFI44L.value <= threshold=1.8637521862983704
node_4040: feature_name=SNX9	SNX9.value > threshold=1.274232804775238

node_4096: feature_name=RALGPS2	RALGPS2.value <= threshold=2.3599878549575806
node_4097: feature_name=HLA-DQB1	HLA-DQB1.value > threshold=2.9625227451324463
node_4111: feature_name=FTH1	FTH1.value > threshold=-0.30264076590538025
node_4115: feature_name=MT-CYB	MT-CYB.value <= threshold=1.042892873287201
Class: COVID-19 Severe	
Rules_436	passed counts:10
node_0: feature_name=LTB	LTB.value <= threshold=0.994031548500061
node_1: feature_name=MTRNR2L2	MTRNR2L2.value <= threshold=2.8784297704696655
node_2: feature_name=TCL1A	TCL1A.value <= threshold=4.798312664031982
node_3: feature_name=MT-CO1	MT-CO1.value > threshold=0.08947191759943962
node_2521: feature_name=LTB	LTB.value <= threshold=0.6038667261600494
node_2522: feature_name=HLA-DQB1	HLA-DQB1.value > threshold=2.1053054332733154
node_3550: feature_name=XIST	XIST.value <= threshold=0.7716337237507105
node_3551: feature_name=CD79B	CD79B.value > threshold=2.9426796436309814
node_4039: feature_name=IFI44L	IFI44L.value <= threshold=1.8637521862983704
node_4040: feature_name=SNX9	SNX9.value <= threshold=1.274232804775238
node_4041: feature_name=CASP8	CASP8.value <= threshold=-0.574097603559494
node_4042: feature_name=NFKBID	NFKBID.value > threshold=1.49756920337677
node_4056: feature_name=MIR24-2	MIR24-2.value > threshold=0.2797542065382004
node_4070: feature_name=FCRL1	FCRL1.value > threshold=-0.2390713170170784
Class: COVID-19 Critical	
Rules_437	passed counts:10
node_0: feature_name=LTB	LTB.value <= threshold=0.994031548500061
node_1: feature_name=MTRNR2L2	MTRNR2L2.value <= threshold=2.8784297704696655
node_2: feature_name=TCL1A	TCL1A.value <= threshold=4.798312664031982
node_3: feature_name=MT-CO1	MT-CO1.value > threshold=0.08947191759943962
node_2521: feature_name=LTB	LTB.value <= threshold=0.6038667261600494
node_2522: feature_name=HLA-DQB1	HLA-DQB1.value > threshold=2.1053054332733154

node_3550: feature_name=XIST	XIST.value <= threshold=0.7716337237507105
node_3551: feature_name=CD79B	CD79B.value <= threshold=2.9426796436309814
node_3552: feature_name=HLA-DQB1	HLA-DQB1.value <= threshold=2.713674306869507
node_3553: feature_name=SNX9	SNX9.value <= threshold=-0.5404067635536194
node_3554: feature_name=VPREB3	VPREB3.value <= threshold=4.4475414752960205
node_3555: feature_name=RALGPS2	RALGPS2.value > threshold=2.7017585039138794
node_3609: feature_name=MX1	MX1.value <= threshold=1.5204657316207886
node_3610: feature_name=FAM129C	FAM129C.value > threshold=-0.30572274327278137
node_3622: feature_name=CASP8	CASP8.value > threshold=-0.5367972254753113
node_3638: feature_name=SLC11A1	SLC11A1.value <= threshold=-0.3386744409799576
Class: COVID-19 Critical	
Rules_438	passed counts:10
node_0: feature_name=LTB	LTB.value <= threshold=0.994031548500061
node_1: feature_name=MTRNR2L2	MTRNR2L2.value <= threshold=2.8784297704696655
node_2: feature_name=TCL1A	TCL1A.value <= threshold=4.798312664031982
node_3: feature_name=MT-CO1	MT-CO1.value > threshold=0.08947191759943962
node_2521: feature_name=LTB	LTB.value <= threshold=0.6038667261600494
node_2522: feature_name=HLA-DQB1	HLA-DQB1.value <= threshold=2.1053054332733154
node_2523: feature_name=SNX9	SNX9.value > threshold=-0.5346486568450928
node_2907: feature_name=NR4A1	NR4A1.value > threshold=2.5433837175369263
node_3459: feature_name=VPREB3	VPREB3.value <= threshold=4.410779237747192
node_3460: feature_name=RALGPS2	RALGPS2.value <= threshold=3.064661979675293
node_3461: feature_name=MT-CO1	MT-CO1.value <= threshold=1.7489010095596313
node_3462: feature_name=HLA-DQA2	HLA-DQA2.value <= threshold=1.794841468334198
node_3463: feature_name=CYBB	CYBB.value > threshold=1.5984815955162048
node_3491: feature_name=PELI1	PELI1.value > threshold=0.13108068704605103
node_3497: feature_name=NFKBID	NFKBID.value <= threshold=4.821499824523926
Class: COVID-19 Critical	

Rules_439	passed counts:10
node_0: feature_name=LTB	LTB.value <= threshold=0.994031548500061
node_1: feature_name=MTRNR2L2	MTRNR2L2.value <= threshold=2.8784297704696655
node_2: feature_name=TCL1A	TCL1A.value <= threshold=4.798312664031982
node_3: feature_name=MT-CO1	MT-CO1.value > threshold=0.08947191759943962
node_2521: feature_name=LTB	LTB.value <= threshold=0.6038667261600494
node_2522: feature_name=HLA-DQB1	HLA-DQB1.value <= threshold=2.1053054332733154
node_2523: feature_name=SNX9	SNX9.value > threshold=-0.5346486568450928
node_2907: feature_name=NR4A1	NR4A1.value > threshold=2.5433837175369263
node_3459: feature_name=VPREB3	VPREB3.value <= threshold=4.410779237747192
node_3460: feature_name=RALGPS2	RALGPS2.value <= threshold=3.064661979675293
node_3461: feature_name=MT-CO1	MT-CO1.value <= threshold=1.7489010095596313
node_3462: feature_name=HLA-DQA2	HLA-DQA2.value <= threshold=1.794841468334198
node_3463: feature_name=CYBB	CYBB.value <= threshold=1.5984815955162048
node_3464: feature_name=MARCKS	MARCKS.value <= threshold=-0.38392288982868195
node_3465: feature_name=OAZ1	OAZ1.value <= threshold=-0.07741016428917646
node_3466: feature_name=XAF1	XAF1.value <= threshold=-0.4615721255540848
node_3467: feature_name=BANK1	BANK1.value <= threshold=2.4423738718032837
Class: COVID-19 Critical	
Rules_440	passed counts:10
node_0: feature_name=LTB	LTB.value <= threshold=0.994031548500061
node_1: feature_name=MTRNR2L2	MTRNR2L2.value <= threshold=2.8784297704696655
node_2: feature_name=TCL1A	TCL1A.value <= threshold=4.798312664031982
node_3: feature_name=MT-CO1	MT-CO1.value > threshold=0.08947191759943962
node_2521: feature_name=LTB	LTB.value <= threshold=0.6038667261600494
node_2522: feature_name=HLA-DQB1	HLA-DQB1.value <= threshold=2.1053054332733154
node_2523: feature_name=SNX9	SNX9.value > threshold=-0.5346486568450928
node_2907: feature_name=NR4A1	NR4A1.value <= threshold=2.5433837175369263
node_2908: feature_name=XIST	XIST.value <= threshold=0.7664470989257097

node_2909: feature_name=CD79B	CD79B.value > threshold=2.9326847791671753
node_3353: feature_name=VPREB3	VPREB3.value > threshold=3.0226117372512817
node_3425: feature_name=OAZ1	OAZ1.value <= threshold=0.47689591348171234
node_3426: feature_name=HLA-DQA1	HLA-DQA1.value > threshold=2.271668791770935
node_3434: feature_name=YWHAH	YWHAH.value <= threshold=-0.3741019368171692
Class: Healthy Control	
Rules_441	passed counts:10
node_0: feature_name=LTB	LTB.value <= threshold=0.994031548500061
node_1: feature_name=MTRNR2L2	MTRNR2L2.value <= threshold=2.8784297704696655
node_2: feature_name=TCL1A	TCL1A.value <= threshold=4.798312664031982
node_3: feature_name=MT-CO1	MT-CO1.value > threshold=0.08947191759943962
node_2521: feature_name=LTB	LTB.value <= threshold=0.6038667261600494
node_2522: feature_name=HLA-DQB1	HLA-DQB1.value <= threshold=2.1053054332733154
node_2523: feature_name=SNX9	SNX9.value > threshold=-0.5346486568450928
node_2907: feature_name=NR4A1	NR4A1.value <= threshold=2.5433837175369263
node_2908: feature_name=XIST	XIST.value <= threshold=0.7664470989257097
node_2909: feature_name=CD79B	CD79B.value <= threshold=2.9326847791671753
node_2910: feature_name=NR4A1	NR4A1.value > threshold=-0.2875557690858841
node_3096: feature_name=NR4A3	NR4A3.value <= threshold=3.314968705177307
node_3097: feature_name=VPREB3	VPREB3.value <= threshold=3.3549938201904297
node_3098: feature_name=RALGPS2	RALGPS2.value <= threshold=5.807050704956055
node_3099: feature_name=CTD-2035E11.3	CTD-2035E11.3.value <= threshold=6.4090416431427
node_3100: feature_name=FAM129C	FAM129C.value <= threshold=6.966361045837402
node_3101: feature_name=HES4	HES4.value <= threshold=4.139557242393494
node_3102: feature_name=CD83	CD83.value > threshold=1.8343153595924377
node_3136: feature_name=PLD4	PLD4.value <= threshold=4.453821897506714
node_3137: feature_name=HLA-DQA2	HLA-DQA2.value <= threshold=-0.10758286714553833
node_3138: feature_name=STAG3	STAG3.value <= threshold=7.365770578384399
node_3139: feature_name=LDLR	LDLR.value <= threshold=2.3423796892166138

node_3140: feature_name=CD79A	CD79A.value <= threshold=4.038331866264343
node_3141: feature_name=DUSP4	DUSP4.value <= threshold=4.829863548278809
node_3142: feature_name=OAZ1	OAZ1.value > threshold=-0.34704728424549103
node_3178: feature_name=MX1	MX1.value > threshold=-0.48719069361686707
node_3184: feature_name=HLA-DPB1	HLA-DPB1.value <= threshold=2.151633381843567
node_3185: feature_name=RGS2	RGS2.value > threshold=1.594672977924347
node_3203: feature_name=MARCH1	MARCH1.value <= threshold=1.3179876208305359
node_3204: feature_name=LTB	LTB.value > threshold=-0.9328101277351379
Class: COVID-19 Critical	
Rules_442	passed counts:10
node_0: feature_name=LTB	LTB.value <= threshold=0.994031548500061
node_1: feature_name=MTRNR2L2	MTRNR2L2.value <= threshold=2.8784297704696655
node_2: feature_name=TCL1A	TCL1A.value <= threshold=4.798312664031982
node_3: feature_name=MT-CO1	MT-CO1.value > threshold=0.08947191759943962
node_2521: feature_name=LTB	LTB.value <= threshold=0.6038667261600494
node_2522: feature_name=HLA-DQB1	HLA-DQB1.value <= threshold=2.1053054332733154
node_2523: feature_name=SNX9	SNX9.value > threshold=-0.5346486568450928
node_2907: feature_name=NR4A1	NR4A1.value <= threshold=2.5433837175369263
node_2908: feature_name=XIST	XIST.value <= threshold=0.7664470989257097
node_2909: feature_name=CD79B	CD79B.value <= threshold=2.9326847791671753
node_2910: feature_name=NR4A1	NR4A1.value <= threshold=-0.2875557690858841
node_2911: feature_name=CTD-2035E11.3	CTD-2035E11.3.value <= threshold=5.262048006057739
node_2912: feature_name=VPREB3	VPREB3.value <= threshold=4.937594890594482
node_2913: feature_name=IRAK3	IRAK3.value <= threshold=4.592883586883545
node_2914: feature_name=IGJ	IGJ.value <= threshold=7.093410015106201
node_2915: feature_name=WDR74	WDR74.value <= threshold=4.077916383743286
node_2916: feature_name=MEF2C	MEF2C.value <= threshold=4.374382972717285
node_2917: feature_name=HIST1H2AC	HIST1H2AC.value <= threshold=3.743621349334717
node_2918: feature_name=STMN1	STMN1.value <= threshold=6.266510248184204

node_2919: feature_name=RALGPS2	RALGPS2.value <= threshold=5.72194766998291
node_2920: feature_name=HOPX	HOPX.value <= threshold=2.5944807529449463
node_2921: feature_name=MARCKS	MARCKS.value <= threshold=4.622651815414429
node_2922: feature_name=CCL3	CCL3.value <= threshold=0.7484713792800903
node_2923: feature_name=TPM1	TPM1.value <= threshold=6.446193695068359
node_2924: feature_name=PLD4	PLD4.value <= threshold=8.038907289505005
node_2925: feature_name=COTL1	COTL1.value > threshold=-1.8123814463615417
node_2929: feature_name=ID3	ID3.value <= threshold=7.534881353378296
node_2930: feature_name=IRF8	IRF8.value <= threshold=4.855781078338623
node_2931: feature_name=AP3M2	AP3M2.value <= threshold=1.8868122100830078
node_2932: feature_name=FCER2	FCER2.value <= threshold=6.877448558807373
node_2933: feature_name=MTRNR2L2	MTRNR2L2.value <= threshold=2.710945725440979
node_2934: feature_name=GNG11	GNG11.value > threshold=2.510925054550171
node_3008: feature_name=DENND3	DENND3.value > threshold=-0.3559011369943619
Class: COVID-19 Critical	
Rules_443	passed counts:10
node_0: feature_name=LTB	LTB.value <= threshold=0.994031548500061
node_1: feature_name=MTRNR2L2	MTRNR2L2.value <= threshold=2.8784297704696655
node_2: feature_name=TCL1A	TCL1A.value <= threshold=4.798312664031982
node_3: feature_name=MT-CO1	MT-CO1.value > threshold=0.08947191759943962
node_2521: feature_name=LTB	LTB.value <= threshold=0.6038667261600494
node_2522: feature_name=HLA-DQB1	HLA-DQB1.value <= threshold=2.1053054332733154
node_2523: feature_name=SNX9	SNX9.value <= threshold=-0.5346486568450928
node_2524: feature_name=HLA-DPB1	HLA-DPB1.value <= threshold=1.9548270106315613
node_2525: feature_name=FAM129C	FAM129C.value <= threshold=3.3393388986587524
node_2526: feature_name=XIST	XIST.value <= threshold=3.6911416053771973
node_2527: feature_name=NR4A1	NR4A1.value > threshold=-0.2557755410671234
node_2625: feature_name=HLA-DPA1	HLA-DPA1.value > threshold=0.9335225224494934
node_2657: feature_name=RALGPS2	RALGPS2.value <= threshold=2.9413715600967407

node_2658: feature_name=HLA-DQB1	HLA-DQB1.value > threshold=1.7503233551979065
node_2692: feature_name=MEF2C	MEF2C.value > threshold=0.2689770758152008
node_2696: feature_name=DAPP1	DAPP1.value <= threshold=2.193618059158325
Class: Healthy Control	
Rules_444	passed counts:10
node_0: feature_name=LTB	LTB.value <= threshold=0.994031548500061
node_1: feature_name=MTRNR2L2	MTRNR2L2.value <= threshold=2.8784297704696655
node_2: feature_name=TCL1A	TCL1A.value <= threshold=4.798312664031982
node_3: feature_name=MT-CO1	MT-CO1.value > threshold=0.08947191759943962
node_2521: feature_name=LTB	LTB.value <= threshold=0.6038667261600494
node_2522: feature_name=HLA-DQB1	HLA-DQB1.value <= threshold=2.1053054332733154
node_2523: feature_name=SNX9	SNX9.value <= threshold=-0.5346486568450928
node_2524: feature_name=HLA-DPB1	HLA-DPB1.value <= threshold=1.9548270106315613
node_2525: feature_name=FAM129C	FAM129C.value <= threshold=3.3393388986587524
node_2526: feature_name=XIST	XIST.value <= threshold=3.6911416053771973
node_2527: feature_name=NR4A1	NR4A1.value <= threshold=-0.2557755410671234
node_2528: feature_name=HOPX	HOPX.value <= threshold=2.776473879814148
node_2529: feature_name=AREGB	AREGB.value <= threshold=-0.10896047577261925
node_2530: feature_name=KIAA0125	KIAA0125.value <= threshold=7.652706623077393
node_2531: feature_name=MT-CO3	MT-CO3.value > threshold=-1.3726763725280762
node_2533: feature_name=TNF	TNF.value <= threshold=3.1101887226104736
node_2534: feature_name=NFKBID	NFKBID.value > threshold=-0.39706873893737793
node_2568: feature_name=TCF4	TCF4.value <= threshold=6.289925575256348
node_2569: feature_name=CD22	CD22.value <= threshold=8.377277374267578
node_2570: feature_name=FERMT3	FERMT3.value <= threshold=2.260508179664612
node_2571: feature_name=MT-ND6	MT-ND6.value <= threshold=3.413356304168701
node_2572: feature_name=ID1	ID1.value <= threshold=1.9121195077896118
node_2573: feature_name=DAPK1	DAPK1.value <= threshold=1.6979314014315605
node_2574: feature_name=IRF4	IRF4.value <= threshold=8.765593528747559

node_2575: feature_name=PLD4	PLD4.value <= threshold=6.6784141063690186
node_2576: feature_name=GPX1	GPX1.value > threshold=-0.9480071663856506
node_2582: feature_name=MYL9	MYL9.value <= threshold=2.9503014087677
node_2583: feature_name=ID1	ID1.value <= threshold=1.3511708974838257
node_2584: feature_name=HLA-DRB5	HLA-DRB5.value > threshold=-0.8129743039608002
node_2588: feature_name=HLA-DQA1	HLA-DQA1.value <= threshold=-0.44121430814266205
node_2589: feature_name=AREGB	AREGB.value > threshold=-0.1138153076171875
Class: COVID-19 Critical	
Rules_445	passed counts:10
node_0: feature_name=LTB	LTB.value <= threshold=0.994031548500061
node_1: feature_name=MTRNR2L2	MTRNR2L2.value <= threshold=2.8784297704696655
node_2: feature_name=TCL1A	TCL1A.value <= threshold=4.798312664031982
node_3: feature_name=MT-CO1	MT-CO1.value <= threshold=0.08947191759943962
node_4: feature_name=NR4A1	NR4A1.value > threshold=1.0725348591804504
node_1622: feature_name=HLA-DQA2	HLA-DQA2.value > threshold=1.9496816992759705
node_2350: feature_name=BCL2A1	BCL2A1.value <= threshold=1.1322240233421326
node_2351: feature_name=CD83	CD83.value > threshold=2.3162450790405273
node_2391: feature_name=CD79B	CD79B.value <= threshold=1.7111427783966064
node_2392: feature_name=TTN	TTN.value > threshold=-0.28553803265094757
node_2430: feature_name=LTB	LTB.value <= threshold=0.3538805693387985
node_2431: feature_name=MT-CO3	MT-CO3.value > threshold=-0.8342754244804382
node_2433: feature_name=MT-CYB	MT-CYB.value > threshold=-0.5145382583141327
node_2435: feature_name=ADAM19	ADAM19.value <= threshold=2.372166395187378
node_2436: feature_name=UTY	UTY.value <= threshold=1.0755369067192078
Class: COVID-19 Critical	
Rules_446	passed counts:10
node_0: feature_name=LTB	LTB.value <= threshold=0.994031548500061
node_1: feature_name=MTRNR2L2	MTRNR2L2.value <= threshold=2.8784297704696655

node_2: feature_name=TCL1A	TCL1A.value <= threshold=4.798312664031982
node_3: feature_name=MT-CO1	MT-CO1.value <= threshold=0.08947191759943962
node_4: feature_name=NR4A1	NR4A1.value > threshold=1.0725348591804504
node_1622: feature_name=HLA-DQA2	HLA-DQA2.value <= threshold=1.9496816992759705
node_1623: feature_name=HLA-DRB5	HLA-DRB5.value <= threshold=2.715766429901123
node_1624: feature_name=HLA-DPA1	HLA-DPA1.value > threshold=1.7050301432609558
node_2056: feature_name=OAZ1	OAZ1.value > threshold=0.456103190779686
node_2266: feature_name=XAF1	XAF1.value <= threshold=-0.4431648850440979
node_2267: feature_name=MT-CO1	MT-CO1.value <= threshold=-0.3505740910768509
node_2268: feature_name=HLA-DRB1	HLA-DRB1.value > threshold=2.1576695442199707
node_2290: feature_name=ATF5	ATF5.value <= threshold=3.288153052330017
Class: COVID-19 Critical	
Rules_447	passed counts:10
node_0: feature_name=LTB	LTB.value <= threshold=0.994031548500061
node_1: feature_name=MTRNR2L2	MTRNR2L2.value <= threshold=2.8784297704696655
node_2: feature_name=TCL1A	TCL1A.value <= threshold=4.798312664031982
node_3: feature_name=MT-CO1	MT-CO1.value <= threshold=0.08947191759943962
node_4: feature_name=NR4A1	NR4A1.value > threshold=1.0725348591804504
node_1622: feature_name=HLA-DQA2	HLA-DQA2.value <= threshold=1.9496816992759705
node_1623: feature_name=HLA-DRB5	HLA-DRB5.value <= threshold=2.715766429901123
node_1624: feature_name=HLA-DPA1	HLA-DPA1.value <= threshold=1.7050301432609558
node_1625: feature_name=CD83	CD83.value <= threshold=2.783489227294922
node_1626: feature_name=XIST	XIST.value <= threshold=0.9589543007314205
node_1627: feature_name=PLD4	PLD4.value > threshold=5.865932464599609
node_1863: feature_name=MT-CO1	MT-CO1.value > threshold=-0.6275922656059265
node_1865: feature_name=MX1	MX1.value <= threshold=1.0075831413269043
Class: Healthy Control	
Rules_448	passed counts:10

node_0: feature_name=LTB	LTB.value <= threshold=0.994031548500061
node_1: feature_name=MTRNR2L2	MTRNR2L2.value <= threshold=2.8784297704696655
node_2: feature_name=TCL1A	TCL1A.value <= threshold=4.798312664031982
node_3: feature_name=MT-CO1	MT-CO1.value <= threshold=0.08947191759943962
node_4: feature_name=NR4A1	NR4A1.value <= threshold=1.0725348591804504
node_5: feature_name=HLA-DPA1	HLA-DPA1.value > threshold=1.6885469555854797
node_861: feature_name=CD83	CD83.value > threshold=3.0563642978668213
node_1539: feature_name=ID1	ID1.value <= threshold=1.4626862406730652
node_1540: feature_name=FTH1	FTH1.value > threshold=0.2985198348760605
node_1590: feature_name=RP5-821D11.7	RP5-821D11.7.value <= threshold=-0.2580558806657791
node_1591: feature_name=HSP90B1	HSP90B1.value > threshold=1.1008275151252747
node_1605: feature_name=MT-CO2	MT-CO2.value <= threshold=-0.15356595814228058
Class: COVID-19 Severe	
Rules_449	passed counts:10
node_0: feature_name=LTB	LTB.value <= threshold=0.994031548500061
node_1: feature_name=MTRNR2L2	MTRNR2L2.value <= threshold=2.8784297704696655
node_2: feature_name=TCL1A	TCL1A.value <= threshold=4.798312664031982
node_3: feature_name=MT-CO1	MT-CO1.value <= threshold=0.08947191759943962
node_4: feature_name=NR4A1	NR4A1.value <= threshold=1.0725348591804504
node_5: feature_name=HLA-DPA1	HLA-DPA1.value > threshold=1.6885469555854797
node_861: feature_name=CD83	CD83.value <= threshold=3.0563642978668213
node_862: feature_name=HLA-DQB1	HLA-DQB1.value > threshold=2.61081063747406
node_1358: feature_name=HLA-DRB5	HLA-DRB5.value > threshold=0.7542744874954224
node_1390: feature_name=HLA-DRB5	HLA-DRB5.value <= threshold=2.6244447231292725
node_1391: feature_name=XIST	XIST.value <= threshold=-0.22017984092235565
node_1392: feature_name=MT-CO3	MT-CO3.value > threshold=0.12647542357444763
node_1472: feature_name=IFI44L	IFI44L.value <= threshold=-0.34079885482788086
node_1473: feature_name=TNFAIP3	TNFAIP3.value <= threshold=-0.0701381303369999
node_1474: feature_name=HSP90B1	HSP90B1.value > threshold=0.9771479368209839

node_1488: feature_name=PLA2G12A	PLA2G12A.value <= threshold=2.156290076673031
Class: COVID-19 Severe	
Rules_450	passed counts:10
node_0: feature_name=LTB	LTB.value <= threshold=0.994031548500061
node_1: feature_name=MTRNR2L2	MTRNR2L2.value <= threshold=2.8784297704696655
node_2: feature_name=TCL1A	TCL1A.value <= threshold=4.798312664031982
node_3: feature_name=MT-CO1	MT-CO1.value <= threshold=0.08947191759943962
node_4: feature_name=NR4A1	NR4A1.value <= threshold=1.0725348591804504
node_5: feature_name=HLA-DPA1	HLA-DPA1.value > threshold=1.6885469555854797
node_861: feature_name=CD83	CD83.value <= threshold=3.0563642978668213
node_862: feature_name=HLA-DQB1	HLA-DQB1.value > threshold=2.61081063747406
node_1358: feature_name=HLA-DRB5	HLA-DRB5.value > threshold=0.7542744874954224
node_1390: feature_name=HLA-DRB5	HLA-DRB5.value <= threshold=2.6244447231292725
node_1391: feature_name=XIST	XIST.value <= threshold=-0.22017984092235565
node_1392: feature_name=MT-CO3	MT-CO3.value <= threshold=0.12647542357444763
node_1393: feature_name=CD79B	CD79B.value <= threshold=4.338301658630371
node_1394: feature_name=LTB	LTB.value <= threshold=0.9865012466907501
node_1395: feature_name=MEF2C	MEF2C.value <= threshold=4.133931398391724
node_1396: feature_name=MEF2C	MEF2C.value > threshold=-0.38076959550380707
node_1418: feature_name=HLA-DPB1	HLA-DPB1.value > threshold=0.8961269855499268
node_1422: feature_name=HLA-DPA1	HLA-DPA1.value <= threshold=2.9194568395614624
node_1423: feature_name=IL1B	IL1B.value <= threshold=2.321333408355713
node_1424: feature_name=TRIB1	TRIB1.value <= threshold=4.043357253074646
node_1425: feature_name=NT5C3A	NT5C3A.value <= threshold=3.4580591917037964
node_1426: feature_name=MT1E	MT1E.value <= threshold=1.7696760445833206
node_1427: feature_name=SESN3	SESN3.value <= threshold=3.1229121685028076
node_1428: feature_name=HLA-DRB1	HLA-DRB1.value <= threshold=2.7549219131469727
node_1429: feature_name=OASL	OASL.value <= threshold=3.688907504081726
node_1430: feature_name=ITM2C	ITM2C.value > threshold=3.4028767347335815

node_1448: feature_name=BACH1	BACH1.value > threshold=-0.5225754976272583
Class: COVID-19 Critical	
Rules_451	passed counts:10
node_0: feature_name=LTB	LTB.value <= threshold=0.994031548500061
node_1: feature_name=MTRNR2L2	MTRNR2L2.value <= threshold=2.8784297704696655
node_2: feature_name=TCL1A	TCL1A.value <= threshold=4.798312664031982
node_3: feature_name=MT-CO1	MT-CO1.value <= threshold=0.08947191759943962
node_4: feature_name=NR4A1	NR4A1.value <= threshold=1.0725348591804504
node_5: feature_name=HLA-DPA1	HLA-DPA1.value > threshold=1.6885469555854797
node_861: feature_name=CD83	CD83.value <= threshold=3.0563642978668213
node_862: feature_name=HLA-DQB1	HLA-DQB1.value > threshold=2.61081063747406
node_1358: feature_name=HLA-DRB5	HLA-DRB5.value <= threshold=0.7542744874954224
node_1359: feature_name=MT-CYB	MT-CYB.value <= threshold=-0.06432783603668213
node_1360: feature_name=NFKBID	NFKBID.value > threshold=2.1380220651626587
node_1368: feature_name=YWHAH	YWHAH.value <= threshold=-0.26376135647296906
node_1369: feature_name=MS4A1	MS4A1.value <= threshold=3.1560086011886597
Class: COVID-19 Severe	
Rules_452	passed counts:10
node_0: feature_name=LTB	LTB.value <= threshold=0.994031548500061
node_1: feature_name=MTRNR2L2	MTRNR2L2.value <= threshold=2.8784297704696655
node_2: feature_name=TCL1A	TCL1A.value <= threshold=4.798312664031982
node_3: feature_name=MT-CO1	MT-CO1.value <= threshold=0.08947191759943962
node_4: feature_name=NR4A1	NR4A1.value <= threshold=1.0725348591804504
node_5: feature_name=HLA-DPA1	HLA-DPA1.value > threshold=1.6885469555854797
node_861: feature_name=CD83	CD83.value <= threshold=3.0563642978668213
node_862: feature_name=HLA-DQB1	HLA-DQB1.value <= threshold=2.61081063747406
node_863: feature_name=MTRNR2L2	MTRNR2L2.value <= threshold=2.0919997692108154
node_864: feature_name=BCL2A1	BCL2A1.value > threshold=1.852618932723999

node_1328: feature_name=ATF5	ATF5.value <= threshold=1.995988368988037
node_1329: feature_name=S100A6	S100A6.value <= threshold=-1.6572160124778748
node_1330: feature_name=JARID2	JARID2.value > threshold=-0.4899669587612152
node_1332: feature_name=JUN	JUN.value <= threshold=1.1276743412017822
Class: COVID-19 Severe	
Rules_453	passed counts:10
node_0: feature_name=LTB	LTB.value <= threshold=0.994031548500061
node_1: feature_name=MTRNR2L2	MTRNR2L2.value <= threshold=2.8784297704696655
node_2: feature_name=TCL1A	TCL1A.value <= threshold=4.798312664031982
node_3: feature_name=MT-CO1	MT-CO1.value <= threshold=0.08947191759943962
node_4: feature_name=NR4A1	NR4A1.value <= threshold=1.0725348591804504
node_5: feature_name=HLA-DPA1	HLA-DPA1.value > threshold=1.6885469555854797
node_861: feature_name=CD83	CD83.value <= threshold=3.0563642978668213
node_862: feature_name=HLA-DQB1	HLA-DQB1.value <= threshold=2.61081063747406
node_863: feature_name=MTRNR2L2	MTRNR2L2.value <= threshold=2.0919997692108154
node_864: feature_name=BCL2A1	BCL2A1.value <= threshold=1.852618932723999
node_865: feature_name=CD79B	CD79B.value <= threshold=4.352142333984375
node_866: feature_name=TNFAIP3	TNFAIP3.value <= threshold=-0.0449496041983366
node_867: feature_name=MT-CO1	MT-CO1.value <= threshold=-0.08531957119703293
node_868: feature_name=MTRNR2L8	MTRNR2L8.value <= threshold=0.8770350813865662
node_869: feature_name=EGR1	EGR1.value > threshold=-0.32270750403404236
node_1015: feature_name=NR4A3	NR4A3.value <= threshold=1.9085431098937988
node_1016: feature_name=ID3	ID3.value <= threshold=2.776647686958313
node_1017: feature_name=XIST	XIST.value <= threshold=2.618046522140503
node_1018: feature_name=CD79B	CD79B.value <= threshold=3.818994164466858
node_1019: feature_name=NFKBID	NFKBID.value <= threshold=3.4191306829452515
node_1020: feature_name=MIAT	MIAT.value <= threshold=3.532225251197815
node_1021: feature_name=STAG3	STAG3.value <= threshold=9.534917831420898
node_1022: feature_name=OASL	OASL.value <= threshold=2.3923760652542114

node_1023: feature_name=HLA-DQB1	HLA-DQB1.value <= threshold=2.572031259536743
node_1024: feature_name=CD79A	CD79A.value > threshold=0.1879405975341797
node_1028: feature_name=NR4A1	NR4A1.value <= threshold=0.7387348711490631
node_1029: feature_name=SLC7A5	SLC7A5.value <= threshold=2.0677570104599
node_1030: feature_name=HLA-DPB1	HLA-DPB1.value <= threshold=2.17708420753479
node_1031: feature_name=MARCH1	MARCH1.value <= threshold=4.443183898925781
node_1032: feature_name=ATF5	ATF5.value <= threshold=6.411617755889893
node_1033: feature_name=MYL9	MYL9.value <= threshold=3.8704605102539062
node_1034: feature_name=LTB	LTB.value > threshold=0.9670123755931854
node_1062: feature_name=YWHAH	YWHAH.value > threshold=-0.3901667594909668
Class: COVID-19 Critical	
Rules_454	passed counts:10
node_0: feature_name=LTB	LTB.value <= threshold=0.994031548500061
node_1: feature_name=MTRNR2L2	MTRNR2L2.value <= threshold=2.8784297704696655
node_2: feature_name=TCL1A	TCL1A.value <= threshold=4.798312664031982
node_3: feature_name=MT-CO1	MT-CO1.value <= threshold=0.08947191759943962
node_4: feature_name=NR4A1	NR4A1.value <= threshold=1.0725348591804504
node_5: feature_name=HLA-DPA1	HLA-DPA1.value > threshold=1.6885469555854797
node_861: feature_name=CD83	CD83.value <= threshold=3.0563642978668213
node_862: feature_name=HLA-DQB1	HLA-DQB1.value <= threshold=2.61081063747406
node_863: feature_name=MTRNR2L2	MTRNR2L2.value <= threshold=2.0919997692108154
node_864: feature_name=BCL2A1	BCL2A1.value <= threshold=1.852618932723999
node_865: feature_name=CD79B	CD79B.value <= threshold=4.352142333984375
node_866: feature_name=TNFAIP3	TNFAIP3.value <= threshold=-0.0449496041983366
node_867: feature_name=MT-CO1	MT-CO1.value <= threshold=-0.08531957119703293
node_868: feature_name=MTRNR2L8	MTRNR2L8.value <= threshold=0.8770350813865662
node_869: feature_name=EGR1	EGR1.value <= threshold=-0.32270750403404236
node_870: feature_name=IGJ	IGJ.value <= threshold=4.618318557739258
node_871: feature_name=DUSP4	DUSP4.value <= threshold=2.8197922706604004

node_872: feature_name=HOPX	HOPX.value <= threshold=2.10397732257843
node_873: feature_name=RGCC	RGCC.value <= threshold=1.2070720195770264
node_874: feature_name=OAZ1	OAZ1.value <= threshold=1.02116060256958
node_875: feature_name=NAIP	NAIP.value <= threshold=3.768904685974121
node_876: feature_name=LDLR	LDLR.value <= threshold=2.9582698345184326
node_877: feature_name=HLA-DPA1	HLA-DPA1.value <= threshold=3.0588693618774414
node_878: feature_name=RHOB	RHOB.value <= threshold=5.08755898475647
node_879: feature_name=SYTL3	SYTL3.value <= threshold=1.5127440690994263
node_880: feature_name=FTH1	FTH1.value > threshold=-1.4152275919914246
node_882: feature_name=CCL3	CCL3.value <= threshold=2.253131687641144
node_883: feature_name=CD83	CD83.value <= threshold=2.8566592931747437
node_884: feature_name=HLA-DRB5	HLA-DRB5.value > threshold=-0.6464254856109619
node_888: feature_name=HSP90B1	HSP90B1.value > threshold=-1.186358630657196
node_892: feature_name=ATG2A	ATG2A.value <= threshold=2.3624722957611084
node_893: feature_name=ICAM1	ICAM1.value <= threshold=2.4306468963623047
node_894: feature_name=CD38	CD38.value <= threshold=3.4977954626083374
node_895: feature_name=HLA-DRB1	HLA-DRB1.value > threshold=0.28603747487068176
node_899: feature_name=XIST	XIST.value <= threshold=1.4272559583187103
node_900: feature_name=BANK1	BANK1.value <= threshold=5.060973644256592
node_901: feature_name=IRF4	IRF4.value <= threshold=6.117950916290283
node_902: feature_name=MTRNR2L2	MTRNR2L2.value <= threshold=1.8410876989364624
node_903: feature_name=TPM1	TPM1.value <= threshold=4.590091228485107
node_904: feature_name=SNORD3B-2	SNORD3B-2.value > threshold=4.349201917648315
node_930: feature_name=CD22	CD22.value <= threshold=5.764584064483643
Class: COVID-19 Critical	
Rules_455	passed counts:10
node_0: feature_name=LTB	LTB.value <= threshold=0.994031548500061
node_1: feature_name=MTRNR2L2	MTRNR2L2.value <= threshold=2.8784297704696655
node_2: feature_name=TCL1A	TCL1A.value <= threshold=4.798312664031982

node_3: feature_name=MT-CO1	MT-CO1.value <= threshold=0.08947191759943962
node_4: feature_name=NR4A1	NR4A1.value <= threshold=1.0725348591804504
node_5: feature_name=HLA-DPA1	HLA-DPA1.value <= threshold=1.6885469555854797
node_6: feature_name=HLA-DRB5	HLA-DRB5.value <= threshold=2.1886194944381714
node_7: feature_name=XIST	XIST.value <= threshold=-0.12328458577394485
node_8: feature_name=SYNE1	SYNE1.value > threshold=-0.7835594415664673
node_16: feature_name=MTRNR2L2	MTRNR2L2.value <= threshold=2.4483247995376587
node_17: feature_name=HLA-DQA2	HLA-DQA2.value <= threshold=2.227178931236267
node_18: feature_name=NR4A1	NR4A1.value > threshold=-0.21380723267793655
node_504: feature_name=LTB	LTB.value <= threshold=0.8758118152618408
node_505: feature_name=TNFAIP3	TNFAIP3.value <= threshold=0.4779366999864578
node_506: feature_name=HLA-DQB1	HLA-DQB1.value <= threshold=2.9689468145370483
node_507: feature_name=MS4A1	MS4A1.value <= threshold=3.7428637742996216
node_508: feature_name=SNX9	SNX9.value <= threshold=3.304862380027771
node_509: feature_name=MARCKS	MARCKS.value > threshold=-0.519626647233963
node_513: feature_name=CD79B	CD79B.value > threshold=3.0890846252441406
node_563: feature_name=NR4A3	NR4A3.value <= threshold=-0.4203310012817383
node_564: feature_name=FERMT3	FERMT3.value <= threshold=0.8333712220191956
Class: COVID-19 Critical	
Rules_456	passed counts:10
node_0: feature_name=LTB	LTB.value <= threshold=0.994031548500061
node_1: feature_name=MTRNR2L2	MTRNR2L2.value <= threshold=2.8784297704696655
node_2: feature_name=TCL1A	TCL1A.value <= threshold=4.798312664031982
node_3: feature_name=MT-CO1	MT-CO1.value <= threshold=0.08947191759943962
node_4: feature_name=NR4A1	NR4A1.value <= threshold=1.0725348591804504
node_5: feature_name=HLA-DPA1	HLA-DPA1.value <= threshold=1.6885469555854797
node_6: feature_name=HLA-DRB5	HLA-DRB5.value <= threshold=2.1886194944381714
node_7: feature_name=XIST	XIST.value <= threshold=-0.12328458577394485
node_8: feature_name=SYNE1	SYNE1.value > threshold=-0.7835594415664673

node_16: feature_name=MTRNR2L2	MTRNR2L2.value <= threshold=2.4483247995376587
node_17: feature_name=HLA-DQA2	HLA-DQA2.value <= threshold=2.227178931236267
node_18: feature_name=NR4A1	NR4A1.value <= threshold=-0.21380723267793655
node_19: feature_name=IRAK3	IRAK3.value <= threshold=4.27159571647644
node_20: feature_name=IFIT2	IFIT2.value <= threshold=7.8175740242004395
node_21: feature_name=CD79A	CD79A.value <= threshold=2.857242226600647
node_22: feature_name=HIST1H2AC	HIST1H2AC.value <= threshold=5.077470302581787
node_23: feature_name=FTH1	FTH1.value <= threshold=0.4158056080341339
node_24: feature_name=ATF3	ATF3.value <= threshold=3.8028353452682495
node_25: feature_name=OASL	OASL.value <= threshold=3.387580633163452
node_26: feature_name=MT-CO1	MT-CO1.value > threshold=-0.7942646145820618
node_74: feature_name=HOPX	HOPX.value > threshold=0.8682331740856171
node_250: feature_name=CD22	CD22.value > threshold=-0.23878339678049088
node_254: feature_name=MT-CO2	MT-CO2.value > threshold=0.39438967406749725
node_274: feature_name=CD79A	CD79A.value <= threshold=2.507172465324402
node_275: feature_name=FCRL1	FCRL1.value <= threshold=1.3748255372047424
Class: COVID-19 Critical	
Rules_457	passed counts:10
node_0: feature_name=LTB	LTB.value <= threshold=0.994031548500061
node_1: feature_name=MTRNR2L2	MTRNR2L2.value <= threshold=2.8784297704696655
node_2: feature_name=TCL1A	TCL1A.value <= threshold=4.798312664031982
node_3: feature_name=MT-CO1	MT-CO1.value <= threshold=0.08947191759943962
node_4: feature_name=NR4A1	NR4A1.value <= threshold=1.0725348591804504
node_5: feature_name=HLA-DPA1	HLA-DPA1.value <= threshold=1.6885469555854797
node_6: feature_name=HLA-DRB5	HLA-DRB5.value <= threshold=2.1886194944381714
node_7: feature_name=XIST	XIST.value <= threshold=-0.12328458577394485
node_8: feature_name=SYNE1	SYNE1.value > threshold=-0.7835594415664673
node_16: feature_name=MTRNR2L2	MTRNR2L2.value <= threshold=2.4483247995376587
node_17: feature_name=HLA-DQA2	HLA-DQA2.value <= threshold=2.227178931236267

node_18: feature_name=NR4A1	NR4A1.value <= threshold=-0.21380723267793655
node_19: feature_name=IRAK3	IRAK3.value <= threshold=4.27159571647644
node_20: feature_name=IFIT2	IFIT2.value <= threshold=7.8175740242004395
node_21: feature_name=CD79A	CD79A.value <= threshold=2.857242226600647
node_22: feature_name=HIST1H2AC	HIST1H2AC.value <= threshold=5.077470302581787
node_23: feature_name=FTH1	FTH1.value <= threshold=0.4158056080341339
node_24: feature_name=ATF3	ATF3.value <= threshold=3.8028353452682495
node_25: feature_name=OASL	OASL.value <= threshold=3.387580633163452
node_26: feature_name=MT-CO1	MT-CO1.value > threshold=-0.7942646145820618
node_74: feature_name=HOPX	HOPX.value <= threshold=0.8682331740856171
node_75: feature_name=RGS2	RGS2.value <= threshold=1.4589954018592834
node_76: feature_name=AHR	AHR.value <= threshold=3.870481848716736
node_77: feature_name=IGJ	IGJ.value <= threshold=4.902882814407349
node_78: feature_name=CD79B	CD79B.value <= threshold=4.87716269493103
node_79: feature_name=HLA-DQB1	HLA-DQB1.value <= threshold=3.6925491094589233
node_80: feature_name=HLA-DRB5	HLA-DRB5.value <= threshold=2.0926843881607056
node_81: feature_name=MCTP1	MCTP1.value <= threshold=4.010472774505615
node_82: feature_name=GNG11	GNG11.value <= threshold=3.627470850944519
node_83: feature_name=HSP90B1	HSP90B1.value > threshold=2.0835131406784058
node_191: feature_name=IFIT3	IFIT3.value > threshold=-0.21995635330677032
Class: COVID-19 Critical	
Rules_458	passed counts:9
node_0: feature_name=LTB	LTB.value > threshold=0.994031548500061
node_5264: feature_name=MT-CO1	MT-CO1.value > threshold=-0.4127991199493408
node_5638: feature_name=OAZ1	OAZ1.value > threshold=-0.2640867084264755
node_6112: feature_name=IFI44L	IFI44L.value <= threshold=2.5893263816833496
node_6113: feature_name=TCL1A	TCL1A.value <= threshold=4.201832294464111
node_6114: feature_name=HLA-DQA2	HLA-DQA2.value > threshold=-0.2270243540406227
node_6542: feature_name=CD79B	CD79B.value > threshold=3.096232771873474

node_6872: feature_name=ICAM1	ICAM1.value <= threshold=-0.2232399433851242
node_6873: feature_name=STAG3	STAG3.value <= threshold=-0.19312061369419098
node_6874: feature_name=CD79A	CD79A.value <= threshold=1.57558012008667
node_6875: feature_name=TNFAIP3	TNFAIP3.value > threshold=-1.4814274907112122
Class: COVID-19 Critical	
Rules_459	passed counts:9
node_0: feature_name=LTB	LTB.value > threshold=0.994031548500061
node_5264: feature_name=MT-CO1	MT-CO1.value > threshold=-0.4127991199493408
node_5638: feature_name=OAZ1	OAZ1.value > threshold=-0.2640867084264755
node_6112: feature_name=IFI44L	IFI44L.value <= threshold=2.5893263816833496
node_6113: feature_name=TCL1A	TCL1A.value <= threshold=4.201832294464111
node_6114: feature_name=HLA-DQA2	HLA-DQA2.value > threshold=-0.2270243540406227
node_6542: feature_name=CD79B	CD79B.value <= threshold=3.096232771873474
node_6543: feature_name=NR4A1	NR4A1.value > threshold=1.421015739440918
node_6811: feature_name=SLC7A5	SLC7A5.value > threshold=1.570515513420105
node_6867: feature_name=HSP90B1	HSP90B1.value > threshold=-0.995681881904602
node_6869: feature_name=MT-CYB	MT-CYB.value > threshold=0.2630006968975067
Class: COVID-19 Critical	
Rules_460	passed counts:9
node_0: feature_name=LTB	LTB.value > threshold=0.994031548500061
node_5264: feature_name=MT-CO1	MT-CO1.value > threshold=-0.4127991199493408
node_5638: feature_name=OAZ1	OAZ1.value > threshold=-0.2640867084264755
node_6112: feature_name=IFI44L	IFI44L.value <= threshold=2.5893263816833496
node_6113: feature_name=TCL1A	TCL1A.value <= threshold=4.201832294464111
node_6114: feature_name=HLA-DQA2	HLA-DQA2.value > threshold=-0.2270243540406227
node_6542: feature_name=CD79B	CD79B.value <= threshold=3.096232771873474
node_6543: feature_name=NR4A1	NR4A1.value <= threshold=1.421015739440918
node_6544: feature_name=LTB	LTB.value > threshold=1.359755277633667

node_6720: feature_name=NFKBIZ	NFKBIZ.value <= threshold=0.2720414251089096
node_6721: feature_name=ATF5	ATF5.value <= threshold=2.4672781229019165
node_6722: feature_name=SNX9	SNX9.value <= threshold=2.0814030170440674
node_6723: feature_name=HLA-DQA2	HLA-DQA2.value <= threshold=4.360614776611328
node_6724: feature_name=MT-CO1	MT-CO1.value <= threshold=-0.12188276648521423
node_6725: feature_name=CD79B	CD79B.value <= threshold=2.3298217058181763
node_6726: feature_name=MARCKS	MARCKS.value <= threshold=2.858885169029236
node_6727: feature_name=AHR	AHR.value <= threshold=-0.3792427331209183
node_6728: feature_name=ADAM19	ADAM19.value <= threshold=4.289629697799683
Class: COVID-19 Critical	
Rules_461	passed counts:9
node_0: feature_name=LTB	LTB.value > threshold=0.994031548500061
node_5264: feature_name=MT-CO1	MT-CO1.value > threshold=-0.4127991199493408
node_5638: feature_name=OAZ1	OAZ1.value > threshold=-0.2640867084264755
node_6112: feature_name=IFI44L	IFI44L.value <= threshold=2.5893263816833496
node_6113: feature_name=TCL1A	TCL1A.value <= threshold=4.201832294464111
node_6114: feature_name=HLA-DQA2	HLA-DQA2.value > threshold=-0.2270243540406227
node_6542: feature_name=CD79B	CD79B.value <= threshold=3.096232771873474
node_6543: feature_name=NR4A1	NR4A1.value <= threshold=1.421015739440918
node_6544: feature_name=LTB	LTB.value <= threshold=1.359755277633667
node_6545: feature_name=NFKBID	NFKBID.value > threshold=1.4294711351394653
node_6643: feature_name=FAM129C	FAM129C.value <= threshold=4.799884080886841
node_6644: feature_name=XIST	XIST.value <= threshold=0.9931697957217693
node_6645: feature_name=GPX1	GPX1.value > threshold=0.3628883808851242
node_6695: feature_name=CD79A	CD79A.value > threshold=2.4297949075698853
node_6699: feature_name=FCRL1	FCRL1.value > threshold=-0.20751195400953293
Class: Healthy Control	
Rules_462	passed counts:9

node_0: feature_name=LTB	LTB.value > threshold=0.994031548500061
node_5264: feature_name=MT-CO1	MT-CO1.value > threshold=-0.4127991199493408
node_5638: feature_name=OAZ1	OAZ1.value > threshold=-0.2640867084264755
node_6112: feature_name=IFI44L	IFI44L.value <= threshold=2.5893263816833496
node_6113: feature_name=TCL1A	TCL1A.value <= threshold=4.201832294464111
node_6114: feature_name=HLA-DQA2	HLA-DQA2.value > threshold=-0.2270243540406227
node_6542: feature_name=CD79B	CD79B.value <= threshold=3.096232771873474
node_6543: feature_name=NR4A1	NR4A1.value <= threshold=1.421015739440918
node_6544: feature_name=LTB	LTB.value <= threshold=1.359755277633667
node_6545: feature_name=NFKBID	NFKBID.value <= threshold=1.4294711351394653
node_6546: feature_name=HLA-DRB1	HLA-DRB1.value > threshold=1.0398632287979126
node_6556: feature_name=CD79B	CD79B.value <= threshold=-0.2919560521841049
node_6557: feature_name=MTRNR2L2	MTRNR2L2.value <= threshold=2.7710195183753967
node_6558: feature_name=JUN	JUN.value > threshold=0.5767147243022919
node_6574: feature_name=MT-ND6	MT-ND6.value > threshold=0.21166645362973213
node_6578: feature_name=HLA-DPA1	HLA-DPA1.value > threshold=1.3230751752853394
Class: Healthy Control	
Rules_463	passed counts:9
node_0: feature_name=LTB	LTB.value > threshold=0.994031548500061
node_5264: feature_name=MT-CO1	MT-CO1.value > threshold=-0.4127991199493408
node_5638: feature_name=OAZ1	OAZ1.value > threshold=-0.2640867084264755
node_6112: feature_name=IFI44L	IFI44L.value <= threshold=2.5893263816833496
node_6113: feature_name=TCL1A	TCL1A.value <= threshold=4.201832294464111
node_6114: feature_name=HLA-DQA2	HLA-DQA2.value <= threshold=-0.2270243540406227
node_6115: feature_name=MTRNR2L2	MTRNR2L2.value <= threshold=2.548917293548584
node_6116: feature_name=ATF5	ATF5.value > threshold=2.041263461112976
node_6490: feature_name=HLA-DPB1	HLA-DPB1.value <= threshold=1.5343759655952454
node_6491: feature_name=MT-ND6	MT-ND6.value > threshold=1.0284687876701355
node_6505: feature_name=HES4	HES4.value <= threshold=-0.17312173545360565

Class: Healthy Control	
Rules_464	passed counts:9
node_0: feature_name=LTB	LTB.value > threshold=0.994031548500061
node_5264: feature_name=MT-CO1	MT-CO1.value > threshold=-0.4127991199493408
node_5638: feature_name=OAZ1	OAZ1.value > threshold=-0.2640867084264755
node_6112: feature_name=IFI44L	IFI44L.value <= threshold=2.5893263816833496
node_6113: feature_name=TCL1A	TCL1A.value <= threshold=4.201832294464111
node_6114: feature_name=HLA-DQA2	HLA-DQA2.value <= threshold=-0.2270243540406227
node_6115: feature_name=MTRNR2L2	MTRNR2L2.value <= threshold=2.548917293548584
node_6116: feature_name=ATF5	ATF5.value <= threshold=2.041263461112976
node_6117: feature_name=JUN	JUN.value > threshold=-0.616259902715683
node_6189: feature_name=MT-CO3	MT-CO3.value > threshold=0.13483521342277527
node_6351: feature_name=XAF1	XAF1.value <= threshold=3.061717987060547
node_6352: feature_name=MT-CO2	MT-CO2.value <= threshold=1.2605181336402893
node_6353: feature_name=ICAM1	ICAM1.value > threshold=-0.3340446650981903
node_6439: feature_name=FAM129C	FAM129C.value <= threshold=-0.24657993018627167
node_6440: feature_name=ITM2C	ITM2C.value <= threshold=-0.2592049688100815
node_6441: feature_name=TCF4	TCF4.value <= threshold=2.291034698486328
Class: COVID-19 Critical	
Rules_465	passed counts:9
node_0: feature_name=LTB	LTB.value > threshold=0.994031548500061
node_5264: feature_name=MT-CO1	MT-CO1.value <= threshold=-0.4127991199493408
node_5265: feature_name=MTRNR2L2	MTRNR2L2.value <= threshold=2.4473838806152344
node_5266: feature_name=TCL1A	TCL1A.value > threshold=5.787256956100464
node_5616: feature_name=XAF1	XAF1.value > threshold=3.0923240184783936
Class: COVID-19 Critical	
Rules_466	passed counts:9

node_0: feature_name=LTB	LTB.value > threshold=0.994031548500061
node_5264: feature_name=MT-CO1	MT-CO1.value <= threshold=-0.4127991199493408
node_5265: feature_name=MTRNR2L2	MTRNR2L2.value <= threshold=2.4473838806152344
node_5266: feature_name=TCL1A	TCL1A.value <= threshold=5.787256956100464
node_5267: feature_name=PLD4	PLD4.value <= threshold=3.544657826423645
node_5268: feature_name=NR4A1	NR4A1.value > threshold=0.8490704298019409
node_5498: feature_name=HLA-DPA1	HLA-DPA1.value > threshold=1.9079211950302124
node_5556: feature_name=CD83	CD83.value > threshold=1.6135562062263489
node_5560: feature_name=OAZ1	OAZ1.value <= threshold=0.11598343029618263
node_5561: feature_name=STMN1	STMN1.value > threshold=-0.2897801846265793
node_5563: feature_name=MARCH1	MARCH1.value <= threshold=2.132397413253784
Class: COVID-19 Severe	
Rules_467	passed counts:9
node_0: feature_name=LTB	LTB.value > threshold=0.994031548500061
node_5264: feature_name=MT-CO1	MT-CO1.value <= threshold=-0.4127991199493408
node_5265: feature_name=MTRNR2L2	MTRNR2L2.value <= threshold=2.4473838806152344
node_5266: feature_name=TCL1A	TCL1A.value <= threshold=5.787256956100464
node_5267: feature_name=PLD4	PLD4.value <= threshold=3.544657826423645
node_5268: feature_name=NR4A1	NR4A1.value <= threshold=0.8490704298019409
node_5269: feature_name=LTB	LTB.value <= threshold=1.5252206921577454
node_5270: feature_name=HLA-DRB5	HLA-DRB5.value <= threshold=2.5117164850234985
node_5271: feature_name=CD79A	CD79A.value <= threshold=3.685201406478882
node_5272: feature_name=BCL2A1	BCL2A1.value > threshold=2.081526756286621
node_5438: feature_name=RALGPS2	RALGPS2.value <= threshold=2.112855315208435
node_5439: feature_name=SYNE1	SYNE1.value <= threshold=-0.5098004937171936
Class: COVID-19 Severe	
Rules_468	passed counts:9
node_0: feature_name=LTB	LTB.value <= threshold=0.994031548500061

node_1: feature_name=MTRNR2L2	MTRNR2L2.value <= threshold=2.8784297704696655
node_2: feature_name=TCL1A	TCL1A.value > threshold=4.798312664031982
node_4912: feature_name=MT-CO1	MT-CO1.value > threshold=-0.3664844036102295
node_5002: feature_name=IFI44L	IFI44L.value <= threshold=2.3101747035980225
node_5003: feature_name=SNX9	SNX9.value <= threshold=2.0247421264648438
node_5004: feature_name=STAG3	STAG3.value <= threshold=6.482141733169556
node_5005: feature_name=BACH1	BACH1.value > threshold=-0.5237349569797516
node_5047: feature_name=RHOB	RHOB.value > threshold=-0.4843696355819702
node_5055: feature_name=FAM129C	FAM129C.value > threshold=-0.2832885980606079
node_5079: feature_name=TP53INP1	TP53INP1.value > threshold=2.9482468366622925
node_5129: feature_name=CD79B	CD79B.value <= threshold=2.402374267578125
node_5130: feature_name=JARID2	JARID2.value <= threshold=2.191186785697937
node_5131: feature_name=SESN3	SESN3.value <= threshold=1.5416949391365051
Class: COVID-19 Critical	
Rules_469	passed counts:9
node_0: feature_name=LTB	LTB.value <= threshold=0.994031548500061
node_1: feature_name=MTRNR2L2	MTRNR2L2.value <= threshold=2.8784297704696655
node_2: feature_name=TCL1A	TCL1A.value <= threshold=4.798312664031982
node_3: feature_name=MT-CO1	MT-CO1.value > threshold=0.08947191759943962
node_2521: feature_name=LTB	LTB.value > threshold=0.6038667261600494
node_4173: feature_name=OAZ1	OAZ1.value > threshold=0.143283873796463
node_4615: feature_name=NR4A1	NR4A1.value <= threshold=0.6621004343032837
node_4616: feature_name=NFKBID	NFKBID.value <= threshold=3.283741593360901
node_4617: feature_name=HLA-DQA1	HLA-DQA1.value <= threshold=2.3435752391815186
node_4618: feature_name=PELI1	PELI1.value <= threshold=1.3271203637123108
node_4619: feature_name=PLD4	PLD4.value <= threshold=3.3385335206985474
node_4620: feature_name=MEF2A	MEF2A.value > threshold=1.5926940441131592
node_4658: feature_name=STAG3	STAG3.value <= threshold=-0.2016931176185608
node_4659: feature_name=HLA-DQB1	HLA-DQB1.value <= threshold=1.983833909034729

node_4660: feature_name=CD79A	CD79A.value <= threshold=2.912865161895752
Class: COVID-19 Critical	
Rules_470	passed counts:9
node_0: feature_name=LTB	LTB.value <= threshold=0.994031548500061
node_1: feature_name=MTRNR2L2	MTRNR2L2.value <= threshold=2.8784297704696655
node_2: feature_name=TCL1A	TCL1A.value <= threshold=4.798312664031982
node_3: feature_name=MT-CO1	MT-CO1.value > threshold=0.08947191759943962
node_2521: feature_name=LTB	LTB.value > threshold=0.6038667261600494
node_4173: feature_name=OAZ1	OAZ1.value <= threshold=0.143283873796463
node_4174: feature_name=PLD4	PLD4.value <= threshold=2.921932101249695
node_4175: feature_name=XIST	XIST.value <= threshold=-0.05067425221204758
node_4176: feature_name=MT-CO1	MT-CO1.value > threshold=1.345594048500061
node_4486: feature_name=RALGPS2	RALGPS2.value <= threshold=2.7596691846847534
node_4487: feature_name=IFIT2	IFIT2.value > threshold=-0.1915586143732071
node_4501: feature_name=DAPK1	DAPK1.value <= threshold=-0.08581509068608284
node_4502: feature_name=MT-CO2	MT-CO2.value > threshold=1.4776776432991028
node_4504: feature_name=CD83	CD83.value > threshold=-0.5638298988342285
Class: Healthy Control	
Rules_471	passed counts:9
node_0: feature_name=LTB	LTB.value <= threshold=0.994031548500061
node_1: feature_name=MTRNR2L2	MTRNR2L2.value <= threshold=2.8784297704696655
node_2: feature_name=TCL1A	TCL1A.value <= threshold=4.798312664031982
node_3: feature_name=MT-CO1	MT-CO1.value > threshold=0.08947191759943962
node_2521: feature_name=LTB	LTB.value > threshold=0.6038667261600494
node_4173: feature_name=OAZ1	OAZ1.value <= threshold=0.143283873796463
node_4174: feature_name=PLD4	PLD4.value <= threshold=2.921932101249695
node_4175: feature_name=XIST	XIST.value <= threshold=-0.05067425221204758
node_4176: feature_name=MT-CO1	MT-CO1.value <= threshold=1.345594048500061

node_4177: feature_name=HLA-DQB1	HLA-DQB1.value > threshold=1.927736520767212
node_4353: feature_name=ITM2C	ITM2C.value > threshold=-0.12329206243157387
node_4459: feature_name=SNX9	SNX9.value <= threshold=0.6323382556438446
node_4460: feature_name=NR4A1	NR4A1.value <= threshold=0.9784677922725677
node_4461: feature_name=MARCKS	MARCKS.value <= threshold=1.4018014669418335
node_4462: feature_name=RALGPS2	RALGPS2.value > threshold=1.3579027652740479
Class: Healthy Control	
Rules_472	passed counts:9
node_0: feature_name=LTB	LTB.value <= threshold=0.994031548500061
node_1: feature_name=MTRNR2L2	MTRNR2L2.value <= threshold=2.8784297704696655
node_2: feature_name=TCL1A	TCL1A.value <= threshold=4.798312664031982
node_3: feature_name=MT-CO1	MT-CO1.value > threshold=0.08947191759943962
node_2521: feature_name=LTB	LTB.value <= threshold=0.6038667261600494
node_2522: feature_name=HLA-DQB1	HLA-DQB1.value > threshold=2.1053054332733154
node_3550: feature_name=XIST	XIST.value <= threshold=0.7716337237507105
node_3551: feature_name=CD79B	CD79B.value > threshold=2.9426796436309814
node_4039: feature_name=IFI44L	IFI44L.value <= threshold=1.8637521862983704
node_4040: feature_name=SNX9	SNX9.value <= threshold=1.274232804775238
node_4041: feature_name=CASP8	CASP8.value <= threshold=-0.574097603559494
node_4042: feature_name=NFKBID	NFKBID.value > threshold=1.49756920337677
node_4056: feature_name=MIR24-2	MIR24-2.value <= threshold=0.2797542065382004
node_4057: feature_name=MEF2C	MEF2C.value <= threshold=2.6051090955734253
node_4058: feature_name=BOD1L1	BOD1L1.value > threshold=-0.476860374212265
node_4062: feature_name=RHOB	RHOB.value <= threshold=1.5522340536117554
node_4063: feature_name=GPX1	GPX1.value <= threshold=0.3275824636220932
Class: COVID-19 Severe	
Rules_473	passed counts:9
node_0: feature_name=LTB	LTB.value <= threshold=0.994031548500061

node_1: feature_name=MTRNR2L2	MTRNR2L2.value <= threshold=2.8784297704696655
node_2: feature_name=TCL1A	TCL1A.value <= threshold=4.798312664031982
node_3: feature_name=MT-CO1	MT-CO1.value > threshold=0.08947191759943962
node_2521: feature_name=LTB	LTB.value <= threshold=0.6038667261600494
node_2522: feature_name=HLA-DQB1	HLA-DQB1.value > threshold=2.1053054332733154
node_3550: feature_name=XIST	XIST.value <= threshold=0.7716337237507105
node_3551: feature_name=CD79B	CD79B.value <= threshold=2.9426796436309814
node_3552: feature_name=HLA-DQB1	HLA-DQB1.value > threshold=2.713674306869507
node_3854: feature_name=MEF2C	MEF2C.value > threshold=-0.3721291869878769
node_3882: feature_name=IFI44L	IFI44L.value <= threshold=2.5516022443771362
node_3883: feature_name=FCER2	FCER2.value > threshold=-0.21487552672624588
node_3977: feature_name=IGJ	IGJ.value > threshold=2.123470664024353
node_4025: feature_name=PELI1	PELI1.value > threshold=-0.6190133392810822
Class: COVID-19 Severe	
Rules_474	passed counts:9
node_0: feature_name=LTB	LTB.value <= threshold=0.994031548500061
node_1: feature_name=MTRNR2L2	MTRNR2L2.value <= threshold=2.8784297704696655
node_2: feature_name=TCL1A	TCL1A.value <= threshold=4.798312664031982
node_3: feature_name=MT-CO1	MT-CO1.value > threshold=0.08947191759943962
node_2521: feature_name=LTB	LTB.value <= threshold=0.6038667261600494
node_2522: feature_name=HLA-DQB1	HLA-DQB1.value > threshold=2.1053054332733154
node_3550: feature_name=XIST	XIST.value <= threshold=0.7716337237507105
node_3551: feature_name=CD79B	CD79B.value <= threshold=2.9426796436309814
node_3552: feature_name=HLA-DQB1	HLA-DQB1.value > threshold=2.713674306869507
node_3854: feature_name=MEF2C	MEF2C.value > threshold=-0.3721291869878769
node_3882: feature_name=IFI44L	IFI44L.value <= threshold=2.5516022443771362
node_3883: feature_name=FCER2	FCER2.value > threshold=-0.21487552672624588
node_3977: feature_name=IGJ	IGJ.value <= threshold=2.123470664024353
node_3978: feature_name=S100A6	S100A6.value > threshold=-1.7107264399528503

node_3982: feature_name=MT-CO1	MT-CO1.value > threshold=1.5725501775741577
node_4020: feature_name=TSPYL2	TSPYL2.value <= threshold=1.4511758089065552
node_4021: feature_name=WDR74	WDR74.value > threshold=-0.4913913458585739
Class: Healthy Control	
Rules_475	passed counts:9
node_0: feature_name=LTB	LTB.value <= threshold=0.994031548500061
node_1: feature_name=MTRNR2L2	MTRNR2L2.value <= threshold=2.8784297704696655
node_2: feature_name=TCL1A	TCL1A.value <= threshold=4.798312664031982
node_3: feature_name=MT-CO1	MT-CO1.value > threshold=0.08947191759943962
node_2521: feature_name=LTB	LTB.value <= threshold=0.6038667261600494
node_2522: feature_name=HLA-DQB1	HLA-DQB1.value > threshold=2.1053054332733154
node_3550: feature_name=XIST	XIST.value <= threshold=0.7716337237507105
node_3551: feature_name=CD79B	CD79B.value <= threshold=2.9426796436309814
node_3552: feature_name=HLA-DQB1	HLA-DQB1.value <= threshold=2.713674306869507
node_3553: feature_name=SNX9	SNX9.value > threshold=-0.5404067635536194
node_3653: feature_name=LTB	LTB.value > threshold=0.021708179265260696
node_3759: feature_name=TCL1A	TCL1A.value <= threshold=3.3921085596084595
node_3760: feature_name=HLA-DRB5	HLA-DRB5.value > threshold=0.5265218019485474
node_3776: feature_name=ID1	ID1.value > threshold=-0.21235637366771698
node_3828: feature_name=RALGPS2	RALGPS2.value <= threshold=2.589961290359497
node_3829: feature_name=RGS2	RGS2.value <= threshold=1.3759892582893372
node_3830: feature_name=IGLL5	IGLL5.value <= threshold=1.807655394077301
Class: COVID-19 Critical	
Rules_476	passed counts:9
node_0: feature_name=LTB	LTB.value <= threshold=0.994031548500061
node_1: feature_name=MTRNR2L2	MTRNR2L2.value <= threshold=2.8784297704696655
node_2: feature_name=TCL1A	TCL1A.value <= threshold=4.798312664031982
node_3: feature_name=MT-CO1	MT-CO1.value > threshold=0.08947191759943962

node_2521: feature_name=LTB	LTB.value <= threshold=0.6038667261600494
node_2522: feature_name=HLA-DQB1	HLA-DQB1.value > threshold=2.1053054332733154
node_3550: feature_name=XIST	XIST.value <= threshold=0.7716337237507105
node_3551: feature_name=CD79B	CD79B.value <= threshold=2.9426796436309814
node_3552: feature_name=HLA-DQB1	HLA-DQB1.value <= threshold=2.713674306869507
node_3553: feature_name=SNX9	SNX9.value > threshold=-0.5404067635536194
node_3653: feature_name=LTB	LTB.value > threshold=0.021708179265260696
node_3759: feature_name=TCL1A	TCL1A.value <= threshold=3.3921085596084595
node_3760: feature_name=HLA-DRB5	HLA-DRB5.value > threshold=0.5265218019485474
node_3776: feature_name=ID1	ID1.value <= threshold=-0.21235637366771698
node_3777: feature_name=RP11-731F5.2	RP11-731F5.2.value <= threshold=2.830894112586975
node_3778: feature_name=CASP8	CASP8.value <= threshold=2.222941279411316
node_3779: feature_name=MT-CO3	MT-CO3.value > threshold=-0.41117167472839355
node_3785: feature_name=HLA-DRB5	HLA-DRB5.value <= threshold=0.8465962707996368
node_3786: feature_name=NFKBID	NFKBID.value > threshold=1.7803236246109009
Class: COVID-19 Critical	
Rules_477	passed counts:9
node_0: feature_name=LTB	LTB.value <= threshold=0.994031548500061
node_1: feature_name=MTRNR2L2	MTRNR2L2.value <= threshold=2.8784297704696655
node_2: feature_name=TCL1A	TCL1A.value <= threshold=4.798312664031982
node_3: feature_name=MT-CO1	MT-CO1.value > threshold=0.08947191759943962
node_2521: feature_name=LTB	LTB.value <= threshold=0.6038667261600494
node_2522: feature_name=HLA-DQB1	HLA-DQB1.value > threshold=2.1053054332733154
node_3550: feature_name=XIST	XIST.value <= threshold=0.7716337237507105
node_3551: feature_name=CD79B	CD79B.value <= threshold=2.9426796436309814
node_3552: feature_name=HLA-DQB1	HLA-DQB1.value <= threshold=2.713674306869507
node_3553: feature_name=SNX9	SNX9.value <= threshold=-0.5404067635536194
node_3554: feature_name=VPREB3	VPREB3.value > threshold=4.4475414752960205
node_3646: feature_name=FCRL1	FCRL1.value <= threshold=-0.21100489795207977

node_3647: feature_name=CD79B	CD79B.value <= threshold=2.7485435009002686
node_3648: feature_name=RGS2	RGS2.value <= threshold=0.15276828408241272
Class: COVID-19 Critical	
Rules_478	passed counts:9
node_0: feature_name=LTB	LTB.value <= threshold=0.994031548500061
node_1: feature_name=MTRNR2L2	MTRNR2L2.value <= threshold=2.8784297704696655
node_2: feature_name=TCL1A	TCL1A.value <= threshold=4.798312664031982
node_3: feature_name=MT-CO1	MT-CO1.value > threshold=0.08947191759943962
node_2521: feature_name=LTB	LTB.value <= threshold=0.6038667261600494
node_2522: feature_name=HLA-DQB1	HLA-DQB1.value <= threshold=2.1053054332733154
node_2523: feature_name=SNX9	SNX9.value > threshold=-0.5346486568450928
node_2907: feature_name=NR4A1	NR4A1.value > threshold=2.5433837175369263
node_3459: feature_name=VPREB3	VPREB3.value <= threshold=4.410779237747192
node_3460: feature_name=RALGPS2	RALGPS2.value > threshold=3.064661979675293
node_3520: feature_name=RGS2	RGS2.value <= threshold=-0.5165578126907349
Class: Healthy Control	
Rules_479	passed counts:9
node_0: feature_name=LTB	LTB.value <= threshold=0.994031548500061
node_1: feature_name=MTRNR2L2	MTRNR2L2.value <= threshold=2.8784297704696655
node_2: feature_name=TCL1A	TCL1A.value <= threshold=4.798312664031982
node_3: feature_name=MT-CO1	MT-CO1.value > threshold=0.08947191759943962
node_2521: feature_name=LTB	LTB.value <= threshold=0.6038667261600494
node_2522: feature_name=HLA-DQB1	HLA-DQB1.value <= threshold=2.1053054332733154
node_2523: feature_name=SNX9	SNX9.value > threshold=-0.5346486568450928
node_2907: feature_name=NR4A1	NR4A1.value <= threshold=2.5433837175369263
node_2908: feature_name=XIST	XIST.value > threshold=0.7664470989257097
node_3440: feature_name=MT-CO1	MT-CO1.value > threshold=0.9336088299751282
node_3448: feature_name=RALGPS2	RALGPS2.value > threshold=3.274242401123047

Class: Healthy Control	
Rules_480	passed counts:9
node_0: feature_name=LTB	LTB.value <= threshold=0.994031548500061
node_1: feature_name=MTRNR2L2	MTRNR2L2.value <= threshold=2.8784297704696655
node_2: feature_name=TCL1A	TCL1A.value <= threshold=4.798312664031982
node_3: feature_name=MT-CO1	MT-CO1.value > threshold=0.08947191759943962
node_2521: feature_name=LTB	LTB.value <= threshold=0.6038667261600494
node_2522: feature_name=HLA-DQB1	HLA-DQB1.value <= threshold=2.1053054332733154
node_2523: feature_name=SNX9	SNX9.value > threshold=-0.5346486568450928
node_2907: feature_name=NR4A1	NR4A1.value <= threshold=2.5433837175369263
node_2908: feature_name=XIST	XIST.value <= threshold=0.7664470989257097
node_2909: feature_name=CD79B	CD79B.value <= threshold=2.9326847791671753
node_2910: feature_name=NR4A1	NR4A1.value > threshold=-0.2875557690858841
node_3096: feature_name=NR4A3	NR4A3.value <= threshold=3.314968705177307
node_3097: feature_name=VPREB3	VPREB3.value <= threshold=3.3549938201904297
node_3098: feature_name=RALGPS2	RALGPS2.value <= threshold=5.807050704956055
node_3099: feature_name=CTD-2035E11.3	CTD-2035E11.3.value <= threshold=6.4090416431427
node_3100: feature_name=FAM129C	FAM129C.value <= threshold=6.966361045837402
node_3101: feature_name=HES4	HES4.value <= threshold=4.139557242393494
node_3102: feature_name=CD83	CD83.value > threshold=1.8343153595924377
node_3136: feature_name=PLD4	PLD4.value > threshold=4.453821897506714
node_3264: feature_name=LTB	LTB.value <= threshold=0.12628396600484848
Class: COVID-19 Critical	
Rules_481	passed counts:9
node_0: feature_name=LTB	LTB.value <= threshold=0.994031548500061
node_1: feature_name=MTRNR2L2	MTRNR2L2.value <= threshold=2.8784297704696655
node_2: feature_name=TCL1A	TCL1A.value <= threshold=4.798312664031982
node_3: feature_name=MT-CO1	MT-CO1.value > threshold=0.08947191759943962

node_2521: feature_name=LTB	LTB.value <= threshold=0.6038667261600494
node_2522: feature_name=HLA-DQB1	HLA-DQB1.value <= threshold=2.1053054332733154
node_2523: feature_name=SNX9	SNX9.value > threshold=-0.5346486568450928
node_2907: feature_name=NR4A1	NR4A1.value <= threshold=2.5433837175369263
node_2908: feature_name=XIST	XIST.value <= threshold=0.7664470989257097
node_2909: feature_name=CD79B	CD79B.value <= threshold=2.9326847791671753
node_2910: feature_name=NR4A1	NR4A1.value > threshold=-0.2875557690858841
node_3096: feature_name=NR4A3	NR4A3.value <= threshold=3.314968705177307
node_3097: feature_name=VPREB3	VPREB3.value <= threshold=3.3549938201904297
node_3098: feature_name=RALGPS2	RALGPS2.value <= threshold=5.807050704956055
node_3099: feature_name=CTD-2035E11.3	CTD-2035E11.3.value <= threshold=6.4090416431427
node_3100: feature_name=FAM129C	FAM129C.value <= threshold=6.966361045837402
node_3101: feature_name=HES4	HES4.value <= threshold=4.139557242393494
node_3102: feature_name=CD83	CD83.value <= threshold=1.8343153595924377
node_3103: feature_name=FCER2	FCER2.value <= threshold=7.255846738815308
node_3104: feature_name=MT-CYB	MT-CYB.value <= threshold=2.3888577222824097
node_3105: feature_name=RGCC	RGCC.value > threshold=-1.2040565013885498
node_3107: feature_name=MCTP1	MCTP1.value <= threshold=6.221277952194214
node_3108: feature_name=JUN	JUN.value > threshold=-0.629857987165451
node_3120: feature_name=RGS2	RGS2.value <= threshold=-0.9065633714199066
node_3121: feature_name=RALGPS2	RALGPS2.value <= threshold=2.04490202665329
Class: COVID-19 Critical	
Rules_482	passed counts:9
node_0: feature_name=LTB	LTB.value <= threshold=0.994031548500061
node_1: feature_name=MTRNR2L2	MTRNR2L2.value <= threshold=2.8784297704696655
node_2: feature_name=TCL1A	TCL1A.value <= threshold=4.798312664031982
node_3: feature_name=MT-CO1	MT-CO1.value > threshold=0.08947191759943962
node_2521: feature_name=LTB	LTB.value <= threshold=0.6038667261600494
node_2522: feature_name=HLA-DQB1	HLA-DQB1.value <= threshold=2.1053054332733154

node_2523: feature_name=SNX9	SNX9.value > threshold=-0.5346486568450928
node_2907: feature_name=NR4A1	NR4A1.value <= threshold=2.5433837175369263
node_2908: feature_name=XIST	XIST.value <= threshold=0.7664470989257097
node_2909: feature_name=CD79B	CD79B.value <= threshold=2.9326847791671753
node_2910: feature_name=NR4A1	NR4A1.value <= threshold=-0.2875557690858841
node_2911: feature_name=CTD-2035E11.3	CTD-2035E11.3.value <= threshold=5.262048006057739
node_2912: feature_name=VPREB3	VPREB3.value <= threshold=4.937594890594482
node_2913: feature_name=IRAK3	IRAK3.value <= threshold=4.592883586883545
node_2914: feature_name=IGJ	IGJ.value <= threshold=7.093410015106201
node_2915: feature_name=WDR74	WDR74.value <= threshold=4.077916383743286
node_2916: feature_name=MEF2C	MEF2C.value <= threshold=4.374382972717285
node_2917: feature_name=HIST1H2AC	HIST1H2AC.value <= threshold=3.743621349334717
node_2918: feature_name=STMN1	STMN1.value <= threshold=6.266510248184204
node_2919: feature_name=RALGPS2	RALGPS2.value <= threshold=5.72194766998291
node_2920: feature_name=HOPX	HOPX.value <= threshold=2.5944807529449463
node_2921: feature_name=MARCKS	MARCKS.value <= threshold=4.622651815414429
node_2922: feature_name=CCL3	CCL3.value <= threshold=0.7484713792800903
node_2923: feature_name=TPM1	TPM1.value <= threshold=6.446193695068359
node_2924: feature_name=PLD4	PLD4.value <= threshold=8.038907289505005
node_2925: feature_name=COTL1	COTL1.value > threshold=-1.8123814463615417
node_2929: feature_name=ID3	ID3.value <= threshold=7.534881353378296
node_2930: feature_name=IRF8	IRF8.value <= threshold=4.855781078338623
node_2931: feature_name=AP3M2	AP3M2.value <= threshold=1.8868122100830078
node_2932: feature_name=FCER2	FCER2.value <= threshold=6.877448558807373
node_2933: feature_name=MTRNR2L2	MTRNR2L2.value <= threshold=2.710945725440979
node_2934: feature_name=GNG11	GNG11.value <= threshold=2.510925054550171
node_2935: feature_name=CD83	CD83.value <= threshold=1.9873459935188293
node_2936: feature_name=MARCH1	MARCH1.value <= threshold=5.006200551986694
node_2937: feature_name=MEF2C	MEF2C.value > threshold=-0.5498070418834686
node_2941: feature_name=FCRL1	FCRL1.value <= threshold=7.093972444534302

node_2942: feature_name=HSP90B1	HSP90B1.value <= threshold=-1.4724047780036926
node_2943: feature_name=ATF3	ATF3.value <= threshold=-0.6969725489616394
Class: COVID-19 Critical	
Rules_483	passed counts:9
node_0: feature_name=LTB	LTB.value <= threshold=0.994031548500061
node_1: feature_name=MTRNR2L2	MTRNR2L2.value <= threshold=2.8784297704696655
node_2: feature_name=TCL1A	TCL1A.value <= threshold=4.798312664031982
node_3: feature_name=MT-CO1	MT-CO1.value <= threshold=0.08947191759943962
node_4: feature_name=NR4A1	NR4A1.value > threshold=1.0725348591804504
node_1622: feature_name=HLA-DQA2	HLA-DQA2.value <= threshold=1.9496816992759705
node_1623: feature_name=HLA-DRB5	HLA-DRB5.value <= threshold=2.715766429901123
node_1624: feature_name=HLA-DPA1	HLA-DPA1.value > threshold=1.7050301432609558
node_2056: feature_name=OAZ1	OAZ1.value <= threshold=0.456103190779686
node_2057: feature_name=HLA-DRB5	HLA-DRB5.value > threshold=0.6839984953403473
node_2101: feature_name=HLA-DRB1	HLA-DRB1.value > threshold=1.6199676990509033
node_2151: feature_name=HLA-DQB1	HLA-DQB1.value <= threshold=3.1961567401885986
node_2152: feature_name=PLD4	PLD4.value > threshold=-0.03259922005236149
node_2244: feature_name=LTB	LTB.value > threshold=0.3717459514737129
node_2250: feature_name=SYNE1	SYNE1.value > threshold=-0.5891828238964081
node_2254: feature_name=AP3M2	AP3M2.value <= threshold=0.6148001402616501
Class: Healthy Control	
Rules_484	passed counts:9
node_0: feature_name=LTB	LTB.value <= threshold=0.994031548500061
node_1: feature_name=MTRNR2L2	MTRNR2L2.value <= threshold=2.8784297704696655
node_2: feature_name=TCL1A	TCL1A.value <= threshold=4.798312664031982
node_3: feature_name=MT-CO1	MT-CO1.value <= threshold=0.08947191759943962
node_4: feature_name=NR4A1	NR4A1.value > threshold=1.0725348591804504
node_1622: feature_name=HLA-DQA2	HLA-DQA2.value <= threshold=1.9496816992759705

node_1623: feature_name=HLA-DRB5	HLA-DRB5.value <= threshold=2.715766429901123
node_1624: feature_name=HLA-DPA1	HLA-DPA1.value <= threshold=1.7050301432609558
node_1625: feature_name=CD83	CD83.value > threshold=2.783489227294922
node_1873: feature_name=IFI44L	IFI44L.value <= threshold=1.5938722491264343
node_1874: feature_name=PLD4	PLD4.value > threshold=4.101001739501953
node_2028: feature_name=SNX9	SNX9.value > threshold=1.4460421800613403
node_2036: feature_name=ID1	ID1.value > threshold=-0.23599714040756226
node_2044: feature_name=MT-CO3	MT-CO3.value <= threshold=0.46381479501724243
Class: COVID-19 Severe	
Rules_485	passed counts:9
node_0: feature_name=LTB	LTB.value <= threshold=0.994031548500061
node_1: feature_name=MTRNR2L2	MTRNR2L2.value <= threshold=2.8784297704696655
node_2: feature_name=TCL1A	TCL1A.value <= threshold=4.798312664031982
node_3: feature_name=MT-CO1	MT-CO1.value <= threshold=0.08947191759943962
node_4: feature_name=NR4A1	NR4A1.value > threshold=1.0725348591804504
node_1622: feature_name=HLA-DQA2	HLA-DQA2.value <= threshold=1.9496816992759705
node_1623: feature_name=HLA-DRB5	HLA-DRB5.value <= threshold=2.715766429901123
node_1624: feature_name=HLA-DPA1	HLA-DPA1.value <= threshold=1.7050301432609558
node_1625: feature_name=CD83	CD83.value > threshold=2.783489227294922
node_1873: feature_name=IFI44L	IFI44L.value <= threshold=1.5938722491264343
node_1874: feature_name=PLD4	PLD4.value <= threshold=4.101001739501953
node_1875: feature_name=RHOB	RHOB.value > threshold=1.304835319519043
node_1965: feature_name=HLA-DQB1	HLA-DQB1.value > threshold=1.5989137291908264
node_1991: feature_name=COTL1	COTL1.value > threshold=-1.2334879636764526
node_1997: feature_name=HSP90B1	HSP90B1.value <= threshold=1.903715431690216
node_1998: feature_name=NFKBID	NFKBID.value <= threshold=1.8163637518882751
node_1999: feature_name=IFI6	IFI6.value <= threshold=-0.5193088352680206
node_2000: feature_name=LTB	LTB.value <= threshold=0.580909252166748
node_2001: feature_name=PELI1	PELI1.value <= threshold=0.9547452330589294

node_2002: feature_name=ATF3	ATF3.value > threshold=-0.4521287828683853
Class: COVID-19 Severe	
Rules_486	passed counts:9
node_0: feature_name=LTB	LTB.value <= threshold=0.994031548500061
node_1: feature_name=MTRNR2L2	MTRNR2L2.value <= threshold=2.8784297704696655
node_2: feature_name=TCL1A	TCL1A.value <= threshold=4.798312664031982
node_3: feature_name=MT-CO1	MT-CO1.value <= threshold=0.08947191759943962
node_4: feature_name=NR4A1	NR4A1.value > threshold=1.0725348591804504
node_1622: feature_name=HLA-DQA2	HLA-DQA2.value <= threshold=1.9496816992759705
node_1623: feature_name=HLA-DRB5	HLA-DRB5.value <= threshold=2.715766429901123
node_1624: feature_name=HLA-DPA1	HLA-DPA1.value <= threshold=1.7050301432609558
node_1625: feature_name=CD83	CD83.value > threshold=2.783489227294922
node_1873: feature_name=IFI44L	IFI44L.value <= threshold=1.5938722491264343
node_1874: feature_name=PLD4	PLD4.value <= threshold=4.101001739501953
node_1875: feature_name=RHOB	RHOB.value > threshold=1.304835319519043
node_1965: feature_name=HLA-DQB1	HLA-DQB1.value <= threshold=1.5989137291908264
node_1966: feature_name=CD79A	CD79A.value > threshold=2.189441442489624
node_1974: feature_name=IGJ	IGJ.value > threshold=1.4252705574035645
node_1984: feature_name=BACH1	BACH1.value > threshold=0.9873552322387695
Class: COVID-19 Severe	
Rules_487	passed counts:9
node_0: feature_name=LTB	LTB.value <= threshold=0.994031548500061
node_1: feature_name=MTRNR2L2	MTRNR2L2.value <= threshold=2.8784297704696655
node_2: feature_name=TCL1A	TCL1A.value <= threshold=4.798312664031982
node_3: feature_name=MT-CO1	MT-CO1.value <= threshold=0.08947191759943962
node_4: feature_name=NR4A1	NR4A1.value > threshold=1.0725348591804504
node_1622: feature_name=HLA-DQA2	HLA-DQA2.value <= threshold=1.9496816992759705
node_1623: feature_name=HLA-DRB5	HLA-DRB5.value <= threshold=2.715766429901123

node_1624: feature_name=HLA-DPA1	HLA-DPA1.value <= threshold=1.7050301432609558
node_1625: feature_name=CD83	CD83.value <= threshold=2.783489227294922
node_1626: feature_name=XIST	XIST.value <= threshold=0.9589543007314205
node_1627: feature_name=PLD4	PLD4.value <= threshold=5.865932464599609
node_1628: feature_name=OAZ1	OAZ1.value <= threshold=0.6137169003486633
node_1629: feature_name=MTRNR2L2	MTRNR2L2.value <= threshold=1.6102792620658875
node_1630: feature_name=IFI6	IFI6.value > threshold=-0.8404486179351807
node_1632: feature_name=NR4A1	NR4A1.value > threshold=1.0916320085525513
node_1638: feature_name=DENND3	DENND3.value <= threshold=3.988452672958374
node_1639: feature_name=HLA-DRB1	HLA-DRB1.value > threshold=1.4092575907707214
node_1749: feature_name=HLA-DQB1	HLA-DQB1.value <= threshold=3.4929215908050537
node_1750: feature_name=RP11-685N3.1	RP11-685N3.1.value <= threshold=9.97533893585205
node_1751: feature_name=RGS1	RGS1.value > threshold=-0.5999330878257751
node_1765: feature_name=MT-ND6	MT-ND6.value <= threshold=2.4838690757751465
node_1766: feature_name=DAPK1	DAPK1.value <= threshold=4.920811414718628
node_1767: feature_name=RGS1	RGS1.value <= threshold=3.3605728149414062
node_1768: feature_name=ANO6	ANO6.value <= threshold=4.00420081615448
node_1769: feature_name=FTH1	FTH1.value <= threshold=1.6050249934196472
node_1770: feature_name=APP	APP.value <= threshold=2.417694091796875
node_1771: feature_name=ITGAX	ITGAX.value <= threshold=2.950524091720581
node_1772: feature_name=PDLIM1	PDLIM1.value <= threshold=5.856243848800659
node_1773: feature_name=RELT	RELT.value <= threshold=2.891391396522522
node_1774: feature_name=RHOB	RHOB.value <= threshold=3.2315229177474976
node_1775: feature_name=NR4A1	NR4A1.value > threshold=1.125234842300415
node_1781: feature_name=LTB	LTB.value <= threshold=0.7502643764019012
node_1782: feature_name=HSP90B1	HSP90B1.value > threshold=1.9601801037788391
node_1784: feature_name=MAP3K2	MAP3K2.value > threshold=-0.4875212460756302
Class: COVID-19 Critical	
Rules_488	passed counts:9

node_0: feature_name=LTB	LTB.value <= threshold=0.994031548500061
node_1: feature_name=MTRNR2L2	MTRNR2L2.value <= threshold=2.8784297704696655
node_2: feature_name=TCL1A	TCL1A.value <= threshold=4.798312664031982
node_3: feature_name=MT-CO1	MT-CO1.value <= threshold=0.08947191759943962
node_4: feature_name=NR4A1	NR4A1.value <= threshold=1.0725348591804504
node_5: feature_name=HLA-DPA1	HLA-DPA1.value > threshold=1.6885469555854797
node_861: feature_name=CD83	CD83.value <= threshold=3.0563642978668213
node_862: feature_name=HLA-DQB1	HLA-DQB1.value <= threshold=2.61081063747406
node_863: feature_name=MTRNR2L2	MTRNR2L2.value <= threshold=2.0919997692108154
node_864: feature_name=BCL2A1	BCL2A1.value <= threshold=1.852618932723999
node_865: feature_name=CD79B	CD79B.value <= threshold=4.352142333984375
node_866: feature_name=TNFAIP3	TNFAIP3.value <= threshold=-0.0449496041983366
node_867: feature_name=MT-CO1	MT-CO1.value <= threshold=-0.08531957119703293
node_868: feature_name=MTRNR2L8	MTRNR2L8.value <= threshold=0.8770350813865662
node_869: feature_name=EGR1	EGR1.value > threshold=-0.32270750403404236
node_1015: feature_name=NR4A3	NR4A3.value <= threshold=1.9085431098937988
node_1016: feature_name=ID3	ID3.value > threshold=2.776647686958313
node_1128: feature_name=MARCKS	MARCKS.value > threshold=-0.29620493948459625
node_1136: feature_name=S100A6	S100A6.value <= threshold=-0.2672681510448456
node_1137: feature_name=FCER2	FCER2.value > threshold=-0.24340790510177612
Class: COVID-19 Severe	
Rules_489	passed counts:9
node_0: feature_name=LTB	LTB.value <= threshold=0.994031548500061
node_1: feature_name=MTRNR2L2	MTRNR2L2.value <= threshold=2.8784297704696655
node_2: feature_name=TCL1A	TCL1A.value <= threshold=4.798312664031982
node_3: feature_name=MT-CO1	MT-CO1.value <= threshold=0.08947191759943962
node_4: feature_name=NR4A1	NR4A1.value <= threshold=1.0725348591804504
node_5: feature_name=HLA-DPA1	HLA-DPA1.value > threshold=1.6885469555854797
node_861: feature_name=CD83	CD83.value <= threshold=3.0563642978668213

node_862: feature_name=HLA-DQB1	HLA-DQB1.value <= threshold=2.61081063747406
node_863: feature_name=MTRNR2L2	MTRNR2L2.value <= threshold=2.0919997692108154
node_864: feature_name=BCL2A1	BCL2A1.value <= threshold=1.852618932723999
node_865: feature_name=CD79B	CD79B.value <= threshold=4.352142333984375
node_866: feature_name=TNFAIP3	TNFAIP3.value <= threshold=-0.0449496041983366
node_867: feature_name=MT-CO1	MT-CO1.value <= threshold=-0.08531957119703293
node_868: feature_name=MTRNR2L8	MTRNR2L8.value <= threshold=0.8770350813865662
node_869: feature_name=EGR1	EGR1.value <= threshold=-0.32270750403404236
node_870: feature_name=IGJ	IGJ.value <= threshold=4.618318557739258
node_871: feature_name=DUSP4	DUSP4.value <= threshold=2.8197922706604004
node_872: feature_name=HOPX	HOPX.value <= threshold=2.10397732257843
node_873: feature_name=RGCC	RGCC.value <= threshold=1.2070720195770264
node_874: feature_name=OAZ1	OAZ1.value <= threshold=1.02116060256958
node_875: feature_name=NAIP	NAIP.value <= threshold=3.768904685974121
node_876: feature_name=LDLR	LDLR.value <= threshold=2.9582698345184326
node_877: feature_name=HLA-DPA1	HLA-DPA1.value <= threshold=3.0588693618774414
node_878: feature_name=RHOB	RHOB.value <= threshold=5.08755898475647
node_879: feature_name=SYTL3	SYTL3.value <= threshold=1.5127440690994263
node_880: feature_name=FTH1	FTH1.value > threshold=-1.4152275919914246
node_882: feature_name=CCL3	CCL3.value <= threshold=2.253131687641144
node_883: feature_name=CD83	CD83.value <= threshold=2.8566592931747437
node_884: feature_name=HLA-DRB5	HLA-DRB5.value > threshold=-0.6464254856109619
node_888: feature_name=HSP90B1	HSP90B1.value > threshold=-1.186358630657196
node_892: feature_name=ATG2A	ATG2A.value > threshold=2.3624722957611084
node_964: feature_name=IGJ	IGJ.value <= threshold=0.7024875283241272
Class: COVID-19 Critical	
Rules_490	passed counts:9
node_0: feature_name=LTB	LTB.value <= threshold=0.994031548500061
node_1: feature_name=MTRNR2L2	MTRNR2L2.value <= threshold=2.8784297704696655

node_2: feature_name=TCL1A	TCL1A.value <= threshold=4.798312664031982
node_3: feature_name=MT-CO1	MT-CO1.value <= threshold=0.08947191759943962
node_4: feature_name=NR4A1	NR4A1.value <= threshold=1.0725348591804504
node_5: feature_name=HLA-DPA1	HLA-DPA1.value > threshold=1.6885469555854797
node_861: feature_name=CD83	CD83.value <= threshold=3.0563642978668213
node_862: feature_name=HLA-DQB1	HLA-DQB1.value <= threshold=2.61081063747406
node_863: feature_name=MTRNR2L2	MTRNR2L2.value <= threshold=2.0919997692108154
node_864: feature_name=BCL2A1	BCL2A1.value <= threshold=1.852618932723999
node_865: feature_name=CD79B	CD79B.value <= threshold=4.352142333984375
node_866: feature_name=TNFAIP3	TNFAIP3.value <= threshold=-0.0449496041983366
node_867: feature_name=MT-CO1	MT-CO1.value <= threshold=-0.08531957119703293
node_868: feature_name=MTRNR2L8	MTRNR2L8.value <= threshold=0.8770350813865662
node_869: feature_name=EGR1	EGR1.value <= threshold=-0.32270750403404236
node_870: feature_name=IGJ	IGJ.value <= threshold=4.618318557739258
node_871: feature_name=DUSP4	DUSP4.value <= threshold=2.8197922706604004
node_872: feature_name=HOPX	HOPX.value <= threshold=2.10397732257843
node_873: feature_name=RGCC	RGCC.value <= threshold=1.2070720195770264
node_874: feature_name=OAZ1	OAZ1.value <= threshold=1.02116060256958
node_875: feature_name=NAIP	NAIP.value <= threshold=3.768904685974121
node_876: feature_name=LDLR	LDLR.value <= threshold=2.9582698345184326
node_877: feature_name=HLA-DPA1	HLA-DPA1.value <= threshold=3.0588693618774414
node_878: feature_name=RHOB	RHOB.value <= threshold=5.08755898475647
node_879: feature_name=SYTL3	SYTL3.value <= threshold=1.5127440690994263
node_880: feature_name=FTH1	FTH1.value > threshold=-1.4152275919914246
node_882: feature_name=CCL3	CCL3.value <= threshold=2.253131687641144
node_883: feature_name=CD83	CD83.value <= threshold=2.8566592931747437
node_884: feature_name=HLA-DRB5	HLA-DRB5.value > threshold=-0.6464254856109619
node_888: feature_name=HSP90B1	HSP90B1.value > threshold=-1.186358630657196
node_892: feature_name=ATG2A	ATG2A.value <= threshold=2.3624722957611084
node_893: feature_name=ICAM1	ICAM1.value > threshold=2.4306468963623047

node_961: feature_name=ICAM1	ICAM1.value > threshold=2.4940208196640015
Class: COVID-19 Critical	
Rules_491	passed counts:9
node_0: feature_name=LTB	LTB.value <= threshold=0.994031548500061
node_1: feature_name=MTRNR2L2	MTRNR2L2.value <= threshold=2.8784297704696655
node_2: feature_name=TCL1A	TCL1A.value <= threshold=4.798312664031982
node_3: feature_name=MT-CO1	MT-CO1.value <= threshold=0.08947191759943962
node_4: feature_name=NR4A1	NR4A1.value <= threshold=1.0725348591804504
node_5: feature_name=HLA-DPA1	HLA-DPA1.value <= threshold=1.6885469555854797
node_6: feature_name=HLA-DRB5	HLA-DRB5.value <= threshold=2.1886194944381714
node_7: feature_name=XIST	XIST.value <= threshold=-0.12328458577394485
node_8: feature_name=SYNE1	SYNE1.value > threshold=-0.7835594415664673
node_16: feature_name=MTRNR2L2	MTRNR2L2.value > threshold=2.4483247995376587
node_708: feature_name=ICAM1	ICAM1.value <= threshold=-0.25065042823553085
Class: COVID-19 Severe	
Rules_492	passed counts:9
node_0: feature_name=LTB	LTB.value <= threshold=0.994031548500061
node_1: feature_name=MTRNR2L2	MTRNR2L2.value <= threshold=2.8784297704696655
node_2: feature_name=TCL1A	TCL1A.value <= threshold=4.798312664031982
node_3: feature_name=MT-CO1	MT-CO1.value <= threshold=0.08947191759943962
node_4: feature_name=NR4A1	NR4A1.value <= threshold=1.0725348591804504
node_5: feature_name=HLA-DPA1	HLA-DPA1.value <= threshold=1.6885469555854797
node_6: feature_name=HLA-DRB5	HLA-DRB5.value <= threshold=2.1886194944381714
node_7: feature_name=XIST	XIST.value <= threshold=-0.12328458577394485
node_8: feature_name=SYNE1	SYNE1.value > threshold=-0.7835594415664673
node_16: feature_name=MTRNR2L2	MTRNR2L2.value <= threshold=2.4483247995376587
node_17: feature_name=HLA-DQA2	HLA-DQA2.value > threshold=2.227178931236267
node_591: feature_name=BCL2A1	BCL2A1.value <= threshold=1.0648428201675415

node_592: feature_name=FTH1	FTH1.value > threshold=0.8842596709728241
node_680: feature_name=PELI1	PELI1.value > threshold=0.20563310384750366
node_684: feature_name=MMD	MMD.value > threshold=-0.2975647747516632
Class: COVID-19 Severe	
Rules_493	passed counts:9
node_0: feature_name=LTB	LTB.value <= threshold=0.994031548500061
node_1: feature_name=MTRNR2L2	MTRNR2L2.value <= threshold=2.8784297704696655
node_2: feature_name=TCL1A	TCL1A.value <= threshold=4.798312664031982
node_3: feature_name=MT-CO1	MT-CO1.value <= threshold=0.08947191759943962
node_4: feature_name=NR4A1	NR4A1.value <= threshold=1.0725348591804504
node_5: feature_name=HLA-DPA1	HLA-DPA1.value <= threshold=1.6885469555854797
node_6: feature_name=HLA-DRB5	HLA-DRB5.value <= threshold=2.1886194944381714
node_7: feature_name=XIST	XIST.value <= threshold=-0.12328458577394485
node_8: feature_name=SYNE1	SYNE1.value > threshold=-0.7835594415664673
node_16: feature_name=MTRNR2L2	MTRNR2L2.value <= threshold=2.4483247995376587
node_17: feature_name=HLA-DQA2	HLA-DQA2.value > threshold=2.227178931236267
node_591: feature_name=BCL2A1	BCL2A1.value <= threshold=1.0648428201675415
node_592: feature_name=FTH1	FTH1.value <= threshold=0.8842596709728241
node_593: feature_name=SNX9	SNX9.value > threshold=3.232524275779724
node_671: feature_name=IRF4	IRF4.value > threshold=-0.20697960257530212
node_677: feature_name=MAP3K2	MAP3K2.value <= threshold=0.689010888338089
Class: COVID-19 Critical	
Rules_494	passed counts:9
node_0: feature_name=LTB	LTB.value <= threshold=0.994031548500061
node_1: feature_name=MTRNR2L2	MTRNR2L2.value <= threshold=2.8784297704696655
node_2: feature_name=TCL1A	TCL1A.value <= threshold=4.798312664031982
node_3: feature_name=MT-CO1	MT-CO1.value <= threshold=0.08947191759943962
node_4: feature_name=NR4A1	NR4A1.value <= threshold=1.0725348591804504

node_5: feature_name=HLA-DPA1	HLA-DPA1.value <= threshold=1.6885469555854797
node_6: feature_name=HLA-DRB5	HLA-DRB5.value <= threshold=2.1886194944381714
node_7: feature_name=XIST	XIST.value <= threshold=-0.12328458577394485
node_8: feature_name=SYNE1	SYNE1.value > threshold=-0.7835594415664673
node_16: feature_name=MTRNR2L2	MTRNR2L2.value <= threshold=2.4483247995376587
node_17: feature_name=HLA-DQA2	HLA-DQA2.value <= threshold=2.227178931236267
node_18: feature_name=NR4A1	NR4A1.value <= threshold=-0.21380723267793655
node_19: feature_name=IRAK3	IRAK3.value <= threshold=4.27159571647644
node_20: feature_name=IFIT2	IFIT2.value <= threshold=7.8175740242004395
node_21: feature_name=CD79A	CD79A.value > threshold=2.857242226600647
node_375: feature_name=LTB	LTB.value > threshold=0.7405790388584137
node_473: feature_name=MS4A1	MS4A1.value > threshold=2.8195884227752686
node_489: feature_name=FCER2	FCER2.value > threshold=-0.2379418984055519
node_495: feature_name=BANK1	BANK1.value > threshold=2.0636932253837585
node_497: feature_name=PLD4	PLD4.value > threshold=-0.15754935890436172
Class: Healthy Control	
Rules_495	passed counts:9
node_0: feature_name=LTB	LTB.value <= threshold=0.994031548500061
node_1: feature_name=MTRNR2L2	MTRNR2L2.value <= threshold=2.8784297704696655
node_2: feature_name=TCL1A	TCL1A.value <= threshold=4.798312664031982
node_3: feature_name=MT-CO1	MT-CO1.value <= threshold=0.08947191759943962
node_4: feature_name=NR4A1	NR4A1.value <= threshold=1.0725348591804504
node_5: feature_name=HLA-DPA1	HLA-DPA1.value <= threshold=1.6885469555854797
node_6: feature_name=HLA-DRB5	HLA-DRB5.value <= threshold=2.1886194944381714
node_7: feature_name=XIST	XIST.value <= threshold=-0.12328458577394485
node_8: feature_name=SYNE1	SYNE1.value > threshold=-0.7835594415664673
node_16: feature_name=MTRNR2L2	MTRNR2L2.value <= threshold=2.4483247995376587
node_17: feature_name=HLA-DQA2	HLA-DQA2.value <= threshold=2.227178931236267
node_18: feature_name=NR4A1	NR4A1.value <= threshold=-0.21380723267793655

node_19: feature_name=IRAK3	IRAK3.value <= threshold=4.27159571647644
node_20: feature_name=IFIT2	IFIT2.value <= threshold=7.8175740242004395
node_21: feature_name=CD79A	CD79A.value <= threshold=2.857242226600647
node_22: feature_name=HIST1H2AC	HIST1H2AC.value <= threshold=5.077470302581787
node_23: feature_name=FTH1	FTH1.value > threshold=0.4158056080341339
node_285: feature_name=EGR1	EGR1.value > threshold=4.240467071533203
node_363: feature_name=NFKBIZ	NFKBIZ.value > threshold=0.4797569662332535
node_367: feature_name=TRIB1	TRIB1.value <= threshold=-0.206153504550457
Class: COVID-19 Critical	
Rules_496	passed counts:9
node_0: feature_name=LTB	LTB.value <= threshold=0.994031548500061
node_1: feature_name=MTRNR2L2	MTRNR2L2.value <= threshold=2.8784297704696655
node_2: feature_name=TCL1A	TCL1A.value <= threshold=4.798312664031982
node_3: feature_name=MT-CO1	MT-CO1.value <= threshold=0.08947191759943962
node_4: feature_name=NR4A1	NR4A1.value <= threshold=1.0725348591804504
node_5: feature_name=HLA-DPA1	HLA-DPA1.value <= threshold=1.6885469555854797
node_6: feature_name=HLA-DRB5	HLA-DRB5.value <= threshold=2.1886194944381714
node_7: feature_name=XIST	XIST.value <= threshold=-0.12328458577394485
node_8: feature_name=SYNE1	SYNE1.value > threshold=-0.7835594415664673
node_16: feature_name=MTRNR2L2	MTRNR2L2.value <= threshold=2.4483247995376587
node_17: feature_name=HLA-DQA2	HLA-DQA2.value <= threshold=2.227178931236267
node_18: feature_name=NR4A1	NR4A1.value <= threshold=-0.21380723267793655
node_19: feature_name=IRAK3	IRAK3.value <= threshold=4.27159571647644
node_20: feature_name=IFIT2	IFIT2.value <= threshold=7.8175740242004395
node_21: feature_name=CD79A	CD79A.value <= threshold=2.857242226600647
node_22: feature_name=HIST1H2AC	HIST1H2AC.value <= threshold=5.077470302581787
node_23: feature_name=FTH1	FTH1.value <= threshold=0.4158056080341339
node_24: feature_name=ATF3	ATF3.value <= threshold=3.8028353452682495
node_25: feature_name=OASL	OASL.value <= threshold=3.387580633163452

node_26: feature_name=MT-CO1	MT-CO1.value > threshold=-0.7942646145820618
node_74: feature_name=HOPX	HOPX.value <= threshold=0.8682331740856171
node_75: feature_name=RGS2	RGS2.value <= threshold=1.4589954018592834
node_76: feature_name=AHR	AHR.value <= threshold=3.870481848716736
node_77: feature_name=IGJ	IGJ.value <= threshold=4.902882814407349
node_78: feature_name=CD79B	CD79B.value <= threshold=4.87716269493103
node_79: feature_name=HLA-DQB1	HLA-DQB1.value <= threshold=3.6925491094589233
node_80: feature_name=HLA-DRB5	HLA-DRB5.value <= threshold=2.0926843881607056
node_81: feature_name=MCTP1	MCTP1.value <= threshold=4.010472774505615
node_82: feature_name=GNG11	GNG11.value <= threshold=3.627470850944519
node_83: feature_name=HSP90B1	HSP90B1.value <= threshold=2.0835131406784058
node_84: feature_name=ATF3	ATF3.value <= threshold=1.1142374873161316
node_85: feature_name=MT-ND6	MT-ND6.value <= threshold=2.7328014373779297
node_86: feature_name=COTL1	COTL1.value > threshold=-1.6524587869644165
node_90: feature_name=MT-CYB	MT-CYB.value > threshold=1.099629819393158
node_172: feature_name=CCL3	CCL3.value > threshold=-0.36862824857234955
Class: COVID-19 Critical	
Rules_497	passed counts:9
node_0: feature_name=LTB	LTB.value <= threshold=0.994031548500061
node_1: feature_name=MTRNR2L2	MTRNR2L2.value <= threshold=2.8784297704696655
node_2: feature_name=TCL1A	TCL1A.value <= threshold=4.798312664031982
node_3: feature_name=MT-CO1	MT-CO1.value <= threshold=0.08947191759943962
node_4: feature_name=NR4A1	NR4A1.value <= threshold=1.0725348591804504
node_5: feature_name=HLA-DPA1	HLA-DPA1.value <= threshold=1.6885469555854797
node_6: feature_name=HLA-DRB5	HLA-DRB5.value <= threshold=2.1886194944381714
node_7: feature_name=XIST	XIST.value <= threshold=-0.12328458577394485
node_8: feature_name=SYNE1	SYNE1.value > threshold=-0.7835594415664673
node_16: feature_name=MTRNR2L2	MTRNR2L2.value <= threshold=2.4483247995376587
node_17: feature_name=HLA-DQA2	HLA-DQA2.value <= threshold=2.227178931236267

node_18: feature_name=NR4A1	NR4A1.value <= threshold=-0.21380723267793655
node_19: feature_name=IRAK3	IRAK3.value <= threshold=4.27159571647644
node_20: feature_name=IFIT2	IFIT2.value <= threshold=7.8175740242004395
node_21: feature_name=CD79A	CD79A.value <= threshold=2.857242226600647
node_22: feature_name=HIST1H2AC	HIST1H2AC.value <= threshold=5.077470302581787
node_23: feature_name=FTH1	FTH1.value <= threshold=0.4158056080341339
node_24: feature_name=ATF3	ATF3.value <= threshold=3.8028353452682495
node_25: feature_name=OASL	OASL.value <= threshold=3.387580633163452
node_26: feature_name=MT-CO1	MT-CO1.value > threshold=-0.7942646145820618
node_74: feature_name=HOPX	HOPX.value <= threshold=0.8682331740856171
node_75: feature_name=RGS2	RGS2.value <= threshold=1.4589954018592834
node_76: feature_name=AHR	AHR.value <= threshold=3.870481848716736
node_77: feature_name=IGJ	IGJ.value <= threshold=4.902882814407349
node_78: feature_name=CD79B	CD79B.value <= threshold=4.87716269493103
node_79: feature_name=HLA-DQB1	HLA-DQB1.value <= threshold=3.6925491094589233
node_80: feature_name=HLA-DRB5	HLA-DRB5.value <= threshold=2.0926843881607056
node_81: feature_name=MCTP1	MCTP1.value <= threshold=4.010472774505615
node_82: feature_name=GNG11	GNG11.value <= threshold=3.627470850944519
node_83: feature_name=HSP90B1	HSP90B1.value <= threshold=2.0835131406784058
node_84: feature_name=ATF3	ATF3.value <= threshold=1.1142374873161316
node_85: feature_name=MT-ND6	MT-ND6.value <= threshold=2.7328014373779297
node_86: feature_name=COTL1	COTL1.value > threshold=-1.6524587869644165
node_90: feature_name=MT-CYB	MT-CYB.value <= threshold=1.099629819393158
node_91: feature_name=RGCC	RGCC.value <= threshold=1.6908138990402222
node_92: feature_name=ILK	ILK.value <= threshold=3.9497616291046143
node_93: feature_name=CTD-2035E11.3	CTD-2035E11.3.value <= threshold=2.4543306827545166
node_94: feature_name=S100A6	S100A6.value <= threshold=0.2867250442504883
node_95: feature_name=HLA-DRB5	HLA-DRB5.value > threshold=-0.9496813416481018
node_99: feature_name=MT-CO3	MT-CO3.value > threshold=-1.55654776096344
node_103: feature_name=ITGAX	ITGAX.value <= threshold=1.8544010519981384

node_104: feature_name=MEF2C	MEF2C.value <= threshold=4.22724461555481
node_105: feature_name=CXorf57	CXorf57.value <= threshold=5.114444732666016
node_106: feature_name=IRAK3	IRAK3.value <= threshold=3.1017903089523315
node_107: feature_name=DUSP4	DUSP4.value <= threshold=2.9122174978256226
node_108: feature_name=OAZ1	OAZ1.value > threshold=-1.8057305812835693
node_112: feature_name=DAPP1	DAPP1.value <= threshold=4.016228437423706
node_113: feature_name=HSP90B1	HSP90B1.value > threshold=-1.2981283068656921
node_119: feature_name=MT-CYB	MT-CYB.value > threshold=0.9500376582145691
node_129: feature_name=PLEC	PLEC.value <= threshold=1.193070411682129
Class: COVID-19 Critical	
Rules_498	passed counts:8
node_0: feature_name=LTB	LTB.value > threshold=0.994031548500061
node_5264: feature_name=MT-CO1	MT-CO1.value > threshold=-0.4127991199493408
node_5638: feature_name=OAZ1	OAZ1.value > threshold=-0.2640867084264755
node_6112: feature_name=IFI44L	IFI44L.value <= threshold=2.5893263816833496
node_6113: feature_name=TCL1A	TCL1A.value > threshold=4.201832294464111
node_6991: feature_name=XAF1	XAF1.value <= threshold=3.4585667848587036
node_6992: feature_name=STAG3	STAG3.value <= threshold=7.686291694641113
node_6993: feature_name=RALGPS2	RALGPS2.value > threshold=-0.4090157449245453
node_6997: feature_name=MTRNR2L2	MTRNR2L2.value <= threshold=3.23723828792572
node_6998: feature_name=TP53INP1	TP53INP1.value <= threshold=6.239925146102905
node_6999: feature_name=MEF2C	MEF2C.value > threshold=-0.44642284512519836
node_7003: feature_name=HSPA1A	HSPA1A.value <= threshold=7.404359817504883
node_7004: feature_name=COTL1	COTL1.value > threshold=-1.6598749160766602
node_7006: feature_name=HIST1H2AC	HIST1H2AC.value > threshold=-0.34910088777542114
node_7008: feature_name=MARCH1	MARCH1.value <= threshold=5.393903970718384
node_7009: feature_name=PELI1	PELI1.value <= threshold=3.565749168395996
node_7010: feature_name=RGS1	RGS1.value <= threshold=2.851950168609619
node_7011: feature_name=HLA-DRB5	HLA-DRB5.value <= threshold=3.0528379678726196

node_7012: feature_name=MEF2A	MEF2A.value <= threshold=3.228689193725586
node_7013: feature_name=MT-CO1	MT-CO1.value > threshold=-0.37944722175598145
node_7017: feature_name=ATG2A	ATG2A.value <= threshold=2.790610194206238
node_7018: feature_name=MEF2C	MEF2C.value > threshold=-0.404978945851326
node_7026: feature_name=MT-CO3	MT-CO3.value > threshold=-1.699916422367096
node_7030: feature_name=CD79A	CD79A.value > threshold=0.17886222898960114
node_7034: feature_name=CCDC141	CCDC141.value <= threshold=5.498055934906006
node_7035: feature_name=SNX9	SNX9.value > threshold=2.924591064453125
node_7045: feature_name=GPX1	GPX1.value <= threshold=0.16466258466243744
Class: Healthy Control	
Rules_499	passed counts:8
node_0: feature_name=LTB	LTB.value > threshold=0.994031548500061
node_5264: feature_name=MT-CO1	MT-CO1.value > threshold=-0.4127991199493408
node_5638: feature_name=OAZ1	OAZ1.value > threshold=-0.2640867084264755
node_6112: feature_name=IFI44L	IFI44L.value <= threshold=2.5893263816833496
node_6113: feature_name=TCL1A	TCL1A.value > threshold=4.201832294464111
node_6991: feature_name=XAF1	XAF1.value <= threshold=3.4585667848587036
node_6992: feature_name=STAG3	STAG3.value <= threshold=7.686291694641113
node_6993: feature_name=RALGPS2	RALGPS2.value > threshold=-0.4090157449245453
node_6997: feature_name=MTRNR2L2	MTRNR2L2.value <= threshold=3.23723828792572
node_6998: feature_name=TP53INP1	TP53INP1.value <= threshold=6.239925146102905
node_6999: feature_name=MEF2C	MEF2C.value > threshold=-0.44642284512519836
node_7003: feature_name=HSPA1A	HSPA1A.value <= threshold=7.404359817504883
node_7004: feature_name=COTL1	COTL1.value > threshold=-1.6598749160766602
node_7006: feature_name=HIST1H2AC	HIST1H2AC.value > threshold=-0.34910088777542114
node_7008: feature_name=MARCH1	MARCH1.value <= threshold=5.393903970718384
node_7009: feature_name=PELI1	PELI1.value <= threshold=3.565749168395996
node_7010: feature_name=RGS1	RGS1.value <= threshold=2.851950168609619
node_7011: feature_name=HLA-DRB5	HLA-DRB5.value <= threshold=3.0528379678726196

node_7012: feature_name=MEF2A	MEF2A.value <= threshold=3.228689193725586
node_7013: feature_name=MT-CO1	MT-CO1.value > threshold=-0.37944722175598145
node_7017: feature_name=ATG2A	ATG2A.value <= threshold=2.790610194206238
node_7018: feature_name=MEF2C	MEF2C.value > threshold=-0.404978945851326
node_7026: feature_name=MT-CO3	MT-CO3.value > threshold=-1.699916422367096
node_7030: feature_name=CD79A	CD79A.value > threshold=0.17886222898960114
node_7034: feature_name=CCDC141	CCDC141.value <= threshold=5.498055934906006
node_7035: feature_name=SNX9	SNX9.value <= threshold=2.924591064453125
node_7036: feature_name=RP5-821D11.7	RP5-821D11.7.value <= threshold=4.530828952789307
node_7037: feature_name=IRAK3	IRAK3.value > threshold=3.0760087966918945
node_7039: feature_name=HBB	HBB.value <= threshold=1.5530832409858704
Class: Healthy Control	
Rules_500	passed counts:8
node_0: feature_name=LTB	LTB.value > threshold=0.994031548500061
node_5264: feature_name=MT-CO1	MT-CO1.value > threshold=-0.4127991199493408
node_5638: feature_name=OAZ1	OAZ1.value > threshold=-0.2640867084264755
node_6112: feature_name=IFI44L	IFI44L.value <= threshold=2.5893263816833496
node_6113: feature_name=TCL1A	TCL1A.value <= threshold=4.201832294464111
node_6114: feature_name=HLA-DQA2	HLA-DQA2.value > threshold=-0.2270243540406227
node_6542: feature_name=CD79B	CD79B.value > threshold=3.096232771873474
node_6872: feature_name=ICAM1	ICAM1.value <= threshold=-0.2232399433851242
node_6873: feature_name=STAG3	STAG3.value > threshold=-0.19312061369419098
node_6939: feature_name=HLA-DRB5	HLA-DRB5.value <= threshold=2.209904909133911
node_6940: feature_name=STAG3	STAG3.value > threshold=7.8730878829956055
node_6962: feature_name=HLA-DQA2	HLA-DQA2.value <= threshold=1.1327991858124733
Class: COVID-19 Critical	
Rules_501	passed counts:8
node_0: feature_name=LTB	LTB.value > threshold=0.994031548500061

node_5264: feature_name=MT-CO1	MT-CO1.value > threshold=-0.4127991199493408
node_5638: feature_name=OAZ1	OAZ1.value > threshold=-0.2640867084264755
node_6112: feature_name=IFI44L	IFI44L.value <= threshold=2.5893263816833496
node_6113: feature_name=TCL1A	TCL1A.value <= threshold=4.201832294464111
node_6114: feature_name=HLA-DQA2	HLA-DQA2.value > threshold=-0.2270243540406227
node_6542: feature_name=CD79B	CD79B.value <= threshold=3.096232771873474
node_6543: feature_name=NR4A1	NR4A1.value <= threshold=1.421015739440918
node_6544: feature_name=LTB	LTB.value > threshold=1.359755277633667
node_6720: feature_name=NFKBIZ	NFKBIZ.value > threshold=0.2720414251089096
node_6794: feature_name=PELI1	PELI1.value <= threshold=1.5861127376556396
node_6795: feature_name=LTB	LTB.value <= threshold=1.5073508024215698
node_6796: feature_name=MEF2A	MEF2A.value <= threshold=2.0854281783103943
node_6797: feature_name=FAM129C	FAM129C.value <= threshold=3.7562291622161865
Class: COVID-19 Critical	
Rules_502	passed counts:8
node_0: feature_name=LTB	LTB.value > threshold=0.994031548500061
node_5264: feature_name=MT-CO1	MT-CO1.value > threshold=-0.4127991199493408
node_5638: feature_name=OAZ1	OAZ1.value > threshold=-0.2640867084264755
node_6112: feature_name=IFI44L	IFI44L.value <= threshold=2.5893263816833496
node_6113: feature_name=TCL1A	TCL1A.value <= threshold=4.201832294464111
node_6114: feature_name=HLA-DQA2	HLA-DQA2.value > threshold=-0.2270243540406227
node_6542: feature_name=CD79B	CD79B.value <= threshold=3.096232771873474
node_6543: feature_name=NR4A1	NR4A1.value <= threshold=1.421015739440918
node_6544: feature_name=LTB	LTB.value > threshold=1.359755277633667
node_6720: feature_name=NFKBIZ	NFKBIZ.value <= threshold=0.2720414251089096
node_6721: feature_name=ATF5	ATF5.value <= threshold=2.4672781229019165
node_6722: feature_name=SNX9	SNX9.value <= threshold=2.0814030170440674
node_6723: feature_name=HLA-DQA2	HLA-DQA2.value <= threshold=4.360614776611328
node_6724: feature_name=MT-CO1	MT-CO1.value > threshold=-0.12188276648521423

node_6736: feature_name=MT1E	MT1E.value <= threshold=-0.1352209895849228
node_6737: feature_name=PELI1	PELI1.value <= threshold=3.1761642694473267
node_6738: feature_name=TRAF1	TRAF1.value <= threshold=-0.2332744374871254
node_6739: feature_name=HLA-DQA2	HLA-DQA2.value > threshold=-0.22670809924602509
node_6741: feature_name=HLA-DRB5	HLA-DRB5.value > threshold=-0.7810345888137817
node_6745: feature_name=GPX1	GPX1.value <= threshold=0.5079085677862167
node_6746: feature_name=MMD	MMD.value > threshold=2.110599994659424
node_6752: feature_name=UTY	UTY.value <= threshold=-0.40934064984321594
Class: Healthy Control	
Rules_503	passed counts:8
node_0: feature_name=LTB	LTB.value > threshold=0.994031548500061
node_5264: feature_name=MT-CO1	MT-CO1.value > threshold=-0.4127991199493408
node_5638: feature_name=OAZ1	OAZ1.value > threshold=-0.2640867084264755
node_6112: feature_name=IFI44L	IFI44L.value <= threshold=2.5893263816833496
node_6113: feature_name=TCL1A	TCL1A.value <= threshold=4.201832294464111
node_6114: feature_name=HLA-DQA2	HLA-DQA2.value > threshold=-0.2270243540406227
node_6542: feature_name=CD79B	CD79B.value <= threshold=3.096232771873474
node_6543: feature_name=NR4A1	NR4A1.value <= threshold=1.421015739440918
node_6544: feature_name=LTB	LTB.value <= threshold=1.359755277633667
node_6545: feature_name=NFKBID	NFKBID.value <= threshold=1.4294711351394653
node_6546: feature_name=HLA-DRB1	HLA-DRB1.value > threshold=1.0398632287979126
node_6556: feature_name=CD79B	CD79B.value <= threshold=-0.2919560521841049
node_6557: feature_name=MTRNR2L2	MTRNR2L2.value <= threshold=2.7710195183753967
node_6558: feature_name=JUN	JUN.value > threshold=0.5767147243022919
node_6574: feature_name=MT-ND6	MT-ND6.value <= threshold=0.21166645362973213
node_6575: feature_name=CD83	CD83.value <= threshold=2.2719725370407104
Class: COVID-19 Critical	
Rules_504	passed counts:8

node_0: feature_name=LTB	LTB.value > threshold=0.994031548500061
node_5264: feature_name=MT-CO1	MT-CO1.value > threshold=-0.4127991199493408
node_5638: feature_name=OAZ1	OAZ1.value > threshold=-0.2640867084264755
node_6112: feature_name=IFI44L	IFI44L.value <= threshold=2.5893263816833496
node_6113: feature_name=TCL1A	TCL1A.value <= threshold=4.201832294464111
node_6114: feature_name=HLA-DQA2	HLA-DQA2.value <= threshold=-0.2270243540406227
node_6115: feature_name=MTRNR2L2	MTRNR2L2.value <= threshold=2.548917293548584
node_6116: feature_name=ATF5	ATF5.value <= threshold=2.041263461112976
node_6117: feature_name=JUN	JUN.value > threshold=-0.616259902715683
node_6189: feature_name=MT-CO3	MT-CO3.value > threshold=0.13483521342277527
node_6351: feature_name=XAF1	XAF1.value <= threshold=3.061717987060547
node_6352: feature_name=MT-CO2	MT-CO2.value > threshold=1.2605181336402893
node_6476: feature_name=ATF5	ATF5.value > threshold=-0.3569986820220947
node_6482: feature_name=CYBB	CYBB.value <= threshold=-0.4226558953523636
node_6483: feature_name=IFI44L	IFI44L.value <= threshold=0.367323100566864
Class: COVID-19 Critical	
Rules_505	passed counts:8
node_0: feature_name=LTB	LTB.value > threshold=0.994031548500061
node_5264: feature_name=MT-CO1	MT-CO1.value > threshold=-0.4127991199493408
node_5638: feature_name=OAZ1	OAZ1.value > threshold=-0.2640867084264755
node_6112: feature_name=IFI44L	IFI44L.value <= threshold=2.5893263816833496
node_6113: feature_name=TCL1A	TCL1A.value <= threshold=4.201832294464111
node_6114: feature_name=HLA-DQA2	HLA-DQA2.value <= threshold=-0.2270243540406227
node_6115: feature_name=MTRNR2L2	MTRNR2L2.value <= threshold=2.548917293548584
node_6116: feature_name=ATF5	ATF5.value <= threshold=2.041263461112976
node_6117: feature_name=JUN	JUN.value > threshold=-0.616259902715683
node_6189: feature_name=MT-CO3	MT-CO3.value > threshold=0.13483521342277527
node_6351: feature_name=XAF1	XAF1.value <= threshold=3.061717987060547
node_6352: feature_name=MT-CO2	MT-CO2.value <= threshold=1.2605181336402893

node_6353: feature_name=ICAM1	ICAM1.value <= threshold=-0.3340446650981903
node_6354: feature_name=OAZ1	OAZ1.value <= threshold=-0.24055946618318558
node_6355: feature_name=MEF2A	MEF2A.value <= threshold=1.281972885131836
node_6356: feature_name=TP53INP1	TP53INP1.value <= threshold=3.1166218519210815
Class: Healthy Control	
Rules_506	passed counts:8
node_0: feature_name=LTB	LTB.value > threshold=0.994031548500061
node_5264: feature_name=MT-CO1	MT-CO1.value > threshold=-0.4127991199493408
node_5638: feature_name=OAZ1	OAZ1.value > threshold=-0.2640867084264755
node_6112: feature_name=IFI44L	IFI44L.value <= threshold=2.5893263816833496
node_6113: feature_name=TCL1A	TCL1A.value <= threshold=4.201832294464111
node_6114: feature_name=HLA-DQA2	HLA-DQA2.value <= threshold=-0.2270243540406227
node_6115: feature_name=MTRNR2L2	MTRNR2L2.value <= threshold=2.548917293548584
node_6116: feature_name=ATF5	ATF5.value <= threshold=2.041263461112976
node_6117: feature_name=JUN	JUN.value > threshold=-0.616259902715683
node_6189: feature_name=MT-CO3	MT-CO3.value <= threshold=0.13483521342277527
node_6190: feature_name=XAF1	XAF1.value <= threshold=2.584952235221863
node_6191: feature_name=HLA-DRB5	HLA-DRB5.value > threshold=0.6955901980400085
node_6239: feature_name=TNFAIP3	TNFAIP3.value > threshold=0.11930759996175766
node_6341: feature_name=PELI1	PELI1.value > threshold=-0.6198236048221588
Class: COVID-19 Critical	
Rules_507	passed counts:8
node_0: feature_name=LTB	LTB.value > threshold=0.994031548500061
node_5264: feature_name=MT-CO1	MT-CO1.value > threshold=-0.4127991199493408
node_5638: feature_name=OAZ1	OAZ1.value > threshold=-0.2640867084264755
node_6112: feature_name=IFI44L	IFI44L.value <= threshold=2.5893263816833496
node_6113: feature_name=TCL1A	TCL1A.value <= threshold=4.201832294464111
node_6114: feature_name=HLA-DQA2	HLA-DQA2.value <= threshold=-0.2270243540406227

node_6115: feature_name=MTRNR2L2	MTRNR2L2.value <= threshold=2.548917293548584
node_6116: feature_name=ATF5	ATF5.value <= threshold=2.041263461112976
node_6117: feature_name=JUN	JUN.value > threshold=-0.616259902715683
node_6189: feature_name=MT-CO3	MT-CO3.value <= threshold=0.13483521342277527
node_6190: feature_name=XAF1	XAF1.value <= threshold=2.584952235221863
node_6191: feature_name=HLA-DRB5	HLA-DRB5.value > threshold=0.6955901980400085
node_6239: feature_name=TNFAIP3	TNFAIP3.value <= threshold=0.11930759996175766
node_6240: feature_name=TP53INP1	TP53INP1.value <= threshold=2.9654784202575684
node_6241: feature_name=HLA-DRB5	HLA-DRB5.value <= threshold=2.5141202211380005
node_6242: feature_name=NFKBID	NFKBID.value <= threshold=3.358878970146179
node_6243: feature_name=JARID2	JARID2.value <= threshold=1.7160060405731201
node_6244: feature_name=HLA-DPA1	HLA-DPA1.value > threshold=0.49491266906261444
node_6246: feature_name=MIR24-2	MIR24-2.value <= threshold=1.8348549008369446
node_6247: feature_name=HSP90B1	HSP90B1.value <= threshold=1.8861054182052612
node_6248: feature_name=NR4A1	NR4A1.value <= threshold=-0.5434737503528595
node_6249: feature_name=MT-CO1	MT-CO1.value <= threshold=0.002460678806528449
node_6250: feature_name=FTH1	FTH1.value > threshold=-0.5194586217403412
node_6254: feature_name=PDLIM1	PDLIM1.value <= threshold=2.35056471824646
node_6255: feature_name=NFKBIZ	NFKBIZ.value > threshold=-0.8181745707988739
Class: COVID-19 Critical	
Rules_508	passed counts:8
node_0: feature_name=LTB	LTB.value > threshold=0.994031548500061
node_5264: feature_name=MT-CO1	MT-CO1.value > threshold=-0.4127991199493408
node_5638: feature_name=OAZ1	OAZ1.value > threshold=-0.2640867084264755
node_6112: feature_name=IFI44L	IFI44L.value <= threshold=2.5893263816833496
node_6113: feature_name=TCL1A	TCL1A.value <= threshold=4.201832294464111
node_6114: feature_name=HLA-DQA2	HLA-DQA2.value <= threshold=-0.2270243540406227
node_6115: feature_name=MTRNR2L2	MTRNR2L2.value <= threshold=2.548917293548584
node_6116: feature_name=ATF5	ATF5.value <= threshold=2.041263461112976

node_6117: feature_name=JUN	JUN.value <= threshold=-0.616259902715683
node_6118: feature_name=NFKBID	NFKBID.value > threshold=1.5264258980751038
node_6166: feature_name=NR4A1	NR4A1.value <= threshold=0.6553736329078674
node_6167: feature_name=WDR74	WDR74.value <= threshold=-0.49743133783340454
node_6168: feature_name=APP	APP.value <= threshold=0.5775387734174728
node_6169: feature_name=HLA-DQB1	HLA-DQB1.value <= threshold=1.6636562943458557
Class: COVID-19 Critical	
Rules_509	passed counts:8
node_0: feature_name=LTB	LTB.value > threshold=0.994031548500061
node_5264: feature_name=MT-CO1	MT-CO1.value <= threshold=-0.4127991199493408
node_5265: feature_name=MTRNR2L2	MTRNR2L2.value <= threshold=2.4473838806152344
node_5266: feature_name=TCL1A	TCL1A.value <= threshold=5.787256956100464
node_5267: feature_name=PLD4	PLD4.value <= threshold=3.544657826423645
node_5268: feature_name=NR4A1	NR4A1.value > threshold=0.8490704298019409
node_5498: feature_name=HLA-DPA1	HLA-DPA1.value > threshold=1.9079211950302124
node_5556: feature_name=CD83	CD83.value <= threshold=1.6135562062263489
node_5557: feature_name=MARCH1	MARCH1.value <= threshold=3.160825490951538
Class: COVID-19 Critical	
Rules_510	passed counts:8
node_0: feature_name=LTB	LTB.value > threshold=0.994031548500061
node_5264: feature_name=MT-CO1	MT-CO1.value <= threshold=-0.4127991199493408
node_5265: feature_name=MTRNR2L2	MTRNR2L2.value <= threshold=2.4473838806152344
node_5266: feature_name=TCL1A	TCL1A.value <= threshold=5.787256956100464
node_5267: feature_name=PLD4	PLD4.value <= threshold=3.544657826423645
node_5268: feature_name=NR4A1	NR4A1.value > threshold=0.8490704298019409
node_5498: feature_name=HLA-DPA1	HLA-DPA1.value <= threshold=1.9079211950302124
node_5499: feature_name=ID3	ID3.value > threshold=-0.17505786567926407
node_5543: feature_name=BCL2A1	BCL2A1.value > threshold=-0.4924587309360504

node_5547: feature_name=IRF8	IRF8.value > threshold=2.243783950805664
Class: COVID-19 Severe	
Rules_511	passed counts:8
node_0: feature_name=LTB	LTB.value > threshold=0.994031548500061
node_5264: feature_name=MT-CO1	MT-CO1.value <= threshold=-0.4127991199493408
node_5265: feature_name=MTRNR2L2	MTRNR2L2.value <= threshold=2.4473838806152344
node_5266: feature_name=TCL1A	TCL1A.value <= threshold=5.787256956100464
node_5267: feature_name=PLD4	PLD4.value <= threshold=3.544657826423645
node_5268: feature_name=NR4A1	NR4A1.value > threshold=0.8490704298019409
node_5498: feature_name=HLA-DPA1	HLA-DPA1.value <= threshold=1.9079211950302124
node_5499: feature_name=ID3	ID3.value > threshold=-0.17505786567926407
node_5543: feature_name=BCL2A1	BCL2A1.value > threshold=-0.4924587309360504
node_5547: feature_name=IRF8	IRF8.value <= threshold=2.243783950805664
node_5548: feature_name=BOD1L1	BOD1L1.value <= threshold=0.25003959238529205
node_5549: feature_name=FERMT3	FERMT3.value <= threshold=1.291577696800232
Class: COVID-19 Critical	
Rules_512	passed counts:8
node_0: feature_name=LTB	LTB.value > threshold=0.994031548500061
node_5264: feature_name=MT-CO1	MT-CO1.value <= threshold=-0.4127991199493408
node_5265: feature_name=MTRNR2L2	MTRNR2L2.value <= threshold=2.4473838806152344
node_5266: feature_name=TCL1A	TCL1A.value <= threshold=5.787256956100464
node_5267: feature_name=PLD4	PLD4.value <= threshold=3.544657826423645
node_5268: feature_name=NR4A1	NR4A1.value > threshold=0.8490704298019409
node_5498: feature_name=HLA-DPA1	HLA-DPA1.value <= threshold=1.9079211950302124
node_5499: feature_name=ID3	ID3.value <= threshold=-0.17505786567926407
node_5500: feature_name=PPIF	PPIF.value > threshold=-0.5180355906486511
node_5512: feature_name=PDLIM1	PDLIM1.value > threshold=2.5938297510147095
node_5526: feature_name=FTH1	FTH1.value > threshold=0.17593900486826897

node_5536: feature_name=BANK1	BANK1.value > threshold=1.7925409078598022
node_5540: feature_name=IFI44L	IFI44L.value > threshold=-0.35279013216495514
Class: COVID-19 Severe	
Rules_513	passed counts:8
node_0: feature_name=LTB	LTB.value > threshold=0.994031548500061
node_5264: feature_name=MT-CO1	MT-CO1.value <= threshold=-0.4127991199493408
node_5265: feature_name=MTRNR2L2	MTRNR2L2.value <= threshold=2.4473838806152344
node_5266: feature_name=TCL1A	TCL1A.value <= threshold=5.787256956100464
node_5267: feature_name=PLD4	PLD4.value <= threshold=3.544657826423645
node_5268: feature_name=NR4A1	NR4A1.value <= threshold=0.8490704298019409
node_5269: feature_name=LTB	LTB.value <= threshold=1.5252206921577454
node_5270: feature_name=HLA-DRB5	HLA-DRB5.value <= threshold=2.5117164850234985
node_5271: feature_name=CD79A	CD79A.value <= threshold=3.685201406478882
node_5272: feature_name=BCL2A1	BCL2A1.value <= threshold=2.081526756286621
node_5273: feature_name=HLA-DPA1	HLA-DPA1.value > threshold=1.8042166829109192
node_5363: feature_name=MS4A1	MS4A1.value > threshold=3.5283708572387695
node_5435: feature_name=MS4A1	MS4A1.value <= threshold=3.809623122215271
Class: Healthy Control	
Rules_514	passed counts:8
node_0: feature_name=LTB	LTB.value <= threshold=0.994031548500061
node_1: feature_name=MTRNR2L2	MTRNR2L2.value <= threshold=2.8784297704696655
node_2: feature_name=TCL1A	TCL1A.value > threshold=4.798312664031982
node_4912: feature_name=MT-CO1	MT-CO1.value > threshold=-0.3664844036102295
node_5002: feature_name=IFI44L	IFI44L.value <= threshold=2.3101747035980225
node_5003: feature_name=SNX9	SNX9.value > threshold=2.0247421264648438
node_5147: feature_name=CD79A	CD79A.value > threshold=2.554050326347351
node_5169: feature_name=CD79B	CD79B.value > threshold=-0.2480710819363594
node_5185: feature_name=OAZ1	OAZ1.value <= threshold=0.2523931488394737

node_5186: feature_name=PDLIM1	PDLIM1.value > threshold=2.7835850715637207
Class: Healthy Control	
Rules_515	passed counts:8
node_0: feature_name=LTB	LTB.value <= threshold=0.994031548500061
node_1: feature_name=MTRNR2L2	MTRNR2L2.value <= threshold=2.8784297704696655
node_2: feature_name=TCL1A	TCL1A.value > threshold=4.798312664031982
node_4912: feature_name=MT-CO1	MT-CO1.value > threshold=-0.3664844036102295
node_5002: feature_name=IFI44L	IFI44L.value <= threshold=2.3101747035980225
node_5003: feature_name=SNX9	SNX9.value <= threshold=2.0247421264648438
node_5004: feature_name=STAG3	STAG3.value <= threshold=6.482141733169556
node_5005: feature_name=BACH1	BACH1.value <= threshold=-0.5237349569797516
node_5006: feature_name=OAZ1	OAZ1.value <= threshold=-0.6512158215045929
node_5007: feature_name=HLA-DQA1	HLA-DQA1.value <= threshold=1.5983093976974487
node_5008: feature_name=CYBB	CYBB.value <= threshold=0.598551407456398
Class: COVID-19 Critical	
Rules_516	passed counts:8
node_0: feature_name=LTB	LTB.value <= threshold=0.994031548500061
node_1: feature_name=MTRNR2L2	MTRNR2L2.value <= threshold=2.8784297704696655
node_2: feature_name=TCL1A	TCL1A.value > threshold=4.798312664031982
node_4912: feature_name=MT-CO1	MT-CO1.value <= threshold=-0.3664844036102295
node_4913: feature_name=CD79A	CD79A.value <= threshold=2.987208843231201
node_4914: feature_name=HLA-DPB1	HLA-DPB1.value > threshold=2.0926655530929565
node_4958: feature_name=KIAA0125	KIAA0125.value > threshold=-0.15961305052042007
node_4970: feature_name=PELI1	PELI1.value <= threshold=1.1742149591445923
Class: Healthy Control	
Rules_517	passed counts:8
node_0: feature_name=LTB	LTB.value <= threshold=0.994031548500061

node_1: feature_name=MTRNR2L2	MTRNR2L2.value <= threshold=2.8784297704696655
node_2: feature_name=TCL1A	TCL1A.value <= threshold=4.798312664031982
node_3: feature_name=MT-CO1	MT-CO1.value > threshold=0.08947191759943962
node_2521: feature_name=LTB	LTB.value > threshold=0.6038667261600494
node_4173: feature_name=OAZ1	OAZ1.value > threshold=0.143283873796463
node_4615: feature_name=NR4A1	NR4A1.value > threshold=0.6621004343032837
node_4817: feature_name=HSP90B1	HSP90B1.value <= threshold=1.1699737310409546
node_4818: feature_name=XAF1	XAF1.value > threshold=2.6752071380615234
Class: COVID-19 Critical	
Rules_518	passed counts:8
node_0: feature_name=LTB	LTB.value <= threshold=0.994031548500061
node_1: feature_name=MTRNR2L2	MTRNR2L2.value <= threshold=2.8784297704696655
node_2: feature_name=TCL1A	TCL1A.value <= threshold=4.798312664031982
node_3: feature_name=MT-CO1	MT-CO1.value > threshold=0.08947191759943962
node_2521: feature_name=LTB	LTB.value > threshold=0.6038667261600494
node_4173: feature_name=OAZ1	OAZ1.value > threshold=0.143283873796463
node_4615: feature_name=NR4A1	NR4A1.value > threshold=0.6621004343032837
node_4817: feature_name=HSP90B1	HSP90B1.value <= threshold=1.1699737310409546
node_4818: feature_name=XAF1	XAF1.value <= threshold=2.6752071380615234
node_4819: feature_name=SNX9	SNX9.value > threshold=2.939147114753723
node_4885: feature_name=CD79B	CD79B.value > threshold=-0.2693563252687454
node_4891: feature_name=TSPYL2	TSPYL2.value > threshold=-0.7927102446556091
Class: COVID-19 Critical	
Rules_519	passed counts:8
node_0: feature_name=LTB	LTB.value <= threshold=0.994031548500061
node_1: feature_name=MTRNR2L2	MTRNR2L2.value <= threshold=2.8784297704696655
node_2: feature_name=TCL1A	TCL1A.value <= threshold=4.798312664031982
node_3: feature_name=MT-CO1	MT-CO1.value > threshold=0.08947191759943962

node_2521: feature_name=LTB	LTB.value > threshold=0.6038667261600494
node_4173: feature_name=OAZ1	OAZ1.value > threshold=0.143283873796463
node_4615: feature_name=NR4A1	NR4A1.value > threshold=0.6621004343032837
node_4817: feature_name=HSP90B1	HSP90B1.value <= threshold=1.1699737310409546
node_4818: feature_name=XAF1	XAF1.value <= threshold=2.6752071380615234
node_4819: feature_name=SNX9	SNX9.value <= threshold=2.939147114753723
node_4820: feature_name=HLA-DQA2	HLA-DQA2.value <= threshold=2.9822239875793457
node_4821: feature_name=ATF5	ATF5.value <= threshold=4.116653323173523
node_4822: feature_name=RALGPS2	RALGPS2.value > threshold=-0.3627814054489136
node_4834: feature_name=CD79B	CD79B.value > threshold=-0.4220905303955078
node_4838: feature_name=MIAT	MIAT.value <= threshold=2.609323263168335
node_4839: feature_name=YWHAH	YWHAH.value <= threshold=4.648661851882935
node_4840: feature_name=HLA-DRB5	HLA-DRB5.value > threshold=-0.8319411873817444
node_4842: feature_name=MX1	MX1.value <= threshold=3.420834183692932
node_4843: feature_name=SLC7A5	SLC7A5.value <= threshold=2.6673996448516846
node_4844: feature_name=PTGS1	PTGS1.value <= threshold=5.0393829345703125
node_4845: feature_name=ATG2A	ATG2A.value <= threshold=2.2149808406829834
node_4846: feature_name=MT-CO2	MT-CO2.value > threshold=-0.6727012395858765
node_4850: feature_name=AHR	AHR.value > threshold=1.3810421824455261
node_4860: feature_name=RP11-731F5.2	RP11-731F5.2.value > threshold=-0.16218073666095734
Class: Healthy Control	
Rules_520	passed counts:8
node_0: feature_name=LTB	LTB.value <= threshold=0.994031548500061
node_1: feature_name=MTRNR2L2	MTRNR2L2.value <= threshold=2.8784297704696655
node_2: feature_name=TCL1A	TCL1A.value <= threshold=4.798312664031982
node_3: feature_name=MT-CO1	MT-CO1.value > threshold=0.08947191759943962
node_2521: feature_name=LTB	LTB.value > threshold=0.6038667261600494
node_4173: feature_name=OAZ1	OAZ1.value > threshold=0.143283873796463
node_4615: feature_name=NR4A1	NR4A1.value <= threshold=0.6621004343032837

node_4616: feature_name=NFKBID	NFKBID.value <= threshold=3.283741593360901
node_4617: feature_name=HLA-DQA1	HLA-DQA1.value <= threshold=2.3435752391815186
node_4618: feature_name=PELI1	PELI1.value <= threshold=1.3271203637123108
node_4619: feature_name=PLD4	PLD4.value <= threshold=3.3385335206985474
node_4620: feature_name=MEF2A	MEF2A.value > threshold=1.5926940441131592
node_4658: feature_name=STAG3	STAG3.value <= threshold=-0.2016931176185608
node_4659: feature_name=HLA-DQB1	HLA-DQB1.value > threshold=1.983833909034729
node_4665: feature_name=MTRNR2L2	MTRNR2L2.value > threshold=-0.2939942181110382
Class: Healthy Control	
Rules_521	passed counts:8
node_0: feature_name=LTB	LTB.value <= threshold=0.994031548500061
node_1: feature_name=MTRNR2L2	MTRNR2L2.value <= threshold=2.8784297704696655
node_2: feature_name=TCL1A	TCL1A.value <= threshold=4.798312664031982
node_3: feature_name=MT-CO1	MT-CO1.value > threshold=0.08947191759943962
node_2521: feature_name=LTB	LTB.value > threshold=0.6038667261600494
node_4173: feature_name=OAZ1	OAZ1.value > threshold=0.143283873796463
node_4615: feature_name=NR4A1	NR4A1.value <= threshold=0.6621004343032837
node_4616: feature_name=NFKBID	NFKBID.value <= threshold=3.283741593360901
node_4617: feature_name=HLA-DQA1	HLA-DQA1.value <= threshold=2.3435752391815186
node_4618: feature_name=PELI1	PELI1.value <= threshold=1.3271203637123108
node_4619: feature_name=PLD4	PLD4.value <= threshold=3.3385335206985474
node_4620: feature_name=MEF2A	MEF2A.value <= threshold=1.5926940441131592
node_4621: feature_name=DAPP1	DAPP1.value <= threshold=2.647637367248535
node_4622: feature_name=STAG3	STAG3.value <= threshold=-0.16521219909191132
node_4623: feature_name=OAZ1	OAZ1.value > threshold=0.2042311355471611
node_4625: feature_name=FAM129C	FAM129C.value <= threshold=-0.23615043610334396
node_4626: feature_name=TCL1A	TCL1A.value > threshold=-0.18263818323612213
Class: COVID-19 Critical	

Rules_522	passed counts:8
node_0: feature_name=LTB	LTB.value <= threshold=0.994031548500061
node_1: feature_name=MTRNR2L2	MTRNR2L2.value <= threshold=2.8784297704696655
node_2: feature_name=TCL1A	TCL1A.value <= threshold=4.798312664031982
node_3: feature_name=MT-CO1	MT-CO1.value > threshold=0.08947191759943962
node_2521: feature_name=LTB	LTB.value > threshold=0.6038667261600494
node_4173: feature_name=OAZ1	OAZ1.value <= threshold=0.143283873796463
node_4174: feature_name=PLD4	PLD4.value <= threshold=2.921932101249695
node_4175: feature_name=XIST	XIST.value <= threshold=-0.05067425221204758
node_4176: feature_name=MT-CO1	MT-CO1.value <= threshold=1.345594048500061
node_4177: feature_name=HLA-DQB1	HLA-DQB1.value > threshold=1.927736520767212
node_4353: feature_name=ITM2C	ITM2C.value <= threshold=-0.12329206243157387
node_4354: feature_name=MX1	MX1.value > threshold=-0.4356398284435272
node_4430: feature_name=CD83	CD83.value <= threshold=3.929547071456909
node_4431: feature_name=SNX9	SNX9.value > threshold=3.212221622467041
node_4447: feature_name=HIST1H2AC	HIST1H2AC.value <= threshold=-0.338812991976738
node_4448: feature_name=CD22	CD22.value > threshold=-0.26052001118659973
Class: COVID-19 Critical	
Rules_523	passed counts:8
node_0: feature_name=LTB	LTB.value <= threshold=0.994031548500061
node_1: feature_name=MTRNR2L2	MTRNR2L2.value <= threshold=2.8784297704696655
node_2: feature_name=TCL1A	TCL1A.value <= threshold=4.798312664031982
node_3: feature_name=MT-CO1	MT-CO1.value > threshold=0.08947191759943962
node_2521: feature_name=LTB	LTB.value > threshold=0.6038667261600494
node_4173: feature_name=OAZ1	OAZ1.value <= threshold=0.143283873796463
node_4174: feature_name=PLD4	PLD4.value <= threshold=2.921932101249695
node_4175: feature_name=XIST	XIST.value <= threshold=-0.05067425221204758
node_4176: feature_name=MT-CO1	MT-CO1.value <= threshold=1.345594048500061
node_4177: feature_name=HLA-DQB1	HLA-DQB1.value > threshold=1.927736520767212

node_4353: feature_name=ITM2C	ITM2C.value <= threshold=-0.12329206243157387
node_4354: feature_name=MX1	MX1.value <= threshold=-0.4356398284435272
node_4355: feature_name=HLA-DQB1	HLA-DQB1.value <= threshold=2.8865538835525513
node_4356: feature_name=SNX9	SNX9.value > threshold=1.002076417207718
node_4384: feature_name=HLA-DRB5	HLA-DRB5.value <= threshold=1.0937854647636414
node_4385: feature_name=IFI6	IFI6.value > threshold=0.09842458367347717
Class: COVID-19 Critical	
Rules_524	passed counts:8
node_0: feature_name=LTB	LTB.value <= threshold=0.994031548500061
node_1: feature_name=MTRNR2L2	MTRNR2L2.value <= threshold=2.8784297704696655
node_2: feature_name=TCL1A	TCL1A.value <= threshold=4.798312664031982
node_3: feature_name=MT-CO1	MT-CO1.value > threshold=0.08947191759943962
node_2521: feature_name=LTB	LTB.value > threshold=0.6038667261600494
node_4173: feature_name=OAZ1	OAZ1.value <= threshold=0.143283873796463
node_4174: feature_name=PLD4	PLD4.value <= threshold=2.921932101249695
node_4175: feature_name=XIST	XIST.value <= threshold=-0.05067425221204758
node_4176: feature_name=MT-CO1	MT-CO1.value <= threshold=1.345594048500061
node_4177: feature_name=HLA-DQB1	HLA-DQB1.value <= threshold=1.927736520767212
node_4178: feature_name=BACH1	BACH1.value > threshold=-0.5349778532981873
node_4236: feature_name=TPM1	TPM1.value <= threshold=3.516477584838867
node_4237: feature_name=NR4A1	NR4A1.value <= threshold=-0.274935707449913
node_4238: feature_name=VPREB3	VPREB3.value <= threshold=5.024149179458618
node_4239: feature_name=YWHAH	YWHAH.value <= threshold=4.7504096031188965
node_4240: feature_name=NAIP	NAIP.value <= threshold=4.030007481575012
node_4241: feature_name=CD38	CD38.value > threshold=-0.3387429714202881
node_4243: feature_name=MS4A1	MS4A1.value <= threshold=5.195746660232544
node_4244: feature_name=TRIB1	TRIB1.value <= threshold=2.9039160013198853
node_4245: feature_name=RGS2	RGS2.value > threshold=-0.8980970978736877
node_4249: feature_name=PLD4	PLD4.value <= threshold=2.8284080028533936

node_4250: feature_name=GRAP2	GRAP2.value <= threshold=2.0700820088386536
node_4251: feature_name=ATF3	ATF3.value <= threshold=2.327788233757019
node_4252: feature_name=ITM2C	ITM2C.value > threshold=3.576403021812439
node_4254: feature_name=DUSP4	DUSP4.value > threshold=-0.27090051770210266
Class: COVID-19 Critical	
Rules_525	passed counts:8
node_0: feature_name=LTB	LTB.value <= threshold=0.994031548500061
node_1: feature_name=MTRNR2L2	MTRNR2L2.value <= threshold=2.8784297704696655
node_2: feature_name=TCL1A	TCL1A.value <= threshold=4.798312664031982
node_3: feature_name=MT-CO1	MT-CO1.value > threshold=0.08947191759943962
node_2521: feature_name=LTB	LTB.value > threshold=0.6038667261600494
node_4173: feature_name=OAZ1	OAZ1.value <= threshold=0.143283873796463
node_4174: feature_name=PLD4	PLD4.value <= threshold=2.921932101249695
node_4175: feature_name=XIST	XIST.value <= threshold=-0.05067425221204758
node_4176: feature_name=MT-CO1	MT-CO1.value <= threshold=1.345594048500061
node_4177: feature_name=HLA-DQB1	HLA-DQB1.value <= threshold=1.927736520767212
node_4178: feature_name=BACH1	BACH1.value <= threshold=-0.5349778532981873
node_4179: feature_name=CD79B	CD79B.value > threshold=1.6518195271492004
node_4201: feature_name=MT-CO2	MT-CO2.value > threshold=0.5007440596818924
node_4223: feature_name=FAM129C	FAM129C.value <= threshold=4.054540395736694
node_4224: feature_name=MEF2A	MEF2A.value <= threshold=-0.4423168897628784
node_4225: feature_name=ENDOD1	ENDOD1.value > threshold=-0.18554074317216873
node_4229: feature_name=PLD4	PLD4.value <= threshold=2.418735980987549
Class: COVID-19 Critical	
Rules_526	passed counts:8
node_0: feature_name=LTB	LTB.value <= threshold=0.994031548500061
node_1: feature_name=MTRNR2L2	MTRNR2L2.value <= threshold=2.8784297704696655
node_2: feature_name=TCL1A	TCL1A.value <= threshold=4.798312664031982

node_3: feature_name=MT-CO1	MT-CO1.value > threshold=0.08947191759943962
node_2521: feature_name=LTB	LTB.value <= threshold=0.6038667261600494
node_2522: feature_name=HLA-DQB1	HLA-DQB1.value > threshold=2.1053054332733154
node_3550: feature_name=XIST	XIST.value > threshold=0.7716337237507105
node_4140: feature_name=HLA-DQA2	HLA-DQA2.value > threshold=-0.1354314684867859
node_4158: feature_name=MT-CO3	MT-CO3.value <= threshold=0.5339524000883102
node_4159: feature_name=HSP90B1	HSP90B1.value <= threshold=1.0332441329956055
node_4160: feature_name=CD38	CD38.value <= threshold=-0.22172659635543823
Class: COVID-19 Critical	
Rules_527	passed counts:8
node_0: feature_name=LTB	LTB.value <= threshold=0.994031548500061
node_1: feature_name=MTRNR2L2	MTRNR2L2.value <= threshold=2.8784297704696655
node_2: feature_name=TCL1A	TCL1A.value <= threshold=4.798312664031982
node_3: feature_name=MT-CO1	MT-CO1.value > threshold=0.08947191759943962
node_2521: feature_name=LTB	LTB.value <= threshold=0.6038667261600494
node_2522: feature_name=HLA-DQB1	HLA-DQB1.value > threshold=2.1053054332733154
node_3550: feature_name=XIST	XIST.value <= threshold=0.7716337237507105
node_3551: feature_name=CD79B	CD79B.value > threshold=2.9426796436309814
node_4039: feature_name=IFI44L	IFI44L.value <= threshold=1.8637521862983704
node_4040: feature_name=SNX9	SNX9.value > threshold=1.274232804775238
node_4096: feature_name=RALGPS2	RALGPS2.value > threshold=2.3599878549575806
node_4118: feature_name=SNX29P2	SNX29P2.value <= threshold=0.880477637052536
node_4119: feature_name=FAM129C	FAM129C.value > threshold=3.415250778198242
node_4125: feature_name=IGJ	IGJ.value > threshold=-0.2685837149620056
node_4129: feature_name=AHR	AHR.value <= threshold=1.625461682677269
Class: Healthy Control	
Rules_528	passed counts:8
node_0: feature_name=LTB	LTB.value <= threshold=0.994031548500061

node_1: feature_name=MTRNR2L2	MTRNR2L2.value <= threshold=2.8784297704696655
node_2: feature_name=TCL1A	TCL1A.value <= threshold=4.798312664031982
node_3: feature_name=MT-CO1	MT-CO1.value > threshold=0.08947191759943962
node_2521: feature_name=LTB	LTB.value <= threshold=0.6038667261600494
node_2522: feature_name=HLA-DQB1	HLA-DQB1.value > threshold=2.1053054332733154
node_3550: feature_name=XIST	XIST.value <= threshold=0.7716337237507105
node_3551: feature_name=CD79B	CD79B.value > threshold=2.9426796436309814
node_4039: feature_name=IFI44L	IFI44L.value <= threshold=1.8637521862983704
node_4040: feature_name=SNX9	SNX9.value <= threshold=1.274232804775238
node_4041: feature_name=CASP8	CASP8.value > threshold=-0.574097603559494
node_4073: feature_name=HLA-DRB5	HLA-DRB5.value > threshold=1.4346495270729065
node_4083: feature_name=NT5C3A	NT5C3A.value <= threshold=-0.45546136796474457
node_4084: feature_name=OAZ1	OAZ1.value > threshold=0.1348155364394188
node_4090: feature_name=CD38	CD38.value <= threshold=-0.24148839712142944
Class: Healthy Control	
Rules_529	passed counts:8
node_0: feature_name=LTB	LTB.value <= threshold=0.994031548500061
node_1: feature_name=MTRNR2L2	MTRNR2L2.value <= threshold=2.8784297704696655
node_2: feature_name=TCL1A	TCL1A.value <= threshold=4.798312664031982
node_3: feature_name=MT-CO1	MT-CO1.value > threshold=0.08947191759943962
node_2521: feature_name=LTB	LTB.value <= threshold=0.6038667261600494
node_2522: feature_name=HLA-DQB1	HLA-DQB1.value > threshold=2.1053054332733154
node_3550: feature_name=XIST	XIST.value <= threshold=0.7716337237507105
node_3551: feature_name=CD79B	CD79B.value > threshold=2.9426796436309814
node_4039: feature_name=IFI44L	IFI44L.value <= threshold=1.8637521862983704
node_4040: feature_name=SNX9	SNX9.value <= threshold=1.274232804775238
node_4041: feature_name=CASP8	CASP8.value <= threshold=-0.574097603559494
node_4042: feature_name=NFKBID	NFKBID.value > threshold=1.49756920337677
node_4056: feature_name=MIR24-2	MIR24-2.value <= threshold=0.2797542065382004

node_4057: feature_name=MEF2C	MEF2C.value <= threshold=2.6051090955734253
node_4058: feature_name=BOD1L1	BOD1L1.value <= threshold=-0.476860374212265
node_4059: feature_name=IGLL5	IGLL5.value <= threshold=2.377661719918251
Class: Healthy Control	
Rules_530	passed counts:8
node_0: feature_name=LTB	LTB.value <= threshold=0.994031548500061
node_1: feature_name=MTRNR2L2	MTRNR2L2.value <= threshold=2.8784297704696655
node_2: feature_name=TCL1A	TCL1A.value <= threshold=4.798312664031982
node_3: feature_name=MT-CO1	MT-CO1.value > threshold=0.08947191759943962
node_2521: feature_name=LTB	LTB.value <= threshold=0.6038667261600494
node_2522: feature_name=HLA-DQB1	HLA-DQB1.value > threshold=2.1053054332733154
node_3550: feature_name=XIST	XIST.value <= threshold=0.7716337237507105
node_3551: feature_name=CD79B	CD79B.value <= threshold=2.9426796436309814
node_3552: feature_name=HLA-DQB1	HLA-DQB1.value > threshold=2.713674306869507
node_3854: feature_name=MEF2C	MEF2C.value > threshold=-0.3721291869878769
node_3882: feature_name=IFI44L	IFI44L.value <= threshold=2.5516022443771362
node_3883: feature_name=FCER2	FCER2.value > threshold=-0.21487552672624588
node_3977: feature_name=IGJ	IGJ.value <= threshold=2.123470664024353
node_3978: feature_name=S100A6	S100A6.value > threshold=-1.7107264399528503
node_3982: feature_name=MT-CO1	MT-CO1.value <= threshold=1.5725501775741577
node_3983: feature_name=SNX29P2	SNX29P2.value > threshold=3.1593780517578125
node_4013: feature_name=KIAA0125	KIAA0125.value <= threshold=-0.16171331703662872
Class: Healthy Control	
Rules_531	passed counts:8
node_0: feature_name=LTB	LTB.value <= threshold=0.994031548500061
node_1: feature_name=MTRNR2L2	MTRNR2L2.value <= threshold=2.8784297704696655
node_2: feature_name=TCL1A	TCL1A.value <= threshold=4.798312664031982
node_3: feature_name=MT-CO1	MT-CO1.value > threshold=0.08947191759943962

node_2521: feature_name=LTB	LTB.value <= threshold=0.6038667261600494
node_2522: feature_name=HLA-DQB1	HLA-DQB1.value > threshold=2.1053054332733154
node_3550: feature_name=XIST	XIST.value <= threshold=0.7716337237507105
node_3551: feature_name=CD79B	CD79B.value <= threshold=2.9426796436309814
node_3552: feature_name=HLA-DQB1	HLA-DQB1.value > threshold=2.713674306869507
node_3854: feature_name=MEF2C	MEF2C.value > threshold=-0.3721291869878769
node_3882: feature_name=IFI44L	IFI44L.value <= threshold=2.5516022443771362
node_3883: feature_name=FCER2	FCER2.value > threshold=-0.21487552672624588
node_3977: feature_name=IGJ	IGJ.value <= threshold=2.123470664024353
node_3978: feature_name=S100A6	S100A6.value <= threshold=-1.7107264399528503
node_3979: feature_name=UTY	UTY.value > threshold=-0.4821864813566208
Class: COVID-19 Severe	
Rules_532	passed counts:8
node_0: feature_name=LTB	LTB.value <= threshold=0.994031548500061
node_1: feature_name=MTRNR2L2	MTRNR2L2.value <= threshold=2.8784297704696655
node_2: feature_name=TCL1A	TCL1A.value <= threshold=4.798312664031982
node_3: feature_name=MT-CO1	MT-CO1.value > threshold=0.08947191759943962
node_2521: feature_name=LTB	LTB.value <= threshold=0.6038667261600494
node_2522: feature_name=HLA-DQB1	HLA-DQB1.value > threshold=2.1053054332733154
node_3550: feature_name=XIST	XIST.value <= threshold=0.7716337237507105
node_3551: feature_name=CD79B	CD79B.value <= threshold=2.9426796436309814
node_3552: feature_name=HLA-DQB1	HLA-DQB1.value > threshold=2.713674306869507
node_3854: feature_name=MEF2C	MEF2C.value > threshold=-0.3721291869878769
node_3882: feature_name=IFI44L	IFI44L.value <= threshold=2.5516022443771362
node_3883: feature_name=FCER2	FCER2.value <= threshold=-0.21487552672624588
node_3884: feature_name=HLA-DQA2	HLA-DQA2.value <= threshold=-0.12580305337905884
node_3885: feature_name=SLC7A5	SLC7A5.value <= threshold=-0.4906871169805527
node_3886: feature_name=VPREB3	VPREB3.value > threshold=5.492124557495117
node_3942: feature_name=LDLR	LDLR.value <= threshold=1.8207427263259888

Class: Healthy Control	
Rules_533	passed counts:8
node_0: feature_name=LTB	LTB.value <= threshold=0.994031548500061
node_1: feature_name=MTRNR2L2	MTRNR2L2.value <= threshold=2.8784297704696655
node_2: feature_name=TCL1A	TCL1A.value <= threshold=4.798312664031982
node_3: feature_name=MT-CO1	MT-CO1.value > threshold=0.08947191759943962
node_2521: feature_name=LTB	LTB.value <= threshold=0.6038667261600494
node_2522: feature_name=HLA-DQB1	HLA-DQB1.value > threshold=2.1053054332733154
node_3550: feature_name=XIST	XIST.value <= threshold=0.7716337237507105
node_3551: feature_name=CD79B	CD79B.value <= threshold=2.9426796436309814
node_3552: feature_name=HLA-DQB1	HLA-DQB1.value <= threshold=2.713674306869507
node_3553: feature_name=SNX9	SNX9.value > threshold=-0.5404067635536194
node_3653: feature_name=LTB	LTB.value > threshold=0.021708179265260696
node_3759: feature_name=TCL1A	TCL1A.value <= threshold=3.3921085596084595
node_3760: feature_name=HLA-DRB5	HLA-DRB5.value > threshold=0.5265218019485474
node_3776: feature_name=ID1	ID1.value <= threshold=-0.21235637366771698
node_3777: feature_name=RP11-731F5.2	RP11-731F5.2.value > threshold=2.830894112586975
node_3817: feature_name=BOD1L1	BOD1L1.value > threshold=-0.4770502895116806
node_3825: feature_name=TRAF1	TRAF1.value <= threshold=1.0712183713912964
Class: COVID-19 Critical	
Rules_534	passed counts:8
node_0: feature_name=LTB	LTB.value <= threshold=0.994031548500061
node_1: feature_name=MTRNR2L2	MTRNR2L2.value <= threshold=2.8784297704696655
node_2: feature_name=TCL1A	TCL1A.value <= threshold=4.798312664031982
node_3: feature_name=MT-CO1	MT-CO1.value > threshold=0.08947191759943962
node_2521: feature_name=LTB	LTB.value <= threshold=0.6038667261600494
node_2522: feature_name=HLA-DQB1	HLA-DQB1.value <= threshold=2.1053054332733154
node_2523: feature_name=SNX9	SNX9.value > threshold=-0.5346486568450928

node_2907: feature_name=NR4A1	NR4A1.value > threshold=2.5433837175369263
node_3459: feature_name=VPREB3	VPREB3.value <= threshold=4.410779237747192
node_3460: feature_name=RALGPS2	RALGPS2.value > threshold=3.064661979675293
node_3520: feature_name=RGS2	RGS2.value > threshold=-0.5165578126907349
node_3522: feature_name=MT-ND6	MT-ND6.value > threshold=1.692318856716156
node_3542: feature_name=CD22	CD22.value <= threshold=8.577749729156494
Class: Healthy Control	
Rules_535	passed counts:8
node_0: feature_name=LTB	LTB.value <= threshold=0.994031548500061
node_1: feature_name=MTRNR2L2	MTRNR2L2.value <= threshold=2.8784297704696655
node_2: feature_name=TCL1A	TCL1A.value <= threshold=4.798312664031982
node_3: feature_name=MT-CO1	MT-CO1.value > threshold=0.08947191759943962
node_2521: feature_name=LTB	LTB.value <= threshold=0.6038667261600494
node_2522: feature_name=HLA-DQB1	HLA-DQB1.value <= threshold=2.1053054332733154
node_2523: feature_name=SNX9	SNX9.value > threshold=-0.5346486568450928
node_2907: feature_name=NR4A1	NR4A1.value > threshold=2.5433837175369263
node_3459: feature_name=VPREB3	VPREB3.value <= threshold=4.410779237747192
node_3460: feature_name=RALGPS2	RALGPS2.value <= threshold=3.064661979675293
node_3461: feature_name=MT-CO1	MT-CO1.value <= threshold=1.7489010095596313
node_3462: feature_name=HLA-DQA2	HLA-DQA2.value <= threshold=1.794841468334198
node_3463: feature_name=CYBB	CYBB.value > threshold=1.5984815955162048
node_3491: feature_name=PELI1	PELI1.value <= threshold=0.13108068704605103
node_3492: feature_name=HLA-DPA1	HLA-DPA1.value > threshold=-0.6165751218795776
Class: Healthy Control	
Rules_536	passed counts:8
node_0: feature_name=LTB	LTB.value <= threshold=0.994031548500061
node_1: feature_name=MTRNR2L2	MTRNR2L2.value <= threshold=2.8784297704696655
node_2: feature_name=TCL1A	TCL1A.value <= threshold=4.798312664031982

node_3: feature_name=MT-CO1	MT-CO1.value > threshold=0.08947191759943962
node_2521: feature_name=LTB	LTB.value <= threshold=0.6038667261600494
node_2522: feature_name=HLA-DQB1	HLA-DQB1.value <= threshold=2.1053054332733154
node_2523: feature_name=SNX9	SNX9.value > threshold=-0.5346486568450928
node_2907: feature_name=NR4A1	NR4A1.value <= threshold=2.5433837175369263
node_2908: feature_name=XIST	XIST.value <= threshold=0.7664470989257097
node_2909: feature_name=CD79B	CD79B.value <= threshold=2.9326847791671753
node_2910: feature_name=NR4A1	NR4A1.value > threshold=-0.2875557690858841
node_3096: feature_name=NR4A3	NR4A3.value <= threshold=3.314968705177307
node_3097: feature_name=VPREB3	VPREB3.value <= threshold=3.3549938201904297
node_3098: feature_name=RALGPS2	RALGPS2.value <= threshold=5.807050704956055
node_3099: feature_name=CTD-2035E11.3	CTD-2035E11.3.value <= threshold=6.4090416431427
node_3100: feature_name=FAM129C	FAM129C.value <= threshold=6.966361045837402
node_3101: feature_name=HES4	HES4.value <= threshold=4.139557242393494
node_3102: feature_name=CD83	CD83.value > threshold=1.8343153595924377
node_3136: feature_name=PLD4	PLD4.value <= threshold=4.453821897506714
node_3137: feature_name=HLA-DQA2	HLA-DQA2.value > threshold=-0.10758286714553833
node_3229: feature_name=FAM160B1	FAM160B1.value > threshold=1.61697119474411
node_3261: feature_name=TNFAIP3	TNFAIP3.value > threshold=-1.4364122152328491
Class: COVID-19 Severe	
Rules_537	passed counts:8
node_0: feature_name=LTB	LTB.value <= threshold=0.994031548500061
node_1: feature_name=MTRNR2L2	MTRNR2L2.value <= threshold=2.8784297704696655
node_2: feature_name=TCL1A	TCL1A.value <= threshold=4.798312664031982
node_3: feature_name=MT-CO1	MT-CO1.value > threshold=0.08947191759943962
node_2521: feature_name=LTB	LTB.value <= threshold=0.6038667261600494
node_2522: feature_name=HLA-DQB1	HLA-DQB1.value <= threshold=2.1053054332733154
node_2523: feature_name=SNX9	SNX9.value > threshold=-0.5346486568450928
node_2907: feature_name=NR4A1	NR4A1.value <= threshold=2.5433837175369263

node_2908: feature_name=XIST	XIST.value <= threshold=0.7664470989257097
node_2909: feature_name=CD79B	CD79B.value <= threshold=2.9326847791671753
node_2910: feature_name=NR4A1	NR4A1.value > threshold=-0.2875557690858841
node_3096: feature_name=NR4A3	NR4A3.value <= threshold=3.314968705177307
node_3097: feature_name=VPREB3	VPREB3.value <= threshold=3.3549938201904297
node_3098: feature_name=RALGPS2	RALGPS2.value <= threshold=5.807050704956055
node_3099: feature_name=CTD-2035E11.3	CTD-2035E11.3.value <= threshold=6.4090416431427
node_3100: feature_name=FAM129C	FAM129C.value <= threshold=6.966361045837402
node_3101: feature_name=HES4	HES4.value <= threshold=4.139557242393494
node_3102: feature_name=CD83	CD83.value > threshold=1.8343153595924377
node_3136: feature_name=PLD4	PLD4.value <= threshold=4.453821897506714
node_3137: feature_name=HLA-DQA2	HLA-DQA2.value > threshold=-0.10758286714553833
node_3229: feature_name=FAM160B1	FAM160B1.value <= threshold=1.61697119474411
node_3230: feature_name=BCL2A1	BCL2A1.value > threshold=0.9140156805515289
node_3250: feature_name=RNF11	RNF11.value <= threshold=-0.5307398736476898
node_3251: feature_name=RP11-731F5.2	RP11-731F5.2.value <= threshold=1.3988762274384499
Class: COVID-19 Severe	
Rules_538	passed counts:8
node_0: feature_name=LTB	LTB.value <= threshold=0.994031548500061
node_1: feature_name=MTRNR2L2	MTRNR2L2.value <= threshold=2.8784297704696655
node_2: feature_name=TCL1A	TCL1A.value <= threshold=4.798312664031982
node_3: feature_name=MT-CO1	MT-CO1.value > threshold=0.08947191759943962
node_2521: feature_name=LTB	LTB.value <= threshold=0.6038667261600494
node_2522: feature_name=HLA-DQB1	HLA-DQB1.value <= threshold=2.1053054332733154
node_2523: feature_name=SNX9	SNX9.value > threshold=-0.5346486568450928
node_2907: feature_name=NR4A1	NR4A1.value <= threshold=2.5433837175369263
node_2908: feature_name=XIST	XIST.value <= threshold=0.7664470989257097
node_2909: feature_name=CD79B	CD79B.value <= threshold=2.9326847791671753
node_2910: feature_name=NR4A1	NR4A1.value <= threshold=-0.2875557690858841

node_2911: feature_name=CTD-2035E11.3	CTD-2035E11.3.value <= threshold=5.262048006057739
node_2912: feature_name=VPREB3	VPREB3.value > threshold=4.937594890594482
node_3072: feature_name=LTB	LTB.value > threshold=0.5297379791736603
node_3092: feature_name=YWHAH	YWHAH.value <= threshold=0.6025846526026726
Class: Healthy Control	
Rules_539	passed counts:8
node_0: feature_name=LTB	LTB.value <= threshold=0.994031548500061
node_1: feature_name=MTRNR2L2	MTRNR2L2.value <= threshold=2.8784297704696655
node_2: feature_name=TCL1A	TCL1A.value <= threshold=4.798312664031982
node_3: feature_name=MT-CO1	MT-CO1.value > threshold=0.08947191759943962
node_2521: feature_name=LTB	LTB.value <= threshold=0.6038667261600494
node_2522: feature_name=HLA-DQB1	HLA-DQB1.value <= threshold=2.1053054332733154
node_2523: feature_name=SNX9	SNX9.value > threshold=-0.5346486568450928
node_2907: feature_name=NR4A1	NR4A1.value <= threshold=2.5433837175369263
node_2908: feature_name=XIST	XIST.value <= threshold=0.7664470989257097
node_2909: feature_name=CD79B	CD79B.value <= threshold=2.9326847791671753
node_2910: feature_name=NR4A1	NR4A1.value <= threshold=-0.2875557690858841
node_2911: feature_name=CTD-2035E11.3	CTD-2035E11.3.value <= threshold=5.262048006057739
node_2912: feature_name=VPREB3	VPREB3.value <= threshold=4.937594890594482
node_2913: feature_name=IRAK3	IRAK3.value <= threshold=4.592883586883545
node_2914: feature_name=IGJ	IGJ.value <= threshold=7.093410015106201
node_2915: feature_name=WDR74	WDR74.value <= threshold=4.077916383743286
node_2916: feature_name=MEF2C	MEF2C.value > threshold=4.374382972717285
node_3058: feature_name=HLA-DRB1	HLA-DRB1.value <= threshold=1.63593327999115
Class: COVID-19 Critical	
Rules_540	passed counts:8
node_0: feature_name=LTB	LTB.value <= threshold=0.994031548500061
node_1: feature_name=MTRNR2L2	MTRNR2L2.value <= threshold=2.8784297704696655

node_2: feature_name=TCL1A	TCL1A.value <= threshold=4.798312664031982
node_3: feature_name=MT-CO1	MT-CO1.value > threshold=0.08947191759943962
node_2521: feature_name=LTB	LTB.value <= threshold=0.6038667261600494
node_2522: feature_name=HLA-DQB1	HLA-DQB1.value <= threshold=2.1053054332733154
node_2523: feature_name=SNX9	SNX9.value > threshold=-0.5346486568450928
node_2907: feature_name=NR4A1	NR4A1.value <= threshold=2.5433837175369263
node_2908: feature_name=XIST	XIST.value <= threshold=0.7664470989257097
node_2909: feature_name=CD79B	CD79B.value <= threshold=2.9326847791671753
node_2910: feature_name=NR4A1	NR4A1.value <= threshold=-0.2875557690858841
node_2911: feature_name=CTD-2035E11.3	CTD-2035E11.3.value <= threshold=5.262048006057739
node_2912: feature_name=VPREB3	VPREB3.value <= threshold=4.937594890594482
node_2913: feature_name=IRAK3	IRAK3.value <= threshold=4.592883586883545
node_2914: feature_name=IGJ	IGJ.value <= threshold=7.093410015106201
node_2915: feature_name=WDR74	WDR74.value <= threshold=4.077916383743286
node_2916: feature_name=MEF2C	MEF2C.value <= threshold=4.374382972717285
node_2917: feature_name=HIST1H2AC	HIST1H2AC.value <= threshold=3.743621349334717
node_2918: feature_name=STMN1	STMN1.value <= threshold=6.266510248184204
node_2919: feature_name=RALGPS2	RALGPS2.value <= threshold=5.72194766998291
node_2920: feature_name=HOPX	HOPX.value <= threshold=2.5944807529449463
node_2921: feature_name=MARCKS	MARCKS.value <= threshold=4.622651815414429
node_2922: feature_name=CCL3	CCL3.value <= threshold=0.7484713792800903
node_2923: feature_name=TPM1	TPM1.value <= threshold=6.446193695068359
node_2924: feature_name=PLD4	PLD4.value <= threshold=8.038907289505005
node_2925: feature_name=COTL1	COTL1.value > threshold=-1.8123814463615417
node_2929: feature_name=ID3	ID3.value <= threshold=7.534881353378296
node_2930: feature_name=IRF8	IRF8.value <= threshold=4.855781078338623
node_2931: feature_name=AP3M2	AP3M2.value > threshold=1.8868122100830078
node_3017: feature_name=MT-ND6	MT-ND6.value > threshold=-0.17867428064346313
Class: COVID-19 Critical	

Rules_541	passed counts:8
node_0: feature_name=LTB	LTB.value <= threshold=0.994031548500061
node_1: feature_name=MTRNR2L2	MTRNR2L2.value <= threshold=2.8784297704696655
node_2: feature_name=TCL1A	TCL1A.value <= threshold=4.798312664031982
node_3: feature_name=MT-CO1	MT-CO1.value > threshold=0.08947191759943962
node_2521: feature_name=LTB	LTB.value <= threshold=0.6038667261600494
node_2522: feature_name=HLA-DQB1	HLA-DQB1.value <= threshold=2.1053054332733154
node_2523: feature_name=SNX9	SNX9.value > threshold=-0.5346486568450928
node_2907: feature_name=NR4A1	NR4A1.value <= threshold=2.5433837175369263
node_2908: feature_name=XIST	XIST.value <= threshold=0.7664470989257097
node_2909: feature_name=CD79B	CD79B.value <= threshold=2.9326847791671753
node_2910: feature_name=NR4A1	NR4A1.value <= threshold=-0.2875557690858841
node_2911: feature_name=CTD-2035E11.3	CTD-2035E11.3.value <= threshold=5.262048006057739
node_2912: feature_name=VPREB3	VPREB3.value <= threshold=4.937594890594482
node_2913: feature_name=IRAK3	IRAK3.value <= threshold=4.592883586883545
node_2914: feature_name=IGJ	IGJ.value <= threshold=7.093410015106201
node_2915: feature_name=WDR74	WDR74.value <= threshold=4.077916383743286
node_2916: feature_name=MEF2C	MEF2C.value <= threshold=4.374382972717285
node_2917: feature_name=HIST1H2AC	HIST1H2AC.value <= threshold=3.743621349334717
node_2918: feature_name=STMN1	STMN1.value <= threshold=6.266510248184204
node_2919: feature_name=RALGPS2	RALGPS2.value <= threshold=5.72194766998291
node_2920: feature_name=HOPX	HOPX.value <= threshold=2.5944807529449463
node_2921: feature_name=MARCKS	MARCKS.value <= threshold=4.622651815414429
node_2922: feature_name=CCL3	CCL3.value <= threshold=0.7484713792800903
node_2923: feature_name=TPM1	TPM1.value <= threshold=6.446193695068359
node_2924: feature_name=PLD4	PLD4.value <= threshold=8.038907289505005
node_2925: feature_name=COTL1	COTL1.value > threshold=-1.8123814463615417
node_2929: feature_name=ID3	ID3.value <= threshold=7.534881353378296
node_2930: feature_name=IRF8	IRF8.value <= threshold=4.855781078338623
node_2931: feature_name=AP3M2	AP3M2.value <= threshold=1.8868122100830078

node_2932: feature_name=FCER2	FCER2.value <= threshold=6.877448558807373
node_2933: feature_name=MTRNR2L2	MTRNR2L2.value <= threshold=2.710945725440979
node_2934: feature_name=GNG11	GNG11.value <= threshold=2.510925054550171
node_2935: feature_name=CD83	CD83.value > threshold=1.9873459935188293
node_2961: feature_name=CD83	CD83.value > threshold=2.0049850940704346
node_2965: feature_name=HES4	HES4.value <= threshold=4.076817035675049
node_2966: feature_name=BCL2A1	BCL2A1.value <= threshold=1.1745486855506897
node_2967: feature_name=NT5C3A	NT5C3A.value <= threshold=3.6911903619766235
node_2968: feature_name=TLN1	TLN1.value <= threshold=2.122964382171631
node_2969: feature_name=MT-CYB	MT-CYB.value > threshold=-0.9192460179328918
node_2973: feature_name=KIAA0125	KIAA0125.value <= threshold=6.9272027015686035
node_2974: feature_name=OAZ1	OAZ1.value > threshold=-1.8451763987541199
node_2978: feature_name=ATG2A	ATG2A.value <= threshold=1.4784215688705444
node_2979: feature_name=MMD	MMD.value > threshold=4.39089298248291
node_2981: feature_name=ENDOD1	ENDOD1.value <= threshold=-0.20693418383598328
Class: COVID-19 Critical	
Rules_542	passed counts:8
node_0: feature_name=LTB	LTB.value <= threshold=0.994031548500061
node_1: feature_name=MTRNR2L2	MTRNR2L2.value <= threshold=2.8784297704696655
node_2: feature_name=TCL1A	TCL1A.value <= threshold=4.798312664031982
node_3: feature_name=MT-CO1	MT-CO1.value > threshold=0.08947191759943962
node_2521: feature_name=LTB	LTB.value <= threshold=0.6038667261600494
node_2522: feature_name=HLA-DQB1	HLA-DQB1.value <= threshold=2.1053054332733154
node_2523: feature_name=SNX9	SNX9.value <= threshold=-0.5346486568450928
node_2524: feature_name=HLA-DPB1	HLA-DPB1.value > threshold=1.9548270106315613
node_2814: feature_name=CD79B	CD79B.value > threshold=-0.28186772763729095
node_2826: feature_name=MT-ND6	MT-ND6.value > threshold=0.17273088544607162
node_2872: feature_name=IFI44L	IFI44L.value > threshold=-0.31519079208374023
Class: COVID-19 Critical	

Rules_543	passed counts:8
node_0: feature_name=LTB	LTB.value <= threshold=0.994031548500061
node_1: feature_name=MTRNR2L2	MTRNR2L2.value <= threshold=2.8784297704696655
node_2: feature_name=TCL1A	TCL1A.value <= threshold=4.798312664031982
node_3: feature_name=MT-CO1	MT-CO1.value > threshold=0.08947191759943962
node_2521: feature_name=LTB	LTB.value <= threshold=0.6038667261600494
node_2522: feature_name=HLA-DQB1	HLA-DQB1.value <= threshold=2.1053054332733154
node_2523: feature_name=SNX9	SNX9.value <= threshold=-0.5346486568450928
node_2524: feature_name=HLA-DPB1	HLA-DPB1.value > threshold=1.9548270106315613
node_2814: feature_name=CD79B	CD79B.value > threshold=-0.28186772763729095
node_2826: feature_name=MT-ND6	MT-ND6.value > threshold=0.17273088544607162
node_2872: feature_name=IFI44L	IFI44L.value <= threshold=-0.31519079208374023
node_2873: feature_name=RALGPS2	RALGPS2.value > threshold=1.6798744797706604
node_2887: feature_name=HLA-DQA2	HLA-DQA2.value <= threshold=5.12495756149292
node_2888: feature_name=MS4A1	MS4A1.value > threshold=3.8452248573303223
node_2900: feature_name=NR4A1	NR4A1.value <= threshold=-0.24637621641159058
node_2901: feature_name=GRASP	GRASP.value <= threshold=3.1462806463241577
Class: COVID-19 Critical	
Rules_544	passed counts:8
node_0: feature_name=LTB	LTB.value <= threshold=0.994031548500061
node_1: feature_name=MTRNR2L2	MTRNR2L2.value <= threshold=2.8784297704696655
node_2: feature_name=TCL1A	TCL1A.value <= threshold=4.798312664031982
node_3: feature_name=MT-CO1	MT-CO1.value > threshold=0.08947191759943962
node_2521: feature_name=LTB	LTB.value <= threshold=0.6038667261600494
node_2522: feature_name=HLA-DQB1	HLA-DQB1.value <= threshold=2.1053054332733154
node_2523: feature_name=SNX9	SNX9.value <= threshold=-0.5346486568450928
node_2524: feature_name=HLA-DPB1	HLA-DPB1.value <= threshold=1.9548270106315613
node_2525: feature_name=FAM129C	FAM129C.value > threshold=3.3393388986587524

node_2721: feature_name=ADAM19	ADAM19.value > threshold=-0.29525794088840485
node_2781: feature_name=XAF1	XAF1.value <= threshold=-0.4296249598264694
node_2782: feature_name=CYBB	CYBB.value <= threshold=-0.5005911737680435
node_2783: feature_name=HLA-DQA1	HLA-DQA1.value > threshold=2.0089213848114014
node_2785: feature_name=GPX1	GPX1.value <= threshold=0.2504514306783676
Class: Healthy Control	
Rules_545	passed counts:8
node_0: feature_name=LTB	LTB.value <= threshold=0.994031548500061
node_1: feature_name=MTRNR2L2	MTRNR2L2.value <= threshold=2.8784297704696655
node_2: feature_name=TCL1A	TCL1A.value <= threshold=4.798312664031982
node_3: feature_name=MT-CO1	MT-CO1.value > threshold=0.08947191759943962
node_2521: feature_name=LTB	LTB.value <= threshold=0.6038667261600494
node_2522: feature_name=HLA-DQB1	HLA-DQB1.value <= threshold=2.1053054332733154
node_2523: feature_name=SNX9	SNX9.value <= threshold=-0.5346486568450928
node_2524: feature_name=HLA-DPB1	HLA-DPB1.value <= threshold=1.9548270106315613
node_2525: feature_name=FAM129C	FAM129C.value > threshold=3.3393388986587524
node_2721: feature_name=ADAM19	ADAM19.value <= threshold=-0.29525794088840485
node_2722: feature_name=NR4A1	NR4A1.value <= threshold=2.1566513776779175
node_2723: feature_name=CD22	CD22.value > threshold=8.345317840576172
node_2769: feature_name=BACH1	BACH1.value <= threshold=1.3255683034658432
node_2770: feature_name=HLA-DQB1	HLA-DQB1.value > threshold=-0.3957219868898392
Class: Healthy Control	
Rules_546	passed counts:8
node_0: feature_name=LTB	LTB.value <= threshold=0.994031548500061
node_1: feature_name=MTRNR2L2	MTRNR2L2.value <= threshold=2.8784297704696655
node_2: feature_name=TCL1A	TCL1A.value <= threshold=4.798312664031982
node_3: feature_name=MT-CO1	MT-CO1.value > threshold=0.08947191759943962
node_2521: feature_name=LTB	LTB.value <= threshold=0.6038667261600494

node_2522: feature_name=HLA-DQB1	HLA-DQB1.value <= threshold=2.1053054332733154
node_2523: feature_name=SNX9	SNX9.value <= threshold=-0.5346486568450928
node_2524: feature_name=HLA-DPB1	HLA-DPB1.value <= threshold=1.9548270106315613
node_2525: feature_name=FAM129C	FAM129C.value <= threshold=3.3393388986587524
node_2526: feature_name=XIST	XIST.value <= threshold=3.6911416053771973
node_2527: feature_name=NR4A1	NR4A1.value > threshold=-0.2557755410671234
node_2625: feature_name=HLA-DPA1	HLA-DPA1.value > threshold=0.9335225224494934
node_2657: feature_name=RALGPS2	RALGPS2.value <= threshold=2.9413715600967407
node_2658: feature_name=HLA-DQB1	HLA-DQB1.value > threshold=1.7503233551979065
node_2692: feature_name=MEF2C	MEF2C.value <= threshold=0.2689770758152008
node_2693: feature_name=MT-CO3	MT-CO3.value > threshold=-0.3096682131290436
Class: COVID-19 Critical	
Rules_547	passed counts:8
node_0: feature_name=LTB	LTB.value <= threshold=0.994031548500061
node_1: feature_name=MTRNR2L2	MTRNR2L2.value <= threshold=2.8784297704696655
node_2: feature_name=TCL1A	TCL1A.value <= threshold=4.798312664031982
node_3: feature_name=MT-CO1	MT-CO1.value > threshold=0.08947191759943962
node_2521: feature_name=LTB	LTB.value <= threshold=0.6038667261600494
node_2522: feature_name=HLA-DQB1	HLA-DQB1.value <= threshold=2.1053054332733154
node_2523: feature_name=SNX9	SNX9.value <= threshold=-0.5346486568450928
node_2524: feature_name=HLA-DPB1	HLA-DPB1.value <= threshold=1.9548270106315613
node_2525: feature_name=FAM129C	FAM129C.value <= threshold=3.3393388986587524
node_2526: feature_name=XIST	XIST.value <= threshold=3.6911416053771973
node_2527: feature_name=NR4A1	NR4A1.value > threshold=-0.2557755410671234
node_2625: feature_name=HLA-DPA1	HLA-DPA1.value > threshold=0.9335225224494934
node_2657: feature_name=RALGPS2	RALGPS2.value <= threshold=2.9413715600967407
node_2658: feature_name=HLA-DQB1	HLA-DQB1.value <= threshold=1.7503233551979065
node_2659: feature_name=TP53INP1	TP53INP1.value <= threshold=-0.2649386376142502
node_2660: feature_name=OAZ1	OAZ1.value <= threshold=1.2488974928855896

node_2661: feature_name=HLA-DPB1	HLA-DPB1.value <= threshold=-0.5502273440361023
node_2662: feature_name=CD22	CD22.value > threshold=-0.268435537815094
Class: COVID-19 Critical	
Rules_548	passed counts:8
node_0: feature_name=LTB	LTB.value <= threshold=0.994031548500061
node_1: feature_name=MTRNR2L2	MTRNR2L2.value <= threshold=2.8784297704696655
node_2: feature_name=TCL1A	TCL1A.value <= threshold=4.798312664031982
node_3: feature_name=MT-CO1	MT-CO1.value <= threshold=0.08947191759943962
node_4: feature_name=NR4A1	NR4A1.value > threshold=1.0725348591804504
node_1622: feature_name=HLA-DQA2	HLA-DQA2.value > threshold=1.9496816992759705
node_2350: feature_name=BCL2A1	BCL2A1.value > threshold=1.1322240233421326
node_2466: feature_name=MS4A1	MS4A1.value <= threshold=2.7321707010269165
node_2467: feature_name=HSP90B1	HSP90B1.value <= threshold=0.8215908706188202
node_2468: feature_name=HLA-DQA1	HLA-DQA1.value > threshold=1.4212530851364136
node_2476: feature_name=MT-CO3	MT-CO3.value > threshold=0.05051383376121521
node_2488: feature_name=SPI1	SPI1.value <= threshold=-0.4845922589302063
node_2489: feature_name=CASP8	CASP8.value <= threshold=0.062011122703552246
Class: COVID-19 Critical	
Rules_549	passed counts:8
node_0: feature_name=LTB	LTB.value <= threshold=0.994031548500061
node_1: feature_name=MTRNR2L2	MTRNR2L2.value <= threshold=2.8784297704696655
node_2: feature_name=TCL1A	TCL1A.value <= threshold=4.798312664031982
node_3: feature_name=MT-CO1	MT-CO1.value <= threshold=0.08947191759943962
node_4: feature_name=NR4A1	NR4A1.value > threshold=1.0725348591804504
node_1622: feature_name=HLA-DQA2	HLA-DQA2.value > threshold=1.9496816992759705
node_2350: feature_name=BCL2A1	BCL2A1.value <= threshold=1.1322240233421326
node_2351: feature_name=CD83	CD83.value > threshold=2.3162450790405273
node_2391: feature_name=CD79B	CD79B.value <= threshold=1.7111427783966064

node_2392: feature_name=TTN	TTN.value <= threshold=-0.28553803265094757
node_2393: feature_name=LDLRAD4	LDLRAD4.value > threshold=3.3328956365585327
node_2425: feature_name=IGJ	IGJ.value <= threshold=-0.30049456655979156
Class: COVID-19 Critical	
Rules_550	passed counts:8
node_0: feature_name=LTB	LTB.value <= threshold=0.994031548500061
node_1: feature_name=MTRNR2L2	MTRNR2L2.value <= threshold=2.8784297704696655
node_2: feature_name=TCL1A	TCL1A.value <= threshold=4.798312664031982
node_3: feature_name=MT-CO1	MT-CO1.value <= threshold=0.08947191759943962
node_4: feature_name=NR4A1	NR4A1.value > threshold=1.0725348591804504
node_1622: feature_name=HLA-DQA2	HLA-DQA2.value <= threshold=1.9496816992759705
node_1623: feature_name=HLA-DRB5	HLA-DRB5.value <= threshold=2.715766429901123
node_1624: feature_name=HLA-DPA1	HLA-DPA1.value <= threshold=1.7050301432609558
node_1625: feature_name=CD83	CD83.value > threshold=2.783489227294922
node_1873: feature_name=IFI44L	IFI44L.value <= threshold=1.5938722491264343
node_1874: feature_name=PLD4	PLD4.value <= threshold=4.101001739501953
node_1875: feature_name=RHOB	RHOB.value > threshold=1.304835319519043
node_1965: feature_name=HLA-DQB1	HLA-DQB1.value > threshold=1.5989137291908264
node_1991: feature_name=COTL1	COTL1.value <= threshold=-1.2334879636764526
node_1992: feature_name=HLA-DRB5	HLA-DRB5.value <= threshold=1.1685267686843872
Class: COVID-19 Severe	
Rules_551	passed counts:8
node_0: feature_name=LTB	LTB.value <= threshold=0.994031548500061
node_1: feature_name=MTRNR2L2	MTRNR2L2.value <= threshold=2.8784297704696655
node_2: feature_name=TCL1A	TCL1A.value <= threshold=4.798312664031982
node_3: feature_name=MT-CO1	MT-CO1.value <= threshold=0.08947191759943962
node_4: feature_name=NR4A1	NR4A1.value > threshold=1.0725348591804504
node_1622: feature_name=HLA-DQA2	HLA-DQA2.value <= threshold=1.9496816992759705

node_1623: feature_name=HLA-DRB5	HLA-DRB5.value <= threshold=2.715766429901123
node_1624: feature_name=HLA-DPA1	HLA-DPA1.value <= threshold=1.7050301432609558
node_1625: feature_name=CD83	CD83.value > threshold=2.783489227294922
node_1873: feature_name=IFI44L	IFI44L.value <= threshold=1.5938722491264343
node_1874: feature_name=PLD4	PLD4.value <= threshold=4.101001739501953
node_1875: feature_name=RHOB	RHOB.value <= threshold=1.304835319519043
node_1876: feature_name=PPIF	PPIF.value > threshold=2.2943923473358154
node_1962: feature_name=HLA-DPA1	HLA-DPA1.value > threshold=1.071113646030426
Class: COVID-19 Severe	
Rules_552	passed counts:8
node_0: feature_name=LTB	LTB.value <= threshold=0.994031548500061
node_1: feature_name=MTRNR2L2	MTRNR2L2.value <= threshold=2.8784297704696655
node_2: feature_name=TCL1A	TCL1A.value <= threshold=4.798312664031982
node_3: feature_name=MT-CO1	MT-CO1.value <= threshold=0.08947191759943962
node_4: feature_name=NR4A1	NR4A1.value > threshold=1.0725348591804504
node_1622: feature_name=HLA-DQA2	HLA-DQA2.value <= threshold=1.9496816992759705
node_1623: feature_name=HLA-DRB5	HLA-DRB5.value <= threshold=2.715766429901123
node_1624: feature_name=HLA-DPA1	HLA-DPA1.value <= threshold=1.7050301432609558
node_1625: feature_name=CD83	CD83.value > threshold=2.783489227294922
node_1873: feature_name=IFI44L	IFI44L.value <= threshold=1.5938722491264343
node_1874: feature_name=PLD4	PLD4.value <= threshold=4.101001739501953
node_1875: feature_name=RHOB	RHOB.value <= threshold=1.304835319519043
node_1876: feature_name=PPIF	PPIF.value <= threshold=2.2943923473358154
node_1877: feature_name=OAZ1	OAZ1.value <= threshold=0.04260866530239582
node_1878: feature_name=ID3	ID3.value <= threshold=5.290210247039795
node_1879: feature_name=NFKBID	NFKBID.value > threshold=-0.40532417595386505
node_1883: feature_name=MEF2C	MEF2C.value > threshold=-0.4597593992948532
node_1887: feature_name=SNX9	SNX9.value <= threshold=1.010923683643341
node_1888: feature_name=HLA-DRB5	HLA-DRB5.value > threshold=-0.4844578504562378

node_1890: feature_name=HLA-DPB1	HLA-DPB1.value > threshold=1.4238839745521545
node_1894: feature_name=NFKBID	NFKBID.value <= threshold=2.7626200914382935
node_1895: feature_name=COTL1	COTL1.value <= threshold=0.2644204944372177
Class: COVID-19 Severe	
Rules_553	passed counts:8
node_0: feature_name=LTB	LTB.value <= threshold=0.994031548500061
node_1: feature_name=MTRNR2L2	MTRNR2L2.value <= threshold=2.8784297704696655
node_2: feature_name=TCL1A	TCL1A.value <= threshold=4.798312664031982
node_3: feature_name=MT-CO1	MT-CO1.value <= threshold=0.08947191759943962
node_4: feature_name=NR4A1	NR4A1.value > threshold=1.0725348591804504
node_1622: feature_name=HLA-DQA2	HLA-DQA2.value <= threshold=1.9496816992759705
node_1623: feature_name=HLA-DRB5	HLA-DRB5.value <= threshold=2.715766429901123
node_1624: feature_name=HLA-DPA1	HLA-DPA1.value <= threshold=1.7050301432609558
node_1625: feature_name=CD83	CD83.value <= threshold=2.783489227294922
node_1626: feature_name=XIST	XIST.value > threshold=0.9589543007314205
node_1870: feature_name=BACH1	BACH1.value > threshold=-0.0758613795042038
Class: COVID-19 Severe	
Rules_554	passed counts:8
node_0: feature_name=LTB	LTB.value <= threshold=0.994031548500061
node_1: feature_name=MTRNR2L2	MTRNR2L2.value <= threshold=2.8784297704696655
node_2: feature_name=TCL1A	TCL1A.value <= threshold=4.798312664031982
node_3: feature_name=MT-CO1	MT-CO1.value <= threshold=0.08947191759943962
node_4: feature_name=NR4A1	NR4A1.value > threshold=1.0725348591804504
node_1622: feature_name=HLA-DQA2	HLA-DQA2.value <= threshold=1.9496816992759705
node_1623: feature_name=HLA-DRB5	HLA-DRB5.value <= threshold=2.715766429901123
node_1624: feature_name=HLA-DPA1	HLA-DPA1.value <= threshold=1.7050301432609558
node_1625: feature_name=CD83	CD83.value <= threshold=2.783489227294922
node_1626: feature_name=XIST	XIST.value <= threshold=0.9589543007314205

node_1627: feature_name=PLD4	PLD4.value <= threshold=5.865932464599609
node_1628: feature_name=OAZ1	OAZ1.value <= threshold=0.6137169003486633
node_1629: feature_name=MTRNR2L2	MTRNR2L2.value <= threshold=1.6102792620658875
node_1630: feature_name=IFI6	IFI6.value > threshold=-0.8404486179351807
node_1632: feature_name=NR4A1	NR4A1.value > threshold=1.0916320085525513
node_1638: feature_name=DENND3	DENND3.value <= threshold=3.988452672958374
node_1639: feature_name=HLA-DRB1	HLA-DRB1.value <= threshold=1.4092575907707214
node_1640: feature_name=HLA-DPA1	HLA-DPA1.value > threshold=1.2675696015357971
node_1708: feature_name=CYBB	CYBB.value <= threshold=1.8550206422805786
node_1709: feature_name=JUN	JUN.value <= threshold=-0.04836222808808088
node_1710: feature_name=MARCH1	MARCH1.value <= threshold=-0.3826435059309006
node_1711: feature_name=MT-CO3	MT-CO3.value <= threshold=-0.03930339030921459
Class: COVID-19 Severe	
Rules_555	passed counts:8
node_0: feature_name=LTB	LTB.value <= threshold=0.994031548500061
node_1: feature_name=MTRNR2L2	MTRNR2L2.value <= threshold=2.8784297704696655
node_2: feature_name=TCL1A	TCL1A.value <= threshold=4.798312664031982
node_3: feature_name=MT-CO1	MT-CO1.value <= threshold=0.08947191759943962
node_4: feature_name=NR4A1	NR4A1.value <= threshold=1.0725348591804504
node_5: feature_name=HLA-DPA1	HLA-DPA1.value > threshold=1.6885469555854797
node_861: feature_name=CD83	CD83.value <= threshold=3.0563642978668213
node_862: feature_name=HLA-DQB1	HLA-DQB1.value <= threshold=2.61081063747406
node_863: feature_name=MTRNR2L2	MTRNR2L2.value <= threshold=2.0919997692108154
node_864: feature_name=BCL2A1	BCL2A1.value > threshold=1.852618932723999
node_1328: feature_name=ATF5	ATF5.value > threshold=1.995988368988037
node_1350: feature_name=NAMPT	NAMPT.value > threshold=-0.5354086756706238
Class: COVID-19 Severe	
Rules_556	passed counts:8

node_0: feature_name=LTB	LTB.value <= threshold=0.994031548500061
node_1: feature_name=MTRNR2L2	MTRNR2L2.value <= threshold=2.8784297704696655
node_2: feature_name=TCL1A	TCL1A.value <= threshold=4.798312664031982
node_3: feature_name=MT-CO1	MT-CO1.value <= threshold=0.08947191759943962
node_4: feature_name=NR4A1	NR4A1.value <= threshold=1.0725348591804504
node_5: feature_name=HLA-DPA1	HLA-DPA1.value > threshold=1.6885469555854797
node_861: feature_name=CD83	CD83.value <= threshold=3.0563642978668213
node_862: feature_name=HLA-DQB1	HLA-DQB1.value <= threshold=2.61081063747406
node_863: feature_name=MTRNR2L2	MTRNR2L2.value <= threshold=2.0919997692108154
node_864: feature_name=BCL2A1	BCL2A1.value <= threshold=1.852618932723999
node_865: feature_name=CD79B	CD79B.value <= threshold=4.352142333984375
node_866: feature_name=TNFAIP3	TNFAIP3.value > threshold=-0.0449496041983366
node_1236: feature_name=HLA-DRB5	HLA-DRB5.value > threshold=1.710871696472168
node_1268: feature_name=MT-CO1	MT-CO1.value > threshold=-0.8004095554351807
node_1282: feature_name=HLA-DQA2	HLA-DQA2.value <= threshold=-0.1890905275940895
node_1283: feature_name=OAZ1	OAZ1.value <= threshold=-0.14406292885541916
node_1284: feature_name=ACSL1	ACSL1.value <= threshold=-0.29594099521636963
Class: COVID-19 Severe	
Rules_557	passed counts:8
node_0: feature_name=LTB	LTB.value <= threshold=0.994031548500061
node_1: feature_name=MTRNR2L2	MTRNR2L2.value <= threshold=2.8784297704696655
node_2: feature_name=TCL1A	TCL1A.value <= threshold=4.798312664031982
node_3: feature_name=MT-CO1	MT-CO1.value <= threshold=0.08947191759943962
node_4: feature_name=NR4A1	NR4A1.value <= threshold=1.0725348591804504
node_5: feature_name=HLA-DPA1	HLA-DPA1.value > threshold=1.6885469555854797
node_861: feature_name=CD83	CD83.value <= threshold=3.0563642978668213
node_862: feature_name=HLA-DQB1	HLA-DQB1.value <= threshold=2.61081063747406
node_863: feature_name=MTRNR2L2	MTRNR2L2.value <= threshold=2.0919997692108154
node_864: feature_name=BCL2A1	BCL2A1.value <= threshold=1.852618932723999

node_865: feature_name=CD79B	CD79B.value <= threshold=4.352142333984375
node_866: feature_name=TNFAIP3	TNFAIP3.value > threshold=-0.0449496041983366
node_1236: feature_name=HLA-DRB5	HLA-DRB5.value <= threshold=1.710871696472168
node_1237: feature_name=HLA-DQA2	HLA-DQA2.value <= threshold=3.6443899869918823
node_1238: feature_name=FAM160B1	FAM160B1.value > threshold=-0.36028963327407837
node_1256: feature_name=RGS2	RGS2.value > threshold=-0.6350596249103546
node_1258: feature_name=XAF1	XAF1.value <= threshold=-0.4413585215806961
Class: COVID-19 Critical	
Rules_558	passed counts:8
node_0: feature_name=LTB	LTB.value <= threshold=0.994031548500061
node_1: feature_name=MTRNR2L2	MTRNR2L2.value <= threshold=2.8784297704696655
node_2: feature_name=TCL1A	TCL1A.value <= threshold=4.798312664031982
node_3: feature_name=MT-CO1	MT-CO1.value <= threshold=0.08947191759943962
node_4: feature_name=NR4A1	NR4A1.value <= threshold=1.0725348591804504
node_5: feature_name=HLA-DPA1	HLA-DPA1.value > threshold=1.6885469555854797
node_861: feature_name=CD83	CD83.value <= threshold=3.0563642978668213
node_862: feature_name=HLA-DQB1	HLA-DQB1.value <= threshold=2.61081063747406
node_863: feature_name=MTRNR2L2	MTRNR2L2.value <= threshold=2.0919997692108154
node_864: feature_name=BCL2A1	BCL2A1.value <= threshold=1.852618932723999
node_865: feature_name=CD79B	CD79B.value <= threshold=4.352142333984375
node_866: feature_name=TNFAIP3	TNFAIP3.value <= threshold=-0.0449496041983366
node_867: feature_name=MT-CO1	MT-CO1.value > threshold=-0.08531957119703293
node_1155: feature_name=IRF8	IRF8.value > threshold=-0.39239931106567383
node_1177: feature_name=PELI1	PELI1.value <= threshold=-0.6234017610549927
node_1178: feature_name=HLA-DRB1	HLA-DRB1.value > threshold=1.3807418942451477
node_1182: feature_name=ANO6	ANO6.value <= threshold=-0.3352542072534561
node_1183: feature_name=MS4A1	MS4A1.value <= threshold=1.8863030076026917
node_1184: feature_name=FERMT3	FERMT3.value <= threshold=0.5012311637401581
Class: COVID-19 Critical	

Rules_559	passed counts:8
node_0: feature_name=LTB	LTB.value <= threshold=0.994031548500061
node_1: feature_name=MTRNR2L2	MTRNR2L2.value <= threshold=2.8784297704696655
node_2: feature_name=TCL1A	TCL1A.value <= threshold=4.798312664031982
node_3: feature_name=MT-CO1	MT-CO1.value <= threshold=0.08947191759943962
node_4: feature_name=NR4A1	NR4A1.value <= threshold=1.0725348591804504
node_5: feature_name=HLA-DPA1	HLA-DPA1.value > threshold=1.6885469555854797
node_861: feature_name=CD83	CD83.value <= threshold=3.0563642978668213
node_862: feature_name=HLA-DQB1	HLA-DQB1.value <= threshold=2.61081063747406
node_863: feature_name=MTRNR2L2	MTRNR2L2.value <= threshold=2.0919997692108154
node_864: feature_name=BCL2A1	BCL2A1.value <= threshold=1.852618932723999
node_865: feature_name=CD79B	CD79B.value <= threshold=4.352142333984375
node_866: feature_name=TNFAIP3	TNFAIP3.value <= threshold=-0.0449496041983366
node_867: feature_name=MT-CO1	MT-CO1.value <= threshold=-0.08531957119703293
node_868: feature_name=MTRNR2L8	MTRNR2L8.value <= threshold=0.8770350813865662
node_869: feature_name=EGR1	EGR1.value > threshold=-0.32270750403404236
node_1015: feature_name=NR4A3	NR4A3.value <= threshold=1.9085431098937988
node_1016: feature_name=ID3	ID3.value <= threshold=2.776647686958313
node_1017: feature_name=XIST	XIST.value <= threshold=2.618046522140503
node_1018: feature_name=CD79B	CD79B.value > threshold=3.818994164466858
node_1120: feature_name=NT5C3A	NT5C3A.value > threshold=-0.5003696829080582
node_1122: feature_name=SPI1	SPI1.value <= threshold=0.4639028459787369
Class: Healthy Control	
Rules_560	passed counts:8
node_0: feature_name=LTB	LTB.value <= threshold=0.994031548500061
node_1: feature_name=MTRNR2L2	MTRNR2L2.value <= threshold=2.8784297704696655
node_2: feature_name=TCL1A	TCL1A.value <= threshold=4.798312664031982
node_3: feature_name=MT-CO1	MT-CO1.value <= threshold=0.08947191759943962

node_4: feature_name=NR4A1	NR4A1.value <= threshold=1.0725348591804504
node_5: feature_name=HLA-DPA1	HLA-DPA1.value > threshold=1.6885469555854797
node_861: feature_name=CD83	CD83.value <= threshold=3.0563642978668213
node_862: feature_name=HLA-DQB1	HLA-DQB1.value <= threshold=2.61081063747406
node_863: feature_name=MTRNR2L2	MTRNR2L2.value <= threshold=2.0919997692108154
node_864: feature_name=BCL2A1	BCL2A1.value <= threshold=1.852618932723999
node_865: feature_name=CD79B	CD79B.value <= threshold=4.352142333984375
node_866: feature_name=TNFAIP3	TNFAIP3.value <= threshold=-0.0449496041983366
node_867: feature_name=MT-CO1	MT-CO1.value <= threshold=-0.08531957119703293
node_868: feature_name=MTRNR2L8	MTRNR2L8.value <= threshold=0.8770350813865662
node_869: feature_name=EGR1	EGR1.value <= threshold=-0.32270750403404236
node_870: feature_name=IGJ	IGJ.value <= threshold=4.618318557739258
node_871: feature_name=DUSP4	DUSP4.value <= threshold=2.8197922706604004
node_872: feature_name=HOPX	HOPX.value <= threshold=2.10397732257843
node_873: feature_name=RGCC	RGCC.value <= threshold=1.2070720195770264
node_874: feature_name=OAZ1	OAZ1.value <= threshold=1.02116060256958
node_875: feature_name=NAIP	NAIP.value <= threshold=3.768904685974121
node_876: feature_name=LDLR	LDLR.value <= threshold=2.9582698345184326
node_877: feature_name=HLA-DPA1	HLA-DPA1.value <= threshold=3.0588693618774414
node_878: feature_name=RHOB	RHOB.value <= threshold=5.08755898475647
node_879: feature_name=SYTL3	SYTL3.value <= threshold=1.5127440690994263
node_880: feature_name=FTH1	FTH1.value > threshold=-1.4152275919914246
node_882: feature_name=CCL3	CCL3.value <= threshold=2.253131687641144
node_883: feature_name=CD83	CD83.value <= threshold=2.8566592931747437
node_884: feature_name=HLA-DRB5	HLA-DRB5.value > threshold=-0.6464254856109619
node_888: feature_name=HSP90B1	HSP90B1.value > threshold=-1.186358630657196
node_892: feature_name=ATG2A	ATG2A.value <= threshold=2.3624722957611084
node_893: feature_name=ICAM1	ICAM1.value <= threshold=2.4306468963623047
node_894: feature_name=CD38	CD38.value <= threshold=3.4977954626083374
node_895: feature_name=HLA-DRB1	HLA-DRB1.value > threshold=0.28603747487068176

node_899: feature_name=XIST	XIST.value <= threshold=1.4272559583187103
node_900: feature_name=BANK1	BANK1.value <= threshold=5.060973644256592
node_901: feature_name=IRF4	IRF4.value <= threshold=6.117950916290283
node_902: feature_name=MTRNR2L2	MTRNR2L2.value <= threshold=1.8410876989364624
node_903: feature_name=TPM1	TPM1.value <= threshold=4.590091228485107
node_904: feature_name=SNORD3B-2	SNORD3B-2.value <= threshold=4.349201917648315
node_905: feature_name=AHR	AHR.value <= threshold=2.268427610397339
node_906: feature_name=OAZ1	OAZ1.value > threshold=-1.4369714260101318
node_914: feature_name=MT-ND6	MT-ND6.value <= threshold=1.7712779641151428
node_915: feature_name=HLA-DPB1	HLA-DPB1.value <= threshold=2.6004384756088257
node_916: feature_name=COTL1	COTL1.value <= threshold=-1.1624112129211426
node_917: feature_name=PLD4	PLD4.value <= threshold=4.473680734634399
Class: COVID-19 Critical	
Rules_561	passed counts:8
node_0: feature_name=LTB	LTB.value <= threshold=0.994031548500061
node_1: feature_name=MTRNR2L2	MTRNR2L2.value <= threshold=2.8784297704696655
node_2: feature_name=TCL1A	TCL1A.value <= threshold=4.798312664031982
node_3: feature_name=MT-CO1	MT-CO1.value <= threshold=0.08947191759943962
node_4: feature_name=NR4A1	NR4A1.value <= threshold=1.0725348591804504
node_5: feature_name=HLA-DPA1	HLA-DPA1.value <= threshold=1.6885469555854797
node_6: feature_name=HLA-DRB5	HLA-DRB5.value <= threshold=2.1886194944381714
node_7: feature_name=XIST	XIST.value <= threshold=-0.12328458577394485
node_8: feature_name=SYNE1	SYNE1.value > threshold=-0.7835594415664673
node_16: feature_name=MTRNR2L2	MTRNR2L2.value <= threshold=2.4483247995376587
node_17: feature_name=HLA-DQA2	HLA-DQA2.value > threshold=2.227178931236267
node_591: feature_name=BCL2A1	BCL2A1.value <= threshold=1.0648428201675415
node_592: feature_name=FTH1	FTH1.value > threshold=0.8842596709728241
node_680: feature_name=PELI1	PELI1.value <= threshold=0.20563310384750366
node_681: feature_name=CD83	CD83.value <= threshold=2.8830333948135376

Class: COVID-19 Critical	
Rules_562	passed counts:8
node_0: feature_name=LTB	LTB.value <= threshold=0.994031548500061
node_1: feature_name=MTRNR2L2	MTRNR2L2.value <= threshold=2.8784297704696655
node_2: feature_name=TCL1A	TCL1A.value <= threshold=4.798312664031982
node_3: feature_name=MT-CO1	MT-CO1.value <= threshold=0.08947191759943962
node_4: feature_name=NR4A1	NR4A1.value <= threshold=1.0725348591804504
node_5: feature_name=HLA-DPA1	HLA-DPA1.value <= threshold=1.6885469555854797
node_6: feature_name=HLA-DRB5	HLA-DRB5.value <= threshold=2.1886194944381714
node_7: feature_name=XIST	XIST.value <= threshold=-0.12328458577394485
node_8: feature_name=SYNE1	SYNE1.value > threshold=-0.7835594415664673
node_16: feature_name=MTRNR2L2	MTRNR2L2.value <= threshold=2.4483247995376587
node_17: feature_name=HLA-DQA2	HLA-DQA2.value <= threshold=2.227178931236267
node_18: feature_name=NR4A1	NR4A1.value <= threshold=-0.21380723267793655
node_19: feature_name=IRAK3	IRAK3.value <= threshold=4.27159571647644
node_20: feature_name=IFIT2	IFIT2.value <= threshold=7.8175740242004395
node_21: feature_name=CD79A	CD79A.value > threshold=2.857242226600647
node_375: feature_name=LTB	LTB.value <= threshold=0.7405790388584137
node_376: feature_name=NFKBIZ	NFKBIZ.value <= threshold=1.7718515396118164
node_377: feature_name=OAZ1	OAZ1.value <= threshold=1.3302325010299683
node_378: feature_name=RHOB	RHOB.value <= threshold=3.0459729433059692
node_379: feature_name=MT-CO3	MT-CO3.value <= threshold=1.6619641780853271
node_380: feature_name=MTRNR2L2	MTRNR2L2.value <= threshold=2.1229071617126465
node_381: feature_name=MAP3K2	MAP3K2.value <= threshold=3.626914143562317
node_382: feature_name=LDLR	LDLR.value <= threshold=2.571519374847412
node_383: feature_name=HLA-DRB1	HLA-DRB1.value > threshold=-0.7626054286956787
node_385: feature_name=CD79A	CD79A.value > threshold=2.857717990875244
node_387: feature_name=FCRL1	FCRL1.value <= threshold=6.850295543670654
node_388: feature_name=PDLIM1	PDLIM1.value <= threshold=4.4097795486450195

node_389: feature_name=MARCH1	MARCH1.value > threshold=-0.35520173609256744
node_413: feature_name=RP11-731F5.2	RP11-731F5.2.value > threshold=6.423685789108276
node_439: feature_name=MTRNR2L2	MTRNR2L2.value <= threshold=0.3647138476371765
Class: COVID-19 Critical	
Rules_563	passed counts:7
node_0: feature_name=LTB	LTB.value > threshold=0.994031548500061
node_5264: feature_name=MT-CO1	MT-CO1.value > threshold=-0.4127991199493408
node_5638: feature_name=OAZ1	OAZ1.value > threshold=-0.2640867084264755
node_6112: feature_name=IFI44L	IFI44L.value > threshold=2.5893263816833496
node_7078: feature_name=XIST	XIST.value <= threshold=1.1465829275548458
node_7079: feature_name=TCL1A	TCL1A.value <= threshold=7.115930080413818
node_7080: feature_name=XAF1	XAF1.value <= threshold=-0.45034870505332947
node_7081: feature_name=NFKBID	NFKBID.value <= threshold=1.3513729572296143
node_7082: feature_name=COTL1	COTL1.value > threshold=-1.377109944820404
Class: Healthy Control	
Rules_564	passed counts:7
node_0: feature_name=LTB	LTB.value > threshold=0.994031548500061
node_5264: feature_name=MT-CO1	MT-CO1.value > threshold=-0.4127991199493408
node_5638: feature_name=OAZ1	OAZ1.value > threshold=-0.2640867084264755
node_6112: feature_name=IFI44L	IFI44L.value <= threshold=2.5893263816833496
node_6113: feature_name=TCL1A	TCL1A.value <= threshold=4.201832294464111
node_6114: feature_name=HLA-DQA2	HLA-DQA2.value > threshold=-0.2270243540406227
node_6542: feature_name=CD79B	CD79B.value > threshold=3.096232771873474
node_6872: feature_name=ICAM1	ICAM1.value <= threshold=-0.2232399433851242
node_6873: feature_name=STAG3	STAG3.value > threshold=-0.19312061369419098
node_6939: feature_name=HLA-DRB5	HLA-DRB5.value <= threshold=2.209904909133911
node_6940: feature_name=STAG3	STAG3.value <= threshold=7.8730878829956055
node_6941: feature_name=FAM129C	FAM129C.value <= threshold=1.668714702129364

node_6942: feature_name=HLA-DPB1	HLA-DPB1.value <= threshold=1.4723529815673828
Class: COVID-19 Critical	
Rules_565	passed counts:7
node_0: feature_name=LTB	LTB.value > threshold=0.994031548500061
node_5264: feature_name=MT-CO1	MT-CO1.value > threshold=-0.4127991199493408
node_5638: feature_name=OAZ1	OAZ1.value > threshold=-0.2640867084264755
node_6112: feature_name=IFI44L	IFI44L.value <= threshold=2.5893263816833496
node_6113: feature_name=TCL1A	TCL1A.value <= threshold=4.201832294464111
node_6114: feature_name=HLA-DQA2	HLA-DQA2.value > threshold=-0.2270243540406227
node_6542: feature_name=CD79B	CD79B.value > threshold=3.096232771873474
node_6872: feature_name=ICAM1	ICAM1.value <= threshold=-0.2232399433851242
node_6873: feature_name=STAG3	STAG3.value <= threshold=-0.19312061369419098
node_6874: feature_name=CD79A	CD79A.value > threshold=1.57558012008667
node_6880: feature_name=MEF2A	MEF2A.value > threshold=2.676826596260071
node_6932: feature_name=FAM129C	FAM129C.value <= threshold=2.9109067916870117
Class: COVID-19 Critical	
Rules_566	passed counts:7
node_0: feature_name=LTB	LTB.value > threshold=0.994031548500061
node_5264: feature_name=MT-CO1	MT-CO1.value > threshold=-0.4127991199493408
node_5638: feature_name=OAZ1	OAZ1.value > threshold=-0.2640867084264755
node_6112: feature_name=IFI44L	IFI44L.value <= threshold=2.5893263816833496
node_6113: feature_name=TCL1A	TCL1A.value <= threshold=4.201832294464111
node_6114: feature_name=HLA-DQA2	HLA-DQA2.value > threshold=-0.2270243540406227
node_6542: feature_name=CD79B	CD79B.value <= threshold=3.096232771873474
node_6543: feature_name=NR4A1	NR4A1.value <= threshold=1.421015739440918
node_6544: feature_name=LTB	LTB.value <= threshold=1.359755277633667
node_6545: feature_name=NFKBID	NFKBID.value > threshold=1.4294711351394653
node_6643: feature_name=FAM129C	FAM129C.value <= threshold=4.799884080886841

node_6644: feature_name=XIST	XIST.value > threshold=0.9931697957217693
node_6706: feature_name=MT-CO3	MT-CO3.value > threshold=-0.2717718631029129
Class: Healthy Control	
Rules_567	passed counts:7
node_0: feature_name=LTB	LTB.value > threshold=0.994031548500061
node_5264: feature_name=MT-CO1	MT-CO1.value > threshold=-0.4127991199493408
node_5638: feature_name=OAZ1	OAZ1.value > threshold=-0.2640867084264755
node_6112: feature_name=IFI44L	IFI44L.value <= threshold=2.5893263816833496
node_6113: feature_name=TCL1A	TCL1A.value <= threshold=4.201832294464111
node_6114: feature_name=HLA-DQA2	HLA-DQA2.value <= threshold=-0.2270243540406227
node_6115: feature_name=MTRNR2L2	MTRNR2L2.value <= threshold=2.548917293548584
node_6116: feature_name=ATF5	ATF5.value <= threshold=2.041263461112976
node_6117: feature_name=JUN	JUN.value > threshold=-0.616259902715683
node_6189: feature_name=MT-CO3	MT-CO3.value > threshold=0.13483521342277527
node_6351: feature_name=XAF1	XAF1.value <= threshold=3.061717987060547
node_6352: feature_name=MT-CO2	MT-CO2.value <= threshold=1.2605181336402893
node_6353: feature_name=ICAM1	ICAM1.value <= threshold=-0.3340446650981903
node_6354: feature_name=OAZ1	OAZ1.value > threshold=-0.24055946618318558
node_6360: feature_name=PPIF	PPIF.value <= threshold=2.070030093193054
node_6361: feature_name=RALGPS2	RALGPS2.value > threshold=-0.3684315085411072
node_6369: feature_name=ANO6	ANO6.value <= threshold=3.504153251647949
node_6370: feature_name=YWHAH	YWHAH.value <= threshold=4.3182291984558105
node_6371: feature_name=LDLR	LDLR.value <= threshold=2.494291305541992
node_6372: feature_name=HLA-DQA1	HLA-DQA1.value > threshold=-0.5716990232467651
node_6374: feature_name=ABCA1	ABCA1.value <= threshold=3.7662240266799927
node_6375: feature_name=MS4A1	MS4A1.value > threshold=1.4995208978652954
node_6387: feature_name=SNORD3B-2	SNORD3B-2.value <= threshold=4.154061555862427
node_6388: feature_name=LTB	LTB.value <= threshold=2.1739100217819214
node_6389: feature_name=BACH1	BACH1.value <= threshold=2.0371285676956177

node_6390: feature_name=HLA-DQB1	HLA-DQB1.value <= threshold=3.0258322954177856
node_6391: feature_name=HLA-DPB1	HLA-DPB1.value > threshold=0.2591289207339287
node_6395: feature_name=CXorf57	CXorf57.value > threshold=3.3742183446884155
node_6419: feature_name=HSP90B1	HSP90B1.value <= threshold=0.9682992100715637
Class: Healthy Control	
Rules_568	passed counts:7
node_0: feature_name=LTB	LTB.value > threshold=0.994031548500061
node_5264: feature_name=MT-CO1	MT-CO1.value > threshold=-0.4127991199493408
node_5638: feature_name=OAZ1	OAZ1.value > threshold=-0.2640867084264755
node_6112: feature_name=IFI44L	IFI44L.value <= threshold=2.5893263816833496
node_6113: feature_name=TCL1A	TCL1A.value <= threshold=4.201832294464111
node_6114: feature_name=HLA-DQA2	HLA-DQA2.value <= threshold=-0.2270243540406227
node_6115: feature_name=MTRNR2L2	MTRNR2L2.value <= threshold=2.548917293548584
node_6116: feature_name=ATF5	ATF5.value <= threshold=2.041263461112976
node_6117: feature_name=JUN	JUN.value > threshold=-0.616259902715683
node_6189: feature_name=MT-CO3	MT-CO3.value > threshold=0.13483521342277527
node_6351: feature_name=XAF1	XAF1.value <= threshold=3.061717987060547
node_6352: feature_name=MT-CO2	MT-CO2.value <= threshold=1.2605181336402893
node_6353: feature_name=ICAM1	ICAM1.value <= threshold=-0.3340446650981903
node_6354: feature_name=OAZ1	OAZ1.value > threshold=-0.24055946618318558
node_6360: feature_name=PPIF	PPIF.value <= threshold=2.070030093193054
node_6361: feature_name=RALGPS2	RALGPS2.value > threshold=-0.3684315085411072
node_6369: feature_name=ANO6	ANO6.value <= threshold=3.504153251647949
node_6370: feature_name=YWHAH	YWHAH.value <= threshold=4.3182291984558105
node_6371: feature_name=LDLR	LDLR.value <= threshold=2.494291305541992
node_6372: feature_name=HLA-DQA1	HLA-DQA1.value > threshold=-0.5716990232467651
node_6374: feature_name=ABCA1	ABCA1.value <= threshold=3.7662240266799927
node_6375: feature_name=MS4A1	MS4A1.value > threshold=1.4995208978652954
node_6387: feature_name=SNORD3B-2	SNORD3B-2.value <= threshold=4.154061555862427

node_6388: feature_name=LTB	LTB.value <= threshold=2.1739100217819214
node_6389: feature_name=BACH1	BACH1.value <= threshold=2.0371285676956177
node_6390: feature_name=HLA-DQB1	HLA-DQB1.value <= threshold=3.0258322954177856
node_6391: feature_name=HLA-DPB1	HLA-DPB1.value <= threshold=0.2591289207339287
node_6392: feature_name=SNX9	SNX9.value <= threshold=0.08137500286102295
Class: Healthy Control	
Rules_569	passed counts:7
node_0: feature_name=LTB	LTB.value > threshold=0.994031548500061
node_5264: feature_name=MT-CO1	MT-CO1.value > threshold=-0.4127991199493408
node_5638: feature_name=OAZ1	OAZ1.value > threshold=-0.2640867084264755
node_6112: feature_name=IFI44L	IFI44L.value <= threshold=2.5893263816833496
node_6113: feature_name=TCL1A	TCL1A.value <= threshold=4.201832294464111
node_6114: feature_name=HLA-DQA2	HLA-DQA2.value <= threshold=-0.2270243540406227
node_6115: feature_name=MTRNR2L2	MTRNR2L2.value <= threshold=2.548917293548584
node_6116: feature_name=ATF5	ATF5.value <= threshold=2.041263461112976
node_6117: feature_name=JUN	JUN.value > threshold=-0.616259902715683
node_6189: feature_name=MT-CO3	MT-CO3.value <= threshold=0.13483521342277527
node_6190: feature_name=XAF1	XAF1.value <= threshold=2.584952235221863
node_6191: feature_name=HLA-DRB5	HLA-DRB5.value > threshold=0.6955901980400085
node_6239: feature_name=TNFAIP3	TNFAIP3.value <= threshold=0.11930759996175766
node_6240: feature_name=TP53INP1	TP53INP1.value > threshold=2.9654784202575684
node_6330: feature_name=LTB	LTB.value <= threshold=1.14329594373703
node_6331: feature_name=SNX9	SNX9.value > threshold=-0.5529437959194183
Class: COVID-19 Critical	
Rules_570	passed counts:7
node_0: feature_name=LTB	LTB.value > threshold=0.994031548500061
node_5264: feature_name=MT-CO1	MT-CO1.value > threshold=-0.4127991199493408
node_5638: feature_name=OAZ1	OAZ1.value > threshold=-0.2640867084264755

node_6112: feature_name=IFI44L	IFI44L.value <= threshold=2.5893263816833496
node_6113: feature_name=TCL1A	TCL1A.value <= threshold=4.201832294464111
node_6114: feature_name=HLA-DQA2	HLA-DQA2.value <= threshold=-0.2270243540406227
node_6115: feature_name=MTRNR2L2	MTRNR2L2.value <= threshold=2.548917293548584
node_6116: feature_name=ATF5	ATF5.value <= threshold=2.041263461112976
node_6117: feature_name=JUN	JUN.value > threshold=-0.616259902715683
node_6189: feature_name=MT-CO3	MT-CO3.value <= threshold=0.13483521342277527
node_6190: feature_name=XAF1	XAF1.value <= threshold=2.584952235221863
node_6191: feature_name=HLA-DRB5	HLA-DRB5.value <= threshold=0.6955901980400085
node_6192: feature_name=NR4A1	NR4A1.value <= threshold=-0.4496448189020157
node_6193: feature_name=HLA-DPB1	HLA-DPB1.value <= threshold=1.7609722018241882
node_6194: feature_name=JUN	JUN.value > threshold=1.172904908657074
Class: Healthy Control	
Rules_571	passed counts:7
node_0: feature_name=LTB	LTB.value > threshold=0.994031548500061
node_5264: feature_name=MT-CO1	MT-CO1.value > threshold=-0.4127991199493408
node_5638: feature_name=OAZ1	OAZ1.value > threshold=-0.2640867084264755
node_6112: feature_name=IFI44L	IFI44L.value <= threshold=2.5893263816833496
node_6113: feature_name=TCL1A	TCL1A.value <= threshold=4.201832294464111
node_6114: feature_name=HLA-DQA2	HLA-DQA2.value <= threshold=-0.2270243540406227
node_6115: feature_name=MTRNR2L2	MTRNR2L2.value <= threshold=2.548917293548584
node_6116: feature_name=ATF5	ATF5.value <= threshold=2.041263461112976
node_6117: feature_name=JUN	JUN.value > threshold=-0.616259902715683
node_6189: feature_name=MT-CO3	MT-CO3.value <= threshold=0.13483521342277527
node_6190: feature_name=XAF1	XAF1.value <= threshold=2.584952235221863
node_6191: feature_name=HLA-DRB5	HLA-DRB5.value <= threshold=0.6955901980400085
node_6192: feature_name=NR4A1	NR4A1.value <= threshold=-0.4496448189020157
node_6193: feature_name=HLA-DPB1	HLA-DPB1.value <= threshold=1.7609722018241882
node_6194: feature_name=JUN	JUN.value <= threshold=1.172904908657074

node_6195: feature_name=MT-ND6	MT-ND6.value > threshold=0.6287261247634888
node_6207: feature_name=RGCC	RGCC.value > threshold=-0.8717423379421234
Class: COVID-19 Critical	
Rules_572	passed counts:7
node_0: feature_name=LTB	LTB.value > threshold=0.994031548500061
node_5264: feature_name=MT-CO1	MT-CO1.value > threshold=-0.4127991199493408
node_5638: feature_name=OAZ1	OAZ1.value <= threshold=-0.2640867084264755
node_5639: feature_name=CD79B	CD79B.value > threshold=2.2998517751693726
node_5925: feature_name=SNX9	SNX9.value > threshold=2.4445992708206177
node_6083: feature_name=RALGPS2	RALGPS2.value <= threshold=4.061077356338501
node_6084: feature_name=HLA-DQB1	HLA-DQB1.value > threshold=2.593139410018921
node_6096: feature_name=WDR74	WDR74.value > threshold=-0.4895023852586746
node_6100: feature_name=RALGPS2	RALGPS2.value <= threshold=3.096191167831421
Class: COVID-19 Critical	
Rules_573	passed counts:7
node_0: feature_name=LTB	LTB.value > threshold=0.994031548500061
node_5264: feature_name=MT-CO1	MT-CO1.value > threshold=-0.4127991199493408
node_5638: feature_name=OAZ1	OAZ1.value <= threshold=-0.2640867084264755
node_5639: feature_name=CD79B	CD79B.value > threshold=2.2998517751693726
node_5925: feature_name=SNX9	SNX9.value > threshold=2.4445992708206177
node_6083: feature_name=RALGPS2	RALGPS2.value <= threshold=4.061077356338501
node_6084: feature_name=HLA-DQB1	HLA-DQB1.value > threshold=2.593139410018921
node_6096: feature_name=WDR74	WDR74.value <= threshold=-0.4895023852586746
node_6097: feature_name=DUSP4	DUSP4.value > threshold=-0.26298217475414276
Class: COVID-19 Severe	
Rules_574	passed counts:7
node_0: feature_name=LTB	LTB.value > threshold=0.994031548500061

node_5264: feature_name=MT-CO1	MT-CO1.value > threshold=-0.4127991199493408
node_5638: feature_name=OAZ1	OAZ1.value <= threshold=-0.2640867084264755
node_5639: feature_name=CD79B	CD79B.value > threshold=2.2998517751693726
node_5925: feature_name=SNX9	SNX9.value <= threshold=2.4445992708206177
node_5926: feature_name=TCL1A	TCL1A.value <= threshold=2.6938670873641968
node_5927: feature_name=PLD4	PLD4.value > threshold=-0.06434785947203636
node_6009: feature_name=MX1	MX1.value > threshold=3.600893020629883
Class: COVID-19 Critical	
Rules_575	passed counts:7
node_0: feature_name=LTB	LTB.value > threshold=0.994031548500061
node_5264: feature_name=MT-CO1	MT-CO1.value > threshold=-0.4127991199493408
node_5638: feature_name=OAZ1	OAZ1.value <= threshold=-0.2640867084264755
node_5639: feature_name=CD79B	CD79B.value > threshold=2.2998517751693726
node_5925: feature_name=SNX9	SNX9.value <= threshold=2.4445992708206177
node_5926: feature_name=TCL1A	TCL1A.value <= threshold=2.6938670873641968
node_5927: feature_name=PLD4	PLD4.value > threshold=-0.06434785947203636
node_6009: feature_name=MX1	MX1.value <= threshold=3.600893020629883
node_6010: feature_name=PRDM1	PRDM1.value <= threshold=-0.407244011759758
node_6011: feature_name=SNORD3B-2	SNORD3B-2.value <= threshold=2.7082353830337524
node_6012: feature_name=NFKBID	NFKBID.value <= threshold=3.0716724395751953
node_6013: feature_name=HLA-DQA2	HLA-DQA2.value > threshold=3.7589778900146484
node_6031: feature_name=SNX9	SNX9.value <= threshold=-0.5354251265525818
Class: Healthy Control	
Rules_576	passed counts:7
node_0: feature_name=LTB	LTB.value > threshold=0.994031548500061
node_5264: feature_name=MT-CO1	MT-CO1.value > threshold=-0.4127991199493408
node_5638: feature_name=OAZ1	OAZ1.value <= threshold=-0.2640867084264755
node_5639: feature_name=CD79B	CD79B.value <= threshold=2.2998517751693726

node_5640: feature_name=MTRNR2L2	MTRNR2L2.value <= threshold=3.5499863624572754
node_5641: feature_name=MT-CO1	MT-CO1.value > threshold=0.22567133605480194
node_5729: feature_name=MARCH1	MARCH1.value > threshold=1.5154643654823303
node_5867: feature_name=NFKBID	NFKBID.value <= threshold=2.6947624683380127
node_5868: feature_name=UTY	UTY.value <= threshold=3.29777729511261
node_5869: feature_name=IFI44L	IFI44L.value > threshold=-0.29640993475914
node_5899: feature_name=BANK1	BANK1.value <= threshold=3.7160801887512207
Class: COVID-19 Critical	
Rules_577	passed counts:7
node_0: feature_name=LTB	LTB.value > threshold=0.994031548500061
node_5264: feature_name=MT-CO1	MT-CO1.value > threshold=-0.4127991199493408
node_5638: feature_name=OAZ1	OAZ1.value <= threshold=-0.2640867084264755
node_5639: feature_name=CD79B	CD79B.value <= threshold=2.2998517751693726
node_5640: feature_name=MTRNR2L2	MTRNR2L2.value <= threshold=3.5499863624572754
node_5641: feature_name=MT-CO1	MT-CO1.value > threshold=0.22567133605480194
node_5729: feature_name=MARCH1	MARCH1.value > threshold=1.5154643654823303
node_5867: feature_name=NFKBID	NFKBID.value <= threshold=2.6947624683380127
node_5868: feature_name=UTY	UTY.value <= threshold=3.29777729511261
node_5869: feature_name=IFI44L	IFI44L.value <= threshold=-0.29640993475914
node_5870: feature_name=FTH1	FTH1.value <= threshold=0.6966870129108429
node_5871: feature_name=ILK	ILK.value > threshold=1.5719919204711914
node_5891: feature_name=IGJ	IGJ.value > threshold=-0.2698028236627579
node_5893: feature_name=MTRNR2L8	MTRNR2L8.value <= threshold=1.7828527092933655
Class: Healthy Control	
Rules_578	passed counts:7
node_0: feature_name=LTB	LTB.value > threshold=0.994031548500061
node_5264: feature_name=MT-CO1	MT-CO1.value > threshold=-0.4127991199493408
node_5638: feature_name=OAZ1	OAZ1.value <= threshold=-0.2640867084264755

node_5639: feature_name=CD79B	CD79B.value <= threshold=2.2998517751693726
node_5640: feature_name=MTRNR2L2	MTRNR2L2.value <= threshold=3.5499863624572754
node_5641: feature_name=MT-CO1	MT-CO1.value > threshold=0.22567133605480194
node_5729: feature_name=MARCH1	MARCH1.value <= threshold=1.5154643654823303
node_5730: feature_name=MT-CO1	MT-CO1.value > threshold=1.7823500633239746
node_5856: feature_name=TTN	TTN.value > threshold=-0.38264039158821106
node_5862: feature_name=BACH1	BACH1.value > threshold=-0.4962465167045593
node_5864: feature_name=FAM129C	FAM129C.value <= threshold=-0.29338543117046356
Class: COVID-19 Critical	
Rules_579	passed counts:7
node_0: feature_name=LTB	LTB.value > threshold=0.994031548500061
node_5264: feature_name=MT-CO1	MT-CO1.value > threshold=-0.4127991199493408
node_5638: feature_name=OAZ1	OAZ1.value <= threshold=-0.2640867084264755
node_5639: feature_name=CD79B	CD79B.value <= threshold=2.2998517751693726
node_5640: feature_name=MTRNR2L2	MTRNR2L2.value <= threshold=3.5499863624572754
node_5641: feature_name=MT-CO1	MT-CO1.value > threshold=0.22567133605480194
node_5729: feature_name=MARCH1	MARCH1.value <= threshold=1.5154643654823303
node_5730: feature_name=MT-CO1	MT-CO1.value <= threshold=1.7823500633239746
node_5731: feature_name=HLA-DPB1	HLA-DPB1.value <= threshold=2.0096163749694824
node_5732: feature_name=NR4A3	NR4A3.value <= threshold=3.509189486503601
node_5733: feature_name=OAZ1	OAZ1.value <= threshold=-0.4392261207103729
node_5734: feature_name=SESN3	SESN3.value <= threshold=-0.45661941170692444
node_5735: feature_name=WDR74	WDR74.value <= threshold=-0.4893670231103897
node_5736: feature_name=NR4A1	NR4A1.value <= threshold=-0.2724379748106003
node_5737: feature_name=HLA-DPA1	HLA-DPA1.value > threshold=1.6106070280075073
node_5745: feature_name=MEF2C	MEF2C.value <= threshold=2.7098671197891235
node_5746: feature_name=MMD	MMD.value <= threshold=-0.28827425837516785
node_5747: feature_name=NAMPT	NAMPT.value <= threshold=2.049232542514801
node_5748: feature_name=HLA-DRB5	HLA-DRB5.value > threshold=-0.44690944254398346

Class: COVID-19 Critical	
Rules_580	passed counts:7
node_0: feature_name=LTB	LTB.value > threshold=0.994031548500061
node_5264: feature_name=MT-CO1	MT-CO1.value > threshold=-0.4127991199493408
node_5638: feature_name=OAZ1	OAZ1.value <= threshold=-0.2640867084264755
node_5639: feature_name=CD79B	CD79B.value <= threshold=2.2998517751693726
node_5640: feature_name=MTRNR2L2	MTRNR2L2.value <= threshold=3.5499863624572754
node_5641: feature_name=MT-CO1	MT-CO1.value <= threshold=0.22567133605480194
node_5642: feature_name=RGS2	RGS2.value <= threshold=1.2649686336517334
node_5643: feature_name=TCL1A	TCL1A.value > threshold=4.771023273468018
node_5703: feature_name=MTRNR2L8	MTRNR2L8.value > threshold=-0.3554762303829193
node_5707: feature_name=SPI1	SPI1.value > threshold=-0.5607293844223022
Class: Healthy Control	
Rules_581	passed counts:7
node_0: feature_name=LTB	LTB.value > threshold=0.994031548500061
node_5264: feature_name=MT-CO1	MT-CO1.value > threshold=-0.4127991199493408
node_5638: feature_name=OAZ1	OAZ1.value <= threshold=-0.2640867084264755
node_5639: feature_name=CD79B	CD79B.value <= threshold=2.2998517751693726
node_5640: feature_name=MTRNR2L2	MTRNR2L2.value <= threshold=3.5499863624572754
node_5641: feature_name=MT-CO1	MT-CO1.value <= threshold=0.22567133605480194
node_5642: feature_name=RGS2	RGS2.value <= threshold=1.2649686336517334
node_5643: feature_name=TCL1A	TCL1A.value <= threshold=4.771023273468018
node_5644: feature_name=IGJ	IGJ.value <= threshold=3.91206431388855
node_5645: feature_name=AREGB	AREGB.value <= threshold=1.5142876282334328
node_5646: feature_name=HLA-DRB1	HLA-DRB1.value > threshold=-0.6253005266189575
node_5648: feature_name=ITM2C	ITM2C.value > threshold=3.2904402017593384
node_5690: feature_name=MT-CYB	MT-CYB.value <= threshold=0.3988293260335922
node_5691: feature_name=MT-CO1	MT-CO1.value <= threshold=0.06089743971824646

Class: COVID-19 Critical	
Rules_582	passed counts:7
node_0: feature_name=LTB	LTB.value > threshold=0.994031548500061
node_5264: feature_name=MT-CO1	MT-CO1.value <= threshold=-0.4127991199493408
node_5265: feature_name=MTRNR2L2	MTRNR2L2.value <= threshold=2.4473838806152344
node_5266: feature_name=TCL1A	TCL1A.value > threshold=5.787256956100464
node_5616: feature_name=XAF1	XAF1.value <= threshold=3.0923240184783936
node_5617: feature_name=CD79A	CD79A.value <= threshold=2.297448992729187
node_5618: feature_name=RGS2	RGS2.value > threshold=-0.5853994488716125
Class: COVID-19 Critical	
Rules_583	passed counts:7
node_0: feature_name=LTB	LTB.value > threshold=0.994031548500061
node_5264: feature_name=MT-CO1	MT-CO1.value <= threshold=-0.4127991199493408
node_5265: feature_name=MTRNR2L2	MTRNR2L2.value <= threshold=2.4473838806152344
node_5266: feature_name=TCL1A	TCL1A.value <= threshold=5.787256956100464
node_5267: feature_name=PLD4	PLD4.value > threshold=3.544657826423645
node_5577: feature_name=MT-CO1	MT-CO1.value > threshold=-0.6592064797878265
node_5601: feature_name=OAZ1	OAZ1.value <= threshold=-0.21202879399061203
node_5602: feature_name=RBKS	RBKS.value > threshold=-0.28591571748256683
Class: COVID-19 Critical	
Rules_584	passed counts:7
node_0: feature_name=LTB	LTB.value > threshold=0.994031548500061
node_5264: feature_name=MT-CO1	MT-CO1.value <= threshold=-0.4127991199493408
node_5265: feature_name=MTRNR2L2	MTRNR2L2.value <= threshold=2.4473838806152344
node_5266: feature_name=TCL1A	TCL1A.value <= threshold=5.787256956100464
node_5267: feature_name=PLD4	PLD4.value <= threshold=3.544657826423645
node_5268: feature_name=NR4A1	NR4A1.value > threshold=0.8490704298019409

node_5498: feature_name=HLA-DPA1	HLA-DPA1.value <= threshold=1.9079211950302124
node_5499: feature_name=ID3	ID3.value <= threshold=-0.17505786567926407
node_5500: feature_name=PPIF	PPIF.value <= threshold=-0.5180355906486511
node_5501: feature_name=LTB	LTB.value > threshold=1.0920450091362
node_5503: feature_name=SNX9	SNX9.value > threshold=-0.5426926016807556
node_5509: feature_name=FTH1	FTH1.value > threshold=-1.0832746624946594
Class: COVID-19 Severe	
Rules_585	passed counts:7
node_0: feature_name=LTB	LTB.value > threshold=0.994031548500061
node_5264: feature_name=MT-CO1	MT-CO1.value <= threshold=-0.4127991199493408
node_5265: feature_name=MTRNR2L2	MTRNR2L2.value <= threshold=2.4473838806152344
node_5266: feature_name=TCL1A	TCL1A.value <= threshold=5.787256956100464
node_5267: feature_name=PLD4	PLD4.value <= threshold=3.544657826423645
node_5268: feature_name=NR4A1	NR4A1.value > threshold=0.8490704298019409
node_5498: feature_name=HLA-DPA1	HLA-DPA1.value <= threshold=1.9079211950302124
node_5499: feature_name=ID3	ID3.value <= threshold=-0.17505786567926407
node_5500: feature_name=PPIF	PPIF.value <= threshold=-0.5180355906486511
node_5501: feature_name=LTB	LTB.value <= threshold=1.0920450091362
Class: COVID-19 Critical	
Rules_586	passed counts:7
node_0: feature_name=LTB	LTB.value > threshold=0.994031548500061
node_5264: feature_name=MT-CO1	MT-CO1.value <= threshold=-0.4127991199493408
node_5265: feature_name=MTRNR2L2	MTRNR2L2.value <= threshold=2.4473838806152344
node_5266: feature_name=TCL1A	TCL1A.value <= threshold=5.787256956100464
node_5267: feature_name=PLD4	PLD4.value <= threshold=3.544657826423645
node_5268: feature_name=NR4A1	NR4A1.value <= threshold=0.8490704298019409
node_5269: feature_name=LTB	LTB.value <= threshold=1.5252206921577454
node_5270: feature_name=HLA-DRB5	HLA-DRB5.value <= threshold=2.5117164850234985

node_5271: feature_name=CD79A	CD79A.value <= threshold=3.685201406478882
node_5272: feature_name=BCL2A1	BCL2A1.value <= threshold=2.081526756286621
node_5273: feature_name=HLA-DPA1	HLA-DPA1.value > threshold=1.8042166829109192
node_5363: feature_name=MS4A1	MS4A1.value <= threshold=3.5283708572387695
node_5364: feature_name=MT-CO3	MT-CO3.value > threshold=0.3994715213775635
node_5430: feature_name=OAZ1	OAZ1.value > threshold=0.05025118589401245
Class: Healthy Control	
Rules_587	passed counts:7
node_0: feature_name=LTB	LTB.value > threshold=0.994031548500061
node_5264: feature_name=MT-CO1	MT-CO1.value <= threshold=-0.4127991199493408
node_5265: feature_name=MTRNR2L2	MTRNR2L2.value <= threshold=2.4473838806152344
node_5266: feature_name=TCL1A	TCL1A.value <= threshold=5.787256956100464
node_5267: feature_name=PLD4	PLD4.value <= threshold=3.544657826423645
node_5268: feature_name=NR4A1	NR4A1.value <= threshold=0.8490704298019409
node_5269: feature_name=LTB	LTB.value <= threshold=1.5252206921577454
node_5270: feature_name=HLA-DRB5	HLA-DRB5.value <= threshold=2.5117164850234985
node_5271: feature_name=CD79A	CD79A.value <= threshold=3.685201406478882
node_5272: feature_name=BCL2A1	BCL2A1.value <= threshold=2.081526756286621
node_5273: feature_name=HLA-DPA1	HLA-DPA1.value > threshold=1.8042166829109192
node_5363: feature_name=MS4A1	MS4A1.value <= threshold=3.5283708572387695
node_5364: feature_name=MT-CO3	MT-CO3.value <= threshold=0.3994715213775635
node_5365: feature_name=TNFAIP3	TNFAIP3.value <= threshold=0.2631131559610367
node_5366: feature_name=MT-CO1	MT-CO1.value <= threshold=-0.44641776382923126
node_5367: feature_name=GPX1	GPX1.value > threshold=0.7746693789958954
node_5417: feature_name=TNFAIP3	TNFAIP3.value <= threshold=-0.46223947405815125
node_5418: feature_name=HLA-DRB5	HLA-DRB5.value <= threshold=2.0004552006721497
Class: COVID-19 Critical	
Rules_588	passed counts:7

node_0: feature_name=LTB	LTB.value > threshold=0.994031548500061
node_5264: feature_name=MT-CO1	MT-CO1.value <= threshold=-0.4127991199493408
node_5265: feature_name=MTRNR2L2	MTRNR2L2.value <= threshold=2.4473838806152344
node_5266: feature_name=TCL1A	TCL1A.value <= threshold=5.787256956100464
node_5267: feature_name=PLD4	PLD4.value <= threshold=3.544657826423645
node_5268: feature_name=NR4A1	NR4A1.value <= threshold=0.8490704298019409
node_5269: feature_name=LTB	LTB.value <= threshold=1.5252206921577454
node_5270: feature_name=HLA-DRB5	HLA-DRB5.value <= threshold=2.5117164850234985
node_5271: feature_name=CD79A	CD79A.value <= threshold=3.685201406478882
node_5272: feature_name=BCL2A1	BCL2A1.value <= threshold=2.081526756286621
node_5273: feature_name=HLA-DPA1	HLA-DPA1.value > threshold=1.8042166829109192
node_5363: feature_name=MS4A1	MS4A1.value <= threshold=3.5283708572387695
node_5364: feature_name=MT-CO3	MT-CO3.value <= threshold=0.3994715213775635
node_5365: feature_name=TNFAIP3	TNFAIP3.value <= threshold=0.2631131559610367
node_5366: feature_name=MT-CO1	MT-CO1.value <= threshold=-0.44641776382923126
node_5367: feature_name=GPX1	GPX1.value <= threshold=0.7746693789958954
node_5368: feature_name=MMD	MMD.value > threshold=3.176570177078247
node_5408: feature_name=MT-CO3	MT-CO3.value <= threshold=-0.25999920815229416
node_5409: feature_name=OAZ1	OAZ1.value > threshold=0.22116785682737827
Class: COVID-19 Critical	
Rules_589	passed counts:7
node_0: feature_name=LTB	LTB.value > threshold=0.994031548500061
node_5264: feature_name=MT-CO1	MT-CO1.value <= threshold=-0.4127991199493408
node_5265: feature_name=MTRNR2L2	MTRNR2L2.value <= threshold=2.4473838806152344
node_5266: feature_name=TCL1A	TCL1A.value <= threshold=5.787256956100464
node_5267: feature_name=PLD4	PLD4.value <= threshold=3.544657826423645
node_5268: feature_name=NR4A1	NR4A1.value <= threshold=0.8490704298019409
node_5269: feature_name=LTB	LTB.value <= threshold=1.5252206921577454
node_5270: feature_name=HLA-DRB5	HLA-DRB5.value <= threshold=2.5117164850234985

node_5271: feature_name=CD79A	CD79A.value <= threshold=3.685201406478882
node_5272: feature_name=BCL2A1	BCL2A1.value <= threshold=2.081526756286621
node_5273: feature_name=HLA-DPA1	HLA-DPA1.value <= threshold=1.8042166829109192
node_5274: feature_name=HSP90B1	HSP90B1.value > threshold=-1.3115270137786865
node_5278: feature_name=OAZ1	OAZ1.value <= threshold=1.0802008509635925
node_5279: feature_name=CD79B	CD79B.value <= threshold=5.875581979751587
node_5280: feature_name=CCDC141	CCDC141.value <= threshold=6.439151048660278
node_5281: feature_name=SYTL3	SYTL3.value <= threshold=1.149337351322174
node_5282: feature_name=GPX1	GPX1.value > threshold=-1.0243326425552368
node_5284: feature_name=MMD	MMD.value <= threshold=6.431635856628418
node_5285: feature_name=MARCKS	MARCKS.value <= threshold=4.034798860549927
node_5286: feature_name=XIST	XIST.value <= threshold=1.148451752960682
node_5287: feature_name=CD83	CD83.value <= threshold=2.894522190093994
node_5288: feature_name=MIR24-2	MIR24-2.value <= threshold=2.76771080493927
node_5289: feature_name=GRASP	GRASP.value > threshold=-0.4039058983325958
node_5303: feature_name=CD38	CD38.value <= threshold=3.721052408218384
node_5304: feature_name=ATF5	ATF5.value <= threshold=6.64192533493042
node_5305: feature_name=CD22	CD22.value <= threshold=5.268923759460449
node_5306: feature_name=CASP8	CASP8.value <= threshold=1.738209843635559
node_5307: feature_name=HSP90B1	HSP90B1.value > threshold=-1.2544044852256775
node_5311: feature_name=HLA-DPB1	HLA-DPB1.value <= threshold=2.4081677198410034
node_5312: feature_name=HSP90B1	HSP90B1.value > threshold=1.8984123468399048
node_5314: feature_name=TTN	TTN.value <= threshold=1.0012509226799011
Class: COVID-19 Critical	
Rules_590	passed counts:7
node_0: feature_name=LTB	LTB.value <= threshold=0.994031548500061
node_1: feature_name=MTRNR2L2	MTRNR2L2.value <= threshold=2.8784297704696655
node_2: feature_name=TCL1A	TCL1A.value > threshold=4.798312664031982
node_4912: feature_name=MT-CO1	MT-CO1.value <= threshold=-0.3664844036102295

node_4913: feature_name=CD79A	CD79A.value <= threshold=2.987208843231201
node_4914: feature_name=HLA-DPB1	HLA-DPB1.value <= threshold=2.0926655530929565
node_4915: feature_name=MX1	MX1.value > threshold=-0.4660506546497345
node_4921: feature_name=NR4A1	NR4A1.value > threshold=1.2700347900390625
node_4949: feature_name=LTB	LTB.value > threshold=0.7105090916156769
node_4955: feature_name=CCR6	CCR6.value > threshold=-0.2789531946182251
Class: Healthy Control	
Rules_591	passed counts:7
node_0: feature_name=LTB	LTB.value <= threshold=0.994031548500061
node_1: feature_name=MTRNR2L2	MTRNR2L2.value <= threshold=2.8784297704696655
node_2: feature_name=TCL1A	TCL1A.value <= threshold=4.798312664031982
node_3: feature_name=MT-CO1	MT-CO1.value > threshold=0.08947191759943962
node_2521: feature_name=LTB	LTB.value > threshold=0.6038667261600494
node_4173: feature_name=OAZ1	OAZ1.value > threshold=0.143283873796463
node_4615: feature_name=NR4A1	NR4A1.value <= threshold=0.6621004343032837
node_4616: feature_name=NFKBID	NFKBID.value <= threshold=3.283741593360901
node_4617: feature_name=HLA-DQA1	HLA-DQA1.value > threshold=2.3435752391815186
node_4719: feature_name=MX1	MX1.value <= threshold=1.851993978023529
node_4720: feature_name=JUN	JUN.value <= threshold=-0.5025404393672943
node_4721: feature_name=RELT	RELT.value > threshold=-0.28893138468265533
node_4735: feature_name=HLA-DQB1	HLA-DQB1.value <= threshold=2.66076397895813
node_4736: feature_name=GPX1	GPX1.value > threshold=-0.8606258034706116
Class: Healthy Control	
Rules_592	passed counts:7
node_0: feature_name=LTB	LTB.value <= threshold=0.994031548500061
node_1: feature_name=MTRNR2L2	MTRNR2L2.value <= threshold=2.8784297704696655
node_2: feature_name=TCL1A	TCL1A.value <= threshold=4.798312664031982
node_3: feature_name=MT-CO1	MT-CO1.value > threshold=0.08947191759943962

node_2521: feature_name=LTB	LTB.value > threshold=0.6038667261600494
node_4173: feature_name=OAZ1	OAZ1.value > threshold=0.143283873796463
node_4615: feature_name=NR4A1	NR4A1.value <= threshold=0.6621004343032837
node_4616: feature_name=NFKBID	NFKBID.value <= threshold=3.283741593360901
node_4617: feature_name=HLA-DQA1	HLA-DQA1.value > threshold=2.3435752391815186
node_4719: feature_name=MX1	MX1.value <= threshold=1.851993978023529
node_4720: feature_name=JUN	JUN.value <= threshold=-0.5025404393672943
node_4721: feature_name=RELT	RELT.value <= threshold=-0.28893138468265533
node_4722: feature_name=SNX29P2	SNX29P2.value > threshold=1.9070574045181274
node_4730: feature_name=NFKBID	NFKBID.value <= threshold=1.8065539002418518
Class: Healthy Control	
Rules_593	passed counts:7
node_0: feature_name=LTB	LTB.value <= threshold=0.994031548500061
node_1: feature_name=MTRNR2L2	MTRNR2L2.value <= threshold=2.8784297704696655
node_2: feature_name=TCL1A	TCL1A.value <= threshold=4.798312664031982
node_3: feature_name=MT-CO1	MT-CO1.value > threshold=0.08947191759943962
node_2521: feature_name=LTB	LTB.value > threshold=0.6038667261600494
node_4173: feature_name=OAZ1	OAZ1.value > threshold=0.143283873796463
node_4615: feature_name=NR4A1	NR4A1.value <= threshold=0.6621004343032837
node_4616: feature_name=NFKBID	NFKBID.value <= threshold=3.283741593360901
node_4617: feature_name=HLA-DQA1	HLA-DQA1.value <= threshold=2.3435752391815186
node_4618: feature_name=PELI1	PELI1.value > threshold=1.3271203637123108
node_4680: feature_name=BANK1	BANK1.value > threshold=-0.3607018291950226
node_4682: feature_name=RGCC	RGCC.value <= threshold=0.60355344414711
node_4683: feature_name=SNX29P2	SNX29P2.value <= threshold=4.192138910293579
node_4684: feature_name=XIST	XIST.value <= threshold=2.375336170196533
node_4685: feature_name=TCF4	TCF4.value > threshold=2.2667007446289062
node_4697: feature_name=MEF2A	MEF2A.value <= threshold=-0.4261530190706253
node_4698: feature_name=RALGPS2	RALGPS2.value <= threshold=2.293534517288208

node_4699: feature_name=OAZ1	OAZ1.value > threshold=0.35008086264133453
Class: COVID-19 Critical	
Rules_594	passed counts:7
node_0: feature_name=LTB	LTB.value <= threshold=0.994031548500061
node_1: feature_name=MTRNR2L2	MTRNR2L2.value <= threshold=2.8784297704696655
node_2: feature_name=TCL1A	TCL1A.value <= threshold=4.798312664031982
node_3: feature_name=MT-CO1	MT-CO1.value > threshold=0.08947191759943962
node_2521: feature_name=LTB	LTB.value > threshold=0.6038667261600494
node_4173: feature_name=OAZ1	OAZ1.value <= threshold=0.143283873796463
node_4174: feature_name=PLD4	PLD4.value <= threshold=2.921932101249695
node_4175: feature_name=XIST	XIST.value <= threshold=-0.05067425221204758
node_4176: feature_name=MT-CO1	MT-CO1.value <= threshold=1.345594048500061
node_4177: feature_name=HLA-DQB1	HLA-DQB1.value > threshold=1.927736520767212
node_4353: feature_name=ITM2C	ITM2C.value <= threshold=-0.12329206243157387
node_4354: feature_name=MX1	MX1.value <= threshold=-0.4356398284435272
node_4355: feature_name=HLA-DQB1	HLA-DQB1.value <= threshold=2.8865538835525513
node_4356: feature_name=SNX9	SNX9.value <= threshold=1.002076417207718
node_4357: feature_name=FAM160B1	FAM160B1.value > threshold=-0.42920351028442383
node_4361: feature_name=SNX9	SNX9.value > threshold=0.011789441108703613
node_4379: feature_name=MS4A1	MS4A1.value > threshold=1.948566198348999
Class: Healthy Control	
Rules_595	passed counts:7
node_0: feature_name=LTB	LTB.value <= threshold=0.994031548500061
node_1: feature_name=MTRNR2L2	MTRNR2L2.value <= threshold=2.8784297704696655
node_2: feature_name=TCL1A	TCL1A.value <= threshold=4.798312664031982
node_3: feature_name=MT-CO1	MT-CO1.value > threshold=0.08947191759943962
node_2521: feature_name=LTB	LTB.value > threshold=0.6038667261600494
node_4173: feature_name=OAZ1	OAZ1.value <= threshold=0.143283873796463

node_4174: feature_name=PLD4	PLD4.value <= threshold=2.921932101249695
node_4175: feature_name=XIST	XIST.value <= threshold=-0.05067425221204758
node_4176: feature_name=MT-CO1	MT-CO1.value <= threshold=1.345594048500061
node_4177: feature_name=HLA-DQB1	HLA-DQB1.value <= threshold=1.927736520767212
node_4178: feature_name=BACH1	BACH1.value > threshold=-0.5349778532981873
node_4236: feature_name=TPM1	TPM1.value <= threshold=3.516477584838867
node_4237: feature_name=NR4A1	NR4A1.value > threshold=-0.274935707449913
node_4277: feature_name=HLA-DPA1	HLA-DPA1.value > threshold=1.8894080519676208
node_4339: feature_name=PARP14	PARP14.value > threshold=0.2787386327981949
node_4347: feature_name=S100A6	S100A6.value > threshold=-1.3354644775390625
Class: COVID-19 Severe	
Rules_596	passed counts:7
node_0: feature_name=LTB	LTB.value <= threshold=0.994031548500061
node_1: feature_name=MTRNR2L2	MTRNR2L2.value <= threshold=2.8784297704696655
node_2: feature_name=TCL1A	TCL1A.value <= threshold=4.798312664031982
node_3: feature_name=MT-CO1	MT-CO1.value > threshold=0.08947191759943962
node_2521: feature_name=LTB	LTB.value > threshold=0.6038667261600494
node_4173: feature_name=OAZ1	OAZ1.value <= threshold=0.143283873796463
node_4174: feature_name=PLD4	PLD4.value <= threshold=2.921932101249695
node_4175: feature_name=XIST	XIST.value <= threshold=-0.05067425221204758
node_4176: feature_name=MT-CO1	MT-CO1.value <= threshold=1.345594048500061
node_4177: feature_name=HLA-DQB1	HLA-DQB1.value <= threshold=1.927736520767212
node_4178: feature_name=BACH1	BACH1.value > threshold=-0.5349778532981873
node_4236: feature_name=TPM1	TPM1.value <= threshold=3.516477584838867
node_4237: feature_name=NR4A1	NR4A1.value <= threshold=-0.274935707449913
node_4238: feature_name=VPREB3	VPREB3.value <= threshold=5.024149179458618
node_4239: feature_name=YWHAH	YWHAH.value <= threshold=4.7504096031188965
node_4240: feature_name=NAIP	NAIP.value <= threshold=4.030007481575012
node_4241: feature_name=CD38	CD38.value > threshold=-0.3387429714202881

node_4243: feature_name=MS4A1	MS4A1.value <= threshold=5.195746660232544
node_4244: feature_name=TRIB1	TRIB1.value <= threshold=2.9039160013198853
node_4245: feature_name=RGS2	RGS2.value <= threshold=-0.8980970978736877
node_4246: feature_name=SLC7A5	SLC7A5.value <= threshold=0.16534416377544403
Class: COVID-19 Critical	
Rules_597	passed counts:7
node_0: feature_name=LTB	LTB.value <= threshold=0.994031548500061
node_1: feature_name=MTRNR2L2	MTRNR2L2.value <= threshold=2.8784297704696655
node_2: feature_name=TCL1A	TCL1A.value <= threshold=4.798312664031982
node_3: feature_name=MT-CO1	MT-CO1.value > threshold=0.08947191759943962
node_2521: feature_name=LTB	LTB.value > threshold=0.6038667261600494
node_4173: feature_name=OAZ1	OAZ1.value <= threshold=0.143283873796463
node_4174: feature_name=PLD4	PLD4.value <= threshold=2.921932101249695
node_4175: feature_name=XIST	XIST.value <= threshold=-0.05067425221204758
node_4176: feature_name=MT-CO1	MT-CO1.value <= threshold=1.345594048500061
node_4177: feature_name=HLA-DQB1	HLA-DQB1.value <= threshold=1.927736520767212
node_4178: feature_name=BACH1	BACH1.value <= threshold=-0.5349778532981873
node_4179: feature_name=CD79B	CD79B.value > threshold=1.6518195271492004
node_4201: feature_name=MT-CO2	MT-CO2.value > threshold=0.5007440596818924
node_4223: feature_name=FAM129C	FAM129C.value <= threshold=4.054540395736694
node_4224: feature_name=MEF2A	MEF2A.value <= threshold=-0.4423168897628784
node_4225: feature_name=ENDOD1	ENDOD1.value <= threshold=-0.18554074317216873
node_4226: feature_name=NFKBIZ	NFKBIZ.value <= threshold=0.8983076810836792
Class: Healthy Control	
Rules_598	passed counts:7
node_0: feature_name=LTB	LTB.value <= threshold=0.994031548500061
node_1: feature_name=MTRNR2L2	MTRNR2L2.value <= threshold=2.8784297704696655
node_2: feature_name=TCL1A	TCL1A.value <= threshold=4.798312664031982

node_3: feature_name=MT-CO1	MT-CO1.value > threshold=0.08947191759943962
node_2521: feature_name=LTB	LTB.value <= threshold=0.6038667261600494
node_2522: feature_name=HLA-DQB1	HLA-DQB1.value > threshold=2.1053054332733154
node_3550: feature_name=XIST	XIST.value <= threshold=0.7716337237507105
node_3551: feature_name=CD79B	CD79B.value > threshold=2.9426796436309814
node_4039: feature_name=IFI44L	IFI44L.value <= threshold=1.8637521862983704
node_4040: feature_name=SNX9	SNX9.value > threshold=1.274232804775238
node_4096: feature_name=RALGPS2	RALGPS2.value <= threshold=2.3599878549575806
node_4097: feature_name=HLA-DQB1	HLA-DQB1.value <= threshold=2.9625227451324463
node_4098: feature_name=CD79A	CD79A.value <= threshold=2.383400559425354
node_4099: feature_name=CXorf57	CXorf57.value <= threshold=-0.2232322245836258
node_4100: feature_name=ATF3	ATF3.value <= threshold=-0.20010782778263092
Class: COVID-19 Critical	
Rules_599	passed counts:7
node_0: feature_name=LTB	LTB.value <= threshold=0.994031548500061
node_1: feature_name=MTRNR2L2	MTRNR2L2.value <= threshold=2.8784297704696655
node_2: feature_name=TCL1A	TCL1A.value <= threshold=4.798312664031982
node_3: feature_name=MT-CO1	MT-CO1.value > threshold=0.08947191759943962
node_2521: feature_name=LTB	LTB.value <= threshold=0.6038667261600494
node_2522: feature_name=HLA-DQB1	HLA-DQB1.value > threshold=2.1053054332733154
node_3550: feature_name=XIST	XIST.value <= threshold=0.7716337237507105
node_3551: feature_name=CD79B	CD79B.value <= threshold=2.9426796436309814
node_3552: feature_name=HLA-DQB1	HLA-DQB1.value > threshold=2.713674306869507
node_3854: feature_name=MEF2C	MEF2C.value > threshold=-0.3721291869878769
node_3882: feature_name=IFI44L	IFI44L.value <= threshold=2.5516022443771362
node_3883: feature_name=FCER2	FCER2.value <= threshold=-0.21487552672624588
node_3884: feature_name=HLA-DQA2	HLA-DQA2.value > threshold=-0.12580305337905884
node_3954: feature_name=SNX9	SNX9.value <= threshold=-0.5673478543758392
node_3955: feature_name=CD83	CD83.value <= threshold=2.9509599208831787

node_3956: feature_name=DAPP1	DAPP1.value <= threshold=-0.2832452803850174
Class: COVID-19 Critical	
Rules_600	passed counts:7
node_0: feature_name=LTB	LTB.value <= threshold=0.994031548500061
node_1: feature_name=MTRNR2L2	MTRNR2L2.value <= threshold=2.8784297704696655
node_2: feature_name=TCL1A	TCL1A.value <= threshold=4.798312664031982
node_3: feature_name=MT-CO1	MT-CO1.value > threshold=0.08947191759943962
node_2521: feature_name=LTB	LTB.value <= threshold=0.6038667261600494
node_2522: feature_name=HLA-DQB1	HLA-DQB1.value > threshold=2.1053054332733154
node_3550: feature_name=XIST	XIST.value <= threshold=0.7716337237507105
node_3551: feature_name=CD79B	CD79B.value <= threshold=2.9426796436309814
node_3552: feature_name=HLA-DQB1	HLA-DQB1.value > threshold=2.713674306869507
node_3854: feature_name=MEF2C	MEF2C.value <= threshold=-0.3721291869878769
node_3855: feature_name=HLA-DRB5	HLA-DRB5.value > threshold=0.7929701805114746
node_3859: feature_name=LTB	LTB.value > threshold=-0.9689712226390839
node_3863: feature_name=RP11-731F5.2	RP11-731F5.2.value <= threshold=3.578703761100769
node_3864: feature_name=COTL1	COTL1.value <= threshold=0.38136887550354004
node_3865: feature_name=HLA-DQA1	HLA-DQA1.value > threshold=2.6825207471847534
node_3873: feature_name=MARCH1	MARCH1.value <= threshold=1.1291348487138748
Class: COVID-19 Critical	
Rules_601	passed counts:7
node_0: feature_name=LTB	LTB.value <= threshold=0.994031548500061
node_1: feature_name=MTRNR2L2	MTRNR2L2.value <= threshold=2.8784297704696655
node_2: feature_name=TCL1A	TCL1A.value <= threshold=4.798312664031982
node_3: feature_name=MT-CO1	MT-CO1.value > threshold=0.08947191759943962
node_2521: feature_name=LTB	LTB.value <= threshold=0.6038667261600494
node_2522: feature_name=HLA-DQB1	HLA-DQB1.value > threshold=2.1053054332733154
node_3550: feature_name=XIST	XIST.value <= threshold=0.7716337237507105

node_3551: feature_name=CD79B	CD79B.value <= threshold=2.9426796436309814
node_3552: feature_name=HLA-DQB1	HLA-DQB1.value <= threshold=2.713674306869507
node_3553: feature_name=SNX9	SNX9.value > threshold=-0.5404067635536194
node_3653: feature_name=LTB	LTB.value > threshold=0.021708179265260696
node_3759: feature_name=TCL1A	TCL1A.value <= threshold=3.3921085596084595
node_3760: feature_name=HLA-DRB5	HLA-DRB5.value > threshold=0.5265218019485474
node_3776: feature_name=ID1	ID1.value <= threshold=-0.21235637366771698
node_3777: feature_name=RP11-731F5.2	RP11-731F5.2.value > threshold=2.830894112586975
node_3817: feature_name=BOD1L1	BOD1L1.value <= threshold=-0.4770502895116806
node_3818: feature_name=APP	APP.value > threshold=-0.40902893245220184
Class: Healthy Control	
Rules_602	passed counts:7
node_0: feature_name=LTB	LTB.value <= threshold=0.994031548500061
node_1: feature_name=MTRNR2L2	MTRNR2L2.value <= threshold=2.8784297704696655
node_2: feature_name=TCL1A	TCL1A.value <= threshold=4.798312664031982
node_3: feature_name=MT-CO1	MT-CO1.value > threshold=0.08947191759943962
node_2521: feature_name=LTB	LTB.value <= threshold=0.6038667261600494
node_2522: feature_name=HLA-DQB1	HLA-DQB1.value > threshold=2.1053054332733154
node_3550: feature_name=XIST	XIST.value <= threshold=0.7716337237507105
node_3551: feature_name=CD79B	CD79B.value <= threshold=2.9426796436309814
node_3552: feature_name=HLA-DQB1	HLA-DQB1.value <= threshold=2.713674306869507
node_3553: feature_name=SNX9	SNX9.value > threshold=-0.5404067635536194
node_3653: feature_name=LTB	LTB.value > threshold=0.021708179265260696
node_3759: feature_name=TCL1A	TCL1A.value <= threshold=3.3921085596084595
node_3760: feature_name=HLA-DRB5	HLA-DRB5.value > threshold=0.5265218019485474
node_3776: feature_name=ID1	ID1.value <= threshold=-0.21235637366771698
node_3777: feature_name=RP11-731F5.2	RP11-731F5.2.value <= threshold=2.830894112586975
node_3778: feature_name=CASP8	CASP8.value <= threshold=2.222941279411316
node_3779: feature_name=MT-CO3	MT-CO3.value > threshold=-0.41117167472839355

node_3785: feature_name=HLA-DRB5	HLA-DRB5.value > threshold=0.8465962707996368
node_3793: feature_name=PDLIM1	PDLIM1.value > threshold=2.90118670463562
node_3813: feature_name=IRF8	IRF8.value > threshold=-0.34634101390838623
Class: COVID-19 Critical	
Rules_603	passed counts:7
node_0: feature_name=LTB	LTB.value <= threshold=0.994031548500061
node_1: feature_name=MTRNR2L2	MTRNR2L2.value <= threshold=2.8784297704696655
node_2: feature_name=TCL1A	TCL1A.value <= threshold=4.798312664031982
node_3: feature_name=MT-CO1	MT-CO1.value > threshold=0.08947191759943962
node_2521: feature_name=LTB	LTB.value <= threshold=0.6038667261600494
node_2522: feature_name=HLA-DQB1	HLA-DQB1.value > threshold=2.1053054332733154
node_3550: feature_name=XIST	XIST.value <= threshold=0.7716337237507105
node_3551: feature_name=CD79B	CD79B.value <= threshold=2.9426796436309814
node_3552: feature_name=HLA-DQB1	HLA-DQB1.value <= threshold=2.713674306869507
node_3553: feature_name=SNX9	SNX9.value > threshold=-0.5404067635536194
node_3653: feature_name=LTB	LTB.value <= threshold=0.021708179265260696
node_3654: feature_name=HLA-DRB5	HLA-DRB5.value > threshold=-0.5633223652839661
node_3660: feature_name=NR4A1	NR4A1.value > threshold=2.2327845096588135
node_3736: feature_name=NFKBID	NFKBID.value > threshold=-0.4191274344921112
node_3738: feature_name=PARP14	PARP14.value <= threshold=-0.49970389902591705
node_3739: feature_name=LDLRAD4	LDLRAD4.value > threshold=-0.268046572804451
node_3747: feature_name=TNFAIP3	TNFAIP3.value > threshold=-1.493474304676056
node_3749: feature_name=NAIP	NAIP.value <= threshold=-0.26066793501377106
Class: COVID-19 Severe	
Rules_604	passed counts:7
node_0: feature_name=LTB	LTB.value <= threshold=0.994031548500061
node_1: feature_name=MTRNR2L2	MTRNR2L2.value <= threshold=2.8784297704696655
node_2: feature_name=TCL1A	TCL1A.value <= threshold=4.798312664031982

node_3: feature_name=MT-CO1	MT-CO1.value > threshold=0.08947191759943962
node_2521: feature_name=LTB	LTB.value <= threshold=0.6038667261600494
node_2522: feature_name=HLA-DQB1	HLA-DQB1.value > threshold=2.1053054332733154
node_3550: feature_name=XIST	XIST.value <= threshold=0.7716337237507105
node_3551: feature_name=CD79B	CD79B.value <= threshold=2.9426796436309814
node_3552: feature_name=HLA-DQB1	HLA-DQB1.value <= threshold=2.713674306869507
node_3553: feature_name=SNX9	SNX9.value > threshold=-0.5404067635536194
node_3653: feature_name=LTB	LTB.value <= threshold=0.021708179265260696
node_3654: feature_name=HLA-DRB5	HLA-DRB5.value > threshold=-0.5633223652839661
node_3660: feature_name=NR4A1	NR4A1.value <= threshold=2.2327845096588135
node_3661: feature_name=FCRL1	FCRL1.value <= threshold=8.662389755249023
node_3662: feature_name=BANK1	BANK1.value > threshold=4.776419401168823
node_3726: feature_name=MT-CO1	MT-CO1.value <= threshold=1.4696794748306274
node_3727: feature_name=GRASP	GRASP.value <= threshold=1.477085903286934
Class: COVID-19 Critical	
Rules_605	passed counts:7
node_0: feature_name=LTB	LTB.value <= threshold=0.994031548500061
node_1: feature_name=MTRNR2L2	MTRNR2L2.value <= threshold=2.8784297704696655
node_2: feature_name=TCL1A	TCL1A.value <= threshold=4.798312664031982
node_3: feature_name=MT-CO1	MT-CO1.value > threshold=0.08947191759943962
node_2521: feature_name=LTB	LTB.value <= threshold=0.6038667261600494
node_2522: feature_name=HLA-DQB1	HLA-DQB1.value <= threshold=2.1053054332733154
node_2523: feature_name=SNX9	SNX9.value > threshold=-0.5346486568450928
node_2907: feature_name=NR4A1	NR4A1.value <= threshold=2.5433837175369263
node_2908: feature_name=XIST	XIST.value > threshold=0.7664470989257097
node_3440: feature_name=MT-CO1	MT-CO1.value > threshold=0.9336088299751282
node_3448: feature_name=RALGPS2	RALGPS2.value <= threshold=3.274242401123047
node_3449: feature_name=S100A6	S100A6.value <= threshold=-1.0691525042057037
node_3450: feature_name=MT-CO1	MT-CO1.value <= threshold=2.2711681127548218

Class: COVID-19 Severe	
Rules_606	passed counts:7
node_0: feature_name=LTB	LTB.value <= threshold=0.994031548500061
node_1: feature_name=MTRNR2L2	MTRNR2L2.value <= threshold=2.8784297704696655
node_2: feature_name=TCL1A	TCL1A.value <= threshold=4.798312664031982
node_3: feature_name=MT-CO1	MT-CO1.value > threshold=0.08947191759943962
node_2521: feature_name=LTB	LTB.value <= threshold=0.6038667261600494
node_2522: feature_name=HLA-DQB1	HLA-DQB1.value <= threshold=2.1053054332733154
node_2523: feature_name=SNX9	SNX9.value > threshold=-0.5346486568450928
node_2907: feature_name=NR4A1	NR4A1.value <= threshold=2.5433837175369263
node_2908: feature_name=XIST	XIST.value <= threshold=0.7664470989257097
node_2909: feature_name=CD79B	CD79B.value <= threshold=2.9326847791671753
node_2910: feature_name=NR4A1	NR4A1.value > threshold=-0.2875557690858841
node_3096: feature_name=NR4A3	NR4A3.value <= threshold=3.314968705177307
node_3097: feature_name=VPREB3	VPREB3.value <= threshold=3.3549938201904297
node_3098: feature_name=RALGPS2	RALGPS2.value <= threshold=5.807050704956055
node_3099: feature_name=CTD-2035E11.3	CTD-2035E11.3.value <= threshold=6.4090416431427
node_3100: feature_name=FAM129C	FAM129C.value > threshold=6.966361045837402
node_3272: feature_name=MT-CYB	MT-CYB.value <= threshold=1.1738299131393433
node_3273: feature_name=MS4A1	MS4A1.value <= threshold=4.263813018798828
node_3274: feature_name=ZNF10	ZNF10.value <= threshold=5.08561897277832
Class: COVID-19 Critical	
Rules_607	passed counts:7
node_0: feature_name=LTB	LTB.value <= threshold=0.994031548500061
node_1: feature_name=MTRNR2L2	MTRNR2L2.value <= threshold=2.8784297704696655
node_2: feature_name=TCL1A	TCL1A.value <= threshold=4.798312664031982
node_3: feature_name=MT-CO1	MT-CO1.value > threshold=0.08947191759943962
node_2521: feature_name=LTB	LTB.value <= threshold=0.6038667261600494

node_2522: feature_name=HLA-DQB1	HLA-DQB1.value <= threshold=2.1053054332733154
node_2523: feature_name=SNX9	SNX9.value > threshold=-0.5346486568450928
node_2907: feature_name=NR4A1	NR4A1.value <= threshold=2.5433837175369263
node_2908: feature_name=XIST	XIST.value <= threshold=0.7664470989257097
node_2909: feature_name=CD79B	CD79B.value <= threshold=2.9326847791671753
node_2910: feature_name=NR4A1	NR4A1.value > threshold=-0.2875557690858841
node_3096: feature_name=NR4A3	NR4A3.value <= threshold=3.314968705177307
node_3097: feature_name=VPREB3	VPREB3.value <= threshold=3.3549938201904297
node_3098: feature_name=RALGPS2	RALGPS2.value <= threshold=5.807050704956055
node_3099: feature_name=CTD-2035E11.3	CTD-2035E11.3.value <= threshold=6.4090416431427
node_3100: feature_name=FAM129C	FAM129C.value <= threshold=6.966361045837402
node_3101: feature_name=HES4	HES4.value <= threshold=4.139557242393494
node_3102: feature_name=CD83	CD83.value > threshold=1.8343153595924377
node_3136: feature_name=PLD4	PLD4.value > threshold=4.453821897506714
node_3264: feature_name=LTB	LTB.value > threshold=0.12628396600484848
node_3266: feature_name=TSPYL2	TSPYL2.value <= threshold=1.1761471629142761
Class: Healthy Control	
Rules_608	passed counts:7
node_0: feature_name=LTB	LTB.value <= threshold=0.994031548500061
node_1: feature_name=MTRNR2L2	MTRNR2L2.value <= threshold=2.8784297704696655
node_2: feature_name=TCL1A	TCL1A.value <= threshold=4.798312664031982
node_3: feature_name=MT-CO1	MT-CO1.value > threshold=0.08947191759943962
node_2521: feature_name=LTB	LTB.value <= threshold=0.6038667261600494
node_2522: feature_name=HLA-DQB1	HLA-DQB1.value <= threshold=2.1053054332733154
node_2523: feature_name=SNX9	SNX9.value <= threshold=-0.5346486568450928
node_2524: feature_name=HLA-DPB1	HLA-DPB1.value > threshold=1.9548270106315613
node_2814: feature_name=CD79B	CD79B.value > threshold=-0.28186772763729095
node_2826: feature_name=MT-ND6	MT-ND6.value <= threshold=0.17273088544607162
node_2827: feature_name=NR4A1	NR4A1.value > threshold=-0.24634335190057755

node_2849: feature_name=FAM129C	FAM129C.value <= threshold=3.089340925216675
node_2850: feature_name=COTL1	COTL1.value <= threshold=-0.5968812704086304
node_2851: feature_name=HLA-DPB1	HLA-DPB1.value <= threshold=2.2867326736450195
Class: COVID-19 Critical	
Rules_609	passed counts:7
node_0: feature_name=LTB	LTB.value <= threshold=0.994031548500061
node_1: feature_name=MTRNR2L2	MTRNR2L2.value <= threshold=2.8784297704696655
node_2: feature_name=TCL1A	TCL1A.value <= threshold=4.798312664031982
node_3: feature_name=MT-CO1	MT-CO1.value > threshold=0.08947191759943962
node_2521: feature_name=LTB	LTB.value <= threshold=0.6038667261600494
node_2522: feature_name=HLA-DQB1	HLA-DQB1.value <= threshold=2.1053054332733154
node_2523: feature_name=SNX9	SNX9.value <= threshold=-0.5346486568450928
node_2524: feature_name=HLA-DPB1	HLA-DPB1.value <= threshold=1.9548270106315613
node_2525: feature_name=FAM129C	FAM129C.value > threshold=3.3393388986587524
node_2721: feature_name=ADAM19	ADAM19.value > threshold=-0.29525794088840485
node_2781: feature_name=XAF1	XAF1.value > threshold=-0.4296249598264694
node_2793: feature_name=PELI1	PELI1.value > threshold=1.8806812763214111
node_2801: feature_name=HLA-DRB1	HLA-DRB1.value <= threshold=0.921924501657486
node_2802: feature_name=TNFAIP3	TNFAIP3.value > threshold=-1.0874561071395874
Class: COVID-19 Critical	
Rules_610	passed counts:7
node_0: feature_name=LTB	LTB.value <= threshold=0.994031548500061
node_1: feature_name=MTRNR2L2	MTRNR2L2.value <= threshold=2.8784297704696655
node_2: feature_name=TCL1A	TCL1A.value <= threshold=4.798312664031982
node_3: feature_name=MT-CO1	MT-CO1.value > threshold=0.08947191759943962
node_2521: feature_name=LTB	LTB.value <= threshold=0.6038667261600494
node_2522: feature_name=HLA-DQB1	HLA-DQB1.value <= threshold=2.1053054332733154
node_2523: feature_name=SNX9	SNX9.value <= threshold=-0.5346486568450928

node_2524: feature_name=HLA-DPB1	HLA-DPB1.value <= threshold=1.9548270106315613
node_2525: feature_name=FAM129C	FAM129C.value > threshold=3.3393388986587524
node_2721: feature_name=ADAM19	ADAM19.value <= threshold=-0.29525794088840485
node_2722: feature_name=NR4A1	NR4A1.value <= threshold=2.1566513776779175
node_2723: feature_name=CD22	CD22.value <= threshold=8.345317840576172
node_2724: feature_name=PLD4	PLD4.value <= threshold=7.507996082305908
node_2725: feature_name=RALGPS2	RALGPS2.value > threshold=4.3808369636535645
node_2751: feature_name=NFKBID	NFKBID.value <= threshold=-0.4283874034881592
node_2752: feature_name=IFIT3	IFIT3.value <= threshold=-0.16047467291355133
Class: Healthy Control	
Rules_611	passed counts:7
node_0: feature_name=LTB	LTB.value <= threshold=0.994031548500061
node_1: feature_name=MTRNR2L2	MTRNR2L2.value <= threshold=2.8784297704696655
node_2: feature_name=TCL1A	TCL1A.value <= threshold=4.798312664031982
node_3: feature_name=MT-CO1	MT-CO1.value > threshold=0.08947191759943962
node_2521: feature_name=LTB	LTB.value <= threshold=0.6038667261600494
node_2522: feature_name=HLA-DQB1	HLA-DQB1.value <= threshold=2.1053054332733154
node_2523: feature_name=SNX9	SNX9.value <= threshold=-0.5346486568450928
node_2524: feature_name=HLA-DPB1	HLA-DPB1.value <= threshold=1.9548270106315613
node_2525: feature_name=FAM129C	FAM129C.value > threshold=3.3393388986587524
node_2721: feature_name=ADAM19	ADAM19.value <= threshold=-0.29525794088840485
node_2722: feature_name=NR4A1	NR4A1.value <= threshold=2.1566513776779175
node_2723: feature_name=CD22	CD22.value <= threshold=8.345317840576172
node_2724: feature_name=PLD4	PLD4.value <= threshold=7.507996082305908
node_2725: feature_name=RALGPS2	RALGPS2.value <= threshold=4.3808369636535645
node_2726: feature_name=VPREB3	VPREB3.value > threshold=4.1944358348846436
node_2746: feature_name=HLA-DPB1	HLA-DPB1.value > threshold=1.3332839012145996
node_2748: feature_name=XAF1	XAF1.value <= threshold=0.7416253834962845
Class: Healthy Control	

Rules_612	passed counts:7
node_0: feature_name=LTB	LTB.value <= threshold=0.994031548500061
node_1: feature_name=MTRNR2L2	MTRNR2L2.value <= threshold=2.8784297704696655
node_2: feature_name=TCL1A	TCL1A.value <= threshold=4.798312664031982
node_3: feature_name=MT-CO1	MT-CO1.value > threshold=0.08947191759943962
node_2521: feature_name=LTB	LTB.value <= threshold=0.6038667261600494
node_2522: feature_name=HLA-DQB1	HLA-DQB1.value <= threshold=2.1053054332733154
node_2523: feature_name=SNX9	SNX9.value <= threshold=-0.5346486568450928
node_2524: feature_name=HLA-DPB1	HLA-DPB1.value <= threshold=1.9548270106315613
node_2525: feature_name=FAM129C	FAM129C.value > threshold=3.3393388986587524
node_2721: feature_name=ADAM19	ADAM19.value <= threshold=-0.29525794088840485
node_2722: feature_name=NR4A1	NR4A1.value <= threshold=2.1566513776779175
node_2723: feature_name=CD22	CD22.value <= threshold=8.345317840576172
node_2724: feature_name=PLD4	PLD4.value <= threshold=7.507996082305908
node_2725: feature_name=RALGPS2	RALGPS2.value <= threshold=4.3808369636535645
node_2726: feature_name=VPREB3	VPREB3.value > threshold=4.1944358348846436
node_2746: feature_name=HLA-DPB1	HLA-DPB1.value <= threshold=1.3332839012145996
Class: COVID-19 Critical	
Rules_613	passed counts:7
node_0: feature_name=LTB	LTB.value <= threshold=0.994031548500061
node_1: feature_name=MTRNR2L2	MTRNR2L2.value <= threshold=2.8784297704696655
node_2: feature_name=TCL1A	TCL1A.value <= threshold=4.798312664031982
node_3: feature_name=MT-CO1	MT-CO1.value <= threshold=0.08947191759943962
node_4: feature_name=NR4A1	NR4A1.value > threshold=1.0725348591804504
node_1622: feature_name=HLA-DQA2	HLA-DQA2.value > threshold=1.9496816992759705
node_2350: feature_name=BCL2A1	BCL2A1.value <= threshold=1.1322240233421326
node_2351: feature_name=CD83	CD83.value > threshold=2.3162450790405273
node_2391: feature_name=CD79B	CD79B.value <= threshold=1.7111427783966064

node_2392: feature_name=TTN	TTN.value <= threshold=-0.28553803265094757
node_2393: feature_name=LDLRAD4	LDLRAD4.value <= threshold=3.3328956365585327
node_2394: feature_name=BCL2A1	BCL2A1.value <= threshold=-0.5811292827129364
node_2395: feature_name=HLA-DQB1	HLA-DQB1.value > threshold=1.635683536529541
node_2401: feature_name=RNF144B	RNF144B.value <= threshold=0.8731776028871536
Class: COVID-19 Critical	
Rules_614	passed counts:7
node_0: feature_name=LTB	LTB.value <= threshold=0.994031548500061
node_1: feature_name=MTRNR2L2	MTRNR2L2.value <= threshold=2.8784297704696655
node_2: feature_name=TCL1A	TCL1A.value <= threshold=4.798312664031982
node_3: feature_name=MT-CO1	MT-CO1.value <= threshold=0.08947191759943962
node_4: feature_name=NR4A1	NR4A1.value > threshold=1.0725348591804504
node_1622: feature_name=HLA-DQA2	HLA-DQA2.value <= threshold=1.9496816992759705
node_1623: feature_name=HLA-DRB5	HLA-DRB5.value <= threshold=2.715766429901123
node_1624: feature_name=HLA-DPA1	HLA-DPA1.value > threshold=1.7050301432609558
node_2056: feature_name=OAZ1	OAZ1.value <= threshold=0.456103190779686
node_2057: feature_name=HLA-DRB5	HLA-DRB5.value > threshold=0.6839984953403473
node_2101: feature_name=HLA-DRB1	HLA-DRB1.value > threshold=1.6199676990509033
node_2151: feature_name=HLA-DQB1	HLA-DQB1.value > threshold=3.1961567401885986
node_2257: feature_name=XAF1	XAF1.value > threshold=2.217243790626526
Class: COVID-19 Critical	
Rules_615	passed counts:7
node_0: feature_name=LTB	LTB.value <= threshold=0.994031548500061
node_1: feature_name=MTRNR2L2	MTRNR2L2.value <= threshold=2.8784297704696655
node_2: feature_name=TCL1A	TCL1A.value <= threshold=4.798312664031982
node_3: feature_name=MT-CO1	MT-CO1.value <= threshold=0.08947191759943962
node_4: feature_name=NR4A1	NR4A1.value > threshold=1.0725348591804504
node_1622: feature_name=HLA-DQA2	HLA-DQA2.value <= threshold=1.9496816992759705

node_1623: feature_name=HLA-DRB5	HLA-DRB5.value <= threshold=2.715766429901123
node_1624: feature_name=HLA-DPA1	HLA-DPA1.value > threshold=1.7050301432609558
node_2056: feature_name=OAZ1	OAZ1.value <= threshold=0.456103190779686
node_2057: feature_name=HLA-DRB5	HLA-DRB5.value > threshold=0.6839984953403473
node_2101: feature_name=HLA-DRB1	HLA-DRB1.value > threshold=1.6199676990509033
node_2151: feature_name=HLA-DQB1	HLA-DQB1.value <= threshold=3.1961567401885986
node_2152: feature_name=PLD4	PLD4.value <= threshold=-0.03259922005236149
node_2153: feature_name=XIST	XIST.value <= threshold=-0.138966154307127
node_2154: feature_name=HLA-DQA1	HLA-DQA1.value > threshold=2.0848299264907837
node_2190: feature_name=RALGPS2	RALGPS2.value <= threshold=4.276920795440674
node_2191: feature_name=RGS2	RGS2.value > threshold=-0.7087429165840149
node_2195: feature_name=GRAP2	GRAP2.value <= threshold=2.2513980865478516
node_2196: feature_name=ID3	ID3.value <= threshold=5.227530002593994
node_2197: feature_name=CD79B	CD79B.value <= threshold=4.226724624633789
node_2198: feature_name=ENDOD1	ENDOD1.value <= threshold=4.877119541168213
node_2199: feature_name=AP3M2	AP3M2.value <= threshold=2.6568219661712646
node_2200: feature_name=ICAM1	ICAM1.value <= threshold=4.384357690811157
node_2201: feature_name=ILK	ILK.value > threshold=2.051138460636139
node_2225: feature_name=ACSL1	ACSL1.value > threshold=-0.3219778835773468
Class: COVID-19 Critical	
Rules_616	passed counts:7
node_0: feature_name=LTB	LTB.value <= threshold=0.994031548500061
node_1: feature_name=MTRNR2L2	MTRNR2L2.value <= threshold=2.8784297704696655
node_2: feature_name=TCL1A	TCL1A.value <= threshold=4.798312664031982
node_3: feature_name=MT-CO1	MT-CO1.value <= threshold=0.08947191759943962
node_4: feature_name=NR4A1	NR4A1.value > threshold=1.0725348591804504
node_1622: feature_name=HLA-DQA2	HLA-DQA2.value <= threshold=1.9496816992759705
node_1623: feature_name=HLA-DRB5	HLA-DRB5.value <= threshold=2.715766429901123
node_1624: feature_name=HLA-DPA1	HLA-DPA1.value > threshold=1.7050301432609558

node_2056: feature_name=OAZ1	OAZ1.value <= threshold=0.456103190779686
node_2057: feature_name=HLA-DRB5	HLA-DRB5.value > threshold=0.6839984953403473
node_2101: feature_name=HLA-DRB1	HLA-DRB1.value > threshold=1.6199676990509033
node_2151: feature_name=HLA-DQB1	HLA-DQB1.value <= threshold=3.1961567401885986
node_2152: feature_name=PLD4	PLD4.value <= threshold=-0.03259922005236149
node_2153: feature_name=XIST	XIST.value <= threshold=-0.138966154307127
node_2154: feature_name=HLA-DQA1	HLA-DQA1.value <= threshold=2.0848299264907837
node_2155: feature_name=LTB	LTB.value > threshold=-0.9993145167827606
node_2161: feature_name=CYBB	CYBB.value <= threshold=1.514441728591919
node_2162: feature_name=CD83	CD83.value > threshold=3.348180890083313
node_2178: feature_name=HLA-DRB1	HLA-DRB1.value <= threshold=2.1453733444213867
node_2179: feature_name=EGR1	EGR1.value <= threshold=5.271394968032837
Class: COVID-19 Severe	
Rules_617	passed counts:7
node_0: feature_name=LTB	LTB.value <= threshold=0.994031548500061
node_1: feature_name=MTRNR2L2	MTRNR2L2.value <= threshold=2.8784297704696655
node_2: feature_name=TCL1A	TCL1A.value <= threshold=4.798312664031982
node_3: feature_name=MT-CO1	MT-CO1.value <= threshold=0.08947191759943962
node_4: feature_name=NR4A1	NR4A1.value > threshold=1.0725348591804504
node_1622: feature_name=HLA-DQA2	HLA-DQA2.value <= threshold=1.9496816992759705
node_1623: feature_name=HLA-DRB5	HLA-DRB5.value <= threshold=2.715766429901123
node_1624: feature_name=HLA-DPA1	HLA-DPA1.value <= threshold=1.7050301432609558
node_1625: feature_name=CD83	CD83.value > threshold=2.783489227294922
node_1873: feature_name=IFI44L	IFI44L.value <= threshold=1.5938722491264343
node_1874: feature_name=PLD4	PLD4.value <= threshold=4.101001739501953
node_1875: feature_name=RHOB	RHOB.value > threshold=1.304835319519043
node_1965: feature_name=HLA-DQB1	HLA-DQB1.value > threshold=1.5989137291908264
node_1991: feature_name=COTL1	COTL1.value > threshold=-1.2334879636764526
node_1997: feature_name=HSP90B1	HSP90B1.value <= threshold=1.903715431690216

node_1998: feature_name=NFKBID	NFKBID.value <= threshold=1.8163637518882751
node_1999: feature_name=IFI6	IFI6.value <= threshold=-0.5193088352680206
node_2000: feature_name=LTB	LTB.value <= threshold=0.580909252166748
node_2001: feature_name=PELI1	PELI1.value > threshold=0.9547452330589294
node_2005: feature_name=MAP3K2	MAP3K2.value > threshold=-0.48303911089897156
node_2007: feature_name=MT-CYB	MT-CYB.value <= threshold=0.129111610352993
Class: COVID-19 Critical	
Rules_618	passed counts:7
node_0: feature_name=LTB	LTB.value <= threshold=0.994031548500061
node_1: feature_name=MTRNR2L2	MTRNR2L2.value <= threshold=2.8784297704696655
node_2: feature_name=TCL1A	TCL1A.value <= threshold=4.798312664031982
node_3: feature_name=MT-CO1	MT-CO1.value <= threshold=0.08947191759943962
node_4: feature_name=NR4A1	NR4A1.value > threshold=1.0725348591804504
node_1622: feature_name=HLA-DQA2	HLA-DQA2.value <= threshold=1.9496816992759705
node_1623: feature_name=HLA-DRB5	HLA-DRB5.value <= threshold=2.715766429901123
node_1624: feature_name=HLA-DPA1	HLA-DPA1.value <= threshold=1.7050301432609558
node_1625: feature_name=CD83	CD83.value > threshold=2.783489227294922
node_1873: feature_name=IFI44L	IFI44L.value <= threshold=1.5938722491264343
node_1874: feature_name=PLD4	PLD4.value <= threshold=4.101001739501953
node_1875: feature_name=RHOB	RHOB.value <= threshold=1.304835319519043
node_1876: feature_name=PPIF	PPIF.value <= threshold=2.2943923473358154
node_1877: feature_name=OAZ1	OAZ1.value > threshold=0.04260866530239582
node_1935: feature_name=RGS1	RGS1.value <= threshold=-0.13325228542089462
node_1936: feature_name=BCL2A1	BCL2A1.value <= threshold=2.528669595718384
node_1937: feature_name=RALGPS2	RALGPS2.value > threshold=2.1082775592803955
node_1945: feature_name=MT-ND6	MT-ND6.value > threshold=-0.9341086447238922
node_1947: feature_name=RHOB	RHOB.value > threshold=-0.4660041630268097
node_1951: feature_name=MCTP1	MCTP1.value <= threshold=-0.19373653829097748
Class: Healthy Control	

Rules_619	passed counts:7
node_0: feature_name=LTB	LTB.value <= threshold=0.994031548500061
node_1: feature_name=MTRNR2L2	MTRNR2L2.value <= threshold=2.8784297704696655
node_2: feature_name=TCL1A	TCL1A.value <= threshold=4.798312664031982
node_3: feature_name=MT-CO1	MT-CO1.value <= threshold=0.08947191759943962
node_4: feature_name=NR4A1	NR4A1.value > threshold=1.0725348591804504
node_1622: feature_name=HLA-DQA2	HLA-DQA2.value <= threshold=1.9496816992759705
node_1623: feature_name=HLA-DRB5	HLA-DRB5.value <= threshold=2.715766429901123
node_1624: feature_name=HLA-DPA1	HLA-DPA1.value <= threshold=1.7050301432609558
node_1625: feature_name=CD83	CD83.value <= threshold=2.783489227294922
node_1626: feature_name=XIST	XIST.value <= threshold=0.9589543007314205
node_1627: feature_name=PLD4	PLD4.value <= threshold=5.865932464599609
node_1628: feature_name=OAZ1	OAZ1.value <= threshold=0.6137169003486633
node_1629: feature_name=MTRNR2L2	MTRNR2L2.value <= threshold=1.6102792620658875
node_1630: feature_name=IFI6	IFI6.value > threshold=-0.8404486179351807
node_1632: feature_name=NR4A1	NR4A1.value > threshold=1.0916320085525513
node_1638: feature_name=DENND3	DENND3.value <= threshold=3.988452672958374
node_1639: feature_name=HLA-DRB1	HLA-DRB1.value <= threshold=1.4092575907707214
node_1640: feature_name=HLA-DPA1	HLA-DPA1.value <= threshold=1.2675696015357971
node_1641: feature_name=TRAF1	TRAF1.value <= threshold=2.918454885482788
node_1642: feature_name=HLA-DPA1	HLA-DPA1.value > threshold=-0.917132705450058
node_1648: feature_name=LTB	LTB.value <= threshold=0.9716593027114868
node_1649: feature_name=LDLRAD4	LDLRAD4.value <= threshold=4.4251909255981445
node_1650: feature_name=BCL2A1	BCL2A1.value > threshold=-0.7959703803062439
node_1652: feature_name=PRDM1	PRDM1.value <= threshold=2.6821553707122803
node_1653: feature_name=FAM129C	FAM129C.value <= threshold=4.9084858894348145
node_1654: feature_name=BOD1L1	BOD1L1.value <= threshold=2.9830023050308228
node_1655: feature_name=HSP90B1	HSP90B1.value <= threshold=2.581766724586487
node_1656: feature_name=HLA-DQB1	HLA-DQB1.value <= threshold=3.3723777532577515

node_1657: feature_name=ANO6	ANO6.value <= threshold=2.491254210472107
node_1658: feature_name=RP5-821D11.7	RP5-821D11.7.value <= threshold=3.2165164947509766
node_1659: feature_name=MARCH1	MARCH1.value <= threshold=4.2638514041900635
node_1660: feature_name=ABCA1	ABCA1.value <= threshold=3.618377923965454
node_1661: feature_name=LTB	LTB.value <= threshold=0.8862588405609131
node_1662: feature_name=SNORD3B-2	SNORD3B-2.value > threshold=4.273592233657837
node_1680: feature_name=HSP90B1	HSP90B1.value <= threshold=1.3086768984794617
Class: COVID-19 Critical	
Rules_620	passed counts:7
node_0: feature_name=LTB	LTB.value <= threshold=0.994031548500061
node_1: feature_name=MTRNR2L2	MTRNR2L2.value <= threshold=2.8784297704696655
node_2: feature_name=TCL1A	TCL1A.value <= threshold=4.798312664031982
node_3: feature_name=MT-CO1	MT-CO1.value <= threshold=0.08947191759943962
node_4: feature_name=NR4A1	NR4A1.value <= threshold=1.0725348591804504
node_5: feature_name=HLA-DPA1	HLA-DPA1.value > threshold=1.6885469555854797
node_861: feature_name=CD83	CD83.value > threshold=3.0563642978668213
node_1539: feature_name=ID1	ID1.value <= threshold=1.4626862406730652
node_1540: feature_name=FTH1	FTH1.value <= threshold=0.2985198348760605
node_1541: feature_name=SESN3	SESN3.value <= threshold=2.7280690670013428
node_1542: feature_name=HOPX	HOPX.value > threshold=-0.46606285870075226
node_1580: feature_name=RALGPS2	RALGPS2.value <= threshold=2.509079933166504
node_1581: feature_name=IGJ	IGJ.value <= threshold=2.693845212459564
Class: COVID-19 Severe	
Rules_621	passed counts:7
node_0: feature_name=LTB	LTB.value <= threshold=0.994031548500061
node_1: feature_name=MTRNR2L2	MTRNR2L2.value <= threshold=2.8784297704696655
node_2: feature_name=TCL1A	TCL1A.value <= threshold=4.798312664031982
node_3: feature_name=MT-CO1	MT-CO1.value <= threshold=0.08947191759943962

node_4: feature_name=NR4A1	NR4A1.value <= threshold=1.0725348591804504
node_5: feature_name=HLA-DPA1	HLA-DPA1.value > threshold=1.6885469555854797
node_861: feature_name=CD83	CD83.value <= threshold=3.0563642978668213
node_862: feature_name=HLA-DQB1	HLA-DQB1.value > threshold=2.61081063747406
node_1358: feature_name=HLA-DRB5	HLA-DRB5.value > threshold=0.7542744874954224
node_1390: feature_name=HLA-DRB5	HLA-DRB5.value <= threshold=2.6244447231292725
node_1391: feature_name=XIST	XIST.value > threshold=-0.22017984092235565
node_1497: feature_name=HLA-DRB5	HLA-DRB5.value > threshold=1.2674232721328735
node_1503: feature_name=HLA-DQA1	HLA-DQA1.value <= threshold=2.4817473888397217
node_1504: feature_name=HLA-DQB1	HLA-DQB1.value > threshold=2.675566077232361
node_1506: feature_name=WDR74	WDR74.value <= threshold=0.3838092237710953
Class: COVID-19 Severe	
Rules_622	passed counts:7
node_0: feature_name=LTB	LTB.value <= threshold=0.994031548500061
node_1: feature_name=MTRNR2L2	MTRNR2L2.value <= threshold=2.8784297704696655
node_2: feature_name=TCL1A	TCL1A.value <= threshold=4.798312664031982
node_3: feature_name=MT-CO1	MT-CO1.value <= threshold=0.08947191759943962
node_4: feature_name=NR4A1	NR4A1.value <= threshold=1.0725348591804504
node_5: feature_name=HLA-DPA1	HLA-DPA1.value > threshold=1.6885469555854797
node_861: feature_name=CD83	CD83.value <= threshold=3.0563642978668213
node_862: feature_name=HLA-DQB1	HLA-DQB1.value > threshold=2.61081063747406
node_1358: feature_name=HLA-DRB5	HLA-DRB5.value > threshold=0.7542744874954224
node_1390: feature_name=HLA-DRB5	HLA-DRB5.value <= threshold=2.6244447231292725
node_1391: feature_name=XIST	XIST.value <= threshold=-0.22017984092235565
node_1392: feature_name=MT-CO3	MT-CO3.value <= threshold=0.12647542357444763
node_1393: feature_name=CD79B	CD79B.value <= threshold=4.338301658630371
node_1394: feature_name=LTB	LTB.value <= threshold=0.9865012466907501
node_1395: feature_name=MEF2C	MEF2C.value <= threshold=4.133931398391724
node_1396: feature_name=MEF2C	MEF2C.value > threshold=-0.38076959550380707

node_1418: feature_name=HLA-DPB1	HLA-DPB1.value <= threshold=0.8961269855499268
node_1419: feature_name=IRF8	IRF8.value <= threshold=3.3565738201141357
Class: COVID-19 Critical	
Rules_623	passed counts:7
node_0: feature_name=LTB	LTB.value <= threshold=0.994031548500061
node_1: feature_name=MTRNR2L2	MTRNR2L2.value <= threshold=2.8784297704696655
node_2: feature_name=TCL1A	TCL1A.value <= threshold=4.798312664031982
node_3: feature_name=MT-CO1	MT-CO1.value <= threshold=0.08947191759943962
node_4: feature_name=NR4A1	NR4A1.value <= threshold=1.0725348591804504
node_5: feature_name=HLA-DPA1	HLA-DPA1.value > threshold=1.6885469555854797
node_861: feature_name=CD83	CD83.value <= threshold=3.0563642978668213
node_862: feature_name=HLA-DQB1	HLA-DQB1.value <= threshold=2.61081063747406
node_863: feature_name=MTRNR2L2	MTRNR2L2.value <= threshold=2.0919997692108154
node_864: feature_name=BCL2A1	BCL2A1.value <= threshold=1.852618932723999
node_865: feature_name=CD79B	CD79B.value <= threshold=4.352142333984375
node_866: feature_name=TNFAIP3	TNFAIP3.value > threshold=-0.0449496041983366
node_1236: feature_name=HLA-DRB5	HLA-DRB5.value > threshold=1.710871696472168
node_1268: feature_name=MT-CO1	MT-CO1.value <= threshold=-0.8004095554351807
node_1269: feature_name=MTRNR2L8	MTRNR2L8.value > threshold=-0.3383208364248276
node_1279: feature_name=MT-CO2	MT-CO2.value <= threshold=-0.16840557008981705
Class: COVID-19 Critical	
Rules_624	passed counts:7
node_0: feature_name=LTB	LTB.value <= threshold=0.994031548500061
node_1: feature_name=MTRNR2L2	MTRNR2L2.value <= threshold=2.8784297704696655
node_2: feature_name=TCL1A	TCL1A.value <= threshold=4.798312664031982
node_3: feature_name=MT-CO1	MT-CO1.value <= threshold=0.08947191759943962
node_4: feature_name=NR4A1	NR4A1.value <= threshold=1.0725348591804504
node_5: feature_name=HLA-DPA1	HLA-DPA1.value > threshold=1.6885469555854797

node_861: feature_name=CD83	CD83.value <= threshold=3.0563642978668213
node_862: feature_name=HLA-DQB1	HLA-DQB1.value <= threshold=2.61081063747406
node_863: feature_name=MTRNR2L2	MTRNR2L2.value <= threshold=2.0919997692108154
node_864: feature_name=BCL2A1	BCL2A1.value <= threshold=1.852618932723999
node_865: feature_name=CD79B	CD79B.value <= threshold=4.352142333984375
node_866: feature_name=TNFAIP3	TNFAIP3.value <= threshold=-0.0449496041983366
node_867: feature_name=MT-CO1	MT-CO1.value <= threshold=-0.08531957119703293
node_868: feature_name=MTRNR2L8	MTRNR2L8.value <= threshold=0.8770350813865662
node_869: feature_name=EGR1	EGR1.value <= threshold=-0.32270750403404236
node_870: feature_name=IGJ	IGJ.value <= threshold=4.618318557739258
node_871: feature_name=DUSP4	DUSP4.value <= threshold=2.8197922706604004
node_872: feature_name=HOPX	HOPX.value <= threshold=2.10397732257843
node_873: feature_name=RGCC	RGCC.value <= threshold=1.2070720195770264
node_874: feature_name=OAZ1	OAZ1.value <= threshold=1.02116060256958
node_875: feature_name=NAIP	NAIP.value <= threshold=3.768904685974121
node_876: feature_name=LDLR	LDLR.value <= threshold=2.9582698345184326
node_877: feature_name=HLA-DPA1	HLA-DPA1.value <= threshold=3.0588693618774414
node_878: feature_name=RHOB	RHOB.value <= threshold=5.08755898475647
node_879: feature_name=SYTL3	SYTL3.value <= threshold=1.5127440690994263
node_880: feature_name=FTH1	FTH1.value > threshold=-1.4152275919914246
node_882: feature_name=CCL3	CCL3.value <= threshold=2.253131687641144
node_883: feature_name=CD83	CD83.value <= threshold=2.8566592931747437
node_884: feature_name=HLA-DRB5	HLA-DRB5.value > threshold=-0.6464254856109619
node_888: feature_name=HSP90B1	HSP90B1.value <= threshold=-1.186358630657196
node_889: feature_name=NFKBID	NFKBID.value > threshold=-0.39772652089595795
Class: COVID-19 Critical	
Rules_625	passed counts:7
node_0: feature_name=LTB	LTB.value <= threshold=0.994031548500061
node_1: feature_name=MTRNR2L2	MTRNR2L2.value <= threshold=2.8784297704696655

node_2: feature_name=TCL1A	TCL1A.value <= threshold=4.798312664031982
node_3: feature_name=MT-CO1	MT-CO1.value <= threshold=0.08947191759943962
node_4: feature_name=NR4A1	NR4A1.value <= threshold=1.0725348591804504
node_5: feature_name=HLA-DPA1	HLA-DPA1.value <= threshold=1.6885469555854797
node_6: feature_name=HLA-DRB5	HLA-DRB5.value > threshold=2.1886194944381714
node_738: feature_name=HLA-DQA2	HLA-DQA2.value <= threshold=-0.20105575770139694
node_739: feature_name=HLA-DQA1	HLA-DQA1.value > threshold=1.7414769530296326
node_763: feature_name=HLA-DRB5	HLA-DRB5.value > threshold=2.6777851581573486
node_799: feature_name=NFKBID	NFKBID.value <= threshold=2.063578724861145
Class: COVID-19 Severe	
Rules_626	passed counts:7
node_0: feature_name=LTB	LTB.value <= threshold=0.994031548500061
node_1: feature_name=MTRNR2L2	MTRNR2L2.value <= threshold=2.8784297704696655
node_2: feature_name=TCL1A	TCL1A.value <= threshold=4.798312664031982
node_3: feature_name=MT-CO1	MT-CO1.value <= threshold=0.08947191759943962
node_4: feature_name=NR4A1	NR4A1.value <= threshold=1.0725348591804504
node_5: feature_name=HLA-DPA1	HLA-DPA1.value <= threshold=1.6885469555854797
node_6: feature_name=HLA-DRB5	HLA-DRB5.value > threshold=2.1886194944381714
node_738: feature_name=HLA-DQA2	HLA-DQA2.value <= threshold=-0.20105575770139694
node_739: feature_name=HLA-DQA1	HLA-DQA1.value <= threshold=1.7414769530296326
node_740: feature_name=TNFAIP3	TNFAIP3.value > threshold=-0.5488012433052063
node_750: feature_name=SNX9	SNX9.value <= threshold=0.20590564608573914
node_751: feature_name=KIAA0125	KIAA0125.value <= threshold=4.1270952224731445
Class: COVID-19 Severe	
Rules_627	passed counts:7
node_0: feature_name=LTB	LTB.value <= threshold=0.994031548500061
node_1: feature_name=MTRNR2L2	MTRNR2L2.value <= threshold=2.8784297704696655
node_2: feature_name=TCL1A	TCL1A.value <= threshold=4.798312664031982

node_3: feature_name=MT-CO1	MT-CO1.value <= threshold=0.08947191759943962
node_4: feature_name=NR4A1	NR4A1.value <= threshold=1.0725348591804504
node_5: feature_name=HLA-DPA1	HLA-DPA1.value <= threshold=1.6885469555854797
node_6: feature_name=HLA-DRB5	HLA-DRB5.value <= threshold=2.1886194944381714
node_7: feature_name=XIST	XIST.value <= threshold=-0.12328458577394485
node_8: feature_name=SYNE1	SYNE1.value > threshold=-0.7835594415664673
node_16: feature_name=MTRNR2L2	MTRNR2L2.value <= threshold=2.4483247995376587
node_17: feature_name=HLA-DQA2	HLA-DQA2.value > threshold=2.227178931236267
node_591: feature_name=BCL2A1	BCL2A1.value <= threshold=1.0648428201675415
node_592: feature_name=FTH1	FTH1.value <= threshold=0.8842596709728241
node_593: feature_name=SNX9	SNX9.value > threshold=3.232524275779724
node_671: feature_name=IRF4	IRF4.value <= threshold=-0.20697960257530212
node_672: feature_name=BANK1	BANK1.value <= threshold=3.245829224586487
Class: COVID-19 Severe	
Rules_628	passed counts:7
node_0: feature_name=LTB	LTB.value <= threshold=0.994031548500061
node_1: feature_name=MTRNR2L2	MTRNR2L2.value <= threshold=2.8784297704696655
node_2: feature_name=TCL1A	TCL1A.value <= threshold=4.798312664031982
node_3: feature_name=MT-CO1	MT-CO1.value <= threshold=0.08947191759943962
node_4: feature_name=NR4A1	NR4A1.value <= threshold=1.0725348591804504
node_5: feature_name=HLA-DPA1	HLA-DPA1.value <= threshold=1.6885469555854797
node_6: feature_name=HLA-DRB5	HLA-DRB5.value <= threshold=2.1886194944381714
node_7: feature_name=XIST	XIST.value <= threshold=-0.12328458577394485
node_8: feature_name=SYNE1	SYNE1.value > threshold=-0.7835594415664673
node_16: feature_name=MTRNR2L2	MTRNR2L2.value <= threshold=2.4483247995376587
node_17: feature_name=HLA-DQA2	HLA-DQA2.value <= threshold=2.227178931236267
node_18: feature_name=NR4A1	NR4A1.value <= threshold=-0.21380723267793655
node_19: feature_name=IRAK3	IRAK3.value <= threshold=4.27159571647644
node_20: feature_name=IFIT2	IFIT2.value <= threshold=7.8175740242004395

node_21: feature_name=CD79A	CD79A.value > threshold=2.857242226600647
node_375: feature_name=LTB	LTB.value > threshold=0.7405790388584137
node_473: feature_name=MS4A1	MS4A1.value <= threshold=2.8195884227752686
node_474: feature_name=HLA-DQA1	HLA-DQA1.value > threshold=-0.44217973947525024
node_478: feature_name=IGLL5	IGLL5.value > threshold=-0.25717009603977203
node_480: feature_name=RGS2	RGS2.value > threshold=1.253660798072815
node_484: feature_name=TSPYL2	TSPYL2.value <= threshold=-0.5976825058460236
Class: COVID-19 Critical	
Rules_629	passed counts:7
node_0: feature_name=LTB	LTB.value <= threshold=0.994031548500061
node_1: feature_name=MTRNR2L2	MTRNR2L2.value <= threshold=2.8784297704696655
node_2: feature_name=TCL1A	TCL1A.value <= threshold=4.798312664031982
node_3: feature_name=MT-CO1	MT-CO1.value <= threshold=0.08947191759943962
node_4: feature_name=NR4A1	NR4A1.value <= threshold=1.0725348591804504
node_5: feature_name=HLA-DPA1	HLA-DPA1.value <= threshold=1.6885469555854797
node_6: feature_name=HLA-DRB5	HLA-DRB5.value <= threshold=2.1886194944381714
node_7: feature_name=XIST	XIST.value <= threshold=-0.12328458577394485
node_8: feature_name=SYNE1	SYNE1.value > threshold=-0.7835594415664673
node_16: feature_name=MTRNR2L2	MTRNR2L2.value <= threshold=2.4483247995376587
node_17: feature_name=HLA-DQA2	HLA-DQA2.value <= threshold=2.227178931236267
node_18: feature_name=NR4A1	NR4A1.value <= threshold=-0.21380723267793655
node_19: feature_name=IRAK3	IRAK3.value <= threshold=4.27159571647644
node_20: feature_name=IFIT2	IFIT2.value <= threshold=7.8175740242004395
node_21: feature_name=CD79A	CD79A.value <= threshold=2.857242226600647
node_22: feature_name=HIST1H2AC	HIST1H2AC.value <= threshold=5.077470302581787
node_23: feature_name=FTH1	FTH1.value <= threshold=0.4158056080341339
node_24: feature_name=ATF3	ATF3.value <= threshold=3.8028353452682495
node_25: feature_name=OASL	OASL.value <= threshold=3.387580633163452
node_26: feature_name=MT-CO1	MT-CO1.value <= threshold=-0.7942646145820618

node_27: feature_name=FCER2	FCER2.value <= threshold=7.268347978591919
node_28: feature_name=CCL3	CCL3.value <= threshold=3.2822797298431396
node_29: feature_name=BCL2A1	BCL2A1.value <= threshold=3.1504160165786743
node_30: feature_name=GRAP2	GRAP2.value <= threshold=3.0517499446868896
node_31: feature_name=EGR1	EGR1.value <= threshold=7.645251512527466
node_32: feature_name=GPX1	GPX1.value <= threshold=0.6752602756023407
node_33: feature_name=CD79B	CD79B.value > threshold=4.365710258483887
node_55: feature_name=CD79B	CD79B.value > threshold=4.435514450073242
Class: COVID-19 Critical	
Rules_630	passed counts:7
node_0: feature_name=LTB	LTB.value <= threshold=0.994031548500061
node_1: feature_name=MTRNR2L2	MTRNR2L2.value <= threshold=2.8784297704696655
node_2: feature_name=TCL1A	TCL1A.value <= threshold=4.798312664031982
node_3: feature_name=MT-CO1	MT-CO1.value <= threshold=0.08947191759943962
node_4: feature_name=NR4A1	NR4A1.value <= threshold=1.0725348591804504
node_5: feature_name=HLA-DPA1	HLA-DPA1.value <= threshold=1.6885469555854797
node_6: feature_name=HLA-DRB5	HLA-DRB5.value <= threshold=2.1886194944381714
node_7: feature_name=XIST	XIST.value <= threshold=-0.12328458577394485
node_8: feature_name=SYNE1	SYNE1.value > threshold=-0.7835594415664673
node_16: feature_name=MTRNR2L2	MTRNR2L2.value <= threshold=2.4483247995376587
node_17: feature_name=HLA-DQA2	HLA-DQA2.value <= threshold=2.227178931236267
node_18: feature_name=NR4A1	NR4A1.value <= threshold=-0.21380723267793655
node_19: feature_name=IRAK3	IRAK3.value <= threshold=4.27159571647644
node_20: feature_name=IFIT2	IFIT2.value <= threshold=7.8175740242004395
node_21: feature_name=CD79A	CD79A.value <= threshold=2.857242226600647
node_22: feature_name=HIST1H2AC	HIST1H2AC.value <= threshold=5.077470302581787
node_23: feature_name=FTH1	FTH1.value <= threshold=0.4158056080341339
node_24: feature_name=ATF3	ATF3.value <= threshold=3.8028353452682495
node_25: feature_name=OASL	OASL.value <= threshold=3.387580633163452

node_26: feature_name=MT-CO1	MT-CO1.value <= threshold=-0.7942646145820618
node_27: feature_name=FCER2	FCER2.value <= threshold=7.268347978591919
node_28: feature_name=CCL3	CCL3.value <= threshold=3.2822797298431396
node_29: feature_name=BCL2A1	BCL2A1.value <= threshold=3.1504160165786743
node_30: feature_name=GRAP2	GRAP2.value <= threshold=3.0517499446868896
node_31: feature_name=EGR1	EGR1.value <= threshold=7.645251512527466
node_32: feature_name=GPX1	GPX1.value <= threshold=0.6752602756023407
node_33: feature_name=CD79B	CD79B.value <= threshold=4.365710258483887
node_34: feature_name=S100A6	S100A6.value > threshold=0.4172801077365875
node_52: feature_name=TPM1	TPM1.value <= threshold=-0.16594571620225906
Class: COVID-19 Critical	
Rules_631	passed counts:7
node_0: feature_name=LTB	LTB.value <= threshold=0.994031548500061
node_1: feature_name=MTRNR2L2	MTRNR2L2.value <= threshold=2.8784297704696655
node_2: feature_name=TCL1A	TCL1A.value <= threshold=4.798312664031982
node_3: feature_name=MT-CO1	MT-CO1.value <= threshold=0.08947191759943962
node_4: feature_name=NR4A1	NR4A1.value <= threshold=1.0725348591804504
node_5: feature_name=HLA-DPA1	HLA-DPA1.value <= threshold=1.6885469555854797
node_6: feature_name=HLA-DRB5	HLA-DRB5.value <= threshold=2.1886194944381714
node_7: feature_name=XIST	XIST.value <= threshold=-0.12328458577394485
node_8: feature_name=SYNE1	SYNE1.value > threshold=-0.7835594415664673
node_16: feature_name=MTRNR2L2	MTRNR2L2.value <= threshold=2.4483247995376587
node_17: feature_name=HLA-DQA2	HLA-DQA2.value <= threshold=2.227178931236267
node_18: feature_name=NR4A1	NR4A1.value <= threshold=-0.21380723267793655
node_19: feature_name=IRAK3	IRAK3.value <= threshold=4.27159571647644
node_20: feature_name=IFIT2	IFIT2.value <= threshold=7.8175740242004395
node_21: feature_name=CD79A	CD79A.value <= threshold=2.857242226600647
node_22: feature_name=HIST1H2AC	HIST1H2AC.value <= threshold=5.077470302581787
node_23: feature_name=FTH1	FTH1.value <= threshold=0.4158056080341339

node_24: feature_name=ATF3	ATF3.value <= threshold=3.8028353452682495
node_25: feature_name=OASL	OASL.value <= threshold=3.387580633163452
node_26: feature_name=MT-CO1	MT-CO1.value <= threshold=-0.7942646145820618
node_27: feature_name=FCER2	FCER2.value <= threshold=7.268347978591919
node_28: feature_name=CCL3	CCL3.value <= threshold=3.2822797298431396
node_29: feature_name=BCL2A1	BCL2A1.value <= threshold=3.1504160165786743
node_30: feature_name=GRAP2	GRAP2.value <= threshold=3.0517499446868896
node_31: feature_name=EGR1	EGR1.value <= threshold=7.645251512527466
node_32: feature_name=GPX1	GPX1.value <= threshold=0.6752602756023407
node_33: feature_name=CD79B	CD79B.value <= threshold=4.365710258483887
node_34: feature_name=S100A6	S100A6.value <= threshold=0.4172801077365875
node_35: feature_name=ID3	ID3.value <= threshold=3.093442916870117
node_36: feature_name=ATF3	ATF3.value > threshold=2.6047290563583374
node_42: feature_name=NAMPT	NAMPT.value > threshold=-0.5723253190517426
Class: COVID-19 Critical	
Rules_632	passed counts:6
node_0: feature_name=LTB	LTB.value > threshold=0.994031548500061
node_5264: feature_name=MT-CO1	MT-CO1.value > threshold=-0.4127991199493408
node_5638: feature_name=OAZ1	OAZ1.value > threshold=-0.2640867084264755
node_6112: feature_name=IFI44L	IFI44L.value > threshold=2.5893263816833496
node_7078: feature_name=XIST	XIST.value > threshold=1.1465829275548458
node_7114: feature_name=PDLIM1	PDLIM1.value > threshold=-0.24314071238040924
Class: Healthy Control	
Rules_633	passed counts:6
node_0: feature_name=LTB	LTB.value > threshold=0.994031548500061
node_5264: feature_name=MT-CO1	MT-CO1.value > threshold=-0.4127991199493408
node_5638: feature_name=OAZ1	OAZ1.value > threshold=-0.2640867084264755
node_6112: feature_name=IFI44L	IFI44L.value > threshold=2.5893263816833496

node_7078: feature_name=XIST	XIST.value <= threshold=1.1465829275548458
node_7079: feature_name=TCL1A	TCL1A.value > threshold=7.115930080413818
node_7111: feature_name=RGS1	RGS1.value <= threshold=0.2852351665496826
Class: Healthy Control	
Rules_634	passed counts:6
node_0: feature_name=LTB	LTB.value > threshold=0.994031548500061
node_5264: feature_name=MT-CO1	MT-CO1.value > threshold=-0.4127991199493408
node_5638: feature_name=OAZ1	OAZ1.value > threshold=-0.2640867084264755
node_6112: feature_name=IFI44L	IFI44L.value <= threshold=2.5893263816833496
node_6113: feature_name=TCL1A	TCL1A.value > threshold=4.201832294464111
node_6991: feature_name=XAF1	XAF1.value <= threshold=3.4585667848587036
node_6992: feature_name=STAG3	STAG3.value > threshold=7.686291694641113
node_7072: feature_name=MT-CO1	MT-CO1.value <= threshold=0.184519425034523
Class: COVID-19 Critical	
Rules_635	passed counts:6
node_0: feature_name=LTB	LTB.value > threshold=0.994031548500061
node_5264: feature_name=MT-CO1	MT-CO1.value > threshold=-0.4127991199493408
node_5638: feature_name=OAZ1	OAZ1.value > threshold=-0.2640867084264755
node_6112: feature_name=IFI44L	IFI44L.value <= threshold=2.5893263816833496
node_6113: feature_name=TCL1A	TCL1A.value > threshold=4.201832294464111
node_6991: feature_name=XAF1	XAF1.value <= threshold=3.4585667848587036
node_6992: feature_name=STAG3	STAG3.value <= threshold=7.686291694641113
node_6993: feature_name=RALGPS2	RALGPS2.value > threshold=-0.4090157449245453
node_6997: feature_name=MTRNR2L2	MTRNR2L2.value <= threshold=3.23723828792572
node_6998: feature_name=TP53INP1	TP53INP1.value <= threshold=6.239925146102905
node_6999: feature_name=MEF2C	MEF2C.value > threshold=-0.44642284512519836
node_7003: feature_name=HSPA1A	HSPA1A.value <= threshold=7.404359817504883
node_7004: feature_name=COTL1	COTL1.value > threshold=-1.6598749160766602

node_7006: feature_name=HIST1H2AC	HIST1H2AC.value > threshold=-0.34910088777542114
node_7008: feature_name=MARCH1	MARCH1.value <= threshold=5.393903970718384
node_7009: feature_name=PELI1	PELI1.value <= threshold=3.565749168395996
node_7010: feature_name=RGS1	RGS1.value <= threshold=2.851950168609619
node_7011: feature_name=HLA-DRB5	HLA-DRB5.value <= threshold=3.0528379678726196
node_7012: feature_name=MEF2A	MEF2A.value <= threshold=3.228689193725586
node_7013: feature_name=MT-CO1	MT-CO1.value > threshold=-0.37944722175598145
node_7017: feature_name=ATG2A	ATG2A.value <= threshold=2.790610194206238
node_7018: feature_name=MEF2C	MEF2C.value > threshold=-0.404978945851326
node_7026: feature_name=MT-CO3	MT-CO3.value > threshold=-1.699916422367096
node_7030: feature_name=CD79A	CD79A.value > threshold=0.17886222898960114
node_7034: feature_name=CCDC141	CCDC141.value <= threshold=5.498055934906006
node_7035: feature_name=SNX9	SNX9.value <= threshold=2.924591064453125
node_7036: feature_name=RP5-821D11.7	RP5-821D11.7.value > threshold=4.530828952789307
node_7042: feature_name=HOPX	HOPX.value <= threshold=-0.4752810001373291
Class: Healthy Control	
Rules_636	passed counts:6
node_0: feature_name=LTB	LTB.value > threshold=0.994031548500061
node_5264: feature_name=MT-CO1	MT-CO1.value > threshold=-0.4127991199493408
node_5638: feature_name=OAZ1	OAZ1.value > threshold=-0.2640867084264755
node_6112: feature_name=IFI44L	IFI44L.value <= threshold=2.5893263816833496
node_6113: feature_name=TCL1A	TCL1A.value <= threshold=4.201832294464111
node_6114: feature_name=HLA-DQA2	HLA-DQA2.value > threshold=-0.2270243540406227
node_6542: feature_name=CD79B	CD79B.value > threshold=3.096232771873474
node_6872: feature_name=ICAM1	ICAM1.value <= threshold=-0.2232399433851242
node_6873: feature_name=STAG3	STAG3.value > threshold=-0.19312061369419098
node_6939: feature_name=HLA-DRB5	HLA-DRB5.value > threshold=2.209904909133911
node_6967: feature_name=TSPYL2	TSPYL2.value <= threshold=-0.6224541068077087
node_6968: feature_name=BANK1	BANK1.value > threshold=2.4764840602874756

Class: Healthy Control	
Rules_637	passed counts:6
node_0: feature_name=LTB	LTB.value > threshold=0.994031548500061
node_5264: feature_name=MT-CO1	MT-CO1.value > threshold=-0.4127991199493408
node_5638: feature_name=OAZ1	OAZ1.value > threshold=-0.2640867084264755
node_6112: feature_name=IFI44L	IFI44L.value <= threshold=2.5893263816833496
node_6113: feature_name=TCL1A	TCL1A.value <= threshold=4.201832294464111
node_6114: feature_name=HLA-DQA2	HLA-DQA2.value > threshold=-0.2270243540406227
node_6542: feature_name=CD79B	CD79B.value > threshold=3.096232771873474
node_6872: feature_name=ICAM1	ICAM1.value <= threshold=-0.2232399433851242
node_6873: feature_name=STAG3	STAG3.value > threshold=-0.19312061369419098
node_6939: feature_name=HLA-DRB5	HLA-DRB5.value <= threshold=2.209904909133911
node_6940: feature_name=STAG3	STAG3.value <= threshold=7.8730878829956055
node_6941: feature_name=FAM129C	FAM129C.value <= threshold=1.668714702129364
node_6942: feature_name=HLA-DPB1	HLA-DPB1.value > threshold=1.4723529815673828
node_6944: feature_name=BOD1L1	BOD1L1.value > threshold=0.9118953347206116
node_6948: feature_name=MT-CO2	MT-CO2.value > threshold=0.5889202803373337
Class: COVID-19 Critical	
Rules_638	passed counts:6
node_0: feature_name=LTB	LTB.value > threshold=0.994031548500061
node_5264: feature_name=MT-CO1	MT-CO1.value > threshold=-0.4127991199493408
node_5638: feature_name=OAZ1	OAZ1.value > threshold=-0.2640867084264755
node_6112: feature_name=IFI44L	IFI44L.value <= threshold=2.5893263816833496
node_6113: feature_name=TCL1A	TCL1A.value <= threshold=4.201832294464111
node_6114: feature_name=HLA-DQA2	HLA-DQA2.value > threshold=-0.2270243540406227
node_6542: feature_name=CD79B	CD79B.value > threshold=3.096232771873474
node_6872: feature_name=ICAM1	ICAM1.value <= threshold=-0.2232399433851242
node_6873: feature_name=STAG3	STAG3.value > threshold=-0.19312061369419098

node_6939: feature_name=HLA-DRB5	HLA-DRB5.value <= threshold=2.209904909133911
node_6940: feature_name=STAG3	STAG3.value <= threshold=7.8730878829956055
node_6941: feature_name=FAM129C	FAM129C.value <= threshold=1.668714702129364
node_6942: feature_name=HLA-DPB1	HLA-DPB1.value > threshold=1.4723529815673828
node_6944: feature_name=BOD1L1	BOD1L1.value > threshold=0.9118953347206116
node_6948: feature_name=MT-CO2	MT-CO2.value <= threshold=0.5889202803373337
node_6949: feature_name=MYL9	MYL9.value > threshold=-0.08132943883538246
Class: Healthy Control	
Rules_639	passed counts:6
node_0: feature_name=LTB	LTB.value > threshold=0.994031548500061
node_5264: feature_name=MT-CO1	MT-CO1.value > threshold=-0.4127991199493408
node_5638: feature_name=OAZ1	OAZ1.value > threshold=-0.2640867084264755
node_6112: feature_name=IFI44L	IFI44L.value <= threshold=2.5893263816833496
node_6113: feature_name=TCL1A	TCL1A.value <= threshold=4.201832294464111
node_6114: feature_name=HLA-DQA2	HLA-DQA2.value > threshold=-0.2270243540406227
node_6542: feature_name=CD79B	CD79B.value <= threshold=3.096232771873474
node_6543: feature_name=NR4A1	NR4A1.value > threshold=1.421015739440918
node_6811: feature_name=SLC7A5	SLC7A5.value <= threshold=1.570515513420105
node_6812: feature_name=XAF1	XAF1.value <= threshold=2.218220591545105
node_6813: feature_name=MEF2C	MEF2C.value <= threshold=-0.35489463806152344
node_6814: feature_name=MIR24-2	MIR24-2.value > threshold=1.5691230297088623
Class: COVID-19 Critical	
Rules_640	passed counts:6
node_0: feature_name=LTB	LTB.value > threshold=0.994031548500061
node_5264: feature_name=MT-CO1	MT-CO1.value > threshold=-0.4127991199493408
node_5638: feature_name=OAZ1	OAZ1.value > threshold=-0.2640867084264755
node_6112: feature_name=IFI44L	IFI44L.value <= threshold=2.5893263816833496
node_6113: feature_name=TCL1A	TCL1A.value <= threshold=4.201832294464111

node_6114: feature_name=HLA-DQA2	HLA-DQA2.value > threshold=-0.2270243540406227
node_6542: feature_name=CD79B	CD79B.value <= threshold=3.096232771873474
node_6543: feature_name=NR4A1	NR4A1.value <= threshold=1.421015739440918
node_6544: feature_name=LTB	LTB.value > threshold=1.359755277633667
node_6720: feature_name=NFKBIZ	NFKBIZ.value <= threshold=0.2720414251089096
node_6721: feature_name=ATF5	ATF5.value <= threshold=2.4672781229019165
node_6722: feature_name=SNX9	SNX9.value <= threshold=2.0814030170440674
node_6723: feature_name=HLA-DQA2	HLA-DQA2.value <= threshold=4.360614776611328
node_6724: feature_name=MT-CO1	MT-CO1.value <= threshold=-0.12188276648521423
node_6725: feature_name=CD79B	CD79B.value > threshold=2.3298217058181763
node_6733: feature_name=HBB	HBB.value <= threshold=0.6237644702196121
Class: Healthy Control	
Rules_641	passed counts:6
node_0: feature_name=LTB	LTB.value > threshold=0.994031548500061
node_5264: feature_name=MT-CO1	MT-CO1.value > threshold=-0.4127991199493408
node_5638: feature_name=OAZ1	OAZ1.value > threshold=-0.2640867084264755
node_6112: feature_name=IFI44L	IFI44L.value <= threshold=2.5893263816833496
node_6113: feature_name=TCL1A	TCL1A.value <= threshold=4.201832294464111
node_6114: feature_name=HLA-DQA2	HLA-DQA2.value > threshold=-0.2270243540406227
node_6542: feature_name=CD79B	CD79B.value <= threshold=3.096232771873474
node_6543: feature_name=NR4A1	NR4A1.value <= threshold=1.421015739440918
node_6544: feature_name=LTB	LTB.value <= threshold=1.359755277633667
node_6545: feature_name=NFKBID	NFKBID.value <= threshold=1.4294711351394653
node_6546: feature_name=HLA-DRB1	HLA-DRB1.value <= threshold=1.0398632287979126
node_6547: feature_name=CASP8	CASP8.value > threshold=0.6416637301445007
node_6553: feature_name=HLA-DRB5	HLA-DRB5.value > threshold=1.0622348487377167
Class: COVID-19 Critical	
Rules_642	passed counts:6

node_0: feature_name=LTB	LTB.value > threshold=0.994031548500061
node_5264: feature_name=MT-CO1	MT-CO1.value > threshold=-0.4127991199493408
node_5638: feature_name=OAZ1	OAZ1.value > threshold=-0.2640867084264755
node_6112: feature_name=IFI44L	IFI44L.value <= threshold=2.5893263816833496
node_6113: feature_name=TCL1A	TCL1A.value <= threshold=4.201832294464111
node_6114: feature_name=HLA-DQA2	HLA-DQA2.value > threshold=-0.2270243540406227
node_6542: feature_name=CD79B	CD79B.value <= threshold=3.096232771873474
node_6543: feature_name=NR4A1	NR4A1.value <= threshold=1.421015739440918
node_6544: feature_name=LTB	LTB.value <= threshold=1.359755277633667
node_6545: feature_name=NFKBID	NFKBID.value <= threshold=1.4294711351394653
node_6546: feature_name=HLA-DRB1	HLA-DRB1.value <= threshold=1.0398632287979126
node_6547: feature_name=CASP8	CASP8.value > threshold=0.6416637301445007
node_6553: feature_name=HLA-DRB5	HLA-DRB5.value <= threshold=1.0622348487377167
Class: Healthy Control	
Rules_643	passed counts:6
node_0: feature_name=LTB	LTB.value > threshold=0.994031548500061
node_5264: feature_name=MT-CO1	MT-CO1.value > threshold=-0.4127991199493408
node_5638: feature_name=OAZ1	OAZ1.value > threshold=-0.2640867084264755
node_6112: feature_name=IFI44L	IFI44L.value <= threshold=2.5893263816833496
node_6113: feature_name=TCL1A	TCL1A.value <= threshold=4.201832294464111
node_6114: feature_name=HLA-DQA2	HLA-DQA2.value <= threshold=-0.2270243540406227
node_6115: feature_name=MTRNR2L2	MTRNR2L2.value <= threshold=2.548917293548584
node_6116: feature_name=ATF5	ATF5.value > threshold=2.041263461112976
node_6490: feature_name=HLA-DPB1	HLA-DPB1.value <= threshold=1.5343759655952454
node_6491: feature_name=MT-ND6	MT-ND6.value <= threshold=1.0284687876701355
node_6492: feature_name=FAM129C	FAM129C.value > threshold=3.5579545497894287
node_6502: feature_name=RNF11	RNF11.value > threshold=-0.5562405586242676
Class: Healthy Control	

Rules_644	passed counts:6
node_0: feature_name=LTB	LTB.value > threshold=0.994031548500061
node_5264: feature_name=MT-CO1	MT-CO1.value > threshold=-0.4127991199493408
node_5638: feature_name=OAZ1	OAZ1.value > threshold=-0.2640867084264755
node_6112: feature_name=IFI44L	IFI44L.value <= threshold=2.5893263816833496
node_6113: feature_name=TCL1A	TCL1A.value <= threshold=4.201832294464111
node_6114: feature_name=HLA-DQA2	HLA-DQA2.value <= threshold=-0.2270243540406227
node_6115: feature_name=MTRNR2L2	MTRNR2L2.value <= threshold=2.548917293548584
node_6116: feature_name=ATF5	ATF5.value <= threshold=2.041263461112976
node_6117: feature_name=JUN	JUN.value > threshold=-0.616259902715683
node_6189: feature_name=MT-CO3	MT-CO3.value > threshold=0.13483521342277527
node_6351: feature_name=XAF1	XAF1.value > threshold=3.061717987060547
node_6487: feature_name=ZNF10	ZNF10.value <= threshold=1.048926368355751
Class: COVID-19 Critical	
Rules_645	passed counts:6
node_0: feature_name=LTB	LTB.value > threshold=0.994031548500061
node_5264: feature_name=MT-CO1	MT-CO1.value > threshold=-0.4127991199493408
node_5638: feature_name=OAZ1	OAZ1.value > threshold=-0.2640867084264755
node_6112: feature_name=IFI44L	IFI44L.value <= threshold=2.5893263816833496
node_6113: feature_name=TCL1A	TCL1A.value <= threshold=4.201832294464111
node_6114: feature_name=HLA-DQA2	HLA-DQA2.value <= threshold=-0.2270243540406227
node_6115: feature_name=MTRNR2L2	MTRNR2L2.value <= threshold=2.548917293548584
node_6116: feature_name=ATF5	ATF5.value <= threshold=2.041263461112976
node_6117: feature_name=JUN	JUN.value > threshold=-0.616259902715683
node_6189: feature_name=MT-CO3	MT-CO3.value > threshold=0.13483521342277527
node_6351: feature_name=XAF1	XAF1.value <= threshold=3.061717987060547
node_6352: feature_name=MT-CO2	MT-CO2.value <= threshold=1.2605181336402893
node_6353: feature_name=ICAM1	ICAM1.value > threshold=-0.3340446650981903
node_6439: feature_name=FAM129C	FAM129C.value > threshold=-0.24657993018627167

node_6455: feature_name=HSP90B1	HSP90B1.value <= threshold=1.5712243914604187
node_6456: feature_name=NR4A1	NR4A1.value <= threshold=-0.6648433208465576
node_6457: feature_name=DAPP1	DAPP1.value <= threshold=0.7670192569494247
Class: Healthy Control	
Rules_646	passed counts:6
node_0: feature_name=LTB	LTB.value > threshold=0.994031548500061
node_5264: feature_name=MT-CO1	MT-CO1.value > threshold=-0.4127991199493408
node_5638: feature_name=OAZ1	OAZ1.value > threshold=-0.2640867084264755
node_6112: feature_name=IFI44L	IFI44L.value <= threshold=2.5893263816833496
node_6113: feature_name=TCL1A	TCL1A.value <= threshold=4.201832294464111
node_6114: feature_name=HLA-DQA2	HLA-DQA2.value <= threshold=-0.2270243540406227
node_6115: feature_name=MTRNR2L2	MTRNR2L2.value <= threshold=2.548917293548584
node_6116: feature_name=ATF5	ATF5.value <= threshold=2.041263461112976
node_6117: feature_name=JUN	JUN.value > threshold=-0.616259902715683
node_6189: feature_name=MT-CO3	MT-CO3.value > threshold=0.13483521342277527
node_6351: feature_name=XAF1	XAF1.value <= threshold=3.061717987060547
node_6352: feature_name=MT-CO2	MT-CO2.value <= threshold=1.2605181336402893
node_6353: feature_name=ICAM1	ICAM1.value <= threshold=-0.3340446650981903
node_6354: feature_name=OAZ1	OAZ1.value > threshold=-0.24055946618318558
node_6360: feature_name=PPIF	PPIF.value <= threshold=2.070030093193054
node_6361: feature_name=RALGPS2	RALGPS2.value > threshold=-0.3684315085411072
node_6369: feature_name=ANO6	ANO6.value <= threshold=3.504153251647949
node_6370: feature_name=YWHAH	YWHAH.value <= threshold=4.3182291984558105
node_6371: feature_name=LDLR	LDLR.value <= threshold=2.494291305541992
node_6372: feature_name=HLA-DQA1	HLA-DQA1.value > threshold=-0.5716990232467651
node_6374: feature_name=ABCA1	ABCA1.value <= threshold=3.7662240266799927
node_6375: feature_name=MS4A1	MS4A1.value > threshold=1.4995208978652954
node_6387: feature_name=SNORD3B-2	SNORD3B-2.value <= threshold=4.154061555862427
node_6388: feature_name=LTB	LTB.value <= threshold=2.1739100217819214

node_6389: feature_name=BACH1	BACH1.value > threshold=2.0371285676956177
node_6425: feature_name=PLA2G12A	PLA2G12A.value <= threshold=-0.22552809119224548
Class: Healthy Control	
Rules_647	passed counts:6
node_0: feature_name=LTB	LTB.value > threshold=0.994031548500061
node_5264: feature_name=MT-CO1	MT-CO1.value > threshold=-0.4127991199493408
node_5638: feature_name=OAZ1	OAZ1.value > threshold=-0.2640867084264755
node_6112: feature_name=IFI44L	IFI44L.value <= threshold=2.5893263816833496
node_6113: feature_name=TCL1A	TCL1A.value <= threshold=4.201832294464111
node_6114: feature_name=HLA-DQA2	HLA-DQA2.value <= threshold=-0.2270243540406227
node_6115: feature_name=MTRNR2L2	MTRNR2L2.value <= threshold=2.548917293548584
node_6116: feature_name=ATF5	ATF5.value <= threshold=2.041263461112976
node_6117: feature_name=JUN	JUN.value > threshold=-0.616259902715683
node_6189: feature_name=MT-CO3	MT-CO3.value > threshold=0.13483521342277527
node_6351: feature_name=XAF1	XAF1.value <= threshold=3.061717987060547
node_6352: feature_name=MT-CO2	MT-CO2.value <= threshold=1.2605181336402893
node_6353: feature_name=ICAM1	ICAM1.value <= threshold=-0.3340446650981903
node_6354: feature_name=OAZ1	OAZ1.value > threshold=-0.24055946618318558
node_6360: feature_name=PPIF	PPIF.value <= threshold=2.070030093193054
node_6361: feature_name=RALGPS2	RALGPS2.value > threshold=-0.3684315085411072
node_6369: feature_name=ANO6	ANO6.value <= threshold=3.504153251647949
node_6370: feature_name=YWHAH	YWHAH.value <= threshold=4.3182291984558105
node_6371: feature_name=LDLR	LDLR.value <= threshold=2.494291305541992
node_6372: feature_name=HLA-DQA1	HLA-DQA1.value > threshold=-0.5716990232467651
node_6374: feature_name=ABCA1	ABCA1.value <= threshold=3.7662240266799927
node_6375: feature_name=MS4A1	MS4A1.value > threshold=1.4995208978652954
node_6387: feature_name=SNORD3B-2	SNORD3B-2.value <= threshold=4.154061555862427
node_6388: feature_name=LTB	LTB.value <= threshold=2.1739100217819214
node_6389: feature_name=BACH1	BACH1.value <= threshold=2.0371285676956177

node_6390: feature_name=HLA-DQB1	HLA-DQB1.value <= threshold=3.0258322954177856
node_6391: feature_name=HLA-DPB1	HLA-DPB1.value > threshold=0.2591289207339287
node_6395: feature_name=CXorf57	CXorf57.value <= threshold=3.3742183446884155
node_6396: feature_name=MMD	MMD.value <= threshold=3.8883349895477295
node_6397: feature_name=IRF8	IRF8.value <= threshold=3.939465045928955
node_6398: feature_name=PPIF	PPIF.value <= threshold=1.1284508109092712
node_6399: feature_name=S100A6	S100A6.value <= threshold=-2.1265565156936646
node_6400: feature_name=MS4A1	MS4A1.value > threshold=2.0918729305267334
Class: Healthy Control	
Rules_648	passed counts:6
node_0: feature_name=LTB	LTB.value > threshold=0.994031548500061
node_5264: feature_name=MT-CO1	MT-CO1.value > threshold=-0.4127991199493408
node_5638: feature_name=OAZ1	OAZ1.value > threshold=-0.2640867084264755
node_6112: feature_name=IFI44L	IFI44L.value <= threshold=2.5893263816833496
node_6113: feature_name=TCL1A	TCL1A.value <= threshold=4.201832294464111
node_6114: feature_name=HLA-DQA2	HLA-DQA2.value <= threshold=-0.2270243540406227
node_6115: feature_name=MTRNR2L2	MTRNR2L2.value <= threshold=2.548917293548584
node_6116: feature_name=ATF5	ATF5.value <= threshold=2.041263461112976
node_6117: feature_name=JUN	JUN.value > threshold=-0.616259902715683
node_6189: feature_name=MT-CO3	MT-CO3.value <= threshold=0.13483521342277527
node_6190: feature_name=XAF1	XAF1.value <= threshold=2.584952235221863
node_6191: feature_name=HLA-DRB5	HLA-DRB5.value > threshold=0.6955901980400085
node_6239: feature_name=TNFAIP3	TNFAIP3.value <= threshold=0.11930759996175766
node_6240: feature_name=TP53INP1	TP53INP1.value <= threshold=2.9654784202575684
node_6241: feature_name=HLA-DRB5	HLA-DRB5.value <= threshold=2.5141202211380005
node_6242: feature_name=NFKBID	NFKBID.value <= threshold=3.358878970146179
node_6243: feature_name=JARID2	JARID2.value <= threshold=1.7160060405731201
node_6244: feature_name=HLA-DPA1	HLA-DPA1.value > threshold=0.49491266906261444
node_6246: feature_name=MIR24-2	MIR24-2.value <= threshold=1.8348549008369446

node_6247: feature_name=HSP90B1	HSP90B1.value <= threshold=1.8861054182052612
node_6248: feature_name=NR4A1	NR4A1.value > threshold=-0.5434737503528595
node_6274: feature_name=CD79A	CD79A.value > threshold=1.2718155980110168
node_6278: feature_name=RP5-821D11.7	RP5-821D11.7.value <= threshold=4.768282890319824
node_6279: feature_name=FAM160B1	FAM160B1.value <= threshold=2.6933162212371826
node_6280: feature_name=ICAM1	ICAM1.value <= threshold=3.578627824783325
node_6281: feature_name=CASP8	CASP8.value <= threshold=1.7487988471984863
node_6282: feature_name=TRAF1	TRAF1.value <= threshold=3.18716561794281
node_6283: feature_name=RNF11	RNF11.value <= threshold=1.9705576300621033
node_6284: feature_name=SNX9	SNX9.value <= threshold=3.318251371383667
node_6285: feature_name=PARP14	PARP14.value <= threshold=2.4342962503433228
node_6286: feature_name=GPX1	GPX1.value <= threshold=1.119508445262909
node_6287: feature_name=IRF8	IRF8.value <= threshold=3.6463273763656616
node_6288: feature_name=TNF	TNF.value > threshold=1.8232123255729675
node_6290: feature_name=RBKS	RBKS.value > threshold=-0.2851060926914215
Class: Healthy Control	
Rules_649	passed counts:6
node_0: feature_name=LTB	LTB.value > threshold=0.994031548500061
node_5264: feature_name=MT-CO1	MT-CO1.value > threshold=-0.4127991199493408
node_5638: feature_name=OAZ1	OAZ1.value > threshold=-0.2640867084264755
node_6112: feature_name=IFI44L	IFI44L.value <= threshold=2.5893263816833496
node_6113: feature_name=TCL1A	TCL1A.value <= threshold=4.201832294464111
node_6114: feature_name=HLA-DQA2	HLA-DQA2.value <= threshold=-0.2270243540406227
node_6115: feature_name=MTRNR2L2	MTRNR2L2.value <= threshold=2.548917293548584
node_6116: feature_name=ATF5	ATF5.value <= threshold=2.041263461112976
node_6117: feature_name=JUN	JUN.value > threshold=-0.616259902715683
node_6189: feature_name=MT-CO3	MT-CO3.value <= threshold=0.13483521342277527
node_6190: feature_name=XAF1	XAF1.value <= threshold=2.584952235221863
node_6191: feature_name=HLA-DRB5	HLA-DRB5.value <= threshold=0.6955901980400085

node_6192: feature_name=NR4A1	NR4A1.value > threshold=-0.4496448189020157
node_6222: feature_name=HSP90B1	HSP90B1.value > threshold=1.1745065450668335
node_6236: feature_name=NR4A1	NR4A1.value <= threshold=2.657681107521057
Class: COVID-19 Severe	
Rules_650	passed counts:6
node_0: feature_name=LTB	LTB.value > threshold=0.994031548500061
node_5264: feature_name=MT-CO1	MT-CO1.value > threshold=-0.4127991199493408
node_5638: feature_name=OAZ1	OAZ1.value > threshold=-0.2640867084264755
node_6112: feature_name=IFI44L	IFI44L.value <= threshold=2.5893263816833496
node_6113: feature_name=TCL1A	TCL1A.value <= threshold=4.201832294464111
node_6114: feature_name=HLA-DQA2	HLA-DQA2.value <= threshold=-0.2270243540406227
node_6115: feature_name=MTRNR2L2	MTRNR2L2.value <= threshold=2.548917293548584
node_6116: feature_name=ATF5	ATF5.value <= threshold=2.041263461112976
node_6117: feature_name=JUN	JUN.value <= threshold=-0.616259902715683
node_6118: feature_name=NFKBID	NFKBID.value > threshold=1.5264258980751038
node_6166: feature_name=NR4A1	NR4A1.value <= threshold=0.6553736329078674
node_6167: feature_name=WDR74	WDR74.value <= threshold=-0.49743133783340454
node_6168: feature_name=APP	APP.value > threshold=0.5775387734174728
node_6176: feature_name=MT-CO3	MT-CO3.value > threshold=-0.19468428194522858
Class: Healthy Control	
Rules_651	passed counts:6
node_0: feature_name=LTB	LTB.value > threshold=0.994031548500061
node_5264: feature_name=MT-CO1	MT-CO1.value > threshold=-0.4127991199493408
node_5638: feature_name=OAZ1	OAZ1.value > threshold=-0.2640867084264755
node_6112: feature_name=IFI44L	IFI44L.value <= threshold=2.5893263816833496
node_6113: feature_name=TCL1A	TCL1A.value <= threshold=4.201832294464111
node_6114: feature_name=HLA-DQA2	HLA-DQA2.value <= threshold=-0.2270243540406227
node_6115: feature_name=MTRNR2L2	MTRNR2L2.value <= threshold=2.548917293548584

node_6116: feature_name=ATF5	ATF5.value <= threshold=2.041263461112976
node_6117: feature_name=JUN	JUN.value <= threshold=-0.616259902715683
node_6118: feature_name=NFKBID	NFKBID.value > threshold=1.5264258980751038
node_6166: feature_name=NR4A1	NR4A1.value <= threshold=0.6553736329078674
node_6167: feature_name=WDR74	WDR74.value <= threshold=-0.49743133783340454
node_6168: feature_name=APP	APP.value <= threshold=0.5775387734174728
node_6169: feature_name=HLA-DQB1	HLA-DQB1.value > threshold=1.6636562943458557
node_6171: feature_name=TCF4	TCF4.value > threshold=-0.27397148311138153
node_6173: feature_name=PDLIM1	PDLIM1.value > threshold=-0.28448981046676636
Class: COVID-19 Severe	
Rules_652	passed counts:6
node_0: feature_name=LTB	LTB.value > threshold=0.994031548500061
node_5264: feature_name=MT-CO1	MT-CO1.value > threshold=-0.4127991199493408
node_5638: feature_name=OAZ1	OAZ1.value > threshold=-0.2640867084264755
node_6112: feature_name=IFI44L	IFI44L.value <= threshold=2.5893263816833496
node_6113: feature_name=TCL1A	TCL1A.value <= threshold=4.201832294464111
node_6114: feature_name=HLA-DQA2	HLA-DQA2.value <= threshold=-0.2270243540406227
node_6115: feature_name=MTRNR2L2	MTRNR2L2.value <= threshold=2.548917293548584
node_6116: feature_name=ATF5	ATF5.value <= threshold=2.041263461112976
node_6117: feature_name=JUN	JUN.value <= threshold=-0.616259902715683
node_6118: feature_name=NFKBID	NFKBID.value <= threshold=1.5264258980751038
node_6119: feature_name=MT-CO1	MT-CO1.value > threshold=0.1564532369375229
node_6133: feature_name=LTB	LTB.value <= threshold=1.2431999444961548
node_6134: feature_name=MT-ND6	MT-ND6.value <= threshold=0.5990295112133026
node_6135: feature_name=MEF2A	MEF2A.value <= threshold=1.0292169749736786
node_6136: feature_name=MX1	MX1.value > threshold=-0.4555272161960602
Class: COVID-19 Critical	
Rules_653	passed counts:6

node_0: feature_name=LTB	LTB.value > threshold=0.994031548500061
node_5264: feature_name=MT-CO1	MT-CO1.value > threshold=-0.4127991199493408
node_5638: feature_name=OAZ1	OAZ1.value > threshold=-0.2640867084264755
node_6112: feature_name=IFI44L	IFI44L.value <= threshold=2.5893263816833496
node_6113: feature_name=TCL1A	TCL1A.value <= threshold=4.201832294464111
node_6114: feature_name=HLA-DQA2	HLA-DQA2.value <= threshold=-0.2270243540406227
node_6115: feature_name=MTRNR2L2	MTRNR2L2.value <= threshold=2.548917293548584
node_6116: feature_name=ATF5	ATF5.value <= threshold=2.041263461112976
node_6117: feature_name=JUN	JUN.value <= threshold=-0.616259902715683
node_6118: feature_name=NFKBID	NFKBID.value <= threshold=1.5264258980751038
node_6119: feature_name=MT-CO1	MT-CO1.value <= threshold=0.1564532369375229
node_6120: feature_name=HLA-DPA1	HLA-DPA1.value > threshold=1.569844663143158
node_6126: feature_name=CASP8	CASP8.value > threshold=0.8988403975963593
Class: COVID-19 Critical	
Rules_654	passed counts:6
node_0: feature_name=LTB	LTB.value > threshold=0.994031548500061
node_5264: feature_name=MT-CO1	MT-CO1.value > threshold=-0.4127991199493408
node_5638: feature_name=OAZ1	OAZ1.value <= threshold=-0.2640867084264755
node_5639: feature_name=CD79B	CD79B.value > threshold=2.2998517751693726
node_5925: feature_name=SNX9	SNX9.value <= threshold=2.4445992708206177
node_5926: feature_name=TCL1A	TCL1A.value <= threshold=2.6938670873641968
node_5927: feature_name=PLD4	PLD4.value > threshold=-0.06434785947203636
node_6009: feature_name=MX1	MX1.value <= threshold=3.600893020629883
node_6010: feature_name=PRDM1	PRDM1.value <= threshold=-0.407244011759758
node_6011: feature_name=SNORD3B-2	SNORD3B-2.value <= threshold=2.7082353830337524
node_6012: feature_name=NFKBID	NFKBID.value <= threshold=3.0716724395751953
node_6013: feature_name=HLA-DQA2	HLA-DQA2.value > threshold=3.7589778900146484
node_6031: feature_name=SNX9	SNX9.value > threshold=-0.5354251265525818
Class: COVID-19 Critical	

Rules_655	passed counts:6
node_0: feature_name=LTB	LTB.value > threshold=0.994031548500061
node_5264: feature_name=MT-CO1	MT-CO1.value > threshold=-0.4127991199493408
node_5638: feature_name=OAZ1	OAZ1.value <= threshold=-0.2640867084264755
node_5639: feature_name=CD79B	CD79B.value > threshold=2.2998517751693726
node_5925: feature_name=SNX9	SNX9.value <= threshold=2.4445992708206177
node_5926: feature_name=TCL1A	TCL1A.value <= threshold=2.6938670873641968
node_5927: feature_name=PLD4	PLD4.value <= threshold=-0.06434785947203636
node_5928: feature_name=ICAM1	ICAM1.value <= threshold=-0.2820792943239212
node_5929: feature_name=MS4A1	MS4A1.value > threshold=2.747320055961609
node_5963: feature_name=RNF144B	RNF144B.value <= threshold=-0.2324080914258957
node_5964: feature_name=HLA-DRB5	HLA-DRB5.value > threshold=2.0430362224578857
node_5982: feature_name=HSP90B1	HSP90B1.value <= threshold=0.5199131667613983
Class: COVID-19 Critical	
Rules_656	passed counts:6
node_0: feature_name=LTB	LTB.value > threshold=0.994031548500061
node_5264: feature_name=MT-CO1	MT-CO1.value > threshold=-0.4127991199493408
node_5638: feature_name=OAZ1	OAZ1.value <= threshold=-0.2640867084264755
node_5639: feature_name=CD79B	CD79B.value > threshold=2.2998517751693726
node_5925: feature_name=SNX9	SNX9.value <= threshold=2.4445992708206177
node_5926: feature_name=TCL1A	TCL1A.value <= threshold=2.6938670873641968
node_5927: feature_name=PLD4	PLD4.value <= threshold=-0.06434785947203636
node_5928: feature_name=ICAM1	ICAM1.value <= threshold=-0.2820792943239212
node_5929: feature_name=MS4A1	MS4A1.value <= threshold=2.747320055961609
node_5930: feature_name=LTB	LTB.value > threshold=1.3533939123153687
node_5954: feature_name=HLA-DPB1	HLA-DPB1.value <= threshold=1.5530678033828735
node_5955: feature_name=PPIF	PPIF.value <= threshold=-0.3640940636396408
Class: COVID-19 Critical	

Rules_657	passed counts:6
node_0: feature_name=LTB	LTB.value > threshold=0.994031548500061
node_5264: feature_name=MT-CO1	MT-CO1.value > threshold=-0.4127991199493408
node_5638: feature_name=OAZ1	OAZ1.value <= threshold=-0.2640867084264755
node_5639: feature_name=CD79B	CD79B.value <= threshold=2.2998517751693726
node_5640: feature_name=MTRNR2L2	MTRNR2L2.value <= threshold=3.5499863624572754
node_5641: feature_name=MT-CO1	MT-CO1.value > threshold=0.22567133605480194
node_5729: feature_name=MARCH1	MARCH1.value > threshold=1.5154643654823303
node_5867: feature_name=NFKBID	NFKBID.value <= threshold=2.6947624683380127
node_5868: feature_name=UTY	UTY.value <= threshold=3.29777729511261
node_5869: feature_name=IFI44L	IFI44L.value <= threshold=-0.29640993475914
node_5870: feature_name=FTH1	FTH1.value <= threshold=0.6966870129108429
node_5871: feature_name=ILK	ILK.value > threshold=1.5719919204711914
node_5891: feature_name=IGJ	IGJ.value <= threshold=-0.2698028236627579
Class: COVID-19 Critical	
Rules_658	passed counts:6
node_0: feature_name=LTB	LTB.value > threshold=0.994031548500061
node_5264: feature_name=MT-CO1	MT-CO1.value > threshold=-0.4127991199493408
node_5638: feature_name=OAZ1	OAZ1.value <= threshold=-0.2640867084264755
node_5639: feature_name=CD79B	CD79B.value <= threshold=2.2998517751693726
node_5640: feature_name=MTRNR2L2	MTRNR2L2.value <= threshold=3.5499863624572754
node_5641: feature_name=MT-CO1	MT-CO1.value > threshold=0.22567133605480194
node_5729: feature_name=MARCH1	MARCH1.value <= threshold=1.5154643654823303
node_5730: feature_name=MT-CO1	MT-CO1.value > threshold=1.7823500633239746
node_5856: feature_name=TTN	TTN.value > threshold=-0.38264039158821106
node_5862: feature_name=BACH1	BACH1.value <= threshold=-0.4962465167045593
Class: Healthy Control	

Rules_659	passed counts:6
node_0: feature_name=LTB	LTB.value > threshold=0.994031548500061
node_5264: feature_name=MT-CO1	MT-CO1.value > threshold=-0.4127991199493408
node_5638: feature_name=OAZ1	OAZ1.value <= threshold=-0.2640867084264755
node_5639: feature_name=CD79B	CD79B.value <= threshold=2.2998517751693726
node_5640: feature_name=MTRNR2L2	MTRNR2L2.value <= threshold=3.5499863624572754
node_5641: feature_name=MT-CO1	MT-CO1.value > threshold=0.22567133605480194
node_5729: feature_name=MARCH1	MARCH1.value <= threshold=1.5154643654823303
node_5730: feature_name=MT-CO1	MT-CO1.value <= threshold=1.7823500633239746
node_5731: feature_name=HLA-DPB1	HLA-DPB1.value <= threshold=2.0096163749694824
node_5732: feature_name=NR4A3	NR4A3.value > threshold=3.509189486503601
node_5822: feature_name=BANK1	BANK1.value <= threshold=2.9566380977630615
Class: COVID-19 Severe	
Rules_660	passed counts:6
node_0: feature_name=LTB	LTB.value > threshold=0.994031548500061
node_5264: feature_name=MT-CO1	MT-CO1.value > threshold=-0.4127991199493408
node_5638: feature_name=OAZ1	OAZ1.value <= threshold=-0.2640867084264755
node_5639: feature_name=CD79B	CD79B.value <= threshold=2.2998517751693726
node_5640: feature_name=MTRNR2L2	MTRNR2L2.value <= threshold=3.5499863624572754
node_5641: feature_name=MT-CO1	MT-CO1.value > threshold=0.22567133605480194
node_5729: feature_name=MARCH1	MARCH1.value <= threshold=1.5154643654823303
node_5730: feature_name=MT-CO1	MT-CO1.value <= threshold=1.7823500633239746
node_5731: feature_name=HLA-DPB1	HLA-DPB1.value <= threshold=2.0096163749694824
node_5732: feature_name=NR4A3	NR4A3.value <= threshold=3.509189486503601
node_5733: feature_name=OAZ1	OAZ1.value > threshold=-0.4392261207103729
node_5803: feature_name=BACH1	BACH1.value <= threshold=1.211863934993744
node_5804: feature_name=MEF2A	MEF2A.value > threshold=0.8741590976715088
node_5808: feature_name=ATF3	ATF3.value <= threshold=-0.38448040187358856
node_5809: feature_name=CYBB	CYBB.value <= threshold=-0.5348981320858002

Class: COVID-19 Critical	
Rules_661	passed counts:6
node_0: feature_name=LTB	LTB.value > threshold=0.994031548500061
node_5264: feature_name=MT-CO1	MT-CO1.value <= threshold=-0.4127991199493408
node_5265: feature_name=MTRNR2L2	MTRNR2L2.value <= threshold=2.4473838806152344
node_5266: feature_name=TCL1A	TCL1A.value > threshold=5.787256956100464
node_5616: feature_name=XAF1	XAF1.value <= threshold=3.0923240184783936
node_5617: feature_name=CD79A	CD79A.value > threshold=2.297448992729187
node_5623: feature_name=MT-CO1	MT-CO1.value <= threshold=-1.1357948780059814
node_5624: feature_name=TTN	TTN.value <= threshold=-0.33193837106227875
node_5625: feature_name=NAMPT	NAMPT.value <= threshold=-0.4733038991689682
Class: COVID-19 Critical	
Rules_662	passed counts:6
node_0: feature_name=LTB	LTB.value > threshold=0.994031548500061
node_5264: feature_name=MT-CO1	MT-CO1.value <= threshold=-0.4127991199493408
node_5265: feature_name=MTRNR2L2	MTRNR2L2.value <= threshold=2.4473838806152344
node_5266: feature_name=TCL1A	TCL1A.value <= threshold=5.787256956100464
node_5267: feature_name=PLD4	PLD4.value <= threshold=3.544657826423645
node_5268: feature_name=NR4A1	NR4A1.value > threshold=0.8490704298019409
node_5498: feature_name=HLA-DPA1	HLA-DPA1.value > threshold=1.9079211950302124
node_5556: feature_name=CD83	CD83.value > threshold=1.6135562062263489
node_5560: feature_name=OAZ1	OAZ1.value > threshold=0.11598343029618263
node_5568: feature_name=MT-CO1	MT-CO1.value <= threshold=-0.8431997299194336
node_5569: feature_name=VCL	VCL.value > threshold=-0.3415001332759857
Class: COVID-19 Severe	
Rules_663	passed counts:6
node_0: feature_name=LTB	LTB.value > threshold=0.994031548500061

node_5264: feature_name=MT-CO1	MT-CO1.value <= threshold=-0.4127991199493408
node_5265: feature_name=MTRNR2L2	MTRNR2L2.value <= threshold=2.4473838806152344
node_5266: feature_name=TCL1A	TCL1A.value <= threshold=5.787256956100464
node_5267: feature_name=PLD4	PLD4.value <= threshold=3.544657826423645
node_5268: feature_name=NR4A1	NR4A1.value > threshold=0.8490704298019409
node_5498: feature_name=HLA-DPA1	HLA-DPA1.value <= threshold=1.9079211950302124
node_5499: feature_name=ID3	ID3.value <= threshold=-0.17505786567926407
node_5500: feature_name=PPIF	PPIF.value <= threshold=-0.5180355906486511
node_5501: feature_name=LTB	LTB.value > threshold=1.0920450091362
node_5503: feature_name=SNX9	SNX9.value <= threshold=-0.5426926016807556
node_5504: feature_name=BACH1	BACH1.value <= threshold=-0.5995040833950043
Class: Healthy Control	
Rules_664	passed counts:6
node_0: feature_name=LTB	LTB.value > threshold=0.994031548500061
node_5264: feature_name=MT-CO1	MT-CO1.value <= threshold=-0.4127991199493408
node_5265: feature_name=MTRNR2L2	MTRNR2L2.value <= threshold=2.4473838806152344
node_5266: feature_name=TCL1A	TCL1A.value <= threshold=5.787256956100464
node_5267: feature_name=PLD4	PLD4.value <= threshold=3.544657826423645
node_5268: feature_name=NR4A1	NR4A1.value <= threshold=0.8490704298019409
node_5269: feature_name=LTB	LTB.value > threshold=1.5252206921577454
node_5471: feature_name=CD79A	CD79A.value <= threshold=2.510846734046936
node_5472: feature_name=CD83	CD83.value <= threshold=-0.5842534303665161
node_5473: feature_name=PELI1	PELI1.value > threshold=0.7717273533344269
Class: COVID-19 Critical	
Rules_665	passed counts:6
node_0: feature_name=LTB	LTB.value > threshold=0.994031548500061
node_5264: feature_name=MT-CO1	MT-CO1.value <= threshold=-0.4127991199493408
node_5265: feature_name=MTRNR2L2	MTRNR2L2.value <= threshold=2.4473838806152344

node_5266: feature_name=TCL1A	TCL1A.value <= threshold=5.787256956100464
node_5267: feature_name=PLD4	PLD4.value <= threshold=3.544657826423645
node_5268: feature_name=NR4A1	NR4A1.value <= threshold=0.8490704298019409
node_5269: feature_name=LTB	LTB.value > threshold=1.5252206921577454
node_5471: feature_name=CD79A	CD79A.value <= threshold=2.510846734046936
node_5472: feature_name=CD83	CD83.value <= threshold=-0.5842534303665161
node_5473: feature_name=PELI1	PELI1.value <= threshold=0.7717273533344269
Class: Healthy Control	
Rules_666	passed counts:6
node_0: feature_name=LTB	LTB.value > threshold=0.994031548500061
node_5264: feature_name=MT-CO1	MT-CO1.value <= threshold=-0.4127991199493408
node_5265: feature_name=MTRNR2L2	MTRNR2L2.value <= threshold=2.4473838806152344
node_5266: feature_name=TCL1A	TCL1A.value <= threshold=5.787256956100464
node_5267: feature_name=PLD4	PLD4.value <= threshold=3.544657826423645
node_5268: feature_name=NR4A1	NR4A1.value <= threshold=0.8490704298019409
node_5269: feature_name=LTB	LTB.value <= threshold=1.5252206921577454
node_5270: feature_name=HLA-DRB5	HLA-DRB5.value <= threshold=2.5117164850234985
node_5271: feature_name=CD79A	CD79A.value <= threshold=3.685201406478882
node_5272: feature_name=BCL2A1	BCL2A1.value <= threshold=2.081526756286621
node_5273: feature_name=HLA-DPA1	HLA-DPA1.value <= threshold=1.8042166829109192
node_5274: feature_name=HSP90B1	HSP90B1.value > threshold=-1.3115270137786865
node_5278: feature_name=OAZ1	OAZ1.value > threshold=1.0802008509635925
node_5350: feature_name=XAF1	XAF1.value <= threshold=-0.4393231123685837
node_5351: feature_name=HLA-DQA1	HLA-DQA1.value > threshold=2.280219793319702
node_5353: feature_name=VPREB3	VPREB3.value <= threshold=4.682476997375488
Class: COVID-19 Critical	
Rules_667	passed counts:6
node_0: feature_name=LTB	LTB.value > threshold=0.994031548500061

node_5264: feature_name=MT-CO1	MT-CO1.value <= threshold=-0.4127991199493408
node_5265: feature_name=MTRNR2L2	MTRNR2L2.value <= threshold=2.4473838806152344
node_5266: feature_name=TCL1A	TCL1A.value <= threshold=5.787256956100464
node_5267: feature_name=PLD4	PLD4.value <= threshold=3.544657826423645
node_5268: feature_name=NR4A1	NR4A1.value <= threshold=0.8490704298019409
node_5269: feature_name=LTB	LTB.value <= threshold=1.5252206921577454
node_5270: feature_name=HLA-DRB5	HLA-DRB5.value <= threshold=2.5117164850234985
node_5271: feature_name=CD79A	CD79A.value <= threshold=3.685201406478882
node_5272: feature_name=BCL2A1	BCL2A1.value <= threshold=2.081526756286621
node_5273: feature_name=HLA-DPA1	HLA-DPA1.value <= threshold=1.8042166829109192
node_5274: feature_name=HSP90B1	HSP90B1.value > threshold=-1.3115270137786865
node_5278: feature_name=OAZ1	OAZ1.value <= threshold=1.0802008509635925
node_5279: feature_name=CD79B	CD79B.value <= threshold=5.875581979751587
node_5280: feature_name=CCDC141	CCDC141.value <= threshold=6.439151048660278
node_5281: feature_name=SYTL3	SYTL3.value <= threshold=1.149337351322174
node_5282: feature_name=GPX1	GPX1.value > threshold=-1.0243326425552368
node_5284: feature_name=MMD	MMD.value <= threshold=6.431635856628418
node_5285: feature_name=MARCKS	MARCKS.value <= threshold=4.034798860549927
node_5286: feature_name=XIST	XIST.value <= threshold=1.148451752960682
node_5287: feature_name=CD83	CD83.value <= threshold=2.894522190093994
node_5288: feature_name=MIR24-2	MIR24-2.value <= threshold=2.76771080493927
node_5289: feature_name=GRASP	GRASP.value > threshold=-0.4039058983325958
node_5303: feature_name=CD38	CD38.value <= threshold=3.721052408218384
node_5304: feature_name=ATF5	ATF5.value <= threshold=6.64192533493042
node_5305: feature_name=CD22	CD22.value <= threshold=5.268923759460449
node_5306: feature_name=CASP8	CASP8.value <= threshold=1.738209843635559
node_5307: feature_name=HSP90B1	HSP90B1.value > threshold=-1.2544044852256775
node_5311: feature_name=HLA-DPB1	HLA-DPB1.value > threshold=2.4081677198410034
node_5317: feature_name=MARCKS	MARCKS.value <= threshold=2.3083163499832153
Class: COVID-19 Critical	

Rules_668	passed counts:6
node_0: feature_name=LTB	LTB.value <= threshold=0.994031548500061
node_1: feature_name=MTRNR2L2	MTRNR2L2.value > threshold=2.8784297704696655
node_5207: feature_name=MTRNR2L8	MTRNR2L8.value <= threshold=3.1915172338485718
node_5208: feature_name=MT-CYB	MT-CYB.value <= threshold=1.3929389119148254
node_5209: feature_name=SLC7A5	SLC7A5.value <= threshold=3.205444812774658
node_5210: feature_name=MT-CO2	MT-CO2.value <= threshold=2.065874218940735
node_5211: feature_name=HLA-DRB5	HLA-DRB5.value <= threshold=2.276706099510193
node_5212: feature_name=TLR2	TLR2.value <= threshold=4.805860757827759
node_5213: feature_name=XIST	XIST.value <= threshold=4.313443303108215
node_5214: feature_name=TTN	TTN.value <= threshold=7.185946702957153
node_5215: feature_name=ITGAX	ITGAX.value <= threshold=3.106598973274231
node_5216: feature_name=PLEC	PLEC.value <= threshold=3.848032832145691
node_5217: feature_name=BCL2A1	BCL2A1.value <= threshold=2.4758780002593994
node_5218: feature_name=ATG2A	ATG2A.value > threshold=3.2491225004196167
node_5224: feature_name=KIAA0125	KIAA0125.value > threshold=-0.1543486788868904
Class: COVID-19 Severe	
Rules_669	passed counts:6
node_0: feature_name=LTB	LTB.value <= threshold=0.994031548500061
node_1: feature_name=MTRNR2L2	MTRNR2L2.value <= threshold=2.8784297704696655
node_2: feature_name=TCL1A	TCL1A.value > threshold=4.798312664031982
node_4912: feature_name=MT-CO1	MT-CO1.value > threshold=-0.3664844036102295
node_5002: feature_name=IFI44L	IFI44L.value <= threshold=2.3101747035980225
node_5003: feature_name=SNX9	SNX9.value > threshold=2.0247421264648438
node_5147: feature_name=CD79A	CD79A.value <= threshold=2.554050326347351
node_5148: feature_name=HLA-DQB1	HLA-DQB1.value > threshold=2.6719621419906616
node_5162: feature_name=GPX1	GPX1.value <= threshold=0.0071204304695129395
node_5163: feature_name=CD79B	CD79B.value > threshold=-0.2608358860015869

node_5165: feature_name=ABCA1	ABCA1.value <= threshold=1.4709488600492477
Class: COVID-19 Severe	
Rules_670	passed counts:6
node_0: feature_name=LTB	LTB.value <= threshold=0.994031548500061
node_1: feature_name=MTRNR2L2	MTRNR2L2.value <= threshold=2.8784297704696655
node_2: feature_name=TCL1A	TCL1A.value > threshold=4.798312664031982
node_4912: feature_name=MT-CO1	MT-CO1.value > threshold=-0.3664844036102295
node_5002: feature_name=IFI44L	IFI44L.value <= threshold=2.3101747035980225
node_5003: feature_name=SNX9	SNX9.value <= threshold=2.0247421264648438
node_5004: feature_name=STAG3	STAG3.value <= threshold=6.482141733169556
node_5005: feature_name=BACH1	BACH1.value > threshold=-0.5237349569797516
node_5047: feature_name=RHOB	RHOB.value <= threshold=-0.4843696355819702
node_5048: feature_name=MT-CO1	MT-CO1.value > threshold=0.6870226263999939
Class: Healthy Control	
Rules_671	passed counts:6
node_0: feature_name=LTB	LTB.value <= threshold=0.994031548500061
node_1: feature_name=MTRNR2L2	MTRNR2L2.value <= threshold=2.8784297704696655
node_2: feature_name=TCL1A	TCL1A.value <= threshold=4.798312664031982
node_3: feature_name=MT-CO1	MT-CO1.value > threshold=0.08947191759943962
node_2521: feature_name=LTB	LTB.value > threshold=0.6038667261600494
node_4173: feature_name=OAZ1	OAZ1.value > threshold=0.143283873796463
node_4615: feature_name=NR4A1	NR4A1.value > threshold=0.6621004343032837
node_4817: feature_name=HSP90B1	HSP90B1.value <= threshold=1.1699737310409546
node_4818: feature_name=XAF1	XAF1.value <= threshold=2.6752071380615234
node_4819: feature_name=SNX9	SNX9.value <= threshold=2.939147114753723
node_4820: feature_name=HLA-DQA2	HLA-DQA2.value > threshold=2.9822239875793457
node_4874: feature_name=MARCH1	MARCH1.value <= threshold=-0.3389042466878891
node_4875: feature_name=ICAM1	ICAM1.value <= threshold=-0.36889341473579407

node_4876: feature_name=CYBB	CYBB.value > threshold=-0.6026962101459503
Class: Healthy Control	
Rules_672	passed counts:6
node_0: feature_name=LTB	LTB.value <= threshold=0.994031548500061
node_1: feature_name=MTRNR2L2	MTRNR2L2.value <= threshold=2.8784297704696655
node_2: feature_name=TCL1A	TCL1A.value <= threshold=4.798312664031982
node_3: feature_name=MT-CO1	MT-CO1.value > threshold=0.08947191759943962
node_2521: feature_name=LTB	LTB.value > threshold=0.6038667261600494
node_4173: feature_name=OAZ1	OAZ1.value > threshold=0.143283873796463
node_4615: feature_name=NR4A1	NR4A1.value <= threshold=0.6621004343032837
node_4616: feature_name=NFKBID	NFKBID.value <= threshold=3.283741593360901
node_4617: feature_name=HLA-DQA1	HLA-DQA1.value <= threshold=2.3435752391815186
node_4618: feature_name=PELI1	PELI1.value > threshold=1.3271203637123108
node_4680: feature_name=BANK1	BANK1.value > threshold=-0.3607018291950226
node_4682: feature_name=RGCC	RGCC.value <= threshold=0.60355344414711
node_4683: feature_name=SNX29P2	SNX29P2.value <= threshold=4.192138910293579
node_4684: feature_name=XIST	XIST.value <= threshold=2.375336170196533
node_4685: feature_name=TCF4	TCF4.value > threshold=2.2667007446289062
node_4697: feature_name=MEF2A	MEF2A.value <= threshold=-0.4261530190706253
node_4698: feature_name=RALGPS2	RALGPS2.value > threshold=2.293534517288208
node_4704: feature_name=HSP90B1	HSP90B1.value <= threshold=1.1924639344215393
Class: Healthy Control	
Rules_673	passed counts:6
node_0: feature_name=LTB	LTB.value <= threshold=0.994031548500061
node_1: feature_name=MTRNR2L2	MTRNR2L2.value <= threshold=2.8784297704696655
node_2: feature_name=TCL1A	TCL1A.value <= threshold=4.798312664031982
node_3: feature_name=MT-CO1	MT-CO1.value > threshold=0.08947191759943962
node_2521: feature_name=LTB	LTB.value > threshold=0.6038667261600494

node_4173: feature_name=OAZ1	OAZ1.value > threshold=0.143283873796463
node_4615: feature_name=NR4A1	NR4A1.value <= threshold=0.6621004343032837
node_4616: feature_name=NFKBID	NFKBID.value <= threshold=3.283741593360901
node_4617: feature_name=HLA-DQA1	HLA-DQA1.value <= threshold=2.3435752391815186
node_4618: feature_name=PELI1	PELI1.value <= threshold=1.3271203637123108
node_4619: feature_name=PLD4	PLD4.value <= threshold=3.3385335206985474
node_4620: feature_name=MEF2A	MEF2A.value <= threshold=1.5926940441131592
node_4621: feature_name=DAPP1	DAPP1.value <= threshold=2.647637367248535
node_4622: feature_name=STAG3	STAG3.value <= threshold=-0.16521219909191132
node_4623: feature_name=OAZ1	OAZ1.value > threshold=0.2042311355471611
node_4625: feature_name=FAM129C	FAM129C.value <= threshold=-0.23615043610334396
node_4626: feature_name=TCL1A	TCL1A.value <= threshold=-0.18263818323612213
node_4627: feature_name=RALGPS2	RALGPS2.value <= threshold=0.4531548172235489
node_4628: feature_name=MT-ND6	MT-ND6.value > threshold=-0.9424236118793488
Class: COVID-19 Critical	
Rules_674	passed counts:6
node_0: feature_name=LTB	LTB.value <= threshold=0.994031548500061
node_1: feature_name=MTRNR2L2	MTRNR2L2.value <= threshold=2.8784297704696655
node_2: feature_name=TCL1A	TCL1A.value <= threshold=4.798312664031982
node_3: feature_name=MT-CO1	MT-CO1.value > threshold=0.08947191759943962
node_2521: feature_name=LTB	LTB.value > threshold=0.6038667261600494
node_4173: feature_name=OAZ1	OAZ1.value <= threshold=0.143283873796463
node_4174: feature_name=PLD4	PLD4.value > threshold=2.921932101249695
node_4558: feature_name=HLA-DQA2	HLA-DQA2.value > threshold=1.5485953092575073
node_4598: feature_name=MTRNR2L8	MTRNR2L8.value <= threshold=2.9248507022857666
node_4599: feature_name=HLA-DRB1	HLA-DRB1.value > threshold=1.4262019395828247
node_4607: feature_name=MT-ND6	MT-ND6.value > threshold=0.19271118938922882
node_4611: feature_name=ICAM1	ICAM1.value > threshold=-0.4038618803024292
Class: Healthy Control	

Rules_675	passed counts:6
node_0: feature_name=LTB	LTB.value <= threshold=0.994031548500061
node_1: feature_name=MTRNR2L2	MTRNR2L2.value <= threshold=2.8784297704696655
node_2: feature_name=TCL1A	TCL1A.value <= threshold=4.798312664031982
node_3: feature_name=MT-CO1	MT-CO1.value > threshold=0.08947191759943962
node_2521: feature_name=LTB	LTB.value > threshold=0.6038667261600494
node_4173: feature_name=OAZ1	OAZ1.value <= threshold=0.143283873796463
node_4174: feature_name=PLD4	PLD4.value > threshold=2.921932101249695
node_4558: feature_name=HLA-DQA2	HLA-DQA2.value > threshold=1.5485953092575073
node_4598: feature_name=MTRNR2L8	MTRNR2L8.value <= threshold=2.9248507022857666
node_4599: feature_name=HLA-DRB1	HLA-DRB1.value > threshold=1.4262019395828247
node_4607: feature_name=MT-ND6	MT-ND6.value <= threshold=0.19271118938922882
node_4608: feature_name=LDLRAD4	LDLRAD4.value <= threshold=0.916767954826355
Class: COVID-19 Severe	
Rules_676	passed counts:6
node_0: feature_name=LTB	LTB.value <= threshold=0.994031548500061
node_1: feature_name=MTRNR2L2	MTRNR2L2.value <= threshold=2.8784297704696655
node_2: feature_name=TCL1A	TCL1A.value <= threshold=4.798312664031982
node_3: feature_name=MT-CO1	MT-CO1.value > threshold=0.08947191759943962
node_2521: feature_name=LTB	LTB.value > threshold=0.6038667261600494
node_4173: feature_name=OAZ1	OAZ1.value <= threshold=0.143283873796463
node_4174: feature_name=PLD4	PLD4.value <= threshold=2.921932101249695
node_4175: feature_name=XIST	XIST.value <= threshold=-0.05067425221204758
node_4176: feature_name=MT-CO1	MT-CO1.value > threshold=1.345594048500061
node_4486: feature_name=RALGPS2	RALGPS2.value > threshold=2.7596691846847534
node_4508: feature_name=SNX9	SNX9.value > threshold=2.5712826251983643
node_4526: feature_name=APP	APP.value > threshold=-0.2580544352531433
node_4534: feature_name=HLA-DQB1	HLA-DQB1.value <= threshold=3.2880821228027344

node_4535: feature_name=BOD1L1	BOD1L1.value <= threshold=-0.47307854890823364
Class: Healthy Control	
Rules_677	passed counts:6
node_0: feature_name=LTB	LTB.value <= threshold=0.994031548500061
node_1: feature_name=MTRNR2L2	MTRNR2L2.value <= threshold=2.8784297704696655
node_2: feature_name=TCL1A	TCL1A.value <= threshold=4.798312664031982
node_3: feature_name=MT-CO1	MT-CO1.value > threshold=0.08947191759943962
node_2521: feature_name=LTB	LTB.value > threshold=0.6038667261600494
node_4173: feature_name=OAZ1	OAZ1.value <= threshold=0.143283873796463
node_4174: feature_name=PLD4	PLD4.value <= threshold=2.921932101249695
node_4175: feature_name=XIST	XIST.value <= threshold=-0.05067425221204758
node_4176: feature_name=MT-CO1	MT-CO1.value > threshold=1.345594048500061
node_4486: feature_name=RALGPS2	RALGPS2.value <= threshold=2.7596691846847534
node_4487: feature_name=IFIT2	IFIT2.value > threshold=-0.1915586143732071
node_4501: feature_name=DAPK1	DAPK1.value > threshold=-0.08581509068608284
Class: COVID-19 Critical	
Rules_678	passed counts:6
node_0: feature_name=LTB	LTB.value <= threshold=0.994031548500061
node_1: feature_name=MTRNR2L2	MTRNR2L2.value <= threshold=2.8784297704696655
node_2: feature_name=TCL1A	TCL1A.value <= threshold=4.798312664031982
node_3: feature_name=MT-CO1	MT-CO1.value > threshold=0.08947191759943962
node_2521: feature_name=LTB	LTB.value > threshold=0.6038667261600494
node_4173: feature_name=OAZ1	OAZ1.value <= threshold=0.143283873796463
node_4174: feature_name=PLD4	PLD4.value <= threshold=2.921932101249695
node_4175: feature_name=XIST	XIST.value <= threshold=-0.05067425221204758
node_4176: feature_name=MT-CO1	MT-CO1.value <= threshold=1.345594048500061
node_4177: feature_name=HLA-DQB1	HLA-DQB1.value > threshold=1.927736520767212
node_4353: feature_name=ITM2C	ITM2C.value <= threshold=-0.12329206243157387

node_4354: feature_name=MX1	MX1.value <= threshold=-0.4356398284435272
node_4355: feature_name=HLA-DQB1	HLA-DQB1.value > threshold=2.8865538835525513
node_4419: feature_name=DAPP1	DAPP1.value > threshold=-0.30035753548145294
node_4425: feature_name=RGS2	RGS2.value <= threshold=0.5943844318389893
Class: COVID-19 Critical	
Rules_679	passed counts:6
node_0: feature_name=LTB	LTB.value <= threshold=0.994031548500061
node_1: feature_name=MTRNR2L2	MTRNR2L2.value <= threshold=2.8784297704696655
node_2: feature_name=TCL1A	TCL1A.value <= threshold=4.798312664031982
node_3: feature_name=MT-CO1	MT-CO1.value > threshold=0.08947191759943962
node_2521: feature_name=LTB	LTB.value > threshold=0.6038667261600494
node_4173: feature_name=OAZ1	OAZ1.value <= threshold=0.143283873796463
node_4174: feature_name=PLD4	PLD4.value <= threshold=2.921932101249695
node_4175: feature_name=XIST	XIST.value <= threshold=-0.05067425221204758
node_4176: feature_name=MT-CO1	MT-CO1.value <= threshold=1.345594048500061
node_4177: feature_name=HLA-DQB1	HLA-DQB1.value > threshold=1.927736520767212
node_4353: feature_name=ITM2C	ITM2C.value <= threshold=-0.12329206243157387
node_4354: feature_name=MX1	MX1.value <= threshold=-0.4356398284435272
node_4355: feature_name=HLA-DQB1	HLA-DQB1.value <= threshold=2.8865538835525513
node_4356: feature_name=SNX9	SNX9.value > threshold=1.002076417207718
node_4384: feature_name=HLA-DRB5	HLA-DRB5.value <= threshold=1.0937854647636414
node_4385: feature_name=IFI6	IFI6.value <= threshold=0.09842458367347717
node_4386: feature_name=FCRL1	FCRL1.value <= threshold=3.549554228782654
node_4387: feature_name=HLA-DPB1	HLA-DPB1.value <= threshold=1.561116874217987
node_4388: feature_name=RP11-685N3.1	RP11-685N3.1.value <= threshold=1.0019370689988136
Class: COVID-19 Critical	
Rules_680	passed counts:6
node_0: feature_name=LTB	LTB.value <= threshold=0.994031548500061

node_1: feature_name=MTRNR2L2	MTRNR2L2.value <= threshold=2.8784297704696655
node_2: feature_name=TCL1A	TCL1A.value <= threshold=4.798312664031982
node_3: feature_name=MT-CO1	MT-CO1.value > threshold=0.08947191759943962
node_2521: feature_name=LTB	LTB.value > threshold=0.6038667261600494
node_4173: feature_name=OAZ1	OAZ1.value <= threshold=0.143283873796463
node_4174: feature_name=PLD4	PLD4.value <= threshold=2.921932101249695
node_4175: feature_name=XIST	XIST.value <= threshold=-0.05067425221204758
node_4176: feature_name=MT-CO1	MT-CO1.value <= threshold=1.345594048500061
node_4177: feature_name=HLA-DQB1	HLA-DQB1.value > threshold=1.927736520767212
node_4353: feature_name=ITM2C	ITM2C.value <= threshold=-0.12329206243157387
node_4354: feature_name=MX1	MX1.value <= threshold=-0.4356398284435272
node_4355: feature_name=HLA-DQB1	HLA-DQB1.value <= threshold=2.8865538835525513
node_4356: feature_name=SNX9	SNX9.value <= threshold=1.002076417207718
node_4357: feature_name=FAM160B1	FAM160B1.value > threshold=-0.42920351028442383
node_4361: feature_name=SNX9	SNX9.value <= threshold=0.011789441108703613
node_4362: feature_name=MT-CO3	MT-CO3.value <= threshold=0.4059640020132065
node_4363: feature_name=RNF11	RNF11.value > threshold=-0.5651727318763733
node_4365: feature_name=IGJ	IGJ.value > threshold=-0.3220001757144928
node_4373: feature_name=SLC7A5	SLC7A5.value <= threshold=-0.5009843707084656
Class: Healthy Control	
Rules_681	passed counts:6
node_0: feature_name=LTB	LTB.value <= threshold=0.994031548500061
node_1: feature_name=MTRNR2L2	MTRNR2L2.value <= threshold=2.8784297704696655
node_2: feature_name=TCL1A	TCL1A.value <= threshold=4.798312664031982
node_3: feature_name=MT-CO1	MT-CO1.value > threshold=0.08947191759943962
node_2521: feature_name=LTB	LTB.value > threshold=0.6038667261600494
node_4173: feature_name=OAZ1	OAZ1.value <= threshold=0.143283873796463
node_4174: feature_name=PLD4	PLD4.value <= threshold=2.921932101249695
node_4175: feature_name=XIST	XIST.value <= threshold=-0.05067425221204758

node_4176: feature_name=MT-CO1	MT-CO1.value <= threshold=1.345594048500061
node_4177: feature_name=HLA-DQB1	HLA-DQB1.value <= threshold=1.927736520767212
node_4178: feature_name=BACH1	BACH1.value <= threshold=-0.5349778532981873
node_4179: feature_name=CD79B	CD79B.value > threshold=1.6518195271492004
node_4201: feature_name=MT-CO2	MT-CO2.value <= threshold=0.5007440596818924
node_4202: feature_name=PELI1	PELI1.value > threshold=0.9399837851524353
node_4212: feature_name=TTN	TTN.value <= threshold=-0.3203035891056061
Class: Healthy Control	
Rules_682	passed counts:6
node_0: feature_name=LTB	LTB.value <= threshold=0.994031548500061
node_1: feature_name=MTRNR2L2	MTRNR2L2.value <= threshold=2.8784297704696655
node_2: feature_name=TCL1A	TCL1A.value <= threshold=4.798312664031982
node_3: feature_name=MT-CO1	MT-CO1.value > threshold=0.08947191759943962
node_2521: feature_name=LTB	LTB.value <= threshold=0.6038667261600494
node_2522: feature_name=HLA-DQB1	HLA-DQB1.value > threshold=2.1053054332733154
node_3550: feature_name=XIST	XIST.value > threshold=0.7716337237507105
node_4140: feature_name=HLA-DQA2	HLA-DQA2.value <= threshold=-0.1354314684867859
node_4141: feature_name=MS4A1	MS4A1.value > threshold=3.907364010810852
node_4153: feature_name=HLA-DQB1	HLA-DQB1.value > threshold=2.3341654539108276
Class: Healthy Control	
Rules_683	passed counts:6
node_0: feature_name=LTB	LTB.value <= threshold=0.994031548500061
node_1: feature_name=MTRNR2L2	MTRNR2L2.value <= threshold=2.8784297704696655
node_2: feature_name=TCL1A	TCL1A.value <= threshold=4.798312664031982
node_3: feature_name=MT-CO1	MT-CO1.value > threshold=0.08947191759943962
node_2521: feature_name=LTB	LTB.value <= threshold=0.6038667261600494
node_2522: feature_name=HLA-DQB1	HLA-DQB1.value > threshold=2.1053054332733154
node_3550: feature_name=XIST	XIST.value <= threshold=0.7716337237507105

node_3551: feature_name=CD79B	CD79B.value <= threshold=2.9426796436309814
node_3552: feature_name=HLA-DQB1	HLA-DQB1.value > threshold=2.713674306869507
node_3854: feature_name=MEF2C	MEF2C.value > threshold=-0.3721291869878769
node_3882: feature_name=IFI44L	IFI44L.value <= threshold=2.5516022443771362
node_3883: feature_name=FCER2	FCER2.value <= threshold=-0.21487552672624588
node_3884: feature_name=HLA-DQA2	HLA-DQA2.value <= threshold=-0.12580305337905884
node_3885: feature_name=SLC7A5	SLC7A5.value <= threshold=-0.4906871169805527
node_3886: feature_name=VPREB3	VPREB3.value <= threshold=5.492124557495117
node_3887: feature_name=CYBB	CYBB.value <= threshold=2.720046043395996
node_3888: feature_name=CD79A	CD79A.value <= threshold=3.3531476259231567
node_3889: feature_name=HLA-DPA1	HLA-DPA1.value > threshold=1.6232598423957825
node_3911: feature_name=HLA-DQA1	HLA-DQA1.value > threshold=3.2045756578445435
node_3923: feature_name=NAMPT	NAMPT.value <= threshold=-0.45362021028995514
Class: COVID-19 Critical	
Rules_684	passed counts:6
node_0: feature_name=LTB	LTB.value <= threshold=0.994031548500061
node_1: feature_name=MTRNR2L2	MTRNR2L2.value <= threshold=2.8784297704696655
node_2: feature_name=TCL1A	TCL1A.value <= threshold=4.798312664031982
node_3: feature_name=MT-CO1	MT-CO1.value > threshold=0.08947191759943962
node_2521: feature_name=LTB	LTB.value <= threshold=0.6038667261600494
node_2522: feature_name=HLA-DQB1	HLA-DQB1.value > threshold=2.1053054332733154
node_3550: feature_name=XIST	XIST.value <= threshold=0.7716337237507105
node_3551: feature_name=CD79B	CD79B.value <= threshold=2.9426796436309814
node_3552: feature_name=HLA-DQB1	HLA-DQB1.value > threshold=2.713674306869507
node_3854: feature_name=MEF2C	MEF2C.value > threshold=-0.3721291869878769
node_3882: feature_name=IFI44L	IFI44L.value <= threshold=2.5516022443771362
node_3883: feature_name=FCER2	FCER2.value <= threshold=-0.21487552672624588
node_3884: feature_name=HLA-DQA2	HLA-DQA2.value <= threshold=-0.12580305337905884
node_3885: feature_name=SLC7A5	SLC7A5.value <= threshold=-0.4906871169805527

node_3886: feature_name=VPREB3	VPREB3.value <= threshold=5.492124557495117
node_3887: feature_name=CYBB	CYBB.value <= threshold=2.720046043395996
node_3888: feature_name=CD79A	CD79A.value <= threshold=3.3531476259231567
node_3889: feature_name=HLA-DPA1	HLA-DPA1.value <= threshold=1.6232598423957825
node_3890: feature_name=MT-CYB	MT-CYB.value > threshold=1.3801624178886414
node_3906: feature_name=BANK1	BANK1.value <= threshold=3.6963043212890625
node_3907: feature_name=SYTL3	SYTL3.value <= threshold=-0.657780647277832
Class: COVID-19 Severe	
Rules_685	passed counts:6
node_0: feature_name=LTB	LTB.value <= threshold=0.994031548500061
node_1: feature_name=MTRNR2L2	MTRNR2L2.value <= threshold=2.8784297704696655
node_2: feature_name=TCL1A	TCL1A.value <= threshold=4.798312664031982
node_3: feature_name=MT-CO1	MT-CO1.value > threshold=0.08947191759943962
node_2521: feature_name=LTB	LTB.value <= threshold=0.6038667261600494
node_2522: feature_name=HLA-DQB1	HLA-DQB1.value > threshold=2.1053054332733154
node_3550: feature_name=XIST	XIST.value <= threshold=0.7716337237507105
node_3551: feature_name=CD79B	CD79B.value <= threshold=2.9426796436309814
node_3552: feature_name=HLA-DQB1	HLA-DQB1.value > threshold=2.713674306869507
node_3854: feature_name=MEF2C	MEF2C.value <= threshold=-0.3721291869878769
node_3855: feature_name=HLA-DRB5	HLA-DRB5.value > threshold=0.7929701805114746
node_3859: feature_name=LTB	LTB.value > threshold=-0.9689712226390839
node_3863: feature_name=RP11-731F5.2	RP11-731F5.2.value <= threshold=3.578703761100769
node_3864: feature_name=COTL1	COTL1.value > threshold=0.38136887550354004
Class: COVID-19 Critical	
Rules_686	passed counts:6
node_0: feature_name=LTB	LTB.value <= threshold=0.994031548500061
node_1: feature_name=MTRNR2L2	MTRNR2L2.value <= threshold=2.8784297704696655
node_2: feature_name=TCL1A	TCL1A.value <= threshold=4.798312664031982

node_3: feature_name=MT-CO1	MT-CO1.value > threshold=0.08947191759943962
node_2521: feature_name=LTB	LTB.value <= threshold=0.6038667261600494
node_2522: feature_name=HLA-DQB1	HLA-DQB1.value > threshold=2.1053054332733154
node_3550: feature_name=XIST	XIST.value <= threshold=0.7716337237507105
node_3551: feature_name=CD79B	CD79B.value <= threshold=2.9426796436309814
node_3552: feature_name=HLA-DQB1	HLA-DQB1.value <= threshold=2.713674306869507
node_3553: feature_name=SNX9	SNX9.value > threshold=-0.5404067635536194
node_3653: feature_name=LTB	LTB.value > threshold=0.021708179265260696
node_3759: feature_name=TCL1A	TCL1A.value <= threshold=3.3921085596084595
node_3760: feature_name=HLA-DRB5	HLA-DRB5.value > threshold=0.5265218019485474
node_3776: feature_name=ID1	ID1.value <= threshold=-0.21235637366771698
node_3777: feature_name=RP11-731F5.2	RP11-731F5.2.value <= threshold=2.830894112586975
node_3778: feature_name=CASP8	CASP8.value <= threshold=2.222941279411316
node_3779: feature_name=MT-CO3	MT-CO3.value > threshold=-0.41117167472839355
node_3785: feature_name=HLA-DRB5	HLA-DRB5.value <= threshold=0.8465962707996368
node_3786: feature_name=NFKBID	NFKBID.value <= threshold=1.7803236246109009
node_3787: feature_name=IRF8	IRF8.value <= threshold=2.439036250114441
Class: COVID-19 Severe	
Rules_687	passed counts:6
node_0: feature_name=LTB	LTB.value <= threshold=0.994031548500061
node_1: feature_name=MTRNR2L2	MTRNR2L2.value <= threshold=2.8784297704696655
node_2: feature_name=TCL1A	TCL1A.value <= threshold=4.798312664031982
node_3: feature_name=MT-CO1	MT-CO1.value > threshold=0.08947191759943962
node_2521: feature_name=LTB	LTB.value <= threshold=0.6038667261600494
node_2522: feature_name=HLA-DQB1	HLA-DQB1.value > threshold=2.1053054332733154
node_3550: feature_name=XIST	XIST.value <= threshold=0.7716337237507105
node_3551: feature_name=CD79B	CD79B.value <= threshold=2.9426796436309814
node_3552: feature_name=HLA-DQB1	HLA-DQB1.value <= threshold=2.713674306869507
node_3553: feature_name=SNX9	SNX9.value > threshold=-0.5404067635536194

node_3653: feature_name=LTB	LTB.value <= threshold=0.021708179265260696
node_3654: feature_name=HLA-DRB5	HLA-DRB5.value > threshold=-0.5633223652839661
node_3660: feature_name=NR4A1	NR4A1.value <= threshold=2.2327845096588135
node_3661: feature_name=FCRL1	FCRL1.value <= threshold=8.662389755249023
node_3662: feature_name=BANK1	BANK1.value <= threshold=4.776419401168823
node_3663: feature_name=IGLL5	IGLL5.value > threshold=1.4806077480316162
node_3705: feature_name=NR4A1	NR4A1.value > threshold=1.2165279388427734
node_3721: feature_name=FCRL1	FCRL1.value > threshold=-0.18543093651533127
node_3723: feature_name=FAM129C	FAM129C.value <= threshold=3.0054107904434204
Class: Healthy Control	
Rules_688	passed counts:6
node_0: feature_name=LTB	LTB.value <= threshold=0.994031548500061
node_1: feature_name=MTRNR2L2	MTRNR2L2.value <= threshold=2.8784297704696655
node_2: feature_name=TCL1A	TCL1A.value <= threshold=4.798312664031982
node_3: feature_name=MT-CO1	MT-CO1.value > threshold=0.08947191759943962
node_2521: feature_name=LTB	LTB.value <= threshold=0.6038667261600494
node_2522: feature_name=HLA-DQB1	HLA-DQB1.value > threshold=2.1053054332733154
node_3550: feature_name=XIST	XIST.value <= threshold=0.7716337237507105
node_3551: feature_name=CD79B	CD79B.value <= threshold=2.9426796436309814
node_3552: feature_name=HLA-DQB1	HLA-DQB1.value <= threshold=2.713674306869507
node_3553: feature_name=SNX9	SNX9.value <= threshold=-0.5404067635536194
node_3554: feature_name=VPREB3	VPREB3.value <= threshold=4.4475414752960205
node_3555: feature_name=RALGPS2	RALGPS2.value > threshold=2.7017585039138794
node_3609: feature_name=MX1	MX1.value <= threshold=1.5204657316207886
node_3610: feature_name=FAM129C	FAM129C.value > threshold=-0.30572274327278137
node_3622: feature_name=CASP8	CASP8.value > threshold=-0.5367972254753113
node_3638: feature_name=SLC11A1	SLC11A1.value > threshold=-0.3386744409799576
node_3640: feature_name=MTRNR2L8	MTRNR2L8.value > threshold=-0.38738344609737396
Class: Healthy Control	

Rules_689	passed counts:6
node_0: feature_name=LTB	LTB.value <= threshold=0.994031548500061
node_1: feature_name=MTRNR2L2	MTRNR2L2.value <= threshold=2.8784297704696655
node_2: feature_name=TCL1A	TCL1A.value <= threshold=4.798312664031982
node_3: feature_name=MT-CO1	MT-CO1.value > threshold=0.08947191759943962
node_2521: feature_name=LTB	LTB.value <= threshold=0.6038667261600494
node_2522: feature_name=HLA-DQB1	HLA-DQB1.value <= threshold=2.1053054332733154
node_2523: feature_name=SNX9	SNX9.value > threshold=-0.5346486568450928
node_2907: feature_name=NR4A1	NR4A1.value > threshold=2.5433837175369263
node_3459: feature_name=VPREB3	VPREB3.value <= threshold=4.410779237747192
node_3460: feature_name=RALGPS2	RALGPS2.value > threshold=3.064661979675293
node_3520: feature_name=RGS2	RGS2.value > threshold=-0.5165578126907349
node_3522: feature_name=MT-ND6	MT-ND6.value <= threshold=1.692318856716156
node_3523: feature_name=ID1	ID1.value > threshold=-0.16399746388196945
node_3539: feature_name=NFKBID	NFKBID.value > threshold=-0.43074318766593933
Class: COVID-19 Severe	
Rules_690	passed counts:6
node_0: feature_name=LTB	LTB.value <= threshold=0.994031548500061
node_1: feature_name=MTRNR2L2	MTRNR2L2.value <= threshold=2.8784297704696655
node_2: feature_name=TCL1A	TCL1A.value <= threshold=4.798312664031982
node_3: feature_name=MT-CO1	MT-CO1.value > threshold=0.08947191759943962
node_2521: feature_name=LTB	LTB.value <= threshold=0.6038667261600494
node_2522: feature_name=HLA-DQB1	HLA-DQB1.value <= threshold=2.1053054332733154
node_2523: feature_name=SNX9	SNX9.value > threshold=-0.5346486568450928
node_2907: feature_name=NR4A1	NR4A1.value > threshold=2.5433837175369263
node_3459: feature_name=VPREB3	VPREB3.value <= threshold=4.410779237747192
node_3460: feature_name=RALGPS2	RALGPS2.value <= threshold=3.064661979675293
node_3461: feature_name=MT-CO1	MT-CO1.value > threshold=1.7489010095596313

node_3517: feature_name=RNF11	RNF11.value <= threshold=1.4480273127555847
Class: Healthy Control	
Rules_691	passed counts:6
node_0: feature_name=LTB	LTB.value <= threshold=0.994031548500061
node_1: feature_name=MTRNR2L2	MTRNR2L2.value <= threshold=2.8784297704696655
node_2: feature_name=TCL1A	TCL1A.value <= threshold=4.798312664031982
node_3: feature_name=MT-CO1	MT-CO1.value > threshold=0.08947191759943962
node_2521: feature_name=LTB	LTB.value <= threshold=0.6038667261600494
node_2522: feature_name=HLA-DQB1	HLA-DQB1.value <= threshold=2.1053054332733154
node_2523: feature_name=SNX9	SNX9.value > threshold=-0.5346486568450928
node_2907: feature_name=NR4A1	NR4A1.value <= threshold=2.5433837175369263
node_2908: feature_name=XIST	XIST.value <= threshold=0.7664470989257097
node_2909: feature_name=CD79B	CD79B.value > threshold=2.9326847791671753
node_3353: feature_name=VPREB3	VPREB3.value > threshold=3.0226117372512817
node_3425: feature_name=OAZ1	OAZ1.value <= threshold=0.47689591348171234
node_3426: feature_name=HLA-DQA1	HLA-DQA1.value > threshold=2.271668791770935
node_3434: feature_name=YWHAH	YWHAH.value > threshold=-0.3741019368171692
node_3436: feature_name=TLN1	TLN1.value <= threshold=0.922350101172924
Class: COVID-19 Critical	
Rules_692	passed counts:6
node_0: feature_name=LTB	LTB.value <= threshold=0.994031548500061
node_1: feature_name=MTRNR2L2	MTRNR2L2.value <= threshold=2.8784297704696655
node_2: feature_name=TCL1A	TCL1A.value <= threshold=4.798312664031982
node_3: feature_name=MT-CO1	MT-CO1.value > threshold=0.08947191759943962
node_2521: feature_name=LTB	LTB.value <= threshold=0.6038667261600494
node_2522: feature_name=HLA-DQB1	HLA-DQB1.value <= threshold=2.1053054332733154
node_2523: feature_name=SNX9	SNX9.value > threshold=-0.5346486568450928
node_2907: feature_name=NR4A1	NR4A1.value <= threshold=2.5433837175369263

node_2908: feature_name=XIST	XIST.value <= threshold=0.7664470989257097
node_2909: feature_name=CD79B	CD79B.value <= threshold=2.9326847791671753
node_2910: feature_name=NR4A1	NR4A1.value <= threshold=-0.2875557690858841
node_2911: feature_name=CTD-2035E11.3	CTD-2035E11.3.value <= threshold=5.262048006057739
node_2912: feature_name=VPREB3	VPREB3.value <= threshold=4.937594890594482
node_2913: feature_name=IRAK3	IRAK3.value <= threshold=4.592883586883545
node_2914: feature_name=IGJ	IGJ.value <= threshold=7.093410015106201
node_2915: feature_name=WDR74	WDR74.value <= threshold=4.077916383743286
node_2916: feature_name=MEF2C	MEF2C.value <= threshold=4.374382972717285
node_2917: feature_name=HIST1H2AC	HIST1H2AC.value <= threshold=3.743621349334717
node_2918: feature_name=STMN1	STMN1.value <= threshold=6.266510248184204
node_2919: feature_name=RALGPS2	RALGPS2.value <= threshold=5.72194766998291
node_2920: feature_name=HOPX	HOPX.value <= threshold=2.5944807529449463
node_2921: feature_name=MARCKS	MARCKS.value <= threshold=4.622651815414429
node_2922: feature_name=CCL3	CCL3.value <= threshold=0.7484713792800903
node_2923: feature_name=TPM1	TPM1.value <= threshold=6.446193695068359
node_2924: feature_name=PLD4	PLD4.value <= threshold=8.038907289505005
node_2925: feature_name=COTL1	COTL1.value > threshold=-1.8123814463615417
node_2929: feature_name=ID3	ID3.value <= threshold=7.534881353378296
node_2930: feature_name=IRF8	IRF8.value > threshold=4.855781078338623
node_3020: feature_name=IRF8	IRF8.value > threshold=5.055942058563232
Class: COVID-19 Critical	
Rules_693	passed counts:6
node_0: feature_name=LTB	LTB.value <= threshold=0.994031548500061
node_1: feature_name=MTRNR2L2	MTRNR2L2.value <= threshold=2.8784297704696655
node_2: feature_name=TCL1A	TCL1A.value <= threshold=4.798312664031982
node_3: feature_name=MT-CO1	MT-CO1.value > threshold=0.08947191759943962
node_2521: feature_name=LTB	LTB.value <= threshold=0.6038667261600494
node_2522: feature_name=HLA-DQB1	HLA-DQB1.value <= threshold=2.1053054332733154

node_2523: feature_name=SNX9	SNX9.value <= threshold=-0.5346486568450928
node_2524: feature_name=HLA-DPB1	HLA-DPB1.value > threshold=1.9548270106315613
node_2814: feature_name=CD79B	CD79B.value > threshold=-0.28186772763729095
node_2826: feature_name=MT-ND6	MT-ND6.value > threshold=0.17273088544607162
node_2872: feature_name=IFI44L	IFI44L.value <= threshold=-0.31519079208374023
node_2873: feature_name=RALGPS2	RALGPS2.value > threshold=1.6798744797706604
node_2887: feature_name=HLA-DQA2	HLA-DQA2.value <= threshold=5.12495756149292
node_2888: feature_name=MS4A1	MS4A1.value > threshold=3.8452248573303223
node_2900: feature_name=NR4A1	NR4A1.value > threshold=-0.24637621641159058
Class: Healthy Control	
Rules_694	passed counts:6
node_0: feature_name=LTB	LTB.value <= threshold=0.994031548500061
node_1: feature_name=MTRNR2L2	MTRNR2L2.value <= threshold=2.8784297704696655
node_2: feature_name=TCL1A	TCL1A.value <= threshold=4.798312664031982
node_3: feature_name=MT-CO1	MT-CO1.value > threshold=0.08947191759943962
node_2521: feature_name=LTB	LTB.value <= threshold=0.6038667261600494
node_2522: feature_name=HLA-DQB1	HLA-DQB1.value <= threshold=2.1053054332733154
node_2523: feature_name=SNX9	SNX9.value <= threshold=-0.5346486568450928
node_2524: feature_name=HLA-DPB1	HLA-DPB1.value <= threshold=1.9548270106315613
node_2525: feature_name=FAM129C	FAM129C.value > threshold=3.3393388986587524
node_2721: feature_name=ADAM19	ADAM19.value > threshold=-0.29525794088840485
node_2781: feature_name=XAF1	XAF1.value <= threshold=-0.4296249598264694
node_2782: feature_name=CYBB	CYBB.value <= threshold=-0.5005911737680435
node_2783: feature_name=HLA-DQA1	HLA-DQA1.value <= threshold=2.0089213848114014
Class: COVID-19 Critical	
Rules_695	passed counts:6
node_0: feature_name=LTB	LTB.value <= threshold=0.994031548500061
node_1: feature_name=MTRNR2L2	MTRNR2L2.value <= threshold=2.8784297704696655

node_2: feature_name=TCL1A	TCL1A.value <= threshold=4.798312664031982
node_3: feature_name=MT-CO1	MT-CO1.value > threshold=0.08947191759943962
node_2521: feature_name=LTB	LTB.value <= threshold=0.6038667261600494
node_2522: feature_name=HLA-DQB1	HLA-DQB1.value <= threshold=2.1053054332733154
node_2523: feature_name=SNX9	SNX9.value <= threshold=-0.5346486568450928
node_2524: feature_name=HLA-DPB1	HLA-DPB1.value <= threshold=1.9548270106315613
node_2525: feature_name=FAM129C	FAM129C.value > threshold=3.3393388986587524
node_2721: feature_name=ADAM19	ADAM19.value <= threshold=-0.29525794088840485
node_2722: feature_name=NR4A1	NR4A1.value <= threshold=2.1566513776779175
node_2723: feature_name=CD22	CD22.value <= threshold=8.345317840576172
node_2724: feature_name=PLD4	PLD4.value <= threshold=7.507996082305908
node_2725: feature_name=RALGPS2	RALGPS2.value <= threshold=4.3808369636535645
node_2726: feature_name=VPREB3	VPREB3.value <= threshold=4.1944358348846436
node_2727: feature_name=LDLRAD4	LDLRAD4.value > threshold=3.293746590614319
node_2741: feature_name=NFKBID	NFKBID.value > threshold=1.385556623339653
Class: COVID-19 Critical	
Rules_696	passed counts:6
node_0: feature_name=LTB	LTB.value <= threshold=0.994031548500061
node_1: feature_name=MTRNR2L2	MTRNR2L2.value <= threshold=2.8784297704696655
node_2: feature_name=TCL1A	TCL1A.value <= threshold=4.798312664031982
node_3: feature_name=MT-CO1	MT-CO1.value > threshold=0.08947191759943962
node_2521: feature_name=LTB	LTB.value <= threshold=0.6038667261600494
node_2522: feature_name=HLA-DQB1	HLA-DQB1.value <= threshold=2.1053054332733154
node_2523: feature_name=SNX9	SNX9.value <= threshold=-0.5346486568450928
node_2524: feature_name=HLA-DPB1	HLA-DPB1.value <= threshold=1.9548270106315613
node_2525: feature_name=FAM129C	FAM129C.value <= threshold=3.3393388986587524
node_2526: feature_name=XIST	XIST.value > threshold=3.6911416053771973
node_2718: feature_name=MEF2C	MEF2C.value <= threshold=4.978461980819702
Class: Healthy Control	

Rules_697	passed counts:6
node_0: feature_name=LTB	LTB.value <= threshold=0.994031548500061
node_1: feature_name=MTRNR2L2	MTRNR2L2.value <= threshold=2.8784297704696655
node_2: feature_name=TCL1A	TCL1A.value <= threshold=4.798312664031982
node_3: feature_name=MT-CO1	MT-CO1.value <= threshold=0.08947191759943962
node_4: feature_name=NR4A1	NR4A1.value > threshold=1.0725348591804504
node_1622: feature_name=HLA-DQA2	HLA-DQA2.value > threshold=1.9496816992759705
node_2350: feature_name=BCL2A1	BCL2A1.value <= threshold=1.1322240233421326
node_2351: feature_name=CD83	CD83.value > threshold=2.3162450790405273
node_2391: feature_name=CD79B	CD79B.value > threshold=1.7111427783966064
node_2443: feature_name=MT-CO1	MT-CO1.value > threshold=-1.1614157557487488
node_2447: feature_name=JUN	JUN.value <= threshold=0.19632036238908768
node_2448: feature_name=PLD4	PLD4.value > threshold=-0.11274048686027527
node_2454: feature_name=TRIB1	TRIB1.value > threshold=-0.36109432578086853
Class: Healthy Control	
Rules_698	passed counts:6
node_0: feature_name=LTB	LTB.value <= threshold=0.994031548500061
node_1: feature_name=MTRNR2L2	MTRNR2L2.value <= threshold=2.8784297704696655
node_2: feature_name=TCL1A	TCL1A.value <= threshold=4.798312664031982
node_3: feature_name=MT-CO1	MT-CO1.value <= threshold=0.08947191759943962
node_4: feature_name=NR4A1	NR4A1.value > threshold=1.0725348591804504
node_1622: feature_name=HLA-DQA2	HLA-DQA2.value > threshold=1.9496816992759705
node_2350: feature_name=BCL2A1	BCL2A1.value <= threshold=1.1322240233421326
node_2351: feature_name=CD83	CD83.value > threshold=2.3162450790405273
node_2391: feature_name=CD79B	CD79B.value > threshold=1.7111427783966064
node_2443: feature_name=MT-CO1	MT-CO1.value <= threshold=-1.1614157557487488
node_2444: feature_name=RNF144B	RNF144B.value > threshold=-0.27276481688022614
Class: COVID-19 Severe	

Rules_699	passed counts:6
node_0: feature_name=LTB	LTB.value <= threshold=0.994031548500061
node_1: feature_name=MTRNR2L2	MTRNR2L2.value <= threshold=2.8784297704696655
node_2: feature_name=TCL1A	TCL1A.value <= threshold=4.798312664031982
node_3: feature_name=MT-CO1	MT-CO1.value <= threshold=0.08947191759943962
node_4: feature_name=NR4A1	NR4A1.value > threshold=1.0725348591804504
node_1622: feature_name=HLA-DQA2	HLA-DQA2.value > threshold=1.9496816992759705
node_2350: feature_name=BCL2A1	BCL2A1.value <= threshold=1.1322240233421326
node_2351: feature_name=CD83	CD83.value > threshold=2.3162450790405273
node_2391: feature_name=CD79B	CD79B.value <= threshold=1.7111427783966064
node_2392: feature_name=TTN	TTN.value > threshold=-0.28553803265094757
node_2430: feature_name=LTB	LTB.value <= threshold=0.3538805693387985
node_2431: feature_name=MT-CO3	MT-CO3.value <= threshold=-0.8342754244804382
Class: COVID-19 Severe	
Rules_700	passed counts:6
node_0: feature_name=LTB	LTB.value <= threshold=0.994031548500061
node_1: feature_name=MTRNR2L2	MTRNR2L2.value <= threshold=2.8784297704696655
node_2: feature_name=TCL1A	TCL1A.value <= threshold=4.798312664031982
node_3: feature_name=MT-CO1	MT-CO1.value <= threshold=0.08947191759943962
node_4: feature_name=NR4A1	NR4A1.value > threshold=1.0725348591804504
node_1622: feature_name=HLA-DQA2	HLA-DQA2.value > threshold=1.9496816992759705
node_2350: feature_name=BCL2A1	BCL2A1.value <= threshold=1.1322240233421326
node_2351: feature_name=CD83	CD83.value > threshold=2.3162450790405273
node_2391: feature_name=CD79B	CD79B.value <= threshold=1.7111427783966064
node_2392: feature_name=TTN	TTN.value <= threshold=-0.28553803265094757
node_2393: feature_name=LDLRAD4	LDLRAD4.value <= threshold=3.3328956365585327
node_2394: feature_name=BCL2A1	BCL2A1.value > threshold=-0.5811292827129364
node_2404: feature_name=SLC7A5	SLC7A5.value <= threshold=1.3126214146614075

node_2405: feature_name=AP3M2	AP3M2.value <= threshold=1.617304801940918
node_2406: feature_name=MT-ND6	MT-ND6.value > threshold=0.7549090385437012
node_2416: feature_name=NAMPT	NAMPT.value <= threshold=-0.5255279242992401
node_2417: feature_name=WDR74	WDR74.value > threshold=-0.49697351455688477
Class: COVID-19 Critical	
Rules_701	passed counts:6
node_0: feature_name=LTB	LTB.value <= threshold=0.994031548500061
node_1: feature_name=MTRNR2L2	MTRNR2L2.value <= threshold=2.8784297704696655
node_2: feature_name=TCL1A	TCL1A.value <= threshold=4.798312664031982
node_3: feature_name=MT-CO1	MT-CO1.value <= threshold=0.08947191759943962
node_4: feature_name=NR4A1	NR4A1.value > threshold=1.0725348591804504
node_1622: feature_name=HLA-DQA2	HLA-DQA2.value > threshold=1.9496816992759705
node_2350: feature_name=BCL2A1	BCL2A1.value <= threshold=1.1322240233421326
node_2351: feature_name=CD83	CD83.value > threshold=2.3162450790405273
node_2391: feature_name=CD79B	CD79B.value <= threshold=1.7111427783966064
node_2392: feature_name=TTN	TTN.value <= threshold=-0.28553803265094757
node_2393: feature_name=LDLRAD4	LDLRAD4.value <= threshold=3.3328956365585327
node_2394: feature_name=BCL2A1	BCL2A1.value <= threshold=-0.5811292827129364
node_2395: feature_name=HLA-DQB1	HLA-DQB1.value <= threshold=1.635683536529541
node_2396: feature_name=S100A6	S100A6.value <= threshold=-0.984266608953476
Class: COVID-19 Severe	
Rules_702	passed counts:6
node_0: feature_name=LTB	LTB.value <= threshold=0.994031548500061
node_1: feature_name=MTRNR2L2	MTRNR2L2.value <= threshold=2.8784297704696655
node_2: feature_name=TCL1A	TCL1A.value <= threshold=4.798312664031982
node_3: feature_name=MT-CO1	MT-CO1.value <= threshold=0.08947191759943962
node_4: feature_name=NR4A1	NR4A1.value > threshold=1.0725348591804504
node_1622: feature_name=HLA-DQA2	HLA-DQA2.value > threshold=1.9496816992759705

node_2350: feature_name=BCL2A1	BCL2A1.value <= threshold=1.1322240233421326
node_2351: feature_name=CD83	CD83.value <= threshold=2.3162450790405273
node_2352: feature_name=LTB	LTB.value > threshold=0.7341383993625641
node_2380: feature_name=VPREB3	VPREB3.value > threshold=3.700833559036255
Class: Healthy Control	
Rules_703	passed counts:6
node_0: feature_name=LTB	LTB.value <= threshold=0.994031548500061
node_1: feature_name=MTRNR2L2	MTRNR2L2.value <= threshold=2.8784297704696655
node_2: feature_name=TCL1A	TCL1A.value <= threshold=4.798312664031982
node_3: feature_name=MT-CO1	MT-CO1.value <= threshold=0.08947191759943962
node_4: feature_name=NR4A1	NR4A1.value > threshold=1.0725348591804504
node_1622: feature_name=HLA-DQA2	HLA-DQA2.value > threshold=1.9496816992759705
node_2350: feature_name=BCL2A1	BCL2A1.value <= threshold=1.1322240233421326
node_2351: feature_name=CD83	CD83.value <= threshold=2.3162450790405273
node_2352: feature_name=LTB	LTB.value > threshold=0.7341383993625641
node_2380: feature_name=VPREB3	VPREB3.value <= threshold=3.700833559036255
node_2381: feature_name=NFKBID	NFKBID.value <= threshold=1.520115613937378
node_2382: feature_name=XAF1	XAF1.value > threshold=-0.45230603218078613
Class: COVID-19 Critical	
Rules_704	passed counts:6
node_0: feature_name=LTB	LTB.value <= threshold=0.994031548500061
node_1: feature_name=MTRNR2L2	MTRNR2L2.value <= threshold=2.8784297704696655
node_2: feature_name=TCL1A	TCL1A.value <= threshold=4.798312664031982
node_3: feature_name=MT-CO1	MT-CO1.value <= threshold=0.08947191759943962
node_4: feature_name=NR4A1	NR4A1.value > threshold=1.0725348591804504
node_1622: feature_name=HLA-DQA2	HLA-DQA2.value <= threshold=1.9496816992759705
node_1623: feature_name=HLA-DRB5	HLA-DRB5.value <= threshold=2.715766429901123
node_1624: feature_name=HLA-DPA1	HLA-DPA1.value > threshold=1.7050301432609558

node_2056: feature_name=OAZ1	OAZ1.value > threshold=0.456103190779686
node_2266: feature_name=XAF1	XAF1.value <= threshold=-0.4431648850440979
node_2267: feature_name=MT-CO1	MT-CO1.value > threshold=-0.3505740910768509
node_2293: feature_name=LTB	LTB.value > threshold=-0.31744880229234695
node_2301: feature_name=CD79A	CD79A.value <= threshold=2.299592971801758
node_2302: feature_name=S100A6	S100A6.value > threshold=-0.7221342325210571
Class: COVID-19 Critical	
Rules_705	passed counts:6
node_0: feature_name=LTB	LTB.value <= threshold=0.994031548500061
node_1: feature_name=MTRNR2L2	MTRNR2L2.value <= threshold=2.8784297704696655
node_2: feature_name=TCL1A	TCL1A.value <= threshold=4.798312664031982
node_3: feature_name=MT-CO1	MT-CO1.value <= threshold=0.08947191759943962
node_4: feature_name=NR4A1	NR4A1.value > threshold=1.0725348591804504
node_1622: feature_name=HLA-DQA2	HLA-DQA2.value <= threshold=1.9496816992759705
node_1623: feature_name=HLA-DRB5	HLA-DRB5.value <= threshold=2.715766429901123
node_1624: feature_name=HLA-DPA1	HLA-DPA1.value > threshold=1.7050301432609558
node_2056: feature_name=OAZ1	OAZ1.value > threshold=0.456103190779686
node_2266: feature_name=XAF1	XAF1.value <= threshold=-0.4431648850440979
node_2267: feature_name=MT-CO1	MT-CO1.value > threshold=-0.3505740910768509
node_2293: feature_name=LTB	LTB.value <= threshold=-0.31744880229234695
node_2294: feature_name=PPIF	PPIF.value <= threshold=-0.4462503343820572
node_2295: feature_name=RALGPS2	RALGPS2.value <= threshold=2.9701075553894043
Class: COVID-19 Critical	
Rules_706	passed counts:6
node_0: feature_name=LTB	LTB.value <= threshold=0.994031548500061
node_1: feature_name=MTRNR2L2	MTRNR2L2.value <= threshold=2.8784297704696655
node_2: feature_name=TCL1A	TCL1A.value <= threshold=4.798312664031982
node_3: feature_name=MT-CO1	MT-CO1.value <= threshold=0.08947191759943962

node_4: feature_name=NR4A1	NR4A1.value > threshold=1.0725348591804504
node_1622: feature_name=HLA-DQA2	HLA-DQA2.value <= threshold=1.9496816992759705
node_1623: feature_name=HLA-DRB5	HLA-DRB5.value <= threshold=2.715766429901123
node_1624: feature_name=HLA-DPA1	HLA-DPA1.value > threshold=1.7050301432609558
node_2056: feature_name=OAZ1	OAZ1.value > threshold=0.456103190779686
node_2266: feature_name=XAF1	XAF1.value <= threshold=-0.4431648850440979
node_2267: feature_name=MT-CO1	MT-CO1.value <= threshold=-0.3505740910768509
node_2268: feature_name=HLA-DRB1	HLA-DRB1.value <= threshold=2.1576695442199707
node_2269: feature_name=MARCH1	MARCH1.value <= threshold=1.492592692375183
node_2270: feature_name=CD83	CD83.value <= threshold=1.7977983355522156
node_2271: feature_name=MARCKS	MARCKS.value > threshold=-0.3426544815301895
Class: COVID-19 Critical	
Rules_707	passed counts:6
node_0: feature_name=LTB	LTB.value <= threshold=0.994031548500061
node_1: feature_name=MTRNR2L2	MTRNR2L2.value <= threshold=2.8784297704696655
node_2: feature_name=TCL1A	TCL1A.value <= threshold=4.798312664031982
node_3: feature_name=MT-CO1	MT-CO1.value <= threshold=0.08947191759943962
node_4: feature_name=NR4A1	NR4A1.value > threshold=1.0725348591804504
node_1622: feature_name=HLA-DQA2	HLA-DQA2.value <= threshold=1.9496816992759705
node_1623: feature_name=HLA-DRB5	HLA-DRB5.value <= threshold=2.715766429901123
node_1624: feature_name=HLA-DPA1	HLA-DPA1.value > threshold=1.7050301432609558
node_2056: feature_name=OAZ1	OAZ1.value <= threshold=0.456103190779686
node_2057: feature_name=HLA-DRB5	HLA-DRB5.value > threshold=0.6839984953403473
node_2101: feature_name=HLA-DRB1	HLA-DRB1.value > threshold=1.6199676990509033
node_2151: feature_name=HLA-DQB1	HLA-DQB1.value <= threshold=3.1961567401885986
node_2152: feature_name=PLD4	PLD4.value > threshold=-0.03259922005236149
node_2244: feature_name=LTB	LTB.value > threshold=0.3717459514737129
node_2250: feature_name=SYNE1	SYNE1.value <= threshold=-0.5891828238964081
node_2251: feature_name=IL8	IL8.value <= threshold=0.5656295120716095

Class: COVID-19 Severe	
Rules_708	passed counts:6
node_0: feature_name=LTB	LTB.value <= threshold=0.994031548500061
node_1: feature_name=MTRNR2L2	MTRNR2L2.value <= threshold=2.8784297704696655
node_2: feature_name=TCL1A	TCL1A.value <= threshold=4.798312664031982
node_3: feature_name=MT-CO1	MT-CO1.value <= threshold=0.08947191759943962
node_4: feature_name=NR4A1	NR4A1.value > threshold=1.0725348591804504
node_1622: feature_name=HLA-DQA2	HLA-DQA2.value <= threshold=1.9496816992759705
node_1623: feature_name=HLA-DRB5	HLA-DRB5.value <= threshold=2.715766429901123
node_1624: feature_name=HLA-DPA1	HLA-DPA1.value > threshold=1.7050301432609558
node_2056: feature_name=OAZ1	OAZ1.value <= threshold=0.456103190779686
node_2057: feature_name=HLA-DRB5	HLA-DRB5.value > threshold=0.6839984953403473
node_2101: feature_name=HLA-DRB1	HLA-DRB1.value > threshold=1.6199676990509033
node_2151: feature_name=HLA-DQB1	HLA-DQB1.value <= threshold=3.1961567401885986
node_2152: feature_name=PLD4	PLD4.value <= threshold=-0.03259922005236149
node_2153: feature_name=XIST	XIST.value <= threshold=-0.138966154307127
node_2154: feature_name=HLA-DQA1	HLA-DQA1.value <= threshold=2.0848299264907837
node_2155: feature_name=LTB	LTB.value > threshold=-0.9993145167827606
node_2161: feature_name=CYBB	CYBB.value > threshold=1.514441728591919
node_2183: feature_name=SNX9	SNX9.value <= threshold=1.4872435331344604
node_2184: feature_name=MARCH1	MARCH1.value <= threshold=3.6078312397003174
Class: Healthy Control	
Rules_709	passed counts:6
node_0: feature_name=LTB	LTB.value <= threshold=0.994031548500061
node_1: feature_name=MTRNR2L2	MTRNR2L2.value <= threshold=2.8784297704696655
node_2: feature_name=TCL1A	TCL1A.value <= threshold=4.798312664031982
node_3: feature_name=MT-CO1	MT-CO1.value <= threshold=0.08947191759943962
node_4: feature_name=NR4A1	NR4A1.value > threshold=1.0725348591804504

node_1622: feature_name=HLA-DQA2	HLA-DQA2.value <= threshold=1.9496816992759705
node_1623: feature_name=HLA-DRB5	HLA-DRB5.value <= threshold=2.715766429901123
node_1624: feature_name=HLA-DPA1	HLA-DPA1.value > threshold=1.7050301432609558
node_2056: feature_name=OAZ1	OAZ1.value <= threshold=0.456103190779686
node_2057: feature_name=HLA-DRB5	HLA-DRB5.value > threshold=0.6839984953403473
node_2101: feature_name=HLA-DRB1	HLA-DRB1.value <= threshold=1.6199676990509033
node_2102: feature_name=CD83	CD83.value > threshold=2.5568599700927734
node_2124: feature_name=MARCH1	MARCH1.value > threshold=-0.34506022930145264
node_2140: feature_name=GRASP	GRASP.value > threshold=2.12925124168396
node_2148: feature_name=RALGPS2	RALGPS2.value <= threshold=2.9612797498703003
Class: COVID-19 Severe	
Rules_710	passed counts:6
node_0: feature_name=LTB	LTB.value <= threshold=0.994031548500061
node_1: feature_name=MTRNR2L2	MTRNR2L2.value <= threshold=2.8784297704696655
node_2: feature_name=TCL1A	TCL1A.value <= threshold=4.798312664031982
node_3: feature_name=MT-CO1	MT-CO1.value <= threshold=0.08947191759943962
node_4: feature_name=NR4A1	NR4A1.value > threshold=1.0725348591804504
node_1622: feature_name=HLA-DQA2	HLA-DQA2.value <= threshold=1.9496816992759705
node_1623: feature_name=HLA-DRB5	HLA-DRB5.value <= threshold=2.715766429901123
node_1624: feature_name=HLA-DPA1	HLA-DPA1.value > threshold=1.7050301432609558
node_2056: feature_name=OAZ1	OAZ1.value <= threshold=0.456103190779686
node_2057: feature_name=HLA-DRB5	HLA-DRB5.value > threshold=0.6839984953403473
node_2101: feature_name=HLA-DRB1	HLA-DRB1.value <= threshold=1.6199676990509033
node_2102: feature_name=CD83	CD83.value > threshold=2.5568599700927734
node_2124: feature_name=MARCH1	MARCH1.value <= threshold=-0.34506022930145264
node_2125: feature_name=FTH1	FTH1.value <= threshold=-0.19983553141355515
node_2126: feature_name=ID3	ID3.value <= threshold=1.2159389927983284
Class: Healthy Control	

Rules_711	passed counts:6
node_0: feature_name=LTB	LTB.value <= threshold=0.994031548500061
node_1: feature_name=MTRNR2L2	MTRNR2L2.value <= threshold=2.8784297704696655
node_2: feature_name=TCL1A	TCL1A.value <= threshold=4.798312664031982
node_3: feature_name=MT-CO1	MT-CO1.value <= threshold=0.08947191759943962
node_4: feature_name=NR4A1	NR4A1.value > threshold=1.0725348591804504
node_1622: feature_name=HLA-DQA2	HLA-DQA2.value <= threshold=1.9496816992759705
node_1623: feature_name=HLA-DRB5	HLA-DRB5.value <= threshold=2.715766429901123
node_1624: feature_name=HLA-DPA1	HLA-DPA1.value > threshold=1.7050301432609558
node_2056: feature_name=OAZ1	OAZ1.value <= threshold=0.456103190779686
node_2057: feature_name=HLA-DRB5	HLA-DRB5.value > threshold=0.6839984953403473
node_2101: feature_name=HLA-DRB1	HLA-DRB1.value <= threshold=1.6199676990509033
node_2102: feature_name=CD83	CD83.value <= threshold=2.5568599700927734
node_2103: feature_name=MT-CO1	MT-CO1.value <= threshold=-0.08588453382253647
node_2104: feature_name=FAM129C	FAM129C.value <= threshold=3.9770365953445435
node_2105: feature_name=OAZ1	OAZ1.value > threshold=0.13203191384673119
node_2113: feature_name=HLA-DQA1	HLA-DQA1.value > threshold=2.55633544921875
Class: Healthy Control	
Rules_712	passed counts:6
node_0: feature_name=LTB	LTB.value <= threshold=0.994031548500061
node_1: feature_name=MTRNR2L2	MTRNR2L2.value <= threshold=2.8784297704696655
node_2: feature_name=TCL1A	TCL1A.value <= threshold=4.798312664031982
node_3: feature_name=MT-CO1	MT-CO1.value <= threshold=0.08947191759943962
node_4: feature_name=NR4A1	NR4A1.value > threshold=1.0725348591804504
node_1622: feature_name=HLA-DQA2	HLA-DQA2.value <= threshold=1.9496816992759705
node_1623: feature_name=HLA-DRB5	HLA-DRB5.value <= threshold=2.715766429901123
node_1624: feature_name=HLA-DPA1	HLA-DPA1.value > threshold=1.7050301432609558
node_2056: feature_name=OAZ1	OAZ1.value <= threshold=0.456103190779686
node_2057: feature_name=HLA-DRB5	HLA-DRB5.value <= threshold=0.6839984953403473

node_2058: feature_name=IFI6	IFI6.value <= threshold=-0.5314981639385223
node_2059: feature_name=CD83	CD83.value > threshold=2.6418607234954834
node_2075: feature_name=NFKBID	NFKBID.value > threshold=3.104505181312561
node_2079: feature_name=HLA-DPA1	HLA-DPA1.value > threshold=1.8489333391189575
Class: COVID-19 Critical	
Rules_713	passed counts:6
node_0: feature_name=LTB	LTB.value <= threshold=0.994031548500061
node_1: feature_name=MTRNR2L2	MTRNR2L2.value <= threshold=2.8784297704696655
node_2: feature_name=TCL1A	TCL1A.value <= threshold=4.798312664031982
node_3: feature_name=MT-CO1	MT-CO1.value <= threshold=0.08947191759943962
node_4: feature_name=NR4A1	NR4A1.value > threshold=1.0725348591804504
node_1622: feature_name=HLA-DQA2	HLA-DQA2.value <= threshold=1.9496816992759705
node_1623: feature_name=HLA-DRB5	HLA-DRB5.value <= threshold=2.715766429901123
node_1624: feature_name=HLA-DPA1	HLA-DPA1.value <= threshold=1.7050301432609558
node_1625: feature_name=CD83	CD83.value > threshold=2.783489227294922
node_1873: feature_name=IFI44L	IFI44L.value <= threshold=1.5938722491264343
node_1874: feature_name=PLD4	PLD4.value <= threshold=4.101001739501953
node_1875: feature_name=RHOB	RHOB.value > threshold=1.304835319519043
node_1965: feature_name=HLA-DQB1	HLA-DQB1.value > threshold=1.5989137291908264
node_1991: feature_name=COTL1	COTL1.value > threshold=-1.2334879636764526
node_1997: feature_name=HSP90B1	HSP90B1.value > threshold=1.903715431690216
node_2023: feature_name=MARCH1	MARCH1.value <= threshold=-0.33349713683128357
Class: COVID-19 Severe	
Rules_714	passed counts:6
node_0: feature_name=LTB	LTB.value <= threshold=0.994031548500061
node_1: feature_name=MTRNR2L2	MTRNR2L2.value <= threshold=2.8784297704696655
node_2: feature_name=TCL1A	TCL1A.value <= threshold=4.798312664031982
node_3: feature_name=MT-CO1	MT-CO1.value <= threshold=0.08947191759943962

node_4: feature_name=NR4A1	NR4A1.value > threshold=1.0725348591804504
node_1622: feature_name=HLA-DQA2	HLA-DQA2.value <= threshold=1.9496816992759705
node_1623: feature_name=HLA-DRB5	HLA-DRB5.value <= threshold=2.715766429901123
node_1624: feature_name=HLA-DPA1	HLA-DPA1.value <= threshold=1.7050301432609558
node_1625: feature_name=CD83	CD83.value > threshold=2.783489227294922
node_1873: feature_name=IFI44L	IFI44L.value <= threshold=1.5938722491264343
node_1874: feature_name=PLD4	PLD4.value <= threshold=4.101001739501953
node_1875: feature_name=RHOB	RHOB.value <= threshold=1.304835319519043
node_1876: feature_name=PPIF	PPIF.value <= threshold=2.2943923473358154
node_1877: feature_name=OAZ1	OAZ1.value > threshold=0.04260866530239582
node_1935: feature_name=RGS1	RGS1.value <= threshold=-0.13325228542089462
node_1936: feature_name=BCL2A1	BCL2A1.value <= threshold=2.528669595718384
node_1937: feature_name=RALGPS2	RALGPS2.value > threshold=2.1082775592803955
node_1945: feature_name=MT-ND6	MT-ND6.value <= threshold=-0.9341086447238922
Class: COVID-19 Critical	
Rules_715	passed counts:6
node_0: feature_name=LTB	LTB.value <= threshold=0.994031548500061
node_1: feature_name=MTRNR2L2	MTRNR2L2.value <= threshold=2.8784297704696655
node_2: feature_name=TCL1A	TCL1A.value <= threshold=4.798312664031982
node_3: feature_name=MT-CO1	MT-CO1.value <= threshold=0.08947191759943962
node_4: feature_name=NR4A1	NR4A1.value > threshold=1.0725348591804504
node_1622: feature_name=HLA-DQA2	HLA-DQA2.value <= threshold=1.9496816992759705
node_1623: feature_name=HLA-DRB5	HLA-DRB5.value <= threshold=2.715766429901123
node_1624: feature_name=HLA-DPA1	HLA-DPA1.value <= threshold=1.7050301432609558
node_1625: feature_name=CD83	CD83.value > threshold=2.783489227294922
node_1873: feature_name=IFI44L	IFI44L.value <= threshold=1.5938722491264343
node_1874: feature_name=PLD4	PLD4.value <= threshold=4.101001739501953
node_1875: feature_name=RHOB	RHOB.value <= threshold=1.304835319519043
node_1876: feature_name=PPIF	PPIF.value <= threshold=2.2943923473358154

node_1877: feature_name=OAZ1	OAZ1.value <= threshold=0.04260866530239582
node_1878: feature_name=ID3	ID3.value > threshold=5.290210247039795
node_1932: feature_name=NAMPT	NAMPT.value > threshold=-0.5633004009723663
Class: COVID-19 Severe	
Rules_716	passed counts:6
node_0: feature_name=LTB	LTB.value <= threshold=0.994031548500061
node_1: feature_name=MTRNR2L2	MTRNR2L2.value <= threshold=2.8784297704696655
node_2: feature_name=TCL1A	TCL1A.value <= threshold=4.798312664031982
node_3: feature_name=MT-CO1	MT-CO1.value <= threshold=0.08947191759943962
node_4: feature_name=NR4A1	NR4A1.value > threshold=1.0725348591804504
node_1622: feature_name=HLA-DQA2	HLA-DQA2.value <= threshold=1.9496816992759705
node_1623: feature_name=HLA-DRB5	HLA-DRB5.value <= threshold=2.715766429901123
node_1624: feature_name=HLA-DPA1	HLA-DPA1.value <= threshold=1.7050301432609558
node_1625: feature_name=CD83	CD83.value > threshold=2.783489227294922
node_1873: feature_name=IFI44L	IFI44L.value <= threshold=1.5938722491264343
node_1874: feature_name=PLD4	PLD4.value <= threshold=4.101001739501953
node_1875: feature_name=RHOB	RHOB.value <= threshold=1.304835319519043
node_1876: feature_name=PPIF	PPIF.value <= threshold=2.2943923473358154
node_1877: feature_name=OAZ1	OAZ1.value <= threshold=0.04260866530239582
node_1878: feature_name=ID3	ID3.value <= threshold=5.290210247039795
node_1879: feature_name=NFKBID	NFKBID.value > threshold=-0.40532417595386505
node_1883: feature_name=MEF2C	MEF2C.value > threshold=-0.4597593992948532
node_1887: feature_name=SNX9	SNX9.value <= threshold=1.010923683643341
node_1888: feature_name=HLA-DRB5	HLA-DRB5.value > threshold=-0.4844578504562378
node_1890: feature_name=HLA-DPB1	HLA-DPB1.value > threshold=1.4238839745521545
node_1894: feature_name=NFKBID	NFKBID.value > threshold=2.7626200914382935
node_1898: feature_name=MIR24-2	MIR24-2.value > threshold=-0.5253734588623047
Class: COVID-19 Critical	

Rules_717	passed counts:6
node_0: feature_name=LTB	LTB.value <= threshold=0.994031548500061
node_1: feature_name=MTRNR2L2	MTRNR2L2.value <= threshold=2.8784297704696655
node_2: feature_name=TCL1A	TCL1A.value <= threshold=4.798312664031982
node_3: feature_name=MT-CO1	MT-CO1.value <= threshold=0.08947191759943962
node_4: feature_name=NR4A1	NR4A1.value > threshold=1.0725348591804504
node_1622: feature_name=HLA-DQA2	HLA-DQA2.value <= threshold=1.9496816992759705
node_1623: feature_name=HLA-DRB5	HLA-DRB5.value <= threshold=2.715766429901123
node_1624: feature_name=HLA-DPA1	HLA-DPA1.value <= threshold=1.7050301432609558
node_1625: feature_name=CD83	CD83.value <= threshold=2.783489227294922
node_1626: feature_name=XIST	XIST.value <= threshold=0.9589543007314205
node_1627: feature_name=PLD4	PLD4.value > threshold=5.865932464599609
node_1863: feature_name=MT-CO1	MT-CO1.value <= threshold=-0.6275922656059265
Class: COVID-19 Critical	
Rules_718	passed counts:6
node_0: feature_name=LTB	LTB.value <= threshold=0.994031548500061
node_1: feature_name=MTRNR2L2	MTRNR2L2.value <= threshold=2.8784297704696655
node_2: feature_name=TCL1A	TCL1A.value <= threshold=4.798312664031982
node_3: feature_name=MT-CO1	MT-CO1.value <= threshold=0.08947191759943962
node_4: feature_name=NR4A1	NR4A1.value > threshold=1.0725348591804504
node_1622: feature_name=HLA-DQA2	HLA-DQA2.value <= threshold=1.9496816992759705
node_1623: feature_name=HLA-DRB5	HLA-DRB5.value <= threshold=2.715766429901123
node_1624: feature_name=HLA-DPA1	HLA-DPA1.value <= threshold=1.7050301432609558
node_1625: feature_name=CD83	CD83.value <= threshold=2.783489227294922
node_1626: feature_name=XIST	XIST.value <= threshold=0.9589543007314205
node_1627: feature_name=PLD4	PLD4.value <= threshold=5.865932464599609
node_1628: feature_name=OAZ1	OAZ1.value <= threshold=0.6137169003486633
node_1629: feature_name=MTRNR2L2	MTRNR2L2.value <= threshold=1.6102792620658875
node_1630: feature_name=IFI6	IFI6.value > threshold=-0.8404486179351807

node_1632: feature_name=NR4A1	NR4A1.value > threshold=1.0916320085525513
node_1638: feature_name=DENND3	DENND3.value <= threshold=3.988452672958374
node_1639: feature_name=HLA-DRB1	HLA-DRB1.value <= threshold=1.4092575907707214
node_1640: feature_name=HLA-DPA1	HLA-DPA1.value > threshold=1.2675696015357971
node_1708: feature_name=CYBB	CYBB.value <= threshold=1.8550206422805786
node_1709: feature_name=JUN	JUN.value > threshold=-0.04836222808808088
node_1723: feature_name=NT5C3A	NT5C3A.value <= threshold=1.9380813241004944
node_1724: feature_name=FAM129C	FAM129C.value <= threshold=4.6762073040008545
node_1725: feature_name=FERMT3	FERMT3.value <= threshold=1.1511099338531494
node_1726: feature_name=GPX1	GPX1.value > threshold=0.10868745669722557
node_1734: feature_name=RALGPS2	RALGPS2.value <= threshold=1.23886239528656
Class: COVID-19 Critical	
Rules_719	passed counts:6
node_0: feature_name=LTB	LTB.value <= threshold=0.994031548500061
node_1: feature_name=MTRNR2L2	MTRNR2L2.value <= threshold=2.8784297704696655
node_2: feature_name=TCL1A	TCL1A.value <= threshold=4.798312664031982
node_3: feature_name=MT-CO1	MT-CO1.value <= threshold=0.08947191759943962
node_4: feature_name=NR4A1	NR4A1.value > threshold=1.0725348591804504
node_1622: feature_name=HLA-DQA2	HLA-DQA2.value <= threshold=1.9496816992759705
node_1623: feature_name=HLA-DRB5	HLA-DRB5.value <= threshold=2.715766429901123
node_1624: feature_name=HLA-DPA1	HLA-DPA1.value <= threshold=1.7050301432609558
node_1625: feature_name=CD83	CD83.value <= threshold=2.783489227294922
node_1626: feature_name=XIST	XIST.value <= threshold=0.9589543007314205
node_1627: feature_name=PLD4	PLD4.value <= threshold=5.865932464599609
node_1628: feature_name=OAZ1	OAZ1.value <= threshold=0.6137169003486633
node_1629: feature_name=MTRNR2L2	MTRNR2L2.value <= threshold=1.6102792620658875
node_1630: feature_name=IFI6	IFI6.value > threshold=-0.8404486179351807
node_1632: feature_name=NR4A1	NR4A1.value > threshold=1.0916320085525513
node_1638: feature_name=DENND3	DENND3.value <= threshold=3.988452672958374

node_1639: feature_name=HLA-DRB1	HLA-DRB1.value <= threshold=1.4092575907707214
node_1640: feature_name=HLA-DPA1	HLA-DPA1.value <= threshold=1.2675696015357971
node_1641: feature_name=TRAF1	TRAF1.value <= threshold=2.918454885482788
node_1642: feature_name=HLA-DPA1	HLA-DPA1.value > threshold=-0.917132705450058
node_1648: feature_name=LTB	LTB.value <= threshold=0.9716593027114868
node_1649: feature_name=LDLRAD4	LDLRAD4.value <= threshold=4.4251909255981445
node_1650: feature_name=BCL2A1	BCL2A1.value > threshold=-0.7959703803062439
node_1652: feature_name=PRDM1	PRDM1.value <= threshold=2.6821553707122803
node_1653: feature_name=FAM129C	FAM129C.value <= threshold=4.9084858894348145
node_1654: feature_name=BOD1L1	BOD1L1.value <= threshold=2.9830023050308228
node_1655: feature_name=HSP90B1	HSP90B1.value <= threshold=2.581766724586487
node_1656: feature_name=HLA-DQB1	HLA-DQB1.value <= threshold=3.3723777532577515
node_1657: feature_name=ANO6	ANO6.value <= threshold=2.491254210472107
node_1658: feature_name=RP5-821D11.7	RP5-821D11.7.value <= threshold=3.2165164947509766
node_1659: feature_name=MARCH1	MARCH1.value <= threshold=4.2638514041900635
node_1660: feature_name=ABCA1	ABCA1.value <= threshold=3.618377923965454
node_1661: feature_name=LTB	LTB.value > threshold=0.8862588405609131
node_1683: feature_name=XAF1	XAF1.value <= threshold=0.26804937422275543
Class: COVID-19 Critical	
Rules_720	passed counts:6
node_0: feature_name=LTB	LTB.value <= threshold=0.994031548500061
node_1: feature_name=MTRNR2L2	MTRNR2L2.value <= threshold=2.8784297704696655
node_2: feature_name=TCL1A	TCL1A.value <= threshold=4.798312664031982
node_3: feature_name=MT-CO1	MT-CO1.value <= threshold=0.08947191759943962
node_4: feature_name=NR4A1	NR4A1.value <= threshold=1.0725348591804504
node_5: feature_name=HLA-DPA1	HLA-DPA1.value > threshold=1.6885469555854797
node_861: feature_name=CD83	CD83.value > threshold=3.0563642978668213
node_1539: feature_name=ID1	ID1.value <= threshold=1.4626862406730652
node_1540: feature_name=FTH1	FTH1.value > threshold=0.2985198348760605

node_1590: feature_name=RP5-821D11.7	RP5-821D11.7.value <= threshold=-0.2580558806657791
node_1591: feature_name=HSP90B1	HSP90B1.value <= threshold=1.1008275151252747
node_1592: feature_name=S100A6	S100A6.value > threshold=-0.45454199612140656
node_1600: feature_name=IRF4	IRF4.value > threshold=-0.20501960068941116
node_1602: feature_name=IFIT3	IFIT3.value <= threshold=-0.17674797028303146
Class: COVID-19 Critical	
Rules_721	passed counts:6
node_0: feature_name=LTB	LTB.value <= threshold=0.994031548500061
node_1: feature_name=MTRNR2L2	MTRNR2L2.value <= threshold=2.8784297704696655
node_2: feature_name=TCL1A	TCL1A.value <= threshold=4.798312664031982
node_3: feature_name=MT-CO1	MT-CO1.value <= threshold=0.08947191759943962
node_4: feature_name=NR4A1	NR4A1.value <= threshold=1.0725348591804504
node_5: feature_name=HLA-DPA1	HLA-DPA1.value > threshold=1.6885469555854797
node_861: feature_name=CD83	CD83.value > threshold=3.0563642978668213
node_1539: feature_name=ID1	ID1.value <= threshold=1.4626862406730652
node_1540: feature_name=FTH1	FTH1.value > threshold=0.2985198348760605
node_1590: feature_name=RP5-821D11.7	RP5-821D11.7.value <= threshold=-0.2580558806657791
node_1591: feature_name=HSP90B1	HSP90B1.value <= threshold=1.1008275151252747
node_1592: feature_name=S100A6	S100A6.value > threshold=-0.45454199612140656
node_1600: feature_name=IRF4	IRF4.value <= threshold=-0.20501960068941116
Class: COVID-19 Severe	
Rules_722	passed counts:6
node_0: feature_name=LTB	LTB.value <= threshold=0.994031548500061
node_1: feature_name=MTRNR2L2	MTRNR2L2.value <= threshold=2.8784297704696655
node_2: feature_name=TCL1A	TCL1A.value <= threshold=4.798312664031982
node_3: feature_name=MT-CO1	MT-CO1.value <= threshold=0.08947191759943962
node_4: feature_name=NR4A1	NR4A1.value <= threshold=1.0725348591804504
node_5: feature_name=HLA-DPA1	HLA-DPA1.value > threshold=1.6885469555854797

node_861: feature_name=CD83	CD83.value <= threshold=3.0563642978668213
node_862: feature_name=HLA-DQB1	HLA-DQB1.value > threshold=2.61081063747406
node_1358: feature_name=HLA-DRB5	HLA-DRB5.value > threshold=0.7542744874954224
node_1390: feature_name=HLA-DRB5	HLA-DRB5.value > threshold=2.6244447231292725
node_1516: feature_name=HLA-DQA2	HLA-DQA2.value <= threshold=-0.18513725697994232
node_1517: feature_name=HLA-DPB1	HLA-DPB1.value > threshold=2.381898283958435
node_1521: feature_name=CD79B	CD79B.value > threshold=1.831205129623413
node_1525: feature_name=NFKBID	NFKBID.value > threshold=0.774367168545723
Class: COVID-19 Severe	
Rules_723	passed counts:6
node_0: feature_name=LTB	LTB.value <= threshold=0.994031548500061
node_1: feature_name=MTRNR2L2	MTRNR2L2.value <= threshold=2.8784297704696655
node_2: feature_name=TCL1A	TCL1A.value <= threshold=4.798312664031982
node_3: feature_name=MT-CO1	MT-CO1.value <= threshold=0.08947191759943962
node_4: feature_name=NR4A1	NR4A1.value <= threshold=1.0725348591804504
node_5: feature_name=HLA-DPA1	HLA-DPA1.value > threshold=1.6885469555854797
node_861: feature_name=CD83	CD83.value <= threshold=3.0563642978668213
node_862: feature_name=HLA-DQB1	HLA-DQB1.value > threshold=2.61081063747406
node_1358: feature_name=HLA-DRB5	HLA-DRB5.value > threshold=0.7542744874954224
node_1390: feature_name=HLA-DRB5	HLA-DRB5.value > threshold=2.6244447231292725
node_1516: feature_name=HLA-DQA2	HLA-DQA2.value <= threshold=-0.18513725697994232
node_1517: feature_name=HLA-DPB1	HLA-DPB1.value > threshold=2.381898283958435
node_1521: feature_name=CD79B	CD79B.value <= threshold=1.831205129623413
node_1522: feature_name=ENDOD1	ENDOD1.value > threshold=-0.23476968705654144
Class: COVID-19 Critical	
Rules_724	passed counts:6
node_0: feature_name=LTB	LTB.value <= threshold=0.994031548500061
node_1: feature_name=MTRNR2L2	MTRNR2L2.value <= threshold=2.8784297704696655

node_2: feature_name=TCL1A	TCL1A.value <= threshold=4.798312664031982
node_3: feature_name=MT-CO1	MT-CO1.value <= threshold=0.08947191759943962
node_4: feature_name=NR4A1	NR4A1.value <= threshold=1.0725348591804504
node_5: feature_name=HLA-DPA1	HLA-DPA1.value > threshold=1.6885469555854797
node_861: feature_name=CD83	CD83.value <= threshold=3.0563642978668213
node_862: feature_name=HLA-DQB1	HLA-DQB1.value > threshold=2.61081063747406
node_1358: feature_name=HLA-DRB5	HLA-DRB5.value <= threshold=0.7542744874954224
node_1359: feature_name=MT-CYB	MT-CYB.value > threshold=-0.06432783603668213
node_1375: feature_name=RP11-731F5.2	RP11-731F5.2.value > threshold=-0.09639483317732811
node_1385: feature_name=HSP90B1	HSP90B1.value <= threshold=0.9035855233669281
Class: COVID-19 Critical	
Rules_725	passed counts:6
node_0: feature_name=LTB	LTB.value <= threshold=0.994031548500061
node_1: feature_name=MTRNR2L2	MTRNR2L2.value <= threshold=2.8784297704696655
node_2: feature_name=TCL1A	TCL1A.value <= threshold=4.798312664031982
node_3: feature_name=MT-CO1	MT-CO1.value <= threshold=0.08947191759943962
node_4: feature_name=NR4A1	NR4A1.value <= threshold=1.0725348591804504
node_5: feature_name=HLA-DPA1	HLA-DPA1.value > threshold=1.6885469555854797
node_861: feature_name=CD83	CD83.value <= threshold=3.0563642978668213
node_862: feature_name=HLA-DQB1	HLA-DQB1.value > threshold=2.61081063747406
node_1358: feature_name=HLA-DRB5	HLA-DRB5.value <= threshold=0.7542744874954224
node_1359: feature_name=MT-CYB	MT-CYB.value <= threshold=-0.06432783603668213
node_1360: feature_name=NFKBID	NFKBID.value > threshold=2.1380220651626587
node_1368: feature_name=YWHAH	YWHAH.value > threshold=-0.26376135647296906
node_1372: feature_name=HLA-DRB1	HLA-DRB1.value > threshold=1.3005402088165283
Class: COVID-19 Critical	
Rules_726	passed counts:6
node_0: feature_name=LTB	LTB.value <= threshold=0.994031548500061

node_1: feature_name=MTRNR2L2	MTRNR2L2.value <= threshold=2.8784297704696655
node_2: feature_name=TCL1A	TCL1A.value <= threshold=4.798312664031982
node_3: feature_name=MT-CO1	MT-CO1.value <= threshold=0.08947191759943962
node_4: feature_name=NR4A1	NR4A1.value <= threshold=1.0725348591804504
node_5: feature_name=HLA-DPA1	HLA-DPA1.value > threshold=1.6885469555854797
node_861: feature_name=CD83	CD83.value <= threshold=3.0563642978668213
node_862: feature_name=HLA-DQB1	HLA-DQB1.value <= threshold=2.61081063747406
node_863: feature_name=MTRNR2L2	MTRNR2L2.value <= threshold=2.0919997692108154
node_864: feature_name=BCL2A1	BCL2A1.value <= threshold=1.852618932723999
node_865: feature_name=CD79B	CD79B.value <= threshold=4.352142333984375
node_866: feature_name=TNFAIP3	TNFAIP3.value > threshold=-0.0449496041983366
node_1236: feature_name=HLA-DRB5	HLA-DRB5.value > threshold=1.710871696472168
node_1268: feature_name=MT-CO1	MT-CO1.value <= threshold=-0.8004095554351807
node_1269: feature_name=MTRNR2L8	MTRNR2L8.value <= threshold=-0.3383208364248276
node_1270: feature_name=PELI1	PELI1.value <= threshold=-0.6302085220813751
node_1271: feature_name=TNF	TNF.value > threshold=-0.4168354868888855
Class: COVID-19 Critical	
Rules_727	passed counts:6
node_0: feature_name=LTB	LTB.value <= threshold=0.994031548500061
node_1: feature_name=MTRNR2L2	MTRNR2L2.value <= threshold=2.8784297704696655
node_2: feature_name=TCL1A	TCL1A.value <= threshold=4.798312664031982
node_3: feature_name=MT-CO1	MT-CO1.value <= threshold=0.08947191759943962
node_4: feature_name=NR4A1	NR4A1.value <= threshold=1.0725348591804504
node_5: feature_name=HLA-DPA1	HLA-DPA1.value > threshold=1.6885469555854797
node_861: feature_name=CD83	CD83.value <= threshold=3.0563642978668213
node_862: feature_name=HLA-DQB1	HLA-DQB1.value <= threshold=2.61081063747406
node_863: feature_name=MTRNR2L2	MTRNR2L2.value <= threshold=2.0919997692108154
node_864: feature_name=BCL2A1	BCL2A1.value <= threshold=1.852618932723999
node_865: feature_name=CD79B	CD79B.value <= threshold=4.352142333984375

node_866: feature_name=TNFAIP3	TNFAIP3.value > threshold=-0.0449496041983366
node_1236: feature_name=HLA-DRB5	HLA-DRB5.value <= threshold=1.710871696472168
node_1237: feature_name=HLA-DQA2	HLA-DQA2.value > threshold=3.6443899869918823
node_1263: feature_name=STAG3	STAG3.value > threshold=-0.1967550590634346
node_1265: feature_name=FCER2	FCER2.value > threshold=-0.23549097776412964
Class: COVID-19 Critical	
Rules_728	passed counts:6
node_0: feature_name=LTB	LTB.value <= threshold=0.994031548500061
node_1: feature_name=MTRNR2L2	MTRNR2L2.value <= threshold=2.8784297704696655
node_2: feature_name=TCL1A	TCL1A.value <= threshold=4.798312664031982
node_3: feature_name=MT-CO1	MT-CO1.value <= threshold=0.08947191759943962
node_4: feature_name=NR4A1	NR4A1.value <= threshold=1.0725348591804504
node_5: feature_name=HLA-DPA1	HLA-DPA1.value > threshold=1.6885469555854797
node_861: feature_name=CD83	CD83.value <= threshold=3.0563642978668213
node_862: feature_name=HLA-DQB1	HLA-DQB1.value <= threshold=2.61081063747406
node_863: feature_name=MTRNR2L2	MTRNR2L2.value <= threshold=2.0919997692108154
node_864: feature_name=BCL2A1	BCL2A1.value <= threshold=1.852618932723999
node_865: feature_name=CD79B	CD79B.value <= threshold=4.352142333984375
node_866: feature_name=TNFAIP3	TNFAIP3.value > threshold=-0.0449496041983366
node_1236: feature_name=HLA-DRB5	HLA-DRB5.value <= threshold=1.710871696472168
node_1237: feature_name=HLA-DQA2	HLA-DQA2.value > threshold=3.6443899869918823
node_1263: feature_name=STAG3	STAG3.value <= threshold=-0.1967550590634346
Class: COVID-19 Severe	
Rules_729	passed counts:6
node_0: feature_name=LTB	LTB.value <= threshold=0.994031548500061
node_1: feature_name=MTRNR2L2	MTRNR2L2.value <= threshold=2.8784297704696655
node_2: feature_name=TCL1A	TCL1A.value <= threshold=4.798312664031982
node_3: feature_name=MT-CO1	MT-CO1.value <= threshold=0.08947191759943962

node_4: feature_name=NR4A1	NR4A1.value <= threshold=1.0725348591804504
node_5: feature_name=HLA-DPA1	HLA-DPA1.value > threshold=1.6885469555854797
node_861: feature_name=CD83	CD83.value <= threshold=3.0563642978668213
node_862: feature_name=HLA-DQB1	HLA-DQB1.value <= threshold=2.61081063747406
node_863: feature_name=MTRNR2L2	MTRNR2L2.value <= threshold=2.0919997692108154
node_864: feature_name=BCL2A1	BCL2A1.value <= threshold=1.852618932723999
node_865: feature_name=CD79B	CD79B.value <= threshold=4.352142333984375
node_866: feature_name=TNFAIP3	TNFAIP3.value <= threshold=-0.0449496041983366
node_867: feature_name=MT-CO1	MT-CO1.value <= threshold=-0.08531957119703293
node_868: feature_name=MTRNR2L8	MTRNR2L8.value <= threshold=0.8770350813865662
node_869: feature_name=EGR1	EGR1.value > threshold=-0.32270750403404236
node_1015: feature_name=NR4A3	NR4A3.value <= threshold=1.9085431098937988
node_1016: feature_name=ID3	ID3.value <= threshold=2.776647686958313
node_1017: feature_name=XIST	XIST.value > threshold=2.618046522140503
node_1125: feature_name=RP11-731F5.2	RP11-731F5.2.value <= threshold=-0.14458340406417847
Class: COVID-19 Severe	
Rules_730	passed counts:6
node_0: feature_name=LTB	LTB.value <= threshold=0.994031548500061
node_1: feature_name=MTRNR2L2	MTRNR2L2.value <= threshold=2.8784297704696655
node_2: feature_name=TCL1A	TCL1A.value <= threshold=4.798312664031982
node_3: feature_name=MT-CO1	MT-CO1.value <= threshold=0.08947191759943962
node_4: feature_name=NR4A1	NR4A1.value <= threshold=1.0725348591804504
node_5: feature_name=HLA-DPA1	HLA-DPA1.value > threshold=1.6885469555854797
node_861: feature_name=CD83	CD83.value <= threshold=3.0563642978668213
node_862: feature_name=HLA-DQB1	HLA-DQB1.value <= threshold=2.61081063747406
node_863: feature_name=MTRNR2L2	MTRNR2L2.value <= threshold=2.0919997692108154
node_864: feature_name=BCL2A1	BCL2A1.value <= threshold=1.852618932723999
node_865: feature_name=CD79B	CD79B.value <= threshold=4.352142333984375
node_866: feature_name=TNFAIP3	TNFAIP3.value <= threshold=-0.0449496041983366

node_867: feature_name=MT-CO1	MT-CO1.value <= threshold=-0.08531957119703293
node_868: feature_name=MTRNR2L8	MTRNR2L8.value <= threshold=0.8770350813865662
node_869: feature_name=EGR1	EGR1.value > threshold=-0.32270750403404236
node_1015: feature_name=NR4A3	NR4A3.value <= threshold=1.9085431098937988
node_1016: feature_name=ID3	ID3.value <= threshold=2.776647686958313
node_1017: feature_name=XIST	XIST.value <= threshold=2.618046522140503
node_1018: feature_name=CD79B	CD79B.value > threshold=3.818994164466858
node_1120: feature_name=NT5C3A	NT5C3A.value <= threshold=-0.5003696829080582
Class: COVID-19 Critical	
Rules_731	passed counts:6
node_0: feature_name=LTB	LTB.value <= threshold=0.994031548500061
node_1: feature_name=MTRNR2L2	MTRNR2L2.value <= threshold=2.8784297704696655
node_2: feature_name=TCL1A	TCL1A.value <= threshold=4.798312664031982
node_3: feature_name=MT-CO1	MT-CO1.value <= threshold=0.08947191759943962
node_4: feature_name=NR4A1	NR4A1.value <= threshold=1.0725348591804504
node_5: feature_name=HLA-DPA1	HLA-DPA1.value > threshold=1.6885469555854797
node_861: feature_name=CD83	CD83.value <= threshold=3.0563642978668213
node_862: feature_name=HLA-DQB1	HLA-DQB1.value <= threshold=2.61081063747406
node_863: feature_name=MTRNR2L2	MTRNR2L2.value <= threshold=2.0919997692108154
node_864: feature_name=BCL2A1	BCL2A1.value <= threshold=1.852618932723999
node_865: feature_name=CD79B	CD79B.value <= threshold=4.352142333984375
node_866: feature_name=TNFAIP3	TNFAIP3.value <= threshold=-0.0449496041983366
node_867: feature_name=MT-CO1	MT-CO1.value <= threshold=-0.08531957119703293
node_868: feature_name=MTRNR2L8	MTRNR2L8.value <= threshold=0.8770350813865662
node_869: feature_name=EGR1	EGR1.value <= threshold=-0.32270750403404236
node_870: feature_name=IGJ	IGJ.value <= threshold=4.618318557739258
node_871: feature_name=DUSP4	DUSP4.value <= threshold=2.8197922706604004
node_872: feature_name=HOPX	HOPX.value <= threshold=2.10397732257843
node_873: feature_name=RGCC	RGCC.value <= threshold=1.2070720195770264

node_874: feature_name=OAZ1	OAZ1.value <= threshold=1.02116060256958
node_875: feature_name=NAIP	NAIP.value <= threshold=3.768904685974121
node_876: feature_name=LDLR	LDLR.value <= threshold=2.9582698345184326
node_877: feature_name=HLA-DPA1	HLA-DPA1.value <= threshold=3.0588693618774414
node_878: feature_name=RHOB	RHOB.value <= threshold=5.08755898475647
node_879: feature_name=SYTL3	SYTL3.value <= threshold=1.5127440690994263
node_880: feature_name=FTH1	FTH1.value > threshold=-1.4152275919914246
node_882: feature_name=CCL3	CCL3.value <= threshold=2.253131687641144
node_883: feature_name=CD83	CD83.value <= threshold=2.8566592931747437
node_884: feature_name=HLA-DRB5	HLA-DRB5.value > threshold=-0.6464254856109619
node_888: feature_name=HSP90B1	HSP90B1.value > threshold=-1.186358630657196
node_892: feature_name=ATG2A	ATG2A.value <= threshold=2.3624722957611084
node_893: feature_name=ICAM1	ICAM1.value <= threshold=2.4306468963623047
node_894: feature_name=CD38	CD38.value <= threshold=3.4977954626083374
node_895: feature_name=HLA-DRB1	HLA-DRB1.value > threshold=0.28603747487068176
node_899: feature_name=XIST	XIST.value <= threshold=1.4272559583187103
node_900: feature_name=BANK1	BANK1.value <= threshold=5.060973644256592
node_901: feature_name=IRF4	IRF4.value <= threshold=6.117950916290283
node_902: feature_name=MTRNR2L2	MTRNR2L2.value <= threshold=1.8410876989364624
node_903: feature_name=TPM1	TPM1.value <= threshold=4.590091228485107
node_904: feature_name=SNORD3B-2	SNORD3B-2.value <= threshold=4.349201917648315
node_905: feature_name=AHR	AHR.value <= threshold=2.268427610397339
node_906: feature_name=OAZ1	OAZ1.value > threshold=-1.4369714260101318
node_914: feature_name=MT-ND6	MT-ND6.value <= threshold=1.7712779641151428
node_915: feature_name=HLA-DPB1	HLA-DPB1.value > threshold=2.6004384756088257
node_921: feature_name=SLC7A5	SLC7A5.value <= threshold=1.4944450855255127
Class: COVID-19 Critical	
Rules_732	passed counts:6
node_0: feature_name=LTB	LTB.value <= threshold=0.994031548500061

node_1: feature_name=MTRNR2L2	MTRNR2L2.value <= threshold=2.8784297704696655
node_2: feature_name=TCL1A	TCL1A.value <= threshold=4.798312664031982
node_3: feature_name=MT-CO1	MT-CO1.value <= threshold=0.08947191759943962
node_4: feature_name=NR4A1	NR4A1.value <= threshold=1.0725348591804504
node_5: feature_name=HLA-DPA1	HLA-DPA1.value <= threshold=1.6885469555854797
node_6: feature_name=HLA-DRB5	HLA-DRB5.value <= threshold=2.1886194944381714
node_7: feature_name=XIST	XIST.value > threshold=-0.12328458577394485
node_711: feature_name=HLA-DQB1	HLA-DQB1.value <= threshold=2.337415099143982
node_712: feature_name=TNF	TNF.value > threshold=0.626040518283844
node_728: feature_name=ADAM19	ADAM19.value > threshold=-0.2985510528087616
Class: COVID-19 Severe	
Rules_733	passed counts:6
node_0: feature_name=LTB	LTB.value <= threshold=0.994031548500061
node_1: feature_name=MTRNR2L2	MTRNR2L2.value <= threshold=2.8784297704696655
node_2: feature_name=TCL1A	TCL1A.value <= threshold=4.798312664031982
node_3: feature_name=MT-CO1	MT-CO1.value <= threshold=0.08947191759943962
node_4: feature_name=NR4A1	NR4A1.value <= threshold=1.0725348591804504
node_5: feature_name=HLA-DPA1	HLA-DPA1.value <= threshold=1.6885469555854797
node_6: feature_name=HLA-DRB5	HLA-DRB5.value <= threshold=2.1886194944381714
node_7: feature_name=XIST	XIST.value <= threshold=-0.12328458577394485
node_8: feature_name=SYNE1	SYNE1.value > threshold=-0.7835594415664673
node_16: feature_name=MTRNR2L2	MTRNR2L2.value <= threshold=2.4483247995376587
node_17: feature_name=HLA-DQA2	HLA-DQA2.value > threshold=2.227178931236267
node_591: feature_name=BCL2A1	BCL2A1.value <= threshold=1.0648428201675415
node_592: feature_name=FTH1	FTH1.value <= threshold=0.8842596709728241
node_593: feature_name=SNX9	SNX9.value <= threshold=3.232524275779724
node_594: feature_name=OAZ1	OAZ1.value <= threshold=1.704061508178711
node_595: feature_name=MT-CYB	MT-CYB.value <= threshold=0.5040059983730316
node_596: feature_name=CD83	CD83.value <= threshold=2.535894751548767

node_597: feature_name=ATF5	ATF5.value <= threshold=5.664362907409668
node_598: feature_name=IGLL5	IGLL5.value <= threshold=6.548790216445923
node_599: feature_name=EGR1	EGR1.value <= threshold=6.419021844863892
node_600: feature_name=PLEC	PLEC.value > threshold=1.6971505880355835
node_630: feature_name=KIAA0125	KIAA0125.value <= threshold=2.18315377086401
node_631: feature_name=PLEC	PLEC.value > threshold=1.7833772897720337
Class: COVID-19 Critical	
Rules_734	passed counts:6
node_0: feature_name=LTB	LTB.value <= threshold=0.994031548500061
node_1: feature_name=MTRNR2L2	MTRNR2L2.value <= threshold=2.8784297704696655
node_2: feature_name=TCL1A	TCL1A.value <= threshold=4.798312664031982
node_3: feature_name=MT-CO1	MT-CO1.value <= threshold=0.08947191759943962
node_4: feature_name=NR4A1	NR4A1.value <= threshold=1.0725348591804504
node_5: feature_name=HLA-DPA1	HLA-DPA1.value <= threshold=1.6885469555854797
node_6: feature_name=HLA-DRB5	HLA-DRB5.value <= threshold=2.1886194944381714
node_7: feature_name=XIST	XIST.value <= threshold=-0.12328458577394485
node_8: feature_name=SYNE1	SYNE1.value > threshold=-0.7835594415664673
node_16: feature_name=MTRNR2L2	MTRNR2L2.value <= threshold=2.4483247995376587
node_17: feature_name=HLA-DQA2	HLA-DQA2.value <= threshold=2.227178931236267
node_18: feature_name=NR4A1	NR4A1.value > threshold=-0.21380723267793655
node_504: feature_name=LTB	LTB.value <= threshold=0.8758118152618408
node_505: feature_name=TNFAIP3	TNFAIP3.value <= threshold=0.4779366999864578
node_506: feature_name=HLA-DQB1	HLA-DQB1.value <= threshold=2.9689468145370483
node_507: feature_name=MS4A1	MS4A1.value <= threshold=3.7428637742996216
node_508: feature_name=SNX9	SNX9.value <= threshold=3.304862380027771
node_509: feature_name=MARCKS	MARCKS.value > threshold=-0.519626647233963
node_513: feature_name=CD79B	CD79B.value <= threshold=3.0890846252441406
node_514: feature_name=GNG11	GNG11.value <= threshold=3.3202054500579834
node_515: feature_name=BANK1	BANK1.value <= threshold=-0.3250492364168167

node_516: feature_name=MT-CO1	MT-CO1.value <= threshold=-0.4365827441215515
Class: COVID-19 Critical	
Rules_735	passed counts:6
node_0: feature_name=LTB	LTB.value <= threshold=0.994031548500061
node_1: feature_name=MTRNR2L2	MTRNR2L2.value <= threshold=2.8784297704696655
node_2: feature_name=TCL1A	TCL1A.value <= threshold=4.798312664031982
node_3: feature_name=MT-CO1	MT-CO1.value <= threshold=0.08947191759943962
node_4: feature_name=NR4A1	NR4A1.value <= threshold=1.0725348591804504
node_5: feature_name=HLA-DPA1	HLA-DPA1.value <= threshold=1.6885469555854797
node_6: feature_name=HLA-DRB5	HLA-DRB5.value <= threshold=2.1886194944381714
node_7: feature_name=XIST	XIST.value <= threshold=-0.12328458577394485
node_8: feature_name=SYNE1	SYNE1.value > threshold=-0.7835594415664673
node_16: feature_name=MTRNR2L2	MTRNR2L2.value <= threshold=2.4483247995376587
node_17: feature_name=HLA-DQA2	HLA-DQA2.value <= threshold=2.227178931236267
node_18: feature_name=NR4A1	NR4A1.value > threshold=-0.21380723267793655
node_504: feature_name=LTB	LTB.value <= threshold=0.8758118152618408
node_505: feature_name=TNFAIP3	TNFAIP3.value <= threshold=0.4779366999864578
node_506: feature_name=HLA-DQB1	HLA-DQB1.value <= threshold=2.9689468145370483
node_507: feature_name=MS4A1	MS4A1.value <= threshold=3.7428637742996216
node_508: feature_name=SNX9	SNX9.value <= threshold=3.304862380027771
node_509: feature_name=MARCKS	MARCKS.value <= threshold=-0.519626647233963
node_510: feature_name=TCL1A	TCL1A.value > threshold=-0.1625603437423706
Class: COVID-19 Critical	
Rules_736	passed counts:6
node_0: feature_name=LTB	LTB.value <= threshold=0.994031548500061
node_1: feature_name=MTRNR2L2	MTRNR2L2.value <= threshold=2.8784297704696655
node_2: feature_name=TCL1A	TCL1A.value <= threshold=4.798312664031982
node_3: feature_name=MT-CO1	MT-CO1.value <= threshold=0.08947191759943962

node_4: feature_name=NR4A1	NR4A1.value <= threshold=1.0725348591804504
node_5: feature_name=HLA-DPA1	HLA-DPA1.value <= threshold=1.6885469555854797
node_6: feature_name=HLA-DRB5	HLA-DRB5.value <= threshold=2.1886194944381714
node_7: feature_name=XIST	XIST.value <= threshold=-0.12328458577394485
node_8: feature_name=SYNE1	SYNE1.value > threshold=-0.7835594415664673
node_16: feature_name=MTRNR2L2	MTRNR2L2.value <= threshold=2.4483247995376587
node_17: feature_name=HLA-DQA2	HLA-DQA2.value <= threshold=2.227178931236267
node_18: feature_name=NR4A1	NR4A1.value <= threshold=-0.21380723267793655
node_19: feature_name=IRAK3	IRAK3.value <= threshold=4.27159571647644
node_20: feature_name=IFIT2	IFIT2.value <= threshold=7.8175740242004395
node_21: feature_name=CD79A	CD79A.value > threshold=2.857242226600647
node_375: feature_name=LTB	LTB.value > threshold=0.7405790388584137
node_473: feature_name=MS4A1	MS4A1.value > threshold=2.8195884227752686
node_489: feature_name=FCER2	FCER2.value > threshold=-0.2379418984055519
node_495: feature_name=BANK1	BANK1.value <= threshold=2.0636932253837585
Class: COVID-19 Critical	
Rules_737	passed counts:6
node_0: feature_name=LTB	LTB.value <= threshold=0.994031548500061
node_1: feature_name=MTRNR2L2	MTRNR2L2.value <= threshold=2.8784297704696655
node_2: feature_name=TCL1A	TCL1A.value <= threshold=4.798312664031982
node_3: feature_name=MT-CO1	MT-CO1.value <= threshold=0.08947191759943962
node_4: feature_name=NR4A1	NR4A1.value <= threshold=1.0725348591804504
node_5: feature_name=HLA-DPA1	HLA-DPA1.value <= threshold=1.6885469555854797
node_6: feature_name=HLA-DRB5	HLA-DRB5.value <= threshold=2.1886194944381714
node_7: feature_name=XIST	XIST.value <= threshold=-0.12328458577394485
node_8: feature_name=SYNE1	SYNE1.value > threshold=-0.7835594415664673
node_16: feature_name=MTRNR2L2	MTRNR2L2.value <= threshold=2.4483247995376587
node_17: feature_name=HLA-DQA2	HLA-DQA2.value <= threshold=2.227178931236267
node_18: feature_name=NR4A1	NR4A1.value <= threshold=-0.21380723267793655

node_19: feature_name=IRAK3	IRAK3.value <= threshold=4.27159571647644
node_20: feature_name=IFIT2	IFIT2.value <= threshold=7.8175740242004395
node_21: feature_name=CD79A	CD79A.value > threshold=2.857242226600647
node_375: feature_name=LTB	LTB.value > threshold=0.7405790388584137
node_473: feature_name=MS4A1	MS4A1.value > threshold=2.8195884227752686
node_489: feature_name=FCER2	FCER2.value <= threshold=-0.2379418984055519
node_490: feature_name=HLA-DPA1	HLA-DPA1.value <= threshold=1.6207503080368042
Class: COVID-19 Severe	
Rules_738	passed counts:6
node_0: feature_name=LTB	LTB.value <= threshold=0.994031548500061
node_1: feature_name=MTRNR2L2	MTRNR2L2.value <= threshold=2.8784297704696655
node_2: feature_name=TCL1A	TCL1A.value <= threshold=4.798312664031982
node_3: feature_name=MT-CO1	MT-CO1.value <= threshold=0.08947191759943962
node_4: feature_name=NR4A1	NR4A1.value <= threshold=1.0725348591804504
node_5: feature_name=HLA-DPA1	HLA-DPA1.value <= threshold=1.6885469555854797
node_6: feature_name=HLA-DRB5	HLA-DRB5.value <= threshold=2.1886194944381714
node_7: feature_name=XIST	XIST.value <= threshold=-0.12328458577394485
node_8: feature_name=SYNE1	SYNE1.value > threshold=-0.7835594415664673
node_16: feature_name=MTRNR2L2	MTRNR2L2.value <= threshold=2.4483247995376587
node_17: feature_name=HLA-DQA2	HLA-DQA2.value <= threshold=2.227178931236267
node_18: feature_name=NR4A1	NR4A1.value <= threshold=-0.21380723267793655
node_19: feature_name=IRAK3	IRAK3.value <= threshold=4.27159571647644
node_20: feature_name=IFIT2	IFIT2.value <= threshold=7.8175740242004395
node_21: feature_name=CD79A	CD79A.value <= threshold=2.857242226600647
node_22: feature_name=HIST1H2AC	HIST1H2AC.value <= threshold=5.077470302581787
node_23: feature_name=FTH1	FTH1.value > threshold=0.4158056080341339
node_285: feature_name=EGR1	EGR1.value > threshold=4.240467071533203
node_363: feature_name=NFKBIZ	NFKBIZ.value <= threshold=0.4797569662332535
node_364: feature_name=EGR1	EGR1.value <= threshold=5.980160474777222

Class: COVID-19 Severe	
Rules_739	passed counts:6
node_0: feature_name=LTB	LTB.value <= threshold=0.994031548500061
node_1: feature_name=MTRNR2L2	MTRNR2L2.value <= threshold=2.8784297704696655
node_2: feature_name=TCL1A	TCL1A.value <= threshold=4.798312664031982
node_3: feature_name=MT-CO1	MT-CO1.value <= threshold=0.08947191759943962
node_4: feature_name=NR4A1	NR4A1.value <= threshold=1.0725348591804504
node_5: feature_name=HLA-DPA1	HLA-DPA1.value <= threshold=1.6885469555854797
node_6: feature_name=HLA-DRB5	HLA-DRB5.value <= threshold=2.1886194944381714
node_7: feature_name=XIST	XIST.value <= threshold=-0.12328458577394485
node_8: feature_name=SYNE1	SYNE1.value > threshold=-0.7835594415664673
node_16: feature_name=MTRNR2L2	MTRNR2L2.value <= threshold=2.4483247995376587
node_17: feature_name=HLA-DQA2	HLA-DQA2.value <= threshold=2.227178931236267
node_18: feature_name=NR4A1	NR4A1.value <= threshold=-0.21380723267793655
node_19: feature_name=IRAK3	IRAK3.value <= threshold=4.27159571647644
node_20: feature_name=IFIT2	IFIT2.value <= threshold=7.8175740242004395
node_21: feature_name=CD79A	CD79A.value <= threshold=2.857242226600647
node_22: feature_name=HIST1H2AC	HIST1H2AC.value <= threshold=5.077470302581787
node_23: feature_name=FTH1	FTH1.value <= threshold=0.4158056080341339
node_24: feature_name=ATF3	ATF3.value <= threshold=3.8028353452682495
node_25: feature_name=OASL	OASL.value <= threshold=3.387580633163452
node_26: feature_name=MT-CO1	MT-CO1.value > threshold=-0.7942646145820618
node_74: feature_name=HOPX	HOPX.value <= threshold=0.8682331740856171
node_75: feature_name=RGS2	RGS2.value > threshold=1.4589954018592834
node_213: feature_name=TRAF1	TRAF1.value <= threshold=2.4759315252304077
node_214: feature_name=IRF8	IRF8.value > threshold=-0.4509534388780594
node_218: feature_name=EGR1	EGR1.value <= threshold=4.4260780811309814
node_219: feature_name=PPIF	PPIF.value <= threshold=1.8276893496513367
node_220: feature_name=RGS2	RGS2.value <= threshold=2.9868245124816895

node_221: feature_name=CD79B	CD79B.value <= threshold=4.210061550140381
node_222: feature_name=CD79A	CD79A.value <= threshold=2.8370617628097534
node_223: feature_name=RP5-821D11.7	RP5-821D11.7.value <= threshold=4.518633961677551
node_224: feature_name=HLA-DQB1	HLA-DQB1.value <= threshold=3.2811548709869385
node_225: feature_name=LDLRAD4	LDLRAD4.value > threshold=4.308608055114746
node_233: feature_name=IL8	IL8.value > threshold=-0.3150452971458435
Class: COVID-19 Critical	
Rules_740	passed counts:5
node_0: feature_name=LTB	LTB.value > threshold=0.994031548500061
node_5264: feature_name=MT-CO1	MT-CO1.value > threshold=-0.4127991199493408
node_5638: feature_name=OAZ1	OAZ1.value > threshold=-0.2640867084264755
node_6112: feature_name=IFI44L	IFI44L.value > threshold=2.5893263816833496
node_7078: feature_name=XIST	XIST.value <= threshold=1.1465829275548458
node_7079: feature_name=TCL1A	TCL1A.value <= threshold=7.115930080413818
node_7080: feature_name=XAF1	XAF1.value > threshold=-0.45034870505332947
node_7088: feature_name=FERMT3	FERMT3.value > threshold=1.994587481021881
node_7108: feature_name=MIR24-2	MIR24-2.value <= threshold=0.44104979932308197
Class: Healthy Control	
Rules_741	passed counts:5
node_0: feature_name=LTB	LTB.value > threshold=0.994031548500061
node_5264: feature_name=MT-CO1	MT-CO1.value > threshold=-0.4127991199493408
node_5638: feature_name=OAZ1	OAZ1.value > threshold=-0.2640867084264755
node_6112: feature_name=IFI44L	IFI44L.value <= threshold=2.5893263816833496
node_6113: feature_name=TCL1A	TCL1A.value > threshold=4.201832294464111
node_6991: feature_name=XAF1	XAF1.value > threshold=3.4585667848587036
node_7075: feature_name=WDR74	WDR74.value <= threshold=1.7098039984703064
Class: COVID-19 Critical	

Rules_742	passed counts:5
node_0: feature_name=LTB	LTB.value > threshold=0.994031548500061
node_5264: feature_name=MT-CO1	MT-CO1.value > threshold=-0.4127991199493408
node_5638: feature_name=OAZ1	OAZ1.value > threshold=-0.2640867084264755
node_6112: feature_name=IFI44L	IFI44L.value <= threshold=2.5893263816833496
node_6113: feature_name=TCL1A	TCL1A.value <= threshold=4.201832294464111
node_6114: feature_name=HLA-DQA2	HLA-DQA2.value > threshold=-0.2270243540406227
node_6542: feature_name=CD79B	CD79B.value > threshold=3.096232771873474
node_6872: feature_name=ICAM1	ICAM1.value <= threshold=-0.2232399433851242
node_6873: feature_name=STAG3	STAG3.value <= threshold=-0.19312061369419098
node_6874: feature_name=CD79A	CD79A.value > threshold=1.57558012008667
node_6880: feature_name=MEF2A	MEF2A.value <= threshold=2.676826596260071
node_6881: feature_name=MTRNR2L2	MTRNR2L2.value <= threshold=2.9562686681747437
node_6882: feature_name=SNX9	SNX9.value <= threshold=3.162803888320923
node_6883: feature_name=MARCKS	MARCKS.value <= threshold=4.3125433921813965
node_6884: feature_name=MX1	MX1.value <= threshold=2.1828908920288086
node_6885: feature_name=ATF5	ATF5.value <= threshold=3.838430166244507
node_6886: feature_name=PELI1	PELI1.value > threshold=1.8930315971374512
node_6910: feature_name=RHOB	RHOB.value <= threshold=-0.40518850088119507
node_6911: feature_name=SNX9	SNX9.value > threshold=0.20708411931991577
Class: COVID-19 Critical	
Rules_743	passed counts:5
node_0: feature_name=LTB	LTB.value > threshold=0.994031548500061
node_5264: feature_name=MT-CO1	MT-CO1.value > threshold=-0.4127991199493408
node_5638: feature_name=OAZ1	OAZ1.value > threshold=-0.2640867084264755
node_6112: feature_name=IFI44L	IFI44L.value <= threshold=2.5893263816833496
node_6113: feature_name=TCL1A	TCL1A.value <= threshold=4.201832294464111
node_6114: feature_name=HLA-DQA2	HLA-DQA2.value > threshold=-0.2270243540406227
node_6542: feature_name=CD79B	CD79B.value > threshold=3.096232771873474

node_6872: feature_name=ICAM1	ICAM1.value <= threshold=-0.2232399433851242
node_6873: feature_name=STAG3	STAG3.value <= threshold=-0.19312061369419098
node_6874: feature_name=CD79A	CD79A.value <= threshold=1.57558012008667
node_6875: feature_name=TNFAIP3	TNFAIP3.value <= threshold=-1.4814274907112122
node_6876: feature_name=CASP8	CASP8.value <= threshold=0.2961290776729584
Class: Healthy Control	
Rules_744	passed counts:5
node_0: feature_name=LTB	LTB.value > threshold=0.994031548500061
node_5264: feature_name=MT-CO1	MT-CO1.value > threshold=-0.4127991199493408
node_5638: feature_name=OAZ1	OAZ1.value > threshold=-0.2640867084264755
node_6112: feature_name=IFI44L	IFI44L.value <= threshold=2.5893263816833496
node_6113: feature_name=TCL1A	TCL1A.value <= threshold=4.201832294464111
node_6114: feature_name=HLA-DQA2	HLA-DQA2.value > threshold=-0.2270243540406227
node_6542: feature_name=CD79B	CD79B.value <= threshold=3.096232771873474
node_6543: feature_name=NR4A1	NR4A1.value > threshold=1.421015739440918
node_6811: feature_name=SLC7A5	SLC7A5.value <= threshold=1.570515513420105
node_6812: feature_name=XAF1	XAF1.value > threshold=2.218220591545105
Class: COVID-19 Critical	
Rules_745	passed counts:5
node_0: feature_name=LTB	LTB.value > threshold=0.994031548500061
node_5264: feature_name=MT-CO1	MT-CO1.value > threshold=-0.4127991199493408
node_5638: feature_name=OAZ1	OAZ1.value > threshold=-0.2640867084264755
node_6112: feature_name=IFI44L	IFI44L.value <= threshold=2.5893263816833496
node_6113: feature_name=TCL1A	TCL1A.value <= threshold=4.201832294464111
node_6114: feature_name=HLA-DQA2	HLA-DQA2.value > threshold=-0.2270243540406227
node_6542: feature_name=CD79B	CD79B.value <= threshold=3.096232771873474
node_6543: feature_name=NR4A1	NR4A1.value > threshold=1.421015739440918
node_6811: feature_name=SLC7A5	SLC7A5.value <= threshold=1.570515513420105

node_6812: feature_name=XAF1	XAF1.value <= threshold=2.218220591545105
node_6813: feature_name=MEF2C	MEF2C.value <= threshold=-0.35489463806152344
node_6814: feature_name=MIR24-2	MIR24-2.value <= threshold=1.5691230297088623
node_6815: feature_name=HLA-DQB1	HLA-DQB1.value > threshold=2.6548476219177246
node_6825: feature_name=IRF8	IRF8.value > threshold=-0.36069250106811523
Class: COVID-19 Severe	
Rules_746	passed counts:5
node_0: feature_name=LTB	LTB.value > threshold=0.994031548500061
node_5264: feature_name=MT-CO1	MT-CO1.value > threshold=-0.4127991199493408
node_5638: feature_name=OAZ1	OAZ1.value > threshold=-0.2640867084264755
node_6112: feature_name=IFI44L	IFI44L.value <= threshold=2.5893263816833496
node_6113: feature_name=TCL1A	TCL1A.value <= threshold=4.201832294464111
node_6114: feature_name=HLA-DQA2	HLA-DQA2.value > threshold=-0.2270243540406227
node_6542: feature_name=CD79B	CD79B.value <= threshold=3.096232771873474
node_6543: feature_name=NR4A1	NR4A1.value <= threshold=1.421015739440918
node_6544: feature_name=LTB	LTB.value > threshold=1.359755277633667
node_6720: feature_name=NFKBIZ	NFKBIZ.value <= threshold=0.2720414251089096
node_6721: feature_name=ATF5	ATF5.value <= threshold=2.4672781229019165
node_6722: feature_name=SNX9	SNX9.value > threshold=2.0814030170440674
node_6780: feature_name=JUN	JUN.value > threshold=0.45471879839897156
node_6784: feature_name=PPIF	PPIF.value <= threshold=-0.2260597050189972
Class: Healthy Control	
Rules_747	passed counts:5
node_0: feature_name=LTB	LTB.value > threshold=0.994031548500061
node_5264: feature_name=MT-CO1	MT-CO1.value > threshold=-0.4127991199493408
node_5638: feature_name=OAZ1	OAZ1.value > threshold=-0.2640867084264755
node_6112: feature_name=IFI44L	IFI44L.value <= threshold=2.5893263816833496
node_6113: feature_name=TCL1A	TCL1A.value <= threshold=4.201832294464111

node_6114: feature_name=HLA-DQA2	HLA-DQA2.value > threshold=-0.2270243540406227
node_6542: feature_name=CD79B	CD79B.value <= threshold=3.096232771873474
node_6543: feature_name=NR4A1	NR4A1.value <= threshold=1.421015739440918
node_6544: feature_name=LTB	LTB.value > threshold=1.359755277633667
node_6720: feature_name=NFKBIZ	NFKBIZ.value <= threshold=0.2720414251089096
node_6721: feature_name=ATF5	ATF5.value <= threshold=2.4672781229019165
node_6722: feature_name=SNX9	SNX9.value <= threshold=2.0814030170440674
node_6723: feature_name=HLA-DQA2	HLA-DQA2.value <= threshold=4.360614776611328
node_6724: feature_name=MT-CO1	MT-CO1.value > threshold=-0.12188276648521423
node_6736: feature_name=MT1E	MT1E.value > threshold=-0.1352209895849228
node_6772: feature_name=ITGAX	ITGAX.value > threshold=-0.3478046655654907
Class: COVID-19 Critical	
Rules_748	passed counts:5
node_0: feature_name=LTB	LTB.value > threshold=0.994031548500061
node_5264: feature_name=MT-CO1	MT-CO1.value > threshold=-0.4127991199493408
node_5638: feature_name=OAZ1	OAZ1.value > threshold=-0.2640867084264755
node_6112: feature_name=IFI44L	IFI44L.value <= threshold=2.5893263816833496
node_6113: feature_name=TCL1A	TCL1A.value <= threshold=4.201832294464111
node_6114: feature_name=HLA-DQA2	HLA-DQA2.value > threshold=-0.2270243540406227
node_6542: feature_name=CD79B	CD79B.value <= threshold=3.096232771873474
node_6543: feature_name=NR4A1	NR4A1.value <= threshold=1.421015739440918
node_6544: feature_name=LTB	LTB.value > threshold=1.359755277633667
node_6720: feature_name=NFKBIZ	NFKBIZ.value <= threshold=0.2720414251089096
node_6721: feature_name=ATF5	ATF5.value <= threshold=2.4672781229019165
node_6722: feature_name=SNX9	SNX9.value <= threshold=2.0814030170440674
node_6723: feature_name=HLA-DQA2	HLA-DQA2.value <= threshold=4.360614776611328
node_6724: feature_name=MT-CO1	MT-CO1.value > threshold=-0.12188276648521423
node_6736: feature_name=MT1E	MT1E.value <= threshold=-0.1352209895849228
node_6737: feature_name=PELI1	PELI1.value <= threshold=3.1761642694473267

node_6738: feature_name=TRAF1	TRAF1.value <= threshold=-0.2332744374871254
node_6739: feature_name=HLA-DQA2	HLA-DQA2.value > threshold=-0.22670809924602509
node_6741: feature_name=HLA-DRB5	HLA-DRB5.value > threshold=-0.7810345888137817
node_6745: feature_name=GPX1	GPX1.value > threshold=0.5079085677862167
node_6757: feature_name=FAM129C	FAM129C.value <= threshold=-0.24598311632871628
node_6758: feature_name=SLC11A1	SLC11A1.value <= threshold=-0.31124286353588104
node_6759: feature_name=CD79A	CD79A.value <= threshold=3.088741898536682
Class: COVID-19 Critical	
Rules_749	passed counts:5
node_0: feature_name=LTB	LTB.value > threshold=0.994031548500061
node_5264: feature_name=MT-CO1	MT-CO1.value > threshold=-0.4127991199493408
node_5638: feature_name=OAZ1	OAZ1.value > threshold=-0.2640867084264755
node_6112: feature_name=IFI44L	IFI44L.value <= threshold=2.5893263816833496
node_6113: feature_name=TCL1A	TCL1A.value <= threshold=4.201832294464111
node_6114: feature_name=HLA-DQA2	HLA-DQA2.value > threshold=-0.2270243540406227
node_6542: feature_name=CD79B	CD79B.value <= threshold=3.096232771873474
node_6543: feature_name=NR4A1	NR4A1.value <= threshold=1.421015739440918
node_6544: feature_name=LTB	LTB.value <= threshold=1.359755277633667
node_6545: feature_name=NFKBID	NFKBID.value > threshold=1.4294711351394653
node_6643: feature_name=FAM129C	FAM129C.value <= threshold=4.799884080886841
node_6644: feature_name=XIST	XIST.value <= threshold=0.9931697957217693
node_6645: feature_name=GPX1	GPX1.value <= threshold=0.3628883808851242
node_6646: feature_name=STMN1	STMN1.value > threshold=2.3225364685058594
node_6690: feature_name=MT-CO1	MT-CO1.value > threshold=-0.025839068926870823
Class: Healthy Control	
Rules_750	passed counts:5
node_0: feature_name=LTB	LTB.value > threshold=0.994031548500061
node_5264: feature_name=MT-CO1	MT-CO1.value > threshold=-0.4127991199493408

node_5638: feature_name=OAZ1	OAZ1.value > threshold=-0.2640867084264755
node_6112: feature_name=IFI44L	IFI44L.value <= threshold=2.5893263816833496
node_6113: feature_name=TCL1A	TCL1A.value <= threshold=4.201832294464111
node_6114: feature_name=HLA-DQA2	HLA-DQA2.value > threshold=-0.2270243540406227
node_6542: feature_name=CD79B	CD79B.value <= threshold=3.096232771873474
node_6543: feature_name=NR4A1	NR4A1.value <= threshold=1.421015739440918
node_6544: feature_name=LTB	LTB.value <= threshold=1.359755277633667
node_6545: feature_name=NFKBID	NFKBID.value <= threshold=1.4294711351394653
node_6546: feature_name=HLA-DRB1	HLA-DRB1.value > threshold=1.0398632287979126
node_6556: feature_name=CD79B	CD79B.value > threshold=-0.2919560521841049
node_6582: feature_name=PELI1	PELI1.value > threshold=-0.6156984269618988
node_6602: feature_name=MT-CO3	MT-CO3.value > threshold=0.46141014993190765
node_6632: feature_name=FAM160B1	FAM160B1.value > threshold=-0.3204421401023865
node_6638: feature_name=RALGPS2	RALGPS2.value > threshold=1.8440747261047363
Class: COVID-19 Critical	
Rules_751	passed counts:5
node_0: feature_name=LTB	LTB.value > threshold=0.994031548500061
node_5264: feature_name=MT-CO1	MT-CO1.value > threshold=-0.4127991199493408
node_5638: feature_name=OAZ1	OAZ1.value > threshold=-0.2640867084264755
node_6112: feature_name=IFI44L	IFI44L.value <= threshold=2.5893263816833496
node_6113: feature_name=TCL1A	TCL1A.value <= threshold=4.201832294464111
node_6114: feature_name=HLA-DQA2	HLA-DQA2.value > threshold=-0.2270243540406227
node_6542: feature_name=CD79B	CD79B.value <= threshold=3.096232771873474
node_6543: feature_name=NR4A1	NR4A1.value <= threshold=1.421015739440918
node_6544: feature_name=LTB	LTB.value <= threshold=1.359755277633667
node_6545: feature_name=NFKBID	NFKBID.value <= threshold=1.4294711351394653
node_6546: feature_name=HLA-DRB1	HLA-DRB1.value > threshold=1.0398632287979126
node_6556: feature_name=CD79B	CD79B.value > threshold=-0.2919560521841049
node_6582: feature_name=PELI1	PELI1.value > threshold=-0.6156984269618988

node_6602: feature_name=MT-CO3	MT-CO3.value <= threshold=0.46141014993190765
node_6603: feature_name=NFKBIZ	NFKBIZ.value <= threshold=0.6470938920974731
node_6604: feature_name=HSP90B1	HSP90B1.value <= threshold=0.4268011748790741
node_6605: feature_name=FCER2	FCER2.value <= threshold=-0.2290492281317711
node_6606: feature_name=HLA-DPB1	HLA-DPB1.value <= threshold=1.5359395146369934
Class: COVID-19 Critical	
Rules_752	passed counts:5
node_0: feature_name=LTB	LTB.value > threshold=0.994031548500061
node_5264: feature_name=MT-CO1	MT-CO1.value > threshold=-0.4127991199493408
node_5638: feature_name=OAZ1	OAZ1.value > threshold=-0.2640867084264755
node_6112: feature_name=IFI44L	IFI44L.value <= threshold=2.5893263816833496
node_6113: feature_name=TCL1A	TCL1A.value <= threshold=4.201832294464111
node_6114: feature_name=HLA-DQA2	HLA-DQA2.value <= threshold=-0.2270243540406227
node_6115: feature_name=MTRNR2L2	MTRNR2L2.value <= threshold=2.548917293548584
node_6116: feature_name=ATF5	ATF5.value <= threshold=2.041263461112976
node_6117: feature_name=JUN	JUN.value > threshold=-0.616259902715683
node_6189: feature_name=MT-CO3	MT-CO3.value > threshold=0.13483521342277527
node_6351: feature_name=XAF1	XAF1.value <= threshold=3.061717987060547
node_6352: feature_name=MT-CO2	MT-CO2.value <= threshold=1.2605181336402893
node_6353: feature_name=ICAM1	ICAM1.value <= threshold=-0.3340446650981903
node_6354: feature_name=OAZ1	OAZ1.value > threshold=-0.24055946618318558
node_6360: feature_name=PPIF	PPIF.value <= threshold=2.070030093193054
node_6361: feature_name=RALGPS2	RALGPS2.value > threshold=-0.3684315085411072
node_6369: feature_name=ANO6	ANO6.value <= threshold=3.504153251647949
node_6370: feature_name=YWHAH	YWHAH.value <= threshold=4.3182291984558105
node_6371: feature_name=LDLR	LDLR.value <= threshold=2.494291305541992
node_6372: feature_name=HLA-DQA1	HLA-DQA1.value > threshold=-0.5716990232467651
node_6374: feature_name=ABCA1	ABCA1.value <= threshold=3.7662240266799927
node_6375: feature_name=MS4A1	MS4A1.value > threshold=1.4995208978652954

node_6387: feature_name=SNORD3B-2	SNORD3B-2.value <= threshold=4.154061555862427
node_6388: feature_name=LTB	LTB.value <= threshold=2.1739100217819214
node_6389: feature_name=BACH1	BACH1.value <= threshold=2.0371285676956177
node_6390: feature_name=HLA-DQB1	HLA-DQB1.value <= threshold=3.0258322954177856
node_6391: feature_name=HLA-DPB1	HLA-DPB1.value > threshold=0.2591289207339287
node_6395: feature_name=CXorf57	CXorf57.value <= threshold=3.3742183446884155
node_6396: feature_name=MMD	MMD.value <= threshold=3.8883349895477295
node_6397: feature_name=IRF8	IRF8.value > threshold=3.939465045928955
node_6413: feature_name=TLN1	TLN1.value > threshold=-0.7442445755004883
Class: Healthy Control	
Rules_753	passed counts:5
node_0: feature_name=LTB	LTB.value > threshold=0.994031548500061
node_5264: feature_name=MT-CO1	MT-CO1.value > threshold=-0.4127991199493408
node_5638: feature_name=OAZ1	OAZ1.value > threshold=-0.2640867084264755
node_6112: feature_name=IFI44L	IFI44L.value <= threshold=2.5893263816833496
node_6113: feature_name=TCL1A	TCL1A.value <= threshold=4.201832294464111
node_6114: feature_name=HLA-DQA2	HLA-DQA2.value <= threshold=-0.2270243540406227
node_6115: feature_name=MTRNR2L2	MTRNR2L2.value <= threshold=2.548917293548584
node_6116: feature_name=ATF5	ATF5.value <= threshold=2.041263461112976
node_6117: feature_name=JUN	JUN.value > threshold=-0.616259902715683
node_6189: feature_name=MT-CO3	MT-CO3.value > threshold=0.13483521342277527
node_6351: feature_name=XAF1	XAF1.value <= threshold=3.061717987060547
node_6352: feature_name=MT-CO2	MT-CO2.value <= threshold=1.2605181336402893
node_6353: feature_name=ICAM1	ICAM1.value <= threshold=-0.3340446650981903
node_6354: feature_name=OAZ1	OAZ1.value > threshold=-0.24055946618318558
node_6360: feature_name=PPIF	PPIF.value <= threshold=2.070030093193054
node_6361: feature_name=RALGPS2	RALGPS2.value <= threshold=-0.3684315085411072
node_6362: feature_name=JUN	JUN.value <= threshold=-0.10340499877929688
node_6363: feature_name=MS4A1	MS4A1.value <= threshold=3.1063270568847656

Class: COVID-19 Critical	
Rules_754	passed counts:5
node_0: feature_name=LTB	LTB.value > threshold=0.994031548500061
node_5264: feature_name=MT-CO1	MT-CO1.value > threshold=-0.4127991199493408
node_5638: feature_name=OAZ1	OAZ1.value > threshold=-0.2640867084264755
node_6112: feature_name=IFI44L	IFI44L.value <= threshold=2.5893263816833496
node_6113: feature_name=TCL1A	TCL1A.value <= threshold=4.201832294464111
node_6114: feature_name=HLA-DQA2	HLA-DQA2.value <= threshold=-0.2270243540406227
node_6115: feature_name=MTRNR2L2	MTRNR2L2.value <= threshold=2.548917293548584
node_6116: feature_name=ATF5	ATF5.value <= threshold=2.041263461112976
node_6117: feature_name=JUN	JUN.value > threshold=-0.616259902715683
node_6189: feature_name=MT-CO3	MT-CO3.value > threshold=0.13483521342277527
node_6351: feature_name=XAF1	XAF1.value <= threshold=3.061717987060547
node_6352: feature_name=MT-CO2	MT-CO2.value <= threshold=1.2605181336402893
node_6353: feature_name=ICAM1	ICAM1.value <= threshold=-0.3340446650981903
node_6354: feature_name=OAZ1	OAZ1.value <= threshold=-0.24055946618318558
node_6355: feature_name=MEF2A	MEF2A.value > threshold=1.281972885131836
Class: COVID-19 Critical	
Rules_755	passed counts:5
node_0: feature_name=LTB	LTB.value > threshold=0.994031548500061
node_5264: feature_name=MT-CO1	MT-CO1.value > threshold=-0.4127991199493408
node_5638: feature_name=OAZ1	OAZ1.value > threshold=-0.2640867084264755
node_6112: feature_name=IFI44L	IFI44L.value <= threshold=2.5893263816833496
node_6113: feature_name=TCL1A	TCL1A.value <= threshold=4.201832294464111
node_6114: feature_name=HLA-DQA2	HLA-DQA2.value <= threshold=-0.2270243540406227
node_6115: feature_name=MTRNR2L2	MTRNR2L2.value <= threshold=2.548917293548584
node_6116: feature_name=ATF5	ATF5.value <= threshold=2.041263461112976
node_6117: feature_name=JUN	JUN.value > threshold=-0.616259902715683

node_6189: feature_name=MT-CO3	MT-CO3.value <= threshold=0.13483521342277527
node_6190: feature_name=XAF1	XAF1.value <= threshold=2.584952235221863
node_6191: feature_name=HLA-DRB5	HLA-DRB5.value > threshold=0.6955901980400085
node_6239: feature_name=TNFAIP3	TNFAIP3.value <= threshold=0.11930759996175766
node_6240: feature_name=TP53INP1	TP53INP1.value <= threshold=2.9654784202575684
node_6241: feature_name=HLA-DRB5	HLA-DRB5.value <= threshold=2.5141202211380005
node_6242: feature_name=NFKBID	NFKBID.value <= threshold=3.358878970146179
node_6243: feature_name=JARID2	JARID2.value <= threshold=1.7160060405731201
node_6244: feature_name=HLA-DPA1	HLA-DPA1.value > threshold=0.49491266906261444
node_6246: feature_name=MIR24-2	MIR24-2.value > threshold=1.8348549008369446
node_6312: feature_name=RP11-685N3.1	RP11-685N3.1.value > threshold=-0.17039766907691956
node_6314: feature_name=MS4A1	MS4A1.value > threshold=1.671911597251892
Class: Healthy Control	
Rules_756	passed counts:5
node_0: feature_name=LTB	LTB.value > threshold=0.994031548500061
node_5264: feature_name=MT-CO1	MT-CO1.value > threshold=-0.4127991199493408
node_5638: feature_name=OAZ1	OAZ1.value > threshold=-0.2640867084264755
node_6112: feature_name=IFI44L	IFI44L.value <= threshold=2.5893263816833496
node_6113: feature_name=TCL1A	TCL1A.value <= threshold=4.201832294464111
node_6114: feature_name=HLA-DQA2	HLA-DQA2.value <= threshold=-0.2270243540406227
node_6115: feature_name=MTRNR2L2	MTRNR2L2.value <= threshold=2.548917293548584
node_6116: feature_name=ATF5	ATF5.value <= threshold=2.041263461112976
node_6117: feature_name=JUN	JUN.value > threshold=-0.616259902715683
node_6189: feature_name=MT-CO3	MT-CO3.value <= threshold=0.13483521342277527
node_6190: feature_name=XAF1	XAF1.value <= threshold=2.584952235221863
node_6191: feature_name=HLA-DRB5	HLA-DRB5.value <= threshold=0.6955901980400085
node_6192: feature_name=NR4A1	NR4A1.value <= threshold=-0.4496448189020157
node_6193: feature_name=HLA-DPB1	HLA-DPB1.value > threshold=1.7609722018241882
node_6213: feature_name=SNX9	SNX9.value > threshold=1.1182581186294556

node_6219: feature_name=CXorf57	CXorf57.value > threshold=-0.21670392155647278
Class: COVID-19 Critical	
Rules_757	passed counts:5
node_0: feature_name=LTB	LTB.value > threshold=0.994031548500061
node_5264: feature_name=MT-CO1	MT-CO1.value > threshold=-0.4127991199493408
node_5638: feature_name=OAZ1	OAZ1.value <= threshold=-0.2640867084264755
node_5639: feature_name=CD79B	CD79B.value > threshold=2.2998517751693726
node_5925: feature_name=SNX9	SNX9.value <= threshold=2.4445992708206177
node_5926: feature_name=TCL1A	TCL1A.value <= threshold=2.6938670873641968
node_5927: feature_name=PLD4	PLD4.value > threshold=-0.06434785947203636
node_6009: feature_name=MX1	MX1.value <= threshold=3.600893020629883
node_6010: feature_name=PRDM1	PRDM1.value <= threshold=-0.407244011759758
node_6011: feature_name=SNORD3B-2	SNORD3B-2.value > threshold=2.7082353830337524
node_6043: feature_name=SESN3	SESN3.value > threshold=-0.45859090983867645
Class: COVID-19 Critical	
Rules_758	passed counts:5
node_0: feature_name=LTB	LTB.value > threshold=0.994031548500061
node_5264: feature_name=MT-CO1	MT-CO1.value > threshold=-0.4127991199493408
node_5638: feature_name=OAZ1	OAZ1.value <= threshold=-0.2640867084264755
node_5639: feature_name=CD79B	CD79B.value > threshold=2.2998517751693726
node_5925: feature_name=SNX9	SNX9.value <= threshold=2.4445992708206177
node_5926: feature_name=TCL1A	TCL1A.value <= threshold=2.6938670873641968
node_5927: feature_name=PLD4	PLD4.value > threshold=-0.06434785947203636
node_6009: feature_name=MX1	MX1.value <= threshold=3.600893020629883
node_6010: feature_name=PRDM1	PRDM1.value <= threshold=-0.407244011759758
node_6011: feature_name=SNORD3B-2	SNORD3B-2.value <= threshold=2.7082353830337524
node_6012: feature_name=NFKBID	NFKBID.value > threshold=3.0716724395751953
node_6034: feature_name=BANK1	BANK1.value <= threshold=2.832552671432495

Class: COVID-19 Critical	
Rules_759	passed counts:5
node_0: feature_name=LTB	LTB.value > threshold=0.994031548500061
node_5264: feature_name=MT-CO1	MT-CO1.value > threshold=-0.4127991199493408
node_5638: feature_name=OAZ1	OAZ1.value <= threshold=-0.2640867084264755
node_5639: feature_name=CD79B	CD79B.value > threshold=2.2998517751693726
node_5925: feature_name=SNX9	SNX9.value <= threshold=2.4445992708206177
node_5926: feature_name=TCL1A	TCL1A.value <= threshold=2.6938670873641968
node_5927: feature_name=PLD4	PLD4.value > threshold=-0.06434785947203636
node_6009: feature_name=MX1	MX1.value <= threshold=3.600893020629883
node_6010: feature_name=PRDM1	PRDM1.value <= threshold=-0.407244011759758
node_6011: feature_name=SNORD3B-2	SNORD3B-2.value <= threshold=2.7082353830337524
node_6012: feature_name=NFKBID	NFKBID.value <= threshold=3.0716724395751953
node_6013: feature_name=HLA-DQA2	HLA-DQA2.value <= threshold=3.7589778900146484
node_6014: feature_name=HLA-DQB1	HLA-DQB1.value <= threshold=3.291059136390686
node_6015: feature_name=ATG2A	ATG2A.value <= threshold=1.4668332934379578
node_6016: feature_name=IRF4	IRF4.value <= threshold=4.835575103759766
node_6017: feature_name=IL1B	IL1B.value <= threshold=2.2611767649650574
node_6018: feature_name=IFI44L	IFI44L.value > threshold=1.2665085792541504
node_6020: feature_name=SPI1	SPI1.value > threshold=-0.7380362749099731
Class: Healthy Control	
Rules_760	passed counts:5
node_0: feature_name=LTB	LTB.value > threshold=0.994031548500061
node_5264: feature_name=MT-CO1	MT-CO1.value > threshold=-0.4127991199493408
node_5638: feature_name=OAZ1	OAZ1.value <= threshold=-0.2640867084264755
node_5639: feature_name=CD79B	CD79B.value > threshold=2.2998517751693726
node_5925: feature_name=SNX9	SNX9.value <= threshold=2.4445992708206177
node_5926: feature_name=TCL1A	TCL1A.value <= threshold=2.6938670873641968

node_5927: feature_name=PLD4	PLD4.value <= threshold=-0.06434785947203636
node_5928: feature_name=ICAM1	ICAM1.value <= threshold=-0.2820792943239212
node_5929: feature_name=MS4A1	MS4A1.value > threshold=2.747320055961609
node_5963: feature_name=RNF144B	RNF144B.value <= threshold=-0.2324080914258957
node_5964: feature_name=HLA-DRB5	HLA-DRB5.value <= threshold=2.0430362224578857
node_5965: feature_name=MX1	MX1.value > threshold=1.2721255421638489
node_5979: feature_name=DAPP1	DAPP1.value > threshold=-0.3016224354505539
Class: COVID-19 Critical	
Rules_761	passed counts:5
node_0: feature_name=LTB	LTB.value > threshold=0.994031548500061
node_5264: feature_name=MT-CO1	MT-CO1.value > threshold=-0.4127991199493408
node_5638: feature_name=OAZ1	OAZ1.value <= threshold=-0.2640867084264755
node_5639: feature_name=CD79B	CD79B.value > threshold=2.2998517751693726
node_5925: feature_name=SNX9	SNX9.value <= threshold=2.4445992708206177
node_5926: feature_name=TCL1A	TCL1A.value <= threshold=2.6938670873641968
node_5927: feature_name=PLD4	PLD4.value <= threshold=-0.06434785947203636
node_5928: feature_name=ICAM1	ICAM1.value <= threshold=-0.2820792943239212
node_5929: feature_name=MS4A1	MS4A1.value > threshold=2.747320055961609
node_5963: feature_name=RNF144B	RNF144B.value <= threshold=-0.2324080914258957
node_5964: feature_name=HLA-DRB5	HLA-DRB5.value <= threshold=2.0430362224578857
node_5965: feature_name=MX1	MX1.value <= threshold=1.2721255421638489
node_5966: feature_name=LDLRAD4	LDLRAD4.value <= threshold=2.3228561878204346
node_5967: feature_name=LTB	LTB.value <= threshold=1.1045992970466614
node_5968: feature_name=PARP14	PARP14.value > threshold=-0.44172780215740204
Class: Healthy Control	
Rules_762	passed counts:5
node_0: feature_name=LTB	LTB.value > threshold=0.994031548500061
node_5264: feature_name=MT-CO1	MT-CO1.value > threshold=-0.4127991199493408

node_5638: feature_name=OAZ1	OAZ1.value <= threshold=-0.2640867084264755
node_5639: feature_name=CD79B	CD79B.value > threshold=2.2998517751693726
node_5925: feature_name=SNX9	SNX9.value <= threshold=2.4445992708206177
node_5926: feature_name=TCL1A	TCL1A.value <= threshold=2.6938670873641968
node_5927: feature_name=PLD4	PLD4.value <= threshold=-0.06434785947203636
node_5928: feature_name=ICAM1	ICAM1.value <= threshold=-0.2820792943239212
node_5929: feature_name=MS4A1	MS4A1.value <= threshold=2.747320055961609
node_5930: feature_name=LTB	LTB.value <= threshold=1.3533939123153687
node_5931: feature_name=CD83	CD83.value > threshold=3.0086376667022705
node_5951: feature_name=TNF	TNF.value <= threshold=1.6858283430337906
Class: Healthy Control	
Rules_763	passed counts:5
node_0: feature_name=LTB	LTB.value > threshold=0.994031548500061
node_5264: feature_name=MT-CO1	MT-CO1.value > threshold=-0.4127991199493408
node_5638: feature_name=OAZ1	OAZ1.value <= threshold=-0.2640867084264755
node_5639: feature_name=CD79B	CD79B.value <= threshold=2.2998517751693726
node_5640: feature_name=MTRNR2L2	MTRNR2L2.value <= threshold=3.5499863624572754
node_5641: feature_name=MT-CO1	MT-CO1.value > threshold=0.22567133605480194
node_5729: feature_name=MARCH1	MARCH1.value > threshold=1.5154643654823303
node_5867: feature_name=NFKBID	NFKBID.value > threshold=2.6947624683380127
node_5903: feature_name=RGCC	RGCC.value > threshold=-0.730547159910202
node_5909: feature_name=SPI1	SPI1.value > threshold=-0.2540571391582489
node_5915: feature_name=MEF2C	MEF2C.value > threshold=3.5967005491256714
node_5919: feature_name=RGS1	RGS1.value > threshold=-0.4445079267024994
Class: Healthy Control	
Rules_764	passed counts:5
node_0: feature_name=LTB	LTB.value > threshold=0.994031548500061
node_5264: feature_name=MT-CO1	MT-CO1.value > threshold=-0.4127991199493408

node_5638: feature_name=OAZ1	OAZ1.value <= threshold=-0.2640867084264755
node_5639: feature_name=CD79B	CD79B.value <= threshold=2.2998517751693726
node_5640: feature_name=MTRNR2L2	MTRNR2L2.value <= threshold=3.5499863624572754
node_5641: feature_name=MT-CO1	MT-CO1.value > threshold=0.22567133605480194
node_5729: feature_name=MARCH1	MARCH1.value > threshold=1.5154643654823303
node_5867: feature_name=NFKBID	NFKBID.value <= threshold=2.6947624683380127
node_5868: feature_name=UTY	UTY.value > threshold=3.29777729511261
Class: COVID-19 Critical	
Rules_765	passed counts:5
node_0: feature_name=LTB	LTB.value > threshold=0.994031548500061
node_5264: feature_name=MT-CO1	MT-CO1.value > threshold=-0.4127991199493408
node_5638: feature_name=OAZ1	OAZ1.value <= threshold=-0.2640867084264755
node_5639: feature_name=CD79B	CD79B.value <= threshold=2.2998517751693726
node_5640: feature_name=MTRNR2L2	MTRNR2L2.value <= threshold=3.5499863624572754
node_5641: feature_name=MT-CO1	MT-CO1.value > threshold=0.22567133605480194
node_5729: feature_name=MARCH1	MARCH1.value > threshold=1.5154643654823303
node_5867: feature_name=NFKBID	NFKBID.value <= threshold=2.6947624683380127
node_5868: feature_name=UTY	UTY.value <= threshold=3.29777729511261
node_5869: feature_name=IFI44L	IFI44L.value <= threshold=-0.29640993475914
node_5870: feature_name=FTH1	FTH1.value <= threshold=0.6966870129108429
node_5871: feature_name=ILK	ILK.value <= threshold=1.5719919204711914
node_5872: feature_name=MARCKS	MARCKS.value > threshold=1.20343416929245
node_5882: feature_name=RALGPS2	RALGPS2.value <= threshold=2.361847758293152
node_5883: feature_name=PELI1	PELI1.value > threshold=1.1635615229606628
Class: COVID-19 Critical	
Rules_766	passed counts:5
node_0: feature_name=LTB	LTB.value > threshold=0.994031548500061
node_5264: feature_name=MT-CO1	MT-CO1.value > threshold=-0.4127991199493408

node_5638: feature_name=OAZ1	OAZ1.value <= threshold=-0.2640867084264755
node_5639: feature_name=CD79B	CD79B.value <= threshold=2.2998517751693726
node_5640: feature_name=MTRNR2L2	MTRNR2L2.value <= threshold=3.5499863624572754
node_5641: feature_name=MT-CO1	MT-CO1.value > threshold=0.22567133605480194
node_5729: feature_name=MARCH1	MARCH1.value <= threshold=1.5154643654823303
node_5730: feature_name=MT-CO1	MT-CO1.value <= threshold=1.7823500633239746
node_5731: feature_name=HLA-DPB1	HLA-DPB1.value > threshold=2.0096163749694824
node_5829: feature_name=LTB	LTB.value <= threshold=1.2124510407447815
node_5830: feature_name=MT-CO1	MT-CO1.value <= threshold=0.41994138062000275
node_5831: feature_name=TNF	TNF.value <= threshold=0.5043804794549942
Class: COVID-19 Severe	
Rules_767	passed counts:5
node_0: feature_name=LTB	LTB.value > threshold=0.994031548500061
node_5264: feature_name=MT-CO1	MT-CO1.value > threshold=-0.4127991199493408
node_5638: feature_name=OAZ1	OAZ1.value <= threshold=-0.2640867084264755
node_5639: feature_name=CD79B	CD79B.value <= threshold=2.2998517751693726
node_5640: feature_name=MTRNR2L2	MTRNR2L2.value <= threshold=3.5499863624572754
node_5641: feature_name=MT-CO1	MT-CO1.value > threshold=0.22567133605480194
node_5729: feature_name=MARCH1	MARCH1.value <= threshold=1.5154643654823303
node_5730: feature_name=MT-CO1	MT-CO1.value <= threshold=1.7823500633239746
node_5731: feature_name=HLA-DPB1	HLA-DPB1.value <= threshold=2.0096163749694824
node_5732: feature_name=NR4A3	NR4A3.value <= threshold=3.509189486503601
node_5733: feature_name=OAZ1	OAZ1.value <= threshold=-0.4392261207103729
node_5734: feature_name=SESN3	SESN3.value <= threshold=-0.45661941170692444
node_5735: feature_name=WDR74	WDR74.value > threshold=-0.4893670231103897
node_5761: feature_name=FCER2	FCER2.value <= threshold=6.041448354721069
node_5762: feature_name=TCL1A	TCL1A.value > threshold=-0.1774759516119957
node_5782: feature_name=HLA-DQB1	HLA-DQB1.value <= threshold=2.203125238418579
Class: Healthy Control	

Rules_768	passed counts:5
node_0: feature_name=LTB	LTB.value > threshold=0.994031548500061
node_5264: feature_name=MT-CO1	MT-CO1.value > threshold=-0.4127991199493408
node_5638: feature_name=OAZ1	OAZ1.value <= threshold=-0.2640867084264755
node_5639: feature_name=CD79B	CD79B.value <= threshold=2.2998517751693726
node_5640: feature_name=MTRNR2L2	MTRNR2L2.value <= threshold=3.5499863624572754
node_5641: feature_name=MT-CO1	MT-CO1.value > threshold=0.22567133605480194
node_5729: feature_name=MARCH1	MARCH1.value <= threshold=1.5154643654823303
node_5730: feature_name=MT-CO1	MT-CO1.value <= threshold=1.7823500633239746
node_5731: feature_name=HLA-DPB1	HLA-DPB1.value <= threshold=2.0096163749694824
node_5732: feature_name=NR4A3	NR4A3.value <= threshold=3.509189486503601
node_5733: feature_name=OAZ1	OAZ1.value <= threshold=-0.4392261207103729
node_5734: feature_name=SESN3	SESN3.value <= threshold=-0.45661941170692444
node_5735: feature_name=WDR74	WDR74.value <= threshold=-0.4893670231103897
node_5736: feature_name=NR4A1	NR4A1.value <= threshold=-0.2724379748106003
node_5737: feature_name=HLA-DPA1	HLA-DPA1.value > threshold=1.6106070280075073
node_5745: feature_name=MEF2C	MEF2C.value <= threshold=2.7098671197891235
node_5746: feature_name=MMD	MMD.value > threshold=-0.28827425837516785
Class: COVID-19 Severe	
Rules_769	passed counts:5
node_0: feature_name=LTB	LTB.value > threshold=0.994031548500061
node_5264: feature_name=MT-CO1	MT-CO1.value > threshold=-0.4127991199493408
node_5638: feature_name=OAZ1	OAZ1.value <= threshold=-0.2640867084264755
node_5639: feature_name=CD79B	CD79B.value <= threshold=2.2998517751693726
node_5640: feature_name=MTRNR2L2	MTRNR2L2.value <= threshold=3.5499863624572754
node_5641: feature_name=MT-CO1	MT-CO1.value <= threshold=0.22567133605480194
node_5642: feature_name=RGS2	RGS2.value > threshold=1.2649686336517334
node_5710: feature_name=HLA-DPB1	HLA-DPB1.value > threshold=1.09694242477417

node_5712: feature_name=PPIF	PPIF.value > threshold=-0.4469884932041168
node_5720: feature_name=CD83	CD83.value > threshold=1.302907645702362
node_5722: feature_name=COTL1	COTL1.value > threshold=0.1188640259206295
Class: COVID-19 Severe	
Rules_770	passed counts:5
node_0: feature_name=LTB	LTB.value > threshold=0.994031548500061
node_5264: feature_name=MT-CO1	MT-CO1.value > threshold=-0.4127991199493408
node_5638: feature_name=OAZ1	OAZ1.value <= threshold=-0.2640867084264755
node_5639: feature_name=CD79B	CD79B.value <= threshold=2.2998517751693726
node_5640: feature_name=MTRNR2L2	MTRNR2L2.value <= threshold=3.5499863624572754
node_5641: feature_name=MT-CO1	MT-CO1.value <= threshold=0.22567133605480194
node_5642: feature_name=RGS2	RGS2.value <= threshold=1.2649686336517334
node_5643: feature_name=TCL1A	TCL1A.value > threshold=4.771023273468018
node_5703: feature_name=MTRNR2L8	MTRNR2L8.value <= threshold=-0.3554762303829193
node_5704: feature_name=FTH1	FTH1.value <= threshold=0.6365696042776108
Class: COVID-19 Critical	
Rules_771	passed counts:5
node_0: feature_name=LTB	LTB.value > threshold=0.994031548500061
node_5264: feature_name=MT-CO1	MT-CO1.value > threshold=-0.4127991199493408
node_5638: feature_name=OAZ1	OAZ1.value <= threshold=-0.2640867084264755
node_5639: feature_name=CD79B	CD79B.value <= threshold=2.2998517751693726
node_5640: feature_name=MTRNR2L2	MTRNR2L2.value <= threshold=3.5499863624572754
node_5641: feature_name=MT-CO1	MT-CO1.value <= threshold=0.22567133605480194
node_5642: feature_name=RGS2	RGS2.value <= threshold=1.2649686336517334
node_5643: feature_name=TCL1A	TCL1A.value <= threshold=4.771023273468018
node_5644: feature_name=IGJ	IGJ.value > threshold=3.91206431388855
node_5698: feature_name=BACH1	BACH1.value <= threshold=1.0095552802085876
Class: Healthy Control	

Rules_772	passed counts:5
node_0: feature_name=LTB	LTB.value > threshold=0.994031548500061
node_5264: feature_name=MT-CO1	MT-CO1.value <= threshold=-0.4127991199493408
node_5265: feature_name=MTRNR2L2	MTRNR2L2.value <= threshold=2.4473838806152344
node_5266: feature_name=TCL1A	TCL1A.value <= threshold=5.787256956100464
node_5267: feature_name=PLD4	PLD4.value > threshold=3.544657826423645
node_5577: feature_name=MT-CO1	MT-CO1.value <= threshold=-0.6592064797878265
node_5578: feature_name=HOPX	HOPX.value > threshold=-0.47573523223400116
node_5584: feature_name=OAZ1	OAZ1.value <= threshold=0.5679030418395996
node_5585: feature_name=MS4A1	MS4A1.value > threshold=2.869006037712097
node_5595: feature_name=YWHAH	YWHAH.value > threshold=-0.35980531573295593
Class: COVID-19 Severe	
Rules_773	passed counts:5
node_0: feature_name=LTB	LTB.value > threshold=0.994031548500061
node_5264: feature_name=MT-CO1	MT-CO1.value <= threshold=-0.4127991199493408
node_5265: feature_name=MTRNR2L2	MTRNR2L2.value <= threshold=2.4473838806152344
node_5266: feature_name=TCL1A	TCL1A.value <= threshold=5.787256956100464
node_5267: feature_name=PLD4	PLD4.value <= threshold=3.544657826423645
node_5268: feature_name=NR4A1	NR4A1.value > threshold=0.8490704298019409
node_5498: feature_name=HLA-DPA1	HLA-DPA1.value <= threshold=1.9079211950302124
node_5499: feature_name=ID3	ID3.value <= threshold=-0.17505786567926407
node_5500: feature_name=PPIF	PPIF.value > threshold=-0.5180355906486511
node_5512: feature_name=PDLIM1	PDLIM1.value > threshold=2.5938297510147095
node_5526: feature_name=FTH1	FTH1.value <= threshold=0.17593900486826897
node_5527: feature_name=MIR24-2	MIR24-2.value > threshold=1.0514202117919922
node_5533: feature_name=BCL2A1	BCL2A1.value <= threshold=1.141773760318756
Class: Healthy Control	

Rules_774	passed counts:5
node_0: feature_name=LTB	LTB.value > threshold=0.994031548500061
node_5264: feature_name=MT-CO1	MT-CO1.value <= threshold=-0.4127991199493408
node_5265: feature_name=MTRNR2L2	MTRNR2L2.value <= threshold=2.4473838806152344
node_5266: feature_name=TCL1A	TCL1A.value <= threshold=5.787256956100464
node_5267: feature_name=PLD4	PLD4.value <= threshold=3.544657826423645
node_5268: feature_name=NR4A1	NR4A1.value <= threshold=0.8490704298019409
node_5269: feature_name=LTB	LTB.value <= threshold=1.5252206921577454
node_5270: feature_name=HLA-DRB5	HLA-DRB5.value <= threshold=2.5117164850234985
node_5271: feature_name=CD79A	CD79A.value <= threshold=3.685201406478882
node_5272: feature_name=BCL2A1	BCL2A1.value <= threshold=2.081526756286621
node_5273: feature_name=HLA-DPA1	HLA-DPA1.value > threshold=1.8042166829109192
node_5363: feature_name=MS4A1	MS4A1.value <= threshold=3.5283708572387695
node_5364: feature_name=MT-CO3	MT-CO3.value <= threshold=0.3994715213775635
node_5365: feature_name=TNFAIP3	TNFAIP3.value > threshold=0.2631131559610367
node_5427: feature_name=HSP90B1	HSP90B1.value > threshold=0.9477921724319458
Class: COVID-19 Severe	
Rules_775	passed counts:5
node_0: feature_name=LTB	LTB.value > threshold=0.994031548500061
node_5264: feature_name=MT-CO1	MT-CO1.value <= threshold=-0.4127991199493408
node_5265: feature_name=MTRNR2L2	MTRNR2L2.value <= threshold=2.4473838806152344
node_5266: feature_name=TCL1A	TCL1A.value <= threshold=5.787256956100464
node_5267: feature_name=PLD4	PLD4.value <= threshold=3.544657826423645
node_5268: feature_name=NR4A1	NR4A1.value <= threshold=0.8490704298019409
node_5269: feature_name=LTB	LTB.value <= threshold=1.5252206921577454
node_5270: feature_name=HLA-DRB5	HLA-DRB5.value <= threshold=2.5117164850234985
node_5271: feature_name=CD79A	CD79A.value <= threshold=3.685201406478882
node_5272: feature_name=BCL2A1	BCL2A1.value <= threshold=2.081526756286621
node_5273: feature_name=HLA-DPA1	HLA-DPA1.value > threshold=1.8042166829109192

node_5363: feature_name=MS4A1	MS4A1.value <= threshold=3.5283708572387695
node_5364: feature_name=MT-CO3	MT-CO3.value <= threshold=0.3994715213775635
node_5365: feature_name=TNFAIP3	TNFAIP3.value <= threshold=0.2631131559610367
node_5366: feature_name=MT-CO1	MT-CO1.value <= threshold=-0.44641776382923126
node_5367: feature_name=GPX1	GPX1.value <= threshold=0.7746693789958954
node_5368: feature_name=MMD	MMD.value <= threshold=3.176570177078247
node_5369: feature_name=FERMT3	FERMT3.value > threshold=-0.7207392454147339
node_5373: feature_name=HBB	HBB.value > threshold=-0.42021533846855164
node_5375: feature_name=MT-CYB	MT-CYB.value <= threshold=0.6712252795696259
node_5376: feature_name=IGLL5	IGLL5.value <= threshold=8.24229383468628
node_5377: feature_name=PLA2G12A	PLA2G12A.value <= threshold=3.9736461639404297
node_5378: feature_name=MEF2C	MEF2C.value <= threshold=4.8988196849823
node_5379: feature_name=ATF3	ATF3.value <= threshold=3.2426276206970215
node_5380: feature_name=RELT	RELT.value > threshold=2.1399883031845093
node_5400: feature_name=IGLL5	IGLL5.value > threshold=-0.23810312896966934
Class: COVID-19 Critical	
Rules_776	passed counts:5
node_0: feature_name=LTB	LTB.value > threshold=0.994031548500061
node_5264: feature_name=MT-CO1	MT-CO1.value <= threshold=-0.4127991199493408
node_5265: feature_name=MTRNR2L2	MTRNR2L2.value <= threshold=2.4473838806152344
node_5266: feature_name=TCL1A	TCL1A.value <= threshold=5.787256956100464
node_5267: feature_name=PLD4	PLD4.value <= threshold=3.544657826423645
node_5268: feature_name=NR4A1	NR4A1.value <= threshold=0.8490704298019409
node_5269: feature_name=LTB	LTB.value <= threshold=1.5252206921577454
node_5270: feature_name=HLA-DRB5	HLA-DRB5.value <= threshold=2.5117164850234985
node_5271: feature_name=CD79A	CD79A.value <= threshold=3.685201406478882
node_5272: feature_name=BCL2A1	BCL2A1.value <= threshold=2.081526756286621
node_5273: feature_name=HLA-DPA1	HLA-DPA1.value > threshold=1.8042166829109192
node_5363: feature_name=MS4A1	MS4A1.value <= threshold=3.5283708572387695

node_5364: feature_name=MT-CO3	MT-CO3.value <= threshold=0.3994715213775635
node_5365: feature_name=TNFAIP3	TNFAIP3.value <= threshold=0.2631131559610367
node_5366: feature_name=MT-CO1	MT-CO1.value <= threshold=-0.44641776382923126
node_5367: feature_name=GPX1	GPX1.value <= threshold=0.7746693789958954
node_5368: feature_name=MMD	MMD.value <= threshold=3.176570177078247
node_5369: feature_name=FERMT3	FERMT3.value > threshold=-0.7207392454147339
node_5373: feature_name=HBB	HBB.value > threshold=-0.42021533846855164
node_5375: feature_name=MT-CYB	MT-CYB.value <= threshold=0.6712252795696259
node_5376: feature_name=IGLL5	IGLL5.value <= threshold=8.24229383468628
node_5377: feature_name=PLA2G12A	PLA2G12A.value <= threshold=3.9736461639404297
node_5378: feature_name=MEF2C	MEF2C.value <= threshold=4.8988196849823
node_5379: feature_name=ATF3	ATF3.value <= threshold=3.2426276206970215
node_5380: feature_name=RELT	RELT.value <= threshold=2.1399883031845093
node_5381: feature_name=SLC7A5	SLC7A5.value <= threshold=2.0621291399002075
node_5382: feature_name=TPM1	TPM1.value <= threshold=1.94612605124712
node_5383: feature_name=HLA-DPA1	HLA-DPA1.value > threshold=1.8114917278289795
node_5387: feature_name=EGR1	EGR1.value <= threshold=3.533252716064453
node_5388: feature_name=SNX9	SNX9.value > threshold=3.2163097858428955
node_5390: feature_name=SNX9	SNX9.value > threshold=3.2634530067443848
Class: COVID-19 Critical	
Rules_777	passed counts:5
node_0: feature_name=LTB	LTB.value > threshold=0.994031548500061
node_5264: feature_name=MT-CO1	MT-CO1.value <= threshold=-0.4127991199493408
node_5265: feature_name=MTRNR2L2	MTRNR2L2.value <= threshold=2.4473838806152344
node_5266: feature_name=TCL1A	TCL1A.value <= threshold=5.787256956100464
node_5267: feature_name=PLD4	PLD4.value <= threshold=3.544657826423645
node_5268: feature_name=NR4A1	NR4A1.value <= threshold=0.8490704298019409
node_5269: feature_name=LTB	LTB.value <= threshold=1.5252206921577454
node_5270: feature_name=HLA-DRB5	HLA-DRB5.value <= threshold=2.5117164850234985

node_5271: feature_name=CD79A	CD79A.value <= threshold=3.685201406478882
node_5272: feature_name=BCL2A1	BCL2A1.value <= threshold=2.081526756286621
node_5273: feature_name=HLA-DPA1	HLA-DPA1.value <= threshold=1.8042166829109192
node_5274: feature_name=HSP90B1	HSP90B1.value > threshold=-1.3115270137786865
node_5278: feature_name=OAZ1	OAZ1.value > threshold=1.0802008509635925
node_5350: feature_name=XAF1	XAF1.value <= threshold=-0.4393231123685837
node_5351: feature_name=HLA-DQA1	HLA-DQA1.value <= threshold=2.280219793319702
Class: Healthy Control	
Rules_778	passed counts:5
node_0: feature_name=LTB	LTB.value > threshold=0.994031548500061
node_5264: feature_name=MT-CO1	MT-CO1.value <= threshold=-0.4127991199493408
node_5265: feature_name=MTRNR2L2	MTRNR2L2.value <= threshold=2.4473838806152344
node_5266: feature_name=TCL1A	TCL1A.value <= threshold=5.787256956100464
node_5267: feature_name=PLD4	PLD4.value <= threshold=3.544657826423645
node_5268: feature_name=NR4A1	NR4A1.value <= threshold=0.8490704298019409
node_5269: feature_name=LTB	LTB.value <= threshold=1.5252206921577454
node_5270: feature_name=HLA-DRB5	HLA-DRB5.value <= threshold=2.5117164850234985
node_5271: feature_name=CD79A	CD79A.value <= threshold=3.685201406478882
node_5272: feature_name=BCL2A1	BCL2A1.value <= threshold=2.081526756286621
node_5273: feature_name=HLA-DPA1	HLA-DPA1.value <= threshold=1.8042166829109192
node_5274: feature_name=HSP90B1	HSP90B1.value > threshold=-1.3115270137786865
node_5278: feature_name=OAZ1	OAZ1.value <= threshold=1.0802008509635925
node_5279: feature_name=CD79B	CD79B.value <= threshold=5.875581979751587
node_5280: feature_name=CCDC141	CCDC141.value <= threshold=6.439151048660278
node_5281: feature_name=SYTL3	SYTL3.value <= threshold=1.149337351322174
node_5282: feature_name=GPX1	GPX1.value > threshold=-1.0243326425552368
node_5284: feature_name=MMD	MMD.value <= threshold=6.431635856628418
node_5285: feature_name=MARCKS	MARCKS.value > threshold=4.034798860549927
node_5343: feature_name=ATG2A	ATG2A.value > threshold=-0.3775705397129059

Class: COVID-19 Critical	
Rules_779	passed counts:5
node_0: feature_name=LTB	LTB.value > threshold=0.994031548500061
node_5264: feature_name=MT-CO1	MT-CO1.value <= threshold=-0.4127991199493408
node_5265: feature_name=MTRNR2L2	MTRNR2L2.value <= threshold=2.4473838806152344
node_5266: feature_name=TCL1A	TCL1A.value <= threshold=5.787256956100464
node_5267: feature_name=PLD4	PLD4.value <= threshold=3.544657826423645
node_5268: feature_name=NR4A1	NR4A1.value <= threshold=0.8490704298019409
node_5269: feature_name=LTB	LTB.value <= threshold=1.5252206921577454
node_5270: feature_name=HLA-DRB5	HLA-DRB5.value <= threshold=2.5117164850234985
node_5271: feature_name=CD79A	CD79A.value <= threshold=3.685201406478882
node_5272: feature_name=BCL2A1	BCL2A1.value <= threshold=2.081526756286621
node_5273: feature_name=HLA-DPA1	HLA-DPA1.value <= threshold=1.8042166829109192
node_5274: feature_name=HSP90B1	HSP90B1.value > threshold=-1.3115270137786865
node_5278: feature_name=OAZ1	OAZ1.value <= threshold=1.0802008509635925
node_5279: feature_name=CD79B	CD79B.value <= threshold=5.875581979751587
node_5280: feature_name=CCDC141	CCDC141.value <= threshold=6.439151048660278
node_5281: feature_name=SYTL3	SYTL3.value <= threshold=1.149337351322174
node_5282: feature_name=GPX1	GPX1.value > threshold=-1.0243326425552368
node_5284: feature_name=MMD	MMD.value <= threshold=6.431635856628418
node_5285: feature_name=MARCKS	MARCKS.value <= threshold=4.034798860549927
node_5286: feature_name=XIST	XIST.value <= threshold=1.148451752960682
node_5287: feature_name=CD83	CD83.value <= threshold=2.894522190093994
node_5288: feature_name=MIR24-2	MIR24-2.value <= threshold=2.76771080493927
node_5289: feature_name=GRASP	GRASP.value > threshold=-0.4039058983325958
node_5303: feature_name=CD38	CD38.value <= threshold=3.721052408218384
node_5304: feature_name=ATF5	ATF5.value <= threshold=6.64192533493042
node_5305: feature_name=CD22	CD22.value <= threshold=5.268923759460449
node_5306: feature_name=CASP8	CASP8.value <= threshold=1.738209843635559

node_5307: feature_name=HSP90B1	HSP90B1.value <= threshold=-1.2544044852256775
node_5308: feature_name=RALGPS2	RALGPS2.value <= threshold=3.17460036277771
Class: COVID-19 Critical	
Rules_780	passed counts:5
node_0: feature_name=LTB	LTB.value > threshold=0.994031548500061
node_5264: feature_name=MT-CO1	MT-CO1.value <= threshold=-0.4127991199493408
node_5265: feature_name=MTRNR2L2	MTRNR2L2.value <= threshold=2.4473838806152344
node_5266: feature_name=TCL1A	TCL1A.value <= threshold=5.787256956100464
node_5267: feature_name=PLD4	PLD4.value <= threshold=3.544657826423645
node_5268: feature_name=NR4A1	NR4A1.value <= threshold=0.8490704298019409
node_5269: feature_name=LTB	LTB.value <= threshold=1.5252206921577454
node_5270: feature_name=HLA-DRB5	HLA-DRB5.value <= threshold=2.5117164850234985
node_5271: feature_name=CD79A	CD79A.value <= threshold=3.685201406478882
node_5272: feature_name=BCL2A1	BCL2A1.value <= threshold=2.081526756286621
node_5273: feature_name=HLA-DPA1	HLA-DPA1.value <= threshold=1.8042166829109192
node_5274: feature_name=HSP90B1	HSP90B1.value <= threshold=-1.3115270137786865
node_5275: feature_name=MT-CO1	MT-CO1.value > threshold=-0.5243768393993378
Class: Healthy Control	
Rules_781	passed counts:5
node_0: feature_name=LTB	LTB.value > threshold=0.994031548500061
node_5264: feature_name=MT-CO1	MT-CO1.value <= threshold=-0.4127991199493408
node_5265: feature_name=MTRNR2L2	MTRNR2L2.value <= threshold=2.4473838806152344
node_5266: feature_name=TCL1A	TCL1A.value <= threshold=5.787256956100464
node_5267: feature_name=PLD4	PLD4.value <= threshold=3.544657826423645
node_5268: feature_name=NR4A1	NR4A1.value <= threshold=0.8490704298019409
node_5269: feature_name=LTB	LTB.value <= threshold=1.5252206921577454
node_5270: feature_name=HLA-DRB5	HLA-DRB5.value <= threshold=2.5117164850234985
node_5271: feature_name=CD79A	CD79A.value <= threshold=3.685201406478882

node_5272: feature_name=BCL2A1	BCL2A1.value <= threshold=2.081526756286621
node_5273: feature_name=HLA-DPA1	HLA-DPA1.value <= threshold=1.8042166829109192
node_5274: feature_name=HSP90B1	HSP90B1.value <= threshold=-1.3115270137786865
node_5275: feature_name=MT-CO1	MT-CO1.value <= threshold=-0.5243768393993378
Class: COVID-19 Critical	
Rules_782	passed counts:5
node_0: feature_name=LTB	LTB.value <= threshold=0.994031548500061
node_1: feature_name=MTRNR2L2	MTRNR2L2.value <= threshold=2.8784297704696655
node_2: feature_name=TCL1A	TCL1A.value > threshold=4.798312664031982
node_4912: feature_name=MT-CO1	MT-CO1.value > threshold=-0.3664844036102295
node_5002: feature_name=IFI44L	IFI44L.value <= threshold=2.3101747035980225
node_5003: feature_name=SNX9	SNX9.value > threshold=2.0247421264648438
node_5147: feature_name=CD79A	CD79A.value > threshold=2.554050326347351
node_5169: feature_name=CD79B	CD79B.value > threshold=-0.2480710819363594
node_5185: feature_name=OAZ1	OAZ1.value <= threshold=0.2523931488394737
node_5186: feature_name=PDLIM1	PDLIM1.value <= threshold=2.7835850715637207
node_5187: feature_name=CTD-2035E11.3	CTD-2035E11.3.value <= threshold=-0.26431451737880707
Class: Healthy Control	
Rules_783	passed counts:5
node_0: feature_name=LTB	LTB.value <= threshold=0.994031548500061
node_1: feature_name=MTRNR2L2	MTRNR2L2.value <= threshold=2.8784297704696655
node_2: feature_name=TCL1A	TCL1A.value > threshold=4.798312664031982
node_4912: feature_name=MT-CO1	MT-CO1.value > threshold=-0.3664844036102295
node_5002: feature_name=IFI44L	IFI44L.value <= threshold=2.3101747035980225
node_5003: feature_name=SNX9	SNX9.value <= threshold=2.0247421264648438
node_5004: feature_name=STAG3	STAG3.value <= threshold=6.482141733169556
node_5005: feature_name=BACH1	BACH1.value > threshold=-0.5237349569797516
node_5047: feature_name=RHOB	RHOB.value > threshold=-0.4843696355819702

node_5055: feature_name=FAM129C	FAM129C.value > threshold=-0.2832885980606079
node_5079: feature_name=TP53INP1	TP53INP1.value <= threshold=2.9482468366622925
node_5080: feature_name=HLA-DRB5	HLA-DRB5.value > threshold=2.6279215812683105
node_5124: feature_name=NFKBID	NFKBID.value > threshold=0.6739058941602707
Class: COVID-19 Critical	
Rules_784	passed counts:5
node_0: feature_name=LTB	LTB.value <= threshold=0.994031548500061
node_1: feature_name=MTRNR2L2	MTRNR2L2.value <= threshold=2.8784297704696655
node_2: feature_name=TCL1A	TCL1A.value > threshold=4.798312664031982
node_4912: feature_name=MT-CO1	MT-CO1.value > threshold=-0.3664844036102295
node_5002: feature_name=IFI44L	IFI44L.value <= threshold=2.3101747035980225
node_5003: feature_name=SNX9	SNX9.value <= threshold=2.0247421264648438
node_5004: feature_name=STAG3	STAG3.value <= threshold=6.482141733169556
node_5005: feature_name=BACH1	BACH1.value > threshold=-0.5237349569797516
node_5047: feature_name=RHOB	RHOB.value > threshold=-0.4843696355819702
node_5055: feature_name=FAM129C	FAM129C.value > threshold=-0.2832885980606079
node_5079: feature_name=TP53INP1	TP53INP1.value <= threshold=2.9482468366622925
node_5080: feature_name=HLA-DRB5	HLA-DRB5.value > threshold=2.6279215812683105
node_5124: feature_name=NFKBID	NFKBID.value <= threshold=0.6739058941602707
node_5125: feature_name=RALGPS2	RALGPS2.value <= threshold=3.7552335262298584
Class: Healthy Control	
Rules_785	passed counts:5
node_0: feature_name=LTB	LTB.value <= threshold=0.994031548500061
node_1: feature_name=MTRNR2L2	MTRNR2L2.value <= threshold=2.8784297704696655
node_2: feature_name=TCL1A	TCL1A.value > threshold=4.798312664031982
node_4912: feature_name=MT-CO1	MT-CO1.value > threshold=-0.3664844036102295
node_5002: feature_name=IFI44L	IFI44L.value <= threshold=2.3101747035980225
node_5003: feature_name=SNX9	SNX9.value <= threshold=2.0247421264648438

node_5004: feature_name=STAG3	STAG3.value <= threshold=6.482141733169556
node_5005: feature_name=BACH1	BACH1.value > threshold=-0.5237349569797516
node_5047: feature_name=RHOB	RHOB.value > threshold=-0.4843696355819702
node_5055: feature_name=FAM129C	FAM129C.value <= threshold=-0.2832885980606079
node_5056: feature_name=CD83	CD83.value > threshold=-0.5592367649078369
node_5060: feature_name=OAZ1	OAZ1.value <= threshold=0.7524859011173248
node_5061: feature_name=FCRL1	FCRL1.value > threshold=5.677775859832764
Class: Healthy Control	
Rules_786	passed counts:5
node_0: feature_name=LTB	LTB.value <= threshold=0.994031548500061
node_1: feature_name=MTRNR2L2	MTRNR2L2.value <= threshold=2.8784297704696655
node_2: feature_name=TCL1A	TCL1A.value <= threshold=4.798312664031982
node_3: feature_name=MT-CO1	MT-CO1.value > threshold=0.08947191759943962
node_2521: feature_name=LTB	LTB.value > threshold=0.6038667261600494
node_4173: feature_name=OAZ1	OAZ1.value > threshold=0.143283873796463
node_4615: feature_name=NR4A1	NR4A1.value > threshold=0.6621004343032837
node_4817: feature_name=HSP90B1	HSP90B1.value <= threshold=1.1699737310409546
node_4818: feature_name=XAF1	XAF1.value <= threshold=2.6752071380615234
node_4819: feature_name=SNX9	SNX9.value <= threshold=2.939147114753723
node_4820: feature_name=HLA-DQA2	HLA-DQA2.value <= threshold=2.9822239875793457
node_4821: feature_name=ATF5	ATF5.value > threshold=4.116653323173523
node_4871: feature_name=BANK1	BANK1.value <= threshold=3.1911901235580444
Class: COVID-19 Critical	
Rules_787	passed counts:5
node_0: feature_name=LTB	LTB.value <= threshold=0.994031548500061
node_1: feature_name=MTRNR2L2	MTRNR2L2.value <= threshold=2.8784297704696655
node_2: feature_name=TCL1A	TCL1A.value <= threshold=4.798312664031982
node_3: feature_name=MT-CO1	MT-CO1.value > threshold=0.08947191759943962

node_2521: feature_name=LTB	LTB.value > threshold=0.6038667261600494
node_4173: feature_name=OAZ1	OAZ1.value > threshold=0.143283873796463
node_4615: feature_name=NR4A1	NR4A1.value > threshold=0.6621004343032837
node_4817: feature_name=HSP90B1	HSP90B1.value <= threshold=1.1699737310409546
node_4818: feature_name=XAF1	XAF1.value <= threshold=2.6752071380615234
node_4819: feature_name=SNX9	SNX9.value <= threshold=2.939147114753723
node_4820: feature_name=HLA-DQA2	HLA-DQA2.value <= threshold=2.9822239875793457
node_4821: feature_name=ATF5	ATF5.value <= threshold=4.116653323173523
node_4822: feature_name=RALGPS2	RALGPS2.value <= threshold=-0.3627814054489136
node_4823: feature_name=HIST1H2AC	HIST1H2AC.value > threshold=-0.2637694925069809
node_4831: feature_name=IRAK3	IRAK3.value <= threshold=0.9354141280055046
Class: COVID-19 Critical	
Rules_788	passed counts:5
node_0: feature_name=LTB	LTB.value <= threshold=0.994031548500061
node_1: feature_name=MTRNR2L2	MTRNR2L2.value <= threshold=2.8784297704696655
node_2: feature_name=TCL1A	TCL1A.value <= threshold=4.798312664031982
node_3: feature_name=MT-CO1	MT-CO1.value > threshold=0.08947191759943962
node_2521: feature_name=LTB	LTB.value > threshold=0.6038667261600494
node_4173: feature_name=OAZ1	OAZ1.value > threshold=0.143283873796463
node_4615: feature_name=NR4A1	NR4A1.value <= threshold=0.6621004343032837
node_4616: feature_name=NFKBID	NFKBID.value <= threshold=3.283741593360901
node_4617: feature_name=HLA-DQA1	HLA-DQA1.value > threshold=2.3435752391815186
node_4719: feature_name=MX1	MX1.value <= threshold=1.851993978023529
node_4720: feature_name=JUN	JUN.value > threshold=-0.5025404393672943
node_4740: feature_name=IRF8	IRF8.value <= threshold=3.838419556617737
node_4741: feature_name=BACH1	BACH1.value <= threshold=0.9568033516407013
node_4742: feature_name=HLA-DQA2	HLA-DQA2.value <= threshold=4.834465265274048
node_4743: feature_name=STAG3	STAG3.value <= threshold=6.6092634201049805
node_4744: feature_name=IFIT2	IFIT2.value > threshold=0.700381800532341

node_4762: feature_name=SPI1	SPI1.value > threshold=0.22979680448770523
Class: Healthy Control	
Rules_789	passed counts:5
node_0: feature_name=LTB	LTB.value <= threshold=0.994031548500061
node_1: feature_name=MTRNR2L2	MTRNR2L2.value <= threshold=2.8784297704696655
node_2: feature_name=TCL1A	TCL1A.value <= threshold=4.798312664031982
node_3: feature_name=MT-CO1	MT-CO1.value > threshold=0.08947191759943962
node_2521: feature_name=LTB	LTB.value > threshold=0.6038667261600494
node_4173: feature_name=OAZ1	OAZ1.value > threshold=0.143283873796463
node_4615: feature_name=NR4A1	NR4A1.value <= threshold=0.6621004343032837
node_4616: feature_name=NFKBID	NFKBID.value <= threshold=3.283741593360901
node_4617: feature_name=HLA-DQA1	HLA-DQA1.value > threshold=2.3435752391815186
node_4719: feature_name=MX1	MX1.value <= threshold=1.851993978023529
node_4720: feature_name=JUN	JUN.value > threshold=-0.5025404393672943
node_4740: feature_name=IRF8	IRF8.value <= threshold=3.838419556617737
node_4741: feature_name=BACH1	BACH1.value <= threshold=0.9568033516407013
node_4742: feature_name=HLA-DQA2	HLA-DQA2.value <= threshold=4.834465265274048
node_4743: feature_name=STAG3	STAG3.value <= threshold=6.6092634201049805
node_4744: feature_name=IFIT2	IFIT2.value > threshold=0.700381800532341
node_4762: feature_name=SPI1	SPI1.value <= threshold=0.22979680448770523
node_4763: feature_name=HLA-DPB1	HLA-DPB1.value <= threshold=2.2211217880249023
Class: COVID-19 Critical	
Rules_790	passed counts:5
node_0: feature_name=LTB	LTB.value <= threshold=0.994031548500061
node_1: feature_name=MTRNR2L2	MTRNR2L2.value <= threshold=2.8784297704696655
node_2: feature_name=TCL1A	TCL1A.value <= threshold=4.798312664031982
node_3: feature_name=MT-CO1	MT-CO1.value > threshold=0.08947191759943962
node_2521: feature_name=LTB	LTB.value > threshold=0.6038667261600494

node_4173: feature_name=OAZ1	OAZ1.value > threshold=0.143283873796463
node_4615: feature_name=NR4A1	NR4A1.value <= threshold=0.6621004343032837
node_4616: feature_name=NFKBID	NFKBID.value <= threshold=3.283741593360901
node_4617: feature_name=HLA-DQA1	HLA-DQA1.value <= threshold=2.3435752391815186
node_4618: feature_name=PELI1	PELI1.value <= threshold=1.3271203637123108
node_4619: feature_name=PLD4	PLD4.value <= threshold=3.3385335206985474
node_4620: feature_name=MEF2A	MEF2A.value <= threshold=1.5926940441131592
node_4621: feature_name=DAPP1	DAPP1.value <= threshold=2.647637367248535
node_4622: feature_name=STAG3	STAG3.value > threshold=-0.16521219909191132
node_4648: feature_name=CD22	CD22.value <= threshold=-0.21456258744001389
node_4649: feature_name=FCRL1	FCRL1.value <= threshold=6.079606294631958
Class: Healthy Control	
Rules_791	passed counts:5
node_0: feature_name=LTB	LTB.value <= threshold=0.994031548500061
node_1: feature_name=MTRNR2L2	MTRNR2L2.value <= threshold=2.8784297704696655
node_2: feature_name=TCL1A	TCL1A.value <= threshold=4.798312664031982
node_3: feature_name=MT-CO1	MT-CO1.value > threshold=0.08947191759943962
node_2521: feature_name=LTB	LTB.value > threshold=0.6038667261600494
node_4173: feature_name=OAZ1	OAZ1.value > threshold=0.143283873796463
node_4615: feature_name=NR4A1	NR4A1.value <= threshold=0.6621004343032837
node_4616: feature_name=NFKBID	NFKBID.value <= threshold=3.283741593360901
node_4617: feature_name=HLA-DQA1	HLA-DQA1.value <= threshold=2.3435752391815186
node_4618: feature_name=PELI1	PELI1.value <= threshold=1.3271203637123108
node_4619: feature_name=PLD4	PLD4.value <= threshold=3.3385335206985474
node_4620: feature_name=MEF2A	MEF2A.value <= threshold=1.5926940441131592
node_4621: feature_name=DAPP1	DAPP1.value <= threshold=2.647637367248535
node_4622: feature_name=STAG3	STAG3.value <= threshold=-0.16521219909191132
node_4623: feature_name=OAZ1	OAZ1.value > threshold=0.2042311355471611
node_4625: feature_name=FAM129C	FAM129C.value > threshold=-0.23615043610334396

node_4639: feature_name=TP53INP1	TP53INP1.value <= threshold=4.941076993942261
node_4640: feature_name=KIAA0125	KIAA0125.value <= threshold=7.376631736755371
node_4641: feature_name=SESN3	SESN3.value > threshold=1.000320941209793
node_4643: feature_name=CASP8	CASP8.value <= threshold=-0.5342620015144348
Class: Healthy Control	
Rules_792	passed counts:5
node_0: feature_name=LTB	LTB.value <= threshold=0.994031548500061
node_1: feature_name=MTRNR2L2	MTRNR2L2.value <= threshold=2.8784297704696655
node_2: feature_name=TCL1A	TCL1A.value <= threshold=4.798312664031982
node_3: feature_name=MT-CO1	MT-CO1.value > threshold=0.08947191759943962
node_2521: feature_name=LTB	LTB.value > threshold=0.6038667261600494
node_4173: feature_name=OAZ1	OAZ1.value > threshold=0.143283873796463
node_4615: feature_name=NR4A1	NR4A1.value <= threshold=0.6621004343032837
node_4616: feature_name=NFKBID	NFKBID.value <= threshold=3.283741593360901
node_4617: feature_name=HLA-DQA1	HLA-DQA1.value <= threshold=2.3435752391815186
node_4618: feature_name=PELI1	PELI1.value <= threshold=1.3271203637123108
node_4619: feature_name=PLD4	PLD4.value <= threshold=3.3385335206985474
node_4620: feature_name=MEF2A	MEF2A.value <= threshold=1.5926940441131592
node_4621: feature_name=DAPP1	DAPP1.value <= threshold=2.647637367248535
node_4622: feature_name=STAG3	STAG3.value <= threshold=-0.16521219909191132
node_4623: feature_name=OAZ1	OAZ1.value <= threshold=0.2042311355471611
Class: COVID-19 Critical	
Rules_793	passed counts:5
node_0: feature_name=LTB	LTB.value <= threshold=0.994031548500061
node_1: feature_name=MTRNR2L2	MTRNR2L2.value <= threshold=2.8784297704696655
node_2: feature_name=TCL1A	TCL1A.value <= threshold=4.798312664031982
node_3: feature_name=MT-CO1	MT-CO1.value > threshold=0.08947191759943962
node_2521: feature_name=LTB	LTB.value > threshold=0.6038667261600494

node_4173: feature_name=OAZ1	OAZ1.value <= threshold=0.143283873796463
node_4174: feature_name=PLD4	PLD4.value > threshold=2.921932101249695
node_4558: feature_name=HLA-DQA2	HLA-DQA2.value > threshold=1.5485953092575073
node_4598: feature_name=MTRNR2L8	MTRNR2L8.value > threshold=2.9248507022857666
Class: Healthy Control	
Rules_794	passed counts:5
node_0: feature_name=LTB	LTB.value <= threshold=0.994031548500061
node_1: feature_name=MTRNR2L2	MTRNR2L2.value <= threshold=2.8784297704696655
node_2: feature_name=TCL1A	TCL1A.value <= threshold=4.798312664031982
node_3: feature_name=MT-CO1	MT-CO1.value > threshold=0.08947191759943962
node_2521: feature_name=LTB	LTB.value > threshold=0.6038667261600494
node_4173: feature_name=OAZ1	OAZ1.value <= threshold=0.143283873796463
node_4174: feature_name=PLD4	PLD4.value > threshold=2.921932101249695
node_4558: feature_name=HLA-DQA2	HLA-DQA2.value > threshold=1.5485953092575073
node_4598: feature_name=MTRNR2L8	MTRNR2L8.value <= threshold=2.9248507022857666
node_4599: feature_name=HLA-DRB1	HLA-DRB1.value > threshold=1.4262019395828247
node_4607: feature_name=MT-ND6	MT-ND6.value <= threshold=0.19271118938922882
node_4608: feature_name=LDLRAD4	LDLRAD4.value > threshold=0.916767954826355
Class: COVID-19 Critical	
Rules_795	passed counts:5
node_0: feature_name=LTB	LTB.value <= threshold=0.994031548500061
node_1: feature_name=MTRNR2L2	MTRNR2L2.value <= threshold=2.8784297704696655
node_2: feature_name=TCL1A	TCL1A.value <= threshold=4.798312664031982
node_3: feature_name=MT-CO1	MT-CO1.value > threshold=0.08947191759943962
node_2521: feature_name=LTB	LTB.value > threshold=0.6038667261600494
node_4173: feature_name=OAZ1	OAZ1.value <= threshold=0.143283873796463
node_4174: feature_name=PLD4	PLD4.value > threshold=2.921932101249695
node_4558: feature_name=HLA-DQA2	HLA-DQA2.value <= threshold=1.5485953092575073

node_4559: feature_name=COTL1	COTL1.value > threshold=-0.42844848334789276
node_4573: feature_name=CD83	CD83.value <= threshold=1.9224650263786316
node_4574: feature_name=ICAM1	ICAM1.value > threshold=-0.3564278930425644
node_4586: feature_name=MARCKS	MARCKS.value <= threshold=-0.035114869475364685
node_4587: feature_name=KIAA0125	KIAA0125.value <= threshold=1.9511559009552002
Class: Healthy Control	
Rules_796	passed counts:5
node_0: feature_name=LTB	LTB.value <= threshold=0.994031548500061
node_1: feature_name=MTRNR2L2	MTRNR2L2.value <= threshold=2.8784297704696655
node_2: feature_name=TCL1A	TCL1A.value <= threshold=4.798312664031982
node_3: feature_name=MT-CO1	MT-CO1.value > threshold=0.08947191759943962
node_2521: feature_name=LTB	LTB.value > threshold=0.6038667261600494
node_4173: feature_name=OAZ1	OAZ1.value <= threshold=0.143283873796463
node_4174: feature_name=PLD4	PLD4.value > threshold=2.921932101249695
node_4558: feature_name=HLA-DQA2	HLA-DQA2.value <= threshold=1.5485953092575073
node_4559: feature_name=COTL1	COTL1.value > threshold=-0.42844848334789276
node_4573: feature_name=CD83	CD83.value <= threshold=1.9224650263786316
node_4574: feature_name=ICAM1	ICAM1.value <= threshold=-0.3564278930425644
node_4575: feature_name=MARCKS	MARCKS.value > threshold=2.6073683500289917
node_4583: feature_name=HOPX	HOPX.value <= threshold=-0.45748303830623627
Class: COVID-19 Critical	
Rules_797	passed counts:5
node_0: feature_name=LTB	LTB.value <= threshold=0.994031548500061
node_1: feature_name=MTRNR2L2	MTRNR2L2.value <= threshold=2.8784297704696655
node_2: feature_name=TCL1A	TCL1A.value <= threshold=4.798312664031982
node_3: feature_name=MT-CO1	MT-CO1.value > threshold=0.08947191759943962
node_2521: feature_name=LTB	LTB.value > threshold=0.6038667261600494
node_4173: feature_name=OAZ1	OAZ1.value <= threshold=0.143283873796463

node_4174: feature_name=PLD4	PLD4.value > threshold=2.921932101249695
node_4558: feature_name=HLA-DQA2	HLA-DQA2.value <= threshold=1.5485953092575073
node_4559: feature_name=COTL1	COTL1.value <= threshold=-0.42844848334789276
node_4560: feature_name=VPREB3	VPREB3.value <= threshold=0.7973381504416466
node_4561: feature_name=ZNF10	ZNF10.value > threshold=3.9046400785446167
Class: Healthy Control	
Rules_798	passed counts:5
node_0: feature_name=LTB	LTB.value <= threshold=0.994031548500061
node_1: feature_name=MTRNR2L2	MTRNR2L2.value <= threshold=2.8784297704696655
node_2: feature_name=TCL1A	TCL1A.value <= threshold=4.798312664031982
node_3: feature_name=MT-CO1	MT-CO1.value > threshold=0.08947191759943962
node_2521: feature_name=LTB	LTB.value > threshold=0.6038667261600494
node_4173: feature_name=OAZ1	OAZ1.value <= threshold=0.143283873796463
node_4174: feature_name=PLD4	PLD4.value <= threshold=2.921932101249695
node_4175: feature_name=XIST	XIST.value <= threshold=-0.05067425221204758
node_4176: feature_name=MT-CO1	MT-CO1.value > threshold=1.345594048500061
node_4486: feature_name=RALGPS2	RALGPS2.value > threshold=2.7596691846847534
node_4508: feature_name=SNX9	SNX9.value <= threshold=2.5712826251983643
node_4509: feature_name=NFKBIZ	NFKBIZ.value > threshold=-0.8055458068847656
node_4511: feature_name=MT-CO3	MT-CO3.value <= threshold=0.9913833141326904
node_4512: feature_name=CD79A	CD79A.value <= threshold=2.6927499771118164
Class: COVID-19 Critical	
Rules_799	passed counts:5
node_0: feature_name=LTB	LTB.value <= threshold=0.994031548500061
node_1: feature_name=MTRNR2L2	MTRNR2L2.value <= threshold=2.8784297704696655
node_2: feature_name=TCL1A	TCL1A.value <= threshold=4.798312664031982
node_3: feature_name=MT-CO1	MT-CO1.value > threshold=0.08947191759943962
node_2521: feature_name=LTB	LTB.value > threshold=0.6038667261600494

node_4173: feature_name=OAZ1	OAZ1.value <= threshold=0.143283873796463
node_4174: feature_name=PLD4	PLD4.value <= threshold=2.921932101249695
node_4175: feature_name=XIST	XIST.value <= threshold=-0.05067425221204758
node_4176: feature_name=MT-CO1	MT-CO1.value <= threshold=1.345594048500061
node_4177: feature_name=HLA-DQB1	HLA-DQB1.value > threshold=1.927736520767212
node_4353: feature_name=ITM2C	ITM2C.value > threshold=-0.12329206243157387
node_4459: feature_name=SNX9	SNX9.value > threshold=0.6323382556438446
node_4469: feature_name=PELI1	PELI1.value <= threshold=0.4953474849462509
node_4470: feature_name=HSP90B1	HSP90B1.value > threshold=0.24037402868270874
node_4474: feature_name=LTB	LTB.value > threshold=0.7805512845516205
Class: COVID-19 Critical	
Rules_800	passed counts:5
node_0: feature_name=LTB	LTB.value <= threshold=0.994031548500061
node_1: feature_name=MTRNR2L2	MTRNR2L2.value <= threshold=2.8784297704696655
node_2: feature_name=TCL1A	TCL1A.value <= threshold=4.798312664031982
node_3: feature_name=MT-CO1	MT-CO1.value > threshold=0.08947191759943962
node_2521: feature_name=LTB	LTB.value > threshold=0.6038667261600494
node_4173: feature_name=OAZ1	OAZ1.value <= threshold=0.143283873796463
node_4174: feature_name=PLD4	PLD4.value <= threshold=2.921932101249695
node_4175: feature_name=XIST	XIST.value <= threshold=-0.05067425221204758
node_4176: feature_name=MT-CO1	MT-CO1.value <= threshold=1.345594048500061
node_4177: feature_name=HLA-DQB1	HLA-DQB1.value > threshold=1.927736520767212
node_4353: feature_name=ITM2C	ITM2C.value > threshold=-0.12329206243157387
node_4459: feature_name=SNX9	SNX9.value <= threshold=0.6323382556438446
node_4460: feature_name=NR4A1	NR4A1.value <= threshold=0.9784677922725677
node_4461: feature_name=MARCKS	MARCKS.value > threshold=1.4018014669418335
node_4465: feature_name=GPX1	GPX1.value > threshold=-0.7114101648330688
Class: COVID-19 Critical	

Rules_801	passed counts:5
node_0: feature_name=LTB	LTB.value <= threshold=0.994031548500061
node_1: feature_name=MTRNR2L2	MTRNR2L2.value <= threshold=2.8784297704696655
node_2: feature_name=TCL1A	TCL1A.value <= threshold=4.798312664031982
node_3: feature_name=MT-CO1	MT-CO1.value > threshold=0.08947191759943962
node_2521: feature_name=LTB	LTB.value > threshold=0.6038667261600494
node_4173: feature_name=OAZ1	OAZ1.value <= threshold=0.143283873796463
node_4174: feature_name=PLD4	PLD4.value <= threshold=2.921932101249695
node_4175: feature_name=XIST	XIST.value <= threshold=-0.05067425221204758
node_4176: feature_name=MT-CO1	MT-CO1.value <= threshold=1.345594048500061
node_4177: feature_name=HLA-DQB1	HLA-DQB1.value > threshold=1.927736520767212
node_4353: feature_name=ITM2C	ITM2C.value <= threshold=-0.12329206243157387
node_4354: feature_name=MX1	MX1.value <= threshold=-0.4356398284435272
node_4355: feature_name=HLA-DQB1	HLA-DQB1.value <= threshold=2.8865538835525513
node_4356: feature_name=SNX9	SNX9.value > threshold=1.002076417207718
node_4384: feature_name=HLA-DRB5	HLA-DRB5.value <= threshold=1.0937854647636414
node_4385: feature_name=IFI6	IFI6.value <= threshold=0.09842458367347717
node_4386: feature_name=FCRL1	FCRL1.value > threshold=3.549554228782654
Class: COVID-19 Critical	
Rules_802	passed counts:5
node_0: feature_name=LTB	LTB.value <= threshold=0.994031548500061
node_1: feature_name=MTRNR2L2	MTRNR2L2.value <= threshold=2.8784297704696655
node_2: feature_name=TCL1A	TCL1A.value <= threshold=4.798312664031982
node_3: feature_name=MT-CO1	MT-CO1.value > threshold=0.08947191759943962
node_2521: feature_name=LTB	LTB.value > threshold=0.6038667261600494
node_4173: feature_name=OAZ1	OAZ1.value <= threshold=0.143283873796463
node_4174: feature_name=PLD4	PLD4.value <= threshold=2.921932101249695
node_4175: feature_name=XIST	XIST.value <= threshold=-0.05067425221204758
node_4176: feature_name=MT-CO1	MT-CO1.value <= threshold=1.345594048500061

node_4177: feature_name=HLA-DQB1	HLA-DQB1.value <= threshold=1.927736520767212
node_4178: feature_name=BACH1	BACH1.value > threshold=-0.5349778532981873
node_4236: feature_name=TPM1	TPM1.value <= threshold=3.516477584838867
node_4237: feature_name=NR4A1	NR4A1.value > threshold=-0.274935707449913
node_4277: feature_name=HLA-DPA1	HLA-DPA1.value > threshold=1.8894080519676208
node_4339: feature_name=PARP14	PARP14.value > threshold=0.2787386327981949
node_4347: feature_name=S100A6	S100A6.value <= threshold=-1.3354644775390625
node_4348: feature_name=SLC7A5	SLC7A5.value > threshold=-0.6056487262248993
Class: Healthy Control	
Rules_803	passed counts:5
node_0: feature_name=LTB	LTB.value <= threshold=0.994031548500061
node_1: feature_name=MTRNR2L2	MTRNR2L2.value <= threshold=2.8784297704696655
node_2: feature_name=TCL1A	TCL1A.value <= threshold=4.798312664031982
node_3: feature_name=MT-CO1	MT-CO1.value > threshold=0.08947191759943962
node_2521: feature_name=LTB	LTB.value > threshold=0.6038667261600494
node_4173: feature_name=OAZ1	OAZ1.value <= threshold=0.143283873796463
node_4174: feature_name=PLD4	PLD4.value <= threshold=2.921932101249695
node_4175: feature_name=XIST	XIST.value <= threshold=-0.05067425221204758
node_4176: feature_name=MT-CO1	MT-CO1.value <= threshold=1.345594048500061
node_4177: feature_name=HLA-DQB1	HLA-DQB1.value <= threshold=1.927736520767212
node_4178: feature_name=BACH1	BACH1.value > threshold=-0.5349778532981873
node_4236: feature_name=TPM1	TPM1.value <= threshold=3.516477584838867
node_4237: feature_name=NR4A1	NR4A1.value > threshold=-0.274935707449913
node_4277: feature_name=HLA-DPA1	HLA-DPA1.value <= threshold=1.8894080519676208
node_4278: feature_name=TRAF1	TRAF1.value <= threshold=2.091539978981018
node_4279: feature_name=STMN1	STMN1.value <= threshold=2.2198781967163086
node_4280: feature_name=PLD4	PLD4.value <= threshold=2.7647364139556885
node_4281: feature_name=IGJ	IGJ.value <= threshold=4.225788354873657
node_4282: feature_name=MEF2C	MEF2C.value > threshold=3.573865532875061

node_4328: feature_name=HLA-DPB1	HLA-DPB1.value > threshold=1.5301921963691711
Class: COVID-19 Critical	
Rules_804	passed counts:5
node_0: feature_name=LTB	LTB.value <= threshold=0.994031548500061
node_1: feature_name=MTRNR2L2	MTRNR2L2.value <= threshold=2.8784297704696655
node_2: feature_name=TCL1A	TCL1A.value <= threshold=4.798312664031982
node_3: feature_name=MT-CO1	MT-CO1.value > threshold=0.08947191759943962
node_2521: feature_name=LTB	LTB.value > threshold=0.6038667261600494
node_4173: feature_name=OAZ1	OAZ1.value <= threshold=0.143283873796463
node_4174: feature_name=PLD4	PLD4.value <= threshold=2.921932101249695
node_4175: feature_name=XIST	XIST.value <= threshold=-0.05067425221204758
node_4176: feature_name=MT-CO1	MT-CO1.value <= threshold=1.345594048500061
node_4177: feature_name=HLA-DQB1	HLA-DQB1.value <= threshold=1.927736520767212
node_4178: feature_name=BACH1	BACH1.value > threshold=-0.5349778532981873
node_4236: feature_name=TPM1	TPM1.value <= threshold=3.516477584838867
node_4237: feature_name=NR4A1	NR4A1.value > threshold=-0.274935707449913
node_4277: feature_name=HLA-DPA1	HLA-DPA1.value <= threshold=1.8894080519676208
node_4278: feature_name=TRAF1	TRAF1.value <= threshold=2.091539978981018
node_4279: feature_name=STMN1	STMN1.value <= threshold=2.2198781967163086
node_4280: feature_name=PLD4	PLD4.value <= threshold=2.7647364139556885
node_4281: feature_name=IGJ	IGJ.value <= threshold=4.225788354873657
node_4282: feature_name=MEF2C	MEF2C.value <= threshold=3.573865532875061
node_4283: feature_name=ILK	ILK.value <= threshold=3.675175428390503
node_4284: feature_name=MT-CO3	MT-CO3.value > threshold=-0.7448426485061646
node_4288: feature_name=TCF4	TCF4.value > threshold=3.9125311374664307
node_4310: feature_name=BOD1L1	BOD1L1.value <= threshold=-0.4781903773546219
node_4311: feature_name=RP11-731F5.2	RP11-731F5.2.value > threshold=-0.1919102445244789
Class: Healthy Control	

Rules_805	passed counts:5
node_0: feature_name=LTB	LTB.value <= threshold=0.994031548500061
node_1: feature_name=MTRNR2L2	MTRNR2L2.value <= threshold=2.8784297704696655
node_2: feature_name=TCL1A	TCL1A.value <= threshold=4.798312664031982
node_3: feature_name=MT-CO1	MT-CO1.value > threshold=0.08947191759943962
node_2521: feature_name=LTB	LTB.value > threshold=0.6038667261600494
node_4173: feature_name=OAZ1	OAZ1.value <= threshold=0.143283873796463
node_4174: feature_name=PLD4	PLD4.value <= threshold=2.921932101249695
node_4175: feature_name=XIST	XIST.value <= threshold=-0.05067425221204758
node_4176: feature_name=MT-CO1	MT-CO1.value <= threshold=1.345594048500061
node_4177: feature_name=HLA-DQB1	HLA-DQB1.value <= threshold=1.927736520767212
node_4178: feature_name=BACH1	BACH1.value > threshold=-0.5349778532981873
node_4236: feature_name=TPM1	TPM1.value <= threshold=3.516477584838867
node_4237: feature_name=NR4A1	NR4A1.value <= threshold=-0.274935707449913
node_4238: feature_name=VPREB3	VPREB3.value > threshold=5.024149179458618
node_4270: feature_name=IRF8	IRF8.value <= threshold=0.23405013978481293
node_4271: feature_name=TNF	TNF.value <= threshold=-0.39863887429237366
Class: Healthy Control	
Rules_806	passed counts:5
node_0: feature_name=LTB	LTB.value <= threshold=0.994031548500061
node_1: feature_name=MTRNR2L2	MTRNR2L2.value <= threshold=2.8784297704696655
node_2: feature_name=TCL1A	TCL1A.value <= threshold=4.798312664031982
node_3: feature_name=MT-CO1	MT-CO1.value > threshold=0.08947191759943962
node_2521: feature_name=LTB	LTB.value > threshold=0.6038667261600494
node_4173: feature_name=OAZ1	OAZ1.value <= threshold=0.143283873796463
node_4174: feature_name=PLD4	PLD4.value <= threshold=2.921932101249695
node_4175: feature_name=XIST	XIST.value <= threshold=-0.05067425221204758
node_4176: feature_name=MT-CO1	MT-CO1.value <= threshold=1.345594048500061
node_4177: feature_name=HLA-DQB1	HLA-DQB1.value <= threshold=1.927736520767212

node_4178: feature_name=BACH1	BACH1.value <= threshold=-0.5349778532981873
node_4179: feature_name=CD79B	CD79B.value > threshold=1.6518195271492004
node_4201: feature_name=MT-CO2	MT-CO2.value > threshold=0.5007440596818924
node_4223: feature_name=FAM129C	FAM129C.value > threshold=4.054540395736694
Class: Healthy Control	
Rules_807	passed counts:5
node_0: feature_name=LTB	LTB.value <= threshold=0.994031548500061
node_1: feature_name=MTRNR2L2	MTRNR2L2.value <= threshold=2.8784297704696655
node_2: feature_name=TCL1A	TCL1A.value <= threshold=4.798312664031982
node_3: feature_name=MT-CO1	MT-CO1.value > threshold=0.08947191759943962
node_2521: feature_name=LTB	LTB.value > threshold=0.6038667261600494
node_4173: feature_name=OAZ1	OAZ1.value <= threshold=0.143283873796463
node_4174: feature_name=PLD4	PLD4.value <= threshold=2.921932101249695
node_4175: feature_name=XIST	XIST.value <= threshold=-0.05067425221204758
node_4176: feature_name=MT-CO1	MT-CO1.value <= threshold=1.345594048500061
node_4177: feature_name=HLA-DQB1	HLA-DQB1.value <= threshold=1.927736520767212
node_4178: feature_name=BACH1	BACH1.value <= threshold=-0.5349778532981873
node_4179: feature_name=CD79B	CD79B.value <= threshold=1.6518195271492004
node_4180: feature_name=FTH1	FTH1.value > threshold=-1.4486562609672546
node_4184: feature_name=FAM160B1	FAM160B1.value <= threshold=1.850276529788971
node_4185: feature_name=HSPA1A	HSPA1A.value <= threshold=1.0704439878463745
node_4186: feature_name=NR4A1	NR4A1.value > threshold=1.1393861174583435
node_4192: feature_name=HLA-DPA1	HLA-DPA1.value > threshold=1.9337060451507568
Class: Healthy Control	
Rules_808	passed counts:5
node_0: feature_name=LTB	LTB.value <= threshold=0.994031548500061
node_1: feature_name=MTRNR2L2	MTRNR2L2.value <= threshold=2.8784297704696655
node_2: feature_name=TCL1A	TCL1A.value <= threshold=4.798312664031982

node_3: feature_name=MT-CO1	MT-CO1.value > threshold=0.08947191759943962
node_2521: feature_name=LTB	LTB.value <= threshold=0.6038667261600494
node_2522: feature_name=HLA-DQB1	HLA-DQB1.value > threshold=2.1053054332733154
node_3550: feature_name=XIST	XIST.value <= threshold=0.7716337237507105
node_3551: feature_name=CD79B	CD79B.value > threshold=2.9426796436309814
node_4039: feature_name=IFI44L	IFI44L.value <= threshold=1.8637521862983704
node_4040: feature_name=SNX9	SNX9.value > threshold=1.274232804775238
node_4096: feature_name=RALGPS2	RALGPS2.value <= threshold=2.3599878549575806
node_4097: feature_name=HLA-DQB1	HLA-DQB1.value <= threshold=2.9625227451324463
node_4098: feature_name=CD79A	CD79A.value <= threshold=2.383400559425354
node_4099: feature_name=CXorf57	CXorf57.value > threshold=-0.2232322245836258
node_4105: feature_name=HLA-DQA1	HLA-DQA1.value <= threshold=2.4148895740509033
Class: COVID-19 Severe	
Rules_809	passed counts:5
node_0: feature_name=LTB	LTB.value <= threshold=0.994031548500061
node_1: feature_name=MTRNR2L2	MTRNR2L2.value <= threshold=2.8784297704696655
node_2: feature_name=TCL1A	TCL1A.value <= threshold=4.798312664031982
node_3: feature_name=MT-CO1	MT-CO1.value > threshold=0.08947191759943962
node_2521: feature_name=LTB	LTB.value <= threshold=0.6038667261600494
node_2522: feature_name=HLA-DQB1	HLA-DQB1.value > threshold=2.1053054332733154
node_3550: feature_name=XIST	XIST.value <= threshold=0.7716337237507105
node_3551: feature_name=CD79B	CD79B.value <= threshold=2.9426796436309814
node_3552: feature_name=HLA-DQB1	HLA-DQB1.value > threshold=2.713674306869507
node_3854: feature_name=MEF2C	MEF2C.value > threshold=-0.3721291869878769
node_3882: feature_name=IFI44L	IFI44L.value <= threshold=2.5516022443771362
node_3883: feature_name=FCER2	FCER2.value > threshold=-0.21487552672624588
node_3977: feature_name=IGJ	IGJ.value <= threshold=2.123470664024353
node_3978: feature_name=S100A6	S100A6.value > threshold=-1.7107264399528503
node_3982: feature_name=MT-CO1	MT-CO1.value <= threshold=1.5725501775741577

node_3983: feature_name=SNX29P2	SNX29P2.value <= threshold=3.1593780517578125
node_3984: feature_name=IL8	IL8.value <= threshold=-0.28026726841926575
node_3985: feature_name=NAMPT	NAMPT.value > threshold=-0.5902779400348663
node_3991: feature_name=PELI1	PELI1.value <= threshold=3.5653313398361206
node_3992: feature_name=IRF8	IRF8.value <= threshold=-0.34819670021533966
node_3993: feature_name=NFKBID	NFKBID.value <= threshold=1.719421625137329
node_3994: feature_name=OAZ1	OAZ1.value > threshold=-0.3672443702816963
Class: Healthy Control	
Rules_810	passed counts:5
node_0: feature_name=LTB	LTB.value <= threshold=0.994031548500061
node_1: feature_name=MTRNR2L2	MTRNR2L2.value <= threshold=2.8784297704696655
node_2: feature_name=TCL1A	TCL1A.value <= threshold=4.798312664031982
node_3: feature_name=MT-CO1	MT-CO1.value > threshold=0.08947191759943962
node_2521: feature_name=LTB	LTB.value <= threshold=0.6038667261600494
node_2522: feature_name=HLA-DQB1	HLA-DQB1.value > threshold=2.1053054332733154
node_3550: feature_name=XIST	XIST.value <= threshold=0.7716337237507105
node_3551: feature_name=CD79B	CD79B.value <= threshold=2.9426796436309814
node_3552: feature_name=HLA-DQB1	HLA-DQB1.value > threshold=2.713674306869507
node_3854: feature_name=MEF2C	MEF2C.value > threshold=-0.3721291869878769
node_3882: feature_name=IFI44L	IFI44L.value <= threshold=2.5516022443771362
node_3883: feature_name=FCER2	FCER2.value > threshold=-0.21487552672624588
node_3977: feature_name=IGJ	IGJ.value <= threshold=2.123470664024353
node_3978: feature_name=S100A6	S100A6.value > threshold=-1.7107264399528503
node_3982: feature_name=MT-CO1	MT-CO1.value <= threshold=1.5725501775741577
node_3983: feature_name=SNX29P2	SNX29P2.value <= threshold=3.1593780517578125
node_3984: feature_name=IL8	IL8.value <= threshold=-0.28026726841926575
node_3985: feature_name=NAMPT	NAMPT.value <= threshold=-0.5902779400348663
node_3986: feature_name=HLA-DQA1	HLA-DQA1.value <= threshold=2.868778705596924
node_3987: feature_name=HLA-DQA2	HLA-DQA2.value <= threshold=0.8097359836101532

Class: COVID-19 Severe	
Rules_811	passed counts:5
node_0: feature_name=LTB	LTB.value <= threshold=0.994031548500061
node_1: feature_name=MTRNR2L2	MTRNR2L2.value <= threshold=2.8784297704696655
node_2: feature_name=TCL1A	TCL1A.value <= threshold=4.798312664031982
node_3: feature_name=MT-CO1	MT-CO1.value > threshold=0.08947191759943962
node_2521: feature_name=LTB	LTB.value <= threshold=0.6038667261600494
node_2522: feature_name=HLA-DQB1	HLA-DQB1.value > threshold=2.1053054332733154
node_3550: feature_name=XIST	XIST.value <= threshold=0.7716337237507105
node_3551: feature_name=CD79B	CD79B.value <= threshold=2.9426796436309814
node_3552: feature_name=HLA-DQB1	HLA-DQB1.value > threshold=2.713674306869507
node_3854: feature_name=MEF2C	MEF2C.value > threshold=-0.3721291869878769
node_3882: feature_name=IFI44L	IFI44L.value <= threshold=2.5516022443771362
node_3883: feature_name=FCER2	FCER2.value <= threshold=-0.21487552672624588
node_3884: feature_name=HLA-DQA2	HLA-DQA2.value > threshold=-0.12580305337905884
node_3954: feature_name=SNX9	SNX9.value <= threshold=-0.5673478543758392
node_3955: feature_name=CD83	CD83.value > threshold=2.9509599208831787
node_3961: feature_name=IGJ	IGJ.value > threshold=-0.25829608738422394
Class: COVID-19 Severe	
Rules_812	passed counts:5
node_0: feature_name=LTB	LTB.value <= threshold=0.994031548500061
node_1: feature_name=MTRNR2L2	MTRNR2L2.value <= threshold=2.8784297704696655
node_2: feature_name=TCL1A	TCL1A.value <= threshold=4.798312664031982
node_3: feature_name=MT-CO1	MT-CO1.value > threshold=0.08947191759943962
node_2521: feature_name=LTB	LTB.value <= threshold=0.6038667261600494
node_2522: feature_name=HLA-DQB1	HLA-DQB1.value > threshold=2.1053054332733154
node_3550: feature_name=XIST	XIST.value <= threshold=0.7716337237507105
node_3551: feature_name=CD79B	CD79B.value <= threshold=2.9426796436309814

node_3552: feature_name=HLA-DQB1	HLA-DQB1.value > threshold=2.713674306869507
node_3854: feature_name=MEF2C	MEF2C.value <= threshold=-0.3721291869878769
node_3855: feature_name=HLA-DRB5	HLA-DRB5.value > threshold=0.7929701805114746
node_3859: feature_name=LTB	LTB.value > threshold=-0.9689712226390839
node_3863: feature_name=RP11-731F5.2	RP11-731F5.2.value > threshold=3.578703761100769
node_3879: feature_name=BOD1L1	BOD1L1.value <= threshold=0.6120122522115707
Class: Healthy Control	
Rules_813	passed counts:5
node_0: feature_name=LTB	LTB.value <= threshold=0.994031548500061
node_1: feature_name=MTRNR2L2	MTRNR2L2.value <= threshold=2.8784297704696655
node_2: feature_name=TCL1A	TCL1A.value <= threshold=4.798312664031982
node_3: feature_name=MT-CO1	MT-CO1.value > threshold=0.08947191759943962
node_2521: feature_name=LTB	LTB.value <= threshold=0.6038667261600494
node_2522: feature_name=HLA-DQB1	HLA-DQB1.value > threshold=2.1053054332733154
node_3550: feature_name=XIST	XIST.value <= threshold=0.7716337237507105
node_3551: feature_name=CD79B	CD79B.value <= threshold=2.9426796436309814
node_3552: feature_name=HLA-DQB1	HLA-DQB1.value <= threshold=2.713674306869507
node_3553: feature_name=SNX9	SNX9.value > threshold=-0.5404067635536194
node_3653: feature_name=LTB	LTB.value > threshold=0.021708179265260696
node_3759: feature_name=TCL1A	TCL1A.value > threshold=3.3921085596084595
node_3841: feature_name=NFKBIZ	NFKBIZ.value > threshold=0.3082800954580307
node_3849: feature_name=HLA-DQA2	HLA-DQA2.value > threshold=-0.24445389211177826
node_3851: feature_name=FCRL1	FCRL1.value <= threshold=6.296805143356323
Class: COVID-19 Critical	
Rules_814	passed counts:5
node_0: feature_name=LTB	LTB.value <= threshold=0.994031548500061
node_1: feature_name=MTRNR2L2	MTRNR2L2.value <= threshold=2.8784297704696655
node_2: feature_name=TCL1A	TCL1A.value <= threshold=4.798312664031982

node_3: feature_name=MT-CO1	MT-CO1.value > threshold=0.08947191759943962
node_2521: feature_name=LTB	LTB.value <= threshold=0.6038667261600494
node_2522: feature_name=HLA-DQB1	HLA-DQB1.value > threshold=2.1053054332733154
node_3550: feature_name=XIST	XIST.value <= threshold=0.7716337237507105
node_3551: feature_name=CD79B	CD79B.value <= threshold=2.9426796436309814
node_3552: feature_name=HLA-DQB1	HLA-DQB1.value <= threshold=2.713674306869507
node_3553: feature_name=SNX9	SNX9.value > threshold=-0.5404067635536194
node_3653: feature_name=LTB	LTB.value > threshold=0.021708179265260696
node_3759: feature_name=TCL1A	TCL1A.value <= threshold=3.3921085596084595
node_3760: feature_name=HLA-DRB5	HLA-DRB5.value > threshold=0.5265218019485474
node_3776: feature_name=ID1	ID1.value <= threshold=-0.21235637366771698
node_3777: feature_name=RP11-731F5.2	RP11-731F5.2.value > threshold=2.830894112586975
node_3817: feature_name=BOD1L1	BOD1L1.value <= threshold=-0.4770502895116806
node_3818: feature_name=APP	APP.value <= threshold=-0.40902893245220184
node_3819: feature_name=MT-CO2	MT-CO2.value <= threshold=0.24372443556785583
Class: COVID-19 Critical	
Rules_815	passed counts:5
node_0: feature_name=LTB	LTB.value <= threshold=0.994031548500061
node_1: feature_name=MTRNR2L2	MTRNR2L2.value <= threshold=2.8784297704696655
node_2: feature_name=TCL1A	TCL1A.value <= threshold=4.798312664031982
node_3: feature_name=MT-CO1	MT-CO1.value > threshold=0.08947191759943962
node_2521: feature_name=LTB	LTB.value <= threshold=0.6038667261600494
node_2522: feature_name=HLA-DQB1	HLA-DQB1.value > threshold=2.1053054332733154
node_3550: feature_name=XIST	XIST.value <= threshold=0.7716337237507105
node_3551: feature_name=CD79B	CD79B.value <= threshold=2.9426796436309814
node_3552: feature_name=HLA-DQB1	HLA-DQB1.value <= threshold=2.713674306869507
node_3553: feature_name=SNX9	SNX9.value > threshold=-0.5404067635536194
node_3653: feature_name=LTB	LTB.value > threshold=0.021708179265260696
node_3759: feature_name=TCL1A	TCL1A.value <= threshold=3.3921085596084595

node_3760: feature_name=HLA-DRB5	HLA-DRB5.value > threshold=0.5265218019485474
node_3776: feature_name=ID1	ID1.value <= threshold=-0.21235637366771698
node_3777: feature_name=RP11-731F5.2	RP11-731F5.2.value <= threshold=2.830894112586975
node_3778: feature_name=CASP8	CASP8.value <= threshold=2.222941279411316
node_3779: feature_name=MT-CO3	MT-CO3.value <= threshold=-0.41117167472839355
node_3780: feature_name=HLA-DRB5	HLA-DRB5.value > threshold=1.6230974197387695
Class: COVID-19 Severe	
Rules_816	passed counts:5
node_0: feature_name=LTB	LTB.value <= threshold=0.994031548500061
node_1: feature_name=MTRNR2L2	MTRNR2L2.value <= threshold=2.8784297704696655
node_2: feature_name=TCL1A	TCL1A.value <= threshold=4.798312664031982
node_3: feature_name=MT-CO1	MT-CO1.value > threshold=0.08947191759943962
node_2521: feature_name=LTB	LTB.value <= threshold=0.6038667261600494
node_2522: feature_name=HLA-DQB1	HLA-DQB1.value > threshold=2.1053054332733154
node_3550: feature_name=XIST	XIST.value <= threshold=0.7716337237507105
node_3551: feature_name=CD79B	CD79B.value <= threshold=2.9426796436309814
node_3552: feature_name=HLA-DQB1	HLA-DQB1.value <= threshold=2.713674306869507
node_3553: feature_name=SNX9	SNX9.value > threshold=-0.5404067635536194
node_3653: feature_name=LTB	LTB.value > threshold=0.021708179265260696
node_3759: feature_name=TCL1A	TCL1A.value <= threshold=3.3921085596084595
node_3760: feature_name=HLA-DRB5	HLA-DRB5.value > threshold=0.5265218019485474
node_3776: feature_name=ID1	ID1.value <= threshold=-0.21235637366771698
node_3777: feature_name=RP11-731F5.2	RP11-731F5.2.value <= threshold=2.830894112586975
node_3778: feature_name=CASP8	CASP8.value <= threshold=2.222941279411316
node_3779: feature_name=MT-CO3	MT-CO3.value <= threshold=-0.41117167472839355
node_3780: feature_name=HLA-DRB5	HLA-DRB5.value <= threshold=1.6230974197387695
node_3781: feature_name=FCER2	FCER2.value <= threshold=4.076342940330505
Class: COVID-19 Critical	

Rules_817	passed counts:5
node_0: feature_name=LTB	LTB.value <= threshold=0.994031548500061
node_1: feature_name=MTRNR2L2	MTRNR2L2.value <= threshold=2.8784297704696655
node_2: feature_name=TCL1A	TCL1A.value <= threshold=4.798312664031982
node_3: feature_name=MT-CO1	MT-CO1.value > threshold=0.08947191759943962
node_2521: feature_name=LTB	LTB.value <= threshold=0.6038667261600494
node_2522: feature_name=HLA-DQB1	HLA-DQB1.value > threshold=2.1053054332733154
node_3550: feature_name=XIST	XIST.value <= threshold=0.7716337237507105
node_3551: feature_name=CD79B	CD79B.value <= threshold=2.9426796436309814
node_3552: feature_name=HLA-DQB1	HLA-DQB1.value <= threshold=2.713674306869507
node_3553: feature_name=SNX9	SNX9.value > threshold=-0.5404067635536194
node_3653: feature_name=LTB	LTB.value > threshold=0.021708179265260696
node_3759: feature_name=TCL1A	TCL1A.value <= threshold=3.3921085596084595
node_3760: feature_name=HLA-DRB5	HLA-DRB5.value <= threshold=0.5265218019485474
node_3761: feature_name=ATF5	ATF5.value > threshold=-0.32008348405361176
node_3769: feature_name=CD83	CD83.value > threshold=3.0822256803512573
node_3773: feature_name=JARID2	JARID2.value > threshold=-0.45200757682323456
Class: COVID-19 Severe	
Rules_818	passed counts:5
node_0: feature_name=LTB	LTB.value <= threshold=0.994031548500061
node_1: feature_name=MTRNR2L2	MTRNR2L2.value <= threshold=2.8784297704696655
node_2: feature_name=TCL1A	TCL1A.value <= threshold=4.798312664031982
node_3: feature_name=MT-CO1	MT-CO1.value > threshold=0.08947191759943962
node_2521: feature_name=LTB	LTB.value <= threshold=0.6038667261600494
node_2522: feature_name=HLA-DQB1	HLA-DQB1.value > threshold=2.1053054332733154
node_3550: feature_name=XIST	XIST.value <= threshold=0.7716337237507105
node_3551: feature_name=CD79B	CD79B.value <= threshold=2.9426796436309814
node_3552: feature_name=HLA-DQB1	HLA-DQB1.value <= threshold=2.713674306869507
node_3553: feature_name=SNX9	SNX9.value > threshold=-0.5404067635536194

node_3653: feature_name=LTB	LTB.value > threshold=0.021708179265260696
node_3759: feature_name=TCL1A	TCL1A.value <= threshold=3.3921085596084595
node_3760: feature_name=HLA-DRB5	HLA-DRB5.value <= threshold=0.5265218019485474
node_3761: feature_name=ATF5	ATF5.value <= threshold=-0.32008348405361176
node_3762: feature_name=MX1	MX1.value > threshold=0.7805783450603485
node_3766: feature_name=CCDC141	CCDC141.value <= threshold=-0.1554054617881775
Class: COVID-19 Critical	
Rules_819	passed counts:5
node_0: feature_name=LTB	LTB.value <= threshold=0.994031548500061
node_1: feature_name=MTRNR2L2	MTRNR2L2.value <= threshold=2.8784297704696655
node_2: feature_name=TCL1A	TCL1A.value <= threshold=4.798312664031982
node_3: feature_name=MT-CO1	MT-CO1.value > threshold=0.08947191759943962
node_2521: feature_name=LTB	LTB.value <= threshold=0.6038667261600494
node_2522: feature_name=HLA-DQB1	HLA-DQB1.value > threshold=2.1053054332733154
node_3550: feature_name=XIST	XIST.value <= threshold=0.7716337237507105
node_3551: feature_name=CD79B	CD79B.value <= threshold=2.9426796436309814
node_3552: feature_name=HLA-DQB1	HLA-DQB1.value <= threshold=2.713674306869507
node_3553: feature_name=SNX9	SNX9.value > threshold=-0.5404067635536194
node_3653: feature_name=LTB	LTB.value <= threshold=0.021708179265260696
node_3654: feature_name=HLA-DRB5	HLA-DRB5.value > threshold=-0.5633223652839661
node_3660: feature_name=NR4A1	NR4A1.value > threshold=2.2327845096588135
node_3736: feature_name=NFKBID	NFKBID.value <= threshold=-0.4191274344921112
Class: Healthy Control	
Rules_820	passed counts:5
node_0: feature_name=LTB	LTB.value <= threshold=0.994031548500061
node_1: feature_name=MTRNR2L2	MTRNR2L2.value <= threshold=2.8784297704696655
node_2: feature_name=TCL1A	TCL1A.value <= threshold=4.798312664031982
node_3: feature_name=MT-CO1	MT-CO1.value > threshold=0.08947191759943962

node_2521: feature_name=LTB	LTB.value <= threshold=0.6038667261600494
node_2522: feature_name=HLA-DQB1	HLA-DQB1.value > threshold=2.1053054332733154
node_3550: feature_name=XIST	XIST.value <= threshold=0.7716337237507105
node_3551: feature_name=CD79B	CD79B.value <= threshold=2.9426796436309814
node_3552: feature_name=HLA-DQB1	HLA-DQB1.value <= threshold=2.713674306869507
node_3553: feature_name=SNX9	SNX9.value > threshold=-0.5404067635536194
node_3653: feature_name=LTB	LTB.value <= threshold=0.021708179265260696
node_3654: feature_name=HLA-DRB5	HLA-DRB5.value > threshold=-0.5633223652839661
node_3660: feature_name=NR4A1	NR4A1.value <= threshold=2.2327845096588135
node_3661: feature_name=FCRL1	FCRL1.value <= threshold=8.662389755249023
node_3662: feature_name=BANK1	BANK1.value <= threshold=4.776419401168823
node_3663: feature_name=IGLL5	IGLL5.value > threshold=1.4806077480316162
node_3705: feature_name=NR4A1	NR4A1.value > threshold=1.2165279388427734
node_3721: feature_name=FCRL1	FCRL1.value <= threshold=-0.18543093651533127
Class: COVID-19 Critical	
Rules_821	passed counts:5
node_0: feature_name=LTB	LTB.value <= threshold=0.994031548500061
node_1: feature_name=MTRNR2L2	MTRNR2L2.value <= threshold=2.8784297704696655
node_2: feature_name=TCL1A	TCL1A.value <= threshold=4.798312664031982
node_3: feature_name=MT-CO1	MT-CO1.value > threshold=0.08947191759943962
node_2521: feature_name=LTB	LTB.value <= threshold=0.6038667261600494
node_2522: feature_name=HLA-DQB1	HLA-DQB1.value > threshold=2.1053054332733154
node_3550: feature_name=XIST	XIST.value <= threshold=0.7716337237507105
node_3551: feature_name=CD79B	CD79B.value <= threshold=2.9426796436309814
node_3552: feature_name=HLA-DQB1	HLA-DQB1.value <= threshold=2.713674306869507
node_3553: feature_name=SNX9	SNX9.value > threshold=-0.5404067635536194
node_3653: feature_name=LTB	LTB.value <= threshold=0.021708179265260696
node_3654: feature_name=HLA-DRB5	HLA-DRB5.value <= threshold=-0.5633223652839661
node_3655: feature_name=BOD1L1	BOD1L1.value > threshold=-0.4774012118577957

Class: COVID-19 Severe	
Rules_822	passed counts:5
node_0: feature_name=LTB	LTB.value <= threshold=0.994031548500061
node_1: feature_name=MTRNR2L2	MTRNR2L2.value <= threshold=2.8784297704696655
node_2: feature_name=TCL1A	TCL1A.value <= threshold=4.798312664031982
node_3: feature_name=MT-CO1	MT-CO1.value > threshold=0.08947191759943962
node_2521: feature_name=LTB	LTB.value <= threshold=0.6038667261600494
node_2522: feature_name=HLA-DQB1	HLA-DQB1.value > threshold=2.1053054332733154
node_3550: feature_name=XIST	XIST.value <= threshold=0.7716337237507105
node_3551: feature_name=CD79B	CD79B.value <= threshold=2.9426796436309814
node_3552: feature_name=HLA-DQB1	HLA-DQB1.value <= threshold=2.713674306869507
node_3553: feature_name=SNX9	SNX9.value > threshold=-0.5404067635536194
node_3653: feature_name=LTB	LTB.value <= threshold=0.021708179265260696
node_3654: feature_name=HLA-DRB5	HLA-DRB5.value <= threshold=-0.5633223652839661
node_3655: feature_name=BOD1L1	BOD1L1.value <= threshold=-0.4774012118577957
node_3656: feature_name=PELI1	PELI1.value > threshold=0.038211315870285034
Class: COVID-19 Critical	
Rules_823	passed counts:5
node_0: feature_name=LTB	LTB.value <= threshold=0.994031548500061
node_1: feature_name=MTRNR2L2	MTRNR2L2.value <= threshold=2.8784297704696655
node_2: feature_name=TCL1A	TCL1A.value <= threshold=4.798312664031982
node_3: feature_name=MT-CO1	MT-CO1.value > threshold=0.08947191759943962
node_2521: feature_name=LTB	LTB.value <= threshold=0.6038667261600494
node_2522: feature_name=HLA-DQB1	HLA-DQB1.value > threshold=2.1053054332733154
node_3550: feature_name=XIST	XIST.value <= threshold=0.7716337237507105
node_3551: feature_name=CD79B	CD79B.value <= threshold=2.9426796436309814
node_3552: feature_name=HLA-DQB1	HLA-DQB1.value <= threshold=2.713674306869507
node_3553: feature_name=SNX9	SNX9.value <= threshold=-0.5404067635536194

node_3554: feature_name=VPREB3	VPREB3.value <= threshold=4.4475414752960205
node_3555: feature_name=RALGPS2	RALGPS2.value > threshold=2.7017585039138794
node_3609: feature_name=MX1	MX1.value <= threshold=1.5204657316207886
node_3610: feature_name=FAM129C	FAM129C.value <= threshold=-0.30572274327278137
node_3611: feature_name=LDLRAD4	LDLRAD4.value > threshold=-0.26634007692337036
node_3619: feature_name=PARP14	PARP14.value > threshold=-0.5616399943828583
Class: Healthy Control	
Rules_824	passed counts:5
node_0: feature_name=LTB	LTB.value <= threshold=0.994031548500061
node_1: feature_name=MTRNR2L2	MTRNR2L2.value <= threshold=2.8784297704696655
node_2: feature_name=TCL1A	TCL1A.value <= threshold=4.798312664031982
node_3: feature_name=MT-CO1	MT-CO1.value > threshold=0.08947191759943962
node_2521: feature_name=LTB	LTB.value <= threshold=0.6038667261600494
node_2522: feature_name=HLA-DQB1	HLA-DQB1.value > threshold=2.1053054332733154
node_3550: feature_name=XIST	XIST.value <= threshold=0.7716337237507105
node_3551: feature_name=CD79B	CD79B.value <= threshold=2.9426796436309814
node_3552: feature_name=HLA-DQB1	HLA-DQB1.value <= threshold=2.713674306869507
node_3553: feature_name=SNX9	SNX9.value <= threshold=-0.5404067635536194
node_3554: feature_name=VPREB3	VPREB3.value <= threshold=4.4475414752960205
node_3555: feature_name=RALGPS2	RALGPS2.value <= threshold=2.7017585039138794
node_3556: feature_name=MEF2C	MEF2C.value > threshold=3.997420072555542
node_3604: feature_name=ICAM1	ICAM1.value <= threshold=-0.2164856493473053
Class: Healthy Control	
Rules_825	passed counts:5
node_0: feature_name=LTB	LTB.value <= threshold=0.994031548500061
node_1: feature_name=MTRNR2L2	MTRNR2L2.value <= threshold=2.8784297704696655
node_2: feature_name=TCL1A	TCL1A.value <= threshold=4.798312664031982
node_3: feature_name=MT-CO1	MT-CO1.value > threshold=0.08947191759943962

node_2521: feature_name=LTB	LTB.value <= threshold=0.6038667261600494
node_2522: feature_name=HLA-DQB1	HLA-DQB1.value <= threshold=2.1053054332733154
node_2523: feature_name=SNX9	SNX9.value > threshold=-0.5346486568450928
node_2907: feature_name=NR4A1	NR4A1.value > threshold=2.5433837175369263
node_3459: feature_name=VPREB3	VPREB3.value <= threshold=4.410779237747192
node_3460: feature_name=RALGPS2	RALGPS2.value <= threshold=3.064661979675293
node_3461: feature_name=MT-CO1	MT-CO1.value <= threshold=1.7489010095596313
node_3462: feature_name=HLA-DQA2	HLA-DQA2.value <= threshold=1.794841468334198
node_3463: feature_name=CYBB	CYBB.value <= threshold=1.5984815955162048
node_3464: feature_name=MARCKS	MARCKS.value > threshold=-0.38392288982868195
node_3474: feature_name=PLD4	PLD4.value <= threshold=7.326558828353882
node_3475: feature_name=IRAK3	IRAK3.value <= threshold=4.625715494155884
node_3476: feature_name=CCL3	CCL3.value <= threshold=1.9986491203308105
node_3477: feature_name=ID1	ID1.value <= threshold=3.170982599258423
node_3478: feature_name=AHR	AHR.value <= threshold=4.220391273498535
node_3479: feature_name=OAZ1	OAZ1.value <= threshold=-1.7742292881011963
node_3480: feature_name=YWHAH	YWHAH.value <= threshold=-0.41138526797294617
Class: COVID-19 Critical	
Rules_826	passed counts:5
node_0: feature_name=LTB	LTB.value <= threshold=0.994031548500061
node_1: feature_name=MTRNR2L2	MTRNR2L2.value <= threshold=2.8784297704696655
node_2: feature_name=TCL1A	TCL1A.value <= threshold=4.798312664031982
node_3: feature_name=MT-CO1	MT-CO1.value > threshold=0.08947191759943962
node_2521: feature_name=LTB	LTB.value <= threshold=0.6038667261600494
node_2522: feature_name=HLA-DQB1	HLA-DQB1.value <= threshold=2.1053054332733154
node_2523: feature_name=SNX9	SNX9.value > threshold=-0.5346486568450928
node_2907: feature_name=NR4A1	NR4A1.value > threshold=2.5433837175369263
node_3459: feature_name=VPREB3	VPREB3.value <= threshold=4.410779237747192
node_3460: feature_name=RALGPS2	RALGPS2.value <= threshold=3.064661979675293

node_3461: feature_name=MT-CO1	MT-CO1.value <= threshold=1.7489010095596313
node_3462: feature_name=HLA-DQA2	HLA-DQA2.value <= threshold=1.794841468334198
node_3463: feature_name=CYBB	CYBB.value <= threshold=1.5984815955162048
node_3464: feature_name=MARCKS	MARCKS.value <= threshold=-0.38392288982868195
node_3465: feature_name=OAZ1	OAZ1.value > threshold=-0.07741016428917646
Class: Healthy Control	
Rules_827	passed counts:5
node_0: feature_name=LTB	LTB.value <= threshold=0.994031548500061
node_1: feature_name=MTRNR2L2	MTRNR2L2.value <= threshold=2.8784297704696655
node_2: feature_name=TCL1A	TCL1A.value <= threshold=4.798312664031982
node_3: feature_name=MT-CO1	MT-CO1.value > threshold=0.08947191759943962
node_2521: feature_name=LTB	LTB.value <= threshold=0.6038667261600494
node_2522: feature_name=HLA-DQB1	HLA-DQB1.value <= threshold=2.1053054332733154
node_2523: feature_name=SNX9	SNX9.value > threshold=-0.5346486568450928
node_2907: feature_name=NR4A1	NR4A1.value <= threshold=2.5433837175369263
node_2908: feature_name=XIST	XIST.value <= threshold=0.7664470989257097
node_2909: feature_name=CD79B	CD79B.value > threshold=2.9326847791671753
node_3353: feature_name=VPREB3	VPREB3.value <= threshold=3.0226117372512817
node_3354: feature_name=CD83	CD83.value > threshold=1.9074636101722717
node_3388: feature_name=BOD1L1	BOD1L1.value <= threshold=-0.4742381125688553
node_3389: feature_name=MT-ND6	MT-ND6.value <= threshold=0.3542506694793701
node_3390: feature_name=JUN	JUN.value <= threshold=0.5178887248039246
node_3391: feature_name=HLA-DRB1	HLA-DRB1.value <= threshold=1.6789699792861938
Class: COVID-19 Severe	
Rules_828	passed counts:5
node_0: feature_name=LTB	LTB.value <= threshold=0.994031548500061
node_1: feature_name=MTRNR2L2	MTRNR2L2.value <= threshold=2.8784297704696655
node_2: feature_name=TCL1A	TCL1A.value <= threshold=4.798312664031982

node_3: feature_name=MT-CO1	MT-CO1.value > threshold=0.08947191759943962
node_2521: feature_name=LTB	LTB.value <= threshold=0.6038667261600494
node_2522: feature_name=HLA-DQB1	HLA-DQB1.value <= threshold=2.1053054332733154
node_2523: feature_name=SNX9	SNX9.value > threshold=-0.5346486568450928
node_2907: feature_name=NR4A1	NR4A1.value <= threshold=2.5433837175369263
node_2908: feature_name=XIST	XIST.value <= threshold=0.7664470989257097
node_2909: feature_name=CD79B	CD79B.value <= threshold=2.9326847791671753
node_2910: feature_name=NR4A1	NR4A1.value > threshold=-0.2875557690858841
node_3096: feature_name=NR4A3	NR4A3.value > threshold=3.314968705177307
node_3328: feature_name=MT-CO3	MT-CO3.value <= threshold=0.35808728635311127
node_3329: feature_name=MMD	MMD.value > threshold=-0.3053935170173645
Class: COVID-19 Severe	
Rules_829	passed counts:5
node_0: feature_name=LTB	LTB.value <= threshold=0.994031548500061
node_1: feature_name=MTRNR2L2	MTRNR2L2.value <= threshold=2.8784297704696655
node_2: feature_name=TCL1A	TCL1A.value <= threshold=4.798312664031982
node_3: feature_name=MT-CO1	MT-CO1.value > threshold=0.08947191759943962
node_2521: feature_name=LTB	LTB.value <= threshold=0.6038667261600494
node_2522: feature_name=HLA-DQB1	HLA-DQB1.value <= threshold=2.1053054332733154
node_2523: feature_name=SNX9	SNX9.value > threshold=-0.5346486568450928
node_2907: feature_name=NR4A1	NR4A1.value <= threshold=2.5433837175369263
node_2908: feature_name=XIST	XIST.value <= threshold=0.7664470989257097
node_2909: feature_name=CD79B	CD79B.value <= threshold=2.9326847791671753
node_2910: feature_name=NR4A1	NR4A1.value > threshold=-0.2875557690858841
node_3096: feature_name=NR4A3	NR4A3.value <= threshold=3.314968705177307
node_3097: feature_name=VPREB3	VPREB3.value <= threshold=3.3549938201904297
node_3098: feature_name=RALGPS2	RALGPS2.value <= threshold=5.807050704956055
node_3099: feature_name=CTD-2035E11.3	CTD-2035E11.3.value <= threshold=6.4090416431427
node_3100: feature_name=FAM129C	FAM129C.value <= threshold=6.966361045837402

node_3101: feature_name=HES4	HES4.value <= threshold=4.139557242393494
node_3102: feature_name=CD83	CD83.value > threshold=1.8343153595924377
node_3136: feature_name=PLD4	PLD4.value <= threshold=4.453821897506714
node_3137: feature_name=HLA-DQA2	HLA-DQA2.value <= threshold=-0.10758286714553833
node_3138: feature_name=STAG3	STAG3.value <= threshold=7.365770578384399
node_3139: feature_name=LDLR	LDLR.value <= threshold=2.3423796892166138
node_3140: feature_name=CD79A	CD79A.value <= threshold=4.038331866264343
node_3141: feature_name=DUSP4	DUSP4.value <= threshold=4.829863548278809
node_3142: feature_name=OAZ1	OAZ1.value > threshold=-0.34704728424549103
node_3178: feature_name=MX1	MX1.value > threshold=-0.48719069361686707
node_3184: feature_name=HLA-DPB1	HLA-DPB1.value <= threshold=2.151633381843567
node_3185: feature_name=RGS2	RGS2.value > threshold=1.594672977924347
node_3203: feature_name=MARCH1	MARCH1.value > threshold=1.3179876208305359
node_3207: feature_name=RP11-685N3.1	RP11-685N3.1.value > threshold=-0.1732286512851715
Class: Healthy Control	
Rules_830	passed counts:5
node_0: feature_name=LTB	LTB.value <= threshold=0.994031548500061
node_1: feature_name=MTRNR2L2	MTRNR2L2.value <= threshold=2.8784297704696655
node_2: feature_name=TCL1A	TCL1A.value <= threshold=4.798312664031982
node_3: feature_name=MT-CO1	MT-CO1.value > threshold=0.08947191759943962
node_2521: feature_name=LTB	LTB.value <= threshold=0.6038667261600494
node_2522: feature_name=HLA-DQB1	HLA-DQB1.value <= threshold=2.1053054332733154
node_2523: feature_name=SNX9	SNX9.value > threshold=-0.5346486568450928
node_2907: feature_name=NR4A1	NR4A1.value <= threshold=2.5433837175369263
node_2908: feature_name=XIST	XIST.value <= threshold=0.7664470989257097
node_2909: feature_name=CD79B	CD79B.value <= threshold=2.9326847791671753
node_2910: feature_name=NR4A1	NR4A1.value > threshold=-0.2875557690858841
node_3096: feature_name=NR4A3	NR4A3.value <= threshold=3.314968705177307
node_3097: feature_name=VPREB3	VPREB3.value <= threshold=3.3549938201904297

node_3098: feature_name=RALGPS2	RALGPS2.value <= threshold=5.807050704956055
node_3099: feature_name=CTD-2035E11.3	CTD-2035E11.3.value <= threshold=6.4090416431427
node_3100: feature_name=FAM129C	FAM129C.value <= threshold=6.966361045837402
node_3101: feature_name=HES4	HES4.value <= threshold=4.139557242393494
node_3102: feature_name=CD83	CD83.value > threshold=1.8343153595924377
node_3136: feature_name=PLD4	PLD4.value <= threshold=4.453821897506714
node_3137: feature_name=HLA-DQA2	HLA-DQA2.value <= threshold=-0.10758286714553833
node_3138: feature_name=STAG3	STAG3.value <= threshold=7.365770578384399
node_3139: feature_name=LDLR	LDLR.value <= threshold=2.3423796892166138
node_3140: feature_name=CD79A	CD79A.value <= threshold=4.038331866264343
node_3141: feature_name=DUSP4	DUSP4.value <= threshold=4.829863548278809
node_3142: feature_name=OAZ1	OAZ1.value <= threshold=-0.34704728424549103
node_3143: feature_name=CSF3R	CSF3R.value <= threshold=1.0170481204986572
node_3144: feature_name=IGLL5	IGLL5.value <= threshold=6.979742765426636
node_3145: feature_name=ANO6	ANO6.value <= threshold=4.378111124038696
node_3146: feature_name=NAMPT	NAMPT.value <= threshold=3.490760326385498
node_3147: feature_name=RP11-731F5.2	RP11-731F5.2.value <= threshold=8.205055236816406
node_3148: feature_name=COTL1	COTL1.value > threshold=-1.5100116729736328
node_3152: feature_name=LTB	LTB.value <= threshold=-1.0809237957000732
node_3153: feature_name=RBKS	RBKS.value <= threshold=-0.29577386379241943
Class: COVID-19 Critical	
Rules_831	passed counts:5
node_0: feature_name=LTB	LTB.value <= threshold=0.9940315485000610
node_1: feature_name=MTRNR2L2	MTRNR2L2.value <= threshold=2.8784297704696655
node_2: feature_name=TCL1A	TCL1A.value <= threshold=4.798312664031982
node_3: feature_name=MT-CO1	MT-CO1.value > threshold=0.08947191759943962
node_2521: feature_name=LTB	LTB.value <= threshold=0.6038667261600494
node_2522: feature_name=HLA-DQB1	HLA-DQB1.value <= threshold=2.1053054332733154
node_2523: feature_name=SNX9	SNX9.value <= threshold=-0.5346486568450928

node_2524: feature_name=HLA-DPB1	HLA-DPB1.value <= threshold=1.9548270106315613
node_2525: feature_name=FAM129C	FAM129C.value > threshold=3.3393388986587524
node_2721: feature_name=ADAM19	ADAM19.value > threshold=-0.29525794088840485
node_2781: feature_name=XAF1	XAF1.value > threshold=-0.4296249598264694
node_2793: feature_name=PELI1	PELI1.value > threshold=1.8806812763214111
node_2801: feature_name=HLA-DRB1	HLA-DRB1.value > threshold=0.921924501657486
node_2805: feature_name=MS4A1	MS4A1.value <= threshold=2.953020453453064
node_2806: feature_name=OAZ1	OAZ1.value <= threshold=1.0253153145313263
Class: COVID-19 Critical	
Rules_832	passed counts:5
node_0: feature_name=LTB	LTB.value <= threshold=0.994031548500061
node_1: feature_name=MTRNR2L2	MTRNR2L2.value <= threshold=2.8784297704696655
node_2: feature_name=TCL1A	TCL1A.value <= threshold=4.798312664031982
node_3: feature_name=MT-CO1	MT-CO1.value > threshold=0.08947191759943962
node_2521: feature_name=LTB	LTB.value <= threshold=0.6038667261600494
node_2522: feature_name=HLA-DQB1	HLA-DQB1.value <= threshold=2.1053054332733154
node_2523: feature_name=SNX9	SNX9.value <= threshold=-0.5346486568450928
node_2524: feature_name=HLA-DPB1	HLA-DPB1.value <= threshold=1.9548270106315613
node_2525: feature_name=FAM129C	FAM129C.value > threshold=3.3393388986587524
node_2721: feature_name=ADAM19	ADAM19.value <= threshold=-0.29525794088840485
node_2722: feature_name=NR4A1	NR4A1.value > threshold=2.1566513776779175
node_2774: feature_name=HLA-DRB1	HLA-DRB1.value <= threshold=0.8161951005458832
Class: COVID-19 Critical	
Rules_833	passed counts:5
node_0: feature_name=LTB	LTB.value <= threshold=0.994031548500061
node_1: feature_name=MTRNR2L2	MTRNR2L2.value <= threshold=2.8784297704696655
node_2: feature_name=TCL1A	TCL1A.value <= threshold=4.798312664031982
node_3: feature_name=MT-CO1	MT-CO1.value > threshold=0.08947191759943962

node_2521: feature_name=LTB	LTB.value <= threshold=0.6038667261600494
node_2522: feature_name=HLA-DQB1	HLA-DQB1.value <= threshold=2.1053054332733154
node_2523: feature_name=SNX9	SNX9.value <= threshold=-0.5346486568450928
node_2524: feature_name=HLA-DPB1	HLA-DPB1.value <= threshold=1.9548270106315613
node_2525: feature_name=FAM129C	FAM129C.value <= threshold=3.3393388986587524
node_2526: feature_name=XIST	XIST.value <= threshold=3.6911416053771973
node_2527: feature_name=NR4A1	NR4A1.value > threshold=-0.2557755410671234
node_2625: feature_name=HLA-DPA1	HLA-DPA1.value > threshold=0.9335225224494934
node_2657: feature_name=RALGPS2	RALGPS2.value <= threshold=2.9413715600967407
node_2658: feature_name=HLA-DQB1	HLA-DQB1.value <= threshold=1.7503233551979065
node_2659: feature_name=TP53INP1	TP53INP1.value <= threshold=-0.2649386376142502
node_2660: feature_name=OAZ1	OAZ1.value <= threshold=1.2488974928855896
node_2661: feature_name=HLA-DPB1	HLA-DPB1.value > threshold=-0.5502273440361023
node_2667: feature_name=SLC7A5	SLC7A5.value <= threshold=-0.5558908581733704
node_2668: feature_name=CCR6	CCR6.value <= threshold=2.199329726397991
Class: COVID-19 Severe	
Rules_834	passed counts:5
node_0: feature_name=LTB	LTB.value <= threshold=0.994031548500061
node_1: feature_name=MTRNR2L2	MTRNR2L2.value <= threshold=2.8784297704696655
node_2: feature_name=TCL1A	TCL1A.value <= threshold=4.798312664031982
node_3: feature_name=MT-CO1	MT-CO1.value > threshold=0.08947191759943962
node_2521: feature_name=LTB	LTB.value <= threshold=0.6038667261600494
node_2522: feature_name=HLA-DQB1	HLA-DQB1.value <= threshold=2.1053054332733154
node_2523: feature_name=SNX9	SNX9.value <= threshold=-0.5346486568450928
node_2524: feature_name=HLA-DPB1	HLA-DPB1.value <= threshold=1.9548270106315613
node_2525: feature_name=FAM129C	FAM129C.value <= threshold=3.3393388986587524
node_2526: feature_name=XIST	XIST.value <= threshold=3.6911416053771973
node_2527: feature_name=NR4A1	NR4A1.value <= threshold=-0.2557755410671234
node_2528: feature_name=HOPX	HOPX.value <= threshold=2.776473879814148

node_2529: feature_name=AREGB	AREGB.value <= threshold=-0.10896047577261925
node_2530: feature_name=KIAA0125	KIAA0125.value <= threshold=7.652706623077393
node_2531: feature_name=MT-CO3	MT-CO3.value > threshold=-1.3726763725280762
node_2533: feature_name=TNF	TNF.value <= threshold=3.1101887226104736
node_2534: feature_name=NFKBID	NFKBID.value > threshold=-0.39706873893737793
node_2568: feature_name=TCF4	TCF4.value <= threshold=6.289925575256348
node_2569: feature_name=CD22	CD22.value <= threshold=8.377277374267578
node_2570: feature_name=FERMT3	FERMT3.value <= threshold=2.260508179664612
node_2571: feature_name=MT-ND6	MT-ND6.value <= threshold=3.413356304168701
node_2572: feature_name=ID1	ID1.value > threshold=1.9121195077896118
node_2606: feature_name=XAF1	XAF1.value <= threshold=-0.4339040219783783
Class: COVID-19 Critical	
Rules_835	passed counts:5
node_0: feature_name=LTB	LTB.value <= threshold=0.994031548500061
node_1: feature_name=MTRNR2L2	MTRNR2L2.value <= threshold=2.8784297704696655
node_2: feature_name=TCL1A	TCL1A.value <= threshold=4.798312664031982
node_3: feature_name=MT-CO1	MT-CO1.value > threshold=0.08947191759943962
node_2521: feature_name=LTB	LTB.value <= threshold=0.6038667261600494
node_2522: feature_name=HLA-DQB1	HLA-DQB1.value <= threshold=2.1053054332733154
node_2523: feature_name=SNX9	SNX9.value <= threshold=-0.5346486568450928
node_2524: feature_name=HLA-DPB1	HLA-DPB1.value <= threshold=1.9548270106315613
node_2525: feature_name=FAM129C	FAM129C.value <= threshold=3.3393388986587524
node_2526: feature_name=XIST	XIST.value <= threshold=3.6911416053771973
node_2527: feature_name=NR4A1	NR4A1.value <= threshold=-0.2557755410671234
node_2528: feature_name=HOPX	HOPX.value <= threshold=2.776473879814148
node_2529: feature_name=AREGB	AREGB.value <= threshold=-0.10896047577261925
node_2530: feature_name=KIAA0125	KIAA0125.value <= threshold=7.652706623077393
node_2531: feature_name=MT-CO3	MT-CO3.value > threshold=-1.3726763725280762
node_2533: feature_name=TNF	TNF.value <= threshold=3.1101887226104736

node_2534: feature_name=NFKBID	NFKBID.value > threshold=-0.39706873893737793
node_2568: feature_name=TCF4	TCF4.value <= threshold=6.289925575256348
node_2569: feature_name=CD22	CD22.value <= threshold=8.377277374267578
node_2570: feature_name=FERMT3	FERMT3.value <= threshold=2.260508179664612
node_2571: feature_name=MT-ND6	MT-ND6.value <= threshold=3.413356304168701
node_2572: feature_name=ID1	ID1.value <= threshold=1.9121195077896118
node_2573: feature_name=DAPK1	DAPK1.value <= threshold=1.6979314014315605
node_2574: feature_name=IRF4	IRF4.value <= threshold=8.765593528747559
node_2575: feature_name=PLD4	PLD4.value <= threshold=6.6784141063690186
node_2576: feature_name=GPX1	GPX1.value > threshold=-0.9480071663856506
node_2582: feature_name=MYL9	MYL9.value <= threshold=2.9503014087677
node_2583: feature_name=ID1	ID1.value <= threshold=1.3511708974838257
node_2584: feature_name=HLA-DRB5	HLA-DRB5.value <= threshold=-0.8129743039608002
node_2585: feature_name=VPREB3	VPREB3.value > threshold=-0.19180332869291306
Class: COVID-19 Critical	
Rules_836	passed counts:5
node_0: feature_name=LTB	LTB.value <= threshold=0.994031548500061
node_1: feature_name=MTRNR2L2	MTRNR2L2.value <= threshold=2.8784297704696655
node_2: feature_name=TCL1A	TCL1A.value <= threshold=4.798312664031982
node_3: feature_name=MT-CO1	MT-CO1.value > threshold=0.08947191759943962
node_2521: feature_name=LTB	LTB.value <= threshold=0.6038667261600494
node_2522: feature_name=HLA-DQB1	HLA-DQB1.value <= threshold=2.1053054332733154
node_2523: feature_name=SNX9	SNX9.value <= threshold=-0.5346486568450928
node_2524: feature_name=HLA-DPB1	HLA-DPB1.value <= threshold=1.9548270106315613
node_2525: feature_name=FAM129C	FAM129C.value <= threshold=3.3393388986587524
node_2526: feature_name=XIST	XIST.value <= threshold=3.6911416053771973
node_2527: feature_name=NR4A1	NR4A1.value <= threshold=-0.2557755410671234
node_2528: feature_name=HOPX	HOPX.value <= threshold=2.776473879814148
node_2529: feature_name=AREGB	AREGB.value <= threshold=-0.10896047577261925

node_2530: feature_name=KIAA0125	KIAA0125.value <= threshold=7.652706623077393
node_2531: feature_name=MT-CO3	MT-CO3.value > threshold=-1.3726763725280762
node_2533: feature_name=TNF	TNF.value <= threshold=3.1101887226104736
node_2534: feature_name=NFKBID	NFKBID.value <= threshold=-0.39706873893737793
node_2535: feature_name=KIAA0125	KIAA0125.value <= threshold=-0.17713353037834167
node_2536: feature_name=MT-CYB	MT-CYB.value > threshold=-0.0009266361594200134
node_2538: feature_name=DENND3	DENND3.value <= threshold=-0.35449540615081787
Class: COVID-19 Critical	
Rules_837	passed counts:5
node_0: feature_name=LTB	LTB.value <= threshold=0.994031548500061
node_1: feature_name=MTRNR2L2	MTRNR2L2.value <= threshold=2.8784297704696655
node_2: feature_name=TCL1A	TCL1A.value <= threshold=4.798312664031982
node_3: feature_name=MT-CO1	MT-CO1.value <= threshold=0.08947191759943962
node_4: feature_name=NR4A1	NR4A1.value > threshold=1.0725348591804504
node_1622: feature_name=HLA-DQA2	HLA-DQA2.value > threshold=1.9496816992759705
node_2350: feature_name=BCL2A1	BCL2A1.value > threshold=1.1322240233421326
node_2466: feature_name=MS4A1	MS4A1.value > threshold=2.7321707010269165
node_2510: feature_name=RHOB	RHOB.value <= threshold=-0.35927484929561615
node_2511: feature_name=HLA-DRB1	HLA-DRB1.value > threshold=1.622136414051056
Class: COVID-19 Severe	
Rules_838	passed counts:5
node_0: feature_name=LTB	LTB.value <= threshold=0.994031548500061
node_1: feature_name=MTRNR2L2	MTRNR2L2.value <= threshold=2.8784297704696655
node_2: feature_name=TCL1A	TCL1A.value <= threshold=4.798312664031982
node_3: feature_name=MT-CO1	MT-CO1.value <= threshold=0.08947191759943962
node_4: feature_name=NR4A1	NR4A1.value > threshold=1.0725348591804504
node_1622: feature_name=HLA-DQA2	HLA-DQA2.value > threshold=1.9496816992759705
node_2350: feature_name=BCL2A1	BCL2A1.value > threshold=1.1322240233421326

node_2466: feature_name=MS4A1	MS4A1.value <= threshold=2.7321707010269165
node_2467: feature_name=HSP90B1	HSP90B1.value <= threshold=0.8215908706188202
node_2468: feature_name=HLA-DQA1	HLA-DQA1.value > threshold=1.4212530851364136
node_2476: feature_name=MT-CO3	MT-CO3.value > threshold=0.05051383376121521
node_2488: feature_name=SPI1	SPI1.value > threshold=-0.4845922589302063
Class: COVID-19 Severe	
Rules_839	passed counts:5
node_0: feature_name=LTB	LTB.value <= threshold=0.994031548500061
node_1: feature_name=MTRNR2L2	MTRNR2L2.value <= threshold=2.8784297704696655
node_2: feature_name=TCL1A	TCL1A.value <= threshold=4.798312664031982
node_3: feature_name=MT-CO1	MT-CO1.value <= threshold=0.08947191759943962
node_4: feature_name=NR4A1	NR4A1.value > threshold=1.0725348591804504
node_1622: feature_name=HLA-DQA2	HLA-DQA2.value > threshold=1.9496816992759705
node_2350: feature_name=BCL2A1	BCL2A1.value <= threshold=1.1322240233421326
node_2351: feature_name=CD83	CD83.value > threshold=2.3162450790405273
node_2391: feature_name=CD79B	CD79B.value > threshold=1.7111427783966064
node_2443: feature_name=MT-CO1	MT-CO1.value > threshold=-1.1614157557487488
node_2447: feature_name=JUN	JUN.value <= threshold=0.19632036238908768
node_2448: feature_name=PLD4	PLD4.value <= threshold=-0.11274048686027527
node_2449: feature_name=RNF11	RNF11.value > threshold=-0.5163591802120209
node_2451: feature_name=RP11-685N3.1	RP11-685N3.1.value > threshold=-0.17591144144535065
Class: COVID-19 Severe	
Rules_840	passed counts:5
node_0: feature_name=LTB	LTB.value <= threshold=0.994031548500061
node_1: feature_name=MTRNR2L2	MTRNR2L2.value <= threshold=2.8784297704696655
node_2: feature_name=TCL1A	TCL1A.value <= threshold=4.798312664031982
node_3: feature_name=MT-CO1	MT-CO1.value <= threshold=0.08947191759943962
node_4: feature_name=NR4A1	NR4A1.value > threshold=1.0725348591804504

node_1622: feature_name=HLA-DQA2	HLA-DQA2.value > threshold=1.9496816992759705
node_2350: feature_name=BCL2A1	BCL2A1.value <= threshold=1.1322240233421326
node_2351: feature_name=CD83	CD83.value > threshold=2.3162450790405273
node_2391: feature_name=CD79B	CD79B.value <= threshold=1.7111427783966064
node_2392: feature_name=TTN	TTN.value <= threshold=-0.28553803265094757
node_2393: feature_name=LDLRAD4	LDLRAD4.value <= threshold=3.3328956365585327
node_2394: feature_name=BCL2A1	BCL2A1.value > threshold=-0.5811292827129364
node_2404: feature_name=SLC7A5	SLC7A5.value <= threshold=1.3126214146614075
node_2405: feature_name=AP3M2	AP3M2.value <= threshold=1.617304801940918
node_2406: feature_name=MT-ND6	MT-ND6.value > threshold=0.7549090385437012
node_2416: feature_name=NAMPT	NAMPT.value > threshold=-0.5255279242992401
Class: COVID-19 Severe	
Rules_841	passed counts:5
node_0: feature_name=LTB	LTB.value <= threshold=0.994031548500061
node_1: feature_name=MTRNR2L2	MTRNR2L2.value <= threshold=2.8784297704696655
node_2: feature_name=TCL1A	TCL1A.value <= threshold=4.798312664031982
node_3: feature_name=MT-CO1	MT-CO1.value <= threshold=0.08947191759943962
node_4: feature_name=NR4A1	NR4A1.value > threshold=1.0725348591804504
node_1622: feature_name=HLA-DQA2	HLA-DQA2.value > threshold=1.9496816992759705
node_2350: feature_name=BCL2A1	BCL2A1.value <= threshold=1.1322240233421326
node_2351: feature_name=CD83	CD83.value <= threshold=2.3162450790405273
node_2352: feature_name=LTB	LTB.value > threshold=0.7341383993625641
node_2380: feature_name=VPREB3	VPREB3.value <= threshold=3.700833559036255
node_2381: feature_name=NFKBID	NFKBID.value > threshold=1.520115613937378
node_2385: feature_name=ATF3	ATF3.value > threshold=-0.4353189915418625
Class: COVID-19 Severe	
Rules_842	passed counts:5
node_0: feature_name=LTB	LTB.value <= threshold=0.994031548500061

node_1: feature_name=MTRNR2L2	MTRNR2L2.value <= threshold=2.8784297704696655
node_2: feature_name=TCL1A	TCL1A.value <= threshold=4.798312664031982
node_3: feature_name=MT-CO1	MT-CO1.value <= threshold=0.08947191759943962
node_4: feature_name=NR4A1	NR4A1.value > threshold=1.0725348591804504
node_1622: feature_name=HLA-DQA2	HLA-DQA2.value > threshold=1.9496816992759705
node_2350: feature_name=BCL2A1	BCL2A1.value <= threshold=1.1322240233421326
node_2351: feature_name=CD83	CD83.value <= threshold=2.3162450790405273
node_2352: feature_name=LTB	LTB.value <= threshold=0.7341383993625641
node_2353: feature_name=TCL1A	TCL1A.value <= threshold=3.942829728126526
node_2354: feature_name=MARCKS	MARCKS.value > threshold=2.9345329999923706
node_2374: feature_name=TCF4	TCF4.value <= threshold=3.1691582202911377
Class: COVID-19 Severe	
Rules_843	passed counts:5
node_0: feature_name=LTB	LTB.value <= threshold=0.994031548500061
node_1: feature_name=MTRNR2L2	MTRNR2L2.value <= threshold=2.8784297704696655
node_2: feature_name=TCL1A	TCL1A.value <= threshold=4.798312664031982
node_3: feature_name=MT-CO1	MT-CO1.value <= threshold=0.08947191759943962
node_4: feature_name=NR4A1	NR4A1.value > threshold=1.0725348591804504
node_1622: feature_name=HLA-DQA2	HLA-DQA2.value <= threshold=1.9496816992759705
node_1623: feature_name=HLA-DRB5	HLA-DRB5.value > threshold=2.715766429901123
node_2337: feature_name=HLA-DRB1	HLA-DRB1.value > threshold=1.9765487909317017
node_2345: feature_name=CD83	CD83.value <= threshold=3.0348469018936157
Class: COVID-19 Critical	
Rules_844	passed counts:5
node_0: feature_name=LTB	LTB.value <= threshold=0.994031548500061
node_1: feature_name=MTRNR2L2	MTRNR2L2.value <= threshold=2.8784297704696655
node_2: feature_name=TCL1A	TCL1A.value <= threshold=4.798312664031982
node_3: feature_name=MT-CO1	MT-CO1.value <= threshold=0.08947191759943962

node_4: feature_name=NR4A1	NR4A1.value > threshold=1.0725348591804504
node_1622: feature_name=HLA-DQA2	HLA-DQA2.value <= threshold=1.9496816992759705
node_1623: feature_name=HLA-DRB5	HLA-DRB5.value <= threshold=2.715766429901123
node_1624: feature_name=HLA-DPA1	HLA-DPA1.value > threshold=1.7050301432609558
node_2056: feature_name=OAZ1	OAZ1.value > threshold=0.456103190779686
node_2266: feature_name=XAF1	XAF1.value > threshold=-0.4431648850440979
node_2312: feature_name=ISG15	ISG15.value <= threshold=-0.3511900007724762
node_2313: feature_name=RALGPS2	RALGPS2.value <= threshold=2.2695717811584473
node_2314: feature_name=FCRL1	FCRL1.value > threshold=4.402489423751831
node_2324: feature_name=IRF4	IRF4.value <= threshold=-0.20331371575593948
Class: Healthy Control	
Rules_845	passed counts:5
node_0: feature_name=LTB	LTB.value <= threshold=0.994031548500061
node_1: feature_name=MTRNR2L2	MTRNR2L2.value <= threshold=2.8784297704696655
node_2: feature_name=TCL1A	TCL1A.value <= threshold=4.798312664031982
node_3: feature_name=MT-CO1	MT-CO1.value <= threshold=0.08947191759943962
node_4: feature_name=NR4A1	NR4A1.value > threshold=1.0725348591804504
node_1622: feature_name=HLA-DQA2	HLA-DQA2.value <= threshold=1.9496816992759705
node_1623: feature_name=HLA-DRB5	HLA-DRB5.value <= threshold=2.715766429901123
node_1624: feature_name=HLA-DPA1	HLA-DPA1.value > threshold=1.7050301432609558
node_2056: feature_name=OAZ1	OAZ1.value > threshold=0.456103190779686
node_2266: feature_name=XAF1	XAF1.value <= threshold=-0.4431648850440979
node_2267: feature_name=MT-CO1	MT-CO1.value > threshold=-0.3505740910768509
node_2293: feature_name=LTB	LTB.value > threshold=-0.31744880229234695
node_2301: feature_name=CD79A	CD79A.value <= threshold=2.299592971801758
node_2302: feature_name=S100A6	S100A6.value <= threshold=-0.7221342325210571
node_2303: feature_name=HOPX	HOPX.value <= threshold=0.3391673117876053
Class: Healthy Control	

Rules_846	passed counts:5
node_0: feature_name=LTB	LTB.value <= threshold=0.994031548500061
node_1: feature_name=MTRNR2L2	MTRNR2L2.value <= threshold=2.8784297704696655
node_2: feature_name=TCL1A	TCL1A.value <= threshold=4.798312664031982
node_3: feature_name=MT-CO1	MT-CO1.value <= threshold=0.08947191759943962
node_4: feature_name=NR4A1	NR4A1.value > threshold=1.0725348591804504
node_1622: feature_name=HLA-DQA2	HLA-DQA2.value <= threshold=1.9496816992759705
node_1623: feature_name=HLA-DRB5	HLA-DRB5.value <= threshold=2.715766429901123
node_1624: feature_name=HLA-DPA1	HLA-DPA1.value > threshold=1.7050301432609558
node_2056: feature_name=OAZ1	OAZ1.value <= threshold=0.456103190779686
node_2057: feature_name=HLA-DRB5	HLA-DRB5.value > threshold=0.6839984953403473
node_2101: feature_name=HLA-DRB1	HLA-DRB1.value > threshold=1.6199676990509033
node_2151: feature_name=HLA-DQB1	HLA-DQB1.value > threshold=3.1961567401885986
node_2257: feature_name=XAF1	XAF1.value <= threshold=2.217243790626526
node_2258: feature_name=MT-CO1	MT-CO1.value <= threshold=-1.0810123085975647
Class: COVID-19 Critical	
Rules_847	passed counts:5
node_0: feature_name=LTB	LTB.value <= threshold=0.994031548500061
node_1: feature_name=MTRNR2L2	MTRNR2L2.value <= threshold=2.8784297704696655
node_2: feature_name=TCL1A	TCL1A.value <= threshold=4.798312664031982
node_3: feature_name=MT-CO1	MT-CO1.value <= threshold=0.08947191759943962
node_4: feature_name=NR4A1	NR4A1.value > threshold=1.0725348591804504
node_1622: feature_name=HLA-DQA2	HLA-DQA2.value <= threshold=1.9496816992759705
node_1623: feature_name=HLA-DRB5	HLA-DRB5.value <= threshold=2.715766429901123
node_1624: feature_name=HLA-DPA1	HLA-DPA1.value > threshold=1.7050301432609558
node_2056: feature_name=OAZ1	OAZ1.value <= threshold=0.456103190779686
node_2057: feature_name=HLA-DRB5	HLA-DRB5.value > threshold=0.6839984953403473
node_2101: feature_name=HLA-DRB1	HLA-DRB1.value > threshold=1.6199676990509033
node_2151: feature_name=HLA-DQB1	HLA-DQB1.value <= threshold=3.1961567401885986

node_2152: feature_name=PLD4	PLD4.value <= threshold=-0.03259922005236149
node_2153: feature_name=XIST	XIST.value > threshold=-0.138966154307127
Class: COVID-19 Severe	
Rules_848	passed counts:5
node_0: feature_name=LTB	LTB.value <= threshold=0.994031548500061
node_1: feature_name=MTRNR2L2	MTRNR2L2.value <= threshold=2.8784297704696655
node_2: feature_name=TCL1A	TCL1A.value <= threshold=4.798312664031982
node_3: feature_name=MT-CO1	MT-CO1.value <= threshold=0.08947191759943962
node_4: feature_name=NR4A1	NR4A1.value > threshold=1.0725348591804504
node_1622: feature_name=HLA-DQA2	HLA-DQA2.value <= threshold=1.9496816992759705
node_1623: feature_name=HLA-DRB5	HLA-DRB5.value <= threshold=2.715766429901123
node_1624: feature_name=HLA-DPA1	HLA-DPA1.value > threshold=1.7050301432609558
node_2056: feature_name=OAZ1	OAZ1.value <= threshold=0.456103190779686
node_2057: feature_name=HLA-DRB5	HLA-DRB5.value > threshold=0.6839984953403473
node_2101: feature_name=HLA-DRB1	HLA-DRB1.value > threshold=1.6199676990509033
node_2151: feature_name=HLA-DQB1	HLA-DQB1.value <= threshold=3.1961567401885986
node_2152: feature_name=PLD4	PLD4.value <= threshold=-0.03259922005236149
node_2153: feature_name=XIST	XIST.value <= threshold=-0.138966154307127
node_2154: feature_name=HLA-DQA1	HLA-DQA1.value <= threshold=2.0848299264907837
node_2155: feature_name=LTB	LTB.value <= threshold=-0.9993145167827606
node_2156: feature_name=MARCH1	MARCH1.value <= threshold=0.7534386068582535
Class: COVID-19 Severe	
Rules_849	passed counts:5
node_0: feature_name=LTB	LTB.value <= threshold=0.994031548500061
node_1: feature_name=MTRNR2L2	MTRNR2L2.value <= threshold=2.8784297704696655
node_2: feature_name=TCL1A	TCL1A.value <= threshold=4.798312664031982
node_3: feature_name=MT-CO1	MT-CO1.value <= threshold=0.08947191759943962
node_4: feature_name=NR4A1	NR4A1.value > threshold=1.0725348591804504

node_1622: feature_name=HLA-DQA2	HLA-DQA2.value <= threshold=1.9496816992759705
node_1623: feature_name=HLA-DRB5	HLA-DRB5.value <= threshold=2.715766429901123
node_1624: feature_name=HLA-DPA1	HLA-DPA1.value > threshold=1.7050301432609558
node_2056: feature_name=OAZ1	OAZ1.value <= threshold=0.456103190779686
node_2057: feature_name=HLA-DRB5	HLA-DRB5.value > threshold=0.6839984953403473
node_2101: feature_name=HLA-DRB1	HLA-DRB1.value <= threshold=1.6199676990509033
node_2102: feature_name=CD83	CD83.value <= threshold=2.5568599700927734
node_2103: feature_name=MT-CO1	MT-CO1.value <= threshold=-0.08588453382253647
node_2104: feature_name=FAM129C	FAM129C.value <= threshold=3.9770365953445435
node_2105: feature_name=OAZ1	OAZ1.value > threshold=0.13203191384673119
node_2113: feature_name=HLA-DQA1	HLA-DQA1.value <= threshold=2.55633544921875
node_2114: feature_name=MARCH1	MARCH1.value <= threshold=2.40384840965271
Class: COVID-19 Critical	
Rules_850	passed counts:5
node_0: feature_name=LTB	LTB.value <= threshold=0.994031548500061
node_1: feature_name=MTRNR2L2	MTRNR2L2.value <= threshold=2.8784297704696655
node_2: feature_name=TCL1A	TCL1A.value <= threshold=4.798312664031982
node_3: feature_name=MT-CO1	MT-CO1.value <= threshold=0.08947191759943962
node_4: feature_name=NR4A1	NR4A1.value > threshold=1.0725348591804504
node_1622: feature_name=HLA-DQA2	HLA-DQA2.value <= threshold=1.9496816992759705
node_1623: feature_name=HLA-DRB5	HLA-DRB5.value <= threshold=2.715766429901123
node_1624: feature_name=HLA-DPA1	HLA-DPA1.value <= threshold=1.7050301432609558
node_1625: feature_name=CD83	CD83.value > threshold=2.783489227294922
node_1873: feature_name=IFI44L	IFI44L.value <= threshold=1.5938722491264343
node_1874: feature_name=PLD4	PLD4.value > threshold=4.101001739501953
node_2028: feature_name=SNX9	SNX9.value > threshold=1.4460421800613403
node_2036: feature_name=ID1	ID1.value <= threshold=-0.23599714040756226
node_2037: feature_name=IGLL5	IGLL5.value > threshold=-0.24608439952135086
Class: COVID-19 Critical	

Rules_851	passed counts:5
node_0: feature_name=LTB	LTB.value <= threshold=0.994031548500061
node_1: feature_name=MTRNR2L2	MTRNR2L2.value <= threshold=2.8784297704696655
node_2: feature_name=TCL1A	TCL1A.value <= threshold=4.798312664031982
node_3: feature_name=MT-CO1	MT-CO1.value <= threshold=0.08947191759943962
node_4: feature_name=NR4A1	NR4A1.value > threshold=1.0725348591804504
node_1622: feature_name=HLA-DQA2	HLA-DQA2.value <= threshold=1.9496816992759705
node_1623: feature_name=HLA-DRB5	HLA-DRB5.value <= threshold=2.715766429901123
node_1624: feature_name=HLA-DPA1	HLA-DPA1.value <= threshold=1.7050301432609558
node_1625: feature_name=CD83	CD83.value > threshold=2.783489227294922
node_1873: feature_name=IFI44L	IFI44L.value <= threshold=1.5938722491264343
node_1874: feature_name=PLD4	PLD4.value <= threshold=4.101001739501953
node_1875: feature_name=RHOB	RHOB.value <= threshold=1.304835319519043
node_1876: feature_name=PPIF	PPIF.value <= threshold=2.2943923473358154
node_1877: feature_name=OAZ1	OAZ1.value <= threshold=0.04260866530239582
node_1878: feature_name=ID3	ID3.value <= threshold=5.290210247039795
node_1879: feature_name=NFKBID	NFKBID.value <= threshold=-0.40532417595386505
node_1880: feature_name=SNX29P2	SNX29P2.value <= threshold=1.4727885127067566
Class: COVID-19 Severe	
Rules_852	passed counts:5
node_0: feature_name=LTB	LTB.value <= threshold=0.994031548500061
node_1: feature_name=MTRNR2L2	MTRNR2L2.value <= threshold=2.8784297704696655
node_2: feature_name=TCL1A	TCL1A.value <= threshold=4.798312664031982
node_3: feature_name=MT-CO1	MT-CO1.value <= threshold=0.08947191759943962
node_4: feature_name=NR4A1	NR4A1.value > threshold=1.0725348591804504
node_1622: feature_name=HLA-DQA2	HLA-DQA2.value <= threshold=1.9496816992759705
node_1623: feature_name=HLA-DRB5	HLA-DRB5.value <= threshold=2.715766429901123
node_1624: feature_name=HLA-DPA1	HLA-DPA1.value <= threshold=1.7050301432609558

node_1625: feature_name=CD83	CD83.value <= threshold=2.783489227294922
node_1626: feature_name=XIST	XIST.value <= threshold=0.9589543007314205
node_1627: feature_name=PLD4	PLD4.value <= threshold=5.865932464599609
node_1628: feature_name=OAZ1	OAZ1.value <= threshold=0.6137169003486633
node_1629: feature_name=MTRNR2L2	MTRNR2L2.value <= threshold=1.6102792620658875
node_1630: feature_name=IFI6	IFI6.value > threshold=-0.8404486179351807
node_1632: feature_name=NR4A1	NR4A1.value > threshold=1.0916320085525513
node_1638: feature_name=DENND3	DENND3.value <= threshold=3.988452672958374
node_1639: feature_name=HLA-DRB1	HLA-DRB1.value > threshold=1.4092575907707214
node_1749: feature_name=HLA-DQB1	HLA-DQB1.value <= threshold=3.4929215908050537
node_1750: feature_name=RP11-685N3.1	RP11-685N3.1.value <= threshold=9.97533893585205
node_1751: feature_name=RGS1	RGS1.value > threshold=-0.5999330878257751
node_1765: feature_name=MT-ND6	MT-ND6.value <= threshold=2.4838690757751465
node_1766: feature_name=DAPK1	DAPK1.value <= threshold=4.920811414718628
node_1767: feature_name=RGS1	RGS1.value <= threshold=3.3605728149414062
node_1768: feature_name=ANO6	ANO6.value <= threshold=4.00420081615448
node_1769: feature_name=FTH1	FTH1.value > threshold=1.6050249934196472
node_1825: feature_name=TNFAIP3	TNFAIP3.value > threshold=-1.4747682809829712
Class: COVID-19 Critical	
Rules_853	passed counts:5
node_0: feature_name=LTB	LTB.value <= threshold=0.994031548500061
node_1: feature_name=MTRNR2L2	MTRNR2L2.value <= threshold=2.8784297704696655
node_2: feature_name=TCL1A	TCL1A.value <= threshold=4.798312664031982
node_3: feature_name=MT-CO1	MT-CO1.value <= threshold=0.08947191759943962
node_4: feature_name=NR4A1	NR4A1.value > threshold=1.0725348591804504
node_1622: feature_name=HLA-DQA2	HLA-DQA2.value <= threshold=1.9496816992759705
node_1623: feature_name=HLA-DRB5	HLA-DRB5.value <= threshold=2.715766429901123
node_1624: feature_name=HLA-DPA1	HLA-DPA1.value <= threshold=1.7050301432609558
node_1625: feature_name=CD83	CD83.value <= threshold=2.783489227294922

node_1626: feature_name=XIST	XIST.value <= threshold=0.9589543007314205
node_1627: feature_name=PLD4	PLD4.value <= threshold=5.865932464599609
node_1628: feature_name=OAZ1	OAZ1.value <= threshold=0.6137169003486633
node_1629: feature_name=MTRNR2L2	MTRNR2L2.value <= threshold=1.6102792620658875
node_1630: feature_name=IFI6	IFI6.value > threshold=-0.8404486179351807
node_1632: feature_name=NR4A1	NR4A1.value > threshold=1.0916320085525513
node_1638: feature_name=DENND3	DENND3.value <= threshold=3.988452672958374
node_1639: feature_name=HLA-DRB1	HLA-DRB1.value > threshold=1.4092575907707214
node_1749: feature_name=HLA-DQB1	HLA-DQB1.value <= threshold=3.4929215908050537
node_1750: feature_name=RP11-685N3.1	RP11-685N3.1.value <= threshold=9.97533893585205
node_1751: feature_name=RGS1	RGS1.value > threshold=-0.5999330878257751
node_1765: feature_name=MT-ND6	MT-ND6.value <= threshold=2.4838690757751465
node_1766: feature_name=DAPK1	DAPK1.value <= threshold=4.920811414718628
node_1767: feature_name=RGS1	RGS1.value <= threshold=3.3605728149414062
node_1768: feature_name=ANO6	ANO6.value <= threshold=4.00420081615448
node_1769: feature_name=FTH1	FTH1.value <= threshold=1.6050249934196472
node_1770: feature_name=APP	APP.value <= threshold=2.417694091796875
node_1771: feature_name=ITGAX	ITGAX.value <= threshold=2.950524091720581
node_1772: feature_name=PDLIM1	PDLIM1.value <= threshold=5.856243848800659
node_1773: feature_name=RELT	RELT.value <= threshold=2.891391396522522
node_1774: feature_name=RHOB	RHOB.value <= threshold=3.2315229177474976
node_1775: feature_name=NR4A1	NR4A1.value <= threshold=1.125234842300415
node_1776: feature_name=IGLL5	IGLL5.value <= threshold=2.0251446962356567
Class: COVID-19 Critical	
Rules_854	passed counts:5
node_0: feature_name=LTB	LTB.value <= threshold=0.994031548500061
node_1: feature_name=MTRNR2L2	MTRNR2L2.value <= threshold=2.8784297704696655
node_2: feature_name=TCL1A	TCL1A.value <= threshold=4.798312664031982
node_3: feature_name=MT-CO1	MT-CO1.value <= threshold=0.08947191759943962

node_4: feature_name=NR4A1	NR4A1.value > threshold=1.0725348591804504
node_1622: feature_name=HLA-DQA2	HLA-DQA2.value <= threshold=1.9496816992759705
node_1623: feature_name=HLA-DRB5	HLA-DRB5.value <= threshold=2.715766429901123
node_1624: feature_name=HLA-DPA1	HLA-DPA1.value <= threshold=1.7050301432609558
node_1625: feature_name=CD83	CD83.value <= threshold=2.783489227294922
node_1626: feature_name=XIST	XIST.value <= threshold=0.9589543007314205
node_1627: feature_name=PLD4	PLD4.value <= threshold=5.865932464599609
node_1628: feature_name=OAZ1	OAZ1.value <= threshold=0.6137169003486633
node_1629: feature_name=MTRNR2L2	MTRNR2L2.value <= threshold=1.6102792620658875
node_1630: feature_name=IFI6	IFI6.value > threshold=-0.8404486179351807
node_1632: feature_name=NR4A1	NR4A1.value <= threshold=1.0916320085525513
node_1633: feature_name=PARP14	PARP14.value > threshold=-0.4991041421890259
node_1635: feature_name=IL8	IL8.value > threshold=-0.3314541280269623
Class: COVID-19 Critical	
Rules_855	passed counts:5
node_0: feature_name=LTB	LTB.value <= threshold=0.994031548500061
node_1: feature_name=MTRNR2L2	MTRNR2L2.value <= threshold=2.8784297704696655
node_2: feature_name=TCL1A	TCL1A.value <= threshold=4.798312664031982
node_3: feature_name=MT-CO1	MT-CO1.value <= threshold=0.08947191759943962
node_4: feature_name=NR4A1	NR4A1.value <= threshold=1.0725348591804504
node_5: feature_name=HLA-DPA1	HLA-DPA1.value > threshold=1.6885469555854797
node_861: feature_name=CD83	CD83.value > threshold=3.0563642978668213
node_1539: feature_name=ID1	ID1.value <= threshold=1.4626862406730652
node_1540: feature_name=FTH1	FTH1.value <= threshold=0.2985198348760605
node_1541: feature_name=SESN3	SESN3.value > threshold=2.7280690670013428
node_1587: feature_name=FAM129C	FAM129C.value <= threshold=3.4011322259902954
Class: COVID-19 Severe	
Rules_856	passed counts:5

node_0: feature_name=LTB	LTB.value <= threshold=0.994031548500061
node_1: feature_name=MTRNR2L2	MTRNR2L2.value <= threshold=2.8784297704696655
node_2: feature_name=TCL1A	TCL1A.value <= threshold=4.798312664031982
node_3: feature_name=MT-CO1	MT-CO1.value <= threshold=0.08947191759943962
node_4: feature_name=NR4A1	NR4A1.value <= threshold=1.0725348591804504
node_5: feature_name=HLA-DPA1	HLA-DPA1.value > threshold=1.6885469555854797
node_861: feature_name=CD83	CD83.value <= threshold=3.0563642978668213
node_862: feature_name=HLA-DQB1	HLA-DQB1.value > threshold=2.61081063747406
node_1358: feature_name=HLA-DRB5	HLA-DRB5.value > threshold=0.7542744874954224
node_1390: feature_name=HLA-DRB5	HLA-DRB5.value <= threshold=2.6244447231292725
node_1391: feature_name=XIST	XIST.value <= threshold=-0.22017984092235565
node_1392: feature_name=MT-CO3	MT-CO3.value <= threshold=0.12647542357444763
node_1393: feature_name=CD79B	CD79B.value <= threshold=4.338301658630371
node_1394: feature_name=LTB	LTB.value <= threshold=0.9865012466907501
node_1395: feature_name=MEF2C	MEF2C.value <= threshold=4.133931398391724
node_1396: feature_name=MEF2C	MEF2C.value > threshold=-0.38076959550380707
node_1418: feature_name=HLA-DPB1	HLA-DPB1.value > threshold=0.8961269855499268
node_1422: feature_name=HLA-DPA1	HLA-DPA1.value <= threshold=2.9194568395614624
node_1423: feature_name=IL1B	IL1B.value <= threshold=2.321333408355713
node_1424: feature_name=TRIB1	TRIB1.value <= threshold=4.043357253074646
node_1425: feature_name=NT5C3A	NT5C3A.value <= threshold=3.4580591917037964
node_1426: feature_name=MT1E	MT1E.value <= threshold=1.7696760445833206
node_1427: feature_name=SESN3	SESN3.value <= threshold=3.1229121685028076
node_1428: feature_name=HLA-DRB1	HLA-DRB1.value <= threshold=2.7549219131469727
node_1429: feature_name=OASL	OASL.value <= threshold=3.688907504081726
node_1430: feature_name=ITM2C	ITM2C.value <= threshold=3.4028767347335815
node_1431: feature_name=HLA-DRB1	HLA-DRB1.value > threshold=0.7038701176643372
node_1435: feature_name=ATF5	ATF5.value <= threshold=3.428827404975891
node_1436: feature_name=DAPP1	DAPP1.value <= threshold=5.408607244491577
node_1437: feature_name=PDLIM1	PDLIM1.value > threshold=4.337203502655029

node_1439: feature_name=IFI44L	IFI44L.value <= threshold=-0.3458602577447891
Class: COVID-19 Critical	
Rules_857	passed counts:5
node_0: feature_name=LTB	LTB.value <= threshold=0.994031548500061
node_1: feature_name=MTRNR2L2	MTRNR2L2.value <= threshold=2.8784297704696655
node_2: feature_name=TCL1A	TCL1A.value <= threshold=4.798312664031982
node_3: feature_name=MT-CO1	MT-CO1.value <= threshold=0.08947191759943962
node_4: feature_name=NR4A1	NR4A1.value <= threshold=1.0725348591804504
node_5: feature_name=HLA-DPA1	HLA-DPA1.value > threshold=1.6885469555854797
node_861: feature_name=CD83	CD83.value <= threshold=3.0563642978668213
node_862: feature_name=HLA-DQB1	HLA-DQB1.value > threshold=2.61081063747406
node_1358: feature_name=HLA-DRB5	HLA-DRB5.value > threshold=0.7542744874954224
node_1390: feature_name=HLA-DRB5	HLA-DRB5.value <= threshold=2.6244447231292725
node_1391: feature_name=XIST	XIST.value <= threshold=-0.22017984092235565
node_1392: feature_name=MT-CO3	MT-CO3.value <= threshold=0.12647542357444763
node_1393: feature_name=CD79B	CD79B.value <= threshold=4.338301658630371
node_1394: feature_name=LTB	LTB.value <= threshold=0.9865012466907501
node_1395: feature_name=MEF2C	MEF2C.value <= threshold=4.133931398391724
node_1396: feature_name=MEF2C	MEF2C.value <= threshold=-0.38076959550380707
node_1397: feature_name=UTY	UTY.value <= threshold=-0.4634757786989212
node_1398: feature_name=MT-CO1	MT-CO1.value > threshold=-0.8070613443851471
node_1402: feature_name=ATF3	ATF3.value > threshold=-0.4212478846311569
node_1404: feature_name=MT-CO2	MT-CO2.value <= threshold=-0.6675371527671814
Class: COVID-19 Severe	
Rules_858	passed counts:5
node_0: feature_name=LTB	LTB.value <= threshold=0.994031548500061
node_1: feature_name=MTRNR2L2	MTRNR2L2.value <= threshold=2.8784297704696655
node_2: feature_name=TCL1A	TCL1A.value <= threshold=4.798312664031982

node_3: feature_name=MT-CO1	MT-CO1.value <= threshold=0.08947191759943962
node_4: feature_name=NR4A1	NR4A1.value <= threshold=1.0725348591804504
node_5: feature_name=HLA-DPA1	HLA-DPA1.value > threshold=1.6885469555854797
node_861: feature_name=CD83	CD83.value <= threshold=3.0563642978668213
node_862: feature_name=HLA-DQB1	HLA-DQB1.value <= threshold=2.61081063747406
node_863: feature_name=MTRNR2L2	MTRNR2L2.value <= threshold=2.0919997692108154
node_864: feature_name=BCL2A1	BCL2A1.value <= threshold=1.852618932723999
node_865: feature_name=CD79B	CD79B.value <= threshold=4.352142333984375
node_866: feature_name=TNFAIP3	TNFAIP3.value <= threshold=-0.0449496041983366
node_867: feature_name=MT-CO1	MT-CO1.value > threshold=-0.08531957119703293
node_1155: feature_name=IRF8	IRF8.value > threshold=-0.39239931106567383
node_1177: feature_name=PELI1	PELI1.value > threshold=-0.6234017610549927
node_1203: feature_name=STMN1	STMN1.value > threshold=-0.2129739671945572
node_1229: feature_name=MT-CO1	MT-CO1.value > threshold=0.028881540521979332
Class: Healthy Control	
Rules_859	passed counts:5
node_0: feature_name=LTB	LTB.value <= threshold=0.994031548500061
node_1: feature_name=MTRNR2L2	MTRNR2L2.value <= threshold=2.8784297704696655
node_2: feature_name=TCL1A	TCL1A.value <= threshold=4.798312664031982
node_3: feature_name=MT-CO1	MT-CO1.value <= threshold=0.08947191759943962
node_4: feature_name=NR4A1	NR4A1.value <= threshold=1.0725348591804504
node_5: feature_name=HLA-DPA1	HLA-DPA1.value > threshold=1.6885469555854797
node_861: feature_name=CD83	CD83.value <= threshold=3.0563642978668213
node_862: feature_name=HLA-DQB1	HLA-DQB1.value <= threshold=2.61081063747406
node_863: feature_name=MTRNR2L2	MTRNR2L2.value <= threshold=2.0919997692108154
node_864: feature_name=BCL2A1	BCL2A1.value <= threshold=1.852618932723999
node_865: feature_name=CD79B	CD79B.value <= threshold=4.352142333984375
node_866: feature_name=TNFAIP3	TNFAIP3.value <= threshold=-0.0449496041983366
node_867: feature_name=MT-CO1	MT-CO1.value > threshold=-0.08531957119703293

node_1155: feature_name=IRF8	IRF8.value > threshold=-0.39239931106567383
node_1177: feature_name=PELI1	PELI1.value > threshold=-0.6234017610549927
node_1203: feature_name=STMN1	STMN1.value > threshold=-0.2129739671945572
node_1229: feature_name=MT-CO1	MT-CO1.value <= threshold=0.028881540521979332
node_1230: feature_name=JUN	JUN.value <= threshold=0.7718976438045502
Class: COVID-19 Critical	
Rules_860	passed counts:5
node_0: feature_name=LTB	LTB.value <= threshold=0.994031548500061
node_1: feature_name=MTRNR2L2	MTRNR2L2.value <= threshold=2.8784297704696655
node_2: feature_name=TCL1A	TCL1A.value <= threshold=4.798312664031982
node_3: feature_name=MT-CO1	MT-CO1.value <= threshold=0.08947191759943962
node_4: feature_name=NR4A1	NR4A1.value <= threshold=1.0725348591804504
node_5: feature_name=HLA-DPA1	HLA-DPA1.value > threshold=1.6885469555854797
node_861: feature_name=CD83	CD83.value <= threshold=3.0563642978668213
node_862: feature_name=HLA-DQB1	HLA-DQB1.value <= threshold=2.61081063747406
node_863: feature_name=MTRNR2L2	MTRNR2L2.value <= threshold=2.0919997692108154
node_864: feature_name=BCL2A1	BCL2A1.value <= threshold=1.852618932723999
node_865: feature_name=CD79B	CD79B.value <= threshold=4.352142333984375
node_866: feature_name=TNFAIP3	TNFAIP3.value <= threshold=-0.0449496041983366
node_867: feature_name=MT-CO1	MT-CO1.value > threshold=-0.08531957119703293
node_1155: feature_name=IRF8	IRF8.value > threshold=-0.39239931106567383
node_1177: feature_name=PELI1	PELI1.value > threshold=-0.6234017610549927
node_1203: feature_name=STMN1	STMN1.value <= threshold=-0.2129739671945572
node_1204: feature_name=HLA-DPA1	HLA-DPA1.value > threshold=1.6937929391860962
node_1210: feature_name=MT-CO2	MT-CO2.value <= threshold=-0.5078963339328766
node_1211: feature_name=OAZ1	OAZ1.value > threshold=-0.9088589698076248
node_1213: feature_name=LTB	LTB.value > threshold=0.30608798563480377
node_1215: feature_name=PELI1	PELI1.value <= threshold=1.7061399221420288
Class: Healthy Control	

Rules_861	passed counts:5
node_0: feature_name=LTB	LTB.value <= threshold=0.994031548500061
node_1: feature_name=MTRNR2L2	MTRNR2L2.value <= threshold=2.8784297704696655
node_2: feature_name=TCL1A	TCL1A.value <= threshold=4.798312664031982
node_3: feature_name=MT-CO1	MT-CO1.value <= threshold=0.08947191759943962
node_4: feature_name=NR4A1	NR4A1.value <= threshold=1.0725348591804504
node_5: feature_name=HLA-DPA1	HLA-DPA1.value > threshold=1.6885469555854797
node_861: feature_name=CD83	CD83.value <= threshold=3.0563642978668213
node_862: feature_name=HLA-DQB1	HLA-DQB1.value <= threshold=2.61081063747406
node_863: feature_name=MTRNR2L2	MTRNR2L2.value <= threshold=2.0919997692108154
node_864: feature_name=BCL2A1	BCL2A1.value <= threshold=1.852618932723999
node_865: feature_name=CD79B	CD79B.value <= threshold=4.352142333984375
node_866: feature_name=TNFAIP3	TNFAIP3.value <= threshold=-0.0449496041983366
node_867: feature_name=MT-CO1	MT-CO1.value > threshold=-0.08531957119703293
node_1155: feature_name=IRF8	IRF8.value > threshold=-0.39239931106567383
node_1177: feature_name=PELI1	PELI1.value <= threshold=-0.6234017610549927
node_1178: feature_name=HLA-DRB1	HLA-DRB1.value > threshold=1.3807418942451477
node_1182: feature_name=ANO6	ANO6.value <= threshold=-0.3352542072534561
node_1183: feature_name=MS4A1	MS4A1.value > threshold=1.8863030076026917
node_1187: feature_name=MT-CO2	MT-CO2.value > threshold=0.32131922245025635
Class: COVID-19 Critical	
Rules_862	passed counts:5
node_0: feature_name=LTB	LTB.value <= threshold=0.994031548500061
node_1: feature_name=MTRNR2L2	MTRNR2L2.value <= threshold=2.8784297704696655
node_2: feature_name=TCL1A	TCL1A.value <= threshold=4.798312664031982
node_3: feature_name=MT-CO1	MT-CO1.value <= threshold=0.08947191759943962
node_4: feature_name=NR4A1	NR4A1.value <= threshold=1.0725348591804504
node_5: feature_name=HLA-DPA1	HLA-DPA1.value > threshold=1.6885469555854797

node_861: feature_name=CD83	CD83.value <= threshold=3.0563642978668213
node_862: feature_name=HLA-DQB1	HLA-DQB1.value <= threshold=2.61081063747406
node_863: feature_name=MTRNR2L2	MTRNR2L2.value <= threshold=2.0919997692108154
node_864: feature_name=BCL2A1	BCL2A1.value <= threshold=1.852618932723999
node_865: feature_name=CD79B	CD79B.value <= threshold=4.352142333984375
node_866: feature_name=TNFAIP3	TNFAIP3.value <= threshold=-0.0449496041983366
node_867: feature_name=MT-CO1	MT-CO1.value > threshold=-0.08531957119703293
node_1155: feature_name=IRF8	IRF8.value <= threshold=-0.39239931106567383
node_1156: feature_name=OAZ1	OAZ1.value > threshold=0.027674595825374126
node_1168: feature_name=TNFAIP3	TNFAIP3.value > threshold=-0.6463479399681091
node_1172: feature_name=RP11-685N3.1	RP11-685N3.1.value <= threshold=-0.17028651386499405
Class: COVID-19 Critical	
Rules_863	passed counts:5
node_0: feature_name=LTB	LTB.value <= threshold=0.994031548500061
node_1: feature_name=MTRNR2L2	MTRNR2L2.value <= threshold=2.8784297704696655
node_2: feature_name=TCL1A	TCL1A.value <= threshold=4.798312664031982
node_3: feature_name=MT-CO1	MT-CO1.value <= threshold=0.08947191759943962
node_4: feature_name=NR4A1	NR4A1.value <= threshold=1.0725348591804504
node_5: feature_name=HLA-DPA1	HLA-DPA1.value > threshold=1.6885469555854797
node_861: feature_name=CD83	CD83.value <= threshold=3.0563642978668213
node_862: feature_name=HLA-DQB1	HLA-DQB1.value <= threshold=2.61081063747406
node_863: feature_name=MTRNR2L2	MTRNR2L2.value <= threshold=2.0919997692108154
node_864: feature_name=BCL2A1	BCL2A1.value <= threshold=1.852618932723999
node_865: feature_name=CD79B	CD79B.value <= threshold=4.352142333984375
node_866: feature_name=TNFAIP3	TNFAIP3.value <= threshold=-0.0449496041983366
node_867: feature_name=MT-CO1	MT-CO1.value <= threshold=-0.08531957119703293
node_868: feature_name=MTRNR2L8	MTRNR2L8.value <= threshold=0.8770350813865662
node_869: feature_name=EGR1	EGR1.value > threshold=-0.32270750403404236
node_1015: feature_name=NR4A3	NR4A3.value > threshold=1.9085431098937988

node_1143: feature_name=RP11-685N3.1	RP11-685N3.1.value <= threshold=-0.16600299626588821
Class: COVID-19 Severe	
Rules_864	passed counts:5
node_0: feature_name=LTB	LTB.value <= threshold=0.994031548500061
node_1: feature_name=MTRNR2L2	MTRNR2L2.value <= threshold=2.8784297704696655
node_2: feature_name=TCL1A	TCL1A.value <= threshold=4.798312664031982
node_3: feature_name=MT-CO1	MT-CO1.value <= threshold=0.08947191759943962
node_4: feature_name=NR4A1	NR4A1.value <= threshold=1.0725348591804504
node_5: feature_name=HLA-DPA1	HLA-DPA1.value > threshold=1.6885469555854797
node_861: feature_name=CD83	CD83.value <= threshold=3.0563642978668213
node_862: feature_name=HLA-DQB1	HLA-DQB1.value <= threshold=2.61081063747406
node_863: feature_name=MTRNR2L2	MTRNR2L2.value <= threshold=2.0919997692108154
node_864: feature_name=BCL2A1	BCL2A1.value <= threshold=1.852618932723999
node_865: feature_name=CD79B	CD79B.value <= threshold=4.352142333984375
node_866: feature_name=TNFAIP3	TNFAIP3.value <= threshold=-0.0449496041983366
node_867: feature_name=MT-CO1	MT-CO1.value <= threshold=-0.08531957119703293
node_868: feature_name=MTRNR2L8	MTRNR2L8.value <= threshold=0.8770350813865662
node_869: feature_name=EGR1	EGR1.value > threshold=-0.32270750403404236
node_1015: feature_name=NR4A3	NR4A3.value <= threshold=1.9085431098937988
node_1016: feature_name=ID3	ID3.value <= threshold=2.776647686958313
node_1017: feature_name=XIST	XIST.value <= threshold=2.618046522140503
node_1018: feature_name=CD79B	CD79B.value <= threshold=3.818994164466858
node_1019: feature_name=NFKBID	NFKBID.value > threshold=3.4191306829452515
node_1111: feature_name=RBKS	RBKS.value > threshold=-0.2493925839662552
Class: COVID-19 Severe	
Rules_865	passed counts:5
node_0: feature_name=LTB	LTB.value <= threshold=0.994031548500061
node_1: feature_name=MTRNR2L2	MTRNR2L2.value <= threshold=2.8784297704696655

node_2: feature_name=TCL1A	TCL1A.value <= threshold=4.798312664031982
node_3: feature_name=MT-CO1	MT-CO1.value <= threshold=0.08947191759943962
node_4: feature_name=NR4A1	NR4A1.value <= threshold=1.0725348591804504
node_5: feature_name=HLA-DPA1	HLA-DPA1.value > threshold=1.6885469555854797
node_861: feature_name=CD83	CD83.value <= threshold=3.0563642978668213
node_862: feature_name=HLA-DQB1	HLA-DQB1.value <= threshold=2.61081063747406
node_863: feature_name=MTRNR2L2	MTRNR2L2.value <= threshold=2.0919997692108154
node_864: feature_name=BCL2A1	BCL2A1.value <= threshold=1.852618932723999
node_865: feature_name=CD79B	CD79B.value <= threshold=4.352142333984375
node_866: feature_name=TNFAIP3	TNFAIP3.value <= threshold=-0.0449496041983366
node_867: feature_name=MT-CO1	MT-CO1.value <= threshold=-0.08531957119703293
node_868: feature_name=MTRNR2L8	MTRNR2L8.value <= threshold=0.8770350813865662
node_869: feature_name=EGR1	EGR1.value > threshold=-0.32270750403404236
node_1015: feature_name=NR4A3	NR4A3.value <= threshold=1.9085431098937988
node_1016: feature_name=ID3	ID3.value <= threshold=2.776647686958313
node_1017: feature_name=XIST	XIST.value <= threshold=2.618046522140503
node_1018: feature_name=CD79B	CD79B.value <= threshold=3.818994164466858
node_1019: feature_name=NFKBID	NFKBID.value <= threshold=3.4191306829452515
node_1020: feature_name=MIAT	MIAT.value <= threshold=3.532225251197815
node_1021: feature_name=STAG3	STAG3.value <= threshold=9.534917831420898
node_1022: feature_name=OASL	OASL.value <= threshold=2.3923760652542114
node_1023: feature_name=HLA-DQB1	HLA-DQB1.value <= threshold=2.572031259536743
node_1024: feature_name=CD79A	CD79A.value > threshold=0.1879405975341797
node_1028: feature_name=NR4A1	NR4A1.value <= threshold=0.7387348711490631
node_1029: feature_name=SLC7A5	SLC7A5.value <= threshold=2.0677570104599
node_1030: feature_name=HLA-DPB1	HLA-DPB1.value > threshold=2.17708420753479
node_1068: feature_name=FAM129C	FAM129C.value > threshold=1.734559953212738
node_1072: feature_name=PRDM1	PRDM1.value <= threshold=-0.4598921239376068
node_1073: feature_name=RGS1	RGS1.value > threshold=0.8619816601276398
Class: COVID-19 Critical	

Rules_866	passed counts:5
node_0: feature_name=LTB	LTB.value <= threshold=0.994031548500061
node_1: feature_name=MTRNR2L2	MTRNR2L2.value <= threshold=2.8784297704696655
node_2: feature_name=TCL1A	TCL1A.value <= threshold=4.798312664031982
node_3: feature_name=MT-CO1	MT-CO1.value <= threshold=0.08947191759943962
node_4: feature_name=NR4A1	NR4A1.value <= threshold=1.0725348591804504
node_5: feature_name=HLA-DPA1	HLA-DPA1.value > threshold=1.6885469555854797
node_861: feature_name=CD83	CD83.value <= threshold=3.0563642978668213
node_862: feature_name=HLA-DQB1	HLA-DQB1.value <= threshold=2.61081063747406
node_863: feature_name=MTRNR2L2	MTRNR2L2.value <= threshold=2.0919997692108154
node_864: feature_name=BCL2A1	BCL2A1.value <= threshold=1.852618932723999
node_865: feature_name=CD79B	CD79B.value <= threshold=4.352142333984375
node_866: feature_name=TNFAIP3	TNFAIP3.value <= threshold=-0.0449496041983366
node_867: feature_name=MT-CO1	MT-CO1.value <= threshold=-0.08531957119703293
node_868: feature_name=MTRNR2L8	MTRNR2L8.value <= threshold=0.8770350813865662
node_869: feature_name=EGR1	EGR1.value <= threshold=-0.32270750403404236
node_870: feature_name=IGJ	IGJ.value <= threshold=4.618318557739258
node_871: feature_name=DUSP4	DUSP4.value <= threshold=2.8197922706604004
node_872: feature_name=HOPX	HOPX.value <= threshold=2.10397732257843
node_873: feature_name=RGCC	RGCC.value <= threshold=1.2070720195770264
node_874: feature_name=OAZ1	OAZ1.value > threshold=1.02116060256958
node_980: feature_name=IFIT3	IFIT3.value <= threshold=-0.2093840092420578
node_981: feature_name=VCL	VCL.value <= threshold=-0.3414648026227951
node_982: feature_name=MS4A1	MS4A1.value <= threshold=3.399829626083374
Class: Healthy Control	
Rules_867	passed counts:5
node_0: feature_name=LTB	LTB.value <= threshold=0.994031548500061
node_1: feature_name=MTRNR2L2	MTRNR2L2.value <= threshold=2.8784297704696655

node_2: feature_name=TCL1A	TCL1A.value <= threshold=4.798312664031982
node_3: feature_name=MT-CO1	MT-CO1.value <= threshold=0.08947191759943962
node_4: feature_name=NR4A1	NR4A1.value <= threshold=1.0725348591804504
node_5: feature_name=HLA-DPA1	HLA-DPA1.value > threshold=1.6885469555854797
node_861: feature_name=CD83	CD83.value <= threshold=3.0563642978668213
node_862: feature_name=HLA-DQB1	HLA-DQB1.value <= threshold=2.61081063747406
node_863: feature_name=MTRNR2L2	MTRNR2L2.value <= threshold=2.0919997692108154
node_864: feature_name=BCL2A1	BCL2A1.value <= threshold=1.852618932723999
node_865: feature_name=CD79B	CD79B.value <= threshold=4.352142333984375
node_866: feature_name=TNFAIP3	TNFAIP3.value <= threshold=-0.0449496041983366
node_867: feature_name=MT-CO1	MT-CO1.value <= threshold=-0.08531957119703293
node_868: feature_name=MTRNR2L8	MTRNR2L8.value <= threshold=0.8770350813865662
node_869: feature_name=EGR1	EGR1.value <= threshold=-0.32270750403404236
node_870: feature_name=IGJ	IGJ.value <= threshold=4.618318557739258
node_871: feature_name=DUSP4	DUSP4.value <= threshold=2.8197922706604004
node_872: feature_name=HOPX	HOPX.value <= threshold=2.10397732257843
node_873: feature_name=RGCC	RGCC.value <= threshold=1.2070720195770264
node_874: feature_name=OAZ1	OAZ1.value <= threshold=1.02116060256958
node_875: feature_name=NAIP	NAIP.value <= threshold=3.768904685974121
node_876: feature_name=LDLR	LDLR.value <= threshold=2.9582698345184326
node_877: feature_name=HLA-DPA1	HLA-DPA1.value <= threshold=3.0588693618774414
node_878: feature_name=RHOB	RHOB.value <= threshold=5.08755898475647
node_879: feature_name=SYTL3	SYTL3.value <= threshold=1.5127440690994263
node_880: feature_name=FTH1	FTH1.value > threshold=-1.4152275919914246
node_882: feature_name=CCL3	CCL3.value <= threshold=2.253131687641144
node_883: feature_name=CD83	CD83.value <= threshold=2.8566592931747437
node_884: feature_name=HLA-DRB5	HLA-DRB5.value > threshold=-0.6464254856109619
node_888: feature_name=HSP90B1	HSP90B1.value > threshold=-1.186358630657196
node_892: feature_name=ATG2A	ATG2A.value <= threshold=2.3624722957611084
node_893: feature_name=ICAM1	ICAM1.value <= threshold=2.4306468963623047

node_894: feature_name=CD38	CD38.value <= threshold=3.4977954626083374
node_895: feature_name=HLA-DRB1	HLA-DRB1.value > threshold=0.28603747487068176
node_899: feature_name=XIST	XIST.value <= threshold=1.4272559583187103
node_900: feature_name=BANK1	BANK1.value <= threshold=5.060973644256592
node_901: feature_name=IRF4	IRF4.value <= threshold=6.117950916290283
node_902: feature_name=MTRNR2L2	MTRNR2L2.value <= threshold=1.8410876989364624
node_903: feature_name=TPM1	TPM1.value <= threshold=4.590091228485107
node_904: feature_name=SNORD3B-2	SNORD3B-2.value <= threshold=4.349201917648315
node_905: feature_name=AHR	AHR.value <= threshold=2.268427610397339
node_906: feature_name=OAZ1	OAZ1.value > threshold=-1.4369714260101318
node_914: feature_name=MT-ND6	MT-ND6.value > threshold=1.7712779641151428
node_924: feature_name=MARCKS	MARCKS.value > threshold=-0.32253237068653107
Class: COVID-19 Critical	
Rules_868	passed counts:5
node_0: feature_name=LTB	LTB.value <= threshold=0.994031548500061
node_1: feature_name=MTRNR2L2	MTRNR2L2.value <= threshold=2.8784297704696655
node_2: feature_name=TCL1A	TCL1A.value <= threshold=4.798312664031982
node_3: feature_name=MT-CO1	MT-CO1.value <= threshold=0.08947191759943962
node_4: feature_name=NR4A1	NR4A1.value <= threshold=1.0725348591804504
node_5: feature_name=HLA-DPA1	HLA-DPA1.value <= threshold=1.6885469555854797
node_6: feature_name=HLA-DRB5	HLA-DRB5.value > threshold=2.1886194944381714
node_738: feature_name=HLA-DQA2	HLA-DQA2.value <= threshold=-0.20105575770139694
node_739: feature_name=HLA-DQA1	HLA-DQA1.value > threshold=1.7414769530296326
node_763: feature_name=HLA-DRB5	HLA-DRB5.value <= threshold=2.6777851581573486
node_764: feature_name=ICAM1	ICAM1.value <= threshold=-0.3619510233402252
node_765: feature_name=HLA-DRB1	HLA-DRB1.value <= threshold=1.5085244178771973
Class: COVID-19 Severe	
Rules_869	passed counts:5

node_0: feature_name=LTB	LTB.value <= threshold=0.994031548500061
node_1: feature_name=MTRNR2L2	MTRNR2L2.value <= threshold=2.8784297704696655
node_2: feature_name=TCL1A	TCL1A.value <= threshold=4.798312664031982
node_3: feature_name=MT-CO1	MT-CO1.value <= threshold=0.08947191759943962
node_4: feature_name=NR4A1	NR4A1.value <= threshold=1.0725348591804504
node_5: feature_name=HLA-DPA1	HLA-DPA1.value <= threshold=1.6885469555854797
node_6: feature_name=HLA-DRB5	HLA-DRB5.value <= threshold=2.1886194944381714
node_7: feature_name=XIST	XIST.value <= threshold=-0.12328458577394485
node_8: feature_name=SYNE1	SYNE1.value > threshold=-0.7835594415664673
node_16: feature_name=MTRNR2L2	MTRNR2L2.value <= threshold=2.4483247995376587
node_17: feature_name=HLA-DQA2	HLA-DQA2.value > threshold=2.227178931236267
node_591: feature_name=BCL2A1	BCL2A1.value > threshold=1.0648428201675415
node_687: feature_name=CD79A	CD79A.value <= threshold=2.49657940864563
node_688: feature_name=MT-ND6	MT-ND6.value > threshold=0.16280143707990646
node_698: feature_name=PARP14	PARP14.value <= threshold=-0.49051935970783234
node_699: feature_name=RNF11	RNF11.value <= threshold=-0.4895625412464142
Class: COVID-19 Severe	
Rules_870	passed counts:5
node_0: feature_name=LTB	LTB.value <= threshold=0.994031548500061
node_1: feature_name=MTRNR2L2	MTRNR2L2.value <= threshold=2.8784297704696655
node_2: feature_name=TCL1A	TCL1A.value <= threshold=4.798312664031982
node_3: feature_name=MT-CO1	MT-CO1.value <= threshold=0.08947191759943962
node_4: feature_name=NR4A1	NR4A1.value <= threshold=1.0725348591804504
node_5: feature_name=HLA-DPA1	HLA-DPA1.value <= threshold=1.6885469555854797
node_6: feature_name=HLA-DRB5	HLA-DRB5.value <= threshold=2.1886194944381714
node_7: feature_name=XIST	XIST.value <= threshold=-0.12328458577394485
node_8: feature_name=SYNE1	SYNE1.value > threshold=-0.7835594415664673
node_16: feature_name=MTRNR2L2	MTRNR2L2.value <= threshold=2.4483247995376587
node_17: feature_name=HLA-DQA2	HLA-DQA2.value > threshold=2.227178931236267

node_591: feature_name=BCL2A1	BCL2A1.value <= threshold=1.0648428201675415
node_592: feature_name=FTH1	FTH1.value <= threshold=0.8842596709728241
node_593: feature_name=SNX9	SNX9.value <= threshold=3.232524275779724
node_594: feature_name=OAZ1	OAZ1.value <= threshold=1.704061508178711
node_595: feature_name=MT-CYB	MT-CYB.value <= threshold=0.5040059983730316
node_596: feature_name=CD83	CD83.value > threshold=2.535894751548767
node_638: feature_name=MTRNR2L8	MTRNR2L8.value > threshold=1.581753134727478
node_654: feature_name=CD79A	CD79A.value > threshold=1.777130126953125
Class: Healthy Control	
Rules_871	passed counts:5
node_0: feature_name=LTB	LTB.value <= threshold=0.994031548500061
node_1: feature_name=MTRNR2L2	MTRNR2L2.value <= threshold=2.8784297704696655
node_2: feature_name=TCL1A	TCL1A.value <= threshold=4.798312664031982
node_3: feature_name=MT-CO1	MT-CO1.value <= threshold=0.08947191759943962
node_4: feature_name=NR4A1	NR4A1.value <= threshold=1.0725348591804504
node_5: feature_name=HLA-DPA1	HLA-DPA1.value <= threshold=1.6885469555854797
node_6: feature_name=HLA-DRB5	HLA-DRB5.value <= threshold=2.1886194944381714
node_7: feature_name=XIST	XIST.value <= threshold=-0.12328458577394485
node_8: feature_name=SYNE1	SYNE1.value > threshold=-0.7835594415664673
node_16: feature_name=MTRNR2L2	MTRNR2L2.value <= threshold=2.4483247995376587
node_17: feature_name=HLA-DQA2	HLA-DQA2.value > threshold=2.227178931236267
node_591: feature_name=BCL2A1	BCL2A1.value <= threshold=1.0648428201675415
node_592: feature_name=FTH1	FTH1.value <= threshold=0.8842596709728241
node_593: feature_name=SNX9	SNX9.value <= threshold=3.232524275779724
node_594: feature_name=OAZ1	OAZ1.value <= threshold=1.704061508178711
node_595: feature_name=MT-CYB	MT-CYB.value <= threshold=0.5040059983730316
node_596: feature_name=CD83	CD83.value <= threshold=2.535894751548767
node_597: feature_name=ATF5	ATF5.value <= threshold=5.664362907409668
node_598: feature_name=IGLL5	IGLL5.value <= threshold=6.548790216445923

node_599: feature_name=EGR1	EGR1.value <= threshold=6.419021844863892
node_600: feature_name=PLEC	PLEC.value <= threshold=1.6971505880355835
node_601: feature_name=CD22	CD22.value <= threshold=6.541341304779053
node_602: feature_name=HSPA1A	HSPA1A.value <= threshold=3.400114059448242
node_603: feature_name=HBB	HBB.value <= threshold=6.7660768032073975
node_604: feature_name=FCRL1	FCRL1.value <= threshold=7.900821685791016
node_605: feature_name=STAG3	STAG3.value <= threshold=9.824122905731201
node_606: feature_name=SPI1	SPI1.value <= threshold=1.346154272556305
node_607: feature_name=TRIB1	TRIB1.value > threshold=2.0271907448768616
node_609: feature_name=HES4	HES4.value > threshold=-0.21790731698274612
Class: COVID-19 Critical	
Rules_872	passed counts:5
node_0: feature_name=LTB	LTB.value <= threshold=0.994031548500061
node_1: feature_name=MTRNR2L2	MTRNR2L2.value <= threshold=2.8784297704696655
node_2: feature_name=TCL1A	TCL1A.value <= threshold=4.798312664031982
node_3: feature_name=MT-CO1	MT-CO1.value <= threshold=0.08947191759943962
node_4: feature_name=NR4A1	NR4A1.value <= threshold=1.0725348591804504
node_5: feature_name=HLA-DPA1	HLA-DPA1.value <= threshold=1.6885469555854797
node_6: feature_name=HLA-DRB5	HLA-DRB5.value <= threshold=2.1886194944381714
node_7: feature_name=XIST	XIST.value <= threshold=-0.12328458577394485
node_8: feature_name=SYNE1	SYNE1.value > threshold=-0.7835594415664673
node_16: feature_name=MTRNR2L2	MTRNR2L2.value <= threshold=2.4483247995376587
node_17: feature_name=HLA-DQA2	HLA-DQA2.value <= threshold=2.227178931236267
node_18: feature_name=NR4A1	NR4A1.value > threshold=-0.21380723267793655
node_504: feature_name=LTB	LTB.value <= threshold=0.8758118152618408
node_505: feature_name=TNFAIP3	TNFAIP3.value > threshold=0.4779366999864578
node_581: feature_name=TCL1A	TCL1A.value <= threshold=3.0026209354400635
Class: COVID-19 Severe	

Rules_873	passed counts:5
node_0: feature_name=LTB	LTB.value <= threshold=0.994031548500061
node_1: feature_name=MTRNR2L2	MTRNR2L2.value <= threshold=2.8784297704696655
node_2: feature_name=TCL1A	TCL1A.value <= threshold=4.798312664031982
node_3: feature_name=MT-CO1	MT-CO1.value <= threshold=0.08947191759943962
node_4: feature_name=NR4A1	NR4A1.value <= threshold=1.0725348591804504
node_5: feature_name=HLA-DPA1	HLA-DPA1.value <= threshold=1.6885469555854797
node_6: feature_name=HLA-DRB5	HLA-DRB5.value <= threshold=2.1886194944381714
node_7: feature_name=XIST	XIST.value <= threshold=-0.12328458577394485
node_8: feature_name=SYNE1	SYNE1.value > threshold=-0.7835594415664673
node_16: feature_name=MTRNR2L2	MTRNR2L2.value <= threshold=2.4483247995376587
node_17: feature_name=HLA-DQA2	HLA-DQA2.value <= threshold=2.227178931236267
node_18: feature_name=NR4A1	NR4A1.value > threshold=-0.21380723267793655
node_504: feature_name=LTB	LTB.value <= threshold=0.8758118152618408
node_505: feature_name=TNFAIP3	TNFAIP3.value <= threshold=0.4779366999864578
node_506: feature_name=HLA-DQB1	HLA-DQB1.value <= threshold=2.9689468145370483
node_507: feature_name=MS4A1	MS4A1.value <= threshold=3.7428637742996216
node_508: feature_name=SNX9	SNX9.value <= threshold=3.304862380027771
node_509: feature_name=MARCKS	MARCKS.value > threshold=-0.519626647233963
node_513: feature_name=CD79B	CD79B.value > threshold=3.0890846252441406
node_563: feature_name=NR4A3	NR4A3.value > threshold=-0.4203310012817383
node_567: feature_name=HSP90B1	HSP90B1.value <= threshold=0.9940841794013977
Class: Healthy Control	
Rules_874	passed counts:5
node_0: feature_name=LTB	LTB.value <= threshold=0.994031548500061
node_1: feature_name=MTRNR2L2	MTRNR2L2.value <= threshold=2.8784297704696655
node_2: feature_name=TCL1A	TCL1A.value <= threshold=4.798312664031982
node_3: feature_name=MT-CO1	MT-CO1.value <= threshold=0.08947191759943962
node_4: feature_name=NR4A1	NR4A1.value <= threshold=1.0725348591804504

node_5: feature_name=HLA-DPA1	HLA-DPA1.value <= threshold=1.6885469555854797
node_6: feature_name=HLA-DRB5	HLA-DRB5.value <= threshold=2.1886194944381714
node_7: feature_name=XIST	XIST.value <= threshold=-0.12328458577394485
node_8: feature_name=SYNE1	SYNE1.value > threshold=-0.7835594415664673
node_16: feature_name=MTRNR2L2	MTRNR2L2.value <= threshold=2.4483247995376587
node_17: feature_name=HLA-DQA2	HLA-DQA2.value <= threshold=2.227178931236267
node_18: feature_name=NR4A1	NR4A1.value > threshold=-0.21380723267793655
node_504: feature_name=LTB	LTB.value <= threshold=0.8758118152618408
node_505: feature_name=TNFAIP3	TNFAIP3.value <= threshold=0.4779366999864578
node_506: feature_name=HLA-DQB1	HLA-DQB1.value <= threshold=2.9689468145370483
node_507: feature_name=MS4A1	MS4A1.value <= threshold=3.7428637742996216
node_508: feature_name=SNX9	SNX9.value <= threshold=3.304862380027771
node_509: feature_name=MARCKS	MARCKS.value > threshold=-0.519626647233963
node_513: feature_name=CD79B	CD79B.value <= threshold=3.0890846252441406
node_514: feature_name=GNG11	GNG11.value <= threshold=3.3202054500579834
node_515: feature_name=BANK1	BANK1.value > threshold=-0.3250492364168167
node_521: feature_name=VPREB3	VPREB3.value <= threshold=8.097821950912476
node_522: feature_name=IRF4	IRF4.value <= threshold=6.5894060134887695
node_523: feature_name=BCL2A1	BCL2A1.value <= threshold=2.7336734533309937
node_524: feature_name=HSPA1A	HSPA1A.value <= threshold=3.192288041114807
node_525: feature_name=MS4A1	MS4A1.value > threshold=-0.3445378392934799
node_529: feature_name=MS4A1	MS4A1.value <= threshold=3.4282803535461426
node_530: feature_name=NR4A3	NR4A3.value <= threshold=2.2900307178497314
node_531: feature_name=OAZ1	OAZ1.value <= threshold=0.8763319551944733
node_532: feature_name=JUN	JUN.value > threshold=-1.1919074058532715
node_536: feature_name=HOPX	HOPX.value <= threshold=1.6209625601768494
node_537: feature_name=IL1B	IL1B.value > threshold=0.27510731667280197
node_539: feature_name=RP11-731F5.2	RP11-731F5.2.value > threshold=-0.1954932063817978
Class: COVID-19 Critical	

Rules_875	passed counts:5
node_0: feature_name=LTB	LTB.value <= threshold=0.994031548500061
node_1: feature_name=MTRNR2L2	MTRNR2L2.value <= threshold=2.8784297704696655
node_2: feature_name=TCL1A	TCL1A.value <= threshold=4.798312664031982
node_3: feature_name=MT-CO1	MT-CO1.value <= threshold=0.08947191759943962
node_4: feature_name=NR4A1	NR4A1.value <= threshold=1.0725348591804504
node_5: feature_name=HLA-DPA1	HLA-DPA1.value <= threshold=1.6885469555854797
node_6: feature_name=HLA-DRB5	HLA-DRB5.value <= threshold=2.1886194944381714
node_7: feature_name=XIST	XIST.value <= threshold=-0.12328458577394485
node_8: feature_name=SYNE1	SYNE1.value > threshold=-0.7835594415664673
node_16: feature_name=MTRNR2L2	MTRNR2L2.value <= threshold=2.4483247995376587
node_17: feature_name=HLA-DQA2	HLA-DQA2.value <= threshold=2.227178931236267
node_18: feature_name=NR4A1	NR4A1.value > threshold=-0.21380723267793655
node_504: feature_name=LTB	LTB.value <= threshold=0.8758118152618408
node_505: feature_name=TNFAIP3	TNFAIP3.value <= threshold=0.4779366999864578
node_506: feature_name=HLA-DQB1	HLA-DQB1.value <= threshold=2.9689468145370483
node_507: feature_name=MS4A1	MS4A1.value <= threshold=3.7428637742996216
node_508: feature_name=SNX9	SNX9.value <= threshold=3.304862380027771
node_509: feature_name=MARCKS	MARCKS.value > threshold=-0.519626647233963
node_513: feature_name=CD79B	CD79B.value <= threshold=3.0890846252441406
node_514: feature_name=GNG11	GNG11.value <= threshold=3.3202054500579834
node_515: feature_name=BANK1	BANK1.value > threshold=-0.3250492364168167
node_521: feature_name=VPREB3	VPREB3.value <= threshold=8.097821950912476
node_522: feature_name=IRF4	IRF4.value <= threshold=6.5894060134887695
node_523: feature_name=BCL2A1	BCL2A1.value <= threshold=2.7336734533309937
node_524: feature_name=HSPA1A	HSPA1A.value <= threshold=3.192288041114807
node_525: feature_name=MS4A1	MS4A1.value <= threshold=-0.3445378392934799
node_526: feature_name=COTL1	COTL1.value > threshold=-0.6093726754188538
Class: COVID-19 Critical	

Rules_876	passed counts:5
node_0: feature_name=LTB	LTB.value <= threshold=0.994031548500061
node_1: feature_name=MTRNR2L2	MTRNR2L2.value <= threshold=2.8784297704696655
node_2: feature_name=TCL1A	TCL1A.value <= threshold=4.798312664031982
node_3: feature_name=MT-CO1	MT-CO1.value <= threshold=0.08947191759943962
node_4: feature_name=NR4A1	NR4A1.value <= threshold=1.0725348591804504
node_5: feature_name=HLA-DPA1	HLA-DPA1.value <= threshold=1.6885469555854797
node_6: feature_name=HLA-DRB5	HLA-DRB5.value <= threshold=2.1886194944381714
node_7: feature_name=XIST	XIST.value <= threshold=-0.12328458577394485
node_8: feature_name=SYNE1	SYNE1.value > threshold=-0.7835594415664673
node_16: feature_name=MTRNR2L2	MTRNR2L2.value <= threshold=2.4483247995376587
node_17: feature_name=HLA-DQA2	HLA-DQA2.value <= threshold=2.227178931236267
node_18: feature_name=NR4A1	NR4A1.value > threshold=-0.21380723267793655
node_504: feature_name=LTB	LTB.value <= threshold=0.8758118152618408
node_505: feature_name=TNFAIP3	TNFAIP3.value <= threshold=0.4779366999864578
node_506: feature_name=HLA-DQB1	HLA-DQB1.value <= threshold=2.9689468145370483
node_507: feature_name=MS4A1	MS4A1.value <= threshold=3.7428637742996216
node_508: feature_name=SNX9	SNX9.value <= threshold=3.304862380027771
node_509: feature_name=MARCKS	MARCKS.value <= threshold=-0.519626647233963
node_510: feature_name=TCL1A	TCL1A.value <= threshold=-0.1625603437423706
Class: COVID-19 Severe	
Rules_877	passed counts:5
node_0: feature_name=LTB	LTB.value <= threshold=0.994031548500061
node_1: feature_name=MTRNR2L2	MTRNR2L2.value <= threshold=2.8784297704696655
node_2: feature_name=TCL1A	TCL1A.value <= threshold=4.798312664031982
node_3: feature_name=MT-CO1	MT-CO1.value <= threshold=0.08947191759943962
node_4: feature_name=NR4A1	NR4A1.value <= threshold=1.0725348591804504
node_5: feature_name=HLA-DPA1	HLA-DPA1.value <= threshold=1.6885469555854797
node_6: feature_name=HLA-DRB5	HLA-DRB5.value <= threshold=2.1886194944381714

node_7: feature_name=XIST	XIST.value <= threshold=-0.12328458577394485
node_8: feature_name=SYNE1	SYNE1.value > threshold=-0.7835594415664673
node_16: feature_name=MTRNR2L2	MTRNR2L2.value <= threshold=2.4483247995376587
node_17: feature_name=HLA-DQA2	HLA-DQA2.value <= threshold=2.227178931236267
node_18: feature_name=NR4A1	NR4A1.value <= threshold=-0.21380723267793655
node_19: feature_name=IRAK3	IRAK3.value <= threshold=4.27159571647644
node_20: feature_name=IFIT2	IFIT2.value <= threshold=7.8175740242004395
node_21: feature_name=CD79A	CD79A.value > threshold=2.857242226600647
node_375: feature_name=LTB	LTB.value <= threshold=0.7405790388584137
node_376: feature_name=NFKBIZ	NFKBIZ.value <= threshold=1.7718515396118164
node_377: feature_name=OAZ1	OAZ1.value > threshold=1.3302325010299683
node_465: feature_name=CD22	CD22.value <= threshold=3.588951587677002
Class: COVID-19 Critical	
Rules_878	passed counts:5
node_0: feature_name=LTB	LTB.value <= threshold=0.994031548500061
node_1: feature_name=MTRNR2L2	MTRNR2L2.value <= threshold=2.8784297704696655
node_2: feature_name=TCL1A	TCL1A.value <= threshold=4.798312664031982
node_3: feature_name=MT-CO1	MT-CO1.value <= threshold=0.08947191759943962
node_4: feature_name=NR4A1	NR4A1.value <= threshold=1.0725348591804504
node_5: feature_name=HLA-DPA1	HLA-DPA1.value <= threshold=1.6885469555854797
node_6: feature_name=HLA-DRB5	HLA-DRB5.value <= threshold=2.1886194944381714
node_7: feature_name=XIST	XIST.value <= threshold=-0.12328458577394485
node_8: feature_name=SYNE1	SYNE1.value > threshold=-0.7835594415664673
node_16: feature_name=MTRNR2L2	MTRNR2L2.value <= threshold=2.4483247995376587
node_17: feature_name=HLA-DQA2	HLA-DQA2.value <= threshold=2.227178931236267
node_18: feature_name=NR4A1	NR4A1.value <= threshold=-0.21380723267793655
node_19: feature_name=IRAK3	IRAK3.value <= threshold=4.27159571647644
node_20: feature_name=IFIT2	IFIT2.value <= threshold=7.8175740242004395
node_21: feature_name=CD79A	CD79A.value > threshold=2.857242226600647

node_375: feature_name=LTB	LTB.value <= threshold=0.7405790388584137
node_376: feature_name=NFKBIZ	NFKBIZ.value <= threshold=1.7718515396118164
node_377: feature_name=OAZ1	OAZ1.value <= threshold=1.3302325010299683
node_378: feature_name=RHOB	RHOB.value > threshold=3.0459729433059692
node_456: feature_name=CD83	CD83.value > threshold=2.2091671228408813
node_462: feature_name=CCL3	CCL3.value <= threshold=-0.3615690618753433
Class: COVID-19 Severe	
Rules_879	passed counts:5
node_0: feature_name=LTB	LTB.value <= threshold=0.994031548500061
node_1: feature_name=MTRNR2L2	MTRNR2L2.value <= threshold=2.8784297704696655
node_2: feature_name=TCL1A	TCL1A.value <= threshold=4.798312664031982
node_3: feature_name=MT-CO1	MT-CO1.value <= threshold=0.08947191759943962
node_4: feature_name=NR4A1	NR4A1.value <= threshold=1.0725348591804504
node_5: feature_name=HLA-DPA1	HLA-DPA1.value <= threshold=1.6885469555854797
node_6: feature_name=HLA-DRB5	HLA-DRB5.value <= threshold=2.1886194944381714
node_7: feature_name=XIST	XIST.value <= threshold=-0.12328458577394485
node_8: feature_name=SYNE1	SYNE1.value > threshold=-0.7835594415664673
node_16: feature_name=MTRNR2L2	MTRNR2L2.value <= threshold=2.4483247995376587
node_17: feature_name=HLA-DQA2	HLA-DQA2.value <= threshold=2.227178931236267
node_18: feature_name=NR4A1	NR4A1.value <= threshold=-0.21380723267793655
node_19: feature_name=IRAK3	IRAK3.value <= threshold=4.27159571647644
node_20: feature_name=IFIT2	IFIT2.value <= threshold=7.8175740242004395
node_21: feature_name=CD79A	CD79A.value > threshold=2.857242226600647
node_375: feature_name=LTB	LTB.value <= threshold=0.7405790388584137
node_376: feature_name=NFKBIZ	NFKBIZ.value <= threshold=1.7718515396118164
node_377: feature_name=OAZ1	OAZ1.value <= threshold=1.3302325010299683
node_378: feature_name=RHOB	RHOB.value <= threshold=3.0459729433059692
node_379: feature_name=MT-CO3	MT-CO3.value <= threshold=1.6619641780853271
node_380: feature_name=MTRNR2L2	MTRNR2L2.value <= threshold=2.1229071617126465

node_381: feature_name=MAP3K2	MAP3K2.value <= threshold=3.626914143562317
node_382: feature_name=LDLR	LDLR.value <= threshold=2.571519374847412
node_383: feature_name=HLA-DRB1	HLA-DRB1.value > threshold=-0.7626054286956787
node_385: feature_name=CD79A	CD79A.value > threshold=2.857717990875244
node_387: feature_name=FCRL1	FCRL1.value <= threshold=6.850295543670654
node_388: feature_name=PDLIM1	PDLIM1.value > threshold=4.4097795486450195
node_442: feature_name=MS4A1	MS4A1.value > threshold=1.781447410583496
Class: COVID-19 Critical	
Rules_880	passed counts:5
node_0: feature_name=LTB	LTB.value <= threshold=0.994031548500061
node_1: feature_name=MTRNR2L2	MTRNR2L2.value <= threshold=2.8784297704696655
node_2: feature_name=TCL1A	TCL1A.value <= threshold=4.798312664031982
node_3: feature_name=MT-CO1	MT-CO1.value <= threshold=0.08947191759943962
node_4: feature_name=NR4A1	NR4A1.value <= threshold=1.0725348591804504
node_5: feature_name=HLA-DPA1	HLA-DPA1.value <= threshold=1.6885469555854797
node_6: feature_name=HLA-DRB5	HLA-DRB5.value <= threshold=2.1886194944381714
node_7: feature_name=XIST	XIST.value <= threshold=-0.12328458577394485
node_8: feature_name=SYNE1	SYNE1.value > threshold=-0.7835594415664673
node_16: feature_name=MTRNR2L2	MTRNR2L2.value <= threshold=2.4483247995376587
node_17: feature_name=HLA-DQA2	HLA-DQA2.value <= threshold=2.227178931236267
node_18: feature_name=NR4A1	NR4A1.value <= threshold=-0.21380723267793655
node_19: feature_name=IRAK3	IRAK3.value <= threshold=4.27159571647644
node_20: feature_name=IFIT2	IFIT2.value <= threshold=7.8175740242004395
node_21: feature_name=CD79A	CD79A.value > threshold=2.857242226600647
node_375: feature_name=LTB	LTB.value <= threshold=0.7405790388584137
node_376: feature_name=NFKBIZ	NFKBIZ.value <= threshold=1.7718515396118164
node_377: feature_name=OAZ1	OAZ1.value <= threshold=1.3302325010299683
node_378: feature_name=RHOB	RHOB.value <= threshold=3.0459729433059692
node_379: feature_name=MT-CO3	MT-CO3.value <= threshold=1.6619641780853271

node_380: feature_name=MTRNR2L2	MTRNR2L2.value <= threshold=2.1229071617126465
node_381: feature_name=MAP3K2	MAP3K2.value <= threshold=3.626914143562317
node_382: feature_name=LDLR	LDLR.value <= threshold=2.571519374847412
node_383: feature_name=HLA-DRB1	HLA-DRB1.value > threshold=-0.7626054286956787
node_385: feature_name=CD79A	CD79A.value > threshold=2.857717990875244
node_387: feature_name=FCRL1	FCRL1.value <= threshold=6.850295543670654
node_388: feature_name=PDLIM1	PDLIM1.value <= threshold=4.4097795486450195
node_389: feature_name=MARCH1	MARCH1.value > threshold=-0.35520173609256744
node_413: feature_name=RP11-731F5.2	RP11-731F5.2.value <= threshold=6.423685789108276
node_414: feature_name=IL1B	IL1B.value <= threshold=1.5673747062683105
node_415: feature_name=MARCKS	MARCKS.value <= threshold=4.112927675247192
node_416: feature_name=TCF4	TCF4.value <= threshold=5.713138103485107
node_417: feature_name=HLA-DPB1	HLA-DPB1.value <= threshold=2.425311803817749
node_418: feature_name=CD79B	CD79B.value > threshold=4.059849739074707
node_424: feature_name=GRASP	GRASP.value <= threshold=0.8325297683477402
Class: COVID-19 Critical	
Rules_881	passed counts:5
node_0: feature_name=LTB	LTB.value <= threshold=0.994031548500061
node_1: feature_name=MTRNR2L2	MTRNR2L2.value <= threshold=2.8784297704696655
node_2: feature_name=TCL1A	TCL1A.value <= threshold=4.798312664031982
node_3: feature_name=MT-CO1	MT-CO1.value <= threshold=0.08947191759943962
node_4: feature_name=NR4A1	NR4A1.value <= threshold=1.0725348591804504
node_5: feature_name=HLA-DPA1	HLA-DPA1.value <= threshold=1.6885469555854797
node_6: feature_name=HLA-DRB5	HLA-DRB5.value <= threshold=2.1886194944381714
node_7: feature_name=XIST	XIST.value <= threshold=-0.12328458577394485
node_8: feature_name=SYNE1	SYNE1.value > threshold=-0.7835594415664673
node_16: feature_name=MTRNR2L2	MTRNR2L2.value <= threshold=2.4483247995376587
node_17: feature_name=HLA-DQA2	HLA-DQA2.value <= threshold=2.227178931236267
node_18: feature_name=NR4A1	NR4A1.value <= threshold=-0.21380723267793655

node_19: feature_name=IRAK3	IRAK3.value <= threshold=4.27159571647644
node_20: feature_name=IFIT2	IFIT2.value <= threshold=7.8175740242004395
node_21: feature_name=CD79A	CD79A.value > threshold=2.857242226600647
node_375: feature_name=LTB	LTB.value <= threshold=0.7405790388584137
node_376: feature_name=NFKBIZ	NFKBIZ.value <= threshold=1.7718515396118164
node_377: feature_name=OAZ1	OAZ1.value <= threshold=1.3302325010299683
node_378: feature_name=RHOB	RHOB.value <= threshold=3.0459729433059692
node_379: feature_name=MT-CO3	MT-CO3.value <= threshold=1.6619641780853271
node_380: feature_name=MTRNR2L2	MTRNR2L2.value <= threshold=2.1229071617126465
node_381: feature_name=MAP3K2	MAP3K2.value <= threshold=3.626914143562317
node_382: feature_name=LDLR	LDLR.value <= threshold=2.571519374847412
node_383: feature_name=HLA-DRB1	HLA-DRB1.value > threshold=-0.7626054286956787
node_385: feature_name=CD79A	CD79A.value > threshold=2.857717990875244
node_387: feature_name=FCRL1	FCRL1.value <= threshold=6.850295543670654
node_388: feature_name=PDLIM1	PDLIM1.value <= threshold=4.4097795486450195
node_389: feature_name=MARCH1	MARCH1.value > threshold=-0.35520173609256744
node_413: feature_name=RP11-731F5.2	RP11-731F5.2.value <= threshold=6.423685789108276
node_414: feature_name=IL1B	IL1B.value <= threshold=1.5673747062683105
node_415: feature_name=MARCKS	MARCKS.value <= threshold=4.112927675247192
node_416: feature_name=TCF4	TCF4.value <= threshold=5.713138103485107
node_417: feature_name=HLA-DPB1	HLA-DPB1.value <= threshold=2.425311803817749
node_418: feature_name=CD79B	CD79B.value <= threshold=4.059849739074707
node_419: feature_name=MEF2C	MEF2C.value > threshold=3.9403620958328247
node_421: feature_name=PDLIM1	PDLIM1.value > threshold=-0.2511497735977173
Class: COVID-19 Critical	
Rules_882	passed counts:5
node_0: feature_name=LTB	LTB.value <= threshold=0.994031548500061
node_1: feature_name=MTRNR2L2	MTRNR2L2.value <= threshold=2.8784297704696655
node_2: feature_name=TCL1A	TCL1A.value <= threshold=4.798312664031982

node_3: feature_name=MT-CO1	MT-CO1.value <= threshold=0.08947191759943962
node_4: feature_name=NR4A1	NR4A1.value <= threshold=1.0725348591804504
node_5: feature_name=HLA-DPA1	HLA-DPA1.value <= threshold=1.6885469555854797
node_6: feature_name=HLA-DRB5	HLA-DRB5.value <= threshold=2.1886194944381714
node_7: feature_name=XIST	XIST.value <= threshold=-0.12328458577394485
node_8: feature_name=SYNE1	SYNE1.value > threshold=-0.7835594415664673
node_16: feature_name=MTRNR2L2	MTRNR2L2.value <= threshold=2.4483247995376587
node_17: feature_name=HLA-DQA2	HLA-DQA2.value <= threshold=2.227178931236267
node_18: feature_name=NR4A1	NR4A1.value <= threshold=-0.21380723267793655
node_19: feature_name=IRAK3	IRAK3.value <= threshold=4.27159571647644
node_20: feature_name=IFIT2	IFIT2.value <= threshold=7.8175740242004395
node_21: feature_name=CD79A	CD79A.value <= threshold=2.857242226600647
node_22: feature_name=HIST1H2AC	HIST1H2AC.value <= threshold=5.077470302581787
node_23: feature_name=FTH1	FTH1.value > threshold=0.4158056080341339
node_285: feature_name=EGR1	EGR1.value <= threshold=4.240467071533203
node_286: feature_name=HLA-DRB1	HLA-DRB1.value > threshold=-0.7553382813930511
node_290: feature_name=TLR2	TLR2.value <= threshold=2.879140257835388
node_291: feature_name=SYNE1	SYNE1.value <= threshold=2.323199510574341
node_292: feature_name=BCL2A1	BCL2A1.value > threshold=1.8940600156784058
node_352: feature_name=HLA-DQB1	HLA-DQB1.value <= threshold=1.2327778339385986
node_353: feature_name=IFIT3	IFIT3.value <= threshold=-0.18512452393770218
Class: COVID-19 Severe	
Rules_883	passed counts:5
node_0: feature_name=LTB	LTB.value <= threshold=0.994031548500061
node_1: feature_name=MTRNR2L2	MTRNR2L2.value <= threshold=2.8784297704696655
node_2: feature_name=TCL1A	TCL1A.value <= threshold=4.798312664031982
node_3: feature_name=MT-CO1	MT-CO1.value <= threshold=0.08947191759943962
node_4: feature_name=NR4A1	NR4A1.value <= threshold=1.0725348591804504
node_5: feature_name=HLA-DPA1	HLA-DPA1.value <= threshold=1.6885469555854797

node_6: feature_name=HLA-DRB5	HLA-DRB5.value <= threshold=2.1886194944381714
node_7: feature_name=XIST	XIST.value <= threshold=-0.12328458577394485
node_8: feature_name=SYNE1	SYNE1.value > threshold=-0.7835594415664673
node_16: feature_name=MTRNR2L2	MTRNR2L2.value <= threshold=2.4483247995376587
node_17: feature_name=HLA-DQA2	HLA-DQA2.value <= threshold=2.227178931236267
node_18: feature_name=NR4A1	NR4A1.value <= threshold=-0.21380723267793655
node_19: feature_name=IRAK3	IRAK3.value <= threshold=4.27159571647644
node_20: feature_name=IFIT2	IFIT2.value <= threshold=7.8175740242004395
node_21: feature_name=CD79A	CD79A.value <= threshold=2.857242226600647
node_22: feature_name=HIST1H2AC	HIST1H2AC.value <= threshold=5.077470302581787
node_23: feature_name=FTH1	FTH1.value > threshold=0.4158056080341339
node_285: feature_name=EGR1	EGR1.value <= threshold=4.240467071533203
node_286: feature_name=HLA-DRB1	HLA-DRB1.value > threshold=-0.7553382813930511
node_290: feature_name=TLR2	TLR2.value <= threshold=2.879140257835388
node_291: feature_name=SYNE1	SYNE1.value <= threshold=2.323199510574341
node_292: feature_name=BCL2A1	BCL2A1.value <= threshold=1.8940600156784058
node_293: feature_name=RALGPS2	RALGPS2.value <= threshold=4.808189153671265
node_294: feature_name=IGLL5	IGLL5.value <= threshold=4.116017818450928
node_295: feature_name=FTH1	FTH1.value > threshold=0.41612209379673004
node_297: feature_name=MCTP1	MCTP1.value <= threshold=4.601342678070068
node_298: feature_name=MT-CO2	MT-CO2.value <= threshold=0.6201252341270447
node_299: feature_name=MARCKS	MARCKS.value <= threshold=3.471073269844055
node_300: feature_name=PPIF	PPIF.value <= threshold=2.8470659255981445
node_301: feature_name=CCL3	CCL3.value <= threshold=1.9901156425476074
node_302: feature_name=MT-CO2	MT-CO2.value > threshold=-2.867768883705139
node_306: feature_name=RHOB	RHOB.value <= threshold=3.9839000701904297
node_307: feature_name=SNX29P2	SNX29P2.value <= threshold=5.035826921463013
node_308: feature_name=MT-CO3	MT-CO3.value <= threshold=0.7808255851268768
node_309: feature_name=RGCC	RGCC.value > threshold=1.070733904838562
node_319: feature_name=RNF144B	RNF144B.value > threshold=-0.2801654040813446

Class: COVID-19 Critical	
Rules_884	passed counts:5
node_0: feature_name=LTB	LTB.value <= threshold=0.994031548500061
node_1: feature_name=MTRNR2L2	MTRNR2L2.value <= threshold=2.8784297704696655
node_2: feature_name=TCL1A	TCL1A.value <= threshold=4.798312664031982
node_3: feature_name=MT-CO1	MT-CO1.value <= threshold=0.08947191759943962
node_4: feature_name=NR4A1	NR4A1.value <= threshold=1.0725348591804504
node_5: feature_name=HLA-DPA1	HLA-DPA1.value <= threshold=1.6885469555854797
node_6: feature_name=HLA-DRB5	HLA-DRB5.value <= threshold=2.1886194944381714
node_7: feature_name=XIST	XIST.value <= threshold=-0.12328458577394485
node_8: feature_name=SYNE1	SYNE1.value > threshold=-0.7835594415664673
node_16: feature_name=MTRNR2L2	MTRNR2L2.value <= threshold=2.4483247995376587
node_17: feature_name=HLA-DQA2	HLA-DQA2.value <= threshold=2.227178931236267
node_18: feature_name=NR4A1	NR4A1.value <= threshold=-0.21380723267793655
node_19: feature_name=IRAK3	IRAK3.value <= threshold=4.27159571647644
node_20: feature_name=IFIT2	IFIT2.value <= threshold=7.8175740242004395
node_21: feature_name=CD79A	CD79A.value <= threshold=2.857242226600647
node_22: feature_name=HIST1H2AC	HIST1H2AC.value <= threshold=5.077470302581787
node_23: feature_name=FTH1	FTH1.value <= threshold=0.4158056080341339
node_24: feature_name=ATF3	ATF3.value <= threshold=3.8028353452682495
node_25: feature_name=OASL	OASL.value <= threshold=3.387580633163452
node_26: feature_name=MT-CO1	MT-CO1.value > threshold=-0.7942646145820618
node_74: feature_name=HOPX	HOPX.value > threshold=0.8682331740856171
node_250: feature_name=CD22	CD22.value > threshold=-0.23878339678049088
node_254: feature_name=MT-CO2	MT-CO2.value > threshold=0.39438967406749725
node_274: feature_name=CD79A	CD79A.value > threshold=2.507172465324402
Class: Healthy Control	
Rules_885	passed counts:5

node_0: feature_name=LTB	LTB.value <= threshold=0.994031548500061
node_1: feature_name=MTRNR2L2	MTRNR2L2.value <= threshold=2.8784297704696655
node_2: feature_name=TCL1A	TCL1A.value <= threshold=4.798312664031982
node_3: feature_name=MT-CO1	MT-CO1.value <= threshold=0.08947191759943962
node_4: feature_name=NR4A1	NR4A1.value <= threshold=1.0725348591804504
node_5: feature_name=HLA-DPA1	HLA-DPA1.value <= threshold=1.6885469555854797
node_6: feature_name=HLA-DRB5	HLA-DRB5.value <= threshold=2.1886194944381714
node_7: feature_name=XIST	XIST.value <= threshold=-0.12328458577394485
node_8: feature_name=SYNE1	SYNE1.value > threshold=-0.7835594415664673
node_16: feature_name=MTRNR2L2	MTRNR2L2.value <= threshold=2.4483247995376587
node_17: feature_name=HLA-DQA2	HLA-DQA2.value <= threshold=2.227178931236267
node_18: feature_name=NR4A1	NR4A1.value <= threshold=-0.21380723267793655
node_19: feature_name=IRAK3	IRAK3.value <= threshold=4.27159571647644
node_20: feature_name=IFIT2	IFIT2.value <= threshold=7.8175740242004395
node_21: feature_name=CD79A	CD79A.value <= threshold=2.857242226600647
node_22: feature_name=HIST1H2AC	HIST1H2AC.value <= threshold=5.077470302581787
node_23: feature_name=FTH1	FTH1.value <= threshold=0.4158056080341339
node_24: feature_name=ATF3	ATF3.value <= threshold=3.8028353452682495
node_25: feature_name=OASL	OASL.value <= threshold=3.387580633163452
node_26: feature_name=MT-CO1	MT-CO1.value > threshold=-0.7942646145820618
node_74: feature_name=HOPX	HOPX.value <= threshold=0.8682331740856171
node_75: feature_name=RGS2	RGS2.value <= threshold=1.4589954018592834
node_76: feature_name=AHR	AHR.value <= threshold=3.870481848716736
node_77: feature_name=IGJ	IGJ.value <= threshold=4.902882814407349
node_78: feature_name=CD79B	CD79B.value <= threshold=4.87716269493103
node_79: feature_name=HLA-DQB1	HLA-DQB1.value <= threshold=3.6925491094589233
node_80: feature_name=HLA-DRB5	HLA-DRB5.value > threshold=2.0926843881607056
node_204: feature_name=DAPP1	DAPP1.value <= threshold=-0.3013480007648468
node_205: feature_name=SESN3	SESN3.value <= threshold=1.3210760951042175
Class: COVID-19 Severe	

Rules_886	passed counts:5
node_0: feature_name=LTB	LTB.value <= threshold=0.994031548500061
node_1: feature_name=MTRNR2L2	MTRNR2L2.value <= threshold=2.8784297704696655
node_2: feature_name=TCL1A	TCL1A.value <= threshold=4.798312664031982
node_3: feature_name=MT-CO1	MT-CO1.value <= threshold=0.08947191759943962
node_4: feature_name=NR4A1	NR4A1.value <= threshold=1.0725348591804504
node_5: feature_name=HLA-DPA1	HLA-DPA1.value <= threshold=1.6885469555854797
node_6: feature_name=HLA-DRB5	HLA-DRB5.value <= threshold=2.1886194944381714
node_7: feature_name=XIST	XIST.value <= threshold=-0.12328458577394485
node_8: feature_name=SYNE1	SYNE1.value > threshold=-0.7835594415664673
node_16: feature_name=MTRNR2L2	MTRNR2L2.value <= threshold=2.4483247995376587
node_17: feature_name=HLA-DQA2	HLA-DQA2.value <= threshold=2.227178931236267
node_18: feature_name=NR4A1	NR4A1.value <= threshold=-0.21380723267793655
node_19: feature_name=IRAK3	IRAK3.value <= threshold=4.27159571647644
node_20: feature_name=IFIT2	IFIT2.value <= threshold=7.8175740242004395
node_21: feature_name=CD79A	CD79A.value <= threshold=2.857242226600647
node_22: feature_name=HIST1H2AC	HIST1H2AC.value <= threshold=5.077470302581787
node_23: feature_name=FTH1	FTH1.value <= threshold=0.4158056080341339
node_24: feature_name=ATF3	ATF3.value <= threshold=3.8028353452682495
node_25: feature_name=OASL	OASL.value <= threshold=3.387580633163452
node_26: feature_name=MT-CO1	MT-CO1.value > threshold=-0.7942646145820618
node_74: feature_name=HOPX	HOPX.value <= threshold=0.8682331740856171
node_75: feature_name=RGS2	RGS2.value <= threshold=1.4589954018592834
node_76: feature_name=AHR	AHR.value <= threshold=3.870481848716736
node_77: feature_name=IGJ	IGJ.value <= threshold=4.902882814407349
node_78: feature_name=CD79B	CD79B.value <= threshold=4.87716269493103
node_79: feature_name=HLA-DQB1	HLA-DQB1.value <= threshold=3.6925491094589233
node_80: feature_name=HLA-DRB5	HLA-DRB5.value <= threshold=2.0926843881607056
node_81: feature_name=MCTP1	MCTP1.value <= threshold=4.010472774505615

node_82: feature_name=GNG11	GNG11.value <= threshold=3.627470850944519
node_83: feature_name=HSP90B1	HSP90B1.value <= threshold=2.0835131406784058
node_84: feature_name=ATF3	ATF3.value <= threshold=1.1142374873161316
node_85: feature_name=MT-ND6	MT-ND6.value <= threshold=2.7328014373779297
node_86: feature_name=COTL1	COTL1.value > threshold=-1.6524587869644165
node_90: feature_name=MT-CYB	MT-CYB.value <= threshold=1.099629819393158
node_91: feature_name=RGCC	RGCC.value <= threshold=1.6908138990402222
node_92: feature_name=ILK	ILK.value <= threshold=3.9497616291046143
node_93: feature_name=CTD-2035E11.3	CTD-2035E11.3.value <= threshold=2.4543306827545166
node_94: feature_name=S100A6	S100A6.value <= threshold=0.2867250442504883
node_95: feature_name=HLA-DRB5	HLA-DRB5.value > threshold=-0.9496813416481018
node_99: feature_name=MT-CO3	MT-CO3.value > threshold=-1.55654776096344
node_103: feature_name=ITGAX	ITGAX.value <= threshold=1.8544010519981384
node_104: feature_name=MEF2C	MEF2C.value <= threshold=4.22724461555481
node_105: feature_name=CXorf57	CXorf57.value <= threshold=5.114444732666016
node_106: feature_name=IRAK3	IRAK3.value > threshold=3.1017903089523315
node_140: feature_name=NR4A3	NR4A3.value > threshold=-0.418536439538002
Class: COVID-19 Critical	
Rules_887	passed counts:5
node_0: feature_name=LTB	LTB.value <= threshold=0.994031548500061
node_1: feature_name=MTRNR2L2	MTRNR2L2.value <= threshold=2.8784297704696655
node_2: feature_name=TCL1A	TCL1A.value <= threshold=4.798312664031982
node_3: feature_name=MT-CO1	MT-CO1.value <= threshold=0.08947191759943962
node_4: feature_name=NR4A1	NR4A1.value <= threshold=1.0725348591804504
node_5: feature_name=HLA-DPA1	HLA-DPA1.value <= threshold=1.6885469555854797
node_6: feature_name=HLA-DRB5	HLA-DRB5.value <= threshold=2.1886194944381714
node_7: feature_name=XIST	XIST.value <= threshold=-0.12328458577394485
node_8: feature_name=SYNE1	SYNE1.value <= threshold=-0.7835594415664673
node_9: feature_name=RGCC	RGCC.value <= threshold=-0.6801793873310089

Class: Healthy Control	
Rules_888	passed counts:4
node_0: feature_name=LTB	LTB.value > threshold=0.994031548500061
node_5264: feature_name=MT-CO1	MT-CO1.value > threshold=-0.4127991199493408
node_5638: feature_name=OAZ1	OAZ1.value > threshold=-0.2640867084264755
node_6112: feature_name=IFI44L	IFI44L.value <= threshold=2.5893263816833496
node_6113: feature_name=TCL1A	TCL1A.value > threshold=4.201832294464111
node_6991: feature_name=XAF1	XAF1.value <= threshold=3.4585667848587036
node_6992: feature_name=STAG3	STAG3.value <= threshold=7.686291694641113
node_6993: feature_name=RALGPS2	RALGPS2.value > threshold=-0.4090157449245453
node_6997: feature_name=MTRNR2L2	MTRNR2L2.value <= threshold=3.23723828792572
node_6998: feature_name=TP53INP1	TP53INP1.value <= threshold=6.239925146102905
node_6999: feature_name=MEF2C	MEF2C.value <= threshold=-0.44642284512519836
node_7000: feature_name=HLA-DPA1	HLA-DPA1.value <= threshold=1.8664597272872925
Class: Healthy Control	
Rules_889	passed counts:4
node_0: feature_name=LTB	LTB.value > threshold=0.994031548500061
node_5264: feature_name=MT-CO1	MT-CO1.value > threshold=-0.4127991199493408
node_5638: feature_name=OAZ1	OAZ1.value > threshold=-0.2640867084264755
node_6112: feature_name=IFI44L	IFI44L.value <= threshold=2.5893263816833496
node_6113: feature_name=TCL1A	TCL1A.value <= threshold=4.201832294464111
node_6114: feature_name=HLA-DQA2	HLA-DQA2.value > threshold=-0.2270243540406227
node_6542: feature_name=CD79B	CD79B.value > threshold=3.096232771873474
node_6872: feature_name=ICAM1	ICAM1.value <= threshold=-0.2232399433851242
node_6873: feature_name=STAG3	STAG3.value > threshold=-0.19312061369419098
node_6939: feature_name=HLA-DRB5	HLA-DRB5.value > threshold=2.209904909133911
node_6967: feature_name=TSPYL2	TSPYL2.value <= threshold=-0.6224541068077087
node_6968: feature_name=BANK1	BANK1.value <= threshold=2.4764840602874756

node_6969: feature_name=FCRL1	FCRL1.value <= threshold=5.541063070297241
Class: COVID-19 Critical	
Rules_890	passed counts:4
node_0: feature_name=LTB	LTB.value > threshold=0.994031548500061
node_5264: feature_name=MT-CO1	MT-CO1.value > threshold=-0.4127991199493408
node_5638: feature_name=OAZ1	OAZ1.value > threshold=-0.2640867084264755
node_6112: feature_name=IFI44L	IFI44L.value <= threshold=2.5893263816833496
node_6113: feature_name=TCL1A	TCL1A.value <= threshold=4.201832294464111
node_6114: feature_name=HLA-DQA2	HLA-DQA2.value > threshold=-0.2270243540406227
node_6542: feature_name=CD79B	CD79B.value > threshold=3.096232771873474
node_6872: feature_name=ICAM1	ICAM1.value <= threshold=-0.2232399433851242
node_6873: feature_name=STAG3	STAG3.value > threshold=-0.19312061369419098
node_6939: feature_name=HLA-DRB5	HLA-DRB5.value <= threshold=2.209904909133911
node_6940: feature_name=STAG3	STAG3.value <= threshold=7.8730878829956055
node_6941: feature_name=FAM129C	FAM129C.value > threshold=1.668714702129364
node_6953: feature_name=ENDOD1	ENDOD1.value > threshold=2.171262264251709
node_6959: feature_name=TTN	TTN.value <= threshold=-0.25319549441337585
Class: Healthy Control	
Rules_891	passed counts:4
node_0: feature_name=LTB	LTB.value > threshold=0.994031548500061
node_5264: feature_name=MT-CO1	MT-CO1.value > threshold=-0.4127991199493408
node_5638: feature_name=OAZ1	OAZ1.value > threshold=-0.2640867084264755
node_6112: feature_name=IFI44L	IFI44L.value <= threshold=2.5893263816833496
node_6113: feature_name=TCL1A	TCL1A.value <= threshold=4.201832294464111
node_6114: feature_name=HLA-DQA2	HLA-DQA2.value > threshold=-0.2270243540406227
node_6542: feature_name=CD79B	CD79B.value <= threshold=3.096232771873474
node_6543: feature_name=NR4A1	NR4A1.value > threshold=1.421015739440918
node_6811: feature_name=SLC7A5	SLC7A5.value > threshold=1.570515513420105

node_6867: feature_name=HSP90B1	HSP90B1.value <= threshold=-0.995681881904602
Class: Healthy Control	
Rules_892	passed counts:4
node_0: feature_name=LTB	LTB.value > threshold=0.994031548500061
node_5264: feature_name=MT-CO1	MT-CO1.value > threshold=-0.4127991199493408
node_5638: feature_name=OAZ1	OAZ1.value > threshold=-0.2640867084264755
node_6112: feature_name=IFI44L	IFI44L.value <= threshold=2.5893263816833496
node_6113: feature_name=TCL1A	TCL1A.value <= threshold=4.201832294464111
node_6114: feature_name=HLA-DQA2	HLA-DQA2.value > threshold=-0.2270243540406227
node_6542: feature_name=CD79B	CD79B.value <= threshold=3.096232771873474
node_6543: feature_name=NR4A1	NR4A1.value <= threshold=1.421015739440918
node_6544: feature_name=LTB	LTB.value > threshold=1.359755277633667
node_6720: feature_name=NFKBIZ	NFKBIZ.value <= threshold=0.2720414251089096
node_6721: feature_name=ATF5	ATF5.value > threshold=2.4672781229019165
node_6787: feature_name=SPI1	SPI1.value <= threshold=-0.4216492027044296
node_6788: feature_name=HLA-DQA1	HLA-DQA1.value <= threshold=2.4788447618484497
Class: Healthy Control	
Rules_893	passed counts:4
node_0: feature_name=LTB	LTB.value > threshold=0.994031548500061
node_5264: feature_name=MT-CO1	MT-CO1.value > threshold=-0.4127991199493408
node_5638: feature_name=OAZ1	OAZ1.value > threshold=-0.2640867084264755
node_6112: feature_name=IFI44L	IFI44L.value <= threshold=2.5893263816833496
node_6113: feature_name=TCL1A	TCL1A.value <= threshold=4.201832294464111
node_6114: feature_name=HLA-DQA2	HLA-DQA2.value > threshold=-0.2270243540406227
node_6542: feature_name=CD79B	CD79B.value <= threshold=3.096232771873474
node_6543: feature_name=NR4A1	NR4A1.value <= threshold=1.421015739440918
node_6544: feature_name=LTB	LTB.value > threshold=1.359755277633667
node_6720: feature_name=NFKBIZ	NFKBIZ.value <= threshold=0.2720414251089096

node_6721: feature_name=ATF5	ATF5.value <= threshold=2.4672781229019165
node_6722: feature_name=SNX9	SNX9.value <= threshold=2.0814030170440674
node_6723: feature_name=HLA-DQA2	HLA-DQA2.value <= threshold=4.360614776611328
node_6724: feature_name=MT-CO1	MT-CO1.value > threshold=-0.12188276648521423
node_6736: feature_name=MT1E	MT1E.value <= threshold=-0.1352209895849228
node_6737: feature_name=PELI1	PELI1.value <= threshold=3.1761642694473267
node_6738: feature_name=TRAF1	TRAF1.value > threshold=-0.2332744374871254
node_6764: feature_name=DUSP4	DUSP4.value > threshold=-0.2568044811487198
Class: COVID-19 Critical	
Rules_894	passed counts:4
node_0: feature_name=LTB	LTB.value > threshold=0.994031548500061
node_5264: feature_name=MT-CO1	MT-CO1.value > threshold=-0.4127991199493408
node_5638: feature_name=OAZ1	OAZ1.value > threshold=-0.2640867084264755
node_6112: feature_name=IFI44L	IFI44L.value <= threshold=2.5893263816833496
node_6113: feature_name=TCL1A	TCL1A.value <= threshold=4.201832294464111
node_6114: feature_name=HLA-DQA2	HLA-DQA2.value > threshold=-0.2270243540406227
node_6542: feature_name=CD79B	CD79B.value <= threshold=3.096232771873474
node_6543: feature_name=NR4A1	NR4A1.value <= threshold=1.421015739440918
node_6544: feature_name=LTB	LTB.value <= threshold=1.359755277633667
node_6545: feature_name=NFKBID	NFKBID.value > threshold=1.4294711351394653
node_6643: feature_name=FAM129C	FAM129C.value > threshold=4.799884080886841
node_6709: feature_name=MT-ND6	MT-ND6.value <= threshold=-0.9825485348701477
node_6710: feature_name=EGR1	EGR1.value > threshold=-0.32739605009555817
Class: COVID-19 Severe	
Rules_895	passed counts:4
node_0: feature_name=LTB	LTB.value > threshold=0.994031548500061
node_5264: feature_name=MT-CO1	MT-CO1.value > threshold=-0.4127991199493408
node_5638: feature_name=OAZ1	OAZ1.value > threshold=-0.2640867084264755

node_6112: feature_name=IFI44L	IFI44L.value <= threshold=2.5893263816833496
node_6113: feature_name=TCL1A	TCL1A.value <= threshold=4.201832294464111
node_6114: feature_name=HLA-DQA2	HLA-DQA2.value > threshold=-0.2270243540406227
node_6542: feature_name=CD79B	CD79B.value <= threshold=3.096232771873474
node_6543: feature_name=NR4A1	NR4A1.value <= threshold=1.421015739440918
node_6544: feature_name=LTB	LTB.value <= threshold=1.359755277633667
node_6545: feature_name=NFKBID	NFKBID.value > threshold=1.4294711351394653
node_6643: feature_name=FAM129C	FAM129C.value <= threshold=4.799884080886841
node_6644: feature_name=XIST	XIST.value <= threshold=0.9931697957217693
node_6645: feature_name=GPX1	GPX1.value > threshold=0.3628883808851242
node_6695: feature_name=CD79A	CD79A.value > threshold=2.4297949075698853
node_6699: feature_name=FCRL1	FCRL1.value <= threshold=-0.20751195400953293
node_6700: feature_name=DAPP1	DAPP1.value <= threshold=-0.2950359880924225
Class: COVID-19 Critical	
Rules_896	passed counts:4
node_0: feature_name=LTB	LTB.value > threshold=0.994031548500061
node_5264: feature_name=MT-CO1	MT-CO1.value > threshold=-0.4127991199493408
node_5638: feature_name=OAZ1	OAZ1.value > threshold=-0.2640867084264755
node_6112: feature_name=IFI44L	IFI44L.value <= threshold=2.5893263816833496
node_6113: feature_name=TCL1A	TCL1A.value <= threshold=4.201832294464111
node_6114: feature_name=HLA-DQA2	HLA-DQA2.value > threshold=-0.2270243540406227
node_6542: feature_name=CD79B	CD79B.value <= threshold=3.096232771873474
node_6543: feature_name=NR4A1	NR4A1.value <= threshold=1.421015739440918
node_6544: feature_name=LTB	LTB.value <= threshold=1.359755277633667
node_6545: feature_name=NFKBID	NFKBID.value <= threshold=1.4294711351394653
node_6546: feature_name=HLA-DRB1	HLA-DRB1.value > threshold=1.0398632287979126
node_6556: feature_name=CD79B	CD79B.value > threshold=-0.2919560521841049
node_6582: feature_name=PELI1	PELI1.value > threshold=-0.6156984269618988
node_6602: feature_name=MT-CO3	MT-CO3.value <= threshold=0.46141014993190765

node_6603: feature_name=NFKBIZ	NFKBIZ.value <= threshold=0.6470938920974731
node_6604: feature_name=HSP90B1	HSP90B1.value > threshold=0.4268011748790741
node_6620: feature_name=MT-CO3	MT-CO3.value > threshold=0.2716837227344513
node_6624: feature_name=CD79B	CD79B.value <= threshold=2.595557451248169
Class: Healthy Control	
Rules_897	passed counts:4
node_0: feature_name=LTB	LTB.value > threshold=0.994031548500061
node_5264: feature_name=MT-CO1	MT-CO1.value > threshold=-0.4127991199493408
node_5638: feature_name=OAZ1	OAZ1.value > threshold=-0.2640867084264755
node_6112: feature_name=IFI44L	IFI44L.value <= threshold=2.5893263816833496
node_6113: feature_name=TCL1A	TCL1A.value <= threshold=4.201832294464111
node_6114: feature_name=HLA-DQA2	HLA-DQA2.value > threshold=-0.2270243540406227
node_6542: feature_name=CD79B	CD79B.value <= threshold=3.096232771873474
node_6543: feature_name=NR4A1	NR4A1.value <= threshold=1.421015739440918
node_6544: feature_name=LTB	LTB.value <= threshold=1.359755277633667
node_6545: feature_name=NFKBID	NFKBID.value <= threshold=1.4294711351394653
node_6546: feature_name=HLA-DRB1	HLA-DRB1.value > threshold=1.0398632287979126
node_6556: feature_name=CD79B	CD79B.value > threshold=-0.2919560521841049
node_6582: feature_name=PELI1	PELI1.value <= threshold=-0.6156984269618988
node_6583: feature_name=LTB	LTB.value > threshold=1.0177513360977173
node_6585: feature_name=CD22	CD22.value <= threshold=-0.24851123988628387
node_6586: feature_name=OAZ1	OAZ1.value > threshold=0.9554091989994049
node_6590: feature_name=CSF3R	CSF3R.value <= threshold=0.9392853081226349
Class: Healthy Control	
Rules_898	passed counts:4
node_0: feature_name=LTB	LTB.value > threshold=0.994031548500061
node_5264: feature_name=MT-CO1	MT-CO1.value > threshold=-0.4127991199493408
node_5638: feature_name=OAZ1	OAZ1.value > threshold=-0.2640867084264755

node_6112: feature_name=IFI44L	IFI44L.value <= threshold=2.5893263816833496
node_6113: feature_name=TCL1A	TCL1A.value <= threshold=4.201832294464111
node_6114: feature_name=HLA-DQA2	HLA-DQA2.value > threshold=-0.2270243540406227
node_6542: feature_name=CD79B	CD79B.value <= threshold=3.096232771873474
node_6543: feature_name=NR4A1	NR4A1.value <= threshold=1.421015739440918
node_6544: feature_name=LTB	LTB.value <= threshold=1.359755277633667
node_6545: feature_name=NFKBID	NFKBID.value <= threshold=1.4294711351394653
node_6546: feature_name=HLA-DRB1	HLA-DRB1.value > threshold=1.0398632287979126
node_6556: feature_name=CD79B	CD79B.value <= threshold=-0.2919560521841049
node_6557: feature_name=MTRNR2L2	MTRNR2L2.value > threshold=2.7710195183753967
Class: COVID-19 Severe	
Rules_899	passed counts:4
node_0: feature_name=LTB	LTB.value > threshold=0.994031548500061
node_5264: feature_name=MT-CO1	MT-CO1.value > threshold=-0.4127991199493408
node_5638: feature_name=OAZ1	OAZ1.value > threshold=-0.2640867084264755
node_6112: feature_name=IFI44L	IFI44L.value <= threshold=2.5893263816833496
node_6113: feature_name=TCL1A	TCL1A.value <= threshold=4.201832294464111
node_6114: feature_name=HLA-DQA2	HLA-DQA2.value > threshold=-0.2270243540406227
node_6542: feature_name=CD79B	CD79B.value <= threshold=3.096232771873474
node_6543: feature_name=NR4A1	NR4A1.value <= threshold=1.421015739440918
node_6544: feature_name=LTB	LTB.value <= threshold=1.359755277633667
node_6545: feature_name=NFKBID	NFKBID.value <= threshold=1.4294711351394653
node_6546: feature_name=HLA-DRB1	HLA-DRB1.value > threshold=1.0398632287979126
node_6556: feature_name=CD79B	CD79B.value <= threshold=-0.2919560521841049
node_6557: feature_name=MTRNR2L2	MTRNR2L2.value <= threshold=2.7710195183753967
node_6558: feature_name=JUN	JUN.value <= threshold=0.5767147243022919
node_6559: feature_name=TLN1	TLN1.value > threshold=-0.08378086984157562
node_6569: feature_name=RALGPS2	RALGPS2.value > threshold=2.019797205924988
Class: Healthy Control	

Rules_900	passed counts:4
node_0: feature_name=LTB	LTB.value > threshold=0.994031548500061
node_5264: feature_name=MT-CO1	MT-CO1.value > threshold=-0.4127991199493408
node_5638: feature_name=OAZ1	OAZ1.value > threshold=-0.2640867084264755
node_6112: feature_name=IFI44L	IFI44L.value <= threshold=2.5893263816833496
node_6113: feature_name=TCL1A	TCL1A.value <= threshold=4.201832294464111
node_6114: feature_name=HLA-DQA2	HLA-DQA2.value <= threshold=-0.2270243540406227
node_6115: feature_name=MTRNR2L2	MTRNR2L2.value <= threshold=2.548917293548584
node_6116: feature_name=ATF5	ATF5.value > threshold=2.041263461112976
node_6490: feature_name=HLA-DPB1	HLA-DPB1.value > threshold=1.5343759655952454
node_6508: feature_name=OAZ1	OAZ1.value <= threshold=0.21525950729846954
node_6509: feature_name=RHOB	RHOB.value > threshold=0.046925410628318787
node_6511: feature_name=RBKS	RBKS.value <= threshold=-0.29246409237384796
Class: Healthy Control	
Rules_901	passed counts:4
node_0: feature_name=LTB	LTB.value > threshold=0.994031548500061
node_5264: feature_name=MT-CO1	MT-CO1.value > threshold=-0.4127991199493408
node_5638: feature_name=OAZ1	OAZ1.value > threshold=-0.2640867084264755
node_6112: feature_name=IFI44L	IFI44L.value <= threshold=2.5893263816833496
node_6113: feature_name=TCL1A	TCL1A.value <= threshold=4.201832294464111
node_6114: feature_name=HLA-DQA2	HLA-DQA2.value <= threshold=-0.2270243540406227
node_6115: feature_name=MTRNR2L2	MTRNR2L2.value <= threshold=2.548917293548584
node_6116: feature_name=ATF5	ATF5.value <= threshold=2.041263461112976
node_6117: feature_name=JUN	JUN.value > threshold=-0.616259902715683
node_6189: feature_name=MT-CO3	MT-CO3.value > threshold=0.13483521342277527
node_6351: feature_name=XAF1	XAF1.value <= threshold=3.061717987060547
node_6352: feature_name=MT-CO2	MT-CO2.value <= threshold=1.2605181336402893
node_6353: feature_name=ICAM1	ICAM1.value > threshold=-0.3340446650981903

node_6439: feature_name=FAM129C	FAM129C.value > threshold=-0.24657993018627167
node_6455: feature_name=HSP90B1	HSP90B1.value <= threshold=1.5712243914604187
node_6456: feature_name=NR4A1	NR4A1.value <= threshold=-0.6648433208465576
node_6457: feature_name=DAPP1	DAPP1.value > threshold=0.7670192569494247
node_6459: feature_name=HLA-DRB1	HLA-DRB1.value <= threshold=1.7536143064498901
Class: COVID-19 Critical	
Rules_902	passed counts:4
node_0: feature_name=LTB	LTB.value > threshold=0.994031548500061
node_5264: feature_name=MT-CO1	MT-CO1.value > threshold=-0.4127991199493408
node_5638: feature_name=OAZ1	OAZ1.value > threshold=-0.2640867084264755
node_6112: feature_name=IFI44L	IFI44L.value <= threshold=2.5893263816833496
node_6113: feature_name=TCL1A	TCL1A.value <= threshold=4.201832294464111
node_6114: feature_name=HLA-DQA2	HLA-DQA2.value <= threshold=-0.2270243540406227
node_6115: feature_name=MTRNR2L2	MTRNR2L2.value <= threshold=2.548917293548584
node_6116: feature_name=ATF5	ATF5.value <= threshold=2.041263461112976
node_6117: feature_name=JUN	JUN.value > threshold=-0.616259902715683
node_6189: feature_name=MT-CO3	MT-CO3.value > threshold=0.13483521342277527
node_6351: feature_name=XAF1	XAF1.value <= threshold=3.061717987060547
node_6352: feature_name=MT-CO2	MT-CO2.value <= threshold=1.2605181336402893
node_6353: feature_name=ICAM1	ICAM1.value > threshold=-0.3340446650981903
node_6439: feature_name=FAM129C	FAM129C.value <= threshold=-0.24657993018627167
node_6440: feature_name=ITM2C	ITM2C.value > threshold=-0.2592049688100815
node_6446: feature_name=S100A6	S100A6.value <= threshold=-0.7434525489807129
node_6447: feature_name=HLA-DPA1	HLA-DPA1.value <= threshold=1.7991477251052856
Class: COVID-19 Critical	
Rules_903	passed counts:4
node_0: feature_name=LTB	LTB.value > threshold=0.994031548500061
node_5264: feature_name=MT-CO1	MT-CO1.value > threshold=-0.4127991199493408

node_5638: feature_name=OAZ1	OAZ1.value > threshold=-0.2640867084264755
node_6112: feature_name=IFI44L	IFI44L.value <= threshold=2.5893263816833496
node_6113: feature_name=TCL1A	TCL1A.value <= threshold=4.201832294464111
node_6114: feature_name=HLA-DQA2	HLA-DQA2.value <= threshold=-0.2270243540406227
node_6115: feature_name=MTRNR2L2	MTRNR2L2.value <= threshold=2.548917293548584
node_6116: feature_name=ATF5	ATF5.value <= threshold=2.041263461112976
node_6117: feature_name=JUN	JUN.value > threshold=-0.616259902715683
node_6189: feature_name=MT-CO3	MT-CO3.value > threshold=0.13483521342277527
node_6351: feature_name=XAF1	XAF1.value <= threshold=3.061717987060547
node_6352: feature_name=MT-CO2	MT-CO2.value <= threshold=1.2605181336402893
node_6353: feature_name=ICAM1	ICAM1.value > threshold=-0.3340446650981903
node_6439: feature_name=FAM129C	FAM129C.value <= threshold=-0.24657993018627167
node_6440: feature_name=ITM2C	ITM2C.value <= threshold=-0.2592049688100815
node_6441: feature_name=TCF4	TCF4.value > threshold=2.291034698486328
node_6443: feature_name=MT-CYB	MT-CYB.value <= threshold=0.6701975166797638
Class: Healthy Control	
Rules_904	passed counts:4
node_0: feature_name=LTB	LTB.value > threshold=0.994031548500061
node_5264: feature_name=MT-CO1	MT-CO1.value > threshold=-0.4127991199493408
node_5638: feature_name=OAZ1	OAZ1.value > threshold=-0.2640867084264755
node_6112: feature_name=IFI44L	IFI44L.value <= threshold=2.5893263816833496
node_6113: feature_name=TCL1A	TCL1A.value <= threshold=4.201832294464111
node_6114: feature_name=HLA-DQA2	HLA-DQA2.value <= threshold=-0.2270243540406227
node_6115: feature_name=MTRNR2L2	MTRNR2L2.value <= threshold=2.548917293548584
node_6116: feature_name=ATF5	ATF5.value <= threshold=2.041263461112976
node_6117: feature_name=JUN	JUN.value > threshold=-0.616259902715683
node_6189: feature_name=MT-CO3	MT-CO3.value > threshold=0.13483521342277527
node_6351: feature_name=XAF1	XAF1.value <= threshold=3.061717987060547
node_6352: feature_name=MT-CO2	MT-CO2.value <= threshold=1.2605181336402893

node_6353: feature_name=ICAM1	ICAM1.value <= threshold=-0.3340446650981903
node_6354: feature_name=OAZ1	OAZ1.value > threshold=-0.24055946618318558
node_6360: feature_name=PPIF	PPIF.value <= threshold=2.070030093193054
node_6361: feature_name=RALGPS2	RALGPS2.value > threshold=-0.3684315085411072
node_6369: feature_name=ANO6	ANO6.value <= threshold=3.504153251647949
node_6370: feature_name=YWHAH	YWHAH.value <= threshold=4.3182291984558105
node_6371: feature_name=LDLR	LDLR.value <= threshold=2.494291305541992
node_6372: feature_name=HLA-DQA1	HLA-DQA1.value > threshold=-0.5716990232467651
node_6374: feature_name=ABCA1	ABCA1.value <= threshold=3.7662240266799927
node_6375: feature_name=MS4A1	MS4A1.value > threshold=1.4995208978652954
node_6387: feature_name=SNORD3B-2	SNORD3B-2.value <= threshold=4.154061555862427
node_6388: feature_name=LTB	LTB.value <= threshold=2.1739100217819214
node_6389: feature_name=BACH1	BACH1.value <= threshold=2.0371285676956177
node_6390: feature_name=HLA-DQB1	HLA-DQB1.value <= threshold=3.0258322954177856
node_6391: feature_name=HLA-DPB1	HLA-DPB1.value > threshold=0.2591289207339287
node_6395: feature_name=CXorf57	CXorf57.value <= threshold=3.3742183446884155
node_6396: feature_name=MMD	MMD.value <= threshold=3.8883349895477295
node_6397: feature_name=IRF8	IRF8.value <= threshold=3.939465045928955
node_6398: feature_name=PPIF	PPIF.value > threshold=1.1284508109092712
node_6408: feature_name=JUN	JUN.value <= threshold=-0.1225685365498066
node_6409: feature_name=FTH1	FTH1.value > threshold=-0.6591627895832062
Class: Healthy Control	
Rules_905	passed counts:4
node_0: feature_name=LTB	LTB.value > threshold=0.994031548500061
node_5264: feature_name=MT-CO1	MT-CO1.value > threshold=-0.4127991199493408
node_5638: feature_name=OAZ1	OAZ1.value > threshold=-0.26408670842647555
node_6112: feature_name=IFI44L	IFI44L.value <= threshold=2.5893263816833496
node_6113: feature_name=TCL1A	TCL1A.value <= threshold=4.201832294464111
node_6114: feature_name=HLA-DQA2	HLA-DQA2.value <= threshold=-0.2270243540406227

node_6115: feature_name=MTRNR2L2	MTRNR2L2.value <= threshold=2.548917293548584
node_6116: feature_name=ATF5	ATF5.value <= threshold=2.041263461112976
node_6117: feature_name=JUN	JUN.value > threshold=-0.616259902715683
node_6189: feature_name=MT-CO3	MT-CO3.value <= threshold=0.13483521342277527
node_6190: feature_name=XAF1	XAF1.value <= threshold=2.584952235221863
node_6191: feature_name=HLA-DRB5	HLA-DRB5.value > threshold=0.6955901980400085
node_6239: feature_name=TNFAIP3	TNFAIP3.value <= threshold=0.11930759996175766
node_6240: feature_name=TP53INP1	TP53INP1.value <= threshold=2.9654784202575684
node_6241: feature_name=HLA-DRB5	HLA-DRB5.value > threshold=2.5141202211380005
node_6325: feature_name=VCL	VCL.value <= threshold=-0.33651162683963776
Class: COVID-19 Severe	
Rules_906	passed counts:4
node_0: feature_name=LTB	LTB.value > threshold=0.994031548500061
node_5264: feature_name=MT-CO1	MT-CO1.value > threshold=-0.4127991199493408
node_5638: feature_name=OAZ1	OAZ1.value > threshold=-0.2640867084264755
node_6112: feature_name=IFI44L	IFI44L.value <= threshold=2.5893263816833496
node_6113: feature_name=TCL1A	TCL1A.value <= threshold=4.201832294464111
node_6114: feature_name=HLA-DQA2	HLA-DQA2.value <= threshold=-0.2270243540406227
node_6115: feature_name=MTRNR2L2	MTRNR2L2.value <= threshold=2.548917293548584
node_6116: feature_name=ATF5	ATF5.value <= threshold=2.041263461112976
node_6117: feature_name=JUN	JUN.value > threshold=-0.616259902715683
node_6189: feature_name=MT-CO3	MT-CO3.value <= threshold=0.13483521342277527
node_6190: feature_name=XAF1	XAF1.value <= threshold=2.584952235221863
node_6191: feature_name=HLA-DRB5	HLA-DRB5.value > threshold=0.6955901980400085
node_6239: feature_name=TNFAIP3	TNFAIP3.value <= threshold=0.11930759996175766
node_6240: feature_name=TP53INP1	TP53INP1.value <= threshold=2.9654784202575684
node_6241: feature_name=HLA-DRB5	HLA-DRB5.value <= threshold=2.5141202211380005
node_6242: feature_name=NFKBID	NFKBID.value <= threshold=3.358878970146179
node_6243: feature_name=JARID2	JARID2.value <= threshold=1.7160060405731201

node_6244: feature_name=HLA-DPA1	HLA-DPA1.value > threshold=0.49491266906261444
node_6246: feature_name=MIR24-2	MIR24-2.value > threshold=1.8348549008369446
node_6312: feature_name=RP11-685N3.1	RP11-685N3.1.value <= threshold=-0.17039766907691956
Class: COVID-19 Critical	
Rules_907	passed counts:4
node_0: feature_name=LTB	LTB.value > threshold=0.994031548500061
node_5264: feature_name=MT-CO1	MT-CO1.value > threshold=-0.4127991199493408
node_5638: feature_name=OAZ1	OAZ1.value > threshold=-0.2640867084264755
node_6112: feature_name=IFI44L	IFI44L.value <= threshold=2.5893263816833496
node_6113: feature_name=TCL1A	TCL1A.value <= threshold=4.201832294464111
node_6114: feature_name=HLA-DQA2	HLA-DQA2.value <= threshold=-0.2270243540406227
node_6115: feature_name=MTRNR2L2	MTRNR2L2.value <= threshold=2.548917293548584
node_6116: feature_name=ATF5	ATF5.value <= threshold=2.041263461112976
node_6117: feature_name=JUN	JUN.value > threshold=-0.616259902715683
node_6189: feature_name=MT-CO3	MT-CO3.value <= threshold=0.13483521342277527
node_6190: feature_name=XAF1	XAF1.value <= threshold=2.584952235221863
node_6191: feature_name=HLA-DRB5	HLA-DRB5.value <= threshold=0.6955901980400085
node_6192: feature_name=NR4A1	NR4A1.value <= threshold=-0.4496448189020157
node_6193: feature_name=HLA-DPB1	HLA-DPB1.value > threshold=1.7609722018241882
node_6213: feature_name=SNX9	SNX9.value <= threshold=1.1182581186294556
node_6214: feature_name=MX1	MX1.value > threshold=1.2671910524368286
Class: COVID-19 Critical	
Rules_908	passed counts:4
node_0: feature_name=LTB	LTB.value > threshold=0.994031548500061
node_5264: feature_name=MT-CO1	MT-CO1.value > threshold=-0.4127991199493408
node_5638: feature_name=OAZ1	OAZ1.value <= threshold=-0.2640867084264755
node_5639: feature_name=CD79B	CD79B.value > threshold=2.2998517751693726
node_5925: feature_name=SNX9	SNX9.value <= threshold=2.4445992708206177

node_5926: feature_name=TCL1A	TCL1A.value <= threshold=2.6938670873641968
node_5927: feature_name=PLD4	PLD4.value > threshold=-0.06434785947203636
node_6009: feature_name=MX1	MX1.value <= threshold=3.600893020629883
node_6010: feature_name=PRDM1	PRDM1.value > threshold=-0.407244011759758
node_6046: feature_name=LTB	LTB.value <= threshold=1.087718427181244
Class: Healthy Control	
Rules_909	passed counts:4
node_0: feature_name=LTB	LTB.value > threshold=0.994031548500061
node_5264: feature_name=MT-CO1	MT-CO1.value > threshold=-0.4127991199493408
node_5638: feature_name=OAZ1	OAZ1.value <= threshold=-0.2640867084264755
node_5639: feature_name=CD79B	CD79B.value > threshold=2.2998517751693726
node_5925: feature_name=SNX9	SNX9.value <= threshold=2.4445992708206177
node_5926: feature_name=TCL1A	TCL1A.value <= threshold=2.6938670873641968
node_5927: feature_name=PLD4	PLD4.value <= threshold=-0.06434785947203636
node_5928: feature_name=ICAM1	ICAM1.value <= threshold=-0.2820792943239212
node_5929: feature_name=MS4A1	MS4A1.value > threshold=2.747320055961609
node_5963: feature_name=RNF144B	RNF144B.value <= threshold=-0.2324080914258957
node_5964: feature_name=HLA-DRB5	HLA-DRB5.value > threshold=2.0430362224578857
node_5982: feature_name=HSP90B1	HSP90B1.value > threshold=0.5199131667613983
node_5984: feature_name=MARCKS	MARCKS.value <= threshold=1.4439378380775452
Class: Healthy Control	
Rules_910	passed counts:4
node_0: feature_name=LTB	LTB.value > threshold=0.994031548500061
node_5264: feature_name=MT-CO1	MT-CO1.value > threshold=-0.4127991199493408
node_5638: feature_name=OAZ1	OAZ1.value <= threshold=-0.2640867084264755
node_5639: feature_name=CD79B	CD79B.value > threshold=2.2998517751693726
node_5925: feature_name=SNX9	SNX9.value <= threshold=2.4445992708206177
node_5926: feature_name=TCL1A	TCL1A.value <= threshold=2.6938670873641968

node_5927: feature_name=PLD4	PLD4.value <= threshold=-0.06434785947203636
node_5928: feature_name=ICAM1	ICAM1.value <= threshold=-0.2820792943239212
node_5929: feature_name=MS4A1	MS4A1.value <= threshold=2.747320055961609
node_5930: feature_name=LTB	LTB.value <= threshold=1.3533939123153687
node_5931: feature_name=CD83	CD83.value <= threshold=3.0086376667022705
node_5932: feature_name=HLA-DQB1	HLA-DQB1.value > threshold=2.98651123046875
Class: COVID-19 Severe	
Rules_911	passed counts:4
node_0: feature_name=LTB	LTB.value > threshold=0.994031548500061
node_5264: feature_name=MT-CO1	MT-CO1.value > threshold=-0.4127991199493408
node_5638: feature_name=OAZ1	OAZ1.value <= threshold=-0.2640867084264755
node_5639: feature_name=CD79B	CD79B.value <= threshold=2.2998517751693726
node_5640: feature_name=MTRNR2L2	MTRNR2L2.value <= threshold=3.5499863624572754
node_5641: feature_name=MT-CO1	MT-CO1.value > threshold=0.22567133605480194
node_5729: feature_name=MARCH1	MARCH1.value <= threshold=1.5154643654823303
node_5730: feature_name=MT-CO1	MT-CO1.value <= threshold=1.7823500633239746
node_5731: feature_name=HLA-DPB1	HLA-DPB1.value > threshold=2.0096163749694824
node_5829: feature_name=LTB	LTB.value > threshold=1.2124510407447815
node_5843: feature_name=TTN	TTN.value > threshold=-0.3821280151605606
node_5851: feature_name=SNX9	SNX9.value > threshold=1.9138266444206238
node_5853: feature_name=DENND3	DENND3.value <= threshold=-0.3534839004278183
Class: COVID-19 Severe	
Rules_912	passed counts:4
node_0: feature_name=LTB	LTB.value > threshold=0.994031548500061
node_5264: feature_name=MT-CO1	MT-CO1.value > threshold=-0.4127991199493408
node_5638: feature_name=OAZ1	OAZ1.value <= threshold=-0.2640867084264755
node_5639: feature_name=CD79B	CD79B.value <= threshold=2.2998517751693726
node_5640: feature_name=MTRNR2L2	MTRNR2L2.value <= threshold=3.5499863624572754

node_5641: feature_name=MT-CO1	MT-CO1.value > threshold=0.22567133605480194
node_5729: feature_name=MARCH1	MARCH1.value <= threshold=1.5154643654823303
node_5730: feature_name=MT-CO1	MT-CO1.value <= threshold=1.7823500633239746
node_5731: feature_name=HLA-DPB1	HLA-DPB1.value > threshold=2.0096163749694824
node_5829: feature_name=LTB	LTB.value > threshold=1.2124510407447815
node_5843: feature_name=TTN	TTN.value <= threshold=-0.3821280151605606
node_5844: feature_name=FAM129C	FAM129C.value > threshold=6.290640115737915
Class: Healthy Control	
Rules_913	passed counts:4
node_0: feature_name=LTB	LTB.value > threshold=0.994031548500061
node_5264: feature_name=MT-CO1	MT-CO1.value > threshold=-0.4127991199493408
node_5638: feature_name=OAZ1	OAZ1.value <= threshold=-0.2640867084264755
node_5639: feature_name=CD79B	CD79B.value <= threshold=2.2998517751693726
node_5640: feature_name=MTRNR2L2	MTRNR2L2.value <= threshold=3.5499863624572754
node_5641: feature_name=MT-CO1	MT-CO1.value > threshold=0.22567133605480194
node_5729: feature_name=MARCH1	MARCH1.value <= threshold=1.5154643654823303
node_5730: feature_name=MT-CO1	MT-CO1.value <= threshold=1.7823500633239746
node_5731: feature_name=HLA-DPB1	HLA-DPB1.value <= threshold=2.0096163749694824
node_5732: feature_name=NR4A3	NR4A3.value <= threshold=3.509189486503601
node_5733: feature_name=OAZ1	OAZ1.value <= threshold=-0.4392261207103729
node_5734: feature_name=SESN3	SESN3.value <= threshold=-0.45661941170692444
node_5735: feature_name=WDR74	WDR74.value > threshold=-0.4893670231103897
node_5761: feature_name=FCER2	FCER2.value <= threshold=6.041448354721069
node_5762: feature_name=TCL1A	TCL1A.value > threshold=-0.1774759516119957
node_5782: feature_name=HLA-DQB1	HLA-DQB1.value > threshold=2.203125238418579
Class: COVID-19 Critical	
Rules_914	passed counts:4
node_0: feature_name=LTB	LTB.value > threshold=0.994031548500061

node_5264: feature_name=MT-CO1	MT-CO1.value > threshold=-0.4127991199493408
node_5638: feature_name=OAZ1	OAZ1.value <= threshold=-0.2640867084264755
node_5639: feature_name=CD79B	CD79B.value <= threshold=2.2998517751693726
node_5640: feature_name=MTRNR2L2	MTRNR2L2.value <= threshold=3.5499863624572754
node_5641: feature_name=MT-CO1	MT-CO1.value > threshold=0.22567133605480194
node_5729: feature_name=MARCH1	MARCH1.value <= threshold=1.5154643654823303
node_5730: feature_name=MT-CO1	MT-CO1.value <= threshold=1.7823500633239746
node_5731: feature_name=HLA-DPB1	HLA-DPB1.value <= threshold=2.0096163749694824
node_5732: feature_name=NR4A3	NR4A3.value <= threshold=3.509189486503601
node_5733: feature_name=OAZ1	OAZ1.value <= threshold=-0.4392261207103729
node_5734: feature_name=SESN3	SESN3.value <= threshold=-0.45661941170692444
node_5735: feature_name=WDR74	WDR74.value <= threshold=-0.4893670231103897
node_5736: feature_name=NR4A1	NR4A1.value <= threshold=-0.2724379748106003
node_5737: feature_name=HLA-DPA1	HLA-DPA1.value > threshold=1.6106070280075073
node_5745: feature_name=MEF2C	MEF2C.value > threshold=2.7098671197891235
Class: Healthy Control	
Rules_915	passed counts:4
node_0: feature_name=LTB	LTB.value > threshold=0.994031548500061
node_5264: feature_name=MT-CO1	MT-CO1.value > threshold=-0.4127991199493408
node_5638: feature_name=OAZ1	OAZ1.value <= threshold=-0.2640867084264755
node_5639: feature_name=CD79B	CD79B.value <= threshold=2.2998517751693726
node_5640: feature_name=MTRNR2L2	MTRNR2L2.value <= threshold=3.5499863624572754
node_5641: feature_name=MT-CO1	MT-CO1.value <= threshold=0.22567133605480194
node_5642: feature_name=RGS2	RGS2.value > threshold=1.2649686336517334
node_5710: feature_name=HLA-DPB1	HLA-DPB1.value > threshold=1.09694242477417
node_5712: feature_name=PPIF	PPIF.value > threshold=-0.4469884932041168
node_5720: feature_name=CD83	CD83.value > threshold=1.302907645702362
node_5722: feature_name=COTL1	COTL1.value <= threshold=0.1188640259206295
node_5723: feature_name=ICAM1	ICAM1.value > threshold=-0.24770931899547577

node_5725: feature_name=PDLIM1	PDLIM1.value <= threshold=2.848420023918152
Class: COVID-19 Severe	
Rules_916	passed counts:4
node_0: feature_name=LTB	LTB.value > threshold=0.994031548500061
node_5264: feature_name=MT-CO1	MT-CO1.value > threshold=-0.4127991199493408
node_5638: feature_name=OAZ1	OAZ1.value <= threshold=-0.2640867084264755
node_5639: feature_name=CD79B	CD79B.value <= threshold=2.2998517751693726
node_5640: feature_name=MTRNR2L2	MTRNR2L2.value <= threshold=3.5499863624572754
node_5641: feature_name=MT-CO1	MT-CO1.value <= threshold=0.22567133605480194
node_5642: feature_name=RGS2	RGS2.value > threshold=1.2649686336517334
node_5710: feature_name=HLA-DPB1	HLA-DPB1.value > threshold=1.09694242477417
node_5712: feature_name=PPIF	PPIF.value > threshold=-0.4469884932041168
node_5720: feature_name=CD83	CD83.value <= threshold=1.302907645702362
Class: Healthy Control	
Rules_917	passed counts:4
node_0: feature_name=LTB	LTB.value > threshold=0.994031548500061
node_5264: feature_name=MT-CO1	MT-CO1.value > threshold=-0.4127991199493408
node_5638: feature_name=OAZ1	OAZ1.value <= threshold=-0.2640867084264755
node_5639: feature_name=CD79B	CD79B.value <= threshold=2.2998517751693726
node_5640: feature_name=MTRNR2L2	MTRNR2L2.value <= threshold=3.5499863624572754
node_5641: feature_name=MT-CO1	MT-CO1.value <= threshold=0.22567133605480194
node_5642: feature_name=RGS2	RGS2.value <= threshold=1.2649686336517334
node_5643: feature_name=TCL1A	TCL1A.value <= threshold=4.771023273468018
node_5644: feature_name=IGJ	IGJ.value <= threshold=3.91206431388855
node_5645: feature_name=AREGB	AREGB.value <= threshold=1.5142876282334328
node_5646: feature_name=HLA-DRB1	HLA-DRB1.value > threshold=-0.6253005266189575
node_5648: feature_name=ITM2C	ITM2C.value > threshold=3.2904402017593384
node_5690: feature_name=MT-CYB	MT-CYB.value > threshold=0.3988293260335922

Class: COVID-19 Severe	
Rules_918	passed counts:4
node_0: feature_name=LTB	LTB.value > threshold=0.994031548500061
node_5264: feature_name=MT-CO1	MT-CO1.value > threshold=-0.4127991199493408
node_5638: feature_name=OAZ1	OAZ1.value <= threshold=-0.2640867084264755
node_5639: feature_name=CD79B	CD79B.value <= threshold=2.2998517751693726
node_5640: feature_name=MTRNR2L2	MTRNR2L2.value <= threshold=3.5499863624572754
node_5641: feature_name=MT-CO1	MT-CO1.value <= threshold=0.22567133605480194
node_5642: feature_name=RGS2	RGS2.value <= threshold=1.2649686336517334
node_5643: feature_name=TCL1A	TCL1A.value <= threshold=4.771023273468018
node_5644: feature_name=IGJ	IGJ.value <= threshold=3.91206431388855
node_5645: feature_name=AREGB	AREGB.value <= threshold=1.5142876282334328
node_5646: feature_name=HLA-DRB1	HLA-DRB1.value > threshold=-0.6253005266189575
node_5648: feature_name=ITM2C	ITM2C.value <= threshold=3.2904402017593384
node_5649: feature_name=PLA2G12A	PLA2G12A.value <= threshold=3.833786725997925
node_5650: feature_name=RGCC	RGCC.value > threshold=-0.10016544535756111
node_5680: feature_name=DENND3	DENND3.value > threshold=1.5598239302635193
node_5684: feature_name=BANK1	BANK1.value > threshold=1.7741180062294006
Class: COVID-19 Severe	
Rules_919	passed counts:4
node_0: feature_name=LTB	LTB.value > threshold=0.994031548500061
node_5264: feature_name=MT-CO1	MT-CO1.value <= threshold=-0.4127991199493408
node_5265: feature_name=MTRNR2L2	MTRNR2L2.value <= threshold=2.4473838806152344
node_5266: feature_name=TCL1A	TCL1A.value > threshold=5.787256956100464
node_5616: feature_name=XAF1	XAF1.value <= threshold=3.0923240184783936
node_5617: feature_name=CD79A	CD79A.value > threshold=2.297448992729187
node_5623: feature_name=MT-CO1	MT-CO1.value <= threshold=-1.1357948780059814
node_5624: feature_name=TTN	TTN.value > threshold=-0.33193837106227875

Class: Healthy Control	
Rules_920	passed counts:4
node_0: feature_name=LTB	LTB.value > threshold=0.994031548500061
node_5264: feature_name=MT-CO1	MT-CO1.value <= threshold=-0.4127991199493408
node_5265: feature_name=MTRNR2L2	MTRNR2L2.value <= threshold=2.4473838806152344
node_5266: feature_name=TCL1A	TCL1A.value <= threshold=5.787256956100464
node_5267: feature_name=PLD4	PLD4.value <= threshold=3.544657826423645
node_5268: feature_name=NR4A1	NR4A1.value > threshold=0.8490704298019409
node_5498: feature_name=HLA-DPA1	HLA-DPA1.value > threshold=1.9079211950302124
node_5556: feature_name=CD83	CD83.value > threshold=1.6135562062263489
node_5560: feature_name=OAZ1	OAZ1.value <= threshold=0.11598343029618263
node_5561: feature_name=STMN1	STMN1.value <= threshold=-0.2897801846265793
Class: COVID-19 Critical	
Rules_921	passed counts:4
node_0: feature_name=LTB	LTB.value > threshold=0.994031548500061
node_5264: feature_name=MT-CO1	MT-CO1.value <= threshold=-0.4127991199493408
node_5265: feature_name=MTRNR2L2	MTRNR2L2.value <= threshold=2.4473838806152344
node_5266: feature_name=TCL1A	TCL1A.value <= threshold=5.787256956100464
node_5267: feature_name=PLD4	PLD4.value <= threshold=3.544657826423645
node_5268: feature_name=NR4A1	NR4A1.value <= threshold=0.8490704298019409
node_5269: feature_name=LTB	LTB.value > threshold=1.5252206921577454
node_5471: feature_name=CD79A	CD79A.value > threshold=2.510846734046936
node_5483: feature_name=XAF1	XAF1.value <= threshold=1.7000007629394531
node_5484: feature_name=RALGPS2	RALGPS2.value <= threshold=2.08430278301239
node_5485: feature_name=HLA-DPB1	HLA-DPB1.value > threshold=2.0922847390174866
Class: Healthy Control	
Rules_922	passed counts:4

node_0: feature_name=LTB	LTB.value > threshold=0.994031548500061
node_5264: feature_name=MT-CO1	MT-CO1.value <= threshold=-0.4127991199493408
node_5265: feature_name=MTRNR2L2	MTRNR2L2.value <= threshold=2.4473838806152344
node_5266: feature_name=TCL1A	TCL1A.value <= threshold=5.787256956100464
node_5267: feature_name=PLD4	PLD4.value <= threshold=3.544657826423645
node_5268: feature_name=NR4A1	NR4A1.value <= threshold=0.8490704298019409
node_5269: feature_name=LTB	LTB.value <= threshold=1.5252206921577454
node_5270: feature_name=HLA-DRB5	HLA-DRB5.value <= threshold=2.5117164850234985
node_5271: feature_name=CD79A	CD79A.value > threshold=3.685201406478882
Class: Healthy Control	
Rules_923	passed counts:4
node_0: feature_name=LTB	LTB.value > threshold=0.994031548500061
node_5264: feature_name=MT-CO1	MT-CO1.value <= threshold=-0.4127991199493408
node_5265: feature_name=MTRNR2L2	MTRNR2L2.value <= threshold=2.4473838806152344
node_5266: feature_name=TCL1A	TCL1A.value <= threshold=5.787256956100464
node_5267: feature_name=PLD4	PLD4.value <= threshold=3.544657826423645
node_5268: feature_name=NR4A1	NR4A1.value <= threshold=0.8490704298019409
node_5269: feature_name=LTB	LTB.value <= threshold=1.5252206921577454
node_5270: feature_name=HLA-DRB5	HLA-DRB5.value <= threshold=2.5117164850234985
node_5271: feature_name=CD79A	CD79A.value <= threshold=3.685201406478882
node_5272: feature_name=BCL2A1	BCL2A1.value <= threshold=2.081526756286621
node_5273: feature_name=HLA-DPA1	HLA-DPA1.value > threshold=1.8042166829109192
node_5363: feature_name=MS4A1	MS4A1.value <= threshold=3.5283708572387695
node_5364: feature_name=MT-CO3	MT-CO3.value <= threshold=0.3994715213775635
node_5365: feature_name=TNFAIP3	TNFAIP3.value <= threshold=0.2631131559610367
node_5366: feature_name=MT-CO1	MT-CO1.value > threshold=-0.44641776382923126
node_5422: feature_name=TCF4	TCF4.value <= threshold=3.0345476865768433
Class: Healthy Control	

Rules_924	passed counts:4
node_0: feature_name=LTB	LTB.value > threshold=0.994031548500061
node_5264: feature_name=MT-CO1	MT-CO1.value <= threshold=-0.4127991199493408
node_5265: feature_name=MTRNR2L2	MTRNR2L2.value <= threshold=2.4473838806152344
node_5266: feature_name=TCL1A	TCL1A.value <= threshold=5.787256956100464
node_5267: feature_name=PLD4	PLD4.value <= threshold=3.544657826423645
node_5268: feature_name=NR4A1	NR4A1.value <= threshold=0.8490704298019409
node_5269: feature_name=LTB	LTB.value <= threshold=1.5252206921577454
node_5270: feature_name=HLA-DRB5	HLA-DRB5.value <= threshold=2.5117164850234985
node_5271: feature_name=CD79A	CD79A.value <= threshold=3.685201406478882
node_5272: feature_name=BCL2A1	BCL2A1.value <= threshold=2.081526756286621
node_5273: feature_name=HLA-DPA1	HLA-DPA1.value > threshold=1.8042166829109192
node_5363: feature_name=MS4A1	MS4A1.value <= threshold=3.5283708572387695
node_5364: feature_name=MT-CO3	MT-CO3.value <= threshold=0.3994715213775635
node_5365: feature_name=TNFAIP3	TNFAIP3.value <= threshold=0.2631131559610367
node_5366: feature_name=MT-CO1	MT-CO1.value <= threshold=-0.44641776382923126
node_5367: feature_name=GPX1	GPX1.value > threshold=0.7746693789958954
node_5417: feature_name=TNFAIP3	TNFAIP3.value > threshold=-0.46223947405815125
Class: COVID-19 Severe	
Rules_925	passed counts:4
node_0: feature_name=LTB	LTB.value > threshold=0.994031548500061
node_5264: feature_name=MT-CO1	MT-CO1.value <= threshold=-0.4127991199493408
node_5265: feature_name=MTRNR2L2	MTRNR2L2.value <= threshold=2.4473838806152344
node_5266: feature_name=TCL1A	TCL1A.value <= threshold=5.787256956100464
node_5267: feature_name=PLD4	PLD4.value <= threshold=3.544657826423645
node_5268: feature_name=NR4A1	NR4A1.value <= threshold=0.8490704298019409
node_5269: feature_name=LTB	LTB.value <= threshold=1.5252206921577454
node_5270: feature_name=HLA-DRB5	HLA-DRB5.value <= threshold=2.5117164850234985
node_5271: feature_name=CD79A	CD79A.value <= threshold=3.685201406478882

node_5272: feature_name=BCL2A1	BCL2A1.value <= threshold=2.081526756286621
node_5273: feature_name=HLA-DPA1	HLA-DPA1.value > threshold=1.8042166829109192
node_5363: feature_name=MS4A1	MS4A1.value <= threshold=3.5283708572387695
node_5364: feature_name=MT-CO3	MT-CO3.value <= threshold=0.3994715213775635
node_5365: feature_name=TNFAIP3	TNFAIP3.value <= threshold=0.2631131559610367
node_5366: feature_name=MT-CO1	MT-CO1.value <= threshold=-0.44641776382923126
node_5367: feature_name=GPX1	GPX1.value <= threshold=0.7746693789958954
node_5368: feature_name=MMD	MMD.value > threshold=3.176570177078247
node_5408: feature_name=MT-CO3	MT-CO3.value > threshold=-0.25999920815229416
node_5414: feature_name=FTH1	FTH1.value <= threshold=0.13472552224993706
Class: Healthy Control	
Rules_926	passed counts:4
node_0: feature_name=LTB	LTB.value > threshold=0.994031548500061
node_5264: feature_name=MT-CO1	MT-CO1.value <= threshold=-0.4127991199493408
node_5265: feature_name=MTRNR2L2	MTRNR2L2.value <= threshold=2.4473838806152344
node_5266: feature_name=TCL1A	TCL1A.value <= threshold=5.787256956100464
node_5267: feature_name=PLD4	PLD4.value <= threshold=3.544657826423645
node_5268: feature_name=NR4A1	NR4A1.value <= threshold=0.8490704298019409
node_5269: feature_name=LTB	LTB.value <= threshold=1.5252206921577454
node_5270: feature_name=HLA-DRB5	HLA-DRB5.value <= threshold=2.5117164850234985
node_5271: feature_name=CD79A	CD79A.value <= threshold=3.685201406478882
node_5272: feature_name=BCL2A1	BCL2A1.value <= threshold=2.081526756286621
node_5273: feature_name=HLA-DPA1	HLA-DPA1.value <= threshold=1.8042166829109192
node_5274: feature_name=HSP90B1	HSP90B1.value > threshold=-1.3115270137786865
node_5278: feature_name=OAZ1	OAZ1.value <= threshold=1.0802008509635925
node_5279: feature_name=CD79B	CD79B.value <= threshold=5.875581979751587
node_5280: feature_name=CCDC141	CCDC141.value <= threshold=6.439151048660278
node_5281: feature_name=SYTL3	SYTL3.value <= threshold=1.149337351322174
node_5282: feature_name=GPX1	GPX1.value > threshold=-1.0243326425552368

node_5284: feature_name=MMD	MMD.value <= threshold=6.431635856628418
node_5285: feature_name=MARCKS	MARCKS.value <= threshold=4.034798860549927
node_5286: feature_name=XIST	XIST.value <= threshold=1.148451752960682
node_5287: feature_name=CD83	CD83.value <= threshold=2.894522190093994
node_5288: feature_name=MIR24-2	MIR24-2.value <= threshold=2.76771080493927
node_5289: feature_name=GRASP	GRASP.value <= threshold=-0.4039058983325958
node_5290: feature_name=SLC7A5	SLC7A5.value <= threshold=-0.4947117418050766
node_5291: feature_name=LTB	LTB.value > threshold=1.2177766561508179
Class: Healthy Control	
Rules_927	passed counts:4
node_0: feature_name=LTB	LTB.value <= threshold=0.994031548500061
node_1: feature_name=MTRNR2L2	MTRNR2L2.value > threshold=2.8784297704696655
node_5207: feature_name=MTRNR2L8	MTRNR2L8.value <= threshold=3.1915172338485718
node_5208: feature_name=MT-CYB	MT-CYB.value <= threshold=1.3929389119148254
node_5209: feature_name=SLC7A5	SLC7A5.value <= threshold=3.205444812774658
node_5210: feature_name=MT-CO2	MT-CO2.value <= threshold=2.065874218940735
node_5211: feature_name=HLA-DRB5	HLA-DRB5.value <= threshold=2.276706099510193
node_5212: feature_name=TLR2	TLR2.value <= threshold=4.805860757827759
node_5213: feature_name=XIST	XIST.value <= threshold=4.313443303108215
node_5214: feature_name=TTN	TTN.value <= threshold=7.185946702957153
node_5215: feature_name=ITGAX	ITGAX.value <= threshold=3.106598973274231
node_5216: feature_name=PLEC	PLEC.value <= threshold=3.848032832145691
node_5217: feature_name=BCL2A1	BCL2A1.value > threshold=2.4758780002593994
node_5227: feature_name=MTRNR2L2	MTRNR2L2.value > threshold=4.1492650508880615
Class: COVID-19 Severe	
Rules_928	passed counts:4
node_0: feature_name=LTB	LTB.value <= threshold=0.994031548500061
node_1: feature_name=MTRNR2L2	MTRNR2L2.value <= threshold=2.8784297704696655

node_2: feature_name=TCL1A	TCL1A.value > threshold=4.798312664031982
node_4912: feature_name=MT-CO1	MT-CO1.value > threshold=-0.3664844036102295
node_5002: feature_name=IFI44L	IFI44L.value <= threshold=2.3101747035980225
node_5003: feature_name=SNX9	SNX9.value <= threshold=2.0247421264648438
node_5004: feature_name=STAG3	STAG3.value > threshold=6.482141733169556
node_5138: feature_name=CCDC141	CCDC141.value > threshold=-0.2267386093735695
node_5144: feature_name=JUN	JUN.value > threshold=0.2501271143555641
Class: Healthy Control	
Rules_929	passed counts:4
node_0: feature_name=LTB	LTB.value <= threshold=0.994031548500061
node_1: feature_name=MTRNR2L2	MTRNR2L2.value <= threshold=2.8784297704696655
node_2: feature_name=TCL1A	TCL1A.value > threshold=4.798312664031982
node_4912: feature_name=MT-CO1	MT-CO1.value > threshold=-0.3664844036102295
node_5002: feature_name=IFI44L	IFI44L.value <= threshold=2.3101747035980225
node_5003: feature_name=SNX9	SNX9.value <= threshold=2.0247421264648438
node_5004: feature_name=STAG3	STAG3.value <= threshold=6.482141733169556
node_5005: feature_name=BACH1	BACH1.value > threshold=-0.5237349569797516
node_5047: feature_name=RHOB	RHOB.value > threshold=-0.4843696355819702
node_5055: feature_name=FAM129C	FAM129C.value <= threshold=-0.2832885980606079
node_5056: feature_name=CD83	CD83.value > threshold=-0.5592367649078369
node_5060: feature_name=OAZ1	OAZ1.value <= threshold=0.7524859011173248
node_5061: feature_name=FCRL1	FCRL1.value <= threshold=5.677775859832764
node_5062: feature_name=RNF144B	RNF144B.value <= threshold=1.2688461616635323
node_5063: feature_name=ID1	ID1.value > threshold=-0.1296444833278656
node_5069: feature_name=SNX29P2	SNX29P2.value > threshold=-0.35507717728614807
Class: Healthy Control	
Rules_930	passed counts:4
node_0: feature_name=LTB	LTB.value <= threshold=0.994031548500061

node_1: feature_name=MTRNR2L2	MTRNR2L2.value <= threshold=2.8784297704696655
node_2: feature_name=TCL1A	TCL1A.value > threshold=4.798312664031982
node_4912: feature_name=MT-CO1	MT-CO1.value <= threshold=-0.3664844036102295
node_4913: feature_name=CD79A	CD79A.value <= threshold=2.987208843231201
node_4914: feature_name=HLA-DPB1	HLA-DPB1.value > threshold=2.0926655530929565
node_4958: feature_name=KIAA0125	KIAA0125.value <= threshold=-0.15961305052042007
node_4959: feature_name=PDLIM1	PDLIM1.value > threshold=3.826790690422058
Class: Healthy Control	
Rules_931	passed counts:4
node_0: feature_name=LTB	LTB.value <= threshold=0.994031548500061
node_1: feature_name=MTRNR2L2	MTRNR2L2.value <= threshold=2.8784297704696655
node_2: feature_name=TCL1A	TCL1A.value > threshold=4.798312664031982
node_4912: feature_name=MT-CO1	MT-CO1.value <= threshold=-0.3664844036102295
node_4913: feature_name=CD79A	CD79A.value <= threshold=2.987208843231201
node_4914: feature_name=HLA-DPB1	HLA-DPB1.value <= threshold=2.0926655530929565
node_4915: feature_name=MX1	MX1.value <= threshold=-0.4660506546497345
node_4916: feature_name=GPX1	GPX1.value > threshold=-0.5090344324707985
Class: Healthy Control	
Rules_932	passed counts:4
node_0: feature_name=LTB	LTB.value <= threshold=0.994031548500061
node_1: feature_name=MTRNR2L2	MTRNR2L2.value <= threshold=2.8784297704696655
node_2: feature_name=TCL1A	TCL1A.value <= threshold=4.798312664031982
node_3: feature_name=MT-CO1	MT-CO1.value > threshold=0.08947191759943962
node_2521: feature_name=LTB	LTB.value > threshold=0.6038667261600494
node_4173: feature_name=OAZ1	OAZ1.value > threshold=0.143283873796463
node_4615: feature_name=NR4A1	NR4A1.value <= threshold=0.6621004343032837
node_4616: feature_name=NFKBID	NFKBID.value <= threshold=3.283741593360901
node_4617: feature_name=HLA-DQA1	HLA-DQA1.value > threshold=2.3435752391815186

node_4719: feature_name=MX1	MX1.value <= threshold=1.851993978023529
node_4720: feature_name=JUN	JUN.value > threshold=-0.5025404393672943
node_4740: feature_name=IRF8	IRF8.value <= threshold=3.838419556617737
node_4741: feature_name=BACH1	BACH1.value > threshold=0.9568033516407013
node_4769: feature_name=TCF4	TCF4.value > threshold=-0.23278788477182388
node_4781: feature_name=ICAM1	ICAM1.value > threshold=-0.24220328778028488
node_4787: feature_name=S100A6	S100A6.value <= threshold=-0.8430019617080688
Class: COVID-19 Critical	
Rules_933	passed counts:4
node_0: feature_name=LTB	LTB.value <= threshold=0.994031548500061
node_1: feature_name=MTRNR2L2	MTRNR2L2.value <= threshold=2.8784297704696655
node_2: feature_name=TCL1A	TCL1A.value <= threshold=4.798312664031982
node_3: feature_name=MT-CO1	MT-CO1.value > threshold=0.08947191759943962
node_2521: feature_name=LTB	LTB.value > threshold=0.6038667261600494
node_4173: feature_name=OAZ1	OAZ1.value > threshold=0.143283873796463
node_4615: feature_name=NR4A1	NR4A1.value <= threshold=0.6621004343032837
node_4616: feature_name=NFKBID	NFKBID.value <= threshold=3.283741593360901
node_4617: feature_name=HLA-DQA1	HLA-DQA1.value > threshold=2.3435752391815186
node_4719: feature_name=MX1	MX1.value <= threshold=1.851993978023529
node_4720: feature_name=JUN	JUN.value > threshold=-0.5025404393672943
node_4740: feature_name=IRF8	IRF8.value <= threshold=3.838419556617737
node_4741: feature_name=BACH1	BACH1.value > threshold=0.9568033516407013
node_4769: feature_name=TCF4	TCF4.value <= threshold=-0.23278788477182388
node_4770: feature_name=FERMT3	FERMT3.value > threshold=1.1583285331726074
Class: Healthy Control	
Rules_934	passed counts:4
node_0: feature_name=LTB	LTB.value <= threshold=0.994031548500061
node_1: feature_name=MTRNR2L2	MTRNR2L2.value <= threshold=2.8784297704696655

node_2: feature_name=TCL1A	TCL1A.value <= threshold=4.798312664031982
node_3: feature_name=MT-CO1	MT-CO1.value > threshold=0.08947191759943962
node_2521: feature_name=LTB	LTB.value > threshold=0.6038667261600494
node_4173: feature_name=OAZ1	OAZ1.value > threshold=0.143283873796463
node_4615: feature_name=NR4A1	NR4A1.value <= threshold=0.6621004343032837
node_4616: feature_name=NFKBID	NFKBID.value <= threshold=3.283741593360901
node_4617: feature_name=HLA-DQA1	HLA-DQA1.value <= threshold=2.3435752391815186
node_4618: feature_name=PELI1	PELI1.value > threshold=1.3271203637123108
node_4680: feature_name=BANK1	BANK1.value > threshold=-0.3607018291950226
node_4682: feature_name=RGCC	RGCC.value > threshold=0.60355344414711
Class: Healthy Control	
Rules_935	passed counts:4
node_0: feature_name=LTB	LTB.value <= threshold=0.994031548500061
node_1: feature_name=MTRNR2L2	MTRNR2L2.value <= threshold=2.8784297704696655
node_2: feature_name=TCL1A	TCL1A.value <= threshold=4.798312664031982
node_3: feature_name=MT-CO1	MT-CO1.value > threshold=0.08947191759943962
node_2521: feature_name=LTB	LTB.value > threshold=0.6038667261600494
node_4173: feature_name=OAZ1	OAZ1.value > threshold=0.143283873796463
node_4615: feature_name=NR4A1	NR4A1.value <= threshold=0.6621004343032837
node_4616: feature_name=NFKBID	NFKBID.value <= threshold=3.283741593360901
node_4617: feature_name=HLA-DQA1	HLA-DQA1.value <= threshold=2.3435752391815186
node_4618: feature_name=PELI1	PELI1.value > threshold=1.3271203637123108
node_4680: feature_name=BANK1	BANK1.value > threshold=-0.3607018291950226
node_4682: feature_name=RGCC	RGCC.value <= threshold=0.60355344414711
node_4683: feature_name=SNX29P2	SNX29P2.value > threshold=4.192138910293579
Class: Healthy Control	
Rules_936	passed counts:4
node_0: feature_name=LTB	LTB.value <= threshold=0.994031548500061

node_1: feature_name=MTRNR2L2	MTRNR2L2.value <= threshold=2.8784297704696655
node_2: feature_name=TCL1A	TCL1A.value <= threshold=4.798312664031982
node_3: feature_name=MT-CO1	MT-CO1.value > threshold=0.08947191759943962
node_2521: feature_name=LTB	LTB.value > threshold=0.6038667261600494
node_4173: feature_name=OAZ1	OAZ1.value > threshold=0.143283873796463
node_4615: feature_name=NR4A1	NR4A1.value <= threshold=0.6621004343032837
node_4616: feature_name=NFKBID	NFKBID.value <= threshold=3.283741593360901
node_4617: feature_name=HLA-DQA1	HLA-DQA1.value <= threshold=2.3435752391815186
node_4618: feature_name=PELI1	PELI1.value > threshold=1.3271203637123108
node_4680: feature_name=BANK1	BANK1.value <= threshold=-0.3607018291950226
Class: COVID-19 Severe	
Rules_937	passed counts:4
node_0: feature_name=LTB	LTB.value <= threshold=0.994031548500061
node_1: feature_name=MTRNR2L2	MTRNR2L2.value <= threshold=2.8784297704696655
node_2: feature_name=TCL1A	TCL1A.value <= threshold=4.798312664031982
node_3: feature_name=MT-CO1	MT-CO1.value > threshold=0.08947191759943962
node_2521: feature_name=LTB	LTB.value > threshold=0.6038667261600494
node_4173: feature_name=OAZ1	OAZ1.value > threshold=0.143283873796463
node_4615: feature_name=NR4A1	NR4A1.value <= threshold=0.6621004343032837
node_4616: feature_name=NFKBID	NFKBID.value <= threshold=3.283741593360901
node_4617: feature_name=HLA-DQA1	HLA-DQA1.value <= threshold=2.3435752391815186
node_4618: feature_name=PELI1	PELI1.value <= threshold=1.3271203637123108
node_4619: feature_name=PLD4	PLD4.value <= threshold=3.3385335206985474
node_4620: feature_name=MEF2A	MEF2A.value > threshold=1.5926940441131592
node_4658: feature_name=STAG3	STAG3.value <= threshold=-0.2016931176185608
node_4659: feature_name=HLA-DQB1	HLA-DQB1.value > threshold=1.983833909034729
node_4665: feature_name=MTRNR2L2	MTRNR2L2.value <= threshold=-0.2939942181110382
Class: COVID-19 Severe	

Rules_938	passed counts:4
node_0: feature_name=LTB	LTB.value <= threshold=0.994031548500061
node_1: feature_name=MTRNR2L2	MTRNR2L2.value <= threshold=2.8784297704696655
node_2: feature_name=TCL1A	TCL1A.value <= threshold=4.798312664031982
node_3: feature_name=MT-CO1	MT-CO1.value > threshold=0.08947191759943962
node_2521: feature_name=LTB	LTB.value > threshold=0.6038667261600494
node_4173: feature_name=OAZ1	OAZ1.value <= threshold=0.143283873796463
node_4174: feature_name=PLD4	PLD4.value <= threshold=2.921932101249695
node_4175: feature_name=XIST	XIST.value > threshold=-0.05067425221204758
node_4539: feature_name=HLA-DQB1	HLA-DQB1.value <= threshold=2.1246572732925415
node_4540: feature_name=ICAM1	ICAM1.value > threshold=-0.22761741280555725
node_4544: feature_name=GRASP	GRASP.value <= threshold=0.3840908855199814
Class: COVID-19 Critical	
Rules_939	passed counts:4
node_0: feature_name=LTB	LTB.value <= threshold=0.994031548500061
node_1: feature_name=MTRNR2L2	MTRNR2L2.value <= threshold=2.8784297704696655
node_2: feature_name=TCL1A	TCL1A.value <= threshold=4.798312664031982
node_3: feature_name=MT-CO1	MT-CO1.value > threshold=0.08947191759943962
node_2521: feature_name=LTB	LTB.value > threshold=0.6038667261600494
node_4173: feature_name=OAZ1	OAZ1.value <= threshold=0.143283873796463
node_4174: feature_name=PLD4	PLD4.value <= threshold=2.921932101249695
node_4175: feature_name=XIST	XIST.value <= threshold=-0.05067425221204758
node_4176: feature_name=MT-CO1	MT-CO1.value > threshold=1.345594048500061
node_4486: feature_name=RALGPS2	RALGPS2.value > threshold=2.7596691846847534
node_4508: feature_name=SNX9	SNX9.value <= threshold=2.5712826251983643
node_4509: feature_name=NFKBIZ	NFKBIZ.value <= threshold=-0.8055458068847656
Class: COVID-19 Critical	
Rules_940	passed counts:4

node_0: feature_name=LTB	LTB.value <= threshold=0.994031548500061
node_1: feature_name=MTRNR2L2	MTRNR2L2.value <= threshold=2.8784297704696655
node_2: feature_name=TCL1A	TCL1A.value <= threshold=4.798312664031982
node_3: feature_name=MT-CO1	MT-CO1.value > threshold=0.08947191759943962
node_2521: feature_name=LTB	LTB.value > threshold=0.6038667261600494
node_4173: feature_name=OAZ1	OAZ1.value <= threshold=0.143283873796463
node_4174: feature_name=PLD4	PLD4.value <= threshold=2.921932101249695
node_4175: feature_name=XIST	XIST.value <= threshold=-0.05067425221204758
node_4176: feature_name=MT-CO1	MT-CO1.value <= threshold=1.345594048500061
node_4177: feature_name=HLA-DQB1	HLA-DQB1.value > threshold=1.927736520767212
node_4353: feature_name=ITM2C	ITM2C.value <= threshold=-0.12329206243157387
node_4354: feature_name=MX1	MX1.value > threshold=-0.4356398284435272
node_4430: feature_name=CD83	CD83.value <= threshold=3.929547071456909
node_4431: feature_name=SNX9	SNX9.value > threshold=3.212221622467041
node_4447: feature_name=HIST1H2AC	HIST1H2AC.value > threshold=-0.338812991976738
Class: COVID-19 Severe	
Rules_941	passed counts:4
node_0: feature_name=LTB	LTB.value <= threshold=0.994031548500061
node_1: feature_name=MTRNR2L2	MTRNR2L2.value <= threshold=2.8784297704696655
node_2: feature_name=TCL1A	TCL1A.value <= threshold=4.798312664031982
node_3: feature_name=MT-CO1	MT-CO1.value > threshold=0.08947191759943962
node_2521: feature_name=LTB	LTB.value > threshold=0.6038667261600494
node_4173: feature_name=OAZ1	OAZ1.value <= threshold=0.143283873796463
node_4174: feature_name=PLD4	PLD4.value <= threshold=2.921932101249695
node_4175: feature_name=XIST	XIST.value <= threshold=-0.05067425221204758
node_4176: feature_name=MT-CO1	MT-CO1.value <= threshold=1.345594048500061
node_4177: feature_name=HLA-DQB1	HLA-DQB1.value > threshold=1.927736520767212
node_4353: feature_name=ITM2C	ITM2C.value <= threshold=-0.12329206243157387
node_4354: feature_name=MX1	MX1.value <= threshold=-0.4356398284435272

node_4355: feature_name=HLA-DQB1	HLA-DQB1.value > threshold=2.8865538835525513
node_4419: feature_name=DAPP1	DAPP1.value > threshold=-0.30035753548145294
node_4425: feature_name=RGS2	RGS2.value > threshold=0.5943844318389893
node_4427: feature_name=ATF3	ATF3.value > threshold=-0.36968526244163513
Class: COVID-19 Severe	
Rules_942	passed counts:4
node_0: feature_name=LTB	LTB.value <= threshold=0.994031548500061
node_1: feature_name=MTRNR2L2	MTRNR2L2.value <= threshold=2.8784297704696655
node_2: feature_name=TCL1A	TCL1A.value <= threshold=4.798312664031982
node_3: feature_name=MT-CO1	MT-CO1.value > threshold=0.08947191759943962
node_2521: feature_name=LTB	LTB.value > threshold=0.6038667261600494
node_4173: feature_name=OAZ1	OAZ1.value <= threshold=0.143283873796463
node_4174: feature_name=PLD4	PLD4.value <= threshold=2.921932101249695
node_4175: feature_name=XIST	XIST.value <= threshold=-0.05067425221204758
node_4176: feature_name=MT-CO1	MT-CO1.value <= threshold=1.345594048500061
node_4177: feature_name=HLA-DQB1	HLA-DQB1.value > threshold=1.927736520767212
node_4353: feature_name=ITM2C	ITM2C.value <= threshold=-0.12329206243157387
node_4354: feature_name=MX1	MX1.value <= threshold=-0.4356398284435272
node_4355: feature_name=HLA-DQB1	HLA-DQB1.value <= threshold=2.8865538835525513
node_4356: feature_name=SNX9	SNX9.value <= threshold=1.002076417207718
node_4357: feature_name=FAM160B1	FAM160B1.value > threshold=-0.42920351028442383
node_4361: feature_name=SNX9	SNX9.value <= threshold=0.011789441108703613
node_4362: feature_name=MT-CO3	MT-CO3.value <= threshold=0.4059640020132065
node_4363: feature_name=RNF11	RNF11.value <= threshold=-0.5651727318763733
Class: COVID-19 Severe	
Rules_943	passed counts:4
node_0: feature_name=LTB	LTB.value <= threshold=0.994031548500061
node_1: feature_name=MTRNR2L2	MTRNR2L2.value <= threshold=2.8784297704696655

node_2: feature_name=TCL1A	TCL1A.value <= threshold=4.798312664031982
node_3: feature_name=MT-CO1	MT-CO1.value > threshold=0.08947191759943962
node_2521: feature_name=LTB	LTB.value > threshold=0.6038667261600494
node_4173: feature_name=OAZ1	OAZ1.value <= threshold=0.143283873796463
node_4174: feature_name=PLD4	PLD4.value <= threshold=2.921932101249695
node_4175: feature_name=XIST	XIST.value <= threshold=-0.05067425221204758
node_4176: feature_name=MT-CO1	MT-CO1.value <= threshold=1.345594048500061
node_4177: feature_name=HLA-DQB1	HLA-DQB1.value <= threshold=1.927736520767212
node_4178: feature_name=BACH1	BACH1.value > threshold=-0.5349778532981873
node_4236: feature_name=TPM1	TPM1.value > threshold=3.516477584838867
Class: Healthy Control	
Rules_944	passed counts:4
node_0: feature_name=LTB	LTB.value <= threshold=0.994031548500061
node_1: feature_name=MTRNR2L2	MTRNR2L2.value <= threshold=2.8784297704696655
node_2: feature_name=TCL1A	TCL1A.value <= threshold=4.798312664031982
node_3: feature_name=MT-CO1	MT-CO1.value > threshold=0.08947191759943962
node_2521: feature_name=LTB	LTB.value > threshold=0.6038667261600494
node_4173: feature_name=OAZ1	OAZ1.value <= threshold=0.143283873796463
node_4174: feature_name=PLD4	PLD4.value <= threshold=2.921932101249695
node_4175: feature_name=XIST	XIST.value <= threshold=-0.05067425221204758
node_4176: feature_name=MT-CO1	MT-CO1.value <= threshold=1.345594048500061
node_4177: feature_name=HLA-DQB1	HLA-DQB1.value <= threshold=1.927736520767212
node_4178: feature_name=BACH1	BACH1.value > threshold=-0.5349778532981873
node_4236: feature_name=TPM1	TPM1.value <= threshold=3.516477584838867
node_4237: feature_name=NR4A1	NR4A1.value > threshold=-0.274935707449913
node_4277: feature_name=HLA-DPA1	HLA-DPA1.value <= threshold=1.8894080519676208
node_4278: feature_name=TRAF1	TRAF1.value <= threshold=2.091539978981018
node_4279: feature_name=STMN1	STMN1.value <= threshold=2.2198781967163086
node_4280: feature_name=PLD4	PLD4.value <= threshold=2.7647364139556885

node_4281: feature_name=IGJ	IGJ.value <= threshold=4.225788354873657
node_4282: feature_name=MEF2C	MEF2C.value > threshold=3.573865532875061
node_4328: feature_name=HLA-DPB1	HLA-DPB1.value <= threshold=1.5301921963691711
node_4329: feature_name=TLN1	TLN1.value > threshold=-0.5842107534408569
Class: Healthy Control	
Rules_945	passed counts:4
node_0: feature_name=LTB	LTB.value <= threshold=0.994031548500061
node_1: feature_name=MTRNR2L2	MTRNR2L2.value <= threshold=2.8784297704696655
node_2: feature_name=TCL1A	TCL1A.value <= threshold=4.798312664031982
node_3: feature_name=MT-CO1	MT-CO1.value > threshold=0.08947191759943962
node_2521: feature_name=LTB	LTB.value > threshold=0.6038667261600494
node_4173: feature_name=OAZ1	OAZ1.value <= threshold=0.143283873796463
node_4174: feature_name=PLD4	PLD4.value <= threshold=2.921932101249695
node_4175: feature_name=XIST	XIST.value <= threshold=-0.05067425221204758
node_4176: feature_name=MT-CO1	MT-CO1.value <= threshold=1.345594048500061
node_4177: feature_name=HLA-DQB1	HLA-DQB1.value <= threshold=1.927736520767212
node_4178: feature_name=BACH1	BACH1.value > threshold=-0.5349778532981873
node_4236: feature_name=TPM1	TPM1.value <= threshold=3.516477584838867
node_4237: feature_name=NR4A1	NR4A1.value <= threshold=-0.274935707449913
node_4238: feature_name=VPREB3	VPREB3.value <= threshold=5.024149179458618
node_4239: feature_name=YWHAH	YWHAH.value <= threshold=4.7504096031188965
node_4240: feature_name=NAIP	NAIP.value <= threshold=4.030007481575012
node_4241: feature_name=CD38	CD38.value > threshold=-0.3387429714202881
node_4243: feature_name=MS4A1	MS4A1.value <= threshold=5.195746660232544
node_4244: feature_name=TRIB1	TRIB1.value <= threshold=2.9039160013198853
node_4245: feature_name=RGS2	RGS2.value > threshold=-0.8980970978736877
node_4249: feature_name=PLD4	PLD4.value <= threshold=2.8284080028533936
node_4250: feature_name=GRAP2	GRAP2.value <= threshold=2.0700820088386536
node_4251: feature_name=ATF3	ATF3.value > threshold=2.327788233757019

node_4257: feature_name=SPI1	SPI1.value <= threshold=0.25308896601200104
Class: COVID-19 Critical	
Rules_946	passed counts:4
node_0: feature_name=LTB	LTB.value <= threshold=0.994031548500061
node_1: feature_name=MTRNR2L2	MTRNR2L2.value <= threshold=2.8784297704696655
node_2: feature_name=TCL1A	TCL1A.value <= threshold=4.798312664031982
node_3: feature_name=MT-CO1	MT-CO1.value > threshold=0.08947191759943962
node_2521: feature_name=LTB	LTB.value > threshold=0.6038667261600494
node_4173: feature_name=OAZ1	OAZ1.value <= threshold=0.143283873796463
node_4174: feature_name=PLD4	PLD4.value <= threshold=2.921932101249695
node_4175: feature_name=XIST	XIST.value <= threshold=-0.05067425221204758
node_4176: feature_name=MT-CO1	MT-CO1.value <= threshold=1.345594048500061
node_4177: feature_name=HLA-DQB1	HLA-DQB1.value <= threshold=1.927736520767212
node_4178: feature_name=BACH1	BACH1.value <= threshold=-0.5349778532981873
node_4179: feature_name=CD79B	CD79B.value <= threshold=1.6518195271492004
node_4180: feature_name=FTH1	FTH1.value <= threshold=-1.4486562609672546
node_4181: feature_name=CD83	CD83.value > threshold=1.2222885489463806
Class: Healthy Control	
Rules_947	passed counts:4
node_0: feature_name=LTB	LTB.value <= threshold=0.994031548500061
node_1: feature_name=MTRNR2L2	MTRNR2L2.value <= threshold=2.8784297704696655
node_2: feature_name=TCL1A	TCL1A.value <= threshold=4.798312664031982
node_3: feature_name=MT-CO1	MT-CO1.value > threshold=0.08947191759943962
node_2521: feature_name=LTB	LTB.value <= threshold=0.6038667261600494
node_2522: feature_name=HLA-DQB1	HLA-DQB1.value > threshold=2.1053054332733154
node_3550: feature_name=XIST	XIST.value > threshold=0.7716337237507105
node_4140: feature_name=HLA-DQA2	HLA-DQA2.value > threshold=-0.1354314684867859
node_4158: feature_name=MT-CO3	MT-CO3.value > threshold=0.5339524000883102

node_4166: feature_name=IL1B	IL1B.value > threshold=-0.16591781377792358
node_4168: feature_name=TSPYL2	TSPYL2.value > threshold=-0.8189249932765961
Class: COVID-19 Severe	
Rules_948	passed counts:4
node_0: feature_name=LTB	LTB.value <= threshold=0.994031548500061
node_1: feature_name=MTRNR2L2	MTRNR2L2.value <= threshold=2.8784297704696655
node_2: feature_name=TCL1A	TCL1A.value <= threshold=4.798312664031982
node_3: feature_name=MT-CO1	MT-CO1.value > threshold=0.08947191759943962
node_2521: feature_name=LTB	LTB.value <= threshold=0.6038667261600494
node_2522: feature_name=HLA-DQB1	HLA-DQB1.value > threshold=2.1053054332733154
node_3550: feature_name=XIST	XIST.value <= threshold=0.7716337237507105
node_3551: feature_name=CD79B	CD79B.value > threshold=2.9426796436309814
node_4039: feature_name=IFI44L	IFI44L.value <= threshold=1.8637521862983704
node_4040: feature_name=SNX9	SNX9.value > threshold=1.274232804775238
node_4096: feature_name=RALGPS2	RALGPS2.value > threshold=2.3599878549575806
node_4118: feature_name=SNX29P2	SNX29P2.value <= threshold=0.880477637052536
node_4119: feature_name=FAM129C	FAM129C.value > threshold=3.415250778198242
node_4125: feature_name=IGJ	IGJ.value <= threshold=-0.2685837149620056
node_4126: feature_name=S100A6	S100A6.value <= threshold=-1.4672366380691528
Class: COVID-19 Critical	
Rules_949	passed counts:4
node_0: feature_name=LTB	LTB.value <= threshold=0.994031548500061
node_1: feature_name=MTRNR2L2	MTRNR2L2.value <= threshold=2.8784297704696655
node_2: feature_name=TCL1A	TCL1A.value <= threshold=4.798312664031982
node_3: feature_name=MT-CO1	MT-CO1.value > threshold=0.08947191759943962
node_2521: feature_name=LTB	LTB.value <= threshold=0.6038667261600494
node_2522: feature_name=HLA-DQB1	HLA-DQB1.value > threshold=2.1053054332733154
node_3550: feature_name=XIST	XIST.value <= threshold=0.7716337237507105

node_3551: feature_name=CD79B	CD79B.value > threshold=2.9426796436309814
node_4039: feature_name=IFI44L	IFI44L.value <= threshold=1.8637521862983704
node_4040: feature_name=SNX9	SNX9.value <= threshold=1.274232804775238
node_4041: feature_name=CASP8	CASP8.value > threshold=-0.574097603559494
node_4073: feature_name=HLA-DRB5	HLA-DRB5.value > threshold=1.4346495270729065
node_4083: feature_name=NT5C3A	NT5C3A.value <= threshold=-0.45546136796474457
node_4084: feature_name=OAZ1	OAZ1.value <= threshold=0.1348155364394188
node_4085: feature_name=NAMPT	NAMPT.value <= threshold=-0.6454389691352844
node_4086: feature_name=AREGB	AREGB.value <= threshold=-0.10599542036652565
Class: Healthy Control	
Rules_950	passed counts:4
node_0: feature_name=LTB	LTB.value <= threshold=0.994031548500061
node_1: feature_name=MTRNR2L2	MTRNR2L2.value <= threshold=2.8784297704696655
node_2: feature_name=TCL1A	TCL1A.value <= threshold=4.798312664031982
node_3: feature_name=MT-CO1	MT-CO1.value > threshold=0.08947191759943962
node_2521: feature_name=LTB	LTB.value <= threshold=0.6038667261600494
node_2522: feature_name=HLA-DQB1	HLA-DQB1.value > threshold=2.1053054332733154
node_3550: feature_name=XIST	XIST.value <= threshold=0.7716337237507105
node_3551: feature_name=CD79B	CD79B.value <= threshold=2.9426796436309814
node_3552: feature_name=HLA-DQB1	HLA-DQB1.value > threshold=2.713674306869507
node_3854: feature_name=MEF2C	MEF2C.value > threshold=-0.3721291869878769
node_3882: feature_name=IFI44L	IFI44L.value <= threshold=2.5516022443771362
node_3883: feature_name=FCER2	FCER2.value > threshold=-0.21487552672624588
node_3977: feature_name=IGJ	IGJ.value <= threshold=2.123470664024353
node_3978: feature_name=S100A6	S100A6.value > threshold=-1.7107264399528503
node_3982: feature_name=MT-CO1	MT-CO1.value <= threshold=1.5725501775741577
node_3983: feature_name=SNX29P2	SNX29P2.value <= threshold=3.1593780517578125
node_3984: feature_name=IL8	IL8.value > threshold=-0.28026726841926575
Class: COVID-19 Severe	

Rules_951	passed counts:4
node_0: feature_name=LTB	LTB.value <= threshold=0.994031548500061
node_1: feature_name=MTRNR2L2	MTRNR2L2.value <= threshold=2.8784297704696655
node_2: feature_name=TCL1A	TCL1A.value <= threshold=4.798312664031982
node_3: feature_name=MT-CO1	MT-CO1.value > threshold=0.08947191759943962
node_2521: feature_name=LTB	LTB.value <= threshold=0.6038667261600494
node_2522: feature_name=HLA-DQB1	HLA-DQB1.value > threshold=2.1053054332733154
node_3550: feature_name=XIST	XIST.value <= threshold=0.7716337237507105
node_3551: feature_name=CD79B	CD79B.value <= threshold=2.9426796436309814
node_3552: feature_name=HLA-DQB1	HLA-DQB1.value > threshold=2.713674306869507
node_3854: feature_name=MEF2C	MEF2C.value > threshold=-0.3721291869878769
node_3882: feature_name=IFI44L	IFI44L.value <= threshold=2.5516022443771362
node_3883: feature_name=FCER2	FCER2.value > threshold=-0.21487552672624588
node_3977: feature_name=IGJ	IGJ.value <= threshold=2.123470664024353
node_3978: feature_name=S100A6	S100A6.value > threshold=-1.7107264399528503
node_3982: feature_name=MT-CO1	MT-CO1.value <= threshold=1.5725501775741577
node_3983: feature_name=SNX29P2	SNX29P2.value <= threshold=3.1593780517578125
node_3984: feature_name=IL8	IL8.value <= threshold=-0.28026726841926575
node_3985: feature_name=NAMPT	NAMPT.value > threshold=-0.5902779400348663
node_3991: feature_name=PELI1	PELI1.value <= threshold=3.5653313398361206
node_3992: feature_name=IRF8	IRF8.value <= threshold=-0.34819670021533966
node_3993: feature_name=NFKBID	NFKBID.value > threshold=1.719421625137329
Class: COVID-19 Critical	
Rules_952	passed counts:4
node_0: feature_name=LTB	LTB.value <= threshold=0.994031548500061
node_1: feature_name=MTRNR2L2	MTRNR2L2.value <= threshold=2.8784297704696655
node_2: feature_name=TCL1A	TCL1A.value <= threshold=4.798312664031982
node_3: feature_name=MT-CO1	MT-CO1.value > threshold=0.08947191759943962

node_2521: feature_name=LTB	LTB.value <= threshold=0.6038667261600494
node_2522: feature_name=HLA-DQB1	HLA-DQB1.value > threshold=2.1053054332733154
node_3550: feature_name=XIST	XIST.value <= threshold=0.7716337237507105
node_3551: feature_name=CD79B	CD79B.value <= threshold=2.9426796436309814
node_3552: feature_name=HLA-DQB1	HLA-DQB1.value > threshold=2.713674306869507
node_3854: feature_name=MEF2C	MEF2C.value > threshold=-0.3721291869878769
node_3882: feature_name=IFI44L	IFI44L.value <= threshold=2.5516022443771362
node_3883: feature_name=FCER2	FCER2.value > threshold=-0.21487552672624588
node_3977: feature_name=IGJ	IGJ.value <= threshold=2.123470664024353
node_3978: feature_name=S100A6	S100A6.value > threshold=-1.7107264399528503
node_3982: feature_name=MT-CO1	MT-CO1.value <= threshold=1.5725501775741577
node_3983: feature_name=SNX29P2	SNX29P2.value <= threshold=3.1593780517578125
node_3984: feature_name=IL8	IL8.value <= threshold=-0.28026726841926575
node_3985: feature_name=NAMPT	NAMPT.value <= threshold=-0.5902779400348663
node_3986: feature_name=HLA-DQA1	HLA-DQA1.value > threshold=2.868778705596924
Class: Healthy Control	
Rules_953	passed counts:4
node_0: feature_name=LTB	LTB.value <= threshold=0.994031548500061
node_1: feature_name=MTRNR2L2	MTRNR2L2.value <= threshold=2.8784297704696655
node_2: feature_name=TCL1A	TCL1A.value <= threshold=4.798312664031982
node_3: feature_name=MT-CO1	MT-CO1.value > threshold=0.08947191759943962
node_2521: feature_name=LTB	LTB.value <= threshold=0.6038667261600494
node_2522: feature_name=HLA-DQB1	HLA-DQB1.value > threshold=2.1053054332733154
node_3550: feature_name=XIST	XIST.value <= threshold=0.7716337237507105
node_3551: feature_name=CD79B	CD79B.value <= threshold=2.9426796436309814
node_3552: feature_name=HLA-DQB1	HLA-DQB1.value > threshold=2.713674306869507
node_3854: feature_name=MEF2C	MEF2C.value > threshold=-0.3721291869878769
node_3882: feature_name=IFI44L	IFI44L.value <= threshold=2.5516022443771362
node_3883: feature_name=FCER2	FCER2.value <= threshold=-0.21487552672624588

node_3884: feature_name=HLA-DQA2	HLA-DQA2.value <= threshold=-0.12580305337905884
node_3885: feature_name=SLC7A5	SLC7A5.value > threshold=-0.4906871169805527
node_3945: feature_name=HLA-DQA1	HLA-DQA1.value <= threshold=1.6766853332519531
node_3946: feature_name=FTH1	FTH1.value > threshold=-0.733003418892622
Class: COVID-19 Severe	
Rules_954	passed counts:4
node_0: feature_name=LTB	LTB.value <= threshold=0.994031548500061
node_1: feature_name=MTRNR2L2	MTRNR2L2.value <= threshold=2.8784297704696655
node_2: feature_name=TCL1A	TCL1A.value <= threshold=4.798312664031982
node_3: feature_name=MT-CO1	MT-CO1.value > threshold=0.08947191759943962
node_2521: feature_name=LTB	LTB.value <= threshold=0.6038667261600494
node_2522: feature_name=HLA-DQB1	HLA-DQB1.value > threshold=2.1053054332733154
node_3550: feature_name=XIST	XIST.value <= threshold=0.7716337237507105
node_3551: feature_name=CD79B	CD79B.value <= threshold=2.9426796436309814
node_3552: feature_name=HLA-DQB1	HLA-DQB1.value > threshold=2.713674306869507
node_3854: feature_name=MEF2C	MEF2C.value > threshold=-0.3721291869878769
node_3882: feature_name=IFI44L	IFI44L.value <= threshold=2.5516022443771362
node_3883: feature_name=FCER2	FCER2.value <= threshold=-0.21487552672624588
node_3884: feature_name=HLA-DQA2	HLA-DQA2.value <= threshold=-0.12580305337905884
node_3885: feature_name=SLC7A5	SLC7A5.value <= threshold=-0.4906871169805527
node_3886: feature_name=VPREB3	VPREB3.value <= threshold=5.492124557495117
node_3887: feature_name=CYBB	CYBB.value > threshold=2.720046043395996
node_3935: feature_name=MT-CO1	MT-CO1.value <= threshold=1.21793794631958
node_3936: feature_name=RHOB	RHOB.value <= threshold=-0.3108775019645691
Class: COVID-19 Severe	
Rules_955	passed counts:4
node_0: feature_name=LTB	LTB.value <= threshold=0.994031548500061
node_1: feature_name=MTRNR2L2	MTRNR2L2.value <= threshold=2.8784297704696655

node_2: feature_name=TCL1A	TCL1A.value <= threshold=4.798312664031982
node_3: feature_name=MT-CO1	MT-CO1.value > threshold=0.08947191759943962
node_2521: feature_name=LTB	LTB.value <= threshold=0.6038667261600494
node_2522: feature_name=HLA-DQB1	HLA-DQB1.value > threshold=2.1053054332733154
node_3550: feature_name=XIST	XIST.value <= threshold=0.7716337237507105
node_3551: feature_name=CD79B	CD79B.value <= threshold=2.9426796436309814
node_3552: feature_name=HLA-DQB1	HLA-DQB1.value > threshold=2.713674306869507
node_3854: feature_name=MEF2C	MEF2C.value > threshold=-0.3721291869878769
node_3882: feature_name=IFI44L	IFI44L.value <= threshold=2.5516022443771362
node_3883: feature_name=FCER2	FCER2.value <= threshold=-0.21487552672624588
node_3884: feature_name=HLA-DQA2	HLA-DQA2.value <= threshold=-0.12580305337905884
node_3885: feature_name=SLC7A5	SLC7A5.value <= threshold=-0.4906871169805527
node_3886: feature_name=VPREB3	VPREB3.value <= threshold=5.492124557495117
node_3887: feature_name=CYBB	CYBB.value <= threshold=2.720046043395996
node_3888: feature_name=CD79A	CD79A.value <= threshold=3.3531476259231567
node_3889: feature_name=HLA-DPA1	HLA-DPA1.value > threshold=1.6232598423957825
node_3911: feature_name=HLA-DQA1	HLA-DQA1.value > threshold=3.2045756578445435
node_3923: feature_name=NAMPT	NAMPT.value > threshold=-0.45362021028995514
node_3925: feature_name=PARP14	PARP14.value <= threshold=1.1388885080814362
Class: Healthy Control	
Rules_956	passed counts:4
node_0: feature_name=LTB	LTB.value <= threshold=0.994031548500061
node_1: feature_name=MTRNR2L2	MTRNR2L2.value <= threshold=2.8784297704696655
node_2: feature_name=TCL1A	TCL1A.value <= threshold=4.798312664031982
node_3: feature_name=MT-CO1	MT-CO1.value > threshold=0.08947191759943962
node_2521: feature_name=LTB	LTB.value <= threshold=0.6038667261600494
node_2522: feature_name=HLA-DQB1	HLA-DQB1.value > threshold=2.1053054332733154
node_3550: feature_name=XIST	XIST.value <= threshold=0.7716337237507105
node_3551: feature_name=CD79B	CD79B.value <= threshold=2.9426796436309814

node_3552: feature_name=HLA-DQB1	HLA-DQB1.value <= threshold=2.713674306869507
node_3553: feature_name=SNX9	SNX9.value > threshold=-0.5404067635536194
node_3653: feature_name=LTB	LTB.value > threshold=0.021708179265260696
node_3759: feature_name=TCL1A	TCL1A.value > threshold=3.3921085596084595
node_3841: feature_name=NFKBIZ	NFKBIZ.value > threshold=0.3082800954580307
node_3849: feature_name=HLA-DQA2	HLA-DQA2.value <= threshold=-0.24445389211177826
Class: COVID-19 Severe	
Rules_957	passed counts:4
node_0: feature_name=LTB	LTB.value <= threshold=0.994031548500061
node_1: feature_name=MTRNR2L2	MTRNR2L2.value <= threshold=2.8784297704696655
node_2: feature_name=TCL1A	TCL1A.value <= threshold=4.798312664031982
node_3: feature_name=MT-CO1	MT-CO1.value > threshold=0.08947191759943962
node_2521: feature_name=LTB	LTB.value <= threshold=0.6038667261600494
node_2522: feature_name=HLA-DQB1	HLA-DQB1.value > threshold=2.1053054332733154
node_3550: feature_name=XIST	XIST.value <= threshold=0.7716337237507105
node_3551: feature_name=CD79B	CD79B.value <= threshold=2.9426796436309814
node_3552: feature_name=HLA-DQB1	HLA-DQB1.value <= threshold=2.713674306869507
node_3553: feature_name=SNX9	SNX9.value > threshold=-0.5404067635536194
node_3653: feature_name=LTB	LTB.value > threshold=0.021708179265260696
node_3759: feature_name=TCL1A	TCL1A.value <= threshold=3.3921085596084595
node_3760: feature_name=HLA-DRB5	HLA-DRB5.value > threshold=0.5265218019485474
node_3776: feature_name=ID1	ID1.value <= threshold=-0.21235637366771698
node_3777: feature_name=RP11-731F5.2	RP11-731F5.2.value <= threshold=2.830894112586975
node_3778: feature_name=CASP8	CASP8.value <= threshold=2.222941279411316
node_3779: feature_name=MT-CO3	MT-CO3.value > threshold=-0.41117167472839355
node_3785: feature_name=HLA-DRB5	HLA-DRB5.value > threshold=0.8465962707996368
node_3793: feature_name=PDLIM1	PDLIM1.value > threshold=2.90118670463562
node_3813: feature_name=IRF8	IRF8.value <= threshold=-0.34634101390838623
Class: COVID-19 Severe	

Rules_958	passed counts:4
node_0: feature_name=LTB	LTB.value <= threshold=0.994031548500061
node_1: feature_name=MTRNR2L2	MTRNR2L2.value <= threshold=2.8784297704696655
node_2: feature_name=TCL1A	TCL1A.value <= threshold=4.798312664031982
node_3: feature_name=MT-CO1	MT-CO1.value > threshold=0.08947191759943962
node_2521: feature_name=LTB	LTB.value <= threshold=0.6038667261600494
node_2522: feature_name=HLA-DQB1	HLA-DQB1.value > threshold=2.1053054332733154
node_3550: feature_name=XIST	XIST.value <= threshold=0.7716337237507105
node_3551: feature_name=CD79B	CD79B.value <= threshold=2.9426796436309814
node_3552: feature_name=HLA-DQB1	HLA-DQB1.value <= threshold=2.713674306869507
node_3553: feature_name=SNX9	SNX9.value > threshold=-0.5404067635536194
node_3653: feature_name=LTB	LTB.value > threshold=0.021708179265260696
node_3759: feature_name=TCL1A	TCL1A.value <= threshold=3.3921085596084595
node_3760: feature_name=HLA-DRB5	HLA-DRB5.value > threshold=0.5265218019485474
node_3776: feature_name=ID1	ID1.value <= threshold=-0.21235637366771698
node_3777: feature_name=RP11-731F5.2	RP11-731F5.2.value <= threshold=2.830894112586975
node_3778: feature_name=CASP8	CASP8.value <= threshold=2.222941279411316
node_3779: feature_name=MT-CO3	MT-CO3.value > threshold=-0.41117167472839355
node_3785: feature_name=HLA-DRB5	HLA-DRB5.value > threshold=0.8465962707996368
node_3793: feature_name=PDLIM1	PDLIM1.value <= threshold=2.90118670463562
node_3794: feature_name=WDR74	WDR74.value <= threshold=3.335801362991333
node_3795: feature_name=MEF2C	MEF2C.value <= threshold=4.397150039672852
node_3796: feature_name=HLA-DPA1	HLA-DPA1.value > threshold=-0.10480117797851562
node_3798: feature_name=FCER2	FCER2.value > threshold=5.688235521316528
node_3808: feature_name=TLN1	TLN1.value > threshold=-0.6995839774608612
Class: COVID-19 Critical	
Rules_959	passed counts:4
node_0: feature_name=LTB	LTB.value <= threshold=0.994031548500061

node_1: feature_name=MTRNR2L2	MTRNR2L2.value <= threshold=2.8784297704696655
node_2: feature_name=TCL1A	TCL1A.value <= threshold=4.798312664031982
node_3: feature_name=MT-CO1	MT-CO1.value > threshold=0.08947191759943962
node_2521: feature_name=LTB	LTB.value <= threshold=0.6038667261600494
node_2522: feature_name=HLA-DQB1	HLA-DQB1.value > threshold=2.1053054332733154
node_3550: feature_name=XIST	XIST.value <= threshold=0.7716337237507105
node_3551: feature_name=CD79B	CD79B.value <= threshold=2.9426796436309814
node_3552: feature_name=HLA-DQB1	HLA-DQB1.value <= threshold=2.713674306869507
node_3553: feature_name=SNX9	SNX9.value > threshold=-0.5404067635536194
node_3653: feature_name=LTB	LTB.value <= threshold=0.021708179265260696
node_3654: feature_name=HLA-DRB5	HLA-DRB5.value > threshold=-0.5633223652839661
node_3660: feature_name=NR4A1	NR4A1.value <= threshold=2.2327845096588135
node_3661: feature_name=FCRL1	FCRL1.value <= threshold=8.662389755249023
node_3662: feature_name=BANK1	BANK1.value > threshold=4.776419401168823
node_3726: feature_name=MT-CO1	MT-CO1.value > threshold=1.4696794748306274
Class: Healthy Control	
Rules_960	passed counts:4
node_0: feature_name=LTB	LTB.value <= threshold=0.994031548500061
node_1: feature_name=MTRNR2L2	MTRNR2L2.value <= threshold=2.8784297704696655
node_2: feature_name=TCL1A	TCL1A.value <= threshold=4.798312664031982
node_3: feature_name=MT-CO1	MT-CO1.value > threshold=0.08947191759943962
node_2521: feature_name=LTB	LTB.value <= threshold=0.6038667261600494
node_2522: feature_name=HLA-DQB1	HLA-DQB1.value > threshold=2.1053054332733154
node_3550: feature_name=XIST	XIST.value <= threshold=0.7716337237507105
node_3551: feature_name=CD79B	CD79B.value <= threshold=2.9426796436309814
node_3552: feature_name=HLA-DQB1	HLA-DQB1.value <= threshold=2.713674306869507
node_3553: feature_name=SNX9	SNX9.value > threshold=-0.5404067635536194
node_3653: feature_name=LTB	LTB.value <= threshold=0.021708179265260696
node_3654: feature_name=HLA-DRB5	HLA-DRB5.value > threshold=-0.5633223652839661

node_3660: feature_name=NR4A1	NR4A1.value <= threshold=2.2327845096588135
node_3661: feature_name=FCRL1	FCRL1.value <= threshold=8.662389755249023
node_3662: feature_name=BANK1	BANK1.value <= threshold=4.776419401168823
node_3663: feature_name=IGLL5	IGLL5.value <= threshold=1.4806077480316162
node_3664: feature_name=COTL1	COTL1.value <= threshold=-1.2335613369941711
node_3665: feature_name=PELI1	PELI1.value > threshold=-0.6213432848453522
Class: COVID-19 Critical	
Rules_961	passed counts:4
node_0: feature_name=LTB	LTB.value <= threshold=0.994031548500061
node_1: feature_name=MTRNR2L2	MTRNR2L2.value <= threshold=2.8784297704696655
node_2: feature_name=TCL1A	TCL1A.value <= threshold=4.798312664031982
node_3: feature_name=MT-CO1	MT-CO1.value > threshold=0.08947191759943962
node_2521: feature_name=LTB	LTB.value <= threshold=0.6038667261600494
node_2522: feature_name=HLA-DQB1	HLA-DQB1.value > threshold=2.1053054332733154
node_3550: feature_name=XIST	XIST.value <= threshold=0.7716337237507105
node_3551: feature_name=CD79B	CD79B.value <= threshold=2.9426796436309814
node_3552: feature_name=HLA-DQB1	HLA-DQB1.value <= threshold=2.713674306869507
node_3553: feature_name=SNX9	SNX9.value > threshold=-0.5404067635536194
node_3653: feature_name=LTB	LTB.value <= threshold=0.021708179265260696
node_3654: feature_name=HLA-DRB5	HLA-DRB5.value > threshold=-0.5633223652839661
node_3660: feature_name=NR4A1	NR4A1.value <= threshold=2.2327845096588135
node_3661: feature_name=FCRL1	FCRL1.value <= threshold=8.662389755249023
node_3662: feature_name=BANK1	BANK1.value <= threshold=4.776419401168823
node_3663: feature_name=IGLL5	IGLL5.value <= threshold=1.4806077480316162
node_3664: feature_name=COTL1	COTL1.value <= threshold=-1.2335613369941711
node_3665: feature_name=PELI1	PELI1.value <= threshold=-0.6213432848453522
node_3666: feature_name=SPI1	SPI1.value <= threshold=-0.021152734756469727
Class: COVID-19 Severe	

Rules_962	passed counts:4
node_0: feature_name=LTB	LTB.value <= threshold=0.994031548500061
node_1: feature_name=MTRNR2L2	MTRNR2L2.value <= threshold=2.8784297704696655
node_2: feature_name=TCL1A	TCL1A.value <= threshold=4.798312664031982
node_3: feature_name=MT-CO1	MT-CO1.value > threshold=0.08947191759943962
node_2521: feature_name=LTB	LTB.value <= threshold=0.6038667261600494
node_2522: feature_name=HLA-DQB1	HLA-DQB1.value > threshold=2.1053054332733154
node_3550: feature_name=XIST	XIST.value <= threshold=0.7716337237507105
node_3551: feature_name=CD79B	CD79B.value <= threshold=2.9426796436309814
node_3552: feature_name=HLA-DQB1	HLA-DQB1.value <= threshold=2.713674306869507
node_3553: feature_name=SNX9	SNX9.value <= threshold=-0.5404067635536194
node_3554: feature_name=VPREB3	VPREB3.value <= threshold=4.4475414752960205
node_3555: feature_name=RALGPS2	RALGPS2.value <= threshold=2.7017585039138794
node_3556: feature_name=MEF2C	MEF2C.value <= threshold=3.997420072555542
node_3557: feature_name=NR4A1	NR4A1.value <= threshold=3.9266018867492676
node_3558: feature_name=CD22	CD22.value > threshold=5.738929986953735
node_3600: feature_name=BANK1	BANK1.value <= threshold=2.892276644706726
Class: Healthy Control	
Rules_963	passed counts:4
node_0: feature_name=LTB	LTB.value <= threshold=0.994031548500061
node_1: feature_name=MTRNR2L2	MTRNR2L2.value <= threshold=2.8784297704696655
node_2: feature_name=TCL1A	TCL1A.value <= threshold=4.798312664031982
node_3: feature_name=MT-CO1	MT-CO1.value > threshold=0.08947191759943962
node_2521: feature_name=LTB	LTB.value <= threshold=0.6038667261600494
node_2522: feature_name=HLA-DQB1	HLA-DQB1.value <= threshold=2.1053054332733154
node_2523: feature_name=SNX9	SNX9.value > threshold=-0.5346486568450928
node_2907: feature_name=NR4A1	NR4A1.value > threshold=2.5433837175369263
node_3459: feature_name=VPREB3	VPREB3.value <= threshold=4.410779237747192
node_3460: feature_name=RALGPS2	RALGPS2.value > threshold=3.064661979675293

node_3520: feature_name=RGS2	RGS2.value > threshold=-0.5165578126907349
node_3522: feature_name=MT-ND6	MT-ND6.value <= threshold=1.692318856716156
node_3523: feature_name=ID1	ID1.value <= threshold=-0.16399746388196945
node_3524: feature_name=HBB	HBB.value > threshold=1.082573562860489
Class: COVID-19 Severe	
Rules_964	passed counts:4
node_0: feature_name=LTB	LTB.value <= threshold=0.994031548500061
node_1: feature_name=MTRNR2L2	MTRNR2L2.value <= threshold=2.8784297704696655
node_2: feature_name=TCL1A	TCL1A.value <= threshold=4.798312664031982
node_3: feature_name=MT-CO1	MT-CO1.value > threshold=0.08947191759943962
node_2521: feature_name=LTB	LTB.value <= threshold=0.6038667261600494
node_2522: feature_name=HLA-DQB1	HLA-DQB1.value <= threshold=2.1053054332733154
node_2523: feature_name=SNX9	SNX9.value > threshold=-0.5346486568450928
node_2907: feature_name=NR4A1	NR4A1.value > threshold=2.5433837175369263
node_3459: feature_name=VPREB3	VPREB3.value <= threshold=4.410779237747192
node_3460: feature_name=RALGPS2	RALGPS2.value <= threshold=3.064661979675293
node_3461: feature_name=MT-CO1	MT-CO1.value <= threshold=1.7489010095596313
node_3462: feature_name=HLA-DQA2	HLA-DQA2.value <= threshold=1.794841468334198
node_3463: feature_name=CYBB	CYBB.value <= threshold=1.5984815955162048
node_3464: feature_name=MARCKS	MARCKS.value <= threshold=-0.38392288982868195
node_3465: feature_name=OAZ1	OAZ1.value <= threshold=-0.07741016428917646
node_3466: feature_name=XAF1	XAF1.value > threshold=-0.4615721255540848
Class: Healthy Control	
Rules_965	passed counts:4
node_0: feature_name=LTB	LTB.value <= threshold=0.994031548500061
node_1: feature_name=MTRNR2L2	MTRNR2L2.value <= threshold=2.8784297704696655
node_2: feature_name=TCL1A	TCL1A.value <= threshold=4.798312664031982
node_3: feature_name=MT-CO1	MT-CO1.value > threshold=0.08947191759943962

node_2521: feature_name=LTB	LTB.value <= threshold=0.6038667261600494
node_2522: feature_name=HLA-DQB1	HLA-DQB1.value <= threshold=2.1053054332733154
node_2523: feature_name=SNX9	SNX9.value > threshold=-0.5346486568450928
node_2907: feature_name=NR4A1	NR4A1.value <= threshold=2.5433837175369263
node_2908: feature_name=XIST	XIST.value > threshold=0.7664470989257097
node_3440: feature_name=MT-CO1	MT-CO1.value > threshold=0.9336088299751282
node_3448: feature_name=RALGPS2	RALGPS2.value <= threshold=3.274242401123047
node_3449: feature_name=S100A6	S100A6.value > threshold=-1.0691525042057037
node_3453: feature_name=CD83	CD83.value > threshold=2.5665323734283447
Class: Healthy Control	
Rules_966	passed counts:4
node_0: feature_name=LTB	LTB.value <= threshold=0.994031548500061
node_1: feature_name=MTRNR2L2	MTRNR2L2.value <= threshold=2.8784297704696655
node_2: feature_name=TCL1A	TCL1A.value <= threshold=4.798312664031982
node_3: feature_name=MT-CO1	MT-CO1.value > threshold=0.08947191759943962
node_2521: feature_name=LTB	LTB.value <= threshold=0.6038667261600494
node_2522: feature_name=HLA-DQB1	HLA-DQB1.value <= threshold=2.1053054332733154
node_2523: feature_name=SNX9	SNX9.value > threshold=-0.5346486568450928
node_2907: feature_name=NR4A1	NR4A1.value <= threshold=2.5433837175369263
node_2908: feature_name=XIST	XIST.value > threshold=0.7664470989257097
node_3440: feature_name=MT-CO1	MT-CO1.value <= threshold=0.9336088299751282
node_3441: feature_name=WDR74	WDR74.value > threshold=1.7392029166221619
Class: Healthy Control	
Rules_967	passed counts:4
node_0: feature_name=LTB	LTB.value <= threshold=0.994031548500061
node_1: feature_name=MTRNR2L2	MTRNR2L2.value <= threshold=2.8784297704696655
node_2: feature_name=TCL1A	TCL1A.value <= threshold=4.798312664031982
node_3: feature_name=MT-CO1	MT-CO1.value > threshold=0.08947191759943962

node_2521: feature_name=LTB	LTB.value <= threshold=0.6038667261600494
node_2522: feature_name=HLA-DQB1	HLA-DQB1.value <= threshold=2.1053054332733154
node_2523: feature_name=SNX9	SNX9.value > threshold=-0.5346486568450928
node_2907: feature_name=NR4A1	NR4A1.value <= threshold=2.5433837175369263
node_2908: feature_name=XIST	XIST.value <= threshold=0.7664470989257097
node_2909: feature_name=CD79B	CD79B.value > threshold=2.9326847791671753
node_3353: feature_name=VPREB3	VPREB3.value <= threshold=3.0226117372512817
node_3354: feature_name=CD83	CD83.value > threshold=1.9074636101722717
node_3388: feature_name=BOD1L1	BOD1L1.value > threshold=-0.4742381125688553
node_3404: feature_name=RALGPS2	RALGPS2.value > threshold=4.456665992736816
node_3420: feature_name=BACH1	BACH1.value > threshold=-0.4849775433540344
node_3422: feature_name=WDR74	WDR74.value <= threshold=-0.48890332877635956
Class: COVID-19 Critical	
Rules_968	passed counts:4
node_0: feature_name=LTB	LTB.value <= threshold=0.994031548500061
node_1: feature_name=MTRNR2L2	MTRNR2L2.value <= threshold=2.8784297704696655
node_2: feature_name=TCL1A	TCL1A.value <= threshold=4.798312664031982
node_3: feature_name=MT-CO1	MT-CO1.value > threshold=0.08947191759943962
node_2521: feature_name=LTB	LTB.value <= threshold=0.6038667261600494
node_2522: feature_name=HLA-DQB1	HLA-DQB1.value <= threshold=2.1053054332733154
node_2523: feature_name=SNX9	SNX9.value > threshold=-0.5346486568450928
node_2907: feature_name=NR4A1	NR4A1.value <= threshold=2.5433837175369263
node_2908: feature_name=XIST	XIST.value <= threshold=0.7664470989257097
node_2909: feature_name=CD79B	CD79B.value > threshold=2.9326847791671753
node_3353: feature_name=VPREB3	VPREB3.value <= threshold=3.0226117372512817
node_3354: feature_name=CD83	CD83.value > threshold=1.9074636101722717
node_3388: feature_name=BOD1L1	BOD1L1.value > threshold=-0.4742381125688553
node_3404: feature_name=RALGPS2	RALGPS2.value > threshold=4.456665992736816
node_3420: feature_name=BACH1	BACH1.value <= threshold=-0.4849775433540344

Class: Healthy Control	
Rules_969	passed counts:4
node_0: feature_name=LTB	LTB.value <= threshold=0.994031548500061
node_1: feature_name=MTRNR2L2	MTRNR2L2.value <= threshold=2.8784297704696655
node_2: feature_name=TCL1A	TCL1A.value <= threshold=4.798312664031982
node_3: feature_name=MT-CO1	MT-CO1.value > threshold=0.08947191759943962
node_2521: feature_name=LTB	LTB.value <= threshold=0.6038667261600494
node_2522: feature_name=HLA-DQB1	HLA-DQB1.value <= threshold=2.1053054332733154
node_2523: feature_name=SNX9	SNX9.value > threshold=-0.5346486568450928
node_2907: feature_name=NR4A1	NR4A1.value <= threshold=2.5433837175369263
node_2908: feature_name=XIST	XIST.value <= threshold=0.7664470989257097
node_2909: feature_name=CD79B	CD79B.value <= threshold=2.9326847791671753
node_2910: feature_name=NR4A1	NR4A1.value > threshold=-0.2875557690858841
node_3096: feature_name=NR4A3	NR4A3.value > threshold=3.314968705177307
node_3328: feature_name=MT-CO3	MT-CO3.value > threshold=0.35808728635311127
node_3332: feature_name=STAG3	STAG3.value > threshold=-0.18329568207263947
Class: Healthy Control	
Rules_970	passed counts:4
node_0: feature_name=LTB	LTB.value <= threshold=0.994031548500061
node_1: feature_name=MTRNR2L2	MTRNR2L2.value <= threshold=2.8784297704696655
node_2: feature_name=TCL1A	TCL1A.value <= threshold=4.798312664031982
node_3: feature_name=MT-CO1	MT-CO1.value > threshold=0.08947191759943962
node_2521: feature_name=LTB	LTB.value <= threshold=0.6038667261600494
node_2522: feature_name=HLA-DQB1	HLA-DQB1.value <= threshold=2.1053054332733154
node_2523: feature_name=SNX9	SNX9.value > threshold=-0.5346486568450928
node_2907: feature_name=NR4A1	NR4A1.value <= threshold=2.5433837175369263
node_2908: feature_name=XIST	XIST.value <= threshold=0.7664470989257097
node_2909: feature_name=CD79B	CD79B.value <= threshold=2.9326847791671753

node_2910: feature_name=NR4A1	NR4A1.value > threshold=-0.2875557690858841
node_3096: feature_name=NR4A3	NR4A3.value > threshold=3.314968705177307
node_3328: feature_name=MT-CO3	MT-CO3.value > threshold=0.35808728635311127
node_3332: feature_name=STAG3	STAG3.value <= threshold=-0.18329568207263947
node_3333: feature_name=SLC7A5	SLC7A5.value > threshold=2.876398801803589
node_3345: feature_name=ATF3	ATF3.value <= threshold=-0.1988486647605896
node_3346: feature_name=HSP90B1	HSP90B1.value > threshold=2.246195077896118
Class: COVID-19 Severe	
Rules_971	passed counts:4
node_0: feature_name=LTB	LTB.value <= threshold=0.994031548500061
node_1: feature_name=MTRNR2L2	MTRNR2L2.value <= threshold=2.8784297704696655
node_2: feature_name=TCL1A	TCL1A.value <= threshold=4.798312664031982
node_3: feature_name=MT-CO1	MT-CO1.value > threshold=0.08947191759943962
node_2521: feature_name=LTB	LTB.value <= threshold=0.6038667261600494
node_2522: feature_name=HLA-DQB1	HLA-DQB1.value <= threshold=2.1053054332733154
node_2523: feature_name=SNX9	SNX9.value > threshold=-0.5346486568450928
node_2907: feature_name=NR4A1	NR4A1.value <= threshold=2.5433837175369263
node_2908: feature_name=XIST	XIST.value <= threshold=0.7664470989257097
node_2909: feature_name=CD79B	CD79B.value <= threshold=2.9326847791671753
node_2910: feature_name=NR4A1	NR4A1.value > threshold=-0.2875557690858841
node_3096: feature_name=NR4A3	NR4A3.value > threshold=3.314968705177307
node_3328: feature_name=MT-CO3	MT-CO3.value > threshold=0.35808728635311127
node_3332: feature_name=STAG3	STAG3.value <= threshold=-0.18329568207263947
node_3333: feature_name=SLC7A5	SLC7A5.value <= threshold=2.876398801803589
node_3334: feature_name=SNX29P2	SNX29P2.value <= threshold=5.85232138633728
node_3335: feature_name=GPX1	GPX1.value > threshold=0.11650833487510681
node_3337: feature_name=ADAM19	ADAM19.value <= threshold=-0.3311994820833206
node_3338: feature_name=MAP3K2	MAP3K2.value <= threshold=-0.45020386576652527
Class: COVID-19 Critical	

Rules_972	passed counts:4
node_0: feature_name=LTB	LTB.value <= threshold=0.994031548500061
node_1: feature_name=MTRNR2L2	MTRNR2L2.value <= threshold=2.8784297704696655
node_2: feature_name=TCL1A	TCL1A.value <= threshold=4.798312664031982
node_3: feature_name=MT-CO1	MT-CO1.value > threshold=0.08947191759943962
node_2521: feature_name=LTB	LTB.value <= threshold=0.6038667261600494
node_2522: feature_name=HLA-DQB1	HLA-DQB1.value <= threshold=2.1053054332733154
node_2523: feature_name=SNX9	SNX9.value > threshold=-0.5346486568450928
node_2907: feature_name=NR4A1	NR4A1.value <= threshold=2.5433837175369263
node_2908: feature_name=XIST	XIST.value <= threshold=0.7664470989257097
node_2909: feature_name=CD79B	CD79B.value <= threshold=2.9326847791671753
node_2910: feature_name=NR4A1	NR4A1.value > threshold=-0.2875557690858841
node_3096: feature_name=NR4A3	NR4A3.value <= threshold=3.314968705177307
node_3097: feature_name=VPREB3	VPREB3.value <= threshold=3.3549938201904297
node_3098: feature_name=RALGPS2	RALGPS2.value > threshold=5.807050704956055
Class: Healthy Control	
Rules_973	passed counts:4
node_0: feature_name=LTB	LTB.value <= threshold=0.994031548500061
node_1: feature_name=MTRNR2L2	MTRNR2L2.value <= threshold=2.8784297704696655
node_2: feature_name=TCL1A	TCL1A.value <= threshold=4.798312664031982
node_3: feature_name=MT-CO1	MT-CO1.value > threshold=0.08947191759943962
node_2521: feature_name=LTB	LTB.value <= threshold=0.6038667261600494
node_2522: feature_name=HLA-DQB1	HLA-DQB1.value <= threshold=2.1053054332733154
node_2523: feature_name=SNX9	SNX9.value > threshold=-0.5346486568450928
node_2907: feature_name=NR4A1	NR4A1.value <= threshold=2.5433837175369263
node_2908: feature_name=XIST	XIST.value <= threshold=0.7664470989257097
node_2909: feature_name=CD79B	CD79B.value <= threshold=2.9326847791671753
node_2910: feature_name=NR4A1	NR4A1.value > threshold=-0.2875557690858841

node_3096: feature_name=NR4A3	NR4A3.value <= threshold=3.314968705177307
node_3097: feature_name=VPREB3	VPREB3.value <= threshold=3.3549938201904297
node_3098: feature_name=RALGPS2	RALGPS2.value <= threshold=5.807050704956055
node_3099: feature_name=CTD-2035E11.3	CTD-2035E11.3.value <= threshold=6.4090416431427
node_3100: feature_name=FAM129C	FAM129C.value > threshold=6.966361045837402
node_3272: feature_name=MT-CYB	MT-CYB.value > threshold=1.1738299131393433
Class: Healthy Control	
Rules_974	passed counts:4
node_0: feature_name=LTB	LTB.value <= threshold=0.994031548500061
node_1: feature_name=MTRNR2L2	MTRNR2L2.value <= threshold=2.8784297704696655
node_2: feature_name=TCL1A	TCL1A.value <= threshold=4.798312664031982
node_3: feature_name=MT-CO1	MT-CO1.value > threshold=0.08947191759943962
node_2521: feature_name=LTB	LTB.value <= threshold=0.6038667261600494
node_2522: feature_name=HLA-DQB1	HLA-DQB1.value <= threshold=2.1053054332733154
node_2523: feature_name=SNX9	SNX9.value > threshold=-0.5346486568450928
node_2907: feature_name=NR4A1	NR4A1.value <= threshold=2.5433837175369263
node_2908: feature_name=XIST	XIST.value <= threshold=0.7664470989257097
node_2909: feature_name=CD79B	CD79B.value <= threshold=2.9326847791671753
node_2910: feature_name=NR4A1	NR4A1.value > threshold=-0.2875557690858841
node_3096: feature_name=NR4A3	NR4A3.value <= threshold=3.314968705177307
node_3097: feature_name=VPREB3	VPREB3.value <= threshold=3.3549938201904297
node_3098: feature_name=RALGPS2	RALGPS2.value <= threshold=5.807050704956055
node_3099: feature_name=CTD-2035E11.3	CTD-2035E11.3.value <= threshold=6.4090416431427
node_3100: feature_name=FAM129C	FAM129C.value <= threshold=6.966361045837402
node_3101: feature_name=HES4	HES4.value <= threshold=4.139557242393494
node_3102: feature_name=CD83	CD83.value > threshold=1.8343153595924377
node_3136: feature_name=PLD4	PLD4.value <= threshold=4.453821897506714
node_3137: feature_name=HLA-DQA2	HLA-DQA2.value <= threshold=-0.10758286714553833
node_3138: feature_name=STAG3	STAG3.value <= threshold=7.365770578384399

node_3139: feature_name=LDLR	LDLR.value <= threshold=2.3423796892166138
node_3140: feature_name=CD79A	CD79A.value <= threshold=4.038331866264343
node_3141: feature_name=DUSP4	DUSP4.value <= threshold=4.829863548278809
node_3142: feature_name=OAZ1	OAZ1.value <= threshold=-0.34704728424549103
node_3143: feature_name=CSF3R	CSF3R.value <= threshold=1.0170481204986572
node_3144: feature_name=IGLL5	IGLL5.value <= threshold=6.979742765426636
node_3145: feature_name=ANO6	ANO6.value <= threshold=4.378111124038696
node_3146: feature_name=NAMPT	NAMPT.value <= threshold=3.490760326385498
node_3147: feature_name=RP11-731F5.2	RP11-731F5.2.value <= threshold=8.205055236816406
node_3148: feature_name=COTL1	COTL1.value <= threshold=-1.5100116729736328
node_3149: feature_name=AHR	AHR.value <= threshold=-0.5316690504550934
Class: COVID-19 Critical	
Rules_975	passed counts:4
node_0: feature_name=LTB	LTB.value <= threshold=0.994031548500061
node_1: feature_name=MTRNR2L2	MTRNR2L2.value <= threshold=2.8784297704696655
node_2: feature_name=TCL1A	TCL1A.value <= threshold=4.798312664031982
node_3: feature_name=MT-CO1	MT-CO1.value > threshold=0.08947191759943962
node_2521: feature_name=LTB	LTB.value <= threshold=0.6038667261600494
node_2522: feature_name=HLA-DQB1	HLA-DQB1.value <= threshold=2.1053054332733154
node_2523: feature_name=SNX9	SNX9.value > threshold=-0.5346486568450928
node_2907: feature_name=NR4A1	NR4A1.value <= threshold=2.5433837175369263
node_2908: feature_name=XIST	XIST.value <= threshold=0.7664470989257097
node_2909: feature_name=CD79B	CD79B.value <= threshold=2.9326847791671753
node_2910: feature_name=NR4A1	NR4A1.value <= threshold=-0.2875557690858841
node_2911: feature_name=CTD-2035E11.3	CTD-2035E11.3.value <= threshold=5.262048006057739
node_2912: feature_name=VPREB3	VPREB3.value <= threshold=4.937594890594482
node_2913: feature_name=IRAK3	IRAK3.value <= threshold=4.592883586883545
node_2914: feature_name=IGJ	IGJ.value <= threshold=7.093410015106201
node_2915: feature_name=WDR74	WDR74.value <= threshold=4.077916383743286

node_2916: feature_name=MEF2C	MEF2C.value <= threshold=4.374382972717285
node_2917: feature_name=HIST1H2AC	HIST1H2AC.value > threshold=3.743621349334717
node_3055: feature_name=ITGAX	ITGAX.value > threshold=-0.2822466939687729
Class: COVID-19 Critical	
Rules_976	passed counts:4
node_0: feature_name=LTB	LTB.value <= threshold=0.994031548500061
node_1: feature_name=MTRNR2L2	MTRNR2L2.value <= threshold=2.8784297704696655
node_2: feature_name=TCL1A	TCL1A.value <= threshold=4.798312664031982
node_3: feature_name=MT-CO1	MT-CO1.value > threshold=0.08947191759943962
node_2521: feature_name=LTB	LTB.value <= threshold=0.6038667261600494
node_2522: feature_name=HLA-DQB1	HLA-DQB1.value <= threshold=2.1053054332733154
node_2523: feature_name=SNX9	SNX9.value <= threshold=-0.5346486568450928
node_2524: feature_name=HLA-DPB1	HLA-DPB1.value > threshold=1.9548270106315613
node_2814: feature_name=CD79B	CD79B.value > threshold=-0.28186772763729095
node_2826: feature_name=MT-ND6	MT-ND6.value > threshold=0.17273088544607162
node_2872: feature_name=IFI44L	IFI44L.value <= threshold=-0.31519079208374023
node_2873: feature_name=RALGPS2	RALGPS2.value > threshold=1.6798744797706604
node_2887: feature_name=HLA-DQA2	HLA-DQA2.value > threshold=5.12495756149292
Class: COVID-19 Critical	
Rules_977	passed counts:4
node_0: feature_name=LTB	LTB.value <= threshold=0.994031548500061
node_1: feature_name=MTRNR2L2	MTRNR2L2.value <= threshold=2.8784297704696655
node_2: feature_name=TCL1A	TCL1A.value <= threshold=4.798312664031982
node_3: feature_name=MT-CO1	MT-CO1.value > threshold=0.08947191759943962
node_2521: feature_name=LTB	LTB.value <= threshold=0.6038667261600494
node_2522: feature_name=HLA-DQB1	HLA-DQB1.value <= threshold=2.1053054332733154
node_2523: feature_name=SNX9	SNX9.value <= threshold=-0.5346486568450928
node_2524: feature_name=HLA-DPB1	HLA-DPB1.value > threshold=1.9548270106315613

node_2814: feature_name=CD79B	CD79B.value > threshold=-0.28186772763729095
node_2826: feature_name=MT-ND6	MT-ND6.value <= threshold=0.17273088544607162
node_2827: feature_name=NR4A1	NR4A1.value > threshold=-0.24634335190057755
node_2849: feature_name=FAM129C	FAM129C.value <= threshold=3.089340925216675
node_2850: feature_name=COTL1	COTL1.value > threshold=-0.5968812704086304
node_2856: feature_name=DENND3	DENND3.value <= threshold=-0.3556941896677017
node_2857: feature_name=ISG15	ISG15.value > threshold=-0.5639307200908661
Class: COVID-19 Critical	
Rules_978	passed counts:4
node_0: feature_name=LTB	LTB.value <= threshold=0.994031548500061
node_1: feature_name=MTRNR2L2	MTRNR2L2.value <= threshold=2.8784297704696655
node_2: feature_name=TCL1A	TCL1A.value <= threshold=4.798312664031982
node_3: feature_name=MT-CO1	MT-CO1.value > threshold=0.08947191759943962
node_2521: feature_name=LTB	LTB.value <= threshold=0.6038667261600494
node_2522: feature_name=HLA-DQB1	HLA-DQB1.value <= threshold=2.1053054332733154
node_2523: feature_name=SNX9	SNX9.value <= threshold=-0.5346486568450928
node_2524: feature_name=HLA-DPB1	HLA-DPB1.value <= threshold=1.9548270106315613
node_2525: feature_name=FAM129C	FAM129C.value > threshold=3.3393388986587524
node_2721: feature_name=ADAM19	ADAM19.value <= threshold=-0.29525794088840485
node_2722: feature_name=NR4A1	NR4A1.value <= threshold=2.1566513776779175
node_2723: feature_name=CD22	CD22.value <= threshold=8.345317840576172
node_2724: feature_name=PLD4	PLD4.value <= threshold=7.507996082305908
node_2725: feature_name=RALGPS2	RALGPS2.value > threshold=4.3808369636535645
node_2751: feature_name=NFKBID	NFKBID.value > threshold=-0.4283874034881592
node_2757: feature_name=MT-CO1	MT-CO1.value > threshold=1.7044780254364014
node_2765: feature_name=NFKBIZ	NFKBIZ.value <= threshold=0.5047688484191895
Class: Healthy Control	
Rules_979	passed counts:4

node_0: feature_name=LTB	LTB.value <= threshold=0.994031548500061
node_1: feature_name=MTRNR2L2	MTRNR2L2.value <= threshold=2.8784297704696655
node_2: feature_name=TCL1A	TCL1A.value <= threshold=4.798312664031982
node_3: feature_name=MT-CO1	MT-CO1.value > threshold=0.08947191759943962
node_2521: feature_name=LTB	LTB.value <= threshold=0.6038667261600494
node_2522: feature_name=HLA-DQB1	HLA-DQB1.value <= threshold=2.1053054332733154
node_2523: feature_name=SNX9	SNX9.value <= threshold=-0.5346486568450928
node_2524: feature_name=HLA-DPB1	HLA-DPB1.value <= threshold=1.9548270106315613
node_2525: feature_name=FAM129C	FAM129C.value > threshold=3.3393388986587524
node_2721: feature_name=ADAM19	ADAM19.value <= threshold=-0.29525794088840485
node_2722: feature_name=NR4A1	NR4A1.value <= threshold=2.1566513776779175
node_2723: feature_name=CD22	CD22.value <= threshold=8.345317840576172
node_2724: feature_name=PLD4	PLD4.value <= threshold=7.507996082305908
node_2725: feature_name=RALGPS2	RALGPS2.value <= threshold=4.3808369636535645
node_2726: feature_name=VPREB3	VPREB3.value <= threshold=4.1944358348846436
node_2727: feature_name=LDLRAD4	LDLRAD4.value > threshold=3.293746590614319
node_2741: feature_name=NFKBID	NFKBID.value <= threshold=1.385556623339653
node_2742: feature_name=HLA-DPA1	HLA-DPA1.value > threshold=-0.5997270047664642
Class: Healthy Control	
Rules_980	passed counts:4
node_0: feature_name=LTB	LTB.value <= threshold=0.994031548500061
node_1: feature_name=MTRNR2L2	MTRNR2L2.value <= threshold=2.8784297704696655
node_2: feature_name=TCL1A	TCL1A.value <= threshold=4.798312664031982
node_3: feature_name=MT-CO1	MT-CO1.value > threshold=0.08947191759943962
node_2521: feature_name=LTB	LTB.value <= threshold=0.6038667261600494
node_2522: feature_name=HLA-DQB1	HLA-DQB1.value <= threshold=2.1053054332733154
node_2523: feature_name=SNX9	SNX9.value <= threshold=-0.5346486568450928
node_2524: feature_name=HLA-DPB1	HLA-DPB1.value <= threshold=1.9548270106315613
node_2525: feature_name=FAM129C	FAM129C.value <= threshold=3.3393388986587524

node_2526: feature_name=XIST	XIST.value <= threshold=3.6911416053771973
node_2527: feature_name=NR4A1	NR4A1.value > threshold=-0.2557755410671234
node_2625: feature_name=HLA-DPA1	HLA-DPA1.value > threshold=0.9335225224494934
node_2657: feature_name=RALGPS2	RALGPS2.value > threshold=2.9413715600967407
node_2701: feature_name=DUSP4	DUSP4.value <= threshold=-0.24480421096086502
node_2702: feature_name=MS4A1	MS4A1.value <= threshold=1.7336164116859436
Class: COVID-19 Critical	
Rules_981	passed counts:4
node_0: feature_name=LTB	LTB.value <= threshold=0.994031548500061
node_1: feature_name=MTRNR2L2	MTRNR2L2.value <= threshold=2.8784297704696655
node_2: feature_name=TCL1A	TCL1A.value <= threshold=4.798312664031982
node_3: feature_name=MT-CO1	MT-CO1.value > threshold=0.08947191759943962
node_2521: feature_name=LTB	LTB.value <= threshold=0.6038667261600494
node_2522: feature_name=HLA-DQB1	HLA-DQB1.value <= threshold=2.1053054332733154
node_2523: feature_name=SNX9	SNX9.value <= threshold=-0.5346486568450928
node_2524: feature_name=HLA-DPB1	HLA-DPB1.value <= threshold=1.9548270106315613
node_2525: feature_name=FAM129C	FAM129C.value <= threshold=3.3393388986587524
node_2526: feature_name=XIST	XIST.value <= threshold=3.6911416053771973
node_2527: feature_name=NR4A1	NR4A1.value > threshold=-0.2557755410671234
node_2625: feature_name=HLA-DPA1	HLA-DPA1.value > threshold=0.9335225224494934
node_2657: feature_name=RALGPS2	RALGPS2.value <= threshold=2.9413715600967407
node_2658: feature_name=HLA-DQB1	HLA-DQB1.value <= threshold=1.7503233551979065
node_2659: feature_name=TP53INP1	TP53INP1.value <= threshold=-0.2649386376142502
node_2660: feature_name=OAZ1	OAZ1.value > threshold=1.2488974928855896
Class: Healthy Control	
Rules_982	passed counts:4
node_0: feature_name=LTB	LTB.value <= threshold=0.994031548500061
node_1: feature_name=MTRNR2L2	MTRNR2L2.value <= threshold=2.8784297704696655

node_2: feature_name=TCL1A	TCL1A.value <= threshold=4.798312664031982
node_3: feature_name=MT-CO1	MT-CO1.value > threshold=0.08947191759943962
node_2521: feature_name=LTB	LTB.value <= threshold=0.6038667261600494
node_2522: feature_name=HLA-DQB1	HLA-DQB1.value <= threshold=2.1053054332733154
node_2523: feature_name=SNX9	SNX9.value <= threshold=-0.5346486568450928
node_2524: feature_name=HLA-DPB1	HLA-DPB1.value <= threshold=1.9548270106315613
node_2525: feature_name=FAM129C	FAM129C.value <= threshold=3.3393388986587524
node_2526: feature_name=XIST	XIST.value <= threshold=3.6911416053771973
node_2527: feature_name=NR4A1	NR4A1.value > threshold=-0.2557755410671234
node_2625: feature_name=HLA-DPA1	HLA-DPA1.value <= threshold=0.9335225224494934
node_2626: feature_name=CD83	CD83.value > threshold=-0.585537850856781
node_2628: feature_name=PDLIM1	PDLIM1.value <= threshold=5.292127370834351
node_2629: feature_name=ISG15	ISG15.value <= threshold=3.357967495918274
node_2630: feature_name=BACH1	BACH1.value <= threshold=4.870309114456177
node_2631: feature_name=LDLRAD4	LDLRAD4.value > threshold=-0.2641522139310837
node_2641: feature_name=CD79B	CD79B.value <= threshold=-0.23006416112184525
node_2642: feature_name=IGJ	IGJ.value > threshold=-0.2551199793815613
Class: Healthy Control	
Rules_983	passed counts:4
node_0: feature_name=LTB	LTB.value <= threshold=0.994031548500061
node_1: feature_name=MTRNR2L2	MTRNR2L2.value <= threshold=2.8784297704696655
node_2: feature_name=TCL1A	TCL1A.value <= threshold=4.798312664031982
node_3: feature_name=MT-CO1	MT-CO1.value > threshold=0.08947191759943962
node_2521: feature_name=LTB	LTB.value <= threshold=0.6038667261600494
node_2522: feature_name=HLA-DQB1	HLA-DQB1.value <= threshold=2.1053054332733154
node_2523: feature_name=SNX9	SNX9.value <= threshold=-0.5346486568450928
node_2524: feature_name=HLA-DPB1	HLA-DPB1.value <= threshold=1.9548270106315613
node_2525: feature_name=FAM129C	FAM129C.value <= threshold=3.3393388986587524
node_2526: feature_name=XIST	XIST.value <= threshold=3.6911416053771973

node_2527: feature_name=NR4A1	NR4A1.value > threshold=-0.2557755410671234
node_2625: feature_name=HLA-DPA1	HLA-DPA1.value <= threshold=0.9335225224494934
node_2626: feature_name=CD83	CD83.value > threshold=-0.585537850856781
node_2628: feature_name=PDLIM1	PDLIM1.value <= threshold=5.292127370834351
node_2629: feature_name=ISG15	ISG15.value <= threshold=3.357967495918274
node_2630: feature_name=BACH1	BACH1.value <= threshold=4.870309114456177
node_2631: feature_name=LDLRAD4	LDLRAD4.value > threshold=-0.2641522139310837
node_2641: feature_name=CD79B	CD79B.value <= threshold=-0.23006416112184525
node_2642: feature_name=IGJ	IGJ.value <= threshold=-0.2551199793815613
node_2643: feature_name=HLA-DQA1	HLA-DQA1.value > threshold=0.15228520333766937
Class: COVID-19 Critical	
Rules_984	passed counts:4
node_0: feature_name=LTB	LTB.value <= threshold=0.994031548500061
node_1: feature_name=MTRNR2L2	MTRNR2L2.value <= threshold=2.8784297704696655
node_2: feature_name=TCL1A	TCL1A.value <= threshold=4.798312664031982
node_3: feature_name=MT-CO1	MT-CO1.value > threshold=0.08947191759943962
node_2521: feature_name=LTB	LTB.value <= threshold=0.6038667261600494
node_2522: feature_name=HLA-DQB1	HLA-DQB1.value <= threshold=2.1053054332733154
node_2523: feature_name=SNX9	SNX9.value <= threshold=-0.5346486568450928
node_2524: feature_name=HLA-DPB1	HLA-DPB1.value <= threshold=1.9548270106315613
node_2525: feature_name=FAM129C	FAM129C.value <= threshold=3.3393388986587524
node_2526: feature_name=XIST	XIST.value <= threshold=3.6911416053771973
node_2527: feature_name=NR4A1	NR4A1.value <= threshold=-0.2557755410671234
node_2528: feature_name=HOPX	HOPX.value > threshold=2.776473879814148
node_2622: feature_name=PPIF	PPIF.value <= threshold=0.7416565716266632
Class: Healthy Control	
Rules_985	passed counts:4
node_0: feature_name=LTB	LTB.value <= threshold=0.994031548500061

node_1: feature_name=MTRNR2L2	MTRNR2L2.value <= threshold=2.8784297704696655
node_2: feature_name=TCL1A	TCL1A.value <= threshold=4.798312664031982
node_3: feature_name=MT-CO1	MT-CO1.value > threshold=0.08947191759943962
node_2521: feature_name=LTB	LTB.value <= threshold=0.6038667261600494
node_2522: feature_name=HLA-DQB1	HLA-DQB1.value <= threshold=2.1053054332733154
node_2523: feature_name=SNX9	SNX9.value <= threshold=-0.5346486568450928
node_2524: feature_name=HLA-DPB1	HLA-DPB1.value <= threshold=1.9548270106315613
node_2525: feature_name=FAM129C	FAM129C.value <= threshold=3.3393388986587524
node_2526: feature_name=XIST	XIST.value <= threshold=3.6911416053771973
node_2527: feature_name=NR4A1	NR4A1.value <= threshold=-0.2557755410671234
node_2528: feature_name=HOPX	HOPX.value <= threshold=2.776473879814148
node_2529: feature_name=AREGB	AREGB.value <= threshold=-0.10896047577261925
node_2530: feature_name=KIAA0125	KIAA0125.value <= threshold=7.652706623077393
node_2531: feature_name=MT-CO3	MT-CO3.value > threshold=-1.3726763725280762
node_2533: feature_name=TNF	TNF.value <= threshold=3.1101887226104736
node_2534: feature_name=NFKBID	NFKBID.value <= threshold=-0.39706873893737793
node_2535: feature_name=KIAA0125	KIAA0125.value > threshold=-0.17713353037834167
node_2543: feature_name=XAF1	XAF1.value > threshold=1.7887146472930908
node_2563: feature_name=HLA-DPA1	HLA-DPA1.value <= threshold=0.6970475614070892
Class: COVID-19 Critical	
Rules_986	passed counts:4
node_0: feature_name=LTB	LTB.value <= threshold=0.994031548500061
node_1: feature_name=MTRNR2L2	MTRNR2L2.value <= threshold=2.8784297704696655
node_2: feature_name=TCL1A	TCL1A.value <= threshold=4.798312664031982
node_3: feature_name=MT-CO1	MT-CO1.value > threshold=0.08947191759943962
node_2521: feature_name=LTB	LTB.value <= threshold=0.6038667261600494
node_2522: feature_name=HLA-DQB1	HLA-DQB1.value <= threshold=2.1053054332733154
node_2523: feature_name=SNX9	SNX9.value <= threshold=-0.5346486568450928
node_2524: feature_name=HLA-DPB1	HLA-DPB1.value <= threshold=1.9548270106315613

node_2525: feature_name=FAM129C	FAM129C.value <= threshold=3.3393388986587524
node_2526: feature_name=XIST	XIST.value <= threshold=3.6911416053771973
node_2527: feature_name=NR4A1	NR4A1.value <= threshold=-0.2557755410671234
node_2528: feature_name=HOPX	HOPX.value <= threshold=2.776473879814148
node_2529: feature_name=AREGB	AREGB.value <= threshold=-0.10896047577261925
node_2530: feature_name=KIAA0125	KIAA0125.value <= threshold=7.652706623077393
node_2531: feature_name=MT-CO3	MT-CO3.value > threshold=-1.3726763725280762
node_2533: feature_name=TNF	TNF.value <= threshold=3.1101887226104736
node_2534: feature_name=NFKBID	NFKBID.value <= threshold=-0.39706873893737793
node_2535: feature_name=KIAA0125	KIAA0125.value <= threshold=-0.17713353037834167
node_2536: feature_name=MT-CYB	MT-CYB.value <= threshold=-0.0009266361594200134
Class: Healthy Control	
Rules_987	passed counts:4
node_0: feature_name=LTB	LTB.value <= threshold=0.994031548500061
node_1: feature_name=MTRNR2L2	MTRNR2L2.value <= threshold=2.8784297704696655
node_2: feature_name=TCL1A	TCL1A.value <= threshold=4.798312664031982
node_3: feature_name=MT-CO1	MT-CO1.value <= threshold=0.08947191759943962
node_4: feature_name=NR4A1	NR4A1.value > threshold=1.0725348591804504
node_1622: feature_name=HLA-DQA2	HLA-DQA2.value > threshold=1.9496816992759705
node_2350: feature_name=BCL2A1	BCL2A1.value <= threshold=1.1322240233421326
node_2351: feature_name=CD83	CD83.value > threshold=2.3162450790405273
node_2391: feature_name=CD79B	CD79B.value > threshold=1.7111427783966064
node_2443: feature_name=MT-CO1	MT-CO1.value > threshold=-1.1614157557487488
node_2447: feature_name=JUN	JUN.value <= threshold=0.19632036238908768
node_2448: feature_name=PLD4	PLD4.value <= threshold=-0.11274048686027527
node_2449: feature_name=RNF11	RNF11.value <= threshold=-0.5163591802120209
Class: COVID-19 Critical	
Rules_988	passed counts:4

node_0: feature_name=LTB	LTB.value <= threshold=0.994031548500061
node_1: feature_name=MTRNR2L2	MTRNR2L2.value <= threshold=2.8784297704696655
node_2: feature_name=TCL1A	TCL1A.value <= threshold=4.798312664031982
node_3: feature_name=MT-CO1	MT-CO1.value <= threshold=0.08947191759943962
node_4: feature_name=NR4A1	NR4A1.value > threshold=1.0725348591804504
node_1622: feature_name=HLA-DQA2	HLA-DQA2.value > threshold=1.9496816992759705
node_2350: feature_name=BCL2A1	BCL2A1.value <= threshold=1.1322240233421326
node_2351: feature_name=CD83	CD83.value > threshold=2.3162450790405273
node_2391: feature_name=CD79B	CD79B.value <= threshold=1.7111427783966064
node_2392: feature_name=TTN	TTN.value <= threshold=-0.28553803265094757
node_2393: feature_name=LDLRAD4	LDLRAD4.value <= threshold=3.3328956365585327
node_2394: feature_name=BCL2A1	BCL2A1.value <= threshold=-0.5811292827129364
node_2395: feature_name=HLA-DQB1	HLA-DQB1.value <= threshold=1.635683536529541
node_2396: feature_name=S100A6	S100A6.value > threshold=-0.984266608953476
node_2398: feature_name=JUN	JUN.value > threshold=-0.3675929445307702
Class: COVID-19 Critical	
Rules_989	passed counts:4
node_0: feature_name=LTB	LTB.value <= threshold=0.994031548500061
node_1: feature_name=MTRNR2L2	MTRNR2L2.value <= threshold=2.8784297704696655
node_2: feature_name=TCL1A	TCL1A.value <= threshold=4.798312664031982
node_3: feature_name=MT-CO1	MT-CO1.value <= threshold=0.08947191759943962
node_4: feature_name=NR4A1	NR4A1.value > threshold=1.0725348591804504
node_1622: feature_name=HLA-DQA2	HLA-DQA2.value > threshold=1.9496816992759705
node_2350: feature_name=BCL2A1	BCL2A1.value <= threshold=1.1322240233421326
node_2351: feature_name=CD83	CD83.value <= threshold=2.3162450790405273
node_2352: feature_name=LTB	LTB.value <= threshold=0.7341383993625641
node_2353: feature_name=TCL1A	TCL1A.value > threshold=3.942829728126526
node_2377: feature_name=HLA-DQB1	HLA-DQB1.value <= threshold=1.957283079624176
Class: Healthy Control	

Rules_990	passed counts:4
node_0: feature_name=LTB	LTB.value <= threshold=0.994031548500061
node_1: feature_name=MTRNR2L2	MTRNR2L2.value <= threshold=2.8784297704696655
node_2: feature_name=TCL1A	TCL1A.value <= threshold=4.798312664031982
node_3: feature_name=MT-CO1	MT-CO1.value <= threshold=0.08947191759943962
node_4: feature_name=NR4A1	NR4A1.value > threshold=1.0725348591804504
node_1622: feature_name=HLA-DQA2	HLA-DQA2.value > threshold=1.9496816992759705
node_2350: feature_name=BCL2A1	BCL2A1.value <= threshold=1.1322240233421326
node_2351: feature_name=CD83	CD83.value <= threshold=2.3162450790405273
node_2352: feature_name=LTB	LTB.value <= threshold=0.7341383993625641
node_2353: feature_name=TCL1A	TCL1A.value <= threshold=3.942829728126526
node_2354: feature_name=MARCKS	MARCKS.value > threshold=2.9345329999923706
node_2374: feature_name=TCF4	TCF4.value > threshold=3.1691582202911377
Class: COVID-19 Critical	
Rules_991	passed counts:4
node_0: feature_name=LTB	LTB.value <= threshold=0.994031548500061
node_1: feature_name=MTRNR2L2	MTRNR2L2.value <= threshold=2.8784297704696655
node_2: feature_name=TCL1A	TCL1A.value <= threshold=4.798312664031982
node_3: feature_name=MT-CO1	MT-CO1.value <= threshold=0.08947191759943962
node_4: feature_name=NR4A1	NR4A1.value > threshold=1.0725348591804504
node_1622: feature_name=HLA-DQA2	HLA-DQA2.value <= threshold=1.9496816992759705
node_1623: feature_name=HLA-DRB5	HLA-DRB5.value <= threshold=2.715766429901123
node_1624: feature_name=HLA-DPA1	HLA-DPA1.value > threshold=1.7050301432609558
node_2056: feature_name=OAZ1	OAZ1.value > threshold=0.456103190779686
node_2266: feature_name=XAF1	XAF1.value > threshold=-0.4431648850440979
node_2312: feature_name=ISG15	ISG15.value <= threshold=-0.3511900007724762
node_2313: feature_name=RALGPS2	RALGPS2.value > threshold=2.2695717811584473
node_2329: feature_name=LTB	LTB.value <= threshold=-0.9258447885513306

Class: COVID-19 Critical	
Rules_992	passed counts:4
node_0: feature_name=LTB	LTB.value <= threshold=0.994031548500061
node_1: feature_name=MTRNR2L2	MTRNR2L2.value <= threshold=2.8784297704696655
node_2: feature_name=TCL1A	TCL1A.value <= threshold=4.798312664031982
node_3: feature_name=MT-CO1	MT-CO1.value <= threshold=0.08947191759943962
node_4: feature_name=NR4A1	NR4A1.value > threshold=1.0725348591804504
node_1622: feature_name=HLA-DQA2	HLA-DQA2.value <= threshold=1.9496816992759705
node_1623: feature_name=HLA-DRB5	HLA-DRB5.value <= threshold=2.715766429901123
node_1624: feature_name=HLA-DPA1	HLA-DPA1.value > threshold=1.7050301432609558
node_2056: feature_name=OAZ1	OAZ1.value <= threshold=0.456103190779686
node_2057: feature_name=HLA-DRB5	HLA-DRB5.value > threshold=0.6839984953403473
node_2101: feature_name=HLA-DRB1	HLA-DRB1.value > threshold=1.6199676990509033
node_2151: feature_name=HLA-DQB1	HLA-DQB1.value <= threshold=3.1961567401885986
node_2152: feature_name=PLD4	PLD4.value <= threshold=-0.03259922005236149
node_2153: feature_name=XIST	XIST.value <= threshold=-0.138966154307127
node_2154: feature_name=HLA-DQA1	HLA-DQA1.value > threshold=2.0848299264907837
node_2190: feature_name=RALGPS2	RALGPS2.value <= threshold=4.276920795440674
node_2191: feature_name=RGS2	RGS2.value > threshold=-0.7087429165840149
node_2195: feature_name=GRAP2	GRAP2.value <= threshold=2.2513980865478516
node_2196: feature_name=ID3	ID3.value <= threshold=5.227530002593994
node_2197: feature_name=CD79B	CD79B.value <= threshold=4.226724624633789
node_2198: feature_name=ENDOD1	ENDOD1.value <= threshold=4.877119541168213
node_2199: feature_name=AP3M2	AP3M2.value <= threshold=2.6568219661712646
node_2200: feature_name=ICAM1	ICAM1.value <= threshold=4.384357690811157
node_2201: feature_name=ILK	ILK.value <= threshold=2.051138460636139
node_2202: feature_name=DUSP4	DUSP4.value <= threshold=5.8240966796875
node_2203: feature_name=NR4A1	NR4A1.value > threshold=1.132164180278778
node_2205: feature_name=MEF2C	MEF2C.value <= threshold=4.1522674560546875

node_2206: feature_name=PLA2G12A	PLA2G12A.value <= threshold=1.7114491239190102
node_2207: feature_name=SNX29P2	SNX29P2.value <= threshold=5.11986231803894
node_2208: feature_name=ADAM19	ADAM19.value <= threshold=3.4296538829803467
node_2209: feature_name=PPIF	PPIF.value > threshold=2.486689567565918
node_2211: feature_name=FAM129C	FAM129C.value <= threshold=3.123738408088684
Class: COVID-19 Critical	
Rules_993	passed counts:4
node_0: feature_name=LTB	LTB.value <= threshold=0.994031548500061
node_1: feature_name=MTRNR2L2	MTRNR2L2.value <= threshold=2.8784297704696655
node_2: feature_name=TCL1A	TCL1A.value <= threshold=4.798312664031982
node_3: feature_name=MT-CO1	MT-CO1.value <= threshold=0.08947191759943962
node_4: feature_name=NR4A1	NR4A1.value > threshold=1.0725348591804504
node_1622: feature_name=HLA-DQA2	HLA-DQA2.value <= threshold=1.9496816992759705
node_1623: feature_name=HLA-DRB5	HLA-DRB5.value <= threshold=2.715766429901123
node_1624: feature_name=HLA-DPA1	HLA-DPA1.value > threshold=1.7050301432609558
node_2056: feature_name=OAZ1	OAZ1.value <= threshold=0.456103190779686
node_2057: feature_name=HLA-DRB5	HLA-DRB5.value > threshold=0.6839984953403473
node_2101: feature_name=HLA-DRB1	HLA-DRB1.value > threshold=1.6199676990509033
node_2151: feature_name=HLA-DQB1	HLA-DQB1.value <= threshold=3.1961567401885986
node_2152: feature_name=PLD4	PLD4.value <= threshold=-0.03259922005236149
node_2153: feature_name=XIST	XIST.value <= threshold=-0.138966154307127
node_2154: feature_name=HLA-DQA1	HLA-DQA1.value > threshold=2.0848299264907837
node_2190: feature_name=RALGPS2	RALGPS2.value <= threshold=4.276920795440674
node_2191: feature_name=RGS2	RGS2.value <= threshold=-0.7087429165840149
node_2192: feature_name=MEF2C	MEF2C.value <= threshold=1.1360841393470764
Class: COVID-19 Severe	
Rules_994	passed counts:4
node_0: feature_name=LTB	LTB.value <= threshold=0.994031548500061

node_1: feature_name=MTRNR2L2	MTRNR2L2.value <= threshold=2.8784297704696655
node_2: feature_name=TCL1A	TCL1A.value <= threshold=4.798312664031982
node_3: feature_name=MT-CO1	MT-CO1.value <= threshold=0.08947191759943962
node_4: feature_name=NR4A1	NR4A1.value > threshold=1.0725348591804504
node_1622: feature_name=HLA-DQA2	HLA-DQA2.value <= threshold=1.9496816992759705
node_1623: feature_name=HLA-DRB5	HLA-DRB5.value <= threshold=2.715766429901123
node_1624: feature_name=HLA-DPA1	HLA-DPA1.value > threshold=1.7050301432609558
node_2056: feature_name=OAZ1	OAZ1.value <= threshold=0.456103190779686
node_2057: feature_name=HLA-DRB5	HLA-DRB5.value > threshold=0.6839984953403473
node_2101: feature_name=HLA-DRB1	HLA-DRB1.value > threshold=1.6199676990509033
node_2151: feature_name=HLA-DQB1	HLA-DQB1.value <= threshold=3.1961567401885986
node_2152: feature_name=PLD4	PLD4.value <= threshold=-0.03259922005236149
node_2153: feature_name=XIST	XIST.value <= threshold=-0.138966154307127
node_2154: feature_name=HLA-DQA1	HLA-DQA1.value <= threshold=2.0848299264907837
node_2155: feature_name=LTB	LTB.value > threshold=-0.9993145167827606
node_2161: feature_name=CYBB	CYBB.value <= threshold=1.514441728591919
node_2162: feature_name=CD83	CD83.value > threshold=3.348180890083313
node_2178: feature_name=HLA-DRB1	HLA-DRB1.value > threshold=2.1453733444213867
Class: COVID-19 Critical	
Rules_995	passed counts:4
node_0: feature_name=LTB	LTB.value <= threshold=0.994031548500061
node_1: feature_name=MTRNR2L2	MTRNR2L2.value <= threshold=2.8784297704696655
node_2: feature_name=TCL1A	TCL1A.value <= threshold=4.798312664031982
node_3: feature_name=MT-CO1	MT-CO1.value <= threshold=0.08947191759943962
node_4: feature_name=NR4A1	NR4A1.value > threshold=1.0725348591804504
node_1622: feature_name=HLA-DQA2	HLA-DQA2.value <= threshold=1.9496816992759705
node_1623: feature_name=HLA-DRB5	HLA-DRB5.value <= threshold=2.715766429901123
node_1624: feature_name=HLA-DPA1	HLA-DPA1.value <= threshold=1.7050301432609558
node_1625: feature_name=CD83	CD83.value > threshold=2.783489227294922

node_1873: feature_name=IFI44L	IFI44L.value <= threshold=1.5938722491264343
node_1874: feature_name=PLD4	PLD4.value > threshold=4.101001739501953
node_2028: feature_name=SNX9	SNX9.value <= threshold=1.4460421800613403
node_2029: feature_name=MT-CO3	MT-CO3.value <= threshold=-0.40799812972545624
node_2030: feature_name=PARP14	PARP14.value > threshold=-0.5147344768047333
Class: COVID-19 Critical	
Rules_996	passed counts:4
node_0: feature_name=LTB	LTB.value <= threshold=0.994031548500061
node_1: feature_name=MTRNR2L2	MTRNR2L2.value <= threshold=2.8784297704696655
node_2: feature_name=TCL1A	TCL1A.value <= threshold=4.798312664031982
node_3: feature_name=MT-CO1	MT-CO1.value <= threshold=0.08947191759943962
node_4: feature_name=NR4A1	NR4A1.value > threshold=1.0725348591804504
node_1622: feature_name=HLA-DQA2	HLA-DQA2.value <= threshold=1.9496816992759705
node_1623: feature_name=HLA-DRB5	HLA-DRB5.value <= threshold=2.715766429901123
node_1624: feature_name=HLA-DPA1	HLA-DPA1.value <= threshold=1.7050301432609558
node_1625: feature_name=CD83	CD83.value > threshold=2.783489227294922
node_1873: feature_name=IFI44L	IFI44L.value <= threshold=1.5938722491264343
node_1874: feature_name=PLD4	PLD4.value <= threshold=4.101001739501953
node_1875: feature_name=RHOB	RHOB.value > threshold=1.304835319519043
node_1965: feature_name=HLA-DQB1	HLA-DQB1.value > threshold=1.5989137291908264
node_1991: feature_name=COTL1	COTL1.value > threshold=-1.2334879636764526
node_1997: feature_name=HSP90B1	HSP90B1.value > threshold=1.903715431690216
node_2023: feature_name=MARCH1	MARCH1.value > threshold=-0.33349713683128357
node_2025: feature_name=COTL1	COTL1.value > threshold=-0.5126059651374817
Class: COVID-19 Critical	
Rules_997	passed counts:4
node_0: feature_name=LTB	LTB.value <= threshold=0.994031548500061
node_1: feature_name=MTRNR2L2	MTRNR2L2.value <= threshold=2.8784297704696655

node_2: feature_name=TCL1A	TCL1A.value <= threshold=4.798312664031982
node_3: feature_name=MT-CO1	MT-CO1.value <= threshold=0.08947191759943962
node_4: feature_name=NR4A1	NR4A1.value > threshold=1.0725348591804504
node_1622: feature_name=HLA-DQA2	HLA-DQA2.value <= threshold=1.9496816992759705
node_1623: feature_name=HLA-DRB5	HLA-DRB5.value <= threshold=2.715766429901123
node_1624: feature_name=HLA-DPA1	HLA-DPA1.value <= threshold=1.7050301432609558
node_1625: feature_name=CD83	CD83.value > threshold=2.783489227294922
node_1873: feature_name=IFI44L	IFI44L.value <= threshold=1.5938722491264343
node_1874: feature_name=PLD4	PLD4.value <= threshold=4.101001739501953
node_1875: feature_name=RHOB	RHOB.value > threshold=1.304835319519043
node_1965: feature_name=HLA-DQB1	HLA-DQB1.value > threshold=1.5989137291908264
node_1991: feature_name=COTL1	COTL1.value > threshold=-1.2334879636764526
node_1997: feature_name=HSP90B1	HSP90B1.value <= threshold=1.903715431690216
node_1998: feature_name=NFKBID	NFKBID.value <= threshold=1.8163637518882751
node_1999: feature_name=IFI6	IFI6.value <= threshold=-0.5193088352680206
node_2000: feature_name=LTB	LTB.value > threshold=0.580909252166748
Class: Healthy Control	
Rules_998	passed counts:4
node_0: feature_name=LTB	LTB.value <= threshold=0.994031548500061
node_1: feature_name=MTRNR2L2	MTRNR2L2.value <= threshold=2.8784297704696655
node_2: feature_name=TCL1A	TCL1A.value <= threshold=4.798312664031982
node_3: feature_name=MT-CO1	MT-CO1.value <= threshold=0.08947191759943962
node_4: feature_name=NR4A1	NR4A1.value > threshold=1.0725348591804504
node_1622: feature_name=HLA-DQA2	HLA-DQA2.value <= threshold=1.9496816992759705
node_1623: feature_name=HLA-DRB5	HLA-DRB5.value <= threshold=2.715766429901123
node_1624: feature_name=HLA-DPA1	HLA-DPA1.value <= threshold=1.7050301432609558
node_1625: feature_name=CD83	CD83.value > threshold=2.783489227294922
node_1873: feature_name=IFI44L	IFI44L.value <= threshold=1.5938722491264343
node_1874: feature_name=PLD4	PLD4.value <= threshold=4.101001739501953

node_1875: feature_name=RHOB	RHOB.value > threshold=1.304835319519043
node_1965: feature_name=HLA-DQB1	HLA-DQB1.value <= threshold=1.5989137291908264
node_1966: feature_name=CD79A	CD79A.value > threshold=2.189441442489624
node_1974: feature_name=IGJ	IGJ.value > threshold=1.4252705574035645
node_1984: feature_name=BACH1	BACH1.value <= threshold=0.9873552322387695
node_1985: feature_name=SLC11A1	SLC11A1.value > threshold=-0.4224514663219452
Class: COVID-19 Critical	
Rules_999	passed counts:4
node_0: feature_name=LTB	LTB.value <= threshold=0.994031548500061
node_1: feature_name=MTRNR2L2	MTRNR2L2.value <= threshold=2.8784297704696655
node_2: feature_name=TCL1A	TCL1A.value <= threshold=4.798312664031982
node_3: feature_name=MT-CO1	MT-CO1.value <= threshold=0.08947191759943962
node_4: feature_name=NR4A1	NR4A1.value > threshold=1.0725348591804504
node_1622: feature_name=HLA-DQA2	HLA-DQA2.value <= threshold=1.9496816992759705
node_1623: feature_name=HLA-DRB5	HLA-DRB5.value <= threshold=2.715766429901123
node_1624: feature_name=HLA-DPA1	HLA-DPA1.value <= threshold=1.7050301432609558
node_1625: feature_name=CD83	CD83.value > threshold=2.783489227294922
node_1873: feature_name=IFI44L	IFI44L.value <= threshold=1.5938722491264343
node_1874: feature_name=PLD4	PLD4.value <= threshold=4.101001739501953
node_1875: feature_name=RHOB	RHOB.value <= threshold=1.304835319519043
node_1876: feature_name=PPIF	PPIF.value <= threshold=2.2943923473358154
node_1877: feature_name=OAZ1	OAZ1.value > threshold=0.04260866530239582
node_1935: feature_name=RGS1	RGS1.value > threshold=-0.13325228542089462
node_1955: feature_name=DUSP4	DUSP4.value > threshold=1.2541037425398827
Class: COVID-19 Critical	
Rules_1000	passed counts:4
node_0: feature_name=LTB	LTB.value <= threshold=0.994031548500061
node_1: feature_name=MTRNR2L2	MTRNR2L2.value <= threshold=2.8784297704696655

node_2: feature_name=TCL1A	TCL1A.value <= threshold=4.798312664031982
node_3: feature_name=MT-CO1	MT-CO1.value <= threshold=0.08947191759943962
node_4: feature_name=NR4A1	NR4A1.value > threshold=1.0725348591804504
node_1622: feature_name=HLA-DQA2	HLA-DQA2.value <= threshold=1.9496816992759705
node_1623: feature_name=HLA-DRB5	HLA-DRB5.value <= threshold=2.715766429901123
node_1624: feature_name=HLA-DPA1	HLA-DPA1.value <= threshold=1.7050301432609558
node_1625: feature_name=CD83	CD83.value > threshold=2.783489227294922
node_1873: feature_name=IFI44L	IFI44L.value <= threshold=1.5938722491264343
node_1874: feature_name=PLD4	PLD4.value <= threshold=4.101001739501953
node_1875: feature_name=RHOB	RHOB.value <= threshold=1.304835319519043
node_1876: feature_name=PPIF	PPIF.value <= threshold=2.2943923473358154
node_1877: feature_name=OAZ1	OAZ1.value > threshold=0.04260866530239582
node_1935: feature_name=RGS1	RGS1.value <= threshold=-0.13325228542089462
node_1936: feature_name=BCL2A1	BCL2A1.value <= threshold=2.528669595718384
node_1937: feature_name=RALGPS2	RALGPS2.value > threshold=2.1082775592803955
node_1945: feature_name=MT-ND6	MT-ND6.value > threshold=-0.9341086447238922
node_1947: feature_name=RHOB	RHOB.value <= threshold=-0.4660041630268097
node_1948: feature_name=CXorf57	CXorf57.value <= threshold=-0.2075229212641716
Class: COVID-19 Severe	
Rules_1001	passed counts:4
node_0: feature_name=LTB	LTB.value <= threshold=0.994031548500061
node_1: feature_name=MTRNR2L2	MTRNR2L2.value <= threshold=2.8784297704696655
node_2: feature_name=TCL1A	TCL1A.value <= threshold=4.798312664031982
node_3: feature_name=MT-CO1	MT-CO1.value <= threshold=0.08947191759943962
node_4: feature_name=NR4A1	NR4A1.value > threshold=1.0725348591804504
node_1622: feature_name=HLA-DQA2	HLA-DQA2.value <= threshold=1.9496816992759705
node_1623: feature_name=HLA-DRB5	HLA-DRB5.value <= threshold=2.715766429901123
node_1624: feature_name=HLA-DPA1	HLA-DPA1.value <= threshold=1.7050301432609558
node_1625: feature_name=CD83	CD83.value > threshold=2.783489227294922

node_1873: feature_name=IFI44L	IFI44L.value <= threshold=1.5938722491264343
node_1874: feature_name=PLD4	PLD4.value <= threshold=4.101001739501953
node_1875: feature_name=RHOB	RHOB.value <= threshold=1.304835319519043
node_1876: feature_name=PPIF	PPIF.value <= threshold=2.2943923473358154
node_1877: feature_name=OAZ1	OAZ1.value <= threshold=0.04260866530239582
node_1878: feature_name=ID3	ID3.value <= threshold=5.290210247039795
node_1879: feature_name=NFKBID	NFKBID.value > threshold=-0.40532417595386505
node_1883: feature_name=MEF2C	MEF2C.value > threshold=-0.4597593992948532
node_1887: feature_name=SNX9	SNX9.value <= threshold=1.010923683643341
node_1888: feature_name=HLA-DRB5	HLA-DRB5.value <= threshold=-0.4844578504562378
Class: COVID-19 Severe	
Rules_1002	passed counts:4
node_0: feature_name=LTB	LTB.value <= threshold=0.994031548500061
node_1: feature_name=MTRNR2L2	MTRNR2L2.value <= threshold=2.8784297704696655
node_2: feature_name=TCL1A	TCL1A.value <= threshold=4.798312664031982
node_3: feature_name=MT-CO1	MT-CO1.value <= threshold=0.08947191759943962
node_4: feature_name=NR4A1	NR4A1.value > threshold=1.0725348591804504
node_1622: feature_name=HLA-DQA2	HLA-DQA2.value <= threshold=1.9496816992759705
node_1623: feature_name=HLA-DRB5	HLA-DRB5.value <= threshold=2.715766429901123
node_1624: feature_name=HLA-DPA1	HLA-DPA1.value <= threshold=1.7050301432609558
node_1625: feature_name=CD83	CD83.value > threshold=2.783489227294922
node_1873: feature_name=IFI44L	IFI44L.value <= threshold=1.5938722491264343
node_1874: feature_name=PLD4	PLD4.value <= threshold=4.101001739501953
node_1875: feature_name=RHOB	RHOB.value <= threshold=1.304835319519043
node_1876: feature_name=PPIF	PPIF.value <= threshold=2.2943923473358154
node_1877: feature_name=OAZ1	OAZ1.value <= threshold=0.04260866530239582
node_1878: feature_name=ID3	ID3.value <= threshold=5.290210247039795
node_1879: feature_name=NFKBID	NFKBID.value > threshold=-0.40532417595386505
node_1883: feature_name=MEF2C	MEF2C.value <= threshold=-0.4597593992948532

node_1884: feature_name=TRAF1	TRAF1.value <= threshold=-0.25039780884981155
Class: COVID-19 Severe	
Rules_1003	passed counts:4
node_0: feature_name=LTB	LTB.value <= threshold=0.994031548500061
node_1: feature_name=MTRNR2L2	MTRNR2L2.value <= threshold=2.8784297704696655
node_2: feature_name=TCL1A	TCL1A.value <= threshold=4.798312664031982
node_3: feature_name=MT-CO1	MT-CO1.value <= threshold=0.08947191759943962
node_4: feature_name=NR4A1	NR4A1.value > threshold=1.0725348591804504
node_1622: feature_name=HLA-DQA2	HLA-DQA2.value <= threshold=1.9496816992759705
node_1623: feature_name=HLA-DRB5	HLA-DRB5.value <= threshold=2.715766429901123
node_1624: feature_name=HLA-DPA1	HLA-DPA1.value <= threshold=1.7050301432609558
node_1625: feature_name=CD83	CD83.value <= threshold=2.783489227294922
node_1626: feature_name=XIST	XIST.value <= threshold=0.9589543007314205
node_1627: feature_name=PLD4	PLD4.value <= threshold=5.865932464599609
node_1628: feature_name=OAZ1	OAZ1.value > threshold=0.6137169003486633
node_1840: feature_name=MT-CO1	MT-CO1.value <= threshold=-0.28350088000297546
node_1841: feature_name=AHR	AHR.value <= threshold=1.9306254982948303
node_1842: feature_name=SYNE1	SYNE1.value > threshold=0.17462080717086792
node_1844: feature_name=MIR24-2	MIR24-2.value > threshold=-0.504669725894928
Class: COVID-19 Critical	
Rules_1004	passed counts:4
node_0: feature_name=LTB	LTB.value <= threshold=0.994031548500061
node_1: feature_name=MTRNR2L2	MTRNR2L2.value <= threshold=2.8784297704696655
node_2: feature_name=TCL1A	TCL1A.value <= threshold=4.798312664031982
node_3: feature_name=MT-CO1	MT-CO1.value <= threshold=0.08947191759943962
node_4: feature_name=NR4A1	NR4A1.value > threshold=1.0725348591804504
node_1622: feature_name=HLA-DQA2	HLA-DQA2.value <= threshold=1.9496816992759705
node_1623: feature_name=HLA-DRB5	HLA-DRB5.value <= threshold=2.715766429901123

node_1624: feature_name=HLA-DPA1	HLA-DPA1.value <= threshold=1.7050301432609558
node_1625: feature_name=CD83	CD83.value <= threshold=2.783489227294922
node_1626: feature_name=XIST	XIST.value <= threshold=0.9589543007314205
node_1627: feature_name=PLD4	PLD4.value <= threshold=5.865932464599609
node_1628: feature_name=OAZ1	OAZ1.value <= threshold=0.6137169003486633
node_1629: feature_name=MTRNR2L2	MTRNR2L2.value <= threshold=1.6102792620658875
node_1630: feature_name=IFI6	IFI6.value > threshold=-0.8404486179351807
node_1632: feature_name=NR4A1	NR4A1.value > threshold=1.0916320085525513
node_1638: feature_name=DENND3	DENND3.value <= threshold=3.988452672958374
node_1639: feature_name=HLA-DRB1	HLA-DRB1.value <= threshold=1.4092575907707214
node_1640: feature_name=HLA-DPA1	HLA-DPA1.value > threshold=1.2675696015357971
node_1708: feature_name=CYBB	CYBB.value <= threshold=1.8550206422805786
node_1709: feature_name=JUN	JUN.value > threshold=-0.04836222808808088
node_1723: feature_name=NT5C3A	NT5C3A.value <= threshold=1.9380813241004944
node_1724: feature_name=FAM129C	FAM129C.value <= threshold=4.6762073040008545
node_1725: feature_name=FERMT3	FERMT3.value <= threshold=1.1511099338531494
node_1726: feature_name=GPX1	GPX1.value > threshold=0.10868745669722557
node_1734: feature_name=RALGPS2	RALGPS2.value > threshold=1.23886239528656
node_1736: feature_name=COTL1	COTL1.value > threshold=-0.8507162630558014
node_1738: feature_name=OAZ1	OAZ1.value > threshold=-0.3851434364914894
Class: Healthy Control	
Rules_1005	passed counts:4
node_0: feature_name=LTB	LTB.value <= threshold=0.994031548500061
node_1: feature_name=MTRNR2L2	MTRNR2L2.value <= threshold=2.8784297704696655
node_2: feature_name=TCL1A	TCL1A.value <= threshold=4.798312664031982
node_3: feature_name=MT-CO1	MT-CO1.value <= threshold=0.08947191759943962
node_4: feature_name=NR4A1	NR4A1.value > threshold=1.0725348591804504
node_1622: feature_name=HLA-DQA2	HLA-DQA2.value <= threshold=1.9496816992759705
node_1623: feature_name=HLA-DRB5	HLA-DRB5.value <= threshold=2.715766429901123

node_1624: feature_name=HLA-DPA1	HLA-DPA1.value <= threshold=1.7050301432609558
node_1625: feature_name=CD83	CD83.value <= threshold=2.783489227294922
node_1626: feature_name=XIST	XIST.value <= threshold=0.9589543007314205
node_1627: feature_name=PLD4	PLD4.value <= threshold=5.865932464599609
node_1628: feature_name=OAZ1	OAZ1.value <= threshold=0.6137169003486633
node_1629: feature_name=MTRNR2L2	MTRNR2L2.value <= threshold=1.6102792620658875
node_1630: feature_name=IFI6	IFI6.value > threshold=-0.8404486179351807
node_1632: feature_name=NR4A1	NR4A1.value > threshold=1.0916320085525513
node_1638: feature_name=DENND3	DENND3.value <= threshold=3.988452672958374
node_1639: feature_name=HLA-DRB1	HLA-DRB1.value <= threshold=1.4092575907707214
node_1640: feature_name=HLA-DPA1	HLA-DPA1.value <= threshold=1.2675696015357971
node_1641: feature_name=TRAF1	TRAF1.value <= threshold=2.918454885482788
node_1642: feature_name=HLA-DPA1	HLA-DPA1.value > threshold=-0.917132705450058
node_1648: feature_name=LTB	LTB.value <= threshold=0.9716593027114868
node_1649: feature_name=LDLRAD4	LDLRAD4.value <= threshold=4.4251909255981445
node_1650: feature_name=BCL2A1	BCL2A1.value > threshold=-0.7959703803062439
node_1652: feature_name=PRDM1	PRDM1.value <= threshold=2.6821553707122803
node_1653: feature_name=FAM129C	FAM129C.value <= threshold=4.9084858894348145
node_1654: feature_name=BOD1L1	BOD1L1.value <= threshold=2.9830023050308228
node_1655: feature_name=HSP90B1	HSP90B1.value <= threshold=2.581766724586487
node_1656: feature_name=HLA-DQB1	HLA-DQB1.value <= threshold=3.3723777532577515
node_1657: feature_name=ANO6	ANO6.value <= threshold=2.491254210472107
node_1658: feature_name=RP5-821D11.7	RP5-821D11.7.value <= threshold=3.2165164947509766
node_1659: feature_name=MARCH1	MARCH1.value <= threshold=4.2638514041900635
node_1660: feature_name=ABCA1	ABCA1.value <= threshold=3.618377923965454
node_1661: feature_name=LTB	LTB.value <= threshold=0.8862588405609131
node_1662: feature_name=SNORD3B-2	SNORD3B-2.value <= threshold=4.273592233657837
node_1663: feature_name=S100A6	S100A6.value <= threshold=0.29081472009420395
node_1664: feature_name=IGLL5	IGLL5.value <= threshold=6.102234125137329
node_1665: feature_name=STAG3	STAG3.value <= threshold=6.671069860458374

node_1666: feature_name=HLA-DQA1	HLA-DQA1.value <= threshold=-0.5025019943714142
node_1667: feature_name=CASP8	CASP8.value > threshold=-0.5568675696849823
Class: COVID-19 Critical	
Rules_1006	passed counts:4
node_0: feature_name=LTB	LTB.value <= threshold=0.994031548500061
node_1: feature_name=MTRNR2L2	MTRNR2L2.value <= threshold=2.8784297704696655
node_2: feature_name=TCL1A	TCL1A.value <= threshold=4.798312664031982
node_3: feature_name=MT-CO1	MT-CO1.value <= threshold=0.08947191759943962
node_4: feature_name=NR4A1	NR4A1.value > threshold=1.0725348591804504
node_1622: feature_name=HLA-DQA2	HLA-DQA2.value <= threshold=1.9496816992759705
node_1623: feature_name=HLA-DRB5	HLA-DRB5.value <= threshold=2.715766429901123
node_1624: feature_name=HLA-DPA1	HLA-DPA1.value <= threshold=1.7050301432609558
node_1625: feature_name=CD83	CD83.value <= threshold=2.783489227294922
node_1626: feature_name=XIST	XIST.value <= threshold=0.9589543007314205
node_1627: feature_name=PLD4	PLD4.value <= threshold=5.865932464599609
node_1628: feature_name=OAZ1	OAZ1.value <= threshold=0.6137169003486633
node_1629: feature_name=MTRNR2L2	MTRNR2L2.value <= threshold=1.6102792620658875
node_1630: feature_name=IFI6	IFI6.value > threshold=-0.8404486179351807
node_1632: feature_name=NR4A1	NR4A1.value <= threshold=1.0916320085525513
node_1633: feature_name=PARP14	PARP14.value <= threshold=-0.4991041421890259
Class: COVID-19 Severe	
Rules_1007	passed counts:4
node_0: feature_name=LTB	LTB.value <= threshold=0.994031548500061
node_1: feature_name=MTRNR2L2	MTRNR2L2.value <= threshold=2.8784297704696655
node_2: feature_name=TCL1A	TCL1A.value <= threshold=4.798312664031982
node_3: feature_name=MT-CO1	MT-CO1.value <= threshold=0.08947191759943962
node_4: feature_name=NR4A1	NR4A1.value <= threshold=1.0725348591804504
node_5: feature_name=HLA-DPA1	HLA-DPA1.value > threshold=1.6885469555854797

node_861: feature_name=CD83	CD83.value > threshold=3.0563642978668213
node_1539: feature_name=ID1	ID1.value > threshold=1.4626862406730652
node_1615: feature_name=CD79B	CD79B.value > threshold=2.151446223258972
node_1619: feature_name=HLA-DQB1	HLA-DQB1.value <= threshold=2.806690812110901
Class: COVID-19 Critical	
Rules_1008	passed counts:4
node_0: feature_name=LTB	LTB.value <= threshold=0.994031548500061
node_1: feature_name=MTRNR2L2	MTRNR2L2.value <= threshold=2.8784297704696655
node_2: feature_name=TCL1A	TCL1A.value <= threshold=4.798312664031982
node_3: feature_name=MT-CO1	MT-CO1.value <= threshold=0.08947191759943962
node_4: feature_name=NR4A1	NR4A1.value <= threshold=1.0725348591804504
node_5: feature_name=HLA-DPA1	HLA-DPA1.value > threshold=1.6885469555854797
node_861: feature_name=CD83	CD83.value > threshold=3.0563642978668213
node_1539: feature_name=ID1	ID1.value <= threshold=1.4626862406730652
node_1540: feature_name=FTH1	FTH1.value > threshold=0.2985198348760605
node_1590: feature_name=RP5-821D11.7	RP5-821D11.7.value > threshold=-0.2580558806657791
node_1610: feature_name=CD79B	CD79B.value > threshold=2.037744402885437
node_1612: feature_name=BCL2A1	BCL2A1.value > threshold=0.11882442235946655
Class: COVID-19 Critical	
Rules_1009	passed counts:4
node_0: feature_name=LTB	LTB.value <= threshold=0.994031548500061
node_1: feature_name=MTRNR2L2	MTRNR2L2.value <= threshold=2.8784297704696655
node_2: feature_name=TCL1A	TCL1A.value <= threshold=4.798312664031982
node_3: feature_name=MT-CO1	MT-CO1.value <= threshold=0.08947191759943962
node_4: feature_name=NR4A1	NR4A1.value <= threshold=1.0725348591804504
node_5: feature_name=HLA-DPA1	HLA-DPA1.value > threshold=1.6885469555854797
node_861: feature_name=CD83	CD83.value > threshold=3.0563642978668213
node_1539: feature_name=ID1	ID1.value <= threshold=1.4626862406730652

node_1540: feature_name=FTH1	FTH1.value <= threshold=0.2985198348760605
node_1541: feature_name=SESN3	SESN3.value <= threshold=2.7280690670013428
node_1542: feature_name=HOPX	HOPX.value <= threshold=-0.46606285870075226
node_1543: feature_name=ATF3	ATF3.value > threshold=1.6206613183021545
Class: COVID-19 Severe	
Rules_1010	passed counts:4
node_0: feature_name=LTB	LTB.value <= threshold=0.994031548500061
node_1: feature_name=MTRNR2L2	MTRNR2L2.value <= threshold=2.8784297704696655
node_2: feature_name=TCL1A	TCL1A.value <= threshold=4.798312664031982
node_3: feature_name=MT-CO1	MT-CO1.value <= threshold=0.08947191759943962
node_4: feature_name=NR4A1	NR4A1.value <= threshold=1.0725348591804504
node_5: feature_name=HLA-DPA1	HLA-DPA1.value > threshold=1.6885469555854797
node_861: feature_name=CD83	CD83.value > threshold=3.0563642978668213
node_1539: feature_name=ID1	ID1.value <= threshold=1.4626862406730652
node_1540: feature_name=FTH1	FTH1.value <= threshold=0.2985198348760605
node_1541: feature_name=SESN3	SESN3.value <= threshold=2.7280690670013428
node_1542: feature_name=HOPX	HOPX.value <= threshold=-0.46606285870075226
node_1543: feature_name=ATF3	ATF3.value <= threshold=1.6206613183021545
node_1544: feature_name=HLA-DQA1	HLA-DQA1.value > threshold=2.5666390657424927
node_1562: feature_name=HLA-DQA1	HLA-DQA1.value <= threshold=2.702773928642273
Class: COVID-19 Severe	
Rules_1011	passed counts:4
node_0: feature_name=LTB	LTB.value <= threshold=0.994031548500061
node_1: feature_name=MTRNR2L2	MTRNR2L2.value <= threshold=2.8784297704696655
node_2: feature_name=TCL1A	TCL1A.value <= threshold=4.798312664031982
node_3: feature_name=MT-CO1	MT-CO1.value <= threshold=0.08947191759943962
node_4: feature_name=NR4A1	NR4A1.value <= threshold=1.0725348591804504
node_5: feature_name=HLA-DPA1	HLA-DPA1.value > threshold=1.6885469555854797

node_861: feature_name=CD83	CD83.value > threshold=3.0563642978668213
node_1539: feature_name=ID1	ID1.value <= threshold=1.4626862406730652
node_1540: feature_name=FTH1	FTH1.value <= threshold=0.2985198348760605
node_1541: feature_name=SESN3	SESN3.value <= threshold=2.7280690670013428
node_1542: feature_name=HOPX	HOPX.value <= threshold=-0.46606285870075226
node_1543: feature_name=ATF3	ATF3.value <= threshold=1.6206613183021545
node_1544: feature_name=HLA-DQA1	HLA-DQA1.value <= threshold=2.5666390657424927
node_1545: feature_name=STMN1	STMN1.value > threshold=0.9797938093543053
node_1559: feature_name=NFKBIZ	NFKBIZ.value <= threshold=-0.8102173805236816
Class: COVID-19 Severe	
Rules_1012	passed counts:4
node_0: feature_name=LTB	LTB.value <= threshold=0.994031548500061
node_1: feature_name=MTRNR2L2	MTRNR2L2.value <= threshold=2.8784297704696655
node_2: feature_name=TCL1A	TCL1A.value <= threshold=4.798312664031982
node_3: feature_name=MT-CO1	MT-CO1.value <= threshold=0.08947191759943962
node_4: feature_name=NR4A1	NR4A1.value <= threshold=1.0725348591804504
node_5: feature_name=HLA-DPA1	HLA-DPA1.value > threshold=1.6885469555854797
node_861: feature_name=CD83	CD83.value <= threshold=3.0563642978668213
node_862: feature_name=HLA-DQB1	HLA-DQB1.value > threshold=2.61081063747406
node_1358: feature_name=HLA-DRB5	HLA-DRB5.value > threshold=0.7542744874954224
node_1390: feature_name=HLA-DRB5	HLA-DRB5.value <= threshold=2.6244447231292725
node_1391: feature_name=XIST	XIST.value > threshold=-0.22017984092235565
node_1497: feature_name=HLA-DRB5	HLA-DRB5.value > threshold=1.2674232721328735
node_1503: feature_name=HLA-DQA1	HLA-DQA1.value <= threshold=2.4817473888397217
node_1504: feature_name=HLA-DQB1	HLA-DQB1.value <= threshold=2.675566077232361
Class: Healthy Control	
Rules_1013	passed counts:4
node_0: feature_name=LTB	LTB.value <= threshold=0.994031548500061

node_1: feature_name=MTRNR2L2	MTRNR2L2.value <= threshold=2.8784297704696655
node_2: feature_name=TCL1A	TCL1A.value <= threshold=4.798312664031982
node_3: feature_name=MT-CO1	MT-CO1.value <= threshold=0.08947191759943962
node_4: feature_name=NR4A1	NR4A1.value <= threshold=1.0725348591804504
node_5: feature_name=HLA-DPA1	HLA-DPA1.value > threshold=1.6885469555854797
node_861: feature_name=CD83	CD83.value <= threshold=3.0563642978668213
node_862: feature_name=HLA-DQB1	HLA-DQB1.value > threshold=2.61081063747406
node_1358: feature_name=HLA-DRB5	HLA-DRB5.value > threshold=0.7542744874954224
node_1390: feature_name=HLA-DRB5	HLA-DRB5.value <= threshold=2.6244447231292725
node_1391: feature_name=XIST	XIST.value <= threshold=-0.22017984092235565
node_1392: feature_name=MT-CO3	MT-CO3.value > threshold=0.12647542357444763
node_1472: feature_name=IFI44L	IFI44L.value <= threshold=-0.34079885482788086
node_1473: feature_name=TNFAIP3	TNFAIP3.value <= threshold=-0.0701381303369999
node_1474: feature_name=HSP90B1	HSP90B1.value <= threshold=0.9771479368209839
node_1475: feature_name=CD79A	CD79A.value > threshold=2.6819008588790894
node_1483: feature_name=LTB	LTB.value <= threshold=0.06463655456900597
Class: COVID-19 Critical	
Rules_1014	passed counts:4
node_0: feature_name=LTB	LTB.value <= threshold=0.994031548500061
node_1: feature_name=MTRNR2L2	MTRNR2L2.value <= threshold=2.8784297704696655
node_2: feature_name=TCL1A	TCL1A.value <= threshold=4.798312664031982
node_3: feature_name=MT-CO1	MT-CO1.value <= threshold=0.08947191759943962
node_4: feature_name=NR4A1	NR4A1.value <= threshold=1.0725348591804504
node_5: feature_name=HLA-DPA1	HLA-DPA1.value > threshold=1.6885469555854797
node_861: feature_name=CD83	CD83.value <= threshold=3.0563642978668213
node_862: feature_name=HLA-DQB1	HLA-DQB1.value > threshold=2.61081063747406
node_1358: feature_name=HLA-DRB5	HLA-DRB5.value > threshold=0.7542744874954224
node_1390: feature_name=HLA-DRB5	HLA-DRB5.value <= threshold=2.6244447231292725
node_1391: feature_name=XIST	XIST.value <= threshold=-0.22017984092235565

node_1392: feature_name=MT-CO3	MT-CO3.value > threshold=0.12647542357444763
node_1472: feature_name=IFI44L	IFI44L.value <= threshold=-0.34079885482788086
node_1473: feature_name=TNFAIP3	TNFAIP3.value <= threshold=-0.0701381303369999
node_1474: feature_name=HSP90B1	HSP90B1.value <= threshold=0.9771479368209839
node_1475: feature_name=CD79A	CD79A.value <= threshold=2.6819008588790894
node_1476: feature_name=HLA-DPA1	HLA-DPA1.value <= threshold=1.8044293522834778
Class: COVID-19 Severe	
Rules_1015	passed counts:4
node_0: feature_name=LTB	LTB.value <= threshold=0.994031548500061
node_1: feature_name=MTRNR2L2	MTRNR2L2.value <= threshold=2.8784297704696655
node_2: feature_name=TCL1A	TCL1A.value <= threshold=4.798312664031982
node_3: feature_name=MT-CO1	MT-CO1.value <= threshold=0.08947191759943962
node_4: feature_name=NR4A1	NR4A1.value <= threshold=1.0725348591804504
node_5: feature_name=HLA-DPA1	HLA-DPA1.value > threshold=1.6885469555854797
node_861: feature_name=CD83	CD83.value <= threshold=3.0563642978668213
node_862: feature_name=HLA-DQB1	HLA-DQB1.value > threshold=2.61081063747406
node_1358: feature_name=HLA-DRB5	HLA-DRB5.value > threshold=0.7542744874954224
node_1390: feature_name=HLA-DRB5	HLA-DRB5.value <= threshold=2.6244447231292725
node_1391: feature_name=XIST	XIST.value <= threshold=-0.22017984092235565
node_1392: feature_name=MT-CO3	MT-CO3.value <= threshold=0.12647542357444763
node_1393: feature_name=CD79B	CD79B.value > threshold=4.338301658630371
node_1469: feature_name=UTY	UTY.value <= threshold=1.6515765190124512
Class: Healthy Control	
Rules_1016	passed counts:4
node_0: feature_name=LTB	LTB.value <= threshold=0.994031548500061
node_1: feature_name=MTRNR2L2	MTRNR2L2.value <= threshold=2.8784297704696655
node_2: feature_name=TCL1A	TCL1A.value <= threshold=4.798312664031982
node_3: feature_name=MT-CO1	MT-CO1.value <= threshold=0.08947191759943962

node_4: feature_name=NR4A1	NR4A1.value <= threshold=1.0725348591804504
node_5: feature_name=HLA-DPA1	HLA-DPA1.value > threshold=1.6885469555854797
node_861: feature_name=CD83	CD83.value <= threshold=3.0563642978668213
node_862: feature_name=HLA-DQB1	HLA-DQB1.value > threshold=2.61081063747406
node_1358: feature_name=HLA-DRB5	HLA-DRB5.value > threshold=0.7542744874954224
node_1390: feature_name=HLA-DRB5	HLA-DRB5.value <= threshold=2.6244447231292725
node_1391: feature_name=XIST	XIST.value <= threshold=-0.22017984092235565
node_1392: feature_name=MT-CO3	MT-CO3.value <= threshold=0.12647542357444763
node_1393: feature_name=CD79B	CD79B.value <= threshold=4.338301658630371
node_1394: feature_name=LTB	LTB.value <= threshold=0.9865012466907501
node_1395: feature_name=MEF2C	MEF2C.value <= threshold=4.133931398391724
node_1396: feature_name=MEF2C	MEF2C.value > threshold=-0.38076959550380707
node_1418: feature_name=HLA-DPB1	HLA-DPB1.value <= threshold=0.8961269855499268
node_1419: feature_name=IRF8	IRF8.value > threshold=3.3565738201141357
Class: COVID-19 Severe	
Rules_1017	passed counts:4
node_0: feature_name=LTB	LTB.value <= threshold=0.994031548500061
node_1: feature_name=MTRNR2L2	MTRNR2L2.value <= threshold=2.8784297704696655
node_2: feature_name=TCL1A	TCL1A.value <= threshold=4.798312664031982
node_3: feature_name=MT-CO1	MT-CO1.value <= threshold=0.08947191759943962
node_4: feature_name=NR4A1	NR4A1.value <= threshold=1.0725348591804504
node_5: feature_name=HLA-DPA1	HLA-DPA1.value > threshold=1.6885469555854797
node_861: feature_name=CD83	CD83.value <= threshold=3.0563642978668213
node_862: feature_name=HLA-DQB1	HLA-DQB1.value <= threshold=2.61081063747406
node_863: feature_name=MTRNR2L2	MTRNR2L2.value <= threshold=2.0919997692108154
node_864: feature_name=BCL2A1	BCL2A1.value > threshold=1.852618932723999
node_1328: feature_name=ATF5	ATF5.value <= threshold=1.995988368988037
node_1329: feature_name=S100A6	S100A6.value <= threshold=-1.6572160124778748
node_1330: feature_name=JARID2	JARID2.value <= threshold=-0.4899669587612152

Class: COVID-19 Critical	
Rules_1018	passed counts:4
node_0: feature_name=LTB	LTB.value <= threshold=0.994031548500061
node_1: feature_name=MTRNR2L2	MTRNR2L2.value <= threshold=2.8784297704696655
node_2: feature_name=TCL1A	TCL1A.value <= threshold=4.798312664031982
node_3: feature_name=MT-CO1	MT-CO1.value <= threshold=0.08947191759943962
node_4: feature_name=NR4A1	NR4A1.value <= threshold=1.0725348591804504
node_5: feature_name=HLA-DPA1	HLA-DPA1.value > threshold=1.6885469555854797
node_861: feature_name=CD83	CD83.value <= threshold=3.0563642978668213
node_862: feature_name=HLA-DQB1	HLA-DQB1.value <= threshold=2.61081063747406
node_863: feature_name=MTRNR2L2	MTRNR2L2.value <= threshold=2.0919997692108154
node_864: feature_name=BCL2A1	BCL2A1.value <= threshold=1.852618932723999
node_865: feature_name=CD79B	CD79B.value > threshold=4.352142333984375
node_1309: feature_name=COTL1	COTL1.value > threshold=0.48328976333141327
node_1321: feature_name=MS4A1	MS4A1.value <= threshold=2.450835347175598
Class: COVID-19 Critical	
Rules_1019	passed counts:4
node_0: feature_name=LTB	LTB.value <= threshold=0.994031548500061
node_1: feature_name=MTRNR2L2	MTRNR2L2.value <= threshold=2.8784297704696655
node_2: feature_name=TCL1A	TCL1A.value <= threshold=4.798312664031982
node_3: feature_name=MT-CO1	MT-CO1.value <= threshold=0.08947191759943962
node_4: feature_name=NR4A1	NR4A1.value <= threshold=1.0725348591804504
node_5: feature_name=HLA-DPA1	HLA-DPA1.value > threshold=1.6885469555854797
node_861: feature_name=CD83	CD83.value <= threshold=3.0563642978668213
node_862: feature_name=HLA-DQB1	HLA-DQB1.value <= threshold=2.61081063747406
node_863: feature_name=MTRNR2L2	MTRNR2L2.value <= threshold=2.0919997692108154
node_864: feature_name=BCL2A1	BCL2A1.value <= threshold=1.852618932723999
node_865: feature_name=CD79B	CD79B.value <= threshold=4.352142333984375

node_866: feature_name=TNFAIP3	TNFAIP3.value > threshold=-0.0449496041983366
node_1236: feature_name=HLA-DRB5	HLA-DRB5.value <= threshold=1.710871696472168
node_1237: feature_name=HLA-DQA2	HLA-DQA2.value <= threshold=3.6443899869918823
node_1238: feature_name=FAM160B1	FAM160B1.value > threshold=-0.36028963327407837
node_1256: feature_name=RGS2	RGS2.value <= threshold=-0.6350596249103546
Class: COVID-19 Severe	
Rules_1020	passed counts:4
node_0: feature_name=LTB	LTB.value <= threshold=0.994031548500061
node_1: feature_name=MTRNR2L2	MTRNR2L2.value <= threshold=2.8784297704696655
node_2: feature_name=TCL1A	TCL1A.value <= threshold=4.798312664031982
node_3: feature_name=MT-CO1	MT-CO1.value <= threshold=0.08947191759943962
node_4: feature_name=NR4A1	NR4A1.value <= threshold=1.0725348591804504
node_5: feature_name=HLA-DPA1	HLA-DPA1.value > threshold=1.6885469555854797
node_861: feature_name=CD83	CD83.value <= threshold=3.0563642978668213
node_862: feature_name=HLA-DQB1	HLA-DQB1.value <= threshold=2.61081063747406
node_863: feature_name=MTRNR2L2	MTRNR2L2.value <= threshold=2.0919997692108154
node_864: feature_name=BCL2A1	BCL2A1.value <= threshold=1.852618932723999
node_865: feature_name=CD79B	CD79B.value <= threshold=4.352142333984375
node_866: feature_name=TNFAIP3	TNFAIP3.value <= threshold=-0.0449496041983366
node_867: feature_name=MT-CO1	MT-CO1.value > threshold=-0.08531957119703293
node_1155: feature_name=IRF8	IRF8.value > threshold=-0.39239931106567383
node_1177: feature_name=PELI1	PELI1.value > threshold=-0.6234017610549927
node_1203: feature_name=STMN1	STMN1.value <= threshold=-0.2129739671945572
node_1204: feature_name=HLA-DPA1	HLA-DPA1.value > threshold=1.6937929391860962
node_1210: feature_name=MT-CO2	MT-CO2.value <= threshold=-0.5078963339328766
node_1211: feature_name=OAZ1	OAZ1.value > threshold=-0.9088589698076248
node_1213: feature_name=LTB	LTB.value > threshold=0.30608798563480377
node_1215: feature_name=PELI1	PELI1.value > threshold=1.7061399221420288
Class: COVID-19 Critical	

Rules_1021	passed counts:4
node_0: feature_name=LTB	LTB.value <= threshold=0.994031548500061
node_1: feature_name=MTRNR2L2	MTRNR2L2.value <= threshold=2.8784297704696655
node_2: feature_name=TCL1A	TCL1A.value <= threshold=4.798312664031982
node_3: feature_name=MT-CO1	MT-CO1.value <= threshold=0.08947191759943962
node_4: feature_name=NR4A1	NR4A1.value <= threshold=1.0725348591804504
node_5: feature_name=HLA-DPA1	HLA-DPA1.value > threshold=1.6885469555854797
node_861: feature_name=CD83	CD83.value <= threshold=3.0563642978668213
node_862: feature_name=HLA-DQB1	HLA-DQB1.value <= threshold=2.61081063747406
node_863: feature_name=MTRNR2L2	MTRNR2L2.value <= threshold=2.0919997692108154
node_864: feature_name=BCL2A1	BCL2A1.value <= threshold=1.852618932723999
node_865: feature_name=CD79B	CD79B.value <= threshold=4.352142333984375
node_866: feature_name=TNFAIP3	TNFAIP3.value <= threshold=-0.0449496041983366
node_867: feature_name=MT-CO1	MT-CO1.value > threshold=-0.08531957119703293
node_1155: feature_name=IRF8	IRF8.value > threshold=-0.39239931106567383
node_1177: feature_name=PELI1	PELI1.value <= threshold=-0.6234017610549927
node_1178: feature_name=HLA-DRB1	HLA-DRB1.value > threshold=1.3807418942451477
node_1182: feature_name=ANO6	ANO6.value <= threshold=-0.3352542072534561
node_1183: feature_name=MS4A1	MS4A1.value > threshold=1.8863030076026917
node_1187: feature_name=MT-CO2	MT-CO2.value <= threshold=0.32131922245025635
node_1188: feature_name=BANK1	BANK1.value > threshold=3.6148797273635864
Class: COVID-19 Critical	
Rules_1022	passed counts:4
node_0: feature_name=LTB	LTB.value <= threshold=0.994031548500061
node_1: feature_name=MTRNR2L2	MTRNR2L2.value <= threshold=2.8784297704696655
node_2: feature_name=TCL1A	TCL1A.value <= threshold=4.798312664031982
node_3: feature_name=MT-CO1	MT-CO1.value <= threshold=0.08947191759943962
node_4: feature_name=NR4A1	NR4A1.value <= threshold=1.0725348591804504

node_5: feature_name=HLA-DPA1	HLA-DPA1.value > threshold=1.6885469555854797
node_861: feature_name=CD83	CD83.value <= threshold=3.0563642978668213
node_862: feature_name=HLA-DQB1	HLA-DQB1.value <= threshold=2.61081063747406
node_863: feature_name=MTRNR2L2	MTRNR2L2.value <= threshold=2.0919997692108154
node_864: feature_name=BCL2A1	BCL2A1.value <= threshold=1.852618932723999
node_865: feature_name=CD79B	CD79B.value <= threshold=4.352142333984375
node_866: feature_name=TNFAIP3	TNFAIP3.value <= threshold=-0.0449496041983366
node_867: feature_name=MT-CO1	MT-CO1.value <= threshold=-0.08531957119703293
node_868: feature_name=MTRNR2L8	MTRNR2L8.value > threshold=0.8770350813865662
node_1148: feature_name=NR4A1	NR4A1.value <= threshold=0.3121328055858612
node_1149: feature_name=SNX29P2	SNX29P2.value > threshold=4.279992580413818
node_1151: feature_name=CD79A	CD79A.value <= threshold=2.6091065406799316
Class: COVID-19 Critical	
Rules_1023	passed counts:4
node_0: feature_name=LTB	LTB.value <= threshold=0.994031548500061
node_1: feature_name=MTRNR2L2	MTRNR2L2.value <= threshold=2.8784297704696655
node_2: feature_name=TCL1A	TCL1A.value <= threshold=4.798312664031982
node_3: feature_name=MT-CO1	MT-CO1.value <= threshold=0.08947191759943962
node_4: feature_name=NR4A1	NR4A1.value <= threshold=1.0725348591804504
node_5: feature_name=HLA-DPA1	HLA-DPA1.value > threshold=1.6885469555854797
node_861: feature_name=CD83	CD83.value <= threshold=3.0563642978668213
node_862: feature_name=HLA-DQB1	HLA-DQB1.value <= threshold=2.61081063747406
node_863: feature_name=MTRNR2L2	MTRNR2L2.value <= threshold=2.0919997692108154
node_864: feature_name=BCL2A1	BCL2A1.value <= threshold=1.852618932723999
node_865: feature_name=CD79B	CD79B.value <= threshold=4.352142333984375
node_866: feature_name=TNFAIP3	TNFAIP3.value <= threshold=-0.0449496041983366
node_867: feature_name=MT-CO1	MT-CO1.value <= threshold=-0.08531957119703293
node_868: feature_name=MTRNR2L8	MTRNR2L8.value <= threshold=0.8770350813865662
node_869: feature_name=EGR1	EGR1.value > threshold=-0.32270750403404236

node_1015: feature_name=NR4A3	NR4A3.value <= threshold=1.9085431098937988
node_1016: feature_name=ID3	ID3.value <= threshold=2.776647686958313
node_1017: feature_name=XIST	XIST.value <= threshold=2.618046522140503
node_1018: feature_name=CD79B	CD79B.value <= threshold=3.818994164466858
node_1019: feature_name=NFKBID	NFKBID.value > threshold=3.4191306829452515
node_1111: feature_name=RBKS	RBKS.value <= threshold=-0.2493925839662552
node_1112: feature_name=CYBB	CYBB.value <= threshold=-0.5100959241390228
node_1113: feature_name=PDLIM1	PDLIM1.value > threshold=-0.2814306765794754
Class: COVID-19 Severe	
Rules_1024	passed counts:4
node_0: feature_name=LTB	LTB.value <= threshold=0.994031548500061
node_1: feature_name=MTRNR2L2	MTRNR2L2.value <= threshold=2.8784297704696655
node_2: feature_name=TCL1A	TCL1A.value <= threshold=4.798312664031982
node_3: feature_name=MT-CO1	MT-CO1.value <= threshold=0.08947191759943962
node_4: feature_name=NR4A1	NR4A1.value <= threshold=1.0725348591804504
node_5: feature_name=HLA-DPA1	HLA-DPA1.value > threshold=1.6885469555854797
node_861: feature_name=CD83	CD83.value <= threshold=3.0563642978668213
node_862: feature_name=HLA-DQB1	HLA-DQB1.value <= threshold=2.61081063747406
node_863: feature_name=MTRNR2L2	MTRNR2L2.value <= threshold=2.0919997692108154
node_864: feature_name=BCL2A1	BCL2A1.value <= threshold=1.852618932723999
node_865: feature_name=CD79B	CD79B.value <= threshold=4.352142333984375
node_866: feature_name=TNFAIP3	TNFAIP3.value <= threshold=-0.0449496041983366
node_867: feature_name=MT-CO1	MT-CO1.value <= threshold=-0.08531957119703293
node_868: feature_name=MTRNR2L8	MTRNR2L8.value <= threshold=0.8770350813865662
node_869: feature_name=EGR1	EGR1.value > threshold=-0.32270750403404236
node_1015: feature_name=NR4A3	NR4A3.value <= threshold=1.9085431098937988
node_1016: feature_name=ID3	ID3.value <= threshold=2.776647686958313
node_1017: feature_name=XIST	XIST.value <= threshold=2.618046522140503
node_1018: feature_name=CD79B	CD79B.value <= threshold=3.818994164466858

node_1019: feature_name=NFKBID	NFKBID.value <= threshold=3.4191306829452515
node_1020: feature_name=MIAT	MIAT.value <= threshold=3.532225251197815
node_1021: feature_name=STAG3	STAG3.value <= threshold=9.534917831420898
node_1022: feature_name=OASL	OASL.value <= threshold=2.3923760652542114
node_1023: feature_name=HLA-DQB1	HLA-DQB1.value <= threshold=2.572031259536743
node_1024: feature_name=CD79A	CD79A.value > threshold=0.1879405975341797
node_1028: feature_name=NR4A1	NR4A1.value > threshold=0.7387348711490631
node_1086: feature_name=WDR74	WDR74.value <= threshold=-0.501454085111618
node_1087: feature_name=TNFAIP3	TNFAIP3.value > threshold=-1.082760363817215
Class: COVID-19 Severe	
Rules_1025	passed counts:4
node_0: feature_name=LTB	LTB.value <= threshold=0.994031548500061
node_1: feature_name=MTRNR2L2	MTRNR2L2.value <= threshold=2.8784297704696655
node_2: feature_name=TCL1A	TCL1A.value <= threshold=4.798312664031982
node_3: feature_name=MT-CO1	MT-CO1.value <= threshold=0.08947191759943962
node_4: feature_name=NR4A1	NR4A1.value <= threshold=1.0725348591804504
node_5: feature_name=HLA-DPA1	HLA-DPA1.value > threshold=1.6885469555854797
node_861: feature_name=CD83	CD83.value <= threshold=3.0563642978668213
node_862: feature_name=HLA-DQB1	HLA-DQB1.value <= threshold=2.61081063747406
node_863: feature_name=MTRNR2L2	MTRNR2L2.value <= threshold=2.0919997692108154
node_864: feature_name=BCL2A1	BCL2A1.value <= threshold=1.852618932723999
node_865: feature_name=CD79B	CD79B.value <= threshold=4.352142333984375
node_866: feature_name=TNFAIP3	TNFAIP3.value <= threshold=-0.0449496041983366
node_867: feature_name=MT-CO1	MT-CO1.value <= threshold=-0.08531957119703293
node_868: feature_name=MTRNR2L8	MTRNR2L8.value <= threshold=0.8770350813865662
node_869: feature_name=EGR1	EGR1.value > threshold=-0.32270750403404236
node_1015: feature_name=NR4A3	NR4A3.value <= threshold=1.9085431098937988
node_1016: feature_name=ID3	ID3.value <= threshold=2.776647686958313
node_1017: feature_name=XIST	XIST.value <= threshold=2.618046522140503

node_1018: feature_name=CD79B	CD79B.value <= threshold=3.818994164466858
node_1019: feature_name=NFKBID	NFKBID.value <= threshold=3.4191306829452515
node_1020: feature_name=MIAT	MIAT.value <= threshold=3.532225251197815
node_1021: feature_name=STAG3	STAG3.value <= threshold=9.534917831420898
node_1022: feature_name=OASL	OASL.value <= threshold=2.3923760652542114
node_1023: feature_name=HLA-DQB1	HLA-DQB1.value <= threshold=2.572031259536743
node_1024: feature_name=CD79A	CD79A.value > threshold=0.1879405975341797
node_1028: feature_name=NR4A1	NR4A1.value <= threshold=0.7387348711490631
node_1029: feature_name=SLC7A5	SLC7A5.value <= threshold=2.0677570104599
node_1030: feature_name=HLA-DPB1	HLA-DPB1.value > threshold=2.17708420753479
node_1068: feature_name=FAM129C	FAM129C.value > threshold=1.734559953212738
node_1072: feature_name=PRDM1	PRDM1.value > threshold=-0.4598921239376068
node_1078: feature_name=RNF11	RNF11.value > threshold=-0.5759164690971375
Class: Healthy Control	
Rules_1026	passed counts:4
node_0: feature_name=LTB	LTB.value <= threshold=0.994031548500061
node_1: feature_name=MTRNR2L2	MTRNR2L2.value <= threshold=2.8784297704696655
node_2: feature_name=TCL1A	TCL1A.value <= threshold=4.798312664031982
node_3: feature_name=MT-CO1	MT-CO1.value <= threshold=0.08947191759943962
node_4: feature_name=NR4A1	NR4A1.value <= threshold=1.0725348591804504
node_5: feature_name=HLA-DPA1	HLA-DPA1.value > threshold=1.6885469555854797
node_861: feature_name=CD83	CD83.value <= threshold=3.0563642978668213
node_862: feature_name=HLA-DQB1	HLA-DQB1.value <= threshold=2.61081063747406
node_863: feature_name=MTRNR2L2	MTRNR2L2.value <= threshold=2.0919997692108154
node_864: feature_name=BCL2A1	BCL2A1.value <= threshold=1.852618932723999
node_865: feature_name=CD79B	CD79B.value <= threshold=4.352142333984375
node_866: feature_name=TNFAIP3	TNFAIP3.value <= threshold=-0.0449496041983366
node_867: feature_name=MT-CO1	MT-CO1.value <= threshold=-0.08531957119703293
node_868: feature_name=MTRNR2L8	MTRNR2L8.value <= threshold=0.8770350813865662

node_869: feature_name=EGR1	EGR1.value > threshold=-0.32270750403404236
node_1015: feature_name=NR4A3	NR4A3.value <= threshold=1.9085431098937988
node_1016: feature_name=ID3	ID3.value <= threshold=2.776647686958313
node_1017: feature_name=XIST	XIST.value <= threshold=2.618046522140503
node_1018: feature_name=CD79B	CD79B.value <= threshold=3.818994164466858
node_1019: feature_name=NFKBID	NFKBID.value <= threshold=3.4191306829452515
node_1020: feature_name=MIAT	MIAT.value <= threshold=3.532225251197815
node_1021: feature_name=STAG3	STAG3.value <= threshold=9.534917831420898
node_1022: feature_name=OASL	OASL.value <= threshold=2.3923760652542114
node_1023: feature_name=HLA-DQB1	HLA-DQB1.value <= threshold=2.572031259536743
node_1024: feature_name=CD79A	CD79A.value > threshold=0.1879405975341797
node_1028: feature_name=NR4A1	NR4A1.value <= threshold=0.7387348711490631
node_1029: feature_name=SLC7A5	SLC7A5.value <= threshold=2.0677570104599
node_1030: feature_name=HLA-DPB1	HLA-DPB1.value > threshold=2.17708420753479
node_1068: feature_name=FAM129C	FAM129C.value > threshold=1.734559953212738
node_1072: feature_name=PRDM1	PRDM1.value <= threshold=-0.4598921239376068
node_1073: feature_name=RGS1	RGS1.value <= threshold=0.8619816601276398
node_1074: feature_name=SPI1	SPI1.value <= threshold=-0.004909217357635498
Class: COVID-19 Severe	
Rules_1027	passed counts:4
node_0: feature_name=LTB	LTB.value <= threshold=0.994031548500061
node_1: feature_name=MTRNR2L2	MTRNR2L2.value <= threshold=2.8784297704696655
node_2: feature_name=TCL1A	TCL1A.value <= threshold=4.798312664031982
node_3: feature_name=MT-CO1	MT-CO1.value <= threshold=0.08947191759943962
node_4: feature_name=NR4A1	NR4A1.value <= threshold=1.0725348591804504
node_5: feature_name=HLA-DPA1	HLA-DPA1.value > threshold=1.6885469555854797
node_861: feature_name=CD83	CD83.value <= threshold=3.0563642978668213
node_862: feature_name=HLA-DQB1	HLA-DQB1.value <= threshold=2.61081063747406
node_863: feature_name=MTRNR2L2	MTRNR2L2.value <= threshold=2.0919997692108154

node_864: feature_name=BCL2A1	BCL2A1.value <= threshold=1.852618932723999
node_865: feature_name=CD79B	CD79B.value <= threshold=4.352142333984375
node_866: feature_name=TNFAIP3	TNFAIP3.value <= threshold=-0.0449496041983366
node_867: feature_name=MT-CO1	MT-CO1.value <= threshold=-0.08531957119703293
node_868: feature_name=MTRNR2L8	MTRNR2L8.value <= threshold=0.8770350813865662
node_869: feature_name=EGR1	EGR1.value > threshold=-0.32270750403404236
node_1015: feature_name=NR4A3	NR4A3.value <= threshold=1.9085431098937988
node_1016: feature_name=ID3	ID3.value <= threshold=2.776647686958313
node_1017: feature_name=XIST	XIST.value <= threshold=2.618046522140503
node_1018: feature_name=CD79B	CD79B.value <= threshold=3.818994164466858
node_1019: feature_name=NFKBID	NFKBID.value <= threshold=3.4191306829452515
node_1020: feature_name=MIAT	MIAT.value <= threshold=3.532225251197815
node_1021: feature_name=STAG3	STAG3.value <= threshold=9.534917831420898
node_1022: feature_name=OASL	OASL.value <= threshold=2.3923760652542114
node_1023: feature_name=HLA-DQB1	HLA-DQB1.value <= threshold=2.572031259536743
node_1024: feature_name=CD79A	CD79A.value > threshold=0.1879405975341797
node_1028: feature_name=NR4A1	NR4A1.value <= threshold=0.7387348711490631
node_1029: feature_name=SLC7A5	SLC7A5.value <= threshold=2.0677570104599
node_1030: feature_name=HLA-DPB1	HLA-DPB1.value <= threshold=2.17708420753479
node_1031: feature_name=MARCH1	MARCH1.value <= threshold=4.443183898925781
node_1032: feature_name=ATF5	ATF5.value <= threshold=6.411617755889893
node_1033: feature_name=MYL9	MYL9.value <= threshold=3.8704605102539062
node_1034: feature_name=LTB	LTB.value <= threshold=0.9670123755931854
node_1035: feature_name=PLD4	PLD4.value <= threshold=7.310958623886108
node_1036: feature_name=CTD-2035E11.3	CTD-2035E11.3.value <= threshold=2.9847536087036133
node_1037: feature_name=PDLIM1	PDLIM1.value > threshold=-0.278710201382637
node_1051: feature_name=MTRNR2L2	MTRNR2L2.value > threshold=1.1439209580421448
node_1053: feature_name=AHR	AHR.value > threshold=-0.4576416164636612
Class: COVID-19 Critical	

Rules_1028	passed counts:4
node_0: feature_name=LTB	LTB.value <= threshold=0.994031548500061
node_1: feature_name=MTRNR2L2	MTRNR2L2.value <= threshold=2.8784297704696655
node_2: feature_name=TCL1A	TCL1A.value <= threshold=4.798312664031982
node_3: feature_name=MT-CO1	MT-CO1.value <= threshold=0.08947191759943962
node_4: feature_name=NR4A1	NR4A1.value <= threshold=1.0725348591804504
node_5: feature_name=HLA-DPA1	HLA-DPA1.value > threshold=1.6885469555854797
node_861: feature_name=CD83	CD83.value <= threshold=3.0563642978668213
node_862: feature_name=HLA-DQB1	HLA-DQB1.value <= threshold=2.61081063747406
node_863: feature_name=MTRNR2L2	MTRNR2L2.value <= threshold=2.0919997692108154
node_864: feature_name=BCL2A1	BCL2A1.value <= threshold=1.852618932723999
node_865: feature_name=CD79B	CD79B.value <= threshold=4.352142333984375
node_866: feature_name=TNFAIP3	TNFAIP3.value <= threshold=-0.0449496041983366
node_867: feature_name=MT-CO1	MT-CO1.value <= threshold=-0.08531957119703293
node_868: feature_name=MTRNR2L8	MTRNR2L8.value <= threshold=0.8770350813865662
node_869: feature_name=EGR1	EGR1.value <= threshold=-0.32270750403404236
node_870: feature_name=IGJ	IGJ.value <= threshold=4.618318557739258
node_871: feature_name=DUSP4	DUSP4.value > threshold=2.8197922706604004
node_1011: feature_name=TP53INP1	TP53INP1.value <= threshold=-0.26541395485401154
Class: COVID-19 Severe	
Rules_1029	passed counts:4
node_0: feature_name=LTB	LTB.value <= threshold=0.994031548500061
node_1: feature_name=MTRNR2L2	MTRNR2L2.value <= threshold=2.8784297704696655
node_2: feature_name=TCL1A	TCL1A.value <= threshold=4.798312664031982
node_3: feature_name=MT-CO1	MT-CO1.value <= threshold=0.08947191759943962
node_4: feature_name=NR4A1	NR4A1.value <= threshold=1.0725348591804504
node_5: feature_name=HLA-DPA1	HLA-DPA1.value > threshold=1.6885469555854797
node_861: feature_name=CD83	CD83.value <= threshold=3.0563642978668213
node_862: feature_name=HLA-DQB1	HLA-DQB1.value <= threshold=2.61081063747406

node_863: feature_name=MTRNR2L2	MTRNR2L2.value <= threshold=2.0919997692108154
node_864: feature_name=BCL2A1	BCL2A1.value <= threshold=1.852618932723999
node_865: feature_name=CD79B	CD79B.value <= threshold=4.352142333984375
node_866: feature_name=TNFAIP3	TNFAIP3.value <= threshold=-0.0449496041983366
node_867: feature_name=MT-CO1	MT-CO1.value <= threshold=-0.08531957119703293
node_868: feature_name=MTRNR2L8	MTRNR2L8.value <= threshold=0.8770350813865662
node_869: feature_name=EGR1	EGR1.value <= threshold=-0.32270750403404236
node_870: feature_name=IGJ	IGJ.value <= threshold=4.618318557739258
node_871: feature_name=DUSP4	DUSP4.value <= threshold=2.8197922706604004
node_872: feature_name=HOPX	HOPX.value > threshold=2.10397732257843
node_1008: feature_name=MT-ND6	MT-ND6.value > threshold=-0.9622937142848969
Class: COVID-19 Critical	
Rules_1030	passed counts:4
node_0: feature_name=LTB	LTB.value <= threshold=0.994031548500061
node_1: feature_name=MTRNR2L2	MTRNR2L2.value <= threshold=2.8784297704696655
node_2: feature_name=TCL1A	TCL1A.value <= threshold=4.798312664031982
node_3: feature_name=MT-CO1	MT-CO1.value <= threshold=0.08947191759943962
node_4: feature_name=NR4A1	NR4A1.value <= threshold=1.0725348591804504
node_5: feature_name=HLA-DPA1	HLA-DPA1.value > threshold=1.6885469555854797
node_861: feature_name=CD83	CD83.value <= threshold=3.0563642978668213
node_862: feature_name=HLA-DQB1	HLA-DQB1.value <= threshold=2.61081063747406
node_863: feature_name=MTRNR2L2	MTRNR2L2.value <= threshold=2.0919997692108154
node_864: feature_name=BCL2A1	BCL2A1.value <= threshold=1.852618932723999
node_865: feature_name=CD79B	CD79B.value <= threshold=4.352142333984375
node_866: feature_name=TNFAIP3	TNFAIP3.value <= threshold=-0.0449496041983366
node_867: feature_name=MT-CO1	MT-CO1.value <= threshold=-0.08531957119703293
node_868: feature_name=MTRNR2L8	MTRNR2L8.value <= threshold=0.8770350813865662
node_869: feature_name=EGR1	EGR1.value <= threshold=-0.32270750403404236
node_870: feature_name=IGJ	IGJ.value <= threshold=4.618318557739258

node_871: feature_name=DUSP4	DUSP4.value <= threshold=2.8197922706604004
node_872: feature_name=HOPX	HOPX.value > threshold=2.10397732257843
node_1008: feature_name=MT-ND6	MT-ND6.value <= threshold=-0.9622937142848969
Class: COVID-19 Severe	
Rules_1031	passed counts:4
node_0: feature_name=LTB	LTB.value <= threshold=0.994031548500061
node_1: feature_name=MTRNR2L2	MTRNR2L2.value <= threshold=2.8784297704696655
node_2: feature_name=TCL1A	TCL1A.value <= threshold=4.798312664031982
node_3: feature_name=MT-CO1	MT-CO1.value <= threshold=0.08947191759943962
node_4: feature_name=NR4A1	NR4A1.value <= threshold=1.0725348591804504
node_5: feature_name=HLA-DPA1	HLA-DPA1.value > threshold=1.6885469555854797
node_861: feature_name=CD83	CD83.value <= threshold=3.0563642978668213
node_862: feature_name=HLA-DQB1	HLA-DQB1.value <= threshold=2.61081063747406
node_863: feature_name=MTRNR2L2	MTRNR2L2.value <= threshold=2.0919997692108154
node_864: feature_name=BCL2A1	BCL2A1.value <= threshold=1.852618932723999
node_865: feature_name=CD79B	CD79B.value <= threshold=4.352142333984375
node_866: feature_name=TNFAIP3	TNFAIP3.value <= threshold=-0.0449496041983366
node_867: feature_name=MT-CO1	MT-CO1.value <= threshold=-0.08531957119703293
node_868: feature_name=MTRNR2L8	MTRNR2L8.value <= threshold=0.8770350813865662
node_869: feature_name=EGR1	EGR1.value <= threshold=-0.32270750403404236
node_870: feature_name=IGJ	IGJ.value <= threshold=4.618318557739258
node_871: feature_name=DUSP4	DUSP4.value <= threshold=2.8197922706604004
node_872: feature_name=HOPX	HOPX.value <= threshold=2.10397732257843
node_873: feature_name=RGCC	RGCC.value <= threshold=1.2070720195770264
node_874: feature_name=OAZ1	OAZ1.value > threshold=1.02116060256958
node_980: feature_name=IFIT3	IFIT3.value > threshold=-0.2093840092420578
node_986: feature_name=ID1	ID1.value <= threshold=0.9667705297470093
node_987: feature_name=HLA-DQB1	HLA-DQB1.value > threshold=-0.5031426399946213
node_989: feature_name=BANK1	BANK1.value > threshold=4.076449275016785

node_997: feature_name=PDLIM1	PDLIM1.value > threshold=-0.24925334006547928
Class: COVID-19 Critical	
Rules_1032	passed counts:4
node_0: feature_name=LTB	LTB.value <= threshold=0.994031548500061
node_1: feature_name=MTRNR2L2	MTRNR2L2.value <= threshold=2.8784297704696655
node_2: feature_name=TCL1A	TCL1A.value <= threshold=4.798312664031982
node_3: feature_name=MT-CO1	MT-CO1.value <= threshold=0.08947191759943962
node_4: feature_name=NR4A1	NR4A1.value <= threshold=1.0725348591804504
node_5: feature_name=HLA-DPA1	HLA-DPA1.value > threshold=1.6885469555854797
node_861: feature_name=CD83	CD83.value <= threshold=3.0563642978668213
node_862: feature_name=HLA-DQB1	HLA-DQB1.value <= threshold=2.61081063747406
node_863: feature_name=MTRNR2L2	MTRNR2L2.value <= threshold=2.0919997692108154
node_864: feature_name=BCL2A1	BCL2A1.value <= threshold=1.852618932723999
node_865: feature_name=CD79B	CD79B.value <= threshold=4.352142333984375
node_866: feature_name=TNFAIP3	TNFAIP3.value <= threshold=-0.0449496041983366
node_867: feature_name=MT-CO1	MT-CO1.value <= threshold=-0.08531957119703293
node_868: feature_name=MTRNR2L8	MTRNR2L8.value <= threshold=0.8770350813865662
node_869: feature_name=EGR1	EGR1.value <= threshold=-0.32270750403404236
node_870: feature_name=IGJ	IGJ.value <= threshold=4.618318557739258
node_871: feature_name=DUSP4	DUSP4.value <= threshold=2.8197922706604004
node_872: feature_name=HOPX	HOPX.value <= threshold=2.10397732257843
node_873: feature_name=RGCC	RGCC.value <= threshold=1.2070720195770264
node_874: feature_name=OAZ1	OAZ1.value <= threshold=1.02116060256958
node_875: feature_name=NAIP	NAIP.value <= threshold=3.768904685974121
node_876: feature_name=LDLR	LDLR.value <= threshold=2.9582698345184326
node_877: feature_name=HLA-DPA1	HLA-DPA1.value <= threshold=3.0588693618774414
node_878: feature_name=RHOB	RHOB.value <= threshold=5.08755898475647
node_879: feature_name=SYTL3	SYTL3.value <= threshold=1.5127440690994263
node_880: feature_name=FTH1	FTH1.value > threshold=-1.4152275919914246

node_882: feature_name=CCL3	CCL3.value <= threshold=2.253131687641144
node_883: feature_name=CD83	CD83.value > threshold=2.8566592931747437
node_967: feature_name=MT-CO3	MT-CO3.value <= threshold=-0.9992981255054474
node_968: feature_name=MAP3K2	MAP3K2.value <= threshold=0.7086937576532364
Class: COVID-19 Severe	
Rules_1033	passed counts:4
node_0: feature_name=LTB	LTB.value <= threshold=0.994031548500061
node_1: feature_name=MTRNR2L2	MTRNR2L2.value <= threshold=2.8784297704696655
node_2: feature_name=TCL1A	TCL1A.value <= threshold=4.798312664031982
node_3: feature_name=MT-CO1	MT-CO1.value <= threshold=0.08947191759943962
node_4: feature_name=NR4A1	NR4A1.value <= threshold=1.0725348591804504
node_5: feature_name=HLA-DPA1	HLA-DPA1.value > threshold=1.6885469555854797
node_861: feature_name=CD83	CD83.value <= threshold=3.0563642978668213
node_862: feature_name=HLA-DQB1	HLA-DQB1.value <= threshold=2.61081063747406
node_863: feature_name=MTRNR2L2	MTRNR2L2.value <= threshold=2.0919997692108154
node_864: feature_name=BCL2A1	BCL2A1.value <= threshold=1.852618932723999
node_865: feature_name=CD79B	CD79B.value <= threshold=4.352142333984375
node_866: feature_name=TNFAIP3	TNFAIP3.value <= threshold=-0.0449496041983366
node_867: feature_name=MT-CO1	MT-CO1.value <= threshold=-0.08531957119703293
node_868: feature_name=MTRNR2L8	MTRNR2L8.value <= threshold=0.8770350813865662
node_869: feature_name=EGR1	EGR1.value <= threshold=-0.32270750403404236
node_870: feature_name=IGJ	IGJ.value <= threshold=4.618318557739258
node_871: feature_name=DUSP4	DUSP4.value <= threshold=2.8197922706604004
node_872: feature_name=HOPX	HOPX.value <= threshold=2.10397732257843
node_873: feature_name=RGCC	RGCC.value <= threshold=1.2070720195770264
node_874: feature_name=OAZ1	OAZ1.value <= threshold=1.02116060256958
node_875: feature_name=NAIP	NAIP.value <= threshold=3.768904685974121
node_876: feature_name=LDLR	LDLR.value <= threshold=2.9582698345184326
node_877: feature_name=HLA-DPA1	HLA-DPA1.value <= threshold=3.0588693618774414

node_878: feature_name=RHOB	RHOB.value <= threshold=5.08755898475647
node_879: feature_name=SYTL3	SYTL3.value <= threshold=1.5127440690994263
node_880: feature_name=FTH1	FTH1.value > threshold=-1.4152275919914246
node_882: feature_name=CCL3	CCL3.value <= threshold=2.253131687641144
node_883: feature_name=CD83	CD83.value <= threshold=2.8566592931747437
node_884: feature_name=HLA-DRB5	HLA-DRB5.value > threshold=-0.6464254856109619
node_888: feature_name=HSP90B1	HSP90B1.value > threshold=-1.186358630657196
node_892: feature_name=ATG2A	ATG2A.value <= threshold=2.3624722957611084
node_893: feature_name=ICAM1	ICAM1.value <= threshold=2.4306468963623047
node_894: feature_name=CD38	CD38.value <= threshold=3.4977954626083374
node_895: feature_name=HLA-DRB1	HLA-DRB1.value > threshold=0.28603747487068176
node_899: feature_name=XIST	XIST.value <= threshold=1.4272559583187103
node_900: feature_name=BANK1	BANK1.value <= threshold=5.060973644256592
node_901: feature_name=IRF4	IRF4.value <= threshold=6.117950916290283
node_902: feature_name=MTRNR2L2	MTRNR2L2.value <= threshold=1.8410876989364624
node_903: feature_name=TPM1	TPM1.value <= threshold=4.590091228485107
node_904: feature_name=SNORD3B-2	SNORD3B-2.value <= threshold=4.349201917648315
node_905: feature_name=AHR	AHR.value > threshold=2.268427610397339
node_927: feature_name=FAM129C	FAM129C.value > threshold=1.2436291128396988
Class: COVID-19 Critical	
Rules_1034	passed counts:4
node_0: feature_name=LTB	LTB.value <= threshold=0.994031548500061
node_1: feature_name=MTRNR2L2	MTRNR2L2.value <= threshold=2.8784297704696655
node_2: feature_name=TCL1A	TCL1A.value <= threshold=4.798312664031982
node_3: feature_name=MT-CO1	MT-CO1.value <= threshold=0.08947191759943962
node_4: feature_name=NR4A1	NR4A1.value <= threshold=1.0725348591804504
node_5: feature_name=HLA-DPA1	HLA-DPA1.value > threshold=1.6885469555854797
node_861: feature_name=CD83	CD83.value <= threshold=3.0563642978668213
node_862: feature_name=HLA-DQB1	HLA-DQB1.value <= threshold=2.61081063747406

node_863: feature_name=MTRNR2L2	MTRNR2L2.value <= threshold=2.0919997692108154
node_864: feature_name=BCL2A1	BCL2A1.value <= threshold=1.852618932723999
node_865: feature_name=CD79B	CD79B.value <= threshold=4.352142333984375
node_866: feature_name=TNFAIP3	TNFAIP3.value <= threshold=-0.0449496041983366
node_867: feature_name=MT-CO1	MT-CO1.value <= threshold=-0.08531957119703293
node_868: feature_name=MTRNR2L8	MTRNR2L8.value <= threshold=0.8770350813865662
node_869: feature_name=EGR1	EGR1.value <= threshold=-0.32270750403404236
node_870: feature_name=IGJ	IGJ.value <= threshold=4.618318557739258
node_871: feature_name=DUSP4	DUSP4.value <= threshold=2.8197922706604004
node_872: feature_name=HOPX	HOPX.value <= threshold=2.10397732257843
node_873: feature_name=RGCC	RGCC.value <= threshold=1.2070720195770264
node_874: feature_name=OAZ1	OAZ1.value <= threshold=1.02116060256958
node_875: feature_name=NAIP	NAIP.value <= threshold=3.768904685974121
node_876: feature_name=LDLR	LDLR.value <= threshold=2.9582698345184326
node_877: feature_name=HLA-DPA1	HLA-DPA1.value <= threshold=3.0588693618774414
node_878: feature_name=RHOB	RHOB.value <= threshold=5.08755898475647
node_879: feature_name=SYTL3	SYTL3.value <= threshold=1.5127440690994263
node_880: feature_name=FTH1	FTH1.value > threshold=-1.4152275919914246
node_882: feature_name=CCL3	CCL3.value <= threshold=2.253131687641144
node_883: feature_name=CD83	CD83.value <= threshold=2.8566592931747437
node_884: feature_name=HLA-DRB5	HLA-DRB5.value > threshold=-0.6464254856109619
node_888: feature_name=HSP90B1	HSP90B1.value > threshold=-1.186358630657196
node_892: feature_name=ATG2A	ATG2A.value <= threshold=2.3624722957611084
node_893: feature_name=ICAM1	ICAM1.value <= threshold=2.4306468963623047
node_894: feature_name=CD38	CD38.value <= threshold=3.4977954626083374
node_895: feature_name=HLA-DRB1	HLA-DRB1.value > threshold=0.28603747487068176
node_899: feature_name=XIST	XIST.value <= threshold=1.4272559583187103
node_900: feature_name=BANK1	BANK1.value <= threshold=5.060973644256592
node_901: feature_name=IRF4	IRF4.value <= threshold=6.117950916290283
node_902: feature_name=MTRNR2L2	MTRNR2L2.value <= threshold=1.8410876989364624

node_903: feature_name=TPM1	TPM1.value <= threshold=4.590091228485107
node_904: feature_name=SNORD3B-2	SNORD3B-2.value <= threshold=4.349201917648315
node_905: feature_name=AHR	AHR.value <= threshold=2.268427610397339
node_906: feature_name=OAZ1	OAZ1.value <= threshold=-1.4369714260101318
node_907: feature_name=PELI1	PELI1.value > threshold=1.937936544418335
node_911: feature_name=MTRNR2L2	MTRNR2L2.value > threshold=-0.2940830886363983
Class: COVID-19 Critical	
Rules_1035	passed counts:4
node_0: feature_name=LTB	LTB.value <= threshold=0.994031548500061
node_1: feature_name=MTRNR2L2	MTRNR2L2.value <= threshold=2.8784297704696655
node_2: feature_name=TCL1A	TCL1A.value <= threshold=4.798312664031982
node_3: feature_name=MT-CO1	MT-CO1.value <= threshold=0.08947191759943962
node_4: feature_name=NR4A1	NR4A1.value <= threshold=1.0725348591804504
node_5: feature_name=HLA-DPA1	HLA-DPA1.value > threshold=1.6885469555854797
node_861: feature_name=CD83	CD83.value <= threshold=3.0563642978668213
node_862: feature_name=HLA-DQB1	HLA-DQB1.value <= threshold=2.61081063747406
node_863: feature_name=MTRNR2L2	MTRNR2L2.value <= threshold=2.0919997692108154
node_864: feature_name=BCL2A1	BCL2A1.value <= threshold=1.852618932723999
node_865: feature_name=CD79B	CD79B.value <= threshold=4.352142333984375
node_866: feature_name=TNFAIP3	TNFAIP3.value <= threshold=-0.0449496041983366
node_867: feature_name=MT-CO1	MT-CO1.value <= threshold=-0.08531957119703293
node_868: feature_name=MTRNR2L8	MTRNR2L8.value <= threshold=0.8770350813865662
node_869: feature_name=EGR1	EGR1.value <= threshold=-0.32270750403404236
node_870: feature_name=IGJ	IGJ.value <= threshold=4.618318557739258
node_871: feature_name=DUSP4	DUSP4.value <= threshold=2.8197922706604004
node_872: feature_name=HOPX	HOPX.value <= threshold=2.10397732257843
node_873: feature_name=RGCC	RGCC.value <= threshold=1.2070720195770264
node_874: feature_name=OAZ1	OAZ1.value <= threshold=1.02116060256958
node_875: feature_name=NAIP	NAIP.value <= threshold=3.768904685974121

node_876: feature_name=LDLR	LDLR.value <= threshold=2.9582698345184326
node_877: feature_name=HLA-DPA1	HLA-DPA1.value <= threshold=3.0588693618774414
node_878: feature_name=RHOB	RHOB.value <= threshold=5.08755898475647
node_879: feature_name=SYTL3	SYTL3.value <= threshold=1.5127440690994263
node_880: feature_name=FTH1	FTH1.value > threshold=-1.4152275919914246
node_882: feature_name=CCL3	CCL3.value <= threshold=2.253131687641144
node_883: feature_name=CD83	CD83.value <= threshold=2.8566592931747437
node_884: feature_name=HLA-DRB5	HLA-DRB5.value > threshold=-0.6464254856109619
node_888: feature_name=HSP90B1	HSP90B1.value > threshold=-1.186358630657196
node_892: feature_name=ATG2A	ATG2A.value <= threshold=2.3624722957611084
node_893: feature_name=ICAM1	ICAM1.value <= threshold=2.4306468963623047
node_894: feature_name=CD38	CD38.value <= threshold=3.4977954626083374
node_895: feature_name=HLA-DRB1	HLA-DRB1.value <= threshold=0.28603747487068176
node_896: feature_name=PTGS1	PTGS1.value > threshold=-0.13729806244373322
Class: COVID-19 Critical	
Rules_1036	passed counts:4
node_0: feature_name=LTB	LTB.value <= threshold=0.994031548500061
node_1: feature_name=MTRNR2L2	MTRNR2L2.value <= threshold=2.8784297704696655
node_2: feature_name=TCL1A	TCL1A.value <= threshold=4.798312664031982
node_3: feature_name=MT-CO1	MT-CO1.value <= threshold=0.08947191759943962
node_4: feature_name=NR4A1	NR4A1.value <= threshold=1.0725348591804504
node_5: feature_name=HLA-DPA1	HLA-DPA1.value <= threshold=1.6885469555854797
node_6: feature_name=HLA-DRB5	HLA-DRB5.value > threshold=2.1886194944381714
node_738: feature_name=HLA-DQA2	HLA-DQA2.value > threshold=-0.20105575770139694
node_804: feature_name=CYBB	CYBB.value > threshold=2.9090086221694946
node_858: feature_name=GRASP	GRASP.value > threshold=-0.3890708088874817
Class: COVID-19 Severe	
Rules_1037	passed counts:4

node_0: feature_name=LTB	LTB.value <= threshold=0.994031548500061
node_1: feature_name=MTRNR2L2	MTRNR2L2.value <= threshold=2.8784297704696655
node_2: feature_name=TCL1A	TCL1A.value <= threshold=4.798312664031982
node_3: feature_name=MT-CO1	MT-CO1.value <= threshold=0.08947191759943962
node_4: feature_name=NR4A1	NR4A1.value <= threshold=1.0725348591804504
node_5: feature_name=HLA-DPA1	HLA-DPA1.value <= threshold=1.6885469555854797
node_6: feature_name=HLA-DRB5	HLA-DRB5.value > threshold=2.1886194944381714
node_738: feature_name=HLA-DQA2	HLA-DQA2.value > threshold=-0.20105575770139694
node_804: feature_name=CYBB	CYBB.value <= threshold=2.9090086221694946
node_805: feature_name=VPREB3	VPREB3.value <= threshold=7.757248401641846
node_806: feature_name=IL8	IL8.value <= threshold=1.7155421376228333
node_807: feature_name=ATF5	ATF5.value <= threshold=6.124105930328369
node_808: feature_name=RP11-731F5.2	RP11-731F5.2.value <= threshold=7.633600234985352
node_809: feature_name=OASL	OASL.value <= threshold=0.9932635426521301
node_810: feature_name=ID1	ID1.value <= threshold=2.8093127012252808
node_811: feature_name=GRASP	GRASP.value > threshold=2.3934966325759888
node_837: feature_name=HLA-DQA2	HLA-DQA2.value <= threshold=-0.19460754096508026
Class: COVID-19 Severe	
Rules_1038	passed counts:4
node_0: feature_name=LTB	LTB.value <= threshold=0.994031548500061
node_1: feature_name=MTRNR2L2	MTRNR2L2.value <= threshold=2.8784297704696655
node_2: feature_name=TCL1A	TCL1A.value <= threshold=4.798312664031982
node_3: feature_name=MT-CO1	MT-CO1.value <= threshold=0.08947191759943962
node_4: feature_name=NR4A1	NR4A1.value <= threshold=1.0725348591804504
node_5: feature_name=HLA-DPA1	HLA-DPA1.value <= threshold=1.6885469555854797
node_6: feature_name=HLA-DRB5	HLA-DRB5.value > threshold=2.1886194944381714
node_738: feature_name=HLA-DQA2	HLA-DQA2.value <= threshold=-0.20105575770139694
node_739: feature_name=HLA-DQA1	HLA-DQA1.value > threshold=1.7414769530296326
node_763: feature_name=HLA-DRB5	HLA-DRB5.value > threshold=2.6777851581573486

node_799: feature_name=NFKBID	NFKBID.value > threshold=2.063578724861145
node_801: feature_name=NFKBIZ	NFKBIZ.value <= threshold=0.6845290064811707
Class: COVID-19 Critical	
Rules_1039	passed counts:4
node_0: feature_name=LTB	LTB.value <= threshold=0.994031548500061
node_1: feature_name=MTRNR2L2	MTRNR2L2.value <= threshold=2.8784297704696655
node_2: feature_name=TCL1A	TCL1A.value <= threshold=4.798312664031982
node_3: feature_name=MT-CO1	MT-CO1.value <= threshold=0.08947191759943962
node_4: feature_name=NR4A1	NR4A1.value <= threshold=1.0725348591804504
node_5: feature_name=HLA-DPA1	HLA-DPA1.value <= threshold=1.6885469555854797
node_6: feature_name=HLA-DRB5	HLA-DRB5.value <= threshold=2.1886194944381714
node_7: feature_name=XIST	XIST.value > threshold=-0.12328458577394485
node_711: feature_name=HLA-DQB1	HLA-DQB1.value > threshold=2.337415099143982
node_731: feature_name=OAZ1	OAZ1.value > threshold=0.6314055621623993
Class: COVID-19 Critical	
Rules_1040	passed counts:4
node_0: feature_name=LTB	LTB.value <= threshold=0.994031548500061
node_1: feature_name=MTRNR2L2	MTRNR2L2.value <= threshold=2.8784297704696655
node_2: feature_name=TCL1A	TCL1A.value <= threshold=4.798312664031982
node_3: feature_name=MT-CO1	MT-CO1.value <= threshold=0.08947191759943962
node_4: feature_name=NR4A1	NR4A1.value <= threshold=1.0725348591804504
node_5: feature_name=HLA-DPA1	HLA-DPA1.value <= threshold=1.6885469555854797
node_6: feature_name=HLA-DRB5	HLA-DRB5.value <= threshold=2.1886194944381714
node_7: feature_name=XIST	XIST.value <= threshold=-0.12328458577394485
node_8: feature_name=SYNE1	SYNE1.value > threshold=-0.7835594415664673
node_16: feature_name=MTRNR2L2	MTRNR2L2.value > threshold=2.4483247995376587
node_708: feature_name=ICAM1	ICAM1.value > threshold=-0.25065042823553085
Class: COVID-19 Critical	

Rules_1041	passed counts:4
node_0: feature_name=LTB	LTB.value <= threshold=0.994031548500061
node_1: feature_name=MTRNR2L2	MTRNR2L2.value <= threshold=2.8784297704696655
node_2: feature_name=TCL1A	TCL1A.value <= threshold=4.798312664031982
node_3: feature_name=MT-CO1	MT-CO1.value <= threshold=0.08947191759943962
node_4: feature_name=NR4A1	NR4A1.value <= threshold=1.0725348591804504
node_5: feature_name=HLA-DPA1	HLA-DPA1.value <= threshold=1.6885469555854797
node_6: feature_name=HLA-DRB5	HLA-DRB5.value <= threshold=2.1886194944381714
node_7: feature_name=XIST	XIST.value <= threshold=-0.12328458577394485
node_8: feature_name=SYNE1	SYNE1.value > threshold=-0.7835594415664673
node_16: feature_name=MTRNR2L2	MTRNR2L2.value <= threshold=2.4483247995376587
node_17: feature_name=HLA-DQA2	HLA-DQA2.value > threshold=2.227178931236267
node_591: feature_name=BCL2A1	BCL2A1.value <= threshold=1.0648428201675415
node_592: feature_name=FTH1	FTH1.value <= threshold=0.8842596709728241
node_593: feature_name=SNX9	SNX9.value <= threshold=3.232524275779724
node_594: feature_name=OAZ1	OAZ1.value <= threshold=1.704061508178711
node_595: feature_name=MT-CYB	MT-CYB.value <= threshold=0.5040059983730316
node_596: feature_name=CD83	CD83.value > threshold=2.535894751548767
node_638: feature_name=MTRNR2L8	MTRNR2L8.value <= threshold=1.581753134727478
node_639: feature_name=FCER2	FCER2.value > threshold=-0.253058984875679
node_643: feature_name=ID3	ID3.value <= threshold=2.6175442934036255
node_644: feature_name=PELI1	PELI1.value > threshold=2.353822112083435
node_650: feature_name=APP	APP.value > threshold=-0.3711509108543396
Class: COVID-19 Critical	
Rules_1042	passed counts:4
node_0: feature_name=LTB	LTB.value <= threshold=0.994031548500061
node_1: feature_name=MTRNR2L2	MTRNR2L2.value <= threshold=2.8784297704696655
node_2: feature_name=TCL1A	TCL1A.value <= threshold=4.798312664031982

node_3: feature_name=MT-CO1	MT-CO1.value <= threshold=0.08947191759943962
node_4: feature_name=NR4A1	NR4A1.value <= threshold=1.0725348591804504
node_5: feature_name=HLA-DPA1	HLA-DPA1.value <= threshold=1.6885469555854797
node_6: feature_name=HLA-DRB5	HLA-DRB5.value <= threshold=2.1886194944381714
node_7: feature_name=XIST	XIST.value <= threshold=-0.12328458577394485
node_8: feature_name=SYNE1	SYNE1.value > threshold=-0.7835594415664673
node_16: feature_name=MTRNR2L2	MTRNR2L2.value <= threshold=2.4483247995376587
node_17: feature_name=HLA-DQA2	HLA-DQA2.value > threshold=2.227178931236267
node_591: feature_name=BCL2A1	BCL2A1.value <= threshold=1.0648428201675415
node_592: feature_name=FTH1	FTH1.value <= threshold=0.8842596709728241
node_593: feature_name=SNX9	SNX9.value <= threshold=3.232524275779724
node_594: feature_name=OAZ1	OAZ1.value <= threshold=1.704061508178711
node_595: feature_name=MT-CYB	MT-CYB.value <= threshold=0.5040059983730316
node_596: feature_name=CD83	CD83.value > threshold=2.535894751548767
node_638: feature_name=MTRNR2L8	MTRNR2L8.value <= threshold=1.581753134727478
node_639: feature_name=FCER2	FCER2.value <= threshold=-0.253058984875679
node_640: feature_name=S100A6	S100A6.value <= threshold=0.20003187807742506
Class: COVID-19 Severe	
Rules_1043	passed counts:4
node_0: feature_name=LTB	LTB.value <= threshold=0.994031548500061
node_1: feature_name=MTRNR2L2	MTRNR2L2.value <= threshold=2.8784297704696655
node_2: feature_name=TCL1A	TCL1A.value <= threshold=4.798312664031982
node_3: feature_name=MT-CO1	MT-CO1.value <= threshold=0.08947191759943962
node_4: feature_name=NR4A1	NR4A1.value <= threshold=1.0725348591804504
node_5: feature_name=HLA-DPA1	HLA-DPA1.value <= threshold=1.6885469555854797
node_6: feature_name=HLA-DRB5	HLA-DRB5.value <= threshold=2.1886194944381714
node_7: feature_name=XIST	XIST.value <= threshold=-0.12328458577394485
node_8: feature_name=SYNE1	SYNE1.value > threshold=-0.7835594415664673
node_16: feature_name=MTRNR2L2	MTRNR2L2.value <= threshold=2.4483247995376587

node_17: feature_name=HLA-DQA2	HLA-DQA2.value <= threshold=2.227178931236267
node_18: feature_name=NR4A1	NR4A1.value > threshold=-0.21380723267793655
node_504: feature_name=LTB	LTB.value <= threshold=0.8758118152618408
node_505: feature_name=TNFAIP3	TNFAIP3.value <= threshold=0.4779366999864578
node_506: feature_name=HLA-DQB1	HLA-DQB1.value > threshold=2.9689468145370483
node_578: feature_name=CCDC141	CCDC141.value > threshold=-0.2039114013314247
Class: COVID-19 Severe	
Rules_1044	passed counts:4
node_0: feature_name=LTB	LTB.value <= threshold=0.994031548500061
node_1: feature_name=MTRNR2L2	MTRNR2L2.value <= threshold=2.8784297704696655
node_2: feature_name=TCL1A	TCL1A.value <= threshold=4.798312664031982
node_3: feature_name=MT-CO1	MT-CO1.value <= threshold=0.08947191759943962
node_4: feature_name=NR4A1	NR4A1.value <= threshold=1.0725348591804504
node_5: feature_name=HLA-DPA1	HLA-DPA1.value <= threshold=1.6885469555854797
node_6: feature_name=HLA-DRB5	HLA-DRB5.value <= threshold=2.1886194944381714
node_7: feature_name=XIST	XIST.value <= threshold=-0.12328458577394485
node_8: feature_name=SYNE1	SYNE1.value > threshold=-0.7835594415664673
node_16: feature_name=MTRNR2L2	MTRNR2L2.value <= threshold=2.4483247995376587
node_17: feature_name=HLA-DQA2	HLA-DQA2.value <= threshold=2.227178931236267
node_18: feature_name=NR4A1	NR4A1.value > threshold=-0.21380723267793655
node_504: feature_name=LTB	LTB.value <= threshold=0.8758118152618408
node_505: feature_name=TNFAIP3	TNFAIP3.value <= threshold=0.4779366999864578
node_506: feature_name=HLA-DQB1	HLA-DQB1.value <= threshold=2.9689468145370483
node_507: feature_name=MS4A1	MS4A1.value <= threshold=3.7428637742996216
node_508: feature_name=SNX9	SNX9.value > threshold=3.304862380027771
node_572: feature_name=CXorf57	CXorf57.value <= threshold=-0.20287973433732986
Class: COVID-19 Severe	
Rules_1045	passed counts:4

node_0: feature_name=LTB	LTB.value <= threshold=0.994031548500061
node_1: feature_name=MTRNR2L2	MTRNR2L2.value <= threshold=2.8784297704696655
node_2: feature_name=TCL1A	TCL1A.value <= threshold=4.798312664031982
node_3: feature_name=MT-CO1	MT-CO1.value <= threshold=0.08947191759943962
node_4: feature_name=NR4A1	NR4A1.value <= threshold=1.0725348591804504
node_5: feature_name=HLA-DPA1	HLA-DPA1.value <= threshold=1.6885469555854797
node_6: feature_name=HLA-DRB5	HLA-DRB5.value <= threshold=2.1886194944381714
node_7: feature_name=XIST	XIST.value <= threshold=-0.12328458577394485
node_8: feature_name=SYNE1	SYNE1.value > threshold=-0.7835594415664673
node_16: feature_name=MTRNR2L2	MTRNR2L2.value <= threshold=2.4483247995376587
node_17: feature_name=HLA-DQA2	HLA-DQA2.value <= threshold=2.227178931236267
node_18: feature_name=NR4A1	NR4A1.value > threshold=-0.21380723267793655
node_504: feature_name=LTB	LTB.value <= threshold=0.8758118152618408
node_505: feature_name=TNFAIP3	TNFAIP3.value <= threshold=0.4779366999864578
node_506: feature_name=HLA-DQB1	HLA-DQB1.value <= threshold=2.9689468145370483
node_507: feature_name=MS4A1	MS4A1.value <= threshold=3.7428637742996216
node_508: feature_name=SNX9	SNX9.value <= threshold=3.304862380027771
node_509: feature_name=MARCKS	MARCKS.value > threshold=-0.519626647233963
node_513: feature_name=CD79B	CD79B.value <= threshold=3.0890846252441406
node_514: feature_name=GNG11	GNG11.value <= threshold=3.3202054500579834
node_515: feature_name=BANK1	BANK1.value <= threshold=-0.3250492364168167
node_516: feature_name=MT-CO1	MT-CO1.value > threshold=-0.4365827441215515
node_518: feature_name=IL8	IL8.value > threshold=-0.3376134932041168
Class: COVID-19 Severe	
Rules_1046	passed counts:4
node_0: feature_name=LTB	LTB.value <= threshold=0.994031548500061
node_1: feature_name=MTRNR2L2	MTRNR2L2.value <= threshold=2.8784297704696655
node_2: feature_name=TCL1A	TCL1A.value <= threshold=4.798312664031982
node_3: feature_name=MT-CO1	MT-CO1.value <= threshold=0.08947191759943962

node_4: feature_name=NR4A1	NR4A1.value <= threshold=1.0725348591804504
node_5: feature_name=HLA-DPA1	HLA-DPA1.value <= threshold=1.6885469555854797
node_6: feature_name=HLA-DRB5	HLA-DRB5.value <= threshold=2.1886194944381714
node_7: feature_name=XIST	XIST.value <= threshold=-0.12328458577394485
node_8: feature_name=SYNE1	SYNE1.value > threshold=-0.7835594415664673
node_16: feature_name=MTRNR2L2	MTRNR2L2.value <= threshold=2.4483247995376587
node_17: feature_name=HLA-DQA2	HLA-DQA2.value <= threshold=2.227178931236267
node_18: feature_name=NR4A1	NR4A1.value <= threshold=-0.21380723267793655
node_19: feature_name=IRAK3	IRAK3.value <= threshold=4.27159571647644
node_20: feature_name=IFIT2	IFIT2.value <= threshold=7.8175740242004395
node_21: feature_name=CD79A	CD79A.value > threshold=2.857242226600647
node_375: feature_name=LTB	LTB.value <= threshold=0.7405790388584137
node_376: feature_name=NFKBIZ	NFKBIZ.value <= threshold=1.7718515396118164
node_377: feature_name=OAZ1	OAZ1.value > threshold=1.3302325010299683
node_465: feature_name=CD22	CD22.value > threshold=3.588951587677002
node_467: feature_name=PPIF	PPIF.value <= threshold=1.0877585858106613
Class: Healthy Control	
Rules_1047	passed counts:4
node_0: feature_name=LTB	LTB.value <= threshold=0.994031548500061
node_1: feature_name=MTRNR2L2	MTRNR2L2.value <= threshold=2.8784297704696655
node_2: feature_name=TCL1A	TCL1A.value <= threshold=4.798312664031982
node_3: feature_name=MT-CO1	MT-CO1.value <= threshold=0.08947191759943962
node_4: feature_name=NR4A1	NR4A1.value <= threshold=1.0725348591804504
node_5: feature_name=HLA-DPA1	HLA-DPA1.value <= threshold=1.6885469555854797
node_6: feature_name=HLA-DRB5	HLA-DRB5.value <= threshold=2.1886194944381714
node_7: feature_name=XIST	XIST.value <= threshold=-0.12328458577394485
node_8: feature_name=SYNE1	SYNE1.value > threshold=-0.7835594415664673
node_16: feature_name=MTRNR2L2	MTRNR2L2.value <= threshold=2.4483247995376587
node_17: feature_name=HLA-DQA2	HLA-DQA2.value <= threshold=2.227178931236267

node_18: feature_name=NR4A1	NR4A1.value <= threshold=-0.21380723267793655
node_19: feature_name=IRAK3	IRAK3.value <= threshold=4.27159571647644
node_20: feature_name=IFIT2	IFIT2.value <= threshold=7.8175740242004395
node_21: feature_name=CD79A	CD79A.value <= threshold=2.857242226600647
node_22: feature_name=HIST1H2AC	HIST1H2AC.value <= threshold=5.077470302581787
node_23: feature_name=FTH1	FTH1.value > threshold=0.4158056080341339
node_285: feature_name=EGR1	EGR1.value <= threshold=4.240467071533203
node_286: feature_name=HLA-DRB1	HLA-DRB1.value > threshold=-0.7553382813930511
node_290: feature_name=TLR2	TLR2.value > threshold=2.879140257835388
node_360: feature_name=HLA-DRB1	HLA-DRB1.value > threshold=1.289023995399475
Class: COVID-19 Severe	
Rules_1048	passed counts:4
node_0: feature_name=LTB	LTB.value <= threshold=0.994031548500061
node_1: feature_name=MTRNR2L2	MTRNR2L2.value <= threshold=2.8784297704696655
node_2: feature_name=TCL1A	TCL1A.value <= threshold=4.798312664031982
node_3: feature_name=MT-CO1	MT-CO1.value <= threshold=0.08947191759943962
node_4: feature_name=NR4A1	NR4A1.value <= threshold=1.0725348591804504
node_5: feature_name=HLA-DPA1	HLA-DPA1.value <= threshold=1.6885469555854797
node_6: feature_name=HLA-DRB5	HLA-DRB5.value <= threshold=2.1886194944381714
node_7: feature_name=XIST	XIST.value <= threshold=-0.12328458577394485
node_8: feature_name=SYNE1	SYNE1.value > threshold=-0.7835594415664673
node_16: feature_name=MTRNR2L2	MTRNR2L2.value <= threshold=2.4483247995376587
node_17: feature_name=HLA-DQA2	HLA-DQA2.value <= threshold=2.227178931236267
node_18: feature_name=NR4A1	NR4A1.value <= threshold=-0.21380723267793655
node_19: feature_name=IRAK3	IRAK3.value <= threshold=4.27159571647644
node_20: feature_name=IFIT2	IFIT2.value <= threshold=7.8175740242004395
node_21: feature_name=CD79A	CD79A.value <= threshold=2.857242226600647
node_22: feature_name=HIST1H2AC	HIST1H2AC.value <= threshold=5.077470302581787
node_23: feature_name=FTH1	FTH1.value > threshold=0.4158056080341339

node_285: feature_name=EGR1	EGR1.value <= threshold=4.240467071533203
node_286: feature_name=HLA-DRB1	HLA-DRB1.value > threshold=-0.7553382813930511
node_290: feature_name=TLR2	TLR2.value <= threshold=2.879140257835388
node_291: feature_name=SYNE1	SYNE1.value <= threshold=2.323199510574341
node_292: feature_name=BCL2A1	BCL2A1.value <= threshold=1.8940600156784058
node_293: feature_name=RALGPS2	RALGPS2.value <= threshold=4.808189153671265
node_294: feature_name=IGLL5	IGLL5.value <= threshold=4.116017818450928
node_295: feature_name=FTH1	FTH1.value > threshold=0.41612209379673004
node_297: feature_name=MCTP1	MCTP1.value <= threshold=4.601342678070068
node_298: feature_name=MT-CO2	MT-CO2.value > threshold=0.6201252341270447
node_342: feature_name=TCF4	TCF4.value <= threshold=-0.24748335778713226
Class: COVID-19 Severe	
Rules_1049	passed counts:4
node_0: feature_name=LTB	LTB.value <= threshold=0.994031548500061
node_1: feature_name=MTRNR2L2	MTRNR2L2.value <= threshold=2.8784297704696655
node_2: feature_name=TCL1A	TCL1A.value <= threshold=4.798312664031982
node_3: feature_name=MT-CO1	MT-CO1.value <= threshold=0.08947191759943962
node_4: feature_name=NR4A1	NR4A1.value <= threshold=1.0725348591804504
node_5: feature_name=HLA-DPA1	HLA-DPA1.value <= threshold=1.6885469555854797
node_6: feature_name=HLA-DRB5	HLA-DRB5.value <= threshold=2.1886194944381714
node_7: feature_name=XIST	XIST.value <= threshold=-0.12328458577394485
node_8: feature_name=SYNE1	SYNE1.value > threshold=-0.7835594415664673
node_16: feature_name=MTRNR2L2	MTRNR2L2.value <= threshold=2.4483247995376587
node_17: feature_name=HLA-DQA2	HLA-DQA2.value <= threshold=2.227178931236267
node_18: feature_name=NR4A1	NR4A1.value <= threshold=-0.21380723267793655
node_19: feature_name=IRAK3	IRAK3.value <= threshold=4.27159571647644
node_20: feature_name=IFIT2	IFIT2.value <= threshold=7.8175740242004395
node_21: feature_name=CD79A	CD79A.value <= threshold=2.857242226600647
node_22: feature_name=HIST1H2AC	HIST1H2AC.value <= threshold=5.077470302581787

node_23: feature_name=FTH1	FTH1.value > threshold=0.4158056080341339
node_285: feature_name=EGR1	EGR1.value <= threshold=4.240467071533203
node_286: feature_name=HLA-DRB1	HLA-DRB1.value <= threshold=-0.7553382813930511
node_287: feature_name=MARCKS	MARCKS.value <= threshold=1.0477054715156555
Class: COVID-19 Severe	
Rules_1050	passed counts:4
node_0: feature_name=LTB	LTB.value <= threshold=0.994031548500061
node_1: feature_name=MTRNR2L2	MTRNR2L2.value <= threshold=2.8784297704696655
node_2: feature_name=TCL1A	TCL1A.value <= threshold=4.798312664031982
node_3: feature_name=MT-CO1	MT-CO1.value <= threshold=0.08947191759943962
node_4: feature_name=NR4A1	NR4A1.value <= threshold=1.0725348591804504
node_5: feature_name=HLA-DPA1	HLA-DPA1.value <= threshold=1.6885469555854797
node_6: feature_name=HLA-DRB5	HLA-DRB5.value <= threshold=2.1886194944381714
node_7: feature_name=XIST	XIST.value <= threshold=-0.12328458577394485
node_8: feature_name=SYNE1	SYNE1.value > threshold=-0.7835594415664673
node_16: feature_name=MTRNR2L2	MTRNR2L2.value <= threshold=2.4483247995376587
node_17: feature_name=HLA-DQA2	HLA-DQA2.value <= threshold=2.227178931236267
node_18: feature_name=NR4A1	NR4A1.value <= threshold=-0.21380723267793655
node_19: feature_name=IRAK3	IRAK3.value <= threshold=4.27159571647644
node_20: feature_name=IFIT2	IFIT2.value <= threshold=7.8175740242004395
node_21: feature_name=CD79A	CD79A.value <= threshold=2.857242226600647
node_22: feature_name=HIST1H2AC	HIST1H2AC.value <= threshold=5.077470302581787
node_23: feature_name=FTH1	FTH1.value <= threshold=0.4158056080341339
node_24: feature_name=ATF3	ATF3.value <= threshold=3.8028353452682495
node_25: feature_name=OASL	OASL.value <= threshold=3.387580633163452
node_26: feature_name=MT-CO1	MT-CO1.value > threshold=-0.7942646145820618
node_74: feature_name=HOPX	HOPX.value > threshold=0.8682331740856171
node_250: feature_name=CD22	CD22.value > threshold=-0.23878339678049088
node_254: feature_name=MT-CO2	MT-CO2.value <= threshold=0.39438967406749725

node_255: feature_name=COTL1	COTL1.value <= threshold=-0.0382253173738718
node_256: feature_name=HLA-DQB1	HLA-DQB1.value > threshold=2.292426109313965
node_270: feature_name=NFKBID	NFKBID.value > threshold=0.7098620384931564
Class: COVID-19 Severe	
Rules_1051	passed counts:4
node_0: feature_name=LTB	LTB.value <= threshold=0.994031548500061
node_1: feature_name=MTRNR2L2	MTRNR2L2.value <= threshold=2.8784297704696655
node_2: feature_name=TCL1A	TCL1A.value <= threshold=4.798312664031982
node_3: feature_name=MT-CO1	MT-CO1.value <= threshold=0.08947191759943962
node_4: feature_name=NR4A1	NR4A1.value <= threshold=1.0725348591804504
node_5: feature_name=HLA-DPA1	HLA-DPA1.value <= threshold=1.6885469555854797
node_6: feature_name=HLA-DRB5	HLA-DRB5.value <= threshold=2.1886194944381714
node_7: feature_name=XIST	XIST.value <= threshold=-0.12328458577394485
node_8: feature_name=SYNE1	SYNE1.value > threshold=-0.7835594415664673
node_16: feature_name=MTRNR2L2	MTRNR2L2.value <= threshold=2.4483247995376587
node_17: feature_name=HLA-DQA2	HLA-DQA2.value <= threshold=2.227178931236267
node_18: feature_name=NR4A1	NR4A1.value <= threshold=-0.21380723267793655
node_19: feature_name=IRAK3	IRAK3.value <= threshold=4.27159571647644
node_20: feature_name=IFIT2	IFIT2.value <= threshold=7.8175740242004395
node_21: feature_name=CD79A	CD79A.value <= threshold=2.857242226600647
node_22: feature_name=HIST1H2AC	HIST1H2AC.value <= threshold=5.077470302581787
node_23: feature_name=FTH1	FTH1.value <= threshold=0.4158056080341339
node_24: feature_name=ATF3	ATF3.value <= threshold=3.8028353452682495
node_25: feature_name=OASL	OASL.value <= threshold=3.387580633163452
node_26: feature_name=MT-CO1	MT-CO1.value > threshold=-0.7942646145820618
node_74: feature_name=HOPX	HOPX.value <= threshold=0.8682331740856171
node_75: feature_name=RGS2	RGS2.value <= threshold=1.4589954018592834
node_76: feature_name=AHR	AHR.value <= threshold=3.870481848716736
node_77: feature_name=IGJ	IGJ.value <= threshold=4.902882814407349

node_78: feature_name=CD79B	CD79B.value <= threshold=4.87716269493103
node_79: feature_name=HLA-DQB1	HLA-DQB1.value <= threshold=3.6925491094589233
node_80: feature_name=HLA-DRB5	HLA-DRB5.value <= threshold=2.0926843881607056
node_81: feature_name=MCTP1	MCTP1.value <= threshold=4.010472774505615
node_82: feature_name=GNG11	GNG11.value > threshold=3.627470850944519
node_194: feature_name=SNX9	SNX9.value <= threshold=-0.5442804098129272
node_195: feature_name=HSPA1A	HSPA1A.value <= threshold=-0.2183535024523735
Class: Healthy Control	
Rules_1052	passed counts:4
node_0: feature_name=LTB	LTB.value <= threshold=0.994031548500061
node_1: feature_name=MTRNR2L2	MTRNR2L2.value <= threshold=2.8784297704696655
node_2: feature_name=TCL1A	TCL1A.value <= threshold=4.798312664031982
node_3: feature_name=MT-CO1	MT-CO1.value <= threshold=0.08947191759943962
node_4: feature_name=NR4A1	NR4A1.value <= threshold=1.0725348591804504
node_5: feature_name=HLA-DPA1	HLA-DPA1.value <= threshold=1.6885469555854797
node_6: feature_name=HLA-DRB5	HLA-DRB5.value <= threshold=2.1886194944381714
node_7: feature_name=XIST	XIST.value <= threshold=-0.12328458577394485
node_8: feature_name=SYNE1	SYNE1.value > threshold=-0.7835594415664673
node_16: feature_name=MTRNR2L2	MTRNR2L2.value <= threshold=2.4483247995376587
node_17: feature_name=HLA-DQA2	HLA-DQA2.value <= threshold=2.227178931236267
node_18: feature_name=NR4A1	NR4A1.value <= threshold=-0.21380723267793655
node_19: feature_name=IRAK3	IRAK3.value <= threshold=4.27159571647644
node_20: feature_name=IFIT2	IFIT2.value <= threshold=7.8175740242004395
node_21: feature_name=CD79A	CD79A.value <= threshold=2.857242226600647
node_22: feature_name=HIST1H2AC	HIST1H2AC.value <= threshold=5.077470302581787
node_23: feature_name=FTH1	FTH1.value <= threshold=0.4158056080341339
node_24: feature_name=ATF3	ATF3.value <= threshold=3.8028353452682495
node_25: feature_name=OASL	OASL.value <= threshold=3.387580633163452
node_26: feature_name=MT-CO1	MT-CO1.value > threshold=-0.7942646145820618

node_74: feature_name=HOPX	HOPX.value <= threshold=0.8682331740856171
node_75: feature_name=RGS2	RGS2.value <= threshold=1.4589954018592834
node_76: feature_name=AHR	AHR.value <= threshold=3.870481848716736
node_77: feature_name=IGJ	IGJ.value <= threshold=4.902882814407349
node_78: feature_name=CD79B	CD79B.value <= threshold=4.87716269493103
node_79: feature_name=HLA-DQB1	HLA-DQB1.value <= threshold=3.6925491094589233
node_80: feature_name=HLA-DRB5	HLA-DRB5.value <= threshold=2.0926843881607056
node_81: feature_name=MCTP1	MCTP1.value <= threshold=4.010472774505615
node_82: feature_name=GNG11	GNG11.value <= threshold=3.627470850944519
node_83: feature_name=HSP90B1	HSP90B1.value <= threshold=2.0835131406784058
node_84: feature_name=ATF3	ATF3.value <= threshold=1.1142374873161316
node_85: feature_name=MT-ND6	MT-ND6.value <= threshold=2.7328014373779297
node_86: feature_name=COTL1	COTL1.value > threshold=-1.6524587869644165
node_90: feature_name=MT-CYB	MT-CYB.value <= threshold=1.099629819393158
node_91: feature_name=RGCC	RGCC.value <= threshold=1.6908138990402222
node_92: feature_name=ILK	ILK.value <= threshold=3.9497616291046143
node_93: feature_name=CTD-2035E11.3	CTD-2035E11.3.value <= threshold=2.4543306827545166
node_94: feature_name=S100A6	S100A6.value <= threshold=0.2867250442504883
node_95: feature_name=HLA-DRB5	HLA-DRB5.value > threshold=-0.9496813416481018
node_99: feature_name=MT-CO3	MT-CO3.value > threshold=-1.55654776096344
node_103: feature_name=ITGAX	ITGAX.value <= threshold=1.8544010519981384
node_104: feature_name=MEF2C	MEF2C.value <= threshold=4.22724461555481
node_105: feature_name=CXorf57	CXorf57.value > threshold=5.114444732666016
node_143: feature_name=SPI1	SPI1.value <= threshold=0.5112176164984703
Class: COVID-19 Critical	
Rules_1053	passed counts:3
node_0: feature_name=LTB	LTB.value > threshold=0.994031548500061
node_5264: feature_name=MT-CO1	MT-CO1.value > threshold=-0.4127991199493408
node_5638: feature_name=OAZ1	OAZ1.value > threshold=-0.2640867084264755

node_6112: feature_name=IFI44L	IFI44L.value > threshold=2.5893263816833496
node_7078: feature_name=XIST	XIST.value <= threshold=1.1465829275548458
node_7079: feature_name=TCL1A	TCL1A.value <= threshold=7.115930080413818
node_7080: feature_name=XAF1	XAF1.value > threshold=-0.45034870505332947
node_7088: feature_name=FERMT3	FERMT3.value <= threshold=1.994587481021881
node_7089: feature_name=CD22	CD22.value > threshold=6.903364181518555
Class: Healthy Control	
Rules_1054	passed counts:3
node_0: feature_name=LTB	LTB.value > threshold=0.994031548500061
node_5264: feature_name=MT-CO1	MT-CO1.value > threshold=-0.4127991199493408
node_5638: feature_name=OAZ1	OAZ1.value > threshold=-0.2640867084264755
node_6112: feature_name=IFI44L	IFI44L.value > threshold=2.5893263816833496
node_7078: feature_name=XIST	XIST.value <= threshold=1.1465829275548458
node_7079: feature_name=TCL1A	TCL1A.value <= threshold=7.115930080413818
node_7080: feature_name=XAF1	XAF1.value <= threshold=-0.45034870505332947
node_7081: feature_name=NFKBID	NFKBID.value > threshold=1.3513729572296143
node_7085: feature_name=CD83	CD83.value > threshold=1.4530883431434631
Class: COVID-19 Severe	
Rules_1055	passed counts:3
node_0: feature_name=LTB	LTB.value > threshold=0.994031548500061
node_5264: feature_name=MT-CO1	MT-CO1.value > threshold=-0.4127991199493408
node_5638: feature_name=OAZ1	OAZ1.value > threshold=-0.2640867084264755
node_6112: feature_name=IFI44L	IFI44L.value > threshold=2.5893263816833496
node_7078: feature_name=XIST	XIST.value <= threshold=1.1465829275548458
node_7079: feature_name=TCL1A	TCL1A.value <= threshold=7.115930080413818
node_7080: feature_name=XAF1	XAF1.value <= threshold=-0.45034870505332947
node_7081: feature_name=NFKBID	NFKBID.value > threshold=1.3513729572296143
node_7085: feature_name=CD83	CD83.value <= threshold=1.4530883431434631

Class: COVID-19 Critical	
Rules_1056	passed counts:3
node_0: feature_name=LTB	LTB.value > threshold=0.994031548500061
node_5264: feature_name=MT-CO1	MT-CO1.value > threshold=-0.4127991199493408
node_5638: feature_name=OAZ1	OAZ1.value > threshold=-0.2640867084264755
node_6112: feature_name=IFI44L	IFI44L.value <= threshold=2.5893263816833496
node_6113: feature_name=TCL1A	TCL1A.value > threshold=4.201832294464111
node_6991: feature_name=XAF1	XAF1.value <= threshold=3.4585667848587036
node_6992: feature_name=STAG3	STAG3.value > threshold=7.686291694641113
node_7072: feature_name=MT-CO1	MT-CO1.value > threshold=0.184519425034523
Class: Healthy Control	
Rules_1057	passed counts:3
node_0: feature_name=LTB	LTB.value > threshold=0.994031548500061
node_5264: feature_name=MT-CO1	MT-CO1.value > threshold=-0.4127991199493408
node_5638: feature_name=OAZ1	OAZ1.value > threshold=-0.2640867084264755
node_6112: feature_name=IFI44L	IFI44L.value <= threshold=2.5893263816833496
node_6113: feature_name=TCL1A	TCL1A.value > threshold=4.201832294464111
node_6991: feature_name=XAF1	XAF1.value <= threshold=3.4585667848587036
node_6992: feature_name=STAG3	STAG3.value <= threshold=7.686291694641113
node_6993: feature_name=RALGPS2	RALGPS2.value > threshold=-0.4090157449245453
node_6997: feature_name=MTRNR2L2	MTRNR2L2.value <= threshold=3.23723828792572
node_6998: feature_name=TP53INP1	TP53INP1.value <= threshold=6.239925146102905
node_6999: feature_name=MEF2C	MEF2C.value > threshold=-0.44642284512519836
node_7003: feature_name=HSPA1A	HSPA1A.value <= threshold=7.404359817504883
node_7004: feature_name=COTL1	COTL1.value > threshold=-1.6598749160766602
node_7006: feature_name=HIST1H2AC	HIST1H2AC.value > threshold=-0.34910088777542114
node_7008: feature_name=MARCH1	MARCH1.value <= threshold=5.393903970718384
node_7009: feature_name=PELI1	PELI1.value <= threshold=3.565749168395996

node_7010: feature_name=RGS1	RGS1.value <= threshold=2.851950168609619
node_7011: feature_name=HLA-DRB5	HLA-DRB5.value > threshold=3.0528379678726196
node_7061: feature_name=HLA-DRB1	HLA-DRB1.value > threshold=1.697178602218628
Class: Healthy Control	
Rules_1058	passed counts:3
node_0: feature_name=LTB	LTB.value > threshold=0.994031548500061
node_5264: feature_name=MT-CO1	MT-CO1.value > threshold=-0.4127991199493408
node_5638: feature_name=OAZ1	OAZ1.value > threshold=-0.2640867084264755
node_6112: feature_name=IFI44L	IFI44L.value <= threshold=2.5893263816833496
node_6113: feature_name=TCL1A	TCL1A.value > threshold=4.201832294464111
node_6991: feature_name=XAF1	XAF1.value <= threshold=3.4585667848587036
node_6992: feature_name=STAG3	STAG3.value <= threshold=7.686291694641113
node_6993: feature_name=RALGPS2	RALGPS2.value > threshold=-0.4090157449245453
node_6997: feature_name=MTRNR2L2	MTRNR2L2.value <= threshold=3.23723828792572
node_6998: feature_name=TP53INP1	TP53INP1.value <= threshold=6.239925146102905
node_6999: feature_name=MEF2C	MEF2C.value > threshold=-0.44642284512519836
node_7003: feature_name=HSPA1A	HSPA1A.value <= threshold=7.404359817504883
node_7004: feature_name=COTL1	COTL1.value > threshold=-1.6598749160766602
node_7006: feature_name=HIST1H2AC	HIST1H2AC.value > threshold=-0.34910088777542114
node_7008: feature_name=MARCH1	MARCH1.value <= threshold=5.393903970718384
node_7009: feature_name=PELI1	PELI1.value <= threshold=3.565749168395996
node_7010: feature_name=RGS1	RGS1.value <= threshold=2.851950168609619
node_7011: feature_name=HLA-DRB5	HLA-DRB5.value <= threshold=3.0528379678726196
node_7012: feature_name=MEF2A	MEF2A.value > threshold=3.228689193725586
node_7056: feature_name=TCL1A	TCL1A.value <= threshold=4.69646143913269
node_7057: feature_name=HLA-DQA2	HLA-DQA2.value <= threshold=3.492533802986145
Class: COVID-19 Critical	
Rules_1059	passed counts:3

node_0: feature_name=LTB	LTB.value > threshold=0.994031548500061
node_5264: feature_name=MT-CO1	MT-CO1.value > threshold=-0.4127991199493408
node_5638: feature_name=OAZ1	OAZ1.value > threshold=-0.2640867084264755
node_6112: feature_name=IFI44L	IFI44L.value <= threshold=2.5893263816833496
node_6113: feature_name=TCL1A	TCL1A.value > threshold=4.201832294464111
node_6991: feature_name=XAF1	XAF1.value <= threshold=3.4585667848587036
node_6992: feature_name=STAG3	STAG3.value <= threshold=7.686291694641113
node_6993: feature_name=RALGPS2	RALGPS2.value > threshold=-0.4090157449245453
node_6997: feature_name=MTRNR2L2	MTRNR2L2.value <= threshold=3.23723828792572
node_6998: feature_name=TP53INP1	TP53INP1.value <= threshold=6.239925146102905
node_6999: feature_name=MEF2C	MEF2C.value > threshold=-0.44642284512519836
node_7003: feature_name=HSPA1A	HSPA1A.value <= threshold=7.404359817504883
node_7004: feature_name=COTL1	COTL1.value > threshold=-1.6598749160766602
node_7006: feature_name=HIST1H2AC	HIST1H2AC.value > threshold=-0.34910088777542114
node_7008: feature_name=MARCH1	MARCH1.value <= threshold=5.393903970718384
node_7009: feature_name=PELI1	PELI1.value <= threshold=3.565749168395996
node_7010: feature_name=RGS1	RGS1.value <= threshold=2.851950168609619
node_7011: feature_name=HLA-DRB5	HLA-DRB5.value <= threshold=3.0528379678726196
node_7012: feature_name=MEF2A	MEF2A.value <= threshold=3.228689193725586
node_7013: feature_name=MT-CO1	MT-CO1.value > threshold=-0.37944722175598145
node_7017: feature_name=ATG2A	ATG2A.value <= threshold=2.790610194206238
node_7018: feature_name=MEF2C	MEF2C.value <= threshold=-0.404978945851326
node_7019: feature_name=NAIP	NAIP.value > threshold=-0.33305495977401733
node_7023: feature_name=HES4	HES4.value > threshold=-0.2065645307302475
Class: COVID-19 Critical	
Rules_1060	passed counts:3
node_0: feature_name=LTB	LTB.value > threshold=0.994031548500061
node_5264: feature_name=MT-CO1	MT-CO1.value > threshold=-0.4127991199493408
node_5638: feature_name=OAZ1	OAZ1.value > threshold=-0.2640867084264755

node_6112: feature_name=IFI44L	IFI44L.value <= threshold=2.5893263816833496
node_6113: feature_name=TCL1A	TCL1A.value > threshold=4.201832294464111
node_6991: feature_name=XAF1	XAF1.value <= threshold=3.4585667848587036
node_6992: feature_name=STAG3	STAG3.value <= threshold=7.686291694641113
node_6993: feature_name=RALGPS2	RALGPS2.value > threshold=-0.4090157449245453
node_6997: feature_name=MTRNR2L2	MTRNR2L2.value <= threshold=3.23723828792572
node_6998: feature_name=TP53INP1	TP53INP1.value <= threshold=6.239925146102905
node_6999: feature_name=MEF2C	MEF2C.value > threshold=-0.44642284512519836
node_7003: feature_name=HSPA1A	HSPA1A.value <= threshold=7.404359817504883
node_7004: feature_name=COTL1	COTL1.value > threshold=-1.6598749160766602
node_7006: feature_name=HIST1H2AC	HIST1H2AC.value > threshold=-0.34910088777542114
node_7008: feature_name=MARCH1	MARCH1.value <= threshold=5.393903970718384
node_7009: feature_name=PELI1	PELI1.value <= threshold=3.565749168395996
node_7010: feature_name=RGS1	RGS1.value <= threshold=2.851950168609619
node_7011: feature_name=HLA-DRB5	HLA-DRB5.value <= threshold=3.0528379678726196
node_7012: feature_name=MEF2A	MEF2A.value <= threshold=3.228689193725586
node_7013: feature_name=MT-CO1	MT-CO1.value <= threshold=-0.37944722175598145
node_7014: feature_name=STAG3	STAG3.value > threshold=1.8744874000549316
Class: COVID-19 Critical	
Rules_1061	passed counts:3
node_0: feature_name=LTB	LTB.value > threshold=0.994031548500061
node_5264: feature_name=MT-CO1	MT-CO1.value > threshold=-0.4127991199493408
node_5638: feature_name=OAZ1	OAZ1.value > threshold=-0.2640867084264755
node_6112: feature_name=IFI44L	IFI44L.value <= threshold=2.5893263816833496
node_6113: feature_name=TCL1A	TCL1A.value > threshold=4.201832294464111
node_6991: feature_name=XAF1	XAF1.value <= threshold=3.4585667848587036
node_6992: feature_name=STAG3	STAG3.value <= threshold=7.686291694641113
node_6993: feature_name=RALGPS2	RALGPS2.value > threshold=-0.4090157449245453
node_6997: feature_name=MTRNR2L2	MTRNR2L2.value <= threshold=3.23723828792572

node_6998: feature_name=TP53INP1	TP53INP1.value <= threshold=6.239925146102905
node_6999: feature_name=MEF2C	MEF2C.value <= threshold=-0.44642284512519836
node_7000: feature_name=HLA-DPA1	HLA-DPA1.value > threshold=1.8664597272872925
Class: COVID-19 Critical	
Rules_1062	passed counts:3
node_0: feature_name=LTB	LTB.value > threshold=0.994031548500061
node_5264: feature_name=MT-CO1	MT-CO1.value > threshold=-0.4127991199493408
node_5638: feature_name=OAZ1	OAZ1.value > threshold=-0.2640867084264755
node_6112: feature_name=IFI44L	IFI44L.value <= threshold=2.5893263816833496
node_6113: feature_name=TCL1A	TCL1A.value <= threshold=4.201832294464111
node_6114: feature_name=HLA-DQA2	HLA-DQA2.value > threshold=-0.2270243540406227
node_6542: feature_name=CD79B	CD79B.value > threshold=3.096232771873474
node_6872: feature_name=ICAM1	ICAM1.value > threshold=-0.2232399433851242
node_6974: feature_name=HSP90B1	HSP90B1.value > threshold=-0.9936705231666565
node_6980: feature_name=TCL1A	TCL1A.value > threshold=-0.17627128213644028
node_6988: feature_name=BOD1L1	BOD1L1.value > threshold=-0.48048292100429535
Class: Healthy Control	
Rules_1063	passed counts:3
node_0: feature_name=LTB	LTB.value > threshold=0.994031548500061
node_5264: feature_name=MT-CO1	MT-CO1.value > threshold=-0.4127991199493408
node_5638: feature_name=OAZ1	OAZ1.value > threshold=-0.2640867084264755
node_6112: feature_name=IFI44L	IFI44L.value <= threshold=2.5893263816833496
node_6113: feature_name=TCL1A	TCL1A.value <= threshold=4.201832294464111
node_6114: feature_name=HLA-DQA2	HLA-DQA2.value > threshold=-0.2270243540406227
node_6542: feature_name=CD79B	CD79B.value > threshold=3.096232771873474
node_6872: feature_name=ICAM1	ICAM1.value > threshold=-0.2232399433851242
node_6974: feature_name=HSP90B1	HSP90B1.value <= threshold=-0.9936705231666565
node_6975: feature_name=FTH1	FTH1.value <= threshold=-0.6905665695667267

Class: COVID-19 Critical	
Rules_1064	passed counts:3
node_0: feature_name=LTB	LTB.value > threshold=0.994031548500061
node_5264: feature_name=MT-CO1	MT-CO1.value > threshold=-0.4127991199493408
node_5638: feature_name=OAZ1	OAZ1.value > threshold=-0.2640867084264755
node_6112: feature_name=IFI44L	IFI44L.value <= threshold=2.5893263816833496
node_6113: feature_name=TCL1A	TCL1A.value <= threshold=4.201832294464111
node_6114: feature_name=HLA-DQA2	HLA-DQA2.value > threshold=-0.2270243540406227
node_6542: feature_name=CD79B	CD79B.value > threshold=3.096232771873474
node_6872: feature_name=ICAM1	ICAM1.value <= threshold=-0.2232399433851242
node_6873: feature_name=STAG3	STAG3.value > threshold=-0.19312061369419098
node_6939: feature_name=HLA-DRB5	HLA-DRB5.value <= threshold=2.209904909133911
node_6940: feature_name=STAG3	STAG3.value <= threshold=7.8730878829956055
node_6941: feature_name=FAM129C	FAM129C.value > threshold=1.668714702129364
node_6953: feature_name=ENDOD1	ENDOD1.value > threshold=2.171262264251709
node_6959: feature_name=TTN	TTN.value > threshold=-0.25319549441337585
Class: COVID-19 Critical	
Rules_1065	passed counts:3
node_0: feature_name=LTB	LTB.value > threshold=0.994031548500061
node_5264: feature_name=MT-CO1	MT-CO1.value > threshold=-0.4127991199493408
node_5638: feature_name=OAZ1	OAZ1.value > threshold=-0.2640867084264755
node_6112: feature_name=IFI44L	IFI44L.value <= threshold=2.5893263816833496
node_6113: feature_name=TCL1A	TCL1A.value <= threshold=4.201832294464111
node_6114: feature_name=HLA-DQA2	HLA-DQA2.value > threshold=-0.2270243540406227
node_6542: feature_name=CD79B	CD79B.value > threshold=3.096232771873474
node_6872: feature_name=ICAM1	ICAM1.value <= threshold=-0.2232399433851242
node_6873: feature_name=STAG3	STAG3.value <= threshold=-0.19312061369419098
node_6874: feature_name=CD79A	CD79A.value > threshold=1.57558012008667

node_6880: feature_name=MEF2A	MEF2A.value <= threshold=2.676826596260071
node_6881: feature_name=MTRNR2L2	MTRNR2L2.value > threshold=2.9562686681747437
Class: COVID-19 Severe	
Rules_1066	passed counts:3
node_0: feature_name=LTB	LTB.value > threshold=0.994031548500061
node_5264: feature_name=MT-CO1	MT-CO1.value > threshold=-0.4127991199493408
node_5638: feature_name=OAZ1	OAZ1.value > threshold=-0.2640867084264755
node_6112: feature_name=IFI44L	IFI44L.value <= threshold=2.5893263816833496
node_6113: feature_name=TCL1A	TCL1A.value <= threshold=4.201832294464111
node_6114: feature_name=HLA-DQA2	HLA-DQA2.value > threshold=-0.2270243540406227
node_6542: feature_name=CD79B	CD79B.value > threshold=3.096232771873474
node_6872: feature_name=ICAM1	ICAM1.value <= threshold=-0.2232399433851242
node_6873: feature_name=STAG3	STAG3.value <= threshold=-0.19312061369419098
node_6874: feature_name=CD79A	CD79A.value > threshold=1.57558012008667
node_6880: feature_name=MEF2A	MEF2A.value <= threshold=2.676826596260071
node_6881: feature_name=MTRNR2L2	MTRNR2L2.value <= threshold=2.9562686681747437
node_6882: feature_name=SNX9	SNX9.value <= threshold=3.162803888320923
node_6883: feature_name=MARCKS	MARCKS.value <= threshold=4.3125433921813965
node_6884: feature_name=MX1	MX1.value > threshold=2.1828908920288086
node_6922: feature_name=ENDOD1	ENDOD1.value > threshold=-0.23167684674263
Class: Healthy Control	
Rules_1067	passed counts:3
node_0: feature_name=LTB	LTB.value > threshold=0.994031548500061
node_5264: feature_name=MT-CO1	MT-CO1.value > threshold=-0.4127991199493408
node_5638: feature_name=OAZ1	OAZ1.value > threshold=-0.2640867084264755
node_6112: feature_name=IFI44L	IFI44L.value <= threshold=2.5893263816833496
node_6113: feature_name=TCL1A	TCL1A.value <= threshold=4.201832294464111
node_6114: feature_name=HLA-DQA2	HLA-DQA2.value > threshold=-0.2270243540406227

node_6542: feature_name=CD79B	CD79B.value > threshold=3.096232771873474
node_6872: feature_name=ICAM1	ICAM1.value <= threshold=-0.2232399433851242
node_6873: feature_name=STAG3	STAG3.value <= threshold=-0.19312061369419098
node_6874: feature_name=CD79A	CD79A.value > threshold=1.57558012008667
node_6880: feature_name=MEF2A	MEF2A.value <= threshold=2.676826596260071
node_6881: feature_name=MTRNR2L2	MTRNR2L2.value <= threshold=2.9562686681747437
node_6882: feature_name=SNX9	SNX9.value <= threshold=3.162803888320923
node_6883: feature_name=MARCKS	MARCKS.value <= threshold=4.3125433921813965
node_6884: feature_name=MX1	MX1.value > threshold=2.1828908920288086
node_6922: feature_name=ENDOD1	ENDOD1.value <= threshold=-0.23167684674263
node_6923: feature_name=RHOB	RHOB.value <= threshold=2.444888472557068
Class: COVID-19 Critical	
Rules_1068	passed counts:3
node_0: feature_name=LTB	LTB.value > threshold=0.994031548500061
node_5264: feature_name=MT-CO1	MT-CO1.value > threshold=-0.4127991199493408
node_5638: feature_name=OAZ1	OAZ1.value > threshold=-0.2640867084264755
node_6112: feature_name=IFI44L	IFI44L.value <= threshold=2.5893263816833496
node_6113: feature_name=TCL1A	TCL1A.value <= threshold=4.201832294464111
node_6114: feature_name=HLA-DQA2	HLA-DQA2.value > threshold=-0.2270243540406227
node_6542: feature_name=CD79B	CD79B.value > threshold=3.096232771873474
node_6872: feature_name=ICAM1	ICAM1.value <= threshold=-0.2232399433851242
node_6873: feature_name=STAG3	STAG3.value <= threshold=-0.19312061369419098
node_6874: feature_name=CD79A	CD79A.value > threshold=1.57558012008667
node_6880: feature_name=MEF2A	MEF2A.value <= threshold=2.676826596260071
node_6881: feature_name=MTRNR2L2	MTRNR2L2.value <= threshold=2.9562686681747437
node_6882: feature_name=SNX9	SNX9.value <= threshold=3.162803888320923
node_6883: feature_name=MARCKS	MARCKS.value <= threshold=4.3125433921813965
node_6884: feature_name=MX1	MX1.value <= threshold=2.1828908920288086
node_6885: feature_name=ATF5	ATF5.value <= threshold=3.838430166244507

node_6886: feature_name=PELI1	PELI1.value > threshold=1.8930315971374512
node_6910: feature_name=RHOB	RHOB.value <= threshold=-0.40518850088119507
node_6911: feature_name=SNX9	SNX9.value <= threshold=0.20708411931991577
Class: Healthy Control	
Rules_1069	passed counts:3
node_0: feature_name=LTB	LTB.value > threshold=0.994031548500061
node_5264: feature_name=MT-CO1	MT-CO1.value > threshold=-0.4127991199493408
node_5638: feature_name=OAZ1	OAZ1.value > threshold=-0.2640867084264755
node_6112: feature_name=IFI44L	IFI44L.value <= threshold=2.5893263816833496
node_6113: feature_name=TCL1A	TCL1A.value <= threshold=4.201832294464111
node_6114: feature_name=HLA-DQA2	HLA-DQA2.value > threshold=-0.2270243540406227
node_6542: feature_name=CD79B	CD79B.value <= threshold=3.096232771873474
node_6543: feature_name=NR4A1	NR4A1.value > threshold=1.421015739440918
node_6811: feature_name=SLC7A5	SLC7A5.value > threshold=1.570515513420105
node_6867: feature_name=HSP90B1	HSP90B1.value > threshold=-0.995681881904602
node_6869: feature_name=MT-CYB	MT-CYB.value <= threshold=0.2630006968975067
Class: COVID-19 Severe	
Rules_1070	passed counts:3
node_0: feature_name=LTB	LTB.value > threshold=0.994031548500061
node_5264: feature_name=MT-CO1	MT-CO1.value > threshold=-0.4127991199493408
node_5638: feature_name=OAZ1	OAZ1.value > threshold=-0.2640867084264755
node_6112: feature_name=IFI44L	IFI44L.value <= threshold=2.5893263816833496
node_6113: feature_name=TCL1A	TCL1A.value <= threshold=4.201832294464111
node_6114: feature_name=HLA-DQA2	HLA-DQA2.value > threshold=-0.2270243540406227
node_6542: feature_name=CD79B	CD79B.value <= threshold=3.096232771873474
node_6543: feature_name=NR4A1	NR4A1.value > threshold=1.421015739440918
node_6811: feature_name=SLC7A5	SLC7A5.value <= threshold=1.570515513420105
node_6812: feature_name=XAF1	XAF1.value <= threshold=2.218220591545105

node_6813: feature_name=MEF2C	MEF2C.value > threshold=-0.35489463806152344
node_6831: feature_name=ACSL1	ACSL1.value <= threshold=2.9197680950164795
node_6832: feature_name=SNX9	SNX9.value <= threshold=4.184023022651672
node_6833: feature_name=HLA-DQB1	HLA-DQB1.value > threshold=-0.5399456918239594
node_6837: feature_name=SYTL3	SYTL3.value <= threshold=1.365163803100586
node_6838: feature_name=MIAT	MIAT.value <= threshold=1.9473694376647472
node_6839: feature_name=TRIB1	TRIB1.value <= threshold=2.461132228374481
node_6840: feature_name=ATF5	ATF5.value <= threshold=5.3038389682769775
node_6841: feature_name=S100A6	S100A6.value <= threshold=-1.8476895093917847
node_6842: feature_name=ICAM1	ICAM1.value <= threshold=-0.3907465785741806
Class: Healthy Control	
Rules_1071	passed counts:3
node_0: feature_name=LTB	LTB.value > threshold=0.994031548500061
node_5264: feature_name=MT-CO1	MT-CO1.value > threshold=-0.4127991199493408
node_5638: feature_name=OAZ1	OAZ1.value > threshold=-0.2640867084264755
node_6112: feature_name=IFI44L	IFI44L.value <= threshold=2.5893263816833496
node_6113: feature_name=TCL1A	TCL1A.value <= threshold=4.201832294464111
node_6114: feature_name=HLA-DQA2	HLA-DQA2.value > threshold=-0.2270243540406227
node_6542: feature_name=CD79B	CD79B.value <= threshold=3.096232771873474
node_6543: feature_name=NR4A1	NR4A1.value > threshold=1.421015739440918
node_6811: feature_name=SLC7A5	SLC7A5.value <= threshold=1.570515513420105
node_6812: feature_name=XAF1	XAF1.value <= threshold=2.218220591545105
node_6813: feature_name=MEF2C	MEF2C.value <= threshold=-0.35489463806152344
node_6814: feature_name=MIR24-2	MIR24-2.value <= threshold=1.5691230297088623
node_6815: feature_name=HLA-DQB1	HLA-DQB1.value <= threshold=2.6548476219177246
node_6816: feature_name=PELI1	PELI1.value > threshold=2.055415987968445
Class: COVID-19 Critical	
Rules_1072	passed counts:3

node_0: feature_name=LTB	LTB.value > threshold=0.994031548500061
node_5264: feature_name=MT-CO1	MT-CO1.value > threshold=-0.4127991199493408
node_5638: feature_name=OAZ1	OAZ1.value > threshold=-0.2640867084264755
node_6112: feature_name=IFI44L	IFI44L.value <= threshold=2.5893263816833496
node_6113: feature_name=TCL1A	TCL1A.value <= threshold=4.201832294464111
node_6114: feature_name=HLA-DQA2	HLA-DQA2.value > threshold=-0.2270243540406227
node_6542: feature_name=CD79B	CD79B.value <= threshold=3.096232771873474
node_6543: feature_name=NR4A1	NR4A1.value <= threshold=1.421015739440918
node_6544: feature_name=LTB	LTB.value > threshold=1.359755277633667
node_6720: feature_name=NFKBIZ	NFKBIZ.value <= threshold=0.2720414251089096
node_6721: feature_name=ATF5	ATF5.value <= threshold=2.4672781229019165
node_6722: feature_name=SNX9	SNX9.value <= threshold=2.0814030170440674
node_6723: feature_name=HLA-DQA2	HLA-DQA2.value > threshold=4.360614776611328
node_6775: feature_name=RP11-731F5.2	RP11-731F5.2.value <= threshold=1.6779262274503708
node_6776: feature_name=TTN	TTN.value > threshold=-0.2564083933830261
Class: COVID-19 Critical	
Rules_1073	passed counts:3
node_0: feature_name=LTB	LTB.value > threshold=0.994031548500061
node_5264: feature_name=MT-CO1	MT-CO1.value > threshold=-0.4127991199493408
node_5638: feature_name=OAZ1	OAZ1.value > threshold=-0.2640867084264755
node_6112: feature_name=IFI44L	IFI44L.value <= threshold=2.5893263816833496
node_6113: feature_name=TCL1A	TCL1A.value <= threshold=4.201832294464111
node_6114: feature_name=HLA-DQA2	HLA-DQA2.value > threshold=-0.2270243540406227
node_6542: feature_name=CD79B	CD79B.value <= threshold=3.096232771873474
node_6543: feature_name=NR4A1	NR4A1.value <= threshold=1.421015739440918
node_6544: feature_name=LTB	LTB.value > threshold=1.359755277633667
node_6720: feature_name=NFKBIZ	NFKBIZ.value <= threshold=0.2720414251089096
node_6721: feature_name=ATF5	ATF5.value <= threshold=2.4672781229019165
node_6722: feature_name=SNX9	SNX9.value <= threshold=2.0814030170440674

node_6723: feature_name=HLA-DQA2	HLA-DQA2.value <= threshold=4.360614776611328
node_6724: feature_name=MT-CO1	MT-CO1.value > threshold=-0.12188276648521423
node_6736: feature_name=MT1E	MT1E.value <= threshold=-0.1352209895849228
node_6737: feature_name=PELI1	PELI1.value > threshold=3.1761642694473267
node_6769: feature_name=HLA-DQB1	HLA-DQB1.value <= threshold=2.9407562017440796
Class: COVID-19 Critical	
Rules_1074	passed counts:3
node_0: feature_name=LTB	LTB.value > threshold=0.994031548500061
node_5264: feature_name=MT-CO1	MT-CO1.value > threshold=-0.4127991199493408
node_5638: feature_name=OAZ1	OAZ1.value > threshold=-0.2640867084264755
node_6112: feature_name=IFI44L	IFI44L.value <= threshold=2.5893263816833496
node_6113: feature_name=TCL1A	TCL1A.value <= threshold=4.201832294464111
node_6114: feature_name=HLA-DQA2	HLA-DQA2.value > threshold=-0.2270243540406227
node_6542: feature_name=CD79B	CD79B.value <= threshold=3.096232771873474
node_6543: feature_name=NR4A1	NR4A1.value <= threshold=1.421015739440918
node_6544: feature_name=LTB	LTB.value > threshold=1.359755277633667
node_6720: feature_name=NFKBIZ	NFKBIZ.value <= threshold=0.2720414251089096
node_6721: feature_name=ATF5	ATF5.value <= threshold=2.4672781229019165
node_6722: feature_name=SNX9	SNX9.value <= threshold=2.0814030170440674
node_6723: feature_name=HLA-DQA2	HLA-DQA2.value <= threshold=4.360614776611328
node_6724: feature_name=MT-CO1	MT-CO1.value <= threshold=-0.12188276648521423
node_6725: feature_name=CD79B	CD79B.value <= threshold=2.3298217058181763
node_6726: feature_name=MARCKS	MARCKS.value > threshold=2.858885169029236
Class: COVID-19 Severe	
Rules_1075	passed counts:3
node_0: feature_name=LTB	LTB.value > threshold=0.994031548500061
node_5264: feature_name=MT-CO1	MT-CO1.value > threshold=-0.4127991199493408
node_5638: feature_name=OAZ1	OAZ1.value > threshold=-0.2640867084264755

node_6112: feature_name=IFI44L	IFI44L.value <= threshold=2.5893263816833496
node_6113: feature_name=TCL1A	TCL1A.value <= threshold=4.201832294464111
node_6114: feature_name=HLA-DQA2	HLA-DQA2.value > threshold=-0.2270243540406227
node_6542: feature_name=CD79B	CD79B.value <= threshold=3.096232771873474
node_6543: feature_name=NR4A1	NR4A1.value <= threshold=1.421015739440918
node_6544: feature_name=LTB	LTB.value > threshold=1.359755277633667
node_6720: feature_name=NFKBIZ	NFKBIZ.value <= threshold=0.2720414251089096
node_6721: feature_name=ATF5	ATF5.value <= threshold=2.4672781229019165
node_6722: feature_name=SNX9	SNX9.value <= threshold=2.0814030170440674
node_6723: feature_name=HLA-DQA2	HLA-DQA2.value <= threshold=4.360614776611328
node_6724: feature_name=MT-CO1	MT-CO1.value <= threshold=-0.12188276648521423
node_6725: feature_name=CD79B	CD79B.value <= threshold=2.3298217058181763
node_6726: feature_name=MARCKS	MARCKS.value <= threshold=2.858885169029236
node_6727: feature_name=AHR	AHR.value > threshold=-0.3792427331209183
Class: Healthy Control	
Rules_1076	passed counts:3
node_0: feature_name=LTB	LTB.value > threshold=0.994031548500061
node_5264: feature_name=MT-CO1	MT-CO1.value > threshold=-0.4127991199493408
node_5638: feature_name=OAZ1	OAZ1.value > threshold=-0.2640867084264755
node_6112: feature_name=IFI44L	IFI44L.value <= threshold=2.5893263816833496
node_6113: feature_name=TCL1A	TCL1A.value <= threshold=4.201832294464111
node_6114: feature_name=HLA-DQA2	HLA-DQA2.value > threshold=-0.2270243540406227
node_6542: feature_name=CD79B	CD79B.value <= threshold=3.096232771873474
node_6543: feature_name=NR4A1	NR4A1.value <= threshold=1.421015739440918
node_6544: feature_name=LTB	LTB.value <= threshold=1.359755277633667
node_6545: feature_name=NFKBID	NFKBID.value > threshold=1.4294711351394653
node_6643: feature_name=FAM129C	FAM129C.value > threshold=4.799884080886841
node_6709: feature_name=MT-ND6	MT-ND6.value > threshold=-0.9825485348701477
node_6717: feature_name=PARP14	PARP14.value > threshold=3.3823366165161133

Class: COVID-19 Critical	
Rules_1077	passed counts:3
node_0: feature_name=LTB	LTB.value > threshold=0.994031548500061
node_5264: feature_name=MT-CO1	MT-CO1.value > threshold=-0.4127991199493408
node_5638: feature_name=OAZ1	OAZ1.value > threshold=-0.2640867084264755
node_6112: feature_name=IFI44L	IFI44L.value <= threshold=2.5893263816833496
node_6113: feature_name=TCL1A	TCL1A.value <= threshold=4.201832294464111
node_6114: feature_name=HLA-DQA2	HLA-DQA2.value > threshold=-0.2270243540406227
node_6542: feature_name=CD79B	CD79B.value <= threshold=3.096232771873474
node_6543: feature_name=NR4A1	NR4A1.value <= threshold=1.421015739440918
node_6544: feature_name=LTB	LTB.value <= threshold=1.359755277633667
node_6545: feature_name=NFKBID	NFKBID.value > threshold=1.4294711351394653
node_6643: feature_name=FAM129C	FAM129C.value > threshold=4.799884080886841
node_6709: feature_name=MT-ND6	MT-ND6.value <= threshold=-0.9825485348701477
node_6710: feature_name=EGR1	EGR1.value <= threshold=-0.32739605009555817
node_6711: feature_name=NR4A3	NR4A3.value <= threshold=1.4683782160282135
Class: COVID-19 Critical	
Rules_1078	passed counts:3
node_0: feature_name=LTB	LTB.value > threshold=0.994031548500061
node_5264: feature_name=MT-CO1	MT-CO1.value > threshold=-0.4127991199493408
node_5638: feature_name=OAZ1	OAZ1.value > threshold=-0.2640867084264755
node_6112: feature_name=IFI44L	IFI44L.value <= threshold=2.5893263816833496
node_6113: feature_name=TCL1A	TCL1A.value <= threshold=4.201832294464111
node_6114: feature_name=HLA-DQA2	HLA-DQA2.value > threshold=-0.2270243540406227
node_6542: feature_name=CD79B	CD79B.value <= threshold=3.096232771873474
node_6543: feature_name=NR4A1	NR4A1.value <= threshold=1.421015739440918
node_6544: feature_name=LTB	LTB.value <= threshold=1.359755277633667
node_6545: feature_name=NFKBID	NFKBID.value > threshold=1.4294711351394653

node_6643: feature_name=FAM129C	FAM129C.value <= threshold=4.799884080886841
node_6644: feature_name=XIST	XIST.value <= threshold=0.9931697957217693
node_6645: feature_name=GPX1	GPX1.value > threshold=0.3628883808851242
node_6695: feature_name=CD79A	CD79A.value > threshold=2.4297949075698853
node_6699: feature_name=FCRL1	FCRL1.value <= threshold=-0.20751195400953293
node_6700: feature_name=DAPP1	DAPP1.value > threshold=-0.2950359880924225
node_6702: feature_name=XIST	XIST.value <= threshold=-0.25556112825870514
Class: COVID-19 Severe	
Rules_1079	passed counts:3
node_0: feature_name=LTB	LTB.value > threshold=0.994031548500061
node_5264: feature_name=MT-CO1	MT-CO1.value > threshold=-0.4127991199493408
node_5638: feature_name=OAZ1	OAZ1.value > threshold=-0.2640867084264755
node_6112: feature_name=IFI44L	IFI44L.value <= threshold=2.5893263816833496
node_6113: feature_name=TCL1A	TCL1A.value <= threshold=4.201832294464111
node_6114: feature_name=HLA-DQA2	HLA-DQA2.value > threshold=-0.2270243540406227
node_6542: feature_name=CD79B	CD79B.value <= threshold=3.096232771873474
node_6543: feature_name=NR4A1	NR4A1.value <= threshold=1.421015739440918
node_6544: feature_name=LTB	LTB.value <= threshold=1.359755277633667
node_6545: feature_name=NFKBID	NFKBID.value > threshold=1.4294711351394653
node_6643: feature_name=FAM129C	FAM129C.value <= threshold=4.799884080886841
node_6644: feature_name=XIST	XIST.value <= threshold=0.9931697957217693
node_6645: feature_name=GPX1	GPX1.value <= threshold=0.3628883808851242
node_6646: feature_name=STMN1	STMN1.value <= threshold=2.3225364685058594
node_6647: feature_name=APP	APP.value <= threshold=4.124613881111145
node_6648: feature_name=FCER2	FCER2.value <= threshold=5.776939868927002
node_6649: feature_name=MEF2C	MEF2C.value > threshold=3.8539518117904663
node_6683: feature_name=MT-ND6	MT-ND6.value > threshold=0.14632150530815125
Class: COVID-19 Critical	

Rules_1080	passed counts:3
node_0: feature_name=LTB	LTB.value > threshold=0.994031548500061
node_5264: feature_name=MT-CO1	MT-CO1.value > threshold=-0.4127991199493408
node_5638: feature_name=OAZ1	OAZ1.value > threshold=-0.2640867084264755
node_6112: feature_name=IFI44L	IFI44L.value <= threshold=2.5893263816833496
node_6113: feature_name=TCL1A	TCL1A.value <= threshold=4.201832294464111
node_6114: feature_name=HLA-DQA2	HLA-DQA2.value > threshold=-0.2270243540406227
node_6542: feature_name=CD79B	CD79B.value <= threshold=3.096232771873474
node_6543: feature_name=NR4A1	NR4A1.value <= threshold=1.421015739440918
node_6544: feature_name=LTB	LTB.value <= threshold=1.359755277633667
node_6545: feature_name=NFKBID	NFKBID.value > threshold=1.4294711351394653
node_6643: feature_name=FAM129C	FAM129C.value <= threshold=4.799884080886841
node_6644: feature_name=XIST	XIST.value <= threshold=0.9931697957217693
node_6645: feature_name=GPX1	GPX1.value <= threshold=0.3628883808851242
node_6646: feature_name=STMN1	STMN1.value <= threshold=2.3225364685058594
node_6647: feature_name=APP	APP.value <= threshold=4.124613881111145
node_6648: feature_name=FCER2	FCER2.value <= threshold=5.776939868927002
node_6649: feature_name=MEF2C	MEF2C.value > threshold=3.8539518117904663
node_6683: feature_name=MT-ND6	MT-ND6.value <= threshold=0.14632150530815125
Class: Healthy Control	
Rules_1081	passed counts:3
node_0: feature_name=LTB	LTB.value > threshold=0.994031548500061
node_5264: feature_name=MT-CO1	MT-CO1.value > threshold=-0.4127991199493408
node_5638: feature_name=OAZ1	OAZ1.value > threshold=-0.2640867084264755
node_6112: feature_name=IFI44L	IFI44L.value <= threshold=2.5893263816833496
node_6113: feature_name=TCL1A	TCL1A.value <= threshold=4.201832294464111
node_6114: feature_name=HLA-DQA2	HLA-DQA2.value > threshold=-0.2270243540406227
node_6542: feature_name=CD79B	CD79B.value <= threshold=3.096232771873474
node_6543: feature_name=NR4A1	NR4A1.value <= threshold=1.421015739440918

node_6544: feature_name=LTB	LTB.value <= threshold=1.359755277633667
node_6545: feature_name=NFKBID	NFKBID.value > threshold=1.4294711351394653
node_6643: feature_name=FAM129C	FAM129C.value <= threshold=4.799884080886841
node_6644: feature_name=XIST	XIST.value <= threshold=0.9931697957217693
node_6645: feature_name=GPX1	GPX1.value <= threshold=0.3628883808851242
node_6646: feature_name=STMN1	STMN1.value <= threshold=2.3225364685058594
node_6647: feature_name=APP	APP.value <= threshold=4.124613881111145
node_6648: feature_name=FCER2	FCER2.value <= threshold=5.776939868927002
node_6649: feature_name=MEF2C	MEF2C.value <= threshold=3.8539518117904663
node_6650: feature_name=RHOB	RHOB.value > threshold=1.7790308594703674
node_6672: feature_name=CD79B	CD79B.value <= threshold=-0.3273680806159973
Class: COVID-19 Severe	
Rules_1082	passed counts:3
node_0: feature_name=LTB	LTB.value > threshold=0.994031548500061
node_5264: feature_name=MT-CO1	MT-CO1.value > threshold=-0.4127991199493408
node_5638: feature_name=OAZ1	OAZ1.value > threshold=-0.2640867084264755
node_6112: feature_name=IFI44L	IFI44L.value <= threshold=2.5893263816833496
node_6113: feature_name=TCL1A	TCL1A.value <= threshold=4.201832294464111
node_6114: feature_name=HLA-DQA2	HLA-DQA2.value > threshold=-0.2270243540406227
node_6542: feature_name=CD79B	CD79B.value <= threshold=3.096232771873474
node_6543: feature_name=NR4A1	NR4A1.value <= threshold=1.421015739440918
node_6544: feature_name=LTB	LTB.value <= threshold=1.359755277633667
node_6545: feature_name=NFKBID	NFKBID.value > threshold=1.4294711351394653
node_6643: feature_name=FAM129C	FAM129C.value <= threshold=4.799884080886841
node_6644: feature_name=XIST	XIST.value <= threshold=0.9931697957217693
node_6645: feature_name=GPX1	GPX1.value <= threshold=0.3628883808851242
node_6646: feature_name=STMN1	STMN1.value <= threshold=2.3225364685058594
node_6647: feature_name=APP	APP.value <= threshold=4.124613881111145
node_6648: feature_name=FCER2	FCER2.value <= threshold=5.776939868927002

node_6649: feature_name=MEF2C	MEF2C.value <= threshold=3.8539518117904663
node_6650: feature_name=RHOB	RHOB.value <= threshold=1.7790308594703674
node_6651: feature_name=HSPA1A	HSPA1A.value <= threshold=3.537449836730957
node_6652: feature_name=MTRNR2L2	MTRNR2L2.value <= threshold=3.4327136278152466
node_6653: feature_name=MT-CO3	MT-CO3.value > threshold=-2.1226823925971985
node_6655: feature_name=TLR2	TLR2.value <= threshold=1.0477308258414268
node_6656: feature_name=PLEC	PLEC.value > threshold=2.035267412662506
node_6666: feature_name=HSPA1A	HSPA1A.value > threshold=-0.22247668355703354
Class: COVID-19 Critical	
Rules_1083	passed counts:3
node_0: feature_name=LTB	LTB.value > threshold=0.994031548500061
node_5264: feature_name=MT-CO1	MT-CO1.value > threshold=-0.4127991199493408
node_5638: feature_name=OAZ1	OAZ1.value > threshold=-0.2640867084264755
node_6112: feature_name=IFI44L	IFI44L.value <= threshold=2.5893263816833496
node_6113: feature_name=TCL1A	TCL1A.value <= threshold=4.201832294464111
node_6114: feature_name=HLA-DQA2	HLA-DQA2.value > threshold=-0.2270243540406227
node_6542: feature_name=CD79B	CD79B.value <= threshold=3.096232771873474
node_6543: feature_name=NR4A1	NR4A1.value <= threshold=1.421015739440918
node_6544: feature_name=LTB	LTB.value <= threshold=1.359755277633667
node_6545: feature_name=NFKBID	NFKBID.value > threshold=1.4294711351394653
node_6643: feature_name=FAM129C	FAM129C.value <= threshold=4.799884080886841
node_6644: feature_name=XIST	XIST.value <= threshold=0.9931697957217693
node_6645: feature_name=GPX1	GPX1.value <= threshold=0.3628883808851242
node_6646: feature_name=STMN1	STMN1.value <= threshold=2.3225364685058594
node_6647: feature_name=APP	APP.value <= threshold=4.124613881111145
node_6648: feature_name=FCER2	FCER2.value <= threshold=5.776939868927002
node_6649: feature_name=MEF2C	MEF2C.value <= threshold=3.8539518117904663
node_6650: feature_name=RHOB	RHOB.value <= threshold=1.7790308594703674
node_6651: feature_name=HSPA1A	HSPA1A.value <= threshold=3.537449836730957

node_6652: feature_name=MTRNR2L2	MTRNR2L2.value <= threshold=3.4327136278152466
node_6653: feature_name=MT-CO3	MT-CO3.value > threshold=-2.1226823925971985
node_6655: feature_name=TLR2	TLR2.value <= threshold=1.0477308258414268
node_6656: feature_name=PLEC	PLEC.value <= threshold=2.035267412662506
node_6657: feature_name=IGJ	IGJ.value > threshold=-0.38578981161117554
node_6661: feature_name=PPIF	PPIF.value > threshold=1.9370648860931396
node_6663: feature_name=BCL2A1	BCL2A1.value > threshold=-0.48100417852401733
Class: COVID-19 Critical	
Rules_1084	passed counts:3
node_0: feature_name=LTB	LTB.value > threshold=0.994031548500061
node_5264: feature_name=MT-CO1	MT-CO1.value > threshold=-0.4127991199493408
node_5638: feature_name=OAZ1	OAZ1.value > threshold=-0.2640867084264755
node_6112: feature_name=IFI44L	IFI44L.value <= threshold=2.5893263816833496
node_6113: feature_name=TCL1A	TCL1A.value <= threshold=4.201832294464111
node_6114: feature_name=HLA-DQA2	HLA-DQA2.value > threshold=-0.2270243540406227
node_6542: feature_name=CD79B	CD79B.value <= threshold=3.096232771873474
node_6543: feature_name=NR4A1	NR4A1.value <= threshold=1.421015739440918
node_6544: feature_name=LTB	LTB.value <= threshold=1.359755277633667
node_6545: feature_name=NFKBID	NFKBID.value > threshold=1.4294711351394653
node_6643: feature_name=FAM129C	FAM129C.value <= threshold=4.799884080886841
node_6644: feature_name=XIST	XIST.value <= threshold=0.9931697957217693
node_6645: feature_name=GPX1	GPX1.value <= threshold=0.3628883808851242
node_6646: feature_name=STMN1	STMN1.value <= threshold=2.3225364685058594
node_6647: feature_name=APP	APP.value <= threshold=4.124613881111145
node_6648: feature_name=FCER2	FCER2.value <= threshold=5.776939868927002
node_6649: feature_name=MEF2C	MEF2C.value <= threshold=3.8539518117904663
node_6650: feature_name=RHOB	RHOB.value <= threshold=1.7790308594703674
node_6651: feature_name=HSPA1A	HSPA1A.value <= threshold=3.537449836730957
node_6652: feature_name=MTRNR2L2	MTRNR2L2.value <= threshold=3.4327136278152466

node_6653: feature_name=MT-CO3	MT-CO3.value > threshold=-2.1226823925971985
node_6655: feature_name=TLR2	TLR2.value <= threshold=1.0477308258414268
node_6656: feature_name=PLEC	PLEC.value <= threshold=2.035267412662506
node_6657: feature_name=IGJ	IGJ.value <= threshold=-0.38578981161117554
node_6658: feature_name=EGR1	EGR1.value > threshold=-0.3111751079559326
Class: COVID-19 Critical	
Rules_1085	passed counts:3
node_0: feature_name=LTB	LTB.value > threshold=0.994031548500061
node_5264: feature_name=MT-CO1	MT-CO1.value > threshold=-0.4127991199493408
node_5638: feature_name=OAZ1	OAZ1.value > threshold=-0.2640867084264755
node_6112: feature_name=IFI44L	IFI44L.value <= threshold=2.5893263816833496
node_6113: feature_name=TCL1A	TCL1A.value <= threshold=4.201832294464111
node_6114: feature_name=HLA-DQA2	HLA-DQA2.value > threshold=-0.2270243540406227
node_6542: feature_name=CD79B	CD79B.value <= threshold=3.096232771873474
node_6543: feature_name=NR4A1	NR4A1.value <= threshold=1.421015739440918
node_6544: feature_name=LTB	LTB.value <= threshold=1.359755277633667
node_6545: feature_name=NFKBID	NFKBID.value <= threshold=1.4294711351394653
node_6546: feature_name=HLA-DRB1	HLA-DRB1.value > threshold=1.0398632287979126
node_6556: feature_name=CD79B	CD79B.value > threshold=-0.2919560521841049
node_6582: feature_name=PELI1	PELI1.value > threshold=-0.6156984269618988
node_6602: feature_name=MT-CO3	MT-CO3.value <= threshold=0.46141014993190765
node_6603: feature_name=NFKBIZ	NFKBIZ.value <= threshold=0.6470938920974731
node_6604: feature_name=HSP90B1	HSP90B1.value <= threshold=0.4268011748790741
node_6605: feature_name=FCER2	FCER2.value > threshold=-0.2290492281317711
node_6613: feature_name=HLA-DRB5	HLA-DRB5.value > threshold=2.3765095472335815
Class: COVID-19 Critical	
Rules_1086	passed counts:3
node_0: feature_name=LTB	LTB.value > threshold=0.994031548500061

node_5264: feature_name=MT-CO1	MT-CO1.value > threshold=-0.4127991199493408
node_5638: feature_name=OAZ1	OAZ1.value > threshold=-0.2640867084264755
node_6112: feature_name=IFI44L	IFI44L.value <= threshold=2.5893263816833496
node_6113: feature_name=TCL1A	TCL1A.value <= threshold=4.201832294464111
node_6114: feature_name=HLA-DQA2	HLA-DQA2.value > threshold=-0.2270243540406227
node_6542: feature_name=CD79B	CD79B.value <= threshold=3.096232771873474
node_6543: feature_name=NR4A1	NR4A1.value <= threshold=1.421015739440918
node_6544: feature_name=LTB	LTB.value <= threshold=1.359755277633667
node_6545: feature_name=NFKBID	NFKBID.value <= threshold=1.4294711351394653
node_6546: feature_name=HLA-DRB1	HLA-DRB1.value > threshold=1.0398632287979126
node_6556: feature_name=CD79B	CD79B.value > threshold=-0.2919560521841049
node_6582: feature_name=PELI1	PELI1.value > threshold=-0.6156984269618988
node_6602: feature_name=MT-CO3	MT-CO3.value <= threshold=0.46141014993190765
node_6603: feature_name=NFKBIZ	NFKBIZ.value <= threshold=0.6470938920974731
node_6604: feature_name=HSP90B1	HSP90B1.value <= threshold=0.4268011748790741
node_6605: feature_name=FCER2	FCER2.value <= threshold=-0.2290492281317711
node_6606: feature_name=HLA-DPB1	HLA-DPB1.value > threshold=1.5359395146369934
node_6608: feature_name=BANK1	BANK1.value <= threshold=2.5472620725631714
Class: COVID-19 Severe	
Rules_1087	passed counts:3
node_0: feature_name=LTB	LTB.value > threshold=0.994031548500061
node_5264: feature_name=MT-CO1	MT-CO1.value > threshold=-0.4127991199493408
node_5638: feature_name=OAZ1	OAZ1.value > threshold=-0.2640867084264755
node_6112: feature_name=IFI44L	IFI44L.value <= threshold=2.5893263816833496
node_6113: feature_name=TCL1A	TCL1A.value <= threshold=4.201832294464111
node_6114: feature_name=HLA-DQA2	HLA-DQA2.value > threshold=-0.2270243540406227
node_6542: feature_name=CD79B	CD79B.value <= threshold=3.096232771873474
node_6543: feature_name=NR4A1	NR4A1.value <= threshold=1.421015739440918
node_6544: feature_name=LTB	LTB.value <= threshold=1.359755277633667

node_6545: feature_name=NFKBID	NFKBID.value <= threshold=1.4294711351394653
node_6546: feature_name=HLA-DRB1	HLA-DRB1.value > threshold=1.0398632287979126
node_6556: feature_name=CD79B	CD79B.value > threshold=-0.2919560521841049
node_6582: feature_name=PELI1	PELI1.value <= threshold=-0.6156984269618988
node_6583: feature_name=LTB	LTB.value > threshold=1.0177513360977173
node_6585: feature_name=CD22	CD22.value <= threshold=-0.24851123988628387
node_6586: feature_name=OAZ1	OAZ1.value <= threshold=0.9554091989994049
node_6587: feature_name=RP11-685N3.1	RP11-685N3.1.value > threshold=-0.16386442631483078
Class: COVID-19 Severe	
Rules_1088	passed counts:3
node_0: feature_name=LTB	LTB.value > threshold=0.994031548500061
node_5264: feature_name=MT-CO1	MT-CO1.value > threshold=-0.4127991199493408
node_5638: feature_name=OAZ1	OAZ1.value > threshold=-0.2640867084264755
node_6112: feature_name=IFI44L	IFI44L.value <= threshold=2.5893263816833496
node_6113: feature_name=TCL1A	TCL1A.value <= threshold=4.201832294464111
node_6114: feature_name=HLA-DQA2	HLA-DQA2.value > threshold=-0.2270243540406227
node_6542: feature_name=CD79B	CD79B.value <= threshold=3.096232771873474
node_6543: feature_name=NR4A1	NR4A1.value <= threshold=1.421015739440918
node_6544: feature_name=LTB	LTB.value <= threshold=1.359755277633667
node_6545: feature_name=NFKBID	NFKBID.value <= threshold=1.4294711351394653
node_6546: feature_name=HLA-DRB1	HLA-DRB1.value > threshold=1.0398632287979126
node_6556: feature_name=CD79B	CD79B.value > threshold=-0.2919560521841049
node_6582: feature_name=PELI1	PELI1.value <= threshold=-0.6156984269618988
node_6583: feature_name=LTB	LTB.value <= threshold=1.0177513360977173
Class: COVID-19 Critical	
Rules_1089	passed counts:3
node_0: feature_name=LTB	LTB.value > threshold=0.994031548500061
node_5264: feature_name=MT-CO1	MT-CO1.value > threshold=-0.4127991199493408

node_5638: feature_name=OAZ1	OAZ1.value > threshold=-0.2640867084264755
node_6112: feature_name=IFI44L	IFI44L.value <= threshold=2.5893263816833496
node_6113: feature_name=TCL1A	TCL1A.value <= threshold=4.201832294464111
node_6114: feature_name=HLA-DQA2	HLA-DQA2.value > threshold=-0.2270243540406227
node_6542: feature_name=CD79B	CD79B.value <= threshold=3.096232771873474
node_6543: feature_name=NR4A1	NR4A1.value <= threshold=1.421015739440918
node_6544: feature_name=LTB	LTB.value <= threshold=1.359755277633667
node_6545: feature_name=NFKBID	NFKBID.value <= threshold=1.4294711351394653
node_6546: feature_name=HLA-DRB1	HLA-DRB1.value > threshold=1.0398632287979126
node_6556: feature_name=CD79B	CD79B.value <= threshold=-0.2919560521841049
node_6557: feature_name=MTRNR2L2	MTRNR2L2.value <= threshold=2.7710195183753967
node_6558: feature_name=JUN	JUN.value <= threshold=0.5767147243022919
node_6559: feature_name=TLN1	TLN1.value > threshold=-0.08378086984157562
node_6569: feature_name=RALGPS2	RALGPS2.value <= threshold=2.019797205924988
node_6570: feature_name=COTL1	COTL1.value > threshold=-0.4552314691245556
Class: COVID-19 Critical	
Rules_1090	passed counts:3
node_0: feature_name=LTB	LTB.value > threshold=0.994031548500061
node_5264: feature_name=MT-CO1	MT-CO1.value > threshold=-0.4127991199493408
node_5638: feature_name=OAZ1	OAZ1.value > threshold=-0.2640867084264755
node_6112: feature_name=IFI44L	IFI44L.value <= threshold=2.5893263816833496
node_6113: feature_name=TCL1A	TCL1A.value <= threshold=4.201832294464111
node_6114: feature_name=HLA-DQA2	HLA-DQA2.value <= threshold=-0.2270243540406227
node_6115: feature_name=MTRNR2L2	MTRNR2L2.value <= threshold=2.548917293548584
node_6116: feature_name=ATF5	ATF5.value > threshold=2.041263461112976
node_6490: feature_name=HLA-DPB1	HLA-DPB1.value > threshold=1.5343759655952454
node_6508: feature_name=OAZ1	OAZ1.value > threshold=0.21525950729846954
node_6514: feature_name=NT5C3A	NT5C3A.value > threshold=-0.556839257478714
node_6516: feature_name=S100A6	S100A6.value <= threshold=-1.8120158910751343

Class: COVID-19 Critical	
Rules_1091	passed counts:3
node_0: feature_name=LTB	LTB.value > threshold=0.994031548500061
node_5264: feature_name=MT-CO1	MT-CO1.value > threshold=-0.4127991199493408
node_5638: feature_name=OAZ1	OAZ1.value > threshold=-0.2640867084264755
node_6112: feature_name=IFI44L	IFI44L.value <= threshold=2.5893263816833496
node_6113: feature_name=TCL1A	TCL1A.value <= threshold=4.201832294464111
node_6114: feature_name=HLA-DQA2	HLA-DQA2.value <= threshold=-0.2270243540406227
node_6115: feature_name=MTRNR2L2	MTRNR2L2.value <= threshold=2.548917293548584
node_6116: feature_name=ATF5	ATF5.value > threshold=2.041263461112976
node_6490: feature_name=HLA-DPB1	HLA-DPB1.value > threshold=1.5343759655952454
node_6508: feature_name=OAZ1	OAZ1.value > threshold=0.21525950729846954
node_6514: feature_name=NT5C3A	NT5C3A.value <= threshold=-0.556839257478714
Class: COVID-19 Critical	
Rules_1092	passed counts:3
node_0: feature_name=LTB	LTB.value > threshold=0.994031548500061
node_5264: feature_name=MT-CO1	MT-CO1.value > threshold=-0.4127991199493408
node_5638: feature_name=OAZ1	OAZ1.value > threshold=-0.2640867084264755
node_6112: feature_name=IFI44L	IFI44L.value <= threshold=2.5893263816833496
node_6113: feature_name=TCL1A	TCL1A.value <= threshold=4.201832294464111
node_6114: feature_name=HLA-DQA2	HLA-DQA2.value <= threshold=-0.2270243540406227
node_6115: feature_name=MTRNR2L2	MTRNR2L2.value <= threshold=2.548917293548584
node_6116: feature_name=ATF5	ATF5.value > threshold=2.041263461112976
node_6490: feature_name=HLA-DPB1	HLA-DPB1.value <= threshold=1.5343759655952454
node_6491: feature_name=MT-ND6	MT-ND6.value <= threshold=1.0284687876701355
node_6492: feature_name=FAM129C	FAM129C.value <= threshold=3.5579545497894287
node_6493: feature_name=WDR74	WDR74.value <= threshold=1.888875424861908
node_6494: feature_name=HSP90B1	HSP90B1.value <= threshold=-1.1869465708732605

node_6495: feature_name=MARCKS	MARCKS.value <= threshold=2.911969542503357
Class: Healthy Control	
Rules_1093	passed counts:3
node_0: feature_name=LTB	LTB.value > threshold=0.994031548500061
node_5264: feature_name=MT-CO1	MT-CO1.value > threshold=-0.4127991199493408
node_5638: feature_name=OAZ1	OAZ1.value > threshold=-0.2640867084264755
node_6112: feature_name=IFI44L	IFI44L.value <= threshold=2.5893263816833496
node_6113: feature_name=TCL1A	TCL1A.value <= threshold=4.201832294464111
node_6114: feature_name=HLA-DQA2	HLA-DQA2.value <= threshold=-0.2270243540406227
node_6115: feature_name=MTRNR2L2	MTRNR2L2.value <= threshold=2.548917293548584
node_6116: feature_name=ATF5	ATF5.value <= threshold=2.041263461112976
node_6117: feature_name=JUN	JUN.value > threshold=-0.616259902715683
node_6189: feature_name=MT-CO3	MT-CO3.value > threshold=0.13483521342277527
node_6351: feature_name=XAF1	XAF1.value <= threshold=3.061717987060547
node_6352: feature_name=MT-CO2	MT-CO2.value > threshold=1.2605181336402893
node_6476: feature_name=ATF5	ATF5.value > threshold=-0.3569986820220947
node_6482: feature_name=CYBB	CYBB.value > threshold=-0.4226558953523636
Class: Healthy Control	
Rules_1094	passed counts:3
node_0: feature_name=LTB	LTB.value > threshold=0.994031548500061
node_5264: feature_name=MT-CO1	MT-CO1.value > threshold=-0.4127991199493408
node_5638: feature_name=OAZ1	OAZ1.value > threshold=-0.2640867084264755
node_6112: feature_name=IFI44L	IFI44L.value <= threshold=2.5893263816833496
node_6113: feature_name=TCL1A	TCL1A.value <= threshold=4.201832294464111
node_6114: feature_name=HLA-DQA2	HLA-DQA2.value <= threshold=-0.2270243540406227
node_6115: feature_name=MTRNR2L2	MTRNR2L2.value <= threshold=2.548917293548584
node_6116: feature_name=ATF5	ATF5.value <= threshold=2.041263461112976
node_6117: feature_name=JUN	JUN.value > threshold=-0.616259902715683

node_6189: feature_name=MT-CO3	MT-CO3.value > threshold=0.13483521342277527
node_6351: feature_name=XAF1	XAF1.value <= threshold=3.061717987060547
node_6352: feature_name=MT-CO2	MT-CO2.value <= threshold=1.2605181336402893
node_6353: feature_name=ICAM1	ICAM1.value <= threshold=-0.3340446650981903
node_6354: feature_name=OAZ1	OAZ1.value > threshold=-0.24055946618318558
node_6360: feature_name=PPIF	PPIF.value > threshold=2.070030093193054
Class: COVID-19 Critical	
Rules_1095	passed counts:3
node_0: feature_name=LTB	LTB.value > threshold=0.994031548500061
node_5264: feature_name=MT-CO1	MT-CO1.value > threshold=-0.4127991199493408
node_5638: feature_name=OAZ1	OAZ1.value > threshold=-0.2640867084264755
node_6112: feature_name=IFI44L	IFI44L.value <= threshold=2.5893263816833496
node_6113: feature_name=TCL1A	TCL1A.value <= threshold=4.201832294464111
node_6114: feature_name=HLA-DQA2	HLA-DQA2.value <= threshold=-0.2270243540406227
node_6115: feature_name=MTRNR2L2	MTRNR2L2.value <= threshold=2.548917293548584
node_6116: feature_name=ATF5	ATF5.value <= threshold=2.041263461112976
node_6117: feature_name=JUN	JUN.value > threshold=-0.616259902715683
node_6189: feature_name=MT-CO3	MT-CO3.value > threshold=0.13483521342277527
node_6351: feature_name=XAF1	XAF1.value <= threshold=3.061717987060547
node_6352: feature_name=MT-CO2	MT-CO2.value <= threshold=1.2605181336402893
node_6353: feature_name=ICAM1	ICAM1.value <= threshold=-0.3340446650981903
node_6354: feature_name=OAZ1	OAZ1.value > threshold=-0.24055946618318558
node_6360: feature_name=PPIF	PPIF.value <= threshold=2.070030093193054
node_6361: feature_name=RALGPS2	RALGPS2.value > threshold=-0.3684315085411072
node_6369: feature_name=ANO6	ANO6.value <= threshold=3.504153251647949
node_6370: feature_name=YWHAH	YWHAH.value <= threshold=4.3182291984558105
node_6371: feature_name=LDLR	LDLR.value <= threshold=2.494291305541992
node_6372: feature_name=HLA-DQA1	HLA-DQA1.value > threshold=-0.5716990232467651
node_6374: feature_name=ABCA1	ABCA1.value <= threshold=3.7662240266799927

node_6375: feature_name=MS4A1	MS4A1.value > threshold=1.4995208978652954
node_6387: feature_name=SNORD3B-2	SNORD3B-2.value > threshold=4.154061555862427
node_6431: feature_name=GPX1	GPX1.value <= threshold=-0.1492191143333912
Class: Healthy Control	
Rules_1096	passed counts:3
node_0: feature_name=LTB	LTB.value > threshold=0.994031548500061
node_5264: feature_name=MT-CO1	MT-CO1.value > threshold=-0.4127991199493408
node_5638: feature_name=OAZ1	OAZ1.value > threshold=-0.2640867084264755
node_6112: feature_name=IFI44L	IFI44L.value <= threshold=2.5893263816833496
node_6113: feature_name=TCL1A	TCL1A.value <= threshold=4.201832294464111
node_6114: feature_name=HLA-DQA2	HLA-DQA2.value <= threshold=-0.2270243540406227
node_6115: feature_name=MTRNR2L2	MTRNR2L2.value <= threshold=2.548917293548584
node_6116: feature_name=ATF5	ATF5.value <= threshold=2.041263461112976
node_6117: feature_name=JUN	JUN.value > threshold=-0.616259902715683
node_6189: feature_name=MT-CO3	MT-CO3.value > threshold=0.13483521342277527
node_6351: feature_name=XAF1	XAF1.value <= threshold=3.061717987060547
node_6352: feature_name=MT-CO2	MT-CO2.value <= threshold=1.2605181336402893
node_6353: feature_name=ICAM1	ICAM1.value <= threshold=-0.3340446650981903
node_6354: feature_name=OAZ1	OAZ1.value > threshold=-0.24055946618318558
node_6360: feature_name=PPIF	PPIF.value <= threshold=2.070030093193054
node_6361: feature_name=RALGPS2	RALGPS2.value > threshold=-0.3684315085411072
node_6369: feature_name=ANO6	ANO6.value <= threshold=3.504153251647949
node_6370: feature_name=YWHAH	YWHAH.value <= threshold=4.3182291984558105
node_6371: feature_name=LDLR	LDLR.value <= threshold=2.494291305541992
node_6372: feature_name=HLA-DQA1	HLA-DQA1.value > threshold=-0.5716990232467651
node_6374: feature_name=ABCA1	ABCA1.value <= threshold=3.7662240266799927
node_6375: feature_name=MS4A1	MS4A1.value > threshold=1.4995208978652954
node_6387: feature_name=SNORD3B-2	SNORD3B-2.value <= threshold=4.154061555862427
node_6388: feature_name=LTB	LTB.value <= threshold=2.1739100217819214

node_6389: feature_name=BACH1	BACH1.value <= threshold=2.0371285676956177
node_6390: feature_name=HLA-DQB1	HLA-DQB1.value <= threshold=3.0258322954177856
node_6391: feature_name=HLA-DPB1	HLA-DPB1.value > threshold=0.2591289207339287
node_6395: feature_name=CXorf57	CXorf57.value <= threshold=3.3742183446884155
node_6396: feature_name=MMD	MMD.value <= threshold=3.8883349895477295
node_6397: feature_name=IRF8	IRF8.value <= threshold=3.939465045928955
node_6398: feature_name=PPIF	PPIF.value > threshold=1.1284508109092712
node_6408: feature_name=JUN	JUN.value <= threshold=-0.1225685365498066
node_6409: feature_name=FTH1	FTH1.value <= threshold=-0.6591627895832062
Class: COVID-19 Critical	
Rules_1097	passed counts:3
node_0: feature_name=LTB	LTB.value > threshold=0.994031548500061
node_5264: feature_name=MT-CO1	MT-CO1.value > threshold=-0.4127991199493408
node_5638: feature_name=OAZ1	OAZ1.value > threshold=-0.2640867084264755
node_6112: feature_name=IFI44L	IFI44L.value <= threshold=2.5893263816833496
node_6113: feature_name=TCL1A	TCL1A.value <= threshold=4.201832294464111
node_6114: feature_name=HLA-DQA2	HLA-DQA2.value <= threshold=-0.2270243540406227
node_6115: feature_name=MTRNR2L2	MTRNR2L2.value <= threshold=2.548917293548584
node_6116: feature_name=ATF5	ATF5.value <= threshold=2.041263461112976
node_6117: feature_name=JUN	JUN.value > threshold=-0.616259902715683
node_6189: feature_name=MT-CO3	MT-CO3.value > threshold=0.13483521342277527
node_6351: feature_name=XAF1	XAF1.value <= threshold=3.061717987060547
node_6352: feature_name=MT-CO2	MT-CO2.value <= threshold=1.2605181336402893
node_6353: feature_name=ICAM1	ICAM1.value <= threshold=-0.3340446650981903
node_6354: feature_name=OAZ1	OAZ1.value > threshold=-0.24055946618318558
node_6360: feature_name=PPIF	PPIF.value <= threshold=2.070030093193054
node_6361: feature_name=RALGPS2	RALGPS2.value > threshold=-0.3684315085411072
node_6369: feature_name=ANO6	ANO6.value <= threshold=3.504153251647949
node_6370: feature_name=YWHAH	YWHAH.value <= threshold=4.3182291984558105

node_6371: feature_name=LDLR	LDLR.value <= threshold=2.494291305541992
node_6372: feature_name=HLA-DQA1	HLA-DQA1.value > threshold=-0.5716990232467651
node_6374: feature_name=ABCA1	ABCA1.value <= threshold=3.7662240266799927
node_6375: feature_name=MS4A1	MS4A1.value <= threshold=1.4995208978652954
node_6376: feature_name=MEF2A	MEF2A.value > threshold=1.7946665287017822
node_6384: feature_name=BOD1L1	BOD1L1.value > threshold=-0.47899603843688965
Class: COVID-19 Critical	
Rules_1098	passed counts:3
node_0: feature_name=LTB	LTB.value > threshold=0.994031548500061
node_5264: feature_name=MT-CO1	MT-CO1.value > threshold=-0.4127991199493408
node_5638: feature_name=OAZ1	OAZ1.value > threshold=-0.2640867084264755
node_6112: feature_name=IFI44L	IFI44L.value <= threshold=2.5893263816833496
node_6113: feature_name=TCL1A	TCL1A.value <= threshold=4.201832294464111
node_6114: feature_name=HLA-DQA2	HLA-DQA2.value <= threshold=-0.2270243540406227
node_6115: feature_name=MTRNR2L2	MTRNR2L2.value <= threshold=2.548917293548584
node_6116: feature_name=ATF5	ATF5.value <= threshold=2.041263461112976
node_6117: feature_name=JUN	JUN.value > threshold=-0.616259902715683
node_6189: feature_name=MT-CO3	MT-CO3.value <= threshold=0.13483521342277527
node_6190: feature_name=XAF1	XAF1.value <= threshold=2.584952235221863
node_6191: feature_name=HLA-DRB5	HLA-DRB5.value > threshold=0.6955901980400085
node_6239: feature_name=TNFAIP3	TNFAIP3.value > threshold=0.11930759996175766
node_6341: feature_name=PELI1	PELI1.value <= threshold=-0.6198236048221588
node_6342: feature_name=AREGB	AREGB.value > threshold=-0.11352234333753586
Class: Healthy Control	
Rules_1099	passed counts:3
node_0: feature_name=LTB	LTB.value > threshold=0.994031548500061
node_5264: feature_name=MT-CO1	MT-CO1.value > threshold=-0.4127991199493408
node_5638: feature_name=OAZ1	OAZ1.value > threshold=-0.2640867084264755

node_6112: feature_name=IFI44L	IFI44L.value <= threshold=2.5893263816833496
node_6113: feature_name=TCL1A	TCL1A.value <= threshold=4.201832294464111
node_6114: feature_name=HLA-DQA2	HLA-DQA2.value <= threshold=-0.2270243540406227
node_6115: feature_name=MTRNR2L2	MTRNR2L2.value <= threshold=2.548917293548584
node_6116: feature_name=ATF5	ATF5.value <= threshold=2.041263461112976
node_6117: feature_name=JUN	JUN.value > threshold=-0.616259902715683
node_6189: feature_name=MT-CO3	MT-CO3.value <= threshold=0.13483521342277527
node_6190: feature_name=XAF1	XAF1.value <= threshold=2.584952235221863
node_6191: feature_name=HLA-DRB5	HLA-DRB5.value > threshold=0.6955901980400085
node_6239: feature_name=TNFAIP3	TNFAIP3.value <= threshold=0.11930759996175766
node_6240: feature_name=TP53INP1	TP53INP1.value <= threshold=2.9654784202575684
node_6241: feature_name=HLA-DRB5	HLA-DRB5.value <= threshold=2.5141202211380005
node_6242: feature_name=NFKBID	NFKBID.value > threshold=3.358878970146179
node_6320: feature_name=IFI6	IFI6.value <= threshold=-0.5788501501083374
Class: COVID-19 Critical	
Rules_1100	passed counts:3
node_0: feature_name=LTB	LTB.value > threshold=0.994031548500061
node_5264: feature_name=MT-CO1	MT-CO1.value > threshold=-0.4127991199493408
node_5638: feature_name=OAZ1	OAZ1.value > threshold=-0.2640867084264755
node_6112: feature_name=IFI44L	IFI44L.value <= threshold=2.5893263816833496
node_6113: feature_name=TCL1A	TCL1A.value <= threshold=4.201832294464111
node_6114: feature_name=HLA-DQA2	HLA-DQA2.value <= threshold=-0.2270243540406227
node_6115: feature_name=MTRNR2L2	MTRNR2L2.value <= threshold=2.548917293548584
node_6116: feature_name=ATF5	ATF5.value <= threshold=2.041263461112976
node_6117: feature_name=JUN	JUN.value > threshold=-0.616259902715683
node_6189: feature_name=MT-CO3	MT-CO3.value <= threshold=0.13483521342277527
node_6190: feature_name=XAF1	XAF1.value <= threshold=2.584952235221863
node_6191: feature_name=HLA-DRB5	HLA-DRB5.value > threshold=0.6955901980400085
node_6239: feature_name=TNFAIP3	TNFAIP3.value <= threshold=0.11930759996175766

node_6240: feature_name=TP53INP1	TP53INP1.value <= threshold=2.9654784202575684
node_6241: feature_name=HLA-DRB5	HLA-DRB5.value <= threshold=2.5141202211380005
node_6242: feature_name=NFKBID	NFKBID.value <= threshold=3.358878970146179
node_6243: feature_name=JARID2	JARID2.value > threshold=1.7160060405731201
node_6317: feature_name=RBKS	RBKS.value <= threshold=2.184031158685684
Class: COVID-19 Critical	
Rules_1101	passed counts:3
node_0: feature_name=LTB	LTB.value > threshold=0.994031548500061
node_5264: feature_name=MT-CO1	MT-CO1.value > threshold=-0.4127991199493408
node_5638: feature_name=OAZ1	OAZ1.value > threshold=-0.2640867084264755
node_6112: feature_name=IFI44L	IFI44L.value <= threshold=2.5893263816833496
node_6113: feature_name=TCL1A	TCL1A.value <= threshold=4.201832294464111
node_6114: feature_name=HLA-DQA2	HLA-DQA2.value <= threshold=-0.2270243540406227
node_6115: feature_name=MTRNR2L2	MTRNR2L2.value <= threshold=2.548917293548584
node_6116: feature_name=ATF5	ATF5.value <= threshold=2.041263461112976
node_6117: feature_name=JUN	JUN.value > threshold=-0.616259902715683
node_6189: feature_name=MT-CO3	MT-CO3.value <= threshold=0.13483521342277527
node_6190: feature_name=XAF1	XAF1.value <= threshold=2.584952235221863
node_6191: feature_name=HLA-DRB5	HLA-DRB5.value > threshold=0.6955901980400085
node_6239: feature_name=TNFAIP3	TNFAIP3.value <= threshold=0.11930759996175766
node_6240: feature_name=TP53INP1	TP53INP1.value <= threshold=2.9654784202575684
node_6241: feature_name=HLA-DRB5	HLA-DRB5.value <= threshold=2.5141202211380005
node_6242: feature_name=NFKBID	NFKBID.value <= threshold=3.358878970146179
node_6243: feature_name=JARID2	JARID2.value <= threshold=1.7160060405731201
node_6244: feature_name=HLA-DPA1	HLA-DPA1.value > threshold=0.49491266906261444
node_6246: feature_name=MIR24-2	MIR24-2.value <= threshold=1.8348549008369446
node_6247: feature_name=HSP90B1	HSP90B1.value <= threshold=1.8861054182052612
node_6248: feature_name=NR4A1	NR4A1.value > threshold=-0.5434737503528595
node_6274: feature_name=CD79A	CD79A.value > threshold=1.2718155980110168

node_6278: feature_name=RP5-821D11.7	RP5-821D11.7.value <= threshold=4.768282890319824
node_6279: feature_name=FAM160B1	FAM160B1.value <= threshold=2.6933162212371826
node_6280: feature_name=ICAM1	ICAM1.value <= threshold=3.578627824783325
node_6281: feature_name=CASP8	CASP8.value <= threshold=1.7487988471984863
node_6282: feature_name=TRAF1	TRAF1.value <= threshold=3.18716561794281
node_6283: feature_name=RNF11	RNF11.value <= threshold=1.9705576300621033
node_6284: feature_name=SNX9	SNX9.value <= threshold=3.318251371383667
node_6285: feature_name=PARP14	PARP14.value <= threshold=2.4342962503433228
node_6286: feature_name=GPX1	GPX1.value <= threshold=1.119508445262909
node_6287: feature_name=IRF8	IRF8.value > threshold=3.6463273763656616
node_6293: feature_name=UTY	UTY.value <= threshold=0.49591951072216034
Class: Healthy Control	
Rules_1102	passed counts:3
node_0: feature_name=LTB	LTB.value > threshold=0.994031548500061
node_5264: feature_name=MT-CO1	MT-CO1.value > threshold=-0.4127991199493408
node_5638: feature_name=OAZ1	OAZ1.value > threshold=-0.2640867084264755
node_6112: feature_name=IFI44L	IFI44L.value <= threshold=2.5893263816833496
node_6113: feature_name=TCL1A	TCL1A.value <= threshold=4.201832294464111
node_6114: feature_name=HLA-DQA2	HLA-DQA2.value <= threshold=-0.2270243540406227
node_6115: feature_name=MTRNR2L2	MTRNR2L2.value <= threshold=2.548917293548584
node_6116: feature_name=ATF5	ATF5.value <= threshold=2.041263461112976
node_6117: feature_name=JUN	JUN.value > threshold=-0.616259902715683
node_6189: feature_name=MT-CO3	MT-CO3.value <= threshold=0.13483521342277527
node_6190: feature_name=XAF1	XAF1.value <= threshold=2.584952235221863
node_6191: feature_name=HLA-DRB5	HLA-DRB5.value > threshold=0.6955901980400085
node_6239: feature_name=TNFAIP3	TNFAIP3.value <= threshold=0.11930759996175766
node_6240: feature_name=TP53INP1	TP53INP1.value <= threshold=2.9654784202575684
node_6241: feature_name=HLA-DRB5	HLA-DRB5.value <= threshold=2.5141202211380005
node_6242: feature_name=NFKBID	NFKBID.value <= threshold=3.358878970146179

node_6243: feature_name=JARID2	JARID2.value <= threshold=1.7160060405731201
node_6244: feature_name=HLA-DPA1	HLA-DPA1.value > threshold=0.49491266906261444
node_6246: feature_name=MIR24-2	MIR24-2.value <= threshold=1.8348549008369446
node_6247: feature_name=HSP90B1	HSP90B1.value <= threshold=1.8861054182052612
node_6248: feature_name=NR4A1	NR4A1.value <= threshold=-0.5434737503528595
node_6249: feature_name=MT-CO1	MT-CO1.value > threshold=0.002460678806528449
node_6261: feature_name=PLD4	PLD4.value <= threshold=5.953886985778809
node_6262: feature_name=STAG3	STAG3.value <= threshold=6.350517749786377
node_6263: feature_name=AHR	AHR.value <= threshold=1.2266070246696472
node_6264: feature_name=ITM2C	ITM2C.value > threshold=3.805675506591797
node_6266: feature_name=JUN	JUN.value > threshold=1.0442078709602356
Class: Healthy Control	
Rules_1103	passed counts:3
node_0: feature_name=LTB	LTB.value > threshold=0.994031548500061
node_5264: feature_name=MT-CO1	MT-CO1.value > threshold=-0.4127991199493408
node_5638: feature_name=OAZ1	OAZ1.value > threshold=-0.2640867084264755
node_6112: feature_name=IFI44L	IFI44L.value <= threshold=2.5893263816833496
node_6113: feature_name=TCL1A	TCL1A.value <= threshold=4.201832294464111
node_6114: feature_name=HLA-DQA2	HLA-DQA2.value <= threshold=-0.2270243540406227
node_6115: feature_name=MTRNR2L2	MTRNR2L2.value <= threshold=2.548917293548584
node_6116: feature_name=ATF5	ATF5.value <= threshold=2.041263461112976
node_6117: feature_name=JUN	JUN.value > threshold=-0.616259902715683
node_6189: feature_name=MT-CO3	MT-CO3.value <= threshold=0.13483521342277527
node_6190: feature_name=XAF1	XAF1.value <= threshold=2.584952235221863
node_6191: feature_name=HLA-DRB5	HLA-DRB5.value <= threshold=0.6955901980400085
node_6192: feature_name=NR4A1	NR4A1.value <= threshold=-0.4496448189020157
node_6193: feature_name=HLA-DPB1	HLA-DPB1.value <= threshold=1.7609722018241882
node_6194: feature_name=JUN	JUN.value <= threshold=1.172904908657074
node_6195: feature_name=MT-ND6	MT-ND6.value <= threshold=0.6287261247634888

node_6196: feature_name=ACSL1	ACSL1.value > threshold=-0.31894539296627045
node_6202: feature_name=RGS2	RGS2.value <= threshold=-0.6272018253803253
Class: Healthy Control	
Rules_1104	passed counts:3
node_0: feature_name=LTB	LTB.value > threshold=0.994031548500061
node_5264: feature_name=MT-CO1	MT-CO1.value > threshold=-0.4127991199493408
node_5638: feature_name=OAZ1	OAZ1.value > threshold=-0.2640867084264755
node_6112: feature_name=IFI44L	IFI44L.value <= threshold=2.5893263816833496
node_6113: feature_name=TCL1A	TCL1A.value <= threshold=4.201832294464111
node_6114: feature_name=HLA-DQA2	HLA-DQA2.value <= threshold=-0.2270243540406227
node_6115: feature_name=MTRNR2L2	MTRNR2L2.value <= threshold=2.548917293548584
node_6116: feature_name=ATF5	ATF5.value <= threshold=2.041263461112976
node_6117: feature_name=JUN	JUN.value <= threshold=-0.616259902715683
node_6118: feature_name=NFKBID	NFKBID.value > threshold=1.5264258980751038
node_6166: feature_name=NR4A1	NR4A1.value > threshold=0.6553736329078674
node_6184: feature_name=RP5-821D11.7	RP5-821D11.7.value > threshold=-0.22415155917406082
node_6186: feature_name=CD83	CD83.value <= threshold=2.3596288561820984
Class: COVID-19 Critical	
Rules_1105	passed counts:3
node_0: feature_name=LTB	LTB.value > threshold=0.994031548500061
node_5264: feature_name=MT-CO1	MT-CO1.value > threshold=-0.4127991199493408
node_5638: feature_name=OAZ1	OAZ1.value > threshold=-0.2640867084264755
node_6112: feature_name=IFI44L	IFI44L.value <= threshold=2.5893263816833496
node_6113: feature_name=TCL1A	TCL1A.value <= threshold=4.201832294464111
node_6114: feature_name=HLA-DQA2	HLA-DQA2.value <= threshold=-0.2270243540406227
node_6115: feature_name=MTRNR2L2	MTRNR2L2.value <= threshold=2.548917293548584
node_6116: feature_name=ATF5	ATF5.value <= threshold=2.041263461112976
node_6117: feature_name=JUN	JUN.value <= threshold=-0.616259902715683

node_6118: feature_name=NFKBID	NFKBID.value > threshold=1.5264258980751038
node_6166: feature_name=NR4A1	NR4A1.value <= threshold=0.6553736329078674
node_6167: feature_name=WDR74	WDR74.value <= threshold=-0.49743133783340454
node_6168: feature_name=APP	APP.value <= threshold=0.5775387734174728
node_6169: feature_name=HLA-DQB1	HLA-DQB1.value > threshold=1.6636562943458557
node_6171: feature_name=TCF4	TCF4.value <= threshold=-0.27397148311138153
Class: COVID-19 Critical	
Rules_1106	passed counts:3
node_0: feature_name=LTB	LTB.value > threshold=0.994031548500061
node_5264: feature_name=MT-CO1	MT-CO1.value > threshold=-0.4127991199493408
node_5638: feature_name=OAZ1	OAZ1.value > threshold=-0.2640867084264755
node_6112: feature_name=IFI44L	IFI44L.value <= threshold=2.5893263816833496
node_6113: feature_name=TCL1A	TCL1A.value <= threshold=4.201832294464111
node_6114: feature_name=HLA-DQA2	HLA-DQA2.value <= threshold=-0.2270243540406227
node_6115: feature_name=MTRNR2L2	MTRNR2L2.value <= threshold=2.548917293548584
node_6116: feature_name=ATF5	ATF5.value <= threshold=2.041263461112976
node_6117: feature_name=JUN	JUN.value <= threshold=-0.616259902715683
node_6118: feature_name=NFKBID	NFKBID.value <= threshold=1.5264258980751038
node_6119: feature_name=MT-CO1	MT-CO1.value > threshold=0.1564532369375229
node_6133: feature_name=LTB	LTB.value > threshold=1.2431999444961548
node_6149: feature_name=MT-CO1	MT-CO1.value > threshold=1.1254711747169495
node_6163: feature_name=MEF2A	MEF2A.value > threshold=1.4936169385910034
Class: COVID-19 Critical	
Rules_1107	passed counts:3
node_0: feature_name=LTB	LTB.value > threshold=0.994031548500061
node_5264: feature_name=MT-CO1	MT-CO1.value > threshold=-0.4127991199493408
node_5638: feature_name=OAZ1	OAZ1.value > threshold=-0.2640867084264755
node_6112: feature_name=IFI44L	IFI44L.value <= threshold=2.5893263816833496

node_6113: feature_name=TCL1A	TCL1A.value <= threshold=4.201832294464111
node_6114: feature_name=HLA-DQA2	HLA-DQA2.value <= threshold=-0.2270243540406227
node_6115: feature_name=MTRNR2L2	MTRNR2L2.value <= threshold=2.548917293548584
node_6116: feature_name=ATF5	ATF5.value <= threshold=2.041263461112976
node_6117: feature_name=JUN	JUN.value <= threshold=-0.616259902715683
node_6118: feature_name=NFKBID	NFKBID.value <= threshold=1.5264258980751038
node_6119: feature_name=MT-CO1	MT-CO1.value > threshold=0.1564532369375229
node_6133: feature_name=LTB	LTB.value > threshold=1.2431999444961548
node_6149: feature_name=MT-CO1	MT-CO1.value > threshold=1.1254711747169495
node_6163: feature_name=MEF2A	MEF2A.value <= threshold=1.4936169385910034
Class: Healthy Control	
Rules_1108	passed counts:3
node_0: feature_name=LTB	LTB.value > threshold=0.994031548500061
node_5264: feature_name=MT-CO1	MT-CO1.value > threshold=-0.4127991199493408
node_5638: feature_name=OAZ1	OAZ1.value > threshold=-0.2640867084264755
node_6112: feature_name=IFI44L	IFI44L.value <= threshold=2.5893263816833496
node_6113: feature_name=TCL1A	TCL1A.value <= threshold=4.201832294464111
node_6114: feature_name=HLA-DQA2	HLA-DQA2.value <= threshold=-0.2270243540406227
node_6115: feature_name=MTRNR2L2	MTRNR2L2.value <= threshold=2.548917293548584
node_6116: feature_name=ATF5	ATF5.value <= threshold=2.041263461112976
node_6117: feature_name=JUN	JUN.value <= threshold=-0.616259902715683
node_6118: feature_name=NFKBID	NFKBID.value <= threshold=1.5264258980751038
node_6119: feature_name=MT-CO1	MT-CO1.value > threshold=0.1564532369375229
node_6133: feature_name=LTB	LTB.value > threshold=1.2431999444961548
node_6149: feature_name=MT-CO1	MT-CO1.value <= threshold=1.1254711747169495
node_6150: feature_name=RP5-821D11.7	RP5-821D11.7.value <= threshold=2.683489441871643
node_6151: feature_name=SLC7A5	SLC7A5.value <= threshold=1.7370842099189758
node_6152: feature_name=OAZ1	OAZ1.value > threshold=-0.12006772682070732
node_6156: feature_name=ICAM1	ICAM1.value > threshold=0.616551861166954

node_6158: feature_name=GRASP	GRASP.value > threshold=-0.4035773426294327
Class: Healthy Control	
Rules_1109	passed counts:3
node_0: feature_name=LTB	LTB.value > threshold=0.994031548500061
node_5264: feature_name=MT-CO1	MT-CO1.value > threshold=-0.4127991199493408
node_5638: feature_name=OAZ1	OAZ1.value > threshold=-0.2640867084264755
node_6112: feature_name=IFI44L	IFI44L.value <= threshold=2.5893263816833496
node_6113: feature_name=TCL1A	TCL1A.value <= threshold=4.201832294464111
node_6114: feature_name=HLA-DQA2	HLA-DQA2.value <= threshold=-0.2270243540406227
node_6115: feature_name=MTRNR2L2	MTRNR2L2.value <= threshold=2.548917293548584
node_6116: feature_name=ATF5	ATF5.value <= threshold=2.041263461112976
node_6117: feature_name=JUN	JUN.value <= threshold=-0.616259902715683
node_6118: feature_name=NFKBID	NFKBID.value <= threshold=1.5264258980751038
node_6119: feature_name=MT-CO1	MT-CO1.value > threshold=0.1564532369375229
node_6133: feature_name=LTB	LTB.value <= threshold=1.2431999444961548
node_6134: feature_name=MT-ND6	MT-ND6.value > threshold=0.5990295112133026
node_6144: feature_name=HSP90B1	HSP90B1.value > threshold=1.085684895515442
Class: COVID-19 Critical	
Rules_1110	passed counts:3
node_0: feature_name=LTB	LTB.value > threshold=0.994031548500061
node_5264: feature_name=MT-CO1	MT-CO1.value > threshold=-0.4127991199493408
node_5638: feature_name=OAZ1	OAZ1.value > threshold=-0.2640867084264755
node_6112: feature_name=IFI44L	IFI44L.value <= threshold=2.5893263816833496
node_6113: feature_name=TCL1A	TCL1A.value <= threshold=4.201832294464111
node_6114: feature_name=HLA-DQA2	HLA-DQA2.value <= threshold=-0.2270243540406227
node_6115: feature_name=MTRNR2L2	MTRNR2L2.value <= threshold=2.548917293548584
node_6116: feature_name=ATF5	ATF5.value <= threshold=2.041263461112976
node_6117: feature_name=JUN	JUN.value <= threshold=-0.616259902715683

node_6118: feature_name=NFKBID	NFKBID.value <= threshold=1.5264258980751038
node_6119: feature_name=MT-CO1	MT-CO1.value <= threshold=0.1564532369375229
node_6120: feature_name=HLA-DPA1	HLA-DPA1.value > threshold=1.569844663143158
node_6126: feature_name=CASP8	CASP8.value <= threshold=0.8988403975963593
node_6127: feature_name=IRF8	IRF8.value > threshold=2.923409581184387
node_6129: feature_name=ZNF10	ZNF10.value <= threshold=-0.2537892237305641
Class: COVID-19 Critical	
Rules_1111	passed counts:3
node_0: feature_name=LTB	LTB.value > threshold=0.994031548500061
node_5264: feature_name=MT-CO1	MT-CO1.value > threshold=-0.4127991199493408
node_5638: feature_name=OAZ1	OAZ1.value > threshold=-0.2640867084264755
node_6112: feature_name=IFI44L	IFI44L.value <= threshold=2.5893263816833496
node_6113: feature_name=TCL1A	TCL1A.value <= threshold=4.201832294464111
node_6114: feature_name=HLA-DQA2	HLA-DQA2.value <= threshold=-0.2270243540406227
node_6115: feature_name=MTRNR2L2	MTRNR2L2.value <= threshold=2.548917293548584
node_6116: feature_name=ATF5	ATF5.value <= threshold=2.041263461112976
node_6117: feature_name=JUN	JUN.value <= threshold=-0.616259902715683
node_6118: feature_name=NFKBID	NFKBID.value <= threshold=1.5264258980751038
node_6119: feature_name=MT-CO1	MT-CO1.value <= threshold=0.1564532369375229
node_6120: feature_name=HLA-DPA1	HLA-DPA1.value <= threshold=1.569844663143158
node_6121: feature_name=ADAM19	ADAM19.value > threshold=0.856033593416214
node_6123: feature_name=HLA-DRB5	HLA-DRB5.value <= threshold=1.6543100476264954
Class: Healthy Control	
Rules_1112	passed counts:3
node_0: feature_name=LTB	LTB.value > threshold=0.994031548500061
node_5264: feature_name=MT-CO1	MT-CO1.value > threshold=-0.4127991199493408
node_5638: feature_name=OAZ1	OAZ1.value <= threshold=-0.2640867084264755
node_5639: feature_name=CD79B	CD79B.value > threshold=2.2998517751693726

node_5925: feature_name=SNX9	SNX9.value > threshold=2.4445992708206177
node_6083: feature_name=RALGPS2	RALGPS2.value > threshold=4.061077356338501
node_6105: feature_name=XAF1	XAF1.value <= threshold=-0.4288857579231262
node_6106: feature_name=CD38	CD38.value > threshold=-0.25612112879753113
Class: COVID-19 Critical	
Rules_1113	passed counts:3
node_0: feature_name=LTB	LTB.value > threshold=0.994031548500061
node_5264: feature_name=MT-CO1	MT-CO1.value > threshold=-0.4127991199493408
node_5638: feature_name=OAZ1	OAZ1.value <= threshold=-0.2640867084264755
node_5639: feature_name=CD79B	CD79B.value > threshold=2.2998517751693726
node_5925: feature_name=SNX9	SNX9.value <= threshold=2.4445992708206177
node_5926: feature_name=TCL1A	TCL1A.value > threshold=2.6938670873641968
node_6052: feature_name=IGLL5	IGLL5.value > threshold=-0.26075856387615204
node_6054: feature_name=IFI44L	IFI44L.value > threshold=1.9543575644493103
node_6080: feature_name=CASP8	CASP8.value > threshold=-0.6888859868049622
Class: COVID-19 Critical	
Rules_1114	passed counts:3
node_0: feature_name=LTB	LTB.value > threshold=0.994031548500061
node_5264: feature_name=MT-CO1	MT-CO1.value > threshold=-0.4127991199493408
node_5638: feature_name=OAZ1	OAZ1.value <= threshold=-0.2640867084264755
node_5639: feature_name=CD79B	CD79B.value > threshold=2.2998517751693726
node_5925: feature_name=SNX9	SNX9.value <= threshold=2.4445992708206177
node_5926: feature_name=TCL1A	TCL1A.value > threshold=2.6938670873641968
node_6052: feature_name=IGLL5	IGLL5.value <= threshold=-0.26075856387615204
Class: COVID-19 Critical	
Rules_1115	passed counts:3
node_0: feature_name=LTB	LTB.value > threshold=0.994031548500061

node_5264: feature_name=MT-CO1	MT-CO1.value > threshold=-0.4127991199493408
node_5638: feature_name=OAZ1	OAZ1.value <= threshold=-0.2640867084264755
node_5639: feature_name=CD79B	CD79B.value > threshold=2.2998517751693726
node_5925: feature_name=SNX9	SNX9.value <= threshold=2.4445992708206177
node_5926: feature_name=TCL1A	TCL1A.value <= threshold=2.6938670873641968
node_5927: feature_name=PLD4	PLD4.value > threshold=-0.06434785947203636
node_6009: feature_name=MX1	MX1.value <= threshold=3.600893020629883
node_6010: feature_name=PRDM1	PRDM1.value <= threshold=-0.407244011759758
node_6011: feature_name=SNORD3B-2	SNORD3B-2.value <= threshold=2.7082353830337524
node_6012: feature_name=NFKBID	NFKBID.value > threshold=3.0716724395751953
node_6034: feature_name=BANK1	BANK1.value > threshold=2.832552671432495
node_6036: feature_name=MAP3K2	MAP3K2.value > threshold=2.1622473001480103
Class: COVID-19 Critical	
Rules_1116	passed counts:3
node_0: feature_name=LTB	LTB.value > threshold=0.994031548500061
node_5264: feature_name=MT-CO1	MT-CO1.value > threshold=-0.4127991199493408
node_5638: feature_name=OAZ1	OAZ1.value <= threshold=-0.2640867084264755
node_5639: feature_name=CD79B	CD79B.value > threshold=2.2998517751693726
node_5925: feature_name=SNX9	SNX9.value <= threshold=2.4445992708206177
node_5926: feature_name=TCL1A	TCL1A.value <= threshold=2.6938670873641968
node_5927: feature_name=PLD4	PLD4.value <= threshold=-0.06434785947203636
node_5928: feature_name=ICAM1	ICAM1.value <= threshold=-0.2820792943239212
node_5929: feature_name=MS4A1	MS4A1.value > threshold=2.747320055961609
node_5963: feature_name=RNF144B	RNF144B.value > threshold=-0.2324080914258957
node_5989: feature_name=YWHAH	YWHAH.value > threshold=-0.27859707176685333
node_5993: feature_name=CD83	CD83.value <= threshold=2.551318883895874
Class: Healthy Control	
Rules_1117	passed counts:3

node_0: feature_name=LTB	LTB.value > threshold=0.994031548500061
node_5264: feature_name=MT-CO1	MT-CO1.value > threshold=-0.4127991199493408
node_5638: feature_name=OAZ1	OAZ1.value <= threshold=-0.2640867084264755
node_5639: feature_name=CD79B	CD79B.value > threshold=2.2998517751693726
node_5925: feature_name=SNX9	SNX9.value <= threshold=2.4445992708206177
node_5926: feature_name=TCL1A	TCL1A.value <= threshold=2.6938670873641968
node_5927: feature_name=PLD4	PLD4.value <= threshold=-0.06434785947203636
node_5928: feature_name=ICAM1	ICAM1.value <= threshold=-0.2820792943239212
node_5929: feature_name=MS4A1	MS4A1.value > threshold=2.747320055961609
node_5963: feature_name=RNF144B	RNF144B.value <= threshold=-0.2324080914258957
node_5964: feature_name=HLA-DRB5	HLA-DRB5.value <= threshold=2.0430362224578857
node_5965: feature_name=MX1	MX1.value <= threshold=1.2721255421638489
node_5966: feature_name=LDLRAD4	LDLRAD4.value <= threshold=2.3228561878204346
node_5967: feature_name=LTB	LTB.value <= threshold=1.1045992970466614
node_5968: feature_name=PARP14	PARP14.value <= threshold=-0.44172780215740204
node_5969: feature_name=BACH1	BACH1.value > threshold=0.13615700602531433
Class: COVID-19 Critical	
Rules_1118	passed counts:3
node_0: feature_name=LTB	LTB.value > threshold=0.994031548500061
node_5264: feature_name=MT-CO1	MT-CO1.value > threshold=-0.4127991199493408
node_5638: feature_name=OAZ1	OAZ1.value <= threshold=-0.2640867084264755
node_5639: feature_name=CD79B	CD79B.value > threshold=2.2998517751693726
node_5925: feature_name=SNX9	SNX9.value <= threshold=2.4445992708206177
node_5926: feature_name=TCL1A	TCL1A.value <= threshold=2.6938670873641968
node_5927: feature_name=PLD4	PLD4.value <= threshold=-0.06434785947203636
node_5928: feature_name=ICAM1	ICAM1.value <= threshold=-0.2820792943239212
node_5929: feature_name=MS4A1	MS4A1.value <= threshold=2.747320055961609
node_5930: feature_name=LTB	LTB.value > threshold=1.3533939123153687
node_5954: feature_name=HLA-DPB1	HLA-DPB1.value > threshold=1.5530678033828735

node_5958: feature_name=FCER2	FCER2.value <= threshold=-0.24438364803791046
Class: COVID-19 Critical	
Rules_1119	passed counts:3
node_0: feature_name=LTB	LTB.value > threshold=0.994031548500061
node_5264: feature_name=MT-CO1	MT-CO1.value > threshold=-0.4127991199493408
node_5638: feature_name=OAZ1	OAZ1.value <= threshold=-0.2640867084264755
node_5639: feature_name=CD79B	CD79B.value <= threshold=2.2998517751693726
node_5640: feature_name=MTRNR2L2	MTRNR2L2.value <= threshold=3.5499863624572754
node_5641: feature_name=MT-CO1	MT-CO1.value > threshold=0.22567133605480194
node_5729: feature_name=MARCH1	MARCH1.value > threshold=1.5154643654823303
node_5867: feature_name=NFKBID	NFKBID.value <= threshold=2.6947624683380127
node_5868: feature_name=UTY	UTY.value <= threshold=3.29777729511261
node_5869: feature_name=IFI44L	IFI44L.value <= threshold=-0.29640993475914
node_5870: feature_name=FTH1	FTH1.value > threshold=0.6966870129108429
node_5896: feature_name=HLA-DQB1	HLA-DQB1.value <= threshold=2.877285599708557
Class: COVID-19 Critical	
Rules_1120	passed counts:3
node_0: feature_name=LTB	LTB.value > threshold=0.994031548500061
node_5264: feature_name=MT-CO1	MT-CO1.value > threshold=-0.4127991199493408
node_5638: feature_name=OAZ1	OAZ1.value <= threshold=-0.2640867084264755
node_5639: feature_name=CD79B	CD79B.value <= threshold=2.2998517751693726
node_5640: feature_name=MTRNR2L2	MTRNR2L2.value <= threshold=3.5499863624572754
node_5641: feature_name=MT-CO1	MT-CO1.value > threshold=0.22567133605480194
node_5729: feature_name=MARCH1	MARCH1.value > threshold=1.5154643654823303
node_5867: feature_name=NFKBID	NFKBID.value <= threshold=2.6947624683380127
node_5868: feature_name=UTY	UTY.value <= threshold=3.29777729511261
node_5869: feature_name=IFI44L	IFI44L.value <= threshold=-0.29640993475914
node_5870: feature_name=FTH1	FTH1.value <= threshold=0.6966870129108429

node_5871: feature_name=ILK	ILK.value <= threshold=1.5719919204711914
node_5872: feature_name=MARCKS	MARCKS.value > threshold=1.20343416929245
node_5882: feature_name=RALGPS2	RALGPS2.value <= threshold=2.361847758293152
node_5883: feature_name=PELI1	PELI1.value <= threshold=1.1635615229606628
node_5884: feature_name=RGS1	RGS1.value > threshold=-0.5901600122451782
Class: COVID-19 Severe	
Rules_1121	passed counts:3
node_0: feature_name=LTB	LTB.value > threshold=0.994031548500061
node_5264: feature_name=MT-CO1	MT-CO1.value > threshold=-0.4127991199493408
node_5638: feature_name=OAZ1	OAZ1.value <= threshold=-0.2640867084264755
node_5639: feature_name=CD79B	CD79B.value <= threshold=2.2998517751693726
node_5640: feature_name=MTRNR2L2	MTRNR2L2.value <= threshold=3.5499863624572754
node_5641: feature_name=MT-CO1	MT-CO1.value > threshold=0.22567133605480194
node_5729: feature_name=MARCH1	MARCH1.value <= threshold=1.5154643654823303
node_5730: feature_name=MT-CO1	MT-CO1.value > threshold=1.7823500633239746
node_5856: feature_name=TTN	TTN.value > threshold=-0.38264039158821106
node_5862: feature_name=BACH1	BACH1.value > threshold=-0.4962465167045593
node_5864: feature_name=FAM129C	FAM129C.value > threshold=-0.29338543117046356
Class: COVID-19 Severe	
Rules_1122	passed counts:3
node_0: feature_name=LTB	LTB.value > threshold=0.994031548500061
node_5264: feature_name=MT-CO1	MT-CO1.value > threshold=-0.4127991199493408
node_5638: feature_name=OAZ1	OAZ1.value <= threshold=-0.2640867084264755
node_5639: feature_name=CD79B	CD79B.value <= threshold=2.2998517751693726
node_5640: feature_name=MTRNR2L2	MTRNR2L2.value <= threshold=3.5499863624572754
node_5641: feature_name=MT-CO1	MT-CO1.value > threshold=0.22567133605480194
node_5729: feature_name=MARCH1	MARCH1.value <= threshold=1.5154643654823303
node_5730: feature_name=MT-CO1	MT-CO1.value <= threshold=1.7823500633239746

node_5731: feature_name=HLA-DPB1	HLA-DPB1.value > threshold=2.0096163749694824
node_5829: feature_name=LTB	LTB.value > threshold=1.2124510407447815
node_5843: feature_name=TTN	TTN.value > threshold=-0.3821280151605606
node_5851: feature_name=SNX9	SNX9.value > threshold=1.9138266444206238
node_5853: feature_name=DENND3	DENND3.value > threshold=-0.3534839004278183
Class: Healthy Control	
Rules_1123	passed counts:3
node_0: feature_name=LTB	LTB.value > threshold=0.994031548500061
node_5264: feature_name=MT-CO1	MT-CO1.value > threshold=-0.4127991199493408
node_5638: feature_name=OAZ1	OAZ1.value <= threshold=-0.2640867084264755
node_5639: feature_name=CD79B	CD79B.value <= threshold=2.2998517751693726
node_5640: feature_name=MTRNR2L2	MTRNR2L2.value <= threshold=3.5499863624572754
node_5641: feature_name=MT-CO1	MT-CO1.value > threshold=0.22567133605480194
node_5729: feature_name=MARCH1	MARCH1.value <= threshold=1.5154643654823303
node_5730: feature_name=MT-CO1	MT-CO1.value <= threshold=1.7823500633239746
node_5731: feature_name=HLA-DPB1	HLA-DPB1.value > threshold=2.0096163749694824
node_5829: feature_name=LTB	LTB.value > threshold=1.2124510407447815
node_5843: feature_name=TTN	TTN.value <= threshold=-0.3821280151605606
node_5844: feature_name=FAM129C	FAM129C.value <= threshold=6.290640115737915
node_5845: feature_name=FCER2	FCER2.value <= threshold=-0.27513572573661804
Class: COVID-19 Severe	
Rules_1124	passed counts:3
node_0: feature_name=LTB	LTB.value > threshold=0.994031548500061
node_5264: feature_name=MT-CO1	MT-CO1.value > threshold=-0.4127991199493408
node_5638: feature_name=OAZ1	OAZ1.value <= threshold=-0.2640867084264755
node_5639: feature_name=CD79B	CD79B.value <= threshold=2.2998517751693726
node_5640: feature_name=MTRNR2L2	MTRNR2L2.value <= threshold=3.5499863624572754
node_5641: feature_name=MT-CO1	MT-CO1.value > threshold=0.22567133605480194

node_5729: feature_name=MARCH1	MARCH1.value <= threshold=1.5154643654823303
node_5730: feature_name=MT-CO1	MT-CO1.value <= threshold=1.7823500633239746
node_5731: feature_name=HLA-DPB1	HLA-DPB1.value > threshold=2.0096163749694824
node_5829: feature_name=LTB	LTB.value <= threshold=1.2124510407447815
node_5830: feature_name=MT-CO1	MT-CO1.value > threshold=0.41994138062000275
node_5834: feature_name=WDR74	WDR74.value > threshold=-0.48832663893699646
node_5838: feature_name=IRF8	IRF8.value <= threshold=-0.29013653099536896
Class: Healthy Control	
Rules_1125	passed counts:3
node_0: feature_name=LTB	LTB.value > threshold=0.994031548500061
node_5264: feature_name=MT-CO1	MT-CO1.value > threshold=-0.4127991199493408
node_5638: feature_name=OAZ1	OAZ1.value <= threshold=-0.2640867084264755
node_5639: feature_name=CD79B	CD79B.value <= threshold=2.2998517751693726
node_5640: feature_name=MTRNR2L2	MTRNR2L2.value <= threshold=3.5499863624572754
node_5641: feature_name=MT-CO1	MT-CO1.value > threshold=0.22567133605480194
node_5729: feature_name=MARCH1	MARCH1.value <= threshold=1.5154643654823303
node_5730: feature_name=MT-CO1	MT-CO1.value <= threshold=1.7823500633239746
node_5731: feature_name=HLA-DPB1	HLA-DPB1.value <= threshold=2.0096163749694824
node_5732: feature_name=NR4A3	NR4A3.value > threshold=3.509189486503601
node_5822: feature_name=BANK1	BANK1.value > threshold=2.9566380977630615
node_5824: feature_name=RALGPS2	RALGPS2.value > threshold=-0.4092569053173065
Class: Healthy Control	
Rules_1126	passed counts:3
node_0: feature_name=LTB	LTB.value > threshold=0.994031548500061
node_5264: feature_name=MT-CO1	MT-CO1.value > threshold=-0.4127991199493408
node_5638: feature_name=OAZ1	OAZ1.value <= threshold=-0.2640867084264755
node_5639: feature_name=CD79B	CD79B.value <= threshold=2.2998517751693726
node_5640: feature_name=MTRNR2L2	MTRNR2L2.value <= threshold=3.5499863624572754

node_5641: feature_name=MT-CO1	MT-CO1.value > threshold=0.22567133605480194
node_5729: feature_name=MARCH1	MARCH1.value <= threshold=1.5154643654823303
node_5730: feature_name=MT-CO1	MT-CO1.value <= threshold=1.7823500633239746
node_5731: feature_name=HLA-DPB1	HLA-DPB1.value <= threshold=2.0096163749694824
node_5732: feature_name=NR4A3	NR4A3.value <= threshold=3.509189486503601
node_5733: feature_name=OAZ1	OAZ1.value > threshold=-0.4392261207103729
node_5803: feature_name=BACH1	BACH1.value <= threshold=1.211863934993744
node_5804: feature_name=MEF2A	MEF2A.value > threshold=0.8741590976715088
node_5808: feature_name=ATF3	ATF3.value <= threshold=-0.38448040187358856
node_5809: feature_name=CYBB	CYBB.value > threshold=-0.5348981320858002
node_5811: feature_name=STMN1	STMN1.value <= threshold=-0.2834131717681885
Class: Healthy Control	
Rules_1127	passed counts:3
node_0: feature_name=LTB	LTB.value > threshold=0.994031548500061
node_5264: feature_name=MT-CO1	MT-CO1.value > threshold=-0.4127991199493408
node_5638: feature_name=OAZ1	OAZ1.value <= threshold=-0.2640867084264755
node_5639: feature_name=CD79B	CD79B.value <= threshold=2.2998517751693726
node_5640: feature_name=MTRNR2L2	MTRNR2L2.value <= threshold=3.5499863624572754
node_5641: feature_name=MT-CO1	MT-CO1.value > threshold=0.22567133605480194
node_5729: feature_name=MARCH1	MARCH1.value <= threshold=1.5154643654823303
node_5730: feature_name=MT-CO1	MT-CO1.value <= threshold=1.7823500633239746
node_5731: feature_name=HLA-DPB1	HLA-DPB1.value <= threshold=2.0096163749694824
node_5732: feature_name=NR4A3	NR4A3.value <= threshold=3.509189486503601
node_5733: feature_name=OAZ1	OAZ1.value <= threshold=-0.4392261207103729
node_5734: feature_name=SESN3	SESN3.value <= threshold=-0.45661941170692444
node_5735: feature_name=WDR74	WDR74.value > threshold=-0.4893670231103897
node_5761: feature_name=FCER2	FCER2.value > threshold=6.041448354721069
Class: Healthy Control	

Rules_1128	passed counts:3
node_0: feature_name=LTB	LTB.value > threshold=0.994031548500061
node_5264: feature_name=MT-CO1	MT-CO1.value > threshold=-0.4127991199493408
node_5638: feature_name=OAZ1	OAZ1.value <= threshold=-0.2640867084264755
node_5639: feature_name=CD79B	CD79B.value <= threshold=2.2998517751693726
node_5640: feature_name=MTRNR2L2	MTRNR2L2.value <= threshold=3.5499863624572754
node_5641: feature_name=MT-CO1	MT-CO1.value > threshold=0.22567133605480194
node_5729: feature_name=MARCH1	MARCH1.value <= threshold=1.5154643654823303
node_5730: feature_name=MT-CO1	MT-CO1.value <= threshold=1.7823500633239746
node_5731: feature_name=HLA-DPB1	HLA-DPB1.value <= threshold=2.0096163749694824
node_5732: feature_name=NR4A3	NR4A3.value <= threshold=3.509189486503601
node_5733: feature_name=OAZ1	OAZ1.value <= threshold=-0.4392261207103729
node_5734: feature_name=SESN3	SESN3.value <= threshold=-0.45661941170692444
node_5735: feature_name=WDR74	WDR74.value <= threshold=-0.4893670231103897
node_5736: feature_name=NR4A1	NR4A1.value > threshold=-0.2724379748106003
node_5754: feature_name=NAMPT	NAMPT.value > threshold=1.352931261062622
Class: COVID-19 Critical	
Rules_1129	passed counts:3
node_0: feature_name=LTB	LTB.value > threshold=0.994031548500061
node_5264: feature_name=MT-CO1	MT-CO1.value > threshold=-0.4127991199493408
node_5638: feature_name=OAZ1	OAZ1.value <= threshold=-0.2640867084264755
node_5639: feature_name=CD79B	CD79B.value <= threshold=2.2998517751693726
node_5640: feature_name=MTRNR2L2	MTRNR2L2.value <= threshold=3.5499863624572754
node_5641: feature_name=MT-CO1	MT-CO1.value > threshold=0.22567133605480194
node_5729: feature_name=MARCH1	MARCH1.value <= threshold=1.5154643654823303
node_5730: feature_name=MT-CO1	MT-CO1.value <= threshold=1.7823500633239746
node_5731: feature_name=HLA-DPB1	HLA-DPB1.value <= threshold=2.0096163749694824
node_5732: feature_name=NR4A3	NR4A3.value <= threshold=3.509189486503601
node_5733: feature_name=OAZ1	OAZ1.value <= threshold=-0.4392261207103729

node_5734: feature_name=SESN3	SESN3.value <= threshold=-0.45661941170692444
node_5735: feature_name=WDR74	WDR74.value <= threshold=-0.4893670231103897
node_5736: feature_name=NR4A1	NR4A1.value <= threshold=-0.2724379748106003
node_5737: feature_name=HLA-DPA1	HLA-DPA1.value <= threshold=1.6106070280075073
node_5738: feature_name=HLA-DRB1	HLA-DRB1.value > threshold=1.5730668902397156
node_5740: feature_name=PLEC	PLEC.value > threshold=-0.4240385890007019
Class: Healthy Control	
Rules_1130	passed counts:3
node_0: feature_name=LTB	LTB.value > threshold=0.994031548500061
node_5264: feature_name=MT-CO1	MT-CO1.value > threshold=-0.4127991199493408
node_5638: feature_name=OAZ1	OAZ1.value <= threshold=-0.2640867084264755
node_5639: feature_name=CD79B	CD79B.value <= threshold=2.2998517751693726
node_5640: feature_name=MTRNR2L2	MTRNR2L2.value <= threshold=3.5499863624572754
node_5641: feature_name=MT-CO1	MT-CO1.value > threshold=0.22567133605480194
node_5729: feature_name=MARCH1	MARCH1.value <= threshold=1.5154643654823303
node_5730: feature_name=MT-CO1	MT-CO1.value <= threshold=1.7823500633239746
node_5731: feature_name=HLA-DPB1	HLA-DPB1.value <= threshold=2.0096163749694824
node_5732: feature_name=NR4A3	NR4A3.value <= threshold=3.509189486503601
node_5733: feature_name=OAZ1	OAZ1.value <= threshold=-0.4392261207103729
node_5734: feature_name=SESN3	SESN3.value <= threshold=-0.45661941170692444
node_5735: feature_name=WDR74	WDR74.value <= threshold=-0.4893670231103897
node_5736: feature_name=NR4A1	NR4A1.value <= threshold=-0.2724379748106003
node_5737: feature_name=HLA-DPA1	HLA-DPA1.value <= threshold=1.6106070280075073
node_5738: feature_name=HLA-DRB1	HLA-DRB1.value > threshold=1.5730668902397156
node_5740: feature_name=PLEC	PLEC.value <= threshold=-0.4240385890007019
node_5741: feature_name=RGCC	RGCC.value <= threshold=-0.17803636193275452
Class: COVID-19 Critical	
Rules_1131	passed counts:3

node_0: feature_name=LTB	LTB.value > threshold=0.994031548500061
node_5264: feature_name=MT-CO1	MT-CO1.value > threshold=-0.4127991199493408
node_5638: feature_name=OAZ1	OAZ1.value <= threshold=-0.2640867084264755
node_5639: feature_name=CD79B	CD79B.value <= threshold=2.2998517751693726
node_5640: feature_name=MTRNR2L2	MTRNR2L2.value <= threshold=3.5499863624572754
node_5641: feature_name=MT-CO1	MT-CO1.value <= threshold=0.22567133605480194
node_5642: feature_name=RGS2	RGS2.value > threshold=1.2649686336517334
node_5710: feature_name=HLA-DPB1	HLA-DPB1.value > threshold=1.09694242477417
node_5712: feature_name=PPIF	PPIF.value <= threshold=-0.4469884932041168
node_5713: feature_name=TCF4	TCF4.value <= threshold=-0.2837526798248291
node_5714: feature_name=FAM129C	FAM129C.value > threshold=2.940014958381653
Class: COVID-19 Critical	
Rules_1132	passed counts:3
node_0: feature_name=LTB	LTB.value > threshold=0.994031548500061
node_5264: feature_name=MT-CO1	MT-CO1.value > threshold=-0.4127991199493408
node_5638: feature_name=OAZ1	OAZ1.value <= threshold=-0.2640867084264755
node_5639: feature_name=CD79B	CD79B.value <= threshold=2.2998517751693726
node_5640: feature_name=MTRNR2L2	MTRNR2L2.value <= threshold=3.5499863624572754
node_5641: feature_name=MT-CO1	MT-CO1.value <= threshold=0.22567133605480194
node_5642: feature_name=RGS2	RGS2.value <= threshold=1.2649686336517334
node_5643: feature_name=TCL1A	TCL1A.value <= threshold=4.771023273468018
node_5644: feature_name=IGJ	IGJ.value <= threshold=3.91206431388855
node_5645: feature_name=AREGB	AREGB.value > threshold=1.5142876282334328
Class: Healthy Control	
Rules_1133	passed counts:3
node_0: feature_name=LTB	LTB.value > threshold=0.994031548500061
node_5264: feature_name=MT-CO1	MT-CO1.value > threshold=-0.4127991199493408
node_5638: feature_name=OAZ1	OAZ1.value <= threshold=-0.2640867084264755

node_5639: feature_name=CD79B	CD79B.value <= threshold=2.2998517751693726
node_5640: feature_name=MTRNR2L2	MTRNR2L2.value <= threshold=3.5499863624572754
node_5641: feature_name=MT-CO1	MT-CO1.value <= threshold=0.22567133605480194
node_5642: feature_name=RGS2	RGS2.value <= threshold=1.2649686336517334
node_5643: feature_name=TCL1A	TCL1A.value <= threshold=4.771023273468018
node_5644: feature_name=IGJ	IGJ.value <= threshold=3.91206431388855
node_5645: feature_name=AREGB	AREGB.value <= threshold=1.5142876282334328
node_5646: feature_name=HLA-DRB1	HLA-DRB1.value > threshold=-0.6253005266189575
node_5648: feature_name=ITM2C	ITM2C.value <= threshold=3.2904402017593384
node_5649: feature_name=PLA2G12A	PLA2G12A.value <= threshold=3.833786725997925
node_5650: feature_name=RGCC	RGCC.value <= threshold=-0.10016544535756111
node_5651: feature_name=CD79B	CD79B.value <= threshold=2.278481364250183
node_5652: feature_name=HIST1H2AC	HIST1H2AC.value <= threshold=3.4233893156051636
node_5653: feature_name=RHOB	RHOB.value <= threshold=4.956467151641846
node_5654: feature_name=CD83	CD83.value > threshold=2.970836043357849
node_5670: feature_name=HLA-DRB1	HLA-DRB1.value <= threshold=1.513556182384491
node_5671: feature_name=CD22	CD22.value <= threshold=1.2871938347816467
Class: COVID-19 Severe	
Rules_1134	passed counts:3
node_0: feature_name=LTB	LTB.value > threshold=0.994031548500061
node_5264: feature_name=MT-CO1	MT-CO1.value <= threshold=-0.4127991199493408
node_5265: feature_name=MTRNR2L2	MTRNR2L2.value <= threshold=2.4473838806152344
node_5266: feature_name=TCL1A	TCL1A.value <= threshold=5.787256956100464
node_5267: feature_name=PLD4	PLD4.value > threshold=3.544657826423645
node_5577: feature_name=MT-CO1	MT-CO1.value <= threshold=-0.6592064797878265
node_5578: feature_name=HOPX	HOPX.value > threshold=-0.47573523223400116
node_5584: feature_name=OAZ1	OAZ1.value <= threshold=0.5679030418395996
node_5585: feature_name=MS4A1	MS4A1.value > threshold=2.869006037712097
node_5595: feature_name=YWHAH	YWHAH.value <= threshold=-0.35980531573295593

Class: Healthy Control	
Rules_1135	passed counts:3
node_0: feature_name=LTB	LTB.value > threshold=0.994031548500061
node_5264: feature_name=MT-CO1	MT-CO1.value <= threshold=-0.4127991199493408
node_5265: feature_name=MTRNR2L2	MTRNR2L2.value <= threshold=2.4473838806152344
node_5266: feature_name=TCL1A	TCL1A.value <= threshold=5.787256956100464
node_5267: feature_name=PLD4	PLD4.value > threshold=3.544657826423645
node_5577: feature_name=MT-CO1	MT-CO1.value <= threshold=-0.6592064797878265
node_5578: feature_name=HOPX	HOPX.value > threshold=-0.47573523223400116
node_5584: feature_name=OAZ1	OAZ1.value <= threshold=0.5679030418395996
node_5585: feature_name=MS4A1	MS4A1.value <= threshold=2.869006037712097
node_5586: feature_name=MT-CO2	MT-CO2.value > threshold=-0.9090828001499176
node_5590: feature_name=PLD4	PLD4.value > threshold=5.566271543502808
node_5592: feature_name=IGJ	IGJ.value > threshold=0.7876871973276138
Class: Healthy Control	
Rules_1136	passed counts:3
node_0: feature_name=LTB	LTB.value > threshold=0.994031548500061
node_5264: feature_name=MT-CO1	MT-CO1.value <= threshold=-0.4127991199493408
node_5265: feature_name=MTRNR2L2	MTRNR2L2.value <= threshold=2.4473838806152344
node_5266: feature_name=TCL1A	TCL1A.value <= threshold=5.787256956100464
node_5267: feature_name=PLD4	PLD4.value > threshold=3.544657826423645
node_5577: feature_name=MT-CO1	MT-CO1.value <= threshold=-0.6592064797878265
node_5578: feature_name=HOPX	HOPX.value > threshold=-0.47573523223400116
node_5584: feature_name=OAZ1	OAZ1.value <= threshold=0.5679030418395996
node_5585: feature_name=MS4A1	MS4A1.value <= threshold=2.869006037712097
node_5586: feature_name=MT-CO2	MT-CO2.value <= threshold=-0.9090828001499176
node_5587: feature_name=AHR	AHR.value <= threshold=-0.46321140229701996
Class: COVID-19 Severe	

Rules_1137	passed counts:3
node_0: feature_name=LTB	LTB.value > threshold=0.994031548500061
node_5264: feature_name=MT-CO1	MT-CO1.value <= threshold=-0.4127991199493408
node_5265: feature_name=MTRNR2L2	MTRNR2L2.value <= threshold=2.4473838806152344
node_5266: feature_name=TCL1A	TCL1A.value <= threshold=5.787256956100464
node_5267: feature_name=PLD4	PLD4.value <= threshold=3.544657826423645
node_5268: feature_name=NR4A1	NR4A1.value > threshold=0.8490704298019409
node_5498: feature_name=HLA-DPA1	HLA-DPA1.value > threshold=1.9079211950302124
node_5556: feature_name=CD83	CD83.value > threshold=1.6135562062263489
node_5560: feature_name=OAZ1	OAZ1.value > threshold=0.11598343029618263
node_5568: feature_name=MT-CO1	MT-CO1.value <= threshold=-0.8431997299194336
node_5569: feature_name=VCL	VCL.value <= threshold=-0.3415001332759857
node_5570: feature_name=MT-CO2	MT-CO2.value > threshold=-0.04905248433351517
Class: Healthy Control	
Rules_1138	passed counts:3
node_0: feature_name=LTB	LTB.value > threshold=0.994031548500061
node_5264: feature_name=MT-CO1	MT-CO1.value <= threshold=-0.4127991199493408
node_5265: feature_name=MTRNR2L2	MTRNR2L2.value <= threshold=2.4473838806152344
node_5266: feature_name=TCL1A	TCL1A.value <= threshold=5.787256956100464
node_5267: feature_name=PLD4	PLD4.value <= threshold=3.544657826423645
node_5268: feature_name=NR4A1	NR4A1.value > threshold=0.8490704298019409
node_5498: feature_name=HLA-DPA1	HLA-DPA1.value > threshold=1.9079211950302124
node_5556: feature_name=CD83	CD83.value > threshold=1.6135562062263489
node_5560: feature_name=OAZ1	OAZ1.value > threshold=0.11598343029618263
node_5568: feature_name=MT-CO1	MT-CO1.value <= threshold=-0.8431997299194336
node_5569: feature_name=VCL	VCL.value <= threshold=-0.3415001332759857
node_5570: feature_name=MT-CO2	MT-CO2.value <= threshold=-0.04905248433351517
Class: COVID-19 Critical	

Rules_1139	passed counts:3
node_0: feature_name=LTB	LTB.value > threshold=0.994031548500061
node_5264: feature_name=MT-CO1	MT-CO1.value <= threshold=-0.4127991199493408
node_5265: feature_name=MTRNR2L2	MTRNR2L2.value <= threshold=2.4473838806152344
node_5266: feature_name=TCL1A	TCL1A.value <= threshold=5.787256956100464
node_5267: feature_name=PLD4	PLD4.value <= threshold=3.544657826423645
node_5268: feature_name=NR4A1	NR4A1.value > threshold=0.8490704298019409
node_5498: feature_name=HLA-DPA1	HLA-DPA1.value <= threshold=1.9079211950302124
node_5499: feature_name=ID3	ID3.value > threshold=-0.17505786567926407
node_5543: feature_name=BCL2A1	BCL2A1.value > threshold=-0.4924587309360504
node_5547: feature_name=IRF8	IRF8.value <= threshold=2.243783950805664
node_5548: feature_name=BOD1L1	BOD1L1.value > threshold=0.25003959238529205
node_5552: feature_name=MT-CO2	MT-CO2.value <= threshold=0.0684196949005127
Class: COVID-19 Severe	
Rules_1140	passed counts:3
node_0: feature_name=LTB	LTB.value > threshold=0.994031548500061
node_5264: feature_name=MT-CO1	MT-CO1.value <= threshold=-0.4127991199493408
node_5265: feature_name=MTRNR2L2	MTRNR2L2.value <= threshold=2.4473838806152344
node_5266: feature_name=TCL1A	TCL1A.value <= threshold=5.787256956100464
node_5267: feature_name=PLD4	PLD4.value <= threshold=3.544657826423645
node_5268: feature_name=NR4A1	NR4A1.value > threshold=0.8490704298019409
node_5498: feature_name=HLA-DPA1	HLA-DPA1.value <= threshold=1.9079211950302124
node_5499: feature_name=ID3	ID3.value <= threshold=-0.17505786567926407
node_5500: feature_name=PPIF	PPIF.value > threshold=-0.5180355906486511
node_5512: feature_name=PDLIM1	PDLIM1.value > threshold=2.5938297510147095
node_5526: feature_name=FTH1	FTH1.value > threshold=0.17593900486826897
node_5536: feature_name=BANK1	BANK1.value <= threshold=1.7925409078598022
node_5537: feature_name=APP	APP.value <= threshold=-0.3681751936674118

Class: COVID-19 Critical	
Rules_1141	passed counts:3
node_0: feature_name=LTB	LTB.value > threshold=0.994031548500061
node_5264: feature_name=MT-CO1	MT-CO1.value <= threshold=-0.4127991199493408
node_5265: feature_name=MTRNR2L2	MTRNR2L2.value <= threshold=2.4473838806152344
node_5266: feature_name=TCL1A	TCL1A.value <= threshold=5.787256956100464
node_5267: feature_name=PLD4	PLD4.value <= threshold=3.544657826423645
node_5268: feature_name=NR4A1	NR4A1.value > threshold=0.8490704298019409
node_5498: feature_name=HLA-DPA1	HLA-DPA1.value <= threshold=1.9079211950302124
node_5499: feature_name=ID3	ID3.value <= threshold=-0.17505786567926407
node_5500: feature_name=PPIF	PPIF.value > threshold=-0.5180355906486511
node_5512: feature_name=PDLIM1	PDLIM1.value > threshold=2.5938297510147095
node_5526: feature_name=FTH1	FTH1.value <= threshold=0.17593900486826897
node_5527: feature_name=MIR24-2	MIR24-2.value <= threshold=1.0514202117919922
node_5528: feature_name=CD22	CD22.value > threshold=3.0746376514434814
node_5530: feature_name=TNFAIP3	TNFAIP3.value > threshold=-1.024448037147522
Class: Healthy Control	
Rules_1142	passed counts:3
node_0: feature_name=LTB	LTB.value > threshold=0.994031548500061
node_5264: feature_name=MT-CO1	MT-CO1.value <= threshold=-0.4127991199493408
node_5265: feature_name=MTRNR2L2	MTRNR2L2.value <= threshold=2.4473838806152344
node_5266: feature_name=TCL1A	TCL1A.value <= threshold=5.787256956100464
node_5267: feature_name=PLD4	PLD4.value <= threshold=3.544657826423645
node_5268: feature_name=NR4A1	NR4A1.value > threshold=0.8490704298019409
node_5498: feature_name=HLA-DPA1	HLA-DPA1.value <= threshold=1.9079211950302124
node_5499: feature_name=ID3	ID3.value <= threshold=-0.17505786567926407
node_5500: feature_name=PPIF	PPIF.value > threshold=-0.5180355906486511
node_5512: feature_name=PDLIM1	PDLIM1.value > threshold=2.5938297510147095

node_5526: feature_name=FTH1	FTH1.value <= threshold=0.17593900486826897
node_5527: feature_name=MIR24-2	MIR24-2.value <= threshold=1.0514202117919922
node_5528: feature_name=CD22	CD22.value > threshold=3.0746376514434814
node_5530: feature_name=TNFAIP3	TNFAIP3.value <= threshold=-1.024448037147522
Class: COVID-19 Critical	
Rules_1143	passed counts:3
node_0: feature_name=LTB	LTB.value > threshold=0.994031548500061
node_5264: feature_name=MT-CO1	MT-CO1.value <= threshold=-0.4127991199493408
node_5265: feature_name=MTRNR2L2	MTRNR2L2.value <= threshold=2.4473838806152344
node_5266: feature_name=TCL1A	TCL1A.value <= threshold=5.787256956100464
node_5267: feature_name=PLD4	PLD4.value <= threshold=3.544657826423645
node_5268: feature_name=NR4A1	NR4A1.value > threshold=0.8490704298019409
node_5498: feature_name=HLA-DPA1	HLA-DPA1.value <= threshold=1.9079211950302124
node_5499: feature_name=ID3	ID3.value <= threshold=-0.17505786567926407
node_5500: feature_name=PPIF	PPIF.value > threshold=-0.5180355906486511
node_5512: feature_name=PDLIM1	PDLIM1.value <= threshold=2.5938297510147095
node_5513: feature_name=HLA-DRB5	HLA-DRB5.value <= threshold=-0.5597687363624573
node_5514: feature_name=FTH1	FTH1.value <= threshold=-0.15839225053787231
Class: COVID-19 Critical	
Rules_1144	passed counts:3
node_0: feature_name=LTB	LTB.value > threshold=0.994031548500061
node_5264: feature_name=MT-CO1	MT-CO1.value <= threshold=-0.4127991199493408
node_5265: feature_name=MTRNR2L2	MTRNR2L2.value <= threshold=2.4473838806152344
node_5266: feature_name=TCL1A	TCL1A.value <= threshold=5.787256956100464
node_5267: feature_name=PLD4	PLD4.value <= threshold=3.544657826423645
node_5268: feature_name=NR4A1	NR4A1.value > threshold=0.8490704298019409
node_5498: feature_name=HLA-DPA1	HLA-DPA1.value <= threshold=1.9079211950302124
node_5499: feature_name=ID3	ID3.value <= threshold=-0.17505786567926407

node_5500: feature_name=PPIF	PPIF.value <= threshold=-0.5180355906486511
node_5501: feature_name=LTB	LTB.value > threshold=1.0920450091362
node_5503: feature_name=SNX9	SNX9.value > threshold=-0.5426926016807556
node_5509: feature_name=FTH1	FTH1.value <= threshold=-1.0832746624946594
Class: COVID-19 Critical	
Rules_1145	passed counts:3
node_0: feature_name=LTB	LTB.value > threshold=0.994031548500061
node_5264: feature_name=MT-CO1	MT-CO1.value <= threshold=-0.4127991199493408
node_5265: feature_name=MTRNR2L2	MTRNR2L2.value <= threshold=2.4473838806152344
node_5266: feature_name=TCL1A	TCL1A.value <= threshold=5.787256956100464
node_5267: feature_name=PLD4	PLD4.value <= threshold=3.544657826423645
node_5268: feature_name=NR4A1	NR4A1.value <= threshold=0.8490704298019409
node_5269: feature_name=LTB	LTB.value > threshold=1.5252206921577454
node_5471: feature_name=CD79A	CD79A.value > threshold=2.510846734046936
node_5483: feature_name=XAF1	XAF1.value <= threshold=1.7000007629394531
node_5484: feature_name=RALGPS2	RALGPS2.value > threshold=2.08430278301239
node_5492: feature_name=FTH1	FTH1.value > threshold=-0.024074797984212637
Class: COVID-19 Critical	
Rules_1146	passed counts:3
node_0: feature_name=LTB	LTB.value > threshold=0.994031548500061
node_5264: feature_name=MT-CO1	MT-CO1.value <= threshold=-0.4127991199493408
node_5265: feature_name=MTRNR2L2	MTRNR2L2.value <= threshold=2.4473838806152344
node_5266: feature_name=TCL1A	TCL1A.value <= threshold=5.787256956100464
node_5267: feature_name=PLD4	PLD4.value <= threshold=3.544657826423645
node_5268: feature_name=NR4A1	NR4A1.value <= threshold=0.8490704298019409
node_5269: feature_name=LTB	LTB.value > threshold=1.5252206921577454
node_5471: feature_name=CD79A	CD79A.value > threshold=2.510846734046936
node_5483: feature_name=XAF1	XAF1.value <= threshold=1.7000007629394531

node_5484: feature_name=RALGPS2	RALGPS2.value <= threshold=2.08430278301239
node_5485: feature_name=HLA-DPB1	HLA-DPB1.value <= threshold=2.0922847390174866
node_5486: feature_name=XAF1	XAF1.value > threshold=-0.4127534031867981
Class: Healthy Control	
Rules_1147	passed counts:3
node_0: feature_name=LTB	LTB.value > threshold=0.994031548500061
node_5264: feature_name=MT-CO1	MT-CO1.value <= threshold=-0.4127991199493408
node_5265: feature_name=MTRNR2L2	MTRNR2L2.value <= threshold=2.4473838806152344
node_5266: feature_name=TCL1A	TCL1A.value <= threshold=5.787256956100464
node_5267: feature_name=PLD4	PLD4.value <= threshold=3.544657826423645
node_5268: feature_name=NR4A1	NR4A1.value <= threshold=0.8490704298019409
node_5269: feature_name=LTB	LTB.value <= threshold=1.5252206921577454
node_5270: feature_name=HLA-DRB5	HLA-DRB5.value > threshold=2.5117164850234985
node_5448: feature_name=HLA-DQB1	HLA-DQB1.value > threshold=2.092399835586548
node_5456: feature_name=SNX9	SNX9.value > threshold=2.15211021900177
node_5464: feature_name=AHR	AHR.value > threshold=-0.3730653375387192
node_5468: feature_name=DUSP4	DUSP4.value <= threshold=-0.248112291097641
Class: COVID-19 Severe	
Rules_1148	passed counts:3
node_0: feature_name=LTB	LTB.value > threshold=0.994031548500061
node_5264: feature_name=MT-CO1	MT-CO1.value <= threshold=-0.4127991199493408
node_5265: feature_name=MTRNR2L2	MTRNR2L2.value <= threshold=2.4473838806152344
node_5266: feature_name=TCL1A	TCL1A.value <= threshold=5.787256956100464
node_5267: feature_name=PLD4	PLD4.value <= threshold=3.544657826423645
node_5268: feature_name=NR4A1	NR4A1.value <= threshold=0.8490704298019409
node_5269: feature_name=LTB	LTB.value <= threshold=1.5252206921577454
node_5270: feature_name=HLA-DRB5	HLA-DRB5.value <= threshold=2.5117164850234985
node_5271: feature_name=CD79A	CD79A.value <= threshold=3.685201406478882

node_5272: feature_name=BCL2A1	BCL2A1.value <= threshold=2.081526756286621
node_5273: feature_name=HLA-DPA1	HLA-DPA1.value > threshold=1.8042166829109192
node_5363: feature_name=MS4A1	MS4A1.value > threshold=3.5283708572387695
node_5435: feature_name=MS4A1	MS4A1.value > threshold=3.809623122215271
Class: COVID-19 Critical	
Rules_1149	passed counts:3
node_0: feature_name=LTB	LTB.value > threshold=0.994031548500061
node_5264: feature_name=MT-CO1	MT-CO1.value <= threshold=-0.4127991199493408
node_5265: feature_name=MTRNR2L2	MTRNR2L2.value <= threshold=2.4473838806152344
node_5266: feature_name=TCL1A	TCL1A.value <= threshold=5.787256956100464
node_5267: feature_name=PLD4	PLD4.value <= threshold=3.544657826423645
node_5268: feature_name=NR4A1	NR4A1.value <= threshold=0.8490704298019409
node_5269: feature_name=LTB	LTB.value <= threshold=1.5252206921577454
node_5270: feature_name=HLA-DRB5	HLA-DRB5.value <= threshold=2.5117164850234985
node_5271: feature_name=CD79A	CD79A.value <= threshold=3.685201406478882
node_5272: feature_name=BCL2A1	BCL2A1.value <= threshold=2.081526756286621
node_5273: feature_name=HLA-DPA1	HLA-DPA1.value > threshold=1.8042166829109192
node_5363: feature_name=MS4A1	MS4A1.value <= threshold=3.5283708572387695
node_5364: feature_name=MT-CO3	MT-CO3.value > threshold=0.3994715213775635
node_5430: feature_name=OAZ1	OAZ1.value <= threshold=0.05025118589401245
node_5431: feature_name=NFKBID	NFKBID.value > threshold=0.7402573078870773
Class: COVID-19 Critical	
Rules_1150	passed counts:3
node_0: feature_name=LTB	LTB.value > threshold=0.994031548500061
node_5264: feature_name=MT-CO1	MT-CO1.value <= threshold=-0.4127991199493408
node_5265: feature_name=MTRNR2L2	MTRNR2L2.value <= threshold=2.4473838806152344
node_5266: feature_name=TCL1A	TCL1A.value <= threshold=5.787256956100464
node_5267: feature_name=PLD4	PLD4.value <= threshold=3.544657826423645

node_5268: feature_name=NR4A1	NR4A1.value <= threshold=0.8490704298019409
node_5269: feature_name=LTB	LTB.value <= threshold=1.5252206921577454
node_5270: feature_name=HLA-DRB5	HLA-DRB5.value <= threshold=2.5117164850234985
node_5271: feature_name=CD79A	CD79A.value <= threshold=3.685201406478882
node_5272: feature_name=BCL2A1	BCL2A1.value <= threshold=2.081526756286621
node_5273: feature_name=HLA-DPA1	HLA-DPA1.value > threshold=1.8042166829109192
node_5363: feature_name=MS4A1	MS4A1.value <= threshold=3.5283708572387695
node_5364: feature_name=MT-CO3	MT-CO3.value <= threshold=0.3994715213775635
node_5365: feature_name=TNFAIP3	TNFAIP3.value <= threshold=0.2631131559610367
node_5366: feature_name=MT-CO1	MT-CO1.value <= threshold=-0.44641776382923126
node_5367: feature_name=GPX1	GPX1.value <= threshold=0.7746693789958954
node_5368: feature_name=MMD	MMD.value > threshold=3.176570177078247
node_5408: feature_name=MT-CO3	MT-CO3.value <= threshold=-0.25999920815229416
node_5409: feature_name=OAZ1	OAZ1.value <= threshold=0.22116785682737827
node_5410: feature_name=CD83	CD83.value > threshold=1.7479740381240845
Class: COVID-19 Severe	
Rules_1151	passed counts:3
node_0: feature_name=LTB	LTB.value > threshold=0.994031548500061
node_5264: feature_name=MT-CO1	MT-CO1.value <= threshold=-0.4127991199493408
node_5265: feature_name=MTRNR2L2	MTRNR2L2.value <= threshold=2.4473838806152344
node_5266: feature_name=TCL1A	TCL1A.value <= threshold=5.787256956100464
node_5267: feature_name=PLD4	PLD4.value <= threshold=3.544657826423645
node_5268: feature_name=NR4A1	NR4A1.value <= threshold=0.8490704298019409
node_5269: feature_name=LTB	LTB.value <= threshold=1.5252206921577454
node_5270: feature_name=HLA-DRB5	HLA-DRB5.value <= threshold=2.5117164850234985
node_5271: feature_name=CD79A	CD79A.value <= threshold=3.685201406478882
node_5272: feature_name=BCL2A1	BCL2A1.value <= threshold=2.081526756286621
node_5273: feature_name=HLA-DPA1	HLA-DPA1.value > threshold=1.8042166829109192
node_5363: feature_name=MS4A1	MS4A1.value <= threshold=3.5283708572387695

node_5364: feature_name=MT-CO3	MT-CO3.value <= threshold=0.3994715213775635
node_5365: feature_name=TNFAIP3	TNFAIP3.value <= threshold=0.2631131559610367
node_5366: feature_name=MT-CO1	MT-CO1.value <= threshold=-0.44641776382923126
node_5367: feature_name=GPX1	GPX1.value <= threshold=0.7746693789958954
node_5368: feature_name=MMD	MMD.value <= threshold=3.176570177078247
node_5369: feature_name=FERMT3	FERMT3.value > threshold=-0.7207392454147339
node_5373: feature_name=HBB	HBB.value > threshold=-0.42021533846855164
node_5375: feature_name=MT-CYB	MT-CYB.value <= threshold=0.6712252795696259
node_5376: feature_name=IGLL5	IGLL5.value <= threshold=8.24229383468628
node_5377: feature_name=PLA2G12A	PLA2G12A.value <= threshold=3.9736461639404297
node_5378: feature_name=MEF2C	MEF2C.value <= threshold=4.8988196849823
node_5379: feature_name=ATF3	ATF3.value <= threshold=3.2426276206970215
node_5380: feature_name=RELT	RELT.value > threshold=2.1399883031845093
node_5400: feature_name=IGLL5	IGLL5.value <= threshold=-0.23810312896966934
Class: Healthy Control	
Rules_1152	passed counts:3
node_0: feature_name=LTB	LTB.value > threshold=0.994031548500061
node_5264: feature_name=MT-CO1	MT-CO1.value <= threshold=-0.4127991199493408
node_5265: feature_name=MTRNR2L2	MTRNR2L2.value <= threshold=2.4473838806152344
node_5266: feature_name=TCL1A	TCL1A.value <= threshold=5.787256956100464
node_5267: feature_name=PLD4	PLD4.value <= threshold=3.544657826423645
node_5268: feature_name=NR4A1	NR4A1.value <= threshold=0.8490704298019409
node_5269: feature_name=LTB	LTB.value <= threshold=1.5252206921577454
node_5270: feature_name=HLA-DRB5	HLA-DRB5.value <= threshold=2.5117164850234985
node_5271: feature_name=CD79A	CD79A.value <= threshold=3.685201406478882
node_5272: feature_name=BCL2A1	BCL2A1.value <= threshold=2.081526756286621
node_5273: feature_name=HLA-DPA1	HLA-DPA1.value <= threshold=1.8042166829109192
node_5274: feature_name=HSP90B1	HSP90B1.value > threshold=-1.3115270137786865
node_5278: feature_name=OAZ1	OAZ1.value <= threshold=1.0802008509635925

node_5279: feature_name=CD79B	CD79B.value <= threshold=5.875581979751587
node_5280: feature_name=CCDC141	CCDC141.value <= threshold=6.439151048660278
node_5281: feature_name=SYTL3	SYTL3.value <= threshold=1.149337351322174
node_5282: feature_name=GPX1	GPX1.value > threshold=-1.0243326425552368
node_5284: feature_name=MMD	MMD.value <= threshold=6.431635856628418
node_5285: feature_name=MARCKS	MARCKS.value > threshold=4.034798860549927
node_5343: feature_name=ATG2A	ATG2A.value <= threshold=-0.3775705397129059
Class: COVID-19 Severe	
Rules_1153	passed counts:3
node_0: feature_name=LTB	LTB.value > threshold=0.994031548500061
node_5264: feature_name=MT-CO1	MT-CO1.value <= threshold=-0.4127991199493408
node_5265: feature_name=MTRNR2L2	MTRNR2L2.value <= threshold=2.4473838806152344
node_5266: feature_name=TCL1A	TCL1A.value <= threshold=5.787256956100464
node_5267: feature_name=PLD4	PLD4.value <= threshold=3.544657826423645
node_5268: feature_name=NR4A1	NR4A1.value <= threshold=0.8490704298019409
node_5269: feature_name=LTB	LTB.value <= threshold=1.5252206921577454
node_5270: feature_name=HLA-DRB5	HLA-DRB5.value <= threshold=2.5117164850234985
node_5271: feature_name=CD79A	CD79A.value <= threshold=3.685201406478882
node_5272: feature_name=BCL2A1	BCL2A1.value <= threshold=2.081526756286621
node_5273: feature_name=HLA-DPA1	HLA-DPA1.value <= threshold=1.8042166829109192
node_5274: feature_name=HSP90B1	HSP90B1.value > threshold=-1.3115270137786865
node_5278: feature_name=OAZ1	OAZ1.value <= threshold=1.0802008509635925
node_5279: feature_name=CD79B	CD79B.value <= threshold=5.875581979751587
node_5280: feature_name=CCDC141	CCDC141.value <= threshold=6.439151048660278
node_5281: feature_name=SYTL3	SYTL3.value <= threshold=1.149337351322174
node_5282: feature_name=GPX1	GPX1.value > threshold=-1.0243326425552368
node_5284: feature_name=MMD	MMD.value <= threshold=6.431635856628418
node_5285: feature_name=MARCKS	MARCKS.value <= threshold=4.034798860549927
node_5286: feature_name=XIST	XIST.value <= threshold=1.148451752960682

node_5287: feature_name=CD83	CD83.value > threshold=2.894522190093994
node_5333: feature_name=RNF11	RNF11.value > threshold=1.2521337270736694
Class: COVID-19 Severe	
Rules_1154	passed counts:3
node_0: feature_name=LTB	LTB.value > threshold=0.994031548500061
node_5264: feature_name=MT-CO1	MT-CO1.value <= threshold=-0.4127991199493408
node_5265: feature_name=MTRNR2L2	MTRNR2L2.value <= threshold=2.4473838806152344
node_5266: feature_name=TCL1A	TCL1A.value <= threshold=5.787256956100464
node_5267: feature_name=PLD4	PLD4.value <= threshold=3.544657826423645
node_5268: feature_name=NR4A1	NR4A1.value <= threshold=0.8490704298019409
node_5269: feature_name=LTB	LTB.value <= threshold=1.5252206921577454
node_5270: feature_name=HLA-DRB5	HLA-DRB5.value <= threshold=2.5117164850234985
node_5271: feature_name=CD79A	CD79A.value <= threshold=3.685201406478882
node_5272: feature_name=BCL2A1	BCL2A1.value <= threshold=2.081526756286621
node_5273: feature_name=HLA-DPA1	HLA-DPA1.value <= threshold=1.8042166829109192
node_5274: feature_name=HSP90B1	HSP90B1.value > threshold=-1.3115270137786865
node_5278: feature_name=OAZ1	OAZ1.value <= threshold=1.0802008509635925
node_5279: feature_name=CD79B	CD79B.value <= threshold=5.875581979751587
node_5280: feature_name=CCDC141	CCDC141.value <= threshold=6.439151048660278
node_5281: feature_name=SYTL3	SYTL3.value <= threshold=1.149337351322174
node_5282: feature_name=GPX1	GPX1.value > threshold=-1.0243326425552368
node_5284: feature_name=MMD	MMD.value <= threshold=6.431635856628418
node_5285: feature_name=MARCKS	MARCKS.value <= threshold=4.034798860549927
node_5286: feature_name=XIST	XIST.value <= threshold=1.148451752960682
node_5287: feature_name=CD83	CD83.value <= threshold=2.894522190093994
node_5288: feature_name=MIR24-2	MIR24-2.value <= threshold=2.76771080493927
node_5289: feature_name=GRASP	GRASP.value > threshold=-0.4039058983325958
node_5303: feature_name=CD38	CD38.value <= threshold=3.721052408218384
node_5304: feature_name=ATF5	ATF5.value <= threshold=6.64192533493042

node_5305: feature_name=CD22	CD22.value > threshold=5.268923759460449
node_5323: feature_name=TNFAIP3	TNFAIP3.value > threshold=-0.3317747786641121
Class: COVID-19 Severe	
Rules_1155	passed counts:3
node_0: feature_name=LTB	LTB.value > threshold=0.994031548500061
node_5264: feature_name=MT-CO1	MT-CO1.value <= threshold=-0.4127991199493408
node_5265: feature_name=MTRNR2L2	MTRNR2L2.value <= threshold=2.4473838806152344
node_5266: feature_name=TCL1A	TCL1A.value <= threshold=5.787256956100464
node_5267: feature_name=PLD4	PLD4.value <= threshold=3.544657826423645
node_5268: feature_name=NR4A1	NR4A1.value <= threshold=0.8490704298019409
node_5269: feature_name=LTB	LTB.value <= threshold=1.5252206921577454
node_5270: feature_name=HLA-DRB5	HLA-DRB5.value <= threshold=2.5117164850234985
node_5271: feature_name=CD79A	CD79A.value <= threshold=3.685201406478882
node_5272: feature_name=BCL2A1	BCL2A1.value <= threshold=2.081526756286621
node_5273: feature_name=HLA-DPA1	HLA-DPA1.value <= threshold=1.8042166829109192
node_5274: feature_name=HSP90B1	HSP90B1.value > threshold=-1.3115270137786865
node_5278: feature_name=OAZ1	OAZ1.value <= threshold=1.0802008509635925
node_5279: feature_name=CD79B	CD79B.value <= threshold=5.875581979751587
node_5280: feature_name=CCDC141	CCDC141.value <= threshold=6.439151048660278
node_5281: feature_name=SYTL3	SYTL3.value <= threshold=1.149337351322174
node_5282: feature_name=GPX1	GPX1.value > threshold=-1.0243326425552368
node_5284: feature_name=MMD	MMD.value <= threshold=6.431635856628418
node_5285: feature_name=MARCKS	MARCKS.value <= threshold=4.034798860549927
node_5286: feature_name=XIST	XIST.value <= threshold=1.148451752960682
node_5287: feature_name=CD83	CD83.value <= threshold=2.894522190093994
node_5288: feature_name=MIR24-2	MIR24-2.value <= threshold=2.76771080493927
node_5289: feature_name=GRASP	GRASP.value > threshold=-0.4039058983325958
node_5303: feature_name=CD38	CD38.value <= threshold=3.721052408218384
node_5304: feature_name=ATF5	ATF5.value <= threshold=6.64192533493042

node_5305: feature_name=CD22	CD22.value <= threshold=5.268923759460449
node_5306: feature_name=CASP8	CASP8.value > threshold=1.738209843635559
node_5320: feature_name=IFIT2	IFIT2.value > threshold=-0.22565429657697678
Class: COVID-19 Critical	
Rules_1156	passed counts:3
node_0: feature_name=LTB	LTB.value > threshold=0.994031548500061
node_5264: feature_name=MT-CO1	MT-CO1.value <= threshold=-0.4127991199493408
node_5265: feature_name=MTRNR2L2	MTRNR2L2.value <= threshold=2.4473838806152344
node_5266: feature_name=TCL1A	TCL1A.value <= threshold=5.787256956100464
node_5267: feature_name=PLD4	PLD4.value <= threshold=3.544657826423645
node_5268: feature_name=NR4A1	NR4A1.value <= threshold=0.8490704298019409
node_5269: feature_name=LTB	LTB.value <= threshold=1.5252206921577454
node_5270: feature_name=HLA-DRB5	HLA-DRB5.value <= threshold=2.5117164850234985
node_5271: feature_name=CD79A	CD79A.value <= threshold=3.685201406478882
node_5272: feature_name=BCL2A1	BCL2A1.value <= threshold=2.081526756286621
node_5273: feature_name=HLA-DPA1	HLA-DPA1.value <= threshold=1.8042166829109192
node_5274: feature_name=HSP90B1	HSP90B1.value > threshold=-1.3115270137786865
node_5278: feature_name=OAZ1	OAZ1.value <= threshold=1.0802008509635925
node_5279: feature_name=CD79B	CD79B.value <= threshold=5.875581979751587
node_5280: feature_name=CCDC141	CCDC141.value <= threshold=6.439151048660278
node_5281: feature_name=SYTL3	SYTL3.value <= threshold=1.149337351322174
node_5282: feature_name=GPX1	GPX1.value > threshold=-1.0243326425552368
node_5284: feature_name=MMD	MMD.value <= threshold=6.431635856628418
node_5285: feature_name=MARCKS	MARCKS.value <= threshold=4.034798860549927
node_5286: feature_name=XIST	XIST.value <= threshold=1.148451752960682
node_5287: feature_name=CD83	CD83.value <= threshold=2.894522190093994
node_5288: feature_name=MIR24-2	MIR24-2.value <= threshold=2.76771080493927
node_5289: feature_name=GRASP	GRASP.value <= threshold=-0.4039058983325958
node_5290: feature_name=SLC7A5	SLC7A5.value > threshold=-0.4947117418050766

node_5294: feature_name=CD83	CD83.value > threshold=2.1984872817993164
node_5298: feature_name=MT-CO1	MT-CO1.value > threshold=-0.6244264245033264
Class: COVID-19 Critical	
Rules_1157	passed counts:3
node_0: feature_name=LTB	LTB.value <= threshold=0.994031548500061
node_1: feature_name=MTRNR2L2	MTRNR2L2.value > threshold=2.8784297704696655
node_5207: feature_name=MTRNR2L8	MTRNR2L8.value > threshold=3.1915172338485718
node_5257: feature_name=SNX29P2	SNX29P2.value > threshold=-0.2883431911468506
node_5261: feature_name=PLD4	PLD4.value <= threshold=-0.03204602375626564
Class: COVID-19 Severe	
Rules_1158	passed counts:3
node_0: feature_name=LTB	LTB.value <= threshold=0.994031548500061
node_1: feature_name=MTRNR2L2	MTRNR2L2.value > threshold=2.8784297704696655
node_5207: feature_name=MTRNR2L8	MTRNR2L8.value <= threshold=3.1915172338485718
node_5208: feature_name=MT-CYB	MT-CYB.value > threshold=1.3929389119148254
node_5242: feature_name=MTRNR2L2	MTRNR2L2.value <= threshold=3.8612314462661743
node_5243: feature_name=RALGPS2	RALGPS2.value > threshold=4.833884000778198
Class: Healthy Control	
Rules_1159	passed counts:3
node_0: feature_name=LTB	LTB.value <= threshold=0.994031548500061
node_1: feature_name=MTRNR2L2	MTRNR2L2.value > threshold=2.8784297704696655
node_5207: feature_name=MTRNR2L8	MTRNR2L8.value <= threshold=3.1915172338485718
node_5208: feature_name=MT-CYB	MT-CYB.value <= threshold=1.3929389119148254
node_5209: feature_name=SLC7A5	SLC7A5.value > threshold=3.205444812774658
Class: COVID-19 Critical	
Rules_1160	passed counts:3

node_0: feature_name=LTB	LTB.value <= threshold=0.994031548500061
node_1: feature_name=MTRNR2L2	MTRNR2L2.value > threshold=2.8784297704696655
node_5207: feature_name=MTRNR2L8	MTRNR2L8.value <= threshold=3.1915172338485718
node_5208: feature_name=MT-CYB	MT-CYB.value <= threshold=1.3929389119148254
node_5209: feature_name=SLC7A5	SLC7A5.value <= threshold=3.205444812774658
node_5210: feature_name=MT-CO2	MT-CO2.value <= threshold=2.065874218940735
node_5211: feature_name=HLA-DRB5	HLA-DRB5.value > threshold=2.276706099510193
node_5235: feature_name=SPI1	SPI1.value > threshold=-0.4083195775747299
Class: COVID-19 Severe	
Rules_1161	passed counts:3
node_0: feature_name=LTB	LTB.value <= threshold=0.994031548500061
node_1: feature_name=MTRNR2L2	MTRNR2L2.value <= threshold=2.8784297704696655
node_2: feature_name=TCL1A	TCL1A.value > threshold=4.798312664031982
node_4912: feature_name=MT-CO1	MT-CO1.value > threshold=-0.3664844036102295
node_5002: feature_name=IFI44L	IFI44L.value > threshold=2.3101747035980225
node_5198: feature_name=MX1	MX1.value <= threshold=-0.4572612792253494
Class: Healthy Control	
Rules_1162	passed counts:3
node_0: feature_name=LTB	LTB.value <= threshold=0.994031548500061
node_1: feature_name=MTRNR2L2	MTRNR2L2.value <= threshold=2.8784297704696655
node_2: feature_name=TCL1A	TCL1A.value > threshold=4.798312664031982
node_4912: feature_name=MT-CO1	MT-CO1.value > threshold=-0.3664844036102295
node_5002: feature_name=IFI44L	IFI44L.value <= threshold=2.3101747035980225
node_5003: feature_name=SNX9	SNX9.value > threshold=2.0247421264648438
node_5147: feature_name=CD79A	CD79A.value > threshold=2.554050326347351
node_5169: feature_name=CD79B	CD79B.value <= threshold=-0.2480710819363594
node_5170: feature_name=HLA-DQB1	HLA-DQB1.value <= threshold=2.405891537666321
node_5171: feature_name=RALGPS2	RALGPS2.value > threshold=3.8303792476654053

node_5177: feature_name=KIAA0125	KIAA0125.value <= threshold=-0.14587634056806564
Class: Healthy Control	
Rules_1163	passed counts:3
node_0: feature_name=LTB	LTB.value <= threshold=0.994031548500061
node_1: feature_name=MTRNR2L2	MTRNR2L2.value <= threshold=2.8784297704696655
node_2: feature_name=TCL1A	TCL1A.value > threshold=4.798312664031982
node_4912: feature_name=MT-CO1	MT-CO1.value > threshold=-0.3664844036102295
node_5002: feature_name=IFI44L	IFI44L.value <= threshold=2.3101747035980225
node_5003: feature_name=SNX9	SNX9.value > threshold=2.0247421264648438
node_5147: feature_name=CD79A	CD79A.value > threshold=2.554050326347351
node_5169: feature_name=CD79B	CD79B.value <= threshold=-0.2480710819363594
node_5170: feature_name=HLA-DQB1	HLA-DQB1.value <= threshold=2.405891537666321
node_5171: feature_name=RALGPS2	RALGPS2.value <= threshold=3.8303792476654053
node_5172: feature_name=RGS1	RGS1.value <= threshold=-0.5179850161075592
node_5173: feature_name=HLA-DPA1	HLA-DPA1.value > threshold=1.6717939376831055
Class: Healthy Control	
Rules_1164	passed counts:3
node_0: feature_name=LTB	LTB.value <= threshold=0.994031548500061
node_1: feature_name=MTRNR2L2	MTRNR2L2.value <= threshold=2.8784297704696655
node_2: feature_name=TCL1A	TCL1A.value > threshold=4.798312664031982
node_4912: feature_name=MT-CO1	MT-CO1.value > threshold=-0.3664844036102295
node_5002: feature_name=IFI44L	IFI44L.value <= threshold=2.3101747035980225
node_5003: feature_name=SNX9	SNX9.value > threshold=2.0247421264648438
node_5147: feature_name=CD79A	CD79A.value <= threshold=2.554050326347351
node_5148: feature_name=HLA-DQB1	HLA-DQB1.value > threshold=2.6719621419906616
node_5162: feature_name=GPX1	GPX1.value > threshold=0.0071204304695129395
Class: Healthy Control	

Rules_1165	passed counts:3
node_0: feature_name=LTB	LTB.value <= threshold=0.994031548500061
node_1: feature_name=MTRNR2L2	MTRNR2L2.value <= threshold=2.8784297704696655
node_2: feature_name=TCL1A	TCL1A.value > threshold=4.798312664031982
node_4912: feature_name=MT-CO1	MT-CO1.value > threshold=-0.3664844036102295
node_5002: feature_name=IFI44L	IFI44L.value <= threshold=2.3101747035980225
node_5003: feature_name=SNX9	SNX9.value > threshold=2.0247421264648438
node_5147: feature_name=CD79A	CD79A.value <= threshold=2.554050326347351
node_5148: feature_name=HLA-DQB1	HLA-DQB1.value > threshold=2.6719621419906616
node_5162: feature_name=GPX1	GPX1.value <= threshold=0.0071204304695129395
node_5163: feature_name=CD79B	CD79B.value <= threshold=-0.2608358860015869
Class: COVID-19 Critical	
Rules_1166	passed counts:3
node_0: feature_name=LTB	LTB.value <= threshold=0.994031548500061
node_1: feature_name=MTRNR2L2	MTRNR2L2.value <= threshold=2.8784297704696655
node_2: feature_name=TCL1A	TCL1A.value > threshold=4.798312664031982
node_4912: feature_name=MT-CO1	MT-CO1.value > threshold=-0.3664844036102295
node_5002: feature_name=IFI44L	IFI44L.value <= threshold=2.3101747035980225
node_5003: feature_name=SNX9	SNX9.value > threshold=2.0247421264648438
node_5147: feature_name=CD79A	CD79A.value <= threshold=2.554050326347351
node_5148: feature_name=HLA-DQB1	HLA-DQB1.value <= threshold=2.6719621419906616
node_5149: feature_name=BANK1	BANK1.value > threshold=4.278962850570679
node_5157: feature_name=PARP14	PARP14.value <= threshold=1.0527673661708832
Class: Healthy Control	
Rules_1167	passed counts:3
node_0: feature_name=LTB	LTB.value <= threshold=0.994031548500061
node_1: feature_name=MTRNR2L2	MTRNR2L2.value <= threshold=2.8784297704696655
node_2: feature_name=TCL1A	TCL1A.value > threshold=4.798312664031982

node_4912: feature_name=MT-CO1	MT-CO1.value > threshold=-0.3664844036102295
node_5002: feature_name=IFI44L	IFI44L.value <= threshold=2.3101747035980225
node_5003: feature_name=SNX9	SNX9.value <= threshold=2.0247421264648438
node_5004: feature_name=STAG3	STAG3.value <= threshold=6.482141733169556
node_5005: feature_name=BACH1	BACH1.value > threshold=-0.5237349569797516
node_5047: feature_name=RHOB	RHOB.value > threshold=-0.4843696355819702
node_5055: feature_name=FAM129C	FAM129C.value > threshold=-0.2832885980606079
node_5079: feature_name=TP53INP1	TP53INP1.value > threshold=2.9482468366622925
node_5129: feature_name=CD79B	CD79B.value <= threshold=2.402374267578125
node_5130: feature_name=JARID2	JARID2.value > threshold=2.191186785697937
Class: Healthy Control	
Rules_1168	passed counts:3
node_0: feature_name=LTB	LTB.value <= threshold=0.994031548500061
node_1: feature_name=MTRNR2L2	MTRNR2L2.value <= threshold=2.8784297704696655
node_2: feature_name=TCL1A	TCL1A.value > threshold=4.798312664031982
node_4912: feature_name=MT-CO1	MT-CO1.value > threshold=-0.3664844036102295
node_5002: feature_name=IFI44L	IFI44L.value <= threshold=2.3101747035980225
node_5003: feature_name=SNX9	SNX9.value <= threshold=2.0247421264648438
node_5004: feature_name=STAG3	STAG3.value <= threshold=6.482141733169556
node_5005: feature_name=BACH1	BACH1.value > threshold=-0.5237349569797516
node_5047: feature_name=RHOB	RHOB.value > threshold=-0.4843696355819702
node_5055: feature_name=FAM129C	FAM129C.value > threshold=-0.2832885980606079
node_5079: feature_name=TP53INP1	TP53INP1.value <= threshold=2.9482468366622925
node_5080: feature_name=HLA-DRB5	HLA-DRB5.value <= threshold=2.6279215812683105
node_5081: feature_name=IFI6	IFI6.value > threshold=1.4884688258171082
Class: COVID-19 Critical	
Rules_1169	passed counts:3
node_0: feature_name=LTB	LTB.value <= threshold=0.994031548500061

node_1: feature_name=MTRNR2L2	MTRNR2L2.value <= threshold=2.8784297704696655
node_2: feature_name=TCL1A	TCL1A.value > threshold=4.798312664031982
node_4912: feature_name=MT-CO1	MT-CO1.value > threshold=-0.3664844036102295
node_5002: feature_name=IFI44L	IFI44L.value <= threshold=2.3101747035980225
node_5003: feature_name=SNX9	SNX9.value <= threshold=2.0247421264648438
node_5004: feature_name=STAG3	STAG3.value <= threshold=6.482141733169556
node_5005: feature_name=BACH1	BACH1.value > threshold=-0.5237349569797516
node_5047: feature_name=RHOB	RHOB.value > threshold=-0.4843696355819702
node_5055: feature_name=FAM129C	FAM129C.value <= threshold=-0.2832885980606079
node_5056: feature_name=CD83	CD83.value > threshold=-0.5592367649078369
node_5060: feature_name=OAZ1	OAZ1.value <= threshold=0.7524859011173248
node_5061: feature_name=FCRL1	FCRL1.value <= threshold=5.677775859832764
node_5062: feature_name=RNF144B	RNF144B.value > threshold=1.2688461616635323
Class: COVID-19 Severe	
Rules_1170	passed counts:3
node_0: feature_name=LTB	LTB.value <= threshold=0.994031548500061
node_1: feature_name=MTRNR2L2	MTRNR2L2.value <= threshold=2.8784297704696655
node_2: feature_name=TCL1A	TCL1A.value > threshold=4.798312664031982
node_4912: feature_name=MT-CO1	MT-CO1.value > threshold=-0.3664844036102295
node_5002: feature_name=IFI44L	IFI44L.value <= threshold=2.3101747035980225
node_5003: feature_name=SNX9	SNX9.value <= threshold=2.0247421264648438
node_5004: feature_name=STAG3	STAG3.value <= threshold=6.482141733169556
node_5005: feature_name=BACH1	BACH1.value > threshold=-0.5237349569797516
node_5047: feature_name=RHOB	RHOB.value <= threshold=-0.4843696355819702
node_5048: feature_name=MT-CO1	MT-CO1.value <= threshold=0.6870226263999939
node_5049: feature_name=HIST1H2AC	HIST1H2AC.value > threshold=1.6820606589317322
Class: Healthy Control	
Rules_1171	passed counts:3

node_0: feature_name=LTB	LTB.value <= threshold=0.994031548500061
node_1: feature_name=MTRNR2L2	MTRNR2L2.value <= threshold=2.8784297704696655
node_2: feature_name=TCL1A	TCL1A.value > threshold=4.798312664031982
node_4912: feature_name=MT-CO1	MT-CO1.value > threshold=-0.3664844036102295
node_5002: feature_name=IFI44L	IFI44L.value <= threshold=2.3101747035980225
node_5003: feature_name=SNX9	SNX9.value <= threshold=2.0247421264648438
node_5004: feature_name=STAG3	STAG3.value <= threshold=6.482141733169556
node_5005: feature_name=BACH1	BACH1.value <= threshold=-0.5237349569797516
node_5006: feature_name=OAZ1	OAZ1.value > threshold=-0.6512158215045929
node_5014: feature_name=MEF2A	MEF2A.value > threshold=3.61627995967865
Class: COVID-19 Critical	
Rules_1172	passed counts:3
node_0: feature_name=LTB	LTB.value <= threshold=0.994031548500061
node_1: feature_name=MTRNR2L2	MTRNR2L2.value <= threshold=2.8784297704696655
node_2: feature_name=TCL1A	TCL1A.value > threshold=4.798312664031982
node_4912: feature_name=MT-CO1	MT-CO1.value <= threshold=-0.3664844036102295
node_4913: feature_name=CD79A	CD79A.value > threshold=2.987208843231201
node_4973: feature_name=IFI44L	IFI44L.value <= threshold=0.7183398455381393
node_4974: feature_name=CD79B	CD79B.value > threshold=1.7606428265571594
node_4982: feature_name=TNFAIP3	TNFAIP3.value > threshold=0.0729537159204483
node_4996: feature_name=EGR1	EGR1.value <= threshold=1.0801724791526794
Class: COVID-19 Critical	
Rules_1173	passed counts:3
node_0: feature_name=LTB	LTB.value <= threshold=0.994031548500061
node_1: feature_name=MTRNR2L2	MTRNR2L2.value <= threshold=2.8784297704696655
node_2: feature_name=TCL1A	TCL1A.value > threshold=4.798312664031982
node_4912: feature_name=MT-CO1	MT-CO1.value <= threshold=-0.3664844036102295
node_4913: feature_name=CD79A	CD79A.value > threshold=2.987208843231201

node_4973: feature_name=IFI44L	IFI44L.value <= threshold=0.7183398455381393
node_4974: feature_name=CD79B	CD79B.value <= threshold=1.7606428265571594
node_4975: feature_name=HLA-DPA1	HLA-DPA1.value > threshold=1.9396945238113403
node_4977: feature_name=MT-CYB	MT-CYB.value > threshold=-0.4501015394926071
Class: Healthy Control	
Rules_1174	passed counts:3
node_0: feature_name=LTB	LTB.value <= threshold=0.994031548500061
node_1: feature_name=MTRNR2L2	MTRNR2L2.value <= threshold=2.8784297704696655
node_2: feature_name=TCL1A	TCL1A.value > threshold=4.798312664031982
node_4912: feature_name=MT-CO1	MT-CO1.value <= threshold=-0.3664844036102295
node_4913: feature_name=CD79A	CD79A.value > threshold=2.987208843231201
node_4973: feature_name=IFI44L	IFI44L.value <= threshold=0.7183398455381393
node_4974: feature_name=CD79B	CD79B.value <= threshold=1.7606428265571594
node_4975: feature_name=HLA-DPA1	HLA-DPA1.value > threshold=1.9396945238113403
node_4977: feature_name=MT-CYB	MT-CYB.value <= threshold=-0.4501015394926071
node_4978: feature_name=MEF2C	MEF2C.value > threshold=1.385680228471756
Class: COVID-19 Severe	
Rules_1175	passed counts:3
node_0: feature_name=LTB	LTB.value <= threshold=0.994031548500061
node_1: feature_name=MTRNR2L2	MTRNR2L2.value <= threshold=2.8784297704696655
node_2: feature_name=TCL1A	TCL1A.value > threshold=4.798312664031982
node_4912: feature_name=MT-CO1	MT-CO1.value <= threshold=-0.3664844036102295
node_4913: feature_name=CD79A	CD79A.value <= threshold=2.987208843231201
node_4914: feature_name=HLA-DPB1	HLA-DPB1.value > threshold=2.0926655530929565
node_4958: feature_name=KIAA0125	KIAA0125.value <= threshold=-0.15961305052042007
node_4959: feature_name=PDLIM1	PDLIM1.value <= threshold=3.826790690422058
node_4960: feature_name=MS4A1	MS4A1.value > threshold=3.352075457572937
node_4966: feature_name=CD83	CD83.value > threshold=0.5404248535633087

Class: COVID-19 Severe	
Rules_1176	passed counts:3
node_0: feature_name=LTB	LTB.value <= threshold=0.994031548500061
node_1: feature_name=MTRNR2L2	MTRNR2L2.value <= threshold=2.8784297704696655
node_2: feature_name=TCL1A	TCL1A.value <= threshold=4.798312664031982
node_3: feature_name=MT-CO1	MT-CO1.value > threshold=0.08947191759943962
node_2521: feature_name=LTB	LTB.value > threshold=0.6038667261600494
node_4173: feature_name=OAZ1	OAZ1.value > threshold=0.143283873796463
node_4615: feature_name=NR4A1	NR4A1.value > threshold=0.6621004343032837
node_4817: feature_name=HSP90B1	HSP90B1.value > threshold=1.1699737310409546
node_4895: feature_name=MS4A1	MS4A1.value > threshold=2.627432107925415
node_4903: feature_name=MARCH1	MARCH1.value > threshold=3.4180619716644287
Class: COVID-19 Critical	
Rules_1177	passed counts:3
node_0: feature_name=LTB	LTB.value <= threshold=0.994031548500061
node_1: feature_name=MTRNR2L2	MTRNR2L2.value <= threshold=2.8784297704696655
node_2: feature_name=TCL1A	TCL1A.value <= threshold=4.798312664031982
node_3: feature_name=MT-CO1	MT-CO1.value > threshold=0.08947191759943962
node_2521: feature_name=LTB	LTB.value > threshold=0.6038667261600494
node_4173: feature_name=OAZ1	OAZ1.value > threshold=0.143283873796463
node_4615: feature_name=NR4A1	NR4A1.value > threshold=0.6621004343032837
node_4817: feature_name=HSP90B1	HSP90B1.value > threshold=1.1699737310409546
node_4895: feature_name=MS4A1	MS4A1.value <= threshold=2.627432107925415
node_4896: feature_name=FTH1	FTH1.value > threshold=0.49300217628479004
node_4898: feature_name=ITGAX	ITGAX.value > threshold=-0.2847445458173752
Class: COVID-19 Critical	
Rules_1178	passed counts:3

node_0: feature_name=LTB	LTB.value <= threshold=0.994031548500061
node_1: feature_name=MTRNR2L2	MTRNR2L2.value <= threshold=2.8784297704696655
node_2: feature_name=TCL1A	TCL1A.value <= threshold=4.798312664031982
node_3: feature_name=MT-CO1	MT-CO1.value > threshold=0.08947191759943962
node_2521: feature_name=LTB	LTB.value > threshold=0.6038667261600494
node_4173: feature_name=OAZ1	OAZ1.value > threshold=0.143283873796463
node_4615: feature_name=NR4A1	NR4A1.value > threshold=0.6621004343032837
node_4817: feature_name=HSP90B1	HSP90B1.value > threshold=1.1699737310409546
node_4895: feature_name=MS4A1	MS4A1.value <= threshold=2.627432107925415
node_4896: feature_name=FTH1	FTH1.value > threshold=0.49300217628479004
node_4898: feature_name=ITGAX	ITGAX.value <= threshold=-0.2847445458173752
node_4899: feature_name=IFIT2	IFIT2.value > threshold=-0.23323790729045868
Class: Healthy Control	
Rules_1179	passed counts:3
node_0: feature_name=LTB	LTB.value <= threshold=0.994031548500061
node_1: feature_name=MTRNR2L2	MTRNR2L2.value <= threshold=2.8784297704696655
node_2: feature_name=TCL1A	TCL1A.value <= threshold=4.798312664031982
node_3: feature_name=MT-CO1	MT-CO1.value > threshold=0.08947191759943962
node_2521: feature_name=LTB	LTB.value > threshold=0.6038667261600494
node_4173: feature_name=OAZ1	OAZ1.value > threshold=0.143283873796463
node_4615: feature_name=NR4A1	NR4A1.value > threshold=0.6621004343032837
node_4817: feature_name=HSP90B1	HSP90B1.value > threshold=1.1699737310409546
node_4895: feature_name=MS4A1	MS4A1.value <= threshold=2.627432107925415
node_4896: feature_name=FTH1	FTH1.value > threshold=0.49300217628479004
node_4898: feature_name=ITGAX	ITGAX.value <= threshold=-0.2847445458173752
node_4899: feature_name=IFIT2	IFIT2.value <= threshold=-0.23323790729045868
Class: COVID-19 Severe	
Rules_1180	passed counts:3

node_0: feature_name=LTB	LTB.value <= threshold=0.994031548500061
node_1: feature_name=MTRNR2L2	MTRNR2L2.value <= threshold=2.8784297704696655
node_2: feature_name=TCL1A	TCL1A.value <= threshold=4.798312664031982
node_3: feature_name=MT-CO1	MT-CO1.value > threshold=0.08947191759943962
node_2521: feature_name=LTB	LTB.value > threshold=0.6038667261600494
node_4173: feature_name=OAZ1	OAZ1.value > threshold=0.143283873796463
node_4615: feature_name=NR4A1	NR4A1.value > threshold=0.6621004343032837
node_4817: feature_name=HSP90B1	HSP90B1.value <= threshold=1.1699737310409546
node_4818: feature_name=XAF1	XAF1.value <= threshold=2.6752071380615234
node_4819: feature_name=SNX9	SNX9.value > threshold=2.939147114753723
node_4885: feature_name=CD79B	CD79B.value <= threshold=-0.2693563252687454
node_4886: feature_name=VCL	VCL.value <= threshold=-0.3364468365907669
Class: Healthy Control	
Rules_1181	passed counts:3
node_0: feature_name=LTB	LTB.value <= threshold=0.994031548500061
node_1: feature_name=MTRNR2L2	MTRNR2L2.value <= threshold=2.8784297704696655
node_2: feature_name=TCL1A	TCL1A.value <= threshold=4.798312664031982
node_3: feature_name=MT-CO1	MT-CO1.value > threshold=0.08947191759943962
node_2521: feature_name=LTB	LTB.value > threshold=0.6038667261600494
node_4173: feature_name=OAZ1	OAZ1.value > threshold=0.143283873796463
node_4615: feature_name=NR4A1	NR4A1.value > threshold=0.6621004343032837
node_4817: feature_name=HSP90B1	HSP90B1.value <= threshold=1.1699737310409546
node_4818: feature_name=XAF1	XAF1.value <= threshold=2.6752071380615234
node_4819: feature_name=SNX9	SNX9.value <= threshold=2.939147114753723
node_4820: feature_name=HLA-DQA2	HLA-DQA2.value <= threshold=2.9822239875793457
node_4821: feature_name=ATF5	ATF5.value <= threshold=4.116653323173523
node_4822: feature_name=RALGPS2	RALGPS2.value <= threshold=-0.3627814054489136
node_4823: feature_name=HIST1H2AC	HIST1H2AC.value <= threshold=-0.2637694925069809
node_4824: feature_name=HLA-DRB5	HLA-DRB5.value <= threshold=0.4284958690404892

node_4825: feature_name=LTB	LTB.value <= threshold=0.8841853141784668
Class: COVID-19 Severe	
Rules_1182	passed counts:3
node_0: feature_name=LTB	LTB.value <= threshold=0.994031548500061
node_1: feature_name=MTRNR2L2	MTRNR2L2.value <= threshold=2.8784297704696655
node_2: feature_name=TCL1A	TCL1A.value <= threshold=4.798312664031982
node_3: feature_name=MT-CO1	MT-CO1.value > threshold=0.08947191759943962
node_2521: feature_name=LTB	LTB.value > threshold=0.6038667261600494
node_4173: feature_name=OAZ1	OAZ1.value > threshold=0.143283873796463
node_4615: feature_name=NR4A1	NR4A1.value <= threshold=0.6621004343032837
node_4616: feature_name=NFKBID	NFKBID.value > threshold=3.283741593360901
node_4804: feature_name=FAM129C	FAM129C.value > threshold=6.735232830047607
Class: Healthy Control	
Rules_1183	passed counts:3
node_0: feature_name=LTB	LTB.value <= threshold=0.994031548500061
node_1: feature_name=MTRNR2L2	MTRNR2L2.value <= threshold=2.8784297704696655
node_2: feature_name=TCL1A	TCL1A.value <= threshold=4.798312664031982
node_3: feature_name=MT-CO1	MT-CO1.value > threshold=0.08947191759943962
node_2521: feature_name=LTB	LTB.value > threshold=0.6038667261600494
node_4173: feature_name=OAZ1	OAZ1.value > threshold=0.143283873796463
node_4615: feature_name=NR4A1	NR4A1.value <= threshold=0.6621004343032837
node_4616: feature_name=NFKBID	NFKBID.value <= threshold=3.283741593360901
node_4617: feature_name=HLA-DQA1	HLA-DQA1.value > threshold=2.3435752391815186
node_4719: feature_name=MX1	MX1.value > threshold=1.851993978023529
node_4795: feature_name=FCRL1	FCRL1.value > threshold=5.9853057861328125
node_4801: feature_name=BCL2A1	BCL2A1.value <= threshold=1.3437456786632538
Class: Healthy Control	

Rules_1184	passed counts:3
node_0: feature_name=LTB	LTB.value <= threshold=0.994031548500061
node_1: feature_name=MTRNR2L2	MTRNR2L2.value <= threshold=2.8784297704696655
node_2: feature_name=TCL1A	TCL1A.value <= threshold=4.798312664031982
node_3: feature_name=MT-CO1	MT-CO1.value > threshold=0.08947191759943962
node_2521: feature_name=LTB	LTB.value > threshold=0.6038667261600494
node_4173: feature_name=OAZ1	OAZ1.value > threshold=0.143283873796463
node_4615: feature_name=NR4A1	NR4A1.value <= threshold=0.6621004343032837
node_4616: feature_name=NFKBID	NFKBID.value <= threshold=3.283741593360901
node_4617: feature_name=HLA-DQA1	HLA-DQA1.value > threshold=2.3435752391815186
node_4719: feature_name=MX1	MX1.value <= threshold=1.851993978023529
node_4720: feature_name=JUN	JUN.value > threshold=-0.5025404393672943
node_4740: feature_name=IRF8	IRF8.value <= threshold=3.838419556617737
node_4741: feature_name=BACH1	BACH1.value > threshold=0.9568033516407013
node_4769: feature_name=TCF4	TCF4.value <= threshold=-0.23278788477182388
node_4770: feature_name=FERMT3	FERMT3.value <= threshold=1.1583285331726074
node_4771: feature_name=MS4A1	MS4A1.value <= threshold=2.3401901721954346
node_4772: feature_name=IFI6	IFI6.value > threshold=-0.5258762538433075
Class: COVID-19 Severe	
Rules_1185	passed counts:3
node_0: feature_name=LTB	LTB.value <= threshold=0.994031548500061
node_1: feature_name=MTRNR2L2	MTRNR2L2.value <= threshold=2.8784297704696655
node_2: feature_name=TCL1A	TCL1A.value <= threshold=4.798312664031982
node_3: feature_name=MT-CO1	MT-CO1.value > threshold=0.08947191759943962
node_2521: feature_name=LTB	LTB.value > threshold=0.6038667261600494
node_4173: feature_name=OAZ1	OAZ1.value > threshold=0.143283873796463
node_4615: feature_name=NR4A1	NR4A1.value <= threshold=0.6621004343032837
node_4616: feature_name=NFKBID	NFKBID.value <= threshold=3.283741593360901
node_4617: feature_name=HLA-DQA1	HLA-DQA1.value > threshold=2.3435752391815186

node_4719: feature_name=MX1	MX1.value <= threshold=1.851993978023529
node_4720: feature_name=JUN	JUN.value > threshold=-0.5025404393672943
node_4740: feature_name=IRF8	IRF8.value <= threshold=3.838419556617737
node_4741: feature_name=BACH1	BACH1.value <= threshold=0.9568033516407013
node_4742: feature_name=HLA-DQA2	HLA-DQA2.value > threshold=4.834465265274048
Class: COVID-19 Critical	
Rules_1186	passed counts:3
node_0: feature_name=LTB	LTB.value <= threshold=0.994031548500061
node_1: feature_name=MTRNR2L2	MTRNR2L2.value <= threshold=2.8784297704696655
node_2: feature_name=TCL1A	TCL1A.value <= threshold=4.798312664031982
node_3: feature_name=MT-CO1	MT-CO1.value > threshold=0.08947191759943962
node_2521: feature_name=LTB	LTB.value > threshold=0.6038667261600494
node_4173: feature_name=OAZ1	OAZ1.value > threshold=0.143283873796463
node_4615: feature_name=NR4A1	NR4A1.value <= threshold=0.6621004343032837
node_4616: feature_name=NFKBID	NFKBID.value <= threshold=3.283741593360901
node_4617: feature_name=HLA-DQA1	HLA-DQA1.value > threshold=2.3435752391815186
node_4719: feature_name=MX1	MX1.value <= threshold=1.851993978023529
node_4720: feature_name=JUN	JUN.value > threshold=-0.5025404393672943
node_4740: feature_name=IRF8	IRF8.value <= threshold=3.838419556617737
node_4741: feature_name=BACH1	BACH1.value <= threshold=0.9568033516407013
node_4742: feature_name=HLA-DQA2	HLA-DQA2.value <= threshold=4.834465265274048
node_4743: feature_name=STAG3	STAG3.value > threshold=6.6092634201049805
Class: COVID-19 Critical	
Rules_1187	passed counts:3
node_0: feature_name=LTB	LTB.value <= threshold=0.994031548500061
node_1: feature_name=MTRNR2L2	MTRNR2L2.value <= threshold=2.8784297704696655
node_2: feature_name=TCL1A	TCL1A.value <= threshold=4.798312664031982
node_3: feature_name=MT-CO1	MT-CO1.value > threshold=0.08947191759943962

node_2521: feature_name=LTB	LTB.value > threshold=0.6038667261600494
node_4173: feature_name=OAZ1	OAZ1.value > threshold=0.143283873796463
node_4615: feature_name=NR4A1	NR4A1.value <= threshold=0.6621004343032837
node_4616: feature_name=NFKBID	NFKBID.value <= threshold=3.283741593360901
node_4617: feature_name=HLA-DQA1	HLA-DQA1.value > threshold=2.3435752391815186
node_4719: feature_name=MX1	MX1.value <= threshold=1.851993978023529
node_4720: feature_name=JUN	JUN.value <= threshold=-0.5025404393672943
node_4721: feature_name=RELT	RELT.value <= threshold=-0.28893138468265533
node_4722: feature_name=SNX29P2	SNX29P2.value > threshold=1.9070574045181274
node_4730: feature_name=NFKBID	NFKBID.value > threshold=1.8065539002418518
node_4732: feature_name=OAZ1	OAZ1.value > threshold=0.24030182510614395
Class: COVID-19 Critical	
Rules_1188	passed counts:3
node_0: feature_name=LTB	LTB.value <= threshold=0.994031548500061
node_1: feature_name=MTRNR2L2	MTRNR2L2.value <= threshold=2.8784297704696655
node_2: feature_name=TCL1A	TCL1A.value <= threshold=4.798312664031982
node_3: feature_name=MT-CO1	MT-CO1.value > threshold=0.08947191759943962
node_2521: feature_name=LTB	LTB.value > threshold=0.6038667261600494
node_4173: feature_name=OAZ1	OAZ1.value > threshold=0.143283873796463
node_4615: feature_name=NR4A1	NR4A1.value <= threshold=0.6621004343032837
node_4616: feature_name=NFKBID	NFKBID.value <= threshold=3.283741593360901
node_4617: feature_name=HLA-DQA1	HLA-DQA1.value <= threshold=2.3435752391815186
node_4618: feature_name=PELI1	PELI1.value > threshold=1.3271203637123108
node_4680: feature_name=BANK1	BANK1.value > threshold=-0.3607018291950226
node_4682: feature_name=RGCC	RGCC.value <= threshold=0.60355344414711
node_4683: feature_name=SNX29P2	SNX29P2.value <= threshold=4.192138910293579
node_4684: feature_name=XIST	XIST.value > threshold=2.375336170196533
Class: Healthy Control	

Rules_1189	passed counts:3
node_0: feature_name=LTB	LTB.value <= threshold=0.994031548500061
node_1: feature_name=MTRNR2L2	MTRNR2L2.value <= threshold=2.8784297704696655
node_2: feature_name=TCL1A	TCL1A.value <= threshold=4.798312664031982
node_3: feature_name=MT-CO1	MT-CO1.value > threshold=0.08947191759943962
node_2521: feature_name=LTB	LTB.value > threshold=0.6038667261600494
node_4173: feature_name=OAZ1	OAZ1.value > threshold=0.143283873796463
node_4615: feature_name=NR4A1	NR4A1.value <= threshold=0.6621004343032837
node_4616: feature_name=NFKBID	NFKBID.value <= threshold=3.283741593360901
node_4617: feature_name=HLA-DQA1	HLA-DQA1.value <= threshold=2.3435752391815186
node_4618: feature_name=PELI1	PELI1.value <= threshold=1.3271203637123108
node_4619: feature_name=PLD4	PLD4.value > threshold=3.3385335206985474
node_4671: feature_name=LDLRAD4	LDLRAD4.value > threshold=2.8038166761398315
node_4677: feature_name=TLR2	TLR2.value > threshold=-0.28493697941303253
Class: COVID-19 Critical	
Rules_1190	passed counts:3
node_0: feature_name=LTB	LTB.value <= threshold=0.994031548500061
node_1: feature_name=MTRNR2L2	MTRNR2L2.value <= threshold=2.8784297704696655
node_2: feature_name=TCL1A	TCL1A.value <= threshold=4.798312664031982
node_3: feature_name=MT-CO1	MT-CO1.value > threshold=0.08947191759943962
node_2521: feature_name=LTB	LTB.value > threshold=0.6038667261600494
node_4173: feature_name=OAZ1	OAZ1.value > threshold=0.143283873796463
node_4615: feature_name=NR4A1	NR4A1.value <= threshold=0.6621004343032837
node_4616: feature_name=NFKBID	NFKBID.value <= threshold=3.283741593360901
node_4617: feature_name=HLA-DQA1	HLA-DQA1.value <= threshold=2.3435752391815186
node_4618: feature_name=PELI1	PELI1.value <= threshold=1.3271203637123108
node_4619: feature_name=PLD4	PLD4.value > threshold=3.3385335206985474
node_4671: feature_name=LDLRAD4	LDLRAD4.value <= threshold=2.8038166761398315
node_4672: feature_name=CYBB	CYBB.value <= threshold=-0.5434646904468536

node_4673: feature_name=ID3	ID3.value > threshold=-0.26271694898605347
Class: COVID-19 Critical	
Rules_1191	passed counts:3
node_0: feature_name=LTB	LTB.value <= threshold=0.994031548500061
node_1: feature_name=MTRNR2L2	MTRNR2L2.value <= threshold=2.8784297704696655
node_2: feature_name=TCL1A	TCL1A.value <= threshold=4.798312664031982
node_3: feature_name=MT-CO1	MT-CO1.value > threshold=0.08947191759943962
node_2521: feature_name=LTB	LTB.value > threshold=0.6038667261600494
node_4173: feature_name=OAZ1	OAZ1.value > threshold=0.143283873796463
node_4615: feature_name=NR4A1	NR4A1.value <= threshold=0.6621004343032837
node_4616: feature_name=NFKBID	NFKBID.value <= threshold=3.283741593360901
node_4617: feature_name=HLA-DQA1	HLA-DQA1.value <= threshold=2.3435752391815186
node_4618: feature_name=PELI1	PELI1.value <= threshold=1.3271203637123108
node_4619: feature_name=PLD4	PLD4.value <= threshold=3.3385335206985474
node_4620: feature_name=MEF2A	MEF2A.value > threshold=1.5926940441131592
node_4658: feature_name=STAG3	STAG3.value <= threshold=-0.2016931176185608
node_4659: feature_name=HLA-DQB1	HLA-DQB1.value <= threshold=1.983833909034729
node_4660: feature_name=CD79A	CD79A.value > threshold=2.912865161895752
node_4662: feature_name=MT-CO3	MT-CO3.value > threshold=0.27337878197431564
Class: Healthy Control	
Rules_1192	passed counts:3
node_0: feature_name=LTB	LTB.value <= threshold=0.994031548500061
node_1: feature_name=MTRNR2L2	MTRNR2L2.value <= threshold=2.8784297704696655
node_2: feature_name=TCL1A	TCL1A.value <= threshold=4.798312664031982
node_3: feature_name=MT-CO1	MT-CO1.value > threshold=0.08947191759943962
node_2521: feature_name=LTB	LTB.value > threshold=0.6038667261600494
node_4173: feature_name=OAZ1	OAZ1.value > threshold=0.143283873796463
node_4615: feature_name=NR4A1	NR4A1.value <= threshold=0.6621004343032837

node_4616: feature_name=NFKBID	NFKBID.value <= threshold=3.283741593360901
node_4617: feature_name=HLA-DQA1	HLA-DQA1.value <= threshold=2.3435752391815186
node_4618: feature_name=PELI1	PELI1.value <= threshold=1.3271203637123108
node_4619: feature_name=PLD4	PLD4.value <= threshold=3.3385335206985474
node_4620: feature_name=MEF2A	MEF2A.value <= threshold=1.5926940441131592
node_4621: feature_name=DAPP1	DAPP1.value <= threshold=2.647637367248535
node_4622: feature_name=STAG3	STAG3.value <= threshold=-0.16521219909191132
node_4623: feature_name=OAZ1	OAZ1.value > threshold=0.2042311355471611
node_4625: feature_name=FAM129C	FAM129C.value > threshold=-0.23615043610334396
node_4639: feature_name=TP53INP1	TP53INP1.value <= threshold=4.941076993942261
node_4640: feature_name=KIAA0125	KIAA0125.value <= threshold=7.376631736755371
node_4641: feature_name=SESN3	SESN3.value > threshold=1.000320941209793
node_4643: feature_name=CASP8	CASP8.value > threshold=-0.5342620015144348
Class: COVID-19 Critical	
Rules_1193	passed counts:3
node_0: feature_name=LTB	LTB.value <= threshold=0.994031548500061
node_1: feature_name=MTRNR2L2	MTRNR2L2.value <= threshold=2.8784297704696655
node_2: feature_name=TCL1A	TCL1A.value <= threshold=4.798312664031982
node_3: feature_name=MT-CO1	MT-CO1.value > threshold=0.08947191759943962
node_2521: feature_name=LTB	LTB.value > threshold=0.6038667261600494
node_4173: feature_name=OAZ1	OAZ1.value > threshold=0.143283873796463
node_4615: feature_name=NR4A1	NR4A1.value <= threshold=0.6621004343032837
node_4616: feature_name=NFKBID	NFKBID.value <= threshold=3.283741593360901
node_4617: feature_name=HLA-DQA1	HLA-DQA1.value <= threshold=2.3435752391815186
node_4618: feature_name=PELI1	PELI1.value <= threshold=1.3271203637123108
node_4619: feature_name=PLD4	PLD4.value <= threshold=3.3385335206985474
node_4620: feature_name=MEF2A	MEF2A.value <= threshold=1.5926940441131592
node_4621: feature_name=DAPP1	DAPP1.value <= threshold=2.647637367248535
node_4622: feature_name=STAG3	STAG3.value <= threshold=-0.16521219909191132

node_4623: feature_name=OAZ1	OAZ1.value > threshold=0.2042311355471611
node_4625: feature_name=FAM129C	FAM129C.value <= threshold=-0.23615043610334396
node_4626: feature_name=TCL1A	TCL1A.value <= threshold=-0.18263818323612213
node_4627: feature_name=RALGPS2	RALGPS2.value <= threshold=0.4531548172235489
node_4628: feature_name=MT-ND6	MT-ND6.value <= threshold=-0.9424236118793488
node_4629: feature_name=AHR	AHR.value > threshold=-0.4223325848579407
Class: Healthy Control	
Rules_1194	passed counts:3
node_0: feature_name=LTB	LTB.value <= threshold=0.994031548500061
node_1: feature_name=MTRNR2L2	MTRNR2L2.value <= threshold=2.8784297704696655
node_2: feature_name=TCL1A	TCL1A.value <= threshold=4.798312664031982
node_3: feature_name=MT-CO1	MT-CO1.value > threshold=0.08947191759943962
node_2521: feature_name=LTB	LTB.value > threshold=0.6038667261600494
node_4173: feature_name=OAZ1	OAZ1.value <= threshold=0.143283873796463
node_4174: feature_name=PLD4	PLD4.value > threshold=2.921932101249695
node_4558: feature_name=HLA-DQA2	HLA-DQA2.value > threshold=1.5485953092575073
node_4598: feature_name=MTRNR2L8	MTRNR2L8.value <= threshold=2.9248507022857666
node_4599: feature_name=HLA-DRB1	HLA-DRB1.value <= threshold=1.4262019395828247
node_4600: feature_name=CD83	CD83.value > threshold=2.5374964475631714
node_4604: feature_name=TCL1A	TCL1A.value > threshold=-0.18191984295845032
Class: Healthy Control	
Rules_1195	passed counts:3
node_0: feature_name=LTB	LTB.value <= threshold=0.994031548500061
node_1: feature_name=MTRNR2L2	MTRNR2L2.value <= threshold=2.8784297704696655
node_2: feature_name=TCL1A	TCL1A.value <= threshold=4.798312664031982
node_3: feature_name=MT-CO1	MT-CO1.value > threshold=0.08947191759943962
node_2521: feature_name=LTB	LTB.value > threshold=0.6038667261600494
node_4173: feature_name=OAZ1	OAZ1.value <= threshold=0.143283873796463

node_4174: feature_name=PLD4	PLD4.value > threshold=2.921932101249695
node_4558: feature_name=HLA-DQA2	HLA-DQA2.value > threshold=1.5485953092575073
node_4598: feature_name=MTRNR2L8	MTRNR2L8.value <= threshold=2.9248507022857666
node_4599: feature_name=HLA-DRB1	HLA-DRB1.value <= threshold=1.4262019395828247
node_4600: feature_name=CD83	CD83.value > threshold=2.5374964475631714
node_4604: feature_name=TCL1A	TCL1A.value <= threshold=-0.18191984295845032
Class: COVID-19 Critical	
Rules_1196	passed counts:3
node_0: feature_name=LTB	LTB.value <= threshold=0.994031548500061
node_1: feature_name=MTRNR2L2	MTRNR2L2.value <= threshold=2.8784297704696655
node_2: feature_name=TCL1A	TCL1A.value <= threshold=4.798312664031982
node_3: feature_name=MT-CO1	MT-CO1.value > threshold=0.08947191759943962
node_2521: feature_name=LTB	LTB.value > threshold=0.6038667261600494
node_4173: feature_name=OAZ1	OAZ1.value <= threshold=0.143283873796463
node_4174: feature_name=PLD4	PLD4.value <= threshold=2.921932101249695
node_4175: feature_name=XIST	XIST.value > threshold=-0.05067425221204758
node_4539: feature_name=HLA-DQB1	HLA-DQB1.value > threshold=2.1246572732925415
node_4549: feature_name=HLA-DQA2	HLA-DQA2.value > threshold=-0.12850885093212128
Class: Healthy Control	
Rules_1197	passed counts:3
node_0: feature_name=LTB	LTB.value <= threshold=0.994031548500061
node_1: feature_name=MTRNR2L2	MTRNR2L2.value <= threshold=2.8784297704696655
node_2: feature_name=TCL1A	TCL1A.value <= threshold=4.798312664031982
node_3: feature_name=MT-CO1	MT-CO1.value > threshold=0.08947191759943962
node_2521: feature_name=LTB	LTB.value > threshold=0.6038667261600494
node_4173: feature_name=OAZ1	OAZ1.value <= threshold=0.143283873796463
node_4174: feature_name=PLD4	PLD4.value <= threshold=2.921932101249695
node_4175: feature_name=XIST	XIST.value > threshold=-0.05067425221204758

node_4539: feature_name=HLA-DQB1	HLA-DQB1.value <= threshold=2.1246572732925415
node_4540: feature_name=ICAM1	ICAM1.value > threshold=-0.22761741280555725
node_4544: feature_name=GRASP	GRASP.value > threshold=0.3840908855199814
node_4546: feature_name=MS4A1	MS4A1.value > threshold=2.620572805404663
Class: COVID-19 Severe	
Rules_1198	passed counts:3
node_0: feature_name=LTB	LTB.value <= threshold=0.994031548500061
node_1: feature_name=MTRNR2L2	MTRNR2L2.value <= threshold=2.8784297704696655
node_2: feature_name=TCL1A	TCL1A.value <= threshold=4.798312664031982
node_3: feature_name=MT-CO1	MT-CO1.value > threshold=0.08947191759943962
node_2521: feature_name=LTB	LTB.value > threshold=0.6038667261600494
node_4173: feature_name=OAZ1	OAZ1.value <= threshold=0.143283873796463
node_4174: feature_name=PLD4	PLD4.value <= threshold=2.921932101249695
node_4175: feature_name=XIST	XIST.value <= threshold=-0.05067425221204758
node_4176: feature_name=MT-CO1	MT-CO1.value > threshold=1.345594048500061
node_4486: feature_name=RALGPS2	RALGPS2.value > threshold=2.7596691846847534
node_4508: feature_name=SNX9	SNX9.value > threshold=2.5712826251983643
node_4526: feature_name=APP	APP.value > threshold=-0.2580544352531433
node_4534: feature_name=HLA-DQB1	HLA-DQB1.value > threshold=3.2880821228027344
Class: COVID-19 Severe	
Rules_1199	passed counts:3
node_0: feature_name=LTB	LTB.value <= threshold=0.994031548500061
node_1: feature_name=MTRNR2L2	MTRNR2L2.value <= threshold=2.8784297704696655
node_2: feature_name=TCL1A	TCL1A.value <= threshold=4.798312664031982
node_3: feature_name=MT-CO1	MT-CO1.value > threshold=0.08947191759943962
node_2521: feature_name=LTB	LTB.value > threshold=0.6038667261600494
node_4173: feature_name=OAZ1	OAZ1.value <= threshold=0.143283873796463
node_4174: feature_name=PLD4	PLD4.value <= threshold=2.921932101249695

node_4175: feature_name=XIST	XIST.value <= threshold=-0.05067425221204758
node_4176: feature_name=MT-CO1	MT-CO1.value > threshold=1.345594048500061
node_4486: feature_name=RALGPS2	RALGPS2.value > threshold=2.7596691846847534
node_4508: feature_name=SNX9	SNX9.value > threshold=2.5712826251983643
node_4526: feature_name=APP	APP.value > threshold=-0.2580544352531433
node_4534: feature_name=HLA-DQB1	HLA-DQB1.value <= threshold=3.2880821228027344
node_4535: feature_name=BOD1L1	BOD1L1.value > threshold=-0.47307854890823364
Class: COVID-19 Critical	
Rules_1200	passed counts:3
node_0: feature_name=LTB	LTB.value <= threshold=0.994031548500061
node_1: feature_name=MTRNR2L2	MTRNR2L2.value <= threshold=2.8784297704696655
node_2: feature_name=TCL1A	TCL1A.value <= threshold=4.798312664031982
node_3: feature_name=MT-CO1	MT-CO1.value > threshold=0.08947191759943962
node_2521: feature_name=LTB	LTB.value > threshold=0.6038667261600494
node_4173: feature_name=OAZ1	OAZ1.value <= threshold=0.143283873796463
node_4174: feature_name=PLD4	PLD4.value <= threshold=2.921932101249695
node_4175: feature_name=XIST	XIST.value <= threshold=-0.05067425221204758
node_4176: feature_name=MT-CO1	MT-CO1.value > threshold=1.345594048500061
node_4486: feature_name=RALGPS2	RALGPS2.value > threshold=2.7596691846847534
node_4508: feature_name=SNX9	SNX9.value <= threshold=2.5712826251983643
node_4509: feature_name=NFKBIZ	NFKBIZ.value > threshold=-0.8055458068847656
node_4511: feature_name=MT-CO3	MT-CO3.value > threshold=0.9913833141326904
node_4515: feature_name=MT-CO2	MT-CO2.value > threshold=2.31252920627594
node_4523: feature_name=GRASP	GRASP.value > threshold=-0.3890672028064728
Class: COVID-19 Critical	
Rules_1201	passed counts:3
node_0: feature_name=LTB	LTB.value <= threshold=0.994031548500061
node_1: feature_name=MTRNR2L2	MTRNR2L2.value <= threshold=2.8784297704696655

node_2: feature_name=TCL1A	TCL1A.value <= threshold=4.798312664031982
node_3: feature_name=MT-CO1	MT-CO1.value > threshold=0.08947191759943962
node_2521: feature_name=LTB	LTB.value > threshold=0.6038667261600494
node_4173: feature_name=OAZ1	OAZ1.value <= threshold=0.143283873796463
node_4174: feature_name=PLD4	PLD4.value <= threshold=2.921932101249695
node_4175: feature_name=XIST	XIST.value <= threshold=-0.05067425221204758
node_4176: feature_name=MT-CO1	MT-CO1.value <= threshold=1.345594048500061
node_4177: feature_name=HLA-DQB1	HLA-DQB1.value > threshold=1.927736520767212
node_4353: feature_name=ITM2C	ITM2C.value <= threshold=-0.12329206243157387
node_4354: feature_name=MX1	MX1.value <= threshold=-0.4356398284435272
node_4355: feature_name=HLA-DQB1	HLA-DQB1.value > threshold=2.8865538835525513
node_4419: feature_name=DAPP1	DAPP1.value <= threshold=-0.30035753548145294
node_4420: feature_name=RHOB	RHOB.value > threshold=1.2027948498725891
node_4422: feature_name=HLA-DPB1	HLA-DPB1.value > threshold=1.7014628052711487
Class: Healthy Control	
Rules_1202	passed counts:3
node_0: feature_name=LTB	LTB.value <= threshold=0.994031548500061
node_1: feature_name=MTRNR2L2	MTRNR2L2.value <= threshold=2.8784297704696655
node_2: feature_name=TCL1A	TCL1A.value <= threshold=4.798312664031982
node_3: feature_name=MT-CO1	MT-CO1.value > threshold=0.08947191759943962
node_2521: feature_name=LTB	LTB.value > threshold=0.6038667261600494
node_4173: feature_name=OAZ1	OAZ1.value <= threshold=0.143283873796463
node_4174: feature_name=PLD4	PLD4.value <= threshold=2.921932101249695
node_4175: feature_name=XIST	XIST.value <= threshold=-0.05067425221204758
node_4176: feature_name=MT-CO1	MT-CO1.value <= threshold=1.345594048500061
node_4177: feature_name=HLA-DQB1	HLA-DQB1.value > threshold=1.927736520767212
node_4353: feature_name=ITM2C	ITM2C.value <= threshold=-0.12329206243157387
node_4354: feature_name=MX1	MX1.value <= threshold=-0.4356398284435272
node_4355: feature_name=HLA-DQB1	HLA-DQB1.value <= threshold=2.8865538835525513

node_4356: feature_name=SNX9	SNX9.value > threshold=1.002076417207718
node_4384: feature_name=HLA-DRB5	HLA-DRB5.value > threshold=1.0937854647636414
node_4396: feature_name=SLC7A5	SLC7A5.value > threshold=1.3017424941062927
node_4416: feature_name=MARCH1	MARCH1.value <= threshold=-0.34805573523044586
Class: Healthy Control	
Rules_1203	passed counts:3
node_0: feature_name=LTB	LTB.value <= threshold=0.994031548500061
node_1: feature_name=MTRNR2L2	MTRNR2L2.value <= threshold=2.8784297704696655
node_2: feature_name=TCL1A	TCL1A.value <= threshold=4.798312664031982
node_3: feature_name=MT-CO1	MT-CO1.value > threshold=0.08947191759943962
node_2521: feature_name=LTB	LTB.value > threshold=0.6038667261600494
node_4173: feature_name=OAZ1	OAZ1.value <= threshold=0.143283873796463
node_4174: feature_name=PLD4	PLD4.value <= threshold=2.921932101249695
node_4175: feature_name=XIST	XIST.value <= threshold=-0.05067425221204758
node_4176: feature_name=MT-CO1	MT-CO1.value <= threshold=1.345594048500061
node_4177: feature_name=HLA-DQB1	HLA-DQB1.value > threshold=1.927736520767212
node_4353: feature_name=ITM2C	ITM2C.value <= threshold=-0.12329206243157387
node_4354: feature_name=MX1	MX1.value <= threshold=-0.4356398284435272
node_4355: feature_name=HLA-DQB1	HLA-DQB1.value <= threshold=2.8865538835525513
node_4356: feature_name=SNX9	SNX9.value > threshold=1.002076417207718
node_4384: feature_name=HLA-DRB5	HLA-DRB5.value > threshold=1.0937854647636414
node_4396: feature_name=SLC7A5	SLC7A5.value <= threshold=1.3017424941062927
node_4397: feature_name=BACH1	BACH1.value <= threshold=2.4516913890838623
node_4398: feature_name=BANK1	BANK1.value > threshold=3.624985694885254
node_4410: feature_name=ATF5	ATF5.value > threshold=1.2557298392057419
Class: COVID-19 Critical	
Rules_1204	passed counts:3
node_0: feature_name=LTB	LTB.value <= threshold=0.994031548500061

node_1: feature_name=MTRNR2L2	MTRNR2L2.value <= threshold=2.8784297704696655
node_2: feature_name=TCL1A	TCL1A.value <= threshold=4.798312664031982
node_3: feature_name=MT-CO1	MT-CO1.value > threshold=0.08947191759943962
node_2521: feature_name=LTB	LTB.value > threshold=0.6038667261600494
node_4173: feature_name=OAZ1	OAZ1.value <= threshold=0.143283873796463
node_4174: feature_name=PLD4	PLD4.value <= threshold=2.921932101249695
node_4175: feature_name=XIST	XIST.value <= threshold=-0.05067425221204758
node_4176: feature_name=MT-CO1	MT-CO1.value <= threshold=1.345594048500061
node_4177: feature_name=HLA-DQB1	HLA-DQB1.value > threshold=1.927736520767212
node_4353: feature_name=ITM2C	ITM2C.value <= threshold=-0.12329206243157387
node_4354: feature_name=MX1	MX1.value <= threshold=-0.4356398284435272
node_4355: feature_name=HLA-DQB1	HLA-DQB1.value <= threshold=2.8865538835525513
node_4356: feature_name=SNX9	SNX9.value > threshold=1.002076417207718
node_4384: feature_name=HLA-DRB5	HLA-DRB5.value > threshold=1.0937854647636414
node_4396: feature_name=SLC7A5	SLC7A5.value <= threshold=1.3017424941062927
node_4397: feature_name=BACH1	BACH1.value <= threshold=2.4516913890838623
node_4398: feature_name=BANK1	BANK1.value > threshold=3.624985694885254
node_4410: feature_name=ATF5	ATF5.value <= threshold=1.2557298392057419
node_4411: feature_name=NR4A1	NR4A1.value <= threshold=2.490843117237091
Class: Healthy Control	
Rules_1205	passed counts:3
node_0: feature_name=LTB	LTB.value <= threshold=0.994031548500061
node_1: feature_name=MTRNR2L2	MTRNR2L2.value <= threshold=2.8784297704696655
node_2: feature_name=TCL1A	TCL1A.value <= threshold=4.798312664031982
node_3: feature_name=MT-CO1	MT-CO1.value > threshold=0.08947191759943962
node_2521: feature_name=LTB	LTB.value > threshold=0.6038667261600494
node_4173: feature_name=OAZ1	OAZ1.value <= threshold=0.143283873796463
node_4174: feature_name=PLD4	PLD4.value <= threshold=2.921932101249695
node_4175: feature_name=XIST	XIST.value <= threshold=-0.05067425221204758

node_4176: feature_name=MT-CO1	MT-CO1.value <= threshold=1.345594048500061
node_4177: feature_name=HLA-DQB1	HLA-DQB1.value <= threshold=1.927736520767212
node_4178: feature_name=BACH1	BACH1.value > threshold=-0.5349778532981873
node_4236: feature_name=TPM1	TPM1.value <= threshold=3.516477584838867
node_4237: feature_name=NR4A1	NR4A1.value > threshold=-0.274935707449913
node_4277: feature_name=HLA-DPA1	HLA-DPA1.value > threshold=1.8894080519676208
node_4339: feature_name=PARP14	PARP14.value <= threshold=0.2787386327981949
node_4340: feature_name=RGS2	RGS2.value > threshold=0.10973784327507019
node_4342: feature_name=SNX9	SNX9.value > threshold=0.07674053311347961
node_4344: feature_name=RNF144B	RNF144B.value > threshold=-0.140806145966053
Class: COVID-19 Critical	
Rules_1206	passed counts:3
node_0: feature_name=LTB	LTB.value <= threshold=0.994031548500061
node_1: feature_name=MTRNR2L2	MTRNR2L2.value <= threshold=2.8784297704696655
node_2: feature_name=TCL1A	TCL1A.value <= threshold=4.798312664031982
node_3: feature_name=MT-CO1	MT-CO1.value > threshold=0.08947191759943962
node_2521: feature_name=LTB	LTB.value > threshold=0.6038667261600494
node_4173: feature_name=OAZ1	OAZ1.value <= threshold=0.143283873796463
node_4174: feature_name=PLD4	PLD4.value <= threshold=2.921932101249695
node_4175: feature_name=XIST	XIST.value <= threshold=-0.05067425221204758
node_4176: feature_name=MT-CO1	MT-CO1.value <= threshold=1.345594048500061
node_4177: feature_name=HLA-DQB1	HLA-DQB1.value <= threshold=1.927736520767212
node_4178: feature_name=BACH1	BACH1.value > threshold=-0.5349778532981873
node_4236: feature_name=TPM1	TPM1.value <= threshold=3.516477584838867
node_4237: feature_name=NR4A1	NR4A1.value > threshold=-0.274935707449913
node_4277: feature_name=HLA-DPA1	HLA-DPA1.value > threshold=1.8894080519676208
node_4339: feature_name=PARP14	PARP14.value <= threshold=0.2787386327981949
node_4340: feature_name=RGS2	RGS2.value > threshold=0.10973784327507019
node_4342: feature_name=SNX9	SNX9.value > threshold=0.07674053311347961

node_4344: feature_name=RNF144B	RNF144B.value <= threshold=-0.140806145966053
Class: COVID-19 Severe	
Rules_1207	passed counts:3
node_0: feature_name=LTB	LTB.value <= threshold=0.994031548500061
node_1: feature_name=MTRNR2L2	MTRNR2L2.value <= threshold=2.8784297704696655
node_2: feature_name=TCL1A	TCL1A.value <= threshold=4.798312664031982
node_3: feature_name=MT-CO1	MT-CO1.value > threshold=0.08947191759943962
node_2521: feature_name=LTB	LTB.value > threshold=0.6038667261600494
node_4173: feature_name=OAZ1	OAZ1.value <= threshold=0.143283873796463
node_4174: feature_name=PLD4	PLD4.value <= threshold=2.921932101249695
node_4175: feature_name=XIST	XIST.value <= threshold=-0.05067425221204758
node_4176: feature_name=MT-CO1	MT-CO1.value <= threshold=1.345594048500061
node_4177: feature_name=HLA-DQB1	HLA-DQB1.value <= threshold=1.927736520767212
node_4178: feature_name=BACH1	BACH1.value > threshold=-0.5349778532981873
node_4236: feature_name=TPM1	TPM1.value <= threshold=3.516477584838867
node_4237: feature_name=NR4A1	NR4A1.value > threshold=-0.274935707449913
node_4277: feature_name=HLA-DPA1	HLA-DPA1.value > threshold=1.8894080519676208
node_4339: feature_name=PARP14	PARP14.value <= threshold=0.2787386327981949
node_4340: feature_name=RGS2	RGS2.value > threshold=0.10973784327507019
node_4342: feature_name=SNX9	SNX9.value <= threshold=0.07674053311347961
Class: Healthy Control	
Rules_1208	passed counts:3
node_0: feature_name=LTB	LTB.value <= threshold=0.994031548500061
node_1: feature_name=MTRNR2L2	MTRNR2L2.value <= threshold=2.8784297704696655
node_2: feature_name=TCL1A	TCL1A.value <= threshold=4.798312664031982
node_3: feature_name=MT-CO1	MT-CO1.value > threshold=0.08947191759943962
node_2521: feature_name=LTB	LTB.value > threshold=0.6038667261600494
node_4173: feature_name=OAZ1	OAZ1.value <= threshold=0.143283873796463

node_4174: feature_name=PLD4	PLD4.value <= threshold=2.921932101249695
node_4175: feature_name=XIST	XIST.value <= threshold=-0.05067425221204758
node_4176: feature_name=MT-CO1	MT-CO1.value <= threshold=1.345594048500061
node_4177: feature_name=HLA-DQB1	HLA-DQB1.value <= threshold=1.927736520767212
node_4178: feature_name=BACH1	BACH1.value > threshold=-0.5349778532981873
node_4236: feature_name=TPM1	TPM1.value <= threshold=3.516477584838867
node_4237: feature_name=NR4A1	NR4A1.value <= threshold=-0.274935707449913
node_4238: feature_name=VPREB3	VPREB3.value <= threshold=5.024149179458618
node_4239: feature_name=YWHAH	YWHAH.value <= threshold=4.7504096031188965
node_4240: feature_name=NAIP	NAIP.value <= threshold=4.030007481575012
node_4241: feature_name=CD38	CD38.value > threshold=-0.3387429714202881
node_4243: feature_name=MS4A1	MS4A1.value <= threshold=5.195746660232544
node_4244: feature_name=TRIB1	TRIB1.value <= threshold=2.9039160013198853
node_4245: feature_name=RGS2	RGS2.value > threshold=-0.8980970978736877
node_4249: feature_name=PLD4	PLD4.value <= threshold=2.8284080028533936
node_4250: feature_name=GRAP2	GRAP2.value > threshold=2.0700820088386536
node_4260: feature_name=KIAA0125	KIAA0125.value > threshold=-0.1811859980225563
Class: COVID-19 Critical	
Rules_1209	passed counts:3
node_0: feature_name=LTB	LTB.value <= threshold=0.994031548500061
node_1: feature_name=MTRNR2L2	MTRNR2L2.value <= threshold=2.8784297704696655
node_2: feature_name=TCL1A	TCL1A.value <= threshold=4.798312664031982
node_3: feature_name=MT-CO1	MT-CO1.value > threshold=0.08947191759943962
node_2521: feature_name=LTB	LTB.value > threshold=0.6038667261600494
node_4173: feature_name=OAZ1	OAZ1.value <= threshold=0.143283873796463
node_4174: feature_name=PLD4	PLD4.value <= threshold=2.921932101249695
node_4175: feature_name=XIST	XIST.value <= threshold=-0.05067425221204758
node_4176: feature_name=MT-CO1	MT-CO1.value <= threshold=1.345594048500061
node_4177: feature_name=HLA-DQB1	HLA-DQB1.value <= threshold=1.927736520767212

node_4178: feature_name=BACH1	BACH1.value <= threshold=-0.5349778532981873
node_4179: feature_name=CD79B	CD79B.value > threshold=1.6518195271492004
node_4201: feature_name=MT-CO2	MT-CO2.value <= threshold=0.5007440596818924
node_4202: feature_name=PELI1	PELI1.value > threshold=0.9399837851524353
node_4212: feature_name=TTN	TTN.value > threshold=-0.3203035891056061
node_4214: feature_name=RGS2	RGS2.value > threshold=0.8960320353507996
node_4218: feature_name=SESN3	SESN3.value <= threshold=1.479651689529419
Class: Healthy Control	
Rules_1210	passed counts:3
node_0: feature_name=LTB	LTB.value <= threshold=0.994031548500061
node_1: feature_name=MTRNR2L2	MTRNR2L2.value <= threshold=2.8784297704696655
node_2: feature_name=TCL1A	TCL1A.value <= threshold=4.798312664031982
node_3: feature_name=MT-CO1	MT-CO1.value > threshold=0.08947191759943962
node_2521: feature_name=LTB	LTB.value > threshold=0.6038667261600494
node_4173: feature_name=OAZ1	OAZ1.value <= threshold=0.143283873796463
node_4174: feature_name=PLD4	PLD4.value <= threshold=2.921932101249695
node_4175: feature_name=XIST	XIST.value <= threshold=-0.05067425221204758
node_4176: feature_name=MT-CO1	MT-CO1.value <= threshold=1.345594048500061
node_4177: feature_name=HLA-DQB1	HLA-DQB1.value <= threshold=1.927736520767212
node_4178: feature_name=BACH1	BACH1.value <= threshold=-0.5349778532981873
node_4179: feature_name=CD79B	CD79B.value > threshold=1.6518195271492004
node_4201: feature_name=MT-CO2	MT-CO2.value <= threshold=0.5007440596818924
node_4202: feature_name=PELI1	PELI1.value <= threshold=0.9399837851524353
node_4203: feature_name=STAG3	STAG3.value > threshold=4.226563453674316
node_4209: feature_name=BOD1L1	BOD1L1.value > threshold=0.3161502480506897
Class: COVID-19 Critical	
Rules_1211	passed counts:3
node_0: feature_name=LTB	LTB.value <= threshold=0.994031548500061

node_1: feature_name=MTRNR2L2	MTRNR2L2.value <= threshold=2.8784297704696655
node_2: feature_name=TCL1A	TCL1A.value <= threshold=4.798312664031982
node_3: feature_name=MT-CO1	MT-CO1.value > threshold=0.08947191759943962
node_2521: feature_name=LTB	LTB.value <= threshold=0.6038667261600494
node_2522: feature_name=HLA-DQB1	HLA-DQB1.value > threshold=2.1053054332733154
node_3550: feature_name=XIST	XIST.value <= threshold=0.7716337237507105
node_3551: feature_name=CD79B	CD79B.value > threshold=2.9426796436309814
node_4039: feature_name=IFI44L	IFI44L.value <= threshold=1.8637521862983704
node_4040: feature_name=SNX9	SNX9.value > threshold=1.274232804775238
node_4096: feature_name=RALGPS2	RALGPS2.value > threshold=2.3599878549575806
node_4118: feature_name=SNX29P2	SNX29P2.value > threshold=0.880477637052536
node_4132: feature_name=BOD1L1	BOD1L1.value > threshold=1.5053239464759827
Class: COVID-19 Critical	
Rules_1212	passed counts:3
node_0: feature_name=LTB	LTB.value <= threshold=0.994031548500061
node_1: feature_name=MTRNR2L2	MTRNR2L2.value <= threshold=2.8784297704696655
node_2: feature_name=TCL1A	TCL1A.value <= threshold=4.798312664031982
node_3: feature_name=MT-CO1	MT-CO1.value > threshold=0.08947191759943962
node_2521: feature_name=LTB	LTB.value <= threshold=0.6038667261600494
node_2522: feature_name=HLA-DQB1	HLA-DQB1.value > threshold=2.1053054332733154
node_3550: feature_name=XIST	XIST.value <= threshold=0.7716337237507105
node_3551: feature_name=CD79B	CD79B.value > threshold=2.9426796436309814
node_4039: feature_name=IFI44L	IFI44L.value <= threshold=1.8637521862983704
node_4040: feature_name=SNX9	SNX9.value > threshold=1.274232804775238
node_4096: feature_name=RALGPS2	RALGPS2.value > threshold=2.3599878549575806
node_4118: feature_name=SNX29P2	SNX29P2.value <= threshold=0.880477637052536
node_4119: feature_name=FAM129C	FAM129C.value > threshold=3.415250778198242
node_4125: feature_name=IGJ	IGJ.value <= threshold=-0.2685837149620056
node_4126: feature_name=S100A6	S100A6.value > threshold=-1.4672366380691528

Class: COVID-19 Severe	
Rules_1213	passed counts:3
node_0: feature_name=LTB	LTB.value <= threshold=0.994031548500061
node_1: feature_name=MTRNR2L2	MTRNR2L2.value <= threshold=2.8784297704696655
node_2: feature_name=TCL1A	TCL1A.value <= threshold=4.798312664031982
node_3: feature_name=MT-CO1	MT-CO1.value > threshold=0.08947191759943962
node_2521: feature_name=LTB	LTB.value <= threshold=0.6038667261600494
node_2522: feature_name=HLA-DQB1	HLA-DQB1.value > threshold=2.1053054332733154
node_3550: feature_name=XIST	XIST.value <= threshold=0.7716337237507105
node_3551: feature_name=CD79B	CD79B.value > threshold=2.9426796436309814
node_4039: feature_name=IFI44L	IFI44L.value <= threshold=1.8637521862983704
node_4040: feature_name=SNX9	SNX9.value > threshold=1.274232804775238
node_4096: feature_name=RALGPS2	RALGPS2.value <= threshold=2.3599878549575806
node_4097: feature_name=HLA-DQB1	HLA-DQB1.value > threshold=2.9625227451324463
node_4111: feature_name=FTH1	FTH1.value <= threshold=-0.30264076590538025
node_4112: feature_name=FTH1	FTH1.value > threshold=-3.5435677766799927
Class: COVID-19 Critical	
Rules_1214	passed counts:3
node_0: feature_name=LTB	LTB.value <= threshold=0.994031548500061
node_1: feature_name=MTRNR2L2	MTRNR2L2.value <= threshold=2.8784297704696655
node_2: feature_name=TCL1A	TCL1A.value <= threshold=4.798312664031982
node_3: feature_name=MT-CO1	MT-CO1.value > threshold=0.08947191759943962
node_2521: feature_name=LTB	LTB.value <= threshold=0.6038667261600494
node_2522: feature_name=HLA-DQB1	HLA-DQB1.value > threshold=2.1053054332733154
node_3550: feature_name=XIST	XIST.value <= threshold=0.7716337237507105
node_3551: feature_name=CD79B	CD79B.value > threshold=2.9426796436309814
node_4039: feature_name=IFI44L	IFI44L.value <= threshold=1.8637521862983704
node_4040: feature_name=SNX9	SNX9.value <= threshold=1.274232804775238

node_4041: feature_name=CASP8	CASP8.value <= threshold=-0.574097603559494
node_4042: feature_name=NFKBID	NFKBID.value > threshold=1.49756920337677
node_4056: feature_name=MIR24-2	MIR24-2.value <= threshold=0.2797542065382004
node_4057: feature_name=MEF2C	MEF2C.value <= threshold=2.6051090955734253
node_4058: feature_name=BOD1L1	BOD1L1.value > threshold=-0.476860374212265
node_4062: feature_name=RHOB	RHOB.value > threshold=1.5522340536117554
node_4066: feature_name=APP	APP.value <= threshold=0.9896390289068222
Class: COVID-19 Critical	
Rules_1215	passed counts:3
node_0: feature_name=LTB	LTB.value <= threshold=0.994031548500061
node_1: feature_name=MTRNR2L2	MTRNR2L2.value <= threshold=2.8784297704696655
node_2: feature_name=TCL1A	TCL1A.value <= threshold=4.798312664031982
node_3: feature_name=MT-CO1	MT-CO1.value > threshold=0.08947191759943962
node_2521: feature_name=LTB	LTB.value <= threshold=0.6038667261600494
node_2522: feature_name=HLA-DQB1	HLA-DQB1.value > threshold=2.1053054332733154
node_3550: feature_name=XIST	XIST.value <= threshold=0.7716337237507105
node_3551: feature_name=CD79B	CD79B.value <= threshold=2.9426796436309814
node_3552: feature_name=HLA-DQB1	HLA-DQB1.value > threshold=2.713674306869507
node_3854: feature_name=MEF2C	MEF2C.value > threshold=-0.3721291869878769
node_3882: feature_name=IFI44L	IFI44L.value <= threshold=2.5516022443771362
node_3883: feature_name=FCER2	FCER2.value > threshold=-0.21487552672624588
node_3977: feature_name=IGJ	IGJ.value > threshold=2.123470664024353
node_4025: feature_name=PELI1	PELI1.value <= threshold=-0.6190133392810822
node_4026: feature_name=VCL	VCL.value > threshold=-0.3592749983072281
Class: Healthy Control	
Rules_1216	passed counts:3
node_0: feature_name=LTB	LTB.value <= threshold=0.994031548500061
node_1: feature_name=MTRNR2L2	MTRNR2L2.value <= threshold=2.8784297704696655

node_2: feature_name=TCL1A	TCL1A.value <= threshold=4.798312664031982
node_3: feature_name=MT-CO1	MT-CO1.value > threshold=0.08947191759943962
node_2521: feature_name=LTB	LTB.value <= threshold=0.6038667261600494
node_2522: feature_name=HLA-DQB1	HLA-DQB1.value > threshold=2.1053054332733154
node_3550: feature_name=XIST	XIST.value <= threshold=0.7716337237507105
node_3551: feature_name=CD79B	CD79B.value <= threshold=2.9426796436309814
node_3552: feature_name=HLA-DQB1	HLA-DQB1.value > threshold=2.713674306869507
node_3854: feature_name=MEF2C	MEF2C.value > threshold=-0.3721291869878769
node_3882: feature_name=IFI44L	IFI44L.value <= threshold=2.5516022443771362
node_3883: feature_name=FCER2	FCER2.value > threshold=-0.21487552672624588
node_3977: feature_name=IGJ	IGJ.value <= threshold=2.123470664024353
node_3978: feature_name=S100A6	S100A6.value > threshold=-1.7107264399528503
node_3982: feature_name=MT-CO1	MT-CO1.value <= threshold=1.5725501775741577
node_3983: feature_name=SNX29P2	SNX29P2.value > threshold=3.1593780517578125
node_4013: feature_name=KIAA0125	KIAA0125.value > threshold=-0.16171331703662872
node_4015: feature_name=SNX9	SNX9.value <= threshold=3.6881524324417114
node_4016: feature_name=GRAP2	GRAP2.value > threshold=-0.4084753841161728
Class: COVID-19 Critical	
Rules_1217	passed counts:3
node_0: feature_name=LTB	LTB.value <= threshold=0.994031548500061
node_1: feature_name=MTRNR2L2	MTRNR2L2.value <= threshold=2.8784297704696655
node_2: feature_name=TCL1A	TCL1A.value <= threshold=4.798312664031982
node_3: feature_name=MT-CO1	MT-CO1.value > threshold=0.08947191759943962
node_2521: feature_name=LTB	LTB.value <= threshold=0.6038667261600494
node_2522: feature_name=HLA-DQB1	HLA-DQB1.value > threshold=2.1053054332733154
node_3550: feature_name=XIST	XIST.value <= threshold=0.7716337237507105
node_3551: feature_name=CD79B	CD79B.value <= threshold=2.9426796436309814
node_3552: feature_name=HLA-DQB1	HLA-DQB1.value > threshold=2.713674306869507
node_3854: feature_name=MEF2C	MEF2C.value > threshold=-0.3721291869878769

node_3882: feature_name=IFI44L	IFI44L.value <= threshold=2.5516022443771362
node_3883: feature_name=FCER2	FCER2.value > threshold=-0.21487552672624588
node_3977: feature_name=IGJ	IGJ.value <= threshold=2.123470664024353
node_3978: feature_name=S100A6	S100A6.value > threshold=-1.7107264399528503
node_3982: feature_name=MT-CO1	MT-CO1.value <= threshold=1.5725501775741577
node_3983: feature_name=SNX29P2	SNX29P2.value <= threshold=3.1593780517578125
node_3984: feature_name=IL8	IL8.value <= threshold=-0.28026726841926575
node_3985: feature_name=NAMPT	NAMPT.value > threshold=-0.5902779400348663
node_3991: feature_name=PELI1	PELI1.value > threshold=3.5653313398361206
node_4009: feature_name=HLA-DRB1	HLA-DRB1.value <= threshold=1.7944364547729492
Class: COVID-19 Severe	
Rules_1218	passed counts:3
node_0: feature_name=LTB	LTB.value <= threshold=0.994031548500061
node_1: feature_name=MTRNR2L2	MTRNR2L2.value <= threshold=2.8784297704696655
node_2: feature_name=TCL1A	TCL1A.value <= threshold=4.798312664031982
node_3: feature_name=MT-CO1	MT-CO1.value > threshold=0.08947191759943962
node_2521: feature_name=LTB	LTB.value <= threshold=0.6038667261600494
node_2522: feature_name=HLA-DQB1	HLA-DQB1.value > threshold=2.1053054332733154
node_3550: feature_name=XIST	XIST.value <= threshold=0.7716337237507105
node_3551: feature_name=CD79B	CD79B.value <= threshold=2.9426796436309814
node_3552: feature_name=HLA-DQB1	HLA-DQB1.value > threshold=2.713674306869507
node_3854: feature_name=MEF2C	MEF2C.value > threshold=-0.3721291869878769
node_3882: feature_name=IFI44L	IFI44L.value <= threshold=2.5516022443771362
node_3883: feature_name=FCER2	FCER2.value > threshold=-0.21487552672624588
node_3977: feature_name=IGJ	IGJ.value <= threshold=2.123470664024353
node_3978: feature_name=S100A6	S100A6.value > threshold=-1.7107264399528503
node_3982: feature_name=MT-CO1	MT-CO1.value <= threshold=1.5725501775741577
node_3983: feature_name=SNX29P2	SNX29P2.value <= threshold=3.1593780517578125
node_3984: feature_name=IL8	IL8.value <= threshold=-0.28026726841926575

node_3985: feature_name=NAMPT	NAMPT.value > threshold=-0.5902779400348663
node_3991: feature_name=PELI1	PELI1.value <= threshold=3.5653313398361206
node_3992: feature_name=IRF8	IRF8.value <= threshold=-0.34819670021533966
node_3993: feature_name=NFKBID	NFKBID.value <= threshold=1.719421625137329
node_3994: feature_name=OAZ1	OAZ1.value <= threshold=-0.3672443702816963
Class: COVID-19 Severe	
Rules_1219	passed counts:3
node_0: feature_name=LTB	LTB.value <= threshold=0.994031548500061
node_1: feature_name=MTRNR2L2	MTRNR2L2.value <= threshold=2.8784297704696655
node_2: feature_name=TCL1A	TCL1A.value <= threshold=4.798312664031982
node_3: feature_name=MT-CO1	MT-CO1.value > threshold=0.08947191759943962
node_2521: feature_name=LTB	LTB.value <= threshold=0.6038667261600494
node_2522: feature_name=HLA-DQB1	HLA-DQB1.value > threshold=2.1053054332733154
node_3550: feature_name=XIST	XIST.value <= threshold=0.7716337237507105
node_3551: feature_name=CD79B	CD79B.value <= threshold=2.9426796436309814
node_3552: feature_name=HLA-DQB1	HLA-DQB1.value > threshold=2.713674306869507
node_3854: feature_name=MEF2C	MEF2C.value > threshold=-0.3721291869878769
node_3882: feature_name=IFI44L	IFI44L.value <= threshold=2.5516022443771362
node_3883: feature_name=FCER2	FCER2.value <= threshold=-0.21487552672624588
node_3884: feature_name=HLA-DQA2	HLA-DQA2.value > threshold=-0.12580305337905884
node_3954: feature_name=SNX9	SNX9.value <= threshold=-0.5673478543758392
node_3955: feature_name=CD83	CD83.value <= threshold=2.9509599208831787
node_3956: feature_name=DAPP1	DAPP1.value > threshold=-0.2832452803850174
node_3958: feature_name=BOD1L1	BOD1L1.value <= threshold=2.6149379312992096
Class: Healthy Control	
Rules_1220	passed counts:3
node_0: feature_name=LTB	LTB.value <= threshold=0.994031548500061
node_1: feature_name=MTRNR2L2	MTRNR2L2.value <= threshold=2.8784297704696655

node_2: feature_name=TCL1A	TCL1A.value <= threshold=4.798312664031982
node_3: feature_name=MT-CO1	MT-CO1.value > threshold=0.08947191759943962
node_2521: feature_name=LTB	LTB.value <= threshold=0.6038667261600494
node_2522: feature_name=HLA-DQB1	HLA-DQB1.value > threshold=2.1053054332733154
node_3550: feature_name=XIST	XIST.value <= threshold=0.7716337237507105
node_3551: feature_name=CD79B	CD79B.value <= threshold=2.9426796436309814
node_3552: feature_name=HLA-DQB1	HLA-DQB1.value > threshold=2.713674306869507
node_3854: feature_name=MEF2C	MEF2C.value > threshold=-0.3721291869878769
node_3882: feature_name=IFI44L	IFI44L.value <= threshold=2.5516022443771362
node_3883: feature_name=FCER2	FCER2.value <= threshold=-0.21487552672624588
node_3884: feature_name=HLA-DQA2	HLA-DQA2.value <= threshold=-0.12580305337905884
node_3885: feature_name=SLC7A5	SLC7A5.value <= threshold=-0.4906871169805527
node_3886: feature_name=VPREB3	VPREB3.value <= threshold=5.492124557495117
node_3887: feature_name=CYBB	CYBB.value <= threshold=2.720046043395996
node_3888: feature_name=CD79A	CD79A.value > threshold=3.3531476259231567
node_3930: feature_name=OAZ1	OAZ1.value > threshold=0.8667621314525604
Class: Healthy Control	
Rules_1221	passed counts:3
node_0: feature_name=LTB	LTB.value <= threshold=0.994031548500061
node_1: feature_name=MTRNR2L2	MTRNR2L2.value <= threshold=2.8784297704696655
node_2: feature_name=TCL1A	TCL1A.value <= threshold=4.798312664031982
node_3: feature_name=MT-CO1	MT-CO1.value > threshold=0.08947191759943962
node_2521: feature_name=LTB	LTB.value <= threshold=0.6038667261600494
node_2522: feature_name=HLA-DQB1	HLA-DQB1.value > threshold=2.1053054332733154
node_3550: feature_name=XIST	XIST.value <= threshold=0.7716337237507105
node_3551: feature_name=CD79B	CD79B.value <= threshold=2.9426796436309814
node_3552: feature_name=HLA-DQB1	HLA-DQB1.value > threshold=2.713674306869507
node_3854: feature_name=MEF2C	MEF2C.value > threshold=-0.3721291869878769
node_3882: feature_name=IFI44L	IFI44L.value <= threshold=2.5516022443771362

node_3883: feature_name=FCER2	FCER2.value <= threshold=-0.21487552672624588
node_3884: feature_name=HLA-DQA2	HLA-DQA2.value <= threshold=-0.12580305337905884
node_3885: feature_name=SLC7A5	SLC7A5.value <= threshold=-0.4906871169805527
node_3886: feature_name=VPREB3	VPREB3.value <= threshold=5.492124557495117
node_3887: feature_name=CYBB	CYBB.value <= threshold=2.720046043395996
node_3888: feature_name=CD79A	CD79A.value <= threshold=3.3531476259231567
node_3889: feature_name=HLA-DPA1	HLA-DPA1.value > threshold=1.6232598423957825
node_3911: feature_name=HLA-DQA1	HLA-DQA1.value <= threshold=3.2045756578445435
node_3912: feature_name=GPX1	GPX1.value > threshold=0.37351667881011963
node_3918: feature_name=EGR1	EGR1.value <= threshold=-0.3214186131954193
Class: COVID-19 Critical	
Rules_1222	passed counts:3
node_0: feature_name=LTB	LTB.value <= threshold=0.994031548500061
node_1: feature_name=MTRNR2L2	MTRNR2L2.value <= threshold=2.8784297704696655
node_2: feature_name=TCL1A	TCL1A.value <= threshold=4.798312664031982
node_3: feature_name=MT-CO1	MT-CO1.value > threshold=0.08947191759943962
node_2521: feature_name=LTB	LTB.value <= threshold=0.6038667261600494
node_2522: feature_name=HLA-DQB1	HLA-DQB1.value > threshold=2.1053054332733154
node_3550: feature_name=XIST	XIST.value <= threshold=0.7716337237507105
node_3551: feature_name=CD79B	CD79B.value <= threshold=2.9426796436309814
node_3552: feature_name=HLA-DQB1	HLA-DQB1.value > threshold=2.713674306869507
node_3854: feature_name=MEF2C	MEF2C.value > threshold=-0.3721291869878769
node_3882: feature_name=IFI44L	IFI44L.value <= threshold=2.5516022443771362
node_3883: feature_name=FCER2	FCER2.value <= threshold=-0.21487552672624588
node_3884: feature_name=HLA-DQA2	HLA-DQA2.value <= threshold=-0.12580305337905884
node_3885: feature_name=SLC7A5	SLC7A5.value <= threshold=-0.4906871169805527
node_3886: feature_name=VPREB3	VPREB3.value <= threshold=5.492124557495117
node_3887: feature_name=CYBB	CYBB.value <= threshold=2.720046043395996
node_3888: feature_name=CD79A	CD79A.value <= threshold=3.3531476259231567

node_3889: feature_name=HLA-DPA1	HLA-DPA1.value <= threshold=1.6232598423957825
node_3890: feature_name=MT-CYB	MT-CYB.value <= threshold=1.3801624178886414
node_3891: feature_name=HLA-DPB1	HLA-DPB1.value <= threshold=-0.6351701319217682
node_3892: feature_name=TCF4	TCF4.value > threshold=-0.21780844777822495
Class: COVID-19 Severe	
Rules_1223	passed counts:3
node_0: feature_name=LTB	LTB.value <= threshold=0.994031548500061
node_1: feature_name=MTRNR2L2	MTRNR2L2.value <= threshold=2.8784297704696655
node_2: feature_name=TCL1A	TCL1A.value <= threshold=4.798312664031982
node_3: feature_name=MT-CO1	MT-CO1.value > threshold=0.08947191759943962
node_2521: feature_name=LTB	LTB.value <= threshold=0.6038667261600494
node_2522: feature_name=HLA-DQB1	HLA-DQB1.value > threshold=2.1053054332733154
node_3550: feature_name=XIST	XIST.value <= threshold=0.7716337237507105
node_3551: feature_name=CD79B	CD79B.value <= threshold=2.9426796436309814
node_3552: feature_name=HLA-DQB1	HLA-DQB1.value > threshold=2.713674306869507
node_3854: feature_name=MEF2C	MEF2C.value <= threshold=-0.3721291869878769
node_3855: feature_name=HLA-DRB5	HLA-DRB5.value > threshold=0.7929701805114746
node_3859: feature_name=LTB	LTB.value > threshold=-0.9689712226390839
node_3863: feature_name=RP11-731F5.2	RP11-731F5.2.value <= threshold=3.578703761100769
node_3864: feature_name=COTL1	COTL1.value <= threshold=0.38136887550354004
node_3865: feature_name=HLA-DQA1	HLA-DQA1.value > threshold=2.6825207471847534
node_3873: feature_name=MARCH1	MARCH1.value > threshold=1.1291348487138748
node_3875: feature_name=NR4A3	NR4A3.value > threshold=-0.420995369553566
Class: COVID-19 Severe	
Rules_1224	passed counts:3
node_0: feature_name=LTB	LTB.value <= threshold=0.994031548500061
node_1: feature_name=MTRNR2L2	MTRNR2L2.value <= threshold=2.8784297704696655
node_2: feature_name=TCL1A	TCL1A.value <= threshold=4.798312664031982

node_3: feature_name=MT-CO1	MT-CO1.value > threshold=0.08947191759943962
node_2521: feature_name=LTB	LTB.value <= threshold=0.6038667261600494
node_2522: feature_name=HLA-DQB1	HLA-DQB1.value > threshold=2.1053054332733154
node_3550: feature_name=XIST	XIST.value <= threshold=0.7716337237507105
node_3551: feature_name=CD79B	CD79B.value <= threshold=2.9426796436309814
node_3552: feature_name=HLA-DQB1	HLA-DQB1.value > threshold=2.713674306869507
node_3854: feature_name=MEF2C	MEF2C.value <= threshold=-0.3721291869878769
node_3855: feature_name=HLA-DRB5	HLA-DRB5.value > threshold=0.7929701805114746
node_3859: feature_name=LTB	LTB.value > threshold=-0.9689712226390839
node_3863: feature_name=RP11-731F5.2	RP11-731F5.2.value <= threshold=3.578703761100769
node_3864: feature_name=COTL1	COTL1.value <= threshold=0.38136887550354004
node_3865: feature_name=HLA-DQA1	HLA-DQA1.value <= threshold=2.6825207471847534
node_3866: feature_name=FCER2	FCER2.value > threshold=4.623371601104736
node_3870: feature_name=ITGAX	ITGAX.value > threshold=-0.31624606251716614
Class: COVID-19 Critical	
Rules_1225	passed counts:3
node_0: feature_name=LTB	LTB.value <= threshold=0.994031548500061
node_1: feature_name=MTRNR2L2	MTRNR2L2.value <= threshold=2.8784297704696655
node_2: feature_name=TCL1A	TCL1A.value <= threshold=4.798312664031982
node_3: feature_name=MT-CO1	MT-CO1.value > threshold=0.08947191759943962
node_2521: feature_name=LTB	LTB.value <= threshold=0.6038667261600494
node_2522: feature_name=HLA-DQB1	HLA-DQB1.value > threshold=2.1053054332733154
node_3550: feature_name=XIST	XIST.value <= threshold=0.7716337237507105
node_3551: feature_name=CD79B	CD79B.value <= threshold=2.9426796436309814
node_3552: feature_name=HLA-DQB1	HLA-DQB1.value <= threshold=2.713674306869507
node_3553: feature_name=SNX9	SNX9.value > threshold=-0.5404067635536194
node_3653: feature_name=LTB	LTB.value > threshold=0.021708179265260696
node_3759: feature_name=TCL1A	TCL1A.value > threshold=3.3921085596084595
node_3841: feature_name=NFKBIZ	NFKBIZ.value <= threshold=0.3082800954580307

node_3842: feature_name=DAPP1	DAPP1.value > threshold=2.181488871574402
node_3844: feature_name=PARP14	PARP14.value > threshold=1.1000317335128784
Class: COVID-19 Critical	
Rules_1226	passed counts:3
node_0: feature_name=LTB	LTB.value <= threshold=0.994031548500061
node_1: feature_name=MTRNR2L2	MTRNR2L2.value <= threshold=2.8784297704696655
node_2: feature_name=TCL1A	TCL1A.value <= threshold=4.798312664031982
node_3: feature_name=MT-CO1	MT-CO1.value > threshold=0.08947191759943962
node_2521: feature_name=LTB	LTB.value <= threshold=0.6038667261600494
node_2522: feature_name=HLA-DQB1	HLA-DQB1.value > threshold=2.1053054332733154
node_3550: feature_name=XIST	XIST.value <= threshold=0.7716337237507105
node_3551: feature_name=CD79B	CD79B.value <= threshold=2.9426796436309814
node_3552: feature_name=HLA-DQB1	HLA-DQB1.value <= threshold=2.713674306869507
node_3553: feature_name=SNX9	SNX9.value > threshold=-0.5404067635536194
node_3653: feature_name=LTB	LTB.value > threshold=0.021708179265260696
node_3759: feature_name=TCL1A	TCL1A.value <= threshold=3.3921085596084595
node_3760: feature_name=HLA-DRB5	HLA-DRB5.value > threshold=0.5265218019485474
node_3776: feature_name=ID1	ID1.value > threshold=-0.21235637366771698
node_3828: feature_name=RALGPS2	RALGPS2.value <= threshold=2.589961290359497
node_3829: feature_name=RGS2	RGS2.value > threshold=1.3759892582893372
node_3833: feature_name=FERMT3	FERMT3.value <= threshold=-0.6294995248317719
Class: Healthy Control	
Rules_1227	passed counts:3
node_0: feature_name=LTB	LTB.value <= threshold=0.994031548500061
node_1: feature_name=MTRNR2L2	MTRNR2L2.value <= threshold=2.8784297704696655
node_2: feature_name=TCL1A	TCL1A.value <= threshold=4.798312664031982
node_3: feature_name=MT-CO1	MT-CO1.value > threshold=0.08947191759943962
node_2521: feature_name=LTB	LTB.value <= threshold=0.6038667261600494

node_2522: feature_name=HLA-DQB1	HLA-DQB1.value > threshold=2.1053054332733154
node_3550: feature_name=XIST	XIST.value <= threshold=0.7716337237507105
node_3551: feature_name=CD79B	CD79B.value <= threshold=2.9426796436309814
node_3552: feature_name=HLA-DQB1	HLA-DQB1.value <= threshold=2.713674306869507
node_3553: feature_name=SNX9	SNX9.value > threshold=-0.5404067635536194
node_3653: feature_name=LTB	LTB.value > threshold=0.021708179265260696
node_3759: feature_name=TCL1A	TCL1A.value <= threshold=3.3921085596084595
node_3760: feature_name=HLA-DRB5	HLA-DRB5.value > threshold=0.5265218019485474
node_3776: feature_name=ID1	ID1.value <= threshold=-0.21235637366771698
node_3777: feature_name=RP11-731F5.2	RP11-731F5.2.value <= threshold=2.830894112586975
node_3778: feature_name=CASP8	CASP8.value <= threshold=2.222941279411316
node_3779: feature_name=MT-CO3	MT-CO3.value > threshold=-0.41117167472839355
node_3785: feature_name=HLA-DRB5	HLA-DRB5.value > threshold=0.8465962707996368
node_3793: feature_name=PDLIM1	PDLIM1.value <= threshold=2.90118670463562
node_3794: feature_name=WDR74	WDR74.value <= threshold=3.335801362991333
node_3795: feature_name=MEF2C	MEF2C.value <= threshold=4.397150039672852
node_3796: feature_name=HLA-DPA1	HLA-DPA1.value > threshold=-0.10480117797851562
node_3798: feature_name=FCER2	FCER2.value <= threshold=5.688235521316528
node_3799: feature_name=FTH1	FTH1.value <= threshold=1.0842017531394958
node_3800: feature_name=ILK	ILK.value > threshold=2.2077265977859497
node_3802: feature_name=RGCC	RGCC.value > threshold=-0.831504613161087
Class: COVID-19 Critical	
Rules_1228	passed counts:3
node_0: feature_name=LTB	LTB.value <= threshold=0.994031548500061
node_1: feature_name=MTRNR2L2	MTRNR2L2.value <= threshold=2.8784297704696655
node_2: feature_name=TCL1A	TCL1A.value <= threshold=4.798312664031982
node_3: feature_name=MT-CO1	MT-CO1.value > threshold=0.08947191759943962
node_2521: feature_name=LTB	LTB.value <= threshold=0.6038667261600494
node_2522: feature_name=HLA-DQB1	HLA-DQB1.value > threshold=2.1053054332733154

node_3550: feature_name=XIST	XIST.value <= threshold=0.7716337237507105
node_3551: feature_name=CD79B	CD79B.value <= threshold=2.9426796436309814
node_3552: feature_name=HLA-DQB1	HLA-DQB1.value <= threshold=2.713674306869507
node_3553: feature_name=SNX9	SNX9.value > threshold=-0.5404067635536194
node_3653: feature_name=LTB	LTB.value <= threshold=0.021708179265260696
node_3654: feature_name=HLA-DRB5	HLA-DRB5.value > threshold=-0.5633223652839661
node_3660: feature_name=NR4A1	NR4A1.value > threshold=2.2327845096588135
node_3736: feature_name=NFKBID	NFKBID.value > threshold=-0.4191274344921112
node_3738: feature_name=PARP14	PARP14.value > threshold=-0.49970389902591705
node_3752: feature_name=STAG3	STAG3.value > threshold=2.935676693916321
node_3756: feature_name=NFKBIZ	NFKBIZ.value > threshold=-0.21134622395038605
Class: COVID-19 Severe	
Rules_1229	passed counts:3
node_0: feature_name=LTB	LTB.value <= threshold=0.994031548500061
node_1: feature_name=MTRNR2L2	MTRNR2L2.value <= threshold=2.8784297704696655
node_2: feature_name=TCL1A	TCL1A.value <= threshold=4.798312664031982
node_3: feature_name=MT-CO1	MT-CO1.value > threshold=0.08947191759943962
node_2521: feature_name=LTB	LTB.value <= threshold=0.6038667261600494
node_2522: feature_name=HLA-DQB1	HLA-DQB1.value > threshold=2.1053054332733154
node_3550: feature_name=XIST	XIST.value <= threshold=0.7716337237507105
node_3551: feature_name=CD79B	CD79B.value <= threshold=2.9426796436309814
node_3552: feature_name=HLA-DQB1	HLA-DQB1.value <= threshold=2.713674306869507
node_3553: feature_name=SNX9	SNX9.value > threshold=-0.5404067635536194
node_3653: feature_name=LTB	LTB.value <= threshold=0.021708179265260696
node_3654: feature_name=HLA-DRB5	HLA-DRB5.value > threshold=-0.5633223652839661
node_3660: feature_name=NR4A1	NR4A1.value > threshold=2.2327845096588135
node_3736: feature_name=NFKBID	NFKBID.value > threshold=-0.4191274344921112
node_3738: feature_name=PARP14	PARP14.value <= threshold=-0.49970389902591705
node_3739: feature_name=LDLRAD4	LDLRAD4.value > threshold=-0.268046572804451

node_3747: feature_name=TNFAIP3	TNFAIP3.value <= threshold=-1.493474304676056
Class: Healthy Control	
Rules_1230	passed counts:3
node_0: feature_name=LTB	LTB.value <= threshold=0.994031548500061
node_1: feature_name=MTRNR2L2	MTRNR2L2.value <= threshold=2.8784297704696655
node_2: feature_name=TCL1A	TCL1A.value <= threshold=4.798312664031982
node_3: feature_name=MT-CO1	MT-CO1.value > threshold=0.08947191759943962
node_2521: feature_name=LTB	LTB.value <= threshold=0.6038667261600494
node_2522: feature_name=HLA-DQB1	HLA-DQB1.value > threshold=2.1053054332733154
node_3550: feature_name=XIST	XIST.value <= threshold=0.7716337237507105
node_3551: feature_name=CD79B	CD79B.value <= threshold=2.9426796436309814
node_3552: feature_name=HLA-DQB1	HLA-DQB1.value <= threshold=2.713674306869507
node_3553: feature_name=SNX9	SNX9.value > threshold=-0.5404067635536194
node_3653: feature_name=LTB	LTB.value <= threshold=0.021708179265260696
node_3654: feature_name=HLA-DRB5	HLA-DRB5.value > threshold=-0.5633223652839661
node_3660: feature_name=NR4A1	NR4A1.value <= threshold=2.2327845096588135
node_3661: feature_name=FCRL1	FCRL1.value <= threshold=8.662389755249023
node_3662: feature_name=BANK1	BANK1.value <= threshold=4.776419401168823
node_3663: feature_name=IGLL5	IGLL5.value <= threshold=1.4806077480316162
node_3664: feature_name=COTL1	COTL1.value > threshold=-1.2335613369941711
node_3670: feature_name=VCL	VCL.value <= threshold=4.393002271652222
node_3671: feature_name=HLA-DQA1	HLA-DQA1.value > threshold=-0.3948168456554413
node_3673: feature_name=HLA-DRB1	HLA-DRB1.value > threshold=-0.6657836139202118
node_3677: feature_name=GNG11	GNG11.value <= threshold=3.942102313041687
node_3678: feature_name=MT-CO2	MT-CO2.value > threshold=0.10530227422714233
node_3692: feature_name=PLA2G12A	PLA2G12A.value > threshold=1.5832663029432297
node_3698: feature_name=IGJ	IGJ.value > threshold=-0.307477742433548
Class: COVID-19 Critical	

Rules_1231	passed counts:3
node_0: feature_name=LTB	LTB.value <= threshold=0.994031548500061
node_1: feature_name=MTRNR2L2	MTRNR2L2.value <= threshold=2.8784297704696655
node_2: feature_name=TCL1A	TCL1A.value <= threshold=4.798312664031982
node_3: feature_name=MT-CO1	MT-CO1.value > threshold=0.08947191759943962
node_2521: feature_name=LTB	LTB.value <= threshold=0.6038667261600494
node_2522: feature_name=HLA-DQB1	HLA-DQB1.value > threshold=2.1053054332733154
node_3550: feature_name=XIST	XIST.value <= threshold=0.7716337237507105
node_3551: feature_name=CD79B	CD79B.value <= threshold=2.9426796436309814
node_3552: feature_name=HLA-DQB1	HLA-DQB1.value <= threshold=2.713674306869507
node_3553: feature_name=SNX9	SNX9.value > threshold=-0.5404067635536194
node_3653: feature_name=LTB	LTB.value <= threshold=0.021708179265260696
node_3654: feature_name=HLA-DRB5	HLA-DRB5.value > threshold=-0.5633223652839661
node_3660: feature_name=NR4A1	NR4A1.value <= threshold=2.2327845096588135
node_3661: feature_name=FCRL1	FCRL1.value <= threshold=8.662389755249023
node_3662: feature_name=BANK1	BANK1.value <= threshold=4.776419401168823
node_3663: feature_name=IGLL5	IGLL5.value <= threshold=1.4806077480316162
node_3664: feature_name=COTL1	COTL1.value > threshold=-1.2335613369941711
node_3670: feature_name=VCL	VCL.value <= threshold=4.393002271652222
node_3671: feature_name=HLA-DQA1	HLA-DQA1.value > threshold=-0.3948168456554413
node_3673: feature_name=HLA-DRB1	HLA-DRB1.value > threshold=-0.6657836139202118
node_3677: feature_name=GNG11	GNG11.value <= threshold=3.942102313041687
node_3678: feature_name=MT-CO2	MT-CO2.value > threshold=0.10530227422714233
node_3692: feature_name=PLA2G12A	PLA2G12A.value <= threshold=1.5832663029432297
node_3693: feature_name=ABCA1	ABCA1.value > threshold=5.887498140335083
node_3695: feature_name=COTL1	COTL1.value > threshold=-0.6816152930259705
Class: COVID-19 Critical	
Rules_1232	passed counts:3
node_0: feature_name=LTB	LTB.value <= threshold=0.994031548500061

node_1: feature_name=MTRNR2L2	MTRNR2L2.value <= threshold=2.8784297704696655
node_2: feature_name=TCL1A	TCL1A.value <= threshold=4.798312664031982
node_3: feature_name=MT-CO1	MT-CO1.value > threshold=0.08947191759943962
node_2521: feature_name=LTB	LTB.value <= threshold=0.6038667261600494
node_2522: feature_name=HLA-DQB1	HLA-DQB1.value > threshold=2.1053054332733154
node_3550: feature_name=XIST	XIST.value <= threshold=0.7716337237507105
node_3551: feature_name=CD79B	CD79B.value <= threshold=2.9426796436309814
node_3552: feature_name=HLA-DQB1	HLA-DQB1.value <= threshold=2.713674306869507
node_3553: feature_name=SNX9	SNX9.value <= threshold=-0.5404067635536194
node_3554: feature_name=VPREB3	VPREB3.value <= threshold=4.4475414752960205
node_3555: feature_name=RALGPS2	RALGPS2.value > threshold=2.7017585039138794
node_3609: feature_name=MX1	MX1.value <= threshold=1.5204657316207886
node_3610: feature_name=FAM129C	FAM129C.value > threshold=-0.30572274327278137
node_3622: feature_name=CASP8	CASP8.value > threshold=-0.5367972254753113
node_3638: feature_name=SLC11A1	SLC11A1.value > threshold=-0.3386744409799576
node_3640: feature_name=MTRNR2L8	MTRNR2L8.value <= threshold=-0.38738344609737396
node_3641: feature_name=ADAM19	ADAM19.value <= threshold=5.024192571640015
Class: COVID-19 Critical	
Rules_1233	passed counts:3
node_0: feature_name=LTB	LTB.value <= threshold=0.994031548500061
node_1: feature_name=MTRNR2L2	MTRNR2L2.value <= threshold=2.8784297704696655
node_2: feature_name=TCL1A	TCL1A.value <= threshold=4.798312664031982
node_3: feature_name=MT-CO1	MT-CO1.value > threshold=0.08947191759943962
node_2521: feature_name=LTB	LTB.value <= threshold=0.6038667261600494
node_2522: feature_name=HLA-DQB1	HLA-DQB1.value > threshold=2.1053054332733154
node_3550: feature_name=XIST	XIST.value <= threshold=0.7716337237507105
node_3551: feature_name=CD79B	CD79B.value <= threshold=2.9426796436309814
node_3552: feature_name=HLA-DQB1	HLA-DQB1.value <= threshold=2.713674306869507
node_3553: feature_name=SNX9	SNX9.value <= threshold=-0.5404067635536194

node_3554: feature_name=VPREB3	VPREB3.value <= threshold=4.4475414752960205
node_3555: feature_name=RALGPS2	RALGPS2.value <= threshold=2.7017585039138794
node_3556: feature_name=MEF2C	MEF2C.value > threshold=3.997420072555542
node_3604: feature_name=ICAM1	ICAM1.value > threshold=-0.2164856493473053
node_3606: feature_name=SLC7A5	SLC7A5.value > threshold=-0.6240677237510681
Class: COVID-19 Critical	
Rules_1234	passed counts:3
node_0: feature_name=LTB	LTB.value <= threshold=0.994031548500061
node_1: feature_name=MTRNR2L2	MTRNR2L2.value <= threshold=2.8784297704696655
node_2: feature_name=TCL1A	TCL1A.value <= threshold=4.798312664031982
node_3: feature_name=MT-CO1	MT-CO1.value > threshold=0.08947191759943962
node_2521: feature_name=LTB	LTB.value <= threshold=0.6038667261600494
node_2522: feature_name=HLA-DQB1	HLA-DQB1.value > threshold=2.1053054332733154
node_3550: feature_name=XIST	XIST.value <= threshold=0.7716337237507105
node_3551: feature_name=CD79B	CD79B.value <= threshold=2.9426796436309814
node_3552: feature_name=HLA-DQB1	HLA-DQB1.value <= threshold=2.713674306869507
node_3553: feature_name=SNX9	SNX9.value <= threshold=-0.5404067635536194
node_3554: feature_name=VPREB3	VPREB3.value <= threshold=4.4475414752960205
node_3555: feature_name=RALGPS2	RALGPS2.value <= threshold=2.7017585039138794
node_3556: feature_name=MEF2C	MEF2C.value <= threshold=3.997420072555542
node_3557: feature_name=NR4A1	NR4A1.value > threshold=3.9266018867492676
Class: Healthy Control	
Rules_1235	passed counts:3
node_0: feature_name=LTB	LTB.value <= threshold=0.994031548500061
node_1: feature_name=MTRNR2L2	MTRNR2L2.value <= threshold=2.8784297704696655
node_2: feature_name=TCL1A	TCL1A.value <= threshold=4.798312664031982
node_3: feature_name=MT-CO1	MT-CO1.value > threshold=0.08947191759943962
node_2521: feature_name=LTB	LTB.value <= threshold=0.6038667261600494

node_2522: feature_name=HLA-DQB1	HLA-DQB1.value > threshold=2.1053054332733154
node_3550: feature_name=XIST	XIST.value <= threshold=0.7716337237507105
node_3551: feature_name=CD79B	CD79B.value <= threshold=2.9426796436309814
node_3552: feature_name=HLA-DQB1	HLA-DQB1.value <= threshold=2.713674306869507
node_3553: feature_name=SNX9	SNX9.value <= threshold=-0.5404067635536194
node_3554: feature_name=VPREB3	VPREB3.value <= threshold=4.4475414752960205
node_3555: feature_name=RALGPS2	RALGPS2.value <= threshold=2.7017585039138794
node_3556: feature_name=MEF2C	MEF2C.value <= threshold=3.997420072555542
node_3557: feature_name=NR4A1	NR4A1.value <= threshold=3.9266018867492676
node_3558: feature_name=CD22	CD22.value <= threshold=5.738929986953735
node_3559: feature_name=IRF4	IRF4.value > threshold=6.277133464813232
node_3597: feature_name=MT-CYB	MT-CYB.value <= threshold=0.9908932447433472
Class: COVID-19 Severe	
Rules_1236	passed counts:3
node_0: feature_name=LTB	LTB.value <= threshold=0.994031548500061
node_1: feature_name=MTRNR2L2	MTRNR2L2.value <= threshold=2.8784297704696655
node_2: feature_name=TCL1A	TCL1A.value <= threshold=4.798312664031982
node_3: feature_name=MT-CO1	MT-CO1.value > threshold=0.08947191759943962
node_2521: feature_name=LTB	LTB.value <= threshold=0.6038667261600494
node_2522: feature_name=HLA-DQB1	HLA-DQB1.value > threshold=2.1053054332733154
node_3550: feature_name=XIST	XIST.value <= threshold=0.7716337237507105
node_3551: feature_name=CD79B	CD79B.value <= threshold=2.9426796436309814
node_3552: feature_name=HLA-DQB1	HLA-DQB1.value <= threshold=2.713674306869507
node_3553: feature_name=SNX9	SNX9.value <= threshold=-0.5404067635536194
node_3554: feature_name=VPREB3	VPREB3.value <= threshold=4.4475414752960205
node_3555: feature_name=RALGPS2	RALGPS2.value <= threshold=2.7017585039138794
node_3556: feature_name=MEF2C	MEF2C.value <= threshold=3.997420072555542
node_3557: feature_name=NR4A1	NR4A1.value <= threshold=3.9266018867492676
node_3558: feature_name=CD22	CD22.value <= threshold=5.738929986953735

node_3559: feature_name=IRF4	IRF4.value <= threshold=6.277133464813232
node_3560: feature_name=MTRNR2L2	MTRNR2L2.value <= threshold=1.1907066702842712
node_3561: feature_name=BACH1	BACH1.value > threshold=-0.6081153750419617
node_3567: feature_name=DUSP4	DUSP4.value > threshold=-0.27051687240600586
node_3569: feature_name=FAM160B1	FAM160B1.value <= threshold=3.54995596408844
node_3570: feature_name=MT-CO2	MT-CO2.value > threshold=-0.5464511513710022
node_3572: feature_name=TLR2	TLR2.value <= threshold=1.2082695588469505
node_3573: feature_name=SYNE1	SYNE1.value > threshold=1.1966111063957214
node_3591: feature_name=IL8	IL8.value > threshold=-0.28917959332466125
Class: COVID-19 Critical	
Rules_1237	passed counts:3
node_0: feature_name=LTB	LTB.value <= threshold=0.994031548500061
node_1: feature_name=MTRNR2L2	MTRNR2L2.value <= threshold=2.8784297704696655
node_2: feature_name=TCL1A	TCL1A.value <= threshold=4.798312664031982
node_3: feature_name=MT-CO1	MT-CO1.value > threshold=0.08947191759943962
node_2521: feature_name=LTB	LTB.value <= threshold=0.6038667261600494
node_2522: feature_name=HLA-DQB1	HLA-DQB1.value <= threshold=2.1053054332733154
node_2523: feature_name=SNX9	SNX9.value > threshold=-0.5346486568450928
node_2907: feature_name=NR4A1	NR4A1.value > threshold=2.5433837175369263
node_3459: feature_name=VPREB3	VPREB3.value <= threshold=4.410779237747192
node_3460: feature_name=RALGPS2	RALGPS2.value > threshold=3.064661979675293
node_3520: feature_name=RGS2	RGS2.value > threshold=-0.5165578126907349
node_3522: feature_name=MT-ND6	MT-ND6.value <= threshold=1.692318856716156
node_3523: feature_name=ID1	ID1.value <= threshold=-0.16399746388196945
node_3524: feature_name=HBB	HBB.value <= threshold=1.082573562860489
node_3525: feature_name=RBKS	RBKS.value > threshold=-0.2546686381101608
node_3529: feature_name=BOD1L1	BOD1L1.value > threshold=2.1491535305976868
node_3535: feature_name=HLA-DPA1	HLA-DPA1.value <= threshold=2.143317461013794
Class: Healthy Control	

Rules_1238	passed counts:3
node_0: feature_name=LTB	LTB.value <= threshold=0.994031548500061
node_1: feature_name=MTRNR2L2	MTRNR2L2.value <= threshold=2.8784297704696655
node_2: feature_name=TCL1A	TCL1A.value <= threshold=4.798312664031982
node_3: feature_name=MT-CO1	MT-CO1.value > threshold=0.08947191759943962
node_2521: feature_name=LTB	LTB.value <= threshold=0.6038667261600494
node_2522: feature_name=HLA-DQB1	HLA-DQB1.value <= threshold=2.1053054332733154
node_2523: feature_name=SNX9	SNX9.value > threshold=-0.5346486568450928
node_2907: feature_name=NR4A1	NR4A1.value > threshold=2.5433837175369263
node_3459: feature_name=VPREB3	VPREB3.value <= threshold=4.410779237747192
node_3460: feature_name=RALGPS2	RALGPS2.value > threshold=3.064661979675293
node_3520: feature_name=RGS2	RGS2.value > threshold=-0.5165578126907349
node_3522: feature_name=MT-ND6	MT-ND6.value <= threshold=1.692318856716156
node_3523: feature_name=ID1	ID1.value <= threshold=-0.16399746388196945
node_3524: feature_name=HBB	HBB.value <= threshold=1.082573562860489
node_3525: feature_name=RBKS	RBKS.value <= threshold=-0.2546686381101608
node_3526: feature_name=ITGAX	ITGAX.value > threshold=-0.4467625021934509
Class: Healthy Control	
Rules_1239	passed counts:3
node_0: feature_name=LTB	LTB.value <= threshold=0.994031548500061
node_1: feature_name=MTRNR2L2	MTRNR2L2.value <= threshold=2.8784297704696655
node_2: feature_name=TCL1A	TCL1A.value <= threshold=4.798312664031982
node_3: feature_name=MT-CO1	MT-CO1.value > threshold=0.08947191759943962
node_2521: feature_name=LTB	LTB.value <= threshold=0.6038667261600494
node_2522: feature_name=HLA-DQB1	HLA-DQB1.value <= threshold=2.1053054332733154
node_2523: feature_name=SNX9	SNX9.value > threshold=-0.5346486568450928
node_2907: feature_name=NR4A1	NR4A1.value > threshold=2.5433837175369263
node_3459: feature_name=VPREB3	VPREB3.value <= threshold=4.410779237747192

node_3460: feature_name=RALGPS2	RALGPS2.value <= threshold=3.064661979675293
node_3461: feature_name=MT-CO1	MT-CO1.value <= threshold=1.7489010095596313
node_3462: feature_name=HLA-DQA2	HLA-DQA2.value <= threshold=1.794841468334198
node_3463: feature_name=CYBB	CYBB.value > threshold=1.5984815955162048
node_3491: feature_name=PELI1	PELI1.value > threshold=0.13108068704605103
node_3497: feature_name=NFKBID	NFKBID.value > threshold=4.821499824523926
node_3499: feature_name=TNFAIP3	TNFAIP3.value > threshold=0.4055858887732029
Class: COVID-19 Severe	
Rules_1240	passed counts:3
node_0: feature_name=LTB	LTB.value <= threshold=0.994031548500061
node_1: feature_name=MTRNR2L2	MTRNR2L2.value <= threshold=2.8784297704696655
node_2: feature_name=TCL1A	TCL1A.value <= threshold=4.798312664031982
node_3: feature_name=MT-CO1	MT-CO1.value > threshold=0.08947191759943962
node_2521: feature_name=LTB	LTB.value <= threshold=0.6038667261600494
node_2522: feature_name=HLA-DQB1	HLA-DQB1.value <= threshold=2.1053054332733154
node_2523: feature_name=SNX9	SNX9.value > threshold=-0.5346486568450928
node_2907: feature_name=NR4A1	NR4A1.value > threshold=2.5433837175369263
node_3459: feature_name=VPREB3	VPREB3.value <= threshold=4.410779237747192
node_3460: feature_name=RALGPS2	RALGPS2.value <= threshold=3.064661979675293
node_3461: feature_name=MT-CO1	MT-CO1.value <= threshold=1.7489010095596313
node_3462: feature_name=HLA-DQA2	HLA-DQA2.value <= threshold=1.794841468334198
node_3463: feature_name=CYBB	CYBB.value <= threshold=1.5984815955162048
node_3464: feature_name=MARCKS	MARCKS.value <= threshold=-0.38392288982868195
node_3465: feature_name=OAZ1	OAZ1.value <= threshold=-0.07741016428917646
node_3466: feature_name=XAF1	XAF1.value <= threshold=-0.4615721255540848
node_3467: feature_name=BANK1	BANK1.value > threshold=2.4423738718032837
node_3469: feature_name=MS4A1	MS4A1.value > threshold=0.8174735009670258
Class: COVID-19 Severe	

Rules_1241	passed counts:3
node_0: feature_name=LTB	LTB.value <= threshold=0.994031548500061
node_1: feature_name=MTRNR2L2	MTRNR2L2.value <= threshold=2.8784297704696655
node_2: feature_name=TCL1A	TCL1A.value <= threshold=4.798312664031982
node_3: feature_name=MT-CO1	MT-CO1.value > threshold=0.08947191759943962
node_2521: feature_name=LTB	LTB.value <= threshold=0.6038667261600494
node_2522: feature_name=HLA-DQB1	HLA-DQB1.value <= threshold=2.1053054332733154
node_2523: feature_name=SNX9	SNX9.value > threshold=-0.5346486568450928
node_2907: feature_name=NR4A1	NR4A1.value <= threshold=2.5433837175369263
node_2908: feature_name=XIST	XIST.value <= threshold=0.7664470989257097
node_2909: feature_name=CD79B	CD79B.value > threshold=2.9326847791671753
node_3353: feature_name=VPREB3	VPREB3.value <= threshold=3.0226117372512817
node_3354: feature_name=CD83	CD83.value > threshold=1.9074636101722717
node_3388: feature_name=BOD1L1	BOD1L1.value > threshold=-0.4742381125688553
node_3404: feature_name=RALGPS2	RALGPS2.value <= threshold=4.456665992736816
node_3405: feature_name=MEF2A	MEF2A.value > threshold=-0.43542933464050293
node_3409: feature_name=MMD	MMD.value > threshold=3.3664941787719727
node_3415: feature_name=MT-ND6	MT-ND6.value <= threshold=0.9634814560413361
Class: COVID-19 Critical	
Rules_1242	passed counts:3
node_0: feature_name=LTB	LTB.value <= threshold=0.994031548500061
node_1: feature_name=MTRNR2L2	MTRNR2L2.value <= threshold=2.8784297704696655
node_2: feature_name=TCL1A	TCL1A.value <= threshold=4.798312664031982
node_3: feature_name=MT-CO1	MT-CO1.value > threshold=0.08947191759943962
node_2521: feature_name=LTB	LTB.value <= threshold=0.6038667261600494
node_2522: feature_name=HLA-DQB1	HLA-DQB1.value <= threshold=2.1053054332733154
node_2523: feature_name=SNX9	SNX9.value > threshold=-0.5346486568450928
node_2907: feature_name=NR4A1	NR4A1.value <= threshold=2.5433837175369263
node_2908: feature_name=XIST	XIST.value <= threshold=0.7664470989257097

node_2909: feature_name=CD79B	CD79B.value > threshold=2.9326847791671753
node_3353: feature_name=VPREB3	VPREB3.value <= threshold=3.0226117372512817
node_3354: feature_name=CD83	CD83.value > threshold=1.9074636101722717
node_3388: feature_name=BOD1L1	BOD1L1.value > threshold=-0.4742381125688553
node_3404: feature_name=RALGPS2	RALGPS2.value <= threshold=4.456665992736816
node_3405: feature_name=MEF2A	MEF2A.value <= threshold=-0.43542933464050293
node_3406: feature_name=NFKBID	NFKBID.value <= threshold=2.221052408218384
Class: Healthy Control	
Rules_1243	passed counts:3
node_0: feature_name=LTB	LTB.value <= threshold=0.994031548500061
node_1: feature_name=MTRNR2L2	MTRNR2L2.value <= threshold=2.8784297704696655
node_2: feature_name=TCL1A	TCL1A.value <= threshold=4.798312664031982
node_3: feature_name=MT-CO1	MT-CO1.value > threshold=0.08947191759943962
node_2521: feature_name=LTB	LTB.value <= threshold=0.6038667261600494
node_2522: feature_name=HLA-DQB1	HLA-DQB1.value <= threshold=2.1053054332733154
node_2523: feature_name=SNX9	SNX9.value > threshold=-0.5346486568450928
node_2907: feature_name=NR4A1	NR4A1.value <= threshold=2.5433837175369263
node_2908: feature_name=XIST	XIST.value <= threshold=0.7664470989257097
node_2909: feature_name=CD79B	CD79B.value > threshold=2.9326847791671753
node_3353: feature_name=VPREB3	VPREB3.value <= threshold=3.0226117372512817
node_3354: feature_name=CD83	CD83.value > threshold=1.9074636101722717
node_3388: feature_name=BOD1L1	BOD1L1.value <= threshold=-0.4742381125688553
node_3389: feature_name=MT-ND6	MT-ND6.value > threshold=0.3542506694793701
node_3399: feature_name=RALGPS2	RALGPS2.value <= threshold=1.4945479035377502
Class: COVID-19 Severe	
Rules_1244	passed counts:3
node_0: feature_name=LTB	LTB.value <= threshold=0.994031548500061
node_1: feature_name=MTRNR2L2	MTRNR2L2.value <= threshold=2.8784297704696655

node_2: feature_name=TCL1A	TCL1A.value <= threshold=4.798312664031982
node_3: feature_name=MT-CO1	MT-CO1.value > threshold=0.08947191759943962
node_2521: feature_name=LTB	LTB.value <= threshold=0.6038667261600494
node_2522: feature_name=HLA-DQB1	HLA-DQB1.value <= threshold=2.1053054332733154
node_2523: feature_name=SNX9	SNX9.value > threshold=-0.5346486568450928
node_2907: feature_name=NR4A1	NR4A1.value <= threshold=2.5433837175369263
node_2908: feature_name=XIST	XIST.value <= threshold=0.7664470989257097
node_2909: feature_name=CD79B	CD79B.value > threshold=2.9326847791671753
node_3353: feature_name=VPREB3	VPREB3.value <= threshold=3.0226117372512817
node_3354: feature_name=CD83	CD83.value <= threshold=1.9074636101722717
node_3355: feature_name=FERMT3	FERMT3.value > threshold=1.6086163520812988
node_3385: feature_name=RALGPS2	RALGPS2.value > threshold=0.7346877902746201
Class: Healthy Control	
Rules_1245	passed counts:3
node_0: feature_name=LTB	LTB.value <= threshold=0.994031548500061
node_1: feature_name=MTRNR2L2	MTRNR2L2.value <= threshold=2.8784297704696655
node_2: feature_name=TCL1A	TCL1A.value <= threshold=4.798312664031982
node_3: feature_name=MT-CO1	MT-CO1.value > threshold=0.08947191759943962
node_2521: feature_name=LTB	LTB.value <= threshold=0.6038667261600494
node_2522: feature_name=HLA-DQB1	HLA-DQB1.value <= threshold=2.1053054332733154
node_2523: feature_name=SNX9	SNX9.value > threshold=-0.5346486568450928
node_2907: feature_name=NR4A1	NR4A1.value <= threshold=2.5433837175369263
node_2908: feature_name=XIST	XIST.value <= threshold=0.7664470989257097
node_2909: feature_name=CD79B	CD79B.value > threshold=2.9326847791671753
node_3353: feature_name=VPREB3	VPREB3.value <= threshold=3.0226117372512817
node_3354: feature_name=CD83	CD83.value <= threshold=1.9074636101722717
node_3355: feature_name=FERMT3	FERMT3.value <= threshold=1.6086163520812988
node_3356: feature_name=IGJ	IGJ.value <= threshold=4.493098735809326
node_3357: feature_name=HLA-DQA1	HLA-DQA1.value > threshold=3.502458691596985

node_3381: feature_name=IGJ	IGJ.value > threshold=-0.29092344641685486
Class: Healthy Control	
Rules_1246	passed counts:3
node_0: feature_name=LTB	LTB.value <= threshold=0.994031548500061
node_1: feature_name=MTRNR2L2	MTRNR2L2.value <= threshold=2.8784297704696655
node_2: feature_name=TCL1A	TCL1A.value <= threshold=4.798312664031982
node_3: feature_name=MT-CO1	MT-CO1.value > threshold=0.08947191759943962
node_2521: feature_name=LTB	LTB.value <= threshold=0.6038667261600494
node_2522: feature_name=HLA-DQB1	HLA-DQB1.value <= threshold=2.1053054332733154
node_2523: feature_name=SNX9	SNX9.value > threshold=-0.5346486568450928
node_2907: feature_name=NR4A1	NR4A1.value <= threshold=2.5433837175369263
node_2908: feature_name=XIST	XIST.value <= threshold=0.7664470989257097
node_2909: feature_name=CD79B	CD79B.value > threshold=2.9326847791671753
node_3353: feature_name=VPREB3	VPREB3.value <= threshold=3.0226117372512817
node_3354: feature_name=CD83	CD83.value <= threshold=1.9074636101722717
node_3355: feature_name=FERMT3	FERMT3.value <= threshold=1.6086163520812988
node_3356: feature_name=IGJ	IGJ.value <= threshold=4.493098735809326
node_3357: feature_name=HLA-DQA1	HLA-DQA1.value <= threshold=3.502458691596985
node_3358: feature_name=ID1	ID1.value <= threshold=1.2194700837135315
node_3359: feature_name=OAZ1	OAZ1.value <= threshold=1.6739212274551392
node_3360: feature_name=HLA-DPB1	HLA-DPB1.value <= threshold=2.48960018157959
node_3361: feature_name=TPM1	TPM1.value <= threshold=3.764936327934265
node_3362: feature_name=DUSP4	DUSP4.value <= threshold=3.401969313621521
node_3363: feature_name=PDLIM1	PDLIM1.value > threshold=4.827272415161133
node_3365: feature_name=IGLL5	IGLL5.value <= threshold=0.9029710590839386
Class: COVID-19 Critical	
Rules_1247	passed counts:3
node_0: feature_name=LTB	LTB.value <= threshold=0.994031548500061

node_1: feature_name=MTRNR2L2	MTRNR2L2.value <= threshold=2.8784297704696655
node_2: feature_name=TCL1A	TCL1A.value <= threshold=4.798312664031982
node_3: feature_name=MT-CO1	MT-CO1.value > threshold=0.08947191759943962
node_2521: feature_name=LTB	LTB.value <= threshold=0.6038667261600494
node_2522: feature_name=HLA-DQB1	HLA-DQB1.value <= threshold=2.1053054332733154
node_2523: feature_name=SNX9	SNX9.value > threshold=-0.5346486568450928
node_2907: feature_name=NR4A1	NR4A1.value <= threshold=2.5433837175369263
node_2908: feature_name=XIST	XIST.value <= threshold=0.7664470989257097
node_2909: feature_name=CD79B	CD79B.value <= threshold=2.9326847791671753
node_2910: feature_name=NR4A1	NR4A1.value > threshold=-0.2875557690858841
node_3096: feature_name=NR4A3	NR4A3.value > threshold=3.314968705177307
node_3328: feature_name=MT-CO3	MT-CO3.value > threshold=0.35808728635311127
node_3332: feature_name=STAG3	STAG3.value <= threshold=-0.18329568207263947
node_3333: feature_name=SLC7A5	SLC7A5.value > threshold=2.876398801803589
node_3345: feature_name=ATF3	ATF3.value > threshold=-0.1988486647605896
node_3349: feature_name=CD79B	CD79B.value <= threshold=-0.24604392796754837
Class: Healthy Control	
Rules_1248	passed counts:3
node_0: feature_name=LTB	LTB.value <= threshold=0.994031548500061
node_1: feature_name=MTRNR2L2	MTRNR2L2.value <= threshold=2.8784297704696655
node_2: feature_name=TCL1A	TCL1A.value <= threshold=4.798312664031982
node_3: feature_name=MT-CO1	MT-CO1.value > threshold=0.08947191759943962
node_2521: feature_name=LTB	LTB.value <= threshold=0.6038667261600494
node_2522: feature_name=HLA-DQB1	HLA-DQB1.value <= threshold=2.1053054332733154
node_2523: feature_name=SNX9	SNX9.value > threshold=-0.5346486568450928
node_2907: feature_name=NR4A1	NR4A1.value <= threshold=2.5433837175369263
node_2908: feature_name=XIST	XIST.value <= threshold=0.7664470989257097
node_2909: feature_name=CD79B	CD79B.value <= threshold=2.9326847791671753
node_2910: feature_name=NR4A1	NR4A1.value > threshold=-0.2875557690858841

node_3096: feature_name=NR4A3	NR4A3.value > threshold=3.314968705177307
node_3328: feature_name=MT-CO3	MT-CO3.value > threshold=0.35808728635311127
node_3332: feature_name=STAG3	STAG3.value <= threshold=-0.18329568207263947
node_3333: feature_name=SLC7A5	SLC7A5.value <= threshold=2.876398801803589
node_3334: feature_name=SNX29P2	SNX29P2.value > threshold=5.85232138633728
Class: Healthy Control	
Rules_1249	passed counts:3
node_0: feature_name=LTB	LTB.value <= threshold=0.994031548500061
node_1: feature_name=MTRNR2L2	MTRNR2L2.value <= threshold=2.8784297704696655
node_2: feature_name=TCL1A	TCL1A.value <= threshold=4.798312664031982
node_3: feature_name=MT-CO1	MT-CO1.value > threshold=0.08947191759943962
node_2521: feature_name=LTB	LTB.value <= threshold=0.6038667261600494
node_2522: feature_name=HLA-DQB1	HLA-DQB1.value <= threshold=2.1053054332733154
node_2523: feature_name=SNX9	SNX9.value > threshold=-0.5346486568450928
node_2907: feature_name=NR4A1	NR4A1.value <= threshold=2.5433837175369263
node_2908: feature_name=XIST	XIST.value <= threshold=0.7664470989257097
node_2909: feature_name=CD79B	CD79B.value <= threshold=2.9326847791671753
node_2910: feature_name=NR4A1	NR4A1.value > threshold=-0.2875557690858841
node_3096: feature_name=NR4A3	NR4A3.value > threshold=3.314968705177307
node_3328: feature_name=MT-CO3	MT-CO3.value > threshold=0.35808728635311127
node_3332: feature_name=STAG3	STAG3.value <= threshold=-0.18329568207263947
node_3333: feature_name=SLC7A5	SLC7A5.value <= threshold=2.876398801803589
node_3334: feature_name=SNX29P2	SNX29P2.value <= threshold=5.85232138633728
node_3335: feature_name=GPX1	GPX1.value > threshold=0.11650833487510681
node_3337: feature_name=ADAM19	ADAM19.value > threshold=-0.3311994820833206
Class: Healthy Control	
Rules_1250	passed counts:3
node_0: feature_name=LTB	LTB.value <= threshold=0.994031548500061

node_1: feature_name=MTRNR2L2	MTRNR2L2.value <= threshold=2.8784297704696655
node_2: feature_name=TCL1A	TCL1A.value <= threshold=4.798312664031982
node_3: feature_name=MT-CO1	MT-CO1.value > threshold=0.08947191759943962
node_2521: feature_name=LTB	LTB.value <= threshold=0.6038667261600494
node_2522: feature_name=HLA-DQB1	HLA-DQB1.value <= threshold=2.1053054332733154
node_2523: feature_name=SNX9	SNX9.value > threshold=-0.5346486568450928
node_2907: feature_name=NR4A1	NR4A1.value <= threshold=2.5433837175369263
node_2908: feature_name=XIST	XIST.value <= threshold=0.7664470989257097
node_2909: feature_name=CD79B	CD79B.value <= threshold=2.9326847791671753
node_2910: feature_name=NR4A1	NR4A1.value > threshold=-0.2875557690858841
node_3096: feature_name=NR4A3	NR4A3.value <= threshold=3.314968705177307
node_3097: feature_name=VPREB3	VPREB3.value > threshold=3.3549938201904297
node_3285: feature_name=OAZ1	OAZ1.value > threshold=0.11267071589827538
node_3309: feature_name=SNX9	SNX9.value <= threshold=1.7356606721878052
node_3310: feature_name=RALGPS2	RALGPS2.value <= threshold=2.1317856311798096
node_3311: feature_name=DAPP1	DAPP1.value > threshold=-0.3117790222167969
Class: COVID-19 Critical	
Rules_1251	passed counts:3
node_0: feature_name=LTB	LTB.value <= threshold=0.994031548500061
node_1: feature_name=MTRNR2L2	MTRNR2L2.value <= threshold=2.8784297704696655
node_2: feature_name=TCL1A	TCL1A.value <= threshold=4.798312664031982
node_3: feature_name=MT-CO1	MT-CO1.value > threshold=0.08947191759943962
node_2521: feature_name=LTB	LTB.value <= threshold=0.6038667261600494
node_2522: feature_name=HLA-DQB1	HLA-DQB1.value <= threshold=2.1053054332733154
node_2523: feature_name=SNX9	SNX9.value > threshold=-0.5346486568450928
node_2907: feature_name=NR4A1	NR4A1.value <= threshold=2.5433837175369263
node_2908: feature_name=XIST	XIST.value <= threshold=0.7664470989257097
node_2909: feature_name=CD79B	CD79B.value <= threshold=2.9326847791671753
node_2910: feature_name=NR4A1	NR4A1.value > threshold=-0.2875557690858841

node_3096: feature_name=NR4A3	NR4A3.value <= threshold=3.314968705177307
node_3097: feature_name=VPREB3	VPREB3.value > threshold=3.3549938201904297
node_3285: feature_name=OAZ1	OAZ1.value <= threshold=0.11267071589827538
node_3286: feature_name=STMN1	STMN1.value > threshold=2.106989860534668
Class: COVID-19 Severe	
Rules_1252	passed counts:3
node_0: feature_name=LTB	LTB.value <= threshold=0.994031548500061
node_1: feature_name=MTRNR2L2	MTRNR2L2.value <= threshold=2.8784297704696655
node_2: feature_name=TCL1A	TCL1A.value <= threshold=4.798312664031982
node_3: feature_name=MT-CO1	MT-CO1.value > threshold=0.08947191759943962
node_2521: feature_name=LTB	LTB.value <= threshold=0.6038667261600494
node_2522: feature_name=HLA-DQB1	HLA-DQB1.value <= threshold=2.1053054332733154
node_2523: feature_name=SNX9	SNX9.value > threshold=-0.5346486568450928
node_2907: feature_name=NR4A1	NR4A1.value <= threshold=2.5433837175369263
node_2908: feature_name=XIST	XIST.value <= threshold=0.7664470989257097
node_2909: feature_name=CD79B	CD79B.value <= threshold=2.9326847791671753
node_2910: feature_name=NR4A1	NR4A1.value > threshold=-0.2875557690858841
node_3096: feature_name=NR4A3	NR4A3.value <= threshold=3.314968705177307
node_3097: feature_name=VPREB3	VPREB3.value > threshold=3.3549938201904297
node_3285: feature_name=OAZ1	OAZ1.value <= threshold=0.11267071589827538
node_3286: feature_name=STMN1	STMN1.value <= threshold=2.106989860534668
node_3287: feature_name=PLD4	PLD4.value <= threshold=4.0678054094314575
node_3288: feature_name=APP	APP.value > threshold=2.4729559421539307
node_3302: feature_name=AHR	AHR.value <= threshold=-0.4360882639884949
Class: Healthy Control	
Rules_1253	passed counts:3
node_0: feature_name=LTB	LTB.value <= threshold=0.994031548500061
node_1: feature_name=MTRNR2L2	MTRNR2L2.value <= threshold=2.8784297704696655

node_2: feature_name=TCL1A	TCL1A.value <= threshold=4.798312664031982
node_3: feature_name=MT-CO1	MT-CO1.value > threshold=0.08947191759943962
node_2521: feature_name=LTB	LTB.value <= threshold=0.6038667261600494
node_2522: feature_name=HLA-DQB1	HLA-DQB1.value <= threshold=2.1053054332733154
node_2523: feature_name=SNX9	SNX9.value > threshold=-0.5346486568450928
node_2907: feature_name=NR4A1	NR4A1.value <= threshold=2.5433837175369263
node_2908: feature_name=XIST	XIST.value <= threshold=0.7664470989257097
node_2909: feature_name=CD79B	CD79B.value <= threshold=2.9326847791671753
node_2910: feature_name=NR4A1	NR4A1.value > threshold=-0.2875557690858841
node_3096: feature_name=NR4A3	NR4A3.value <= threshold=3.314968705177307
node_3097: feature_name=VPREB3	VPREB3.value <= threshold=3.3549938201904297
node_3098: feature_name=RALGPS2	RALGPS2.value <= threshold=5.807050704956055
node_3099: feature_name=CTD-2035E11.3	CTD-2035E11.3.value <= threshold=6.4090416431427
node_3100: feature_name=FAM129C	FAM129C.value <= threshold=6.966361045837402
node_3101: feature_name=HES4	HES4.value > threshold=4.139557242393494
node_3269: feature_name=MCTP1	MCTP1.value <= threshold=-0.11753645166754723
Class: COVID-19 Severe	
Rules_1254	passed counts:3
node_0: feature_name=LTB	LTB.value <= threshold=0.994031548500061
node_1: feature_name=MTRNR2L2	MTRNR2L2.value <= threshold=2.8784297704696655
node_2: feature_name=TCL1A	TCL1A.value <= threshold=4.798312664031982
node_3: feature_name=MT-CO1	MT-CO1.value > threshold=0.08947191759943962
node_2521: feature_name=LTB	LTB.value <= threshold=0.6038667261600494
node_2522: feature_name=HLA-DQB1	HLA-DQB1.value <= threshold=2.1053054332733154
node_2523: feature_name=SNX9	SNX9.value > threshold=-0.5346486568450928
node_2907: feature_name=NR4A1	NR4A1.value <= threshold=2.5433837175369263
node_2908: feature_name=XIST	XIST.value <= threshold=0.7664470989257097
node_2909: feature_name=CD79B	CD79B.value <= threshold=2.9326847791671753
node_2910: feature_name=NR4A1	NR4A1.value > threshold=-0.2875557690858841

node_3096: feature_name=NR4A3	NR4A3.value <= threshold=3.314968705177307
node_3097: feature_name=VPREB3	VPREB3.value <= threshold=3.3549938201904297
node_3098: feature_name=RALGPS2	RALGPS2.value <= threshold=5.807050704956055
node_3099: feature_name=CTD-2035E11.3	CTD-2035E11.3.value <= threshold=6.4090416431427
node_3100: feature_name=FAM129C	FAM129C.value <= threshold=6.966361045837402
node_3101: feature_name=HES4	HES4.value <= threshold=4.139557242393494
node_3102: feature_name=CD83	CD83.value > threshold=1.8343153595924377
node_3136: feature_name=PLD4	PLD4.value <= threshold=4.453821897506714
node_3137: feature_name=HLA-DQA2	HLA-DQA2.value > threshold=-0.10758286714553833
node_3229: feature_name=FAM160B1	FAM160B1.value > threshold=1.61697119474411
node_3261: feature_name=TNFAIP3	TNFAIP3.value <= threshold=-1.4364122152328491
Class: COVID-19 Critical	
Rules_1255	passed counts:3
node_0: feature_name=LTB	LTB.value <= threshold=0.994031548500061
node_1: feature_name=MTRNR2L2	MTRNR2L2.value <= threshold=2.8784297704696655
node_2: feature_name=TCL1A	TCL1A.value <= threshold=4.798312664031982
node_3: feature_name=MT-CO1	MT-CO1.value > threshold=0.08947191759943962
node_2521: feature_name=LTB	LTB.value <= threshold=0.6038667261600494
node_2522: feature_name=HLA-DQB1	HLA-DQB1.value <= threshold=2.1053054332733154
node_2523: feature_name=SNX9	SNX9.value > threshold=-0.5346486568450928
node_2907: feature_name=NR4A1	NR4A1.value <= threshold=2.5433837175369263
node_2908: feature_name=XIST	XIST.value <= threshold=0.7664470989257097
node_2909: feature_name=CD79B	CD79B.value <= threshold=2.9326847791671753
node_2910: feature_name=NR4A1	NR4A1.value > threshold=-0.2875557690858841
node_3096: feature_name=NR4A3	NR4A3.value <= threshold=3.314968705177307
node_3097: feature_name=VPREB3	VPREB3.value <= threshold=3.3549938201904297
node_3098: feature_name=RALGPS2	RALGPS2.value <= threshold=5.807050704956055
node_3099: feature_name=CTD-2035E11.3	CTD-2035E11.3.value <= threshold=6.4090416431427
node_3100: feature_name=FAM129C	FAM129C.value <= threshold=6.966361045837402

node_3101: feature_name=HES4	HES4.value <= threshold=4.139557242393494
node_3102: feature_name=CD83	CD83.value > threshold=1.8343153595924377
node_3136: feature_name=PLD4	PLD4.value <= threshold=4.453821897506714
node_3137: feature_name=HLA-DQA2	HLA-DQA2.value > threshold=-0.10758286714553833
node_3229: feature_name=FAM160B1	FAM160B1.value <= threshold=1.61697119474411
node_3230: feature_name=BCL2A1	BCL2A1.value > threshold=0.9140156805515289
node_3250: feature_name=RNF11	RNF11.value <= threshold=-0.5307398736476898
node_3251: feature_name=RP11-731F5.2	RP11-731F5.2.value > threshold=1.3988762274384499
node_3253: feature_name=GPX1	GPX1.value <= threshold=-0.8558085560798645
Class: COVID-19 Critical	
Rules_1256	passed counts:3
node_0: feature_name=LTB	LTB.value <= threshold=0.994031548500061
node_1: feature_name=MTRNR2L2	MTRNR2L2.value <= threshold=2.8784297704696655
node_2: feature_name=TCL1A	TCL1A.value <= threshold=4.798312664031982
node_3: feature_name=MT-CO1	MT-CO1.value > threshold=0.08947191759943962
node_2521: feature_name=LTB	LTB.value <= threshold=0.6038667261600494
node_2522: feature_name=HLA-DQB1	HLA-DQB1.value <= threshold=2.1053054332733154
node_2523: feature_name=SNX9	SNX9.value > threshold=-0.5346486568450928
node_2907: feature_name=NR4A1	NR4A1.value <= threshold=2.5433837175369263
node_2908: feature_name=XIST	XIST.value <= threshold=0.7664470989257097
node_2909: feature_name=CD79B	CD79B.value <= threshold=2.9326847791671753
node_2910: feature_name=NR4A1	NR4A1.value > threshold=-0.2875557690858841
node_3096: feature_name=NR4A3	NR4A3.value <= threshold=3.314968705177307
node_3097: feature_name=VPREB3	VPREB3.value <= threshold=3.3549938201904297
node_3098: feature_name=RALGPS2	RALGPS2.value <= threshold=5.807050704956055
node_3099: feature_name=CTD-2035E11.3	CTD-2035E11.3.value <= threshold=6.4090416431427
node_3100: feature_name=FAM129C	FAM129C.value <= threshold=6.966361045837402
node_3101: feature_name=HES4	HES4.value <= threshold=4.139557242393494
node_3102: feature_name=CD83	CD83.value > threshold=1.8343153595924377

node_3136: feature_name=PLD4	PLD4.value <= threshold=4.453821897506714
node_3137: feature_name=HLA-DQA2	HLA-DQA2.value <= threshold=-0.10758286714553833
node_3138: feature_name=STAG3	STAG3.value > threshold=7.365770578384399
node_3226: feature_name=PPIF	PPIF.value <= threshold=-0.17593920975923538
Class: COVID-19 Severe	
Rules_1257	passed counts:3
node_0: feature_name=LTB	LTB.value <= threshold=0.994031548500061
node_1: feature_name=MTRNR2L2	MTRNR2L2.value <= threshold=2.8784297704696655
node_2: feature_name=TCL1A	TCL1A.value <= threshold=4.798312664031982
node_3: feature_name=MT-CO1	MT-CO1.value > threshold=0.08947191759943962
node_2521: feature_name=LTB	LTB.value <= threshold=0.6038667261600494
node_2522: feature_name=HLA-DQB1	HLA-DQB1.value <= threshold=2.1053054332733154
node_2523: feature_name=SNX9	SNX9.value > threshold=-0.5346486568450928
node_2907: feature_name=NR4A1	NR4A1.value <= threshold=2.5433837175369263
node_2908: feature_name=XIST	XIST.value <= threshold=0.7664470989257097
node_2909: feature_name=CD79B	CD79B.value <= threshold=2.9326847791671753
node_2910: feature_name=NR4A1	NR4A1.value > threshold=-0.2875557690858841
node_3096: feature_name=NR4A3	NR4A3.value <= threshold=3.314968705177307
node_3097: feature_name=VPREB3	VPREB3.value <= threshold=3.3549938201904297
node_3098: feature_name=RALGPS2	RALGPS2.value <= threshold=5.807050704956055
node_3099: feature_name=CTD-2035E11.3	CTD-2035E11.3.value <= threshold=6.4090416431427
node_3100: feature_name=FAM129C	FAM129C.value <= threshold=6.966361045837402
node_3101: feature_name=HES4	HES4.value <= threshold=4.139557242393494
node_3102: feature_name=CD83	CD83.value > threshold=1.8343153595924377
node_3136: feature_name=PLD4	PLD4.value <= threshold=4.453821897506714
node_3137: feature_name=HLA-DQA2	HLA-DQA2.value <= threshold=-0.10758286714553833
node_3138: feature_name=STAG3	STAG3.value <= threshold=7.365770578384399
node_3139: feature_name=LDLR	LDLR.value <= threshold=2.3423796892166138
node_3140: feature_name=CD79A	CD79A.value <= threshold=4.038331866264343

node_3141: feature_name=DUSP4	DUSP4.value <= threshold=4.829863548278809
node_3142: feature_name=OAZ1	OAZ1.value > threshold=-0.34704728424549103
node_3178: feature_name=MX1	MX1.value > threshold=-0.48719069361686707
node_3184: feature_name=HLA-DPB1	HLA-DPB1.value > threshold=2.151633381843567
node_3210: feature_name=TSPYL2	TSPYL2.value > threshold=-0.8144445717334747
Class: Healthy Control	
Rules_1258	passed counts:3
node_0: feature_name=LTB	LTB.value <= threshold=0.994031548500061
node_1: feature_name=MTRNR2L2	MTRNR2L2.value <= threshold=2.8784297704696655
node_2: feature_name=TCL1A	TCL1A.value <= threshold=4.798312664031982
node_3: feature_name=MT-CO1	MT-CO1.value > threshold=0.08947191759943962
node_2521: feature_name=LTB	LTB.value <= threshold=0.6038667261600494
node_2522: feature_name=HLA-DQB1	HLA-DQB1.value <= threshold=2.1053054332733154
node_2523: feature_name=SNX9	SNX9.value > threshold=-0.5346486568450928
node_2907: feature_name=NR4A1	NR4A1.value <= threshold=2.5433837175369263
node_2908: feature_name=XIST	XIST.value <= threshold=0.7664470989257097
node_2909: feature_name=CD79B	CD79B.value <= threshold=2.9326847791671753
node_2910: feature_name=NR4A1	NR4A1.value > threshold=-0.2875557690858841
node_3096: feature_name=NR4A3	NR4A3.value <= threshold=3.314968705177307
node_3097: feature_name=VPREB3	VPREB3.value <= threshold=3.3549938201904297
node_3098: feature_name=RALGPS2	RALGPS2.value <= threshold=5.807050704956055
node_3099: feature_name=CTD-2035E11.3	CTD-2035E11.3.value <= threshold=6.4090416431427
node_3100: feature_name=FAM129C	FAM129C.value <= threshold=6.966361045837402
node_3101: feature_name=HES4	HES4.value <= threshold=4.139557242393494
node_3102: feature_name=CD83	CD83.value > threshold=1.8343153595924377
node_3136: feature_name=PLD4	PLD4.value <= threshold=4.453821897506714
node_3137: feature_name=HLA-DQA2	HLA-DQA2.value <= threshold=-0.10758286714553833
node_3138: feature_name=STAG3	STAG3.value <= threshold=7.365770578384399
node_3139: feature_name=LDLR	LDLR.value <= threshold=2.3423796892166138

node_3140: feature_name=CD79A	CD79A.value <= threshold=4.038331866264343
node_3141: feature_name=DUSP4	DUSP4.value <= threshold=4.829863548278809
node_3142: feature_name=OAZ1	OAZ1.value > threshold=-0.34704728424549103
node_3178: feature_name=MX1	MX1.value > threshold=-0.48719069361686707
node_3184: feature_name=HLA-DPB1	HLA-DPB1.value <= threshold=2.151633381843567
node_3185: feature_name=RGS2	RGS2.value <= threshold=1.594672977924347
node_3186: feature_name=RELT	RELT.value > threshold=2.0173692107200623
node_3198: feature_name=SNX9	SNX9.value > threshold=2.805444598197937
Class: COVID-19 Critical	
Rules_1259	passed counts:3
node_0: feature_name=LTB	LTB.value <= threshold=0.994031548500061
node_1: feature_name=MTRNR2L2	MTRNR2L2.value <= threshold=2.8784297704696655
node_2: feature_name=TCL1A	TCL1A.value <= threshold=4.798312664031982
node_3: feature_name=MT-CO1	MT-CO1.value > threshold=0.08947191759943962
node_2521: feature_name=LTB	LTB.value <= threshold=0.6038667261600494
node_2522: feature_name=HLA-DQB1	HLA-DQB1.value <= threshold=2.1053054332733154
node_2523: feature_name=SNX9	SNX9.value > threshold=-0.5346486568450928
node_2907: feature_name=NR4A1	NR4A1.value <= threshold=2.5433837175369263
node_2908: feature_name=XIST	XIST.value <= threshold=0.7664470989257097
node_2909: feature_name=CD79B	CD79B.value <= threshold=2.9326847791671753
node_2910: feature_name=NR4A1	NR4A1.value > threshold=-0.2875557690858841
node_3096: feature_name=NR4A3	NR4A3.value <= threshold=3.314968705177307
node_3097: feature_name=VPREB3	VPREB3.value <= threshold=3.3549938201904297
node_3098: feature_name=RALGPS2	RALGPS2.value <= threshold=5.807050704956055
node_3099: feature_name=CTD-2035E11.3	CTD-2035E11.3.value <= threshold=6.4090416431427
node_3100: feature_name=FAM129C	FAM129C.value <= threshold=6.966361045837402
node_3101: feature_name=HES4	HES4.value <= threshold=4.139557242393494
node_3102: feature_name=CD83	CD83.value > threshold=1.8343153595924377
node_3136: feature_name=PLD4	PLD4.value <= threshold=4.453821897506714

node_3137: feature_name=HLA-DQA2	HLA-DQA2.value <= threshold=-0.10758286714553833
node_3138: feature_name=STAG3	STAG3.value <= threshold=7.365770578384399
node_3139: feature_name=LDLR	LDLR.value <= threshold=2.3423796892166138
node_3140: feature_name=CD79A	CD79A.value <= threshold=4.038331866264343
node_3141: feature_name=DUSP4	DUSP4.value <= threshold=4.829863548278809
node_3142: feature_name=OAZ1	OAZ1.value > threshold=-0.34704728424549103
node_3178: feature_name=MX1	MX1.value <= threshold=-0.48719069361686707
node_3179: feature_name=HSP90B1	HSP90B1.value <= threshold=0.9721381366252899
Class: Healthy Control	
Rules_1260	passed counts:3
node_0: feature_name=LTB	LTB.value <= threshold=0.994031548500061
node_1: feature_name=MTRNR2L2	MTRNR2L2.value <= threshold=2.8784297704696655
node_2: feature_name=TCL1A	TCL1A.value <= threshold=4.798312664031982
node_3: feature_name=MT-CO1	MT-CO1.value > threshold=0.08947191759943962
node_2521: feature_name=LTB	LTB.value <= threshold=0.6038667261600494
node_2522: feature_name=HLA-DQB1	HLA-DQB1.value <= threshold=2.1053054332733154
node_2523: feature_name=SNX9	SNX9.value > threshold=-0.5346486568450928
node_2907: feature_name=NR4A1	NR4A1.value <= threshold=2.5433837175369263
node_2908: feature_name=XIST	XIST.value <= threshold=0.7664470989257097
node_2909: feature_name=CD79B	CD79B.value <= threshold=2.9326847791671753
node_2910: feature_name=NR4A1	NR4A1.value > threshold=-0.2875557690858841
node_3096: feature_name=NR4A3	NR4A3.value <= threshold=3.314968705177307
node_3097: feature_name=VPREB3	VPREB3.value <= threshold=3.3549938201904297
node_3098: feature_name=RALGPS2	RALGPS2.value <= threshold=5.807050704956055
node_3099: feature_name=CTD-2035E11.3	CTD-2035E11.3.value <= threshold=6.4090416431427
node_3100: feature_name=FAM129C	FAM129C.value <= threshold=6.966361045837402
node_3101: feature_name=HES4	HES4.value <= threshold=4.139557242393494
node_3102: feature_name=CD83	CD83.value <= threshold=1.8343153595924377
node_3103: feature_name=FCER2	FCER2.value <= threshold=7.255846738815308

node_3104: feature_name=MT-CYB	MT-CYB.value <= threshold=2.3888577222824097
node_3105: feature_name=RGCC	RGCC.value > threshold=-1.2040565013885498
node_3107: feature_name=MCTP1	MCTP1.value <= threshold=6.221277952194214
node_3108: feature_name=JUN	JUN.value > threshold=-0.629857987165451
node_3120: feature_name=RGS2	RGS2.value > threshold=-0.9065633714199066
node_3124: feature_name=MT1E	MT1E.value <= threshold=0.8591758906841278
node_3125: feature_name=SPI1	SPI1.value > threshold=1.2638847827911377
node_3127: feature_name=HOPX	HOPX.value <= threshold=-0.4769510328769684
Class: COVID-19 Critical	
Rules_1261	passed counts:3
node_0: feature_name=LTB	LTB.value <= threshold=0.994031548500061
node_1: feature_name=MTRNR2L2	MTRNR2L2.value <= threshold=2.8784297704696655
node_2: feature_name=TCL1A	TCL1A.value <= threshold=4.798312664031982
node_3: feature_name=MT-CO1	MT-CO1.value > threshold=0.08947191759943962
node_2521: feature_name=LTB	LTB.value <= threshold=0.6038667261600494
node_2522: feature_name=HLA-DQB1	HLA-DQB1.value <= threshold=2.1053054332733154
node_2523: feature_name=SNX9	SNX9.value > threshold=-0.5346486568450928
node_2907: feature_name=NR4A1	NR4A1.value <= threshold=2.5433837175369263
node_2908: feature_name=XIST	XIST.value <= threshold=0.7664470989257097
node_2909: feature_name=CD79B	CD79B.value <= threshold=2.9326847791671753
node_2910: feature_name=NR4A1	NR4A1.value <= threshold=-0.2875557690858841
node_2911: feature_name=CTD-2035E11.3	CTD-2035E11.3.value > threshold=5.262048006057739
Class: COVID-19 Severe	
Rules_1262	passed counts:3
node_0: feature_name=LTB	LTB.value <= threshold=0.994031548500061
node_1: feature_name=MTRNR2L2	MTRNR2L2.value <= threshold=2.8784297704696655
node_2: feature_name=TCL1A	TCL1A.value <= threshold=4.798312664031982
node_3: feature_name=MT-CO1	MT-CO1.value > threshold=0.08947191759943962

node_2521: feature_name=LTB	LTB.value <= threshold=0.6038667261600494
node_2522: feature_name=HLA-DQB1	HLA-DQB1.value <= threshold=2.1053054332733154
node_2523: feature_name=SNX9	SNX9.value > threshold=-0.5346486568450928
node_2907: feature_name=NR4A1	NR4A1.value <= threshold=2.5433837175369263
node_2908: feature_name=XIST	XIST.value <= threshold=0.7664470989257097
node_2909: feature_name=CD79B	CD79B.value <= threshold=2.9326847791671753
node_2910: feature_name=NR4A1	NR4A1.value <= threshold=-0.2875557690858841
node_2911: feature_name=CTD-2035E11.3	CTD-2035E11.3.value <= threshold=5.262048006057739
node_2912: feature_name=VPREB3	VPREB3.value > threshold=4.937594890594482
node_3072: feature_name=LTB	LTB.value > threshold=0.5297379791736603
node_3092: feature_name=YWHAH	YWHAH.value > threshold=0.6025846526026726
Class: COVID-19 Critical	
Rules_1263	passed counts:3
node_0: feature_name=LTB	LTB.value <= threshold=0.994031548500061
node_1: feature_name=MTRNR2L2	MTRNR2L2.value <= threshold=2.8784297704696655
node_2: feature_name=TCL1A	TCL1A.value <= threshold=4.798312664031982
node_3: feature_name=MT-CO1	MT-CO1.value > threshold=0.08947191759943962
node_2521: feature_name=LTB	LTB.value <= threshold=0.6038667261600494
node_2522: feature_name=HLA-DQB1	HLA-DQB1.value <= threshold=2.1053054332733154
node_2523: feature_name=SNX9	SNX9.value > threshold=-0.5346486568450928
node_2907: feature_name=NR4A1	NR4A1.value <= threshold=2.5433837175369263
node_2908: feature_name=XIST	XIST.value <= threshold=0.7664470989257097
node_2909: feature_name=CD79B	CD79B.value <= threshold=2.9326847791671753
node_2910: feature_name=NR4A1	NR4A1.value <= threshold=-0.2875557690858841
node_2911: feature_name=CTD-2035E11.3	CTD-2035E11.3.value <= threshold=5.262048006057739
node_2912: feature_name=VPREB3	VPREB3.value <= threshold=4.937594890594482
node_2913: feature_name=IRAK3	IRAK3.value <= threshold=4.592883586883545
node_2914: feature_name=IGJ	IGJ.value <= threshold=7.093410015106201
node_2915: feature_name=WDR74	WDR74.value <= threshold=4.077916383743286

node_2916: feature_name=MEF2C	MEF2C.value > threshold=4.374382972717285
node_3058: feature_name=HLA-DRB1	HLA-DRB1.value > threshold=1.63593327999115
node_3060: feature_name=TCF4	TCF4.value <= threshold=3.4358397722244263
Class: Healthy Control	
Rules_1264	passed counts:3
node_0: feature_name=LTB	LTB.value <= threshold=0.994031548500061
node_1: feature_name=MTRNR2L2	MTRNR2L2.value <= threshold=2.8784297704696655
node_2: feature_name=TCL1A	TCL1A.value <= threshold=4.798312664031982
node_3: feature_name=MT-CO1	MT-CO1.value > threshold=0.08947191759943962
node_2521: feature_name=LTB	LTB.value <= threshold=0.6038667261600494
node_2522: feature_name=HLA-DQB1	HLA-DQB1.value <= threshold=2.1053054332733154
node_2523: feature_name=SNX9	SNX9.value > threshold=-0.5346486568450928
node_2907: feature_name=NR4A1	NR4A1.value <= threshold=2.5433837175369263
node_2908: feature_name=XIST	XIST.value <= threshold=0.7664470989257097
node_2909: feature_name=CD79B	CD79B.value <= threshold=2.9326847791671753
node_2910: feature_name=NR4A1	NR4A1.value <= threshold=-0.2875557690858841
node_2911: feature_name=CTD-2035E11.3	CTD-2035E11.3.value <= threshold=5.262048006057739
node_2912: feature_name=VPREB3	VPREB3.value <= threshold=4.937594890594482
node_2913: feature_name=IRAK3	IRAK3.value <= threshold=4.592883586883545
node_2914: feature_name=IGJ	IGJ.value <= threshold=7.093410015106201
node_2915: feature_name=WDR74	WDR74.value <= threshold=4.077916383743286
node_2916: feature_name=MEF2C	MEF2C.value <= threshold=4.374382972717285
node_2917: feature_name=HIST1H2AC	HIST1H2AC.value > threshold=3.743621349334717
node_3055: feature_name=ITGAX	ITGAX.value <= threshold=-0.2822466939687729
Class: Healthy Control	
Rules_1265	passed counts:3
node_0: feature_name=LTB	LTB.value <= threshold=0.994031548500061
node_1: feature_name=MTRNR2L2	MTRNR2L2.value <= threshold=2.8784297704696655

node_2: feature_name=TCL1A	TCL1A.value <= threshold=4.798312664031982
node_3: feature_name=MT-CO1	MT-CO1.value > threshold=0.08947191759943962
node_2521: feature_name=LTB	LTB.value <= threshold=0.6038667261600494
node_2522: feature_name=HLA-DQB1	HLA-DQB1.value <= threshold=2.1053054332733154
node_2523: feature_name=SNX9	SNX9.value > threshold=-0.5346486568450928
node_2907: feature_name=NR4A1	NR4A1.value <= threshold=2.5433837175369263
node_2908: feature_name=XIST	XIST.value <= threshold=0.7664470989257097
node_2909: feature_name=CD79B	CD79B.value <= threshold=2.9326847791671753
node_2910: feature_name=NR4A1	NR4A1.value <= threshold=-0.2875557690858841
node_2911: feature_name=CTD-2035E11.3	CTD-2035E11.3.value <= threshold=5.262048006057739
node_2912: feature_name=VPREB3	VPREB3.value <= threshold=4.937594890594482
node_2913: feature_name=IRAK3	IRAK3.value <= threshold=4.592883586883545
node_2914: feature_name=IGJ	IGJ.value <= threshold=7.093410015106201
node_2915: feature_name=WDR74	WDR74.value <= threshold=4.077916383743286
node_2916: feature_name=MEF2C	MEF2C.value <= threshold=4.374382972717285
node_2917: feature_name=HIST1H2AC	HIST1H2AC.value <= threshold=3.743621349334717
node_2918: feature_name=STMN1	STMN1.value <= threshold=6.266510248184204
node_2919: feature_name=RALGPS2	RALGPS2.value <= threshold=5.72194766998291
node_2920: feature_name=HOPX	HOPX.value <= threshold=2.5944807529449463
node_2921: feature_name=MARCKS	MARCKS.value <= threshold=4.622651815414429
node_2922: feature_name=CCL3	CCL3.value <= threshold=0.7484713792800903
node_2923: feature_name=TPM1	TPM1.value <= threshold=6.446193695068359
node_2924: feature_name=PLD4	PLD4.value <= threshold=8.038907289505005
node_2925: feature_name=COTL1	COTL1.value > threshold=-1.8123814463615417
node_2929: feature_name=ID3	ID3.value <= threshold=7.534881353378296
node_2930: feature_name=IRF8	IRF8.value <= threshold=4.855781078338623
node_2931: feature_name=AP3M2	AP3M2.value <= threshold=1.8868122100830078
node_2932: feature_name=FCER2	FCER2.value <= threshold=6.877448558807373
node_2933: feature_name=MTRNR2L2	MTRNR2L2.value <= threshold=2.710945725440979
node_2934: feature_name=GNG11	GNG11.value <= threshold=2.510925054550171

node_2935: feature_name=CD83	CD83.value > threshold=1.9873459935188293
node_2961: feature_name=CD83	CD83.value > threshold=2.0049850940704346
node_2965: feature_name=HES4	HES4.value <= threshold=4.076817035675049
node_2966: feature_name=BCL2A1	BCL2A1.value > threshold=1.1745486855506897
node_2996: feature_name=LTB	LTB.value > threshold=-0.9909347593784332
node_2998: feature_name=VPREB3	VPREB3.value > threshold=2.2705172896385193
node_3000: feature_name=HLA-DPB1	HLA-DPB1.value > threshold=1.313808262348175
Class: Healthy Control	
Rules_1266	passed counts:3
node_0: feature_name=LTB	LTB.value <= threshold=0.994031548500061
node_1: feature_name=MTRNR2L2	MTRNR2L2.value <= threshold=2.8784297704696655
node_2: feature_name=TCL1A	TCL1A.value <= threshold=4.798312664031982
node_3: feature_name=MT-CO1	MT-CO1.value > threshold=0.08947191759943962
node_2521: feature_name=LTB	LTB.value <= threshold=0.6038667261600494
node_2522: feature_name=HLA-DQB1	HLA-DQB1.value <= threshold=2.1053054332733154
node_2523: feature_name=SNX9	SNX9.value > threshold=-0.5346486568450928
node_2907: feature_name=NR4A1	NR4A1.value <= threshold=2.5433837175369263
node_2908: feature_name=XIST	XIST.value <= threshold=0.7664470989257097
node_2909: feature_name=CD79B	CD79B.value <= threshold=2.9326847791671753
node_2910: feature_name=NR4A1	NR4A1.value <= threshold=-0.2875557690858841
node_2911: feature_name=CTD-2035E11.3	CTD-2035E11.3.value <= threshold=5.262048006057739
node_2912: feature_name=VPREB3	VPREB3.value <= threshold=4.937594890594482
node_2913: feature_name=IRAK3	IRAK3.value <= threshold=4.592883586883545
node_2914: feature_name=IGJ	IGJ.value <= threshold=7.093410015106201
node_2915: feature_name=WDR74	WDR74.value <= threshold=4.077916383743286
node_2916: feature_name=MEF2C	MEF2C.value <= threshold=4.374382972717285
node_2917: feature_name=HIST1H2AC	HIST1H2AC.value <= threshold=3.743621349334717
node_2918: feature_name=STMN1	STMN1.value <= threshold=6.266510248184204
node_2919: feature_name=RALGPS2	RALGPS2.value <= threshold=5.72194766998291

node_2920: feature_name=HOPX	HOPX.value <= threshold=2.5944807529449463
node_2921: feature_name=MARCKS	MARCKS.value <= threshold=4.622651815414429
node_2922: feature_name=CCL3	CCL3.value <= threshold=0.7484713792800903
node_2923: feature_name=TPM1	TPM1.value <= threshold=6.446193695068359
node_2924: feature_name=PLD4	PLD4.value <= threshold=8.038907289505005
node_2925: feature_name=COTL1	COTL1.value > threshold=-1.8123814463615417
node_2929: feature_name=ID3	ID3.value <= threshold=7.534881353378296
node_2930: feature_name=IRF8	IRF8.value <= threshold=4.855781078338623
node_2931: feature_name=AP3M2	AP3M2.value <= threshold=1.8868122100830078
node_2932: feature_name=FCER2	FCER2.value <= threshold=6.877448558807373
node_2933: feature_name=MTRNR2L2	MTRNR2L2.value <= threshold=2.710945725440979
node_2934: feature_name=GNG11	GNG11.value <= threshold=2.510925054550171
node_2935: feature_name=CD83	CD83.value > threshold=1.9873459935188293
node_2961: feature_name=CD83	CD83.value > threshold=2.0049850940704346
node_2965: feature_name=HES4	HES4.value <= threshold=4.076817035675049
node_2966: feature_name=BCL2A1	BCL2A1.value <= threshold=1.1745486855506897
node_2967: feature_name=NT5C3A	NT5C3A.value <= threshold=3.6911903619766235
node_2968: feature_name=TLN1	TLN1.value <= threshold=2.122964382171631
node_2969: feature_name=MT-CYB	MT-CYB.value > threshold=-0.9192460179328918
node_2973: feature_name=KIAA0125	KIAA0125.value > threshold=6.9272027015686035
node_2987: feature_name=MTRNR2L8	MTRNR2L8.value <= threshold=0.20285551249980927
Class: COVID-19 Critical	
Rules_1267	passed counts:3
node_0: feature_name=LTB	LTB.value <= threshold=0.994031548500061
node_1: feature_name=MTRNR2L2	MTRNR2L2.value <= threshold=2.8784297704696655
node_2: feature_name=TCL1A	TCL1A.value <= threshold=4.798312664031982
node_3: feature_name=MT-CO1	MT-CO1.value > threshold=0.08947191759943962
node_2521: feature_name=LTB	LTB.value <= threshold=0.6038667261600494
node_2522: feature_name=HLA-DQB1	HLA-DQB1.value <= threshold=2.1053054332733154

node_2523: feature_name=SNX9	SNX9.value > threshold=-0.5346486568450928
node_2907: feature_name=NR4A1	NR4A1.value <= threshold=2.5433837175369263
node_2908: feature_name=XIST	XIST.value <= threshold=0.7664470989257097
node_2909: feature_name=CD79B	CD79B.value <= threshold=2.9326847791671753
node_2910: feature_name=NR4A1	NR4A1.value <= threshold=-0.2875557690858841
node_2911: feature_name=CTD-2035E11.3	CTD-2035E11.3.value <= threshold=5.262048006057739
node_2912: feature_name=VPREB3	VPREB3.value <= threshold=4.937594890594482
node_2913: feature_name=IRAK3	IRAK3.value <= threshold=4.592883586883545
node_2914: feature_name=IGJ	IGJ.value <= threshold=7.093410015106201
node_2915: feature_name=WDR74	WDR74.value <= threshold=4.077916383743286
node_2916: feature_name=MEF2C	MEF2C.value <= threshold=4.374382972717285
node_2917: feature_name=HIST1H2AC	HIST1H2AC.value <= threshold=3.743621349334717
node_2918: feature_name=STMN1	STMN1.value <= threshold=6.266510248184204
node_2919: feature_name=RALGPS2	RALGPS2.value <= threshold=5.72194766998291
node_2920: feature_name=HOPX	HOPX.value <= threshold=2.5944807529449463
node_2921: feature_name=MARCKS	MARCKS.value <= threshold=4.622651815414429
node_2922: feature_name=CCL3	CCL3.value <= threshold=0.7484713792800903
node_2923: feature_name=TPM1	TPM1.value <= threshold=6.446193695068359
node_2924: feature_name=PLD4	PLD4.value <= threshold=8.038907289505005
node_2925: feature_name=COTL1	COTL1.value > threshold=-1.8123814463615417
node_2929: feature_name=ID3	ID3.value <= threshold=7.534881353378296
node_2930: feature_name=IRF8	IRF8.value <= threshold=4.855781078338623
node_2931: feature_name=AP3M2	AP3M2.value <= threshold=1.8868122100830078
node_2932: feature_name=FCER2	FCER2.value <= threshold=6.877448558807373
node_2933: feature_name=MTRNR2L2	MTRNR2L2.value <= threshold=2.710945725440979
node_2934: feature_name=GNG11	GNG11.value <= threshold=2.510925054550171
node_2935: feature_name=CD83	CD83.value > threshold=1.9873459935188293
node_2961: feature_name=CD83	CD83.value > threshold=2.0049850940704346
node_2965: feature_name=HES4	HES4.value <= threshold=4.076817035675049
node_2966: feature_name=BCL2A1	BCL2A1.value <= threshold=1.1745486855506897

node_2967: feature_name=NT5C3A	NT5C3A.value <= threshold=3.6911903619766235
node_2968: feature_name=TLN1	TLN1.value <= threshold=2.122964382171631
node_2969: feature_name=MT-CYB	MT-CYB.value > threshold=-0.9192460179328918
node_2973: feature_name=KIAA0125	KIAA0125.value <= threshold=6.9272027015686035
node_2974: feature_name=OAZ1	OAZ1.value <= threshold=-1.8451763987541199
node_2975: feature_name=FTH1	FTH1.value <= threshold=0.543534591794014
Class: COVID-19 Critical	
Rules_1268	passed counts:3
node_0: feature_name=LTB	LTB.value <= threshold=0.994031548500061
node_1: feature_name=MTRNR2L2	MTRNR2L2.value <= threshold=2.8784297704696655
node_2: feature_name=TCL1A	TCL1A.value <= threshold=4.798312664031982
node_3: feature_name=MT-CO1	MT-CO1.value > threshold=0.08947191759943962
node_2521: feature_name=LTB	LTB.value <= threshold=0.6038667261600494
node_2522: feature_name=HLA-DQB1	HLA-DQB1.value <= threshold=2.1053054332733154
node_2523: feature_name=SNX9	SNX9.value > threshold=-0.5346486568450928
node_2907: feature_name=NR4A1	NR4A1.value <= threshold=2.5433837175369263
node_2908: feature_name=XIST	XIST.value <= threshold=0.7664470989257097
node_2909: feature_name=CD79B	CD79B.value <= threshold=2.9326847791671753
node_2910: feature_name=NR4A1	NR4A1.value <= threshold=-0.2875557690858841
node_2911: feature_name=CTD-2035E11.3	CTD-2035E11.3.value <= threshold=5.262048006057739
node_2912: feature_name=VPREB3	VPREB3.value <= threshold=4.937594890594482
node_2913: feature_name=IRAK3	IRAK3.value <= threshold=4.592883586883545
node_2914: feature_name=IGJ	IGJ.value <= threshold=7.093410015106201
node_2915: feature_name=WDR74	WDR74.value <= threshold=4.077916383743286
node_2916: feature_name=MEF2C	MEF2C.value <= threshold=4.374382972717285
node_2917: feature_name=HIST1H2AC	HIST1H2AC.value <= threshold=3.743621349334717
node_2918: feature_name=STMN1	STMN1.value <= threshold=6.266510248184204
node_2919: feature_name=RALGPS2	RALGPS2.value <= threshold=5.72194766998291
node_2920: feature_name=HOPX	HOPX.value <= threshold=2.5944807529449463

node_2921: feature_name=MARCKS	MARCKS.value <= threshold=4.622651815414429
node_2922: feature_name=CCL3	CCL3.value <= threshold=0.7484713792800903
node_2923: feature_name=TPM1	TPM1.value <= threshold=6.446193695068359
node_2924: feature_name=PLD4	PLD4.value <= threshold=8.038907289505005
node_2925: feature_name=COTL1	COTL1.value > threshold=-1.8123814463615417
node_2929: feature_name=ID3	ID3.value <= threshold=7.534881353378296
node_2930: feature_name=IRF8	IRF8.value <= threshold=4.855781078338623
node_2931: feature_name=AP3M2	AP3M2.value <= threshold=1.8868122100830078
node_2932: feature_name=FCER2	FCER2.value <= threshold=6.877448558807373
node_2933: feature_name=MTRNR2L2	MTRNR2L2.value <= threshold=2.710945725440979
node_2934: feature_name=GNG11	GNG11.value <= threshold=2.510925054550171
node_2935: feature_name=CD83	CD83.value > threshold=1.9873459935188293
node_2961: feature_name=CD83	CD83.value <= threshold=2.0049850940704346
node_2962: feature_name=HSP90B1	HSP90B1.value > threshold=0.18583302199840546
Class: COVID-19 Severe	
Rules_1269	passed counts:3
node_0: feature_name=LTB	LTB.value <= threshold=0.994031548500061
node_1: feature_name=MTRNR2L2	MTRNR2L2.value <= threshold=2.8784297704696655
node_2: feature_name=TCL1A	TCL1A.value <= threshold=4.798312664031982
node_3: feature_name=MT-CO1	MT-CO1.value > threshold=0.08947191759943962
node_2521: feature_name=LTB	LTB.value <= threshold=0.6038667261600494
node_2522: feature_name=HLA-DQB1	HLA-DQB1.value <= threshold=2.1053054332733154
node_2523: feature_name=SNX9	SNX9.value > threshold=-0.5346486568450928
node_2907: feature_name=NR4A1	NR4A1.value <= threshold=2.5433837175369263
node_2908: feature_name=XIST	XIST.value <= threshold=0.7664470989257097
node_2909: feature_name=CD79B	CD79B.value <= threshold=2.9326847791671753
node_2910: feature_name=NR4A1	NR4A1.value <= threshold=-0.2875557690858841
node_2911: feature_name=CTD-2035E11.3	CTD-2035E11.3.value <= threshold=5.262048006057739
node_2912: feature_name=VPREB3	VPREB3.value <= threshold=4.937594890594482

node_2913: feature_name=IRAK3	IRAK3.value <= threshold=4.592883586883545
node_2914: feature_name=IGJ	IGJ.value <= threshold=7.093410015106201
node_2915: feature_name=WDR74	WDR74.value <= threshold=4.077916383743286
node_2916: feature_name=MEF2C	MEF2C.value <= threshold=4.374382972717285
node_2917: feature_name=HIST1H2AC	HIST1H2AC.value <= threshold=3.743621349334717
node_2918: feature_name=STMN1	STMN1.value <= threshold=6.266510248184204
node_2919: feature_name=RALGPS2	RALGPS2.value <= threshold=5.72194766998291
node_2920: feature_name=HOPX	HOPX.value <= threshold=2.5944807529449463
node_2921: feature_name=MARCKS	MARCKS.value <= threshold=4.622651815414429
node_2922: feature_name=CCL3	CCL3.value <= threshold=0.7484713792800903
node_2923: feature_name=TPM1	TPM1.value <= threshold=6.446193695068359
node_2924: feature_name=PLD4	PLD4.value <= threshold=8.038907289505005
node_2925: feature_name=COTL1	COTL1.value > threshold=-1.8123814463615417
node_2929: feature_name=ID3	ID3.value <= threshold=7.534881353378296
node_2930: feature_name=IRF8	IRF8.value <= threshold=4.855781078338623
node_2931: feature_name=AP3M2	AP3M2.value <= threshold=1.8868122100830078
node_2932: feature_name=FCER2	FCER2.value <= threshold=6.877448558807373
node_2933: feature_name=MTRNR2L2	MTRNR2L2.value <= threshold=2.710945725440979
node_2934: feature_name=GNG11	GNG11.value <= threshold=2.510925054550171
node_2935: feature_name=CD83	CD83.value > threshold=1.9873459935188293
node_2961: feature_name=CD83	CD83.value <= threshold=2.0049850940704346
node_2962: feature_name=HSP90B1	HSP90B1.value <= threshold=0.18583302199840546
Class: COVID-19 Critical	
Rules_1270	passed counts:3
node_0: feature_name=LTB	LTB.value <= threshold=0.994031548500061
node_1: feature_name=MTRNR2L2	MTRNR2L2.value <= threshold=2.8784297704696655
node_2: feature_name=TCL1A	TCL1A.value <= threshold=4.798312664031982
node_3: feature_name=MT-CO1	MT-CO1.value > threshold=0.08947191759943962
node_2521: feature_name=LTB	LTB.value <= threshold=0.6038667261600494

node_2522: feature_name=HLA-DQB1	HLA-DQB1.value <= threshold=2.1053054332733154
node_2523: feature_name=SNX9	SNX9.value > threshold=-0.5346486568450928
node_2907: feature_name=NR4A1	NR4A1.value <= threshold=2.5433837175369263
node_2908: feature_name=XIST	XIST.value <= threshold=0.7664470989257097
node_2909: feature_name=CD79B	CD79B.value <= threshold=2.9326847791671753
node_2910: feature_name=NR4A1	NR4A1.value <= threshold=-0.2875557690858841
node_2911: feature_name=CTD-2035E11.3	CTD-2035E11.3.value <= threshold=5.262048006057739
node_2912: feature_name=VPREB3	VPREB3.value <= threshold=4.937594890594482
node_2913: feature_name=IRAK3	IRAK3.value <= threshold=4.592883586883545
node_2914: feature_name=IGJ	IGJ.value <= threshold=7.093410015106201
node_2915: feature_name=WDR74	WDR74.value <= threshold=4.077916383743286
node_2916: feature_name=MEF2C	MEF2C.value <= threshold=4.374382972717285
node_2917: feature_name=HIST1H2AC	HIST1H2AC.value <= threshold=3.743621349334717
node_2918: feature_name=STMN1	STMN1.value <= threshold=6.266510248184204
node_2919: feature_name=RALGPS2	RALGPS2.value <= threshold=5.72194766998291
node_2920: feature_name=HOPX	HOPX.value <= threshold=2.5944807529449463
node_2921: feature_name=MARCKS	MARCKS.value <= threshold=4.622651815414429
node_2922: feature_name=CCL3	CCL3.value <= threshold=0.7484713792800903
node_2923: feature_name=TPM1	TPM1.value <= threshold=6.446193695068359
node_2924: feature_name=PLD4	PLD4.value <= threshold=8.038907289505005
node_2925: feature_name=COTL1	COTL1.value > threshold=-1.8123814463615417
node_2929: feature_name=ID3	ID3.value <= threshold=7.534881353378296
node_2930: feature_name=IRF8	IRF8.value <= threshold=4.855781078338623
node_2931: feature_name=AP3M2	AP3M2.value <= threshold=1.8868122100830078
node_2932: feature_name=FCER2	FCER2.value <= threshold=6.877448558807373
node_2933: feature_name=MTRNR2L2	MTRNR2L2.value <= threshold=2.710945725440979
node_2934: feature_name=GNG11	GNG11.value <= threshold=2.510925054550171
node_2935: feature_name=CD83	CD83.value <= threshold=1.9873459935188293
node_2936: feature_name=MARCH1	MARCH1.value > threshold=5.006200551986694
node_2958: feature_name=ITM2C	ITM2C.value > threshold=-0.19988703727722168

Class: COVID-19 Critical	
Rules_1271	passed counts:3
node_0: feature_name=LTB	LTB.value <= threshold=0.994031548500061
node_1: feature_name=MTRNR2L2	MTRNR2L2.value <= threshold=2.8784297704696655
node_2: feature_name=TCL1A	TCL1A.value <= threshold=4.798312664031982
node_3: feature_name=MT-CO1	MT-CO1.value > threshold=0.08947191759943962
node_2521: feature_name=LTB	LTB.value <= threshold=0.6038667261600494
node_2522: feature_name=HLA-DQB1	HLA-DQB1.value <= threshold=2.1053054332733154
node_2523: feature_name=SNX9	SNX9.value > threshold=-0.5346486568450928
node_2907: feature_name=NR4A1	NR4A1.value <= threshold=2.5433837175369263
node_2908: feature_name=XIST	XIST.value <= threshold=0.7664470989257097
node_2909: feature_name=CD79B	CD79B.value <= threshold=2.9326847791671753
node_2910: feature_name=NR4A1	NR4A1.value <= threshold=-0.2875557690858841
node_2911: feature_name=CTD-2035E11.3	CTD-2035E11.3.value <= threshold=5.262048006057739
node_2912: feature_name=VPREB3	VPREB3.value <= threshold=4.937594890594482
node_2913: feature_name=IRAK3	IRAK3.value <= threshold=4.592883586883545
node_2914: feature_name=IGJ	IGJ.value <= threshold=7.093410015106201
node_2915: feature_name=WDR74	WDR74.value <= threshold=4.077916383743286
node_2916: feature_name=MEF2C	MEF2C.value <= threshold=4.374382972717285
node_2917: feature_name=HIST1H2AC	HIST1H2AC.value <= threshold=3.743621349334717
node_2918: feature_name=STMN1	STMN1.value <= threshold=6.266510248184204
node_2919: feature_name=RALGPS2	RALGPS2.value <= threshold=5.72194766998291
node_2920: feature_name=HOPX	HOPX.value <= threshold=2.5944807529449463
node_2921: feature_name=MARCKS	MARCKS.value <= threshold=4.622651815414429
node_2922: feature_name=CCL3	CCL3.value <= threshold=0.7484713792800903
node_2923: feature_name=TPM1	TPM1.value <= threshold=6.446193695068359
node_2924: feature_name=PLD4	PLD4.value <= threshold=8.038907289505005
node_2925: feature_name=COTL1	COTL1.value > threshold=-1.8123814463615417
node_2929: feature_name=ID3	ID3.value <= threshold=7.534881353378296

node_2930: feature_name=IRF8	IRF8.value <= threshold=4.855781078338623
node_2931: feature_name=AP3M2	AP3M2.value <= threshold=1.8868122100830078
node_2932: feature_name=FCER2	FCER2.value <= threshold=6.877448558807373
node_2933: feature_name=MTRNR2L2	MTRNR2L2.value <= threshold=2.710945725440979
node_2934: feature_name=GNG11	GNG11.value <= threshold=2.510925054550171
node_2935: feature_name=CD83	CD83.value <= threshold=1.9873459935188293
node_2936: feature_name=MARCH1	MARCH1.value <= threshold=5.006200551986694
node_2937: feature_name=MEF2C	MEF2C.value > threshold=-0.5498070418834686
node_2941: feature_name=FCRL1	FCRL1.value > threshold=7.093972444534302
node_2955: feature_name=SYNE1	SYNE1.value <= threshold=-0.5938310325145721
Class: COVID-19 Critical	
Rules_1272	passed counts:3
node_0: feature_name=LTB	LTB.value <= threshold=0.994031548500061
node_1: feature_name=MTRNR2L2	MTRNR2L2.value <= threshold=2.8784297704696655
node_2: feature_name=TCL1A	TCL1A.value <= threshold=4.798312664031982
node_3: feature_name=MT-CO1	MT-CO1.value > threshold=0.08947191759943962
node_2521: feature_name=LTB	LTB.value <= threshold=0.6038667261600494
node_2522: feature_name=HLA-DQB1	HLA-DQB1.value <= threshold=2.1053054332733154
node_2523: feature_name=SNX9	SNX9.value > threshold=-0.5346486568450928
node_2907: feature_name=NR4A1	NR4A1.value <= threshold=2.5433837175369263
node_2908: feature_name=XIST	XIST.value <= threshold=0.7664470989257097
node_2909: feature_name=CD79B	CD79B.value <= threshold=2.9326847791671753
node_2910: feature_name=NR4A1	NR4A1.value <= threshold=-0.2875557690858841
node_2911: feature_name=CTD-2035E11.3	CTD-2035E11.3.value <= threshold=5.262048006057739
node_2912: feature_name=VPREB3	VPREB3.value <= threshold=4.937594890594482
node_2913: feature_name=IRAK3	IRAK3.value <= threshold=4.592883586883545
node_2914: feature_name=IGJ	IGJ.value <= threshold=7.093410015106201
node_2915: feature_name=WDR74	WDR74.value <= threshold=4.077916383743286
node_2916: feature_name=MEF2C	MEF2C.value <= threshold=4.374382972717285

node_2917: feature_name=HIST1H2AC	HIST1H2AC.value <= threshold=3.743621349334717
node_2918: feature_name=STMN1	STMN1.value <= threshold=6.266510248184204
node_2919: feature_name=RALGPS2	RALGPS2.value <= threshold=5.72194766998291
node_2920: feature_name=HOPX	HOPX.value <= threshold=2.5944807529449463
node_2921: feature_name=MARCKS	MARCKS.value <= threshold=4.622651815414429
node_2922: feature_name=CCL3	CCL3.value <= threshold=0.7484713792800903
node_2923: feature_name=TPM1	TPM1.value <= threshold=6.446193695068359
node_2924: feature_name=PLD4	PLD4.value <= threshold=8.038907289505005
node_2925: feature_name=COTL1	COTL1.value > threshold=-1.8123814463615417
node_2929: feature_name=ID3	ID3.value <= threshold=7.534881353378296
node_2930: feature_name=IRF8	IRF8.value <= threshold=4.855781078338623
node_2931: feature_name=AP3M2	AP3M2.value <= threshold=1.8868122100830078
node_2932: feature_name=FCER2	FCER2.value <= threshold=6.877448558807373
node_2933: feature_name=MTRNR2L2	MTRNR2L2.value <= threshold=2.710945725440979
node_2934: feature_name=GNG11	GNG11.value <= threshold=2.510925054550171
node_2935: feature_name=CD83	CD83.value <= threshold=1.9873459935188293
node_2936: feature_name=MARCH1	MARCH1.value <= threshold=5.006200551986694
node_2937: feature_name=MEF2C	MEF2C.value <= threshold=-0.5498070418834686
node_2938: feature_name=HLA-DPA1	HLA-DPA1.value > threshold=0.29968614876270294
Class: COVID-19 Critical	
Rules_1273	passed counts:3
node_0: feature_name=LTB	LTB.value <= threshold=0.994031548500061
node_1: feature_name=MTRNR2L2	MTRNR2L2.value <= threshold=2.8784297704696655
node_2: feature_name=TCL1A	TCL1A.value <= threshold=4.798312664031982
node_3: feature_name=MT-CO1	MT-CO1.value > threshold=0.08947191759943962
node_2521: feature_name=LTB	LTB.value <= threshold=0.6038667261600494
node_2522: feature_name=HLA-DQB1	HLA-DQB1.value <= threshold=2.1053054332733154
node_2523: feature_name=SNX9	SNX9.value <= threshold=-0.5346486568450928
node_2524: feature_name=HLA-DPB1	HLA-DPB1.value > threshold=1.9548270106315613

node_2814: feature_name=CD79B	CD79B.value > threshold=-0.28186772763729095
node_2826: feature_name=MT-ND6	MT-ND6.value > threshold=0.17273088544607162
node_2872: feature_name=IFI44L	IFI44L.value <= threshold=-0.31519079208374023
node_2873: feature_name=RALGPS2	RALGPS2.value > threshold=1.6798744797706604
node_2887: feature_name=HLA-DQA2	HLA-DQA2.value <= threshold=5.12495756149292
node_2888: feature_name=MS4A1	MS4A1.value > threshold=3.8452248573303223
node_2900: feature_name=NR4A1	NR4A1.value <= threshold=-0.24637621641159058
node_2901: feature_name=GRASP	GRASP.value > threshold=3.1462806463241577
Class: Healthy Control	
Rules_1274	passed counts:3
node_0: feature_name=LTB	LTB.value <= threshold=0.994031548500061
node_1: feature_name=MTRNR2L2	MTRNR2L2.value <= threshold=2.8784297704696655
node_2: feature_name=TCL1A	TCL1A.value <= threshold=4.798312664031982
node_3: feature_name=MT-CO1	MT-CO1.value > threshold=0.08947191759943962
node_2521: feature_name=LTB	LTB.value <= threshold=0.6038667261600494
node_2522: feature_name=HLA-DQB1	HLA-DQB1.value <= threshold=2.1053054332733154
node_2523: feature_name=SNX9	SNX9.value <= threshold=-0.5346486568450928
node_2524: feature_name=HLA-DPB1	HLA-DPB1.value > threshold=1.9548270106315613
node_2814: feature_name=CD79B	CD79B.value > threshold=-0.28186772763729095
node_2826: feature_name=MT-ND6	MT-ND6.value <= threshold=0.17273088544607162
node_2827: feature_name=NR4A1	NR4A1.value > threshold=-0.24634335190057755
node_2849: feature_name=FAM129C	FAM129C.value <= threshold=3.089340925216675
node_2850: feature_name=COTL1	COTL1.value > threshold=-0.5968812704086304
node_2856: feature_name=DENND3	DENND3.value <= threshold=-0.3556941896677017
node_2857: feature_name=ISG15	ISG15.value <= threshold=-0.5639307200908661
node_2858: feature_name=MX1	MX1.value <= threshold=0.23652145266532898
Class: Healthy Control	
Rules_1275	passed counts:3

node_0: feature_name=LTB	LTB.value <= threshold=0.994031548500061
node_1: feature_name=MTRNR2L2	MTRNR2L2.value <= threshold=2.8784297704696655
node_2: feature_name=TCL1A	TCL1A.value <= threshold=4.798312664031982
node_3: feature_name=MT-CO1	MT-CO1.value > threshold=0.08947191759943962
node_2521: feature_name=LTB	LTB.value <= threshold=0.6038667261600494
node_2522: feature_name=HLA-DQB1	HLA-DQB1.value <= threshold=2.1053054332733154
node_2523: feature_name=SNX9	SNX9.value <= threshold=-0.5346486568450928
node_2524: feature_name=HLA-DPB1	HLA-DPB1.value > threshold=1.9548270106315613
node_2814: feature_name=CD79B	CD79B.value > threshold=-0.28186772763729095
node_2826: feature_name=MT-ND6	MT-ND6.value <= threshold=0.17273088544607162
node_2827: feature_name=NR4A1	NR4A1.value <= threshold=-0.24634335190057755
node_2828: feature_name=NR4A3	NR4A3.value > threshold=1.609707236289978
node_2844: feature_name=MARCKS	MARCKS.value <= threshold=-0.17721937596797943
Class: Healthy Control	
Rules_1276	passed counts:3
node_0: feature_name=LTB	LTB.value <= threshold=0.994031548500061
node_1: feature_name=MTRNR2L2	MTRNR2L2.value <= threshold=2.8784297704696655
node_2: feature_name=TCL1A	TCL1A.value <= threshold=4.798312664031982
node_3: feature_name=MT-CO1	MT-CO1.value > threshold=0.08947191759943962
node_2521: feature_name=LTB	LTB.value <= threshold=0.6038667261600494
node_2522: feature_name=HLA-DQB1	HLA-DQB1.value <= threshold=2.1053054332733154
node_2523: feature_name=SNX9	SNX9.value <= threshold=-0.5346486568450928
node_2524: feature_name=HLA-DPB1	HLA-DPB1.value > threshold=1.9548270106315613
node_2814: feature_name=CD79B	CD79B.value <= threshold=-0.28186772763729095
node_2815: feature_name=HLA-DRB5	HLA-DRB5.value <= threshold=0.7097251415252686
node_2816: feature_name=HOPX	HOPX.value <= threshold=-0.4761084169149399
Class: COVID-19 Severe	
Rules_1277	passed counts:3

node_0: feature_name=LTB	LTB.value <= threshold=0.994031548500061
node_1: feature_name=MTRNR2L2	MTRNR2L2.value <= threshold=2.8784297704696655
node_2: feature_name=TCL1A	TCL1A.value <= threshold=4.798312664031982
node_3: feature_name=MT-CO1	MT-CO1.value > threshold=0.08947191759943962
node_2521: feature_name=LTB	LTB.value <= threshold=0.6038667261600494
node_2522: feature_name=HLA-DQB1	HLA-DQB1.value <= threshold=2.1053054332733154
node_2523: feature_name=SNX9	SNX9.value <= threshold=-0.5346486568450928
node_2524: feature_name=HLA-DPB1	HLA-DPB1.value <= threshold=1.9548270106315613
node_2525: feature_name=FAM129C	FAM129C.value > threshold=3.3393388986587524
node_2721: feature_name=ADAM19	ADAM19.value <= threshold=-0.29525794088840485
node_2722: feature_name=NR4A1	NR4A1.value > threshold=2.1566513776779175
node_2774: feature_name=HLA-DRB1	HLA-DRB1.value > threshold=0.8161951005458832
node_2776: feature_name=UTY	UTY.value > threshold=2.9337798357009888
Class: COVID-19 Critical	
Rules_1278	passed counts:3
node_0: feature_name=LTB	LTB.value <= threshold=0.994031548500061
node_1: feature_name=MTRNR2L2	MTRNR2L2.value <= threshold=2.8784297704696655
node_2: feature_name=TCL1A	TCL1A.value <= threshold=4.798312664031982
node_3: feature_name=MT-CO1	MT-CO1.value > threshold=0.08947191759943962
node_2521: feature_name=LTB	LTB.value <= threshold=0.6038667261600494
node_2522: feature_name=HLA-DQB1	HLA-DQB1.value <= threshold=2.1053054332733154
node_2523: feature_name=SNX9	SNX9.value <= threshold=-0.5346486568450928
node_2524: feature_name=HLA-DPB1	HLA-DPB1.value <= threshold=1.9548270106315613
node_2525: feature_name=FAM129C	FAM129C.value > threshold=3.3393388986587524
node_2721: feature_name=ADAM19	ADAM19.value <= threshold=-0.29525794088840485
node_2722: feature_name=NR4A1	NR4A1.value <= threshold=2.1566513776779175
node_2723: feature_name=CD22	CD22.value <= threshold=8.345317840576172
node_2724: feature_name=PLD4	PLD4.value > threshold=7.507996082305908
Class: Healthy Control	

Rules_1279	passed counts:3
node_0: feature_name=LTB	LTB.value <= threshold=0.994031548500061
node_1: feature_name=MTRNR2L2	MTRNR2L2.value <= threshold=2.8784297704696655
node_2: feature_name=TCL1A	TCL1A.value <= threshold=4.798312664031982
node_3: feature_name=MT-CO1	MT-CO1.value > threshold=0.08947191759943962
node_2521: feature_name=LTB	LTB.value <= threshold=0.6038667261600494
node_2522: feature_name=HLA-DQB1	HLA-DQB1.value <= threshold=2.1053054332733154
node_2523: feature_name=SNX9	SNX9.value <= threshold=-0.5346486568450928
node_2524: feature_name=HLA-DPB1	HLA-DPB1.value <= threshold=1.9548270106315613
node_2525: feature_name=FAM129C	FAM129C.value <= threshold=3.3393388986587524
node_2526: feature_name=XIST	XIST.value <= threshold=3.6911416053771973
node_2527: feature_name=NR4A1	NR4A1.value > threshold=-0.2557755410671234
node_2625: feature_name=HLA-DPA1	HLA-DPA1.value > threshold=0.9335225224494934
node_2657: feature_name=RALGPS2	RALGPS2.value > threshold=2.9413715600967407
node_2701: feature_name=DUSP4	DUSP4.value <= threshold=-0.24480421096086502
node_2702: feature_name=MS4A1	MS4A1.value > threshold=1.7336164116859436
node_2704: feature_name=TLN1	TLN1.value > threshold=0.8644028902053833
node_2712: feature_name=TP53INP1	TP53INP1.value > threshold=-0.2623787075281143
Class: COVID-19 Critical	
Rules_1280	passed counts:3
node_0: feature_name=LTB	LTB.value <= threshold=0.994031548500061
node_1: feature_name=MTRNR2L2	MTRNR2L2.value <= threshold=2.8784297704696655
node_2: feature_name=TCL1A	TCL1A.value <= threshold=4.798312664031982
node_3: feature_name=MT-CO1	MT-CO1.value > threshold=0.08947191759943962
node_2521: feature_name=LTB	LTB.value <= threshold=0.6038667261600494
node_2522: feature_name=HLA-DQB1	HLA-DQB1.value <= threshold=2.1053054332733154
node_2523: feature_name=SNX9	SNX9.value <= threshold=-0.5346486568450928
node_2524: feature_name=HLA-DPB1	HLA-DPB1.value <= threshold=1.9548270106315613

node_2525: feature_name=FAM129C	FAM129C.value <= threshold=3.3393388986587524
node_2526: feature_name=XIST	XIST.value <= threshold=3.6911416053771973
node_2527: feature_name=NR4A1	NR4A1.value > threshold=-0.2557755410671234
node_2625: feature_name=HLA-DPA1	HLA-DPA1.value > threshold=0.9335225224494934
node_2657: feature_name=RALGPS2	RALGPS2.value <= threshold=2.9413715600967407
node_2658: feature_name=HLA-DQB1	HLA-DQB1.value <= threshold=1.7503233551979065
node_2659: feature_name=TP53INP1	TP53INP1.value > threshold=-0.2649386376142502
node_2675: feature_name=VPREB3	VPREB3.value > threshold=5.346607446670532
Class: Healthy Control	
Rules_1281	passed counts:3
node_0: feature_name=LTB	LTB.value <= threshold=0.994031548500061
node_1: feature_name=MTRNR2L2	MTRNR2L2.value <= threshold=2.8784297704696655
node_2: feature_name=TCL1A	TCL1A.value <= threshold=4.798312664031982
node_3: feature_name=MT-CO1	MT-CO1.value > threshold=0.08947191759943962
node_2521: feature_name=LTB	LTB.value <= threshold=0.6038667261600494
node_2522: feature_name=HLA-DQB1	HLA-DQB1.value <= threshold=2.1053054332733154
node_2523: feature_name=SNX9	SNX9.value <= threshold=-0.5346486568450928
node_2524: feature_name=HLA-DPB1	HLA-DPB1.value <= threshold=1.9548270106315613
node_2525: feature_name=FAM129C	FAM129C.value <= threshold=3.3393388986587524
node_2526: feature_name=XIST	XIST.value <= threshold=3.6911416053771973
node_2527: feature_name=NR4A1	NR4A1.value > threshold=-0.2557755410671234
node_2625: feature_name=HLA-DPA1	HLA-DPA1.value > threshold=0.9335225224494934
node_2657: feature_name=RALGPS2	RALGPS2.value <= threshold=2.9413715600967407
node_2658: feature_name=HLA-DQB1	HLA-DQB1.value <= threshold=1.7503233551979065
node_2659: feature_name=TP53INP1	TP53INP1.value <= threshold=-0.2649386376142502
node_2660: feature_name=OAZ1	OAZ1.value <= threshold=1.2488974928855896
node_2661: feature_name=HLA-DPB1	HLA-DPB1.value > threshold=-0.5502273440361023
node_2667: feature_name=SLC7A5	SLC7A5.value > threshold=-0.5558908581733704
node_2671: feature_name=CTD-2035E11.3	CTD-2035E11.3.value <= threshold=-0.26417140662670135

Class: COVID-19 Critical	
Rules_1282	passed counts:3
node_0: feature_name=LTB	LTB.value <= threshold=0.994031548500061
node_1: feature_name=MTRNR2L2	MTRNR2L2.value <= threshold=2.8784297704696655
node_2: feature_name=TCL1A	TCL1A.value <= threshold=4.798312664031982
node_3: feature_name=MT-CO1	MT-CO1.value > threshold=0.08947191759943962
node_2521: feature_name=LTB	LTB.value <= threshold=0.6038667261600494
node_2522: feature_name=HLA-DQB1	HLA-DQB1.value <= threshold=2.1053054332733154
node_2523: feature_name=SNX9	SNX9.value <= threshold=-0.5346486568450928
node_2524: feature_name=HLA-DPB1	HLA-DPB1.value <= threshold=1.9548270106315613
node_2525: feature_name=FAM129C	FAM129C.value <= threshold=3.3393388986587524
node_2526: feature_name=XIST	XIST.value <= threshold=3.6911416053771973
node_2527: feature_name=NR4A1	NR4A1.value > threshold=-0.2557755410671234
node_2625: feature_name=HLA-DPA1	HLA-DPA1.value <= threshold=0.9335225224494934
node_2626: feature_name=CD83	CD83.value > threshold=-0.585537850856781
node_2628: feature_name=PDLIM1	PDLIM1.value > threshold=5.292127370834351
node_2654: feature_name=MARCKS	MARCKS.value <= threshold=1.5881791897118092
Class: Healthy Control	
Rules_1283	passed counts:3
node_0: feature_name=LTB	LTB.value <= threshold=0.994031548500061
node_1: feature_name=MTRNR2L2	MTRNR2L2.value <= threshold=2.8784297704696655
node_2: feature_name=TCL1A	TCL1A.value <= threshold=4.798312664031982
node_3: feature_name=MT-CO1	MT-CO1.value > threshold=0.08947191759943962
node_2521: feature_name=LTB	LTB.value <= threshold=0.6038667261600494
node_2522: feature_name=HLA-DQB1	HLA-DQB1.value <= threshold=2.1053054332733154
node_2523: feature_name=SNX9	SNX9.value <= threshold=-0.5346486568450928
node_2524: feature_name=HLA-DPB1	HLA-DPB1.value <= threshold=1.9548270106315613
node_2525: feature_name=FAM129C	FAM129C.value <= threshold=3.3393388986587524

node_2526: feature_name=XIST	XIST.value <= threshold=3.6911416053771973
node_2527: feature_name=NR4A1	NR4A1.value > threshold=-0.2557755410671234
node_2625: feature_name=HLA-DPA1	HLA-DPA1.value <= threshold=0.9335225224494934
node_2626: feature_name=CD83	CD83.value <= threshold=-0.585537850856781
Class: Healthy Control	
Rules_1284	passed counts:3
node_0: feature_name=LTB	LTB.value <= threshold=0.994031548500061
node_1: feature_name=MTRNR2L2	MTRNR2L2.value <= threshold=2.8784297704696655
node_2: feature_name=TCL1A	TCL1A.value <= threshold=4.798312664031982
node_3: feature_name=MT-CO1	MT-CO1.value > threshold=0.08947191759943962
node_2521: feature_name=LTB	LTB.value <= threshold=0.6038667261600494
node_2522: feature_name=HLA-DQB1	HLA-DQB1.value <= threshold=2.1053054332733154
node_2523: feature_name=SNX9	SNX9.value <= threshold=-0.5346486568450928
node_2524: feature_name=HLA-DPB1	HLA-DPB1.value <= threshold=1.9548270106315613
node_2525: feature_name=FAM129C	FAM129C.value <= threshold=3.3393388986587524
node_2526: feature_name=XIST	XIST.value <= threshold=3.6911416053771973
node_2527: feature_name=NR4A1	NR4A1.value <= threshold=-0.2557755410671234
node_2528: feature_name=HOPX	HOPX.value <= threshold=2.776473879814148
node_2529: feature_name=AREGB	AREGB.value > threshold=-0.10896047577261925
Class: Healthy Control	
Rules_1285	passed counts:3
node_0: feature_name=LTB	LTB.value <= threshold=0.994031548500061
node_1: feature_name=MTRNR2L2	MTRNR2L2.value <= threshold=2.8784297704696655
node_2: feature_name=TCL1A	TCL1A.value <= threshold=4.798312664031982
node_3: feature_name=MT-CO1	MT-CO1.value > threshold=0.08947191759943962
node_2521: feature_name=LTB	LTB.value <= threshold=0.6038667261600494
node_2522: feature_name=HLA-DQB1	HLA-DQB1.value <= threshold=2.1053054332733154
node_2523: feature_name=SNX9	SNX9.value <= threshold=-0.5346486568450928

node_2524: feature_name=HLA-DPB1	HLA-DPB1.value <= threshold=1.9548270106315613
node_2525: feature_name=FAM129C	FAM129C.value <= threshold=3.3393388986587524
node_2526: feature_name=XIST	XIST.value <= threshold=3.6911416053771973
node_2527: feature_name=NR4A1	NR4A1.value <= threshold=-0.2557755410671234
node_2528: feature_name=HOPX	HOPX.value <= threshold=2.776473879814148
node_2529: feature_name=AREGB	AREGB.value <= threshold=-0.10896047577261925
node_2530: feature_name=KIAA0125	KIAA0125.value > threshold=7.652706623077393
node_2618: feature_name=MEF2A	MEF2A.value > threshold=-0.44190549850463867
Class: Healthy Control	
Rules_1286	passed counts:3
node_0: feature_name=LTB	LTB.value <= threshold=0.994031548500061
node_1: feature_name=MTRNR2L2	MTRNR2L2.value <= threshold=2.8784297704696655
node_2: feature_name=TCL1A	TCL1A.value <= threshold=4.798312664031982
node_3: feature_name=MT-CO1	MT-CO1.value > threshold=0.08947191759943962
node_2521: feature_name=LTB	LTB.value <= threshold=0.6038667261600494
node_2522: feature_name=HLA-DQB1	HLA-DQB1.value <= threshold=2.1053054332733154
node_2523: feature_name=SNX9	SNX9.value <= threshold=-0.5346486568450928
node_2524: feature_name=HLA-DPB1	HLA-DPB1.value <= threshold=1.9548270106315613
node_2525: feature_name=FAM129C	FAM129C.value <= threshold=3.3393388986587524
node_2526: feature_name=XIST	XIST.value <= threshold=3.6911416053771973
node_2527: feature_name=NR4A1	NR4A1.value <= threshold=-0.2557755410671234
node_2528: feature_name=HOPX	HOPX.value <= threshold=2.776473879814148
node_2529: feature_name=AREGB	AREGB.value <= threshold=-0.10896047577261925
node_2530: feature_name=KIAA0125	KIAA0125.value <= threshold=7.652706623077393
node_2531: feature_name=MT-CO3	MT-CO3.value > threshold=-1.3726763725280762
node_2533: feature_name=TNF	TNF.value <= threshold=3.1101887226104736
node_2534: feature_name=NFKBID	NFKBID.value > threshold=-0.39706873893737793
node_2568: feature_name=TCF4	TCF4.value <= threshold=6.289925575256348
node_2569: feature_name=CD22	CD22.value <= threshold=8.377277374267578

node_2570: feature_name=FERMT3	FERMT3.value <= threshold=2.260508179664612
node_2571: feature_name=MT-ND6	MT-ND6.value <= threshold=3.413356304168701
node_2572: feature_name=ID1	ID1.value <= threshold=1.9121195077896118
node_2573: feature_name=DAPK1	DAPK1.value <= threshold=1.6979314014315605
node_2574: feature_name=IRF4	IRF4.value <= threshold=8.765593528747559
node_2575: feature_name=PLD4	PLD4.value <= threshold=6.6784141063690186
node_2576: feature_name=GPX1	GPX1.value > threshold=-0.9480071663856506
node_2582: feature_name=MYL9	MYL9.value > threshold=2.9503014087677
node_2596: feature_name=ID1	ID1.value > threshold=-0.49309042096138
Class: COVID-19 Critical	
Rules_1287	passed counts:3
node_0: feature_name=LTB	LTB.value <= threshold=0.994031548500061
node_1: feature_name=MTRNR2L2	MTRNR2L2.value <= threshold=2.8784297704696655
node_2: feature_name=TCL1A	TCL1A.value <= threshold=4.798312664031982
node_3: feature_name=MT-CO1	MT-CO1.value > threshold=0.08947191759943962
node_2521: feature_name=LTB	LTB.value <= threshold=0.6038667261600494
node_2522: feature_name=HLA-DQB1	HLA-DQB1.value <= threshold=2.1053054332733154
node_2523: feature_name=SNX9	SNX9.value <= threshold=-0.5346486568450928
node_2524: feature_name=HLA-DPB1	HLA-DPB1.value <= threshold=1.9548270106315613
node_2525: feature_name=FAM129C	FAM129C.value <= threshold=3.3393388986587524
node_2526: feature_name=XIST	XIST.value <= threshold=3.6911416053771973
node_2527: feature_name=NR4A1	NR4A1.value <= threshold=-0.2557755410671234
node_2528: feature_name=HOPX	HOPX.value <= threshold=2.776473879814148
node_2529: feature_name=AREGB	AREGB.value <= threshold=-0.10896047577261925
node_2530: feature_name=KIAA0125	KIAA0125.value <= threshold=7.652706623077393
node_2531: feature_name=MT-CO3	MT-CO3.value > threshold=-1.3726763725280762
node_2533: feature_name=TNF	TNF.value <= threshold=3.1101887226104736
node_2534: feature_name=NFKBID	NFKBID.value > threshold=-0.39706873893737793
node_2568: feature_name=TCF4	TCF4.value <= threshold=6.289925575256348

node_2569: feature_name=CD22	CD22.value <= threshold=8.377277374267578
node_2570: feature_name=FERMT3	FERMT3.value <= threshold=2.260508179664612
node_2571: feature_name=MT-ND6	MT-ND6.value <= threshold=3.413356304168701
node_2572: feature_name=ID1	ID1.value <= threshold=1.9121195077896118
node_2573: feature_name=DAPK1	DAPK1.value <= threshold=1.6979314014315605
node_2574: feature_name=IRF4	IRF4.value <= threshold=8.765593528747559
node_2575: feature_name=PLD4	PLD4.value <= threshold=6.6784141063690186
node_2576: feature_name=GPX1	GPX1.value > threshold=-0.9480071663856506
node_2582: feature_name=MYL9	MYL9.value <= threshold=2.9503014087677
node_2583: feature_name=ID1	ID1.value > threshold=1.3511708974838257
node_2593: feature_name=LDLRAD4	LDLRAD4.value <= threshold=1.1256642043590546
Class: COVID-19 Critical	
Rules_1288	passed counts:3
node_0: feature_name=LTB	LTB.value <= threshold=0.994031548500061
node_1: feature_name=MTRNR2L2	MTRNR2L2.value <= threshold=2.8784297704696655
node_2: feature_name=TCL1A	TCL1A.value <= threshold=4.798312664031982
node_3: feature_name=MT-CO1	MT-CO1.value > threshold=0.08947191759943962
node_2521: feature_name=LTB	LTB.value <= threshold=0.6038667261600494
node_2522: feature_name=HLA-DQB1	HLA-DQB1.value <= threshold=2.1053054332733154
node_2523: feature_name=SNX9	SNX9.value <= threshold=-0.5346486568450928
node_2524: feature_name=HLA-DPB1	HLA-DPB1.value <= threshold=1.9548270106315613
node_2525: feature_name=FAM129C	FAM129C.value <= threshold=3.3393388986587524
node_2526: feature_name=XIST	XIST.value <= threshold=3.6911416053771973
node_2527: feature_name=NR4A1	NR4A1.value <= threshold=-0.2557755410671234
node_2528: feature_name=HOPX	HOPX.value <= threshold=2.776473879814148
node_2529: feature_name=AREGB	AREGB.value <= threshold=-0.10896047577261925
node_2530: feature_name=KIAA0125	KIAA0125.value <= threshold=7.652706623077393
node_2531: feature_name=MT-CO3	MT-CO3.value > threshold=-1.3726763725280762
node_2533: feature_name=TNF	TNF.value <= threshold=3.1101887226104736

node_2534: feature_name=NFKBID	NFKBID.value > threshold=-0.39706873893737793
node_2568: feature_name=TCF4	TCF4.value <= threshold=6.289925575256348
node_2569: feature_name=CD22	CD22.value <= threshold=8.377277374267578
node_2570: feature_name=FERMT3	FERMT3.value <= threshold=2.260508179664612
node_2571: feature_name=MT-ND6	MT-ND6.value <= threshold=3.413356304168701
node_2572: feature_name=ID1	ID1.value <= threshold=1.9121195077896118
node_2573: feature_name=DAPK1	DAPK1.value <= threshold=1.6979314014315605
node_2574: feature_name=IRF4	IRF4.value <= threshold=8.765593528747559
node_2575: feature_name=PLD4	PLD4.value <= threshold=6.6784141063690186
node_2576: feature_name=GPX1	GPX1.value <= threshold=-0.9480071663856506
node_2577: feature_name=ILK	ILK.value > threshold=-0.4419245272874832
node_2579: feature_name=IRAK3	IRAK3.value <= threshold=0.6731721460819244
Class: Healthy Control	
Rules_1289	passed counts:3
node_0: feature_name=LTB	LTB.value <= threshold=0.994031548500061
node_1: feature_name=MTRNR2L2	MTRNR2L2.value <= threshold=2.8784297704696655
node_2: feature_name=TCL1A	TCL1A.value <= threshold=4.798312664031982
node_3: feature_name=MT-CO1	MT-CO1.value > threshold=0.08947191759943962
node_2521: feature_name=LTB	LTB.value <= threshold=0.6038667261600494
node_2522: feature_name=HLA-DQB1	HLA-DQB1.value <= threshold=2.1053054332733154
node_2523: feature_name=SNX9	SNX9.value <= threshold=-0.5346486568450928
node_2524: feature_name=HLA-DPB1	HLA-DPB1.value <= threshold=1.9548270106315613
node_2525: feature_name=FAM129C	FAM129C.value <= threshold=3.3393388986587524
node_2526: feature_name=XIST	XIST.value <= threshold=3.6911416053771973
node_2527: feature_name=NR4A1	NR4A1.value <= threshold=-0.2557755410671234
node_2528: feature_name=HOPX	HOPX.value <= threshold=2.776473879814148
node_2529: feature_name=AREGB	AREGB.value <= threshold=-0.10896047577261925
node_2530: feature_name=KIAA0125	KIAA0125.value <= threshold=7.652706623077393
node_2531: feature_name=MT-CO3	MT-CO3.value > threshold=-1.3726763725280762

node_2533: feature_name=TNF	TNF.value <= threshold=3.1101887226104736
node_2534: feature_name=NFKBID	NFKBID.value <= threshold=-0.39706873893737793
node_2535: feature_name=KIAA0125	KIAA0125.value > threshold=-0.17713353037834167
node_2543: feature_name=XAF1	XAF1.value > threshold=1.7887146472930908
node_2563: feature_name=HLA-DPA1	HLA-DPA1.value > threshold=0.6970475614070892
node_2565: feature_name=ICAM1	ICAM1.value <= threshold=-0.28189119696617126
Class: Healthy Control	
Rules_1290	passed counts:3
node_0: feature_name=LTB	LTB.value <= threshold=0.994031548500061
node_1: feature_name=MTRNR2L2	MTRNR2L2.value <= threshold=2.8784297704696655
node_2: feature_name=TCL1A	TCL1A.value <= threshold=4.798312664031982
node_3: feature_name=MT-CO1	MT-CO1.value > threshold=0.08947191759943962
node_2521: feature_name=LTB	LTB.value <= threshold=0.6038667261600494
node_2522: feature_name=HLA-DQB1	HLA-DQB1.value <= threshold=2.1053054332733154
node_2523: feature_name=SNX9	SNX9.value <= threshold=-0.5346486568450928
node_2524: feature_name=HLA-DPB1	HLA-DPB1.value <= threshold=1.9548270106315613
node_2525: feature_name=FAM129C	FAM129C.value <= threshold=3.3393388986587524
node_2526: feature_name=XIST	XIST.value <= threshold=3.6911416053771973
node_2527: feature_name=NR4A1	NR4A1.value <= threshold=-0.2557755410671234
node_2528: feature_name=HOPX	HOPX.value <= threshold=2.776473879814148
node_2529: feature_name=AREGB	AREGB.value <= threshold=-0.10896047577261925
node_2530: feature_name=KIAA0125	KIAA0125.value <= threshold=7.652706623077393
node_2531: feature_name=MT-CO3	MT-CO3.value > threshold=-1.3726763725280762
node_2533: feature_name=TNF	TNF.value <= threshold=3.1101887226104736
node_2534: feature_name=NFKBID	NFKBID.value <= threshold=-0.39706873893737793
node_2535: feature_name=KIAA0125	KIAA0125.value > threshold=-0.17713353037834167
node_2543: feature_name=XAF1	XAF1.value <= threshold=1.7887146472930908
node_2544: feature_name=TLR2	TLR2.value <= threshold=2.253062665462494
node_2545: feature_name=HLA-DRB5	HLA-DRB5.value <= threshold=3.099335193634033

node_2546: feature_name=HLA-DQB1	HLA-DQB1.value <= threshold=2.057031512260437
node_2547: feature_name=CCL3	CCL3.value <= threshold=2.8681111335754395
node_2548: feature_name=MIAT	MIAT.value <= threshold=8.097615242004395
node_2549: feature_name=NT5C3A	NT5C3A.value <= threshold=3.9882057905197144
node_2550: feature_name=PLD4	PLD4.value <= threshold=2.694515687879175
node_2551: feature_name=MT-ND6	MT-ND6.value > threshold=2.745527148246765
node_2553: feature_name=SESN3	SESN3.value > threshold=-0.4803318381309509
Class: COVID-19 Critical	
Rules_1291	passed counts:3
node_0: feature_name=LTB	LTB.value <= threshold=0.994031548500061
node_1: feature_name=MTRNR2L2	MTRNR2L2.value <= threshold=2.8784297704696655
node_2: feature_name=TCL1A	TCL1A.value <= threshold=4.798312664031982
node_3: feature_name=MT-CO1	MT-CO1.value <= threshold=0.08947191759943962
node_4: feature_name=NR4A1	NR4A1.value > threshold=1.0725348591804504
node_1622: feature_name=HLA-DQA2	HLA-DQA2.value > threshold=1.9496816992759705
node_2350: feature_name=BCL2A1	BCL2A1.value > threshold=1.1322240233421326
node_2466: feature_name=MS4A1	MS4A1.value <= threshold=2.7321707010269165
node_2467: feature_name=HSP90B1	HSP90B1.value <= threshold=0.8215908706188202
node_2468: feature_name=HLA-DQA1	HLA-DQA1.value > threshold=1.4212530851364136
node_2476: feature_name=MT-CO3	MT-CO3.value <= threshold=0.05051383376121521
node_2477: feature_name=CCR6	CCR6.value > threshold=-0.2537315636873245
node_2485: feature_name=BOD1L1	BOD1L1.value > threshold=-0.4756772369146347
Class: COVID-19 Critical	
Rules_1292	passed counts:3
node_0: feature_name=LTB	LTB.value <= threshold=0.994031548500061
node_1: feature_name=MTRNR2L2	MTRNR2L2.value <= threshold=2.8784297704696655
node_2: feature_name=TCL1A	TCL1A.value <= threshold=4.798312664031982
node_3: feature_name=MT-CO1	MT-CO1.value <= threshold=0.08947191759943962

node_4: feature_name=NR4A1	NR4A1.value > threshold=1.0725348591804504
node_1622: feature_name=HLA-DQA2	HLA-DQA2.value > threshold=1.9496816992759705
node_2350: feature_name=BCL2A1	BCL2A1.value > threshold=1.1322240233421326
node_2466: feature_name=MS4A1	MS4A1.value <= threshold=2.7321707010269165
node_2467: feature_name=HSP90B1	HSP90B1.value <= threshold=0.8215908706188202
node_2468: feature_name=HLA-DQA1	HLA-DQA1.value > threshold=1.4212530851364136
node_2476: feature_name=MT-CO3	MT-CO3.value <= threshold=0.05051383376121521
node_2477: feature_name=CCR6	CCR6.value <= threshold=-0.2537315636873245
node_2478: feature_name=S100A6	S100A6.value > threshold=-0.21191135048866272
node_2480: feature_name=HLA-DQB1	HLA-DQB1.value > threshold=0.9742099642753601
node_2482: feature_name=PLEC	PLEC.value <= threshold=-0.45634281635284424
Class: COVID-19 Critical	
Rules_1293	passed counts:3
node_0: feature_name=LTB	LTB.value <= threshold=0.994031548500061
node_1: feature_name=MTRNR2L2	MTRNR2L2.value <= threshold=2.8784297704696655
node_2: feature_name=TCL1A	TCL1A.value <= threshold=4.798312664031982
node_3: feature_name=MT-CO1	MT-CO1.value <= threshold=0.08947191759943962
node_4: feature_name=NR4A1	NR4A1.value > threshold=1.0725348591804504
node_1622: feature_name=HLA-DQA2	HLA-DQA2.value > threshold=1.9496816992759705
node_2350: feature_name=BCL2A1	BCL2A1.value > threshold=1.1322240233421326
node_2466: feature_name=MS4A1	MS4A1.value <= threshold=2.7321707010269165
node_2467: feature_name=HSP90B1	HSP90B1.value <= threshold=0.8215908706188202
node_2468: feature_name=HLA-DQA1	HLA-DQA1.value > threshold=1.4212530851364136
node_2476: feature_name=MT-CO3	MT-CO3.value <= threshold=0.05051383376121521
node_2477: feature_name=CCR6	CCR6.value <= threshold=-0.2537315636873245
node_2478: feature_name=S100A6	S100A6.value > threshold=-0.21191135048866272
node_2480: feature_name=HLA-DQB1	HLA-DQB1.value <= threshold=0.9742099642753601
Class: COVID-19 Severe	

Rules_1294	passed counts:3
node_0: feature_name=LTB	LTB.value <= threshold=0.994031548500061
node_1: feature_name=MTRNR2L2	MTRNR2L2.value <= threshold=2.8784297704696655
node_2: feature_name=TCL1A	TCL1A.value <= threshold=4.798312664031982
node_3: feature_name=MT-CO1	MT-CO1.value <= threshold=0.08947191759943962
node_4: feature_name=NR4A1	NR4A1.value > threshold=1.0725348591804504
node_1622: feature_name=HLA-DQA2	HLA-DQA2.value > threshold=1.9496816992759705
node_2350: feature_name=BCL2A1	BCL2A1.value > threshold=1.1322240233421326
node_2466: feature_name=MS4A1	MS4A1.value <= threshold=2.7321707010269165
node_2467: feature_name=HSP90B1	HSP90B1.value <= threshold=0.8215908706188202
node_2468: feature_name=HLA-DQA1	HLA-DQA1.value <= threshold=1.4212530851364136
node_2469: feature_name=STMN1	STMN1.value > threshold=-0.25645384192466736
node_2473: feature_name=NFKBIZ	NFKBIZ.value <= threshold=-0.023992985486984253
Class: COVID-19 Severe	
Rules_1295	passed counts:3
node_0: feature_name=LTB	LTB.value <= threshold=0.994031548500061
node_1: feature_name=MTRNR2L2	MTRNR2L2.value <= threshold=2.8784297704696655
node_2: feature_name=TCL1A	TCL1A.value <= threshold=4.798312664031982
node_3: feature_name=MT-CO1	MT-CO1.value <= threshold=0.08947191759943962
node_4: feature_name=NR4A1	NR4A1.value > threshold=1.0725348591804504
node_1622: feature_name=HLA-DQA2	HLA-DQA2.value > threshold=1.9496816992759705
node_2350: feature_name=BCL2A1	BCL2A1.value <= threshold=1.1322240233421326
node_2351: feature_name=CD83	CD83.value > threshold=2.3162450790405273
node_2391: feature_name=CD79B	CD79B.value <= threshold=1.7111427783966064
node_2392: feature_name=TTN	TTN.value > threshold=-0.28553803265094757
node_2430: feature_name=LTB	LTB.value <= threshold=0.3538805693387985
node_2431: feature_name=MT-CO3	MT-CO3.value > threshold=-0.8342754244804382
node_2433: feature_name=MT-CYB	MT-CYB.value <= threshold=-0.5145382583141327
Class: COVID-19 Severe	

Rules_1296	passed counts:3
node_0: feature_name=LTB	LTB.value <= threshold=0.994031548500061
node_1: feature_name=MTRNR2L2	MTRNR2L2.value <= threshold=2.8784297704696655
node_2: feature_name=TCL1A	TCL1A.value <= threshold=4.798312664031982
node_3: feature_name=MT-CO1	MT-CO1.value <= threshold=0.08947191759943962
node_4: feature_name=NR4A1	NR4A1.value > threshold=1.0725348591804504
node_1622: feature_name=HLA-DQA2	HLA-DQA2.value > threshold=1.9496816992759705
node_2350: feature_name=BCL2A1	BCL2A1.value <= threshold=1.1322240233421326
node_2351: feature_name=CD83	CD83.value > threshold=2.3162450790405273
node_2391: feature_name=CD79B	CD79B.value <= threshold=1.7111427783966064
node_2392: feature_name=TTN	TTN.value <= threshold=-0.28553803265094757
node_2393: feature_name=LDLRAD4	LDLRAD4.value <= threshold=3.3328956365585327
node_2394: feature_name=BCL2A1	BCL2A1.value > threshold=-0.5811292827129364
node_2404: feature_name=SLC7A5	SLC7A5.value > threshold=1.3126214146614075
node_2422: feature_name=PPIF	PPIF.value > threshold=-0.42122700810432434
Class: Healthy Control	
Rules_1297	passed counts:3
node_0: feature_name=LTB	LTB.value <= threshold=0.994031548500061
node_1: feature_name=MTRNR2L2	MTRNR2L2.value <= threshold=2.8784297704696655
node_2: feature_name=TCL1A	TCL1A.value <= threshold=4.798312664031982
node_3: feature_name=MT-CO1	MT-CO1.value <= threshold=0.08947191759943962
node_4: feature_name=NR4A1	NR4A1.value > threshold=1.0725348591804504
node_1622: feature_name=HLA-DQA2	HLA-DQA2.value > threshold=1.9496816992759705
node_2350: feature_name=BCL2A1	BCL2A1.value <= threshold=1.1322240233421326
node_2351: feature_name=CD83	CD83.value > threshold=2.3162450790405273
node_2391: feature_name=CD79B	CD79B.value <= threshold=1.7111427783966064
node_2392: feature_name=TTN	TTN.value <= threshold=-0.28553803265094757
node_2393: feature_name=LDLRAD4	LDLRAD4.value <= threshold=3.3328956365585327

node_2394: feature_name=BCL2A1	BCL2A1.value > threshold=-0.5811292827129364
node_2404: feature_name=SLC7A5	SLC7A5.value <= threshold=1.3126214146614075
node_2405: feature_name=AP3M2	AP3M2.value > threshold=1.617304801940918
Class: COVID-19 Critical	
Rules_1298	passed counts:3
node_0: feature_name=LTB	LTB.value <= threshold=0.994031548500061
node_1: feature_name=MTRNR2L2	MTRNR2L2.value <= threshold=2.8784297704696655
node_2: feature_name=TCL1A	TCL1A.value <= threshold=4.798312664031982
node_3: feature_name=MT-CO1	MT-CO1.value <= threshold=0.08947191759943962
node_4: feature_name=NR4A1	NR4A1.value > threshold=1.0725348591804504
node_1622: feature_name=HLA-DQA2	HLA-DQA2.value > threshold=1.9496816992759705
node_2350: feature_name=BCL2A1	BCL2A1.value <= threshold=1.1322240233421326
node_2351: feature_name=CD83	CD83.value <= threshold=2.3162450790405273
node_2352: feature_name=LTB	LTB.value <= threshold=0.7341383993625641
node_2353: feature_name=TCL1A	TCL1A.value <= threshold=3.942829728126526
node_2354: feature_name=MARCKS	MARCKS.value <= threshold=2.9345329999923706
node_2355: feature_name=ACSL1	ACSL1.value <= threshold=2.564934253692627
node_2356: feature_name=HLA-DQB1	HLA-DQB1.value <= threshold=-0.0751032829284668
node_2357: feature_name=TLR2	TLR2.value > threshold=-0.27227045595645905
Class: COVID-19 Severe	
Rules_1299	passed counts:3
node_0: feature_name=LTB	LTB.value <= threshold=0.994031548500061
node_1: feature_name=MTRNR2L2	MTRNR2L2.value <= threshold=2.8784297704696655
node_2: feature_name=TCL1A	TCL1A.value <= threshold=4.798312664031982
node_3: feature_name=MT-CO1	MT-CO1.value <= threshold=0.08947191759943962
node_4: feature_name=NR4A1	NR4A1.value > threshold=1.0725348591804504
node_1622: feature_name=HLA-DQA2	HLA-DQA2.value <= threshold=1.9496816992759705
node_1623: feature_name=HLA-DRB5	HLA-DRB5.value <= threshold=2.715766429901123

node_1624: feature_name=HLA-DPA1	HLA-DPA1.value > threshold=1.7050301432609558
node_2056: feature_name=OAZ1	OAZ1.value > threshold=0.456103190779686
node_2266: feature_name=XAF1	XAF1.value <= threshold=-0.4431648850440979
node_2267: feature_name=MT-CO1	MT-CO1.value > threshold=-0.3505740910768509
node_2293: feature_name=LTB	LTB.value <= threshold=-0.31744880229234695
node_2294: feature_name=PPIF	PPIF.value > threshold=-0.4462503343820572
node_2298: feature_name=RNF11	RNF11.value <= threshold=-0.5209715664386749
Class: Healthy Control	
Rules_1300	passed counts:3
node_0: feature_name=LTB	LTB.value <= threshold=0.994031548500061
node_1: feature_name=MTRNR2L2	MTRNR2L2.value <= threshold=2.8784297704696655
node_2: feature_name=TCL1A	TCL1A.value <= threshold=4.798312664031982
node_3: feature_name=MT-CO1	MT-CO1.value <= threshold=0.08947191759943962
node_4: feature_name=NR4A1	NR4A1.value > threshold=1.0725348591804504
node_1622: feature_name=HLA-DQA2	HLA-DQA2.value <= threshold=1.9496816992759705
node_1623: feature_name=HLA-DRB5	HLA-DRB5.value <= threshold=2.715766429901123
node_1624: feature_name=HLA-DPA1	HLA-DPA1.value > threshold=1.7050301432609558
node_2056: feature_name=OAZ1	OAZ1.value > threshold=0.456103190779686
node_2266: feature_name=XAF1	XAF1.value <= threshold=-0.4431648850440979
node_2267: feature_name=MT-CO1	MT-CO1.value <= threshold=-0.3505740910768509
node_2268: feature_name=HLA-DRB1	HLA-DRB1.value <= threshold=2.1576695442199707
node_2269: feature_name=MARCH1	MARCH1.value > threshold=1.492592692375183
node_2283: feature_name=FAM129C	FAM129C.value <= threshold=-0.2708045244216919
node_2284: feature_name=NFKBID	NFKBID.value > threshold=-0.3932444006204605
Class: COVID-19 Critical	
Rules_1301	passed counts:3
node_0: feature_name=LTB	LTB.value <= threshold=0.994031548500061
node_1: feature_name=MTRNR2L2	MTRNR2L2.value <= threshold=2.8784297704696655

node_2: feature_name=TCL1A	TCL1A.value <= threshold=4.798312664031982
node_3: feature_name=MT-CO1	MT-CO1.value <= threshold=0.08947191759943962
node_4: feature_name=NR4A1	NR4A1.value > threshold=1.0725348591804504
node_1622: feature_name=HLA-DQA2	HLA-DQA2.value <= threshold=1.9496816992759705
node_1623: feature_name=HLA-DRB5	HLA-DRB5.value <= threshold=2.715766429901123
node_1624: feature_name=HLA-DPA1	HLA-DPA1.value > threshold=1.7050301432609558
node_2056: feature_name=OAZ1	OAZ1.value <= threshold=0.456103190779686
node_2057: feature_name=HLA-DRB5	HLA-DRB5.value > threshold=0.6839984953403473
node_2101: feature_name=HLA-DRB1	HLA-DRB1.value > threshold=1.6199676990509033
node_2151: feature_name=HLA-DQB1	HLA-DQB1.value <= threshold=3.1961567401885986
node_2152: feature_name=PLD4	PLD4.value <= threshold=-0.03259922005236149
node_2153: feature_name=XIST	XIST.value <= threshold=-0.138966154307127
node_2154: feature_name=HLA-DQA1	HLA-DQA1.value > threshold=2.0848299264907837
node_2190: feature_name=RALGPS2	RALGPS2.value <= threshold=4.276920795440674
node_2191: feature_name=RGS2	RGS2.value > threshold=-0.7087429165840149
node_2195: feature_name=GRAP2	GRAP2.value <= threshold=2.2513980865478516
node_2196: feature_name=ID3	ID3.value <= threshold=5.227530002593994
node_2197: feature_name=CD79B	CD79B.value > threshold=4.226724624633789
node_2235: feature_name=GRASP	GRASP.value > threshold=-0.3945188969373703
Class: Healthy Control	
Rules_1302	passed counts:3
node_0: feature_name=LTB	LTB.value <= threshold=0.994031548500061
node_1: feature_name=MTRNR2L2	MTRNR2L2.value <= threshold=2.8784297704696655
node_2: feature_name=TCL1A	TCL1A.value <= threshold=4.798312664031982
node_3: feature_name=MT-CO1	MT-CO1.value <= threshold=0.08947191759943962
node_4: feature_name=NR4A1	NR4A1.value > threshold=1.0725348591804504
node_1622: feature_name=HLA-DQA2	HLA-DQA2.value <= threshold=1.9496816992759705
node_1623: feature_name=HLA-DRB5	HLA-DRB5.value <= threshold=2.715766429901123
node_1624: feature_name=HLA-DPA1	HLA-DPA1.value > threshold=1.7050301432609558

node_2056: feature_name=OAZ1	OAZ1.value <= threshold=0.456103190779686
node_2057: feature_name=HLA-DRB5	HLA-DRB5.value > threshold=0.6839984953403473
node_2101: feature_name=HLA-DRB1	HLA-DRB1.value > threshold=1.6199676990509033
node_2151: feature_name=HLA-DQB1	HLA-DQB1.value <= threshold=3.1961567401885986
node_2152: feature_name=PLD4	PLD4.value <= threshold=-0.03259922005236149
node_2153: feature_name=XIST	XIST.value <= threshold=-0.138966154307127
node_2154: feature_name=HLA-DQA1	HLA-DQA1.value > threshold=2.0848299264907837
node_2190: feature_name=RALGPS2	RALGPS2.value <= threshold=4.276920795440674
node_2191: feature_name=RGS2	RGS2.value > threshold=-0.7087429165840149
node_2195: feature_name=GRAP2	GRAP2.value <= threshold=2.2513980865478516
node_2196: feature_name=ID3	ID3.value <= threshold=5.227530002593994
node_2197: feature_name=CD79B	CD79B.value <= threshold=4.226724624633789
node_2198: feature_name=ENDOD1	ENDOD1.value <= threshold=4.877119541168213
node_2199: feature_name=AP3M2	AP3M2.value <= threshold=2.6568219661712646
node_2200: feature_name=ICAM1	ICAM1.value <= threshold=4.384357690811157
node_2201: feature_name=ILK	ILK.value > threshold=2.051138460636139
node_2225: feature_name=ACSL1	ACSL1.value <= threshold=-0.3219778835773468
node_2226: feature_name=TCL1A	TCL1A.value <= threshold=4.052885293960571
Class: Healthy Control	
Rules_1303	passed counts:3
node_0: feature_name=LTB	LTB.value <= threshold=0.994031548500061
node_1: feature_name=MTRNR2L2	MTRNR2L2.value <= threshold=2.8784297704696655
node_2: feature_name=TCL1A	TCL1A.value <= threshold=4.798312664031982
node_3: feature_name=MT-CO1	MT-CO1.value <= threshold=0.08947191759943962
node_4: feature_name=NR4A1	NR4A1.value > threshold=1.0725348591804504
node_1622: feature_name=HLA-DQA2	HLA-DQA2.value <= threshold=1.9496816992759705
node_1623: feature_name=HLA-DRB5	HLA-DRB5.value <= threshold=2.715766429901123
node_1624: feature_name=HLA-DPA1	HLA-DPA1.value > threshold=1.7050301432609558
node_2056: feature_name=OAZ1	OAZ1.value <= threshold=0.456103190779686

node_2057: feature_name=HLA-DRB5	HLA-DRB5.value > threshold=0.6839984953403473
node_2101: feature_name=HLA-DRB1	HLA-DRB1.value > threshold=1.6199676990509033
node_2151: feature_name=HLA-DQB1	HLA-DQB1.value <= threshold=3.1961567401885986
node_2152: feature_name=PLD4	PLD4.value <= threshold=-0.03259922005236149
node_2153: feature_name=XIST	XIST.value <= threshold=-0.138966154307127
node_2154: feature_name=HLA-DQA1	HLA-DQA1.value > threshold=2.0848299264907837
node_2190: feature_name=RALGPS2	RALGPS2.value <= threshold=4.276920795440674
node_2191: feature_name=RGS2	RGS2.value <= threshold=-0.7087429165840149
node_2192: feature_name=MEF2C	MEF2C.value > threshold=1.1360841393470764
Class: COVID-19 Critical	
Rules_1304	passed counts:3
node_0: feature_name=LTB	LTB.value <= threshold=0.994031548500061
node_1: feature_name=MTRNR2L2	MTRNR2L2.value <= threshold=2.8784297704696655
node_2: feature_name=TCL1A	TCL1A.value <= threshold=4.798312664031982
node_3: feature_name=MT-CO1	MT-CO1.value <= threshold=0.08947191759943962
node_4: feature_name=NR4A1	NR4A1.value > threshold=1.0725348591804504
node_1622: feature_name=HLA-DQA2	HLA-DQA2.value <= threshold=1.9496816992759705
node_1623: feature_name=HLA-DRB5	HLA-DRB5.value <= threshold=2.715766429901123
node_1624: feature_name=HLA-DPA1	HLA-DPA1.value > threshold=1.7050301432609558
node_2056: feature_name=OAZ1	OAZ1.value <= threshold=0.456103190779686
node_2057: feature_name=HLA-DRB5	HLA-DRB5.value > threshold=0.6839984953403473
node_2101: feature_name=HLA-DRB1	HLA-DRB1.value > threshold=1.6199676990509033
node_2151: feature_name=HLA-DQB1	HLA-DQB1.value <= threshold=3.1961567401885986
node_2152: feature_name=PLD4	PLD4.value <= threshold=-0.03259922005236149
node_2153: feature_name=XIST	XIST.value <= threshold=-0.138966154307127
node_2154: feature_name=HLA-DQA1	HLA-DQA1.value <= threshold=2.0848299264907837
node_2155: feature_name=LTB	LTB.value > threshold=-0.9993145167827606
node_2161: feature_name=CYBB	CYBB.value > threshold=1.514441728591919
node_2183: feature_name=SNX9	SNX9.value > threshold=1.4872435331344604

node_2187: feature_name=EGR1	EGR1.value > threshold=-0.3245360553264618
Class: COVID-19 Severe	
Rules_1305	passed counts:3
node_0: feature_name=LTB	LTB.value <= threshold=0.994031548500061
node_1: feature_name=MTRNR2L2	MTRNR2L2.value <= threshold=2.8784297704696655
node_2: feature_name=TCL1A	TCL1A.value <= threshold=4.798312664031982
node_3: feature_name=MT-CO1	MT-CO1.value <= threshold=0.08947191759943962
node_4: feature_name=NR4A1	NR4A1.value > threshold=1.0725348591804504
node_1622: feature_name=HLA-DQA2	HLA-DQA2.value <= threshold=1.9496816992759705
node_1623: feature_name=HLA-DRB5	HLA-DRB5.value <= threshold=2.715766429901123
node_1624: feature_name=HLA-DPA1	HLA-DPA1.value > threshold=1.7050301432609558
node_2056: feature_name=OAZ1	OAZ1.value <= threshold=0.456103190779686
node_2057: feature_name=HLA-DRB5	HLA-DRB5.value > threshold=0.6839984953403473
node_2101: feature_name=HLA-DRB1	HLA-DRB1.value > threshold=1.6199676990509033
node_2151: feature_name=HLA-DQB1	HLA-DQB1.value <= threshold=3.1961567401885986
node_2152: feature_name=PLD4	PLD4.value <= threshold=-0.03259922005236149
node_2153: feature_name=XIST	XIST.value <= threshold=-0.138966154307127
node_2154: feature_name=HLA-DQA1	HLA-DQA1.value <= threshold=2.0848299264907837
node_2155: feature_name=LTB	LTB.value > threshold=-0.9993145167827606
node_2161: feature_name=CYBB	CYBB.value > threshold=1.514441728591919
node_2183: feature_name=SNX9	SNX9.value > threshold=1.4872435331344604
node_2187: feature_name=EGR1	EGR1.value <= threshold=-0.3245360553264618
Class: COVID-19 Critical	
Rules_1306	passed counts:3
node_0: feature_name=LTB	LTB.value <= threshold=0.994031548500061
node_1: feature_name=MTRNR2L2	MTRNR2L2.value <= threshold=2.8784297704696655
node_2: feature_name=TCL1A	TCL1A.value <= threshold=4.798312664031982
node_3: feature_name=MT-CO1	MT-CO1.value <= threshold=0.08947191759943962

node_4: feature_name=NR4A1	NR4A1.value > threshold=1.0725348591804504
node_1622: feature_name=HLA-DQA2	HLA-DQA2.value <= threshold=1.9496816992759705
node_1623: feature_name=HLA-DRB5	HLA-DRB5.value <= threshold=2.715766429901123
node_1624: feature_name=HLA-DPA1	HLA-DPA1.value > threshold=1.7050301432609558
node_2056: feature_name=OAZ1	OAZ1.value <= threshold=0.456103190779686
node_2057: feature_name=HLA-DRB5	HLA-DRB5.value > threshold=0.6839984953403473
node_2101: feature_name=HLA-DRB1	HLA-DRB1.value > threshold=1.6199676990509033
node_2151: feature_name=HLA-DQB1	HLA-DQB1.value <= threshold=3.1961567401885986
node_2152: feature_name=PLD4	PLD4.value <= threshold=-0.03259922005236149
node_2153: feature_name=XIST	XIST.value <= threshold=-0.138966154307127
node_2154: feature_name=HLA-DQA1	HLA-DQA1.value <= threshold=2.0848299264907837
node_2155: feature_name=LTB	LTB.value > threshold=-0.9993145167827606
node_2161: feature_name=CYBB	CYBB.value <= threshold=1.514441728591919
node_2162: feature_name=CD83	CD83.value <= threshold=3.348180890083313
node_2163: feature_name=ID1	ID1.value > threshold=1.9755518436431885
node_2175: feature_name=TLN1	TLN1.value > threshold=-0.638816773891449
Class: COVID-19 Critical	
Rules_1307	passed counts:3
node_0: feature_name=LTB	LTB.value <= threshold=0.994031548500061
node_1: feature_name=MTRNR2L2	MTRNR2L2.value <= threshold=2.8784297704696655
node_2: feature_name=TCL1A	TCL1A.value <= threshold=4.798312664031982
node_3: feature_name=MT-CO1	MT-CO1.value <= threshold=0.08947191759943962
node_4: feature_name=NR4A1	NR4A1.value > threshold=1.0725348591804504
node_1622: feature_name=HLA-DQA2	HLA-DQA2.value <= threshold=1.9496816992759705
node_1623: feature_name=HLA-DRB5	HLA-DRB5.value <= threshold=2.715766429901123
node_1624: feature_name=HLA-DPA1	HLA-DPA1.value > threshold=1.7050301432609558
node_2056: feature_name=OAZ1	OAZ1.value <= threshold=0.456103190779686
node_2057: feature_name=HLA-DRB5	HLA-DRB5.value > threshold=0.6839984953403473
node_2101: feature_name=HLA-DRB1	HLA-DRB1.value > threshold=1.6199676990509033

node_2151: feature_name=HLA-DQB1	HLA-DQB1.value <= threshold=3.1961567401885986
node_2152: feature_name=PLD4	PLD4.value <= threshold=-0.03259922005236149
node_2153: feature_name=XIST	XIST.value <= threshold=-0.138966154307127
node_2154: feature_name=HLA-DQA1	HLA-DQA1.value <= threshold=2.0848299264907837
node_2155: feature_name=LTB	LTB.value > threshold=-0.9993145167827606
node_2161: feature_name=CYBB	CYBB.value <= threshold=1.514441728591919
node_2162: feature_name=CD83	CD83.value <= threshold=3.348180890083313
node_2163: feature_name=ID1	ID1.value > threshold=1.9755518436431885
node_2175: feature_name=TLN1	TLN1.value <= threshold=-0.638816773891449
Class: COVID-19 Severe	
Rules_1308	passed counts:3
node_0: feature_name=LTB	LTB.value <= threshold=0.994031548500061
node_1: feature_name=MTRNR2L2	MTRNR2L2.value <= threshold=2.8784297704696655
node_2: feature_name=TCL1A	TCL1A.value <= threshold=4.798312664031982
node_3: feature_name=MT-CO1	MT-CO1.value <= threshold=0.08947191759943962
node_4: feature_name=NR4A1	NR4A1.value > threshold=1.0725348591804504
node_1622: feature_name=HLA-DQA2	HLA-DQA2.value <= threshold=1.9496816992759705
node_1623: feature_name=HLA-DRB5	HLA-DRB5.value <= threshold=2.715766429901123
node_1624: feature_name=HLA-DPA1	HLA-DPA1.value > threshold=1.7050301432609558
node_2056: feature_name=OAZ1	OAZ1.value <= threshold=0.456103190779686
node_2057: feature_name=HLA-DRB5	HLA-DRB5.value > threshold=0.6839984953403473
node_2101: feature_name=HLA-DRB1	HLA-DRB1.value <= threshold=1.6199676990509033
node_2102: feature_name=CD83	CD83.value > threshold=2.5568599700927734
node_2124: feature_name=MARCH1	MARCH1.value <= threshold=-0.34506022930145264
node_2125: feature_name=FTH1	FTH1.value <= threshold=-0.19983553141355515
node_2126: feature_name=ID3	ID3.value > threshold=1.2159389927983284
Class: COVID-19 Severe	
Rules_1309	passed counts:3

node_0: feature_name=LTB	LTB.value <= threshold=0.994031548500061
node_1: feature_name=MTRNR2L2	MTRNR2L2.value <= threshold=2.8784297704696655
node_2: feature_name=TCL1A	TCL1A.value <= threshold=4.798312664031982
node_3: feature_name=MT-CO1	MT-CO1.value <= threshold=0.08947191759943962
node_4: feature_name=NR4A1	NR4A1.value > threshold=1.0725348591804504
node_1622: feature_name=HLA-DQA2	HLA-DQA2.value <= threshold=1.9496816992759705
node_1623: feature_name=HLA-DRB5	HLA-DRB5.value <= threshold=2.715766429901123
node_1624: feature_name=HLA-DPA1	HLA-DPA1.value > threshold=1.7050301432609558
node_2056: feature_name=OAZ1	OAZ1.value <= threshold=0.456103190779686
node_2057: feature_name=HLA-DRB5	HLA-DRB5.value > threshold=0.6839984953403473
node_2101: feature_name=HLA-DRB1	HLA-DRB1.value <= threshold=1.6199676990509033
node_2102: feature_name=CD83	CD83.value <= threshold=2.5568599700927734
node_2103: feature_name=MT-CO1	MT-CO1.value <= threshold=-0.08588453382253647
node_2104: feature_name=FAM129C	FAM129C.value > threshold=3.9770365953445435
Class: Healthy Control	
Rules_1310	passed counts:3
node_0: feature_name=LTB	LTB.value <= threshold=0.994031548500061
node_1: feature_name=MTRNR2L2	MTRNR2L2.value <= threshold=2.8784297704696655
node_2: feature_name=TCL1A	TCL1A.value <= threshold=4.798312664031982
node_3: feature_name=MT-CO1	MT-CO1.value <= threshold=0.08947191759943962
node_4: feature_name=NR4A1	NR4A1.value > threshold=1.0725348591804504
node_1622: feature_name=HLA-DQA2	HLA-DQA2.value <= threshold=1.9496816992759705
node_1623: feature_name=HLA-DRB5	HLA-DRB5.value <= threshold=2.715766429901123
node_1624: feature_name=HLA-DPA1	HLA-DPA1.value > threshold=1.7050301432609558
node_2056: feature_name=OAZ1	OAZ1.value <= threshold=0.456103190779686
node_2057: feature_name=HLA-DRB5	HLA-DRB5.value <= threshold=0.6839984953403473
node_2058: feature_name=IFI6	IFI6.value > threshold=-0.5314981639385223
node_2082: feature_name=XAF1	XAF1.value > threshold=-0.4537480026483536
node_2086: feature_name=RALGPS2	RALGPS2.value > threshold=3.8138545751571655

node_2096: feature_name=SNX9	SNX9.value > threshold=2.156690239906311
Class: COVID-19 Severe	
Rules_1311	passed counts:3
node_0: feature_name=LTB	LTB.value <= threshold=0.994031548500061
node_1: feature_name=MTRNR2L2	MTRNR2L2.value <= threshold=2.8784297704696655
node_2: feature_name=TCL1A	TCL1A.value <= threshold=4.798312664031982
node_3: feature_name=MT-CO1	MT-CO1.value <= threshold=0.08947191759943962
node_4: feature_name=NR4A1	NR4A1.value > threshold=1.0725348591804504
node_1622: feature_name=HLA-DQA2	HLA-DQA2.value <= threshold=1.9496816992759705
node_1623: feature_name=HLA-DRB5	HLA-DRB5.value <= threshold=2.715766429901123
node_1624: feature_name=HLA-DPA1	HLA-DPA1.value > threshold=1.7050301432609558
node_2056: feature_name=OAZ1	OAZ1.value <= threshold=0.456103190779686
node_2057: feature_name=HLA-DRB5	HLA-DRB5.value <= threshold=0.6839984953403473
node_2058: feature_name=IFI6	IFI6.value > threshold=-0.5314981639385223
node_2082: feature_name=XAF1	XAF1.value <= threshold=-0.4537480026483536
node_2083: feature_name=CCR6	CCR6.value <= threshold=-0.2884496599435806
Class: COVID-19 Severe	
Rules_1312	passed counts:3
node_0: feature_name=LTB	LTB.value <= threshold=0.994031548500061
node_1: feature_name=MTRNR2L2	MTRNR2L2.value <= threshold=2.8784297704696655
node_2: feature_name=TCL1A	TCL1A.value <= threshold=4.798312664031982
node_3: feature_name=MT-CO1	MT-CO1.value <= threshold=0.08947191759943962
node_4: feature_name=NR4A1	NR4A1.value > threshold=1.0725348591804504
node_1622: feature_name=HLA-DQA2	HLA-DQA2.value <= threshold=1.9496816992759705
node_1623: feature_name=HLA-DRB5	HLA-DRB5.value <= threshold=2.715766429901123
node_1624: feature_name=HLA-DPA1	HLA-DPA1.value > threshold=1.7050301432609558
node_2056: feature_name=OAZ1	OAZ1.value <= threshold=0.456103190779686
node_2057: feature_name=HLA-DRB5	HLA-DRB5.value <= threshold=0.6839984953403473

node_2058: feature_name=IFI6	IFI6.value <= threshold=-0.5314981639385223
node_2059: feature_name=CD83	CD83.value > threshold=2.6418607234954834
node_2075: feature_name=NFKBID	NFKBID.value > threshold=3.104505181312561
node_2079: feature_name=HLA-DPA1	HLA-DPA1.value <= threshold=1.8489333391189575
Class: COVID-19 Severe	
Rules_1313	passed counts:3
node_0: feature_name=LTB	LTB.value <= threshold=0.994031548500061
node_1: feature_name=MTRNR2L2	MTRNR2L2.value <= threshold=2.8784297704696655
node_2: feature_name=TCL1A	TCL1A.value <= threshold=4.798312664031982
node_3: feature_name=MT-CO1	MT-CO1.value <= threshold=0.08947191759943962
node_4: feature_name=NR4A1	NR4A1.value > threshold=1.0725348591804504
node_1622: feature_name=HLA-DQA2	HLA-DQA2.value <= threshold=1.9496816992759705
node_1623: feature_name=HLA-DRB5	HLA-DRB5.value <= threshold=2.715766429901123
node_1624: feature_name=HLA-DPA1	HLA-DPA1.value > threshold=1.7050301432609558
node_2056: feature_name=OAZ1	OAZ1.value <= threshold=0.456103190779686
node_2057: feature_name=HLA-DRB5	HLA-DRB5.value <= threshold=0.6839984953403473
node_2058: feature_name=IFI6	IFI6.value <= threshold=-0.5314981639385223
node_2059: feature_name=CD83	CD83.value <= threshold=2.6418607234954834
node_2060: feature_name=HLA-DRB1	HLA-DRB1.value > threshold=1.92746239900589
node_2070: feature_name=FTH1	FTH1.value > threshold=0.9439846873283386
Class: COVID-19 Critical	
Rules_1314	passed counts:3
node_0: feature_name=LTB	LTB.value <= threshold=0.994031548500061
node_1: feature_name=MTRNR2L2	MTRNR2L2.value <= threshold=2.8784297704696655
node_2: feature_name=TCL1A	TCL1A.value <= threshold=4.798312664031982
node_3: feature_name=MT-CO1	MT-CO1.value <= threshold=0.08947191759943962
node_4: feature_name=NR4A1	NR4A1.value > threshold=1.0725348591804504
node_1622: feature_name=HLA-DQA2	HLA-DQA2.value <= threshold=1.9496816992759705

node_1623: feature_name=HLA-DRB5	HLA-DRB5.value <= threshold=2.715766429901123
node_1624: feature_name=HLA-DPA1	HLA-DPA1.value > threshold=1.7050301432609558
node_2056: feature_name=OAZ1	OAZ1.value <= threshold=0.456103190779686
node_2057: feature_name=HLA-DRB5	HLA-DRB5.value <= threshold=0.6839984953403473
node_2058: feature_name=IFI6	IFI6.value <= threshold=-0.5314981639385223
node_2059: feature_name=CD83	CD83.value <= threshold=2.6418607234954834
node_2060: feature_name=HLA-DRB1	HLA-DRB1.value <= threshold=1.92746239900589
node_2061: feature_name=FCRL1	FCRL1.value > threshold=1.318458966910839
node_2065: feature_name=VPREB3	VPREB3.value <= threshold=-0.1917331963777542
Class: COVID-19 Severe	
Rules_1315	passed counts:3
node_0: feature_name=LTB	LTB.value <= threshold=0.994031548500061
node_1: feature_name=MTRNR2L2	MTRNR2L2.value <= threshold=2.8784297704696655
node_2: feature_name=TCL1A	TCL1A.value <= threshold=4.798312664031982
node_3: feature_name=MT-CO1	MT-CO1.value <= threshold=0.08947191759943962
node_4: feature_name=NR4A1	NR4A1.value > threshold=1.0725348591804504
node_1622: feature_name=HLA-DQA2	HLA-DQA2.value <= threshold=1.9496816992759705
node_1623: feature_name=HLA-DRB5	HLA-DRB5.value <= threshold=2.715766429901123
node_1624: feature_name=HLA-DPA1	HLA-DPA1.value <= threshold=1.7050301432609558
node_1625: feature_name=CD83	CD83.value > threshold=2.783489227294922
node_1873: feature_name=IFI44L	IFI44L.value <= threshold=1.5938722491264343
node_1874: feature_name=PLD4	PLD4.value > threshold=4.101001739501953
node_2028: feature_name=SNX9	SNX9.value > threshold=1.4460421800613403
node_2036: feature_name=ID1	ID1.value <= threshold=-0.23599714040756226
node_2037: feature_name=IGLL5	IGLL5.value <= threshold=-0.24608439952135086
node_2038: feature_name=MS4A1	MS4A1.value <= threshold=2.4277244806289673
Class: COVID-19 Severe	
Rules_1316	passed counts:3

node_0: feature_name=LTB	LTB.value <= threshold=0.994031548500061
node_1: feature_name=MTRNR2L2	MTRNR2L2.value <= threshold=2.8784297704696655
node_2: feature_name=TCL1A	TCL1A.value <= threshold=4.798312664031982
node_3: feature_name=MT-CO1	MT-CO1.value <= threshold=0.08947191759943962
node_4: feature_name=NR4A1	NR4A1.value > threshold=1.0725348591804504
node_1622: feature_name=HLA-DQA2	HLA-DQA2.value <= threshold=1.9496816992759705
node_1623: feature_name=HLA-DRB5	HLA-DRB5.value <= threshold=2.715766429901123
node_1624: feature_name=HLA-DPA1	HLA-DPA1.value <= threshold=1.7050301432609558
node_1625: feature_name=CD83	CD83.value > threshold=2.783489227294922
node_1873: feature_name=IFI44L	IFI44L.value <= threshold=1.5938722491264343
node_1874: feature_name=PLD4	PLD4.value <= threshold=4.101001739501953
node_1875: feature_name=RHOB	RHOB.value > threshold=1.304835319519043
node_1965: feature_name=HLA-DQB1	HLA-DQB1.value > threshold=1.5989137291908264
node_1991: feature_name=COTL1	COTL1.value <= threshold=-1.2334879636764526
node_1992: feature_name=HLA-DRB5	HLA-DRB5.value > threshold=1.1685267686843872
node_1994: feature_name=RP5-821D11.7	RP5-821D11.7.value <= threshold=1.3020035326480865
Class: COVID-19 Critical	
Rules_1317	passed counts:3
node_0: feature_name=LTB	LTB.value <= threshold=0.994031548500061
node_1: feature_name=MTRNR2L2	MTRNR2L2.value <= threshold=2.8784297704696655
node_2: feature_name=TCL1A	TCL1A.value <= threshold=4.798312664031982
node_3: feature_name=MT-CO1	MT-CO1.value <= threshold=0.08947191759943962
node_4: feature_name=NR4A1	NR4A1.value > threshold=1.0725348591804504
node_1622: feature_name=HLA-DQA2	HLA-DQA2.value <= threshold=1.9496816992759705
node_1623: feature_name=HLA-DRB5	HLA-DRB5.value <= threshold=2.715766429901123
node_1624: feature_name=HLA-DPA1	HLA-DPA1.value <= threshold=1.7050301432609558
node_1625: feature_name=CD83	CD83.value > threshold=2.783489227294922
node_1873: feature_name=IFI44L	IFI44L.value <= threshold=1.5938722491264343
node_1874: feature_name=PLD4	PLD4.value <= threshold=4.101001739501953

node_1875: feature_name=RHOB	RHOB.value > threshold=1.304835319519043
node_1965: feature_name=HLA-DQB1	HLA-DQB1.value <= threshold=1.5989137291908264
node_1966: feature_name=CD79A	CD79A.value > threshold=2.189441442489624
node_1974: feature_name=IGJ	IGJ.value <= threshold=1.4252705574035645
node_1975: feature_name=PRDM1	PRDM1.value > threshold=-0.4423440992832184
Class: COVID-19 Severe	
Rules_1318	passed counts:3
node_0: feature_name=LTB	LTB.value <= threshold=0.994031548500061
node_1: feature_name=MTRNR2L2	MTRNR2L2.value <= threshold=2.8784297704696655
node_2: feature_name=TCL1A	TCL1A.value <= threshold=4.798312664031982
node_3: feature_name=MT-CO1	MT-CO1.value <= threshold=0.08947191759943962
node_4: feature_name=NR4A1	NR4A1.value > threshold=1.0725348591804504
node_1622: feature_name=HLA-DQA2	HLA-DQA2.value <= threshold=1.9496816992759705
node_1623: feature_name=HLA-DRB5	HLA-DRB5.value <= threshold=2.715766429901123
node_1624: feature_name=HLA-DPA1	HLA-DPA1.value <= threshold=1.7050301432609558
node_1625: feature_name=CD83	CD83.value > threshold=2.783489227294922
node_1873: feature_name=IFI44L	IFI44L.value <= threshold=1.5938722491264343
node_1874: feature_name=PLD4	PLD4.value <= threshold=4.101001739501953
node_1875: feature_name=RHOB	RHOB.value > threshold=1.304835319519043
node_1965: feature_name=HLA-DQB1	HLA-DQB1.value <= threshold=1.5989137291908264
node_1966: feature_name=CD79A	CD79A.value > threshold=2.189441442489624
node_1974: feature_name=IGJ	IGJ.value <= threshold=1.4252705574035645
node_1975: feature_name=PRDM1	PRDM1.value <= threshold=-0.4423440992832184
node_1976: feature_name=CCR6	CCR6.value <= threshold=0.9063289687037468
node_1977: feature_name=GRASP	GRASP.value <= threshold=-0.39720863103866577
node_1978: feature_name=TRIB1	TRIB1.value > threshold=-0.2772701233625412
Class: COVID-19 Severe	
Rules_1319	passed counts:3

node_0: feature_name=LTB	LTB.value <= threshold=0.994031548500061
node_1: feature_name=MTRNR2L2	MTRNR2L2.value <= threshold=2.8784297704696655
node_2: feature_name=TCL1A	TCL1A.value <= threshold=4.798312664031982
node_3: feature_name=MT-CO1	MT-CO1.value <= threshold=0.08947191759943962
node_4: feature_name=NR4A1	NR4A1.value > threshold=1.0725348591804504
node_1622: feature_name=HLA-DQA2	HLA-DQA2.value <= threshold=1.9496816992759705
node_1623: feature_name=HLA-DRB5	HLA-DRB5.value <= threshold=2.715766429901123
node_1624: feature_name=HLA-DPA1	HLA-DPA1.value <= threshold=1.7050301432609558
node_1625: feature_name=CD83	CD83.value > threshold=2.783489227294922
node_1873: feature_name=IFI44L	IFI44L.value <= threshold=1.5938722491264343
node_1874: feature_name=PLD4	PLD4.value <= threshold=4.101001739501953
node_1875: feature_name=RHOB	RHOB.value > threshold=1.304835319519043
node_1965: feature_name=HLA-DQB1	HLA-DQB1.value <= threshold=1.5989137291908264
node_1966: feature_name=CD79A	CD79A.value <= threshold=2.189441442489624
node_1967: feature_name=TNF	TNF.value > threshold=2.0489413142204285
node_1971: feature_name=ITM2C	ITM2C.value > threshold=-0.27933938801288605
Class: COVID-19 Critical	
Rules_1320	passed counts:3
node_0: feature_name=LTB	LTB.value <= threshold=0.994031548500061
node_1: feature_name=MTRNR2L2	MTRNR2L2.value <= threshold=2.8784297704696655
node_2: feature_name=TCL1A	TCL1A.value <= threshold=4.798312664031982
node_3: feature_name=MT-CO1	MT-CO1.value <= threshold=0.08947191759943962
node_4: feature_name=NR4A1	NR4A1.value > threshold=1.0725348591804504
node_1622: feature_name=HLA-DQA2	HLA-DQA2.value <= threshold=1.9496816992759705
node_1623: feature_name=HLA-DRB5	HLA-DRB5.value <= threshold=2.715766429901123
node_1624: feature_name=HLA-DPA1	HLA-DPA1.value <= threshold=1.7050301432609558
node_1625: feature_name=CD83	CD83.value > threshold=2.783489227294922
node_1873: feature_name=IFI44L	IFI44L.value <= threshold=1.5938722491264343
node_1874: feature_name=PLD4	PLD4.value <= threshold=4.101001739501953

node_1875: feature_name=RHOB	RHOB.value <= threshold=1.304835319519043
node_1876: feature_name=PPIF	PPIF.value <= threshold=2.2943923473358154
node_1877: feature_name=OAZ1	OAZ1.value > threshold=0.04260866530239582
node_1935: feature_name=RGS1	RGS1.value > threshold=-0.13325228542089462
node_1955: feature_name=DUSP4	DUSP4.value <= threshold=1.2541037425398827
node_1956: feature_name=BACH1	BACH1.value > threshold=2.0189278721809387
Class: COVID-19 Critical	
Rules_1321	passed counts:3
node_0: feature_name=LTB	LTB.value <= threshold=0.994031548500061
node_1: feature_name=MTRNR2L2	MTRNR2L2.value <= threshold=2.8784297704696655
node_2: feature_name=TCL1A	TCL1A.value <= threshold=4.798312664031982
node_3: feature_name=MT-CO1	MT-CO1.value <= threshold=0.08947191759943962
node_4: feature_name=NR4A1	NR4A1.value > threshold=1.0725348591804504
node_1622: feature_name=HLA-DQA2	HLA-DQA2.value <= threshold=1.9496816992759705
node_1623: feature_name=HLA-DRB5	HLA-DRB5.value <= threshold=2.715766429901123
node_1624: feature_name=HLA-DPA1	HLA-DPA1.value <= threshold=1.7050301432609558
node_1625: feature_name=CD83	CD83.value > threshold=2.783489227294922
node_1873: feature_name=IFI44L	IFI44L.value <= threshold=1.5938722491264343
node_1874: feature_name=PLD4	PLD4.value <= threshold=4.101001739501953
node_1875: feature_name=RHOB	RHOB.value <= threshold=1.304835319519043
node_1876: feature_name=PPIF	PPIF.value <= threshold=2.2943923473358154
node_1877: feature_name=OAZ1	OAZ1.value > threshold=0.04260866530239582
node_1935: feature_name=RGS1	RGS1.value <= threshold=-0.13325228542089462
node_1936: feature_name=BCL2A1	BCL2A1.value > threshold=2.528669595718384
Class: COVID-19 Severe	
Rules_1322	passed counts:3
node_0: feature_name=LTB	LTB.value <= threshold=0.994031548500061
node_1: feature_name=MTRNR2L2	MTRNR2L2.value <= threshold=2.8784297704696655

node_2: feature_name=TCL1A	TCL1A.value <= threshold=4.798312664031982
node_3: feature_name=MT-CO1	MT-CO1.value <= threshold=0.08947191759943962
node_4: feature_name=NR4A1	NR4A1.value > threshold=1.0725348591804504
node_1622: feature_name=HLA-DQA2	HLA-DQA2.value <= threshold=1.9496816992759705
node_1623: feature_name=HLA-DRB5	HLA-DRB5.value <= threshold=2.715766429901123
node_1624: feature_name=HLA-DPA1	HLA-DPA1.value <= threshold=1.7050301432609558
node_1625: feature_name=CD83	CD83.value > threshold=2.783489227294922
node_1873: feature_name=IFI44L	IFI44L.value <= threshold=1.5938722491264343
node_1874: feature_name=PLD4	PLD4.value <= threshold=4.101001739501953
node_1875: feature_name=RHOB	RHOB.value <= threshold=1.304835319519043
node_1876: feature_name=PPIF	PPIF.value <= threshold=2.2943923473358154
node_1877: feature_name=OAZ1	OAZ1.value <= threshold=0.04260866530239582
node_1878: feature_name=ID3	ID3.value <= threshold=5.290210247039795
node_1879: feature_name=NFKBID	NFKBID.value > threshold=-0.40532417595386505
node_1883: feature_name=MEF2C	MEF2C.value > threshold=-0.4597593992948532
node_1887: feature_name=SNX9	SNX9.value > threshold=1.010923683643341
node_1901: feature_name=RBKS	RBKS.value > threshold=4.642704963684082
node_1929: feature_name=NFKBIZ	NFKBIZ.value > threshold=0.7106845676898956
Class: COVID-19 Critical	
Rules_1323	passed counts:3
node_0: feature_name=LTB	LTB.value <= threshold=0.994031548500061
node_1: feature_name=MTRNR2L2	MTRNR2L2.value <= threshold=2.8784297704696655
node_2: feature_name=TCL1A	TCL1A.value <= threshold=4.798312664031982
node_3: feature_name=MT-CO1	MT-CO1.value <= threshold=0.08947191759943962
node_4: feature_name=NR4A1	NR4A1.value > threshold=1.0725348591804504
node_1622: feature_name=HLA-DQA2	HLA-DQA2.value <= threshold=1.9496816992759705
node_1623: feature_name=HLA-DRB5	HLA-DRB5.value <= threshold=2.715766429901123
node_1624: feature_name=HLA-DPA1	HLA-DPA1.value <= threshold=1.7050301432609558
node_1625: feature_name=CD83	CD83.value > threshold=2.783489227294922

node_1873: feature_name=IFI44L	IFI44L.value <= threshold=1.5938722491264343
node_1874: feature_name=PLD4	PLD4.value <= threshold=4.101001739501953
node_1875: feature_name=RHOB	RHOB.value <= threshold=1.304835319519043
node_1876: feature_name=PPIF	PPIF.value <= threshold=2.2943923473358154
node_1877: feature_name=OAZ1	OAZ1.value <= threshold=0.04260866530239582
node_1878: feature_name=ID3	ID3.value <= threshold=5.290210247039795
node_1879: feature_name=NFKBID	NFKBID.value > threshold=-0.40532417595386505
node_1883: feature_name=MEF2C	MEF2C.value > threshold=-0.4597593992948532
node_1887: feature_name=SNX9	SNX9.value > threshold=1.010923683643341
node_1901: feature_name=RBKS	RBKS.value > threshold=4.642704963684082
node_1929: feature_name=NFKBIZ	NFKBIZ.value <= threshold=0.7106845676898956
Class: COVID-19 Severe	
Rules_1324	passed counts:3
node_0: feature_name=LTB	LTB.value <= threshold=0.994031548500061
node_1: feature_name=MTRNR2L2	MTRNR2L2.value <= threshold=2.8784297704696655
node_2: feature_name=TCL1A	TCL1A.value <= threshold=4.798312664031982
node_3: feature_name=MT-CO1	MT-CO1.value <= threshold=0.08947191759943962
node_4: feature_name=NR4A1	NR4A1.value > threshold=1.0725348591804504
node_1622: feature_name=HLA-DQA2	HLA-DQA2.value <= threshold=1.9496816992759705
node_1623: feature_name=HLA-DRB5	HLA-DRB5.value <= threshold=2.715766429901123
node_1624: feature_name=HLA-DPA1	HLA-DPA1.value <= threshold=1.7050301432609558
node_1625: feature_name=CD83	CD83.value > threshold=2.783489227294922
node_1873: feature_name=IFI44L	IFI44L.value <= threshold=1.5938722491264343
node_1874: feature_name=PLD4	PLD4.value <= threshold=4.101001739501953
node_1875: feature_name=RHOB	RHOB.value <= threshold=1.304835319519043
node_1876: feature_name=PPIF	PPIF.value <= threshold=2.2943923473358154
node_1877: feature_name=OAZ1	OAZ1.value <= threshold=0.04260866530239582
node_1878: feature_name=ID3	ID3.value <= threshold=5.290210247039795
node_1879: feature_name=NFKBID	NFKBID.value > threshold=-0.40532417595386505

node_1883: feature_name=MEF2C	MEF2C.value > threshold=-0.4597593992948532
node_1887: feature_name=SNX9	SNX9.value > threshold=1.010923683643341
node_1901: feature_name=RBKS	RBKS.value <= threshold=4.642704963684082
node_1902: feature_name=TSPYL2	TSPYL2.value <= threshold=2.4923402070999146
node_1903: feature_name=MARCKS	MARCKS.value <= threshold=4.944992542266846
node_1904: feature_name=RELT	RELT.value <= threshold=2.6679846048355103
node_1905: feature_name=TCL1A	TCL1A.value <= threshold=4.318726301193237
node_1906: feature_name=ID1	ID1.value <= threshold=4.261367559432983
node_1907: feature_name=IGJ	IGJ.value > threshold=-0.3538896441459656
node_1915: feature_name=HLA-DRB5	HLA-DRB5.value > threshold=2.2267305850982666
node_1917: feature_name=CD22	CD22.value > threshold=-0.22111180424690247
node_1919: feature_name=MT-CO3	MT-CO3.value > threshold=-0.8478020578622818
Class: COVID-19 Severe	
Rules_1325	passed counts:3
node_0: feature_name=LTB	LTB.value <= threshold=0.994031548500061
node_1: feature_name=MTRNR2L2	MTRNR2L2.value <= threshold=2.8784297704696655
node_2: feature_name=TCL1A	TCL1A.value <= threshold=4.798312664031982
node_3: feature_name=MT-CO1	MT-CO1.value <= threshold=0.08947191759943962
node_4: feature_name=NR4A1	NR4A1.value > threshold=1.0725348591804504
node_1622: feature_name=HLA-DQA2	HLA-DQA2.value <= threshold=1.9496816992759705
node_1623: feature_name=HLA-DRB5	HLA-DRB5.value <= threshold=2.715766429901123
node_1624: feature_name=HLA-DPA1	HLA-DPA1.value <= threshold=1.7050301432609558
node_1625: feature_name=CD83	CD83.value > threshold=2.783489227294922
node_1873: feature_name=IFI44L	IFI44L.value <= threshold=1.5938722491264343
node_1874: feature_name=PLD4	PLD4.value <= threshold=4.101001739501953
node_1875: feature_name=RHOB	RHOB.value <= threshold=1.304835319519043
node_1876: feature_name=PPIF	PPIF.value <= threshold=2.2943923473358154
node_1877: feature_name=OAZ1	OAZ1.value <= threshold=0.04260866530239582
node_1878: feature_name=ID3	ID3.value <= threshold=5.290210247039795

node_1879: feature_name=NFKBID	NFKBID.value > threshold=-0.40532417595386505
node_1883: feature_name=MEF2C	MEF2C.value > threshold=-0.4597593992948532
node_1887: feature_name=SNX9	SNX9.value > threshold=1.010923683643341
node_1901: feature_name=RBKS	RBKS.value <= threshold=4.642704963684082
node_1902: feature_name=TSPYL2	TSPYL2.value <= threshold=2.4923402070999146
node_1903: feature_name=MARCKS	MARCKS.value <= threshold=4.944992542266846
node_1904: feature_name=RELT	RELT.value <= threshold=2.6679846048355103
node_1905: feature_name=TCL1A	TCL1A.value <= threshold=4.318726301193237
node_1906: feature_name=ID1	ID1.value <= threshold=4.261367559432983
node_1907: feature_name=IGJ	IGJ.value <= threshold=-0.3538896441459656
node_1908: feature_name=JARID2	JARID2.value > threshold=1.1454014778137207
node_1912: feature_name=RGS2	RGS2.value <= threshold=-0.09703248739242554
Class: COVID-19 Severe	
Rules_1326	passed counts:3
node_0: feature_name=LTB	LTB.value <= threshold=0.994031548500061
node_1: feature_name=MTRNR2L2	MTRNR2L2.value <= threshold=2.8784297704696655
node_2: feature_name=TCL1A	TCL1A.value <= threshold=4.798312664031982
node_3: feature_name=MT-CO1	MT-CO1.value <= threshold=0.08947191759943962
node_4: feature_name=NR4A1	NR4A1.value > threshold=1.0725348591804504
node_1622: feature_name=HLA-DQA2	HLA-DQA2.value <= threshold=1.9496816992759705
node_1623: feature_name=HLA-DRB5	HLA-DRB5.value <= threshold=2.715766429901123
node_1624: feature_name=HLA-DPA1	HLA-DPA1.value <= threshold=1.7050301432609558
node_1625: feature_name=CD83	CD83.value <= threshold=2.783489227294922
node_1626: feature_name=XIST	XIST.value <= threshold=0.9589543007314205
node_1627: feature_name=PLD4	PLD4.value <= threshold=5.865932464599609
node_1628: feature_name=OAZ1	OAZ1.value > threshold=0.6137169003486633
node_1840: feature_name=MT-CO1	MT-CO1.value > threshold=-0.28350088000297546
node_1850: feature_name=CD83	CD83.value > threshold=2.1227927207946777
node_1856: feature_name=WDR74	WDR74.value > threshold=1.5303557515144348

Class: COVID-19 Critical	
Rules_1327	passed counts:3
node_0: feature_name=LTB	LTB.value <= threshold=0.994031548500061
node_1: feature_name=MTRNR2L2	MTRNR2L2.value <= threshold=2.8784297704696655
node_2: feature_name=TCL1A	TCL1A.value <= threshold=4.798312664031982
node_3: feature_name=MT-CO1	MT-CO1.value <= threshold=0.08947191759943962
node_4: feature_name=NR4A1	NR4A1.value > threshold=1.0725348591804504
node_1622: feature_name=HLA-DQA2	HLA-DQA2.value <= threshold=1.9496816992759705
node_1623: feature_name=HLA-DRB5	HLA-DRB5.value <= threshold=2.715766429901123
node_1624: feature_name=HLA-DPA1	HLA-DPA1.value <= threshold=1.7050301432609558
node_1625: feature_name=CD83	CD83.value <= threshold=2.783489227294922
node_1626: feature_name=XIST	XIST.value <= threshold=0.9589543007314205
node_1627: feature_name=PLD4	PLD4.value <= threshold=5.865932464599609
node_1628: feature_name=OAZ1	OAZ1.value > threshold=0.6137169003486633
node_1840: feature_name=MT-CO1	MT-CO1.value > threshold=-0.28350088000297546
node_1850: feature_name=CD83	CD83.value > threshold=2.1227927207946777
node_1856: feature_name=WDR74	WDR74.value <= threshold=1.5303557515144348
node_1857: feature_name=BCL2A1	BCL2A1.value > threshold=1.396297037601471
node_1859: feature_name=STAG3	STAG3.value > threshold=-0.1998264193534851
Class: COVID-19 Critical	
Rules_1328	passed counts:3
node_0: feature_name=LTB	LTB.value <= threshold=0.994031548500061
node_1: feature_name=MTRNR2L2	MTRNR2L2.value <= threshold=2.8784297704696655
node_2: feature_name=TCL1A	TCL1A.value <= threshold=4.798312664031982
node_3: feature_name=MT-CO1	MT-CO1.value <= threshold=0.08947191759943962
node_4: feature_name=NR4A1	NR4A1.value > threshold=1.0725348591804504
node_1622: feature_name=HLA-DQA2	HLA-DQA2.value <= threshold=1.9496816992759705
node_1623: feature_name=HLA-DRB5	HLA-DRB5.value <= threshold=2.715766429901123

node_1624: feature_name=HLA-DPA1	HLA-DPA1.value <= threshold=1.7050301432609558
node_1625: feature_name=CD83	CD83.value <= threshold=2.783489227294922
node_1626: feature_name=XIST	XIST.value <= threshold=0.9589543007314205
node_1627: feature_name=PLD4	PLD4.value <= threshold=5.865932464599609
node_1628: feature_name=OAZ1	OAZ1.value <= threshold=0.6137169003486633
node_1629: feature_name=MTRNR2L2	MTRNR2L2.value > threshold=1.6102792620658875
Class: COVID-19 Severe	
Rules_1329	passed counts:3
node_0: feature_name=LTB	LTB.value <= threshold=0.994031548500061
node_1: feature_name=MTRNR2L2	MTRNR2L2.value <= threshold=2.8784297704696655
node_2: feature_name=TCL1A	TCL1A.value <= threshold=4.798312664031982
node_3: feature_name=MT-CO1	MT-CO1.value <= threshold=0.08947191759943962
node_4: feature_name=NR4A1	NR4A1.value > threshold=1.0725348591804504
node_1622: feature_name=HLA-DQA2	HLA-DQA2.value <= threshold=1.9496816992759705
node_1623: feature_name=HLA-DRB5	HLA-DRB5.value <= threshold=2.715766429901123
node_1624: feature_name=HLA-DPA1	HLA-DPA1.value <= threshold=1.7050301432609558
node_1625: feature_name=CD83	CD83.value <= threshold=2.783489227294922
node_1626: feature_name=XIST	XIST.value <= threshold=0.9589543007314205
node_1627: feature_name=PLD4	PLD4.value <= threshold=5.865932464599609
node_1628: feature_name=OAZ1	OAZ1.value <= threshold=0.6137169003486633
node_1629: feature_name=MTRNR2L2	MTRNR2L2.value <= threshold=1.6102792620658875
node_1630: feature_name=IFI6	IFI6.value > threshold=-0.8404486179351807
node_1632: feature_name=NR4A1	NR4A1.value > threshold=1.0916320085525513
node_1638: feature_name=DENND3	DENND3.value <= threshold=3.988452672958374
node_1639: feature_name=HLA-DRB1	HLA-DRB1.value > threshold=1.4092575907707214
node_1749: feature_name=HLA-DQB1	HLA-DQB1.value > threshold=3.4929215908050537
node_1835: feature_name=MT-CO1	MT-CO1.value > threshold=-0.7759651243686676
Class: COVID-19 Severe	

Rules_1330	passed counts:3
node_0: feature_name=LTB	LTB.value <= threshold=0.994031548500061
node_1: feature_name=MTRNR2L2	MTRNR2L2.value <= threshold=2.8784297704696655
node_2: feature_name=TCL1A	TCL1A.value <= threshold=4.798312664031982
node_3: feature_name=MT-CO1	MT-CO1.value <= threshold=0.08947191759943962
node_4: feature_name=NR4A1	NR4A1.value > threshold=1.0725348591804504
node_1622: feature_name=HLA-DQA2	HLA-DQA2.value <= threshold=1.9496816992759705
node_1623: feature_name=HLA-DRB5	HLA-DRB5.value <= threshold=2.715766429901123
node_1624: feature_name=HLA-DPA1	HLA-DPA1.value <= threshold=1.7050301432609558
node_1625: feature_name=CD83	CD83.value <= threshold=2.783489227294922
node_1626: feature_name=XIST	XIST.value <= threshold=0.9589543007314205
node_1627: feature_name=PLD4	PLD4.value <= threshold=5.865932464599609
node_1628: feature_name=OAZ1	OAZ1.value <= threshold=0.6137169003486633
node_1629: feature_name=MTRNR2L2	MTRNR2L2.value <= threshold=1.6102792620658875
node_1630: feature_name=IFI6	IFI6.value > threshold=-0.8404486179351807
node_1632: feature_name=NR4A1	NR4A1.value > threshold=1.0916320085525513
node_1638: feature_name=DENND3	DENND3.value <= threshold=3.988452672958374
node_1639: feature_name=HLA-DRB1	HLA-DRB1.value > threshold=1.4092575907707214
node_1749: feature_name=HLA-DQB1	HLA-DQB1.value > threshold=3.4929215908050537
node_1835: feature_name=MT-CO1	MT-CO1.value <= threshold=-0.7759651243686676
Class: COVID-19 Critical	
Rules_1331	passed counts:3
node_0: feature_name=LTB	LTB.value <= threshold=0.994031548500061
node_1: feature_name=MTRNR2L2	MTRNR2L2.value <= threshold=2.8784297704696655
node_2: feature_name=TCL1A	TCL1A.value <= threshold=4.798312664031982
node_3: feature_name=MT-CO1	MT-CO1.value <= threshold=0.08947191759943962
[truncated: 685,032 more chars]
